# Trends in adult body-mass index in 200 countries from 1975 to 2014: a pooled analysis of 1698 population-based measurement studies with 19·2 million participants

**DOI:** 10.1016/S0140-6736(16)30054-X

**Published:** 2016-04-02

**Authors:** NCD Risk Factor Collaboration, Pooled Analysis and Writing (*equal contribution), Mariachiara Di Cesare, James Bentham, Gretchen A Stevens, Bin Zhou, Goodarz Danaei, Yuan Lu, Honor Bixby, Melanie J Cowan, Leanne M Riley, Kaveh Hajifathalian, Léa Fortunato, Cristina Taddei, James E Bennett, Nayu Ikeda, Young-Ho Khang, Catherine Kyobutungi, Avula Laxmaiah, Yanping Li, Hsien-Ho Lin, J Jaime Miranda, Aya Mostafa, Maria L Turley, Christopher J Paciorek, Marc Gunter, Majid Ezzati, Country and Regional Data (*equal contribution; listed alphabetically), Ziad A Abdeen, Zargar Abdul Hamid, Niveen M Abu-Rmeileh, Benjamin Acosta-Cazares, Robert Adams, Wichai Aekplakorn, Carlos A Aguilar-Salinas, Alireza Ahmadvand, Wolfgang Ahrens, Mohamed M Ali, Ala’a Alkerwi, Mar Alvarez-Pedrerol, Eman Aly, Philippe Amouyel, Antoinette Amuzu, Lars Bo Andersen, Sigmund A Anderssen, Dolores S Andrade, Ranjit Mohan Anjana, Hajer Aounallah-Skhiri, Inger Ariansen, Tahir Aris, Nimmathota Arlappa, Dominique Arveiler, Felix K Assah, Mária Avdicová, Fereidoun Azizi, Bontha V Babu, Nagalla Balakrishna, Piotr Bandosz, José R Banegas, Carlo M Barbagallo, Alberto Barceló, Amina Barkat, Mauro V Barros, Iqbal Bata, Anwar M Batieha, Rosangela L Batista, Louise A Baur, Robert Beaglehole, Habiba Ben Romdhane, Mikhail Benet, Antonio Bernabe-Ortiz, Gailute Bernotiene, Heloisa Bettiol, Aroor Bhagyalaxmi, Sumit Bharadwaj, Santosh K Bhargava, Zaid Bhatti, Zulfiqar A Bhutta, HongSheng Bi, Yufang Bi, Peter Bjerregaard, Espen Bjertness, Marius B Bjertness, Cecilia Björkelund, Margaret Blake, Anneke Blokstra, Simona Bo, Martin Bobak, Lynne M Boddy, Bernhard O Boehm, Heiner Boeing, Carlos P Boissonnet, Vanina Bongard, Pascal Bovet, Lutgart Braeckman, Marjolijn C E Bragt, Imperia Brajkovich, Francesco Branca, Juergen Breckenkamp, Hermann Brenner, Lizzy M Brewster, Garry R Brian, Graziella Bruno, H B(as) Bueno-de-Mesquita, Anna Bugge, Con Burns, Antonio Cabrera de León, Joseph Cacciottolo, Tilema Cama, Christine Cameron, José Camolas, Günay Can, Ana Paula C Cândido, Vincenzo Capuano, Viviane C Cardoso, Maria J Carvalho, Felipe F Casanueva, Juan-Pablo Casas, Carmelo A Caserta, Katia Castetbon, Snehalatha Chamukuttan, Angelique W Chan, Queenie Chan, Himanshu K Chaturvedi, Nishi Chaturvedi, Chien-Jen Chen, Fangfang Chen, Huashuai Chen, Shuohua Chen, Zhengming Chen, Ching-Yu Cheng, Angela Chetrit, Arnaud Chiolero, Shu-Ti Chiou, Adela Chirita-Emandi, Yumi Cho, Kaare Christensen, Jerzy Chudek, Renata Cifkova, Frank Claessens, Els Clays, Hans Concin, Cyrus Cooper, Rachel Cooper, Tara C Coppinger, Simona Costanzo, Dominique Cottel, Chris Cowell, Cora L Craig, Ana B Crujeiras, Graziella D’Arrigo, Eleonora d’Orsi, Jean Dallongeville, Albertino Damasceno, Camilla T Damsgaard, Goodarz Danaei, Rachel Dankner, Luc Dauchet, Guy De Backer, Dirk De Bacquer, Giovanni de Gaetano, Stefaan De Henauw, Delphine De Smedt, Mohan Deepa, Alexander D Deev, Abbas Dehghan, Hélène Delisle, Francis Delpeuch, Klodian Dhana, Augusto F Di Castelnuovo, Juvenal Soares Dias-da-Costa, Alejandro Diaz, Shirin Djalalinia, Ha T P Do, Annette J Dobson, Chiara Donfrancesco, Angela Döring, Kouamelan Doua, Wojciech Drygas, Eruke E Egbagbe, Robert Eggertsen, Ulf Ekelund, Jalila El Ati, Paul Elliott, Reina Engle-Stone, Rajiv T Erasmus, Cihangir Erem, Louise Eriksen, Jorge Escobedo-de la Peña, Alun Evans, David Faeh, Caroline H Fall, Farshad Farzadfar, Francisco J Felix-Redondo, Trevor S Ferguson, Daniel Fernández-Bergés, Daniel Ferrante, Marika Ferrari, Catterina Ferreccio, Jean Ferrieres, Joseph D Finn, Krista Fischer, Eric Monterubio Flores, Bernhard Föger, Leng Huat Foo, Ann-Sofie Forslund, Stephen P Fortmann, Heba M Fouad, Damian K Francis, Maria do Carmo Franco, Oscar H Franco, Guillermo Frontera, Flavio D Fuchs, Sandra C Fuchs, Yuki Fujita, Takuro Furusawa, Zbigniew Gaciong, Mihai Gafencu, Dickman Gareta, Sarah P Garnett, Jean-Michel Gaspoz, Magda Gasull, Louise Gates, Johanna M Geleijnse, Anoosheh Ghasemian, Simona Giampaoli, Francesco Gianfagna, Jonathan Giovannelli, Aleksander Giwercman, Rebecca A Goldsmith, Marcela Gonzalez Gross, Juan P González Rivas, Mariano Bonet Gorbea, Frederic Gottrand, Sidsel Graff-Iversen, Dušan Grafnetter, Aneta Grajda, Maria G Grammatikopoulou, Ronald D Gregor, Tomasz Grodzicki, Anders Grøntved, Grabriella Gruden, Vera Grujic, Dongfeng Gu, Ong Peng Guan, Vilmundur Gudnason, Ramiro Guerrero, Idris Guessous, Andre L Guimaraes, Martin C Gulliford, Johanna Gunnlaugsdottir, Marc Gunter, Xiu H Guo, Yin Guo, Prakash C Gupta, Oye Gureje, Beata Gurzkowska, Laura Gutierrez, Felix Gutzwiller, Jytte Halkjær, Rebecca Hardy, Rachakulla Hari Kumar, Alison J Hayes, Jiang He, Marleen Elisabeth Hendriks, Leticia Hernandez Cadena, Ramin Heshmat, Ilpo Tapani Hihtaniemi, Sai Yin Ho, Suzanne C Ho, Michael Hobbs, Albert Hofman, Claudia M Hormiga, Bernardo L Horta, Leila Houti, Thein Thein Htay, Aung Soe Htet, Maung Maung Than Htike, Yonghua Hu, Abdullatif S Hussieni, Chinh Nguyen Huu, Inge Huybrechts, Nahla Hwalla, Licia Iacoviello, Anna G Iannone, M Mohsen Ibrahim, Nayu Ikeda, M Arfan Ikram, Vilma E Irazola, Muhammad Islam, Masanori Iwasaki, Rod T Jackson, Jeremy M Jacobs, Tazeen Jafar, Kazi M Jamil, Konrad Jamrozik, Grazyna Jasienska, Chao Qiang Jiang, Michel Joffres, Mattias Johansson, Jost B Jonas, Torben Jørgensen, Pradeep Joshi, Anne Juolevi, Gregor Jurak, Vesna Jureša, Rudolf Kaaks, Anthony Kafatos, Ofra Kalter-Leibovici, Efthymios Kapantais, Amir Kasaeian, Joanne Katz, Prabhdeep Kaur, Maryam Kavousi, Ulrich Keil, Lital Keinan Boker, Roya Kelishadi, Han H C G Kemper, Andre P Kengne, Mathilde Kersting, Timothy Key, Yousef Saleh Khader, Davood Khalili, Young-Ho Khang, Kay-Tee H Khaw, Ilse M S L Khouw, Stefan Kiechl, Japhet Killewo, Jeongseon Kim, Yutaka Kiyohara, Jeannette Klimont, Elin Kolle, Patrick Kolsteren, Paul Korrovits, Seppo Koskinen, Katsuyasu Kouda, Slawomir Koziel, Wolfgang Kratzer, Steinar Krokstad, Daan Kromhout, Herculina S Kruger, Krzysztof Kula, Zbigniew Kulaga, R Krishna Kumar, Yadlapalli S Kusuma, Kari Kuulasmaa, Catherine Kyobutungi, Fatima Zahra Laamiri, Tiina Laatikainen, Carl Lachat, Youcef Laid, Tai Hing Lam, Orlando Landrove, Vera Lanska, Georg Lappas, Lars E Laugsand, Avula Laxmaiah, Khanh Le Nguyen Bao, Tuyen D Le, Catherine Leclercq, Jeannette Lee, Jeonghee Lee, Terho Lehtimäki, Rampal Lekhraj, Luz M León-Muñoz, Yanping Li, Wei-Yen Lim, M Fernanda Lima-Costa, Hsien-Ho Lin, Xu Lin, Allan Linneberg, Lauren Lissner, Mieczyslaw Litwin, Jing Liu, Roberto Lorbeer, Paulo A Lotufo, José Eugenio Lozano, Dalia Luksiene, Annamari Lundqvist, Nuno Lunet, Per Lytsy, Guansheng Ma, Suka Machi, Stefania Maggi, Dianna J Magliano, Marcia Makdisse, Reza Malekzadeh, Rahul Malhotra, Kodavanti Mallikharjuna Rao, Yannis Manios, Jim I Mann, Enzo Manzato, Paula Margozzini, Oonagh Markey, Pedro Marques-Vidal, Jaume Marrugat, Yves Martin-Prevel, Reynaldo Martorell, Shariq R Masoodi, Tandi E Matsha, Artur Mazur, Jean Claude N Mbanya, Shelly R McFarlane, Stephen T McGarvey, Martin McKee, Stela McLachlan, Rachael M McLean, Breige A McNulty, Safiah Md Yusof, Sounnia Mediene-Benchekor, Aline Meirhaeghe, Christa Meisinger, Larissa L Mendes, Ana Maria B Menezes, Gert B M Mensink, Indrapal I Meshram, Andres Metspalu, Jie Mi, Kim F Michaelsen, Kairit Mikkel, Jody C Miller, Juan Francisco Miquel, J Jaime Miranda, Marjeta Mišigoj-Duraković, Mostafa K Mohamed, Kazem Mohammad, Noushin Mohammadifard, Viswanathan Mohan, Muhammad Fadhli Mohd Yusoff, Drude Molbo, Niels C Møller, Dénes Molnár, Charles K Mondo, Eric A Monterrubio, Kotsedi Daniel K Monyeki, Leila B Moreira, Alain Morejon, Luis A Moreno, Karen Morgan, Erik Lykke Mortensen, George Moschonis, Malgorzata Mossakowska, Aya Mostafa, Jorge Mota, Mohammad Esmaeel Motlagh, Jorge Motta, Thet Thet Mu, Maria Lorenza Muiesan, Martina Müller-Nurasyid, Neil Murphy, Jaakko Mursu, Elaine M Murtagh, Kamarul Imran Musa, Vera Musil, Gabriele Nagel, Harunobu Nakamura, Jana Námešná, Ei Ei K Nang, Vinay B Nangia, Martin Nankap, Sameer Narake, Eva Maria Navarrete-Muñoz, Ilona Nenko, Martin Neovius, Flavio Nervi, Hannelore K Neuhauser, Nguyen D Nguyen, Quang Ngoc Nguyen, Ramfis E Nieto-Martínez, Guang Ning, Toshiharu Ninomiya, Sania Nishtar, Marianna Noale, Teresa Norat, Davide Noto, Mohannad Al Nsour, Dermot O’Reilly, Angélica M Ochoa-Avilés, Kyungwon Oh, Iman H Olayan, Maria Teresa Anselmo Olinto, Maciej Oltarzewski, Mohd A Omar, Altan Onat, Pedro Ordunez, Ana P Ortiz, Merete Osler, Clive Osmond, Sergej M Ostojic, Johanna A Otero, Kim Overvad, Fred Michel Paccaud, Cristina Padez, Andrzej Pajak, Domenico Palli, Alberto Palloni, Luigi Palmieri, Songhomitra Panda-Jonas, Francesco Panza, Winsome R Parnell, Mahboubeh Parsaeian, Mangesh S Pednekar, Petra H Peeters, Sergio Viana Peixoto, Alexandre C Pereira, Cynthia M Pérez, Annette Peters, Niloofar Peykari, Son Thai Pham, Iris Pigeot, Hynek Pikhart, Aida Pilav, Lorenza Pilotto, Francesco Pistelli, Freda Pitakaka, Aleksandra Piwonska, Jerzy Piwonski, Pedro Plans-Rubió, Bee Koon Poh, Miquel Porta, Marileen L P Portegies, Dimitrios Poulimeneas, Rajendra Pradeepa, Mathur Prashant, Jacqueline F Price, Maria Puiu, Margus Punab, Radwan F Qasrawi, Mostafa Qorbani, Tran Quoc Bao, Ivana Radic, Ricardas Radisauskas, Mahmudur Rahman, Olli Raitakari, Manu Raj, Sudha Ramachandra Rao, Ambady Ramachandran, Jacqueline Ramke, Rafel Ramos, Sanjay Rampal, Finn Rasmussen, Josep Redon, Paul Ferdinand M Reganit, Robespierre Ribeiro, Elio Riboli, Fernando Rigo, Tobias Floris Rinke de Wit, Raphael M Ritti-Dias, Juan A Rivera, Sian M Robinson, Cynthia Robitaille, Fernando Rodríguez-Artalejo, María del Cristo Rodriguez-Perez, Laura A Rodríguez-Villamizar, Rosalba Rojas-Martinez, Nipa Rojroongwasinkul, Dora Romaguera, Kimmo Ronkainen, Annika Rosengren, Ian Rouse, Adolfo Rubinstein, Frank J Rühli, Ornelas Rui, Blanca Sandra Ruiz-Betancourt, Andrea R V Russo Horimoto, Marcin Rutkowski, Charumathi Sabanayagam, Harshpal S Sachdev, Olfa Saidi, Benoit Salanave, Eduardo Salazar Martinez, Veikko Salomaa, Jukka T Salonen, Massimo Salvetti, Jose Sánchez-Abanto, Sandjaja, Susana Sans, Diana A Santos, Osvaldo Santos, Renata Nunes dos Santos, Rute Santos, Luis B Sardinha, Nizal Sarrafzadegan, Kai-Uwe Saum, Savvas C Savva, Marcia Scazufca, Angelika Schaffrath Rosario, Herman Schargrodsky, Anja Schienkiewitz, Ida Maria Schmidt, Ione J Schneider, Constance Schultsz, Aletta E Schutte, Aye Aye Sein, Abhijit Sen, Idowu O Senbanjo, Sadaf G Sepanlou, Svetlana A Shalnova, Jonathan E Shaw, Kenji Shibuya, Youchan Shin, Rahman Shiri, Rosalynn Siantar, Abla M Sibai, Antonio M Silva, Diego Augusto Santos Silva, Mary Simon, Judith Simons, Leon A Simons, Michael Sjostrom, Jolanta Slowikowska-Hilczer, Przemyslaw Slusarczyk, Liam Smeeth, Margaret C Smith, Marieke B Snijder, Hung-Kwan So, Eugène Sobngwi, Stefan Soderberg, Moesijanti Y E Soekatri, Vincenzo Solfrizzi, Emily Sonestedt, Thorkild I A Sørensen, Maroje Sorić, Charles Sossa Jérome, Aicha Soumare, Jan A Staessen, Gregor Starc, Maria G Stathopoulou, Kaspar Staub, Bill Stavreski, Jostein Steene-Johannessen, Peter Stehle, Aryeh D Stein, George S Stergiou, Jochanan Stessman, Jutta Stieber, Doris Stöckl, Tanja Stocks, Jakub Stokwiszewski, Gareth Stratton, Maria Wany Strufaldi, Chien-An Sun, Johan Sundström, Yn-Tz Sung, Jordi Sunyer, Paibul Suriyawongpaisal, Boyd A Swinburn, Rody G Sy, Lucjan Szponar, E Shyong Tai, Mari-Liis Tammesoo, Abdonas Tamosiunas, Line Tang, Xun Tang, Frank Tanser, Yong Tao, Mohammed Tarawneh, Jakob Tarp, Carolina B Tarqui-Mamani, Anne Taylor, Félicité Tchibindat, Lutgarde Thijs, Betina H Thuesen, Anne Tjonneland, Hanna K Tolonen, Janne S Tolstrup, Murat Topbas, Roman Topór-Madry, Maties Torrent, Pierre Traissac, Antonia Trichopoulou, Dimitrios Trichopoulos, Oanh TH Trinh, Atul Trivedi, Lechaba Tshepo, Marshall K Tulloch-Reid, Tomi-Pekka Tuomainen, Jaakko Tuomilehto, Maria L Turley, Per Tynelius, Themistoklis Tzotzas, Christophe Tzourio, Peter Ueda, Flora AM Ukoli, Hanno Ulmer, Belgin Unal, Gonzalo Valdivia, Susana Vale, Damaskini Valvi, Yvonne T van der Schouw, Koen Van Herck, Hoang Van Minh, Irene G M van Valkengoed, Dirk Vanderschueren, Diego Vanuzzo, Lars Vatten, Tomas Vega, Gustavo Velasquez-Melendez, Giovanni Veronesi, W M Monique Verschuren, Giovanni Viegi, Lucie Viet, Eira Viikari-Juntura, Paolo Vineis, Jesus Vioque, Jyrki K Virtanen, Sophie Visvikis-Siest, Bharathi Viswanathan, Peter Vollenweider, Sari Voutilainen, Martine Vrijheid, Alisha N Wade, Aline Wagner, Janette Walton, Wan Nazaimoon Wan Mohamud, Ming-Dong Wang, Qian Wang, Ya Xing Wang, S Goya Wannamethee, Nicholas Wareham, Deepa Weerasekera, Peter H Whincup, Kurt Widhalm, Indah S Widyahening, Andrzej Wiecek, Rainford J Wilks, Johann Willeit, Bogdan Wojtyniak, Jyh Eiin Wong, Tien Yin Wong, Jean Woo, Mark Woodward, Frederick C Wu, JianFeng Wu, Shou Ling Wu, Haiquan Xu, Liang Xu, Uruwan Yamborisut, Weili Yan, Xiaoguang Yang, Nazan Yardim, Xingwang Ye, Panayiotis K Yiallouros, Akihiro Yoshihara, Qi Sheng You, Novie O Younger-Coleman, Ahmad F Yusoff, Ahmad A Zainuddin, Sabina Zambon, Tomasz Zdrojewski, Yi Zeng, Dong Zhao, Wenhua Zhao, Yingfeng Zheng, Maigeng Zhou, Dan Zhu, Esther Zimmermann, Julio Zuñiga Cisneros

**Affiliations:** Imperial College London, London, UK; Middlesex University, London, UK); Imperial College London, London, UK; World Health Organization, Geneva, Switzerland; Imperial College London, London, UK; Harvard T H Chan School of Public Health, Boston, MA, USA; Harvard T H Chan School of Public Health, Boston, MA, USA; Imperial College London, London, UK; World Health Organization, Geneva, Switzerland; World Health Organization, Geneva, Switzerland; Harvard T H Chan School of Public Health, Boston, MA, USA; Imperial College London, London, UK; University of Florence, Florence, Italy; Imperial College London, London, UK; National Institute of Health and Nutrition, Tokyo, Japan; Seoul National University, Seoul, South Korea; African Population and Health Research Center, Nairobi, Kenya; Indian Council of Medical Research, New Delhi, India; Harvard T H Chan School of Public Health, Boston, MA, USA; National Taiwan University, Taipei, Taiwan; Universidad Peruana Cayetano Heredia, Lima, Peru; Ain Shams University, Cairo, Egypt; Ministry of Health, Wellington, New Zealand; University of California, Berkeley, CA, USA; Imperial College London, London, UK; Imperial College London, London, UK; Al-Quds University, Palestine; Center for Diabetes and Endocrine Care, India; Birzeit University, Palestine; Institute Mexicano del Seguro Social, Mexico; The University of Adelaide, Australia; Mahidol University, Thailand; Instituto Nacional de Ciencias Médicas y Nutricion, Mexico; Non-Communicable Diseases Research Center, Iran; Leibniz Institute for Prevention Research and Epidemiology—BIPS, Germany; World Health Organization Regional Office for the Eastern Mediterranean, Egypt; Luxembourg Institute of Health, Luxembourg; Centre for Research in Environmental Epidemiology, Spain; World Health Organization Regional Office for the Eastern Mediterranean, Egypt; Lille University and Hospital, France; London School of Hygiene & Tropical Medicine, UK; University of Southern Denmark, Denmark; Norwegian School of Sport Sciences, Norway; Universidad de Cuenca, Ecuador; Madras Diabetes Research Foundation, India; National Institute of Public Health, Tunisia; Norwegian Institute of Public Health, Norway; Ministry of Health, Malaysia; Indian Council of Medical Research, India; Strasbourg University and Hospital, France; University of Yaoundé 1, Cameroon; Regional Authority of Public Health, Banska Bystrica, Slovakia; Shahid Beheshti University of Medical Sciences, Iran; Indian Council of Medical Research, India; Indian Council of Medical Research, India; Medical University of Gdansk, Poland; Universidad Autónoma de Madrid, Spain; University of Palermo, Italy; Pan American Health Organization, USA; Université Mohammed V de Rabat, Morocco; University of Pernambuco, Brazil; Dalhousie University, Canada; Jordan University of Science and Technology, Jordan; Federal University of Maranhao, Brazil; University of Sydney, Australia; University of Auckland, New Zealand; University Tunis El Manar, Tunisia; University Medical Science, Cuba; Universidad Peruana Cayetano Heredia, Peru; Lithuanian University of Health Sciences, Lithuania; University of São Paulo, Brazil; B J Medical College, India; Chirayu Medical College, India; Sunder Lal Jain Hospital, India; The Aga Khan University, Pakistan; The Aga Khan University, Pakistan; Shandong University of Traditional Chinese Medicine, China; Shanghai Jiao-Tong University School of Medicine, China; University of Southern Denmark, Denmark; University of Greenland, Greenland; University of Oslo, Norway; University of Oslo, Norway; University of Gothenburg, Sweden; NatCen Social Research, UK; National Institute for Public Health and the Environment, Netherlands; University of Turin, Italy; University College London, UK; Liverpool John Moores University, UK; Nanyang Technological University, Singapore; German Institute of Human Nutrition, Germany; CEMIC, Argentina; Toulouse University School of Medicine, France; Ministry of Health, Seychelles; University of Lausanne, Switzerland; Ghent University, Belgium; FrieslandCampina, Singapore; Universidad Central de Venezuela, Venezuela; World Health Organization, Switzerland; Bielefeld University, Germany; German Cancer Research Center, Germany; University of Amsterdam, Netherlands; The Fred Hollows Foundation New Zealand, New Zealand; University of Turin, Italy; National Institute for Public Health and the Environment, Netherlands; University of Southern Denmark, Denmark; Cork Institute of Technology, Ireland; University La Laguna, Spain; University of Malta, Malta; Ministry of Health, Tonga; Canadian Fitness and Lifestyle Research Institute, Canada; Hospital Santa Maria, CHLN, Portugal; Istanbul University, Turkey; Universidade Federal de Juiz de Fora, Brazil; Cardiologia di Mercato S. Severino, Italy; University of São Paulo, Brazil; University of Porto, Portugal; Santiago de Compostela University, Spain; University College London, UK; Associazione Calabrese di Epatologia, Italy; French Institute for Health Surveillance, France; India Diabetes Research Foundation, India; Duke-NUS Graduate Medical School, Singapore; Imperial College London, UK; National Institute of Medical Statistics, India; University College London, UK; Academia Sinica, Taiwan; Capital Institute of Pediatrics, China; Duke University, USA; Kailuan General Hospital, China; University of Oxford, UK; Duke-NUS Graduate Medical School, Singapore; The Gertner Institute for Epidemiology and Health Policy Research, Israel; Lausanne University Hospital, Switzerland; Ministry of Health and Welfare, Taiwan; Victor Babeş University of Medicine and Pharmacy, Romania; Korea Centers for Disease Control and Prevention, South Korea; University of Southern Denmark, Denmark; Medical University of Silesia, Poland; Charles University in Prague, Czech Republic; Katholieke Universiteit Leuven, Belgium; Ghent University, Belgium; Agency for Preventive and Social Medicine, Austria; University of Southampton, UK; University College London, UK; Cork Institute of Technology, Ireland; IRCCS Istituto Neurologico Mediterraneo Neuromed, Italy; Institut Pasteur de Lille, France; Westmead University of Sydney, Australia; Canadian Fitness and Lifestyle Research Institute, Canada; CIBEROBN, Spain; National Council of Research, Italy; Federal University of Santa Catarina, Brazil; Institut Pasteur de Lille, France; Eduardo Mondlane University, Mozambique; University of Copenhagen, Denmark; Harvard T H Chan School of Public Health, USA; The Gertner Institute for Epidemiology and Health Policy Research, Israel; Lille University Hospital, France; Ghent University, Belgium; Ghent University, Belgium; IRCCS Istituto Neurologico Mediterraneo Neuromed, Italy; Ghent University, Belgium; Ghent University, Belgium; Madras Diabetes Research Foundation, India; National Research Centre for Preventive Medicine, Russia; Erasmus Medical Center Rotterdam, Netherlands; University of Montreal, Canada; Institut de Recherche pour le Développement, France; Erasmus Medical Center Rotterdam, Netherlands; IRCCS Istituto Neurologico Mediterraneo Neuromed, Italy; Universidade do Vale do Rio dos Sinos, Brazil; National Council of Scientific and Technical Research, Argentina; Non-Communicable Diseases Research Center, Iran; National Institute of Nutrition, Vietnam; University of Queensland, Australia; Istituto Superiore di Sanità, Italy; Helmholtz Zentrum München, Germany; Ministère de la Santé et de la Lutte Contre le Sida, Côte d’Ivoire; The Cardinal Wyszynski Institute of Cardiology, Poland; University of Benin College of Medical Sciences, Nigeria; University of Gothenburg, Sweden; Norwegian School of Sport Sciences, Norway; National Institute of Nutrition and Food Technology, Tunisia; Imperial College London, UK; University of California Davis, USA; University of Stellenbosch, South Africa; Karadeniz Technical University, Turkey; University of Southern Denmark, Denmark; Institute Mexicano del Seguro Social, Mexico; The Queen’s University of Belfast, UK; University of Zurich, Switzerland; University of Southampton, UK; Tehran University of Medical Sciences, Iran; Centro de Salud Villanueva Norte, Spain; The University of the West Indies, Jamaica; Hospital Don Benito-Villanueva de la Serena, Spain; Ministry of Health, Argentina; Council for Agriculture Research and Economics, Italy; Pontificia Universidad Católica de Chile, Chile; Toulouse University School of Medicine, France; University of Manchester, UK; University of Tartu, Estonia; Instituto Nacional de Salud Pública, Mexico; Agency for Preventive and Social Medicine, Austria; Universiti Sains Malaysia, Malaysia; Luleå University, Sweden; Stanford University, USA; WHO Regional Office for the Eastern Mediterranean, Egypt; The University of the West Indies, Jamaica; Federal University of São Paulo, Brazil; Erasmus Medical Center Rotterdam, Netherlands; Hospital Universitario Son Espases, Spain; Hospital de Clinicas de Porto Alegre, Brazil; Universidade Federal do Rio Grande do Sul, Brazil; Kinki University Faculty of Medicine, Japan; Kyoto University, Japan; Medical University of Warsaw, Poland; Victor Babeş University of Medicine and Pharmacy, Romania; University of KwaZulu-Natal, South Africa; University of Sydney, Australia; Geneva University Hospitals, Switzerland; CIBER en Epidemiología y Salud Pública, Spain; Australian Bureau of Statistics, Australia; Wageningen University, Netherlands; Non-Communicable Diseases Research Center, Iran; Istituto Superiore di Sanità, Italy; University of Insubria, Italy; Lille University Hospital, France; Lund University, Sweden; Nutrition Department, Ministry of Health, Israel; Universidad Politécnica de Madrid, Spain; The Andes Clinic of Cardio-Metabolic Studies, Venezuela; National Institute of Hygiene, Epidemiology and Microbiology, Cuba; Université de Lille 2, France; Norwegian Institute of Public Health, Norway; Institute for Clinical and Experimental Medicine, Czech Republic; The Children’s Memorial Health Institute, Poland; Alexander Technological Educational Institute, Greece; Dalhousie University, Canada; Jagiellonian University Medical College, Poland; University of Southern Denmark, Denmark; University of Turin, Italy; Institute of Public Health of Vojvodina, Serbia; National Center of Cardiovascular Diseases, China; Singapore Eye Research Institute, Singapore; Icelandic Heart Association, Iceland; Universidad Icesi, Colombia; Geneva University Hospitals, Switzerland; State University of Montes Claros, Brazil; King’s College London, UK; Icelandic Heart Association, Iceland; Imperial College London, UK; Capital Medical University, China; Capital Medical University, China; Healis-Sekhsaria Institute for Public Health, India; University of Ibadan, Nigeria; The Children’s Memorial Health Institute, Poland; Institute for Clinical Effectiveness and Health Policy, Argentina; University of Zurich, Switzerland; Danish Cancer Society Research Centre, Denmark; University College London, UK; Indian Council of Medical Research, India; University of Sydney, Australia; Tulane University, USA; University of Amsterdam Academic Medical Center, Netherlands; National Institute of Public Health, Mexico; Tehran University of Medical Sciences, Iran; Imperial College London, UK; University of Hong Kong, China; The Chinese University of Hong Kong, China; University of Western Australia, Australia; Erasmus Medical Center Rotterdam, Netherlands; Fundación Oftalmológica de Santander, Colombia; Universidade Federal de Pelotas, Brazil; University of Oran 1, Algeria; Ministry of Health, Myanmar; University of Oslo, Norway; Ministry of Health, Myanmar; Peking University Health Science Center, China; Birzeit University, Palestine; National Institute of Nutrition, Vietnam; International Agency for Research on Cancer, France; American University of Beirut, Lebanon; IRCCS Istituto Neurologico Mediterraneo Neuromed, Italy; Cardiologia di Mercato S. Severino, Italy; Cairo University, Egypt; National Institute of Health and Nutrition, Japan; Erasmus Medical Center Rotterdam, Netherlands; Institute for Clinical Effectiveness and Health Policy, Argentina; Aga Khan University, Pakistan; Niigata University, Japan; University of Auckland, New Zealand; Hadassah University Medical Center, Israel; Duke-NUS Graduate Medical School, Singapore; Kuwait Institute for Scientific Research, Kuwait; University of Adelaide, Australia; deceased; Jagiellonian University Medical College, Poland; Guangzhou 12th Hospital, China; Simon Fraser University, Canada; International Agency for Research on Cancer, France; Ruprecht-Karls-University of Heidelberg, Germany; Research Centre for Prevention and Health, Denmark; World Health Organization Country Office, India; National Institute for Health and Welfare, Finland; University of Ljubljana, Slovenia; University of Zagreb, Croatia; German Cancer Research Center, Germany; University of Crete, Greece; The Gertner Institute for Epidemiology and Health Policy Research, Israel; Hellenic Medical Association for Obesity, Greece; Non-Communicable Diseases Research Center, Iran; Johns Hopkins Bloomberg School of Public Health, USA; National Institute of Epidemiology, India; Erasmus Medical Center Rotterdam, Netherlands; University of Münster, Germany; University of Haifa, Israel; Research Institute for Primordial Prevention of Non-Communicable Disease, Iran; VU University Medical Center, Netherlands; South African Medical Research Council, South Africa; Research Institute of Child Nutrition (FKE), Germany; University of Oxford, UK; Jordan University of Science and Technology, Jordan; Shahid Beheshti University of Medical Sciences, Iran; Seoul National University, South Korea; University of Cambridge, UK; FrieslandCampina, Singapore; Medical University Innsbruck, Austria; Muhimbili University of Health and Allied Sciences, Tanzania; National Cancer Center, South Korea; Kyushu University, Japan; Statistics Austria, Austria; Norwegian School of Sport Sciences, Norway; Institute of Tropical Medicine, Belgium; Tartu University Clinics, Estonia; National Institute for Health and Welfare, Finland; Kinki University Faculty of Medicine, Japan; Polish Academy of Sciences Anthropology Unit in Wroclaw, Poland; University Hospital Ulm, Germany; Norwegian University of Science and Technology, Norway; Wageningen University, Netherlands; North-West University, South Africa; Medical University of Lodz, Poland; The Children’s Memorial Health Institute, Poland; Amrita Institute of Medical Sciences, India; All India Institute of Medical Sciences, India; National Institute for Health and Welfare, Finland; African Population and Health Research Center, Kenya; Université Mohammed V de Rabat, Morocco; National Institute for Health and Welfare, Finland; Ghent University, Belgium; National Institute of Public Health of Algeria, Algeria; University of Hong Kong, China; Ministerio de Salud Pública, Cuba; Institute for Clinical and Experimental Medicine, Czech Republic; Sahlgrenska Academy, Sweden; Norwegian University of Science and Technology, Norway; Indian Council of Medical Research, India; National Institute of Nutrition, Vietnam; National Institute of Nutrition, Vietnam; Food and Agriculture Organization, Italy; National University of Singapore, Singapore; National Cancer Center, South Korea; Tampere University Hospital, Finland; Universiti Putra Malaysia, Malaysia; Universidad Autónoma de Madrid, Spain; Harvard T H Chan School of Public Health, USA; National University of Singapore, Singapore; Oswaldo Cruz Foundation Rene Rachou Research Institute, Brazil; National Taiwan University, Taiwan; University of Chinese Academy of Sciences, China; Research Centre for Prevention and Health, Denmark; University of Gothenburg, Sweden; The Children’s Memorial Health Institute, Poland; Capital Medical University, Beijing Anzhen Hospital, China; University Medicine Greifswald, Germany; University of São Paulo, Brazil; Consejería de Sanidad Junta de Castilla y León, Spain; Lithuanian University of Health Sciences, Lithuania; National Institute for Health and Welfare, Finland; University of Porto Medical School, Portugal; University of Uppsala, Sweden; Peking University, China; The Jikei University School of Medicine, Japan; National Research Council, Italy; Baker IDI Heart and Diabetes Institute, Australia; Hospital Israelita Albert Einstein, Brazil; Tehran University of Medical Sciences, Iran; Duke-NUS Graduate Medical School, Singapore; Indian Council of Medical Research, India; Harokopio University of Athens, Greece; University of Otago, New Zealand; University of Padova, Italy; Pontificia Universidad Católica de Chile, Chile; University of Reading, UK; Lausanne University Hospital, Switzerland; Institut Hospital del Mar d’Investigacions Mèdiques, Spain; Institut de Recherche pour le Développement, France; Emory University, USA; Sher-i-Kashmir Institute of Medical Sciences, India; Cape Peninsula University of Technology, South Africa; University of Rzeszow, Poland; University of Yaoundé 1, Cameroon; The University of the West Indies, Jamaica; Brown University, USA; London School of Hygiene & Tropical Medicine, UK; University of Edinburgh, UK; University of Otago, New Zealand; University College Dublin, Ireland; Universiti Teknologi MARA, Malaysia; University of Oran 1, Algeria; Institut National de la Santé et de la Recherche Médicale, France; Helmholtz Zentrum München, Germany; Universidade Federal de Juiz de Fora, Brazil; Universidade Federal de Pelotas, Brazil; Robert Koch Institute, Germany; Indian Council of Medical Research, India; University of Tartu, Estonia; Capital Institute of Pediatrics, China; University of Copenhagen, Denmark; University of Tartu, Estonia; University of Otago, New Zealand; Pontificia Universidad Católica de Chile, Chile; Universidad Peruana Cayetano Heredia, Peru; University of Zagreb, Croatia; Ain Shams University, Egypt; Tehran University of Medical Sciences, Iran; Isfahan Cardiovascular Research Center, Iran; Madras Diabetes Research Foundation, India; Ministry of Health, Malaysia; University of Copenhagen, Denmark; University of Southern Denmark, Denmark; University of Pécs, Hungary; Mulago Hospital, Uganda; Instituto Nacional de Salud Pública, Mexico; University of Limpopo, South Africa; Universidade Federal do Rio Grande do Sul, Brazil; University Medical Science, Cuba; Universidad de Zaragoza, Spain; RCSI Dublin, Ireland; University of Copenhagen, Denmark; Harokopio University of Athens, Greece; International Institute of Molecular and Cell Biology, Poland; Ain Shams University, Egypt; University of Porto, Portugal; Ahvaz Jundishapur University of Medical Sciences, Iran; Gorgas Memorial Institute of Public Health, Panama; Ministry of Health, Myanmar; University of Brescia, Italy; Helmholtz Zentrum München, Germany; Imperial College London, UK; University of Eastern Finland, Finland; Mary Immaculate College, Ireland; Universiti Sains Malaysia, Kota Bharu, Malaysia; University of Zagreb, Croatia; Ulm University, Germany; Kobe University, Japan; Regional Authority of Public Health, Banska Bystrica, Slovakia; National University of Singapore, Singapore; Suraj Eye Institute, India; Helen Keller International, Cameroon; Healis-Sekhsaria Institute for Public Health, India; CIBER en Epidemiología y Salud Pública, Spain; Jagiellonian University Medical College, Poland; Karolinska Institutet, Sweden; Pontificia Universidad Católica de Chile, Chile; Robert Koch Institute, Germany; University of Pharmacy and Medicine of Ho Chi Minh City, Vietnam; Hanoi Medical University, Vietnam; Universidad Centro-Occidental Lisandro Alvarado, Venezuela; Shanghai Jiao-Tong University School of Medicine, China; Kyushu University, Japan; Heartfile, Pakistan; National Research Council, Italy; Imperial College London, UK; University of Palermo, Italy; Eastern Mediterranean Public Health Network, Jordan; The Queen’s University of Belfast, UK; Universidad de Cuenca, Ecuador; Korea Centers for Disease Control and Prevention, South Korea; Kuwait Institute for Scientific Research, Kuwait; University of Vale do Rio dos Sinos, Brazil; National Food and Nutrition Institute, Poland; Ministry of Health, Malaysia; Istanbul University, Turkey; Pan American Health Organization, USA; University of Puerto Rico, Puerto Rico; Research Center for Prevention and Health, Denmark; MRC Lifecourse Epidemiology Unit, UK; University of Novi Sad, Serbia; Fundación Oftalmológica de Santander, Colombia; Aarhus University, Denmark; Institute for Social and Preventive Medicine, Switzerland; University of Coimbra, Portugal; Jagiellonian University Medical College, Poland; Cancer Prevention and Research Institute, Italy; University of Madison-Wisconsin, USA; Istituto Superiore di Sanità, Italy; Ruprecht-Karls-University of Heidelberg, Germany; University of Bari, Italy; University of Otago, New Zealand; Non-Communicable Diseases Research Center, Iran; Healis-Sekhsaria Institute for Public Health, India; University Medical Center Utrecht, Netherlands; Oswaldo Cruz Foundation Rene Rachou Research Institute, Brazil; Heart Institute (InCor), Brazil; University of Puerto Rico Medical Sciences Campus, Puerto Rico; Helmholtz Zentrum München, Germany; Non-Communicable Diseases Research Center, Iran; Vietnam National Heart Institute, Vietnam; Leibniz Institute for Prevention Research and Epidemiology—BIPS, Germany; University College London, UK; Federal Ministry of Health, Bosnia and Herzegovina; Cardiovascular Prevention Centre Udine, Italy; University Hospital of Pisa, Italy; University of New South Wales, Australia; The Cardinal Wyszynski Institute of Cardiology, Poland; The Cardinal Wyszynski Institute of Cardiology, Poland; Public Health Agency of Catalonia, Spain; Universiti Kebangsaan Malaysia, Malaysia; Institut Hospital del Mar d’Investigacions Mèdiques, Spain; Erasmus Medical Center Rotterdam, Netherlands; Alexander Technological Educational Institute, Greece; Madras Diabetes Research Foundation, India; Indian Council of Medical Research, India; University of Edinburgh, UK; Victor Babeş University of Medicine and Pharmacy, Romania; Tartu University Clinics, Estonia; Al-Quds University, Palestine; Alborz University of Medical Sciences, Iran; Ministry of Health, Vietnam; Institute of Public Health of Vojvodina, Serbia; Lithuanian University of Health Sciences, Lithuania; Institute of Epidemiology Disease Control and Research, Bangladesh; Turku University Hospital, Finland; Amrita Institute of Medical Sciences, India; National Institute of Epidemiology, India; India Diabetes Research Foundation, India; University of New South Wales, Australia; Institut Universitari d’Investigació en Atenció Primària Jordi Gol, Spain; University of Malaya, Malaysia; Karolinska Institutet, Sweden; University of Valencia, Spain; University of the Philippines, Philippines; Department of Health, Brazil; Imperial College London, UK; Health Center San Agustín, Spain; PharmAccess Foundation, Netherlands; Hospital Israelita Albert Einstein, Brazil; Instituto Nacional de Salud Pública, Mexico; University of Southampton, UK; Public Health Agency of Canada, Canada; Universidad Autónoma de Madrid, Spain; Canarian Health Service, Spain; Universidad Industrial de Santander, Colombia; Instituto Nacional de Salud Pública, Mexico; Mahidol University, Thailand; CIBEROBN, Spain; University of Eastern Finland, Finland; University of Gothenburg, Sweden; Fiji National University, Fiji; Institute for Clinical Effectiveness and Health Policy, Argentina; University of Zurich, Switzerland; University of Madeira, Portugal; Instituto Mexicano del Seguro Social, Mexico; Heart Institute (InCor), Brazil; Medical University of Gdansk, Poland; Singapore Eye Research Institute, Singapore; Sitaram Bhartia Institute of Science and Research, India; Faculty of Medicine of Tunis, Tunisia; French Institute for Health Surveillance, France; National Institute of Public Health, Mexico; National Institute for Health and Welfare, Finland; University of Helsinki, Finland; University of Brescia, Italy; National Institute of Health, Peru; Ministry of Health, Indonesia; Catalan Department of Health, Spain; University of Lisbon, Portugal; Institute of Preventive Medicine, Portugal; University of São Paulo, Brazil; University of Porto, Portugal; University of Lisbon, Portugal; Isfahan Cardiovascular Research Center, Iran; German Cancer Research Center, Germany; Research and Education Institute of Child Health, Cyprus; University of São Paulo, Brazil; Robert Koch Institute, Germany; Hospital Italiano de Buenos Aires, Argentina; Robert Koch Institute, Germany; Rigshospitalet, Denmark; Federal University of Santa Catarina, Brazil; University of Amsterdam Academic Medical Center, Netherlands; MRC North-West University, South Africa; Ministry of Health, Thailand; Norwegian University of Science and Technology, Norway; Lagos State University College of Medicine, Nigeria; Digestive Diseases Research Institute, Iran; National Research Centre for Preventive Medicine, Russia; Baker IDI Heart and Diabetes Institute, Australia; The University of Tokyo, Japan; Singapore Eye Research Institute, Singapore; Finnish Institute of Occupational Health, Finland; Singapore Eye Research Institute, Singapore; American University of Beirut, Lebanon; Federal University of Maranhao, Brazil; Federal University of Santa Catarina, Brazil; India Diabetes Research Foundation, India; St Vincent’s Hospital, Australia; University of New South Wales, Australia; Karolinska Institutet, Sweden; Medical University of Lodz, Poland; International Institute of Molecular and Cell Biology, Poland; London School of Hygiene & Tropical Medicine, UK; University of Oxford, UK; University of Amsterdam Academic Medical Center, Netherlands; The Chinese University of Hong Kong, China; University of Yaoundé 1, Cameroon; Umeå University, Sweden; Health Polytechnics Institute, Indonesia; University of Bari, Italy; Lund University, Sweden; University of Copenhagen, Denmark; University of Zagreb, Croatia; Institut Régional de Santé Publique, West Africa; University of Bordeaux, France; University of Leuven, Belgium; University of Ljubljana, Slovenia; INSERM, France; University of Zurich, Switzerland; Heart Foundation, Australia; Norwegian School of Sport Sciences, Norway; Bonn University, Germany; Emory University, USA; Sotiria Hospital, Greece; Hadassah University Medical Center, Israel; Helmholtz Zentrum München, Germany; Helmholtz Zentrum München, Germany; Lund University, Sweden; National Institute of Public Health-National Institute of Hygiene, Poland; Swansea University, UK; Federal University of São Paulo, Brazil; Fu Jen Catholic University, Taiwan; Uppsala University, Sweden; The Chinese University of Hong Kong, China; Centre for Research in Environmental Epidemiology, Spain; Mahidol University, Thailand; The University of Auckland, New Zealand; University of the Philippines, Philippines; National Food and Nutrition Institute, Poland; National University of Singapore, Singapore; University of Tartu, Estonia; Lithuanian University of Health Sciences, Lithuania; Research Centre for Prevention and Health, Denmark; Peking University Health Science Center, China; University of KwaZulu-Natal, South Africa; Peking University, China; Ministry of Health, Jordan; University of Southern Denmark, Denmark; National Institute of Health, Peru; The University of Adelaide, Australia; UNICEF, Cameroon; University of Leuven, Belgium; Research Centre for Prevention and Health, Denmark; Danish Cancer Society Research Centre, Denmark; National Institute for Health and Welfare, Finland; University of Southern Denmark, Denmark; Karadeniz Technical University, Turkey; Jagiellonian University Medical College, Poland; IB-SALUT Area de Salut de Menorca, Spain; Institut de Recherche pour le Développement, France; Hellenic Health Foundation, Greece; Harvard T H Chan School of Public Health, USA; deceased; University of Pharmacy and Medicine of Ho Chi Minh City, Vietnam; Government Medical College, India; Sefako Makgatho Health Science University, South Africa; The University of the West Indies, Jamaica; University of Eastern Finland, Finland; Dasman Diabetes Institute, Kuwait; Ministry of Health, New Zealand; Karolinska Institutet, Sweden; Hellenic Medical Association for Obesity, Greece; University of Bordeaux, France; Harvard T H Chan School of Public Health, USA; Meharry Medical College, USA; Medical University of Innsbruck, Austria; Dokuz Eylul University, Turkey; Pontificia Universidad Católica de Chile, Chile; University of Porto, Portugal; Harvard T H Chan School of Public Health, USA; University Medical Center Utrecht, Netherlands; Ghent University, Belgium; Hanoi Medical University, Vietnam; University of Amsterdam Academic Medical Center, Netherlands; Katholieke Universiteit Leuven, Belgium; Centro di Prevenzione Cardiovascolare Udine, Italy; Norwegian University of Science and Technology, Norway; Consejería de Sanidad Junta de Castilla y León, Spain; Universidade Federal de Minas Gerais, Brazil; University of Insubria, Italy; National Institute for Public Health and the Environment, Netherlands; Italian National Research Council, Italy; National Institute for Public Health and the Environment, Netherlands; Finnish Institute of Occupational Health, Finland; Imperial College London, UK; Universidad Miguel Hernandez, Spain; University of Eastern Finland, Finland; INSERM, France; Ministry of Health, Seychelles; Lausanne University Hospital, Switzerland; University of Eastern Finland, Finland; Centre for Research in Environmental Epidemiology, Spain; University of the Witwatersrand, South Africa; University of Strasbourg, France; University College Cork, Ireland; Institute for Medical Research, Malaysia; Public Health Agency of Canada, Canada; Xinjiang Medical University, China; Beijing Tongren Hospital, China; University College London, UK; University of Cambridge, UK; Ministry of Health, New Zealand; St George’s, University of London, UK; Medical University of Vienna, Austria; Universitas Indonesia, Indonesia; Medical University of Silesia, Poland; The University of the West Indies, Jamaica; Medical University Innsbruck, Austria; National Institute of Public Health-National Institute of Hygiene, Poland; Universiti Kebangsaan Malaysia, Malaysia; Duke-NUS Graduate Medical School, Singapore; The Chinese University of Hong Kong, China; University of Sydney, Australia; University of Oxford, UK; University of Manchester, UK; Shandong University of Traditional Chinese Medicine, China; Kailuan General Hospital, China; Institute of Food and Nutrition Development of Ministry of Agriculture, China; Capital Medical University, China; Mahidol University, Thailand; Fudan University, China; Chinese Center for Disease Control and Prevention, China; Ministry of Health, Turkey; University of Chinese Academy of Sciences, China; Cyprus University of Technology, Cyprus; Niigata University, Japan; Capital Medical University, China; The University of the West Indies, Jamaica; Ministry of Health, Malaysia; Universiti Teknologi MARA, Malaysia; University of Padova, Italy; Medical University of Gdansk, Poland; Duke University, USA; Peking University, China; Capital Medical University Beijing Anzhen Hospital, China; Chinese Center for Disease Control and Prevention, China; Singapore Eye Research Institute, Singapore; Chinese Center for Disease Control and Prevention, China; Inner Mongolia Medical University, China; Bispebjerg and Frederiksberg Hospitals, Denmark; Gorgas Memorial Institute of Public Health, Panama

## Abstract

**Background:**

Underweight and severe and morbid obesity are associated with highly elevated risks of adverse health outcomes. We estimated trends in mean body-mass index (BMI), which characterises its population distribution, and in the prevalences of a complete set of BMI categories for adults in all countries.

**Methods:**

We analysed, with use of a consistent protocol, population-based studies that had measured height and weight in adults aged 18 years and older. We applied a Bayesian hierarchical model to these data to estimate trends from 1975 to 2014 in mean BMI and in the prevalences of BMI categories (<18·5 kg/m^2^ [underweight], 18·5 kg/m^2^ to <20 kg/m^2^, 20 kg/m^2^ to <25 kg/m^2^, 25 kg/m^2^ to <30 kg/m^2^, 30 kg/m^2^ to <35 kg/m^2^, 35 kg/m^2^ to <40 kg/m^2^, ≥40 kg/m^2^ [morbid obesity]), by sex in 200 countries and territories, organised in 21 regions. We calculated the posterior probability of meeting the target of halting by 2025 the rise in obesity at its 2010 levels, if post-2000 trends continue.

**Findings:**

We used 1698 population-based data sources, with more than 19·2 million adult participants (9·9 million men and 9·3 million women) in 186 of 200 countries for which estimates were made. Global age-standardised mean BMI increased from 21·7 kg/m^2^ (95% credible interval 21·3–22·1) in 1975 to 24·2 kg/m^2^ (24·0–24·4) in 2014 in men, and from 22·1 kg/m^2^ (21·7–22·5) in 1975 to 24·4 kg/m^2^ (24·2–24·6) in 2014 in women. Regional mean BMIs in 2014 for men ranged from 21·4 kg/m^2^ in central Africa and south Asia to 29·2 kg/m^2^ (28·6–29·8) in Polynesia and Micronesia; for women the range was from 21·8 kg/m^2^ (21·4–22·3) in south Asia to 32·2 kg/m^2^ (31·5–32·8) in Polynesia and Micronesia. Over these four decades, age-standardised global prevalence of underweight decreased from 13·8% (10·5–17·4) to 8·8% (7·4–10·3) in men and from 14·6% (11·6–17·9) to 9·7% (8·3–11·1) in women. South Asia had the highest prevalence of underweight in 2014, 23·4% (17·8–29·2) in men and 24·0% (18·9–29·3) in women. Age-standardised prevalence of obesity increased from 3·2% (2·4–4·1) in 1975 to 10·8% (9·7–12·0) in 2014 in men, and from 6·4% (5·1–7·8) to 14·9% (13·6–16·1) in women. 2·3% (2·0–2·7) of the world’s men and 5·0% (4·4–5·6) of women were severely obese (ie, have BMI ≥35 kg/m^2^). Globally, prevalence of morbid obesity was 0·64% (0·46–0·86) in men and 1·6% (1·3–1·9) in women.

**Interpretation:**

If post-2000 trends continue, the probability of meeting the global obesity target is virtually zero. Rather, if these trends continue, by 2025, global obesity prevalence will reach 18% in men and surpass 21% in women; severe obesity will surpass 6% in men and 9% in women. Nonetheless, underweight remains prevalent in the world’s poorest regions, especially in south Asia.

**Funding:**

Wellcome Trust, Grand Challenges Canada.

## Introduction

High body-mass index (BMI) is an important risk factor for cardiovascular and kidney diseases, diabetes, some cancers, and musculoskeletal disorders.^1–7^ Concerns about the health and economic burden of increasing BMI have led to adiposity being included among the global non-communicable disease (NCD) targets, with a target of halting, by 2025, the rise in the prevalence of obesity at its 2010 level.^8,9^ Information on whether countries are on track to achieve this target is needed to support accountability towards the global NCD commitments.^10^

Two previous studies^11–13^ estimated global trends in the prevalence of overweight and obesity. However, the largest health benefits of weight management are achieved by shifting the population distribution of BMI. The only global report on mean BMI, which characterises distributional shifts, estimated trends to 2008,^11^ before the global target was agreed. Epidemiological studies have shown substantial risks in people with very high BMI—eg, severe (≥35 kg/m^2^) or morbid (≥40 kg/m^2^) obesity.^14^ Being underweight is also associated with increased risk of morbidity and mortality (ie, a so-called J-shaped association) and with adverse pregnancy outcomes.^4,6,15,16^ Very few analyses of trends in underweight,^17^ especially for men, and in severe and morbid obesity have been done. Finally, no information is available on the likelihood of individual countries or the world as a whole achieving the global obesity target.

We pooled population-based data to estimate trends from 1975 to 2014 in both mean BMI and in prevalence of BMI categories ranging from underweight to morbid obesity. We also estimated the probability of achieving the global obesity target.

## Methods

### Study design

We analysed population-based studies that had measured height and weight in adults aged 18 years and older with use of a consistent protocol. We estimated trends in mean BMI and prevalence of BMI categories (<18·5 kg/m^2^ [underweight], 18 · 5 kg/m^2^ to <20 kg/m^2^, 20 kg/m^2^ to <25 kg/m^2^, 25 kg/m^2^ to <30 kg/m^2^, 30 kg/m^2^ to <35 kg/m^2^, 35 kg/m^2^ to <40 kg/m^2^, and ≥40 kg/m^2^ [morbid obesity]) from 1975 to 2014, in 200 countries and territories. We report results for these categories, and for total obesity (BMI ≥30 kg/m^2^) and severe obesity (BMI ≥35 kg/m^2^). Countries and territories were organised into 21 regions, mostly on the basis of geography and national income (appendix pp 10, 11). The exception was a region consisting of high-income English-speaking countries because BMI and other cardiometabolic risk factors have similar trends in these countries, which can be distinct from other countries in their geographical region. Our analysis covered men and women aged 18 years and older, consistent with the Global Monitoring Framework for NCDs.^8^

Our study had two steps: first, we identified, accessed, and reanalysed population-based studies that had measured height and weight; then, we used a statistical model to estimate mean BMI and prevalences of BMI categories for all countries and years.

### Data sources

We used multiple routes for identifying and accessing data, including from publicly available sources and through requests to various national and international organisations, as described in the appendix (pp 2–5). We used data sources that were representative of a national, subnational, or community population and had measured height and weight. We did not use self-reported height and weight because they are subject to biases that vary by geography, time, age, sex, and socioeconomic characteristics.^18–20^ Because of these variations, present approaches to correcting self-reported data leave residual bias and error. Our data inclusion and exclusion criteria were designed to ensure population representativeness (appendix pp 2–5).

### Statistical analysis

The statistical method is described in a statistical paper^21^ and in the appendix of a previous paper.^22^ In summary, the model had a hierarchical structure in which estimates for each country and year were informed by the country and year’s own data, if available, and by data from other years in the same country and in other countries, especially those in the same region with data for similar time periods. The hierarchical structure shares information to a greater degree when data are non-existent or weakly informative (eg, have a small sample size or are not national), and to a lesser extent in data-rich countries and regions.

The model incorporated non-linear time trends and age patterns; national versus subnational and community representativeness; and whether data covered both rural and urban areas versus only one of them. The model also included covariates that help predict BMI, including national income (natural logarithm of per-person gross domestic product adjusted for inflation and purchasing power), proportion of population living in urban areas, mean number of years of education, and summary measures of availability of different food types for human consumption as described elsewhere.^23,24^ We also did an analysis without the use of covariates and compared the estimates with and without covariates. Estimates with and without covariates were virtually identical in most countries (appendix pp 147,148) with the exception of a few countries that had no data and whose covariates (eg, national income) differed from those of their region (eg, Brunei, Bermuda, and North Korea). We report estimates for the model with covariates because it had better fit to data, as measured by the deviance information criterion.

We analysed mean BMI and each prevalence of a BMI category separately. We rescaled the estimated prevalence of different categories so that their sum was 1·0 in each age, sex, country, and year. The mean scaling factor across draws was 1·05 for men and 1·07 for women—ie, the sum of each separately estimated prevalence was close to 1·0. Estimates for regions and the world were calculated as population-weighted means of the constituent country estimates by age group and sex. For presentation, we age-standardised each estimated mean and prevalence to the WHO standard population,^25^ by taking weighted means of age-sex-specific estimates, with use of age weights from the standard population. We tested how well our statistical model predicted mean BMI and the prevalence of each BMI category when a country-year did not have data as described in the appendix (pp 8,9), which showed that it performed very well in terms of its prediction validity.

We estimated mean change in BMI (absolute change for mean BMI and relative change for prevalence of BMI categories) over the 40 years of analysis, which we report as change per decade. We also report the posterior probability that an estimated increase or decrease in mean BMI or prevalence of a BMI category represented a truly increasing or decreasing trend. Additionally, we made separate estimates of change for pre-2000 and post-2000 years to assess whether the increasing recognition of adiposity as an “epidemic” in the 1990s,^26^ and the subsequent public health attention and response,^27,28^ might have slowed down its rise. Finally, we calculated the posterior probability of meeting the global obesity target if post-2000 trends continue.

### Role of the funding source

The funder of the study had no role in study design, data collection, data analysis, data interpretation, or writing of the report. MDC, JB, and Country and Regional Data Group members had full access to the data in the study and the corresponding author had final responsibility for the decision to submit for publication.

## Results

We accessed and used 1698 population-based data sources, with more than 19·2 million participants (9·9 million men and 9·3 million women) aged 18 years or older whose height and weight had been measured, in 186 of 200 countries for which estimates were made (appendix pp 143, 144); these 186 countries covered 99% of the world’s population. 159 countries had at least two data sources, which allowed more reliable trend estimates. 827 sources (49%) were national, 236 (14%) were subnational, and the remaining 635 (37%) were community-based (appendix pp 145, 146). The mean number of data sources per country varied between regions from 2·8 data sources in Polynesia and Micronesia to 34·7 data sources in high-income Asia Pacific. 525 data sources (31%) were from years before 1995 and another 1173 (69%) data sources from 1995 and later. 1314 (77%) sources had data on men and women, 144 (8%) only on men, and 240 (14%) only on women.

**Figure 1:**
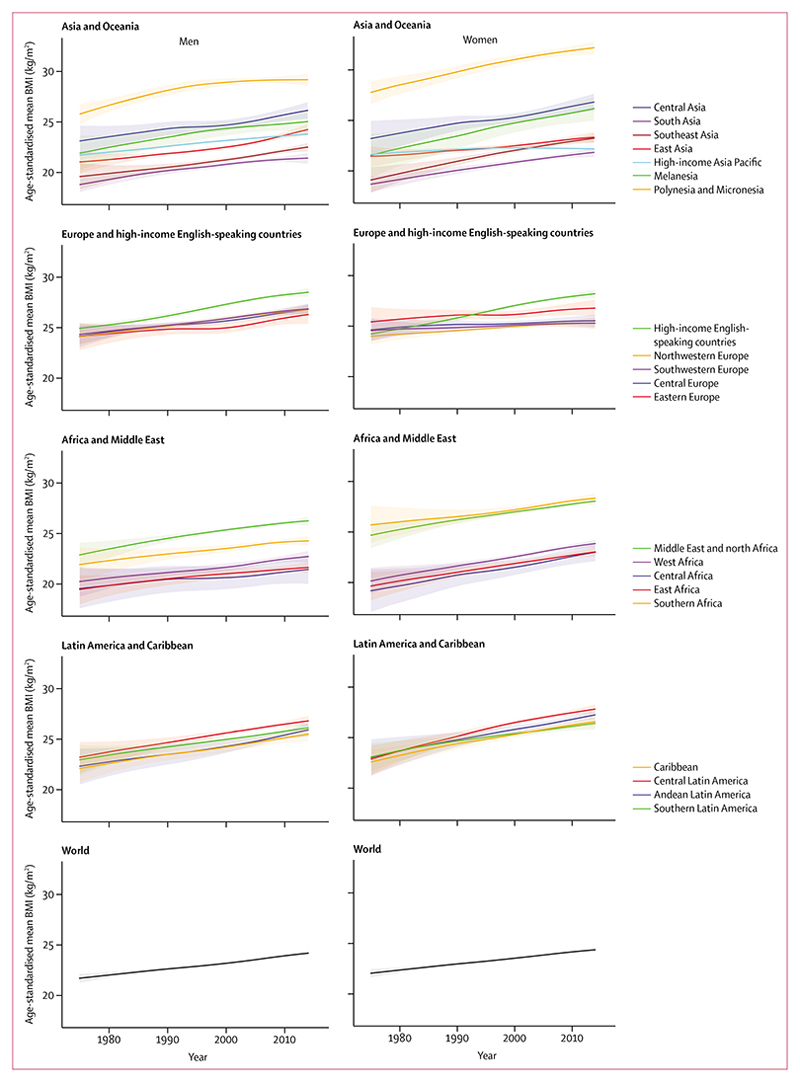
Trends in age-standardised mean BMI by sex and region Lighter colours are 95% credible intervals. See appendix (pp 155–355) for results by sex and country. BMI=body-mass index.

Global age-standardised mean BMI in men increased from 21·7 kg/m^2^ (95% CrI 21·3–22·1) in 1975 to 24·2 kg/m^2^ (24·0–24·4) in 2014, and in women from 22·1 kg/m^2^ (21·7–22·5) in 1975 to 24·4 kg/m^2^ (24·2–24·6) in 2014 (figure 1); the posterior probability that the observed trends were true increases was greater than 0·9999 for both sexes. The mean increases of 0·63 kg/m^2^ per decade (0·53–0·73) for men and 0·59 kg/m^2^ per decade (0·49–0·70) for women are equivalent to the world’s population having become on average more than 1·5 kg heavier each decade.

Regional mean BMI in 2014 in men ranged from 21·4 kg/m^2^ in central Africa and south Asia to 29·2 kg/m^2^ (95% CrI 28·6–29·8) in Polynesia and Micronesia (figure 1). In women, the range was from 21·8 kg/m^2^ (21·4–22·3) in south Asia to 32·2 kg/m^2^ (31·5–32·8) in Polynesia and Micronesia. Mean BMI was also high in men and women in high-income English-speaking countries, and in women in southern Africa and in the Middle East and north Africa.

The largest increase in men’s mean BMI occurred in high-income English-speaking countries (1·00 kg/m^2^ per decade; posterior probability >0·9999) and in women in central Latin America (1·27 kg/m^2^ per decade; posterior probability >0·9999). The increase in women’s mean BMI was also more than 1·00 kg/m^2^ per decade in Melanesia, Polynesia and Micronesia, high-income English-speaking countries, southeast Asia, Andean Latin America, and the Caribbean. Because of these trends, men and women in high-income English-speaking countries in 2014 had substantially higher BMIs than those in continental Europe, whereas in 1975 their BMI had been similar or lower, especially for women (figure 1). By contrast with these large increases, the rise in women’s mean BMI was less than 0·2 kg/m^2^ per decade in central Europe, southwestern Europe, and high-income Asia Pacific.

**figure 2:**
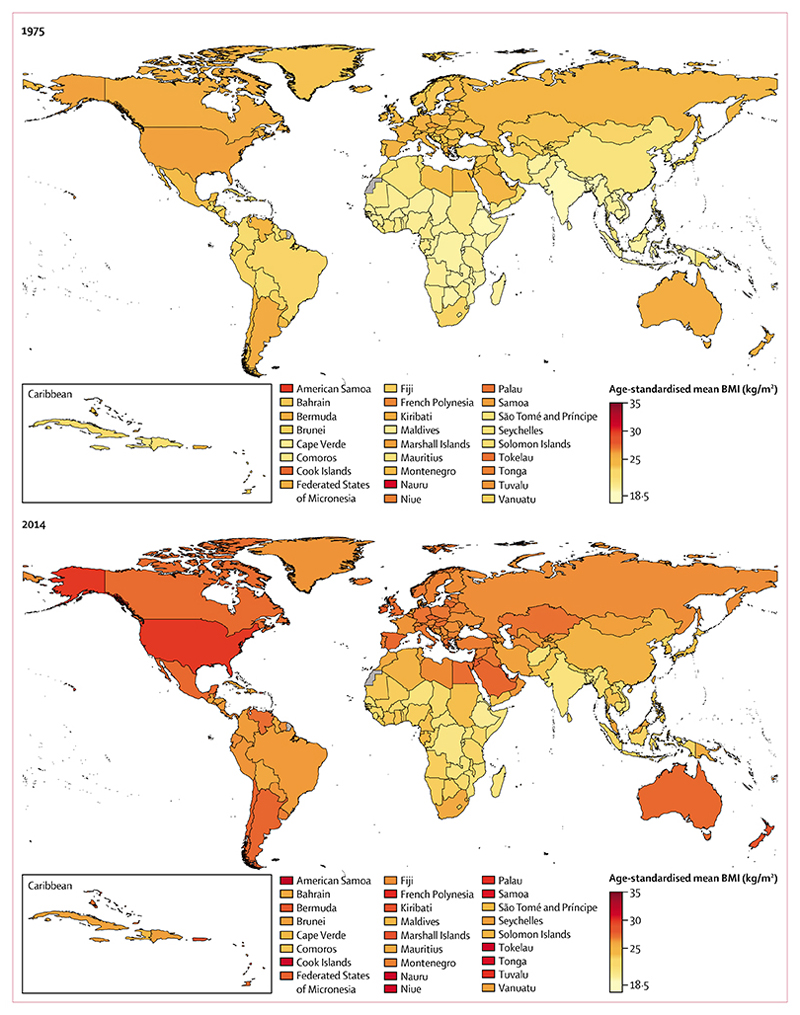
Age-standardised mean BMI in men by country in 1975 and 2014 See appendix (pp 56–64) for numerical results. BMI=body-mass index.

**figure 3:**
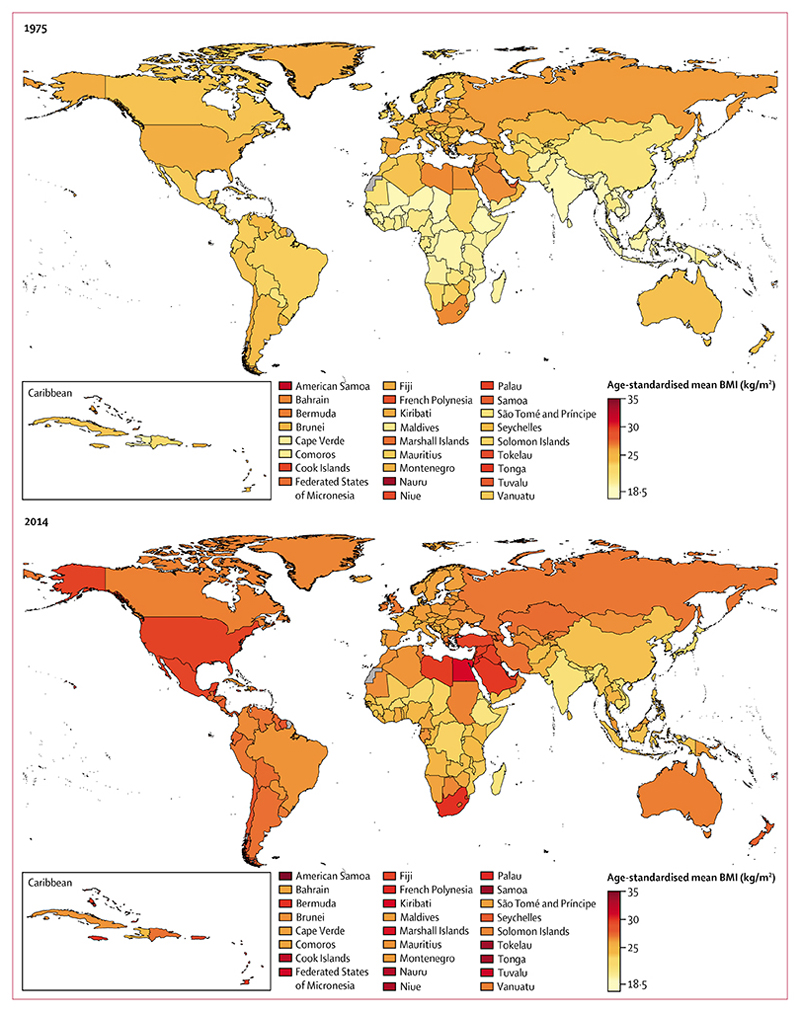
Age-standardised mean BMI in women by country in 1975 and 2014 See appendix (pp 56–64) for numerical results. BMI=body-mass index.

In 1975, age-standardised mean BMI was less than 19 kg/m^2^ in men in Timor-Leste, Burundi, India, Ethiopia, Vietnam, Rwanda, Eritrea, and Bangladesh (figure 2), and 17–18 kg/m^2^ in women in Bangladesh, Nepal, Timor-Leste, Burundi, Cambodia, and Vietnam (figure 3). In the same year, men and women in Nauru and women in American Samoa already had mean BMIs of more than 30 kg/m^2^.^29,30^ By 2014, age-standardised mean BMI was more than 20·0 kg/m^2^ in men and more than 20·7 kg/m^2^ in women in every country, with Ethiopia, Eritrea, and Timor-Leste having the lowest BMIs for both sexes. At the same time, in American Samoa, the age-standardised mean BMIs were 32·2 kg/m^2^ (95% CrI 30·5–33·7) for men and 34·8 kg/m^2^ (33·2–36·3) for women, with mean BMI also more than 30 kg/m^2^ in both sexes in some other islands in Polynesia and Micronesia, and in women in some countries in the Middle East and north Africa (eg, Egypt and Kuwait) and the Caribbean.

**Figure 4:**
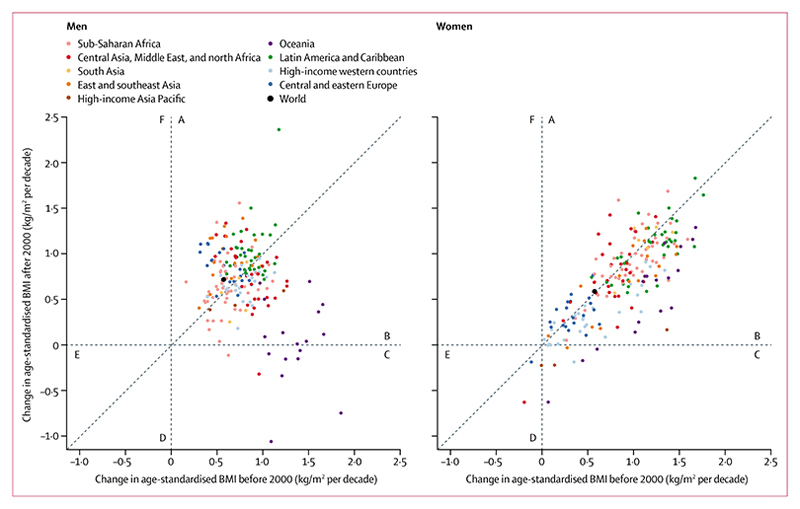
Comparison of mean change in age-standardised mean BMI before and after the year 2000 Each point represents one country. (A) countries in which mean BMI increased more rapidly after 2000 than it had before 2000. (B) countries in which mean BMI increased more slowly after 2000 than it had before 2000. (C) countries in which mean BMI increased before 2000 but decreased after 2000. (D) countries in which mean BMI decreased more rapidly after 2000 than it had before 2000. (E) countries in which mean BMI decreased more slowly after 2000 than it had before 2000. (F) countries in which BMI decreased before 2000 but increased after 2000. BMI=body-mass index.

From 1975 to 2014, trends in men’s BMI ranged from virtually flat in Nauru (albeit at a very high level), North Korea, and several countries in sub-Saharan Africa, to an increase of more than 1·5 kg/m^2^ per decade. Similarly, women’s BMI did not change in Bahrain and Nauru (both starting at high BMIs), Singapore, Japan, North Korea, and several European countries, but increased by more than 1·5 kg/m^2^ per decade in some countries. BMI increased more slowly after the year 2000 than in the preceding 25 years in Oceania and in most high-income countries for both sexes, and for women in most countries in Latin America and the Caribbean (figure 4). By contrast, the post-2000 increase was steeper than pre-2000 in men in central and eastern Europe, east and southeast Asia, and most countries in Latin America and the Caribbean. In other regions, increases in BMI before and after 2000 were similar or they had a mixture of slow-down and acceleration. The standard deviation of BMI also increased from 1975 to 2014 (appendix pp 149, 150), which contributed to an increase in the prevalence of people at either or both extremes of BMI.

Mean BMI in 2014 varied more across countries in women than it did in men. For example, the difference in women’s mean BMI between American Samoa (the country with the highest mean BMI) and Timor-Leste (the country with the lowest mean BMI) was 14·1 kg/m^2^ in 2014, which is equivalent to about a 35 kg difference in the mean weight per person, whereas in men, the difference in mean BMI was 12·1 kg/m^2^, which is also equivalent to about a 35 kg difference in the mean weight per person (because men tend to be taller). Although male and female BMIs were correlated across countries, women on average had higher BMI than did men in 141 countries in 2014 (appendix pp 151, 152). The main exceptions from this sex pattern were countries in Europe and in high-income Asia Pacific and English-speaking countries. Changes in male and female BMI were weakly correlated across countries.

**Figure 5:**
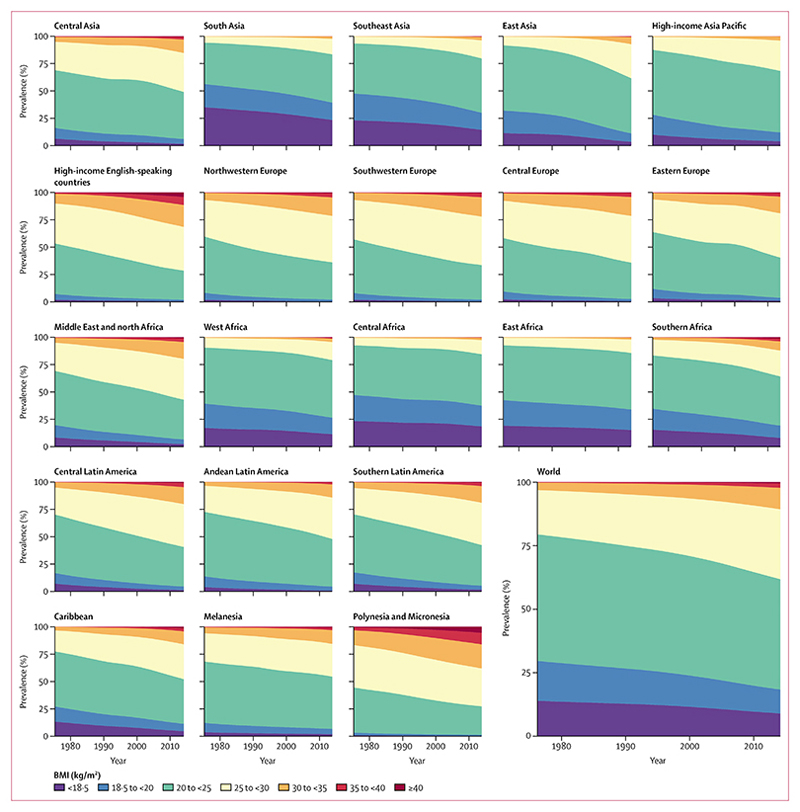
Trends in age-standardised prevalence of BMI categories in men by region See appendix (pp 155–355) for results by country. BMI=body-mass index.

**Figure 6:**
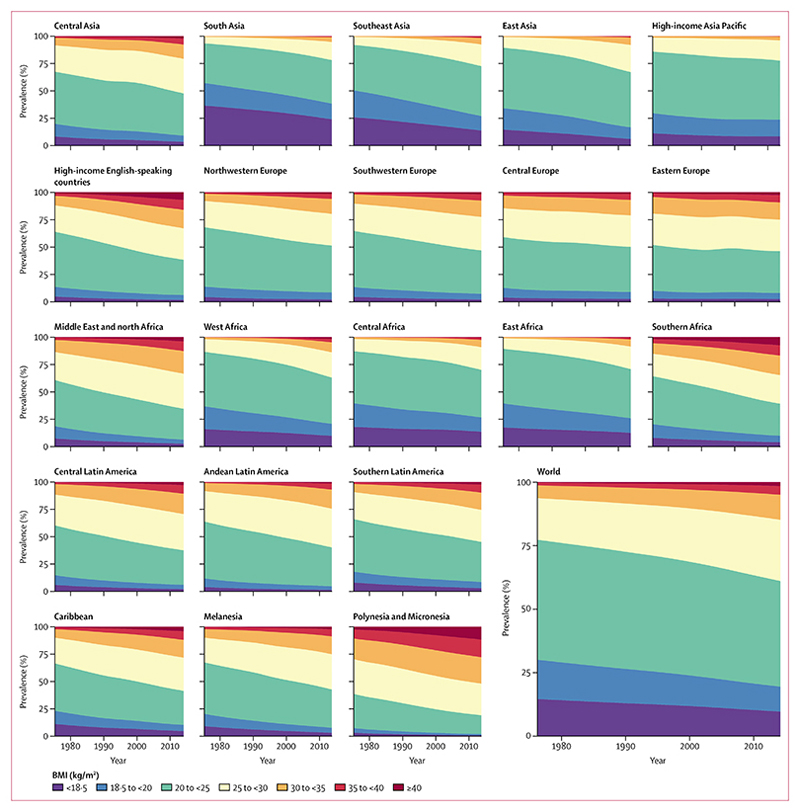
Trends in age-standardised prevalence of BMI categories in women by region See appendix (pp 155–355) for results by country. BMI=body-mass index.

From 1975 to 2014, global age-standardised prevalence of underweight (BMI <18 · 5 kg/m^2^) decreased from 13·8% (95% CrI 10·5–17·4) to 8·8% (7·4–10·3) in men (figure 5) and from 14·6% (11·6–17·9) to 9·7% (8·3–11·1) in women (figure 6). Compared with the fall in underweight, prevalence of obesity (BMI ≥30 kg/m^2^) increased by a larger amount—from 3·2% (2·4–4·1) in 1975 to 10·8% (9·7–12·0) in 2014 in men, and from 6·4% (5·1–7·8) to 14·9% (13·6–16·1) in women. Prevalence of obesity surpassed that of underweight in 2004 in women and in 2011 in men. 2·3% (2·0–2·7) of the world’s men and 5·0% (4·4–5·6) of women were severely obese in 2014. The global prevalence of morbid obesity (BMI ≥40 kg/m^2^) was 0·64% (0·46–0·86) in men and 1·6% (1·3–1·9) in women in 2014.

Age-standardised underweight prevalence in south Asia, where it is most common, decreased from more than 35% in both sexes in 1975 to 23·4% (95% CrI 17·8–29·2) in men and 24·0% (18·9–29·3) in women in 2014 (figures 5, 6). Underweight prevalence also remained higher than 12% in women and higher than 15% in men in central and east Africa in 2014, despite some reductions. At the other extreme, more than 38% of men and more than 50% of women in Polynesia and Micronesia were obese in 2014. Obesity prevalence also surpassed 30% in men and women in high-income English-speaking countries, and in women in southern Africa and in the Middle East and north Africa.

**Figure 7:**
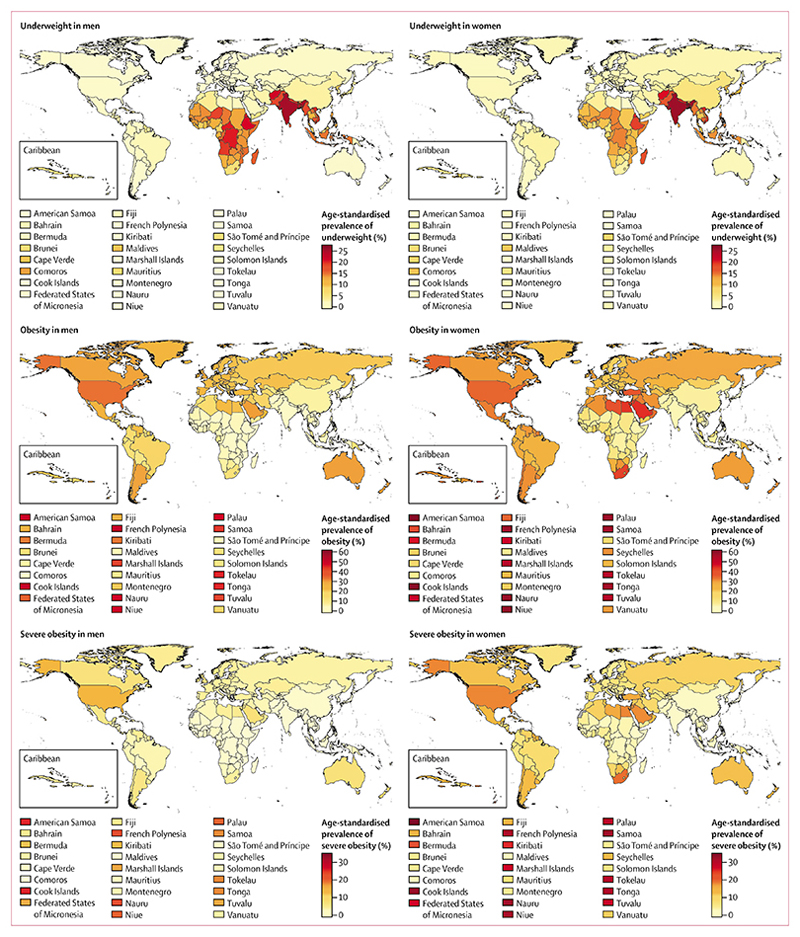
Age-standardised prevalence of underweight, obesity, and severe obesity by sex and country in 2014 Underweight (BMI <18·5 kg/m^2^); obesity (BMI ≥30 kg/m^2^); and severe obesity (BMI ≥35 kg/m^2^). See appendix (pp 65–107) for numerical results for all BMI ranges. BMI=body-mass index.

Age-standardised prevalence of underweight in 2014 was less than 1% in men in 68 countries and in women in 11 countries (figure 7). At the other extreme, more than 20% of men in India, Bangladesh, Timor-Leste, Afghanistan, Eritrea, and Ethiopia, and a quarter or more of women in Bangladesh and India are still underweight. In 1975, the proportion had been as high as 37% in Indian and Bangladeshi women.

In 2014, more men were obese than underweight in 136 (68%) of 200 countries; in 113 of these countries, more men were severely obese than underweight. For women, obesity surpassed underweight in 165 (83%) countries and severe obesity surpassed underweight in 135 countries. Obesity prevalence was less than 1% in men in Burundi and Timor-Leste and 1–2% in another 15 countries in central, east, and west Africa and in south and southeast Asia. The lowest prevalences in women were in Timor-Leste, Japan,Vietnam, North Korea, Cambodia, Laos, and Bangladesh, all less than 5%. At the other extreme, more than 45% of men in six island nations in Polynesia and Micronesia, and more than 50% of women in 11 such island nations were obese. The prevalence of obesity in women in several Caribbean and Middle Eastern countries was 40–50%. Severe obesity surpassed 20% in men and 30% in women in some Polynesian and Micronesian islands, reaching 33·4% (95% CrI 23·6–43·5) in American Samoa in 2014. More than 15% of women in Nauru and American Samoa were morbidly obese.

**Figure 8:**
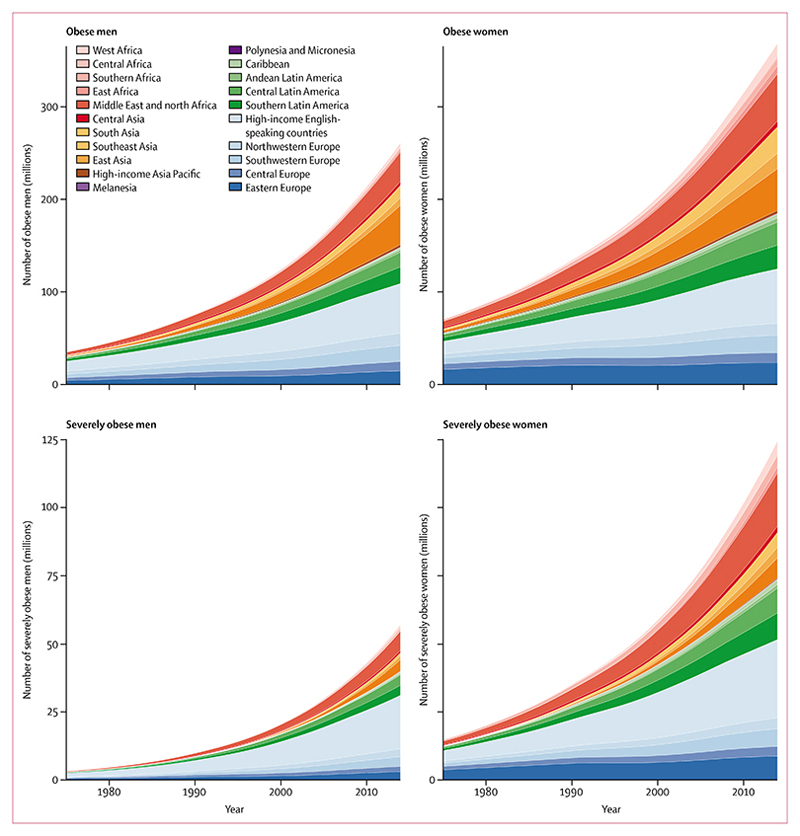
Trends in the number of obese and severely obese people by region A person is obese if they have a body-mass index (BMI) of 30 kg/m^2^ or higher, or is severely obese if they have a BMI of 35 kg/m^2^ or higher.

In 2014, about 266 million men (95% CrI 240–295 million) and 375 million women (344–407 million) were obese in the world, compared with 34 million men (26–44 million) and 71 million women (57–87 million) in 1975 (figure 8). 58 million (49–68 million) of these men and 126 million (112–141 million) of these women were severely obese in 2014. 18·4% of the world’s obese adults (118 million) lived in high-income English-speaking countries and these countries contained an even larger share of the world’s severely obese people (27·1%; 50 million), followed by 13·9% (26 million) in the Middle East and north Africa.

**Figure 9:**
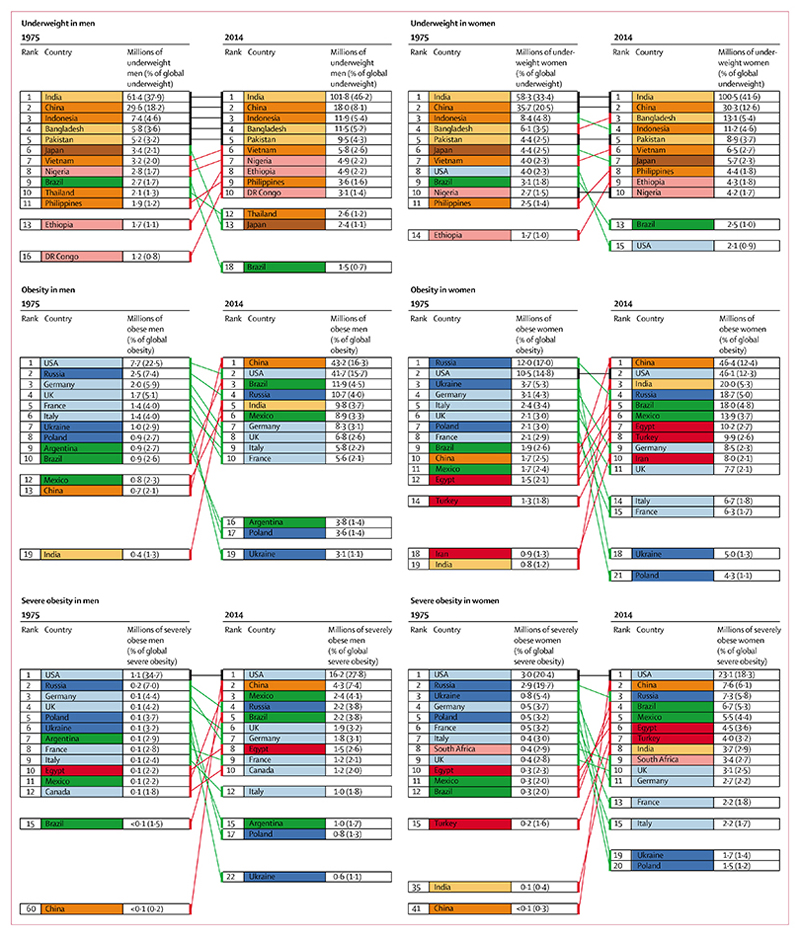
Ten countries with the largest number of underweight, obese, and severely obese men and women in 1975 and 2014 Colours for each country indicate its region, using the same colour scheme as in figure 4. Underweight (BMI <18·5 kg/m^2^); obesity (BMI ≥30 kg/m^2^); and severe obesity (BMI ≥35 kg/m^2^). BMI=body-mass index.

Countries where large numbers of underweight people lived in 1975 and in 2014 were mostly large countries in Asia and sub-Saharan Africa, with an increasing share of underweight people living in south Asia over time (figure 9). By contrast with this stability of underweight geography, countries with the largest number of obese and severely obese people changed over these four decades, with more middle-income countries joining the USA, especially for women. In 2014, slightly more obese men and women lived in China than in the USA, and even for severe obesity, China moved from 60th place for men and 41st place for women in 1975, to 2nd rank for both men and women in 2014. Nonetheless, more than one in four severely obese men and almost one in five severely obese women in the world still live in the USA.

If post-2000 trends continue, every country has a less than 50% probability of meeting the global obesity target, with Nauru having the highest probability of about 45% (appendix pp 153, 154). The probability of achieving the target is less than 10% for men in 194 countries, and for women in 174 countries. At the global level, the probability of meeting the target is virtually zero. Rather, if present trends continue, by 2025, global obesity prevalence will reach 18% in men and surpass 21% in women; severe obesity will surpass 9% in women and 6% in men, and will be larger than the projected prevalence of underweight in women.

## Discussion

Over the past four decades, we have transitioned from a world in which underweight prevalence was more than double that of obesity, to one in which more people are obese than underweight, both globally and in all regions except parts of sub-Saharan Africa and Asia. The rate of increase in BMI since 2000 has been slower than in the preceding decades in high-income countries, where adiposity became an explicit public health concern around this time,^27,28^ and in some middle-income countries. However, because the rate of BMI increase has accelerated in some other regions, the global increase in BMI has not slowed down. If post-2000 trends continue, not only will the world not meet the global target for halting the increase in obesity, but also severe obesity will surpass underweight in women by 2025. Nonetheless, underweight remains a public health problem in south Asia and central and east Africa.

We estimated a slightly larger increase in mean BMI since 1980 than Finucane and colleagues did,^11^ especially in men, because our estimates for 1980 were lower, globally and in most regions; this difference might be because our study included substantially more data, from a larger number of countries. Our global estimates of overweight prevalence are similar to those reported by Stevens and colleagues^13^ for 2008, and by Ng and colleagues for 2013.^12^ Our estimates for obesity for the same years are slightly lower than those of Stevens and colleagues and slightly higher than those of Ng and colleagues. Furthermore, we estimated a lower prevalence of obesity for 1980 than Ng and colleagues had, which means we have attributed a larger role to the rise over the past few decades for the present extent of obesity. Differences between our study and that of Ng and colleagues were greater at the regional level; for example, our estimates for obesity prevalence in men in south Asia and central, east, and west Africa were less than half of those by Ng and colleagues. None of these previous works had estimated underweight or severe and morbid obesity, which are important clinical and public health outcomes.

The strengths of our study include its unique scope of making consistent estimates of mean BMI and the prevalence of all BMI categories with clinical and public health relevance, including the first-ever estimates of underweight and severe and morbid obesity. These estimates helped reveal the details of the transition from underweight to overweight and obesity throughout the world. We also reported on the probability of each country meeting the global obesity target. We put great emphasis on data quality and used only population-based data sources that had measured height and weight to avoid the bias in self-reported data. Characteristics and quality of data sources were verified by Collaborating Group members (appendix pp 2–5). Data were analysed according to a common protocol to obtain the required mean and prevalence by age and sex, which in turn minimised reliance on models for filling such gaps, as done in previous studies.^11–13^ Finally, we pooled data using a statistical model designed to take into account the epidemiological features of outcomes such as BMI, and one that used all available data while giving more weight to national data than subnational and community studies.

Despite our efforts in identifying and accessing countrylevel data, some countries had few data sources, especially those in Polynesia and Micronesia, the Caribbean, and central Asia. Additionally, only 42% of sources included people older than 70 years. In view of ageing trends throughout the world, older people should be included in health and nutrition surveys, which have traditionally focused on childbearing ages. Even measured height and weight data can have error depending on how closely measurement protocols are followed. Although data held by Collaborating Group members were analysed to provide all needed details by sex and age group and BMI level, individual participant data could not be accessed for 19 · 4% of data used in our analysis, hence conversions across categories were still needed; nonetheless, the conversion regressions had high predictive accuracy (appendix pp 41–55). A novel component of our study is that we estimated the prevalences of a complete set of BMI categories, but the uncertainty intervals for BMIs of 30 kg/m^2^ or more and 35 kg/m^2^ or more, prevalences that span more than one of the analysed categories, could be affected by the fact that we combined posterior distributions across Bayesian models. We did not estimate trends in measures of adiposity other than BMI, such as waist circumference and waist-to-hip ratio, because these were measured in less than half of all the data sources and their measurement became more common after the 1980s. A systematic review^31^ of epidemiological studies reported that, taken together, studies that had measured BMI and either waist circumference or waist-to-hip ratio do not show that any of the measures of adiposity have “superior discriminatory capability” of adverse cardiometabolic outcomes; any reported difference was “too small to be of any clinical relevance”. We did not analyse children and adolescents for two reasons. First, because childhood and adolescence is a period of rapid growth, BMI cutoffs used to define underweight, overweight, and obesity for children and adolescents are different from those for adults, and vary by age and sex.^32^ Second, time trends in children’s and adolescents’ obesity are different from those of adults.^33^

Our results have several implications. First, the global focus on the obesity epidemic has largely overshadowed the persistence of underweight in some countries. Our results show the need to address the remaining underweight problem and by doing so reduce risks to pregnant women and their newborn infants,^15^ mortality from tuberculosis and other respiratory diseases,^34^ and possibly all-cause mortality, which has a J-shaped association.^2,3^ To address this problem will require social and food policies that enhance food security in poor households, but also avoid overconsumption of processed carbohydrates and other unhealthy foods. Second, although adiposity has been consistently shown to be an independent risk factor for several NCDs in individuallevel epidemiological studies, at the population level, the effect of rising BMI on the course of mortality reduction has so far been somewhat small in high-income countries,^35,36^ possibly because pharmacological treatment has helped reduce blood pressure and serum cholesterol and manage diabetes complications, which are mediators of the effects of BMI on cardiovascular diseases. In low-income countries, where health systems might not have the capacity to identify and treat hypertension, dyslipidaemia, and diabetes, adiposity might have a larger effect on population health. Furthermore, we have shown that some high-income and middle-income regions are now facing an epidemic of severe obesity. Even antihypertensive drugs, statins, and glucose lowering drugs will not be able to fully address the hazards of such high BMI levels,^7^ and bariatric surgery might be the most effective intervention for weight loss and disease prevention and remission.^37^ However, long-term health outcomes of bariatric surgery are largely unknown and it is not accessible to most people in low-income and middle-income countries because of financial and health system barriers.

Present interventions and policies have not been able to stop the rise in BMI in most countries.^38–40^ The global NCD target on obesity, although ambitious in view of past trends, has engendered a new look at policies that could slow down and stop the worldwide increase in BMI.^40–42^ To avoid an epidemic of severe obesity, the next step must be to implement these policies, and to systematically assess their effect.^43^

## Supplementary Material

Appendix

## Research in context

### Evidence before this study

We searched MEDLINE (via PubMed) for manuscripts published in any language between Jan 1, 1950, and March 12, 2013, using the search terms “body size”[mh:noexp] OR “body height”[mh:noexp] OR “body weight”[mh:noexp] OR “birth weight”[mh:noexp] OR “overweight”[mh:noexp] OR “obesity”[mh] OR “thinness”[mh:noexp] OR “Waist-Hip Ratio”[mh:noexp] or “Waist Circumference”[mh:noexp] or “body mass index” [mh:noexp]) AND (“Humans”[mh]) AND(“1950”[PDAT] : “2013”[PDAT]) AND (“Health Surveys”[mh] OR “Epidemiological Monitoring”[mh] OR “Prevalence”[mh]) NOT Comment[ptyp] NOT Case Reports[ptyp]. Articles were screened according to the inclusion and exclusion criteria described in the appendix (pp 2–5).

The only global study on trends in mean body-mass index (BMI), which characterises shifts in the population distribution of BMI, reported trends to 2008 (before the global target on obesity was agreed) and no recent data are available. Two previous studies estimated global trends in the prevalence of overweight and obesity. Neither study reported trends in underweight, which is associated with increased risk of morbidity, mortality, and adverse pregnancy outcomes, or in high levels of BMI (eg, ≥35 or ≥40 kg/m^2^), which are associated with substantial risks of many non-communicable diseases.

### Added value of this study

This study provides the longest and most complete picture of trends in adult BMI, including, for the first time, in underweight and severe and morbid obesity, which are of enormous clinical and public health interest. We were able to robustly depict this rich picture by reanalysing and pooling hundreds of population-based sources with measurements of height and weight according to a common protocol. We also systematically projected recent trends into the future, and assessed the probability of the global obesity target being achieved.

### Implications of all the available evidence

The world has transitioned from an era when underweight prevalence was more than double that of obesity, to one in which more people are obese than underweight. However, underweight remains a public health problem in the world’s poorest regions—namely south Asia and central and east Africa. If present trends continue, not only will the world not meet the global obesity target, but severe obesity will also surpass underweight in women by 2025.
