# Supplementary material for: Trends in adult body-mass index in 200 countries from 1975 to 2014: a pooled analysis of 1698 population-based measurement studies with 19·2 million participants
Source: Lancet. Author manuscript; Available in PMC 2023 Sep 28. (PMC7615134; doi:10.1016/S0140-6736(16)30054-X)
Supplement: Appendix [file EMS187842-supplement-Appendix.pdf]

THE LANCET

Supplementary appendix

This appendix formed part of the original submission and has been peer reviewed. We post it as supplied by the authors.

Supplement to: NCD Risk Factor Collaboration (NCD-RisC). Trends in adult body-mass index in 200 countries from 1975 to 2014: a pooled analysis of 1698 population-based measurement studies with 19·2 million participants. *Lancet* 2016; **387:** 1377–96.

Appendix

Trends in adult body-mass index in 200 countries from 1975 to 2014: a pooled analysis of 1698 population-based measurement studies with 19·2 million participants

NCD Risk Factor Collaboration (NCD-RisC)

Table of contents

Appendix 1: Data sources 2
Appendix 2: Converting among mean BMI and prevalences of different BMI categories 6
Appendix 3: Validation of statistical model 8
Appendix Table 1: List of analysis regions and "super-regions", and countries in each region. . 10
Appendix Table 2: Data sources used in the analysis. 12
Appendix Table 3: Coefficients of regressions to estimate the prevalences of different BMI categories when information was available on mean BMI. 41
Appendix Table 4: Age-standardised mean BMI by sex and country in 1975, 1985, 1995, 2005, and 2014. 56
Appendix Table 5: Age-standardised prevalences of BMI <18.5 kg/m^2, 18.5 to <20 kg/m^2, 20 to <25 kg/m^2, 25 to <30 kg/m^2, 30 to <35 kg/m^2, 35 to <40 kg/m^2, ≥40 kg/m^2 by sex and country in 1975, 1985, 1995, 2005, and 2014. 65
Appendix Table 6: Results of model validation.. 108
Appendix Figure 1: Flowchart of secondary search for data sources. 141
Appendix Figure 2: Number of data sources used in the analysis, by country. 143
Appendix Figure 3: Number of data sources by region and year. 145
Appendix Figure 4: Comparison of age-standardised mean BMI estimated using models with and without covariates. 147
Appendix Figure 5: Standard deviation (SD) of BMI in 1975 vs. 2014, for ages 20-24 years, 45-49 years and 70-74 years... 149
Appendix Figure 6: Male vs. female age standardised mean BMI in 1975 and 2014, and change from 1975 to 2014.. 151
Appendix Figure 7: Probability of achieving the target of halting the rise of obesity compared to its 2010 levels by country if post-2000 trends continue. 153
Appendix Figure 8: Trends in age-standardised mean BMI and prevalences of BMI categories by sex and country. 155

Appendix 1: Data sources

Data inclusion and exclusion

Data sources were included in NCD-RisC database if:

- measured data on height, weight, waist circumference, or hip circumference were available;
- study participants were five years of age and older;
- data were collected using a probabilistic sampling method with a defined sampling frame;
- data were representative of the general population at the national, subnational, or community level;
- data were collected in or after 1950;
- data were from the countries and territories listed in Appendix Table 1.

We excluded all self-reported data because they are subject to bias. We also excluded data sources on population subgroups whose anthropometric status may differ systematically from the general population, including:

- studies that had included or excluded people based on their health status or cardiovascular risk;
- ethnic minorities;
- specific educational, occupational, or socioeconomic subgroups of the population; and
- those recruited through health facilities, with the exception noted below.

We used school-based data in countries where secondary school enrolment was 70% or higher, and used data whose sampling frame was health insurance schemes in countries where at least 80% of the population were insured. In addition, we used data collected through general practice and primary care clinics in high-income countries with universal insurance, because in these

settings contact with the primary care systems tends to be at least as good as response rates for population-based surveys. No studies were excluded based on the level of BMI. In this paper, we used BMI data on data from the NCD-RisC database for years 1975 through 2014, and among participants aged 18 years and older.

Primary data access

We used multiple routes for identifying and accessing data. We accessed publicly available population-based multi-country and national measurement surveys (e.g., Demographic and Health Surveys, and surveys identified via the Inter-University Consortium for Political and Social Research and European Health Interview & Health Examination Surveys Database) as well as the World Health Organization (WHO) STEPwise approach to Surveillance (STEPS) surveys. We requested, via WHO and its regional offices, from ministries of health and other national health agencies to identify and access population-based surveys. Requests were also sent via the World Heart Federation to its national partners. We made a similar request to the NCD Risk Factor Collaboration (NCD-RisC), a worldwide network of health researchers and practitioners working on NCD risk factors. To identify major sources not accessed through the above routes, we searched and reviewed published studies, as detailed below, and invited all eligible studies to join NCD-RisC.

Anonymised individual record data from sources included in NCD-RisC were re-analysed by the Pooling and Writing Group or by data holders according to a common protocol. All re-analysed data sources included mean BMI and prevalences for the BMI categories listed in Methods section of the main paper, in standard age groups (18 years, 19 years, 20-29 years, followed by

10 year age groups and 80+ years), as well as sample sizes and standard errors. Before calculating means and prevalences, we dropped participants with implausible BMI levels, defined as BMI < 10 kg/m^2 or BMI > 80 kg/m^2 (<0.2% of all subjects). All analyses incorporated appropriate sample weights and complex survey design when applicable. To ensure summaries were prepared according to the study protocol, the Pooling and Writing Group provided computer code to NCD-RisC members who requested assistance. We also recorded information about the study population, period of measurement, sampling approach, and measurement methods. This information was used to establish that each data source was population-based, and to assess whether it covered the whole country, multiple subnational regions, or one or a small number of communities, and whether it was rural, urban, or combined. All submitted data were checked by at least two independent members of the Pooling and Writing Group. Questions and clarifications were discussed with the Collaborating Group members and resolved before data were incorporated in the database.

Finally, we obtained data not accessed through the above routes by extracting from published reports all additional national health surveys identified through the above-described strategies, three subnational STEPS surveys, and six MONICA sites. We also extracted data compiled for a previous global pooling.[11] These sources had information on only some of our quantities of interest, e.g., only on mean and/or some prevalences. We used data that had all of the relevant quantities of interest (mean BMI as well as all the above-mentioned prevalences) to develop regression equations for predicting the missing metrics from those available. These regression equations are described in Appendix 2 and their coefficients are provided in Appendix Table 3.

We identified duplicate data sources by comparing studies from the same country and year. Additionally, NCD-RisC members received the list of all data sources in the database and were asked to ensure that the included data from their country met the inclusion criteria and that there were no duplicates. Data sources used in the analysis are listed in Appendix Table 2.

Secondary search for additional data sources

To identify any major sources not accessed through the above routes, we searched Medline (via PubMed) for articles published between 1st January 1950 and 12th March 2013 using the search terms "body size"[mh:noexp] OR "body height"[mh:noexp] OR "body weight"[mh:noexp] OR "birth weight"[mh:noexp] OR "overweight"[mh:noexp] OR "obesity"[mh] OR "thinness"[mh:noexp] OR "Waist-Hip Ratio"[mh:noexp] or "Waist Circumference"[mh:noexp] or "body mass index" [mh:noexp]) AND ("Humans"[mh]) AND("1950"[PDAT] : "2013"[PDAT]) AND ("Health Surveys"[mh] OR "Epidemiological Monitoring"[mh] OR "Prevalence"[mh]) NOT Comment[ptyp] NOT Case Reports[ptyp]. Articles were screened according to the inclusion and exclusion criteria described above. The number of articles identified and retained is summarised in Appendix Figure 1. As described above, we contacted the corresponding authors of all eligible studies and invited them to join NCD-RisC. We did similar searches for other cardio-metabolic risk factors including blood pressure, serum cholesterol, and blood glucose. All eligible studies were invited to join NCD-RisC and were requested to analyse data on all cardio-metabolic risk factors.

Appendix 2: Converting among mean BMI and prevalences of different BMI categories

As described in the main text, we estimated mean BMI as well as prevalences of the following BMI categories: <18.5 kg/m^2, 18.5 to <20 kg/m^2, 20 to <25 kg/m^2, 25 to <30 kg/m^2, 30 to <35 kg/m^2, 35 to <40 kg/m^2, ≥40 kg/m^2. In 19.4% of our data points, those extracted from published studies or from a previous global pooling, data were available for a subset of these outcomes, and/or were available for prevalences of other BMI ranges. In order to use these data, we developed conversion (or cross-walking) regressions to estimate all of our outcome variables from the available data. All sources of uncertainty in the conversion, including the sampling uncertainty of the original data, the uncertainty of the regression coefficients and random effects, and the regression residuals, were carried forward by using repeated draws from their respective distributions. We accounted for the correlation among the uncertainties of regression coefficients and random effects, by drawing from their joint posterior distribution.

Estimating prevalences for BMI categories from mean BMI

15.9% of all data used in the analysis had information on mean BMI but not on some or all of the prevalence outcomes. For these data, the cross-walking regressions used information on mean to estimate the missing prevalences. The dependent variable in each of the seven regressions for estimating prevalences of BMI categories from mean BMI was prevalence, fitted using generalised linear mixed models with a probit link; the independent variables were mean BMI (transformed as detailed in Appendix Table 3 to provide better fit to data), age, sex, year of data, and natural logarithm of per-capita gross domestic product (GDP). There were also statistical interactions between mean BMI and age and sex. The regression also included random effects for regions, to allow for the possibility that at each mean BMI level, prevalences of various BMI

categories may vary across regions. The coefficients of these regressions were estimated from the 80.6% of data for which all outcomes of interest were available, excluding data points for which there were fewer than 25 subjects. The regression coefficients and number of data points used in estimating the coefficients are shown in Appendix Table 3.

Estimating mean BMI from prevalences of various BMI ranges

3.5% of our data had information on prevalences of only selected BMI categories, some of which overlapped with our outcomes of interest (e.g., BMI of 25 to <30 kg/m^2) and others that were different from those for which we made estimates (e.g., BMI ≥30 kg/m^2). We first converted these data to mean BMI, which was then used to estimate the prevalences used in our analysis as described above. The dependent variable in these regressions was the inverse of mean BMI; the independent variables were the probit-transformed prevalence, age, sex, year and ln(GDP). We used this specification because the relationship between inverse mean BMI and the probit of prevalence was found to be closer to linear than relationships between other functions of mean BMI and prevalence. There were also statistical interactions between probit-transformed prevalence and age and sex. As above, the regressions also included random effects for regions.

Appendix 3: Validation of statistical model

We tested how well our statistical model, which is described in the main text, predicts missing data in two different tests. In Test 1, we held out all data from 10% of countries with data (i.e., created the appearance of countries with no data where we actually had data). The countries whose data were withheld were randomly selected from the following three groups: data-rich (6 years or more of data for women and 5 years or more for men, with at least one year of data after 2000), data-poor (2 years of data for women and 1 year for men), and average data availability (3 to 5 years of data or 6 years or more of data with no data after 2000 for women, and one fewer year of data for men). In total, there were 66 data-rich countries for women and 72 for men; 52 data-poor countries for women and 48 for men; and 68 countries for women and 58 for men had average data availability.

We fitted the model to the data from the remaining 90% of countries and made estimates of the held-out observations. In Test 2, we assessed other patterns of missing data by holding out 10% of our data sources, again from a mix of data-rich, data-poor, and average-data countries, as defined above. For a given country, we either held out a random one third of the country's data or all of the country's 2000-2014 data to determine, respectively, how well we filled in the gaps for countries with intermittent data and how well we estimated in countries without recent data. We fitted the model to the remaining 90% of the dataset and made estimates of the held-out observations. We repeated each test five times, holding out a different subset of data in each repetition. In both tests, we calculated the differences between the held-out data and the estimates. We also checked the 95% CrIs of the estimates; in a model with good external predictive validity, 95% of held-out values would be included in the 95% CrIs.

The estimates of mean BMI were unbiased as evidenced by median errors that were very close to zero globally, and less than ±0.13 kg/m^2 in every subset of withheld data except for those in relation to age group in Test 1 where median errors ranged from -0.63 to 0.17 kg/m^2 (Appendix Table 6). Even the 25th and 75th percentiles of errors rarely exceeded ±0.8 kg/m^2. The 95% credible intervals of estimated mean BMIs covered 94-98% of true data, which implies good estimation of uncertainty; among subgroups of data, coverage was rarely < 90%. Median errors for prevalences of different BMI categories were also small, and ranged between -0.24 percentage points (BMI 18.5 to <20 kg/m^2 in men) to 1.95 percentage points (BMI 20 to <25 kg/m^2 in men). Median absolute errors ranged from 0.15 to 6.33 percentage points. Estimates for women had slightly larger errors than those for men, possibly because there is more variation in female BMI within and across regions (see below) making it harder to estimate missing country-years. For comparison, median absolute differences for the same BMI categories between pairs of nationally representative surveys done in the same country and in the same year ranged 0.9-5.4 percentage points, indicating that our estimates perform as well as running two parallel surveys in the same country and year. Coverage for different prevalence categories ranged between 56% and 99%, and was generally smaller at the tails of the distribution, because some age-sex groups had zero prevalences of extreme categories; the estimates, and their CrIs, can become very close to zero but not precisely zero.

Appendix Table 1: List of analysis regions and "super-regions", and countries in each region. The hierarchical structure of the statistical model consisted of country, region, super-region, and world.

We did not have data on population by age group for American Samoa, Bermuda, French Polynesia, Greenland and Tokelau. Country-specific estimates were made but were not used in calculation regional and global prevalences because the latter requires weighting by age-specific population.

Super-region	Region
**Sub-Saharan Africa (48)**	**Central Africa (6)**: Angola, Central African Republic, Congo, DR Congo, Equatorial Guinea, Gabon
	**East Africa (17)**: Burundi, Comoros, Djibouti, Eritrea, Ethiopia, Kenya, Madagascar, Malawi, Mauritius, Mozambique, Rwanda, Seychelles, Somalia, Sudan, Tanzania, Uganda, Zambia
	**Southern Africa (6)**: Botswana, Lesotho, Namibia, South Africa, Swaziland, Zimbabwe
	**West Africa (19)**: Benin, Burkina Faso, Cabo Verde, Cameroon, Chad, Cote d'Ivoire, Gambia, Ghana, Guinea, Guinea Bissau, Liberia, Mali, Mauritania, Niger, Nigeria, Sao Tome and Principe, Senegal, Sierra Leone, Togo
**Central Asia, Middle East and North Africa (28)**	**Central Asia (9)**: Armenia, Azerbaijan, Georgia, Kazakhstan, Kyrgyzstan, Mongolia, Tajikistan, Turkmenistan, Uzbekistan
	**Middle East and North Africa (19)**: Algeria, Bahrain, Egypt, Iran, Iraq, Jordan, Kuwait, Lebanon, Libya, Morocco, Occupied Palestinian Territory, Oman, Qatar, Saudi Arabia, Syrian Arab Republic, Tunisia, Turkey, United Arab Emirates, Yemen
**South Asia (6)**	**South Asia (6)**: Afghanistan, Bangladesh, Bhutan, India, Nepal, Pakistan
**East and South East Asia (16)**	**East Asia (4)**: China, China (Hong Kong SAR), North Korea, Taiwan
	**South East Asia (12)**: Brunei Darussalam, Cambodia, Indonesia, Lao PDR, Malaysia, Maldives, Myanmar, Philippines, Sri Lanka, Thailand, Timor-Leste, Viet Nam
**Oceania (17)**	**Polynesia and Micronesia (13)**: American Samoa, Cook Islands, French Polynesia, Kiribati, Marshall Islands, Micronesia (Federated States of), Nauru, Niue, Palau, Samoa, Tokelau, Tonga, Tuvalu
	**Melanesia (4):** Fiji, Papua New Guinea, Solomon Islands, Vanuatu
**High-income Asia Pacific (3)**	**High-income Asia Pacific (3)**: Japan, Singapore, South Korea
**Latin America and Caribbean (35)**	**Andean Latin America (3)**: Bolivia, Ecuador, Peru
	**Caribbean (18)**: Antigua and Barbuda, Bahamas, Barbados, Belize, Bermuda, Cuba, Dominica, Dominican Republic, Grenada, Guyana, Haiti, Jamaica, Puerto Rico, Saint Kitts and Nevis, Saint Lucia, Saint Vincent and the Grenadines, Suriname, Trinidad and Tobago
	**Central Latin America (9)**: Colombia, Costa Rica, El Salvador, Guatemala, Honduras, Mexico, Nicaragua, Panama, Venezuela
	**Southern Latin America (5)**: Argentina, Brazil, Chile, Paraguay, Uruguay
**High-income Western countries (27)**	**High-income English-speaking countries* (6)**: Australia, Canada, Ireland, New Zealand, United Kingdom, United States of America
	**North Western Europe (12)**: Austria, Belgium, Denmark, Finland, Germany, Greenland, Iceland, Luxembourg, Netherlands, Norway, Sweden, Switzerland
	**South Western Europe (9)**: Andorra, Cyprus, France, Greece, Israel, Italy, Malta, Portugal, Spain
**Central and Eastern Europe (20)**	**Central Europe (13)**: Albania, Bosnia and Herzegovina, Bulgaria, Croatia, Czech Republic, Hungary, Macedonia (TFYR), Montenegro, Poland, Romania, Serbia, Slovakia, Slovenia
	**Eastern Europe (7)**: Belarus, Estonia, Latvia, Lithuania, Moldova, Russian Federation, Ukraine

* Although high-income English-speaking countries are geographically separated, they exhibit remarkably similar trends in cardio-metabolic risk factors and outcomes. They were therefore grouped together so that the statistical model shares information amongst them more than it does with other countries that are geographically closer but epidemiologically more distinct. This similarity is also seen in results, e.g., in Figure 2 in the main paper.

Appendix Table 2: Data sources used in the analysis.

Country	Data years	Survey/study name	Level of representativness	Rural, urban, or both	Age range as used for global analysis: Male	Age range as used for global analysis: Female	Sample size: Male	Sample size: Female
Afghanistan	2013	National Nutrition Survey	National	both		18-49		12987
Albania	2001	Shapo et al., Public Health Nutr 6:471-77, 2003	Community	urban	24+	24+	535	585
Albania	2008-2009	DHS	National	both	18-49	18-49	2534	6372
Algeria	2003	STEPS	Community	both	25-64	25-64	1612	2435
Algeria	2005	Transition and Health Impact in North Africa	National	both	35-70	35-70	2004	2737
Algeria	2007-2009	The ISOR (InSulino-resistance in ORan) study	Community	urban	30-64	30-64	378	409
American Samoa	1976-1978	McGarvey, Am J Clin Nutr 53(6 Suppl):1586S-1594S, 1991	National	both	18+	18+	850	1191
American Samoa	1990	McGarvey, Pac Health Dialog 8(1):157-62, 2001	National	both	25+	25+	359	484
American Samoa	1994	McGarvey, Pac Health Dialog 8(1):157-62, 2001	National	both	25+	25+	165	241
American Samoa	2004	STEPS	National	both	25-64	25-64	949	1060
Argentina	1981-1985	Hernandez et al., Diabetes Res Clin Pract 3:277-83, 1987	Community	urban	20-74	20-74	395	414
Argentina	1985-1986	INTERSALT	Community	urban	20-59	20-59	100	100
Argentina	1995-1998	de Sereday et al., Diabetes Metab 30:335-9, 2004	Subnational	urban	18-74	18-74	924	1246
Argentina	2003	CEDES-Programa VIGI+A-Banco Mundial, 2004	Community	urban	18-74	18-74	151	176
Argentina	2004-2005	CArdiovascular Risk factors Multiple Evaluation in Latin America	Community	urban	25-64	25-64	733	742
Argentina	2005	Encuensta Nacional de Nutricion y Salud 2005	National	both		18-49		4803
Argentina	2006	Virasoro Survey	Community	urban	18-84	18-84	261	306
Argentina	2008-2011	The VELA Project	Community	rural	18+	18+	146	273
Argentina	2011-2012	CESCAS Study	Community	urban	35-74	35-74	1584	2395
Armenia	1998	The health and nutritional status of children and women in Armenia	National	both		18-45		2420
Armenia	2000	DHS	National	both		18-49		5264
Armenia	2005	DHS	National	both	18-49	18-49	1025	5545
Australia	1975	Busselton Health Study	Community	urban	18+	18+	373	418
Australia	1978	Busselton Health Study	Community	urban	18+	18+	462	550
Australia	1980	Sydney coronary heart disease prevention program	Community	urban	25-64	25-64	705	689
Australia	1980	Risk Factor Prevalence Survey	National	urban	25-64	25-64	2764	2839
Australia	1981	Busselton Health Study	Community	urban	18+	18+	225	290
Australia	1983	MONICA, Newcastle	Subnational	urban	35-64	35-64	1215	1244
Australia	1983	MONICA, Perth inner	Community	urban	25-64	25-64	861	921
Australia	1983	Risk Factor Prevalence Survey	National	urban	25-64	25-64	3733	3863
Australia	1988-1989	Dubbo Study of Australian Elderly	Community	urban	59+	59+	877	1219
Australia	1988-1989	MONICA, Newcastle	Subnational	urban	35-64	35-64	672	671
Australia	1988-1989	MONICA, Newcastle	Community	urban	25-34	25-34	70	84
Australia	1988-1989	MONICA, Perth inner	Community	urban	25-64	25-64	403	405
Australia	1988-1989	MONICA, Perth outer	Community	urban	25-64	25-64	412	419
Australia	1989	Risk Factor Prevalence Surveys 1989	National	urban	18+	18+	4497	4678
Australia	1992-1993	Australia Longitudinal Study of Ageing	Community	urban	65+	65+	814	746
Australia	1994	MONICA, Newcastle	Subnational	urban	35-64	35-64	637	688
Australia	1994	MONICA, Perth inner	Community	urban	25-64	25-64	363	349
Australia	1994	MONICA, Perth outer	Community	urban	25-64	25-64	373	387
Australia	1995-1996	National Nutrition Study	National	both	18+	18+	4864	5352
Australia	1996-1998	Western Australian AAA Screening Program	Community	urban	65-84		12194	
Australia	1999-2000	The Australian Diabetes, Obesity and Lifestyle Study 1999-2000	National	both	25+	25+	4991	6070
Australia	1999-2003	North West Adelaide Health Study	Community	urban	18+	18+	1932	2122
Australia	2000	Perth children	Community	both	25	25	266	334
Australia	2004-2005	Janus et al., Med J Aust 187:147-52, 2007	Community	rural	25-74	25-74	383	423
Australia	2004-2005	The Australian Diabetes, Obesity and Lifestyle Study 2004-2005	National	both	30+	30+	2874	3472
Australia	2004-2006	North West Adelaide Health Study	Community	urban	20+	20+	1523	1679
Australia	2007-2008	National Health Survey	National	both	18+	18+	5279	5655
Australia	2008-2010	North West Adelaide Health Study	Community	urban	24+	24+	1168	1318
Australia	2011-2012	Australian Health Survey	National	both	18+	18+	9975	10849
Australia	2012	The Australian Diabetes, Obesity and Lifestyle Study 2012	National	both	37+	37+	2048	2526
Austria	1977	Rhomberg, Wien Klin Wochenschr Suppl 127:1-30, 1981	Community	rural	20-64	20-64	295	316
Austria	1986	CINDI	Community	both	25-64	25-64	657	715
Austria	1992	Vorarlberg Health Monitoring and Promotion Programme	Subnational	rural	18+	18+	14161	18835
Austria	1998	Vorarlberg Health Monitoring and Promotion Programme	Subnational	rural	18+	18+	16153	20915
Austria	2004	Vorarlberg Health Monitoring and Promotion Programme	Subnational	rural	18+	18+	20160	23893
Azerbaijan	1996	Health and Nutrition Survey	National	both	19-59	19-59	121	295
Azerbaijan	2001	Reproductive Health Survey (RHS)	National	both		18-44		1726
Azerbaijan	2006	DHS	National	both	18-59	18-49	2208	7044
Bahrain	1991-1992	al-Mannai et al., J R Soc Health 116:30-2, 7-40, 1996	Community	both	20+	20+	137	153

Country	Data years	Survey/study name	Level of representativness		Age range as used for global analysis		Sample size	
				Rural, urban, or both	Male	Female	Male	Female
Bahrain	1998-1999	National Nutrition Survey	National	both	19+	19+	1120	1181
Bahrain	2007	STEPS	National	both	20-64	20-64	854	858
Bahrain		Musaiger et al., Ann Hum Biol 28:346-50, 2001	Community	both	30+	30+	298	216
Bangladesh	1996-1997	DHS	National	both		18-49		3761
Bangladesh	1998	Zaman et al., J Health Popul Nutr 21:162-63, 2003	Community	rural	20+	20+	290	379
Bangladesh	1999-2000	Hussain et al., Eur J Public Health, 17:291-96, 2007	Community	rural	20-59	20-59	2037	2720
Bangladesh	1999-2000	DHS	National	both		18-49		4324
Bangladesh	2000-2004	Nutritional Surveillance Project	National	rural		18-45		224251
Bangladesh	2002	STEPS	National	rural	25-64	25-64	2086	2038
Bangladesh	2002	STEPS	National	urban	25-64	25-64	3533	3737
Bangladesh	2004	DHS	National	both		18-49		9798
Bangladesh	2007	DHS	National	both		18-49		9669
Bangladesh	2011	DHS	National	both	18+	18+	5252	17642
Bangladesh		Rahman et al., Hypertension 33:74-8, 1999	Community	rural	30+	30+	965	643
Barbados	1987-1992	Barbados Eye Study	National	both	40-84	40-84	1980	2627
Barbados	1991-1994	Cooper et al., Am J Public Health 87(2):160-68, 1997	Community	urban	25-100	25-100	329	482
Barbados	1997-2002	The Barbados Incidence Studies of Eye Diseases II	National	both	40-84	40-84	1004	1441
Barbados	1999-2000	The Survey on Health, Well-Being, and Aging in Latin America and the Caribbean (SABE)	Community	urban	60+	60+	559	866
Belgium	1983-1985	MONICA, Luxembourg	Community	urban	35-64	35-64	944	936
Belgium	1984-1985	Belgian Interuniversity Research on Nutrition and Health	National	both	25-74	25-74	5897	5289
Belgium	1985-1986	INTERSALT, Ghent	Community	urban	20-59	20-59	100	100
Belgium	1985-1987	INTERSALT, Charleroi	Community	urban	20-59	20-59	82	75
Belgium	1985-1987	MONICA, Charleroi	Community	urban	25-64	25-64	347	327
Belgium	1985-1987	MONICA, Ghent	Community	urban	25-64	25-64	549	459
Belgium	1985-1990	Flemish Study on Environment, Genes and Health Outcomes	Community	rural	20-90	20-90	656	692
Belgium	1987-1990	MONICA, Charleroi	Community	urban	25-64	25-64	325	301
Belgium	1988-1990	MONICA, Ghent	Community	urban	25-64	25-64	456	449
Belgium	1990-1992	MONICA, Ghent	Community	urban	25-64	25-64	507	475
Belgium	1990-1993	MONICA, Charleroi	Community	urban	25-64	25-64	337	332
Belgium	1991-1994	Flemish Study on Environment, Genes and Health Outcomes	Community	rural	26-88	26-88	393	416
Belgium	1992-1995	Flemish Study on Environment, Genes and Health Outcomes	Community	rural	27-89	27-89	298	312
Belgium	1994-1996	BIRNH Elderly: Belgian Interuniversity Research on Nutrition and Health in the Elderly	National	both	65-89	65-89	1142	953
Belgium	1996-1998	Flemish Study on Environment, Genes and Health Outcomes	Community	rural	18-84	18-84	354	347
Belgium	1998	Flemish Study on Environment, Genes and Health Outcomes	Community	rural	32-86	32-86	320	349
Belgium	1998-2000	Flemish Study on Environment, Genes and Health Outcomes	Community	rural	18-80	18-80	198	196
Belgium	1999-2001	Flemish Study on Environment, Genes and Health Outcomes	Community	rural	18-81	18-81	213	227
Belgium	2001	Flemish Study on Environment, Genes and Health Outcomes	Community	rural	18-78	18-78	230	200
Belgium	2002-2003	Flemish Study on Environment, Genes and Health Outcomes	Community	rural	18-81	18-81	169	181
Belgium	2002-2004	SPAH	Subnational	both	18-75	18-75	2595	2308
Belgium	2002-2005	Flemish Study on Environment, Genes and Health Outcomes	Community	rural	18-88	18-88	391	404
Belgium	2003	The European Male Ageing Study	Community	both	40+		433	
Belgium	2005-2008	Flemish Study on Environment, Genes and Health Outcomes	Community	rural	18-89	18-89	449	455
Belgium	2008	The European Male Ageing Study	Community	both	40+		383	
Belgium	2009-2013	Flemish Study on Environment, Genes and Health Outcomes	Community	rural	20-88	20-88	330	335
Belgium	2010-2015	Flemish Study on Environment, Genes and Health Outcomes	Community	rural	18-87	18-87	388	409
Belize	2004-2005	CAMDI	National	both	20+	20+	599	1019
Benin	1996	DHS	National	both		18-49		2292
Benin	2001	DHS	National	both		18-49		4787
Benin	2006	DHS	National	both		18-49		13299
Benin	2007	STEPS	Community	urban	25-64	25-64	955	1508
Benin	2008	STEPS	National	both	25-64	25-64	3430	3365
Benin	2011-2012	DHS	National	both		18-49		12836
Bhutan	2007	STEPS	Community	urban	25-74	25-74	1125	1318
Bhutan	2014	STEPS	National	both	18-69	18-69	1069	1674
Bolivia	1994	DHS	National	both		18-49		2286
Bolivia	1998	DHS	National	both		18-49		4126
Bolivia	2003	DHS	National	both		18-49		13974
Bolivia	2005	Reyes-Garcia et al., Soc Sci Med 69:571-78, 2009	Community	rural	18-84		206	
Bolivia	2005-2007	Baya Botti et al., Nutr Hosp 24(3):304-11, 2009	National	both	18-18	18-18	137	131
Bolivia	2008	DHS	National	both		18-49		13497
Bosnia and Herzegovina	2002	Non-communicable disease risk factor survey, Federation of B&H	Subnational	both	25-64	25-64	1118	1613

Country	Data years	Survey/study name	Level of representativness		Age range as used for global analysis		Sample size	
				Rural, urban, or both	Male	Female	Male	Female
Bosnia and Herzegovina	2012	Non-communicable disease risk factor survey, Federation of B&H	Subnational	rural	18+	18+	1191	1274
Bosnia and Herzegovina	2012	Non-communicable disease risk factor survey, Federation of B&H	Subnational	urban	18+	18+	591	697
Botswana	2007	STEPS	National	both	25-64	25-64	1243	2577
Brazil	1989	Pesquisa Nacional sobre Saude e Nutricao	National	both	18+	18+	16783	17897
Brazil	1990-1991	Fornes et al., Rev Saude Publica 36:12-8, 2002	Community	urban	20+	20+	432	613
Brazil	1991-1993	EPIDOSO	Community	urban	65+	65+	269	473
Brazil	1992-1998	Moraes et al., Int J Cardiol 90:205-11, 2003	Community	urban	18+	18+	438	543
Brazil	1995	Health and Nutrition Survey of Rio de Janeiro	Community	urban	60+	60+	248	385
Brazil	1995-1996	Cohort study from Porto Alegre	Community	both	18+	18+	489	596
Brazil	1996	DHS	National	both		18-49		3081
Brazil	1996-1997	The Bambui Cohort Study of Ageing	Community	urban	18+	18+	931	1335
Brazil	1997	PPV	Subnational	both	20+	20+	8063	9121
Brazil	1998	Belo Horizonte Heart Study	Community	urban	18-19	18-19	31	43
Brazil	1999-2000	Pelotas cross-sectional survey	Community	urban	20-69	20-69	839	1096
Brazil	1999-2000	The Survey on Health, Well-Being, and Aging in Latin America and the Caribbean (SABE)	Community	urban	60+	60+	732	1064
Brazil	2001	de Freitas et al., Arq Bras Cardiol 88:191-99	Community	urban	18+	18+	310	331
Brazil	2001-2003	Bustos et al., Nutr Metab Cardiovasc Dis 17:581-89, 2007	Community	both	22-28	22-28	992	1064
Brazil	2002-2003	Pesquisa de Orcamentos Familiares	National	both	18+	18+	55681	56389
Brazil	2003	PNAFS	Community	urban	20+	20+	1155	1941
Brazil	2003	Women health in Southern Brazil	Community	urban		20-60		986
Brazil	2003-2005	São Paulo Health & Ageing Study	Community	urban	65+	65+	783	1198
Brazil	2004-2005	Pimenta et al., Arq Bras Cardiol 90:386-92, 2008	Community	rural	18+	18-84	291	286
Brazil	2004-2006	Hearts of Brazil	National	urban	18+	18+	550	626
Brazil	2006	Krause et al., J Aging Phys Act 17:387-97, 2009	Community	urban	60+	60+	93	1069
Brazil	2006	ATITUDE	Subnational	both	18-21	18-21	1325	1734
Brazil	2006	Pesquisa Nacional de Demografia e Saude 2006	National	both		18-49		13375
Brazil	2006-2007	SOFT study	Community	urban	18+	18+	739	1099
Brazil	2008	The Bambui Cohort Study of Ageing	Community	urban	71+	71+	248	456
Brazil	2008-2009	Pesquisa de Orcamentos Familiares	National	both	18+	18+	62035	65691
Brazil	2009-2010	EPIFLORIPA Adults	Community	urban	25-59	25-59	631	795
Brazil	2009-2010	Condições de Saúde de Idosos de Florianópolis - EpiFloripa Idoso	Community	urban	60+	60+	594	1052
Brazil	2010-2013	Baependi Heart Study	Community	rural	18+	18+	607	884
Brazil	2011	ATITUDE	Subnational	both	18-19	18-19	688	844
Brazil	2012-2013	Prevalence of Leptin Polymorphism Gln223Arg	Community	urban	18+	18+	282	523
Brazil	2014	Brazilian Guide to the Physical Fitnees related to Health Assessment and Lifestyle Habits	Community	urban	18-19	18-19	73	45
Bulgaria	2004	National Nutrition Survey	National	both	18+	18+	515	515
Burkina Faso	1992-1993	DHS	National	both		18-49		3410
Burkina Faso	1998-1999	DHS	National	both		18-49		3300
Burkina Faso	2003	DHS	National	both		18-49		9360
Burkina Faso	2004	Ouedraogo et al., Public Health Nutr 11:1280-87, 2008	Community	urban	35+	35+	956	1066
Burkina Faso	2010	DHS	National	both		18-49		6730
Burkina Faso	2013	STEPS	National	both	25-64	25-64	2223	2250
Burundi	2010	DHS	National	both		18-49		3471
Cabo Verde	2007	STEPS	National	both	25-64	25-64	658	1066
Cambodia	2000	DHS	National	both		18-49		5888
Cambodia	2005	DHS	National	both		18-49		6952
Cambodia	2008	Anthropometrics Survey	National	both		18-49		5955
Cambodia	2010	DHS	National	both		18-49		7650
Cambodia	2010	STEPS	National	both	25-64	25-64	1881	3343
Cameroon	1998	DHS	National	both		18-49		1574
Cameroon	1998-1999	ENHIP	Community	rural	18+	18+	490	683
Cameroon	1998-1999	ENHIP	Community	urban	18+	18+	431	538
Cameroon	2003	STEPS	Subnational	urban	18+	18+	3331	4991
Cameroon	2004	DHS	National	both		18-49		3924
Cameroon	2007	Cameroon Burden of Diabetes - Second Survey	Subnational	urban	18+	18+	3122	4123
Cameroon	2009	National Survey of Micronutrient Status and Consumption of Fortifiable Foods	National	both		18-49		788
Cameroon	2011	DHS	National	both		18-49		6281
Canada	1981	Canada Fitness Survey	National	both	18-64	18-64	5133	5774
Canada	1985-1986	INTERSAL, St Johns	Community	urban	20-59	20-59	100	100
Canada	1985-1988	MONICA, Halifax	Community	both	25-64	25-64	438	420
Canada	1986-1992	Canada Heart Health Survey	National	both	18-74	18-74	9644	9777

Country	Data years	Survey/study name	Level of representativness		Age range as used for global analysis		Sample size	
				Rural, urban, or both	Male	Female	Male	Female
Canada	1991-1992	Canadian Study of Health and Aging	Community	both	70+	70+	236	348
Canada	1993	Chen et al., Int J Obes Relat Metab Disord 22:771-77, 1998	Community	rural	18-74	18-74	803	988
Canada	1995	MONICA, Halifax	Community	both	25-64	25-64	274	287
Canada	1995-1997	CaMos	Subnational	both	25+	25+	2792	6302
Canada	1996	Canadian Study of Health and Aging	Community	both	70+	70+	236	348
Canada	2005	CCHS	National	both	18+	18+	1684.112	2030.825
Canada	2007-2011	Canadian Health Measures Survey	National	both	18-79	18-79	3528	3994
Canada	2008	CCHS	National	both	18+	18+	1689.482	1987.779
Canada		PEI Nutrition Survey	Subnational	both	18-74	18-74	1000	995
Central African Republic	1994-1995	DHS	National	both		18-49		1945
Central African Republic	2010	STEPS	Community	both	25-64	25-64	1846	1967
Chad	1996-1997	DHS	National	both		18-49		3581
Chad	2004	DHS	National	both		18-49		2840
Chad	2008	STEPS	Community	urban	25-64	25-64	995	845
Chile	1989	INCLEN	Community	urban	35-65		199	
Chile	1992-1993	Miquel et al., Gastroenterology 115: 937-46, 1998	Community	urban	18+	18+	657	1031
Chile	1999-2000	The Survey on Health, Well-Being, and Aging in Latin America and the Caribbean (SABE)	Community	urban	60+	60+	410	806
Chile	2000	Nervi et al., J Hepatol 45: 299 -305, 2006	Community	urban	18+	18+	335	624
Chile	2001-2003	Bustos et al., Nutr Metab Cardiovasc Dis 17:581-89, 2007	Community	both	22-28	22-28	436	562
Chile	2003	Encuesta Nacional de Salud	National	both	18+	18+	1545	1849
Chile	2004-2005	CArdiovascular Risk factors Multiple Evaluation in Latin America	Community	urban	25-64	25-64	783	865
Chile	2005	Palomo et al., Rev Med Chil 135:904-12, 2007	Community	urban	18-74	18-74	339	668
Chile	2009-2010	Encuesta Nacional de Salud	National	both	18+	18+	1871	2833
Chile	2011-2012	CESCAS Study	Community	urban	35-74	35-74	922	1027
Chile	2013	Global School-based Student Health Survey	National	both	18-19	18-19	82	55
China	1979-1982	East Beijing Study 1	Community	urban	20-84	20-84	361	380
China	1982	China National Nutritional Survey	National	both	18-94	18-94	8918	8753
China	1983	Sino-MONICA Shanghai	Subnational	both	30-64	30-64	624	630
China	1983-1984	Wang et al., Zhonghua Liu Xing Bing Xue Za Zhi 24:272-75, 2003	Community	both	25-64	25-64	877	832
China	1984-1985	MONICA, Beijing	Community	both	25-64	25-64	813	856
China	1984-1985	Sino-MONICA Beijing	Subnational	both	25-64	25-64	813	857
China	1986	INTERSALT, Beijing	Community	urban	20-59	20-59	100	100
China	1986	INTERSALT, Nanning	Community	both	20-59	20-59	100	100
China	1986	INTERSALT, Tianjin	Community	urban	20-59	20-59	100	100
China	1986-1989	Ewang et al., Zhonghua Liu Xing Bing Xue Za Zhi 26:394-9, 2005	Community	both	45-64		18244	
China	1986-1989	Sino-MONICA Shanghai	Subnational	both	25-64	25-64	675	753
China	1987	INCLEN	Community	urban	35-65		989	
China	1988	Sino-MONICA Hebei	Subnational	both	25-64		800	
China	1988	Sino-MONICA Heilongjiang	Subnational	both	25-64	25-64	800	800
China	1988	Sino-MONICA Henan	Subnational	both	25-64	25-64	345	427
China	1988	Sino-MONICA Neimenggu	Subnational	both	25-64	25-64	396	400
China	1988	Sino-MONICA Sichuan	Subnational	both	25-64	25-64	312	334
China	1988	Sino-MONICA Shandong	Subnational	both	25-64	25-64	211	225
China	1988-1989	Wang et al., Zhonghua Liu Xing Bing Xue Za Zhi 24:272-75, 2003	Community	both	25-64	25-64	873	731
China	1988-1989	MONICA, Beijing	Community	both	25-64	25-64	701	862
China	1988-1989	Sino-MONICA Beijing	Subnational	both	25-64	25-64	701	862
China	1988-1989	Sino-MONICA Jilin	Subnational	both	25-64	25-64	380	400
China	1988-1989	Sino-MONICA Jiangxi	Subnational	both	25-64	25-64	379	386
China	1988-1989	Sino-MONICA Liaoning	Subnational	both	25-64	25-64	728	734
China	1988-1990	East Beijing Study 2	Community	urban	20-84	20-84	135	148
China	1989	The Tianjin Project	Community	urban	18-64	18-64	3894	3971
China	1989†	China Health and Nutrition Survey	National	both	18-45	18-45	2356	2549
China	1989	Sino-MONICA Fujian	Subnational	both	25-64	25-64	179	191
China	1989	Sino-MONICA Jiangsu	Subnational	both	25-64	25-64	398	399
China	1990-1991	China Prospective Study	National	both	40-79		221194	
China	1991	China National Hypertension Survey Epidemiology Follow-up Study	National	both	40+	40+	75696	79040
China	1991†	China Health and Nutrition Survey	National	both	18+	18+	4047	4470
China	1991	Sino-MONICA Shanghai	Subnational	both	30-64	30-64	564	624
China	1991-1992	Fangshan Cohort Study	Community	urban	34-86	34-86	871	1736
China	1992	Huashan Study	Community	urban	35-75	35-75	892	965
China	1992	China National Nutritional Survey	National	both	18-92	18-92	23814	27157

Country	Data years	Survey/study name	Level of representativness		Age range as used for global analysis		Sample size	
				Rural, urban, or both	Male	Female	Male	Female
China	1992	Sino-MONICA Sichuan	Subnational	both	25-64	25-64	608	526
China	1992-1993	Anzhen 02 Cohort Study	Community	urban	34-65	34-65	2032	2120
China	1993	Wang et al., Zhonghua Liu Xing Bing Xue Za Zhi 24:272-75, 2003	Community	both	25-64	25-64	822	617
China	1993†	China Health and Nutrition Survey	National	both	18+	18+	3864	4235
China	1993	MONICA, Beijing	Community	both	25-64	25-64	613	816
China	1993	Sino-MONICA Anhui	Subnational	both	25-64	25-64	193	195
China	1993	Sino-MONICA Beijing	Subnational	both	25-64	25-64	613	816
China	1993	Sino-MONICA Jiangsu	Subnational	both	25-64	25-64	462	365
China	1993	Sino-MONICA Liaoning	Subnational	both	25-64	25-64	493	500
China	1996	Wang et al., Zhonghua Liu Xing Bing Xue Za Zhi 24:272-75, 2003	Community	both	25-64	25-64	735	721
China	1996	The Tianjin Project	Community	urban	18-64	18-64	722	717
China	1996-2003	Wu et al., Osteoporos Int 15:751-59, 2004	Community	urban		18+		3418
China	1997†	China Health and Nutrition Survey	National	both	18+	18+	4164	4401
China	1997	International collaborative study of macronutrients, micronutrients and blood pressure	Community	rural	40-59	40-59	133	139
China	1997	International collaborative study of macronutrients, micronutrients and blood pressure	Community	rural	40-59	40-59	140	138
China	1997	International collaborative study of macronutrients, micronutrients and blood pressure	Community	rural	40-59	40-59	143	146
China	1997-2000	Shanghai Women's Study	Community	urban		40-70		74881
China	1998	Shanghai Diabetes Study	Community	urban	25+	25+	1264	1768
China	1998-2000	Jia et al., Obes Rev 3:157-65, 2002	Community	urban	20+	20+	1106	1670
China	1999	Wang et al., Zhonghua Liu Xing Bing Xue Za Zhi 24:272-75, 2003	Community	both	25-64	25-64	818	685
China	1999-2000	Xu et al., Public Health Nutr 8:47-51, 2005	Community	both	35+	35+	18194	18902
China	2000†	China Health and Nutrition Survey	National	both	18+	18+	4515	4934
China	2000-2001	The International Collaborative Study of Cardiovascular Disease in ASIA	National	both	35-74	35-74	7511	8006
China	2001	Shanghai Diabetes Study	Community	urban	25+	25+	1264	1768
China	2001-2006	Shanghai Men's Study	Community	urban	40-74		61392	
China	2002	Ma et al., Zhonghua Liu Xing Bing Xue Za Zhi 25:1035-8, 2004	Subnational	both	18+	18+	7352	7352
China	2002	China National Nutritional and Health Survey	National	both	18-101	18-101	64630	75015
China	2002-2003	Fan et al., J Gastroenterol Hepatol 20:1825-32, 2005	Community	urban	18+	18-74	5502	7767
China	2002-2006	SMHS	Community	urban	40-65		48018	
China	2004	Tian et al., Prev Med 48:59-63, 2009	Community	rural	18+	18+	1022669	1163313
China	2004	Beijing Child and Adolescent Metabolic Syndrome study	Community	both	18-18	18-18	248	258
China	2004†	China Health and Nutrition Survey	National	both	18+	18+	4345	4748
China	2004-2006	Pang et al., Intern Med 47:893-97, 2008	Community	rural	35+	35+	22963	22962
China	2004-2008	Xinjiang Children and Adolescent Survey	Subnational	both	18-18	18-18	113	123
China	2005	Ye et al., J Am Coll Cardiol 49:1798-805, 2007	Community	urban	50-70	50-70	743	906
China	2005-2006	Zhou et al., World J Gastroenterol 13:6419-24, 2007	Community	urban	18-79	18-79	1101	2063
China	2005-2006	Hong Kong Growth Survey	Subnational	both	18-19	18-19	920	951
China	2006	Beijing Eye Study	Community	both	45+	45+	1394	1820
China	2006†	China Health and Nutrition Survey	National	both	18+	18+	4250	4781
China	2007	Beijing Child and Adolescent Metabolic Syndrome study	Community	urban	18-18	18-18	20	18
China	2007-2008	China National Diabetes & Metabolic Disorders Study	National	both	20+	20+	18419	27820
China	2007-2010	SAGE	National	both	50+	50+	5759	6617
China	2008-2010	Fangshan Family-based Ischemic Stroke Study in China (FISSIC) program	Community	rural	40+	40+	19478	36449
China	2009†	China Health and Nutrition Survey	National	both	18+	18+	4489	4936
China	2010	China Noncommunicable Disease Surveillance	National	both	18+	18+	45066	53452
China	2011	Beijing Eye Study	Community	both	50+	50+	1467	1895
China	2011	Beijing Pediatric Eye Study	Community	both	18-18	18-18	615	588
China	2011†	China Health and Nutrition Survey	National	both	18+	18+	5886	6645
China	2011	Nutrition and Health of Aging Population in China	Community	both	55-77	55-77	952	1296
China	2012	Shandong Children Study	Community	rural	18-18	18-18	53	44
China	2012	Shandong Children Study	Community	urban	18-18		4	
China	2012-2013	The Kailuan Study	Community	urban	18+	18+	80921	21385
China	2013	Ejina Children Study	Community	both	18-21	18-21	28	25
China		Hua et al., Zhonghua Nei Ke Za Zhi 36:18-20, 1997	Community	rural	60+	60+	288	335
China		Chen et al., Zhonghua Yi Xue Za Zhi 85(40):2830-4, 2005	Subnational	both	35-85	35-85	13549	10315
China (Hong Kong SAR)	1985-1986	Shatin New Town Study	Community	urban	70+	70+	276	669
China (Hong Kong SAR)	1991	The Hong Kong study on Health, health risk and quality of life in the Chinese elderly cohort	Community	both	70+	70+	943	944
China (Hong Kong SAR)	1995-1996	Hong Kong Cardiovascular Risk Factor Prevalence Study 1995-1996	National	both	25-74	25-74	1412	1478
Colombia	1986	INTERSALT	Community	rural	20-59	20-59	96	95
Colombia	1995	DHS	National	both		18-49		3242
Colombia	2000	DHS	National	both		18-49		3158

Country	Data years	Survey/study name	Level of representativness		Age range as used for global analysis		Sample size	
				Rural, urban, or both	Male	Female	Male	Female
Colombia	2001	CINDI/CARMEN - Bucaramaga	Community	urban	18-74	18-74	627	1218
Colombia	2002	CINDI/CARMEN - Bogota	Community	urban	18-74	18-74	322	570
Colombia	2004-2005	CArdiovascular Risk factors Multiple Evaluation in Latin America	Community	urban	25-64	25-64	738	812
Colombia	2005	DHS	National	both	18-64	18-64	25094	38620
Colombia	2005	Encuesta Nacional de Situacion Nutricional	National	both		18-49		2783
Colombia	2007	Encuesta Nacional de Salud	National	both	18-69	18-69	5462	7686
Colombia	2010	DHS	National	both	18-64	18-64	39546	51623
Colombia	2010	STEPS	Subnational	both	18-64	18-64	922	1239
Comoros	1996	DHS	National	both		18-49		773
Comoros	2011	STEPS	National	both	25-64	25-64	1541	3500
Comoros	2012	DHS	National	both		18-49		4105
Congo	1986	Maire et al., Rev Epidemiol Sante Publique 40:252-58, 1992	Community	urban		18-45		558
Congo	1986	Enquête Brazzaville 1986	Community	urban		18-50		935
Congo	1987	Maire et al., Rev Epidemiol Sante Publique 40:252-58, 1992	Community	rural		18-45		750
Congo	1987	Enquête Nationale Congo 1987	National	rural		18-49		1264
Congo	1991	Enquête Brazzaville 1991	Community	urban	18-90	18-90	1200	1806
Congo	1996	Enquête Brazzaville 1996	Community	urban	18-90	18-90	1346	1818
Congo	2004	STEPS	Community	urban	25-64	25-64	1013	956
Congo	2005	DHS	National	both		18-49		5390
Congo	2011-2012	DHS	National	both		18-49		4415
Cook Islands	2003	STEPS	National	both	25-64	25-64	925	958
Costa Rica	2004	CAMDI	Community	urban	20+	20+	304	624
Costa Rica	2004-2006	Costa Rican Longevity and Healthy Aging Study Pre-1945 Cohort Wave 1	National	both	60+	60+	1163	1350
Costa Rica	2006-2008	Costa Rican Longevity and Healthy Aging Study Pre-1945 Cohort Wave 2	National	both	62+	62+	944	1102
Costa Rica	2008-2009	Encuesta Nacional de Nutricion 2008-2009	National	both		45+		661
Costa Rica	2009-2010	Costa Rican Longevity and Healthy Aging Study Pre-1945 Cohort Wave 3	National	both	64+	64+	737	890
Costa Rica	2010-2011	Costa Rican Longevity and Healthy Aging Study 1945-1955 Cohort Wave 1	National	both	54-66	54-66	1058	1676
Costa Rica	2012-2014	Costa Rican Longevity and Healthy Aging Study 1945-1955 Cohort Wave 2	National	both	56-68	56-68	867	1470
Cote d'Ivoire	1994	DHS	National	both		18-49		2981
Cote d'Ivoire	1998-1999	DHS	National	both		18-49		2278
Cote d'Ivoire	2005	STEPS	National	rural	18-64	18-64	853	963
Cote d'Ivoire	2005	STEPS	National	urban	18-64	18-64	982	1289
Cote d'Ivoire	2011-2012	DHS	National	both		18-49		4023
Croatia	1997-1999	Budak A et al., Lijec Vjesn 125(1-2):32-5, 2003	National	both	25-100	25-100	1763	2684
Croatia	2006-2008	The Cardiovascular risk factors in school age – intervention model development	National	both	18-20	18-20	190	202
Cuba	1981-1982	Berdasco, Eur J Clin Nutr 1994; 48 Suppl 3:S155-63; discussion S64, 1994	Subnational	both	20-59	20-59	11355	18708
Cuba	1999-2000	The Survey on Health, Well-Being, and Aging in Latin America and the Caribbean (SABE)	Community	urban	60+	60+	630	1044
Cuba	2001-2002	National Survey of Risk Factors	National	both	20-60	20-60	11425.5	11425.5
Cuba	2010	National Risk Factor Survey	National	both	18+	18+	3200	3731
Cuba	2011	Non communicable disease risk factor in Cienfuegos	Community	urban	18-80	18-80	593	849
Cyprus	1999-2000	Countrywide Integrated Noncommunicable Diseases Intervention Programme Cyprus	National	both	25-65	25-65	457	546
Cyprus	2007-2008	Asthma Study Cyprus	National	both	18-18	18-18	13	22
Czech Republic	1985	Czech-MONICA	National	both	25-64	25-64	1243	1303
Czech Republic	1988	Czech-MONICA	National	both	25-64	25-64	1357	1408
Czech Republic	1992	Czech-MONICA	National	both	25-64	25-64	1131	1207
Czech Republic	1997-1998	post-Czech-MONICA	National	both	25-64	25-64	1527	1665
Czech Republic	2000-2001	post-Czech-MONICA	National	both	25-64	25-64	1628	1690
Czech Republic	2002-2005	Health, Alcohol and Psychosocial factors In Eastern Europe	Subnational	urban	45-69	45-69	3247	3888
Czech Republic	2007-2008	post-Czech-MONICA	National	both	25-64	25-64	1717	1861
Denmark	1972*	The Danish Conscription Database	National	both	18-26		22013	
Denmark	1973*	The Danish Conscription Database	National	both	18-26		13980	
Denmark	1974*	The Danish Conscription Database	National	both	18-26		14824	
Denmark	1974-1975	Drivsholm et al., Diabet Med 18:126-32, 2001	Subnational	urban	60	60	360	306
Denmark	1975	The Danish Conscription Database	National	both	18-26		21359	
Denmark	1976	The Danish Conscription Database	National	both	18-26		27366	
Denmark	1977	The Danish Conscription Database	National	both	18-26		25629	
Denmark	1978	The Danish Conscription Database	National	both	18-26		13576	
Denmark	1979	The Danish Conscription Database	National	both	18-26		6660	
Denmark	1980	The Danish Conscription Database	National	both	18-26		2895	
Denmark	1981	The Danish Conscription Database	National	both	18-26		1749	
Denmark	1982	The Danish Conscription Database	National	both	18-26		1184	

Country	Data years	Survey/study name	Level of representativness		Age range as used for global analysis		Sample size	
				Rural, urban, or both	Male	Female	Male	Female
Denmark	1982-1984	MONICA, Glostrup	Community	urban	30-61	30-61	1940	1844
Denmark	1982-1984	Obesity Research Group-Copenhagen City Heart Study 2	Subnational	both	22-63		1140	
Denmark	1983	The Danish Conscription Database	National	both	18-26		761	
Denmark	1984	The Danish Conscription Database	National	both	18-26		378	
Denmark	1984-1985	The Epidemiology of Gallstones in a 70 Year-Old Danish Population	Community	both	70	70	202	172
Denmark	1985	INTERSALT	Community	urban	20-59	20-59	99	100
Denmark	1986-1987	MONICA, Glostrup	Community	urban	29-61	29-61	746	753
Denmark	1987	Nilsson et al., J Intern Med 237:479-86, 1995	Community	urban	51		439	
Denmark	1991-1992	MONICA, Glostrup	Community	urban	29-61	29-61	808	816
Denmark	1992-1994	Obesity Research Group-Copenhagen City Heart Study 3	Subnational	both	32-73		922	
Denmark	1993-1997	EPIC Aarhus	Community	urban	50-65	50-65	8430	8717
Denmark	1993-1997	EPIC Copenhagen	Community	urban	50-65	50-65	18729	21133
Denmark	1996-1997	Drivsholm et al., Diabet Med 18:126-32, 2001	Subnational	urban	60	60	325	370
Denmark	2002-2003	Odense Androgen Study	Community	urban	20-29		783	
Denmark	2006-2008	The Health2006 Cohort	Community	urban	18-71	18-71	1553	1916
Denmark	2007-2008	The Danish Health Examination Survey 2007-2008	National	both	18+	18+	7349	10648
Denmark	2009-2010	The European Youth Heart Study	Community	both	18-28	18-28	305	333
Dominica	2007	STEPS	National	both	18-64	18-64	417	526
Dominican Republic	1991	DHS	National	both		18-49		2112
Dominican Republic	1993	Aono et al., J Epidemiol 7(4):238-43, 1997	National	both	20-70	20-70	767	1149
Dominican Republic	1996	DHS	National	both		18-49		6431
Dominican Republic	1996-1998	Estudio De Los Factores De Riesgo Cardiovascular Y Sindrome Metabolico En La Republica Dominicana	National	both	18-85	18-85	1349	2613
Dominican Republic	2013	DHS	National	both	18-59	18-49	9281	7906
DR Congo	2001	Multiple Indicator Cluster Survey Round 2	National	both		18-49		5399
DR Congo	2005	STEPS	Community	urban	18+	18+	677	1031
DR Congo	2007	DHS	National	both		18-49		3614
DR Congo	2013-2014	DHS	National	both		18-49		7034
Ecuador	2003-2004	Garcia et al., Int J Cardiol 110:263-4, 2006	Community	urban	25-64		166	
Ecuador	2004	Ecuador Encuesta Demografica y de Salud Materna e Infantil	National	both		18-50		3533
Ecuador	2004-2005	CArdiovascular Risk factors Multiple Evaluation in Latin America	Community	urban	25-64	25-64	813	814
Ecuador	2011-2013	Encuesta Nacional de Salud y Nutricion	National	both	18-59	18-59	13188	17273
Egypt	1991-1993	The Egyptian National Hypertension Study	National	both	25+	25+	970	1322
Egypt	1992	DHS	National	both		18-49		4818
Egypt	1995	DHS	National	both		18-49		6705
Egypt	2000	DHS	National	both		18-49		13906
Egypt	2002	National Survey of Smoking, Obesity, Blood Pressure and Blood Glucose	National	both	18+	18+	2913	3643
Egypt	2003	DHS	National	both		18-49		8134
Egypt	2003-2004	Marzouk et al., Gut 56(8):1105-10, 2007	Community	rural	25+	25+	322	456
Egypt	2005	DHS	National	both		18-49		17299
Egypt	2005	STEPS	National	both	18-65	18-65	4273	4032
Egypt	2007-2009	Mostafa et al., Gut 59(8):1135-40, 2010	Community	rural	35+	35+	642	843
Egypt	2008	DHS	National	both	18-59	18-49	6480	17059
Egypt	2011	STEPS	National	both	18-65	18-65	1659	2842
Egypt	2014	DHS	National	both		18-49		19241
El Salvador	2002-2003	Ecuesta Nacional de Salud Familiar	National	both		18-49		3748
El Salvador	2004	CAMDI	Community	urban	20+	20+	396	811
El Salvador	2008	Ecuesta Nacional de Salud Familiar	National	both		18-49		6283
Equatorial Guinea	2011	DHS	National	both		18-49		1074
Eritrea	1995	DHS	National	both		18-49		1621
Eritrea	2002	DHS	National	both		18-49		3223
Eritrea	2004	STEPS	National	both	18-64	18-64	1044	1025
Eritrea	2010	STEPS	National	both	25-74	25-74	1712	4283
Estonia	1984-1986	Abina et al., Blood Press 12:111-21, 2003	Community	urban	20-54	30-54	2477	851
Estonia	1992-1994	Abina et al., Blood Press 12:111-21, 2003	Community	urban	20-54	20-54	921	678
Estonia	1997	Pomerleau et al., Public Health Nutrition 3: 3-10, 2000	National	both	19-64	19-64	525	629
Estonia	1999-2001	Abina et al., Blood Press 12:111-21, 2003	Community	urban	20-54	20-54	635	692
Estonia	2002	Estonian Biobank	National	both	18+	18+	89	217
Estonia	2003	The European Male Ageing Study	Community	both	40+		416	
Estonia	2003	Estonian Biobank	National	both	18+	18+	2695	5688
Estonia	2004	Estonian Biobank	National	both	18+	18+	527	947
Estonia	2007	Estonian Biobank	National	both	18+	18+	1000	2187

Country	Data years	Survey/study name	Level of representativness		Age range as used for global analysis		Sample size	
				Rural, urban, or both	Male	Female	Male	Female
Estonia	2008	The European Male Ageing Study	Community	both	40+		305	
Estonia	2008	Estonian Biobank	National	both	18+	18+	5147	10990
Estonia	2009	Estonian Biobank	National	both	18+	18+	3963	6493
Estonia	2010	Estonian Biobank	National	both	18+	18+	4052	7045
Estonia	2011	Estonian Biobank	National	both	18+	18+	111	176
Estonia	2012	Estonian Biobank	National	both	18+	18+	85	130
Estonia	2013	Estonian Biobank	National	both	18+	18+	106	143
Ethiopia	2000	DHS	National	both		18-49		11776
Ethiopia	2005	DHS	National	both		18-49		5248
Ethiopia	2006	STEPS	Community	urban	25-64	25-64	1643	2309
Ethiopia	2011	DHS	National	both	18-59	18-49	12625	12836
Fiji	2002	STEPS	National	both	18-64	18-64	2498	3605
Fiji	2005-2007	Pacific Obesity Prevention in Communities - Ma'alahi Youth Project	Subnational	both	18-19	18-19	288	329
Fiji	2007-2008	Pacific Obesity Prevention in Communities - Ma'alahi Youth Project	Subnational	both	18-22	18-22	398	458
Fiji	2009	Fiji Eye Health Survey 2009	National	both	40+	40+	582	776
Fiji	2011	STEPS	National	both	25-64	25-64	1124	1416
Finland	1980	Young Finns Study 1980	National	rural	18-18	18-18	121	149
Finland	1980	Young Finns Study 1980	National	urban	18-18	18-18	131	130
Finland	1982	MONICA, North Karelia/Kuopio/Turku/Loimaa	Subnational	both	25-64	25-64	4550	4659
Finland	1983	Young Finns Study 1983	National	rural	18-21	18-21	166	198
Finland	1983	Young Finns Study 1983	National	urban	18-21	18-21	184	204
Finland	1984	Finnish cohort of the FINE study	Community	rural	65-84		673	
Finland	1984-1989	Kuopio Ischaemic Heart Disease Risk factor Study	Subnational	both	42-61		2670	
Finland	1985	INTERSALT, Turku	Community	urban	20-59	20-59	100	100
Finland	1985-1986	INTERSALT, Joensuu	Community	urban	20-59	20-59	100	100
Finland	1986	Young Finns Study 1986	National	rural	18-24	18-24	205	236
Finland	1986	Young Finns Study 1986	National	urban	18-24	18-24	262	331
Finland	1987	MONICA, North Karelia/Kuopio/Turku/Loimaa	Subnational	both	25-64	25-64	2896	3151
Finland	1989	Finnish cohort of the FINE study	Community	rural	70-89		446	
Finland	1991-1993	Kuopio Ischaemic Heart Disease Risk factor Study	Subnational	both	46-64		1037	
Finland	1992	The National FINRISK Study	Subnational	both	25-64	25-64	2849	3201
Finland	1994	Finnish cohort of the FINE study	Community	rural	75-94		266	
Finland	1997	The National FINRISK Study	National	both	25-75	25-74	4128	4131
Finland	1998-2001	Kuopio Ischaemic Heart Disease Risk factor Study	Subnational	both	53-73	53-73	854	920
Finland	2000	Finnish cohort of the FINE study	Community	rural	81-96		92	
Finland	2000-2001	Health Survey	National	both	30+	30+	3247	3928
Finland	2001	Young Finns Study 2001	National	rural	24-39	24-39	346	393
Finland	2001	Young Finns Study 2001	National	urban	24-39	24-39	658	769
Finland	2002	The National FINRISK Study	National	both	25-74	25-74	3299	3826
Finland	2004-2005	FIN-D2D	Subnational	both	45-74	45-74	1364	1461
Finland	2005	Mantyselka et al., Rheumatology (Oxford) 47(8):1235-38, 2008	Community	rural	30-65	30-65	230	241
Finland	2005-2008	Kuopio Ischaemic Heart Disease Risk factor Study	Subnational	both	62-82	60-82	1241	634
Finland	2007	The National FINRISK Study	National	both	25-74	25-74	2934	3323
Finland	2007	Young Finns Study 2007	National	rural	30-45	30-45	374	431
Finland	2007	Young Finns Study 2007	National	urban	30-45	30-45	602	714
Finland	2011	Young Finns Study 2011	National	rural	34-49	34-49	364	424
Finland	2011	Young Finns Study 2011	National	urban	34-49	34-49	506	636
Finland	2011-2012	Health Survey	National	both	30+	30+	2587	3180
Finland	2012	The National FINRISK Study	National	both	25-74	25-74	2774	3052
France	1985-1987	MONICA, Strasbourg	Subnational	both	35-64	35-64	664	713
France	1985-1987	MONICA, Strasbourg	Community	both	25-34	25-34	65	78
France	1985-1987	MONICA, Toulouse	Subnational	both	35-64	35-64	675	644
France	1986-1989	MONICA, Lille	Community	urban	25-64	25-64	878	732
France	1988-1991	MONICA, Toulouse	Subnational	both	35-64		586	
France	1994-1996	MONICA, Toulouse	Subnational	both	35-64	35-64	608	566
France	1995-1997	MONICA, Lille	Community	urban	36-67	36-67	598	590
France	1995-1997	MONICA, Strasbourg	Subnational	both	35-64	35-64	526	523
France	1996-2003	Jaquet et al., Diabetologia 48(5):849-55, 2005	Community	urban	18-34	18-34	173	164
France	1999-2001	The Three city Study	Community	urban	65+	65+	2423	3778
France	2004-2006	National Monitoring of Arterial Risk in Lille (MONA LISA Lille)	Community	urban	35-75	35-75	783	795
France	2005-2007	National Monitoring of Arterial Risk in Bas-Rhin (MONA LISA Bas-Rhin)	Community	both	35-74	35-74	780	787

Country	Data years	Survey/study name	Level of representativness		Age range as used for global analysis		Sample size	
				Rural, urban, or both	Male	Female	Male	Female
France	2006-2007	Etude Nationale Nutrition Santé	National	both	18-74	18-74	876	1512
France	2006-2008	The Three city Study	Community	urban	72+	72+	768	1217
France	2011-2013	Enquête LIttorale Souffle Air Biologie EnvironnemenT (ELISABET) Dunkerque	Community	urban	40-64	40-64	761	812
France	2011-2013	Enquête LIttorale Souffle Air Biologie EnvironnemenT (ELISABET) Lille	Community	urban	40-64	40-64	758	857
French Polynesia	2010	STEPS	National	both	18-64	18-64	1458	1916
Gabon	2000	DHS	National	both		18-49		2314
Gabon	2009	STEPS	Community	urban	18-64	18-64	1009	1443
Gabon	2012	DHS	National	both		18-49		4365
Gambia	1996-1997	National Survey of Blindness and Low Vision	National	both	18+	18+	1733	2071
Gambia	2003	Siervo et al., Eur J Clin Nutr 60(4):455-63, 2006	Community	urban	18-50	18-50	50	50
Gambia	2010	STEPS	National	both	25-64	25-64	1610	1917
Gambia	2013	DHS	National	both		18-49		3605
Georgia	2010	STEPS	National	both	18-64	18-64	1842	4460
Germany	1982	MONICA, Erfurt	Community	urban	25-64	25-64	106	103
Germany	1982-1984	MONICA, Chemnitz	Community	urban	25-64	25-64	267	295
Germany	1982-1984	MONICA, Zwickau	Community	urban	25-64	25-64	246	276
Germany	1982-1985	MONICA, Rest of Karl-Marx-Stadt County	Subnational	urban	25-64	25-64	592	657
Germany	1982-1985	MONICA, Rest of DDR-MONICA	Subnational	urban	25-64	25-64	235	232
Germany	1983-1984	MONICA, Halle County	Subnational	urban	25-64	25-64	1110	1172
Germany	1983-1987	MONICA, Rhein-Neckar Region	Community	urban	35-64	35-64	1489	1609
Germany	1984	German Cardiovascular Prevention Study (GCP) - National Health Survey 1984	Subnational	both	25-69	25-69	2415	2366
Germany	1984	MONICA, Bremen North/West	Community	urban	25-64	25-64	813	852
Germany	1984-1985	MONICA, Berlin-Lichtenberg	Community	urban	25-64	25-64	593	635
Germany	1984-1985	MONICA, Augsburg	Community	both	25-64	25-64	2005	1961
Germany	1984-1986	MONICA, Cottbus County	Community	urban	25-64	25-64	657	739
Germany	1985-1986	INTERSALT, Cottbus	Community	urban	20-59	20-59	99	99
Germany	1985-1986	INTERSALT, Heidelberg	Community	urban	20-59	20-59	97	99
Germany	1985-1986	CINDI	Subnational	both	25-64	25-64	1875	1990
Germany	1985-1986	INTERSALT, Bernried	Community	urban	20-59	20-59	99	98
Germany	1987-1988	MONICA, Erfurt	Community	urban	25-64	25-64	871	909
Germany	1988	German Cardiovascular Prevention Study (GCP) - National Health Survey 1988	Subnational	both	25-69	25-69	2642	2678
Germany	1988	MONICA, Berlin-Lichtenberg	Community	urban	25-64	25-64	690	728
Germany	1988	MONICA, Bremen North/West	Community	urban	25-69	25-69	619	632
Germany	1988	MONICA, Bremen Center/South/East	Community	urban	25-69	25-69	499	582
Germany	1988	MONICA, Chemnitz	Community	urban	25-64	25-64	288	382
Germany	1988	MONICA, Zwickau	Community	urban	25-64	25-64	193	250
Germany	1988-1989	CINDI	Subnational	both	25-64	25-64	1361	1435
Germany	1988-1989	MONICA, Halle County	Subnational	urban	25-64	25-64	959	1201
Germany	1988-1989	MONICA, Rest of Karl-Marx-Stadt County	Subnational	urban	25-64	25-64	541	626
Germany	1989-1990	MONICA, Cottbus County	Community	urban	25-64	25-64	539	529
Germany	1989-1990	MONICA, Augsburg	Community	both	25-64	25-64	1933	1944
Germany	1990-1992	European Community Respiratory Health Survey, Hamburg	Community	urban	20-47	20-47	146	138
Germany	1990-1992	European Community Respiratory Health Survey, Erfurt	Community	urban	20-47	20-47	146	124
Germany	1991-1992	MONICA, Bremen North/West	Community	urban	25-69	25-69	599	671
Germany	1991-1992	MONICA, Bremen Center/South/East	Community	urban	25-69	25-69	524	546
Germany	1991-1992	CINDI	Subnational	both	25-64	25-64	1326	1400
Germany	1991-1992	German Cardiovascular Prevention Study (GCP) - National Health Survey 1991	Subnational	both	25-69	25-69	2599	2670
Germany	1991-1992	First National Examination of life conditions, Environment and Health in East Germany 1991/92	Subnational	both	25-69	25-69	1042	1155
Germany	1991-1992	MONICA, Erfurt	Community	urban	25-64	25-64	587	572
Germany	1993-1994	MONICA, Chemnitz	Community	urban	25-64	25-64	408	424
Germany	1993-1994	MONICA, Zwickau	Community	urban	25-64	25-64	139	186
Germany	1994-1995	MONICA, Augsburg	Community	both	25-64	25-64	1898	1968
Germany	1994-1998	EPIC Heidelberg	Community	urban	40-64	35-64	11680	13458
Germany	1994-1998	EPIC Potsdam	Community	urban	40-64	35-64	10224	15995
Germany	1997-1999	German National Health Interview and Examination Survey	National	both	18-79	18-79	3435	3608
Germany	1997-2001	Study of Health in Pomerania (SHIP-0) baseline study	Subnational	both	20-80	20-80	2111	2187
Germany	1999-2001	KORA S4 Study: Kooperative Research in the Region of Augsburg Survey 4	Subnational	both	24-75	24-75	2076	2148
Germany	2000-2001	European Community Respiratory Health Survey, Hamburg	Community	urban	30-57	30-57	146	138
Germany	2000-2001	European Community Respiratory Health Survey, Erfurt	Community	urban	30-57	30-57	146	124
Germany	2000-2002	Epidemiological study of the chances of prevention, early recognition and optimal treatment of chronic diseases in an elderly population (ESTHER)	Subnational	both	50-75	50-75	4420	5361
Germany	2000-2003	Heinz Nixdorf RECALL Study	Community	urban	45-74	45-74	2375	2393

Country	Data years	Survey/study name	Level of representativness	Rural, urban, or both	Age range as used for global analysis		Sample size	
					Male	Female	Male	Female
Germany	2002	Echinoccoccus Multilocularis and Internal Diseases in Leutkirch	Community	urban	18-65	18-65	840	950
Germany	2002-2006	Study of Health in Pomerania (SHIP-1) 5-year follow-up	Subnational	both	25-85	25-85	1583	1707
Germany	2006-2008	KORA F4 Study: Kooperative Research in the Region of Augsburg Follow-Up of Survey 4	Subnational	both	31-81	31-81	1480	1583
Germany	2008-2011	Epidemiological study of the chances of prevention, early recognition and optimal treatment of chronic diseases in an elderly population (ESTHER)	Subnational	both	58-84	58-84	1468	1622
Germany	2008-2011	German Health Interview and Examination Survey for adults 2008-11 (DEGS1)	National	both	18-79	18-79	3389	3650
Ghana	1993	DHS	National	both		18-49		1754
Ghana	1998	DHS	National	both		18-49		2053
Ghana	2001	Addo et al., Ethn Dis 16(4):894-99, 2006	Community	rural	18+	18+	89	206
Ghana	2001-2002	Cappuccio et al., Hypertension 43(5):1017-22, 2004	Community	both	35-84	35-84	194	338
Ghana	2002	Amoah et al., Ethn Dis 13(2 Suppl 2):S97-101, 2003	Community	both	25+	25+	1859	2947
Ghana	2003	Women's Health Study of Accra	Community	urban		18+		1328
Ghana	2003	DHS	National	both		18-49		4311
Ghana	2006	STEPS	Community	urban	25+	25+	841	1634
Ghana	2007-2008	SAGE	National	both	50+	50+	2192	1987
Ghana	2008	DHS	National	both		18-49		3884
Ghana		Amoah, Public Health Nutr 6(8):751-7,2003	Community	both	25+	25+	1857	2875
Greece	1991	Seven Countries Study	Subnational	both	70-89		177	
Greece	1991-1999	EPIC	National	both	19-86	19-86	11578	16477
Greece	2000-2001	Karalis et al., BMC Public Health7:351, 2007	Community	rural	18+	18+	73	87
Greece	2003	National Epidemiological Survey	National	both	18-19	18-19	987	1150
Greece	2010-2012	ADONUT	National	both	18-19	18-19	1531	1641
Greenland	2005-2010	Population Health Survey in Greenland	National	both	18+	18+	1336	1714
Grenada	2011	STEPS	National	both	25-64	25-64	438	636
Guatemala	1995	DHS	National	both		18-49		4864
Guatemala	1998-1999	DHS	National	both		18-49		2322
Guatemala	2001-2002	CAMDI	Community	urban	20+	20+	293	638
Guatemala	2002	Guatemala Reproductive Health Survey	National	both	18-60	18-50	1775	6049
Guatemala	2003-2005	The Institute of Nutrition of Central America and Panama Nutrition Supplementation Trial Cohort	Community	both	25-41	25-41	268	288
Guatemala	2008-2009	Encuesta Nacional de Salud Materno Infantil	National	both		18-49		13669
Guinea	1999	DHS	National	both		18-49		3233
Guinea	2005	DHS	National	both		18-49		3091
Guinea	2009	STEPS	Subnational	both	18-64	18-64	1044	1119
Guinea	2012	DHS	National	both		18-49		3624
Guinea Bissau	2010	Multiple Indicator Cluster Survey	National	both		18-49		6654
Guyana	2009	DHS	National	both	18-49	18-49	2954	4009
Haiti	1994-1995	DHS	National	both		18-49		1868
Haiti	2000	DHS	National	both		18-49		7781
Haiti	2005-2006	DHS	National	both		18-49		4192
Haiti	2012	DHS	National	both		18-49		7565
Honduras	1996	Honduras National Micronutrient Survey	National	both		20-40		722
Honduras	2003-2004	CAMDI	Community	urban	20+	20+	428	764
Honduras	2005-2006	DHS	National	both		18-49		15463
Honduras	2011-2012	DHS	National	both		18-49		18078
Hungary	1982-1983	MONICA, Pecs	Community	urban	35-64	35-64	823	861
Hungary	1982-1984	MONICA, Budapest	Community	urban	35-64	35-64	774	737
Hungary	1985	INTERSALT	Community	rural	20-59	20-59	100	100
Hungary	1985-1988	First Hungarian Representative Nutrition Survey	National	both	18+	18+	3079	8916
Hungary	1987-1988	MONICA, Budapest	Community	urban	35-64	35-64	1413	1594
Hungary	1987-1988	MONICA, Pecs	Community	urban	35-64	35-64	1573	1510
Hungary	1992-1994	Nutrition survey of the Hungarian population in a Randomized Trial between 1992-1994	National	both	18-35	18-35	388	1281
Hungary	2003	The European Male Ageing Study	Community	both	40+		428	
Hungary	2008	The European Male Ageing Study	Community	both	40+		349	
Iceland	1983	MONICA, Arnes County	Community	rural	25-64	25-64	388	450
Iceland	1983	MONICA, Reykjavik	Subnational	urban	25-64	25-64	434	461
Iceland	1985-1986	INTERSALT	Community	urban	20-59	20-59	100	100
Iceland	1988-1989	MONICA, Arnes County	Community	rural	25-64	25-64	385	435
Iceland	1988-1989	MONICA, Reykjavik	Subnational	urban	25-64	25-64	414	443
Iceland	1993-1994	MONICA, Arnes County	Community	rural	25-64	25-64	422	484
Iceland	1993-1994	MONICA, Reykjavik	Subnational	urban	25-64	25-64	441	448
India	1975-1979	Diet and Nutritional status of Rural population and Prevalnce of Hypertension	National	rural	18+	18+	22574	17782
India	1986	INTERSALT	Community	urban	20-59	20-59	100	99

Country	Data years	Survey/study name	Level of representativness		Age range as used for global analysis		Sample size	
				Rural, urban, or both	Male	Female	Male	Female
India	1988-1989	Rmachandran et al., Diabetes Res Clin Pract 58(1):55-60, 2002	Community	urban	20-74	20-74	455	437
India	1988-1990	NNMB survey	Subnational	rural	20+	20+	9447	11914
India	1991-1994	Prabhakaran et al., Chronic Illn 3(1):8-19, 2007	Community	rural	35-64	35-64	542	630
India	1991-1994	Prabhakaran et al., Chronic Illn 3(1):8-19, 2007	Community	urban	35-64	35-64	1388	1455
India	1991-1995	Reddy et al., Obes Rev 3(3):197-202, 2002	Community	rural	35-64	35-64	1070	1332
India	1991-1995	Reddy et al., Obes Rev 3(3):197-202, 2002	Community	urban	35-64	35-64	1456	1594
India	1991-1997	Mumbai Cohort Study	Community	urban	35+	35+	88658	59515
India	1992-1994	JHW-Rural	Community	rural	20-59	20-59	1700	1063
India	1993-1994	Khongsdier, Eur J Clin Nutr 56(6):484-89, 2002	Community	both	18-59		575	
India	1993-1995	JHW-1	Community	urban	20-59	20-59	1294	655
India	1995	Shobana et al., Diabetes Res Clin Pract 42(3):181–86, 1998	Community	urban	20-74	20-74	1061	1093
India	1995-1996	Kusuma et al., Ann Hum Biol 29(5):502-12, 2002	Community	both	18-84	18-84	747	737
India	1995-1996	Epidemiology of blood pressure across cross-cultural populations of Visakhapatnam district, Andhra Pradesh, India	Community	rural	19-76	19-76	209	228
India	1995-1997	Aravind Comprehensive Eye Survey	Community	rural	40-90	40-90	2308	2830
India	1995-1997	Kashmiri Adults	Subnational	both	40+	40+	2496	2587
India	1996-1997	Diet and Nutritional status of Rural population and Prevalnce of Hypertension	National	rural	18+	18+	12735	17890
India	1996-1999	Chennai Urban Population Study	Community	urban	20+	20+	557	705
India	1997	Ramachandran et al., Diabetes Res Clin Pract 44(3):207–13, 1999	Community	rural	20-74	20-74	738	879
India	1998-1999	DHS	National	both		18-49		75599
India	1998-2001	Chennai Prospective Study	Community	urban	35+	35+	264848	235968
India	1999-2001	JHW-2	Community	urban	20-59	20-59	468	486
India	1999-2002	New Delhi Birth Cohort	Community	urban	26-33	26-33	886	638
India	2000	Ramachandran et al., Diabet Med,20(3):220-24, 2003	Subnational	urban	20-75	20-75	4640	5257
India	2001	Diet and Nutritional status of Rural population and Prevalnce of Hypertension	National	rural	18+	18+	11074	17317
India	2001-2004	Chennai Urban Rural Epidemiology Study	Community	urban	20+	20+	1094	1254
India	2002-2003	JHW-3	Community	urban	20-59	20-59	179	195
India	2002-2003	Blood Pressure epidemiology in tribal, rural and urban communities of Orissa with special reference to physical and social parameters	Community	rural	18-80	18-80	200	186
India	2003-2005	WHO-ICMR NCD risk factor surveillance study	National	rural	18-69	18-69	6266	6478
India	2003-2005	WHO-ICMR NCD risk factor surveillance study	National	urban	18-69	18-69	7164	7296
India	2004-2005	JHW-4	Community	urban	20-59	20-59	413	473
India	2005-2006	DHS	National	both	18-54	18-49	64045	102354
India	2005-2006	Diet and Nutritional status of Rural population and Prevalnce of Hypertension	National	rural	18+	18+	14036	18598
India	2005-2007	Prevalence of cardiovascular risk factors in rural Tamil Nadu	Community	rural	25-65	25-65	4927	5573
India	2006	Ramachandran et al., Diabetes Care 31(5):893-98, 2008	Community	both	20+	20+	3321	3745
India	2006-2007	Kusuma et al., Asia Pac J Public Health 21(4):497-507, 2009	Community	urban	18-74	18-74	182	192
India	2006-2008	Central India Eye and Medical Study	Community	rural	30+	30+	2190	2520
India	2006-2008	Kashmiri Young Adults	Subnational	both	20-40	20-40	2119	905
India	2006-2009	New Delhi Birth Cohort	Community	urban	33-38	33-38	650	445
India	2007-2008	Integrated Disease Surveillance Project Non-communicable Disease Risk Factors Survey	Subnational	both	18-64	18-64	2494	3196
India	2007-2008	Integrated Disease Surveillance Project Non-communicable Disease Risk Factors Survey	Subnational	both	18-64	18-64	1525	2931
India	2007-2008	Integrated Disease Surveillance Project Non-communicable Disease Risk Factors Survey	Subnational	both	18-64	18-64	2489	2641
India	2007-2008	Integrated Disease Surveillance Project Non-communicable Disease Risk Factors Survey	Subnational	both	18-64	18-64	2767	2701
India	2007-2008	Integrated Disease Surveillance Project Non-communicable Disease Risk Factors Survey	Subnational	both	18-64	18-64	2023	1884
India	2007-2008	Integrated Disease Surveillance Project Non-communicable Disease Risk Factors Survey	Subnational	both	18-64	18-64	1895	2773
India	2007-2008	Integrated Disease Surveillance Project Non-communicable Disease Risk Factors Survey	Subnational	both	18-64	18-64	1821	2827
India	2007-2008	SAGE	National	both	50+	50+	3214	3149
India	2007-2009	Prevalence of NCD risk factor in people above 15 year in Rural area Nagpur using WHO STEP approach	Community	rural	18+	18+	1828	1681
India	2008-2010	ICMR India Diabetes Study	National	both	20+	20+	6959	6847
India	2009-2010	Baseline Survey for the assessment of prevalence of risk factors of NCDs in Gandhinagar District	Community	rural	18-64	18-64	679	654
India	2009-2010	Baseline Survey for the assessment of prevalence of risk factors of NCDs in Gandhinagar District	Community	urban	18-64	18-64	696	723
India	2011-2012	Diet and Nutritional status of Rural population and Prevalnce of Hypertension	National	rural	18+	18+	24079	32208
Indonesia	1983-1987	Strickland et al., Eur J Clin Nutr 48 Suppl 3: S98-108; discussion S-9, 1994	Community	both	18+	18+	447	564
Indonesia	1993-1994	Indonesian Family Life Surveys	National	both	18+	18+	5739	7059
Indonesia	1997-1998	Indonesian Family Life Surveys	National	both	18+	18+	7712	9482
Indonesia	2000-2001	Indonesian Family Life Surveys	National	both	18+	18+	10549	11431
Indonesia	2001	Ng et al., Bull World Health Organ 84(4):305-13, 2006	Community	both	18-74	18-74	1261	1234
Indonesia	2001	STEPS/SURKESNAS	National	both	18-64	18-64	4100	4775
Indonesia	2006	Jakarta Non Communicable Disease Risk Factor Surveillance	Community	urban	25-64	25-64	641	950
Indonesia	2007-2008	Indonesian Family Life Surveys	National	both	18+	18+	12615	14101
Indonesia	2013	Population Health Basic Health Research 2013 (Riskesdas 2013)	National	both	18+	18+	314793	342182
Iran	1990-1991	National Health Survey I	National	both	18-18	18-18	284	405

Country	Data years	Survey/study name	Level of representativness		Age range as used for global analysis		Sample size	
				Rural, urban, or both	Male	Female	Male	Female
Iran	1997-1998	Khadivzadeh, East Mediterr Health J 8(4-5):612-18, 2002	Community	urban		18-49		1513.377
Iran	1999-2000	National Health Survey II	National	both	18-99	18-99	13702	16618
Iran	1999-2001	Tehran Lipid and Glucose Study	Community	urban	18+	18+	4555	6125
Iran	2000	ASADABADI Study	Community	urban	18+	18+	132	168
Iran	2001	Isfahan Healthy Heart Program, Arak rural	Community	rural	19+	19+	1023	1080
Iran	2001	Isfahan Healthy Heart Program, Arak urban	Community	urban	19+	19+	2084	2124
Iran	2001	Isfahan Healthy Heart Program, Isfahan rural	Community	rural	19+	19+	232	233
Iran	2001	Isfahan Healthy Heart Program, Isfahan urban	Community	urban	19+	19+	1760	1912
Iran	2001	Isfahan Healthy Heart Program, Najaf Abad rural	Community	rural	19+	19+	405	416
Iran	2001	Isfahan Healthy Heart Program, Najaf Abad urban	Community	urban	19+	19+	573	571
Iran	2002-2005	Tehran Lipid and Glucose Study	Community	urban	18+	18+	2153	2766
Iran	2003-2004	The Persian Gulf Healthy Heart Study	Subnational	urban	25-64	25-64	1615	1528
Iran	2003-2004	Childhood and Adolescence Surveillance and Prevention of Adult Noncommunicable Disease R1	National	both	18-18	18-18	370	362
Iran	2004	Hajian-Tilaki et al., Obes Rev 8(1):3-10, 2007	Community	urban	20-70	20-70	1800	1800
Iran	2004-2008	Golestan Cohort Study	Subnational	both	40-75	40-75	21229	28808
Iran	2005	Provincial Non-Communicable Disease Surveillance Survey 2005	National	both	18-64	18-64	37998	37520
Iran	2005-2006	Rashidy-Pour, Obes Rev (1):2-6, 2009	Subnational	both	30-70	30-70	1695	2104
Iran	2005-2008	Tehran Lipid and Glucose Study	Community	urban	18+	18+	2531	3259
Iran	2006	Provincial Non-Communicable Disease Surveillance Survey 2006	National	both	18-65	18-65	14130	14008
Iran	2007	Isfahan Healthy Heart Program, Arak rural	Community	rural	19+	19+	1028	1024
Iran	2007	Isfahan Healthy Heart Program, Arak urban	Community	urban	19+	19+	1424	1359
Iran	2007	Isfahan Healthy Heart Program, Isfahan rural	Community	rural	19+	19+	155	151
Iran	2007	Isfahan Healthy Heart Program, Isfahan urban	Community	urban	19+	19+	1309	1302
Iran	2007	Isfahan Healthy Heart Program, Najaf Abad rural	Community	rural	19+	19+	254	253
Iran	2007	Isfahan Healthy Heart Program, Najaf Abad urban	Community	urban	19+	19+	494	542
Iran	2007	Provincial Non-Communicable Disease Surveillance Survey 2007	National	both	18-64	18-64	2211	2209
Iran	2007	Provincial Non-Communicable Disease Surveillance Survey 2007	National	both	18-64	18-64	13840	13805
Iran	2008	Provincial Non-Communicable Disease Surveillance Survey 2008	National	both	18-64	18-64	13692	13601
Iran	2008-2011	Tehran Lipid and Glucose Study	Community	urban	20+	20+	4622	5884
Iran	2009	Provincial Non-Communicable Disease Surveillance Survey 2009	National	both	18-64	18-64	13919	13717
Iran	2009-2010	Childhood and Adolescence Surveillance and Prevention of Adult Noncommunicable Disease R3	National	both	18-18	18-18	497	532
Iran	2011	Provincial Non-Communicable Disease Surveillance Survey 2011	National	both	18-69	18-69	4075	5738
Iran	2011-2012	Childhood and Adolescence Surveillance and Prevention of Adult Noncommunicable Disease R4	National	both	18-18	18-18	287	269
Iraq	2006	STEPS	National	both	25-64	25-64	2251	2252
Ireland	1997-1999	North/South Ireland Food Consumption Survey	National	both	18-64	18-64	613	698
Ireland	1998	Survey of Lifestye, Attitudes and Nutritional in Ireland 1998	National	both	18+	18+	123	296
Ireland	2002	Survey of Lifestye, Attitudes and Nutritional in Ireland 2002	National	both	18+	18+	164	215
Ireland	2006-2007	Survey of Lifestye, Attitudes and Nutritional in Ireland 2006-2007	National	both	40+	18+	524	675
Ireland	2008-2010	National Adult Nutrition Survey	National	both	18+	18+	658	696
Ireland	2009-2011	The Irish Longitudinal Study on Ageing	National	both	50+	50+	2693	3170
Israel	1985-1986	MONICA, Tel Aviv	Community	urban	35-64	35-64	653	685
Israel	1990-1991	The Jerusalem Longitudinal Cohort Study	Community	urban	69-70	69-70	244	199
Israel	1997-1998	The Jerusalem Longitudinal Cohort Study	Community	urban	76-77	76-77	418	425
Israel	1999-2001	Mabat First Israeli National Health and Nutrition Survey	National	both	25-64	25-64	1371	1410
Israel	1999-2005	The Israel Glucose Intolerance, Obesity and Hypertention Study	National	urban	58-94	58-94	514	527
Israel	2002-2007	Hadera District Study	Subnational	urban	25-78	25-78	548	538
Israel	2003-2004	Mabat Youth First Israeli National Health and Nutrition Survey in 7th-12th grade students 2003-4	National	both	18-18	18-18	50	34
Israel	2005-2006	The Jerusalem Longitudinal Cohort Study	Community	urban	83-85	83-85	490	583
Israel	2005-2006	Mabat Zahav National Health and Nutrition Survey ages 65 and over 2005-6	National	both	65+	65+	743	819
Italy	1980-1982	Po river delta Epidemiological Study - first survey	Community	rural	18-64	18-64	1212	1333
Italy	1982-1987	MONICA, Latina	Community	rural	24-66	24-66	852	868
Italy	1983-1996	Malattie cardiovascolari ATerosclerotiche Istituto Superiore di Sanità	Community	rural	18-77	18-77	3948	4488
Italy	1985	Finland, Italy, Netherlands, Elderly (Fine-Italy)	Community	rural	65-84		650	
Italy	1985	INTERSALT, Naples	Community	urban	20-59	20-59	100	100
Italy	1985-1988	Pisa Epidemiological Study - first survey	Community	urban	18-90	18-90	1502	1755
Italy	1986	INTERSALT, Bassiano	Community	urban	20-59	20-59	99	100
Italy	1986	INTERSALT, Gubbio	Community	urban	20-59	20-59	99	100
Italy	1986	INTERSALT, Mirano	Community	urban	20-59	20-59	100	100
Italy	1986	MONICA, Friuli	Subnational	urban	25-64	25-64	921	918
Italy	1986-1987	MONICA, Brianza	Subnational	urban	25-64	25-64	814	832
Italy	1988-1991	Po river delta Epidemiological Study - second survey	Community	rural	18-73	18-73	1142	1307

Country	Data years	Survey/study name	Level of representativness	Rural, urban, or both	Age range as used for global analysis Male	Female	Sample size Male	Female
Italy	1989	MONICA, Friuli	Subnational	urban	25-64	25-64	902	900
Italy	1989	Ventimiglia Heart Study	Community	rural	18+	18+	486	594
Italy	1989-1990	MONICA, Brianza	Subnational	urban	25-64	25-64	787	786
Italy	1990	Bruneck Study	Community	rural	40-79	40-79	469	450
Italy	1991-1993	Pisa Epidemiological Study - second survey	Community	urban	18-97	18-97	1164	1440
Italy	1992-1993	Italian Longitudinal Study on Aging	National	both	65-84	65-84	1666	1455
Italy	1992-1998	Vobarno Study	Community	both	25-64	35-64	265	309
Italy	1993-1994	MONICA, Brianza	Subnational	urban	25-64	25-64	801	856
Italy	1993-1998	EPIC Florence	Community	urban	24-72	24-72	3498	9968
Italy	1994	MONICA, Friuli	Subnational	urban	25-64	25-64	882	888
Italy	1995	Bruneck Study	Community	rural	45-84	45-84	411	408
Italy	1995-1996	Italian Longitudinal Study on Aging	National	both	69-90	69-90	970	779
Italy	1995-1999	PROgetto Veneto Anziani	Subnational	both	65+	65+	1187	1722
Italy	1997-1999	Lucca CUORE Study	Community	urban	18-84	18-84	897	1123
Italy	1998-1999	progetto VIP	Community	both	25-74	25-74	599	600
Italy	1998-2000	InCHIANTI study	Community	both	18+	18+	560	681
Italy	1998-2002	Osservatorio Epidemiologico Cardiovascolare/Health Examination Survey	National	both	35-74	35-74	4870	4752
Italy	2000	Bruneck Study	Community	rural	50-89	50-89	331	361
Italy	2000-2001	Italian Longitudinal Study on Aging	National	both	73-93	73-93	557	473
Italy	2000-2003	PROgetto Veneto Anziani	Subnational	both	67+	67+	795	1331
Italy	2001-2003	The Study of Asti	Community	both	45-64	45-64	780	878
Italy	2002-2005	PROgetto Veneto Anziani	Subnational	both	68+	68+	621	1138
Italy	2003	The European Male Ageing Study	Community	both	40+		433	
Italy	2004-2005	Italian Project on the Epidemiolofy of Alzheimer's disease	National	both	65-84	65-84	1569	1421
Italy	2004-2005	Vobarno study	Community	both	55-74	55-74	99	113
Italy	2004-2008	Cardiolab project	National	urban	40+	40+	19152	14782
Italy	2005	Bruneck Study	Community	rural	55-93	55-93	264	307
Italy	2005-2010	Moli-sani Study	Subnational	both	35+	35+	11694	12614
Italy	2008	The European Male Ageing Study	Community	both	40+		346	
Italy	2008-2009	progetto VIP	Community	both	25-74	25-74	596	596
Italy	2008-2012	Osservatorio Epidemiologico Cardiovascolare/Health Examination Survey	National	both	35-80	35-80	4368	4332
Italy	2010	Bruneck Study	Community	rural	60-98	60-98	225	259
Italy	2010-2012	CArdiovascular risk MEtabolic syndrome LIver and Autoimmunity diseases (CA.ME.LI.A)	Community	both	18-75	18-75	477	514
Italy	2011-2012	Vobarno study	Community	both	49-62	49-62	107	143
Jamaica	1993	Zohoori et al., West Indian Med J 52(2):111-17, 2003	Community	urban	25-74	25-74	845	1245
Jamaica	1994-1995	Cooper et al., Am J Public Health 87(2):160-68, 1997	Community	urban	25-100	25-100	597	833
Jamaica	1998	Ragoobirsingh et al., Diabetes Obes Metab 6(1):23–27, 2004	National	both	18+	18+	552	945
Jamaica	2000-2001	Jamaica Health and Lifestyle Survey	National	both	18-74	18-74	608	1231
Jamaica	2006-2007	Jamaica Youth Risk and Resiliency Behaviour Survey 2006	National	both	18-19	18-19	150	188
Jamaica	2007-2008	Jamaica Health and Lifestyle Survey	National	both	18-74	18-74	821	1822
Japan	1975	National Nutrition Survey	National	both	18-99	18-99	4611	6628
Japan	1976	National Nutrition Survey	National	both	18-99	18-99	5521	7789
Japan	1977	National Nutrition Survey	National	both	18-99	18-99	4434	6240
Japan	1978	National Nutrition Survey	National	both	18-99	18-99	4782	6613
Japan	1979	National Nutrition Survey	National	both	18-99	18-99	5068	6923
Japan	1980	National Nutrition Survey	National	both	18-99	18-99	5806	7337
Japan	1980-1983	Aito Town Study	Community	rural	20-77	20-77	741	970
Japan	1981	National Nutrition Survey	National	both	18-99	18-99	4126	6034
Japan	1982	National Nutrition Survey	National	both	18-99	18-99	4755	6709
Japan	1983	National Nutrition Survey	National	both	18-99	18-99	4579	6548
Japan	1984	National Nutrition Survey	National	both	18-99	18-99	4537	6286
Japan	1985	INTERSALT, Osaka	Community	urban	20-59	20-59	100	97
Japan	1985	INTERSALT, Tochigi	Community	urban	20-59	20-59	95	99
Japan	1985	INTERSALT, Toyama	Community	urban	20-59	20-59	100	100
Japan	1985	National Nutrition Survey	National	both	18-99	18-99	5383	6869
Japan	1985-1986	Akabane Study	Community	urban	40-69	40-69	812	1022
Japan	1986	National Nutrition Survey	National	both	18-99	18-99	5275	6741
Japan	1987	Konan Town Study	Community	rural	20-79	20-79	70	88
Japan	1987	National Nutrition Survey	National	both	18-99	18-99	4719	6485
Japan	1988	Konan Town Study	Community	rural	20-79	20-79	76	85
Japan	1988	National Nutrition Survey	National	both	18-99	18-99	5126	6397

Country	Data years	Survey/study name	Level of representativness		Age range as used for global analysis		Sample size	
				Rural, urban, or both	Male	Female	Male	Female
Japan	1989	Aito Town Study	Community	rural	18-74	18-84	529	525
Japan	1989	Konan Town Study	Community	rural	20-79	20-79	58	63
Japan	1989	National Nutrition Survey	National	both	18-99	18-99	4325	5571
Japan	1990	Konan Town Study	Community	rural	20-79	20-79	27	51
Japan	1990	National Nutrition Survey	National	both	18-99	18-99	4583	5928
Japan	1991	Konan Town Study	Community	rural	20-79	20-79	93	116
Japan	1991	Shigaraki Town Study	Community	rural	30-89	30-89	230	319
Japan	1991	National Nutrition Survey	National	both	18-99	18-99	4667	5860
Japan	1992	Konan Town Study	Community	rural	20-79	20-79	45	47
Japan	1992	Shigaraki Town Study	Community	rural	30-89	30-89	288	385
Japan	1992	National Nutrition Survey	National	both	18-99	18-99	4348	5458
Japan	1993	Konan Town Study	Community	rural	20-79	20-79	54	65
Japan	1993	Shigaraki Town Study	Community	rural	30-89	30-89	301	452
Japan	1993	National Nutrition Survey	National	both	18-99	18-99	4414	5519
Japan	1994	Konan Town Study	Community	rural	20-79	20-79	43	59
Japan	1994	Shigaraki Town Study	Community	rural	30-89	30-89	251	336
Japan	1994	National Nutrition Survey	National	both	18-99	18-99	4303	5318
Japan	1995	Konan Town Study	Community	rural	20-79	20-79	45	61
Japan	1995	Shigaraki Town Study	Community	rural	30-89	30-89	300	470
Japan	1995	National Nutrition Survey	National	both	18-99	18-99	4326	5286
Japan	1996	Shigaraki Town Study	Community	rural	30-89	30-89	86	152
Japan	1996	National Nutrition Survey	National	both	18-99	18-99	4303	5230
Japan	1996-1997	International collaborative study of macronutrients, micronutrients and blood pressure	Community	both	40-59	40-59	130	129
Japan	1997	Shigaraki Town Study	Community	rural	30-89	30-89	61	100
Japan	1997	National Nutrition Survey	National	both	18-99	18-99	4173	5161
Japan	1997-1998	International collaborative study of macronutrients, micronutrients and blood pressure	Community	urban	40-59	40-59	149	148
Japan	1997-1998	International collaborative study of macronutrients, micronutrients and blood pressure	Community	urban	40-59	40-59	149	150
Japan	1997-1998	International collaborative study of macronutrients, micronutrients and blood pressure	Community	urban	40-59	40-59	146	144
Japan	1997-2000	Sudo et al., J Orthop Sci 13(5):413-18, 2008	Community	rural	55+	45+	261	785
Japan	1998	Niigata Study	Community	both	70	70	287	284
Japan	1998	National Nutrition Survey	National	both	18-99	18-99	4407	5327
Japan	1999	Niigata Study	Community	both	71	71	245	216
Japan	1999	National Nutrition Survey	National	both	18-99	18-99	3610	4635
Japan	2000	Niigata Study	Community	both	72	72	233	202
Japan	2000	National Nutrition Survey	National	both	18-99	18-99	3863	4612
Japan	2001	The Japan Association of Health Service Database	Subnational	both	20+	20+	1471868	1231378
Japan	2001	Niigata Study	Community	both	73	73	235	201
Japan	2001	National Nutrition Survey	National	both	18-99	18-99	3708	4675
Japan	2002	Niigata Study	Community	both	74	74	228	202
Japan	2002	National Nutrition Survey	National	both	18-99	18-99	3472	4279
Japan	2002-2003	The Hisayama Study	Community	both	40+	40+	1414	1884
Japan	2003	National Health and Nutrition Survey	National	both	18-99	18-99	3432	4307
Japan	2003	Niigata Study	Community	both	75	75	215	189
Japan	2004	National Health and Nutrition Survey	National	both	18-99	18-99	2816	3468
Japan	2004	Niigata Study	Community	both	76	76	215	185
Japan	2005	National Health and Nutrition Survey	National	both	18-99	18-99	2698	3315
Japan	2005	Niigata Study	Community	both	77	77	203	184
Japan	2006	National Health and Nutrition Survey	National	both	18-99	18-99	2964	3641
Japan	2006	Niigata Study	Community	both	78	78	199	194
Japan	2007	National Health and Nutrition Survey	National	both	18-99	18-99	2990	3607
Japan	2007	Niigata Study	Community	both	79	79	183	192
Japan	2008		Community	urban	40+	40+	6562	11944
Japan	2008	National Health and Nutrition Survey	National	both	18-99	18-99	3059	3740
Japan	2008	Niigata Study	Community	both	80	80	174	180
Japan	2009	National Health and Nutrition Survey	National	both	18-99	18-99	2979	3685
Japan	2010	National Health and Nutrition Survey	National	both	18-99	18-99	2770	3410
Japan	2011	National Health and Nutrition Survey	National	both	18-99	18-99	2620	3178
Japan	2011	The Tokyo Health Service Association Database	Community	urban	20+	20+	82453	54028
Japan	2012	National Health and Nutrition Survey	National	both	18-99	18-99	9817	12182
Japan		Japanese Population-Based Osteoporosis Study	Subnational	both		18-79		3222
Jordan	1994-1996	Ajlouni, Int J Obes Relat Metab Disord 22(7), 1998	Subnational	both	25+	25+	1047	1787

Country	Data years	Survey/study name	Level of representativness		Age range as used for global analysis		Sample size	
				Rural, urban, or both	Male	Female	Male	Female
Jordan	1997	DHS	National	both		18-49		3067
Jordan	2002	DHS	National	both		18-49		4910
Jordan	2004	Khader et al., Metab Syndr Relat Disord 6(2):113-20, 2008	Community	both	25+	25-59	394	548
Jordan	2004	Behavioural Risk Factor Surveillence Survey	National	both	18+	18+	236	473
Jordan	2007	Behavioural Risk Factor Surveillence Survey	National	both	18+	18+	332	433
Jordan	2007	DHS	National	both		18-49		4504
Jordan	2009	DHS	National	both		18-49		4112
Jordan	2009	Metablic abnomalities and vitamin D study	National	both	18+	18+	1144	3350
Jordan	2012	DHS	National	both		18-49		6434
Kazakhstan	1995	DHS	National	both		18-49		3138
Kazakhstan	1999	DHS	National	both		18-49		2005
Kazakhstan		Balakhmetova et l., Ter Arkh 63(1):17-20, 1991	Community	urban	20-54		2886	
Kenya	1985	INTERSALT	Community	rural	20-59	20-59	90	86
Kenya	1993	DHS	National	both		18-49		3308
Kenya	1998	DHS	National	both		18-49		3233
Kenya	2003	DHS	National	both		18-49		6227
Kenya	2008-2009	DHS	National	both		18-49		6796
Kiribati	1981	Epidemiological survey of Kiribati	Subnational	rural	20+	20+	473	531
Kiribati	1981	Epidemiological survey of Kiribati	Subnational	urban	20+	20+	939	906
Kiribati	2004	STEPS	National	both	18-64	18-64	729	895
Kuwait	1980-1981	al-Isa, Ann Nutr Metab 41(5):307-14, 1997	Community	both	18+		959	
Kuwait	1993-1994	al-Isa, Ann Nutr Metab 41(5):307-14, 1997	Community	both	18+		1730	
Kuwait	1995-1996	Abdella et al., Diabetes Res Clin Pract 42(3):187-96, 1998	Subnational	both	20-84	20-84	1099	1892
Kuwait	1995-1996	Abdella et al., Diabetes Res and Clin Pract 42(3):187–196, 1998	Subnational	both	20-84	20-84	1099	1892
Kuwait	1998	Abiaka et al., , Biol Trace Elem Res 91(1):33-43, 2003	National	both	18-80	18-80	178	233
Kuwait	2006	STEPS	National	both	20-65	20-65	918	1298
Kuwait	2008-2009	National Nutrition Program for the State of Kuwait	National	both	18+	18+	484	568
Kuwait	2008-2010	World Health Survey	National	both	18+	18+	1842	1619
Kyrgyzstan	1993	Kyrgyzstan Multipurpose Poverty Surveys	National	both	18-60	18-60	2457	2457
Kyrgyzstan	1997	DHS	National	both		18-49		3120
Kyrgyzstan	2012	DHS	National	both		18-49		6489
Kyrgyzstan	2013	STEPS	National	both	25-64	25-64	942	1600
Lao PDR	2006	Multiple Indicator Cluster Survey 3	National	both		18-49		698
Lao PDR	2008	STEPS	Community	urban	25-64	25-64	1568	2352
Lao PDR	2013	STEPS	National	both	18-64	18-64	984	1461
Latvia	1997	Pomerleau et al., Public Health Nutrition 3: 3-10, 2000	National	both	19-50	19-50	703	732
Latvia	2008-2009	Ērglis et al., Medicina (Kaunas) 48(6):310-16, 2012	National	both	25-74	25-74	1376	2431
Lebanon	1997	Population and Housing Survey	National	both	18+	18+	501	715
Lebanon	2008-2009	STEPS	National	both	18+	18+	1261	1365
Lesotho	1993	National survey on iodine, vitamin A and iron status of women and children in Lesotho	National	both		20-65		792
Lesotho	2004-2005	DHS	National	both		18-49		2703
Lesotho	2009-2010	DHS	National	both	18-59	18-49	2704	3200
Lesotho	2012	STEPS	National	both	25-64	25-64	726	1442
Liberia	2006-2007	DHS	National	both		18-49		5675
Liberia	2011	STEPS	National	both	25-64	25-64	998	1253
Liberia	2013	DHS	National	both	18-49	18-49	3675	4143
Libya	1998-1999	Kadiki et al., Diabetes Metab 27(6):647-54, 2001	Community	both	18+	18+	228	398
Libya	2009	STEPS	National	both	25-64	25-64	1678	1563
Lithuania	1977-1980	Multifactorial Prevention of Ishaemic Heart Disease, Kaunas	Community	urban	45-59		5691	
Lithuania	1983-1985	MONICA, Kaunas	Community	urban	35-64	35-64	728	735
Lithuania	1986-1987	MONICA, Kaunas	Community	urban	35-64	35-64	894	868
Lithuania	1992-1993	MONICA, Kaunas	Community	urban	35-64	35-64	610	621
Lithuania	1997	Pomerleau et al., Public Health Nutrition 3: 3-10, 2000	National	both	19-49	19-49	697	751
Lithuania	2001-2002	MONICA4	Community	urban	35-69	35-69	625	776
Lithuania	2002		National	both	24-70	24-70	977	927
Lithuania	2006-2008	Health, Alcohol and Psychosocial factors In Eastern Europe	Community	urban	45-75	45-75	3231	3874
Luxembourg	2007-2009	Observation of cardiovascular risk factors in Luxembourg (ORISCAV-LUX)	National	both	18-69	18-69	656	695
Macedonia (TFYR)	1999	Multiple Indicator Cluster Survey	National	both		18-45		1029
Madagascar	1997	Mauny et al., Ann Trop Med Parasitol 97(6):645-54, 2003	Community	both	18+	18+	248	283
Madagascar	1997	DHS	National	both		18-49		2506
Madagascar	2003-2004	DHS	National	both		18-49		6367

Country	Data years	Survey/study name	Level of representativness		Age range as used for global analysis		Sample size	
				Rural, urban, or both	Male	Female	Male	Female
Madagascar	2005	STEPS	Community	urban	25-64	25-64	2596	2490
Madagascar	2008-2009	DHS	National	both		18-49		6723
Malawi	1992	DHS	National	both		18-49		2267
Malawi	1996	Chilima et al., Eur J Clin Nutr 52(9):643-9	Community	rural	55-94	55-94	86	185
Malawi	2000	DHS	National	both		18-49		9973
Malawi	2004	DHS	National	both		18-49		8563
Malawi	2009	STEPS	National	both	25-64	25-64	1666	3185
Malawi	2010	DHS	National	both		18-49		6006
Malaysia	1996	National Health and Morbidity Survey	National	both	30+	30+	9925	10943
Malaysia	2002-2003	Malaysian Adult Nutrition Survey	National	both	18-59	18-59	3302	3395
Malaysia	2004	Variation in the prevalence, awareness, and control of diabetes in a multiethnic	National	both	18+	18+	5879	8056
Malaysia	2005	STEPS	National	both	25-64	25-64	1286	1286
Malaysia	2006	The third national health and morbidity survey (NHMS III)	National	both	18+	18+	15029	17828
Malaysia	2008	Metabolic Syndrome Study in Malaysia	National	both	18+	18+	1522	2814
Malaysia	2009-2012	Foo LH et al. APJCN	Community	urban	18-19	18-19	21	30
Malaysia	2011	The National Health and Morbidity Survey	National	both	18+	18+	8029	8782
Malaysia	2012	Global School-based Student Health Survey	National	both		18-18		84
Maldives	2001	Multiple Indicator Cluster Survey	National	both		18-50		1145
Maldives	2004	STEPS	Subnational	urban	25-64	25-64	933	1086
Maldives	2009	DHS	National	both		18-49		5213
Maldives	2011	STEPS	National	both	18-64	18-64	588	999
Maldives	2014	Global School-based Student Health Survey	National	both	18-19	18-19	122	161
Mali	1995-1996	DHS	National	both		18-49		4116
Mali	1997	Torheim et al., Eur J Clin Nutr 58(4):594-604, 2004	Subnational	rural	18-44	18-44	237	337
Mali	1999	Torheim et al., Public Health Nutr 8(4):387-94, 2005	Subnational	rural		18-44		191
Mali	2001	DHS	National	both		18-49		9281
Mali	2006	DHS	National	both		18-49		10726
Mali	2007	STEPS	Community	both	18-64	18-64	931	1313
Mali	2012-2013	DHS	National	both		18-49		4156
Malta	1984	MONICA, Malta	Community	urban	25-64	25-64	948	929
Malta	1986	INTERSALT	Community	rural	20-59	20-59	100	100
Marshall Islands	2002	STEPS	National	both	18-64	18-64	686	1057
Mauritania	2000-2001	DHS	National	both		18-49		2635
Mauritania	2006	STEPS	Community	urban	18-64	18-64	1065	1218
Mauritius	1987	Mauritius non communicable disease survey	National	both	25-74	25-74	2347	2653
Mauritius	1992	Mauritius non communicable disease survey	National	both	25-74	25-74	2985	3477
Mauritius	1998	Mauritius non communicable disease survey	National	both	25-74	25-74	2566	3248
Mauritius	2009	Mauritius non communicable diseases survey	National	both	19+	19+	2860	3389
Mexico	1988-1989	Encuesta Nacional de Nutricion	National	both		18-49		12043
Mexico	1992-1993	Encuesta Nacional de Enfermedades Cronicas	National	urban	20-69	20-69	6040	8287
Mexico	1996	Sanchez-Castillo et al., Eur J Clin Nutr 55(10):833-40, 2001	Community	rural	18+	18+	104	149
Mexico	1998-1999	Encuesta Nacional de Nutricion	National	both		18-49		13886
Mexico	1998-2004	Mexico City Prospective Study	Community	urban	35-84	35-84	51768	105313
Mexico	1999-2000	The Survey on Health, Well-Being, and Aging in Latin America and the Caribbean (SABE)	Community	urban	60+	60+	359	548
Mexico	2000	Encuesta Nacional de Salud	National	both	18+	18+	14702	30283
Mexico	2001	The Mexican Health and Aging Study	National	both	50+	50+	1031	1224
Mexico	2002	Encuesta Nacional Sobre Niveles de vida de los Hogares	National	both	18+	18+	7334	9089
Mexico	2003	The Mexican Health and Aging Study	National	both	50+	50+	893	1162
Mexico	2004-2005	CArdiovascular Risk factors Multiple Evaluation in Latin America	Community	urban	25-64	25-64	833	894
Mexico	2005	Encuesta Nacional Sobre Niveles de vida de los Hogares	National	both	18+	18+	7551	9084
Mexico	2006	Encuesta Nacional de Salud y Nutrición	National	both	18+	18+	14471	21429
Mexico	2006	PREVENIMSS National Coverage Surveys	Subnational	both	20+	20+	8715	11315
Mexico	2009-2010	SAGE	National	both	50+	50+	796	1236
Mexico	2011-2012	Encuesta Nacional de Salud Y Nutricion	National	both	18+	18+	17511	23418
Mexico	2012	The Mexican Health and Aging Study	National	both	50+	50+	786	1106
Micronesia (Federated States of)	2002	STEPS	Subnational	both	25-64	25-64	591	892
Micronesia (Federated States of)	2006	STEPS	Subnational	both	25-64	25-64	628	1160
Micronesia (Federated States of)	2008	STEPS	Subnational	both	25-64	25-64	876	1264
Micronesia (Federated States of)	2009	STEPS	Subnational	both	18-64	18-64	208	411
Micronesia (Federated States of)	2009	STEPS	Subnational	both	18-64	18-64	392	518
Moldova	2005	DHS	National	both		18-49		6268

Country	Data years	Survey/study name	Level of representativness		Age range as used for global analysis		Sample size	
				Rural, urban, or both	Male	Female	Male	Female
Moldova	2013	STEPS	National	both	18-69	18-69	1712	2776
Mongolia	1999	National Nutrition Survey	National	both	35-65	35-65	907	1317
Mongolia	2004	National Nutrition Survey	National	both	18-74	18-74	248	360
Mongolia	2005	STEPS	National	both	18-64	18-64	1513	1590
Mongolia	2009	STEPS	National	both	18-64	18-64	2079	2965
Mongolia	2013	Global School-based Student Health Survey	National	both	18-19	18-19	86	100
Mongolia	2013	STEPS	National	both	18-64	18-64	2317	2807
Morocco	1992	DHS	National	both		18-49		2873
Morocco	2000	National Survey 2000	National	both	20+	20+	755	1047
Morocco	2003-2004	DHS	National	both		18-49		13944
Mozambique	1997	DHS	National	both		18-49		3132
Mozambique	2003	DHS	National	both		18-49		9241
Mozambique	2005	STEPS	National	both	25-64	25-64	1276	1684
Mozambique	2011	DHS	National	both		18-49		10396
Myanmar	2003-2004	STEPS	Subnational	both	25-74	25-74	1992	2454
Myanmar	2009-2010	STEPS	National	both	18-64	18-64	2732	4276
Myanmar	2013-2014	STEPS	Subnational	both	25-74	25-74	745	741
Namibia	1992	DHS	National	both		18-49		2221
Namibia	2005	STEPS	National	both	24-64	24-64	1390	1776
Namibia	2006-2007	DHS	National	both		18-49		7700
Namibia	2006-2009	Okambilimbili Survey	Community	urban	18+	18+	752	949
Namibia	2013	DHS	National	both		18-64		4632
Nauru	1975-1976	Trends in the prevalence and incidence of non-insulin-dependent diabetes mellitus and impaired glucose tolerance	Subnational	both	18+	18+	233	240
Nauru	1982	Trends in the prevalence and incidence of non-insulin-dependent diabetes mellitus and impaired glucose tolerance	National	both	20+	20+	701	773
Nauru	1987	Trends in the prevalence and incidence of non-insulin-dependent diabetes mellitus and impaired glucose tolerance	National	both	20+	20+	555	667
Nauru	1994	Trends in the prevalence and incidence of non-insulin-dependent diabetes mellitus and impaired glucose tolerance	National	both	25+	25+	647	731
Nauru	2004	STEPS	National	both	18-64	18-64	1011	1078
Nauru	2006	STEPS	National	both	18-65	18-65	244	230
Nepal	1996	DHS	National	both		18-49		3333
Nepal	1997	Ohno et al., Asia Pac J Public Health 18(3):20-9, 2006	Community	rural	18-75	18-75	36	41
Nepal	2001	DHS	National	both		18-49		7646
Nepal	2003	STEPS	Subnational	both	25-64	25-64	1010	996
Nepal	2005	STEPS	Subnational	both	18-64	18-64	3650	4084
Nepal	2006	DHS	National	both		18-49		8677
Nepal	2011	DHS	National	both		18-49		5076
Nepal	2013	STEPS	National	both	18-69	18-69	1276	2703
Netherlands	1985	INTERSALT	Community	urban	20-59	20-59	100	99
Netherlands	1985	Zutphen Elderly Study	Community	both	65+		886	
Netherlands	1989-1993	The Rotterdam Study, first subcohort	Community	urban	55+	55+	2807	4103
Netherlands	1992-1993	LASA	Subnational	both	55+	55+	1197	1206
Netherlands	1993-1995	The Rotterdam Study, first subcohort	Community	urban	56+	56+	2214	3105
Netherlands	1993-1997	EPIC Bilthoven	Community	urban	20-59	20-59	9941	12021
Netherlands	1993-1997	EPIC Utrecht	Community	both		49-70		17335
Netherlands	1997-1999	The Rotterdam Study, first subcohort	Community	urban	61+	61+	1718	2361
Netherlands	1998-2001	Regenboog Project	National	both	18-89	18-89	2582	2490
Netherlands	2000-2001	The Rotterdam Study, second subcohort	Community	urban	55+	55+	1210	1468
Netherlands	2001-2003	Surinamese in the Netherlands: Study on Ethnicity and Health	Community	urban	35-60	35-60	251	257
Netherlands	2002-2004	The Rotterdam Study, first subcohort	Community	urban	64+	64+	1206	1708
Netherlands	2003-2007	Doetinchem Cohort Study (4th measurement)	Subnational	urban	36-74	36-74	2125	2352
Netherlands	2004-2005	The Rotterdam Study, second subcohort	Community	urban	58+	58+	964	1244
Netherlands	2005-2006	LASA (Wave F)	National	both	55+	55+	789	958
Netherlands	2006-2008	The Rotterdam Study, third subcohort	Community	urban	45+	45+	1547	2029
Netherlands	2009-2010	Measuring the Netherlands (NL de Maat)	Subnational	both	30-70	30-70	1781	2014
Netherlands	2009-2011	The Rotterdam Study, first subcohort	Community	urban	72+	72+	690	1006
Netherlands	2011-2012	The Rotterdam Study, second subcohort	Community	urban	65+	65+	735	934
Netherlands	2011-2013	Healthy Life in an Urban Setting	Community	urban	18-71	18-71	858	1011
Netherlands	2012-2014	The Rotterdam Study, third subcohort	Community	urban	52+	52+	1256	1639
New Zealand	1982	MONICA, Auckland	Community	urban	35-64	35-64	1019	568
New Zealand	1989	The Life in New Zealand Survey	National	both	18+	18+	1345	1485
New Zealand	1990-1993	Williams, N Z Med J 113(1114):308-11, 2000	Community	both	18-21	18-21	932	859
New Zealand	1993-1994	MONICA, Auckland	Community	urban	35-64	35-64	723	674

Country	Data years	Survey/study name	Level of representativness		Age range as used for global analysis		Sample size	
				Rural, urban, or both	Male	Female	Male	Female
New Zealand	1996-1997	National Nutrition Survey	National	both	18+	18+	1776	2423
New Zealand	2002-2003	New Zealand Health Survey	National	both	18+	18+	4438	6550
New Zealand	2006-2007	New Zealand Health Survey	National	both	18+	18+	4864	6271
New Zealand	2008-2009	The Adult Nutrition Survey	National	both	18+	18+	1754	2215
New Zealand	2011-2012	New Zealand Health Survey	National	both	18+	18+	4396	5948
New Zealand	2012-2013	New Zealand Health Survey	National	both	18+	18+	4967	6602
New Zealand	2013-2014	New Zealand Health Survey	National	both	18+	18+	5396	6807
Nicaragua	1997-1998	DHS	National	both		18-49		10322
Nicaragua	2001	DHS	National	both		18-49		10195
Nicaragua	2003-2004	CAMDI	Community	urban	20+	20+	773	916
Nicaragua	2003-2005	Sistema Integrado de Vigilancia de Intervenciones Nutricionales (SIVIN)	National	both		18-50		1115
Nicaragua	2006-2007	Encuesta Nicaraguense de Demografia y Salud	National	both		18-49		11765
Niger	1992	DHS	National	both		18-49		3207
Niger	1998	DHS	National	both		18-49		3257
Niger	2006	DHS	National	both		18-49		3590
Niger	2007	STEPS	National	both	18-64	18-64	1356	1131
Niger	2012	DHS	National	both		18-49		3936
Nigeria	1990	Non-communicable diseases National Survey	National	rural	18+	18+	3618.915	3681.719
Nigeria	1990	Non-communicable diseases National Survey	National	urban	18+	18+	1616.934	1644.995
Nigeria	1991-1994	Cooper et al., Am J Public Health 87(2):160-68, 1997	Community	both	20-100	20-100	910	1080
Nigeria	1999	DHS	National	both		18-49		2134
Nigeria	1999-2009	Prostate cancer dietary risk factors study	Subnational	both	35+		627	
Nigeria	2003	DHS	National	both		18-49		5664
Nigeria	2006	Senbanjo et al., West Afr J Med 30(6):425-31, 2011	Community	urban	18-19	18-19	10	12
Nigeria	2007	Ibadan Study of Ageing	Subnational	both	60+	60+	642	914
Nigeria	2008	DHS	National	both		18-49		25420
Nigeria	2008	Ibadan Study of Ageing	Subnational	both	61+	61+	453	656
Nigeria	2009	Ibadan Study of Ageing	Subnational	both	62+	62+	420	619
Nigeria	2009	Community Health Plan - Kwara Central Survey	Community	rural	18+	18+	1247	1418
Nigeria	2011	Community Health Plan - Kwara Central Survey	Community	rural	18+	18+	396	494
Nigeria	2013	DHS	National	both		18-49		29350
Nigeria	2013	Community Health Plan - Kwara Central Survey	Community	rural	18+	18+	358	422
Niue	2011	STEPS	National	both	18-100	18-100	376	454
Norway	1979-1980	Jacobsen et al., Scand J Soc Med 16(2):101-4, 1988	Community	urban	20-54	20-49	8309	7820
Norway	1984-1986	HUNT1 Study	Subnational	rural	20+	20+	36517	37811
Norway	1986-1987	Tromsø Study 3	Community	urban	20-61	20-56	10367	9798
Norway	1994-1995	Tromsø Study 4	Community	urban	25+	25+	12774	13813
Norway	1995-1997	HUNT2 study	Subnational	rural	20+	20+	30285	33599
Norway	1995-1997	Young-HUNT1 Study	Subnational	rural	18-21	18-21	634	686
Norway	1997-1999	HUSK	Community	urban	47-73	47-73	1635	1670
Norway	2000-2001	Young-HUNT2 Study	Subnational	rural	18-21	18-21	379	498
Norway	2000-2003	the Oslo cohort (HUBBRO), the Oppland and Hedmark cohort (OPPHED), and the Troms and Finnmark cohort (TROFINN) of COHORT NORWAY	Subnational	both	30-76	30-76	16825	20592
Norway	2001-2002	Tromsø Study 5	Community	urban	30-84	30-84	2974	4152
Norway	2006-2008	HUNT3 Study	Subnational	rural	20+	20+	22860	27553
Norway	2006-2008	Young-HUNT3 Study	Subnational	rural	18-21	18-21	442	519
Norway	2007-2008	Tromsø Study 6	Community	urban	30-87	30-87	6044	6880
Occupied Palestinian Territory	1996	Stene et al., Eur J Clin Nutr 55(9):805-11, 2001	Community	rural	30-65	30-65	208	269
Occupied Palestinian Territory	1996-1998	Ramallah study	Community	rural	18-64	18-64	206	442
Occupied Palestinian Territory	1996-1998	Ramallah study	Community	urban	18-64	18-64	182	458
Occupied Palestinian Territory	1999-2000	The First National Health and Nutrition Survey	National	both	18-64	18-64	1736	1869
Occupied Palestinian Territory	2010	STEPS	National	both	18-64	18-64	1851	3364
Oman	1991	Oman National Health survey	National	both	20+	20+	2128	2958
Oman	2000	Oman National Health survey	National	both	20+	20+	3069	3331
Oman	2001	Al-Lawati et al., Diabetes Care 26(6):1781-85, 2003	Community	urban	20+	20+	755	756
Oman	2006	STEPS	Community	urban	20-59	20-59	540	732
Oman	2008	The Oman World Health Survey	National	both	18+	18+	2258.548	2227.527
Pakistan	1990-1994	MHS	Community	urban	18+	18+	432	478
Pakistan	1990-1994	National Health Survey Of Pakistan 1990-1994	National	both	18+	18+	3789	4346
Pakistan	1999	Shah et al., Trop Med Int Health 9(4):526-32, 2004	Community	both	18+	18+	1391	2754
Pakistan	2004-2005	COBRA-1	Community	urban	40+	40+	1500	1635
Pakistan	2005	STEPS	National	both	25-65	25-65	787	1071

Country	Data years	Survey/study name	Level of representativness		Age range as used for global analysis		Sample size	
				Rural, urban, or both	Male	Female	Male	Female
Pakistan	2011	National and Nutritional Survey	National	both		18-49		21848
Pakistan	2012-2013	DHS	National	both		18-49		4105
Palau	2013	STEPS	National	both	25-64	25-64	1029	1124
Panama	2003	Second Living Standards Survey	National	both	18-75	18-75	6844	7100
Panama	2010-2011	Prevalencia de factores de riesgo asociados a enfermedad cardiovascular 2010-2011	Subnational	both	18+	18+	1067	2469
Papua New Guinea	1985-1986	INTERSALT	Community	rural	20-59	20-59	88	74
Papua New Guinea	2007	STEPS	National	both	18-64	18-64	1281	1317
Peru	1991-1992	DHS	National	both		18-49		5120
Peru	1996	DHS	National	both		18-49		10616
Peru	2000	DHS	National	both		18-49		22078
Peru	2002-2003	Mohanna et al., High Alt Med Biol 7(3):245-55, 2006	Community	rural	25-74	25-74	38	64
Peru	2003-2007	FRENT	Subnational	urban	18+	18+	1052	2147
Peru	2004-2005	CArdiovascular Risk factors Multiple Evaluation in Latin America	Community	urban	25-64	25-64	769	876
Peru	2004-2005	Encuesta Nacional de Indicadores Nutricionales, Bioquímicos, Socioeconómicos y Culturales Relacionados con las Enfermedades Crónicas Degenerativas	National	both	20-95	20-95	2087	2094
Peru	2004-2006	DHS	National	both		18-49		5030
Peru	2007-2008	DHS	National	both		18-49		18357
Peru	2007-2008	PERU MIGRANT Study	Community	both	35+	35+	405	442
Peru	2009	DHS	National	both		18-49		20119
Peru	2009-2010	Componente Nutricional Encuesta Nacional de Hogares	National	both	18+	18+	17730	21934
Peru	2009-2012	CRONICAS Cohort Study	Subnational	both	35+	35+	1557	1660
Peru	2010	DHS	National	both		18-49		19529
Peru	2011	DHS	National	both		18-49		19429
Peru	2012	DHS	National	both		18-49		20859
Peru	2013	DHS	National	both		18-49		20063
Peru	2014	DHS	National	both		18-49		21991
Philippines	1983-1984	Cebu Longitudinal Health and Nutrition Survey Baseline 2-Month Followup	Community	both		18-50		2783
Philippines	1983-1984	Cebu Longitudinal Health and Nutrition Survey Baseline 4-Month Followup	Community	both		18-50		2659
Philippines	1983-1984	Cebu Longitudinal Health and Nutrition Survey Baseline 6-Month Followup	Community	both		18-50		2545
Philippines	1984-1985	Cebu Longitudinal Health and Nutrition Survey Baseline 8-Month Followup	Community	both		18-50		2435
Philippines	1984-1985	Cebu Longitudinal Health and Nutrition Survey Baseline 10-Month Followup	Community	both		18-50		2317
Philippines	1984-1985	Cebu Longitudinal Health and Nutrition Survey Baseline 12-Month Followup	Community	both		18-50		2235
Philippines	1984-1985	Cebu Longitudinal Health and Nutrition Survey Baseline 14-Month Followup	Community	both		18-50		2169
Philippines	1984-1985	Cebu Longitudinal Health and Nutrition Survey Baseline 16-Month Followup	Community	both		18-50		2113
Philippines	1984-1985	Cebu Longitudinal Health and Nutrition Survey Baseline 18-Month Followup	Community	both		18-50		2066
Philippines	1985-1986	Cebu Longitudinal Health and Nutrition Survey Baseline 20-Month Followup	Community	both		18-50		2038
Philippines	1985-1986	Cebu Longitudinal Health and Nutrition Survey Baseline 22-Month Followup	Community	both		18-50		2006
Philippines	1985-1986	Cebu Longitudinal Health and Nutrition Survey Baseline 24-Month Followup	Community	both		18-50		2015
Philippines	1988	INCLEN	Community	rural	35-65		274	
Philippines	1991-1992	Cebu Longitudinal Health and Nutrition Survey 1991 Mother Followup	Community	both		22-55		2195
Philippines	1993	4th National Nutrition Survey Philippine	National	both	20-70	20-70	4383	4754
Philippines	1993	National Safe Motherhood Survey	National	both		18-49		7181
Philippines	1994-1995	Cebu Longitudinal Health and Nutrition Survey 1994-1995 Mother Followup	Community	both		18-59		2552
Philippines	1998	5th National Nutrition Survey Philippine	National	both	20-60	20-60	1323	1340
Philippines	1998-1999	Cebu Longitudinal Health and Nutrition Survey 1998-1999 Mother Followup	Community	both		18-59		1911
Philippines	2002	Cebu Longitudinal Health and Nutrition Survey 2002 Child Followup	Community	both	18-19	18-19	1084	897
Philippines	2002	Cebu Longitudinal Health and Nutrition Survey 2002 Mother Followup	Community	both		32-66		2080
Philippines	2003-2004	National Nutrition and Health Survey	National	both	18+	18+	30231	33295
Philippines	2005	Cebu Longitudinal Health and Nutrition Survey 2005 Child Followup	Community	both	20-22	20-22	1006	831
Philippines	2005	Cebu Longitudinal Health and Nutrition Survey 2005 Mother Followup	Community	both		35-69		2001
Philippines	2007	Cebu Longitudinal Health and Nutrition Survey 2007 Child Followup	Community	both	23-24	23-24	937	751
Philippines	2007	Cebu Longitudinal Health and Nutrition Survey 2007 Mother Followup	Community	both		38-71		1925
Philippines	2009	Cebu Longitudinal Health and Nutrition Survey 2009 Child Followup	Community	both	24-26	24-26	864	718
Philippines	2009	Life Course Study in Cardiovascular Disease Epidemiology	Subnational	both	20-50	20-50	1329	1743
Poland	1983-1984	MONICA, Tarnobrzeg Voivodship	Community	rural	35-64	35-64	1236	1441
Poland	1983-1985	MONICA, Warsaw	Community	urban	35-64	35-64	1297	1327
Poland	1986	Poland Conscripts 10% Sample Cohort	National	both	18-19		29421	
Poland	1986	INTERSALT, Krakow	Community	urban	20-59	20-59	100	100
Poland	1986	INTERSALT, Warsaw	Community	urban	20-59	20-59	100	100
Poland	1987-1988	MONICA, Tarnobrzeg Voivodship	Community	rural	35-64	35-64	616	672
Poland	1988-1989	MONICA, Warsaw	Community	urban	35-64	35-64	705	713
Poland	1992-1993	MONICA, Tarnobrzeg Voivodship	Community	rural	35-64	35-64	618	692

Country	Data years	Survey/study name	Level of representativness		Age range as used for global analysis		Sample size	
				Rural, urban, or both	Male	Female	Male	Female
Poland	1993	MONICA, Warsaw	Community	urban	35-64	35-64	751	763
Poland	1995	Poland Conscripts 10% Sample Cohort	National	both	18-19		31043	
Poland	2000	Binkowska-Bury et al., Neuro Endocrinol Lett 34(8):814-20, 2013	Subnational	both	19-20		3003	
Poland	2000-2001	Household Food Consumption and Anthropometric Survey	National	both	18+	18+	1287	1613
Poland	2001	Binkowska-Bury et al., Neuro Endocrinol Lett 34(8):814-20, 2013	Subnational	both	19-20		3420	
Poland	2001	Poland Conscripts 10% Sample Cohort	National	both	18-19		31213	
Poland	2002	Binkowska-Bury et al., Neuro Endocrinol Lett 34(8):814-20, 2013	Subnational	both	19-20		3544	
Poland	2002-2005	Health, Alcohol and Psychosocial factors In Eastern Europe	Community	urban	45-69	45-69	4454	4720
Poland	2003	Binkowska-Bury et al., Neuro Endocrinol Lett 34(8):814-20, 2013	Subnational	both	19-20		3633	
Poland	2003	The European Male Ageing Study	Community	both	40+		406	
Poland	2003-2005	National Multicenter Health Survey in Poland. Project WOBASZ	National	both	20-74	20-74	6245	6907
Poland	2003-2013	Mogielica Human Ecology Study	Community	rural	21+	21+	353	896
Poland	2004	Binkowska-Bury et al., Neuro Endocrinol Lett 34(8):814-20, 2013	Subnational	both	19-20		3538	
Poland	2005	Binkowska-Bury et al., Neuro Endocrinol Lett 34(8):814-20, 2013	Subnational	both	19-20		3308	
Poland	2006	Binkowska-Bury et al., Neuro Endocrinol Lett 34(8):814-20, 2013	Subnational	both	19-20		3701	
Poland	2006-2007	National Multicenter Health Survey in Poland. Project WOBASZ	National	both	75+	75+	541	533
Poland	2007	Binkowska-Bury et al., Neuro Endocrinol Lett 34(8):814-20, 2013	Subnational	both	19-20		3612	
Poland	2007-2009	Elaboration of the reference range of arterial blood pressure for the population of children and adolescents in Poland	National	both	18-19	18-19	435	591
Poland	2007-2011	Medical, psychological and socioeconomic aspects of aging in Poland	National	both	55+	55+	2750	2582
Poland	2008	Binkowska-Bury et al., Neuro Endocrinol Lett 34(8):814-20, 2013	Subnational	both	19-20		3435	
Poland	2008	The European Male Ageing Study	Community	both	40+		310	
Poland	2009	Binkowska-Bury et al., Neuro Endocrinol Lett 34(8):814-20, 2013	Subnational	both	19-20		3405	
Poland	2010	Binkowska-Bury et al., Neuro Endocrinol Lett 34(8):814-20, 2013	Subnational	both	19-20		3317	
Poland	2011	NATPOL	National	both	18-79	18-79	1158	1235
Portugal	1985	Body Mass Index of Portuguese Conscript	National	both	18-20		29420	
Portugal	1986	Body Mass Index of Portuguese Conscript	National	both	18-20		70504	
Portugal	1986	INTERSALT	Community	both	20-59	20-59	99	99
Portugal	1987	Body Mass Index of Portuguese Conscript	National	both	18-20		68079	
Portugal	1988	Body Mass Index of Portuguese Conscript	National	both	18-20		67573	
Portugal	1989	Body Mass Index of Portuguese Conscript	National	both	18-20		68827	
Portugal	1990	Body Mass Index of Portuguese Conscript	National	both	18-20		44359	
Portugal	1991	Body Mass Index of Portuguese Conscript	National	both	18-20		19552	
Portugal	1992	Body Mass Index of Portuguese Conscript	National	both	18-20		52393	
Portugal	1993	Body Mass Index of Portuguese Conscript	National	both	18-20		59780	
Portugal	1994	Body Mass Index of Portuguese Conscript	National	both	18-20		55511	
Portugal	1995	Body Mass Index of Portuguese Conscript	National	both	18-20		68221	
Portugal	1996	Body Mass Index of Portuguese Conscript	National	both	18-21		106097	
Portugal	1997	Body Mass Index of Portuguese Conscript	National	both	18-21		61215	
Portugal	1998	Body Mass Index of Portuguese Conscript	National	both	18-21		41027	
Portugal	1999	Body Mass Index of Portuguese Conscript	National	both	18-21		54187	
Portugal	1999-2003	EPIPorto	Community	urban	18+	18+	932	1507
Portugal	2000	Body Mass Index of Portuguese Conscript	National	both	18-21		53326	
Portugal	2003-2005	Estudo de Prevalencia da Obesidade e Consumos Alimentares em Portugal	National	both	18-64	18-64	3796	4320
Portugal	2007-2009	Portuguese National Survey of Physical Activity and Physical Fitness	National	both	18+	18+	4693	7178
Portugal	2010-2012	Exercise for Elderly	Community	urban	60-84	60-84	48	104
Puerto Rico	2002-2003	Puerto Rican Elderly: Health Conditions	National	both	60+	60+	1914	2850
Puerto Rico	2005-2007	Perez et al., Ethn Dis 18(4):434-41, 2008	Community	urban	18-84	18-84	275	529
Puerto Rico	2006-2007	Puerto Rican Elderly: Health Conditions	National	both	60+	60+	1056	1669
Puerto Rico	2010-2013	HPV Infection in a Population-Based Sample of Puerto Rican Women	Subnational	both		18-64		557
Qatar	2006	GCC	National	both	18+	18+	1859	2018
Qatar	2012	STEPS	National	both	18-64	18-64	1034	1352
Romania	1986-1987	MONICA, Bucharest	Community	urban	35-64	35-64	702	873
Romania	1997	Somatometria	National	both	18-75	18-75	3142	4063
Romania	2009-2011	Study on children in Dolj County, South Romania	Subnational	both	18-21	18-21	87	38
Romania	2014	Timis County Study	Community	urban	18-19	18-19	8	5
Russian Federation	1984-1986	MONICA, Moscow (control)	Community	urban	35-64	35-64	774	642
Russian Federation	1984-1986	MONICA, Moscow, Leninsky district	Community	urban	35-64	35-64	553	622
Russian Federation	1984-1986	MONICA, Moscow, Cheremushkinsky district	Community	urban	35-64	35-64	580	579
Russian Federation	1985	MONICA, Novosibirsk (intervention)	Community	urban	25-64	25-64	797	818
Russian Federation	1985-1986	MONICA, Novosibirsk, Kirowsky district	Community	urban	25-64	25-64	758	774
Russian Federation	1985-1986	MONICA, Novosibirsk, Leninsky district	Community	urban	25-64	25-64	624	624

Country	Data years	Survey/study name	Level of representativness		Age range as used for global analysis		Sample size	
				Rural, urban, or both	Male	Female	Male	Female
Russian Federation	1986	INTERSALT	Community	urban	20-59	20-59	97	97
Russian Federation	1988	MONICA, Novosibirsk (intervention)	Community	urban	25-64	25-64	837	852
Russian Federation	1988-1989	MONICA, Moscow (control)	Community	urban	35-64	35-64	620	581
Russian Federation	1988-1989	MONICA, Moscow, Leninsky district	Community	urban	35-64	35-64	597	612
Russian Federation	1988-1989	MONICA, Novosibirsk, Kirowsky district	Community	urban	25-64	25-64	871	705
Russian Federation	1992	CINDI	Community	rural	25-64	25-64	377	453
Russian Federation	1992	Puska et al., Int J Epidemiol 22(6):1048-55, 1993	Community	both	25-64	25-64	379	458
Russian Federation	1992-1993	Russia Longitudinal Monitoring Survey-Higher School of Economics Round II	National	both	18+	18+	4362	5940
Russian Federation	1992-1995	MONICA, Moscow (control)	Community	urban	35-64	35-64	556	527
Russian Federation	1992-1995	MONICA, Moscow, Leninsky district	Community	urban	35-64	35-64	538	858
Russian Federation	1993	Russia Longitudinal Monitoring Survey-Higher School of Economics Round III	National	both	18+	18+	4522	6327
Russian Federation	1993-1994	Russia Longitudinal Monitoring Survey-Higher School of Economics Round IV	National	both	18+	18+	4037	5669
Russian Federation	1994	Russia Longitudinal Monitoring Survey-Higher School of Economics Round V	National	both	18+	18+	3575	4704
Russian Federation	1994-1995	MONICA, Novosibirsk (intervention)	Community	urban	25-64	25-64	820	860
Russian Federation	1995	MONICA, Novosibirsk, Kirowsky district	Community	urban	25-64	25-64	771	787
Russian Federation	1995	Russia Longitudinal Monitoring Survey-Higher School of Economics Round VI	National	both	18+	18+	3365	4459
Russian Federation	1996	Russia Longitudinal Monitoring Survey-Higher School of Economics Round VII	National	both	18+	18+	3318	4428
Russian Federation	1998-1999	Russia Longitudinal Monitoring Survey-Higher School of Economics Round VIII	National	both	18+	18+	3433	4553
Russian Federation	2000	Russia Longitudinal Monitoring Survey-Higher School of Economics Round IX	National	both	18+	18+	3521	4771
Russian Federation	2001	Russia Longitudinal Monitoring Survey-Higher School of Economics Round X	National	both	18+	18+	3881	5387
Russian Federation	2002	Russia Longitudinal Monitoring Survey-Higher School of Economics Round XI	National	both	18+	18+	4070	5542
Russian Federation	2002-2005	Health, Alcohol and Psychosocial factors In Eastern Europe	Community	urban	45-69	45-69	4208	5040
Russian Federation	2003	Russia Longitudinal Monitoring Survey-Higher School of Economics Round XII	National	both	18+	18+	4132	5631
Russian Federation	2004	Russia Longitudinal Monitoring Survey-Higher School of Economics Round XIII	National	both	18+	18+	4149	5645
Russian Federation	2005	Russia Longitudinal Monitoring Survey-Higher School of Economics Round XIV	National	both	18+	18+	4025	5498
Russian Federation	2007-2010	SAGE	National	both	50+	50+	1254	2251
Rwanda	2000	DHS	National	both		18-49		7591
Rwanda	2005	DHS	National	both		18-49		4381
Rwanda	2010	DHS	National	both	18-59	18-49	5536	5559
Rwanda	2012	STEPS	National	both	18-64	18-64	2486	3995
Saint Kitts and Nevis	2007	STEPS	Subnational	both	25-64	25-64	511	849
Saint Lucia	1981	Population Study of Blood Pressure and Associated Factors in St Lucia, West Indies	National	both	18+	18+	168	191
Saint Lucia	1991-1994	Cooper et al., Am J Public Health 87(2):160-68, 1997	Community	urban	25-100	25-100	491	593
Saint Lucia	2012	STEPS	National	both	25-64	25-64	587	939
Samoa	1979-1982	McGarvey, Am J Clin Nutr 53(6 Suppl):1586S-1594S, 1991	National	both	18+	18+	247	297
Samoa	1991	McGarvey, Pac Health Dialog 8(1):157-62, 2001	National	both	25+	25+	347	381
Samoa	1995	McGarvey, Pac Health Dialog 8(1):157-62, 2001	National	both	25+	25+	156	153
Samoa	2002	STEPS	National	both	25-64	25-64	1181	1334
Samoa	2010	Samoan Genome-Wide Association Study	National	both	24-65	24-65	1402	2061
Samoa	2013	STEPS	National	both	18-64	18-64	605	918
Sao Tome and Principe	2008-2009	DHS	National	both	18-59	18-49	1896	1961
Sao Tome and Principe	2009	STEPS	National	both	25-64	25-64	998	1287
Saudi Arabia	1985-1988	National Nutrition Survey	National	both	18-75	18-75	4356	5944
Saudi Arabia	1987-1992	Evaluation of the nutritional status of the people of Saudi Arabia	National	both	18-60	18-60	2318	3429
Saudi Arabia	1989-1994	National Nutrition Survey	National	both	18-40	18-40	2481	3294
Saudi Arabia	1990-1993	National Epidemiological Household Survey	National	both	18-60	18-60	4882	4509
Saudi Arabia	1990-1993	Saudi National Survey	National	both	30-70	30-70	1612	1648
Saudi Arabia	1992-1995	Saudi Health Information Survey	National	both	18-50	18-50	2812	5223
Saudi Arabia	1995	National Household Survey	National	both	20-70	20-70	7121.212	7073.522
Saudi Arabia	1995-2000	National Epidemiological Health Survey	National	both	30-70	30-70	8215	9008
Saudi Arabia	2004-2005	Al-Baghli et al., Saudi Med J 29(9):1319-25, 2008	Subnational	both	30+	30+	97254	97254
Saudi Arabia	2005	STEPS	National	both	18-64	18-64	2245	2345
Saudi Arabia	2007	World Health Survey	National	both	18+	18+	93	77
Saudi Arabia	2013	Saudi Health Interview Survey	National	both	18+	18+	4694	4948
Senegal	1984	Maire et al., Rev Epidemiol Sante Publique 40:252-58, 1992	National	rural		18-45		1628
Senegal	1986	Maire et al., Rev Epidemiol Sante Publique 40:252-58, 1992	Community	urban		18-45		616
Senegal	1992-1993	DHS	National	both		18-49		2867
Senegal	2003	Holdsworth et al., Int J Obes Relat Metab Disord 28(12):1561-68, 2004	Community	urban		20-49		301
Senegal	2005	DHS	National	both		18-49		3451
Senegal	2010-2011	DHS	National	both	18-59	18-49	3944	4705
Senegal		Astagneau et al., J Hypertens 10(9):1095-101, 1992	Community	urban	18+	18+	651	707

Country	Data years	Survey/study name	Level of representativness		Age range as used for global analysis		Sample size	
				Rural, urban, or both	Male	Female	Male	Female
Serbia	1984	MONICA, Novi Sad	Community	urban	25-64	25-64	798	777
Serbia	1988-1989	MONICA, Novi Sad	Community	urban	25-64	25-64	778	791
Serbia	1994-1995	MONICA, Novi Sad	Community	urban	25-64	25-64	600	670
Serbia	2000	Health Status, Health Needs and Utilization of Health Care of the Population of Serbia	National	both	18+	18+	4279	5375
Serbia	2006	National Health Survey	National	both	18+	18+	6766	7445
Serbia	2013	National Health Survey	National	both	18+	18+	6262	7202
Serbia	2013-2014	Stay Fit for Lifelong Health; The Prevalence of Lifestyle Health Conditions in Serbian Population	National	urban	18-65		1366	
Seychelles	1989	Seychelles Heart Survey I	National	both	25-64	25-64	513	568
Seychelles	1994	Seychelles Heart Survey II	National	both	25-64	25-64	499	563
Seychelles	2003	School Screening Program	National	both	18-19	18-19	177	229
Seychelles	2004	Seychelles Heart Survey III	National	both	25-64	25-64	568	687
Seychelles	2013-2014	Seychelles Heart Survey IV	National	both	25-64	25-64	531	698
Sierra Leone	2008	DHS	National	both		18-49		2964
Sierra Leone	2009	STEPS	National	both	25-64	25-64	2200	2318
Sierra Leone	2013	DHS	National	both	18-59	18-49	6127	6408
Singapore	1982-1985	Thyroid Heart Study	National	both	18-91	18-91	1030	990
Singapore	1992	National Health Survey 1992	National	both	18-64	18-64	1743.208	1704.147
Singapore	1993-1995	NUH Heart Study	National	both	26-89	26-89	498	484
Singapore	1998	National Health Survey 1998	National	both	18-69	18-69	2283.741	2264.736
Singapore	2004	National Health Survey 2004	National	both	18-74	18-74	2059.15	2094.5
Singapore	2004-2007	Combined follow up of Singapore Cardiovascular Cohort study and Singapore Prospective study	National	both	24-95	24-95	2471	2686
Singapore	2009	Social Isolation, Health and Lifestyles Survey (SIHLS) 2009	National	both	60+	60+	2038	2382
Singapore	2009-2011	The Singapore Chinese Eye Study	Community	both	40+	40+	1652	1679
Singapore	2012-2013	Singapore Health Study 2012	National	both	18-79	18-79	956	1026
Slovakia	1993	Countrywide Integrated Noncommunicable Diseases Intervention Programme	National	both	18-64	18-64	762	1217
Slovakia	1998	Countrywide Integrated Noncommunicable Diseases Intervention Programme	National	both	18-64	18-64	857	1046
Slovakia	2003	Countrywide Integrated Noncommunicable Diseases Intervention Programme	National	both	18-64	18-64	622	867
Slovakia	2008	Countrywide Integrated Noncommunicable Diseases Intervention Programme	National	both	18-64	18-64	391	561
Slovakia	2011-2012	Europan Health Examination Survey	National	both	18-64	18-64	884	1080
Slovenia	2014	Analysis of Children's Development in Slovenia (ACDSi), 15-19 year olds	National	urban	18-19	18-19	144	126
Slovenia	2014	the SLOFIT monitoring system	National	both	18-21	18-21	6460	6410
Solomon Islands	2006	STEPS	Subnational	both	18-64	18-64	990	1305
Solomon Islands	2009-2010	Furusawa et al., N Z Med J 124(1333):17-28, 2011	Subnational	rural	18-87	18-87	153	215
Solomon Islands	2009-2010	Furusawa et al., N Z Med J 124(1333):17-28, 2011	Community	urban	18-70	18-70	22	57
South Africa	1989	Temple et al., Ethn Dis 11(3):431-7, 2001	Community	both	18+	18+	457	614
South Africa	1990	Steyn et al., East Afr Med J 75(1):35-40, 1998	Community	urban	18-64	18-64	292	373
South Africa	1996	Temple et al., Ethn Dis 11(3):431-7, 2001	Community	both	18+	18+	302	406
South Africa	1998	DHS	National	both	18+	18+	4987	7107
South Africa	2002	The 1st South African National Youth Risk Behaviour Survey	National	both	18-18	18-18	475	420
South Africa	2002-2003	SASPI	Community	rural	35+	35+	80	275
South Africa	2003	DHS	National	both	18+	18+	2825	4121
South Africa	2003-2004	Africa Centre Biomeasure Survey	Community	rural	25-49	25-49	778	1693
South Africa	2004-2006	Li et al., Curationis 30(4):79-87, 2007	Community	both	18-40	18-40	334	270
South Africa	2007-2008	SAGE	National	both	50+	50+	1543	2061
South Africa	2008	National Income Dynamics Study Wave I	National	both	18+	18+	4813	7327
South Africa	2008	The 2nd South African National Youth Risk Behaviour Survey	National	both	18-18	18-18	526	431
South Africa	2008-2009	Cape Town Bellville South Cohort Study - Baseline evaluation I	Community	urban	18+	18+	220	715
South Africa	2010	Africa Centre Biomeasure Survey	Community	rural	18+	18+	2271	5718
South Africa	2010-2011	National Income Dynamics Study Wave II	National	both	18+	18+	30799	49072
South Africa	2012	National Income Dynamics Study Wave III	National	both	18+	18+	33193	54314
South Africa	2012	South African National Health and Nutrition Examination Survey	National	both	18+	18+	2274	4320
South Korea	1986	INTERSALT	Community	urban	20-59	20-59	100	98
South Korea	1990	Korean National Blood Pressure Survey	National	both	30+	30+	9734	12620
South Korea	1992-1993	Park et al., Diabetes Res Clin Pract 34 Suppl:S65-72, 1996	Subnational	both	30-89	30+	1077	1392
South Korea	1998	Korea National Health and Nutrition Examination Survey	National	both	18+	18+	3741	4482
South Korea	2001	Kim et al., Br J Psychiatry 185:102-7, 2004	Community	both	65+	65+	300	432
South Korea	2001	Korea National Health and Nutrition Examination Survey	National	both	18+	18+	2974	3762
South Korea	2005	Korea National Health and Nutrition Examination Survey	National	both	18+	18+	2392	3157
South Korea	2007	Korea National Health and Nutrition Examination Survey	National	both	18+	18+	1277	1756
South Korea	2008	Korea National Health and Nutrition Examination Survey	National	both	18+	18+	2875	3944
South Korea	2009	Korea National Health and Nutrition Examination Survey	National	both	18+	18+	3313	4266

Country	Data years	Survey/study name	Level of representativness		Age range as used for global analysis		Sample size	
				Rural, urban, or both	Male	Female	Male	Female
South Korea	2010	Korea National Health and Nutrition Examination Survey	National	both	18+	18+	2755	3585
South Korea	2011	Korea National Health and Nutrition Examination Survey	National	both	18+	18+	2678	3561
South Korea	2012	Korea National Health and Nutrition Examination Survey	National	both	18+	18+	2530	3481
South Korea	2013	Korea National Health and Nutrition Examination Survey	National	both	18+	18+	2514	3309
Spain	1985	INTERSALT, Manresa	Community	urban	20-59	20-59	100	100
Spain	1986	INTERSALT, Torrejo	Community	urban	20-59	20-59	100	100
Spain	1986-1988	MONICA, Catalonia	Community	urban	25-64	25-64	1251	1271
Spain	1989	Cardiovascular Risk Study in Catalonia	Subnational	both	18+	18+	316	362
Spain	1989-1994	SEEDO	Subnational	both	25-60	25-60	2533	2855
Spain	1990	Banegas et al., Hypertension 32(6):998-1002, 1998	National	both	35-65	35-65	810	1203
Spain	1990-1992	MONICA, Catalonia	Community	urban	25-64	25-64	1719	1191
Spain	1990-2000	SEEDO	Subnational	both	25-60	25-60	4707	5178
Spain	1992	CINDI	Subnational	both	25-64	25-64	1194	1454
Spain	1992	ENCAT	Community	both	18-80	18-80	786	952
Spain	1994	HNSV	Community	both	18+	18+	602	749
Spain	1994-1996	MONICA, Catalonia	Community	urban	25-64	25-64	1800	1628
Spain	1996	Guía Study	Community	urban	30+	30+	305	384
Spain	1996-2002	Castells et al., J Epidemiol Community Health 60(4):316-21, 2006	Community	urban		50-69		26963
Spain	1999-2000	ENIB	Community	both	20-60	20-60	498	702
Spain	1999-2000	Factores de riesgo en las islas Baleares: Estudio CORSAIB	Subnational	both	35-75	35-75	811	864
Spain	2000-2001	Regidor et al., J Hum Hypertens 20(1):73-82, 2006	National	both	60+	60+	1318	2281
Spain	2000-2001	EUREYE Study	Subnational	both	65+	65+	274	324
Spain	2000-2005	CDC of the Canary Islands	Community	both	18-75	18-75	2883	3770
Spain	2001-2002	Catalan Health Interview Survey	Subnational	both	18-74	18-74	597	745
Spain	2002-2003	ENCAT	Community	both	18-80	18-80	712	813
Spain	2003	The European Male Ageing Study	Community	both	40+		405	
Spain	2003-2005	Registre Gironi del Cor	Subnational	both	35-79	35-79	2951	3266
Spain	2004	Vioque J et al. Obesity 2008; 16: 664-670	Community	urban	24+	24+	87	115
Spain	2004	Cardiovascular Risk Study in Castilla y León	Subnational	both	18+	18+	1842	2019
Spain	2004-2006	PREVICTUS	National	both	60+	60+	3193	3640
Spain	2007-2009	Harmonizing Equation of Risk in Mediterraneon countries EXtremadura	Subnational	both	25-79	25-79	1298	1498
Spain	2008	The European Male Ageing Study	Community	both	40+		272	
Spain	2008-2010	Study on Nutrition and Cardiovascular Risk in Spain	National	both	18+	18+	5756	6397
Spain		Soriguer et al., Eur J Epidemiol 19(1):33-40, 2004	Community	rural	18-65	18-65	613	613
Sri Lanka	2003	Wijewardene et al., Ceylon Med J 50:62-70, 2005	Subnational	both	30-65	30-65	275	296
Sri Lanka	2003	Wijewardene et al., Ceylon Med J 50:62-70, 2005	Subnational	both	30-65	30-65	139	192
Sri Lanka	2003	Wijewardene et al., Ceylon Med J 50:62-70, 2005	Subnational	both	30-65	30-65	1891	2410
Sri Lanka	2003	Wijewardene et al., Ceylon Med J 50:62-70, 2005	Subnational	both	30-65	30-65	387	457
Sri Lanka	2006	STEPS	National	both	18-64	18-64	5682	5920
Sri Lanka	2006-2007	DHS	National	both		18-49		12539
Sudan	2005	STEPS	National	both	25-64	25-64	626	881
Swaziland	2006-2007	DHS	National	both	18-49	18-49	3252	3970
Sweden	1972*	The Swedish Conscription Study	National	both	18-19		47452	
Sweden	1973*	The Swedish Conscription Study	National	both	18-19		49837	
Sweden	1974*	The Swedish Conscription Study	National	both	18-19		44124	
Sweden	1975	The Swedish Conscription Study	National	both	18-19		39347	
Sweden	1976	The Swedish Conscription Study	National	both	18-19		35965	
Sweden	1977	The Swedish Conscription Study	National	both	18-19		33234	
Sweden	1978	The Swedish Conscription Study	National	both	18-19		12356	
Sweden	1979	The Swedish Conscription Study	National	both	18-19		20636	
Sweden	1980	The Swedish Conscription Study	National	both	18-19		31886	
Sweden	1980-1984	Uppsala Longitudinal Study of Adult Men	Community	both	60		1841	
Sweden	1981	The Swedish Conscription Study	National	both	18-19		32417	
Sweden	1982	The Swedish Conscription Study	National	both	18-19		32166	
Sweden	1983	The Swedish Conscription Study	National	both	18-19		32130	
Sweden	1984	The Swedish Conscription Study	National	both	18-19		20326	
Sweden	1985	MONICA Gothenburg	Community	urban	25-64	25-64	666	702
Sweden	1985	The Swedish Conscription Study	National	both	18-19		10362	
Sweden	1985-1989	Västerbotten Intervention Project	Subnational	both	25-64	25-64	1676	1554
Sweden	1985-1996	EPIC Umea	Subnational	both	24-72	24-72	12359	13217
Sweden	1986	MONICA, Northern Sweden	Subnational	urban	25-64	25-64	819	795

Country	Data years	Survey/study name	Level of representativness		Age range as used for global analysis		Sample size	
				Rural, urban, or both	Male	Female	Male	Female
Sweden	1986	The Swedish Conscription Study	National	both	18-19		34680	
Sweden	1987	The Swedish Conscription Study	National	both	18-19		39485	
Sweden	1988	The Swedish Conscription Study	National	both	18-19		35942	
Sweden	1989	The Swedish Conscription Study	National	both	18-19		38408	
Sweden	1990	MONICA, Northern Sweden	Subnational	urban	25-64	25-64	761	793
Sweden	1990	MONICA Gothenburg	Community	urban	25-64	25-64	775	775
Sweden	1990	The Swedish Conscription Study	National	both	18-19		39863	
Sweden	1990-1992	Västerbotten Intervention Project	Subnational	both	25-64	25-64	7263	7804
Sweden	1991	The Swedish Conscription Study	National	both	18-19		40433	
Sweden	1991-1995	Uppsala Longitudinal Study of Adult Men	Community	both	70		1215	
Sweden	1991-1996	Malmö Diet and Cancer	Community	urban	45-73	45-73	12096	18293
Sweden	1992	The Swedish Conscription Study	National	both	18-19		38470	
Sweden	1993	The Swedish Conscription Study	National	both	18-19		36756	
Sweden	1993-1995	Västerbotten Intervention Project	Subnational	both	25-64	25-64	9804	10727
Sweden	1994	Helicobacter Pylori	Community	urban	56-65	56-65	170	217
Sweden	1994	MONICA, Northern Sweden	Subnational	urban	25-64	25-64	736	761
Sweden	1994	The Swedish Conscription Study	National	both	18-19		34653	
Sweden	1994-1996	Kungsholmen Project	Community	urban	75+	75+	160	160
Sweden	1995	MONICA Gothenburg	Community	urban	25-64	25-64	745	867
Sweden	1995	The Swedish Conscription Study	National	both	18-19		33711	
Sweden	1996	The Swedish Conscription Study	National	both	18-19		31145	
Sweden	1996-1998	Västerbotten Intervention Project	Subnational	both	25-64	25-64	8327	8893
Sweden	1997	The Swedish Conscription Study	National	both	18-19		29332	
Sweden	1997-2001	Uppsala Longitudinal Study of Adult Men	Community	both	77		783	
Sweden	1998	The Swedish Conscription Study	National	both	18-19		35718	
Sweden	1998-2001	The Kalixanda study	Community	both	20+	20+	508	483
Sweden	1999	MONICA Northern Sweden	Subnational	both	25-74	25-74	889	930
Sweden	1999	The Swedish Conscription Study	National	both	18-19		31841	
Sweden	1999-2003	Västerbotten Intervention Project	Subnational	both	25-64	25-64	6354	6384
Sweden	2000	The Swedish Conscription Study	National	both	18-19		25534	
Sweden	2000-2001	H70 Study	Community	urban	70	70	242	270
Sweden	2001	The Swedish Conscription Study	National	both	18-19		25241	
Sweden	2001-2003	Uppsala Longitudinal Study of Adult Men	Community	both	82		511	
Sweden	2001-2004	Swedish INTERGENE Cohort Study	Subnational	both	24-76	24-76	1694	1906
Sweden	2001-2004	Prospective Investigation of the Vasculature in Uppsala Seniors (PIVUS)	Community	both	70	70	507	509
Sweden	2002	The Swedish Conscription Study	National	both	18-19		21219	
Sweden	2003	The European Male Ageing Study	Community	both	40+		396	
Sweden	2003	The Swedish Conscription Study	National	both	18-19		26570	
Sweden	2003-2004	Welin et al., BMC Public Health 8:403, 2008	Community	urban	50	50	595	655
Sweden	2003-2004	Welin et al., BMC Public Health 8:403, 2008	Community	urban	60		667	
Sweden	2004	MONICA Northern Sweden	Subnational	both	25-74	25-74	908	958
Sweden	2004	The Swedish Conscription Study	National	both	18-19		24904	
Sweden	2004-2005	Population Study of Women in Gothenburg	Community	urban		38-50		494
Sweden	2005-2006	H70 Study	Community	urban	75	75	320	422
Sweden	2006-2009	Prospective Investigation of the Vasculature in Uppsala Seniors (PIVUS)	Community	both	75	75	407	419
Sweden	2008	The European Male Ageing Study	Community	both	40+		353	
Sweden	2009	MONICA Northern Sweden	Subnational	both	25-74	25-74	849	861
Sweden	2014	MONICA Northern Sweden	Subnational	both	25-74	25-74	753	801
Switzerland	1984-1986	The Swiss MONICA Study Wave I	Subnational	both	25-74	25-74	1744	1689
Switzerland	1988-1989	The Swiss MONICA Study Wave II	Subnational	both	25-74	25-74	1778	1684
Switzerland	1992-1993	The Swiss MONICA Study Wave III	Subnational	both	25-74	25-74	1577	1672
Switzerland	2003-2006	Cohorte Lausannoise	Community	urban	35-75	35-75	3186	3536
Switzerland	2004	Body Mass Index of Swiss conscripts	National	both	18-20		20491	
Switzerland	2005	Body Mass Index of Swiss conscripts	National	both	18-21		32131	
Switzerland	2006	Body Mass Index of Swiss conscripts	National	both	18-22		34530	
Switzerland	2007	Body Mass Index of Swiss conscripts	National	both	18-23		36194	
Switzerland	2007-2012	Bus Santé Study	Subnational	both	20-80	20-80	1884	1911
Switzerland	2008	Body Mass Index of Swiss conscripts	National	both	18-24		34497	
Switzerland	2009	Body Mass Index of Swiss conscripts	National	both	18-25		34896	
Switzerland	2009-2012	Cohorte Lausannoise	Community	urban	40-81	40-81	2176	2494
Switzerland	2010	Body Mass Index of Swiss conscripts	National	both	18-26		37214	

Country	Data years	Survey/study name	Level of representativness		Age range as used for global analysis		Sample size	
				Rural, urban, or both	Male	Female	Male	Female
Switzerland	2011	Body Mass Index of Swiss conscripts	National	both	18-27		38108	
Switzerland	2012	Body Mass Index of Swiss conscripts	National	both	18-28		36938	
Syrian Arab Republic	2002	National Survey on non-communicable diseases and factors affecting their development	National	both	18-64	18-64	3155	4045
Taiwan	1985	INTERSALT	Community	rural	20-59	20-59	89	92
Taiwan	1989-1991	Chiu et al., J Gerontol A Biol Sci Med Sci 55(11):M684-90, 2000	Subnational	both	65+	65+	1322	1308
Taiwan	1993-1994	The Kinmen Neurological Disorders Survey	Community	urban	50+	50+	672	593
Taiwan	1993-1996	Nutrition and Health Survey in Taiwan 1993-1996	National	both	18+	18+	1506	1704
Taiwan	1999-2000	Nutrition and Health Survey in Taiwan 1999-2000	National	both	65+	65+	1271	1202
Taiwan	2002	Taiwanese Survey on Hypertension, Hyperglycemia and Hyperlipidemia	National	both	18+	18+	3293	3313
Taiwan	2004-2005	TCHS	Community	urban	40+	40+	1147	1212
Taiwan	2005-2008	Nutrition and Health Survey in Taiwan 2005-2008	National	both	19+	19+	1311	1355
Taiwan	2007	Taiwanese Survey on Hypertension, Hyperglycemia and Hyperlipidemia	National	both	20+	20+	2155	2469
Tajikistan	2003	Micronutrient Status Survey	National	both		18-49		1806
Tajikistan	2012	DHS	National	both		18-49		7724
Tanzania	1991-1992	DHS	National	both		18-49		4405
Tanzania	1996	DHS	National	both		18-49		3766
Tanzania	1996-1997	Aspray et al., Trans R Soc Trop Med Hyg 94:637-44, 2000	Community	rural	18+	18+	251	324
Tanzania	1996-1997	Aspray et al., Trans R Soc Trop Med Hyg 94:637-44, 2000	Community	urban	18+	18+	117	118
Tanzania	1998-1999	Bovet et al., Int J Epidemiol 31(1):240-7, 2002	Community	urban	25-64	25-64	3593	5646
Tanzania	2004-2005	DHS	National	both		18-49		7854
Tanzania	2010	DHS	National	both		18-49		7746
Tanzania	2011	STEPS	Subnational	both	25-64	25-64	1008	1517
Tanzania	2012	STEPS	National	both	25-64	25-64	2581	2820
Tanzania	2014	Dar es Salaam Urban Cohort Hypertension Study	Community	urban	40+	40+	965	1266
Thailand	1987	INCLEN	Community	rural	35-65		244	
Thailand	1989	INCLEN	Community	rural	35-65		209	
Thailand	1989	INCLEN	Community	urban	35-65		207	
Thailand	1991	Thailand National Health Examination Survey I	National	both	18+	18+	5771	8140
Thailand	1995	The Fourth National Nutrition Survey of Thailand- 1995	National	both	20-60	20-60	1405	3631
Thailand	1997	Thailand National Health Examination Survey II	National	both	18-59	18-59	1183	2023
Thailand	2000	InterASIA	National	both	35+	35+	2092	3211
Thailand	2003-2004	The Fifth National Nutrition Survey of Thailand	National	both	19+	19+	1961	3366
Thailand	2004	Thailand National Health Examination Survey III	National	both	18+	18+	18442	19856
Thailand	2009	Thailand National Health Examination Survey IV	National	both	18-98	18-98	9262	10164
Timor-Leste	2009-2010	DHS	National	both		18-49		10009
Timor-Leste	2009-2010	Timor-Leste Eye Health Survey	Subnational	both	40+	40+	245	247
Togo	1998	DHS	National	both		18-49		3277
Togo	2010	STEPS	National	both	18-64	18-64	1897	1961
Togo	2013-2014	DHS	National	both		18-49		3877
Tokelau	2005	STEPS	National	both	18-64	18-64	241	267
Tokelau	2014	Global School-based Student Health Survey	National	both	18-19		2	
Tonga	2004	STEPS	National	both	18-64	18-64	390	546
Tonga	2005-2007	Pacific Obesity Prevention in Communities - Ma’alahi Youth Project	Subnational	both	18-19	18-19	105	137
Tonga	2007-2008	Pacific Obesity Prevention in Communities - Ma’alahi Youth Project	Subnational	both	18-22	18-22	144	218
Tonga	2011	STEPS	National	both	18-64	18-64	878	1401
Trinidad and Tobago	1985	INTERSALT	Community	urban	20-59	20-59	84	92
Trinidad and Tobago	2001	Adult Survey	National	both	25+	25+	198	267
Tunisia	1996-1997	Arian Healthy Project 1997	Community	both	35-65	35-65	2664	2711
Tunisia	1996-1997	Tunisian National Nutrition Survey 1996-1997	National	both	18+	18+	1397	2674
Tunisia	2005	Aounallah et al., Public Health 12(1):98, 2012	National	both	18-19	18-19	451	583
Tunisia	2005	Tunisian National Survey	National	both	35-71	35-71	3417	4590
Tunisia	2009-2010	ObeMaghreb	Subnational	urban	18-49	18-49	998	696
Turkey	1990	Turkish Adult Risk Factor Study	National	both	20+	20+	1338	1369
Turkey	1993	DHS	National	both		18-49		2396
Turkey	1998	DHS	National	both		18-49		2305
Turkey	1998	Turkish Adult Risk Factor Study	National	both	28+	28+	877	909
Turkey	1998-1999	Erem et al., Diabetes Res Clin Pract 54(3):203-08, 2001	Community	urban	20+	20+	1324	1322
Turkey	2000	MDHS	Subnational	urban		18-49		1420
Turkey	2000	Turkish Adult Risk Factor Study	National	both	30+	30+	890	938
Turkey	2000-2002	The Healthy Nutrition for Healthy Heart Study	National	both	25-84	25-84	4718	10631
Turkey	2001	Yumuk et al., Diabetes Res Clin Pract 70(2):151-58, 2005	Community	urban	20+	20+	1042	1789

Country	Data years	Survey/study name	Level of representativness		Age range as used for global analysis		Sample size	
				Rural, urban, or both	Male	Female	Male	Female
Turkey	2001-2002	Turkish Adult Risk Factor Study	National	both	32+	32+	1098	1210
Turkey	2002	Onal et al., Blood Press 13(1):31-6, 2004	Subnational	urban	25+	25+	67	355
Turkey	2003	DHS	National	both		18-49		3014
Turkey	2003	Prevalence, awareness, treatment and control of hypertension in Turkey in 2003	National	both	18+	18+	1988	2847
Turkey	2003-2004	Turkish Adult Risk Factor Study	National	both	34+	34+	479	987
Turkey	2004	Nationally Representative Cross-sectional Survey	National	both	20+	20+	2110	2154
Turkey	2005-2006	Turkish Adult Risk Factor Study	National	both	33+	33+	1029	1088
Turkey	2007	Natinal Household survey	National	both	20-85	20-85	2263	1842
Turkey	2007-2008	Turkish Adult Risk Factor Study	National	both	35+	35+	1101	1133
Turkey	2009-2010	Turkish Adult Risk Factor Study	National	both	37+	37+	466	507
Turkey	2009-2012	Prevalence of diabetes and associated risk factors among adult population in Trabzon city	Subnational	both	20+	20+	1583	2138
Turkey	2011	Chronic Disease Risk Factor Survey	National	both	18+	18+	7434.65	8317.07
Turkey	2012-2013	Turkish Adult Risk Factor Study	National	both	37+	37+	1012	1087
Turkmenistan	2000	DHS	National	both		18-49		2084
Turkmenistan	2013	STEPS	National	both	18-64	18-64	1929	2858
Tuvalu	1976	The Funafuti Survey	Subnational	both	18+	18+	280	289
Uganda	1995	DHS	National	both		18-49		3117
Uganda	2000-2001	DHS	National	both		18-49		4984
Uganda	2006	DHS	National	both	18-54	18-49	2092	2140
Uganda	2011	DHS	National	both	18-54	18-49	1988	2120
Uganda	2011-2012	The Prevalence and Distribution of Non-communicable Diseases and Their Risk Factors in Kasese District, Uganda	Subnational	both	25-79	25-79	277	221
Ukraine	2002	National Micronutrient Survey	National	both		18-50		816
United Arab Emirates	1989-1990	el Mugamer et al., J Trop Med Hyg 98(6):407-15, 1995	Community	both	20+	20+	123	199
United Arab Emirates	1999-2000	Emirates National Diabetes and Coronary Artery Disease Risk Factor Study	National	both	20-80	20-80	2822	3743
United Arab Emirates	2000-2001	carter et al., J Health Popul Nutr 22(1):75-83, 2004	Community	both		20-79		521
United Kingdom	1982	MRC National Survey of Health and Development	National	both	36	36	1632	1648
United Kingdom	1983-1984	MONICA, Belfast	Subnational	both	25-64	25-64	1158	1183
United Kingdom	1984-1986	Scottish Heart Health Survey	Subnational	both	40-59	40-59	4364	4464
United Kingdom	1985	INTERSALT, Birmingham	Community	urban	20-59	20-59	100	100
United Kingdom	1985	INTERSALT, Wales	Community	urban	20-59	20-59	100	99
United Kingdom	1985-1986	INTERSALT, Belfast	Community	urban	20-59	20-59	99	100
United Kingdom	1986-1987	Dietary and Nutritional Survey of British Adults 1986-1987	National	both	18-64	18-64	1100	1125
United Kingdom	1986-1987	MONICA, Belfast	Subnational	both	25-64	25-64	1155	1185
United Kingdom	1987-1988	Edinburgh Artery Study	Community	urban	54-75	54-75	808	783
United Kingdom	1989	MRC National Survey of Health and Development	National	both	43	43	1617	1608
United Kingdom	1991	National Child Development Study (1958 British Cohort Study)	National	both	33	33	5426	5606
United Kingdom	1991-1992	Health Survey for England	National	both	18+	18+	3004	3331
United Kingdom	1991-1992	MONICA, Belfast	Subnational	both	25-64	25-64	998	996
United Kingdom	1992	MONICA, Glasgow	Community	urban	25-64	25-64	696	775
United Kingdom	1992-1993	Whickham Survey	Community	urban	35+	35+	676	784
United Kingdom	1992-1994	Edinburgh Artery Study	Community	urban	60-81	60-81	580	582
United Kingdom	1993	Health Survey for England	National	both	18+	18+	7225	8060
United Kingdom	1993-1997	EPIC Norfolk	Subnational	both	40-79	40-79	11574	13995
United Kingdom	1993-2000	EPIC Oxford	Subnational	both	20-98	20-98	10851	37605
United Kingdom	1994	Health Survey for England	National	both	18+	18+	6628	7725
United Kingdom	1994-1995	National Diet and Nutrition Survey 1994-1995	National	both	65+	65+	701	687
United Kingdom	1995	Health Survey for England	National	both	18+	18+	6525	7483
United Kingdom	1995	MONICA, Glasgow	Community	urban	25-64	25-64	855	958
United Kingdom	1995	Scottish Healthy Survey 1995	Subnational	both	18-64	18-64	3208	3903
United Kingdom	1996	Health Survey for England	National	both	18+	18+	6751	7793
United Kingdom	1997	Health Survey for England	National	both	18+	18+	3583	4109
United Kingdom	1997	National Diet and Nutrition Survey 1997	National	both	18-18	18-18	49	53
United Kingdom	1997-1999	International collaborative study of macronutrients, micronutrients and blood pressure	Community	urban	40-59	40-59	141	138
United Kingdom	1998	Health Survey for England	National	both	18+	18+	6382	7497
United Kingdom	1998	Scottish Healthy Survey 1998	Subnational	both	18-74	18-74	3506	4444
United Kingdom	1998-1999	International collaborative study of macronutrients, micronutrients and blood pressure	Community	urban	40-59	40-59	125	97
United Kingdom	1998-2000	The British Regional Heart Study	National	urban	60-79		4138	
United Kingdom	1999	Health Survey for England	National	both	18+	18+	3104	3574
United Kingdom	1999	MRC National Survey of Health and Development	National	both	53	53	1452	1496
United Kingdom	1999-2001	British Women's Heart and Health Study	National	both		60-79		3678
United Kingdom	1999-2001	Edinburgh Artery Study	Community	urban	66-87	66-87	373	404

Country	Data years	Survey/study name	Level of representativness		Age range as used for global analysis		Sample size	
				Rural, urban, or both	Male	Female	Male	Female
United Kingdom	1999-2004	Hertfordshire Cohort Study	Subnational	both	59-73	59-73	1571	1416
United Kingdom	2000	Health Survey for England	National	both	18+	18+	3254	3830
United Kingdom	2000-2001	National Diet and Nutrition Survey 2000-2001	National	both	19-64	19-64	807	973
United Kingdom	2001	Health Survey for England	National	both	18+	18+	6073	7197
United Kingdom	2002	Health Survey for England	National	both	18+	18+	3703	4507
United Kingdom	2003	The European Male Ageing Study	Community	both	40+		394	
United Kingdom	2003	Health Survey for England	National	both	18+	18+	5787	6856
United Kingdom	2003	Scottish Healthy Survey 2003	Subnational	both	18+	18+	2922	3583
United Kingdom	2004	Health Survey for England	National	both	18+	18+	2365	3050
United Kingdom	2004-2005	English Longitudinal Study of Ageing Wave 2 2004-2005	National	both	52+	52+	3259	3966
United Kingdom	2005	Health Survey for England	National	both	18+	18+	3768	4422
United Kingdom	2006	Health Survey for England	National	both	18+	18+	5359	6298
United Kingdom	2006-2011	MRC National Survey of Health and Development	National	both	60-64	60-64	1061	1157
United Kingdom	2007	Health Survey for England	National	both	18+	18+	2633	3122
United Kingdom	2008	The European Male Ageing Study	Community	both	40+		301	
United Kingdom	2008	Health Survey for England	National	both	18+	18+	5660	6767
United Kingdom	2008	Scottish Healthy Survey 2008	Subnational	both	18+	18+	2392	2950
United Kingdom	2008-2009	English Longitudinal Study of Ageing Wave 4 2008-2009	National	both	50+	50+	3540	4296
United Kingdom	2008-2011	National Diet and Nutrition Survey 2008/09-2010/11	National	both	18+	18+	638	803
United Kingdom	2009	Health Survey for England	National	both	18+	18+	1790	2054
United Kingdom	2009	Scottish Healthy Survey 2009	Subnational	both	18+	18+	2757	3380
United Kingdom	2010	Health Survey for England	National	both	18+	18+	3046	3737
United Kingdom	2010	Scottish Healthy Survey 2010	Subnational	both	18+	18+	2616	3257
United Kingdom	2011	Health Survey for England	National	both	18+	18+	3099	3769
United Kingdom	2011	Scottish Healthy Survey 2011	Subnational	both	18+	18+	2688	3311
United Kingdom	2012	Health Survey for England	National	both	18+	18+	3033	3687
United Kingdom	2012	Scottish Healthy Survey 2012	Subnational	both	18+	18+	1835	2175
United Kingdom	2012-2013	English Longitudinal Study of Ageing Wave 6 2012-2013	National	both	50+	50+	3257	4015
United Kingdom	2013	Health Survey for England	National	both	18+	18+	3246	3882
United States of America	1971-1975*	US NHANES I	National	both	18-74	18-74	5250	8195
United States of America	1976-1980	US NHANES II	National	both	18-74	18-74	5919	6583
United States of America	1979-1980	MONICA, Stanford	Subnational	urban	25-64	25-64	703	806
United States of America	1980-1982	The Minnesota Heart Survey	Community	both	25-75	25-75	1611	1837
United States of America	1985-1986	INTERSALT, Chicago	Community	urban	20-59	20-59	97	99
United States of America	1985-1986	MONICA, Stanford	Subnational	urban	25-64	25-64	713	848
United States of America	1985-1987	The Minnesota Heart Survey	Community	both	25-75	25-75	5220	2421
United States of America	1986	INTERSALT, Goodman	Community	urban	20-59	20-59	192	192
United States of America	1986	INTERSALT, Jackson	Community	urban	20-59	20-59	184	199
United States of America	1988-1994	US NHANES III	National	both	18+	18+	8271	9096
United States of America	1989-1990	MONICA, Stanford	Subnational	urban	25-64	25-64	720	842
United States of America	1996	National Longitudinal Study of Adolescent Health Wave II	National	both	18-21	18-21	503	449
United States of America	1996-1997	International collaborative study of macronutrients, micronutrients and blood pressure	Community	urban	40-59	40-59	146	134
United States of America	1996-1997	International collaborative study of macronutrients, micronutrients and blood pressure	Community	urban	40-59	40-59	132	134
United States of America	1996-1997	International collaborative study of macronutrients, micronutrients and blood pressure	Community	urban	40-59	40-59	132	128
United States of America	1996-1997	Study of Women's Health Across the Nation	National	both		40-55		3199
United States of America	1996-1998	International collaborative study of macronutrients, micronutrients and blood pressure	Community	urban	40-59	40-59	130	130
United States of America	1997-1998	International collaborative study of macronutrients, micronutrients and blood pressure	Community	urban	40-59	40-59	271	276
United States of America	1997-1998	International collaborative study of macronutrients, micronutrients and blood pressure	Community	urban	40-59	40-59	156	159
United States of America	1997-1999	Study of Women's Health Across the Nation	National	both		40-55		2760
United States of America	1998-2000	Study of Women's Health Across the Nation	National	both		40-55		2596
United States of America	1999-2000	US NHANES 1999-2000	National	both	18+	18+	2301	2319
United States of America	1999-2001	Study of Women's Health Across the Nation	National	both		40-56		2507
United States of America	2000-2002	Study of Women's Health Across the Nation	National	both		40-57		2440
United States of America	2001-2002	National Longitudinal Study of Adolescent Health Wave III	National	both	18-28	18-28	2139	2443
United States of America	2001-2002	US NHANES 2001-2002	National	both	18+	18+	2506	2419
United States of America	2003-2004	US NHANES 2003-2004	National	both	18+	18+	2503	2446
United States of America	2005-2006	US NHANES 2005-2006	National	both	18+	18+	2511	2372
United States of America	2005-2006	National Social Life Health and Aging Project	National	both	57-85	57-85	1355	1435
United States of America	2007-2008	US NHANES 2007-2008	National	both	18+	18+	2902	2925
United States of America	2008-2009	National Longitudinal Study of Adolescent Health Wave IV	National	both	24-34	24-34	2317	2725
United States of America	2009-2010	US NHANES 2009-2010	National	both	18+	18+	3051	3165

Country	Data years	Survey/study name	Level of representativness	Rural, urban, or both	Age range as used for global analysis		Sample size	
					Male	Female	Male	Female
United States of America	2010-2011	National Social Life Health and Aging Project	National	both	36-99	36-99	1452	1738
United States of America	2011-2012	US NHANES 2011-2012	National	both	18+	18+	2737	2731
Uruguay	1999-2000	The Survey on Health, Well-Being, and Aging in Latin America and the Caribbean (SABE)	Community	urban	60+	60+	492	828
Uruguay	2006	STEPS	National	both	25-64	25-64	261	641
Uruguay	2011-2012	CESCAS Study	Community	urban	35-74	35-74	650	927
Uzbekistan	1996	DHS	National	both		18-49		3499
Uzbekistan	2002	DHS	National	both	18-59	18-49	2062	4559
Vanuatu	1996	Second National Nutrition Survey	National	both		18-50		1353
Vanuatu	1998	Vanuatu Non-comunicable Disease Survey	National	both	20-60	20-60	533	730
Vanuatu	2005	STEPS	Subnational	both	18-60	18-60	583	701
Vanuatu	2011	STEPS	National	both	25-64	25-64	2251	2181
Venezuela	1999-2001	Florez et al., Diabetes Res Clin Pract 69(1):63-77, 2005	Subnational	both	18+	18+	1134	2599
Venezuela	2000	Diaz et al., Invest Clin 46(2):111-19, 2005	Community	urban	60+	60+	42	59
Venezuela	2004-2005	CArdiovascular Risk factors Multiple Evaluation in Latin America	Community	urban	25-64	25-64	713	1123
Venezuela	2005-2006	Brajkovich et al., Rev Ven Endoc Metab 4(3):31-32, 2006	Community	urban	20-65	20-65	205	439
Venezuela	2007-2008	Venezuelan Study of Metabolic Syndrome, Obesity and Lifestyle (VEMSOLS)	Community	urban	20+	20+	107	230
Venezuela	2008-2009	Venezuelan Study of Metabolic Syndrome, Obesity and Lifestyle (VEMSOLS)	Community	rural	20+	20+	51	89
Venezuela	2010-2011	Venezuelan Study of Metabolic Syndrome, Obesity and Lifestyle (VEMSOLS)	Community	urban	20+	20+	66	193
Viet Nam	1981-1985	National Nutrition Survey	Subnational	rural	18+	18+	4815	7985
Viet Nam	1987-1989	General Nutrition Survey	National	both	18-70	18-70	13776	17271
Viet Nam	1992-1993	Living Standard Survey	National	both	18+	18+	5620	6497
Viet Nam	1997-1998	Living Standard Survey	National	both	18+	18+	7509	8761
Viet Nam	2000	General Nutrition Study	National	both	20-59	20-59	9039	9328
Viet Nam	2000	National Nutrition Survey	National	both	20+	20+	8985	9464
Viet Nam	2001-2002	Viet Nam National Health Survey 2001-2002	National	both	18+	18+	42413	48738
Viet Nam	2004	Cuong et al., Eur J Clin Nutr 61(5):673-81, 2007	Community	urban	20-60	20-60	717	771
Viet Nam	2005	STEPS Bavi district	Subnational	rural	25-64	25-64	987	997
Viet Nam	2005	National Adult Overweight Survey	National	both	25-64	25-64	8474	8725
Viet Nam	2005	Non-communicable disease risk factors in Ho Chi Minh City	Community	both	25-64	25-64	908	1063
Viet Nam	2008-2009	The survey on diabetes and its risk factors in 2 northern provinces of Vietnam (DM-S)	Subnational	both	25+	25+	830	1446
Viet Nam	2009	STEPS	National	both	25-64	25-64	6738	7804
Viet Nam	2009-2010	General Nutrition Survey	National	both	20+	20+	10810	11729
Viet Nam	2013	Global School-based Student Health Survey	National	both	18-19	18-19	121	140
Yemen	1997	DHS	National	both		18-49		5123
Yemen	2005-2006	Yemen Household Budget Survey 2005-2006	National	both	18+	18+	1557	1738
Yemen	2013	DHS	National	both		18-49		18900
Zambia	1992	DHS	National	both		18-49		3175
Zambia	1996	DHS	National	both		18-49		3789
Zambia	2001-2002	DHS	National	both		18-49		5800
Zambia	2003	Kelly et al., Am J Clin Nut 88(4):1010-17, 2008	Community	urban	18-74	18-84	132	217
Zambia	2007	DHS	National	both		18-49		5459
Zambia	2008	STEPS	Community	urban	25+	25+	626	1214
Zambia	2013-2014	DHS	National	both		18-49		12813
Zimbabwe	1985-1986	INTERSALT	Community	urban	20-59	20-59	100	95
Zimbabwe	1991	Zinyowera et al., Cent Afr J Med 40(2):33-8, 1994	Community	both	18+	18+	775	734
Zimbabwe	1994	DHS	National	both		18-49		1934
Zimbabwe	1995	Mufunda et al., J Hum Hypertens 14(1):65-73, 2000	Community	urban	25+	25+	384	391
Zimbabwe	1999	DHS	National	both		18-49		4324
Zimbabwe	2005	STEPS	National	both	25-64	25-64	431	1556
Zimbabwe	2005-2006	DHS	National	both		18-49		7017
Zimbabwe	2010-2011	DHS	National	both	18-54	18-49	6284	7216

* National studies for the 3 years prior to 1975 were assigned to 1975 so that they can inform the estimates in countries with older national data.

† This research uses data from China Health and Nutrition Survey (CHNS). We thank the National Institute of Nutrition and Food Safety, China Center for Disease Control and Prevention, Carolina Population Center (5 R24 HD050924), the University of North Carolina at Chapel Hill, the NIH (R01-HD30880, DK056350, R24 HD050924, and R01-HD38700) and the Fogarty International Center, NIH for financial support for the CHNS data collection and analysis files from 1989 to 2011 and future surveys, and the China-Japan Friendship Hospital, Ministry of Health for support for CHNS 2009.

Appendix Table 3: Coefficients of regressions to estimate the prevalences of different BMI categories when information was available on mean BMI. The dependent variables in all regressions were prevalences, fitted using a generalised linear mixed model fitted with a probit link. For each regression, 11,595 data points were used to fit the model.

BMI <18.5 kg/m^2	
**Variable**	**Coefficient (95% CI)**
Intercept	-7.38 (-7.49, -7.26)
Inverse mean BMI	121.0 (120.0, 122.0)
Mean age of age group	-0.00061 (-0.00144, 0.00023)
Male sex	-0.79 (-0.82, -0.76)
Study mid-year (per one more recent year since 1975)	0.00091 (0.00075, 0.00107)
Natural logarithm of per-capita gross domestic product (GDP)	0.074 (0.071, 0.077)
Inverse mean BMI * mean age of age group	0.078 (0.059, 0.097)
Inverse mean BMI * male sex	14.4 (13.8, 15.1)

Traditional R^2 is not clearly defined for mixed-effect models. The pseudo-R^2 for the model, which describes the proportion of variance explained by both fixed and random factors, was 0.90.

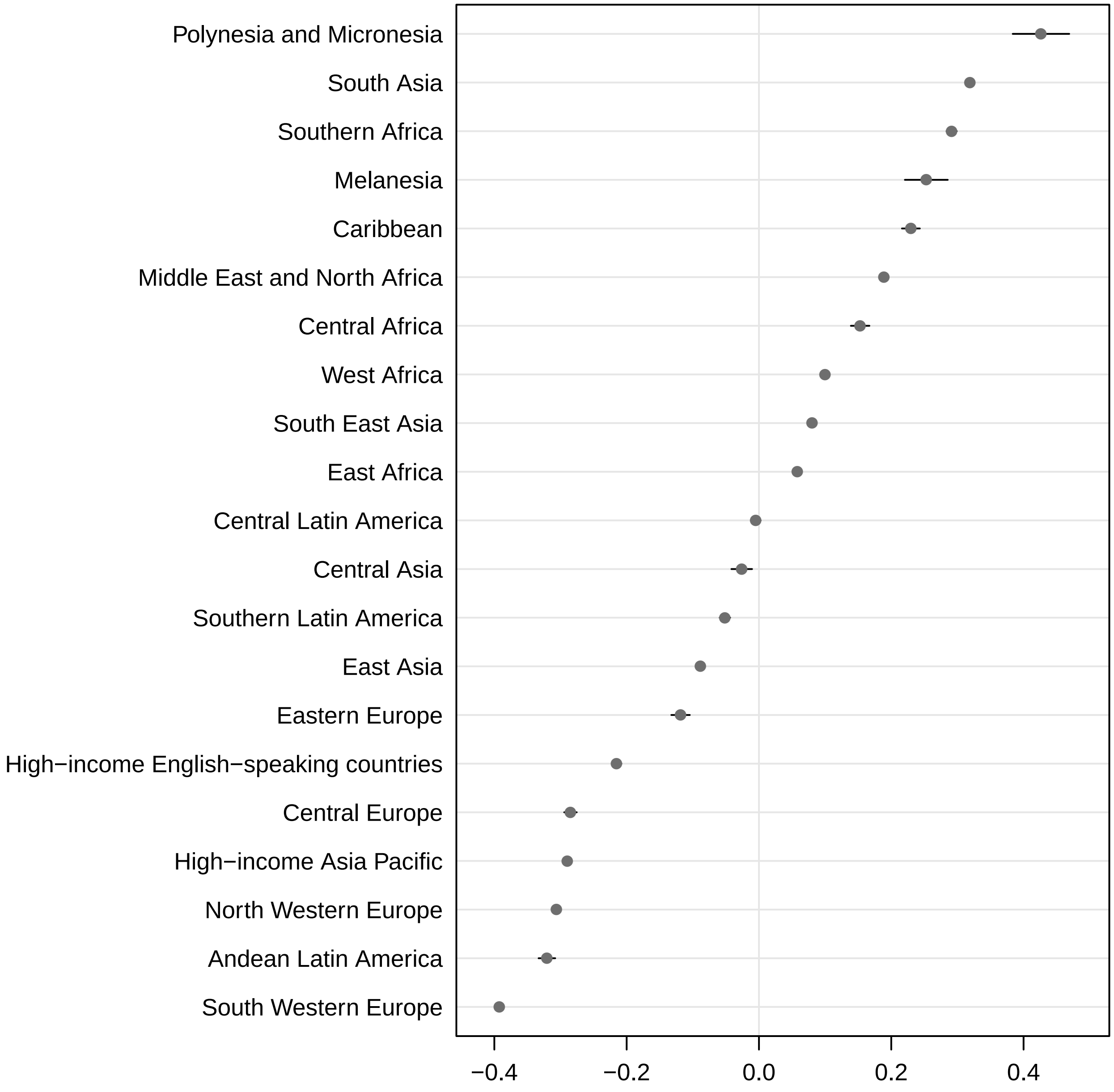

Polynesia and Micronesia
South Asia
Southern Africa
Melanesia
Caribbean
Middle East and North Africa
Central Africa
West Africa
South East Asia
East Africa
Central Latin America
Central Asia
Southern Latin America
East Asia
Eastern Europe
High−income English−speaking countries
Central Europe
High−income Asia Pacific
North Western Europe
Andean Latin America
South Western Europe
−0.4
−0.2
0.0
0.2
0.4

18.5 ≤ BMI <20 kg/m^2	
**Variable**	**Coefficient (95% CI)**
Intercept	-0.40 (-0.44, -0.35)
Inverse (mean BMI – 19.25)	-90.7 (-91.6, -89.8)
Mean age of age group	-0.0030 (-0.0031, -0.0028)
Male sex	0.155 (0.150, 0.161)
Study mid-year (per one more recent year since 1975)	0.00027 (0.00013, 0.00040)
Natural logarithm of per-capita gross domestic product (GDP)	0.0134 (0.0108, 0.0160)
[Inverse (mean BMI – 19.25)] * mean age of age group	-0.054 (-0.071, -0.036)
[Inverse (mean BMI – 19.25)] * male sex	-26.3 (-26.9, -25.7)

Traditional R^2 is not clearly defined for mixed-effect models. The pseudo-R^2 for the model, which describes the proportion of variance explained by both fixed and random factors, was 0.87.

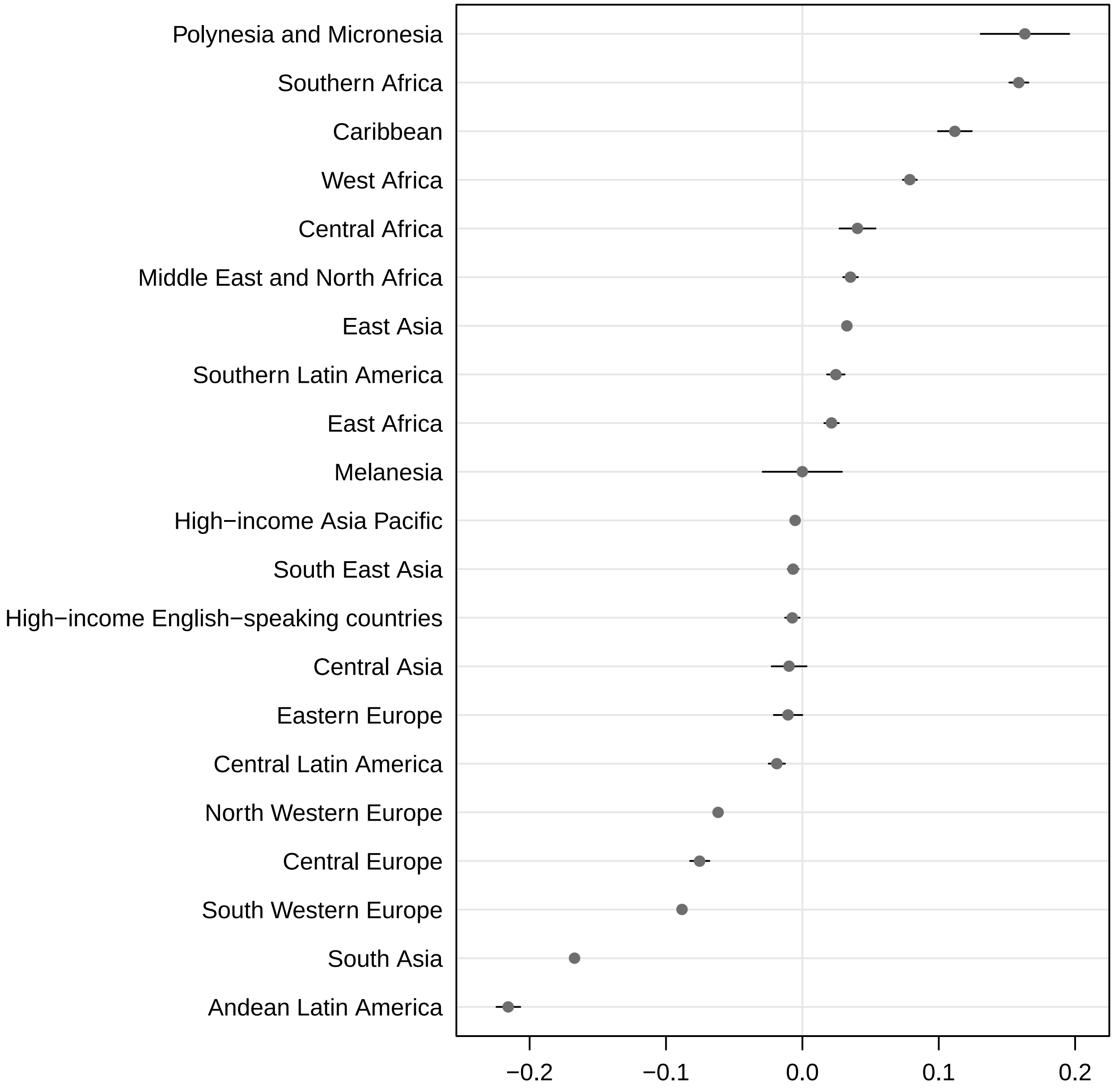
Polynesia and Micronesia
Southern Africa
Caribbean
West Africa
Central Africa
Middle East and North Africa
East Asia
Southern Latin America
East Africa
Melanesia
High−income Asia Pacific
South East Asia
High−income English−speaking countries
Central Asia
Eastern Europe
Central Latin America
North Western Europe
Central Europe
South Western Europe
South Asia
Andean Latin America
−0.2
−0.1
0.0
0.1
0.2

$20 \le$ BMI <25 kg/m^2	
**Variable**	**Coefficient (95% CI)**
Intercept	0.46 (0.42, 0.50)
Inverse (mean BMI – 22.5)	-58.3 (-59.2, -57.4)
Mean age of age group	0.00049 (0.00041, 0.00057)
Male sex	0.144 (0.142, 0.147)
Study mid-year (per one more recent year since 1975)	-0.0024 (-0.0025, -0.0023)
Natural logarithm of per-capita gross domestic product (GDP)	-0.020 (-0.022, -0.018)
[Inverse (mean BMI – 22.5)] * mean age of age group	-0.69 (-0.71, -0.67)
[Inverse (mean BMI – 22.5)] * male sex	-25.7 (-26.2, -25.1)

Traditional R^2 is not clearly defined for mixed-effect models. The pseudo-R^2 for the model, which describes the proportion of variance explained by both fixed and random factors, was 0.85.

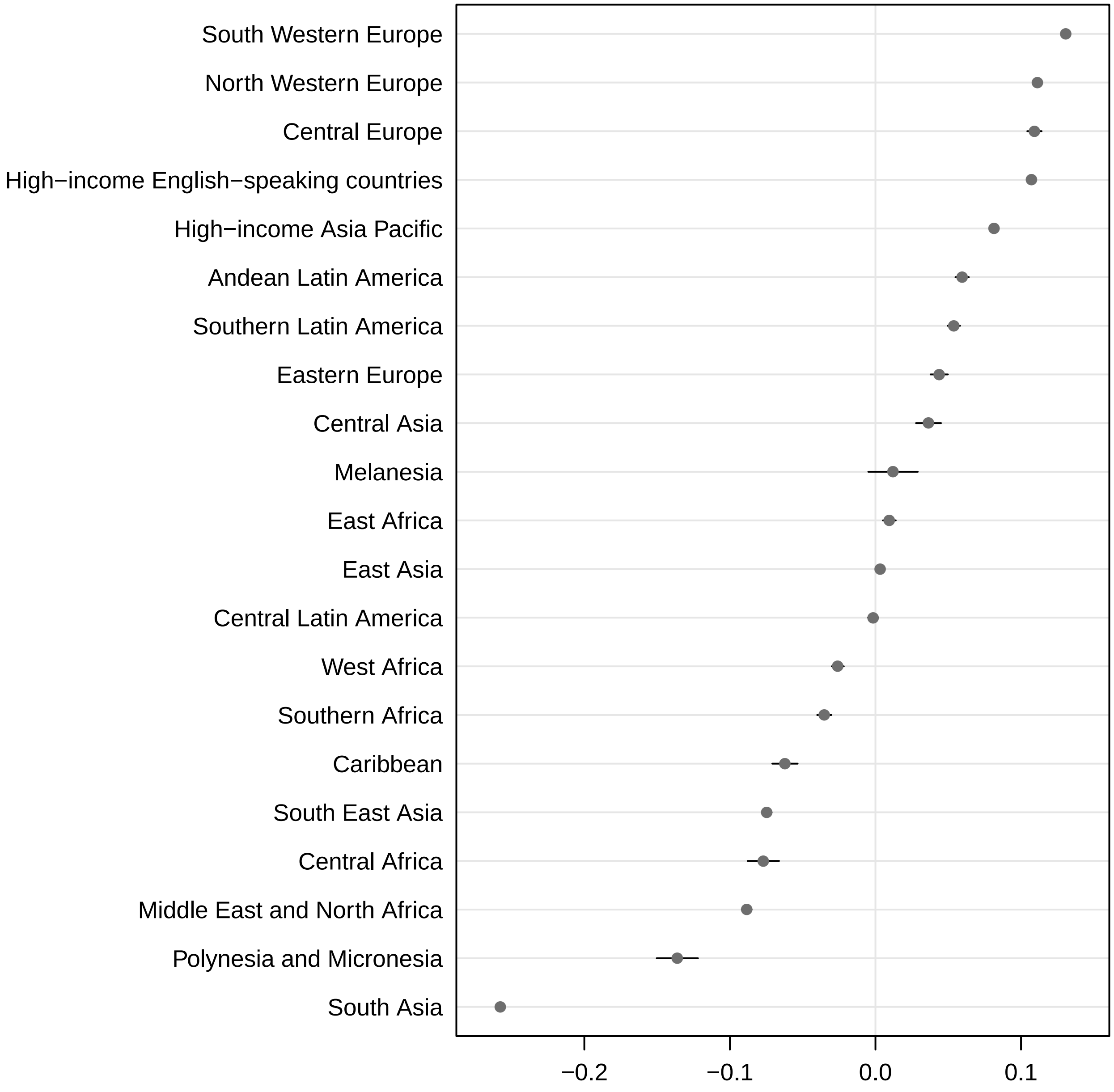
South Western Europe
North Western Europe
Central Europe
High−income English−speaking countries
High−income Asia Pacific
Andean Latin America
Southern Latin America
Eastern Europe
Central Asia
Melanesia
East Africa
East Asia
Central Latin America
West Africa
Southern Africa
Caribbean
South East Asia
Central Africa
Middle East and North Africa
Polynesia and Micronesia
South Asia
−0.2
−0.1
0.0
0.1

25 ≤ BMI <30 kg/m^2	
**Variable**	**Coefficient (95% CI)**
Intercept	-0.41 (-0.45, -0.38)
Inverse (mean BMI – 27.5)	-120.0 (-121.0, -120.0)
Mean age of age group	0.00089 (0.00079, 0.00100)
Male sex	0.290 (0.287, 0.293)
Study mid-year (per one more recent year since 1975)	-0.00025 (-0.00036, -0.00013)
Natural logarithm of per-capita gross domestic product (GDP)	0.022 (0.020, 0.024)
[Inverse (mean BMI – 27.5)] * mean age of age group	0.44 (0.43, 0.46)
[Inverse (mean BMI – 27.5)] * male sex	-36.2 (-36.6, -35.7)

Traditional R^2 is not clearly defined for mixed-effect models. The pseudo-R^2 for the model, which describes the proportion of variance explained by both fixed and random factors, was 0.91.

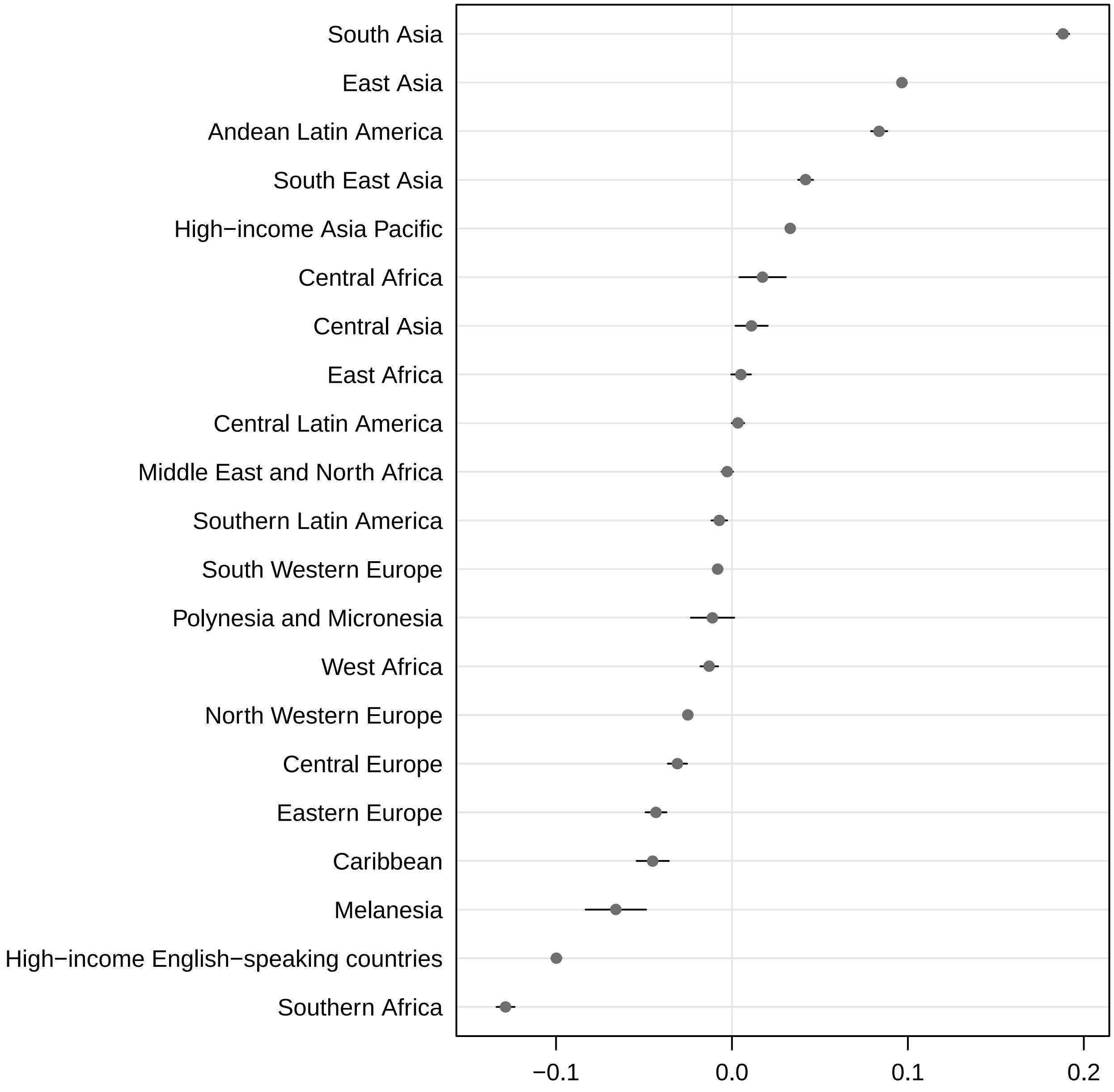
South Asia
East Asia
Andean Latin America
South East Asia
High−income Asia Pacific
Central Africa
Central Asia
East Africa
Central Latin America
Middle East and North Africa
Southern Latin America
South Western Europe
Polynesia and Micronesia
West Africa
North Western Europe
Central Europe
Eastern Europe
Caribbean
Melanesia
High−income English−speaking countries
Southern Africa
−0.1
0.0
0.1
0.2

30 ≤ BMI <35 kg/m^2	
**Variable**	**Coefficient (95% CI)**
Intercept	-0.74 (-0.78, -0.70)
Inverse (mean BMI – 32.5)	-97.2 (-98.2, -96.2)
Mean age of age group	0.0021 (0.0019, 0.0023)
Male sex	0.158 (0.152, 0.165)
Study mid-year (per one more recent year since 1975)	0.0028 (0.0026, 0.0030)
Natural logarithm of per-capita gross domestic product (GDP)	0.022 (0.019, 0.025)
[Inverse (mean BMI – 32.5)] * mean age of age group	-0.20 (-0.22, -0.18)
[Inverse (mean BMI – 32.5)] * male sex	-25.7 (-26.3, -25.1)

Traditional R^2 is not clearly defined for mixed-effect models. The pseudo-R^2 for the model, which describes the proportion of variance explained by both fixed and random factors, was 0.91.

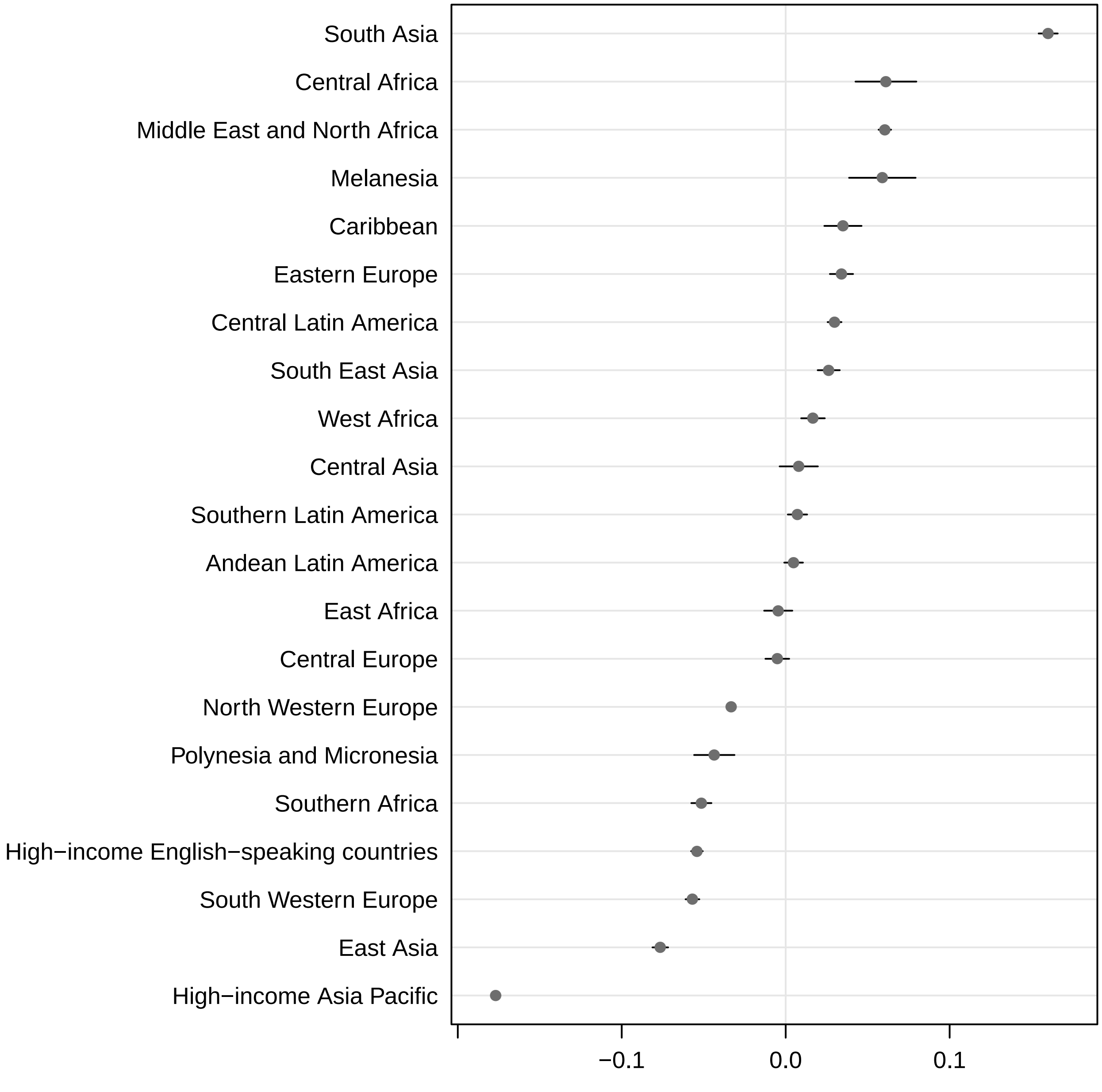
South Asia
Central Africa
Middle East and North Africa
Melanesia
Caribbean
Eastern Europe
Central Latin America
South East Asia
West Africa
Central Asia
Southern Latin America
Andean Latin America
East Africa
Central Europe
North Western Europe
Polynesia and Micronesia
Southern Africa
High−income English−speaking countries
South Western Europe
East Asia
High−income Asia Pacific
−0.1
0.0
0.1

**35 ≤ BMI <40 kg/m²**	
**Variable**	**Coefficient (95% CI)**
Intercept	-1.07 (-1.13, -1.00)
Inverse (mean BMI – 37.5)	-85.4 (-87.0, -83.7)
Mean age of age group	0.0047 (0.0042, 0.0051)
Male sex	-0.167 (-0.181, -0.152)
Study mid-year (per one more recent year since 1975)	0.0051 (0.0048, 0.0054)
Natural logarithm of per-capita gross domestic product (GDP)	0.022 (0.017, 0.027)
[Inverse (mean BMI – 37.5)] * mean age of age group	-0.61 (-0.64, -0.57)
[Inverse (mean BMI – 37.5)] * male sex	-4.89 (-6.01, -3.77)

Traditional R^2 is not clearly defined for mixed-effect models. The pseudo-R^2 for the model, which describes the proportion of variance explained by both fixed and random factors, was 0.86.

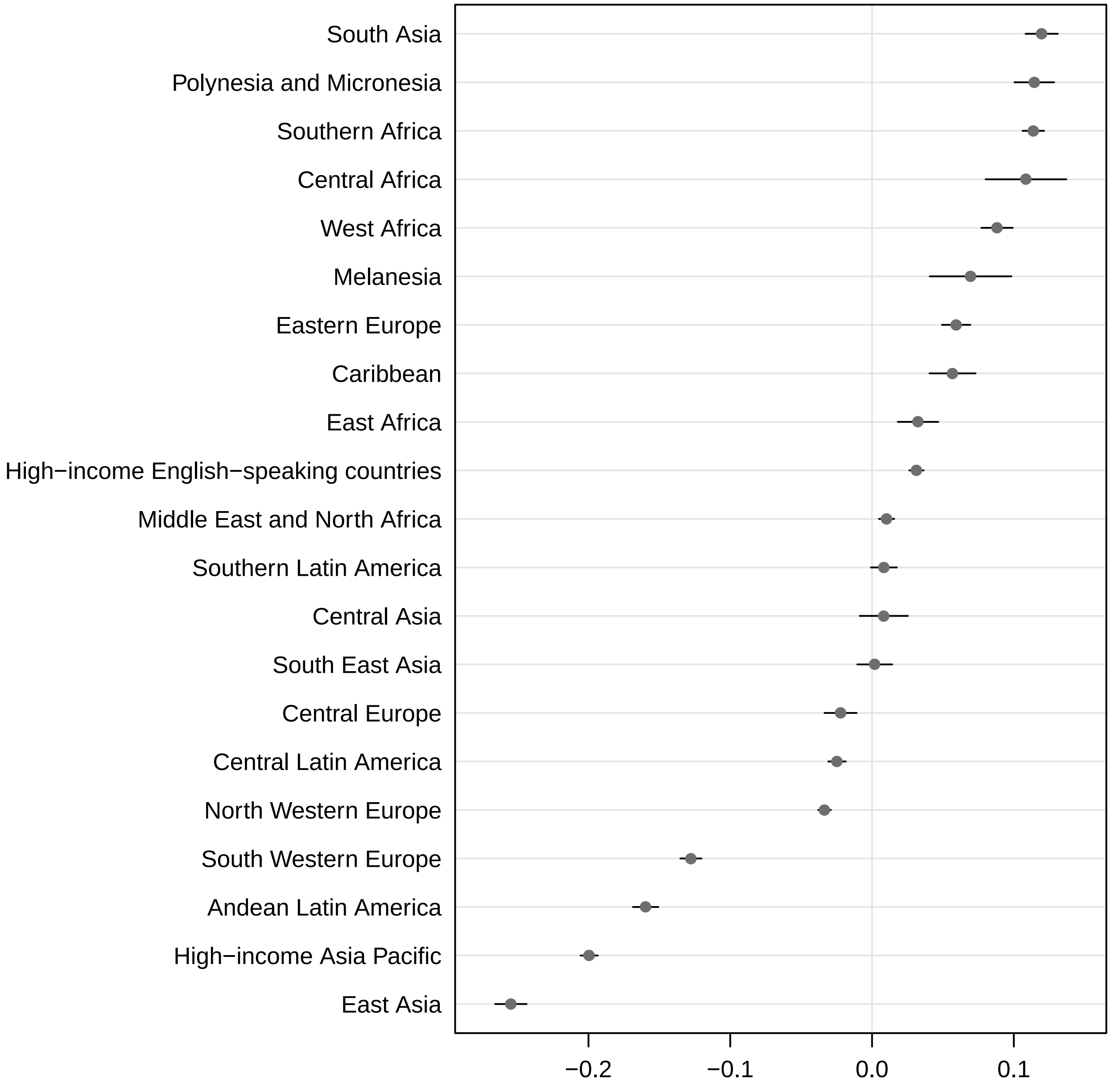
South Asia
Polynesia and Micronesia
Southern Africa
Central Africa
West Africa
Melanesia
Eastern Europe
Caribbean
East Africa
High−income English−speaking countries
Middle East and North Africa
Southern Latin America
Central Asia
South East Asia
Central Europe
Central Latin America
North Western Europe
South Western Europe
Andean Latin America
High−income Asia Pacific
East Asia
−0.2
−0.1
0.0
0.1

BMI ≥40 kg/m^2	
**Variable**	**Coefficient (95% CI)**
Intercept	1.16 (1.02, 1.29)
Inverse mean BMI	-97.4 (-99.9, -94.8)
Mean age of age group	0.0068 (0.0047, 0.0090)
Male sex	-0.66 (-0.73, -0.59)
Study mid-year (per one more recent year since 1975)	0.0060 (0.0056, 0.0064)
Natural logarithm of per-capita gross domestic product (GDP)	0.042 (0.036, 0.048)
Inverse mean BMI * mean age of age group	-0.295 (-0.352, -0.238)
Inverse mean BMI * male sex	9.74 (7.98, 11.50)

Traditional R^2 is not clearly defined for mixed-effect models. The pseudo-R^2 for the model, which describes the proportion of variance explained by both fixed and random factors, was 0.81.

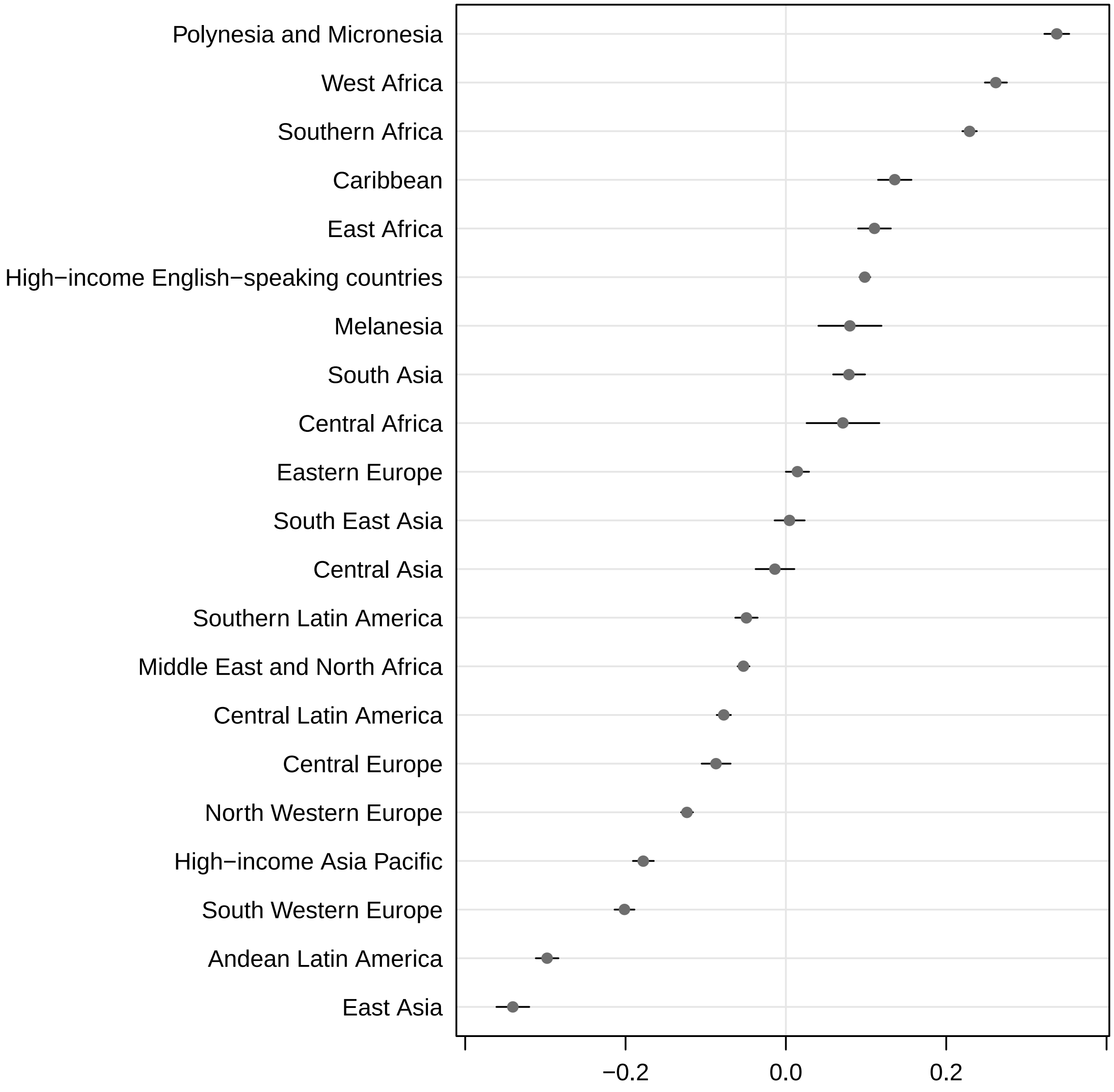
Polynesia and Micronesia
West Africa
Southern Africa
Caribbean
East Africa
High−income English−speaking countries
Melanesia
South Asia
Central Africa
Eastern Europe
South East Asia
Central Asia
Southern Latin America
Middle East and North Africa
Central Latin America
Central Europe
North Western Europe
High−income Asia Pacific
South Western Europe
Andean Latin America
East Asia
−0.2
0.0
0.2

Appendix Table 4: Age-standardised mean BMI by sex and country in 1975, 1985, 1995, 2005, and 2014. Numbers in brackets show 95% credible intervals. Estimates for all countries and years can be downloaded from www.ncdrisc.org.

Men

Country	Year				
	1975	1985	1995	2005	2014
Afghanistan	19.5 (17.0-21.8)	20.1 (18.1-22.2)	20.6 (18.7-22.6)	21.0 (19.1-23.0)	21.6 (19.4-23.8)
Albania	23.7 (21.7-25.7)	24.4 (23.1-25.8)	24.9 (23.9-25.9)	25.8 (25.1-26.5)	26.5 (25.5-27.6)
Algeria	21.4 (19.3-23.5)	22.4 (21.2-23.7)	23.2 (22.4-24.2)	23.8 (23.2-24.5)	24.3 (23.0-25.5)
American Samoa	29.0 (28.1-30.0)	31.6 (31.0-32.3)	33.2 (32.5-33.8)	32.9 (32.2-33.6)	32.2 (30.5-33.7)
Andorra	25.2 (22.7-27.6)	25.9 (23.9-27.9)	26.4 (24.4-28.3)	27.2 (25.2-29.1)	27.7 (25.4-29.9)
Angola	19.9 (17.2-22.7)	20.7 (18.4-22.9)	21.2 (19.1-23.3)	21.8 (19.8-23.9)	22.8 (20.4-25.1)
Antigua and Barbuda	23.0 (20.4-25.5)	23.9 (21.9-25.9)	25.0 (23.1-27.0)	26.1 (24.2-28.0)	27.2 (25.0-29.5)
Argentina	24.7 (23.0-26.4)	25.4 (24.5-26.2)	26.0 (25.2-26.7)	26.7 (26.1-27.4)	27.6 (26.5-28.6)
Armenia	23.0 (20.8-25.3)	23.7 (22.3-25.1)	24.0 (22.9-25.1)	24.8 (24.0-25.6)	25.9 (24.5-27.3)
Australia	24.6 (23.9-25.4)	25.2 (24.9-25.6)	26.1 (25.8-26.4)	27.0 (26.7-27.3)	27.5 (26.9-28.1)
Austria	24.1 (22.9-25.4)	24.7 (24.0-25.5)	25.4 (24.7-26.1)	26.1 (25.2-26.9)	26.6 (25.1-28.1)
Azerbaijan	23.2 (21.1-25.3)	24.1 (23.0-25.3)	24.6 (23.8-25.4)	25.1 (24.4-25.7)	26.4 (25.1-27.6)
Bahamas	23.9 (21.5-26.3)	24.7 (22.7-26.7)	25.6 (23.6-27.5)	26.5 (24.6-28.4)	27.2 (25.0-29.4)
Bahrain	23.1 (20.9-25.3)	24.1 (23.1-25.3)	25.1 (24.4-25.8)	25.3 (24.7-25.9)	24.9 (23.6-26.2)
Bangladesh	18.9 (17.1-20.7)	19.6 (18.7-20.7)	20.2 (19.6-20.8)	20.6 (20.2-21.1)	20.8 (20.0-21.7)
Barbados	23.2 (21.3-25.1)	24.1 (23.1-25.0)	24.8 (24.1-25.5)	25.7 (24.8-26.6)	26.7 (25.0-28.3)
Belarus	24.0 (21.5-26.5)	24.7 (22.6-26.7)	25.1 (23.1-27.1)	25.8 (23.9-27.8)	26.7 (24.6-28.9)
Belgium	24.4 (23.2-25.7)	24.9 (24.5-25.4)	25.5 (25.1-25.9)	26.2 (25.8-26.7)	26.9 (25.9-27.9)
Belize	23.6 (21.4-25.7)	24.4 (23.0-25.8)	25.3 (24.2-26.4)	26.4 (25.5-27.3)	27.3 (25.9-28.7)
Benin	20.2 (18.0-22.3)	20.8 (19.4-22.1)	21.3 (20.2-22.4)	22.0 (21.3-22.7)	22.5 (21.5-23.6)
Bermuda	24.7 (22.1-27.2)	25.4 (23.3-27.5)	26.1 (24.1-28.1)	26.9 (25.0-28.9)	28.0 (25.7-30.2)
Bhutan	19.6 (17.6-21.5)	20.5 (19.0-22.0)	21.6 (20.2-22.8)	22.5 (21.6-23.5)	23.3 (22.5-24.0)
Bolivia	21.8 (19.5-24.1)	22.5 (21.0-24.2)	23.2 (21.9-24.6)	24.1 (23.0-25.2)	25.0 (23.5-26.6)
Bosnia and Herzegovina	22.4 (20.2-24.6)	23.2 (21.7-24.6)	23.8 (22.6-25.0)	25.3 (24.4-26.1)	25.9 (24.9-26.8)
Botswana	19.1 (16.9-21.3)	20.2 (18.8-21.7)	21.3 (20.2-22.5)	22.0 (21.2-22.8)	22.7 (21.4-23.9)
Brazil	22.2 (20.5-24.0)	23.3 (22.6-24.0)	24.1 (23.7-24.5)	24.9 (24.6-25.2)	25.6 (24.9-26.3)
Brunei Darussalam	22.7 (19.9-25.6)	23.5 (21.3-25.7)	24.1 (22.1-26.1)	24.9 (22.8-26.8)	25.5 (23.2-27.8)
Bulgaria	24.1 (22.1-26.0)	25.0 (23.8-26.3)	25.6 (24.6-26.6)	26.0 (25.2-26.8)	26.6 (25.3-28.0)
Burkina Faso	19.6 (17.3-21.8)	20.1 (18.6-21.6)	20.6 (19.4-21.9)	21.4 (20.5-22.3)	22.1 (21.3-22.9)
Burundi	18.5 (15.8-21.3)	19.3 (17.1-21.6)	20.1 (18.0-22.1)	20.4 (18.4-22.3)	20.8 (18.5-22.9)
Cabo Verde	20.2 (17.9-22.3)	21.0 (19.5-22.3)	21.8 (20.7-23.0)	23.1 (22.3-23.9)	24.0 (22.7-25.2)
Cambodia	19.0 (16.8-21.1)	19.5 (18.0-20.9)	20.1 (18.8-21.3)	20.9 (20.0-21.7)	21.8 (20.8-22.8)
Cameroon	20.7 (18.6-22.8)	21.6 (20.3-22.8)	22.1 (21.3-23.0)	22.9 (22.1-23.6)	23.5 (22.1-24.8)
Canada	24.6 (23.5-25.7)	25.5 (25.0-25.9)	26.4 (25.9-26.8)	27.1 (26.7-27.6)	27.4 (26.5-28.3)
Central African Republic	19.8 (17.3-22.3)	20.2 (18.3-22.1)	20.6 (18.9-22.3)	21.0 (19.6-22.4)	21.5 (19.9-23.0)
Chad	19.6 (17.3-21.9)	20.0 (18.3-21.6)	20.5 (19.0-21.9)	21.2 (20.0-22.5)	22.0 (20.4-23.5)
Chile	24.2 (22.3-26.2)	24.9 (23.9-25.9)	25.8 (25.2-26.4)	26.8 (26.3-27.2)	27.7 (26.9-28.4)
China	21.0 (19.9-22.1)	21.5 (21.2-21.8)	22.1 (21.9-22.3)	23.0 (22.7-23.2)	24.3 (23.7-24.8)
China (Hong Kong SAR)	22.1 (20.3-24.0)	22.8 (21.9-23.8)	23.6 (22.9-24.3)	24.2 (23.2-25.3)	25.1 (23.3-27.0)
Colombia	21.9 (19.9-23.7)	22.7 (21.7-23.7)	23.6 (22.8-24.4)	24.6 (24.2-25.0)	25.5 (24.7-26.4)
Comoros	20.8 (18.4-22.9)	21.4 (19.8-22.9)	22.0 (20.7-23.3)	22.6 (21.6-23.5)	23.1 (22.2-24.0)
Congo	19.7 (17.7-21.8)	20.6 (19.4-21.9)	21.2 (20.3-22.1)	21.7 (20.6-22.7)	22.2 (20.5-24.0)
Cook Islands	27.5 (25.6-29.5)	29.5 (28.2-30.8)	31.0 (29.9-32.0)	31.4 (30.5-32.3)	31.5 (29.8-33.0)
Costa Rica	22.9 (20.7-25.1)	24.0 (22.6-25.5)	25.0 (23.9-26.1)	25.9 (25.2-26.6)	26.5 (25.7-27.4)
Cote d'Ivoire	21.0 (18.9-23.1)	21.6 (20.3-22.8)	22.0 (21.1-23.0)	22.7 (22.1-23.3)	23.3 (22.0-24.5)
Croatia	23.6 (21.6-25.6)	24.6 (23.4-25.7)	25.2 (24.4-26.1)	26.2 (25.3-27.0)	27.0 (25.5-28.5)
Cuba	22.1 (20.5-23.7)	23.1 (22.2-24.0)	23.7 (22.9-24.5)	24.3 (23.8-24.9)	25.3 (24.3-26.1)
Cyprus	23.9 (21.9-25.9)	25.1 (23.9-26.3)	26.2 (25.3-27.1)	27.2 (26.3-28.1)	27.8 (26.3-29.4)
Czech Republic	25.6 (24.1-27.0)	26.4 (25.8-26.9)	26.7 (26.3-27.1)	27.1 (26.7-27.6)	27.6 (26.5-28.7)
Denmark	23.2 (22.7-23.6)	24.1 (23.7-24.4)	24.9 (24.4-25.4)	25.6 (25.1-26.1)	26.0 (25.0-26.9)
Djibouti	21.1 (18.6-23.7)	21.5 (19.4-23.7)	22.0 (20.0-24.0)	22.2 (20.2-24.1)	22.4 (20.2-24.6)
Dominica	21.4 (19.3-23.7)	22.2 (20.8-23.7)	23.2 (22.0-24.4)	24.1 (23.2-24.9)	24.9 (23.7-26.2)
Dominican Republic	21.7 (19.7-23.7)	22.8 (21.9-23.8)	23.7 (23.2-24.3)	24.8 (24.1-25.4)	25.4 (24.6-26.2)
DR Congo	19.3 (17.0-21.7)	19.9 (18.2-21.6)	20.2 (18.7-21.7)	20.3 (19.0-21.6)	20.8 (19.1-22.6)
Ecuador	22.4 (20.2-24.6)	23.4 (21.8-24.9)	24.2 (23.0-25.5)	25.2 (24.4-26.0)	26.4 (25.6-27.2)
Egypt	24.1 (22.1-26.0)	25.4 (24.5-26.3)	26.3 (25.8-26.9)	26.9 (26.5-27.2)	27.3 (26.5-28.1)

El Salvador	23.1 (20.8-25.4)	23.9 (22.3-25.6)	24.8 (23.4-26.2)	25.8 (24.6-27.1)	26.5 (24.8-28.2)
Equatorial Guinea	19.7 (16.9-22.5)	20.4 (18.2-22.7)	21.0 (18.9-23.2)	23.1 (21.0-25.2)	24.2 (21.7-26.6)
Eritrea	18.7 (16.6-20.9)	19.4 (18.1-20.7)	20.0 (19.0-21.0)	20.3 (19.7-20.9)	20.2 (19.3-21.1)
Estonia	24.2 (22.4-25.9)	24.7 (23.8-25.5)	25.1 (24.6-25.6)	26.1 (25.8-26.4)	26.8 (26.2-27.3)
Ethiopia	18.6 (16.4-20.8)	19.2 (17.8-20.7)	19.6 (18.4-20.8)	19.8 (19.0-20.6)	20.1 (19.2-21.0)
Fiji	22.8 (20.7-24.8)	23.9 (22.7-25.2)	24.8 (23.9-25.7)	25.7 (25.1-26.2)	26.2 (25.4-27.0)
Finland	24.4 (23.3-25.5)	25.2 (24.8-25.6)	25.9 (25.5-26.2)	26.3 (26.1-26.6)	26.4 (25.9-26.9)
France	24.3 (22.7-25.9)	24.8 (24.1-25.4)	25.1 (24.6-25.6)	25.6 (25.1-26.1)	26.1 (25.2-27.0)
French Polynesia	26.5 (24.5-28.7)	28.3 (26.8-29.9)	29.5 (28.2-30.9)	29.7 (28.8-30.6)	29.5 (28.5-30.5)
Gabon	20.8 (18.3-23.3)	22.1 (20.3-24.0)	22.9 (21.3-24.6)	23.6 (22.2-24.9)	24.1 (22.5-25.7)
Gambia	19.9 (17.6-22.1)	20.2 (19.0-21.4)	20.8 (20.0-21.5)	22.0 (21.3-22.6)	23.1 (22.1-24.2)
Georgia	23.9 (21.7-26.1)	24.9 (23.3-26.4)	25.2 (23.9-26.5)	26.0 (25.1-26.8)	27.0 (26.0-28.0)
Germany	24.3 (23.3-25.4)	25.1 (24.7-25.4)	25.8 (25.4-26.1)	26.5 (26.1-26.8)	27.1 (26.2-27.9)
Ghana	20.6 (18.5-22.7)	21.1 (19.9-22.3)	21.7 (20.9-22.5)	22.5 (21.9-23.1)	23.3 (22.2-24.5)
Greece	24.9 (23.2-26.7)	25.7 (24.9-26.6)	26.4 (25.8-27.0)	27.0 (26.3-27.7)	27.4 (26.1-28.7)
Greenland	23.3 (21.6-25.1)	24.2 (23.0-25.5)	24.9 (23.8-26.0)	25.6 (24.8-26.4)	26.1 (25.0-27.2)
Grenada	21.4 (19.2-23.6)	22.3 (20.8-23.9)	23.3 (21.9-24.6)	24.3 (23.3-25.3)	25.2 (24.2-26.1)
Guatemala	22.2 (20.1-24.3)	23.1 (21.8-24.3)	23.8 (23.0-24.7)	24.8 (24.1-25.5)	25.5 (24.1-26.9)
Guinea	19.9 (17.6-22.2)	20.6 (19.0-22.2)	21.1 (19.6-22.5)	21.7 (20.6-22.9)	22.2 (20.8-23.6)
Guinea Bissau	20.1 (17.4-22.6)	20.6 (18.6-22.7)	21.3 (19.3-23.2)	21.8 (20.0-23.7)	22.3 (20.1-24.4)
Guyana	21.6 (19.6-23.8)	22.4 (21.0-23.9)	23.1 (21.8-24.3)	24.1 (23.3-25.0)	25.0 (23.9-26.1)
Haiti	21.0 (18.4-23.5)	21.7 (19.6-23.7)	22.3 (20.3-24.3)	23.2 (21.4-25.2)	24.0 (21.8-26.3)
Honduras	22.0 (19.8-24.2)	22.9 (21.4-24.5)	23.8 (22.5-25.1)	24.7 (23.5-25.9)	25.5 (23.8-27.1)
Hungary	24.5 (23.0-26.0)	25.4 (24.9-26.0)	26.1 (25.4-26.8)	26.8 (26.0-27.7)	27.4 (26.0-28.9)
Iceland	24.1 (22.7-25.4)	25.0 (24.3-25.6)	25.6 (24.9-26.3)	26.3 (25.3-27.3)	26.8 (25.2-28.4)
India	18.6 (17.8-19.4)	19.6 (19.2-20.1)	20.3 (20.0-20.6)	20.9 (20.7-21.2)	21.2 (20.5-21.8)
Indonesia	19.3 (17.6-21.1)	20.0 (19.2-20.8)	20.7 (20.3-21.1)	21.5 (21.1-21.8)	22.3 (21.7-23.0)
Iran	21.9 (20.0-23.8)	22.9 (21.9-23.8)	23.7 (23.2-24.2)	24.6 (24.4-24.9)	25.3 (24.6-25.9)
Iraq	24.0 (21.9-25.9)	24.9 (23.6-26.2)	25.5 (24.5-26.6)	26.3 (25.5-27.0)	26.9 (25.7-28.1)
Ireland	24.0 (22.4-25.8)	25.0 (24.0-26.0)	26.1 (25.5-26.7)	27.3 (26.9-27.7)	27.8 (27.0-28.6)
Israel	24.4 (22.7-26.2)	25.0 (24.1-25.9)	25.6 (25.0-26.2)	26.4 (25.9-26.9)	27.1 (25.9-28.3)
Italy	24.4 (23.1-25.7)	24.9 (24.5-25.4)	25.5 (25.2-25.8)	26.4 (26.1-26.7)	27.0 (26.2-27.7)
Jamaica	21.8 (19.9-23.7)	22.4 (21.5-23.4)	23.3 (22.7-23.9)	24.5 (24.0-25.0)	25.6 (24.5-26.7)
Japan	22.0 (21.6-22.4)	22.3 (22.1-22.6)	22.8 (22.6-23.0)	23.3 (23.0-23.5)	23.6 (23.0-24.1)
Jordan	23.7 (21.7-25.7)	25.1 (23.9-26.1)	26.1 (25.3-26.8)	27.0 (26.6-27.5)	27.9 (27.0-28.9)
Kazakhstan	23.4 (21.5-25.4)	24.4 (23.1-25.6)	25.1 (23.8-26.4)	26.0 (24.5-27.4)	27.1 (25.2-29.0)
Kenya	19.5 (17.5-21.4)	20.4 (19.1-21.7)	21.2 (19.9-22.5)	21.8 (20.3-23.2)	22.3 (20.5-24.1)
Kiribati	24.8 (23.5-26.2)	26.6 (25.8-27.4)	27.8 (27.0-28.7)	28.3 (27.5-29.1)	28.2 (26.8-29.7)
Kuwait	25.1 (23.4-26.8)	26.1 (25.3-26.9)	27.0 (26.4-27.5)	27.9 (27.5-28.4)	28.2 (27.3-29.1)
Kyrgyzstan	22.6 (20.6-24.7)	23.4 (22.3-24.4)	24.1 (23.2-24.9)	24.7 (23.9-25.5)	25.6 (24.8-26.5)
Lao PDR	19.4 (17.2-21.5)	19.8 (18.3-21.2)	20.2 (18.9-21.5)	21.2 (20.3-22.1)	22.3 (21.5-23.1)
Latvia	24.2 (22.3-26.1)	24.8 (23.7-26.0)	25.1 (24.3-25.9)	25.8 (25.1-26.5)	26.8 (25.6-28.1)
Lebanon	23.8 (21.8-25.8)	24.8 (23.7-25.9)	25.8 (25.1-26.5)	26.8 (26.2-27.4)	27.6 (26.5-28.6)
Lesotho	19.8 (17.5-22.0)	20.5 (19.0-22.0)	21.2 (19.9-22.5)	21.9 (21.1-22.8)	22.7 (22.0-23.5)
Liberia	21.6 (19.4-23.8)	22.2 (20.7-23.8)	22.0 (20.6-23.5)	22.7 (21.8-23.6)	23.2 (22.5-23.9)
Libya	23.6 (21.4-25.7)	24.9 (23.6-26.2)	25.6 (24.6-26.6)	26.1 (25.4-26.9)	26.4 (25.3-27.4)
Lithuania	25.0 (23.6-26.4)	25.5 (24.8-26.2)	25.7 (25.1-26.2)	26.4 (25.7-27.0)	27.4 (26.0-28.8)
Luxembourg	24.4 (22.7-26.3)	25.3 (24.0-26.6)	26.2 (25.1-27.4)	27.0 (26.2-27.8)	27.5 (26.4-28.6)
Macedonia (TFYR)	23.8 (21.2-26.1)	24.6 (22.5-26.6)	25.1 (23.2-27.0)	25.7 (23.8-27.6)	26.3 (24.2-28.5)
Madagascar	19.5 (17.4-21.7)	20.2 (18.8-21.5)	20.7 (19.6-21.7)	20.9 (19.9-22.0)	21.2 (19.6-22.7)
Malawi	19.9 (17.7-22.0)	20.6 (19.2-21.9)	21.2 (20.1-22.2)	21.8 (21.1-22.5)	22.3 (21.3-23.4)
Malaysia	21.1 (19.1-23.1)	22.0 (20.9-23.0)	22.9 (22.3-23.5)	24.1 (23.8-24.4)	25.1 (24.4-25.9)
Maldives	20.1 (17.8-22.1)	20.8 (19.4-22.1)	21.6 (20.4-22.7)	22.8 (22.0-23.6)	24.0 (23.2-24.9)
Mali	20.2 (18.0-22.5)	20.7 (19.3-22.1)	21.2 (20.1-22.3)	22.0 (21.0-23.1)	22.8 (21.3-24.3)
Malta	25.3 (23.5-27.0)	26.0 (24.9-27.1)	26.5 (25.4-27.7)	27.2 (25.9-28.6)	27.8 (25.9-29.5)
Marshall Islands	25.4 (23.5-27.4)	27.0 (25.8-28.3)	28.2 (27.2-29.1)	28.2 (27.4-29.1)	28.1 (26.5-29.6)
Mauritania	20.6 (18.3-22.9)	21.3 (19.7-22.9)	21.9 (20.5-23.2)	22.5 (21.3-23.7)	23.1 (21.5-24.7)
Mauritius	21.4 (19.7-23.0)	22.4 (21.8-23.1)	23.5 (23.0-24.0)	24.3 (23.8-24.8)	24.8 (23.8-25.8)
Mexico	23.8 (21.8-25.6)	24.9 (23.9-25.8)	25.8 (25.4-26.3)	26.8 (26.5-27.0)	27.5 (26.9-28.0)

Micronesia (Federated States of)	24.6 (22.8-26.6)	26.3 (25.0-27.5)	27.3 (26.3-28.4)	27.6 (26.9-28.3)	27.7 (26.6-28.9)
Moldova	23.9 (21.7-26.0)	24.5 (22.9-26.0)	24.8 (23.3-26.2)	25.3 (24.2-26.3)	26.3 (25.5-27.2)
Mongolia	22.2 (20.1-24.3)	23.0 (21.8-24.2)	23.6 (22.8-24.3)	24.2 (23.8-24.7)	25.3 (24.7-25.9)
Montenegro	23.7 (21.2-26.2)	24.8 (22.8-26.8)	25.4 (23.5-27.3)	26.1 (24.2-27.9)	26.7 (24.6-28.8)
Morocco	21.8 (19.7-23.8)	22.7 (21.5-23.9)	23.5 (22.7-24.4)	24.3 (23.4-25.1)	24.8 (23.3-26.4)
Mozambique	19.4 (17.1-21.5)	20.0 (18.6-21.4)	20.6 (19.5-21.6)	21.1 (20.4-21.9)	21.5 (20.2-22.9)
Myanmar	19.3 (17.1-21.5)	19.7 (18.3-21.1)	20.1 (19.0-21.3)	21.0 (20.3-21.7)	22.0 (21.3-22.8)
Namibia	20.2 (17.9-22.3)	20.8 (19.5-22.3)	21.4 (20.3-22.4)	22.1 (21.4-22.8)	22.8 (21.5-24.0)
Nauru	30.7 (29.6-31.8)	32.6 (32.1-33.2)	33.4 (32.8-34.0)	32.5 (31.9-33.1)	31.6 (30.2-33.1)
Nepal	19.7 (17.8-21.5)	20.4 (19.2-21.6)	21.2 (20.3-22.1)	22.0 (21.4-22.7)	22.7 (21.9-23.4)
Netherlands	23.2 (21.8-24.6)	23.9 (23.2-24.6)	24.7 (24.2-25.2)	25.5 (25.2-25.9)	25.9 (25.1-26.6)
New Zealand	24.5 (23.1-25.8)	25.3 (24.7-25.9)	26.3 (25.8-26.7)	27.3 (27.0-27.7)	28.0 (27.5-28.4)
Nicaragua	23.2 (20.9-25.3)	23.9 (22.3-25.5)	24.4 (23.0-25.8)	25.3 (24.0-26.5)	25.9 (24.3-27.6)
Niger	19.7 (17.4-21.8)	20.0 (18.5-21.4)	20.4 (19.3-21.5)	20.9 (20.1-21.7)	21.3 (20.0-22.5)
Nigeria	20.1 (18.2-21.9)	20.8 (20.1-21.6)	21.3 (20.8-21.9)	22.0 (21.5-22.5)	22.7 (21.9-23.6)
Niue	26.8 (24.8-28.8)	28.9 (27.3-30.4)	30.3 (28.9-31.7)	30.9 (29.9-31.9)	31.2 (30.2-32.3)
North Korea	21.1 (18.5-23.7)	21.6 (19.6-23.7)	21.9 (19.8-23.9)	21.8 (19.7-24.0)	22.3 (19.7-24.8)
Norway	23.4 (22.1-24.7)	24.5 (23.8-25.1)	25.4 (24.8-25.9)	26.4 (25.8-26.9)	27.0 (25.9-28.2)
Occupied Palestinian Territory	24.3 (22.2-26.2)	25.1 (24.0-26.2)	25.9 (25.2-26.6)	26.9 (26.4-27.5)	27.5 (26.5-28.6)
Oman	22.0 (20.0-23.8)	23.4 (22.5-24.2)	24.6 (24.0-25.1)	25.4 (24.9-25.9)	26.0 (24.9-27.0)
Pakistan	20.0 (18.3-21.7)	20.8 (19.9-21.7)	21.6 (21.1-22.2)	22.6 (22.0-23.2)	23.4 (22.2-24.7)
Palau	27.0 (25.0-29.3)	28.7 (27.1-30.3)	29.7 (28.2-31.3)	29.8 (28.7-30.9)	29.4 (28.5-30.3)
Panama	22.8 (20.7-25.0)	23.8 (22.4-25.1)	24.6 (23.6-25.6)	25.5 (24.7-26.3)	26.4 (25.2-27.5)
Papua New Guinea	21.7 (19.7-23.6)	22.7 (21.6-23.9)	23.8 (22.8-24.8)	24.4 (23.6-25.1)	24.9 (23.6-26.1)
Paraguay	22.4 (19.9-25.0)	23.3 (21.3-25.4)	24.3 (22.3-26.3)	25.2 (23.2-27.2)	26.0 (23.8-28.2)
Peru	22.5 (20.3-24.6)	23.2 (21.8-24.6)	23.8 (22.9-24.8)	24.8 (24.3-25.3)	25.9 (25.1-26.8)
Philippines	20.8 (19.0-22.7)	21.3 (20.5-22.2)	21.7 (21.2-22.2)	22.3 (21.9-22.8)	23.1 (22.0-24.2)
Poland	24.4 (22.9-25.8)	25.0 (24.5-25.6)	25.4 (24.9-25.8)	26.1 (25.8-26.4)	26.9 (26.3-27.7)
Portugal	23.6 (22.2-25.0)	24.3 (23.8-24.8)	25.1 (24.7-25.5)	26.1 (25.7-26.5)	26.8 (25.8-27.8)
Puerto Rico	24.2 (22.2-26.3)	25.4 (24.0-26.8)	26.5 (25.4-27.6)	27.5 (26.6-28.4)	28.2 (26.9-29.6)
Qatar	24.4 (22.3-26.4)	26.0 (24.6-27.3)	26.7 (25.7-27.8)	27.8 (27.1-28.4)	28.5 (27.7-29.3)
Romania	23.3 (21.6-25.1)	24.2 (23.3-25.1)	24.6 (23.9-25.3)	25.3 (24.5-26.0)	26.2 (24.8-27.6)
Russian Federation	24.0 (22.5-25.5)	24.5 (24.0-25.0)	24.7 (24.5-25.0)	25.3 (25.0-25.6)	26.2 (25.2-27.2)
Rwanda	18.7 (16.4-20.9)	19.5 (17.8-21.1)	20.1 (18.8-21.4)	20.7 (19.9-21.5)	21.3 (20.6-22.0)
Saint Kitts and Nevis	23.5 (21.4-25.6)	24.6 (23.0-26.1)	25.7 (24.4-27.1)	27.1 (25.9-28.3)	28.2 (26.6-29.8)
Saint Lucia	22.7 (21.3-24.1)	23.5 (22.7-24.3)	24.9 (23.8-25.8)	26.9 (26.0-27.7)	29.1 (28.1-30.0)
Saint Vincent and the Grenadines	22.0 (19.5-24.5)	22.9 (20.9-24.8)	23.9 (22.0-25.9)	25.1 (23.3-27.0)	26.2 (24.1-28.3)
Samoa	25.5 (24.2-26.7)	27.2 (26.6-27.8)	28.6 (28.1-29.2)	29.5 (29.0-30.0)	30.1 (29.4-30.8)
Sao Tome and Principe	21.4 (19.2-23.5)	21.9 (20.4-23.3)	22.4 (21.3-23.6)	23.2 (22.5-23.9)	23.9 (23.0-24.8)
Saudi Arabia	23.9 (22.2-25.6)	25.4 (24.8-26.0)	26.4 (26.1-26.7)	27.1 (26.7-27.4)	27.5 (26.9-28.2)
Senegal	19.9 (18.0-21.7)	20.4 (19.2-21.5)	20.8 (19.7-21.8)	21.4 (20.5-22.2)	21.8 (20.9-22.7)
Serbia	23.8 (22.2-25.4)	24.8 (24.0-25.6)	25.4 (24.8-26.0)	25.9 (25.4-26.3)	26.3 (25.8-26.8)
Seychelles	21.5 (19.7-23.3)	22.6 (21.8-23.4)	23.7 (23.2-24.2)	24.7 (24.2-25.2)	25.5 (24.7-26.2)
Sierra Leone	20.4 (18.2-22.5)	20.9 (19.4-22.4)	21.2 (20.0-22.4)	21.5 (20.7-22.4)	22.0 (21.3-22.7)
Singapore	21.9 (20.6-23.3)	22.8 (22.2-23.3)	23.4 (23.0-23.8)	23.8 (23.4-24.2)	24.2 (23.6-24.9)
Slovakia	24.1 (22.4-25.9)	25.1 (24.3-26.0)	25.8 (25.3-26.4)	26.4 (26.0-26.8)	27.1 (26.4-27.9)
Slovenia	23.5 (21.3-25.7)	24.5 (22.9-26.1)	25.3 (23.8-26.8)	26.1 (24.7-27.4)	26.6 (25.2-28.1)
Solomon Islands	21.7 (19.5-24.0)	23.0 (21.5-24.6)	24.0 (22.8-25.3)	24.2 (23.3-25.1)	24.2 (23.0-25.3)
Somalia	20.0 (17.5-22.5)	20.5 (18.5-22.6)	20.9 (19.0-22.9)	21.1 (19.2-23.1)	21.3 (19.0-23.5)
South Africa	22.4 (20.5-24.3)	23.2 (22.2-24.1)	23.8 (23.2-24.3)	24.4 (24.1-24.7)	24.8 (24.3-25.4)
South Korea	20.4 (18.7-22.0)	21.8 (21.1-22.5)	23.0 (22.5-23.4)	23.7 (23.4-24.0)	24.2 (23.7-24.7)
Spain	24.3 (22.7-25.8)	25.1 (24.6-25.7)	26.0 (25.6-26.3)	26.9 (26.6-27.2)	27.5 (26.5-28.4)
Sri Lanka	19.4 (17.4-21.3)	19.9 (18.6-21.0)	20.5 (19.7-21.3)	21.5 (20.9-22.0)	22.6 (21.4-23.8)
Sudan	20.7 (18.5-22.7)	21.5 (20.2-22.8)	22.3 (21.3-23.4)	23.1 (22.3-23.8)	23.6 (22.2-24.9)
Suriname	22.8 (20.3-25.3)	23.8 (21.8-25.7)	24.4 (22.4-26.2)	25.3 (23.4-27.1)	26.2 (24.0-28.4)
Swaziland	20.6 (18.4-22.7)	21.6 (20.1-23.0)	22.5 (21.3-23.6)	23.3 (22.5-24.1)	24.0 (22.7-25.2)
Sweden	23.3 (22.9-23.8)	24.2 (23.9-24.6)	25.1 (24.8-25.4)	26.1 (25.8-26.4)	26.7 (25.8-27.5)
Switzerland	23.9 (22.5-25.5)	24.7 (23.9-25.5)	25.4 (24.8-26.0)	25.9 (25.4-26.4)	26.5 (25.8-27.2)
Syrian Arab Republic	23.3 (21.2-25.3)	24.5 (23.3-25.7)	25.5 (24.6-26.4)	26.3 (25.5-27.1)	26.6 (25.2-28.0)

Taiwan	21.2 (19.4-22.8)	22.2 (21.4-23.0)	23.1 (22.7-23.6)	24.0 (23.6-24.4)	24.9 (23.8-25.9)
Tajikistan	22.9 (20.3-25.4)	23.6 (21.6-25.6)	24.0 (22.1-25.9)	24.3 (22.4-26.2)	25.2 (23.1-27.4)
Tanzania	20.0 (18.0-22.1)	20.9 (19.6-22.0)	21.5 (20.6-22.4)	21.9 (21.3-22.6)	22.3 (21.5-23.0)
Thailand	20.1 (18.3-21.8)	20.9 (20.2-21.6)	21.8 (21.5-22.2)	22.7 (22.3-23.1)	23.5 (22.5-24.4)
Timor-Leste	18.5 (16.3-20.7)	18.8 (17.2-20.5)	19.3 (17.8-20.8)	19.7 (18.4-21.1)	20.3 (18.9-21.8)
Togo	20.3 (18.1-22.4)	20.8 (19.3-22.3)	21.3 (20.0-22.5)	22.0 (21.1-22.8)	22.5 (21.5-23.5)
Tokelau	27.2 (25.1-29.4)	29.2 (27.7-30.7)	30.6 (29.4-31.9)	31.2 (30.3-32.1)	31.5 (30.0-32.9)
Tonga	26.6 (24.8-28.6)	28.5 (27.3-29.8)	29.8 (28.8-30.9)	30.2 (29.6-30.9)	30.2 (29.2-31.1)
Trinidad and Tobago	23.0 (21.0-25.0)	24.0 (22.9-25.2)	24.8 (23.9-25.6)	26.1 (25.1-27.1)	27.4 (25.7-29.2)
Tunisia	21.6 (19.7-23.6)	22.6 (21.6-23.6)	23.5 (23.0-24.1)	24.5 (24.0-25.0)	25.1 (24.0-26.2)
Turkey	22.8 (21.0-24.5)	24.0 (23.2-24.8)	25.3 (24.9-25.7)	26.3 (26.1-26.6)	26.8 (26.3-27.4)
Turkmenistan	23.0 (20.7-25.2)	23.7 (22.1-25.2)	24.5 (23.1-25.9)	25.2 (24.1-26.2)	26.3 (25.5-27.1)
Tuvalu	26.0 (24.7-27.3)	27.7 (26.4-29.0)	29.1 (27.6-30.6)	29.3 (27.8-30.9)	29.2 (27.3-31.1)
Uganda	19.0 (16.9-21.1)	19.5 (18.2-21.0)	20.1 (19.0-21.2)	20.8 (20.1-21.4)	21.4 (20.6-22.2)
Ukraine	24.2 (21.7-26.7)	24.8 (22.9-26.8)	25.1 (23.3-27.1)	25.5 (23.7-27.4)	26.3 (24.0-28.5)
United Arab Emirates	24.7 (22.4-26.8)	25.6 (24.4-26.7)	26.4 (25.7-27.1)	27.3 (26.5-28.1)	27.4 (25.9-28.9)
United Kingdom	24.1 (23.0-25.2)	24.8 (24.5-25.2)	25.8 (25.6-26.0)	26.9 (26.7-27.1)	27.4 (27.0-27.8)
United States of America	25.2 (24.6-25.8)	25.9 (25.5-26.3)	27.0 (26.7-27.4)	28.2 (27.9-28.4)	28.9 (28.4-29.5)
Uruguay	23.9 (21.8-26.0)	24.5 (23.2-25.8)	25.1 (24.1-26.2)	25.9 (25.2-26.7)	26.8 (25.7-28.0)
Uzbekistan	22.7 (20.5-24.8)	23.5 (22.2-24.8)	24.2 (23.2-25.1)	24.8 (24.0-25.6)	25.8 (24.3-27.3)
Vanuatu	22.7 (20.7-24.8)	23.9 (22.7-25.1)	24.9 (24.1-25.6)	25.3 (24.7-25.9)	25.3 (24.4-26.2)
Venezuela	24.1 (22.0-26.2)	25.0 (23.8-26.3)	25.8 (24.9-26.7)	26.5 (25.9-27.2)	27.3 (26.2-28.4)
Viet Nam	18.7 (17.1-20.2)	19.0 (18.5-19.6)	19.5 (19.2-19.9)	20.4 (20.1-20.7)	21.2 (20.5-21.8)
Yemen	20.8 (18.7-22.9)	21.7 (20.4-23.1)	22.5 (21.5-23.6)	23.1 (22.4-23.9)	23.4 (22.2-24.6)
Zambia	19.5 (17.3-21.7)	20.1 (18.7-21.6)	20.5 (19.3-21.7)	20.8 (19.8-21.8)	21.3 (19.9-22.7)
Zimbabwe	20.5 (18.6-22.4)	21.2 (20.3-22.2)	21.6 (20.9-22.4)	21.9 (21.4-22.5)	22.0 (21.1-22.9)

Women

Country	Year				
	1975	1985	1995	2005	2014
Afghanistan	19.4 (16.5-22.2)	20.5 (18.3-22.7)	21.5 (19.8-23.1)	22.5 (21.3-23.7)	23.7 (22.5-24.8)
Albania	24.1 (21.4-26.7)	24.5 (22.7-26.4)	24.8 (23.5-26.0)	25.4 (24.5-26.2)	25.7 (24.6-26.8)
Algeria	23.3 (20.7-25.8)	24.2 (22.5-25.9)	24.9 (23.8-26.0)	25.5 (24.7-26.4)	26.0 (24.7-27.2)
American Samoa	32.2 (31.2-33.1)	33.4 (32.7-34.0)	34.3 (33.7-35.0)	34.7 (33.9-35.6)	34.8 (33.2-36.3)
Andorra	26.2 (22.8-29.7)	26.5 (23.6-29.4)	26.5 (23.8-29.2)	26.7 (23.9-29.4)	26.6 (23.6-29.7)
Angola	19.6 (16.0-23.1)	20.7 (17.6-23.7)	21.8 (18.9-24.6)	22.9 (20.1-25.8)	24.1 (21.0-27.2)
Antigua and Barbuda	24.2 (20.9-27.5)	25.5 (22.5-28.3)	26.9 (24.1-29.6)	28.0 (25.3-30.9)	29.1 (26.1-32.2)
Argentina	23.6 (21.8-25.5)	24.6 (23.5-25.7)	25.5 (24.7-26.3)	26.4 (25.7-27.0)	27.2 (26.2-28.3)
Armenia	24.4 (21.7-27.0)	25.3 (23.6-26.9)	25.8 (24.7-26.7)	26.6 (25.6-27.4)	27.4 (25.8-28.9)
Australia	23.4 (22.6-24.2)	24.3 (23.9-24.7)	25.3 (24.9-25.6)	26.2 (25.9-26.6)	26.8 (26.2-27.4)
Austria	23.9 (22.4-25.4)	24.0 (23.0-25.0)	24.1 (23.3-24.9)	24.4 (23.3-25.4)	24.5 (22.8-26.1)
Azerbaijan	24.2 (21.7-26.8)	25.2 (23.5-26.8)	25.8 (24.7-26.8)	26.7 (25.7-27.6)	27.9 (26.5-29.4)
Bahamas	25.6 (22.3-29.0)	26.6 (23.8-29.6)	27.5 (24.8-30.2)	28.3 (25.6-31.1)	28.9 (25.8-31.9)
Bahrain	26.5 (24.1-29.1)	26.2 (24.8-27.7)	26.1 (25.3-27.0)	25.7 (24.9-26.4)	25.1 (23.7-26.4)
Bangladesh	17.2 (15.3-19.1)	18.2 (17.1-19.3)	19.2 (18.7-19.8)	20.4 (20.0-20.7)	21.4 (20.6-22.2)
Barbados	25.5 (23.6-27.5)	26.4 (25.3-27.6)	27.2 (26.3-28.1)	28.1 (27.0-29.3)	29.0 (27.1-30.9)
Belarus	24.6 (21.1-28.1)	25.4 (22.5-28.3)	25.8 (23.1-28.5)	26.2 (23.5-28.9)	26.6 (23.5-29.6)
Belgium	24.9 (23.7-26.2)	25.0 (24.4-25.5)	25.0 (24.6-25.4)	25.1 (24.5-25.8)	25.1 (24.1-26.2)
Belize	25.4 (22.6-28.0)	26.6 (24.7-28.4)	27.8 (26.6-29.1)	29.2 (28.1-30.2)	30.2 (28.8-31.6)
Benin	18.8 (16.5-21.0)	20.3 (18.9-21.6)	21.6 (20.8-22.4)	23.1 (22.5-23.6)	24.2 (23.4-25.0)
Bermuda	26.8 (23.2-30.4)	27.4 (24.3-30.4)	27.9 (25.0-30.8)	28.5 (25.7-31.3)	29.2 (26.2-32.3)
Bhutan	19.0 (16.0-21.8)	20.3 (18.2-22.4)	21.8 (20.3-23.2)	23.1 (22.1-24.1)	24.2 (23.3-25.0)
Bolivia	22.4 (20.1-24.7)	23.7 (22.4-25.1)	25.0 (24.2-25.9)	26.3 (25.5-27.1)	27.4 (26.1-28.7)
Bosnia and Herzegovina	22.3 (19.4-25.1)	22.9 (20.9-24.9)	23.5 (22.1-24.8)	24.5 (23.5-25.6)	24.6 (23.5-25.7)
Botswana	22.2 (19.3-25.1)	23.6 (21.6-25.7)	25.0 (23.7-26.4)	25.8 (24.8-26.8)	26.4 (25.2-27.7)
Brazil	22.7 (21.0-24.3)	23.9 (23.1-24.7)	24.7 (24.3-25.1)	25.4 (25.1-25.7)	26.1 (25.3-26.7)
Brunei Darussalam	24.4 (20.7-28.2)	25.1 (21.9-28.2)	25.6 (22.7-28.5)	26.0 (23.2-28.9)	26.1 (23.0-29.3)
Bulgaria	24.3 (21.7-26.8)	24.8 (23.1-26.5)	25.0 (23.8-26.1)	25.1 (24.1-26.0)	25.2 (23.8-26.7)
Burkina Faso	18.4 (16.5-20.4)	19.4 (18.3-20.5)	20.3 (19.7-20.9)	21.4 (20.9-21.8)	22.0 (21.3-22.7)
Burundi	17.7 (14.7-20.7)	18.8 (16.6-20.9)	19.8 (18.3-21.4)	20.6 (19.5-21.8)	21.4 (20.1-22.6)
Cabo Verde	19.7 (17.0-22.5)	21.3 (19.5-23.2)	22.8 (21.5-24.2)	24.4 (23.4-25.5)	25.3 (24.0-26.6)
Cambodia	17.9 (15.4-20.3)	18.9 (17.4-20.5)	20.1 (19.1-21.0)	21.0 (20.5-21.6)	21.9 (21.0-22.7)
Cameroon	20.3 (18.1-22.5)	21.8 (20.4-23.1)	22.9 (22.2-23.6)	24.1 (23.6-24.6)	25.0 (24.2-25.8)
Canada	23.3 (22.3-24.3)	24.4 (23.8-24.9)	25.4 (25.0-25.9)	26.3 (25.8-26.7)	26.7 (25.8-27.5)
Central African Republic	19.2 (16.9-21.5)	20.2 (18.8-21.6)	21.2 (20.2-22.2)	22.3 (21.2-23.4)	23.3 (21.7-25.0)
Chad	18.3 (16.0-20.5)	19.2 (17.8-20.6)	20.3 (19.3-21.1)	21.5 (20.6-22.3)	22.6 (21.2-23.9)
Chile	24.8 (22.5-27.2)	25.7 (24.2-27.2)	26.6 (25.8-27.5)	27.4 (26.8-27.9)	28.0 (27.1-28.9)
China	21.4 (20.4-22.5)	21.8 (21.4-22.2)	22.2 (22.0-22.5)	22.8 (22.6-23.1)	23.4 (22.8-23.9)
China (Hong Kong SAR)	22.3 (20.1-24.5)	22.7 (21.4-23.9)	22.9 (22.1-23.7)	22.8 (21.5-24.1)	22.8 (20.6-25.0)
Colombia	22.7 (20.7-24.7)	24.0 (22.9-25.2)	25.1 (24.5-25.7)	25.9 (25.5-26.3)	26.4 (25.6-27.2)
Comoros	20.2 (17.6-22.5)	21.3 (19.7-22.7)	22.5 (21.5-23.4)	23.8 (23.1-24.5)	25.1 (24.2-26.0)
Congo	19.7 (18.1-21.2)	21.1 (20.4-21.9)	22.2 (21.6-22.8)	23.2 (22.6-23.8)	24.0 (23.0-24.9)
Cook Islands	28.7 (26.1-31.2)	30.2 (28.5-32.0)	31.5 (30.3-32.8)	32.2 (31.1-33.4)	32.5 (30.9-34.1)
Costa Rica	21.9 (19.2-24.6)	23.4 (21.6-25.3)	24.9 (23.7-26.1)	26.2 (25.4-27.0)	27.0 (26.1-27.9)
Cote d'Ivoire	20.5 (18.3-22.7)	21.5 (20.2-22.8)	22.4 (21.6-23.1)	23.3 (22.7-23.9)	24.0 (23.0-24.9)
Croatia	23.2 (20.8-25.5)	24.0 (22.5-25.4)	24.4 (23.3-25.3)	24.8 (23.7-25.8)	25.2 (23.5-26.8)
Cuba	23.3 (21.5-25.1)	24.4 (23.2-25.5)	24.9 (24.1-25.7)	25.4 (24.8-26.0)	26.0 (25.1-26.9)
Cyprus	23.2 (20.5-25.7)	24.6 (22.9-26.4)	25.5 (24.4-26.8)	26.1 (25.0-27.4)	26.4 (24.7-28.2)
Czech Republic	26.3 (24.8-27.7)	26.5 (25.8-27.2)	26.2 (25.7-26.8)	26.0 (25.4-26.7)	25.8 (24.7-26.9)
Denmark	24.2 (23.0-25.4)	24.3 (23.6-25.1)	24.4 (23.8-25.0)	24.5 (23.8-25.1)	24.5 (23.4-25.5)
Djibouti	22.5 (19.1-25.9)	22.7 (19.7-25.6)	23.2 (20.4-26.0)	23.4 (20.6-26.3)	23.7 (20.7-26.8)
Dominica	24.0 (21.3-26.6)	25.3 (23.3-27.2)	26.7 (25.3-28.1)	27.7 (26.7-28.9)	28.8 (27.4-30.1)
Dominican Republic	22.0 (20.2-23.8)	23.6 (22.7-24.5)	24.8 (24.4-25.3)	26.2 (25.6-26.9)	27.1 (26.2-28.1)
DR Congo	19.0 (16.6-21.6)	19.9 (18.3-21.6)	20.8 (19.8-21.8)	21.5 (20.9-22.1)	22.5 (21.7-23.3)
Ecuador	22.5 (19.6-25.4)	24.0 (22.1-25.9)	25.3 (24.0-26.7)	26.5 (25.7-27.3)	27.6 (26.6-28.5)
Egypt	26.0 (24.2-27.8)	27.4 (26.5-28.2)	28.5 (28.1-28.9)	29.6 (29.3-29.9)	30.6 (30.0-31.2)

El Salvador	22.7 (20.1-25.3)	24.1 (22.4-25.8)	25.6 (24.6-26.7)	27.0 (26.3-27.7)	28.0 (26.9-29.1)
Equatorial Guinea	19.6 (16.6-22.6)	20.9 (18.6-23.1)	22.2 (20.4-23.9)	24.7 (23.5-25.9)	25.8 (24.5-27.1)
Eritrea	18.4 (16.3-20.5)	19.0 (17.7-20.2)	19.7 (19.1-20.4)	20.5 (20.0-21.0)	21.0 (20.0-21.9)
Estonia	24.7 (22.8-26.7)	25.0 (24.0-26.1)	25.1 (24.5-25.6)	25.5 (25.2-25.8)	25.8 (25.2-26.3)
Ethiopia	18.5 (16.1-21.0)	19.1 (17.5-20.7)	19.7 (18.8-20.6)	20.3 (19.7-21.0)	21.0 (20.0-21.9)
Fiji	24.6 (21.9-27.3)	25.9 (24.1-27.7)	27.0 (25.9-28.1)	28.0 (27.4-28.6)	28.6 (27.7-29.4)
Finland	23.6 (22.4-24.8)	24.4 (23.8-25.0)	25.1 (24.7-25.4)	25.4 (25.1-25.7)	25.4 (24.9-26.0)
France	24.2 (22.6-25.8)	24.2 (23.4-25.1)	24.3 (23.7-24.9)	24.4 (23.9-25.0)	24.4 (23.4-25.3)
French Polynesia	28.2 (25.3-31.3)	29.0 (26.8-31.3)	29.5 (27.9-31.2)	29.6 (28.5-30.7)	29.6 (28.4-30.7)
Gabon	20.8 (17.9-23.5)	22.7 (20.8-24.4)	23.9 (22.7-25.1)	25.2 (24.2-26.0)	26.2 (25.0-27.2)
Gambia	19.2 (17.0-21.5)	20.6 (19.2-22.0)	22.0 (21.2-22.8)	23.4 (22.8-23.9)	24.4 (23.6-25.1)
Georgia	23.7 (20.7-26.8)	24.9 (22.7-27.1)	25.3 (23.8-26.8)	26.2 (25.2-27.2)	27.2 (26.1-28.2)
Germany	24.1 (23.0-25.2)	24.5 (24.0-24.9)	24.9 (24.5-25.2)	25.3 (24.8-25.7)	25.6 (24.7-26.4)
Ghana	20.2 (18.1-22.1)	21.3 (20.1-22.4)	22.6 (22.0-23.2)	23.9 (23.5-24.3)	24.9 (24.0-25.8)
Greece	25.6 (23.4-27.9)	26.1 (24.8-27.4)	26.4 (25.6-27.2)	26.9 (25.9-27.8)	27.1 (25.5-28.7)
Greenland	24.9 (22.5-27.4)	25.5 (23.8-27.3)	25.9 (24.6-27.2)	26.3 (25.4-27.2)	26.5 (25.4-27.7)
Grenada	23.0 (20.2-25.8)	24.4 (22.4-26.5)	25.9 (24.3-27.4)	27.3 (26.2-28.5)	28.6 (27.4-29.7)
Guatemala	21.8 (19.6-24.0)	23.3 (22.1-24.6)	24.8 (24.1-25.5)	26.2 (25.6-26.8)	27.3 (26.2-28.3)
Guinea	19.0 (16.6-21.3)	20.4 (18.9-21.9)	21.4 (20.5-22.4)	22.5 (21.8-23.3)	23.3 (22.4-24.3)
Guinea Bissau	19.5 (16.7-22.3)	20.7 (18.7-22.7)	22.0 (20.5-23.6)	23.1 (21.9-24.2)	23.8 (22.5-25.1)
Guyana	22.6 (19.8-25.4)	23.8 (21.8-25.8)	25.0 (23.5-26.5)	26.4 (25.3-27.5)	27.5 (26.3-28.8)
Haiti	19.3 (17.1-21.5)	20.5 (19.1-21.9)	21.6 (20.8-22.6)	22.9 (22.2-23.7)	23.9 (22.9-25.0)
Honduras	20.6 (18.2-22.9)	22.4 (21.0-23.8)	24.1 (23.3-25.0)	25.9 (25.3-26.5)	27.3 (26.4-28.2)
Hungary	24.6 (23.2-26.2)	25.1 (24.4-25.8)	25.2 (24.3-26.1)	25.4 (24.0-26.9)	25.5 (23.4-27.8)
Iceland	23.7 (22.2-25.2)	24.4 (23.6-25.2)	24.7 (23.8-25.6)	25.0 (23.7-26.4)	25.2 (23.2-27.2)
India	18.8 (17.8-19.6)	19.6 (19.1-20.1)	20.4 (20.0-20.7)	21.0 (20.8-21.3)	21.5 (21.0-22.1)
Indonesia	19.0 (17.2-20.9)	20.3 (19.4-21.3)	21.6 (21.1-22.1)	22.6 (22.2-23.0)	23.4 (22.7-24.1)
Iran	23.8 (21.7-25.9)	24.7 (23.5-25.9)	25.4 (24.8-26.0)	26.3 (26.0-26.5)	27.0 (26.4-27.7)
Iraq	26.2 (23.5-28.8)	27.1 (25.2-28.9)	27.4 (26.1-28.7)	28.0 (27.0-29.0)	28.7 (27.4-30.1)
Ireland	22.4 (20.5-24.5)	23.8 (22.6-25.0)	25.1 (24.4-25.8)	26.4 (25.9-26.8)	27.0 (26.1-27.8)
Israel	24.7 (22.6-26.8)	25.3 (24.1-26.5)	25.9 (25.2-26.6)	26.3 (25.7-26.9)	26.7 (25.4-27.9)
Italy	24.3 (23.1-25.7)	24.5 (23.9-25.0)	24.6 (24.3-25.0)	24.9 (24.5-25.2)	25.1 (24.4-25.9)
Jamaica	23.5 (21.3-25.7)	24.8 (23.5-26.1)	26.3 (25.6-27.0)	27.8 (27.2-28.4)	29.2 (28.1-30.3)
Japan	22.0 (21.6-22.4)	22.1 (21.8-22.3)	22.1 (21.9-22.3)	22.0 (21.8-22.2)	21.7 (21.2-22.2)
Jordan	26.7 (24.5-28.8)	28.0 (26.8-29.3)	28.8 (28.2-29.4)	29.3 (28.9-29.6)	29.6 (29.0-30.3)
Kazakhstan	24.0 (21.5-26.5)	25.0 (23.5-26.5)	25.8 (24.8-26.7)	26.6 (25.3-27.7)	27.3 (25.3-29.2)
Kenya	19.7 (17.7-21.5)	20.9 (19.8-22.0)	22.0 (21.3-22.8)	23.0 (22.3-23.8)	24.0 (22.9-25.1)
Kiribati	24.9 (23.3-26.4)	26.6 (25.6-27.6)	28.3 (27.4-29.2)	29.8 (28.9-30.8)	30.8 (29.2-32.3)
Kuwait	28.7 (26.5-31.1)	29.0 (27.6-30.3)	29.3 (28.6-30.1)	29.9 (29.4-30.4)	30.3 (29.4-31.1)
Kyrgyzstan	21.9 (19.7-24.1)	23.2 (21.9-24.5)	24.4 (23.6-25.2)	25.6 (24.9-26.4)	26.9 (26.1-27.7)
Lao PDR	18.6 (16.0-21.3)	19.6 (17.7-21.4)	20.6 (19.3-21.9)	21.7 (20.9-22.6)	22.7 (21.8-23.5)
Latvia	24.4 (22.0-26.7)	25.0 (23.5-26.5)	25.1 (24.2-26.1)	25.5 (24.7-26.4)	26.0 (24.7-27.3)
Lebanon	24.4 (22.1-26.6)	25.3 (23.9-26.8)	25.9 (25.1-26.8)	26.6 (25.9-27.3)	27.2 (26.1-28.3)
Lesotho	23.9 (21.7-26.2)	24.7 (23.4-26.1)	25.6 (24.7-26.4)	26.4 (25.6-27.0)	27.0 (26.1-27.8)
Liberia	20.3 (17.5-23.0)	21.7 (19.7-23.5)	22.1 (20.6-23.5)	23.5 (22.5-24.3)	24.9 (23.9-25.7)
Libya	26.4 (23.7-29.0)	27.4 (25.7-29.2)	27.9 (26.6-29.0)	28.4 (27.4-29.3)	28.8 (27.7-30.0)
Lithuania	25.6 (23.7-27.5)	26.0 (24.9-27.0)	25.9 (25.2-26.5)	25.9 (25.2-26.6)	26.1 (24.7-27.5)
Luxembourg	23.8 (21.2-26.3)	24.2 (22.4-26.1)	24.7 (23.3-26.2)	25.2 (24.3-26.3)	25.4 (24.2-26.7)
Macedonia (TFYR)	24.5 (21.9-27.0)	25.0 (23.2-26.7)	25.2 (23.9-26.5)	25.3 (23.9-26.7)	25.4 (23.5-27.4)
Madagascar	19.4 (17.1-21.7)	19.9 (18.5-21.3)	20.3 (19.6-21.1)	20.7 (20.1-21.3)	21.0 (20.0-22.0)
Malawi	19.6 (17.6-21.5)	20.6 (19.5-21.7)	21.5 (20.9-22.1)	22.4 (21.9-22.9)	23.2 (22.4-24.0)
Malaysia	20.5 (18.3-22.7)	22.1 (20.8-23.4)	23.5 (22.8-24.2)	24.8 (24.5-25.2)	25.7 (25.0-26.5)
Maldives	20.4 (17.8-22.7)	22.1 (20.4-23.7)	23.4 (22.4-24.4)	24.7 (24.0-25.3)	25.4 (24.6-26.3)
Mali	18.4 (16.3-20.6)	19.7 (18.3-20.9)	20.8 (19.9-21.6)	22.1 (21.3-22.7)	22.9 (22.0-23.8)
Malta	26.1 (24.1-28.1)	26.5 (25.1-28.0)	26.6 (25.1-28.1)	26.8 (24.9-28.6)	26.7 (24.2-29.2)
Marshall Islands	27.3 (24.8-29.8)	28.4 (26.7-30.1)	29.5 (28.3-30.6)	30.0 (28.9-31.0)	30.2 (28.4-31.9)
Mauritania	21.6 (19.0-24.1)	23.1 (21.3-24.7)	24.4 (23.2-25.4)	25.4 (24.4-26.4)	26.3 (24.7-27.8)
Mauritius	22.9 (21.2-24.5)	23.7 (22.9-24.5)	24.7 (24.1-25.2)	25.4 (24.8-26.1)	26.0 (25.0-27.1)
Mexico	22.9 (21.1-24.6)	24.7 (23.8-25.6)	26.3 (25.9-26.8)	27.7 (27.4-28.0)	28.6 (28.0-29.2)

Micronesia (Federated States of)	27.3 (24.8-29.8)	28.6 (26.8-30.3)	29.7 (28.5-30.9)	30.6 (29.7-31.4)	31.2 (30.0-32.4)
Moldova	24.9 (22.2-27.6)	25.7 (23.9-27.6)	26.1 (24.8-27.4)	26.6 (25.8-27.5)	27.3 (26.4-28.2)
Mongolia	22.4 (20.0-25.0)	23.6 (22.0-25.2)	24.4 (23.5-25.3)	25.2 (24.7-25.7)	26.1 (25.4-26.9)
Montenegro	24.3 (20.8-27.7)	25.1 (22.2-28.0)	25.4 (22.6-28.1)	25.6 (22.7-28.4)	25.6 (22.5-28.7)
Morocco	23.0 (20.9-25.0)	24.0 (22.9-25.1)	24.8 (24.2-25.5)	25.6 (24.9-26.3)	26.2 (25.0-27.5)
Mozambique	19.3 (17.0-21.7)	20.4 (18.9-21.8)	21.4 (20.5-22.3)	22.3 (21.7-22.9)	22.9 (22.0-23.9)
Myanmar	18.7 (15.9-21.5)	19.8 (17.9-21.8)	21.0 (19.7-22.3)	22.2 (21.4-23.0)	23.3 (22.4-24.2)
Namibia	22.4 (20.3-24.5)	23.0 (21.8-24.2)	23.7 (22.9-24.4)	24.5 (24.0-25.1)	25.1 (24.4-25.9)
Nauru	33.6 (32.5-34.7)	34.1 (33.5-34.7)	34.0 (33.4-34.6)	33.3 (32.6-34.1)	32.9 (31.4-34.2)
Nepal	17.2 (15.2-19.3)	18.4 (17.1-19.6)	19.7 (19.0-20.4)	21.0 (20.6-21.5)	22.2 (21.5-22.9)
Netherlands	23.4 (21.7-25.1)	23.9 (22.9-24.8)	24.4 (23.8-25.0)	24.9 (24.5-25.4)	25.0 (24.2-25.8)
New Zealand	23.5 (22.1-24.9)	24.7 (23.9-25.4)	25.8 (25.4-26.4)	27.0 (26.6-27.4)	27.7 (27.2-28.2)
Nicaragua	23.2 (20.8-25.5)	24.4 (22.9-25.8)	25.5 (24.7-26.3)	26.8 (26.2-27.4)	27.8 (26.7-28.9)
Niger	18.7 (16.7-20.7)	19.5 (18.3-20.7)	20.4 (19.6-21.2)	21.4 (20.7-22.0)	22.1 (21.1-23.0)
Nigeria	20.8 (19.1-22.6)	21.7 (20.8-22.6)	22.4 (21.9-22.9)	23.2 (22.8-23.6)	23.9 (23.3-24.6)
Niue	28.4 (25.6-31.2)	30.2 (28.0-32.3)	31.5 (29.9-33.2)	32.5 (31.3-33.7)	33.2 (32.0-34.4)
North Korea	22.0 (18.4-25.5)	22.3 (19.3-25.3)	22.3 (19.4-25.1)	22.1 (19.0-25.0)	22.2 (18.8-25.6)
Norway	22.7 (21.2-24.1)	23.7 (22.9-24.5)	24.5 (23.9-25.1)	25.1 (24.4-25.9)	25.4 (24.1-26.6)
Occupied Palestinian Territory	25.8 (23.6-28.0)	26.8 (25.4-28.1)	27.7 (26.8-28.5)	28.7 (28.1-29.4)	29.5 (28.5-30.5)
Oman	23.9 (21.9-26.0)	25.1 (24.0-26.2)	25.7 (25.1-26.4)	26.0 (25.4-26.6)	26.1 (25.0-27.2)
Pakistan	20.0 (18.3-21.8)	21.2 (20.2-22.2)	22.3 (21.7-23.0)	23.4 (23.0-23.9)	24.4 (23.7-25.1)
Palau	28.8 (25.8-31.9)	29.3 (27.0-31.6)	29.8 (28.0-31.6)	29.9 (28.6-31.2)	29.6 (28.5-30.8)
Panama	22.9 (20.2-25.5)	24.3 (22.5-26.0)	25.5 (24.4-26.7)	26.7 (25.8-27.6)	27.8 (26.5-29.0)
Papua New Guinea	20.8 (18.3-23.2)	22.1 (20.4-23.7)	23.5 (22.2-24.7)	24.7 (23.5-25.6)	25.7 (24.3-27.1)
Paraguay	21.4 (17.9-25.1)	22.8 (19.8-26.0)	24.1 (21.3-27.1)	25.2 (22.3-28.1)	26.1 (22.9-29.2)
Peru	23.4 (21.4-25.5)	24.5 (23.5-25.6)	25.3 (24.8-25.8)	26.1 (25.8-26.4)	27.0 (26.5-27.4)
Philippines	19.3 (18.0-20.5)	20.7 (20.2-21.3)	21.9 (21.5-22.3)	22.7 (22.2-23.2)	23.4 (22.3-24.4)
Poland	25.2 (23.7-26.7)	25.4 (24.6-26.2)	25.4 (24.9-26.0)	25.6 (25.2-26.0)	25.8 (25.1-26.5)
Portugal	24.2 (22.1-26.3)	24.6 (23.2-25.9)	25.0 (24.2-25.9)	25.4 (24.8-25.9)	25.4 (24.4-26.4)
Puerto Rico	24.4 (21.8-27.0)	25.8 (24.0-27.6)	27.0 (25.8-28.2)	27.8 (26.9-28.6)	28.3 (27.2-29.5)
Qatar	26.6 (23.9-29.3)	28.1 (26.3-30.0)	28.4 (27.2-29.7)	29.2 (28.4-30.0)	29.8 (28.9-30.7)
Romania	23.8 (21.7-25.9)	24.5 (23.2-25.7)	24.7 (23.9-25.4)	24.9 (23.8-25.9)	25.1 (23.4-26.8)
Russian Federation	25.6 (24.2-27.0)	26.1 (25.5-26.7)	26.2 (26.0-26.5)	26.6 (26.2-27.0)	27.0 (26.1-27.9)
Rwanda	18.5 (16.0-20.9)	19.6 (17.9-21.1)	20.7 (19.7-21.6)	21.8 (21.1-22.4)	22.6 (21.8-23.4)
Saint Kitts and Nevis	24.9 (22.1-27.8)	26.5 (24.4-28.7)	27.9 (26.2-29.7)	29.3 (27.7-30.9)	30.5 (28.7-32.4)
Saint Lucia	23.3 (21.9-24.7)	24.9 (24.0-25.8)	26.6 (25.7-27.5)	28.3 (27.4-29.2)	30.0 (29.0-31.1)
Saint Vincent and the Grenadines	22.8 (19.4-26.0)	24.1 (21.3-27.0)	25.6 (22.9-28.3)	26.9 (24.2-29.6)	27.9 (24.9-30.8)
Samoa	27.8 (26.5-29.1)	29.4 (28.7-30.1)	31.1 (30.6-31.7)	32.7 (32.1-33.2)	33.8 (33.0-34.6)
Sao Tome and Principe	21.4 (18.5-24.0)	22.4 (20.4-24.3)	23.6 (22.2-24.9)	24.6 (23.8-25.5)	25.5 (24.5-26.5)
Saudi Arabia	26.1 (24.5-27.8)	27.2 (26.5-27.9)	27.9 (27.5-28.2)	28.3 (27.9-28.8)	28.9 (28.2-29.7)
Senegal	20.8 (19.4-22.2)	21.7 (21.0-22.5)	22.5 (21.9-23.1)	23.4 (22.8-24.0)	24.0 (23.0-24.8)
Serbia	24.1 (22.3-26.0)	24.7 (23.7-25.7)	24.9 (24.3-25.6)	25.1 (24.6-25.6)	25.3 (24.5-26.0)
Seychelles	23.8 (22.0-25.6)	25.1 (24.2-26.1)	26.2 (25.6-26.8)	27.2 (26.5-27.8)	27.8 (27.0-28.7)
Sierra Leone	20.6 (18.0-23.3)	21.5 (19.7-23.4)	22.2 (20.9-23.4)	22.9 (22.1-23.7)	23.6 (22.8-24.4)
Singapore	23.1 (21.6-24.4)	23.4 (22.7-24.1)	23.5 (23.0-23.9)	23.2 (22.8-23.6)	23.1 (22.4-23.7)
Slovakia	23.9 (21.9-25.9)	24.6 (23.5-25.8)	24.9 (24.2-25.6)	25.1 (24.5-25.7)	25.4 (24.5-26.3)
Slovenia	24.3 (21.3-27.2)	25.1 (22.6-27.4)	25.5 (23.4-27.6)	26.0 (24.0-27.9)	26.3 (24.0-28.5)
Solomon Islands	22.3 (19.2-25.4)	23.7 (21.5-25.9)	24.9 (23.4-26.5)	25.8 (24.6-26.9)	26.4 (25.1-27.7)
Somalia	20.2 (16.9-23.6)	20.9 (18.1-23.8)	21.5 (18.8-24.3)	22.0 (19.3-24.9)	22.5 (19.5-25.6)
South Africa	26.4 (24.3-28.6)	27.0 (25.8-28.2)	27.6 (26.9-28.2)	28.4 (28.0-28.8)	29.2 (28.7-29.8)
South Korea	19.7 (18.0-21.4)	21.6 (20.8-22.5)	22.8 (22.3-23.2)	22.9 (22.6-23.3)	23.2 (22.7-23.7)
Spain	25.1 (23.6-26.6)	25.4 (24.7-26.1)	25.6 (25.2-26.0)	25.8 (25.4-26.2)	25.8 (24.9-26.7)
Sri Lanka	19.2 (16.7-21.7)	20.2 (18.5-21.9)	21.4 (20.4-22.4)	22.5 (21.9-23.1)	23.6 (22.5-24.6)
Sudan	22.4 (19.6-25.0)	23.5 (21.6-25.4)	24.7 (23.4-26.0)	25.8 (24.7-26.9)	26.6 (25.1-28.0)
Suriname	24.3 (20.9-27.6)	25.6 (22.7-28.5)	26.3 (23.5-29.0)	27.1 (24.4-29.9)	28.0 (25.1-31.1)
Swaziland	24.3 (21.3-27.2)	25.6 (23.5-27.6)	26.7 (25.3-28.2)	27.8 (26.7-29.0)	28.8 (27.4-30.2)
Sweden	23.5 (22.0-25.0)	23.9 (23.2-24.7)	24.4 (24.0-24.9)	25.0 (24.5-25.5)	25.3 (24.4-26.3)
Switzerland	23.2 (21.5-25.0)	23.5 (22.5-24.6)	23.9 (23.1-24.7)	23.9 (23.0-24.7)	23.7 (22.4-24.9)
Syrian Arab Republic	25.2 (22.6-27.7)	26.4 (24.7-28.0)	27.2 (26.1-28.3)	28.1 (27.1-29.1)	28.8 (27.3-30.3)

Taiwan	21.3 (19.4-23.1)	22.2 (21.2-23.2)	22.7 (22.2-23.2)	22.9 (22.4-23.4)	23.0 (21.9-24.1)
Tajikistan	22.3 (19.4-25.2)	23.1 (21.0-25.1)	23.6 (22.3-24.9)	24.3 (23.3-25.3)	25.4 (24.3-26.5)
Tanzania	19.9 (17.9-21.8)	21.0 (20.0-22.0)	22.0 (21.4-22.5)	22.9 (22.5-23.4)	23.8 (23.2-24.5)
Thailand	20.9 (19.1-22.6)	22.0 (21.1-23.0)	23.1 (22.6-23.5)	23.9 (23.4-24.3)	24.5 (23.5-25.4)
Timor-Leste	17.5 (14.9-20.3)	18.4 (16.5-20.4)	19.4 (18.1-20.8)	20.1 (19.2-21.0)	20.8 (19.8-21.7)
Togo	19.1 (16.6-21.5)	20.4 (18.8-21.9)	21.6 (20.6-22.6)	23.1 (22.3-23.8)	24.3 (23.4-25.1)
Tokelau	28.4 (25.5-31.2)	30.0 (27.9-32.0)	31.5 (30.0-32.9)	32.7 (31.5-33.8)	33.7 (32.0-35.2)
Tonga	29.0 (26.3-31.6)	30.5 (28.6-32.3)	31.9 (30.5-33.1)	32.9 (31.9-33.7)	33.5 (32.3-34.5)
Trinidad and Tobago	24.0 (21.8-26.2)	25.2 (23.7-26.7)	26.2 (25.1-27.2)	27.6 (26.4-28.8)	28.8 (26.9-30.7)
Tunisia	24.1 (21.8-26.4)	25.1 (23.8-26.4)	25.8 (25.1-26.6)	26.4 (25.8-27.1)	26.9 (25.8-28.0)
Turkey	25.0 (23.2-26.7)	26.1 (25.3-27.0)	27.2 (26.8-27.6)	28.0 (27.7-28.2)	28.6 (28.0-29.1)
Turkmenistan	22.3 (19.7-24.8)	23.2 (21.5-24.8)	24.2 (23.1-25.3)	25.2 (24.4-26.0)	26.4 (25.5-27.4)
Tuvalu	27.8 (26.1-29.5)	29.2 (27.6-30.7)	30.5 (28.8-32.3)	31.2 (29.2-33.2)	31.5 (28.9-34.0)
Uganda	19.6 (17.5-21.8)	20.4 (19.1-21.6)	21.2 (20.4-21.9)	22.1 (21.5-22.7)	23.0 (22.1-23.9)
Ukraine	25.0 (22.3-27.7)	25.5 (23.6-27.5)	25.7 (24.3-27.1)	25.8 (24.5-27.1)	26.1 (24.3-28.0)
United Arab Emirates	27.8 (25.3-30.3)	27.9 (26.5-29.3)	28.2 (27.3-29.0)	28.8 (27.9-29.7)	28.9 (27.3-30.5)
United Kingdom	23.4 (22.3-24.4)	24.4 (24.0-24.8)	25.5 (25.3-25.8)	26.5 (26.3-26.7)	27.0 (26.6-27.4)
United States of America	24.6 (23.9-25.2)	25.5 (25.1-26.0)	26.8 (26.4-27.1)	27.9 (27.7-28.2)	28.7 (28.1-29.3)
Uruguay	24.2 (21.6-26.9)	25.0 (23.2-26.8)	25.7 (24.4-26.9)	26.3 (25.4-27.2)	27.0 (25.8-28.2)
Uzbekistan	22.1 (19.6-24.6)	23.2 (21.6-24.7)	24.2 (23.2-25.2)	25.2 (24.1-26.2)	26.4 (24.7-28.0)
Vanuatu	23.0 (20.5-25.3)	24.2 (22.6-25.6)	25.2 (24.3-26.1)	26.1 (25.3-26.8)	26.7 (25.5-27.8)
Venezuela	23.9 (21.4-26.7)	24.9 (23.3-26.7)	25.9 (24.8-27.0)	26.5 (25.8-27.3)	27.1 (25.9-28.2)
Viet Nam	18.0 (16.5-19.4)	18.7 (18.1-19.4)	19.6 (19.2-20.0)	20.5 (20.2-20.8)	21.1 (20.4-21.8)
Yemen	20.1 (17.8-22.3)	20.9 (19.5-22.3)	21.9 (21.1-22.7)	22.8 (22.3-23.4)	23.5 (22.7-24.4)
Zambia	21.0 (19.1-22.9)	21.8 (20.7-22.8)	22.2 (21.7-22.8)	22.8 (22.4-23.3)	23.6 (22.8-24.3)
Zimbabwe	24.1 (22.2-26.1)	24.4 (23.4-25.5)	24.7 (24.1-25.2)	24.9 (24.4-25.4)	25.0 (24.1-25.9)

Appendix Table 5: Age-standardised prevalences of BMI <18.5 kg/m^2, 18.5 to <20 kg/m^2, 20 to <25 kg/m^2, 25 to <30 kg/m^2, 30 to <35 kg/m^2, 35 to <40 kg/m^2, ≥40 kg/m^2 by sex and country in 1975, 1985, 1995, 2005, and 2014. Numbers in brackets show 95% credible intervals. Estimates for all countries and years can be downloaded from www.ncdrisc.org.

Men, BMI <18.5 kg/m^2

Country	Year				
	1975	1985	1995	2005	2014
Afghanistan	0.295 (0.153-0.446)	0.277 (0.154-0.402)	0.255 (0.142-0.375)	0.232 (0.126-0.350)	0.210 (0.102-0.335)
Albania	0.026 (0.005-0.073)	0.021 (0.006-0.049)	0.016 (0.005-0.036)	0.010 (0.004-0.022)	0.006 (0.002-0.015)
Algeria	0.124 (0.045-0.234)	0.102 (0.048-0.180)	0.083 (0.042-0.142)	0.064 (0.032-0.109)	0.044 (0.017-0.086)
American Samoa	0.005 (0.001-0.014)	0.004 (0.001-0.008)	0.003 (0.001-0.006)	0.002 (0.000-0.004)	0.001 (0.000-0.004)
Andorra	0.021 (0.005-0.059)	0.013 (0.004-0.033)	0.009 (0.002-0.021)	0.005 (0.001-0.013)	0.004 (0.001-0.011)
Angola	0.226 (0.091-0.379)	0.225 (0.107-0.355)	0.216 (0.106-0.337)	0.206 (0.099-0.325)	0.177 (0.068-0.311)
Antigua and Barbuda	0.093 (0.025-0.201)	0.074 (0.024-0.153)	0.051 (0.017-0.108)	0.039 (0.012-0.084)	0.026 (0.006-0.068)
Argentina	0.022 (0.005-0.057)	0.015 (0.005-0.031)	0.010 (0.004-0.021)	0.006 (0.003-0.013)	0.004 (0.001-0.010)
Armenia	0.064 (0.013-0.169)	0.045 (0.012-0.108)	0.034 (0.011-0.078)	0.024 (0.008-0.053)	0.013 (0.003-0.034)
Australia	0.018 (0.008-0.034)	0.010 (0.006-0.016)	0.008 (0.004-0.012)	0.006 (0.003-0.009)	0.004 (0.002-0.008)
Austria	0.027 (0.010-0.058)	0.017 (0.008-0.032)	0.012 (0.006-0.022)	0.009 (0.004-0.016)	0.006 (0.002-0.014)
Azerbaijan	0.056 (0.014-0.139)	0.038 (0.012-0.087)	0.029 (0.010-0.062)	0.021 (0.007-0.046)	0.013 (0.003-0.032)
Bahamas	0.091 (0.025-0.200)	0.068 (0.022-0.142)	0.047 (0.015-0.100)	0.029 (0.009-0.064)	0.019 (0.005-0.048)
Bahrain	0.105 (0.038-0.206)	0.077 (0.037-0.137)	0.055 (0.028-0.093)	0.035 (0.017-0.063)	0.023 (0.008-0.050)
Bangladesh	0.316 (0.189-0.439)	0.308 (0.218-0.400)	0.292 (0.217-0.367)	0.264 (0.194-0.335)	0.231 (0.147-0.321)
Barbados	0.108 (0.040-0.203)	0.080 (0.037-0.136)	0.063 (0.033-0.103)	0.045 (0.021-0.080)	0.031 (0.010-0.069)
Belarus	0.030 (0.006-0.080)	0.021 (0.005-0.052)	0.017 (0.004-0.040)	0.012 (0.003-0.029)	0.007 (0.002-0.020)
Belgium	0.018 (0.006-0.037)	0.011 (0.006-0.018)	0.007 (0.004-0.011)	0.004 (0.002-0.007)	0.003 (0.001-0.006)
Belize	0.125 (0.039-0.252)	0.096 (0.038-0.179)	0.074 (0.032-0.134)	0.051 (0.022-0.093)	0.038 (0.013-0.081)
Benin	0.164 (0.061-0.295)	0.157 (0.076-0.256)	0.151 (0.080-0.234)	0.135 (0.075-0.208)	0.119 (0.057-0.197)
Bermuda	0.074 (0.017-0.178)	0.049 (0.014-0.114)	0.033 (0.009-0.077)	0.024 (0.007-0.055)	0.015 (0.003-0.039)
Bhutan	0.237 (0.108-0.389)	0.219 (0.113-0.336)	0.196 (0.104-0.299)	0.163 (0.085-0.251)	0.127 (0.058-0.218)
Bolivia	0.050 (0.010-0.132)	0.037 (0.009-0.090)	0.027 (0.008-0.065)	0.019 (0.006-0.043)	0.013 (0.003-0.035)
Bosnia and Herzegovina	0.033 (0.005-0.099)	0.025 (0.006-0.062)	0.019 (0.006-0.043)	0.011 (0.004-0.023)	0.007 (0.002-0.015)
Botswana	0.200 (0.084-0.337)	0.191 (0.097-0.298)	0.170 (0.088-0.266)	0.145 (0.075-0.237)	0.114 (0.048-0.210)
Brazil	0.093 (0.036-0.181)	0.067 (0.037-0.108)	0.046 (0.031-0.067)	0.031 (0.021-0.043)	0.021 (0.011-0.037)
Brunei Darussalam	0.170 (0.045-0.339)	0.141 (0.045-0.274)	0.118 (0.044-0.224)	0.089 (0.033-0.174)	0.069 (0.020-0.152)
Bulgaria	0.020 (0.004-0.055)	0.012 (0.004-0.029)	0.008 (0.003-0.020)	0.007 (0.002-0.016)	0.005 (0.001-0.014)
Burkina Faso	0.176 (0.067-0.315)	0.169 (0.083-0.270)	0.163 (0.090-0.247)	0.149 (0.086-0.224)	0.137 (0.070-0.223)
Burundi	0.208 (0.071-0.371)	0.199 (0.084-0.336)	0.187 (0.085-0.310)	0.174 (0.082-0.286)	0.159 (0.066-0.275)
Cabo Verde	0.183 (0.068-0.326)	0.166 (0.079-0.269)	0.150 (0.076-0.239)	0.116 (0.057-0.191)	0.092 (0.037-0.171)
Cambodia	0.236 (0.104-0.382)	0.229 (0.130-0.336)	0.220 (0.132-0.316)	0.196 (0.119-0.284)	0.163 (0.084-0.259)
Cameroon	0.132 (0.051-0.244)	0.118 (0.061-0.191)	0.108 (0.062-0.166)	0.096 (0.057-0.144)	0.079 (0.036-0.139)
Canada	0.015 (0.005-0.032)	0.009 (0.005-0.016)	0.007 (0.003-0.011)	0.005 (0.002-0.008)	0.004 (0.001-0.008)
Central African Republic	0.233 (0.097-0.392)	0.225 (0.105-0.359)	0.223 (0.116-0.342)	0.209 (0.113-0.318)	0.191 (0.092-0.307)
Chad	0.192 (0.079-0.328)	0.183 (0.093-0.292)	0.170 (0.091-0.267)	0.154 (0.084-0.240)	0.148 (0.070-0.248)
Chile	0.028 (0.007-0.076)	0.018 (0.007-0.041)	0.011 (0.005-0.022)	0.006 (0.003-0.011)	0.003 (0.001-0.007)
China	0.114 (0.055-0.193)	0.108 (0.079-0.140)	0.093 (0.074-0.112)	0.060 (0.047-0.077)	0.034 (0.019-0.054)
China (Hong Kong SAR)	0.111 (0.035-0.232)	0.082 (0.035-0.151)	0.059 (0.026-0.109)	0.039 (0.015-0.078)	0.023 (0.006-0.059)
Colombia	0.080 (0.026-0.173)	0.059 (0.026-0.111)	0.043 (0.022-0.074)	0.028 (0.016-0.046)	0.019 (0.008-0.036)
Comoros	0.158 (0.057-0.288)	0.157 (0.074-0.261)	0.149 (0.074-0.244)	0.137 (0.069-0.222)	0.123 (0.056-0.211)
Congo	0.226 (0.114-0.354)	0.211 (0.124-0.308)	0.194 (0.120-0.281)	0.181 (0.109-0.264)	0.157 (0.074-0.260)
Cook Islands	0.008 (0.001-0.025)	0.005 (0.001-0.014)	0.004 (0.001-0.009)	0.002 (0.001-0.006)	0.002 (0.000-0.005)
Costa Rica	0.073 (0.021-0.168)	0.052 (0.020-0.106)	0.036 (0.015-0.071)	0.023 (0.010-0.043)	0.015 (0.006-0.031)
Cote d'Ivoire	0.131 (0.047-0.246)	0.124 (0.058-0.207)	0.116 (0.058-0.188)	0.099 (0.051-0.162)	0.083 (0.036-0.152)
Croatia	0.030 (0.007-0.079)	0.020 (0.006-0.047)	0.015 (0.005-0.033)	0.009 (0.003-0.021)	0.006 (0.001-0.015)
Cuba	0.146 (0.063-0.260)	0.112 (0.058-0.184)	0.089 (0.048-0.146)	0.071 (0.040-0.115)	0.048 (0.022-0.088)
Cyprus	0.021 (0.005-0.058)	0.015 (0.004-0.035)	0.009 (0.003-0.021)	0.006 (0.002-0.014)	0.004 (0.001-0.012)
Czech Republic	0.009 (0.002-0.022)	0.006 (0.002-0.012)	0.004 (0.002-0.008)	0.003 (0.001-0.006)	0.002 (0.001-0.006)
Denmark	0.025 (0.012-0.045)	0.015 (0.008-0.025)	0.010 (0.005-0.016)	0.006 (0.003-0.011)	0.005 (0.002-0.009)
Djibouti	0.198 (0.072-0.356)	0.186 (0.074-0.332)	0.169 (0.076-0.289)	0.153 (0.066-0.261)	0.129 (0.044-0.246)
Dominica	0.120 (0.042-0.241)	0.094 (0.037-0.179)	0.069 (0.028-0.132)	0.043 (0.017-0.084)	0.029 (0.009-0.066)
Dominican Republic	0.131 (0.052-0.244)	0.109 (0.055-0.181)	0.087 (0.047-0.140)	0.057 (0.029-0.094)	0.037 (0.014-0.074)
DR Congo	0.237 (0.109-0.376)	0.228 (0.124-0.348)	0.221 (0.124-0.331)	0.212 (0.120-0.318)	0.191 (0.089-0.309)
Ecuador	0.039 (0.007-0.108)	0.025 (0.006-0.063)	0.018 (0.005-0.043)	0.012 (0.004-0.028)	0.008 (0.002-0.021)
Egypt	0.067 (0.022-0.146)	0.048 (0.023-0.086)	0.036 (0.019-0.060)	0.025 (0.014-0.041)	0.017 (0.007-0.033)
El Salvador	0.078 (0.019-0.186)	0.060 (0.020-0.129)	0.045 (0.016-0.092)	0.029 (0.011-0.058)	0.019 (0.006-0.044)
Equatorial Guinea	0.223 (0.085-0.386)	0.208 (0.092-0.339)	0.197 (0.089-0.320)	0.169 (0.069-0.295)	0.145 (0.044-0.294)
Eritrea	0.240 (0.117-0.374)	0.234 (0.136-0.336)	0.225 (0.141-0.314)	0.215 (0.140-0.297)	0.204 (0.118-0.301)
Estonia	0.026 (0.008-0.060)	0.018 (0.008-0.034)	0.015 (0.008-0.025)	0.009 (0.005-0.014)	0.006 (0.003-0.011)
Ethiopia	0.226 (0.101-0.369)	0.218 (0.119-0.329)	0.212 (0.126-0.307)	0.209 (0.130-0.295)	0.202 (0.116-0.301)
Fiji	0.058 (0.012-0.139)	0.044 (0.014-0.096)	0.036 (0.013-0.075)	0.028 (0.012-0.055)	0.022 (0.008-0.048)
Finland	0.015 (0.006-0.030)	0.009 (0.005-0.015)	0.006 (0.003-0.010)	0.004 (0.002-0.007)	0.003 (0.001-0.006)
France	0.020 (0.007-0.042)	0.013 (0.006-0.024)	0.009 (0.005-0.017)	0.007 (0.003-0.012)	0.005 (0.002-0.012)
French Polynesia	0.008 (0.001-0.025)	0.006 (0.001-0.016)	0.004 (0.001-0.010)	0.002 (0.001-0.006)	0.002 (0.000-0.005)

Gabon	0.187 (0.068-0.339)	0.168 (0.071-0.286)	0.149 (0.064-0.257)	0.128 (0.053-0.223)	0.107 (0.039-0.207)
Gambia	0.186 (0.080-0.318)	0.180 (0.099-0.274)	0.164 (0.094-0.248)	0.139 (0.080-0.213)	0.121 (0.056-0.204)
Georgia	0.058 (0.014-0.144)	0.040 (0.012-0.091)	0.032 (0.010-0.072)	0.023 (0.008-0.050)	0.016 (0.005-0.036)
Germany	0.015 (0.007-0.029)	0.009 (0.006-0.014)	0.007 (0.004-0.010)	0.005 (0.003-0.007)	0.003 (0.001-0.006)
Ghana	0.165 (0.073-0.279)	0.158 (0.090-0.240)	0.146 (0.091-0.211)	0.120 (0.074-0.173)	0.095 (0.046-0.161)
Greece	0.021 (0.006-0.053)	0.013 (0.005-0.027)	0.008 (0.003-0.018)	0.006 (0.002-0.012)	0.004 (0.001-0.011)
Greenland	0.027 (0.008-0.063)	0.017 (0.006-0.035)	0.011 (0.004-0.024)	0.008 (0.003-0.017)	0.006 (0.002-0.013)
Grenada	0.117 (0.040-0.235)	0.094 (0.039-0.177)	0.070 (0.029-0.134)	0.050 (0.020-0.096)	0.034 (0.011-0.074)
Guatemala	0.089 (0.028-0.195)	0.071 (0.029-0.137)	0.056 (0.025-0.102)	0.037 (0.017-0.069)	0.026 (0.008-0.055)
Guinea	0.176 (0.062-0.318)	0.166 (0.076-0.272)	0.160 (0.080-0.253)	0.144 (0.077-0.224)	0.129 (0.061-0.214)
Guinea Bissau	0.172 (0.063-0.311)	0.168 (0.077-0.277)	0.157 (0.077-0.259)	0.147 (0.072-0.239)	0.131 (0.054-0.232)
Guyana	0.126 (0.043-0.249)	0.105 (0.043-0.194)	0.087 (0.037-0.160)	0.062 (0.025-0.119)	0.051 (0.017-0.108)
Haiti	0.154 (0.050-0.296)	0.135 (0.053-0.244)	0.122 (0.049-0.222)	0.098 (0.036-0.184)	0.076 (0.021-0.163)
Honduras	0.091 (0.025-0.201)	0.073 (0.026-0.147)	0.057 (0.022-0.113)	0.039 (0.015-0.077)	0.028 (0.008-0.063)
Hungary	0.019 (0.006-0.045)	0.012 (0.005-0.023)	0.008 (0.004-0.016)	0.007 (0.003-0.013)	0.005 (0.001-0.012)
Iceland	0.013 (0.004-0.031)	0.006 (0.002-0.013)	0.005 (0.002-0.010)	0.003 (0.001-0.007)	0.002 (0.001-0.005)
India	0.364 (0.265-0.465)	0.340 (0.277-0.404)	0.316 (0.275-0.358)	0.282 (0.244-0.319)	0.245 (0.181-0.310)
Indonesia	0.233 (0.131-0.345)	0.227 (0.160-0.298)	0.213 (0.158-0.271)	0.185 (0.136-0.237)	0.149 (0.092-0.215)
Iran	0.108 (0.040-0.205)	0.085 (0.048-0.134)	0.066 (0.045-0.093)	0.046 (0.034-0.060)	0.029 (0.016-0.047)
Iraq	0.085 (0.025-0.192)	0.066 (0.025-0.134)	0.055 (0.022-0.108)	0.041 (0.016-0.080)	0.029 (0.010-0.065)
Ireland	0.016 (0.004-0.040)	0.010 (0.003-0.022)	0.007 (0.003-0.014)	0.005 (0.002-0.009)	0.004 (0.001-0.009)
Israel	0.021 (0.006-0.049)	0.014 (0.006-0.028)	0.009 (0.004-0.018)	0.006 (0.003-0.012)	0.005 (0.001-0.011)
Italy	0.019 (0.007-0.039)	0.012 (0.007-0.019)	0.008 (0.005-0.013)	0.006 (0.003-0.009)	0.004 (0.002-0.009)
Jamaica	0.132 (0.054-0.234)	0.113 (0.064-0.175)	0.091 (0.057-0.134)	0.062 (0.036-0.096)	0.042 (0.017-0.080)
Japan	0.092 (0.062-0.128)	0.073 (0.058-0.090)	0.059 (0.047-0.072)	0.050 (0.039-0.064)	0.045 (0.027-0.069)
Jordan	0.061 (0.017-0.145)	0.040 (0.017-0.077)	0.026 (0.012-0.047)	0.016 (0.007-0.028)	0.010 (0.004-0.021)
Kazakhstan	0.067 (0.018-0.157)	0.046 (0.015-0.100)	0.033 (0.011-0.074)	0.022 (0.007-0.052)	0.012 (0.003-0.033)
Kenya	0.195 (0.081-0.325)	0.179 (0.091-0.283)	0.163 (0.084-0.258)	0.151 (0.077-0.243)	0.133 (0.056-0.237)
Kiribati	0.010 (0.002-0.028)	0.008 (0.002-0.019)	0.006 (0.002-0.015)	0.004 (0.001-0.010)	0.003 (0.001-0.010)
Kuwait	0.053 (0.016-0.120)	0.034 (0.016-0.060)	0.024 (0.013-0.041)	0.013 (0.007-0.022)	0.008 (0.003-0.016)
Kyrgyzstan	0.066 (0.017-0.160)	0.048 (0.016-0.105)	0.037 (0.013-0.078)	0.028 (0.011-0.060)	0.020 (0.006-0.046)
Lao PDR	0.224 (0.106-0.353)	0.219 (0.127-0.320)	0.209 (0.128-0.297)	0.186 (0.115-0.268)	0.153 (0.080-0.240)
Latvia	0.031 (0.007-0.080)	0.021 (0.007-0.048)	0.017 (0.006-0.038)	0.012 (0.004-0.027)	0.007 (0.002-0.017)
Lebanon	0.066 (0.021-0.144)	0.047 (0.019-0.094)	0.028 (0.012-0.056)	0.015 (0.006-0.031)	0.010 (0.003-0.023)
Lesotho	0.192 (0.077-0.330)	0.179 (0.090-0.284)	0.168 (0.090-0.258)	0.154 (0.086-0.235)	0.135 (0.067-0.223)
Liberia	0.148 (0.056-0.273)	0.142 (0.064-0.243)	0.137 (0.061-0.241)	0.116 (0.059-0.188)	0.098 (0.045-0.171)
Libya	0.080 (0.020-0.185)	0.054 (0.022-0.106)	0.040 (0.018-0.076)	0.029 (0.013-0.054)	0.020 (0.007-0.044)
Lithuania	0.019 (0.005-0.046)	0.012 (0.004-0.025)	0.009 (0.004-0.018)	0.006 (0.003-0.012)	0.003 (0.001-0.009)
Luxembourg	0.018 (0.005-0.046)	0.011 (0.003-0.026)	0.006 (0.002-0.016)	0.005 (0.002-0.011)	0.004 (0.001-0.009)
Macedonia (TFYR)	0.029 (0.006-0.082)	0.020 (0.005-0.048)	0.015 (0.004-0.036)	0.010 (0.003-0.024)	0.007 (0.002-0.019)
Madagascar	0.198 (0.087-0.327)	0.191 (0.106-0.289)	0.182 (0.106-0.270)	0.173 (0.102-0.256)	0.161 (0.080-0.263)
Malawi	0.188 (0.073-0.330)	0.178 (0.090-0.283)	0.173 (0.095-0.264)	0.158 (0.090-0.237)	0.140 (0.066-0.229)
Malaysia	0.194 (0.096-0.310)	0.165 (0.098-0.243)	0.131 (0.083-0.189)	0.099 (0.066-0.140)	0.075 (0.039-0.122)
Maldives	0.185 (0.081-0.319)	0.178 (0.095-0.275)	0.150 (0.079-0.237)	0.114 (0.055-0.193)	0.093 (0.040-0.169)
Mali	0.178 (0.065-0.316)	0.170 (0.082-0.276)	0.168 (0.090-0.263)	0.155 (0.088-0.237)	0.138 (0.065-0.228)
Malta	0.030 (0.006-0.084)	0.016 (0.005-0.038)	0.011 (0.004-0.024)	0.006 (0.002-0.015)	0.005 (0.001-0.012)
Marshall Islands	0.011 (0.002-0.036)	0.008 (0.002-0.022)	0.006 (0.002-0.014)	0.004 (0.001-0.010)	0.003 (0.001-0.009)
Mauritania	0.177 (0.070-0.313)	0.174 (0.087-0.280)	0.165 (0.087-0.261)	0.147 (0.078-0.232)	0.124 (0.054-0.218)
Mauritius	0.164 (0.073-0.280)	0.138 (0.078-0.209)	0.113 (0.065-0.172)	0.089 (0.048-0.141)	0.071 (0.028-0.133)
Mexico	0.069 (0.024-0.153)	0.045 (0.022-0.082)	0.027 (0.016-0.044)	0.016 (0.010-0.024)	0.010 (0.005-0.018)
Micronesia (Federated States of)	0.016 (0.003-0.045)	0.012 (0.004-0.030)	0.010 (0.003-0.023)	0.008 (0.003-0.017)	0.006 (0.002-0.015)
Moldova	0.037 (0.008-0.094)	0.028 (0.008-0.065)	0.025 (0.008-0.056)	0.023 (0.008-0.050)	0.015 (0.004-0.036)
Mongolia	0.069 (0.018-0.160)	0.052 (0.020-0.104)	0.041 (0.019-0.076)	0.030 (0.015-0.053)	0.020 (0.008-0.040)
Montenegro	0.031 (0.005-0.092)	0.021 (0.005-0.054)	0.015 (0.004-0.037)	0.009 (0.002-0.021)	0.005 (0.001-0.015)
Morocco	0.106 (0.033-0.219)	0.087 (0.037-0.164)	0.070 (0.032-0.128)	0.051 (0.023-0.096)	0.036 (0.012-0.077)
Mozambique	0.183 (0.068-0.336)	0.178 (0.084-0.294)	0.174 (0.092-0.270)	0.162 (0.090-0.249)	0.153 (0.076-0.252)
Myanmar	0.255 (0.109-0.414)	0.244 (0.140-0.357)	0.230 (0.146-0.320)	0.209 (0.140-0.283)	0.167 (0.098-0.247)
Namibia	0.174 (0.065-0.309)	0.167 (0.083-0.267)	0.160 (0.086-0.246)	0.137 (0.075-0.214)	0.111 (0.050-0.193)
Nauru	0.004 (0.001-0.012)	0.003 (0.001-0.006)	0.002 (0.001-0.005)	0.002 (0.000-0.004)	0.001 (0.000-0.004)
Nepal	0.275 (0.145-0.417)	0.261 (0.163-0.368)	0.241 (0.157-0.331)	0.212 (0.135-0.294)	0.180 (0.098-0.272)
Netherlands	0.029 (0.011-0.061)	0.021 (0.011-0.035)	0.014 (0.008-0.022)	0.008 (0.005-0.013)	0.005 (0.002-0.011)
New Zealand	0.016 (0.005-0.036)	0.009 (0.004-0.017)	0.006 (0.003-0.011)	0.005 (0.003-0.009)	0.004 (0.002-0.008)
Nicaragua	0.088 (0.024-0.201)	0.068 (0.024-0.139)	0.057 (0.023-0.111)	0.042 (0.017-0.082)	0.031 (0.010-0.070)
Niger	0.200 (0.087-0.336)	0.200 (0.106-0.307)	0.194 (0.110-0.289)	0.183 (0.106-0.268)	0.171 (0.087-0.271)
Nigeria	0.176 (0.084-0.285)	0.162 (0.102-0.230)	0.156 (0.108-0.212)	0.135 (0.096-0.181)	0.110 (0.065-0.165)
Niue	0.007 (0.001-0.024)	0.005 (0.001-0.014)	0.004 (0.001-0.010)	0.002 (0.001-0.006)	0.002 (0.000-0.005)
North Korea	0.142 (0.047-0.280)	0.120 (0.047-0.231)	0.106 (0.041-0.205)	0.102 (0.035-0.203)	0.089 (0.022-0.206)
Norway	0.036 (0.015-0.072)	0.021 (0.011-0.035)	0.014 (0.008-0.023)	0.009 (0.005-0.014)	0.006 (0.002-0.012)

Occupied Palestinian Territory	0.050 (0.013-0.120)	0.038 (0.014-0.077)	0.028 (0.012-0.053)	0.019 (0.008-0.036)	0.015 (0.005-0.034)
Oman	0.116 (0.039-0.235)	0.090 (0.045-0.153)	0.067 (0.038-0.106)	0.046 (0.025-0.074)	0.031 (0.012-0.058)
Pakistan	0.290 (0.171-0.416)	0.264 (0.182-0.355)	0.234 (0.166-0.310)	0.202 (0.136-0.276)	0.170 (0.090-0.263)
Palau	0.009 (0.001-0.030)	0.006 (0.001-0.016)	0.004 (0.001-0.011)	0.003 (0.001-0.008)	0.002 (0.000-0.006)
Panama	0.073 (0.021-0.168)	0.050 (0.018-0.103)	0.037 (0.015-0.074)	0.025 (0.011-0.048)	0.017 (0.006-0.036)
Papua New Guinea	0.033 (0.005-0.097)	0.027 (0.007-0.070)	0.024 (0.007-0.058)	0.022 (0.007-0.049)	0.019 (0.005-0.048)
Paraguay	0.051 (0.010-0.133)	0.039 (0.010-0.093)	0.027 (0.008-0.065)	0.018 (0.005-0.043)	0.013 (0.003-0.034)
Peru	0.039 (0.010-0.102)	0.028 (0.009-0.061)	0.020 (0.008-0.040)	0.013 (0.006-0.023)	0.008 (0.003-0.017)
Philippines	0.194 (0.094-0.312)	0.179 (0.108-0.260)	0.165 (0.104-0.234)	0.144 (0.090-0.207)	0.123 (0.063-0.203)
Poland	0.025 (0.008-0.055)	0.017 (0.009-0.030)	0.014 (0.007-0.022)	0.010 (0.006-0.016)	0.007 (0.003-0.014)
Portugal	0.026 (0.008-0.060)	0.020 (0.009-0.036)	0.013 (0.007-0.021)	0.008 (0.004-0.013)	0.006 (0.002-0.012)
Puerto Rico	0.087 (0.028-0.187)	0.063 (0.025-0.124)	0.042 (0.017-0.082)	0.024 (0.010-0.049)	0.015 (0.005-0.035)
Qatar	0.079 (0.024-0.177)	0.047 (0.016-0.098)	0.032 (0.013-0.061)	0.018 (0.008-0.036)	0.011 (0.004-0.025)
Romania	0.030 (0.007-0.078)	0.018 (0.006-0.039)	0.014 (0.005-0.029)	0.011 (0.004-0.023)	0.007 (0.002-0.018)
Russian Federation	0.036 (0.013-0.073)	0.024 (0.014-0.037)	0.020 (0.014-0.027)	0.016 (0.010-0.023)	0.010 (0.004-0.020)
Rwanda	0.174 (0.060-0.331)	0.163 (0.068-0.284)	0.156 (0.075-0.256)	0.143 (0.076-0.223)	0.129 (0.062-0.214)
Saint Kitts and Nevis	0.097 (0.028-0.204)	0.069 (0.023-0.139)	0.055 (0.020-0.109)	0.036 (0.013-0.075)	0.026 (0.007-0.062)
Saint Lucia	0.115 (0.045-0.214)	0.097 (0.049-0.160)	0.076 (0.038-0.125)	0.049 (0.022-0.089)	0.036 (0.010-0.079)
Saint Vincent and the Grenadines	0.116 (0.035-0.241)	0.097 (0.037-0.183)	0.074 (0.028-0.143)	0.053 (0.019-0.107)	0.033 (0.009-0.080)
Samoa	0.004 (0.001-0.012)	0.003 (0.001-0.007)	0.002 (0.000-0.004)	0.001 (0.000-0.003)	0.001 (0.000-0.002)
Sao Tome and Principe	0.131 (0.047-0.249)	0.124 (0.055-0.213)	0.107 (0.048-0.184)	0.086 (0.041-0.146)	0.066 (0.026-0.126)
Saudi Arabia	0.081 (0.026-0.170)	0.050 (0.026-0.084)	0.035 (0.021-0.052)	0.024 (0.014-0.038)	0.016 (0.007-0.030)
Senegal	0.193 (0.087-0.318)	0.186 (0.102-0.284)	0.173 (0.097-0.261)	0.155 (0.088-0.236)	0.140 (0.068-0.229)
Serbia	0.029 (0.008-0.069)	0.019 (0.008-0.038)	0.014 (0.007-0.027)	0.010 (0.005-0.017)	0.007 (0.003-0.015)
Seychelles	0.140 (0.058-0.242)	0.114 (0.057-0.187)	0.091 (0.048-0.149)	0.067 (0.034-0.114)	0.052 (0.021-0.102)
Sierra Leone	0.169 (0.066-0.301)	0.163 (0.080-0.266)	0.156 (0.084-0.246)	0.143 (0.082-0.217)	0.119 (0.060-0.195)
Singapore	0.136 (0.060-0.237)	0.092 (0.053-0.139)	0.064 (0.038-0.095)	0.050 (0.031-0.075)	0.038 (0.019-0.066)
Slovakia	0.017 (0.004-0.043)	0.011 (0.004-0.023)	0.008 (0.003-0.015)	0.006 (0.002-0.011)	0.004 (0.001-0.009)
Slovenia	0.025 (0.005-0.069)	0.016 (0.004-0.038)	0.011 (0.003-0.027)	0.007 (0.002-0.017)	0.005 (0.001-0.014)
Solomon Islands	0.033 (0.005-0.096)	0.027 (0.006-0.070)	0.025 (0.007-0.058)	0.023 (0.008-0.050)	0.020 (0.006-0.046)
Somalia	0.185 (0.066-0.332)	0.180 (0.080-0.301)	0.175 (0.083-0.289)	0.161 (0.074-0.267)	0.148 (0.060-0.266)
South Africa	0.147 (0.068-0.246)	0.129 (0.078-0.190)	0.112 (0.075-0.156)	0.092 (0.065-0.123)	0.067 (0.040-0.102)
South Korea	0.132 (0.058-0.224)	0.093 (0.057-0.138)	0.063 (0.041-0.090)	0.045 (0.031-0.063)	0.028 (0.015-0.046)
Spain	0.023 (0.008-0.048)	0.013 (0.007-0.021)	0.008 (0.005-0.012)	0.005 (0.003-0.008)	0.004 (0.002-0.008)
Sri Lanka	0.226 (0.111-0.349)	0.214 (0.127-0.303)	0.203 (0.130-0.280)	0.182 (0.118-0.255)	0.159 (0.081-0.255)
Sudan	0.168 (0.064-0.303)	0.158 (0.073-0.263)	0.154 (0.077-0.248)	0.136 (0.067-0.223)	0.118 (0.047-0.212)
Suriname	0.122 (0.040-0.246)	0.100 (0.038-0.193)	0.083 (0.032-0.163)	0.054 (0.019-0.111)	0.037 (0.010-0.086)
Swaziland	0.162 (0.058-0.297)	0.150 (0.067-0.255)	0.136 (0.063-0.225)	0.115 (0.052-0.196)	0.098 (0.037-0.189)
Sweden	0.025 (0.012-0.047)	0.016 (0.010-0.024)	0.011 (0.007-0.017)	0.007 (0.004-0.011)	0.005 (0.002-0.009)
Switzerland	0.026 (0.008-0.059)	0.016 (0.007-0.031)	0.011 (0.006-0.020)	0.009 (0.005-0.015)	0.007 (0.003-0.013)
Syrian Arab Republic	0.088 (0.026-0.197)	0.061 (0.022-0.122)	0.047 (0.019-0.092)	0.032 (0.013-0.064)	0.023 (0.007-0.053)
Taiwan	0.117 (0.048-0.207)	0.090 (0.051-0.142)	0.061 (0.037-0.092)	0.041 (0.024-0.063)	0.032 (0.013-0.062)
Tajikistan	0.069 (0.016-0.171)	0.053 (0.015-0.121)	0.043 (0.012-0.100)	0.038 (0.011-0.089)	0.028 (0.007-0.074)
Tanzania	0.169 (0.069-0.300)	0.165 (0.089-0.257)	0.160 (0.094-0.237)	0.152 (0.095-0.217)	0.133 (0.073-0.206)
Thailand	0.199 (0.098-0.310)	0.182 (0.119-0.251)	0.159 (0.110-0.212)	0.130 (0.089-0.176)	0.105 (0.056-0.169)
Timor-Leste	0.249 (0.122-0.385)	0.252 (0.153-0.358)	0.245 (0.155-0.341)	0.230 (0.147-0.322)	0.215 (0.124-0.319)
Togo	0.164 (0.063-0.293)	0.156 (0.075-0.259)	0.148 (0.077-0.237)	0.134 (0.073-0.213)	0.119 (0.055-0.203)
Tokelau	0.008 (0.001-0.034)	0.006 (0.001-0.019)	0.005 (0.001-0.013)	0.003 (0.001-0.009)	0.003 (0.000-0.008)
Tonga	0.006 (0.001-0.021)	0.004 (0.001-0.012)	0.003 (0.001-0.008)	0.002 (0.001-0.006)	0.002 (0.000-0.005)
Trinidad and Tobago	0.091 (0.019-0.216)	0.069 (0.020-0.148)	0.057 (0.021-0.110)	0.040 (0.013-0.083)	0.027 (0.005-0.074)
Tunisia	0.107 (0.040-0.213)	0.083 (0.041-0.143)	0.063 (0.033-0.105)	0.043 (0.022-0.072)	0.030 (0.012-0.059)
Turkey	0.057 (0.019-0.125)	0.042 (0.022-0.073)	0.030 (0.018-0.045)	0.018 (0.012-0.026)	0.010 (0.005-0.018)
Turkmenistan	0.067 (0.017-0.164)	0.048 (0.015-0.107)	0.035 (0.012-0.079)	0.027 (0.010-0.058)	0.017 (0.005-0.039)
Tuvalu	0.008 (0.001-0.024)	0.006 (0.001-0.016)	0.005 (0.001-0.012)	0.003 (0.001-0.008)	0.002 (0.000-0.007)
Uganda	0.156 (0.055-0.297)	0.148 (0.067-0.253)	0.140 (0.068-0.230)	0.130 (0.069-0.204)	0.115 (0.057-0.191)
Ukraine	0.032 (0.006-0.087)	0.022 (0.006-0.055)	0.018 (0.005-0.044)	0.015 (0.004-0.036)	0.009 (0.002-0.026)
United Arab Emirates	0.064 (0.013-0.165)	0.037 (0.013-0.077)	0.024 (0.011-0.046)	0.017 (0.007-0.033)	0.015 (0.005-0.033)
United Kingdom	0.022 (0.009-0.043)	0.015 (0.010-0.023)	0.012 (0.008-0.016)	0.009 (0.006-0.012)	0.006 (0.004-0.010)
United States of America	0.021 (0.010-0.039)	0.013 (0.007-0.020)	0.009 (0.006-0.014)	0.007 (0.005-0.011)	0.006 (0.003-0.011)
Uruguay	0.040 (0.008-0.112)	0.029 (0.009-0.067)	0.020 (0.007-0.044)	0.012 (0.004-0.025)	0.008 (0.002-0.019)
Uzbekistan	0.066 (0.015-0.164)	0.048 (0.015-0.108)	0.036 (0.012-0.079)	0.028 (0.010-0.061)	0.021 (0.005-0.051)
Vanuatu	0.031 (0.005-0.089)	0.025 (0.006-0.061)	0.022 (0.007-0.048)	0.019 (0.007-0.040)	0.016 (0.005-0.037)
Venezuela	0.052 (0.014-0.132)	0.035 (0.013-0.075)	0.023 (0.010-0.045)	0.014 (0.006-0.026)	0.009 (0.003-0.019)
Viet Nam	0.277 (0.166-0.389)	0.273 (0.205-0.343)	0.260 (0.211-0.313)	0.231 (0.186-0.280)	0.184 (0.119-0.253)
Yemen	0.106 (0.029-0.231)	0.091 (0.035-0.174)	0.078 (0.034-0.144)	0.064 (0.028-0.118)	0.051 (0.018-0.105)
Zambia	0.194 (0.087-0.322)	0.189 (0.101-0.290)	0.178 (0.102-0.268)	0.162 (0.095-0.242)	0.139 (0.067-0.229)
Zimbabwe	0.177 (0.077-0.298)	0.166 (0.092-0.251)	0.155 (0.091-0.225)	0.136 (0.080-0.203)	0.121 (0.058-0.202)

Women, BMI <18.5 kg/m^2

Country	Year				
	1975	1985	1995	2005	2014
Afghanistan	0.302 (0.165-0.441)	0.279 (0.164-0.394)	0.254 (0.149-0.358)	0.228 (0.132-0.330)	0.197 (0.101-0.306)
Albania	0.051 (0.014-0.121)	0.043 (0.016-0.090)	0.040 (0.016-0.078)	0.032 (0.014-0.062)	0.027 (0.010-0.057)
Algeria	0.110 (0.039-0.224)	0.086 (0.038-0.154)	0.067 (0.033-0.115)	0.053 (0.026-0.090)	0.041 (0.018-0.078)
American Samoa	0.011 (0.003-0.026)	0.008 (0.003-0.017)	0.006 (0.002-0.013)	0.005 (0.002-0.011)	0.005 (0.001-0.012)
Andorra	0.031 (0.008-0.076)	0.023 (0.007-0.052)	0.020 (0.007-0.043)	0.017 (0.006-0.038)	0.016 (0.004-0.038)
Angola	0.181 (0.064-0.326)	0.168 (0.072-0.287)	0.155 (0.070-0.262)	0.141 (0.062-0.243)	0.117 (0.044-0.222)
Antigua and Barbuda	0.084 (0.023-0.184)	0.064 (0.021-0.135)	0.045 (0.015-0.094)	0.034 (0.011-0.073)	0.024 (0.006-0.058)
Argentina	0.035 (0.011-0.078)	0.027 (0.012-0.051)	0.022 (0.011-0.039)	0.017 (0.008-0.029)	0.013 (0.005-0.025)
Armenia	0.068 (0.018-0.169)	0.054 (0.020-0.112)	0.048 (0.020-0.089)	0.040 (0.018-0.074)	0.029 (0.011-0.063)
Australia	0.046 (0.025-0.076)	0.034 (0.022-0.049)	0.026 (0.017-0.038)	0.022 (0.014-0.031)	0.018 (0.010-0.030)
Austria	0.059 (0.025-0.110)	0.046 (0.024-0.077)	0.038 (0.021-0.062)	0.033 (0.017-0.055)	0.029 (0.012-0.055)
Azerbaijan	0.071 (0.019-0.161)	0.053 (0.020-0.111)	0.045 (0.018-0.085)	0.037 (0.016-0.070)	0.027 (0.009-0.057)
Bahamas	0.060 (0.016-0.141)	0.047 (0.016-0.100)	0.037 (0.013-0.076)	0.029 (0.010-0.061)	0.025 (0.008-0.056)
Bahrain	0.075 (0.024-0.160)	0.062 (0.028-0.112)	0.050 (0.025-0.087)	0.040 (0.020-0.071)	0.034 (0.014-0.066)
Bangladesh	0.373 (0.261-0.489)	0.357 (0.276-0.443)	0.337 (0.270-0.400)	0.305 (0.243-0.364)	0.265 (0.186-0.343)
Barbados	0.094 (0.035-0.187)	0.072 (0.034-0.126)	0.056 (0.029-0.095)	0.040 (0.019-0.071)	0.029 (0.010-0.061)
Belarus	0.037 (0.008-0.096)	0.030 (0.009-0.069)	0.028 (0.010-0.062)	0.027 (0.009-0.057)	0.023 (0.007-0.053)
Belgium	0.033 (0.014-0.063)	0.027 (0.016-0.041)	0.022 (0.014-0.033)	0.019 (0.011-0.030)	0.019 (0.009-0.034)
Belize	0.086 (0.025-0.189)	0.068 (0.026-0.135)	0.054 (0.023-0.100)	0.039 (0.017-0.073)	0.030 (0.011-0.063)
Benin	0.147 (0.060-0.264)	0.131 (0.069-0.209)	0.122 (0.071-0.184)	0.108 (0.065-0.161)	0.095 (0.051-0.152)
Bermuda	0.042 (0.009-0.111)	0.035 (0.009-0.082)	0.029 (0.008-0.067)	0.026 (0.008-0.057)	0.023 (0.006-0.054)
Bhutan	0.256 (0.129-0.399)	0.230 (0.127-0.344)	0.197 (0.109-0.295)	0.157 (0.085-0.243)	0.120 (0.056-0.202)
Bolivia	0.048 (0.012-0.119)	0.034 (0.012-0.072)	0.024 (0.010-0.048)	0.018 (0.008-0.035)	0.015 (0.005-0.033)
Bosnia and Herzegovina	0.064 (0.014-0.162)	0.054 (0.018-0.113)	0.048 (0.020-0.091)	0.036 (0.017-0.065)	0.030 (0.013-0.057)
Botswana	0.151 (0.050-0.290)	0.124 (0.050-0.226)	0.093 (0.040-0.168)	0.076 (0.034-0.140)	0.063 (0.025-0.125)
Brazil	0.102 (0.045-0.190)	0.076 (0.046-0.119)	0.059 (0.041-0.081)	0.045 (0.032-0.060)	0.034 (0.019-0.053)
Brunei Darussalam	0.163 (0.043-0.339)	0.131 (0.043-0.258)	0.104 (0.040-0.196)	0.082 (0.031-0.159)	0.071 (0.022-0.149)
Bulgaria	0.033 (0.009-0.080)	0.026 (0.009-0.054)	0.025 (0.010-0.049)	0.025 (0.010-0.050)	0.026 (0.009-0.055)
Burkina Faso	0.189 (0.087-0.310)	0.177 (0.103-0.265)	0.166 (0.106-0.237)	0.153 (0.100-0.217)	0.141 (0.082-0.215)
Burundi	0.171 (0.057-0.316)	0.161 (0.065-0.279)	0.151 (0.067-0.251)	0.141 (0.068-0.228)	0.129 (0.057-0.222)
Cabo Verde	0.149 (0.053-0.282)	0.132 (0.059-0.227)	0.110 (0.052-0.189)	0.089 (0.043-0.152)	0.075 (0.031-0.141)
Cambodia	0.265 (0.139-0.402)	0.246 (0.155-0.342)	0.222 (0.144-0.302)	0.193 (0.126-0.264)	0.160 (0.093-0.241)
Cameroon	0.120 (0.048-0.222)	0.098 (0.051-0.159)	0.087 (0.052-0.131)	0.076 (0.047-0.112)	0.064 (0.033-0.107)
Canada	0.043 (0.020-0.079)	0.030 (0.017-0.048)	0.022 (0.013-0.035)	0.017 (0.009-0.027)	0.014 (0.006-0.026)
Central African Republic	0.174 (0.065-0.307)	0.164 (0.076-0.271)	0.162 (0.084-0.257)	0.156 (0.084-0.245)	0.146 (0.067-0.244)
Chad	0.194 (0.087-0.323)	0.182 (0.100-0.278)	0.172 (0.102-0.251)	0.157 (0.094-0.231)	0.142 (0.073-0.228)
Chile	0.034 (0.010-0.083)	0.025 (0.010-0.050)	0.019 (0.009-0.033)	0.013 (0.007-0.023)	0.010 (0.004-0.019)
China	0.146 (0.079-0.230)	0.127 (0.096-0.162)	0.107 (0.088-0.129)	0.081 (0.064-0.101)	0.060 (0.037-0.089)
China (Hong Kong SAR)	0.093 (0.027-0.203)	0.079 (0.033-0.149)	0.072 (0.033-0.126)	0.067 (0.029-0.126)	0.061 (0.019-0.137)
Colombia	0.065 (0.022-0.137)	0.049 (0.024-0.087)	0.040 (0.022-0.064)	0.033 (0.020-0.050)	0.028 (0.014-0.049)
Comoros	0.137 (0.052-0.255)	0.127 (0.061-0.215)	0.115 (0.060-0.189)	0.105 (0.056-0.168)	0.094 (0.045-0.159)
Congo	0.175 (0.084-0.284)	0.158 (0.095-0.230)	0.146 (0.094-0.208)	0.135 (0.084-0.195)	0.122 (0.063-0.199)
Cook Islands	0.026 (0.007-0.067)	0.017 (0.005-0.039)	0.012 (0.004-0.025)	0.008 (0.003-0.018)	0.007 (0.002-0.016)
Costa Rica	0.076 (0.022-0.171)	0.057 (0.023-0.110)	0.041 (0.019-0.073)	0.030 (0.015-0.052)	0.024 (0.010-0.045)
Cote d'Ivoire	0.119 (0.047-0.225)	0.107 (0.054-0.178)	0.100 (0.055-0.156)	0.088 (0.050-0.138)	0.079 (0.040-0.136)
Croatia	0.049 (0.014-0.112)	0.038 (0.014-0.077)	0.034 (0.013-0.066)	0.028 (0.011-0.057)	0.025 (0.008-0.056)
Cuba	0.110 (0.041-0.211)	0.085 (0.041-0.147)	0.071 (0.038-0.117)	0.059 (0.033-0.096)	0.046 (0.022-0.083)
Cyprus	0.048 (0.013-0.114)	0.032 (0.011-0.070)	0.025 (0.009-0.053)	0.020 (0.007-0.042)	0.017 (0.005-0.040)
Czech Republic	0.021 (0.007-0.046)	0.017 (0.008-0.032)	0.018 (0.009-0.031)	0.018 (0.009-0.031)	0.018 (0.007-0.036)
Denmark	0.057 (0.025-0.104)	0.045 (0.025-0.073)	0.038 (0.021-0.061)	0.034 (0.019-0.054)	0.031 (0.015-0.055)
Djibouti	0.131 (0.039-0.274)	0.125 (0.043-0.249)	0.123 (0.049-0.225)	0.124 (0.050-0.223)	0.116 (0.041-0.223)
Dominica	0.097 (0.031-0.204)	0.072 (0.029-0.138)	0.052 (0.022-0.099)	0.039 (0.016-0.075)	0.031 (0.011-0.064)
Dominican Republic	0.107 (0.041-0.204)	0.084 (0.043-0.139)	0.068 (0.038-0.107)	0.051 (0.028-0.085)	0.040 (0.017-0.077)
DR Congo	0.183 (0.075-0.311)	0.171 (0.088-0.271)	0.163 (0.092-0.249)	0.157 (0.094-0.233)	0.143 (0.073-0.228)
Ecuador	0.042 (0.010-0.109)	0.028 (0.009-0.064)	0.021 (0.008-0.043)	0.016 (0.006-0.032)	0.013 (0.004-0.028)
Egypt	0.041 (0.012-0.097)	0.029 (0.013-0.053)	0.021 (0.012-0.034)	0.014 (0.008-0.022)	0.009 (0.004-0.017)
El Salvador	0.072 (0.022-0.164)	0.055 (0.022-0.108)	0.042 (0.019-0.076)	0.031 (0.015-0.056)	0.024 (0.010-0.049)
Equatorial Guinea	0.161 (0.054-0.299)	0.149 (0.064-0.259)	0.136 (0.062-0.235)	0.103 (0.043-0.190)	0.084 (0.027-0.175)
Eritrea	0.237 (0.124-0.363)	0.229 (0.144-0.323)	0.220 (0.146-0.300)	0.207 (0.137-0.284)	0.197 (0.117-0.288)
Estonia	0.032 (0.010-0.070)	0.029 (0.014-0.052)	0.030 (0.017-0.048)	0.027 (0.017-0.041)	0.024 (0.013-0.040)
Ethiopia	0.212 (0.097-0.344)	0.203 (0.115-0.302)	0.196 (0.122-0.280)	0.188 (0.121-0.264)	0.173 (0.099-0.260)
Fiji	0.065 (0.018-0.160)	0.047 (0.017-0.103)	0.035 (0.014-0.069)	0.026 (0.011-0.048)	0.021 (0.008-0.042)
Finland	0.041 (0.019-0.075)	0.029 (0.017-0.045)	0.023 (0.014-0.034)	0.020 (0.013-0.029)	0.019 (0.010-0.032)
France	0.057 (0.024-0.107)	0.047 (0.026-0.074)	0.040 (0.023-0.062)	0.034 (0.020-0.053)	0.031 (0.015-0.055)
French Polynesia	0.031 (0.008-0.080)	0.022 (0.007-0.049)	0.016 (0.005-0.035)	0.012 (0.004-0.026)	0.010 (0.003-0.022)
Gabon	0.125 (0.039-0.250)	0.097 (0.038-0.183)	0.083 (0.037-0.150)	0.075 (0.035-0.130)	0.070 (0.030-0.132)

Gambia	0.168 (0.071-0.291)	0.153 (0.081-0.243)	0.138 (0.079-0.209)	0.121 (0.071-0.183)	0.111 (0.058-0.183)
Georgia	0.074 (0.020-0.176)	0.055 (0.019-0.119)	0.049 (0.019-0.100)	0.041 (0.017-0.083)	0.035 (0.013-0.074)
Germany	0.039 (0.020-0.067)	0.030 (0.020-0.042)	0.025 (0.017-0.034)	0.021 (0.014-0.031)	0.020 (0.010-0.033)
Ghana	0.123 (0.052-0.220)	0.112 (0.063-0.171)	0.098 (0.062-0.140)	0.080 (0.051-0.117)	0.066 (0.034-0.109)
Greece	0.033 (0.011-0.076)	0.024 (0.010-0.049)	0.019 (0.008-0.038)	0.016 (0.006-0.032)	0.015 (0.005-0.033)
Greenland	0.052 (0.017-0.109)	0.038 (0.015-0.075)	0.031 (0.013-0.060)	0.026 (0.011-0.050)	0.024 (0.009-0.049)
Grenada	0.099 (0.032-0.208)	0.078 (0.030-0.152)	0.058 (0.024-0.112)	0.041 (0.017-0.080)	0.029 (0.010-0.062)
Guatemala	0.067 (0.021-0.151)	0.051 (0.020-0.099)	0.038 (0.018-0.068)	0.028 (0.014-0.050)	0.022 (0.009-0.046)
Guinea	0.153 (0.058-0.277)	0.138 (0.067-0.225)	0.131 (0.071-0.200)	0.120 (0.070-0.182)	0.112 (0.059-0.178)
Guinea Bissau	0.155 (0.057-0.283)	0.143 (0.065-0.240)	0.126 (0.060-0.214)	0.120 (0.060-0.199)	0.112 (0.050-0.196)
Guyana	0.116 (0.039-0.231)	0.093 (0.037-0.176)	0.075 (0.032-0.140)	0.053 (0.022-0.101)	0.041 (0.014-0.087)
Haiti	0.162 (0.064-0.292)	0.140 (0.069-0.235)	0.121 (0.063-0.195)	0.099 (0.052-0.163)	0.082 (0.035-0.155)
Honduras	0.087 (0.027-0.187)	0.067 (0.027-0.128)	0.051 (0.024-0.090)	0.038 (0.019-0.067)	0.030 (0.013-0.058)
Hungary	0.040 (0.016-0.081)	0.034 (0.017-0.059)	0.033 (0.017-0.057)	0.033 (0.015-0.060)	0.033 (0.012-0.067)
Iceland	0.035 (0.014-0.072)	0.025 (0.012-0.045)	0.022 (0.011-0.038)	0.020 (0.009-0.036)	0.018 (0.007-0.037)
India	0.374 (0.283-0.471)	0.349 (0.289-0.412)	0.322 (0.280-0.363)	0.287 (0.250-0.325)	0.249 (0.191-0.311)
Indonesia	0.262 (0.161-0.373)	0.236 (0.168-0.309)	0.202 (0.150-0.257)	0.168 (0.123-0.218)	0.136 (0.084-0.198)
Iran	0.096 (0.039-0.183)	0.072 (0.041-0.113)	0.056 (0.037-0.078)	0.042 (0.030-0.055)	0.031 (0.018-0.048)
Iraq	0.069 (0.020-0.156)	0.053 (0.020-0.107)	0.047 (0.019-0.093)	0.040 (0.016-0.079)	0.033 (0.012-0.071)
Ireland	0.050 (0.019-0.103)	0.034 (0.015-0.062)	0.024 (0.011-0.043)	0.017 (0.008-0.030)	0.014 (0.006-0.027)
Israel	0.028 (0.009-0.062)	0.023 (0.010-0.043)	0.019 (0.009-0.034)	0.016 (0.007-0.029)	0.015 (0.005-0.030)
Italy	0.043 (0.020-0.077)	0.035 (0.022-0.051)	0.029 (0.020-0.041)	0.023 (0.015-0.035)	0.020 (0.010-0.034)
Jamaica	0.098 (0.040-0.187)	0.076 (0.040-0.123)	0.060 (0.035-0.091)	0.044 (0.025-0.069)	0.034 (0.015-0.063)
Japan	0.108 (0.077-0.145)	0.099 (0.081-0.120)	0.094 (0.078-0.112)	0.094 (0.076-0.115)	0.100 (0.068-0.140)
Jordan	0.045 (0.014-0.104)	0.030 (0.014-0.056)	0.021 (0.011-0.035)	0.015 (0.008-0.025)	0.012 (0.006-0.022)
Kazakhstan	0.082 (0.024-0.187)	0.062 (0.023-0.129)	0.050 (0.020-0.097)	0.039 (0.016-0.077)	0.028 (0.009-0.063)
Kenya	0.171 (0.075-0.289)	0.152 (0.082-0.236)	0.133 (0.077-0.204)	0.116 (0.066-0.180)	0.099 (0.047-0.172)
Kiribati	0.062 (0.022-0.132)	0.042 (0.017-0.084)	0.029 (0.011-0.060)	0.019 (0.007-0.040)	0.015 (0.004-0.036)
Kuwait	0.031 (0.008-0.080)	0.024 (0.010-0.046)	0.021 (0.010-0.035)	0.016 (0.008-0.027)	0.013 (0.006-0.025)
Kyrgyzstan	0.092 (0.027-0.206)	0.072 (0.029-0.141)	0.059 (0.027-0.107)	0.048 (0.023-0.085)	0.038 (0.016-0.074)
Lao PDR	0.251 (0.132-0.380)	0.229 (0.137-0.327)	0.204 (0.125-0.289)	0.172 (0.106-0.248)	0.140 (0.076-0.218)
Latvia	0.033 (0.008-0.081)	0.028 (0.010-0.060)	0.028 (0.011-0.057)	0.026 (0.011-0.050)	0.023 (0.008-0.048)
Lebanon	0.066 (0.022-0.143)	0.048 (0.020-0.093)	0.033 (0.015-0.062)	0.023 (0.010-0.046)	0.022 (0.009-0.043)
Lesotho	0.122 (0.038-0.250)	0.102 (0.042-0.186)	0.084 (0.040-0.147)	0.069 (0.034-0.117)	0.057 (0.025-0.105)
Liberia	0.129 (0.047-0.242)	0.114 (0.053-0.199)	0.111 (0.050-0.194)	0.101 (0.052-0.164)	0.090 (0.043-0.153)
Libya	0.056 (0.014-0.142)	0.035 (0.013-0.074)	0.028 (0.012-0.054)	0.023 (0.010-0.044)	0.020 (0.007-0.040)
Lithuania	0.026 (0.007-0.061)	0.020 (0.008-0.040)	0.019 (0.009-0.036)	0.018 (0.008-0.033)	0.015 (0.006-0.033)
Luxembourg	0.045 (0.015-0.100)	0.035 (0.013-0.073)	0.027 (0.010-0.058)	0.023 (0.009-0.046)	0.022 (0.008-0.046)
Macedonia (TFYR)	0.045 (0.012-0.109)	0.037 (0.013-0.078)	0.035 (0.013-0.069)	0.032 (0.012-0.065)	0.029 (0.010-0.065)
Madagascar	0.212 (0.103-0.335)	0.198 (0.119-0.287)	0.186 (0.120-0.262)	0.177 (0.115-0.249)	0.165 (0.093-0.251)
Malawi	0.159 (0.065-0.282)	0.145 (0.076-0.228)	0.134 (0.080-0.201)	0.120 (0.073-0.179)	0.105 (0.054-0.174)
Malaysia	0.215 (0.112-0.335)	0.175 (0.107-0.256)	0.136 (0.087-0.193)	0.106 (0.071-0.147)	0.086 (0.049-0.135)
Maldives	0.206 (0.097-0.335)	0.178 (0.099-0.270)	0.148 (0.085-0.224)	0.113 (0.062-0.179)	0.089 (0.043-0.153)
Mali	0.164 (0.071-0.283)	0.152 (0.085-0.237)	0.143 (0.086-0.212)	0.130 (0.079-0.189)	0.118 (0.064-0.187)
Malta	0.031 (0.008-0.078)	0.024 (0.009-0.050)	0.020 (0.008-0.041)	0.016 (0.006-0.035)	0.015 (0.004-0.035)
Marshall Islands	0.030 (0.008-0.078)	0.021 (0.007-0.048)	0.015 (0.006-0.033)	0.012 (0.004-0.026)	0.011 (0.003-0.026)
Mauritania	0.153 (0.060-0.280)	0.135 (0.065-0.228)	0.122 (0.061-0.202)	0.110 (0.056-0.182)	0.098 (0.044-0.176)
Mauritius	0.125 (0.052-0.223)	0.108 (0.058-0.170)	0.089 (0.050-0.138)	0.070 (0.038-0.112)	0.056 (0.024-0.104)
Mexico	0.057 (0.020-0.123)	0.040 (0.020-0.072)	0.030 (0.018-0.047)	0.022 (0.014-0.033)	0.017 (0.009-0.030)
Micronesia (Federated States of)	0.052 (0.015-0.121)	0.036 (0.014-0.071)	0.025 (0.011-0.049)	0.018 (0.008-0.033)	0.013 (0.005-0.027)
Moldova	0.043 (0.011-0.107)	0.035 (0.013-0.076)	0.034 (0.014-0.069)	0.035 (0.015-0.068)	0.030 (0.012-0.062)
Mongolia	0.077 (0.022-0.178)	0.060 (0.024-0.120)	0.050 (0.024-0.088)	0.042 (0.022-0.070)	0.034 (0.016-0.062)
Montenegro	0.052 (0.012-0.132)	0.038 (0.011-0.087)	0.033 (0.011-0.070)	0.029 (0.010-0.062)	0.026 (0.008-0.061)
Morocco	0.099 (0.033-0.208)	0.077 (0.034-0.140)	0.060 (0.030-0.104)	0.047 (0.023-0.083)	0.037 (0.015-0.072)
Mozambique	0.161 (0.060-0.293)	0.149 (0.072-0.245)	0.138 (0.076-0.214)	0.129 (0.074-0.195)	0.118 (0.058-0.194)
Myanmar	0.255 (0.116-0.411)	0.236 (0.134-0.345)	0.213 (0.132-0.297)	0.185 (0.121-0.257)	0.148 (0.086-0.223)
Namibia	0.157 (0.056-0.293)	0.139 (0.067-0.227)	0.124 (0.069-0.193)	0.104 (0.061-0.159)	0.087 (0.045-0.145)
Nauru	0.007 (0.002-0.018)	0.006 (0.002-0.012)	0.005 (0.002-0.011)	0.006 (0.002-0.012)	0.006 (0.001-0.014)
Nepal	0.316 (0.194-0.440)	0.296 (0.202-0.391)	0.268 (0.191-0.345)	0.231 (0.165-0.298)	0.190 (0.121-0.269)
Netherlands	0.050 (0.021-0.095)	0.038 (0.020-0.062)	0.030 (0.017-0.047)	0.024 (0.014-0.038)	0.022 (0.011-0.039)
New Zealand	0.047 (0.021-0.089)	0.034 (0.019-0.055)	0.025 (0.015-0.039)	0.019 (0.012-0.029)	0.016 (0.009-0.027)
Nicaragua	0.067 (0.021-0.148)	0.052 (0.022-0.101)	0.044 (0.021-0.079)	0.034 (0.017-0.061)	0.028 (0.012-0.057)
Niger	0.189 (0.087-0.311)	0.177 (0.102-0.267)	0.169 (0.104-0.242)	0.158 (0.099-0.226)	0.144 (0.079-0.223)
Nigeria	0.169 (0.080-0.281)	0.152 (0.095-0.219)	0.138 (0.095-0.187)	0.117 (0.082-0.158)	0.094 (0.057-0.139)
Niue	0.039 (0.010-0.102)	0.023 (0.007-0.054)	0.015 (0.005-0.034)	0.010 (0.003-0.022)	0.007 (0.002-0.016)
North Korea	0.140 (0.047-0.277)	0.120 (0.048-0.225)	0.110 (0.045-0.206)	0.111 (0.041-0.212)	0.108 (0.032-0.228)
Norway	0.066 (0.030-0.120)	0.045 (0.026-0.071)	0.034 (0.021-0.053)	0.028 (0.016-0.043)	0.025 (0.012-0.044)
Occupied Palestinian Territory	0.038 (0.011-0.091)	0.028 (0.012-0.057)	0.023 (0.010-0.042)	0.018 (0.008-0.033)	0.016 (0.006-0.033)
Oman	0.139 (0.054-0.262)	0.097 (0.051-0.162)	0.070 (0.041-0.111)	0.055 (0.032-0.088)	0.045 (0.021-0.082)

Pakistan	0.287 (0.177-0.403)	0.259 (0.180-0.346)	0.227 (0.163-0.296)	0.194 (0.134-0.259)	0.163 (0.097-0.242)
Palau	0.026 (0.006-0.068)	0.019 (0.006-0.043)	0.014 (0.005-0.031)	0.011 (0.004-0.025)	0.010 (0.003-0.023)
Panama	0.069 (0.020-0.161)	0.051 (0.020-0.103)	0.040 (0.018-0.074)	0.030 (0.014-0.055)	0.024 (0.010-0.046)
Papua New Guinea	0.099 (0.027-0.226)	0.077 (0.027-0.162)	0.060 (0.023-0.121)	0.046 (0.018-0.092)	0.035 (0.011-0.078)
Paraguay	0.079 (0.020-0.185)	0.059 (0.018-0.131)	0.044 (0.015-0.097)	0.032 (0.011-0.070)	0.024 (0.007-0.055)
Peru	0.036 (0.010-0.089)	0.026 (0.011-0.053)	0.021 (0.011-0.035)	0.016 (0.009-0.024)	0.012 (0.006-0.020)
Philippines	0.252 (0.160-0.350)	0.219 (0.165-0.278)	0.188 (0.143-0.237)	0.164 (0.121-0.214)	0.143 (0.084-0.212)
Poland	0.033 (0.012-0.065)	0.028 (0.015-0.046)	0.027 (0.015-0.042)	0.025 (0.014-0.039)	0.023 (0.011-0.041)
Portugal	0.054 (0.017-0.116)	0.043 (0.019-0.080)	0.032 (0.016-0.055)	0.023 (0.012-0.040)	0.019 (0.008-0.038)
Puerto Rico	0.079 (0.026-0.166)	0.055 (0.023-0.104)	0.039 (0.018-0.073)	0.031 (0.015-0.055)	0.027 (0.011-0.052)
Qatar	0.050 (0.015-0.121)	0.034 (0.012-0.072)	0.028 (0.012-0.054)	0.021 (0.009-0.040)	0.018 (0.007-0.036)
Romania	0.049 (0.015-0.109)	0.036 (0.015-0.072)	0.033 (0.015-0.062)	0.031 (0.014-0.059)	0.028 (0.010-0.059)
Russian Federation	0.030 (0.012-0.059)	0.026 (0.016-0.039)	0.027 (0.019-0.037)	0.027 (0.018-0.039)	0.025 (0.012-0.044)
Rwanda	0.136 (0.044-0.263)	0.123 (0.049-0.218)	0.114 (0.054-0.188)	0.102 (0.053-0.165)	0.090 (0.043-0.153)
Saint Kitts and Nevis	0.088 (0.026-0.191)	0.064 (0.024-0.131)	0.048 (0.019-0.093)	0.034 (0.013-0.069)	0.025 (0.007-0.058)
Saint Lucia	0.100 (0.038-0.192)	0.078 (0.038-0.136)	0.060 (0.030-0.101)	0.042 (0.020-0.076)	0.030 (0.011-0.064)
Saint Vincent and the Grenadines	0.110 (0.033-0.229)	0.086 (0.031-0.170)	0.065 (0.025-0.125)	0.047 (0.018-0.094)	0.034 (0.010-0.075)
Samoa	0.024 (0.007-0.060)	0.015 (0.005-0.032)	0.010 (0.004-0.020)	0.006 (0.002-0.012)	0.004 (0.001-0.009)
Sao Tome and Principe	0.135 (0.052-0.253)	0.122 (0.057-0.210)	0.105 (0.052-0.179)	0.089 (0.046-0.150)	0.076 (0.034-0.137)
Saudi Arabia	0.069 (0.023-0.146)	0.043 (0.023-0.072)	0.033 (0.020-0.050)	0.027 (0.016-0.041)	0.022 (0.010-0.038)
Senegal	0.161 (0.074-0.272)	0.154 (0.089-0.233)	0.145 (0.088-0.211)	0.132 (0.080-0.195)	0.119 (0.061-0.191)
Serbia	0.049 (0.017-0.104)	0.040 (0.019-0.070)	0.037 (0.020-0.060)	0.034 (0.019-0.056)	0.032 (0.015-0.058)
Seychelles	0.102 (0.038-0.196)	0.079 (0.037-0.138)	0.063 (0.031-0.106)	0.047 (0.023-0.080)	0.038 (0.015-0.072)
Sierra Leone	0.147 (0.058-0.270)	0.137 (0.066-0.227)	0.131 (0.069-0.207)	0.122 (0.068-0.186)	0.106 (0.054-0.171)
Singapore	0.113 (0.051-0.202)	0.097 (0.058-0.146)	0.088 (0.055-0.128)	0.084 (0.054-0.121)	0.079 (0.045-0.124)
Slovakia	0.048 (0.016-0.104)	0.037 (0.017-0.068)	0.032 (0.017-0.055)	0.030 (0.017-0.050)	0.028 (0.013-0.052)
Slovenia	0.052 (0.014-0.123)	0.039 (0.014-0.083)	0.034 (0.013-0.068)	0.028 (0.011-0.058)	0.026 (0.009-0.058)
Solomon Islands	0.079 (0.018-0.196)	0.058 (0.018-0.133)	0.043 (0.016-0.091)	0.033 (0.013-0.066)	0.026 (0.009-0.056)
Somalia	0.164 (0.059-0.305)	0.157 (0.068-0.271)	0.152 (0.071-0.256)	0.147 (0.069-0.250)	0.141 (0.059-0.253)
South Africa	0.067 (0.023-0.137)	0.056 (0.028-0.093)	0.047 (0.028-0.070)	0.039 (0.026-0.056)	0.033 (0.019-0.050)
South Korea	0.135 (0.064-0.231)	0.098 (0.061-0.144)	0.074 (0.049-0.104)	0.064 (0.045-0.086)	0.052 (0.032-0.079)
Spain	0.032 (0.014-0.062)	0.024 (0.013-0.039)	0.019 (0.011-0.029)	0.015 (0.009-0.025)	0.014 (0.006-0.025)
Sri Lanka	0.243 (0.129-0.372)	0.214 (0.132-0.304)	0.186 (0.120-0.259)	0.151 (0.097-0.216)	0.119 (0.060-0.195)
Sudan	0.144 (0.052-0.269)	0.129 (0.058-0.224)	0.117 (0.055-0.200)	0.103 (0.048-0.178)	0.090 (0.037-0.166)
Suriname	0.091 (0.026-0.195)	0.068 (0.024-0.137)	0.058 (0.022-0.114)	0.045 (0.017-0.090)	0.036 (0.011-0.078)
Swaziland	0.121 (0.034-0.256)	0.098 (0.035-0.189)	0.078 (0.030-0.148)	0.061 (0.024-0.116)	0.048 (0.016-0.102)
Sweden	0.044 (0.019-0.084)	0.035 (0.021-0.054)	0.030 (0.019-0.043)	0.025 (0.015-0.038)	0.022 (0.011-0.038)
Switzerland	0.071 (0.028-0.137)	0.052 (0.026-0.087)	0.040 (0.022-0.066)	0.037 (0.020-0.061)	0.035 (0.016-0.063)
Syrian Arab Republic	0.079 (0.025-0.176)	0.056 (0.022-0.112)	0.046 (0.020-0.086)	0.034 (0.014-0.065)	0.027 (0.009-0.057)
Taiwan	0.156 (0.073-0.256)	0.117 (0.068-0.175)	0.086 (0.054-0.126)	0.071 (0.043-0.105)	0.067 (0.032-0.119)
Tajikistan	0.098 (0.028-0.216)	0.080 (0.030-0.163)	0.069 (0.029-0.134)	0.061 (0.027-0.116)	0.048 (0.018-0.099)
Tanzania	0.163 (0.070-0.278)	0.147 (0.081-0.222)	0.135 (0.085-0.192)	0.124 (0.082-0.175)	0.109 (0.064-0.167)
Thailand	0.210 (0.110-0.325)	0.177 (0.111-0.252)	0.144 (0.096-0.198)	0.112 (0.074-0.156)	0.091 (0.049-0.147)
Timor-Leste	0.272 (0.149-0.396)	0.258 (0.160-0.358)	0.239 (0.151-0.328)	0.218 (0.138-0.300)	0.195 (0.110-0.288)
Togo	0.146 (0.057-0.262)	0.134 (0.067-0.219)	0.123 (0.066-0.195)	0.110 (0.061-0.171)	0.097 (0.050-0.162)
Tokelau	0.043 (0.007-0.131)	0.026 (0.006-0.067)	0.016 (0.005-0.038)	0.010 (0.003-0.023)	0.007 (0.001-0.017)
Tonga	0.019 (0.004-0.052)	0.011 (0.003-0.027)	0.007 (0.002-0.016)	0.005 (0.001-0.010)	0.003 (0.001-0.008)
Trinidad and Tobago	0.096 (0.025-0.218)	0.070 (0.023-0.146)	0.053 (0.021-0.101)	0.035 (0.013-0.070)	0.023 (0.006-0.058)
Tunisia	0.094 (0.033-0.193)	0.069 (0.032-0.121)	0.052 (0.027-0.087)	0.040 (0.021-0.066)	0.031 (0.014-0.058)
Turkey	0.058 (0.022-0.122)	0.041 (0.023-0.068)	0.029 (0.019-0.043)	0.021 (0.014-0.031)	0.016 (0.009-0.027)
Turkmenistan	0.088 (0.025-0.199)	0.071 (0.027-0.145)	0.057 (0.024-0.109)	0.047 (0.021-0.088)	0.035 (0.014-0.072)
Tuvalu	0.033 (0.009-0.081)	0.022 (0.007-0.047)	0.014 (0.005-0.031)	0.010 (0.003-0.022)	0.008 (0.002-0.019)
Uganda	0.157 (0.061-0.277)	0.142 (0.068-0.232)	0.128 (0.069-0.203)	0.116 (0.064-0.180)	0.104 (0.054-0.170)
Ukraine	0.030 (0.007-0.080)	0.026 (0.008-0.059)	0.026 (0.009-0.056)	0.027 (0.009-0.059)	0.024 (0.007-0.057)
United Arab Emirates	0.054 (0.013-0.135)	0.036 (0.014-0.072)	0.027 (0.013-0.050)	0.022 (0.010-0.040)	0.021 (0.008-0.041)
United Kingdom	0.046 (0.023-0.082)	0.034 (0.023-0.048)	0.026 (0.019-0.034)	0.021 (0.015-0.027)	0.018 (0.011-0.026)
United States of America	0.049 (0.027-0.080)	0.035 (0.023-0.051)	0.026 (0.018-0.038)	0.021 (0.014-0.030)	0.019 (0.011-0.030)
Uruguay	0.042 (0.011-0.106)	0.033 (0.012-0.072)	0.027 (0.011-0.053)	0.020 (0.009-0.039)	0.015 (0.005-0.032)
Uzbekistan	0.092 (0.026-0.212)	0.072 (0.027-0.149)	0.059 (0.025-0.114)	0.049 (0.021-0.096)	0.038 (0.013-0.082)
Vanuatu	0.084 (0.021-0.196)	0.061 (0.022-0.127)	0.045 (0.019-0.084)	0.034 (0.015-0.062)	0.026 (0.010-0.054)
Venezuela	0.036 (0.010-0.087)	0.027 (0.010-0.054)	0.021 (0.009-0.039)	0.017 (0.008-0.030)	0.014 (0.005-0.028)
Viet Nam	0.310 (0.200-0.418)	0.293 (0.222-0.365)	0.268 (0.214-0.321)	0.229 (0.185-0.276)	0.186 (0.126-0.253)
Yemen	0.168 (0.061-0.310)	0.143 (0.067-0.241)	0.120 (0.063-0.197)	0.098 (0.052-0.162)	0.083 (0.039-0.148)
Zambia	0.142 (0.058-0.256)	0.126 (0.067-0.201)	0.119 (0.070-0.178)	0.108 (0.066-0.160)	0.094 (0.051-0.153)
Zimbabwe	0.105 (0.036-0.209)	0.085 (0.040-0.148)	0.071 (0.039-0.116)	0.062 (0.034-0.100)	0.056 (0.026-0.105)

Men, 18.5 ≤ BMI < 20 kg/m^2

Country	Year				
	1975	1985	1995	2005	2014
Afghanistan	0.215 (0.113-0.335)	0.204 (0.123-0.298)	0.196 (0.124-0.278)	0.190 (0.122-0.265)	0.176 (0.102-0.259)
Albania	0.095 (0.042-0.178)	0.076 (0.040-0.125)	0.061 (0.034-0.096)	0.041 (0.024-0.064)	0.026 (0.013-0.047)
Algeria	0.132 (0.067-0.224)	0.107 (0.065-0.165)	0.086 (0.054-0.128)	0.071 (0.045-0.106)	0.055 (0.031-0.092)
American Samoa	0.016 (0.006-0.030)	0.011 (0.005-0.020)	0.008 (0.004-0.015)	0.007 (0.003-0.012)	0.006 (0.002-0.012)
Andorra	0.053 (0.023-0.098)	0.035 (0.018-0.059)	0.025 (0.014-0.043)	0.017 (0.010-0.029)	0.014 (0.007-0.027)
Angola	0.250 (0.130-0.380)	0.232 (0.140-0.339)	0.214 (0.133-0.309)	0.199 (0.121-0.289)	0.170 (0.089-0.276)
Antigua and Barbuda	0.125 (0.059-0.217)	0.106 (0.059-0.168)	0.080 (0.044-0.128)	0.065 (0.034-0.105)	0.047 (0.020-0.089)
Argentina	0.057 (0.025-0.105)	0.044 (0.024-0.074)	0.036 (0.021-0.056)	0.027 (0.016-0.042)	0.021 (0.011-0.035)
Armenia	0.089 (0.033-0.181)	0.073 (0.034-0.135)	0.066 (0.033-0.116)	0.054 (0.028-0.090)	0.035 (0.016-0.066)
Australia	0.054 (0.032-0.085)	0.039 (0.028-0.054)	0.030 (0.021-0.041)	0.022 (0.016-0.030)	0.016 (0.010-0.025)
Austria	0.074 (0.039-0.123)	0.052 (0.032-0.078)	0.037 (0.024-0.055)	0.028 (0.018-0.042)	0.021 (0.011-0.037)
Azerbaijan	0.095 (0.041-0.178)	0.075 (0.038-0.130)	0.064 (0.033-0.108)	0.054 (0.028-0.088)	0.034 (0.015-0.063)
Bahamas	0.099 (0.046-0.173)	0.079 (0.042-0.127)	0.059 (0.032-0.095)	0.044 (0.024-0.071)	0.035 (0.016-0.062)
Bahrain	0.088 (0.039-0.159)	0.067 (0.039-0.107)	0.051 (0.032-0.079)	0.038 (0.024-0.058)	0.029 (0.015-0.051)
Bangladesh	0.219 (0.125-0.331)	0.210 (0.142-0.286)	0.200 (0.145-0.261)	0.187 (0.138-0.243)	0.170 (0.110-0.241)
Barbados	0.128 (0.064-0.212)	0.103 (0.062-0.156)	0.087 (0.055-0.128)	0.067 (0.041-0.101)	0.051 (0.024-0.090)
Belarus	0.086 (0.039-0.156)	0.063 (0.033-0.105)	0.050 (0.027-0.082)	0.038 (0.021-0.062)	0.024 (0.011-0.045)
Belgium	0.053 (0.027-0.090)	0.037 (0.025-0.054)	0.026 (0.018-0.036)	0.018 (0.012-0.026)	0.014 (0.008-0.023)
Belize	0.132 (0.061-0.234)	0.116 (0.066-0.182)	0.102 (0.061-0.152)	0.081 (0.049-0.122)	0.068 (0.034-0.114)
Benin	0.222 (0.121-0.346)	0.206 (0.131-0.291)	0.194 (0.131-0.263)	0.181 (0.126-0.240)	0.167 (0.106-0.237)
Bermuda	0.068 (0.027-0.132)	0.052 (0.025-0.092)	0.039 (0.020-0.070)	0.032 (0.017-0.055)	0.023 (0.010-0.044)
Bhutan	0.228 (0.124-0.355)	0.215 (0.136-0.307)	0.195 (0.129-0.268)	0.169 (0.112-0.232)	0.140 (0.084-0.211)
Bolivia	0.107 (0.044-0.203)	0.086 (0.042-0.152)	0.071 (0.036-0.121)	0.056 (0.029-0.095)	0.045 (0.021-0.081)
Bosnia and Herzegovina	0.097 (0.034-0.210)	0.081 (0.037-0.149)	0.067 (0.035-0.113)	0.041 (0.023-0.066)	0.027 (0.014-0.046)
Botswana	0.238 (0.132-0.359)	0.215 (0.137-0.304)	0.174 (0.109-0.248)	0.148 (0.093-0.216)	0.120 (0.066-0.188)
Brazil	0.123 (0.066-0.203)	0.096 (0.063-0.139)	0.075 (0.053-0.101)	0.056 (0.040-0.074)	0.041 (0.024-0.063)
Brunei Darussalam	0.190 (0.076-0.336)	0.156 (0.075-0.261)	0.123 (0.067-0.194)	0.093 (0.052-0.146)	0.074 (0.035-0.131)
Bulgaria	0.068 (0.031-0.127)	0.045 (0.024-0.075)	0.034 (0.019-0.055)	0.028 (0.016-0.045)	0.020 (0.010-0.037)
Burkina Faso	0.239 (0.128-0.369)	0.228 (0.149-0.320)	0.215 (0.149-0.286)	0.196 (0.140-0.261)	0.182 (0.115-0.259)
Burundi	0.285 (0.151-0.432)	0.270 (0.163-0.388)	0.252 (0.159-0.352)	0.240 (0.156-0.332)	0.227 (0.138-0.328)
Cabo Verde	0.225 (0.118-0.354)	0.207 (0.131-0.292)	0.182 (0.120-0.252)	0.146 (0.097-0.205)	0.121 (0.068-0.187)
Cambodia	0.257 (0.142-0.384)	0.252 (0.171-0.338)	0.239 (0.168-0.313)	0.219 (0.156-0.288)	0.191 (0.122-0.272)
Cameroon	0.220 (0.125-0.334)	0.200 (0.135-0.278)	0.183 (0.128-0.243)	0.168 (0.123-0.221)	0.151 (0.094-0.217)
Canada	0.049 (0.027-0.082)	0.035 (0.022-0.050)	0.026 (0.016-0.037)	0.018 (0.011-0.026)	0.014 (0.008-0.023)
Central African Republic	0.231 (0.116-0.367)	0.222 (0.126-0.334)	0.217 (0.135-0.313)	0.212 (0.136-0.299)	0.202 (0.117-0.303)
Chad	0.228 (0.128-0.352)	0.215 (0.137-0.306)	0.201 (0.137-0.276)	0.185 (0.128-0.252)	0.172 (0.105-0.251)
Chile	0.068 (0.029-0.132)	0.053 (0.028-0.086)	0.040 (0.024-0.061)	0.028 (0.017-0.042)	0.020 (0.011-0.035)
China	0.207 (0.128-0.301)	0.193 (0.154-0.235)	0.164 (0.139-0.191)	0.117 (0.095-0.141)	0.076 (0.050-0.108)
China (Hong Kong SAR)	0.120 (0.048-0.218)	0.100 (0.053-0.164)	0.079 (0.046-0.126)	0.062 (0.034-0.101)	0.043 (0.018-0.085)
Colombia	0.101 (0.048-0.179)	0.081 (0.047-0.126)	0.066 (0.042-0.098)	0.053 (0.035-0.076)	0.042 (0.024-0.068)
Comoros	0.217 (0.117-0.334)	0.207 (0.129-0.295)	0.195 (0.127-0.271)	0.186 (0.124-0.254)	0.175 (0.109-0.252)
Congo	0.230 (0.132-0.342)	0.211 (0.136-0.298)	0.193 (0.126-0.270)	0.179 (0.117-0.251)	0.158 (0.088-0.244)
Cook Islands	0.020 (0.007-0.043)	0.016 (0.007-0.028)	0.011 (0.005-0.020)	0.008 (0.003-0.014)	0.006 (0.002-0.011)
Costa Rica	0.107 (0.050-0.191)	0.082 (0.046-0.133)	0.062 (0.037-0.097)	0.046 (0.028-0.068)	0.034 (0.018-0.057)
Cote d'Ivoire	0.208 (0.113-0.318)	0.190 (0.121-0.271)	0.175 (0.118-0.243)	0.159 (0.109-0.215)	0.142 (0.085-0.209)
Croatia	0.087 (0.039-0.162)	0.061 (0.033-0.102)	0.046 (0.026-0.074)	0.030 (0.017-0.049)	0.018 (0.009-0.034)
Cuba	0.120 (0.061-0.200)	0.097 (0.058-0.146)	0.083 (0.052-0.124)	0.076 (0.048-0.112)	0.058 (0.031-0.095)
Cyprus	0.081 (0.037-0.148)	0.056 (0.030-0.092)	0.036 (0.020-0.059)	0.025 (0.014-0.040)	0.019 (0.009-0.036)
Czech Republic	0.043 (0.021-0.079)	0.030 (0.018-0.048)	0.024 (0.014-0.036)	0.018 (0.011-0.027)	0.013 (0.006-0.023)
Denmark	0.070 (0.042-0.109)	0.048 (0.032-0.067)	0.033 (0.022-0.047)	0.024 (0.015-0.035)	0.019 (0.010-0.031)
Djibouti	0.179 (0.083-0.303)	0.167 (0.084-0.275)	0.159 (0.091-0.244)	0.155 (0.092-0.235)	0.144 (0.074-0.235)
Dominica	0.138 (0.067-0.232)	0.114 (0.067-0.177)	0.090 (0.054-0.138)	0.069 (0.041-0.107)	0.054 (0.027-0.094)
Dominican Republic	0.152 (0.079-0.250)	0.128 (0.078-0.189)	0.108 (0.069-0.155)	0.080 (0.051-0.116)	0.060 (0.031-0.098)
DR Congo	0.237 (0.128-0.359)	0.223 (0.134-0.326)	0.213 (0.134-0.307)	0.212 (0.137-0.297)	0.201 (0.113-0.302)
Ecuador	0.106 (0.044-0.200)	0.079 (0.037-0.137)	0.062 (0.031-0.103)	0.048 (0.026-0.078)	0.036 (0.017-0.064)
Egypt	0.104 (0.050-0.188)	0.083 (0.051-0.126)	0.068 (0.044-0.097)	0.053 (0.035-0.075)	0.040 (0.022-0.063)
El Salvador	0.105 (0.047-0.191)	0.085 (0.045-0.139)	0.069 (0.039-0.110)	0.053 (0.030-0.082)	0.042 (0.021-0.072)
Equatorial Guinea	0.235 (0.115-0.369)	0.223 (0.128-0.333)	0.208 (0.124-0.306)	0.163 (0.087-0.255)	0.130 (0.054-0.237)
Eritrea	0.230 (0.131-0.344)	0.220 (0.145-0.307)	0.208 (0.145-0.279)	0.196 (0.140-0.260)	0.188 (0.120-0.264)
Estonia	0.070 (0.035-0.126)	0.053 (0.032-0.080)	0.043 (0.028-0.063)	0.031 (0.021-0.043)	0.021 (0.012-0.034)
Ethiopia	0.235 (0.128-0.359)	0.226 (0.145-0.320)	0.219 (0.152-0.296)	0.217 (0.157-0.285)	0.206 (0.137-0.286)
Fiji	0.072 (0.026-0.147)	0.056 (0.024-0.104)	0.045 (0.021-0.081)	0.035 (0.018-0.061)	0.029 (0.014-0.053)
Finland	0.065 (0.036-0.105)	0.042 (0.029-0.059)	0.029 (0.020-0.040)	0.022 (0.015-0.030)	0.017 (0.010-0.027)
France	0.055 (0.028-0.094)	0.039 (0.024-0.060)	0.029 (0.018-0.043)	0.021 (0.013-0.032)	0.018 (0.009-0.030)
French Polynesia	0.020 (0.007-0.045)	0.014 (0.006-0.030)	0.010 (0.004-0.020)	0.007 (0.003-0.013)	0.005 (0.002-0.011)
Gabon	0.229 (0.113-0.363)	0.185 (0.100-0.286)	0.155 (0.087-0.242)	0.131 (0.073-0.208)	0.113 (0.056-0.193)
Gambia	0.217 (0.120-0.335)	0.204 (0.134-0.287)	0.184 (0.125-0.252)	0.164 (0.113-0.224)	0.149 (0.089-0.222)

Georgia	0.093 (0.040-0.179)	0.073 (0.036-0.128)	0.065 (0.033-0.110)	0.053 (0.028-0.089)	0.039 (0.019-0.071)
Germany	0.058 (0.034-0.092)	0.039 (0.028-0.051)	0.028 (0.020-0.036)	0.021 (0.015-0.029)	0.016 (0.009-0.026)
Ghana	0.204 (0.113-0.313)	0.191 (0.125-0.269)	0.177 (0.124-0.237)	0.157 (0.109-0.211)	0.134 (0.080-0.200)
Greece	0.065 (0.031-0.118)	0.043 (0.025-0.070)	0.031 (0.018-0.049)	0.022 (0.013-0.036)	0.018 (0.008-0.032)
Greenland	0.071 (0.037-0.122)	0.046 (0.028-0.074)	0.032 (0.019-0.052)	0.024 (0.014-0.038)	0.018 (0.009-0.031)
Grenada	0.139 (0.067-0.235)	0.121 (0.070-0.187)	0.098 (0.058-0.149)	0.076 (0.045-0.116)	0.056 (0.028-0.098)
Guatemala	0.116 (0.055-0.205)	0.097 (0.054-0.154)	0.081 (0.048-0.123)	0.062 (0.038-0.094)	0.050 (0.026-0.086)
Guinea	0.223 (0.115-0.356)	0.210 (0.132-0.304)	0.199 (0.134-0.275)	0.185 (0.129-0.248)	0.174 (0.110-0.248)
Guinea Bissau	0.224 (0.121-0.344)	0.211 (0.135-0.302)	0.192 (0.125-0.270)	0.182 (0.123-0.251)	0.172 (0.103-0.253)
Guyana	0.143 (0.072-0.240)	0.124 (0.073-0.193)	0.111 (0.068-0.167)	0.087 (0.051-0.134)	0.073 (0.036-0.125)
Haiti	0.180 (0.091-0.297)	0.164 (0.096-0.248)	0.149 (0.089-0.220)	0.130 (0.077-0.196)	0.113 (0.056-0.192)
Honduras	0.128 (0.060-0.230)	0.107 (0.060-0.175)	0.090 (0.053-0.140)	0.071 (0.043-0.110)	0.058 (0.030-0.099)
Hungary	0.054 (0.027-0.096)	0.038 (0.023-0.058)	0.028 (0.017-0.043)	0.022 (0.013-0.034)	0.016 (0.008-0.028)
Iceland	0.051 (0.026-0.089)	0.030 (0.018-0.048)	0.024 (0.014-0.037)	0.018 (0.011-0.029)	0.013 (0.006-0.024)
India	0.211 (0.133-0.298)	0.202 (0.153-0.257)	0.192 (0.159-0.228)	0.178 (0.150-0.207)	0.159 (0.116-0.210)
Indonesia	0.257 (0.166-0.357)	0.241 (0.180-0.306)	0.218 (0.170-0.268)	0.189 (0.146-0.234)	0.159 (0.107-0.219)
Iran	0.133 (0.070-0.221)	0.105 (0.069-0.150)	0.087 (0.063-0.115)	0.067 (0.053-0.085)	0.050 (0.032-0.073)
Iraq	0.098 (0.046-0.176)	0.079 (0.045-0.126)	0.070 (0.042-0.107)	0.060 (0.036-0.092)	0.047 (0.024-0.079)
Ireland	0.062 (0.029-0.113)	0.042 (0.023-0.068)	0.030 (0.017-0.047)	0.020 (0.011-0.031)	0.016 (0.008-0.028)
Israel	0.051 (0.023-0.095)	0.038 (0.022-0.062)	0.029 (0.017-0.045)	0.022 (0.013-0.034)	0.017 (0.009-0.031)
Italy	0.059 (0.032-0.098)	0.042 (0.029-0.058)	0.030 (0.022-0.040)	0.022 (0.016-0.030)	0.018 (0.010-0.028)
Jamaica	0.135 (0.072-0.220)	0.118 (0.077-0.173)	0.102 (0.070-0.141)	0.079 (0.054-0.112)	0.062 (0.033-0.099)
Japan	0.173 (0.131-0.219)	0.145 (0.122-0.170)	0.119 (0.100-0.140)	0.102 (0.084-0.122)	0.090 (0.063-0.125)
Jordan	0.094 (0.042-0.172)	0.071 (0.042-0.109)	0.052 (0.032-0.077)	0.039 (0.025-0.058)	0.030 (0.016-0.049)
Kazakhstan	0.095 (0.041-0.174)	0.073 (0.036-0.124)	0.058 (0.029-0.099)	0.045 (0.023-0.076)	0.028 (0.012-0.053)
Kenya	0.243 (0.139-0.360)	0.222 (0.146-0.309)	0.203 (0.138-0.277)	0.192 (0.131-0.260)	0.176 (0.105-0.261)
Kiribati	0.026 (0.010-0.053)	0.020 (0.009-0.036)	0.017 (0.008-0.030)	0.013 (0.006-0.023)	0.012 (0.005-0.023)
Kuwait	0.065 (0.026-0.129)	0.041 (0.023-0.064)	0.032 (0.020-0.047)	0.021 (0.013-0.031)	0.016 (0.008-0.027)
Kyrgyzstan	0.105 (0.045-0.199)	0.088 (0.045-0.151)	0.076 (0.040-0.127)	0.067 (0.036-0.110)	0.054 (0.026-0.094)
Lao PDR	0.262 (0.160-0.375)	0.250 (0.172-0.332)	0.238 (0.171-0.307)	0.213 (0.154-0.277)	0.183 (0.118-0.256)
Latvia	0.073 (0.033-0.135)	0.054 (0.029-0.088)	0.044 (0.025-0.071)	0.035 (0.020-0.055)	0.021 (0.010-0.039)
Lebanon	0.089 (0.043-0.157)	0.067 (0.038-0.106)	0.049 (0.028-0.078)	0.034 (0.019-0.056)	0.027 (0.014-0.047)
Lesotho	0.235 (0.127-0.362)	0.224 (0.145-0.317)	0.208 (0.140-0.286)	0.191 (0.131-0.261)	0.173 (0.107-0.252)
Liberia	0.211 (0.116-0.328)	0.193 (0.121-0.280)	0.190 (0.116-0.278)	0.185 (0.125-0.252)	0.169 (0.103-0.245)
Libya	0.101 (0.039-0.193)	0.063 (0.035-0.100)	0.049 (0.030-0.074)	0.039 (0.025-0.058)	0.032 (0.017-0.054)
Lithuania	0.079 (0.037-0.139)	0.054 (0.032-0.085)	0.043 (0.026-0.064)	0.032 (0.019-0.048)	0.019 (0.009-0.034)
Luxembourg	0.060 (0.030-0.105)	0.039 (0.021-0.065)	0.025 (0.013-0.042)	0.019 (0.011-0.031)	0.015 (0.007-0.026)
Macedonia (TFYR)	0.082 (0.036-0.152)	0.059 (0.031-0.100)	0.046 (0.025-0.075)	0.034 (0.018-0.056)	0.024 (0.011-0.044)
Madagascar	0.226 (0.127-0.341)	0.211 (0.139-0.292)	0.199 (0.137-0.268)	0.192 (0.137-0.256)	0.184 (0.115-0.264)
Malawi	0.239 (0.128-0.368)	0.230 (0.146-0.324)	0.222 (0.153-0.299)	0.215 (0.152-0.283)	0.204 (0.131-0.283)
Malaysia	0.221 (0.134-0.326)	0.190 (0.130-0.257)	0.154 (0.108-0.205)	0.119 (0.085-0.157)	0.092 (0.057-0.138)
Maldives	0.227 (0.125-0.343)	0.206 (0.131-0.286)	0.177 (0.112-0.247)	0.137 (0.082-0.203)	0.109 (0.059-0.176)
Mali	0.226 (0.122-0.351)	0.215 (0.137-0.304)	0.205 (0.138-0.281)	0.192 (0.132-0.259)	0.174 (0.107-0.255)
Malta	0.051 (0.018-0.109)	0.035 (0.018-0.060)	0.027 (0.015-0.044)	0.018 (0.010-0.030)	0.014 (0.006-0.026)
Marshall Islands	0.020 (0.007-0.044)	0.016 (0.007-0.030)	0.012 (0.006-0.023)	0.011 (0.005-0.019)	0.009 (0.004-0.019)
Mauritania	0.224 (0.123-0.344)	0.202 (0.127-0.287)	0.182 (0.119-0.255)	0.166 (0.110-0.232)	0.147 (0.086-0.222)
Mauritius	0.188 (0.104-0.291)	0.167 (0.109-0.234)	0.143 (0.096-0.197)	0.118 (0.078-0.168)	0.097 (0.052-0.159)
Mexico	0.095 (0.048-0.165)	0.069 (0.042-0.105)	0.049 (0.033-0.070)	0.036 (0.024-0.048)	0.027 (0.015-0.042)
Micronesia (Federated States of)	0.034 (0.012-0.071)	0.027 (0.013-0.050)	0.022 (0.012-0.039)	0.019 (0.010-0.031)	0.016 (0.007-0.029)
Moldova	0.104 (0.047-0.191)	0.081 (0.043-0.132)	0.069 (0.038-0.111)	0.063 (0.035-0.100)	0.044 (0.022-0.077)
Mongolia	0.097 (0.041-0.187)	0.080 (0.041-0.136)	0.069 (0.038-0.111)	0.058 (0.034-0.089)	0.043 (0.023-0.072)
Montenegro	0.095 (0.038-0.186)	0.064 (0.032-0.112)	0.045 (0.025-0.074)	0.030 (0.016-0.048)	0.020 (0.010-0.037)
Morocco	0.125 (0.059-0.221)	0.105 (0.062-0.162)	0.087 (0.054-0.130)	0.070 (0.044-0.105)	0.056 (0.029-0.094)
Mozambique	0.232 (0.116-0.372)	0.221 (0.133-0.325)	0.214 (0.140-0.296)	0.206 (0.143-0.275)	0.198 (0.126-0.280)
Myanmar	0.238 (0.115-0.386)	0.235 (0.149-0.330)	0.229 (0.162-0.303)	0.213 (0.157-0.275)	0.180 (0.119-0.248)
Namibia	0.225 (0.118-0.346)	0.206 (0.128-0.294)	0.188 (0.122-0.262)	0.161 (0.107-0.224)	0.136 (0.080-0.207)
Nauru	0.009 (0.003-0.018)	0.006 (0.003-0.012)	0.005 (0.002-0.010)	0.005 (0.002-0.010)	0.005 (0.002-0.012)
Nepal	0.223 (0.123-0.341)	0.212 (0.136-0.297)	0.197 (0.136-0.265)	0.180 (0.124-0.243)	0.163 (0.101-0.237)
Netherlands	0.077 (0.042-0.126)	0.055 (0.036-0.080)	0.039 (0.026-0.055)	0.026 (0.018-0.036)	0.020 (0.012-0.033)
New Zealand	0.051 (0.027-0.088)	0.036 (0.022-0.054)	0.027 (0.017-0.039)	0.021 (0.014-0.030)	0.016 (0.010-0.026)
Nicaragua	0.107 (0.049-0.188)	0.088 (0.046-0.142)	0.080 (0.045-0.125)	0.067 (0.040-0.103)	0.057 (0.029-0.099)
Niger	0.239 (0.134-0.359)	0.232 (0.151-0.322)	0.221 (0.151-0.296)	0.212 (0.150-0.280)	0.207 (0.132-0.289)
Nigeria	0.225 (0.135-0.332)	0.204 (0.145-0.273)	0.189 (0.143-0.242)	0.168 (0.127-0.211)	0.139 (0.093-0.194)
Niue	0.028 (0.010-0.061)	0.019 (0.008-0.037)	0.014 (0.006-0.025)	0.010 (0.004-0.017)	0.006 (0.002-0.013)
North Korea	0.179 (0.088-0.290)	0.164 (0.098-0.245)	0.156 (0.097-0.232)	0.163 (0.094-0.247)	0.159 (0.076-0.267)
Norway	0.080 (0.044-0.131)	0.050 (0.032-0.074)	0.035 (0.023-0.051)	0.023 (0.015-0.034)	0.017 (0.009-0.030)
Occupied Palestinian Territory	0.086 (0.039-0.158)	0.071 (0.038-0.115)	0.060 (0.035-0.092)	0.049 (0.029-0.073)	0.043 (0.021-0.076)
Oman	0.130 (0.059-0.228)	0.092 (0.056-0.141)	0.065 (0.042-0.095)	0.048 (0.031-0.071)	0.035 (0.019-0.059)
Pakistan	0.204 (0.118-0.305)	0.189 (0.127-0.258)	0.173 (0.123-0.231)	0.158 (0.110-0.213)	0.141 (0.086-0.211)
Palau	0.021 (0.007-0.048)	0.015 (0.007-0.027)	0.011 (0.005-0.020)	0.008 (0.004-0.015)	0.007 (0.003-0.014)

Panama	0.098 (0.047-0.177)	0.075 (0.042-0.124)	0.060 (0.035-0.095)	0.046 (0.028-0.071)	0.034 (0.018-0.058)
Papua New Guinea	0.088 (0.031-0.183)	0.075 (0.032-0.140)	0.065 (0.030-0.116)	0.058 (0.029-0.101)	0.050 (0.022-0.094)
Paraguay	0.116 (0.052-0.211)	0.098 (0.051-0.163)	0.074 (0.041-0.121)	0.055 (0.030-0.090)	0.044 (0.021-0.079)
Peru	0.092 (0.040-0.169)	0.072 (0.038-0.121)	0.059 (0.033-0.095)	0.046 (0.028-0.071)	0.034 (0.018-0.057)
Philippines	0.222 (0.131-0.324)	0.204 (0.141-0.273)	0.186 (0.131-0.243)	0.169 (0.121-0.222)	0.149 (0.094-0.215)
Poland	0.065 (0.033-0.115)	0.048 (0.031-0.072)	0.039 (0.026-0.055)	0.028 (0.019-0.040)	0.019 (0.011-0.031)
Portugal	0.083 (0.039-0.146)	0.064 (0.039-0.098)	0.042 (0.028-0.062)	0.026 (0.017-0.039)	0.019 (0.010-0.033)
Puerto Rico	0.113 (0.057-0.190)	0.083 (0.047-0.132)	0.059 (0.033-0.094)	0.042 (0.024-0.067)	0.032 (0.016-0.056)
Qatar	0.079 (0.035-0.145)	0.055 (0.028-0.096)	0.040 (0.023-0.064)	0.027 (0.015-0.042)	0.019 (0.009-0.033)
Romania	0.084 (0.038-0.154)	0.055 (0.031-0.090)	0.042 (0.025-0.067)	0.032 (0.019-0.051)	0.021 (0.010-0.038)
Russian Federation	0.085 (0.044-0.140)	0.063 (0.044-0.086)	0.051 (0.039-0.066)	0.041 (0.029-0.054)	0.027 (0.014-0.044)
Rwanda	0.275 (0.144-0.418)	0.262 (0.157-0.375)	0.245 (0.161-0.338)	0.229 (0.156-0.309)	0.214 (0.135-0.302)
Saint Kitts and Nevis	0.130 (0.064-0.218)	0.104 (0.057-0.163)	0.086 (0.050-0.133)	0.064 (0.036-0.099)	0.049 (0.023-0.088)
Saint Lucia	0.147 (0.076-0.242)	0.127 (0.078-0.189)	0.103 (0.065-0.150)	0.076 (0.046-0.115)	0.058 (0.027-0.102)
Saint Vincent and the Grenadines	0.140 (0.068-0.239)	0.122 (0.070-0.189)	0.100 (0.058-0.153)	0.077 (0.046-0.119)	0.057 (0.028-0.097)
Samoa	0.022 (0.008-0.048)	0.016 (0.008-0.030)	0.012 (0.006-0.021)	0.009 (0.004-0.015)	0.006 (0.002-0.013)
Sao Tome and Principe	0.203 (0.110-0.314)	0.187 (0.118-0.270)	0.164 (0.104-0.233)	0.147 (0.098-0.204)	0.128 (0.076-0.192)
Saudi Arabia	0.103 (0.045-0.190)	0.064 (0.040-0.097)	0.045 (0.031-0.061)	0.034 (0.023-0.048)	0.025 (0.014-0.041)
Senegal	0.204 (0.112-0.318)	0.193 (0.124-0.276)	0.181 (0.122-0.254)	0.168 (0.115-0.232)	0.156 (0.096-0.228)
Serbia	0.090 (0.044-0.161)	0.065 (0.039-0.099)	0.049 (0.031-0.072)	0.035 (0.022-0.051)	0.026 (0.014-0.042)
Seychelles	0.179 (0.095-0.277)	0.148 (0.090-0.214)	0.123 (0.077-0.179)	0.097 (0.060-0.144)	0.078 (0.041-0.131)
Sierra Leone	0.215 (0.117-0.334)	0.203 (0.131-0.290)	0.197 (0.135-0.270)	0.189 (0.136-0.252)	0.170 (0.110-0.240)
Singapore	0.166 (0.090-0.260)	0.132 (0.089-0.182)	0.102 (0.072-0.138)	0.082 (0.058-0.112)	0.062 (0.038-0.095)
Slovakia	0.063 (0.029-0.116)	0.044 (0.025-0.070)	0.032 (0.019-0.049)	0.024 (0.014-0.036)	0.017 (0.008-0.028)
Slovenia	0.082 (0.035-0.154)	0.055 (0.029-0.092)	0.040 (0.022-0.066)	0.027 (0.015-0.044)	0.019 (0.009-0.035)
Solomon Islands	0.091 (0.031-0.186)	0.077 (0.032-0.144)	0.067 (0.032-0.121)	0.063 (0.032-0.107)	0.056 (0.026-0.104)
Somalia	0.213 (0.106-0.340)	0.203 (0.118-0.305)	0.198 (0.122-0.282)	0.192 (0.120-0.272)	0.187 (0.106-0.283)
South Africa	0.180 (0.100-0.274)	0.158 (0.106-0.219)	0.138 (0.099-0.183)	0.119 (0.089-0.153)	0.096 (0.064-0.135)
South Korea	0.229 (0.146-0.324)	0.169 (0.124-0.220)	0.119 (0.088-0.156)	0.092 (0.070-0.119)	0.061 (0.039-0.090)
Spain	0.062 (0.031-0.105)	0.041 (0.027-0.058)	0.028 (0.020-0.038)	0.019 (0.013-0.026)	0.015 (0.008-0.024)
Sri Lanka	0.232 (0.138-0.344)	0.223 (0.153-0.300)	0.206 (0.147-0.269)	0.183 (0.129-0.240)	0.155 (0.091-0.233)
Sudan	0.220 (0.121-0.338)	0.203 (0.128-0.288)	0.188 (0.122-0.259)	0.171 (0.113-0.236)	0.154 (0.091-0.232)
Suriname	0.128 (0.062-0.216)	0.101 (0.056-0.159)	0.089 (0.052-0.141)	0.069 (0.040-0.111)	0.053 (0.027-0.094)
Swaziland	0.232 (0.125-0.351)	0.207 (0.128-0.299)	0.185 (0.118-0.263)	0.162 (0.104-0.232)	0.142 (0.078-0.224)
Sweden	0.067 (0.039-0.106)	0.048 (0.034-0.065)	0.036 (0.026-0.047)	0.026 (0.018-0.035)	0.019 (0.011-0.030)
Switzerland	0.076 (0.036-0.134)	0.050 (0.029-0.077)	0.033 (0.020-0.047)	0.025 (0.016-0.037)	0.020 (0.011-0.033)
Syrian Arab Republic	0.113 (0.052-0.198)	0.085 (0.048-0.134)	0.072 (0.043-0.109)	0.057 (0.034-0.086)	0.047 (0.024-0.080)
Taiwan	0.189 (0.108-0.280)	0.149 (0.101-0.205)	0.109 (0.075-0.149)	0.081 (0.055-0.114)	0.066 (0.035-0.107)
Tajikistan	0.109 (0.046-0.201)	0.093 (0.046-0.157)	0.084 (0.043-0.140)	0.082 (0.043-0.137)	0.066 (0.030-0.119)
Tanzania	0.228 (0.121-0.350)	0.216 (0.139-0.305)	0.207 (0.144-0.277)	0.199 (0.143-0.258)	0.182 (0.118-0.253)
Thailand	0.238 (0.147-0.345)	0.219 (0.158-0.286)	0.195 (0.147-0.248)	0.161 (0.121-0.206)	0.130 (0.080-0.190)
Timor-Leste	0.253 (0.152-0.369)	0.245 (0.168-0.330)	0.232 (0.165-0.306)	0.221 (0.158-0.291)	0.205 (0.132-0.285)
Togo	0.222 (0.125-0.344)	0.209 (0.133-0.296)	0.196 (0.132-0.269)	0.184 (0.130-0.244)	0.172 (0.108-0.246)
Tokelau	0.036 (0.009-0.093)	0.025 (0.009-0.056)	0.019 (0.008-0.036)	0.014 (0.006-0.026)	0.010 (0.004-0.022)
Tonga	0.023 (0.008-0.051)	0.017 (0.008-0.032)	0.013 (0.006-0.024)	0.010 (0.005-0.018)	0.008 (0.003-0.016)
Trinidad and Tobago	0.154 (0.061-0.277)	0.125 (0.060-0.208)	0.104 (0.059-0.162)	0.076 (0.040-0.123)	0.052 (0.019-0.106)
Tunisia	0.119 (0.058-0.210)	0.096 (0.059-0.147)	0.078 (0.051-0.114)	0.061 (0.040-0.089)	0.048 (0.027-0.079)
Turkey	0.099 (0.049-0.172)	0.075 (0.048-0.109)	0.054 (0.038-0.075)	0.039 (0.028-0.051)	0.028 (0.017-0.042)
Turkmenistan	0.097 (0.040-0.184)	0.081 (0.040-0.139)	0.066 (0.034-0.112)	0.056 (0.030-0.093)	0.038 (0.018-0.067)
Tuvalu	0.026 (0.009-0.054)	0.020 (0.009-0.037)	0.015 (0.007-0.027)	0.012 (0.006-0.021)	0.010 (0.004-0.019)
Uganda	0.244 (0.128-0.373)	0.238 (0.147-0.338)	0.231 (0.151-0.316)	0.218 (0.151-0.294)	0.198 (0.128-0.277)
Ukraine	0.085 (0.038-0.153)	0.063 (0.033-0.102)	0.051 (0.028-0.084)	0.044 (0.024-0.072)	0.031 (0.014-0.056)
United Arab Emirates	0.071 (0.023-0.153)	0.046 (0.022-0.080)	0.034 (0.019-0.052)	0.025 (0.015-0.039)	0.023 (0.011-0.041)
United Kingdom	0.058 (0.033-0.093)	0.044 (0.032-0.059)	0.034 (0.026-0.043)	0.025 (0.020-0.032)	0.019 (0.013-0.028)
United States of America	0.051 (0.030-0.080)	0.036 (0.024-0.050)	0.025 (0.018-0.035)	0.018 (0.013-0.025)	0.014 (0.008-0.022)
Uruguay	0.070 (0.028-0.139)	0.057 (0.029-0.099)	0.047 (0.026-0.075)	0.036 (0.021-0.056)	0.029 (0.014-0.050)
Uzbekistan	0.102 (0.042-0.197)	0.085 (0.041-0.149)	0.073 (0.038-0.122)	0.065 (0.034-0.107)	0.050 (0.023-0.091)
Vanuatu	0.074 (0.025-0.157)	0.061 (0.026-0.117)	0.053 (0.025-0.095)	0.046 (0.023-0.080)	0.040 (0.018-0.074)
Venezuela	0.074 (0.033-0.138)	0.054 (0.029-0.088)	0.042 (0.024-0.064)	0.032 (0.019-0.048)	0.024 (0.012-0.040)
Viet Nam	0.253 (0.159-0.353)	0.251 (0.189-0.314)	0.240 (0.194-0.290)	0.215 (0.173-0.259)	0.178 (0.123-0.239)
Yemen	0.153 (0.070-0.267)	0.131 (0.075-0.205)	0.111 (0.068-0.167)	0.095 (0.058-0.143)	0.082 (0.043-0.137)
Zambia	0.209 (0.118-0.314)	0.191 (0.121-0.271)	0.185 (0.124-0.257)	0.179 (0.124-0.243)	0.163 (0.099-0.240)
Zimbabwe	0.226 (0.128-0.339)	0.207 (0.135-0.286)	0.191 (0.130-0.256)	0.177 (0.122-0.236)	0.171 (0.107-0.248)

Women, 18.5 ≤ BMI < 20 kg/m^2

Country	Year				
	1975	1985	1995	2005	2014
Afghanistan	0.228 (0.132-0.340)	0.208 (0.139-0.288)	0.192 (0.136-0.257)	0.179 (0.127-0.236)	0.161 (0.103-0.230)
Albania	0.119 (0.054-0.207)	0.104 (0.061-0.157)	0.095 (0.062-0.136)	0.083 (0.054-0.117)	0.074 (0.042-0.118)
Algeria	0.122 (0.057-0.221)	0.094 (0.057-0.147)	0.073 (0.049-0.102)	0.056 (0.038-0.078)	0.044 (0.025-0.069)
American Samoa	0.021 (0.008-0.042)	0.016 (0.007-0.029)	0.013 (0.006-0.023)	0.012 (0.005-0.020)	0.011 (0.004-0.022)
Andorra	0.064 (0.031-0.113)	0.053 (0.030-0.082)	0.049 (0.030-0.074)	0.045 (0.028-0.067)	0.044 (0.023-0.073)
Angola	0.233 (0.121-0.363)	0.204 (0.124-0.297)	0.176 (0.116-0.247)	0.151 (0.098-0.215)	0.124 (0.068-0.197)
Antigua and Barbuda	0.112 (0.051-0.201)	0.088 (0.050-0.140)	0.066 (0.039-0.099)	0.051 (0.030-0.081)	0.037 (0.016-0.070)
Argentina	0.068 (0.030-0.125)	0.059 (0.034-0.093)	0.053 (0.033-0.076)	0.048 (0.032-0.067)	0.044 (0.025-0.069)
Armenia	0.098 (0.037-0.197)	0.083 (0.042-0.143)	0.077 (0.046-0.117)	0.067 (0.043-0.097)	0.054 (0.028-0.088)
Australia	0.093 (0.058-0.138)	0.079 (0.058-0.103)	0.069 (0.052-0.088)	0.059 (0.045-0.076)	0.052 (0.034-0.074)
Austria	0.104 (0.060-0.161)	0.092 (0.064-0.128)	0.083 (0.058-0.112)	0.073 (0.050-0.099)	0.065 (0.038-0.099)
Azerbaijan	0.108 (0.043-0.203)	0.088 (0.046-0.148)	0.078 (0.047-0.118)	0.067 (0.042-0.100)	0.050 (0.026-0.086)
Bahamas	0.077 (0.035-0.139)	0.062 (0.035-0.097)	0.050 (0.030-0.076)	0.042 (0.025-0.063)	0.037 (0.019-0.063)
Bahrain	0.064 (0.027-0.123)	0.054 (0.031-0.088)	0.046 (0.029-0.068)	0.037 (0.024-0.055)	0.032 (0.017-0.052)
Bangladesh	0.212 (0.127-0.311)	0.194 (0.136-0.258)	0.179 (0.136-0.226)	0.162 (0.125-0.203)	0.145 (0.099-0.200)
Barbados	0.120 (0.059-0.213)	0.094 (0.055-0.144)	0.076 (0.049-0.110)	0.056 (0.035-0.082)	0.041 (0.020-0.072)
Belarus	0.084 (0.036-0.152)	0.069 (0.039-0.109)	0.063 (0.040-0.094)	0.060 (0.038-0.088)	0.053 (0.028-0.088)
Belgium	0.078 (0.043-0.128)	0.073 (0.051-0.101)	0.067 (0.049-0.089)	0.063 (0.045-0.085)	0.063 (0.039-0.095)
Belize	0.117 (0.054-0.209)	0.100 (0.059-0.155)	0.083 (0.053-0.119)	0.064 (0.041-0.092)	0.052 (0.028-0.086)
Benin	0.215 (0.123-0.325)	0.185 (0.126-0.256)	0.162 (0.119-0.211)	0.139 (0.106-0.178)	0.119 (0.079-0.164)
Bermuda	0.048 (0.018-0.099)	0.043 (0.021-0.075)	0.037 (0.020-0.061)	0.036 (0.020-0.056)	0.032 (0.015-0.056)
Bhutan	0.246 (0.145-0.360)	0.223 (0.149-0.304)	0.197 (0.139-0.258)	0.170 (0.122-0.220)	0.146 (0.096-0.205)
Bolivia	0.088 (0.033-0.170)	0.067 (0.034-0.113)	0.052 (0.030-0.081)	0.043 (0.026-0.064)	0.038 (0.020-0.064)
Bosnia and Herzegovina	0.127 (0.050-0.240)	0.110 (0.059-0.180)	0.101 (0.064-0.150)	0.083 (0.054-0.119)	0.078 (0.046-0.118)
Botswana	0.193 (0.091-0.317)	0.153 (0.089-0.230)	0.108 (0.069-0.156)	0.087 (0.057-0.123)	0.071 (0.040-0.111)
Brazil	0.113 (0.057-0.191)	0.090 (0.058-0.129)	0.076 (0.056-0.101)	0.068 (0.051-0.088)	0.062 (0.040-0.091)
Brunei Darussalam	0.173 (0.071-0.310)	0.146 (0.076-0.235)	0.120 (0.073-0.174)	0.098 (0.061-0.142)	0.089 (0.047-0.144)
Bulgaria	0.082 (0.039-0.145)	0.069 (0.041-0.103)	0.066 (0.043-0.094)	0.067 (0.044-0.095)	0.067 (0.037-0.105)
Burkina Faso	0.236 (0.135-0.350)	0.211 (0.146-0.284)	0.186 (0.140-0.239)	0.158 (0.121-0.204)	0.139 (0.095-0.195)
Burundi	0.265 (0.147-0.397)	0.241 (0.149-0.341)	0.211 (0.140-0.287)	0.182 (0.125-0.246)	0.156 (0.098-0.224)
Cabo Verde	0.219 (0.120-0.335)	0.187 (0.125-0.261)	0.155 (0.108-0.205)	0.123 (0.087-0.163)	0.105 (0.065-0.156)
Cambodia	0.264 (0.158-0.386)	0.243 (0.172-0.324)	0.216 (0.163-0.273)	0.186 (0.143-0.233)	0.155 (0.105-0.212)
Cameroon	0.194 (0.106-0.300)	0.161 (0.107-0.226)	0.137 (0.099-0.179)	0.117 (0.085-0.148)	0.098 (0.062-0.139)
Canada	0.087 (0.051-0.136)	0.070 (0.048-0.096)	0.057 (0.040-0.077)	0.047 (0.033-0.063)	0.041 (0.025-0.063)
Central African Republic	0.212 (0.110-0.338)	0.191 (0.116-0.281)	0.177 (0.117-0.245)	0.160 (0.107-0.220)	0.142 (0.080-0.217)
Chad	0.220 (0.128-0.332)	0.195 (0.133-0.268)	0.172 (0.126-0.224)	0.144 (0.108-0.189)	0.124 (0.080-0.179)
Chile	0.068 (0.029-0.132)	0.055 (0.030-0.091)	0.048 (0.030-0.071)	0.042 (0.027-0.060)	0.037 (0.020-0.060)
China	0.197 (0.119-0.288)	0.182 (0.143-0.224)	0.161 (0.137-0.188)	0.130 (0.105-0.157)	0.104 (0.070-0.147)
China (Hong Kong SAR)	0.090 (0.034-0.178)	0.088 (0.046-0.144)	0.089 (0.055-0.134)	0.095 (0.059-0.142)	0.097 (0.050-0.167)
Colombia	0.092 (0.042-0.168)	0.073 (0.043-0.114)	0.061 (0.041-0.086)	0.053 (0.037-0.071)	0.047 (0.029-0.071)
Comoros	0.201 (0.111-0.311)	0.182 (0.121-0.255)	0.161 (0.114-0.216)	0.142 (0.104-0.187)	0.122 (0.079-0.175)
Congo	0.194 (0.105-0.298)	0.166 (0.108-0.232)	0.143 (0.101-0.194)	0.128 (0.090-0.172)	0.113 (0.066-0.171)
Cook Islands	0.034 (0.014-0.067)	0.025 (0.012-0.042)	0.018 (0.009-0.030)	0.013 (0.007-0.023)	0.011 (0.005-0.022)
Costa Rica	0.106 (0.046-0.194)	0.082 (0.046-0.132)	0.062 (0.039-0.092)	0.048 (0.031-0.069)	0.041 (0.023-0.066)
Cote d'Ivoire	0.189 (0.104-0.294)	0.165 (0.110-0.230)	0.145 (0.106-0.189)	0.124 (0.092-0.161)	0.108 (0.069-0.153)
Croatia	0.107 (0.052-0.184)	0.088 (0.053-0.131)	0.079 (0.052-0.112)	0.070 (0.046-0.100)	0.063 (0.036-0.100)
Cuba	0.096 (0.045-0.171)	0.076 (0.045-0.118)	0.066 (0.043-0.095)	0.059 (0.039-0.084)	0.050 (0.028-0.081)
Cyprus	0.110 (0.054-0.186)	0.080 (0.048-0.121)	0.064 (0.041-0.092)	0.054 (0.034-0.078)	0.048 (0.026-0.079)
Czech Republic	0.059 (0.028-0.103)	0.052 (0.031-0.076)	0.053 (0.035-0.075)	0.054 (0.036-0.077)	0.056 (0.032-0.088)
Denmark	0.088 (0.049-0.139)	0.077 (0.052-0.109)	0.071 (0.050-0.098)	0.067 (0.046-0.094)	0.064 (0.038-0.098)
Djibouti	0.150 (0.068-0.259)	0.138 (0.074-0.226)	0.132 (0.083-0.193)	0.129 (0.083-0.185)	0.121 (0.066-0.192)
Dominica	0.116 (0.056-0.201)	0.090 (0.054-0.138)	0.070 (0.045-0.101)	0.056 (0.035-0.082)	0.046 (0.025-0.075)
Dominican Republic	0.134 (0.067-0.227)	0.105 (0.066-0.156)	0.087 (0.059-0.120)	0.066 (0.045-0.093)	0.055 (0.031-0.087)
DR Congo	0.212 (0.114-0.329)	0.189 (0.120-0.270)	0.168 (0.113-0.232)	0.152 (0.104-0.209)	0.135 (0.079-0.204)
Ecuador	0.090 (0.035-0.178)	0.065 (0.033-0.111)	0.050 (0.028-0.079)	0.042 (0.025-0.063)	0.036 (0.019-0.060)
Egypt	0.100 (0.044-0.190)	0.074 (0.044-0.114)	0.055 (0.036-0.076)	0.038 (0.026-0.052)	0.026 (0.014-0.041)
El Salvador	0.107 (0.048-0.192)	0.085 (0.048-0.133)	0.067 (0.042-0.097)	0.053 (0.035-0.076)	0.045 (0.026-0.072)
Equatorial Guinea	0.212 (0.110-0.339)	0.190 (0.118-0.278)	0.164 (0.108-0.234)	0.123 (0.075-0.183)	0.098 (0.048-0.171)
Eritrea	0.215 (0.123-0.325)	0.196 (0.133-0.268)	0.175 (0.129-0.229)	0.153 (0.115-0.197)	0.133 (0.087-0.190)
Estonia	0.070 (0.032-0.124)	0.064 (0.039-0.095)	0.065 (0.045-0.088)	0.062 (0.045-0.082)	0.060 (0.038-0.088)
Ethiopia	0.221 (0.125-0.338)	0.202 (0.137-0.280)	0.183 (0.134-0.241)	0.167 (0.126-0.214)	0.148 (0.099-0.210)
Fiji	0.081 (0.027-0.169)	0.062 (0.027-0.115)	0.048 (0.024-0.083)	0.038 (0.020-0.061)	0.032 (0.015-0.056)
Finland	0.109 (0.065-0.167)	0.088 (0.063-0.117)	0.075 (0.057-0.096)	0.069 (0.052-0.089)	0.067 (0.045-0.097)
France	0.085 (0.045-0.142)	0.076 (0.049-0.112)	0.069 (0.047-0.097)	0.060 (0.041-0.085)	0.056 (0.033-0.087)
French Polynesia	0.032 (0.012-0.066)	0.025 (0.011-0.046)	0.019 (0.009-0.034)	0.016 (0.008-0.027)	0.013 (0.006-0.026)

Gabon	0.195 (0.089-0.323)	0.147 (0.083-0.228)	0.121 (0.075-0.176)	0.103 (0.065-0.149)	0.096 (0.053-0.151)
Gambia	0.206 (0.115-0.313)	0.180 (0.121-0.249)	0.152 (0.109-0.202)	0.127 (0.092-0.168)	0.113 (0.070-0.166)
Georgia	0.105 (0.041-0.204)	0.084 (0.043-0.142)	0.076 (0.045-0.119)	0.066 (0.041-0.098)	0.055 (0.030-0.091)
Germany	0.093 (0.057-0.140)	0.084 (0.063-0.109)	0.076 (0.059-0.097)	0.068 (0.051-0.090)	0.063 (0.040-0.092)
Ghana	0.187 (0.104-0.288)	0.164 (0.111-0.227)	0.140 (0.103-0.182)	0.114 (0.083-0.150)	0.094 (0.058-0.137)
Greece	0.085 (0.042-0.142)	0.068 (0.042-0.101)	0.059 (0.039-0.084)	0.052 (0.033-0.074)	0.049 (0.027-0.079)
Greenland	0.092 (0.050-0.150)	0.077 (0.049-0.112)	0.068 (0.046-0.095)	0.061 (0.042-0.085)	0.057 (0.034-0.089)
Grenada	0.129 (0.061-0.224)	0.104 (0.062-0.160)	0.080 (0.050-0.116)	0.059 (0.038-0.087)	0.043 (0.022-0.073)
Guatemala	0.118 (0.052-0.213)	0.094 (0.054-0.149)	0.075 (0.049-0.108)	0.058 (0.039-0.082)	0.048 (0.027-0.076)
Guinea	0.213 (0.115-0.331)	0.185 (0.122-0.257)	0.164 (0.119-0.216)	0.141 (0.106-0.183)	0.124 (0.082-0.173)
Guinea Bissau	0.218 (0.125-0.327)	0.191 (0.129-0.264)	0.160 (0.113-0.214)	0.142 (0.104-0.185)	0.128 (0.082-0.183)
Guyana	0.129 (0.062-0.225)	0.104 (0.062-0.159)	0.084 (0.054-0.122)	0.061 (0.037-0.090)	0.048 (0.024-0.081)
Haiti	0.173 (0.089-0.284)	0.147 (0.093-0.215)	0.121 (0.082-0.168)	0.099 (0.067-0.138)	0.084 (0.047-0.134)
Honduras	0.127 (0.059-0.225)	0.099 (0.057-0.155)	0.078 (0.052-0.112)	0.062 (0.043-0.087)	0.051 (0.030-0.080)
Hungary	0.075 (0.038-0.128)	0.067 (0.043-0.100)	0.066 (0.044-0.094)	0.066 (0.044-0.094)	0.065 (0.037-0.102)
Iceland	0.081 (0.043-0.131)	0.065 (0.041-0.096)	0.063 (0.043-0.089)	0.061 (0.041-0.087)	0.058 (0.034-0.091)
India	0.204 (0.130-0.288)	0.186 (0.139-0.238)	0.171 (0.140-0.203)	0.156 (0.131-0.183)	0.143 (0.105-0.187)
Indonesia	0.250 (0.159-0.349)	0.221 (0.162-0.285)	0.188 (0.146-0.233)	0.156 (0.122-0.192)	0.131 (0.088-0.182)
Iran	0.120 (0.058-0.208)	0.092 (0.059-0.136)	0.073 (0.054-0.095)	0.055 (0.043-0.069)	0.040 (0.026-0.059)
Iraq	0.092 (0.041-0.171)	0.072 (0.041-0.113)	0.063 (0.040-0.091)	0.053 (0.035-0.076)	0.042 (0.024-0.068)
Ireland	0.128 (0.070-0.204)	0.099 (0.063-0.142)	0.077 (0.050-0.106)	0.058 (0.037-0.081)	0.048 (0.027-0.075)
Israel	0.062 (0.029-0.112)	0.055 (0.033-0.085)	0.050 (0.032-0.073)	0.047 (0.030-0.069)	0.046 (0.024-0.075)
Italy	0.089 (0.050-0.139)	0.078 (0.056-0.106)	0.069 (0.053-0.090)	0.060 (0.044-0.080)	0.053 (0.033-0.081)
Jamaica	0.107 (0.052-0.184)	0.087 (0.055-0.130)	0.070 (0.048-0.097)	0.054 (0.036-0.075)	0.042 (0.023-0.069)
Japan	0.173 (0.131-0.219)	0.164 (0.139-0.191)	0.160 (0.138-0.184)	0.162 (0.137-0.189)	0.170 (0.126-0.218)
Jordan	0.089 (0.038-0.173)	0.064 (0.038-0.098)	0.047 (0.030-0.066)	0.036 (0.024-0.049)	0.028 (0.016-0.045)
Kazakhstan	0.112 (0.045-0.208)	0.091 (0.049-0.153)	0.077 (0.047-0.116)	0.065 (0.040-0.095)	0.050 (0.025-0.084)
Kenya	0.225 (0.128-0.339)	0.194 (0.133-0.264)	0.166 (0.122-0.215)	0.142 (0.104-0.183)	0.118 (0.075-0.171)
Kiribati	0.045 (0.019-0.090)	0.032 (0.016-0.055)	0.024 (0.012-0.041)	0.017 (0.008-0.029)	0.014 (0.006-0.028)
Kuwait	0.048 (0.017-0.103)	0.038 (0.020-0.062)	0.033 (0.020-0.049)	0.027 (0.017-0.039)	0.022 (0.012-0.037)
Kyrgyzstan	0.129 (0.054-0.238)	0.108 (0.059-0.177)	0.092 (0.057-0.139)	0.079 (0.050-0.113)	0.063 (0.035-0.101)
Lao PDR	0.274 (0.173-0.381)	0.249 (0.179-0.324)	0.222 (0.168-0.279)	0.188 (0.145-0.237)	0.157 (0.107-0.218)
Latvia	0.067 (0.029-0.124)	0.058 (0.033-0.092)	0.057 (0.036-0.086)	0.056 (0.036-0.082)	0.051 (0.028-0.084)
Lebanon	0.084 (0.038-0.152)	0.063 (0.036-0.101)	0.048 (0.029-0.073)	0.036 (0.021-0.056)	0.031 (0.017-0.051)
Lesotho	0.185 (0.086-0.307)	0.156 (0.090-0.233)	0.124 (0.080-0.176)	0.097 (0.065-0.136)	0.077 (0.045-0.120)
Liberia	0.200 (0.113-0.311)	0.169 (0.111-0.238)	0.154 (0.101-0.217)	0.141 (0.101-0.185)	0.122 (0.077-0.175)
Libya	0.088 (0.034-0.175)	0.057 (0.031-0.091)	0.046 (0.029-0.068)	0.038 (0.024-0.055)	0.032 (0.017-0.051)
Lithuania	0.077 (0.035-0.136)	0.061 (0.036-0.092)	0.057 (0.038-0.082)	0.054 (0.035-0.076)	0.047 (0.026-0.077)
Luxembourg	0.096 (0.052-0.157)	0.084 (0.052-0.123)	0.073 (0.046-0.108)	0.066 (0.043-0.093)	0.063 (0.037-0.098)
Macedonia (TFYR)	0.094 (0.044-0.166)	0.080 (0.047-0.123)	0.075 (0.048-0.109)	0.072 (0.048-0.105)	0.069 (0.039-0.108)
Madagascar	0.207 (0.116-0.315)	0.185 (0.126-0.254)	0.165 (0.123-0.217)	0.147 (0.111-0.189)	0.131 (0.086-0.188)
Malawi	0.226 (0.126-0.350)	0.203 (0.137-0.282)	0.181 (0.133-0.235)	0.158 (0.119-0.201)	0.134 (0.088-0.189)
Malaysia	0.227 (0.136-0.335)	0.187 (0.131-0.253)	0.149 (0.110-0.194)	0.120 (0.090-0.154)	0.101 (0.065-0.146)
Maldives	0.238 (0.141-0.350)	0.202 (0.138-0.275)	0.164 (0.113-0.221)	0.123 (0.081-0.173)	0.101 (0.060-0.154)
Mali	0.222 (0.127-0.334)	0.197 (0.135-0.268)	0.174 (0.127-0.226)	0.151 (0.114-0.192)	0.132 (0.086-0.185)
Malta	0.066 (0.027-0.130)	0.057 (0.033-0.088)	0.053 (0.033-0.078)	0.046 (0.029-0.069)	0.044 (0.023-0.074)
Marshall Islands	0.032 (0.012-0.066)	0.024 (0.011-0.043)	0.019 (0.009-0.032)	0.016 (0.008-0.028)	0.015 (0.006-0.029)
Mauritania	0.225 (0.127-0.343)	0.188 (0.124-0.262)	0.159 (0.109-0.215)	0.138 (0.096-0.183)	0.120 (0.074-0.176)
Mauritius	0.167 (0.091-0.263)	0.146 (0.095-0.206)	0.123 (0.085-0.167)	0.099 (0.067-0.136)	0.079 (0.043-0.127)
Mexico	0.083 (0.037-0.156)	0.064 (0.038-0.099)	0.050 (0.034-0.068)	0.040 (0.028-0.054)	0.034 (0.020-0.053)
Micronesia (Federated States of)	0.050 (0.020-0.099)	0.037 (0.018-0.063)	0.028 (0.015-0.044)	0.021 (0.011-0.032)	0.016 (0.007-0.028)
Moldova	0.100 (0.045-0.181)	0.082 (0.047-0.128)	0.076 (0.049-0.110)	0.075 (0.049-0.107)	0.065 (0.037-0.103)
Mongolia	0.122 (0.048-0.230)	0.101 (0.054-0.166)	0.089 (0.055-0.132)	0.078 (0.051-0.111)	0.065 (0.037-0.102)
Montenegro	0.116 (0.050-0.212)	0.089 (0.049-0.142)	0.077 (0.048-0.113)	0.070 (0.045-0.102)	0.066 (0.037-0.106)
Morocco	0.130 (0.061-0.233)	0.101 (0.062-0.155)	0.078 (0.054-0.110)	0.060 (0.041-0.084)	0.046 (0.026-0.074)
Mozambique	0.222 (0.118-0.348)	0.197 (0.127-0.280)	0.175 (0.125-0.233)	0.157 (0.117-0.203)	0.141 (0.094-0.199)
Myanmar	0.244 (0.126-0.381)	0.226 (0.147-0.317)	0.204 (0.148-0.264)	0.176 (0.131-0.223)	0.140 (0.093-0.194)
Namibia	0.166 (0.074-0.284)	0.140 (0.082-0.215)	0.117 (0.079-0.164)	0.093 (0.065-0.128)	0.078 (0.047-0.120)
Nauru	0.011 (0.004-0.025)	0.010 (0.004-0.019)	0.010 (0.005-0.018)	0.012 (0.005-0.021)	0.012 (0.004-0.026)
Nepal	0.236 (0.142-0.344)	0.218 (0.150-0.291)	0.195 (0.146-0.248)	0.171 (0.130-0.215)	0.149 (0.101-0.206)
Netherlands	0.109 (0.062-0.169)	0.095 (0.065-0.129)	0.080 (0.058-0.107)	0.067 (0.048-0.090)	0.063 (0.040-0.093)
New Zealand	0.090 (0.052-0.140)	0.076 (0.052-0.105)	0.063 (0.045-0.084)	0.051 (0.037-0.067)	0.044 (0.029-0.064)
Nicaragua	0.104 (0.047-0.189)	0.083 (0.048-0.131)	0.070 (0.045-0.101)	0.058 (0.039-0.081)	0.050 (0.028-0.080)
Niger	0.237 (0.139-0.351)	0.217 (0.151-0.293)	0.192 (0.143-0.247)	0.168 (0.127-0.215)	0.149 (0.099-0.206)
Nigeria	0.208 (0.117-0.317)	0.178 (0.123-0.241)	0.153 (0.116-0.195)	0.126 (0.097-0.158)	0.101 (0.069-0.142)
Niue	0.051 (0.019-0.104)	0.032 (0.015-0.058)	0.022 (0.011-0.039)	0.015 (0.008-0.026)	0.011 (0.004-0.021)
North Korea	0.145 (0.070-0.247)	0.138 (0.085-0.203)	0.139 (0.092-0.194)	0.151 (0.093-0.218)	0.156 (0.078-0.254)
Norway	0.103 (0.058-0.163)	0.081 (0.055-0.113)	0.068 (0.049-0.092)	0.058 (0.041-0.079)	0.055 (0.033-0.084)

Occupied Palestinian Territory	0.088 (0.040-0.163)	0.069 (0.040-0.111)	0.057 (0.036-0.083)	0.045 (0.029-0.064)	0.037 (0.020-0.061)
Oman	0.125 (0.053-0.229)	0.084 (0.049-0.131)	0.059 (0.040-0.086)	0.046 (0.031-0.067)	0.037 (0.020-0.060)
Pakistan	0.210 (0.124-0.310)	0.190 (0.132-0.255)	0.171 (0.125-0.219)	0.154 (0.113-0.197)	0.139 (0.090-0.196)
Palau	0.026 (0.009-0.056)	0.021 (0.010-0.037)	0.017 (0.008-0.029)	0.014 (0.007-0.025)	0.013 (0.006-0.025)
Panama	0.091 (0.039-0.166)	0.070 (0.039-0.113)	0.057 (0.035-0.084)	0.045 (0.029-0.065)	0.037 (0.020-0.060)
Papua New Guinea	0.120 (0.042-0.239)	0.095 (0.043-0.167)	0.075 (0.039-0.125)	0.060 (0.033-0.098)	0.048 (0.023-0.086)
Paraguay	0.128 (0.059-0.222)	0.106 (0.061-0.162)	0.084 (0.053-0.123)	0.070 (0.044-0.101)	0.060 (0.033-0.095)
Peru	0.071 (0.027-0.140)	0.054 (0.028-0.093)	0.046 (0.028-0.068)	0.039 (0.026-0.053)	0.033 (0.020-0.050)
Philippines	0.227 (0.148-0.321)	0.199 (0.152-0.251)	0.171 (0.137-0.209)	0.152 (0.119-0.189)	0.133 (0.087-0.188)
Poland	0.078 (0.040-0.134)	0.068 (0.044-0.098)	0.066 (0.045-0.089)	0.062 (0.043-0.085)	0.059 (0.036-0.091)
Portugal	0.121 (0.060-0.200)	0.103 (0.064-0.150)	0.081 (0.055-0.111)	0.065 (0.044-0.089)	0.056 (0.032-0.088)
Puerto Rico	0.088 (0.041-0.159)	0.062 (0.036-0.099)	0.047 (0.028-0.071)	0.040 (0.024-0.060)	0.036 (0.019-0.060)
Qatar	0.059 (0.025-0.116)	0.044 (0.021-0.075)	0.037 (0.022-0.056)	0.029 (0.017-0.044)	0.024 (0.012-0.040)
Romania	0.099 (0.048-0.171)	0.079 (0.048-0.118)	0.072 (0.048-0.102)	0.069 (0.047-0.098)	0.064 (0.036-0.102)
Russian Federation	0.067 (0.033-0.112)	0.059 (0.040-0.080)	0.058 (0.044-0.075)	0.058 (0.042-0.077)	0.055 (0.032-0.085)
Rwanda	0.254 (0.136-0.381)	0.229 (0.142-0.324)	0.199 (0.134-0.269)	0.168 (0.118-0.223)	0.144 (0.092-0.208)
Saint Kitts and Nevis	0.120 (0.058-0.209)	0.091 (0.052-0.140)	0.073 (0.046-0.107)	0.055 (0.033-0.081)	0.041 (0.019-0.074)
Saint Lucia	0.134 (0.067-0.227)	0.107 (0.066-0.159)	0.084 (0.055-0.117)	0.061 (0.038-0.089)	0.043 (0.020-0.075)
Saint Vincent and the Grenadines	0.128 (0.062-0.224)	0.102 (0.062-0.156)	0.080 (0.051-0.115)	0.062 (0.040-0.090)	0.048 (0.025-0.080)
Samoa	0.044 (0.017-0.088)	0.030 (0.014-0.053)	0.021 (0.011-0.035)	0.014 (0.007-0.023)	0.009 (0.004-0.018)
Sao Tome and Principe	0.187 (0.102-0.294)	0.162 (0.106-0.230)	0.131 (0.090-0.180)	0.113 (0.080-0.151)	0.099 (0.062-0.144)
Saudi Arabia	0.093 (0.038-0.179)	0.060 (0.036-0.091)	0.046 (0.032-0.062)	0.038 (0.026-0.052)	0.031 (0.017-0.049)
Senegal	0.187 (0.105-0.287)	0.167 (0.114-0.230)	0.149 (0.109-0.195)	0.129 (0.096-0.169)	0.113 (0.072-0.161)
Serbia	0.108 (0.053-0.184)	0.091 (0.057-0.132)	0.083 (0.058-0.114)	0.078 (0.054-0.105)	0.074 (0.044-0.111)
Seychelles	0.149 (0.077-0.246)	0.118 (0.072-0.175)	0.098 (0.064-0.138)	0.076 (0.050-0.109)	0.061 (0.034-0.099)
Sierra Leone	0.198 (0.111-0.306)	0.176 (0.116-0.246)	0.159 (0.114-0.212)	0.141 (0.105-0.181)	0.118 (0.078-0.163)
Singapore	0.147 (0.080-0.233)	0.132 (0.091-0.179)	0.126 (0.093-0.162)	0.130 (0.097-0.165)	0.125 (0.084-0.173)
Slovakia	0.102 (0.051-0.172)	0.084 (0.053-0.122)	0.076 (0.051-0.105)	0.072 (0.050-0.098)	0.066 (0.039-0.103)
Slovenia	0.106 (0.050-0.187)	0.084 (0.051-0.126)	0.075 (0.050-0.108)	0.067 (0.044-0.094)	0.062 (0.035-0.099)
Solomon Islands	0.121 (0.042-0.247)	0.093 (0.042-0.169)	0.073 (0.038-0.120)	0.058 (0.033-0.092)	0.048 (0.024-0.085)
Somalia	0.206 (0.109-0.321)	0.187 (0.117-0.269)	0.172 (0.118-0.238)	0.158 (0.107-0.220)	0.144 (0.085-0.217)
South Africa	0.109 (0.047-0.194)	0.091 (0.052-0.140)	0.076 (0.051-0.105)	0.062 (0.045-0.082)	0.052 (0.033-0.077)
South Korea	0.237 (0.155-0.326)	0.178 (0.132-0.227)	0.141 (0.105-0.179)	0.133 (0.104-0.166)	0.110 (0.074-0.156)
Spain	0.090 (0.049-0.147)	0.073 (0.049-0.101)	0.062 (0.045-0.083)	0.054 (0.038-0.073)	0.050 (0.030-0.078)
Sri Lanka	0.236 (0.139-0.344)	0.212 (0.148-0.283)	0.182 (0.135-0.234)	0.149 (0.111-0.193)	0.118 (0.070-0.178)
Sudan	0.210 (0.116-0.322)	0.186 (0.122-0.259)	0.162 (0.113-0.216)	0.140 (0.100-0.187)	0.122 (0.076-0.176)
Suriname	0.105 (0.049-0.188)	0.078 (0.044-0.122)	0.069 (0.043-0.102)	0.057 (0.035-0.084)	0.047 (0.025-0.080)
Swaziland	0.182 (0.084-0.304)	0.146 (0.083-0.219)	0.114 (0.072-0.164)	0.089 (0.057-0.126)	0.070 (0.038-0.112)
Sweden	0.089 (0.051-0.140)	0.081 (0.057-0.110)	0.075 (0.055-0.097)	0.068 (0.050-0.088)	0.063 (0.039-0.092)
Switzerland	0.109 (0.057-0.181)	0.090 (0.059-0.132)	0.073 (0.051-0.102)	0.068 (0.048-0.094)	0.066 (0.040-0.101)
Syrian Arab Republic	0.114 (0.053-0.208)	0.084 (0.050-0.132)	0.069 (0.045-0.100)	0.052 (0.034-0.075)	0.040 (0.022-0.066)
Taiwan	0.159 (0.082-0.253)	0.130 (0.084-0.184)	0.112 (0.079-0.151)	0.104 (0.073-0.142)	0.106 (0.060-0.165)
Tajikistan	0.129 (0.053-0.239)	0.111 (0.059-0.184)	0.098 (0.059-0.146)	0.086 (0.054-0.127)	0.068 (0.037-0.110)
Tanzania	0.215 (0.120-0.326)	0.189 (0.128-0.259)	0.168 (0.124-0.216)	0.147 (0.112-0.186)	0.125 (0.084-0.172)
Thailand	0.244 (0.146-0.354)	0.210 (0.150-0.276)	0.178 (0.133-0.222)	0.142 (0.106-0.177)	0.117 (0.074-0.166)
Timor-Leste	0.261 (0.162-0.366)	0.239 (0.170-0.313)	0.214 (0.159-0.274)	0.191 (0.143-0.242)	0.170 (0.113-0.233)
Togo	0.215 (0.124-0.324)	0.190 (0.130-0.262)	0.166 (0.121-0.216)	0.143 (0.107-0.181)	0.124 (0.083-0.173)
Tokelau	0.070 (0.021-0.163)	0.046 (0.019-0.089)	0.030 (0.014-0.052)	0.019 (0.010-0.034)	0.013 (0.005-0.027)
Tonga	0.044 (0.016-0.091)	0.030 (0.014-0.053)	0.020 (0.010-0.034)	0.014 (0.007-0.023)	0.010 (0.004-0.019)
Trinidad and Tobago	0.153 (0.063-0.281)	0.119 (0.061-0.199)	0.091 (0.056-0.134)	0.062 (0.035-0.095)	0.042 (0.016-0.083)
Tunisia	0.108 (0.050-0.196)	0.081 (0.049-0.126)	0.063 (0.042-0.091)	0.048 (0.032-0.068)	0.037 (0.021-0.059)
Turkey	0.108 (0.051-0.191)	0.078 (0.048-0.116)	0.055 (0.039-0.074)	0.040 (0.029-0.053)	0.031 (0.019-0.046)
Turkmenistan	0.115 (0.047-0.219)	0.099 (0.053-0.161)	0.084 (0.050-0.126)	0.071 (0.045-0.104)	0.055 (0.030-0.091)
Tuvalu	0.051 (0.021-0.098)	0.035 (0.017-0.060)	0.023 (0.012-0.039)	0.017 (0.009-0.029)	0.014 (0.006-0.027)
Uganda	0.225 (0.124-0.345)	0.206 (0.132-0.291)	0.183 (0.125-0.250)	0.159 (0.112-0.211)	0.131 (0.086-0.188)
Ukraine	0.073 (0.032-0.133)	0.062 (0.036-0.097)	0.060 (0.038-0.088)	0.061 (0.039-0.088)	0.057 (0.030-0.093)
United Arab Emirates	0.059 (0.019-0.130)	0.044 (0.023-0.077)	0.036 (0.022-0.055)	0.028 (0.018-0.042)	0.026 (0.014-0.043)
United Kingdom	0.085 (0.050-0.130)	0.072 (0.053-0.093)	0.061 (0.049-0.074)	0.051 (0.041-0.062)	0.044 (0.031-0.060)
United States of America	0.088 (0.056-0.127)	0.072 (0.053-0.095)	0.059 (0.045-0.077)	0.049 (0.037-0.063)	0.043 (0.028-0.061)
Uruguay	0.071 (0.028-0.137)	0.062 (0.033-0.102)	0.056 (0.034-0.085)	0.052 (0.033-0.076)	0.049 (0.027-0.078)
Uzbekistan	0.126 (0.054-0.236)	0.104 (0.055-0.173)	0.089 (0.054-0.133)	0.076 (0.047-0.111)	0.060 (0.031-0.099)
Vanuatu	0.106 (0.036-0.214)	0.080 (0.036-0.143)	0.061 (0.033-0.100)	0.048 (0.028-0.076)	0.039 (0.019-0.069)
Venezuela	0.071 (0.031-0.136)	0.057 (0.032-0.094)	0.048 (0.030-0.072)	0.042 (0.028-0.060)	0.038 (0.022-0.062)
Viet Nam	0.245 (0.154-0.346)	0.229 (0.170-0.294)	0.207 (0.166-0.253)	0.178 (0.144-0.217)	0.147 (0.101-0.201)
Yemen	0.165 (0.078-0.289)	0.134 (0.078-0.205)	0.105 (0.069-0.151)	0.081 (0.054-0.116)	0.065 (0.037-0.103)
Zambia	0.184 (0.100-0.287)	0.160 (0.106-0.225)	0.148 (0.107-0.196)	0.133 (0.099-0.173)	0.112 (0.074-0.163)
Zimbabwe	0.165 (0.077-0.272)	0.134 (0.081-0.198)	0.110 (0.074-0.150)	0.092 (0.064-0.125)	0.081 (0.048-0.126)

Men, 20 ≤ BMI < 25 kg/m^2

Country	Year				
	1975	1985	1995	2005	2014
Afghanistan	0.419 (0.299-0.552)	0.437 (0.342-0.542)	0.456 (0.371-0.548)	0.474 (0.388-0.561)	0.481 (0.383-0.584)
Albania	0.543 (0.425-0.655)	0.527 (0.447-0.608)	0.509 (0.440-0.578)	0.448 (0.389-0.506)	0.384 (0.310-0.461)
Algeria	0.485 (0.373-0.596)	0.469 (0.394-0.543)	0.449 (0.390-0.507)	0.423 (0.374-0.473)	0.386 (0.321-0.454)
American Samoa	0.328 (0.244-0.423)	0.284 (0.226-0.346)	0.241 (0.192-0.294)	0.210 (0.163-0.259)	0.190 (0.136-0.251)
Andorra	0.459 (0.368-0.550)	0.410 (0.349-0.474)	0.364 (0.312-0.417)	0.309 (0.265-0.356)	0.284 (0.226-0.347)
Angola	0.442 (0.318-0.577)	0.450 (0.348-0.558)	0.463 (0.366-0.563)	0.466 (0.371-0.563)	0.467 (0.356-0.580)
Antigua and Barbuda	0.513 (0.394-0.628)	0.507 (0.421-0.587)	0.470 (0.397-0.539)	0.443 (0.374-0.509)	0.395 (0.304-0.488)
Argentina	0.481 (0.374-0.591)	0.447 (0.371-0.528)	0.410 (0.350-0.472)	0.362 (0.311-0.413)	0.309 (0.246-0.374)
Armenia	0.541 (0.399-0.675)	0.524 (0.421-0.623)	0.517 (0.430-0.604)	0.479 (0.405-0.551)	0.402 (0.322-0.490)
Australia	0.472 (0.397-0.549)	0.427 (0.380-0.475)	0.381 (0.343-0.419)	0.329 (0.296-0.363)	0.284 (0.236-0.334)
Austria	0.508 (0.427-0.590)	0.462 (0.411-0.516)	0.418 (0.370-0.467)	0.379 (0.336-0.425)	0.348 (0.284-0.416)
Azerbaijan	0.528 (0.408-0.640)	0.513 (0.422-0.601)	0.512 (0.429-0.590)	0.487 (0.413-0.556)	0.402 (0.318-0.488)
Bahamas	0.476 (0.372-0.580)	0.456 (0.383-0.532)	0.416 (0.355-0.481)	0.373 (0.319-0.429)	0.338 (0.269-0.410)
Bahrain	0.420 (0.315-0.524)	0.405 (0.341-0.471)	0.374 (0.324-0.426)	0.336 (0.294-0.381)	0.302 (0.244-0.362)
Bangladesh	0.403 (0.293-0.517)	0.414 (0.336-0.496)	0.426 (0.363-0.492)	0.443 (0.382-0.503)	0.458 (0.378-0.540)
Barbados	0.506 (0.401-0.613)	0.496 (0.424-0.568)	0.479 (0.420-0.539)	0.450 (0.389-0.508)	0.405 (0.322-0.490)
Belarus	0.537 (0.426-0.637)	0.502 (0.429-0.571)	0.469 (0.408-0.529)	0.426 (0.371-0.480)	0.360 (0.289-0.433)
Belgium	0.504 (0.414-0.590)	0.451 (0.400-0.500)	0.395 (0.356-0.436)	0.344 (0.306-0.381)	0.314 (0.261-0.372)
Belize	0.525 (0.400-0.644)	0.516 (0.432-0.600)	0.500 (0.434-0.566)	0.468 (0.409-0.527)	0.430 (0.351-0.508)
Benin	0.536 (0.410-0.657)	0.544 (0.451-0.634)	0.547 (0.472-0.621)	0.551 (0.485-0.615)	0.546 (0.467-0.625)
Bermuda	0.432 (0.321-0.545)	0.395 (0.315-0.481)	0.358 (0.291-0.429)	0.325 (0.267-0.385)	0.272 (0.206-0.345)
Bhutan	0.463 (0.338-0.593)	0.478 (0.384-0.575)	0.491 (0.410-0.576)	0.507 (0.434-0.582)	0.514 (0.425-0.599)
Bolivia	0.585 (0.457-0.701)	0.571 (0.478-0.660)	0.544 (0.462-0.622)	0.500 (0.430-0.569)	0.452 (0.367-0.535)
Bosnia and Herzegovina	0.601 (0.442-0.742)	0.586 (0.480-0.686)	0.559 (0.476-0.638)	0.471 (0.406-0.536)	0.417 (0.343-0.490)
Botswana	0.472 (0.351-0.601)	0.477 (0.384-0.572)	0.481 (0.400-0.564)	0.476 (0.401-0.550)	0.459 (0.371-0.545)
Brazil	0.543 (0.439-0.647)	0.517 (0.452-0.585)	0.483 (0.429-0.534)	0.439 (0.395-0.483)	0.386 (0.323-0.449)
Brunei Darussalam	0.450 (0.295-0.603)	0.462 (0.343-0.580)	0.460 (0.369-0.546)	0.440 (0.365-0.514)	0.409 (0.315-0.504)
Bulgaria	0.491 (0.389-0.590)	0.440 (0.375-0.505)	0.404 (0.348-0.460)	0.383 (0.331-0.435)	0.339 (0.272-0.411)
Burkina Faso	0.513 (0.383-0.645)	0.523 (0.429-0.616)	0.525 (0.449-0.601)	0.530 (0.463-0.597)	0.531 (0.449-0.611)
Burundi	0.469 (0.323-0.622)	0.484 (0.364-0.607)	0.502 (0.397-0.608)	0.518 (0.426-0.615)	0.527 (0.425-0.632)
Cabo Verde	0.520 (0.386-0.657)	0.529 (0.435-0.623)	0.527 (0.450-0.607)	0.519 (0.452-0.585)	0.496 (0.411-0.577)
Cambodia	0.458 (0.328-0.590)	0.466 (0.376-0.559)	0.476 (0.400-0.556)	0.497 (0.428-0.568)	0.521 (0.436-0.606)
Cameroon	0.554 (0.437-0.666)	0.562 (0.480-0.640)	0.564 (0.500-0.627)	0.560 (0.505-0.613)	0.552 (0.474-0.628)
Canada	0.490 (0.412-0.567)	0.442 (0.395-0.489)	0.389 (0.348-0.432)	0.331 (0.295-0.369)	0.293 (0.241-0.348)
Central African Republic	0.471 (0.332-0.621)	0.480 (0.365-0.598)	0.475 (0.376-0.576)	0.479 (0.387-0.573)	0.480 (0.374-0.587)
Chad	0.505 (0.381-0.630)	0.522 (0.430-0.618)	0.532 (0.451-0.610)	0.542 (0.469-0.614)	0.534 (0.445-0.620)
Chile	0.528 (0.413-0.642)	0.494 (0.413-0.574)	0.444 (0.386-0.503)	0.382 (0.335-0.430)	0.329 (0.270-0.392)
China	0.596 (0.497-0.690)	0.593 (0.547-0.639)	0.583 (0.551-0.614)	0.558 (0.524-0.591)	0.503 (0.443-0.559)
China (Hong Kong SAR)	0.530 (0.401-0.651)	0.514 (0.432-0.596)	0.492 (0.423-0.563)	0.456 (0.388-0.524)	0.396 (0.310-0.492)
Colombia	0.546 (0.430-0.658)	0.523 (0.447-0.601)	0.487 (0.429-0.547)	0.443 (0.397-0.491)	0.397 (0.332-0.464)
Comoros	0.538 (0.415-0.661)	0.543 (0.447-0.638)	0.548 (0.464-0.627)	0.553 (0.480-0.625)	0.554 (0.468-0.637)
Congo	0.465 (0.346-0.585)	0.474 (0.383-0.565)	0.480 (0.398-0.562)	0.472 (0.389-0.552)	0.463 (0.363-0.563)
Cook Islands	0.383 (0.282-0.497)	0.326 (0.264-0.393)	0.265 (0.211-0.323)	0.216 (0.171-0.264)	0.183 (0.134-0.239)
Costa Rica	0.536 (0.419-0.649)	0.513 (0.434-0.590)	0.472 (0.411-0.534)	0.422 (0.370-0.470)	0.367 (0.301-0.433)
Cote d'Ivoire	0.547 (0.430-0.662)	0.558 (0.472-0.642)	0.561 (0.488-0.632)	0.561 (0.498-0.622)	0.552 (0.473-0.626)
Croatia	0.529 (0.414-0.637)	0.491 (0.420-0.562)	0.456 (0.396-0.517)	0.398 (0.345-0.453)	0.336 (0.268-0.407)
Cuba	0.479 (0.373-0.585)	0.458 (0.385-0.532)	0.442 (0.380-0.506)	0.424 (0.367-0.482)	0.375 (0.304-0.450)
Cyprus	0.537 (0.434-0.635)	0.494 (0.427-0.560)	0.422 (0.368-0.479)	0.367 (0.319-0.417)	0.334 (0.268-0.406)
Czech Republic	0.449 (0.352-0.543)	0.402 (0.343-0.462)	0.367 (0.316-0.418)	0.330 (0.285-0.376)	0.293 (0.231-0.356)
Denmark	0.514 (0.438-0.586)	0.467 (0.422-0.514)	0.410 (0.368-0.453)	0.358 (0.317-0.401)	0.330 (0.273-0.390)
Djibouti	0.499 (0.363-0.636)	0.510 (0.388-0.629)	0.504 (0.407-0.599)	0.495 (0.403-0.584)	0.479 (0.370-0.587)
Dominica	0.526 (0.409-0.640)	0.526 (0.443-0.610)	0.489 (0.422-0.555)	0.454 (0.393-0.514)	0.407 (0.333-0.483)
Dominican Republic	0.533 (0.416-0.648)	0.525 (0.446-0.602)	0.508 (0.444-0.571)	0.466 (0.409-0.521)	0.409 (0.331-0.486)
DR Congo	0.454 (0.329-0.582)	0.464 (0.363-0.568)	0.470 (0.376-0.566)	0.471 (0.378-0.562)	0.471 (0.360-0.582)
Ecuador	0.591 (0.465-0.709)	0.570 (0.478-0.659)	0.533 (0.454-0.610)	0.488 (0.420-0.556)	0.431 (0.351-0.513)
Egypt	0.529 (0.410-0.641)	0.497 (0.428-0.565)	0.468 (0.415-0.520)	0.435 (0.389-0.480)	0.389 (0.325-0.457)
El Salvador	0.552 (0.432-0.667)	0.538 (0.451-0.620)	0.510 (0.442-0.576)	0.463 (0.407-0.519)	0.417 (0.344-0.490)
Equatorial Guinea	0.474 (0.333-0.622)	0.483 (0.371-0.599)	0.488 (0.386-0.594)	0.468 (0.362-0.574)	0.446 (0.317-0.577)
Eritrea	0.462 (0.344-0.587)	0.472 (0.382-0.565)	0.481 (0.404-0.558)	0.486 (0.417-0.555)	0.489 (0.403-0.574)
Estonia	0.526 (0.423-0.622)	0.495 (0.435-0.555)	0.473 (0.424-0.522)	0.420 (0.381-0.461)	0.366 (0.306-0.427)
Ethiopia	0.475 (0.349-0.608)	0.486 (0.390-0.584)	0.491 (0.408-0.574)	0.489 (0.419-0.560)	0.488 (0.400-0.575)
Fiji	0.495 (0.356-0.622)	0.459 (0.355-0.560)	0.425 (0.337-0.517)	0.384 (0.309-0.460)	0.358 (0.276-0.441)
Finland	0.540 (0.459-0.621)	0.486 (0.440-0.534)	0.432 (0.391-0.473)	0.389 (0.353-0.426)	0.360 (0.305-0.418)
France	0.471 (0.382-0.556)	0.426 (0.370-0.483)	0.381 (0.335-0.429)	0.337 (0.293-0.382)	0.308 (0.249-0.371)
French Polynesia	0.304 (0.212-0.405)	0.262 (0.194-0.335)	0.223 (0.169-0.284)	0.193 (0.148-0.242)	0.167 (0.120-0.220)

Gabon	0.470 (0.342-0.605)	0.481 (0.375-0.587)	0.480 (0.380-0.574)	0.466 (0.375-0.556)	0.441 (0.339-0.544)
Gambia	0.508 (0.387-0.628)	0.515 (0.425-0.600)	0.519 (0.443-0.591)	0.522 (0.456-0.587)	0.511 (0.427-0.593)
Georgia	0.528 (0.403-0.649)	0.502 (0.410-0.592)	0.507 (0.419-0.587)	0.468 (0.393-0.538)	0.419 (0.339-0.500)
Germany	0.516 (0.437-0.592)	0.454 (0.411-0.497)	0.405 (0.368-0.442)	0.373 (0.338-0.408)	0.341 (0.286-0.399)
Ghana	0.538 (0.424-0.651)	0.548 (0.466-0.630)	0.553 (0.485-0.621)	0.563 (0.499-0.626)	0.552 (0.470-0.629)
Greece	0.495 (0.404-0.586)	0.439 (0.380-0.499)	0.392 (0.341-0.444)	0.344 (0.299-0.391)	0.311 (0.250-0.376)
Greenland	0.502 (0.421-0.586)	0.449 (0.395-0.504)	0.403 (0.357-0.451)	0.359 (0.319-0.402)	0.321 (0.264-0.386)
Grenada	0.535 (0.419-0.649)	0.531 (0.448-0.611)	0.509 (0.442-0.574)	0.480 (0.419-0.538)	0.435 (0.356-0.516)
Guatemala	0.548 (0.426-0.667)	0.538 (0.454-0.620)	0.516 (0.449-0.581)	0.482 (0.425-0.538)	0.443 (0.365-0.520)
Guinea	0.519 (0.383-0.656)	0.524 (0.426-0.621)	0.524 (0.441-0.606)	0.531 (0.461-0.600)	0.533 (0.450-0.614)
Guinea Bissau	0.515 (0.390-0.641)	0.517 (0.423-0.616)	0.518 (0.435-0.601)	0.524 (0.449-0.598)	0.524 (0.435-0.611)
Guyana	0.515 (0.401-0.627)	0.517 (0.433-0.599)	0.520 (0.449-0.587)	0.492 (0.428-0.557)	0.450 (0.364-0.534)
Haiti	0.523 (0.395-0.648)	0.535 (0.440-0.632)	0.538 (0.452-0.623)	0.527 (0.450-0.602)	0.502 (0.404-0.598)
Honduras	0.556 (0.434-0.678)	0.546 (0.460-0.634)	0.523 (0.455-0.591)	0.486 (0.429-0.543)	0.444 (0.365-0.520)
Hungary	0.457 (0.362-0.545)	0.410 (0.353-0.466)	0.376 (0.324-0.429)	0.349 (0.303-0.395)	0.308 (0.247-0.373)
Iceland	0.524 (0.438-0.609)	0.463 (0.405-0.525)	0.419 (0.369-0.472)	0.377 (0.331-0.424)	0.336 (0.273-0.403)
India	0.369 (0.275-0.463)	0.388 (0.327-0.448)	0.401 (0.358-0.443)	0.418 (0.380-0.456)	0.432 (0.369-0.500)
Indonesia	0.452 (0.350-0.555)	0.461 (0.395-0.530)	0.472 (0.419-0.527)	0.489 (0.439-0.540)	0.494 (0.421-0.566)
Iran	0.467 (0.359-0.568)	0.457 (0.394-0.517)	0.442 (0.399-0.485)	0.406 (0.375-0.439)	0.356 (0.300-0.411)
Iraq	0.489 (0.376-0.598)	0.464 (0.391-0.535)	0.448 (0.387-0.507)	0.432 (0.382-0.482)	0.387 (0.320-0.455)
Ireland	0.510 (0.416-0.604)	0.462 (0.397-0.527)	0.410 (0.355-0.462)	0.344 (0.296-0.390)	0.309 (0.251-0.373)
Israel	0.470 (0.373-0.569)	0.424 (0.360-0.488)	0.373 (0.322-0.424)	0.328 (0.283-0.375)	0.294 (0.236-0.357)
Italy	0.490 (0.408-0.574)	0.446 (0.399-0.493)	0.401 (0.365-0.440)	0.353 (0.317-0.389)	0.321 (0.263-0.379)
Jamaica	0.495 (0.392-0.600)	0.497 (0.430-0.566)	0.484 (0.430-0.540)	0.455 (0.405-0.507)	0.413 (0.341-0.483)
Japan	0.597 (0.543-0.647)	0.609 (0.577-0.640)	0.606 (0.577-0.635)	0.594 (0.565-0.624)	0.576 (0.518-0.630)
Jordan	0.514 (0.397-0.631)	0.473 (0.406-0.540)	0.430 (0.375-0.482)	0.385 (0.340-0.430)	0.335 (0.274-0.400)
Kazakhstan	0.510 (0.390-0.624)	0.490 (0.402-0.574)	0.468 (0.391-0.544)	0.431 (0.362-0.500)	0.362 (0.280-0.447)
Kenya	0.486 (0.369-0.612)	0.503 (0.413-0.599)	0.515 (0.434-0.597)	0.519 (0.443-0.593)	0.520 (0.427-0.611)
Kiribati	0.421 (0.314-0.532)	0.395 (0.318-0.478)	0.362 (0.292-0.437)	0.315 (0.258-0.380)	0.295 (0.222-0.380)
Kuwait	0.399 (0.288-0.505)	0.368 (0.305-0.430)	0.336 (0.291-0.383)	0.281 (0.241-0.320)	0.249 (0.198-0.304)
Kyrgyzstan	0.549 (0.413-0.677)	0.540 (0.443-0.631)	0.530 (0.447-0.609)	0.516 (0.440-0.589)	0.475 (0.388-0.560)
Lao PDR	0.458 (0.347-0.573)	0.472 (0.389-0.558)	0.485 (0.412-0.562)	0.504 (0.439-0.575)	0.520 (0.438-0.602)
Latvia	0.511 (0.407-0.611)	0.475 (0.407-0.538)	0.453 (0.396-0.510)	0.412 (0.362-0.462)	0.339 (0.271-0.408)
Lebanon	0.479 (0.373-0.579)	0.446 (0.377-0.516)	0.395 (0.336-0.457)	0.343 (0.287-0.399)	0.319 (0.258-0.385)
Lesotho	0.491 (0.363-0.626)	0.498 (0.404-0.596)	0.504 (0.422-0.585)	0.506 (0.433-0.578)	0.503 (0.417-0.586)
Liberia	0.543 (0.421-0.661)	0.550 (0.457-0.643)	0.563 (0.466-0.655)	0.564 (0.493-0.633)	0.559 (0.475-0.642)
Libya	0.448 (0.328-0.566)	0.405 (0.339-0.471)	0.379 (0.328-0.431)	0.355 (0.312-0.397)	0.329 (0.270-0.391)
Lithuania	0.529 (0.425-0.622)	0.489 (0.423-0.554)	0.459 (0.403-0.515)	0.411 (0.361-0.460)	0.341 (0.274-0.408)
Luxembourg	0.503 (0.421-0.589)	0.451 (0.393-0.514)	0.392 (0.334-0.454)	0.346 (0.300-0.394)	0.309 (0.250-0.377)
Macedonia (TFYR)	0.516 (0.407-0.621)	0.483 (0.411-0.555)	0.453 (0.389-0.519)	0.414 (0.359-0.472)	0.367 (0.294-0.441)
Madagascar	0.486 (0.372-0.606)	0.500 (0.413-0.589)	0.510 (0.434-0.587)	0.514 (0.444-0.584)	0.511 (0.420-0.599)
Malawi	0.507 (0.374-0.643)	0.517 (0.423-0.614)	0.521 (0.441-0.603)	0.530 (0.458-0.601)	0.539 (0.453-0.624)
Malaysia	0.480 (0.374-0.586)	0.500 (0.424-0.575)	0.508 (0.445-0.570)	0.493 (0.442-0.544)	0.458 (0.385-0.530)
Maldives	0.503 (0.385-0.621)	0.512 (0.424-0.601)	0.534 (0.452-0.615)	0.546 (0.464-0.625)	0.518 (0.431-0.604)
Mali	0.510 (0.381-0.637)	0.518 (0.425-0.610)	0.513 (0.432-0.592)	0.515 (0.442-0.584)	0.513 (0.428-0.598)
Malta	0.487 (0.357-0.613)	0.423 (0.353-0.492)	0.377 (0.323-0.432)	0.322 (0.274-0.370)	0.284 (0.222-0.351)
Marshall Islands	0.385 (0.279-0.505)	0.346 (0.274-0.427)	0.298 (0.240-0.359)	0.267 (0.212-0.326)	0.243 (0.176-0.321)
Mauritania	0.498 (0.378-0.623)	0.509 (0.415-0.604)	0.508 (0.427-0.593)	0.507 (0.433-0.579)	0.497 (0.407-0.586)
Mauritius	0.515 (0.403-0.626)	0.522 (0.446-0.599)	0.515 (0.448-0.582)	0.495 (0.431-0.559)	0.467 (0.377-0.554)
Mexico	0.525 (0.415-0.632)	0.482 (0.413-0.555)	0.431 (0.379-0.484)	0.377 (0.335-0.417)	0.328 (0.272-0.388)
Micronesia (Federated States of)	0.416 (0.308-0.532)	0.385 (0.311-0.465)	0.350 (0.286-0.417)	0.317 (0.265-0.374)	0.288 (0.221-0.360)
Moldova	0.549 (0.436-0.652)	0.528 (0.452-0.601)	0.519 (0.455-0.584)	0.508 (0.446-0.569)	0.452 (0.375-0.529)
Mongolia	0.551 (0.412-0.682)	0.536 (0.441-0.628)	0.522 (0.441-0.599)	0.494 (0.424-0.563)	0.438 (0.357-0.519)
Montenegro	0.490 (0.366-0.609)	0.451 (0.373-0.530)	0.424 (0.363-0.484)	0.382 (0.331-0.434)	0.339 (0.271-0.410)
Morocco	0.513 (0.392-0.632)	0.502 (0.423-0.580)	0.480 (0.416-0.543)	0.452 (0.397-0.506)	0.412 (0.339-0.483)
Mozambique	0.525 (0.380-0.666)	0.533 (0.423-0.640)	0.533 (0.445-0.620)	0.533 (0.458-0.608)	0.529 (0.440-0.619)
Myanmar	0.459 (0.315-0.612)	0.468 (0.368-0.573)	0.477 (0.399-0.555)	0.488 (0.426-0.552)	0.506 (0.433-0.584)
Namibia	0.478 (0.355-0.606)	0.490 (0.397-0.583)	0.496 (0.416-0.573)	0.497 (0.428-0.566)	0.487 (0.403-0.569)
Nauru	0.262 (0.183-0.354)	0.222 (0.169-0.279)	0.203 (0.156-0.253)	0.202 (0.154-0.255)	0.191 (0.129-0.264)
Nepal	0.440 (0.323-0.564)	0.455 (0.368-0.549)	0.469 (0.398-0.547)	0.488 (0.422-0.557)	0.501 (0.418-0.586)
Netherlands	0.518 (0.435-0.598)	0.484 (0.433-0.535)	0.440 (0.396-0.484)	0.384 (0.346-0.423)	0.348 (0.292-0.405)
New Zealand	0.481 (0.396-0.564)	0.436 (0.381-0.490)	0.387 (0.343-0.431)	0.338 (0.302-0.373)	0.291 (0.246-0.340)
Nicaragua	0.541 (0.425-0.657)	0.533 (0.451-0.617)	0.523 (0.455-0.593)	0.491 (0.433-0.548)	0.453 (0.372-0.533)
Niger	0.487 (0.363-0.614)	0.492 (0.398-0.588)	0.498 (0.418-0.578)	0.504 (0.433-0.574)	0.510 (0.421-0.597)
Nigeria	0.499 (0.390-0.609)	0.516 (0.442-0.587)	0.519 (0.461-0.577)	0.525 (0.476-0.574)	0.515 (0.446-0.582)
Niue	0.414 (0.298-0.538)	0.357 (0.281-0.440)	0.303 (0.242-0.369)	0.253 (0.204-0.306)	0.206 (0.152-0.267)
North Korea	0.567 (0.440-0.688)	0.573 (0.480-0.659)	0.573 (0.487-0.652)	0.569 (0.476-0.658)	0.563 (0.444-0.673)
Norway	0.514 (0.433-0.596)	0.464 (0.413-0.515)	0.415 (0.372-0.460)	0.361 (0.322-0.402)	0.323 (0.266-0.383)

Occupied Palestinian Territory	0.499 (0.380-0.609)	0.482 (0.402-0.561)	0.461 (0.397-0.524)	0.425 (0.373-0.479)	0.397 (0.321-0.477)
Oman	0.462 (0.344-0.576)	0.441 (0.370-0.510)	0.403 (0.351-0.455)	0.366 (0.323-0.410)	0.325 (0.263-0.390)
Pakistan	0.426 (0.316-0.539)	0.444 (0.366-0.522)	0.458 (0.393-0.525)	0.472 (0.408-0.536)	0.479 (0.395-0.561)
Palau	0.330 (0.230-0.438)	0.296 (0.235-0.362)	0.255 (0.202-0.312)	0.220 (0.176-0.270)	0.195 (0.144-0.253)
Panama	0.531 (0.417-0.646)	0.509 (0.432-0.586)	0.475 (0.415-0.539)	0.427 (0.377-0.475)	0.369 (0.302-0.438)
Papua New Guinea	0.570 (0.426-0.701)	0.562 (0.456-0.662)	0.540 (0.447-0.629)	0.520 (0.436-0.603)	0.492 (0.395-0.586)
Paraguay	0.584 (0.463-0.698)	0.562 (0.478-0.644)	0.525 (0.457-0.593)	0.477 (0.416-0.536)	0.428 (0.347-0.502)
Peru	0.585 (0.467-0.696)	0.563 (0.472-0.649)	0.531 (0.454-0.606)	0.489 (0.425-0.551)	0.430 (0.354-0.504)
Philippines	0.488 (0.380-0.595)	0.502 (0.426-0.575)	0.512 (0.448-0.577)	0.515 (0.455-0.573)	0.510 (0.428-0.590)
Poland	0.474 (0.378-0.563)	0.440 (0.382-0.497)	0.411 (0.362-0.459)	0.367 (0.325-0.409)	0.321 (0.264-0.381)
Portugal	0.543 (0.441-0.638)	0.518 (0.453-0.580)	0.465 (0.413-0.515)	0.397 (0.353-0.442)	0.353 (0.291-0.419)
Puerto Rico	0.507 (0.402-0.612)	0.480 (0.405-0.554)	0.425 (0.357-0.488)	0.369 (0.312-0.426)	0.320 (0.252-0.393)
Qatar	0.448 (0.339-0.556)	0.373 (0.299-0.449)	0.343 (0.289-0.396)	0.290 (0.244-0.337)	0.248 (0.193-0.309)
Romania	0.500 (0.390-0.605)	0.455 (0.389-0.523)	0.430 (0.374-0.486)	0.390 (0.340-0.441)	0.332 (0.266-0.400)
Russian Federation	0.516 (0.421-0.602)	0.488 (0.438-0.537)	0.466 (0.428-0.503)	0.428 (0.388-0.469)	0.360 (0.296-0.427)
Rwanda	0.504 (0.359-0.649)	0.520 (0.403-0.635)	0.533 (0.436-0.628)	0.547 (0.465-0.627)	0.554 (0.460-0.644)
Saint Kitts and Nevis	0.534 (0.421-0.643)	0.526 (0.445-0.606)	0.490 (0.422-0.556)	0.450 (0.385-0.513)	0.404 (0.318-0.491)
Saint Lucia	0.532 (0.421-0.638)	0.528 (0.451-0.601)	0.505 (0.443-0.568)	0.472 (0.409-0.534)	0.426 (0.338-0.515)
Saint Vincent and the Grenadines	0.541 (0.422-0.658)	0.536 (0.451-0.617)	0.512 (0.442-0.580)	0.467 (0.405-0.527)	0.415 (0.338-0.494)
Samoa	0.441 (0.327-0.562)	0.407 (0.326-0.490)	0.349 (0.285-0.416)	0.296 (0.244-0.352)	0.259 (0.196-0.329)
Sao Tome and Principe	0.561 (0.443-0.674)	0.571 (0.482-0.655)	0.581 (0.505-0.655)	0.582 (0.518-0.645)	0.565 (0.485-0.644)
Saudi Arabia	0.467 (0.349-0.584)	0.425 (0.357-0.489)	0.381 (0.335-0.423)	0.345 (0.304-0.384)	0.304 (0.247-0.367)
Senegal	0.497 (0.377-0.613)	0.506 (0.421-0.594)	0.513 (0.436-0.589)	0.513 (0.447-0.579)	0.508 (0.428-0.588)
Serbia	0.507 (0.402-0.607)	0.472 (0.406-0.538)	0.445 (0.388-0.501)	0.411 (0.362-0.459)	0.374 (0.307-0.438)
Seychelles	0.524 (0.417-0.631)	0.528 (0.450-0.607)	0.514 (0.444-0.584)	0.489 (0.423-0.556)	0.452 (0.367-0.538)
Sierra Leone	0.516 (0.395-0.636)	0.524 (0.431-0.614)	0.528 (0.450-0.605)	0.534 (0.469-0.599)	0.542 (0.464-0.617)
Singapore	0.587 (0.477-0.690)	0.590 (0.531-0.646)	0.576 (0.528-0.621)	0.554 (0.509-0.597)	0.521 (0.459-0.582)
Slovakia	0.502 (0.396-0.600)	0.462 (0.399-0.527)	0.421 (0.370-0.474)	0.384 (0.338-0.432)	0.334 (0.268-0.402)
Slovenia	0.505 (0.393-0.611)	0.466 (0.399-0.534)	0.428 (0.371-0.486)	0.376 (0.326-0.429)	0.334 (0.263-0.409)
Solomon Islands	0.610 (0.464-0.735)	0.591 (0.482-0.692)	0.572 (0.480-0.660)	0.560 (0.476-0.641)	0.535 (0.438-0.629)
Somalia	0.507 (0.371-0.642)	0.512 (0.406-0.618)	0.513 (0.420-0.606)	0.519 (0.432-0.607)	0.518 (0.413-0.619)
South Africa	0.486 (0.379-0.592)	0.490 (0.419-0.559)	0.486 (0.428-0.544)	0.468 (0.421-0.516)	0.434 (0.369-0.499)
South Korea	0.574 (0.477-0.664)	0.598 (0.542-0.651)	0.590 (0.543-0.637)	0.572 (0.531-0.613)	0.532 (0.471-0.590)
Spain	0.510 (0.418-0.599)	0.454 (0.401-0.507)	0.396 (0.356-0.437)	0.343 (0.306-0.380)	0.311 (0.255-0.370)
Sri Lanka	0.471 (0.362-0.583)	0.484 (0.405-0.565)	0.495 (0.427-0.563)	0.509 (0.446-0.572)	0.514 (0.422-0.602)
Sudan	0.512 (0.387-0.638)	0.520 (0.428-0.613)	0.521 (0.438-0.602)	0.519 (0.444-0.591)	0.512 (0.421-0.600)
Suriname	0.496 (0.388-0.607)	0.482 (0.404-0.560)	0.465 (0.397-0.534)	0.434 (0.373-0.494)	0.390 (0.314-0.467)
Swaziland	0.499 (0.375-0.628)	0.509 (0.414-0.603)	0.512 (0.431-0.595)	0.512 (0.436-0.587)	0.505 (0.408-0.598)
Sweden	0.521 (0.442-0.595)	0.476 (0.431-0.521)	0.432 (0.395-0.470)	0.388 (0.353-0.423)	0.350 (0.296-0.409)
Switzerland	0.487 (0.394-0.581)	0.445 (0.384-0.504)	0.393 (0.349-0.439)	0.361 (0.322-0.401)	0.331 (0.276-0.391)
Syrian Arab Republic	0.507 (0.389-0.621)	0.471 (0.399-0.546)	0.457 (0.398-0.517)	0.427 (0.378-0.476)	0.397 (0.327-0.466)
Taiwan	0.558 (0.462-0.653)	0.561 (0.497-0.624)	0.543 (0.488-0.599)	0.507 (0.450-0.562)	0.478 (0.397-0.559)
Tajikistan	0.535 (0.411-0.655)	0.534 (0.438-0.627)	0.541 (0.456-0.624)	0.548 (0.467-0.624)	0.512 (0.412-0.605)
Tanzania	0.526 (0.400-0.655)	0.531 (0.442-0.624)	0.533 (0.461-0.605)	0.531 (0.469-0.592)	0.533 (0.456-0.608)
Thailand	0.478 (0.375-0.582)	0.496 (0.425-0.563)	0.512 (0.455-0.568)	0.522 (0.471-0.571)	0.506 (0.432-0.580)
Timor-Leste	0.451 (0.339-0.570)	0.454 (0.369-0.545)	0.464 (0.390-0.543)	0.477 (0.404-0.552)	0.484 (0.394-0.577)
Togo	0.537 (0.411-0.659)	0.547 (0.455-0.640)	0.549 (0.472-0.627)	0.554 (0.487-0.622)	0.553 (0.468-0.633)
Tokelau	0.419 (0.266-0.579)	0.388 (0.283-0.497)	0.347 (0.271-0.428)	0.303 (0.240-0.371)	0.262 (0.186-0.349)
Tonga	0.399 (0.285-0.520)	0.362 (0.284-0.442)	0.318 (0.253-0.385)	0.275 (0.221-0.331)	0.246 (0.183-0.316)
Trinidad and Tobago	0.506 (0.367-0.642)	0.502 (0.399-0.602)	0.504 (0.427-0.576)	0.469 (0.393-0.542)	0.409 (0.300-0.520)
Tunisia	0.489 (0.376-0.602)	0.467 (0.397-0.541)	0.442 (0.387-0.498)	0.409 (0.366-0.456)	0.371 (0.309-0.433)
Turkey	0.495 (0.390-0.595)	0.468 (0.405-0.529)	0.422 (0.376-0.468)	0.376 (0.338-0.413)	0.331 (0.279-0.385)
Turkmenistan	0.533 (0.404-0.652)	0.523 (0.430-0.615)	0.502 (0.423-0.579)	0.481 (0.411-0.551)	0.419 (0.338-0.503)
Tuvalu	0.445 (0.329-0.571)	0.402 (0.323-0.487)	0.339 (0.277-0.407)	0.293 (0.240-0.351)	0.261 (0.195-0.334)
Uganda	0.536 (0.399-0.674)	0.547 (0.442-0.651)	0.554 (0.463-0.645)	0.557 (0.478-0.637)	0.562 (0.478-0.644)
Ukraine	0.524 (0.418-0.623)	0.491 (0.422-0.557)	0.466 (0.407-0.526)	0.447 (0.390-0.508)	0.386 (0.311-0.464)
United Arab Emirates	0.383 (0.257-0.510)	0.356 (0.282-0.432)	0.328 (0.279-0.381)	0.296 (0.253-0.340)	0.285 (0.228-0.345)
United Kingdom	0.474 (0.395-0.550)	0.439 (0.394-0.483)	0.394 (0.363-0.427)	0.342 (0.315-0.368)	0.297 (0.255-0.341)
United States of America	0.453 (0.378-0.528)	0.410 (0.363-0.455)	0.353 (0.315-0.391)	0.293 (0.261-0.325)	0.252 (0.208-0.300)
Uruguay	0.507 (0.384-0.626)	0.477 (0.397-0.560)	0.440 (0.377-0.502)	0.388 (0.335-0.443)	0.335 (0.268-0.404)
Uzbekistan	0.535 (0.400-0.664)	0.527 (0.426-0.624)	0.514 (0.429-0.594)	0.506 (0.430-0.578)	0.461 (0.371-0.552)
Vanuatu	0.555 (0.410-0.684)	0.549 (0.443-0.649)	0.530 (0.438-0.616)	0.504 (0.425-0.583)	0.473 (0.379-0.565)
Venezuela	0.523 (0.413-0.632)	0.480 (0.406-0.553)	0.441 (0.382-0.501)	0.399 (0.351-0.446)	0.345 (0.282-0.412)
Viet Nam	0.418 (0.318-0.523)	0.424 (0.360-0.491)	0.436 (0.384-0.486)	0.458 (0.410-0.504)	0.480 (0.407-0.549)
Yemen	0.521 (0.392-0.645)	0.528 (0.437-0.617)	0.525 (0.452-0.596)	0.516 (0.451-0.581)	0.499 (0.414-0.584)
Zambia	0.490 (0.376-0.606)	0.496 (0.408-0.585)	0.502 (0.424-0.580)	0.509 (0.441-0.577)	0.514 (0.430-0.598)
Zimbabwe	0.492 (0.376-0.609)	0.504 (0.422-0.587)	0.509 (0.440-0.581)	0.514 (0.448-0.578)	0.513 (0.430-0.595)

Women, 20 ≤ BMI < 25 kg/m^2

Country	Year				
	1975	1985	1995	2005	2014
Afghanistan	0.399 (0.285-0.521)	0.419 (0.331-0.514)	0.435 (0.361-0.514)	0.444 (0.375-0.514)	0.440 (0.361-0.522)
Albania	0.487 (0.367-0.604)	0.481 (0.402-0.561)	0.482 (0.418-0.545)	0.463 (0.407-0.518)	0.445 (0.369-0.522)
Algeria	0.412 (0.296-0.530)	0.389 (0.317-0.466)	0.364 (0.313-0.416)	0.329 (0.287-0.373)	0.301 (0.242-0.362)
American Samoa	0.223 (0.157-0.298)	0.198 (0.152-0.247)	0.173 (0.134-0.214)	0.155 (0.118-0.196)	0.145 (0.099-0.198)
Andorra	0.451 (0.357-0.546)	0.427 (0.361-0.492)	0.407 (0.354-0.462)	0.374 (0.325-0.423)	0.360 (0.291-0.431)
Angola	0.458 (0.329-0.590)	0.465 (0.366-0.565)	0.463 (0.379-0.546)	0.442 (0.363-0.522)	0.412 (0.318-0.507)
Antigua and Barbuda	0.436 (0.325-0.548)	0.403 (0.327-0.482)	0.359 (0.298-0.422)	0.320 (0.261-0.383)	0.281 (0.202-0.369)
Argentina	0.474 (0.369-0.575)	0.451 (0.376-0.523)	0.418 (0.360-0.476)	0.386 (0.338-0.437)	0.347 (0.283-0.416)
Armenia	0.481 (0.338-0.617)	0.461 (0.365-0.559)	0.450 (0.379-0.525)	0.418 (0.358-0.479)	0.385 (0.307-0.465)
Australia	0.526 (0.457-0.597)	0.497 (0.454-0.540)	0.452 (0.416-0.487)	0.398 (0.365-0.432)	0.354 (0.301-0.407)
Austria	0.537 (0.456-0.617)	0.521 (0.469-0.573)	0.496 (0.452-0.543)	0.462 (0.418-0.505)	0.434 (0.368-0.502)
Azerbaijan	0.455 (0.324-0.576)	0.438 (0.348-0.525)	0.434 (0.364-0.504)	0.408 (0.348-0.471)	0.352 (0.274-0.433)
Bahamas	0.388 (0.289-0.491)	0.354 (0.284-0.422)	0.320 (0.266-0.379)	0.287 (0.237-0.340)	0.267 (0.206-0.334)
Bahrain	0.305 (0.213-0.404)	0.304 (0.243-0.367)	0.283 (0.240-0.329)	0.259 (0.223-0.298)	0.240 (0.189-0.296)
Bangladesh	0.355 (0.259-0.457)	0.368 (0.299-0.438)	0.376 (0.323-0.430)	0.384 (0.336-0.433)	0.387 (0.319-0.457)
Barbados	0.445 (0.338-0.555)	0.422 (0.346-0.498)	0.389 (0.330-0.448)	0.341 (0.287-0.398)	0.302 (0.227-0.383)
Belarus	0.475 (0.359-0.584)	0.450 (0.376-0.524)	0.432 (0.374-0.490)	0.414 (0.358-0.468)	0.393 (0.319-0.468)
Belgium	0.515 (0.428-0.601)	0.493 (0.441-0.544)	0.464 (0.425-0.503)	0.434 (0.393-0.473)	0.413 (0.352-0.475)
Belize	0.472 (0.350-0.592)	0.448 (0.369-0.526)	0.408 (0.350-0.468)	0.364 (0.312-0.417)	0.328 (0.256-0.404)
Benin	0.516 (0.403-0.629)	0.519 (0.444-0.594)	0.502 (0.444-0.557)	0.472 (0.425-0.518)	0.435 (0.374-0.497)
Bermuda	0.331 (0.232-0.437)	0.308 (0.237-0.384)	0.284 (0.226-0.347)	0.257 (0.206-0.309)	0.230 (0.169-0.298)
Bhutan	0.428 (0.307-0.550)	0.446 (0.355-0.537)	0.455 (0.382-0.529)	0.458 (0.395-0.522)	0.449 (0.373-0.527)
Bolivia	0.535 (0.413-0.648)	0.512 (0.425-0.594)	0.469 (0.400-0.535)	0.419 (0.363-0.476)	0.374 (0.301-0.449)
Bosnia and Herzegovina	0.530 (0.378-0.669)	0.521 (0.421-0.616)	0.499 (0.424-0.571)	0.446 (0.383-0.507)	0.451 (0.379-0.525)
Botswana	0.483 (0.349-0.616)	0.454 (0.360-0.548)	0.403 (0.334-0.472)	0.356 (0.300-0.414)	0.312 (0.246-0.381)
Brazil	0.484 (0.379-0.582)	0.459 (0.395-0.525)	0.434 (0.387-0.481)	0.407 (0.367-0.448)	0.374 (0.312-0.436)
Brunei Darussalam	0.392 (0.236-0.550)	0.407 (0.293-0.519)	0.416 (0.338-0.496)	0.407 (0.340-0.474)	0.399 (0.314-0.492)
Bulgaria	0.464 (0.356-0.569)	0.452 (0.382-0.520)	0.445 (0.391-0.503)	0.427 (0.373-0.481)	0.410 (0.333-0.487)
Burkina Faso	0.489 (0.373-0.606)	0.496 (0.417-0.577)	0.492 (0.430-0.551)	0.472 (0.418-0.523)	0.442 (0.376-0.508)
Burundi	0.487 (0.360-0.617)	0.497 (0.395-0.602)	0.502 (0.417-0.587)	0.498 (0.426-0.570)	0.481 (0.402-0.560)
Cabo Verde	0.519 (0.395-0.640)	0.517 (0.436-0.596)	0.500 (0.436-0.563)	0.456 (0.403-0.511)	0.413 (0.343-0.484)
Cambodia	0.415 (0.299-0.538)	0.434 (0.358-0.516)	0.454 (0.393-0.519)	0.471 (0.416-0.526)	0.478 (0.408-0.548)
Cameroon	0.535 (0.425-0.642)	0.530 (0.458-0.600)	0.516 (0.464-0.567)	0.480 (0.436-0.523)	0.435 (0.372-0.496)
Canada	0.532 (0.458-0.603)	0.487 (0.441-0.534)	0.435 (0.393-0.475)	0.382 (0.345-0.421)	0.346 (0.292-0.403)
Central African Republic	0.494 (0.364-0.623)	0.497 (0.396-0.596)	0.485 (0.403-0.566)	0.468 (0.395-0.541)	0.445 (0.355-0.537)
Chad	0.481 (0.369-0.600)	0.491 (0.412-0.573)	0.484 (0.421-0.547)	0.462 (0.407-0.518)	0.428 (0.355-0.499)
Chile	0.474 (0.359-0.585)	0.437 (0.359-0.516)	0.395 (0.339-0.450)	0.356 (0.310-0.404)	0.322 (0.262-0.385)
China	0.553 (0.452-0.646)	0.555 (0.505-0.601)	0.547 (0.515-0.578)	0.531 (0.496-0.566)	0.504 (0.439-0.564)
China (Hong Kong SAR)	0.513 (0.374-0.642)	0.508 (0.418-0.593)	0.501 (0.435-0.566)	0.502 (0.434-0.570)	0.501 (0.402-0.594)
Colombia	0.478 (0.363-0.590)	0.447 (0.373-0.520)	0.411 (0.359-0.463)	0.377 (0.336-0.418)	0.345 (0.285-0.408)
Comoros	0.517 (0.400-0.630)	0.516 (0.433-0.594)	0.506 (0.441-0.568)	0.485 (0.430-0.540)	0.456 (0.388-0.525)
Congo	0.473 (0.361-0.583)	0.462 (0.381-0.540)	0.447 (0.380-0.512)	0.425 (0.362-0.486)	0.397 (0.317-0.478)
Cook Islands	0.291 (0.202-0.393)	0.234 (0.179-0.291)	0.188 (0.145-0.234)	0.157 (0.120-0.197)	0.140 (0.098-0.189)
Costa Rica	0.479 (0.360-0.595)	0.455 (0.376-0.533)	0.411 (0.353-0.467)	0.361 (0.315-0.408)	0.321 (0.261-0.382)
Cote d'Ivoire	0.517 (0.407-0.624)	0.515 (0.441-0.586)	0.500 (0.446-0.555)	0.473 (0.427-0.519)	0.439 (0.376-0.502)
Croatia	0.486 (0.371-0.595)	0.466 (0.393-0.541)	0.452 (0.394-0.510)	0.435 (0.380-0.489)	0.424 (0.350-0.503)
Cuba	0.415 (0.311-0.521)	0.378 (0.309-0.448)	0.353 (0.298-0.410)	0.322 (0.271-0.374)	0.290 (0.226-0.360)
Cyprus	0.549 (0.446-0.645)	0.492 (0.425-0.559)	0.444 (0.390-0.498)	0.406 (0.353-0.459)	0.381 (0.310-0.458)
Czech Republic	0.437 (0.334-0.535)	0.419 (0.354-0.481)	0.415 (0.364-0.466)	0.409 (0.360-0.460)	0.404 (0.333-0.479)
Denmark	0.525 (0.442-0.601)	0.499 (0.446-0.550)	0.478 (0.433-0.522)	0.461 (0.415-0.506)	0.439 (0.375-0.502)
Djibouti	0.508 (0.377-0.631)	0.505 (0.393-0.610)	0.473 (0.392-0.551)	0.444 (0.369-0.519)	0.412 (0.319-0.506)
Dominica	0.450 (0.342-0.560)	0.430 (0.355-0.505)	0.386 (0.328-0.446)	0.351 (0.299-0.405)	0.310 (0.245-0.379)
Dominican Republic	0.469 (0.358-0.577)	0.439 (0.365-0.511)	0.406 (0.351-0.461)	0.356 (0.309-0.405)	0.316 (0.248-0.385)
DR Congo	0.478 (0.359-0.599)	0.485 (0.392-0.575)	0.483 (0.403-0.557)	0.470 (0.399-0.540)	0.445 (0.358-0.531)
Ecuador	0.533 (0.410-0.646)	0.497 (0.412-0.580)	0.452 (0.385-0.519)	0.410 (0.353-0.468)	0.370 (0.298-0.444)
Egypt	0.447 (0.331-0.568)	0.399 (0.332-0.470)	0.355 (0.310-0.401)	0.306 (0.269-0.343)	0.263 (0.212-0.317)
El Salvador	0.473 (0.352-0.590)	0.454 (0.373-0.534)	0.414 (0.356-0.473)	0.367 (0.321-0.413)	0.333 (0.272-0.398)
Equatorial Guinea	0.502 (0.369-0.636)	0.500 (0.403-0.599)	0.488 (0.406-0.569)	0.414 (0.334-0.493)	0.373 (0.270-0.479)
Eritrea	0.453 (0.338-0.568)	0.457 (0.378-0.537)	0.452 (0.389-0.515)	0.437 (0.383-0.495)	0.416 (0.343-0.489)
Estonia	0.432 (0.324-0.533)	0.421 (0.356-0.484)	0.424 (0.375-0.472)	0.422 (0.379-0.464)	0.424 (0.357-0.489)
Ethiopia	0.473 (0.353-0.595)	0.478 (0.393-0.563)	0.475 (0.409-0.543)	0.460 (0.401-0.516)	0.436 (0.363-0.508)
Fiji	0.410 (0.272-0.550)	0.378 (0.275-0.484)	0.341 (0.260-0.428)	0.300 (0.232-0.373)	0.273 (0.201-0.353)
Finland	0.563 (0.483-0.637)	0.530 (0.482-0.576)	0.488 (0.449-0.527)	0.449 (0.411-0.488)	0.428 (0.372-0.486)
France	0.514 (0.422-0.601)	0.493 (0.433-0.552)	0.463 (0.413-0.513)	0.429 (0.383-0.478)	0.398 (0.332-0.467)
French Polynesia	0.235 (0.154-0.332)	0.205 (0.148-0.271)	0.182 (0.136-0.235)	0.166 (0.125-0.211)	0.153 (0.108-0.207)
Gabon	0.473 (0.339-0.601)	0.466 (0.372-0.558)	0.446 (0.371-0.519)	0.407 (0.340-0.474)	0.379 (0.297-0.465)
Gambia	0.499 (0.384-0.615)	0.499 (0.422-0.576)	0.487 (0.426-0.548)	0.456 (0.404-0.508)	0.417 (0.348-0.488)
Georgia	0.465 (0.334-0.593)	0.433 (0.343-0.523)	0.438 (0.362-0.511)	0.412 (0.349-0.474)	0.376 (0.297-0.454)

Germany	0.549 (0.472-0.621)	0.524 (0.481-0.566)	0.491 (0.456-0.526)	0.456 (0.419-0.494)	0.431 (0.373-0.492)
Ghana	0.525 (0.417-0.630)	0.524 (0.451-0.593)	0.502 (0.447-0.555)	0.464 (0.413-0.514)	0.415 (0.351-0.482)
Greece	0.499 (0.407-0.591)	0.467 (0.407-0.528)	0.439 (0.388-0.490)	0.404 (0.356-0.453)	0.384 (0.316-0.453)
Greenland	0.522 (0.438-0.603)	0.493 (0.436-0.548)	0.466 (0.422-0.512)	0.434 (0.392-0.477)	0.408 (0.344-0.472)
Grenada	0.472 (0.361-0.584)	0.444 (0.366-0.521)	0.402 (0.340-0.461)	0.354 (0.299-0.408)	0.310 (0.240-0.386)
Guatemala	0.491 (0.368-0.609)	0.467 (0.386-0.547)	0.435 (0.377-0.494)	0.393 (0.343-0.440)	0.356 (0.288-0.425)
Guinea	0.519 (0.400-0.640)	0.517 (0.437-0.597)	0.503 (0.441-0.564)	0.479 (0.429-0.530)	0.449 (0.384-0.512)
Guinea Bissau	0.509 (0.394-0.622)	0.510 (0.427-0.590)	0.497 (0.430-0.562)	0.473 (0.415-0.527)	0.443 (0.371-0.512)
Guyana	0.447 (0.332-0.563)	0.428 (0.348-0.506)	0.396 (0.333-0.460)	0.346 (0.289-0.406)	0.302 (0.231-0.378)
Haiti	0.460 (0.343-0.579)	0.455 (0.370-0.536)	0.440 (0.372-0.504)	0.414 (0.351-0.474)	0.381 (0.297-0.466)
Honduras	0.501 (0.381-0.618)	0.479 (0.399-0.559)	0.445 (0.387-0.504)	0.403 (0.354-0.451)	0.357 (0.290-0.423)
Hungary	0.451 (0.353-0.546)	0.439 (0.376-0.502)	0.442 (0.388-0.495)	0.429 (0.377-0.478)	0.417 (0.345-0.493)
Iceland	0.548 (0.457-0.632)	0.508 (0.445-0.569)	0.481 (0.430-0.531)	0.454 (0.402-0.505)	0.435 (0.364-0.505)
India	0.356 (0.263-0.446)	0.375 (0.314-0.435)	0.385 (0.344-0.427)	0.391 (0.355-0.429)	0.391 (0.332-0.453)
Indonesia	0.412 (0.313-0.513)	0.432 (0.367-0.500)	0.448 (0.399-0.500)	0.455 (0.413-0.500)	0.450 (0.384-0.516)
Iran	0.397 (0.284-0.511)	0.380 (0.316-0.448)	0.357 (0.317-0.400)	0.322 (0.293-0.352)	0.287 (0.237-0.339)
Iraq	0.404 (0.297-0.519)	0.373 (0.305-0.447)	0.355 (0.304-0.409)	0.323 (0.279-0.368)	0.288 (0.229-0.351)
Ireland	0.586 (0.502-0.668)	0.559 (0.500-0.618)	0.507 (0.457-0.556)	0.443 (0.396-0.490)	0.388 (0.325-0.453)
Israel	0.471 (0.367-0.569)	0.438 (0.372-0.503)	0.402 (0.351-0.456)	0.379 (0.330-0.430)	0.358 (0.291-0.430)
Italy	0.506 (0.420-0.591)	0.484 (0.433-0.533)	0.455 (0.416-0.495)	0.423 (0.382-0.463)	0.395 (0.330-0.460)
Jamaica	0.425 (0.323-0.532)	0.409 (0.341-0.477)	0.369 (0.318-0.419)	0.329 (0.283-0.376)	0.290 (0.227-0.355)
Japan	0.566 (0.512-0.617)	0.570 (0.537-0.602)	0.568 (0.539-0.597)	0.558 (0.526-0.590)	0.542 (0.485-0.598)
Jordan	0.432 (0.315-0.553)	0.371 (0.307-0.438)	0.331 (0.284-0.378)	0.292 (0.254-0.330)	0.258 (0.207-0.314)
Kazakhstan	0.459 (0.328-0.580)	0.442 (0.352-0.530)	0.422 (0.353-0.493)	0.395 (0.332-0.457)	0.367 (0.284-0.453)
Kenya	0.494 (0.379-0.608)	0.502 (0.424-0.579)	0.499 (0.437-0.559)	0.477 (0.422-0.531)	0.443 (0.370-0.515)
Kiribati	0.309 (0.211-0.417)	0.288 (0.220-0.363)	0.254 (0.199-0.316)	0.211 (0.165-0.263)	0.188 (0.134-0.251)
Kuwait	0.278 (0.184-0.385)	0.284 (0.226-0.347)	0.266 (0.225-0.310)	0.244 (0.208-0.281)	0.226 (0.177-0.279)
Kyrgyzstan	0.503 (0.366-0.634)	0.493 (0.398-0.583)	0.477 (0.406-0.547)	0.457 (0.394-0.516)	0.420 (0.339-0.498)
Lao PDR	0.410 (0.303-0.524)	0.437 (0.357-0.517)	0.460 (0.394-0.526)	0.476 (0.419-0.533)	0.480 (0.406-0.550)
Latvia	0.430 (0.319-0.539)	0.407 (0.339-0.475)	0.407 (0.352-0.465)	0.399 (0.348-0.451)	0.396 (0.320-0.472)
Lebanon	0.400 (0.296-0.508)	0.369 (0.302-0.442)	0.340 (0.285-0.398)	0.303 (0.252-0.358)	0.275 (0.219-0.334)
Lesotho	0.506 (0.366-0.638)	0.489 (0.395-0.580)	0.449 (0.379-0.520)	0.395 (0.337-0.455)	0.342 (0.273-0.414)
Liberia	0.521 (0.408-0.631)	0.521 (0.442-0.598)	0.529 (0.450-0.603)	0.499 (0.442-0.554)	0.455 (0.383-0.524)
Libya	0.348 (0.230-0.472)	0.323 (0.259-0.390)	0.308 (0.263-0.354)	0.285 (0.248-0.323)	0.265 (0.213-0.321)
Lithuania	0.433 (0.323-0.539)	0.406 (0.338-0.471)	0.399 (0.349-0.452)	0.391 (0.343-0.440)	0.377 (0.307-0.451)
Luxembourg	0.553 (0.470-0.633)	0.538 (0.477-0.597)	0.508 (0.447-0.565)	0.461 (0.412-0.510)	0.435 (0.368-0.502)
Macedonia (TFYR)	0.460 (0.346-0.567)	0.450 (0.377-0.522)	0.441 (0.383-0.499)	0.439 (0.385-0.494)	0.430 (0.354-0.506)
Madagascar	0.465 (0.354-0.581)	0.475 (0.399-0.554)	0.473 (0.412-0.534)	0.455 (0.402-0.510)	0.427 (0.352-0.501)
Malawi	0.512 (0.389-0.633)	0.517 (0.434-0.597)	0.513 (0.452-0.573)	0.497 (0.446-0.549)	0.470 (0.397-0.540)
Malaysia	0.434 (0.326-0.541)	0.456 (0.382-0.531)	0.465 (0.406-0.521)	0.450 (0.403-0.495)	0.421 (0.354-0.491)
Maldives	0.459 (0.345-0.574)	0.476 (0.394-0.556)	0.489 (0.418-0.558)	0.486 (0.414-0.553)	0.470 (0.392-0.548)
Mali	0.504 (0.387-0.619)	0.506 (0.426-0.584)	0.493 (0.430-0.554)	0.469 (0.416-0.520)	0.439 (0.369-0.509)
Malta	0.502 (0.370-0.623)	0.455 (0.383-0.525)	0.420 (0.365-0.474)	0.381 (0.330-0.434)	0.356 (0.286-0.432)
Marshall Islands	0.289 (0.198-0.393)	0.255 (0.193-0.322)	0.214 (0.167-0.263)	0.191 (0.149-0.239)	0.174 (0.121-0.235)
Mauritania	0.502 (0.381-0.619)	0.509 (0.424-0.590)	0.492 (0.426-0.559)	0.463 (0.404-0.522)	0.425 (0.350-0.500)
Mauritius	0.507 (0.394-0.614)	0.492 (0.421-0.563)	0.462 (0.403-0.520)	0.419 (0.363-0.475)	0.369 (0.291-0.449)
Mexico	0.442 (0.331-0.550)	0.406 (0.337-0.475)	0.368 (0.322-0.417)	0.329 (0.292-0.366)	0.297 (0.243-0.354)
Micronesia (Federated States of)	0.325 (0.224-0.439)	0.293 (0.227-0.369)	0.257 (0.205-0.314)	0.221 (0.178-0.266)	0.191 (0.141-0.247)
Moldova	0.480 (0.359-0.592)	0.458 (0.383-0.531)	0.454 (0.395-0.513)	0.450 (0.393-0.506)	0.434 (0.358-0.510)
Mongolia	0.521 (0.384-0.649)	0.500 (0.408-0.591)	0.481 (0.410-0.551)	0.457 (0.397-0.517)	0.414 (0.336-0.490)
Montenegro	0.461 (0.332-0.586)	0.442 (0.359-0.525)	0.440 (0.380-0.502)	0.440 (0.385-0.496)	0.431 (0.354-0.507)
Morocco	0.450 (0.328-0.572)	0.425 (0.349-0.503)	0.393 (0.341-0.447)	0.357 (0.312-0.403)	0.319 (0.257-0.385)
Mozambique	0.521 (0.389-0.646)	0.527 (0.434-0.616)	0.519 (0.449-0.586)	0.494 (0.437-0.549)	0.462 (0.390-0.532)
Myanmar	0.437 (0.302-0.583)	0.454 (0.360-0.551)	0.470 (0.401-0.542)	0.477 (0.420-0.534)	0.479 (0.412-0.548)
Namibia	0.446 (0.314-0.580)	0.437 (0.349-0.528)	0.410 (0.346-0.477)	0.369 (0.319-0.423)	0.330 (0.268-0.397)
Nauru	0.161 (0.105-0.228)	0.147 (0.107-0.191)	0.149 (0.113-0.189)	0.158 (0.117-0.202)	0.153 (0.102-0.216)
Nepal	0.389 (0.284-0.500)	0.408 (0.331-0.488)	0.423 (0.363-0.485)	0.435 (0.383-0.488)	0.439 (0.369-0.508)
Netherlands	0.545 (0.461-0.623)	0.526 (0.474-0.576)	0.495 (0.455-0.535)	0.457 (0.419-0.494)	0.429 (0.368-0.491)
New Zealand	0.534 (0.454-0.611)	0.502 (0.452-0.553)	0.452 (0.412-0.492)	0.387 (0.350-0.422)	0.344 (0.293-0.394)
Nicaragua	0.480 (0.366-0.591)	0.464 (0.388-0.541)	0.437 (0.380-0.495)	0.391 (0.345-0.439)	0.351 (0.285-0.421)
Niger	0.477 (0.364-0.595)	0.485 (0.407-0.566)	0.482 (0.420-0.545)	0.470 (0.415-0.525)	0.450 (0.379-0.519)
Nigeria	0.484 (0.373-0.597)	0.492 (0.423-0.562)	0.482 (0.433-0.532)	0.454 (0.411-0.496)	0.409 (0.349-0.469)
Niue	0.322 (0.217-0.442)	0.261 (0.193-0.335)	0.215 (0.166-0.267)	0.178 (0.139-0.221)	0.149 (0.104-0.199)
North Korea	0.534 (0.407-0.649)	0.535 (0.449-0.616)	0.536 (0.461-0.607)	0.528 (0.443-0.607)	0.515 (0.404-0.620)
Norway	0.521 (0.442-0.600)	0.490 (0.438-0.542)	0.452 (0.409-0.495)	0.416 (0.376-0.457)	0.399 (0.337-0.463)
Occupied Palestinian Territory	0.434 (0.321-0.552)	0.404 (0.328-0.484)	0.374 (0.316-0.432)	0.327 (0.280-0.374)	0.293 (0.228-0.362)
Oman	0.388 (0.263-0.512)	0.358 (0.288-0.433)	0.317 (0.272-0.364)	0.286 (0.249-0.327)	0.258 (0.205-0.317)
Pakistan	0.412 (0.303-0.519)	0.426 (0.350-0.504)	0.436 (0.378-0.497)	0.441 (0.388-0.497)	0.438 (0.367-0.512)
Palau	0.207 (0.132-0.294)	0.199 (0.149-0.254)	0.179 (0.138-0.224)	0.161 (0.123-0.202)	0.150 (0.105-0.201)
Panama	0.461 (0.346-0.575)	0.431 (0.354-0.509)	0.395 (0.339-0.454)	0.347 (0.301-0.395)	0.301 (0.238-0.365)
Papua New Guinea	0.480 (0.326-0.628)	0.468 (0.353-0.577)	0.436 (0.345-0.524)	0.402 (0.324-0.478)	0.360 (0.271-0.452)

Paraguay	0.536 (0.422-0.643)	0.513 (0.435-0.590)	0.481 (0.419-0.542)	0.448 (0.391-0.504)	0.404 (0.330-0.477)
Peru	0.508 (0.391-0.624)	0.474 (0.391-0.558)	0.442 (0.379-0.506)	0.394 (0.346-0.443)	0.341 (0.280-0.400)
Philippines	0.414 (0.317-0.508)	0.439 (0.379-0.498)	0.456 (0.409-0.503)	0.461 (0.417-0.507)	0.455 (0.386-0.525)
Poland	0.456 (0.354-0.552)	0.445 (0.383-0.503)	0.433 (0.386-0.482)	0.412 (0.364-0.458)	0.397 (0.329-0.467)
Portugal	0.542 (0.439-0.640)	0.522 (0.454-0.586)	0.489 (0.436-0.541)	0.458 (0.410-0.507)	0.429 (0.361-0.502)
Puerto Rico	0.398 (0.294-0.502)	0.352 (0.284-0.422)	0.300 (0.247-0.356)	0.268 (0.221-0.315)	0.248 (0.191-0.309)
Qatar	0.338 (0.236-0.447)	0.261 (0.197-0.333)	0.254 (0.209-0.301)	0.225 (0.187-0.265)	0.207 (0.158-0.263)
Romania	0.462 (0.352-0.569)	0.447 (0.379-0.516)	0.439 (0.386-0.493)	0.426 (0.374-0.477)	0.413 (0.338-0.490)
Russian Federation	0.407 (0.311-0.502)	0.387 (0.334-0.440)	0.384 (0.346-0.421)	0.379 (0.338-0.421)	0.373 (0.305-0.442)
Rwanda	0.523 (0.393-0.648)	0.532 (0.433-0.628)	0.536 (0.461-0.611)	0.524 (0.459-0.586)	0.495 (0.419-0.569)
Saint Kitts and Nevis	0.457 (0.343-0.569)	0.419 (0.341-0.499)	0.376 (0.316-0.439)	0.336 (0.279-0.394)	0.296 (0.221-0.379)
Saint Lucia	0.473 (0.364-0.582)	0.452 (0.379-0.526)	0.412 (0.355-0.472)	0.374 (0.316-0.433)	0.322 (0.240-0.407)
Saint Vincent and the Grenadines	0.464 (0.347-0.581)	0.439 (0.360-0.517)	0.398 (0.340-0.458)	0.354 (0.301-0.406)	0.321 (0.252-0.396)
Samoa	0.326 (0.221-0.440)	0.290 (0.222-0.367)	0.245 (0.193-0.301)	0.196 (0.155-0.241)	0.164 (0.118-0.216)
Sao Tome and Principe	0.510 (0.393-0.621)	0.509 (0.428-0.585)	0.490 (0.426-0.552)	0.462 (0.407-0.515)	0.423 (0.356-0.492)
Saudi Arabia	0.347 (0.234-0.470)	0.317 (0.256-0.383)	0.294 (0.255-0.335)	0.270 (0.236-0.305)	0.240 (0.192-0.292)
Senegal	0.495 (0.385-0.604)	0.490 (0.417-0.563)	0.475 (0.417-0.533)	0.449 (0.399-0.500)	0.415 (0.347-0.482)
Serbia	0.477 (0.368-0.584)	0.467 (0.397-0.534)	0.457 (0.405-0.510)	0.454 (0.406-0.502)	0.439 (0.369-0.507)
Seychelles	0.496 (0.383-0.604)	0.466 (0.388-0.544)	0.427 (0.363-0.491)	0.378 (0.318-0.438)	0.327 (0.254-0.405)
Sierra Leone	0.512 (0.398-0.621)	0.509 (0.431-0.587)	0.497 (0.434-0.557)	0.474 (0.422-0.525)	0.438 (0.374-0.501)
Singapore	0.558 (0.452-0.658)	0.547 (0.487-0.605)	0.535 (0.488-0.583)	0.528 (0.482-0.572)	0.513 (0.449-0.575)
Slovakia	0.492 (0.384-0.594)	0.471 (0.404-0.533)	0.453 (0.401-0.504)	0.435 (0.387-0.484)	0.412 (0.338-0.486)
Slovenia	0.476 (0.363-0.587)	0.464 (0.390-0.536)	0.446 (0.390-0.504)	0.429 (0.376-0.484)	0.417 (0.338-0.493)
Solomon Islands	0.501 (0.345-0.650)	0.474 (0.358-0.585)	0.437 (0.345-0.526)	0.401 (0.325-0.477)	0.364 (0.279-0.452)
Somalia	0.515 (0.387-0.637)	0.514 (0.419-0.608)	0.504 (0.424-0.582)	0.490 (0.415-0.563)	0.462 (0.372-0.550)
South Africa	0.423 (0.307-0.538)	0.393 (0.318-0.469)	0.359 (0.305-0.414)	0.312 (0.272-0.354)	0.274 (0.223-0.328)
South Korea	0.546 (0.454-0.636)	0.571 (0.514-0.626)	0.563 (0.515-0.611)	0.557 (0.515-0.597)	0.535 (0.470-0.596)
Spain	0.520 (0.426-0.606)	0.491 (0.434-0.544)	0.454 (0.412-0.497)	0.418 (0.377-0.458)	0.390 (0.325-0.455)
Sri Lanka	0.427 (0.318-0.539)	0.449 (0.373-0.527)	0.464 (0.403-0.525)	0.470 (0.415-0.525)	0.457 (0.375-0.540)
Sudan	0.513 (0.395-0.629)	0.516 (0.434-0.598)	0.502 (0.434-0.568)	0.479 (0.419-0.537)	0.449 (0.375-0.526)
Suriname	0.405 (0.298-0.512)	0.371 (0.298-0.445)	0.354 (0.294-0.415)	0.326 (0.274-0.382)	0.291 (0.225-0.363)
Swaziland	0.489 (0.353-0.622)	0.473 (0.381-0.565)	0.428 (0.359-0.499)	0.383 (0.325-0.442)	0.337 (0.263-0.414)
Sweden	0.531 (0.450-0.610)	0.510 (0.461-0.559)	0.484 (0.445-0.523)	0.452 (0.413-0.492)	0.426 (0.365-0.488)
Switzerland	0.520 (0.427-0.610)	0.506 (0.446-0.566)	0.471 (0.425-0.517)	0.445 (0.402-0.489)	0.427 (0.363-0.491)
Syrian Arab Republic	0.445 (0.327-0.566)	0.413 (0.339-0.489)	0.386 (0.332-0.439)	0.350 (0.305-0.397)	0.316 (0.254-0.380)
Taiwan	0.514 (0.408-0.615)	0.521 (0.453-0.589)	0.518 (0.464-0.572)	0.508 (0.454-0.560)	0.494 (0.413-0.573)
Tajikistan	0.477 (0.342-0.601)	0.474 (0.378-0.564)	0.475 (0.402-0.547)	0.460 (0.395-0.525)	0.419 (0.334-0.500)
Tanzania	0.511 (0.394-0.626)	0.517 (0.436-0.591)	0.507 (0.449-0.561)	0.482 (0.435-0.527)	0.445 (0.382-0.507)
Thailand	0.443 (0.336-0.550)	0.466 (0.395-0.536)	0.480 (0.425-0.532)	0.480 (0.434-0.526)	0.461 (0.394-0.527)
Timor-Leste	0.411 (0.308-0.522)	0.429 (0.353-0.512)	0.447 (0.382-0.518)	0.461 (0.400-0.524)	0.465 (0.390-0.542)
Togo	0.513 (0.403-0.627)	0.515 (0.437-0.592)	0.504 (0.444-0.564)	0.477 (0.427-0.527)	0.443 (0.379-0.505)
Tokelau	0.335 (0.196-0.497)	0.298 (0.205-0.406)	0.254 (0.192-0.323)	0.211 (0.161-0.264)	0.176 (0.119-0.242)
Tonga	0.317 (0.213-0.433)	0.275 (0.206-0.350)	0.229 (0.177-0.283)	0.187 (0.146-0.231)	0.161 (0.116-0.214)
Trinidad and Tobago	0.435 (0.292-0.581)	0.421 (0.315-0.524)	0.399 (0.329-0.469)	0.344 (0.277-0.413)	0.290 (0.200-0.395)
Tunisia	0.428 (0.310-0.546)	0.394 (0.326-0.466)	0.359 (0.311-0.408)	0.322 (0.284-0.363)	0.289 (0.236-0.347)
Turkey	0.431 (0.322-0.543)	0.395 (0.330-0.460)	0.344 (0.304-0.385)	0.303 (0.271-0.337)	0.269 (0.221-0.318)
Turkmenistan	0.480 (0.349-0.607)	0.471 (0.380-0.560)	0.445 (0.376-0.514)	0.418 (0.358-0.479)	0.372 (0.296-0.451)
Tuvalu	0.363 (0.254-0.482)	0.310 (0.241-0.386)	0.245 (0.194-0.298)	0.204 (0.162-0.249)	0.178 (0.128-0.234)
Uganda	0.522 (0.401-0.643)	0.530 (0.440-0.620)	0.528 (0.454-0.600)	0.511 (0.448-0.573)	0.480 (0.409-0.549)
Ukraine	0.434 (0.325-0.539)	0.415 (0.348-0.483)	0.411 (0.357-0.466)	0.413 (0.358-0.470)	0.404 (0.327-0.483)
United Arab Emirates	0.278 (0.170-0.407)	0.278 (0.209-0.352)	0.267 (0.221-0.315)	0.236 (0.197-0.276)	0.229 (0.178-0.285)
United Kingdom	0.539 (0.465-0.612)	0.503 (0.462-0.544)	0.451 (0.421-0.480)	0.393 (0.365-0.420)	0.353 (0.309-0.399)
United States of America	0.489 (0.421-0.557)	0.451 (0.406-0.495)	0.399 (0.363-0.434)	0.346 (0.314-0.377)	0.309 (0.262-0.357)
Uruguay	0.472 (0.357-0.583)	0.441 (0.361-0.519)	0.406 (0.348-0.467)	0.374 (0.324-0.425)	0.330 (0.264-0.397)
Uzbekistan	0.496 (0.355-0.627)	0.488 (0.390-0.581)	0.468 (0.395-0.540)	0.443 (0.378-0.506)	0.396 (0.310-0.479)
Vanuatu	0.475 (0.328-0.623)	0.453 (0.345-0.559)	0.412 (0.329-0.495)	0.367 (0.297-0.437)	0.324 (0.246-0.408)
Venezuela	0.430 (0.317-0.543)	0.406 (0.332-0.482)	0.371 (0.316-0.427)	0.340 (0.297-0.385)	0.311 (0.252-0.376)
Viet Nam	0.383 (0.288-0.482)	0.401 (0.341-0.464)	0.421 (0.375-0.469)	0.443 (0.400-0.486)	0.460 (0.394-0.526)
Yemen	0.425 (0.292-0.560)	0.427 (0.338-0.520)	0.413 (0.347-0.478)	0.386 (0.329-0.443)	0.363 (0.288-0.438)
Zambia	0.499 (0.385-0.609)	0.497 (0.420-0.572)	0.485 (0.425-0.544)	0.463 (0.412-0.514)	0.424 (0.356-0.494)
Zimbabwe	0.487 (0.358-0.610)	0.470 (0.384-0.552)	0.435 (0.373-0.496)	0.398 (0.345-0.451)	0.366 (0.297-0.438)

Men, 25 ≤ BMI < 30 kg/m^2

Country	Year				
	1975	1985	1995	2005	2014
Afghanistan	0.067 (0.031-0.120)	0.076 (0.046-0.115)	0.086 (0.056-0.122)	0.094 (0.065-0.130)	0.115 (0.072-0.169)
Albania	0.291 (0.186-0.400)	0.315 (0.243-0.392)	0.334 (0.270-0.402)	0.381 (0.324-0.444)	0.419 (0.341-0.502)
Algeria	0.223 (0.137-0.312)	0.265 (0.204-0.328)	0.297 (0.246-0.349)	0.325 (0.277-0.370)	0.357 (0.290-0.423)
American Samoa	0.358 (0.265-0.451)	0.351 (0.285-0.416)	0.338 (0.276-0.402)	0.333 (0.275-0.398)	0.329 (0.248-0.418)
Andorra	0.371 (0.288-0.459)	0.406 (0.342-0.469)	0.431 (0.378-0.490)	0.446 (0.395-0.498)	0.442 (0.371-0.514)
Angola	0.076 (0.033-0.138)	0.085 (0.046-0.130)	0.094 (0.057-0.138)	0.108 (0.069-0.154)	0.144 (0.085-0.213)
Antigua and Barbuda	0.231 (0.141-0.338)	0.257 (0.191-0.334)	0.303 (0.242-0.370)	0.314 (0.254-0.377)	0.321 (0.237-0.415)
Argentina	0.329 (0.222-0.433)	0.355 (0.277-0.431)	0.374 (0.311-0.435)	0.394 (0.339-0.447)	0.399 (0.332-0.469)
Armenia	0.249 (0.137-0.374)	0.284 (0.193-0.376)	0.300 (0.222-0.382)	0.336 (0.270-0.408)	0.388 (0.305-0.471)
Australia	0.357 (0.281-0.432)	0.397 (0.349-0.444)	0.420 (0.381-0.461)	0.426 (0.390-0.462)	0.420 (0.365-0.475)
Austria	0.326 (0.248-0.404)	0.379 (0.326-0.429)	0.409 (0.362-0.457)	0.417 (0.373-0.462)	0.417 (0.347-0.487)
Azerbaijan	0.269 (0.170-0.374)	0.302 (0.226-0.382)	0.312 (0.243-0.388)	0.333 (0.269-0.403)	0.373 (0.289-0.459)
Bahamas	0.263 (0.171-0.359)	0.295 (0.225-0.366)	0.333 (0.272-0.396)	0.357 (0.300-0.414)	0.363 (0.288-0.443)
Bahrain	0.294 (0.204-0.390)	0.328 (0.265-0.384)	0.356 (0.305-0.405)	0.381 (0.334-0.424)	0.398 (0.330-0.464)
Bangladesh	0.058 (0.024-0.109)	0.064 (0.038-0.095)	0.076 (0.053-0.103)	0.096 (0.072-0.127)	0.124 (0.086-0.174)
Barbados	0.222 (0.137-0.316)	0.264 (0.203-0.328)	0.290 (0.236-0.345)	0.313 (0.260-0.371)	0.326 (0.246-0.413)
Belarus	0.291 (0.205-0.397)	0.329 (0.266-0.399)	0.351 (0.293-0.414)	0.376 (0.321-0.433)	0.405 (0.329-0.480)
Belgium	0.338 (0.257-0.429)	0.390 (0.343-0.441)	0.429 (0.389-0.470)	0.449 (0.411-0.488)	0.448 (0.386-0.507)
Belize	0.181 (0.100-0.280)	0.222 (0.161-0.289)	0.253 (0.201-0.311)	0.293 (0.239-0.346)	0.314 (0.238-0.389)
Benin	0.073 (0.035-0.130)	0.085 (0.052-0.122)	0.095 (0.066-0.128)	0.114 (0.085-0.147)	0.137 (0.094-0.192)
Bermuda	0.305 (0.200-0.414)	0.347 (0.262-0.426)	0.372 (0.301-0.443)	0.373 (0.309-0.435)	0.364 (0.284-0.445)
Bhutan	0.068 (0.030-0.126)	0.082 (0.049-0.124)	0.106 (0.073-0.149)	0.139 (0.104-0.185)	0.178 (0.127-0.242)
Bolivia	0.228 (0.133-0.329)	0.260 (0.183-0.346)	0.294 (0.224-0.371)	0.335 (0.274-0.402)	0.366 (0.288-0.449)
Bosnia and Herzegovina	0.238 (0.123-0.383)	0.267 (0.182-0.371)	0.298 (0.228-0.377)	0.369 (0.307-0.433)	0.409 (0.338-0.483)
Botswana	0.084 (0.040-0.144)	0.105 (0.067-0.151)	0.146 (0.103-0.193)	0.177 (0.132-0.225)	0.214 (0.154-0.283)
Brazil	0.205 (0.125-0.290)	0.260 (0.202-0.322)	0.307 (0.261-0.357)	0.349 (0.308-0.390)	0.381 (0.320-0.446)
Brunei Darussalam	0.168 (0.067-0.287)	0.204 (0.117-0.303)	0.239 (0.171-0.318)	0.276 (0.213-0.343)	0.295 (0.209-0.389)
Bulgaria	0.346 (0.252-0.449)	0.390 (0.327-0.455)	0.409 (0.352-0.465)	0.412 (0.358-0.466)	0.425 (0.348-0.500)
Burkina Faso	0.069 (0.027-0.129)	0.076 (0.043-0.115)	0.089 (0.061-0.120)	0.109 (0.079-0.141)	0.125 (0.082-0.171)
Burundi	0.038 (0.012-0.085)	0.045 (0.020-0.085)	0.056 (0.030-0.094)	0.063 (0.039-0.098)	0.078 (0.045-0.122)
Cabo Verde	0.066 (0.027-0.125)	0.089 (0.054-0.130)	0.122 (0.086-0.161)	0.176 (0.135-0.220)	0.215 (0.151-0.282)
Cambodia	0.048 (0.019-0.100)	0.051 (0.029-0.080)	0.062 (0.041-0.086)	0.081 (0.058-0.109)	0.109 (0.070-0.154)
Cameroon	0.087 (0.043-0.147)	0.108 (0.073-0.151)	0.126 (0.092-0.165)	0.147 (0.113-0.182)	0.171 (0.119-0.233)
Canada	0.362 (0.289-0.438)	0.397 (0.352-0.443)	0.417 (0.374-0.460)	0.426 (0.386-0.467)	0.422 (0.361-0.485)
Central African Republic	0.060 (0.024-0.115)	0.068 (0.036-0.116)	0.077 (0.045-0.121)	0.090 (0.055-0.131)	0.109 (0.061-0.168)
Chad	0.071 (0.032-0.124)	0.074 (0.043-0.110)	0.087 (0.057-0.118)	0.103 (0.074-0.136)	0.116 (0.073-0.167)
Chile	0.288 (0.182-0.405)	0.322 (0.244-0.399)	0.356 (0.296-0.416)	0.387 (0.335-0.436)	0.399 (0.333-0.463)
China	0.079 (0.035-0.138)	0.101 (0.073-0.131)	0.147 (0.126-0.170)	0.230 (0.202-0.259)	0.313 (0.259-0.368)
China (Hong Kong SAR)	0.210 (0.110-0.321)	0.261 (0.184-0.336)	0.305 (0.239-0.369)	0.353 (0.284-0.418)	0.403 (0.304-0.493)
Colombia	0.232 (0.134-0.333)	0.277 (0.206-0.346)	0.318 (0.261-0.374)	0.358 (0.310-0.405)	0.384 (0.314-0.453)
Comoros	0.081 (0.038-0.137)	0.086 (0.053-0.130)	0.098 (0.066-0.137)	0.110 (0.081-0.146)	0.128 (0.086-0.177)
Congo	0.073 (0.035-0.125)	0.093 (0.057-0.142)	0.114 (0.073-0.164)	0.139 (0.097-0.191)	0.173 (0.109-0.255)
Cook Islands	0.375 (0.260-0.490)	0.382 (0.310-0.455)	0.364 (0.296-0.434)	0.343 (0.283-0.408)	0.324 (0.249-0.404)
Costa Rica	0.245 (0.147-0.349)	0.292 (0.221-0.364)	0.336 (0.282-0.394)	0.371 (0.324-0.422)	0.393 (0.328-0.465)
Cote d'Ivoire	0.105 (0.055-0.175)	0.115 (0.077-0.164)	0.129 (0.094-0.170)	0.153 (0.118-0.194)	0.180 (0.129-0.239)
Croatia	0.303 (0.206-0.415)	0.345 (0.280-0.416)	0.369 (0.310-0.431)	0.403 (0.348-0.459)	0.430 (0.355-0.508)
Cuba	0.211 (0.132-0.303)	0.261 (0.199-0.322)	0.290 (0.233-0.346)	0.308 (0.256-0.362)	0.341 (0.269-0.413)
Cyprus	0.320 (0.231-0.416)	0.362 (0.299-0.427)	0.407 (0.351-0.464)	0.423 (0.370-0.477)	0.418 (0.342-0.492)
Czech Republic	0.387 (0.297-0.485)	0.411 (0.352-0.471)	0.426 (0.373-0.479)	0.437 (0.388-0.488)	0.445 (0.376-0.516)
Denmark	0.324 (0.260-0.391)	0.375 (0.329-0.418)	0.416 (0.372-0.460)	0.438 (0.393-0.484)	0.440 (0.373-0.504)
Djibouti	0.106 (0.049-0.183)	0.116 (0.060-0.190)	0.140 (0.092-0.197)	0.162 (0.112-0.218)	0.199 (0.128-0.280)
Dominica	0.186 (0.109-0.281)	0.220 (0.158-0.288)	0.272 (0.218-0.332)	0.318 (0.263-0.376)	0.343 (0.267-0.421)
Dominican Republic	0.162 (0.088-0.249)	0.200 (0.143-0.264)	0.238 (0.186-0.293)	0.294 (0.245-0.345)	0.334 (0.259-0.411)
DR Congo	0.068 (0.031-0.120)	0.078 (0.045-0.124)	0.086 (0.053-0.131)	0.094 (0.057-0.140)	0.119 (0.063-0.183)
Ecuador	0.234 (0.137-0.341)	0.276 (0.198-0.363)	0.313 (0.243-0.391)	0.347 (0.282-0.417)	0.380 (0.300-0.460)
Egypt	0.244 (0.152-0.346)	0.287 (0.228-0.349)	0.310 (0.263-0.360)	0.327 (0.285-0.372)	0.342 (0.277-0.408)
El Salvador	0.227 (0.133-0.330)	0.263 (0.195-0.339)	0.298 (0.242-0.360)	0.338 (0.287-0.391)	0.365 (0.293-0.440)
Equatorial Guinea	0.063 (0.025-0.121)	0.079 (0.043-0.128)	0.096 (0.058-0.143)	0.155 (0.098-0.222)	0.187 (0.103-0.288)
Eritrea	0.064 (0.029-0.115)	0.068 (0.041-0.106)	0.079 (0.054-0.111)	0.092 (0.068-0.122)	0.104 (0.066-0.148)
Estonia	0.302 (0.212-0.401)	0.333 (0.276-0.391)	0.349 (0.299-0.397)	0.380 (0.337-0.420)	0.403 (0.340-0.464)
Ethiopia	0.061 (0.025-0.111)	0.066 (0.038-0.103)	0.072 (0.048-0.104)	0.078 (0.055-0.105)	0.091 (0.057-0.131)
Fiji	0.282 (0.178-0.402)	0.314 (0.229-0.412)	0.329 (0.250-0.417)	0.342 (0.271-0.417)	0.341 (0.257-0.429)
Finland	0.316 (0.242-0.392)	0.365 (0.320-0.410)	0.395 (0.353-0.436)	0.405 (0.367-0.442)	0.406 (0.347-0.465)
France	0.380 (0.295-0.467)	0.418 (0.359-0.472)	0.442 (0.391-0.491)	0.453 (0.402-0.501)	0.449 (0.376-0.518)
French Polynesia	0.410 (0.302-0.521)	0.401 (0.315-0.488)	0.383 (0.309-0.461)	0.361 (0.298-0.426)	0.339 (0.265-0.416)

Gabon	0.104 (0.047-0.182)	0.142 (0.086-0.212)	0.173 (0.112-0.245)	0.207 (0.144-0.280)	0.239 (0.157-0.339)
Gambia	0.083 (0.039-0.148)	0.092 (0.058-0.134)	0.116 (0.082-0.157)	0.148 (0.110-0.190)	0.176 (0.114-0.241)
Georgia	0.265 (0.165-0.380)	0.303 (0.226-0.390)	0.308 (0.238-0.387)	0.340 (0.277-0.415)	0.363 (0.289-0.444)
Germany	0.339 (0.265-0.414)	0.396 (0.353-0.436)	0.422 (0.385-0.459)	0.423 (0.388-0.458)	0.420 (0.357-0.478)
Ghana	0.086 (0.043-0.141)	0.093 (0.059-0.134)	0.109 (0.074-0.148)	0.134 (0.097-0.176)	0.166 (0.114-0.226)
Greece	0.351 (0.266-0.442)	0.403 (0.346-0.464)	0.432 (0.381-0.485)	0.447 (0.397-0.498)	0.445 (0.372-0.514)
Greenland	0.330 (0.256-0.406)	0.384 (0.333-0.436)	0.412 (0.365-0.459)	0.421 (0.374-0.464)	0.420 (0.352-0.487)
Grenada	0.183 (0.110-0.275)	0.217 (0.162-0.286)	0.259 (0.207-0.321)	0.293 (0.241-0.350)	0.320 (0.247-0.398)
Guatemala	0.214 (0.117-0.317)	0.248 (0.176-0.322)	0.281 (0.225-0.344)	0.320 (0.271-0.374)	0.347 (0.272-0.424)
Guinea	0.077 (0.034-0.147)	0.093 (0.057-0.142)	0.105 (0.072-0.145)	0.123 (0.091-0.157)	0.138 (0.095-0.192)
Guinea Bissau	0.083 (0.039-0.143)	0.095 (0.059-0.136)	0.118 (0.080-0.157)	0.126 (0.094-0.163)	0.144 (0.098-0.197)
Guyana	0.189 (0.111-0.280)	0.215 (0.157-0.281)	0.229 (0.177-0.285)	0.269 (0.214-0.324)	0.287 (0.214-0.372)
Haiti	0.130 (0.068-0.210)	0.148 (0.096-0.207)	0.164 (0.113-0.219)	0.202 (0.152-0.257)	0.242 (0.167-0.321)
Honduras	0.200 (0.102-0.301)	0.235 (0.163-0.306)	0.273 (0.216-0.331)	0.317 (0.268-0.369)	0.347 (0.274-0.425)
Hungary	0.367 (0.282-0.460)	0.400 (0.346-0.459)	0.417 (0.363-0.471)	0.421 (0.373-0.469)	0.432 (0.364-0.499)
Iceland	0.335 (0.257-0.421)	0.392 (0.332-0.449)	0.414 (0.363-0.464)	0.423 (0.374-0.473)	0.431 (0.363-0.499)
India	0.053 (0.024-0.091)	0.066 (0.043-0.092)	0.083 (0.065-0.104)	0.109 (0.089-0.131)	0.141 (0.101-0.190)
Indonesia	0.056 (0.025-0.099)	0.068 (0.044-0.094)	0.089 (0.066-0.115)	0.120 (0.094-0.149)	0.162 (0.114-0.215)
Iran	0.251 (0.167-0.341)	0.289 (0.239-0.344)	0.316 (0.279-0.357)	0.355 (0.324-0.386)	0.394 (0.339-0.454)
Iraq	0.265 (0.175-0.363)	0.300 (0.240-0.369)	0.319 (0.266-0.378)	0.331 (0.281-0.379)	0.354 (0.287-0.419)
Ireland	0.348 (0.259-0.441)	0.394 (0.330-0.460)	0.420 (0.368-0.476)	0.429 (0.381-0.480)	0.411 (0.342-0.480)
Israel	0.362 (0.272-0.461)	0.401 (0.336-0.466)	0.431 (0.376-0.485)	0.447 (0.396-0.498)	0.446 (0.374-0.518)
Italy	0.363 (0.281-0.445)	0.403 (0.358-0.449)	0.430 (0.393-0.469)	0.445 (0.407-0.484)	0.443 (0.381-0.504)
Jamaica	0.206 (0.129-0.292)	0.227 (0.169-0.285)	0.257 (0.206-0.307)	0.302 (0.252-0.349)	0.331 (0.264-0.400)
Japan	0.131 (0.099-0.170)	0.164 (0.140-0.190)	0.201 (0.177-0.226)	0.229 (0.203-0.256)	0.251 (0.203-0.307)
Jordan	0.264 (0.166-0.368)	0.312 (0.251-0.373)	0.345 (0.295-0.399)	0.371 (0.326-0.417)	0.388 (0.322-0.456)
Kazakhstan	0.270 (0.173-0.377)	0.307 (0.234-0.385)	0.329 (0.260-0.401)	0.354 (0.289-0.423)	0.385 (0.301-0.469)
Kenya	0.072 (0.033-0.125)	0.089 (0.055-0.130)	0.108 (0.076-0.145)	0.121 (0.089-0.159)	0.142 (0.093-0.205)
Kiribati	0.390 (0.280-0.508)	0.386 (0.295-0.476)	0.382 (0.299-0.459)	0.375 (0.303-0.443)	0.362 (0.267-0.458)
Kuwait	0.351 (0.244-0.459)	0.380 (0.315-0.441)	0.391 (0.340-0.440)	0.409 (0.365-0.453)	0.407 (0.344-0.470)
Kyrgyzstan	0.240 (0.135-0.354)	0.271 (0.193-0.358)	0.291 (0.221-0.368)	0.308 (0.244-0.380)	0.338 (0.260-0.422)
Lao PDR	0.054 (0.023-0.101)	0.056 (0.034-0.088)	0.064 (0.043-0.090)	0.087 (0.063-0.117)	0.125 (0.083-0.175)
Latvia	0.306 (0.217-0.410)	0.342 (0.282-0.409)	0.356 (0.301-0.412)	0.378 (0.328-0.429)	0.411 (0.338-0.485)
Lebanon	0.294 (0.208-0.387)	0.331 (0.268-0.397)	0.381 (0.320-0.441)	0.418 (0.358-0.479)	0.412 (0.345-0.479)
Lesotho	0.077 (0.032-0.141)	0.090 (0.055-0.137)	0.106 (0.075-0.146)	0.124 (0.093-0.161)	0.145 (0.097-0.196)
Liberia	0.089 (0.045-0.151)	0.102 (0.064-0.148)	0.098 (0.057-0.148)	0.119 (0.084-0.161)	0.144 (0.095-0.206)
Libya	0.300 (0.191-0.409)	0.354 (0.292-0.417)	0.377 (0.324-0.429)	0.389 (0.344-0.434)	0.395 (0.332-0.460)
Lithuania	0.301 (0.213-0.399)	0.336 (0.275-0.401)	0.351 (0.296-0.409)	0.375 (0.328-0.424)	0.399 (0.331-0.470)
Luxembourg	0.347 (0.266-0.428)	0.396 (0.335-0.455)	0.429 (0.368-0.488)	0.433 (0.381-0.484)	0.429 (0.358-0.498)
Macedonia (TFYR)	0.313 (0.219-0.420)	0.351 (0.282-0.425)	0.371 (0.309-0.439)	0.400 (0.342-0.459)	0.424 (0.347-0.504)
Madagascar	0.085 (0.044-0.143)	0.091 (0.059-0.133)	0.098 (0.069-0.136)	0.107 (0.080-0.141)	0.123 (0.081-0.173)
Malawi	0.062 (0.025-0.114)	0.070 (0.041-0.108)	0.078 (0.052-0.108)	0.089 (0.065-0.117)	0.103 (0.067-0.149)
Malaysia	0.098 (0.049-0.158)	0.131 (0.088-0.176)	0.176 (0.135-0.222)	0.224 (0.184-0.268)	0.261 (0.198-0.325)
Maldives	0.082 (0.036-0.146)	0.098 (0.059-0.141)	0.127 (0.081-0.173)	0.176 (0.116-0.234)	0.225 (0.157-0.301)
Mali	0.080 (0.035-0.135)	0.088 (0.053-0.129)	0.101 (0.068-0.140)	0.117 (0.083-0.154)	0.139 (0.090-0.198)
Malta	0.332 (0.216-0.459)	0.393 (0.326-0.463)	0.420 (0.364-0.475)	0.437 (0.384-0.490)	0.436 (0.359-0.511)
Marshall Islands	0.367 (0.252-0.488)	0.373 (0.286-0.458)	0.371 (0.301-0.441)	0.369 (0.300-0.439)	0.364 (0.272-0.459)
Mauritania	0.092 (0.046-0.163)	0.102 (0.062-0.148)	0.123 (0.081-0.169)	0.147 (0.108-0.191)	0.179 (0.123-0.244)
Mauritius	0.118 (0.064-0.184)	0.151 (0.104-0.202)	0.191 (0.145-0.242)	0.231 (0.180-0.284)	0.255 (0.179-0.339)
Mexico	0.250 (0.155-0.344)	0.309 (0.236-0.373)	0.354 (0.301-0.406)	0.384 (0.342-0.427)	0.398 (0.334-0.460)
Micronesia (Federated States of)	0.378 (0.271-0.496)	0.380 (0.302-0.463)	0.372 (0.305-0.442)	0.357 (0.300-0.415)	0.337 (0.256-0.419)
Moldova	0.269 (0.173-0.371)	0.300 (0.233-0.372)	0.311 (0.248-0.373)	0.317 (0.259-0.373)	0.360 (0.290-0.432)
Mongolia	0.242 (0.137-0.362)	0.274 (0.192-0.361)	0.296 (0.222-0.369)	0.324 (0.258-0.390)	0.360 (0.280-0.439)
Montenegro	0.329 (0.218-0.451)	0.373 (0.297-0.454)	0.391 (0.332-0.454)	0.418 (0.366-0.475)	0.438 (0.364-0.514)
Morocco	0.221 (0.132-0.320)	0.255 (0.193-0.324)	0.289 (0.235-0.348)	0.323 (0.277-0.375)	0.354 (0.285-0.424)
Mozambique	0.057 (0.020-0.107)	0.064 (0.033-0.106)	0.074 (0.045-0.110)	0.089 (0.062-0.121)	0.105 (0.065-0.152)
Myanmar	0.045 (0.015-0.105)	0.050 (0.025-0.088)	0.060 (0.038-0.091)	0.082 (0.058-0.111)	0.126 (0.083-0.177)
Namibia	0.112 (0.055-0.189)	0.121 (0.076-0.173)	0.133 (0.094-0.175)	0.161 (0.123-0.206)	0.191 (0.134-0.259)
Nauru	0.328 (0.237-0.433)	0.331 (0.268-0.398)	0.329 (0.271-0.391)	0.339 (0.276-0.407)	0.342 (0.253-0.439)
Nepal	0.060 (0.026-0.109)	0.068 (0.039-0.102)	0.084 (0.058-0.117)	0.106 (0.078-0.140)	0.133 (0.088-0.186)
Netherlands	0.327 (0.252-0.404)	0.370 (0.321-0.420)	0.406 (0.363-0.449)	0.434 (0.397-0.475)	0.443 (0.381-0.503)
New Zealand	0.357 (0.274-0.443)	0.395 (0.338-0.449)	0.417 (0.369-0.464)	0.418 (0.378-0.457)	0.408 (0.355-0.458)
Nicaragua	0.223 (0.127-0.322)	0.257 (0.182-0.327)	0.275 (0.211-0.338)	0.307 (0.257-0.361)	0.333 (0.253-0.406)
Niger	0.069 (0.032-0.120)	0.070 (0.039-0.104)	0.079 (0.050-0.111)	0.090 (0.062-0.121)	0.096 (0.060-0.141)
Nigeria	0.094 (0.048-0.160)	0.108 (0.075-0.148)	0.120 (0.090-0.154)	0.146 (0.117-0.178)	0.183 (0.132-0.238)
Niue	0.391 (0.272-0.516)	0.394 (0.307-0.485)	0.379 (0.306-0.456)	0.352 (0.291-0.418)	0.320 (0.240-0.401)
North Korea	0.105 (0.054-0.172)	0.131 (0.087-0.178)	0.150 (0.107-0.195)	0.150 (0.103-0.207)	0.169 (0.097-0.262)
Norway	0.313 (0.237-0.384)	0.372 (0.323-0.420)	0.400 (0.356-0.444)	0.412 (0.370-0.451)	0.413 (0.347-0.479)

Occupied Palestinian Territory	0.293 (0.195-0.403)	0.315 (0.245-0.390)	0.334 (0.275-0.397)	0.355 (0.304-0.410)	0.364 (0.280-0.444)
Oman	0.254 (0.161-0.359)	0.304 (0.241-0.370)	0.343 (0.293-0.392)	0.369 (0.322-0.414)	0.382 (0.313-0.452)
Pakistan	0.074 (0.033-0.124)	0.093 (0.061-0.132)	0.117 (0.085-0.155)	0.142 (0.109-0.182)	0.170 (0.116-0.230)
Palau	0.387 (0.276-0.504)	0.379 (0.307-0.457)	0.364 (0.299-0.432)	0.351 (0.291-0.414)	0.342 (0.265-0.423)
Panama	0.254 (0.146-0.354)	0.299 (0.230-0.368)	0.332 (0.276-0.391)	0.365 (0.319-0.415)	0.382 (0.313-0.453)
Papua New Guinea	0.255 (0.146-0.390)	0.268 (0.183-0.370)	0.281 (0.205-0.370)	0.289 (0.216-0.364)	0.294 (0.209-0.383)
Paraguay	0.219 (0.125-0.319)	0.255 (0.186-0.325)	0.299 (0.239-0.359)	0.340 (0.284-0.396)	0.365 (0.294-0.445)
Peru	0.243 (0.146-0.348)	0.276 (0.196-0.358)	0.309 (0.240-0.384)	0.345 (0.286-0.410)	0.377 (0.302-0.454)
Philippines	0.091 (0.046-0.150)	0.106 (0.073-0.146)	0.123 (0.089-0.159)	0.148 (0.112-0.186)	0.177 (0.121-0.238)
Poland	0.350 (0.261-0.446)	0.379 (0.324-0.436)	0.393 (0.345-0.443)	0.411 (0.370-0.455)	0.423 (0.358-0.486)
Portugal	0.309 (0.224-0.408)	0.340 (0.285-0.403)	0.386 (0.338-0.436)	0.421 (0.376-0.468)	0.428 (0.352-0.496)
Puerto Rico	0.237 (0.151-0.327)	0.278 (0.211-0.345)	0.318 (0.256-0.378)	0.348 (0.293-0.403)	0.360 (0.291-0.436)
Qatar	0.291 (0.199-0.388)	0.354 (0.280-0.430)	0.373 (0.318-0.431)	0.385 (0.330-0.440)	0.383 (0.308-0.457)
Romania	0.325 (0.227-0.431)	0.375 (0.309-0.441)	0.389 (0.334-0.444)	0.407 (0.356-0.459)	0.430 (0.358-0.503)
Russian Federation	0.300 (0.219-0.390)	0.335 (0.289-0.383)	0.352 (0.316-0.388)	0.374 (0.333-0.412)	0.407 (0.341-0.473)
Rwanda	0.045 (0.016-0.097)	0.053 (0.025-0.098)	0.062 (0.035-0.101)	0.075 (0.050-0.110)	0.093 (0.058-0.141)
Saint Kitts and Nevis	0.206 (0.124-0.299)	0.247 (0.182-0.318)	0.283 (0.226-0.345)	0.311 (0.253-0.373)	0.315 (0.233-0.405)
Saint Lucia	0.182 (0.108-0.274)	0.212 (0.154-0.276)	0.253 (0.202-0.312)	0.296 (0.239-0.358)	0.311 (0.231-0.403)
Saint Vincent and the Grenadines	0.178 (0.102-0.276)	0.208 (0.151-0.274)	0.250 (0.197-0.311)	0.297 (0.243-0.353)	0.334 (0.257-0.410)
Samoa	0.387 (0.268-0.505)	0.387 (0.302-0.476)	0.380 (0.308-0.456)	0.362 (0.300-0.426)	0.342 (0.261-0.424)
Sao Tome and Principe	0.096 (0.048-0.160)	0.105 (0.069-0.149)	0.127 (0.090-0.170)	0.153 (0.115-0.195)	0.188 (0.128-0.252)
Saudi Arabia	0.281 (0.179-0.396)	0.338 (0.277-0.403)	0.366 (0.324-0.410)	0.376 (0.337-0.416)	0.374 (0.309-0.440)
Senegal	0.097 (0.051-0.161)	0.103 (0.062-0.148)	0.116 (0.079-0.157)	0.138 (0.102-0.178)	0.157 (0.106-0.211)
Serbia	0.317 (0.223-0.419)	0.358 (0.294-0.424)	0.377 (0.320-0.433)	0.400 (0.352-0.449)	0.417 (0.351-0.486)
Seychelles	0.139 (0.080-0.212)	0.177 (0.124-0.236)	0.218 (0.164-0.277)	0.256 (0.198-0.316)	0.277 (0.206-0.353)
Sierra Leone	0.093 (0.046-0.158)	0.100 (0.063-0.145)	0.106 (0.074-0.141)	0.117 (0.087-0.149)	0.139 (0.095-0.189)
Singapore	0.103 (0.059-0.160)	0.172 (0.133-0.214)	0.232 (0.195-0.273)	0.273 (0.236-0.314)	0.317 (0.263-0.377)
Slovakia	0.353 (0.257-0.460)	0.385 (0.323-0.452)	0.408 (0.354-0.459)	0.419 (0.372-0.466)	0.427 (0.354-0.500)
Slovenia	0.332 (0.233-0.441)	0.373 (0.309-0.443)	0.395 (0.339-0.455)	0.422 (0.368-0.477)	0.434 (0.358-0.513)
Solomon Islands	0.224 (0.122-0.349)	0.247 (0.163-0.344)	0.257 (0.184-0.339)	0.258 (0.189-0.331)	0.264 (0.178-0.350)
Somalia	0.088 (0.040-0.154)	0.095 (0.055-0.146)	0.103 (0.067-0.148)	0.114 (0.075-0.158)	0.127 (0.075-0.187)
South Africa	0.158 (0.097-0.240)	0.182 (0.137-0.239)	0.203 (0.160-0.250)	0.228 (0.190-0.270)	0.257 (0.201-0.318)
South Korea	0.064 (0.034-0.109)	0.135 (0.102-0.175)	0.212 (0.173-0.252)	0.265 (0.227-0.301)	0.333 (0.274-0.393)
Spain	0.338 (0.256-0.428)	0.392 (0.340-0.443)	0.427 (0.386-0.468)	0.442 (0.403-0.481)	0.438 (0.374-0.500)
Sri Lanka	0.068 (0.032-0.119)	0.075 (0.048-0.107)	0.090 (0.064-0.122)	0.112 (0.082-0.144)	0.140 (0.090-0.202)
Sudan	0.093 (0.047-0.154)	0.108 (0.070-0.157)	0.122 (0.085-0.166)	0.148 (0.110-0.191)	0.173 (0.118-0.240)
Suriname	0.212 (0.128-0.303)	0.246 (0.181-0.315)	0.271 (0.213-0.333)	0.313 (0.255-0.370)	0.335 (0.261-0.410)
Swaziland	0.100 (0.050-0.168)	0.120 (0.079-0.169)	0.143 (0.103-0.192)	0.169 (0.127-0.220)	0.186 (0.125-0.260)
Sweden	0.316 (0.245-0.388)	0.364 (0.324-0.404)	0.394 (0.359-0.431)	0.408 (0.374-0.444)	0.412 (0.353-0.471)
Switzerland	0.351 (0.257-0.442)	0.399 (0.337-0.459)	0.425 (0.377-0.471)	0.428 (0.386-0.470)	0.426 (0.365-0.486)
Syrian Arab Republic	0.247 (0.159-0.347)	0.311 (0.242-0.379)	0.329 (0.274-0.386)	0.357 (0.310-0.404)	0.374 (0.304-0.442)
Taiwan	0.130 (0.078-0.190)	0.185 (0.139-0.231)	0.255 (0.204-0.303)	0.314 (0.259-0.368)	0.339 (0.259-0.420)
Tajikistan	0.246 (0.143-0.360)	0.270 (0.188-0.356)	0.275 (0.200-0.351)	0.271 (0.204-0.344)	0.302 (0.222-0.396)
Tanzania	0.073 (0.032-0.128)	0.081 (0.049-0.121)	0.091 (0.063-0.128)	0.103 (0.079-0.136)	0.127 (0.088-0.174)
Thailand	0.081 (0.041-0.135)	0.095 (0.062-0.132)	0.120 (0.090-0.155)	0.158 (0.127-0.192)	0.201 (0.145-0.265)
Timor-Leste	0.046 (0.020-0.091)	0.047 (0.027-0.073)	0.056 (0.035-0.082)	0.066 (0.046-0.094)	0.086 (0.054-0.130)
Togo	0.072 (0.033-0.125)	0.081 (0.050-0.119)	0.095 (0.067-0.128)	0.111 (0.084-0.144)	0.129 (0.088-0.180)
Tokelau	0.398 (0.245-0.565)	0.395 (0.288-0.512)	0.378 (0.292-0.468)	0.350 (0.270-0.428)	0.316 (0.216-0.416)
Tonga	0.404 (0.288-0.526)	0.400 (0.316-0.492)	0.385 (0.313-0.463)	0.364 (0.305-0.431)	0.339 (0.259-0.425)
Trinidad and Tobago	0.218 (0.107-0.342)	0.254 (0.170-0.349)	0.266 (0.201-0.338)	0.294 (0.229-0.366)	0.304 (0.208-0.416)
Tunisia	0.244 (0.158-0.340)	0.291 (0.226-0.356)	0.328 (0.272-0.382)	0.362 (0.310-0.406)	0.386 (0.322-0.449)
Turkey	0.296 (0.204-0.398)	0.334 (0.276-0.396)	0.367 (0.321-0.412)	0.395 (0.355-0.433)	0.409 (0.354-0.467)
Turkmenistan	0.254 (0.154-0.364)	0.286 (0.209-0.370)	0.312 (0.243-0.389)	0.329 (0.268-0.397)	0.362 (0.283-0.445)
Tuvalu	0.375 (0.257-0.501)	0.378 (0.294-0.467)	0.374 (0.303-0.445)	0.364 (0.300-0.429)	0.353 (0.273-0.442)
Uganda	0.062 (0.025-0.116)	0.064 (0.034-0.107)	0.072 (0.039-0.111)	0.088 (0.055-0.127)	0.111 (0.070-0.165)
Ukraine	0.294 (0.207-0.396)	0.331 (0.272-0.398)	0.349 (0.292-0.408)	0.362 (0.303-0.417)	0.399 (0.323-0.475)
United Arab Emirates	0.348 (0.224-0.477)	0.394 (0.316-0.471)	0.412 (0.357-0.467)	0.404 (0.351-0.453)	0.396 (0.325-0.465)
United Kingdom	0.360 (0.285-0.437)	0.394 (0.351-0.436)	0.416 (0.383-0.448)	0.423 (0.394-0.452)	0.415 (0.367-0.462)
United States of America	0.368 (0.296-0.443)	0.398 (0.352-0.444)	0.412 (0.370-0.453)	0.407 (0.371-0.442)	0.392 (0.337-0.446)
Uruguay	0.289 (0.184-0.407)	0.320 (0.246-0.400)	0.348 (0.288-0.410)	0.379 (0.326-0.434)	0.393 (0.320-0.470)
Uzbekistan	0.254 (0.139-0.376)	0.284 (0.200-0.376)	0.307 (0.236-0.383)	0.317 (0.253-0.386)	0.343 (0.259-0.427)
Vanuatu	0.275 (0.166-0.405)	0.284 (0.197-0.377)	0.291 (0.216-0.371)	0.296 (0.229-0.366)	0.299 (0.219-0.384)
Venezuela	0.281 (0.180-0.385)	0.329 (0.259-0.401)	0.359 (0.303-0.418)	0.385 (0.338-0.434)	0.404 (0.337-0.471)
Viet Nam	0.050 (0.025-0.101)	0.051 (0.031-0.076)	0.062 (0.042-0.084)	0.090 (0.066-0.115)	0.140 (0.097-0.194)
Yemen	0.200 (0.116-0.305)	0.220 (0.156-0.294)	0.241 (0.186-0.306)	0.261 (0.207-0.319)	0.282 (0.207-0.360)
Zambia	0.098 (0.054-0.156)	0.111 (0.073-0.157)	0.118 (0.084-0.156)	0.129 (0.099-0.165)	0.150 (0.106-0.208)
Zimbabwe	0.097 (0.049-0.161)	0.111 (0.073-0.154)	0.126 (0.092-0.167)	0.145 (0.110-0.183)	0.159 (0.105-0.219)

Women, 25 ≤ BMI < 30 kg/m^2

Country	Year				
	1975	1985	1995	2005	2014
Afghanistan	0.062 (0.031-0.108)	0.080 (0.050-0.118)	0.099 (0.071-0.135)	0.120 (0.090-0.157)	0.152 (0.107-0.206)
Albania	0.235 (0.144-0.339)	0.252 (0.187-0.324)	0.261 (0.209-0.317)	0.274 (0.226-0.325)	0.283 (0.217-0.353)
Algeria	0.235 (0.147-0.336)	0.268 (0.206-0.333)	0.293 (0.247-0.342)	0.309 (0.265-0.355)	0.316 (0.252-0.383)
American Samoa	0.311 (0.226-0.403)	0.294 (0.232-0.363)	0.277 (0.221-0.336)	0.265 (0.208-0.323)	0.257 (0.182-0.336)
Andorra	0.285 (0.201-0.373)	0.301 (0.243-0.364)	0.307 (0.257-0.359)	0.310 (0.262-0.359)	0.310 (0.244-0.380)
Angola	0.108 (0.053-0.183)	0.133 (0.086-0.189)	0.160 (0.116-0.211)	0.189 (0.142-0.243)	0.220 (0.154-0.295)
Antigua and Barbuda	0.246 (0.155-0.350)	0.273 (0.204-0.350)	0.294 (0.231-0.357)	0.301 (0.238-0.366)	0.303 (0.216-0.392)
Argentina	0.288 (0.196-0.387)	0.299 (0.233-0.370)	0.305 (0.251-0.361)	0.304 (0.257-0.349)	0.295 (0.233-0.360)
Armenia	0.260 (0.156-0.382)	0.285 (0.210-0.371)	0.295 (0.235-0.360)	0.307 (0.255-0.362)	0.319 (0.249-0.396)
Australia	0.237 (0.174-0.301)	0.261 (0.222-0.302)	0.282 (0.248-0.318)	0.294 (0.261-0.326)	0.297 (0.246-0.350)
Austria	0.230 (0.166-0.302)	0.251 (0.207-0.298)	0.269 (0.231-0.311)	0.283 (0.245-0.322)	0.292 (0.231-0.353)
Azerbaijan	0.256 (0.161-0.363)	0.282 (0.212-0.358)	0.295 (0.237-0.355)	0.308 (0.254-0.364)	0.320 (0.245-0.398)
Bahamas	0.285 (0.196-0.383)	0.299 (0.236-0.369)	0.305 (0.250-0.365)	0.303 (0.250-0.358)	0.298 (0.229-0.371)
Bahrain	0.300 (0.203-0.401)	0.312 (0.248-0.376)	0.318 (0.267-0.368)	0.322 (0.276-0.369)	0.324 (0.258-0.391)
Bangladesh	0.055 (0.028-0.092)	0.072 (0.047-0.101)	0.093 (0.069-0.119)	0.121 (0.096-0.149)	0.155 (0.114-0.204)
Barbados	0.229 (0.145-0.325)	0.257 (0.193-0.326)	0.277 (0.224-0.332)	0.295 (0.242-0.349)	0.300 (0.223-0.381)
Belarus	0.266 (0.174-0.374)	0.286 (0.222-0.355)	0.292 (0.240-0.346)	0.292 (0.243-0.342)	0.294 (0.224-0.368)
Belgium	0.269 (0.192-0.351)	0.281 (0.235-0.330)	0.291 (0.256-0.330)	0.297 (0.261-0.336)	0.296 (0.237-0.354)
Belize	0.236 (0.146-0.341)	0.260 (0.198-0.330)	0.282 (0.231-0.337)	0.300 (0.251-0.352)	0.307 (0.237-0.382)
Benin	0.106 (0.058-0.167)	0.137 (0.098-0.181)	0.166 (0.131-0.203)	0.196 (0.164-0.228)	0.221 (0.172-0.272)
Bermuda	0.307 (0.207-0.415)	0.308 (0.233-0.389)	0.309 (0.247-0.376)	0.302 (0.246-0.361)	0.288 (0.216-0.367)
Bhutan	0.064 (0.031-0.109)	0.088 (0.056-0.127)	0.123 (0.089-0.162)	0.163 (0.128-0.205)	0.202 (0.150-0.264)
Bolivia	0.257 (0.162-0.360)	0.291 (0.220-0.368)	0.323 (0.265-0.385)	0.341 (0.289-0.397)	0.343 (0.270-0.417)
Bosnia and Herzegovina	0.215 (0.115-0.346)	0.238 (0.162-0.328)	0.254 (0.195-0.320)	0.280 (0.226-0.336)	0.283 (0.219-0.351)
Botswana	0.131 (0.066-0.217)	0.181 (0.121-0.251)	0.230 (0.176-0.291)	0.253 (0.201-0.307)	0.263 (0.196-0.334)
Brazil	0.228 (0.153-0.315)	0.261 (0.210-0.317)	0.280 (0.239-0.322)	0.288 (0.253-0.325)	0.290 (0.235-0.348)
Brunei Darussalam	0.193 (0.093-0.320)	0.216 (0.136-0.313)	0.238 (0.178-0.306)	0.255 (0.201-0.313)	0.256 (0.184-0.339)
Bulgaria	0.280 (0.189-0.381)	0.292 (0.230-0.360)	0.292 (0.243-0.346)	0.291 (0.244-0.342)	0.287 (0.218-0.360)
Burkina Faso	0.080 (0.040-0.134)	0.103 (0.069-0.143)	0.131 (0.100-0.166)	0.168 (0.136-0.201)	0.193 (0.146-0.245)
Burundi	0.072 (0.031-0.131)	0.093 (0.054-0.144)	0.120 (0.082-0.168)	0.150 (0.110-0.194)	0.182 (0.128-0.242)
Cabo Verde	0.101 (0.053-0.163)	0.137 (0.095-0.182)	0.179 (0.139-0.223)	0.218 (0.179-0.259)	0.236 (0.181-0.296)
Cambodia	0.053 (0.023-0.096)	0.071 (0.045-0.102)	0.098 (0.072-0.127)	0.130 (0.102-0.160)	0.165 (0.122-0.215)
Cameroon	0.128 (0.075-0.195)	0.169 (0.125-0.216)	0.196 (0.158-0.235)	0.224 (0.192-0.259)	0.246 (0.195-0.302)
Canada	0.242 (0.180-0.310)	0.274 (0.233-0.318)	0.297 (0.259-0.336)	0.310 (0.272-0.347)	0.312 (0.256-0.371)
Central African Republic	0.106 (0.053-0.176)	0.127 (0.081-0.183)	0.146 (0.103-0.192)	0.168 (0.125-0.214)	0.191 (0.132-0.259)
Chad	0.092 (0.049-0.148)	0.113 (0.078-0.153)	0.140 (0.107-0.175)	0.174 (0.140-0.211)	0.201 (0.150-0.258)
Chile	0.293 (0.195-0.402)	0.312 (0.242-0.386)	0.316 (0.264-0.370)	0.313 (0.270-0.360)	0.303 (0.242-0.368)
China	0.096 (0.053-0.152)	0.123 (0.096-0.155)	0.159 (0.136-0.183)	0.208 (0.180-0.238)	0.249 (0.196-0.306)
China (Hong Kong SAR)	0.242 (0.142-0.362)	0.252 (0.184-0.329)	0.254 (0.197-0.314)	0.250 (0.194-0.308)	0.250 (0.171-0.335)
Colombia	0.274 (0.181-0.373)	0.306 (0.242-0.374)	0.326 (0.277-0.376)	0.335 (0.295-0.377)	0.334 (0.272-0.397)
Comoros	0.124 (0.069-0.191)	0.146 (0.103-0.194)	0.172 (0.133-0.216)	0.197 (0.162-0.236)	0.220 (0.169-0.275)
Congo	0.133 (0.076-0.204)	0.167 (0.119-0.220)	0.194 (0.149-0.242)	0.215 (0.170-0.263)	0.231 (0.169-0.296)
Cook Islands	0.329 (0.226-0.444)	0.310 (0.241-0.384)	0.288 (0.226-0.354)	0.274 (0.217-0.334)	0.264 (0.193-0.339)
Costa Rica	0.254 (0.162-0.356)	0.287 (0.221-0.360)	0.319 (0.266-0.374)	0.332 (0.286-0.379)	0.330 (0.267-0.396)
Cote d'Ivoire	0.143 (0.086-0.214)	0.168 (0.125-0.216)	0.190 (0.153-0.229)	0.217 (0.183-0.254)	0.236 (0.184-0.291)
Croatia	0.243 (0.157-0.345)	0.267 (0.206-0.332)	0.276 (0.227-0.329)	0.285 (0.238-0.335)	0.287 (0.221-0.360)
Cuba	0.262 (0.173-0.360)	0.290 (0.226-0.357)	0.305 (0.251-0.361)	0.316 (0.265-0.368)	0.320 (0.253-0.391)
Cyprus	0.226 (0.149-0.314)	0.273 (0.216-0.333)	0.293 (0.245-0.343)	0.303 (0.255-0.352)	0.307 (0.240-0.380)
Czech Republic	0.294 (0.204-0.393)	0.301 (0.242-0.366)	0.299 (0.250-0.349)	0.295 (0.249-0.342)	0.291 (0.226-0.361)
Denmark	0.250 (0.182-0.324)	0.273 (0.227-0.320)	0.284 (0.244-0.325)	0.289 (0.248-0.331)	0.293 (0.233-0.354)
Djibouti	0.171 (0.093-0.267)	0.185 (0.116-0.264)	0.202 (0.147-0.264)	0.209 (0.158-0.265)	0.220 (0.151-0.297)
Dominica	0.236 (0.155-0.334)	0.269 (0.208-0.337)	0.295 (0.242-0.351)	0.304 (0.254-0.356)	0.305 (0.237-0.373)
Dominican Republic	0.221 (0.141-0.313)	0.261 (0.205-0.323)	0.284 (0.238-0.336)	0.301 (0.256-0.349)	0.305 (0.240-0.374)
DR Congo	0.111 (0.060-0.174)	0.132 (0.089-0.181)	0.154 (0.112-0.199)	0.176 (0.133-0.222)	0.201 (0.144-0.268)
Ecuador	0.261 (0.162-0.370)	0.301 (0.228-0.380)	0.330 (0.270-0.394)	0.343 (0.291-0.398)	0.345 (0.274-0.421)
Egypt	0.263 (0.168-0.372)	0.289 (0.232-0.353)	0.302 (0.259-0.348)	0.309 (0.271-0.350)	0.305 (0.245-0.367)
El Salvador	0.255 (0.164-0.357)	0.287 (0.222-0.358)	0.314 (0.263-0.370)	0.329 (0.285-0.374)	0.331 (0.268-0.398)
Equatorial Guinea	0.110 (0.054-0.182)	0.136 (0.089-0.190)	0.168 (0.121-0.220)	0.224 (0.166-0.288)	0.246 (0.163-0.338)
Eritrea	0.086 (0.044-0.142)	0.104 (0.069-0.145)	0.130 (0.098-0.165)	0.159 (0.125-0.196)	0.184 (0.133-0.240)
Estonia	0.290 (0.200-0.392)	0.296 (0.237-0.360)	0.294 (0.250-0.341)	0.292 (0.255-0.330)	0.289 (0.232-0.349)
Ethiopia	0.086 (0.044-0.147)	0.105 (0.070-0.148)	0.127 (0.094-0.162)	0.152 (0.119-0.187)	0.182 (0.133-0.237)
Fiji	0.272 (0.159-0.398)	0.296 (0.209-0.388)	0.314 (0.242-0.390)	0.325 (0.260-0.392)	0.325 (0.252-0.406)
Finland	0.221 (0.159-0.291)	0.255 (0.215-0.299)	0.279 (0.245-0.317)	0.288 (0.254-0.324)	0.287 (0.232-0.342)
France	0.244 (0.171-0.323)	0.260 (0.210-0.313)	0.276 (0.231-0.323)	0.289 (0.243-0.335)	0.296 (0.232-0.366)
French Polynesia	0.306 (0.202-0.419)	0.296 (0.216-0.384)	0.286 (0.217-0.361)	0.279 (0.220-0.344)	0.272 (0.201-0.352)
Gabon	0.158 (0.082-0.255)	0.204 (0.141-0.276)	0.230 (0.175-0.291)	0.249 (0.195-0.305)	0.251 (0.183-0.323)
Gambia	0.110 (0.061-0.172)	0.138 (0.097-0.183)	0.171 (0.132-0.213)	0.204 (0.168-0.243)	0.221 (0.168-0.278)
Georgia	0.254 (0.161-0.365)	0.285 (0.213-0.363)	0.295 (0.235-0.358)	0.307 (0.254-0.365)	0.310 (0.241-0.386)

Germany	0.236 (0.174-0.305)	0.255 (0.217-0.295)	0.271 (0.239-0.303)	0.281 (0.248-0.315)	0.284 (0.228-0.341)
Ghana	0.136 (0.080-0.203)	0.159 (0.116-0.207)	0.190 (0.152-0.229)	0.222 (0.183-0.263)	0.243 (0.187-0.302)
Greece	0.261 (0.181-0.349)	0.285 (0.231-0.343)	0.299 (0.252-0.346)	0.309 (0.262-0.356)	0.309 (0.244-0.380)
Greenland	0.243 (0.174-0.317)	0.267 (0.220-0.319)	0.282 (0.242-0.324)	0.290 (0.252-0.330)	0.292 (0.233-0.356)
Grenada	0.223 (0.140-0.319)	0.255 (0.194-0.323)	0.283 (0.230-0.340)	0.301 (0.249-0.355)	0.307 (0.235-0.386)
Guatemala	0.245 (0.154-0.348)	0.280 (0.211-0.351)	0.310 (0.258-0.365)	0.328 (0.282-0.376)	0.333 (0.266-0.402)
Guinea	0.105 (0.056-0.171)	0.139 (0.096-0.186)	0.165 (0.127-0.205)	0.194 (0.161-0.229)	0.216 (0.169-0.270)
Guinea Bissau	0.103 (0.056-0.165)	0.132 (0.092-0.177)	0.171 (0.132-0.214)	0.192 (0.155-0.230)	0.210 (0.157-0.266)
Guyana	0.221 (0.138-0.319)	0.253 (0.190-0.323)	0.278 (0.223-0.336)	0.300 (0.243-0.358)	0.306 (0.230-0.385)
Haiti	0.165 (0.097-0.251)	0.199 (0.145-0.260)	0.232 (0.183-0.283)	0.264 (0.214-0.317)	0.283 (0.212-0.358)
Honduras	0.223 (0.136-0.324)	0.263 (0.198-0.331)	0.296 (0.246-0.351)	0.317 (0.272-0.364)	0.323 (0.257-0.388)
Hungary	0.275 (0.188-0.372)	0.282 (0.225-0.344)	0.281 (0.232-0.333)	0.282 (0.237-0.330)	0.283 (0.217-0.353)
Iceland	0.253 (0.178-0.332)	0.281 (0.225-0.339)	0.290 (0.245-0.339)	0.293 (0.247-0.340)	0.292 (0.228-0.361)
India	0.060 (0.032-0.095)	0.080 (0.055-0.107)	0.104 (0.084-0.126)	0.134 (0.113-0.156)	0.166 (0.127-0.211)
Indonesia	0.070 (0.036-0.114)	0.098 (0.069-0.131)	0.135 (0.106-0.167)	0.172 (0.143-0.203)	0.202 (0.154-0.255)
Iran	0.254 (0.167-0.359)	0.290 (0.236-0.351)	0.314 (0.276-0.354)	0.333 (0.303-0.365)	0.345 (0.290-0.401)
Iraq	0.278 (0.184-0.379)	0.303 (0.238-0.370)	0.313 (0.263-0.365)	0.315 (0.268-0.364)	0.316 (0.247-0.388)
Ireland	0.185 (0.125-0.254)	0.226 (0.179-0.278)	0.263 (0.221-0.308)	0.286 (0.244-0.332)	0.298 (0.238-0.361)
Israel	0.287 (0.201-0.384)	0.301 (0.241-0.364)	0.311 (0.263-0.363)	0.315 (0.268-0.364)	0.314 (0.247-0.386)
Italy	0.257 (0.185-0.335)	0.273 (0.228-0.319)	0.289 (0.253-0.326)	0.305 (0.268-0.344)	0.316 (0.256-0.381)
Jamaica	0.247 (0.163-0.343)	0.274 (0.218-0.335)	0.292 (0.245-0.341)	0.303 (0.259-0.351)	0.304 (0.239-0.373)
Japan	0.138 (0.106-0.175)	0.148 (0.127-0.171)	0.155 (0.136-0.177)	0.159 (0.138-0.183)	0.159 (0.121-0.200)
Jordan	0.278 (0.181-0.386)	0.299 (0.240-0.362)	0.312 (0.267-0.361)	0.312 (0.273-0.354)	0.306 (0.246-0.371)
Kazakhstan	0.247 (0.156-0.353)	0.274 (0.206-0.348)	0.292 (0.236-0.351)	0.306 (0.251-0.362)	0.316 (0.240-0.400)
Kenya	0.099 (0.053-0.160)	0.131 (0.092-0.178)	0.164 (0.128-0.203)	0.199 (0.163-0.240)	0.228 (0.171-0.292)
Kiribati	0.322 (0.220-0.437)	0.338 (0.261-0.422)	0.338 (0.268-0.412)	0.326 (0.261-0.394)	0.313 (0.230-0.402)
Kuwait	0.300 (0.195-0.410)	0.315 (0.251-0.381)	0.314 (0.266-0.364)	0.310 (0.266-0.355)	0.303 (0.241-0.370)
Kyrgyzstan	0.217 (0.128-0.322)	0.249 (0.180-0.323)	0.272 (0.215-0.332)	0.292 (0.240-0.348)	0.312 (0.242-0.386)
Lao PDR	0.061 (0.030-0.104)	0.078 (0.051-0.112)	0.102 (0.075-0.133)	0.138 (0.109-0.171)	0.174 (0.131-0.225)
Latvia	0.286 (0.193-0.395)	0.295 (0.233-0.364)	0.294 (0.242-0.348)	0.294 (0.246-0.343)	0.292 (0.225-0.363)
Lebanon	0.289 (0.201-0.390)	0.316 (0.252-0.384)	0.337 (0.280-0.396)	0.346 (0.288-0.404)	0.332 (0.266-0.400)
Lesotho	0.142 (0.072-0.232)	0.180 (0.124-0.247)	0.220 (0.170-0.278)	0.252 (0.204-0.304)	0.267 (0.203-0.337)
Liberia	0.127 (0.073-0.193)	0.158 (0.113-0.208)	0.167 (0.118-0.222)	0.193 (0.156-0.235)	0.219 (0.166-0.279)
Libya	0.275 (0.167-0.391)	0.306 (0.241-0.373)	0.319 (0.270-0.369)	0.322 (0.279-0.366)	0.318 (0.258-0.382)
Lithuania	0.278 (0.186-0.387)	0.294 (0.233-0.360)	0.295 (0.247-0.347)	0.296 (0.251-0.344)	0.293 (0.226-0.363)
Luxembourg	0.232 (0.164-0.307)	0.250 (0.199-0.305)	0.267 (0.216-0.320)	0.284 (0.240-0.328)	0.286 (0.224-0.353)
Macedonia (TFYR)	0.264 (0.174-0.370)	0.279 (0.215-0.348)	0.283 (0.232-0.337)	0.286 (0.236-0.335)	0.289 (0.222-0.361)
Madagascar	0.103 (0.057-0.167)	0.125 (0.088-0.168)	0.149 (0.114-0.185)	0.176 (0.142-0.210)	0.203 (0.150-0.260)
Malawi	0.093 (0.047-0.156)	0.120 (0.081-0.163)	0.146 (0.112-0.184)	0.177 (0.145-0.213)	0.208 (0.156-0.264)
Malaysia	0.106 (0.059-0.164)	0.145 (0.105-0.191)	0.185 (0.147-0.227)	0.215 (0.182-0.252)	0.232 (0.180-0.289)
Maldives	0.089 (0.047-0.145)	0.125 (0.086-0.171)	0.162 (0.120-0.209)	0.205 (0.155-0.258)	0.232 (0.170-0.298)
Mali	0.097 (0.052-0.158)	0.123 (0.085-0.167)	0.152 (0.115-0.190)	0.182 (0.149-0.217)	0.204 (0.155-0.260)
Malta	0.271 (0.167-0.391)	0.287 (0.224-0.354)	0.295 (0.245-0.345)	0.300 (0.252-0.351)	0.300 (0.232-0.373)
Marshall Islands	0.335 (0.225-0.453)	0.328 (0.250-0.411)	0.311 (0.249-0.378)	0.299 (0.240-0.363)	0.287 (0.209-0.370)
Mauritania	0.101 (0.052-0.164)	0.134 (0.093-0.182)	0.166 (0.125-0.213)	0.192 (0.152-0.235)	0.211 (0.157-0.272)
Mauritius	0.163 (0.098-0.244)	0.192 (0.142-0.248)	0.223 (0.178-0.273)	0.249 (0.203-0.299)	0.264 (0.196-0.339)
Mexico	0.288 (0.194-0.388)	0.314 (0.254-0.380)	0.330 (0.286-0.377)	0.333 (0.296-0.371)	0.328 (0.270-0.386)
Micronesia (Federated States of)	0.308 (0.207-0.421)	0.313 (0.240-0.393)	0.310 (0.248-0.375)	0.303 (0.250-0.360)	0.295 (0.225-0.370)
Moldova	0.255 (0.161-0.364)	0.279 (0.215-0.347)	0.285 (0.233-0.341)	0.283 (0.234-0.335)	0.287 (0.221-0.357)
Mongolia	0.218 (0.129-0.328)	0.250 (0.180-0.329)	0.270 (0.212-0.332)	0.286 (0.234-0.338)	0.302 (0.235-0.376)
Montenegro	0.246 (0.147-0.367)	0.273 (0.201-0.354)	0.284 (0.229-0.341)	0.285 (0.238-0.337)	0.287 (0.220-0.357)
Morocco	0.228 (0.143-0.333)	0.266 (0.207-0.333)	0.294 (0.247-0.345)	0.312 (0.266-0.360)	0.320 (0.255-0.388)
Mozambique	0.088 (0.042-0.152)	0.113 (0.073-0.162)	0.143 (0.107-0.185)	0.172 (0.137-0.209)	0.198 (0.148-0.253)
Myanmar	0.062 (0.023-0.120)	0.079 (0.046-0.120)	0.103 (0.074-0.137)	0.137 (0.108-0.170)	0.179 (0.135-0.231)
Namibia	0.159 (0.082-0.259)	0.189 (0.128-0.258)	0.218 (0.167-0.273)	0.246 (0.199-0.295)	0.262 (0.202-0.327)
Nauru	0.300 (0.211-0.398)	0.280 (0.218-0.347)	0.276 (0.220-0.334)	0.278 (0.217-0.342)	0.269 (0.184-0.361)
Nepal	0.053 (0.025-0.091)	0.069 (0.044-0.101)	0.096 (0.071-0.125)	0.131 (0.104-0.162)	0.169 (0.125-0.221)
Netherlands	0.229 (0.164-0.304)	0.254 (0.212-0.303)	0.278 (0.243-0.318)	0.295 (0.260-0.331)	0.297 (0.241-0.354)
New Zealand	0.230 (0.165-0.300)	0.256 (0.212-0.302)	0.279 (0.243-0.318)	0.293 (0.261-0.327)	0.292 (0.246-0.341)
Nicaragua	0.254 (0.165-0.353)	0.281 (0.219-0.348)	0.302 (0.252-0.357)	0.316 (0.272-0.364)	0.318 (0.252-0.385)
Niger	0.087 (0.046-0.144)	0.106 (0.072-0.147)	0.131 (0.098-0.167)	0.160 (0.128-0.195)	0.187 (0.141-0.241)
Nigeria	0.118 (0.066-0.187)	0.145 (0.105-0.190)	0.174 (0.141-0.209)	0.211 (0.179-0.243)	0.241 (0.193-0.292)
Niue	0.305 (0.196-0.429)	0.305 (0.226-0.392)	0.295 (0.230-0.365)	0.284 (0.227-0.345)	0.270 (0.201-0.346)
North Korea	0.157 (0.092-0.240)	0.175 (0.126-0.232)	0.183 (0.139-0.231)	0.179 (0.128-0.235)	0.184 (0.115-0.263)
Norway	0.234 (0.166-0.307)	0.269 (0.223-0.318)	0.289 (0.250-0.330)	0.299 (0.259-0.339)	0.300 (0.239-0.362)
Occupied Palestinian Territory	0.286 (0.191-0.395)	0.307 (0.237-0.383)	0.318 (0.263-0.374)	0.317 (0.272-0.367)	0.310 (0.239-0.386)
Oman	0.233 (0.137-0.344)	0.285 (0.220-0.355)	0.315 (0.267-0.363)	0.324 (0.280-0.369)	0.325 (0.261-0.394)
Pakistan	0.077 (0.041-0.126)	0.101 (0.069-0.141)	0.127 (0.097-0.165)	0.153 (0.122-0.191)	0.178 (0.133-0.232)
Palau	0.300 (0.197-0.416)	0.302 (0.232-0.378)	0.292 (0.231-0.358)	0.283 (0.223-0.342)	0.274 (0.200-0.354)
Panama	0.275 (0.183-0.380)	0.307 (0.241-0.379)	0.328 (0.277-0.385)	0.339 (0.294-0.386)	0.334 (0.270-0.402)
Papua New Guinea	0.215 (0.114-0.338)	0.249 (0.165-0.343)	0.280 (0.208-0.360)	0.305 (0.239-0.376)	0.321 (0.238-0.409)

Paraguay	0.206 (0.129-0.298)	0.241 (0.183-0.306)	0.268 (0.218-0.322)	0.284 (0.237-0.337)	0.293 (0.229-0.361)
Peru	0.292 (0.193-0.398)	0.322 (0.247-0.398)	0.340 (0.282-0.401)	0.355 (0.310-0.403)	0.360 (0.300-0.422)
Philippines	0.096 (0.056-0.147)	0.125 (0.093-0.160)	0.154 (0.124-0.186)	0.176 (0.147-0.209)	0.198 (0.148-0.252)
Poland	0.268 (0.185-0.364)	0.278 (0.223-0.337)	0.281 (0.236-0.328)	0.285 (0.245-0.328)	0.286 (0.225-0.352)
Portugal	0.211 (0.136-0.297)	0.236 (0.183-0.294)	0.266 (0.222-0.313)	0.287 (0.244-0.331)	0.297 (0.234-0.367)
Puerto Rico	0.272 (0.182-0.368)	0.299 (0.234-0.366)	0.307 (0.251-0.366)	0.303 (0.253-0.356)	0.296 (0.231-0.365)
Qatar	0.312 (0.213-0.418)	0.308 (0.234-0.385)	0.316 (0.263-0.371)	0.311 (0.263-0.360)	0.303 (0.238-0.373)
Romania	0.259 (0.170-0.364)	0.280 (0.219-0.346)	0.286 (0.237-0.337)	0.290 (0.244-0.339)	0.293 (0.227-0.363)
Russian Federation	0.288 (0.201-0.380)	0.296 (0.246-0.348)	0.294 (0.260-0.329)	0.290 (0.253-0.329)	0.286 (0.223-0.352)
Rwanda	0.082 (0.038-0.146)	0.107 (0.064-0.160)	0.135 (0.094-0.182)	0.172 (0.131-0.216)	0.206 (0.151-0.268)
Saint Kitts and Nevis	0.233 (0.149-0.332)	0.266 (0.201-0.340)	0.286 (0.230-0.345)	0.297 (0.241-0.355)	0.299 (0.218-0.383)
Saint Lucia	0.216 (0.135-0.313)	0.250 (0.190-0.316)	0.276 (0.223-0.331)	0.294 (0.240-0.350)	0.302 (0.221-0.388)
Saint Vincent and the Grenadines	0.219 (0.136-0.316)	0.255 (0.193-0.323)	0.282 (0.229-0.338)	0.299 (0.246-0.351)	0.307 (0.236-0.382)
Samoa	0.314 (0.207-0.431)	0.313 (0.238-0.393)	0.301 (0.238-0.367)	0.288 (0.232-0.345)	0.278 (0.210-0.351)
Sao Tome and Principe	0.137 (0.080-0.207)	0.161 (0.117-0.212)	0.196 (0.154-0.243)	0.221 (0.180-0.264)	0.239 (0.181-0.298)
Saudi Arabia	0.269 (0.165-0.385)	0.300 (0.238-0.365)	0.311 (0.269-0.353)	0.310 (0.271-0.349)	0.301 (0.243-0.362)
Senegal	0.131 (0.078-0.197)	0.152 (0.109-0.198)	0.174 (0.136-0.215)	0.200 (0.165-0.238)	0.221 (0.170-0.277)
Serbia	0.246 (0.158-0.346)	0.263 (0.204-0.327)	0.270 (0.223-0.320)	0.272 (0.231-0.316)	0.275 (0.214-0.337)
Seychelles	0.187 (0.113-0.274)	0.225 (0.166-0.289)	0.248 (0.195-0.305)	0.264 (0.212-0.321)	0.268 (0.199-0.344)
Sierra Leone	0.122 (0.070-0.190)	0.146 (0.105-0.195)	0.165 (0.129-0.206)	0.190 (0.157-0.225)	0.215 (0.167-0.265)
Singapore	0.159 (0.095-0.235)	0.186 (0.145-0.234)	0.200 (0.165-0.240)	0.201 (0.168-0.239)	0.212 (0.162-0.266)
Slovakia	0.246 (0.160-0.348)	0.269 (0.212-0.332)	0.280 (0.234-0.328)	0.284 (0.240-0.329)	0.288 (0.224-0.358)
Slovenia	0.243 (0.155-0.351)	0.265 (0.205-0.332)	0.275 (0.225-0.328)	0.285 (0.238-0.335)	0.290 (0.222-0.363)
Solomon Islands	0.217 (0.116-0.346)	0.257 (0.171-0.354)	0.289 (0.216-0.370)	0.314 (0.249-0.384)	0.328 (0.245-0.414)
Somalia	0.103 (0.054-0.169)	0.123 (0.080-0.176)	0.144 (0.103-0.190)	0.162 (0.119-0.208)	0.183 (0.127-0.246)
South Africa	0.219 (0.130-0.318)	0.237 (0.175-0.303)	0.250 (0.203-0.301)	0.260 (0.222-0.299)	0.260 (0.210-0.314)
South Korea	0.078 (0.045-0.121)	0.140 (0.108-0.177)	0.192 (0.156-0.230)	0.207 (0.175-0.243)	0.241 (0.190-0.300)
Spain	0.251 (0.175-0.332)	0.276 (0.229-0.328)	0.293 (0.255-0.333)	0.302 (0.264-0.340)	0.306 (0.246-0.368)
Sri Lanka	0.086 (0.045-0.139)	0.110 (0.076-0.150)	0.141 (0.108-0.178)	0.178 (0.143-0.217)	0.216 (0.154-0.283)
Sudan	0.115 (0.063-0.181)	0.142 (0.099-0.190)	0.172 (0.131-0.215)	0.200 (0.160-0.244)	0.222 (0.165-0.285)
Suriname	0.256 (0.166-0.357)	0.286 (0.222-0.356)	0.297 (0.240-0.354)	0.304 (0.252-0.356)	0.304 (0.234-0.376)
Swaziland	0.149 (0.077-0.239)	0.189 (0.129-0.259)	0.227 (0.173-0.289)	0.256 (0.205-0.310)	0.273 (0.201-0.349)
Sweden	0.257 (0.188-0.329)	0.275 (0.232-0.319)	0.288 (0.252-0.325)	0.298 (0.261-0.336)	0.303 (0.245-0.362)
Switzerland	0.232 (0.158-0.317)	0.261 (0.210-0.317)	0.289 (0.248-0.332)	0.298 (0.257-0.338)	0.299 (0.238-0.362)
Syrian Arab Republic	0.250 (0.161-0.353)	0.289 (0.226-0.357)	0.303 (0.254-0.356)	0.318 (0.273-0.365)	0.324 (0.259-0.392)
Taiwan	0.148 (0.091-0.219)	0.192 (0.146-0.244)	0.227 (0.185-0.273)	0.247 (0.203-0.294)	0.252 (0.185-0.323)
Tajikistan	0.224 (0.134-0.332)	0.250 (0.181-0.326)	0.267 (0.209-0.327)	0.284 (0.230-0.342)	0.309 (0.236-0.388)
Tanzania	0.099 (0.053-0.160)	0.127 (0.089-0.172)	0.156 (0.122-0.193)	0.185 (0.154-0.219)	0.214 (0.166-0.266)
Thailand	0.092 (0.049-0.147)	0.124 (0.089-0.165)	0.157 (0.125-0.194)	0.193 (0.162-0.229)	0.217 (0.165-0.274)
Timor-Leste	0.053 (0.025-0.092)	0.070 (0.044-0.100)	0.092 (0.066-0.121)	0.114 (0.087-0.144)	0.141 (0.099-0.191)
Togo	0.110 (0.061-0.169)	0.133 (0.095-0.175)	0.162 (0.128-0.198)	0.194 (0.161-0.227)	0.218 (0.169-0.270)
Tokelau	0.282 (0.145-0.444)	0.296 (0.194-0.409)	0.298 (0.224-0.381)	0.294 (0.230-0.365)	0.284 (0.200-0.377)
Tonga	0.314 (0.199-0.436)	0.315 (0.236-0.399)	0.305 (0.240-0.377)	0.293 (0.236-0.353)	0.283 (0.212-0.357)
Trinidad and Tobago	0.213 (0.107-0.343)	0.248 (0.160-0.345)	0.275 (0.211-0.344)	0.297 (0.230-0.365)	0.301 (0.202-0.407)
Tunisia	0.255 (0.165-0.359)	0.292 (0.231-0.359)	0.315 (0.268-0.363)	0.326 (0.285-0.368)	0.328 (0.266-0.391)
Turkey	0.265 (0.177-0.366)	0.299 (0.242-0.362)	0.322 (0.281-0.365)	0.327 (0.293-0.363)	0.322 (0.267-0.378)
Turkmenistan	0.238 (0.148-0.343)	0.263 (0.196-0.337)	0.286 (0.228-0.346)	0.303 (0.251-0.357)	0.319 (0.247-0.397)
Tuvalu	0.314 (0.208-0.430)	0.320 (0.243-0.399)	0.308 (0.244-0.374)	0.295 (0.237-0.357)	0.282 (0.206-0.364)
Uganda	0.089 (0.044-0.151)	0.111 (0.071-0.161)	0.141 (0.102-0.186)	0.176 (0.135-0.221)	0.210 (0.157-0.270)
Ukraine	0.289 (0.195-0.394)	0.300 (0.237-0.367)	0.301 (0.249-0.353)	0.296 (0.245-0.349)	0.294 (0.225-0.368)
United Arab Emirates	0.279 (0.162-0.407)	0.308 (0.233-0.388)	0.321 (0.269-0.376)	0.318 (0.272-0.365)	0.317 (0.254-0.386)
United Kingdom	0.238 (0.175-0.303)	0.265 (0.227-0.302)	0.289 (0.261-0.317)	0.301 (0.277-0.327)	0.302 (0.259-0.346)
United States of America	0.241 (0.183-0.302)	0.262 (0.223-0.303)	0.277 (0.243-0.310)	0.282 (0.251-0.312)	0.280 (0.232-0.330)
Uruguay	0.282 (0.182-0.390)	0.294 (0.224-0.370)	0.299 (0.245-0.356)	0.299 (0.250-0.349)	0.293 (0.228-0.363)
Uzbekistan	0.224 (0.134-0.334)	0.256 (0.186-0.337)	0.281 (0.223-0.344)	0.299 (0.245-0.357)	0.320 (0.245-0.399)
Vanuatu	0.229 (0.123-0.356)	0.265 (0.180-0.362)	0.295 (0.225-0.371)	0.314 (0.253-0.382)	0.323 (0.244-0.407)
Venezuela	0.316 (0.218-0.421)	0.336 (0.267-0.408)	0.348 (0.294-0.403)	0.350 (0.303-0.397)	0.343 (0.277-0.408)
Viet Nam	0.059 (0.029-0.099)	0.073 (0.048-0.101)	0.096 (0.071-0.121)	0.133 (0.104-0.160)	0.175 (0.126-0.224)
Yemen	0.179 (0.097-0.281)	0.214 (0.151-0.286)	0.249 (0.196-0.302)	0.279 (0.228-0.332)	0.297 (0.224-0.369)
Zambia	0.146 (0.086-0.222)	0.174 (0.126-0.225)	0.191 (0.150-0.233)	0.212 (0.175-0.251)	0.235 (0.183-0.294)
Zimbabwe	0.169 (0.093-0.262)	0.205 (0.146-0.271)	0.235 (0.186-0.289)	0.257 (0.211-0.306)	0.269 (0.206-0.337)

Men, 30 ≤ BMI < 35 kg/m^2

Country	Year				
	1975	1985	1995	2005	2014
Afghanistan	0.004 (0.001-0.012)	0.006 (0.002-0.013)	0.007 (0.003-0.016)	0.010 (0.004-0.019)	0.016 (0.006-0.033)
Albania	0.041 (0.014-0.087)	0.056 (0.027-0.095)	0.071 (0.039-0.111)	0.104 (0.066-0.151)	0.139 (0.083-0.205)
Algeria	0.033 (0.012-0.068)	0.053 (0.028-0.086)	0.077 (0.046-0.113)	0.103 (0.069-0.143)	0.132 (0.083-0.193)
American Samoa	0.200 (0.124-0.289)	0.217 (0.155-0.287)	0.231 (0.168-0.297)	0.239 (0.176-0.304)	0.242 (0.164-0.330)
Andorra	0.086 (0.041-0.149)	0.118 (0.072-0.172)	0.142 (0.094-0.197)	0.174 (0.125-0.229)	0.190 (0.124-0.260)
Angola	0.005 (0.001-0.015)	0.008 (0.002-0.018)	0.012 (0.004-0.024)	0.018 (0.008-0.035)	0.035 (0.013-0.070)
Antigua and Barbuda	0.036 (0.012-0.076)	0.051 (0.024-0.090)	0.081 (0.045-0.128)	0.108 (0.064-0.161)	0.139 (0.073-0.221)
Argentina	0.100 (0.048-0.173)	0.123 (0.077-0.178)	0.143 (0.099-0.193)	0.168 (0.126-0.217)	0.198 (0.140-0.265)
Armenia	0.050 (0.014-0.112)	0.064 (0.027-0.116)	0.070 (0.034-0.118)	0.089 (0.050-0.139)	0.130 (0.074-0.200)
Australia	0.090 (0.053-0.138)	0.111 (0.083-0.143)	0.136 (0.108-0.168)	0.168 (0.138-0.199)	0.194 (0.147-0.243)
Austria	0.060 (0.030-0.100)	0.082 (0.054-0.118)	0.108 (0.075-0.147)	0.137 (0.100-0.179)	0.161 (0.105-0.223)
Azerbaijan	0.047 (0.016-0.099)	0.063 (0.031-0.108)	0.072 (0.038-0.117)	0.089 (0.051-0.136)	0.141 (0.080-0.214)
Bahamas	0.065 (0.027-0.121)	0.089 (0.051-0.141)	0.120 (0.076-0.175)	0.149 (0.101-0.203)	0.166 (0.104-0.236)
Bahrain	0.079 (0.034-0.143)	0.105 (0.065-0.152)	0.135 (0.093-0.182)	0.165 (0.120-0.213)	0.186 (0.124-0.251)
Bangladesh	0.003 (0.001-0.007)	0.004 (0.001-0.008)	0.006 (0.003-0.011)	0.009 (0.005-0.016)	0.016 (0.007-0.030)
Barbados	0.035 (0.012-0.073)	0.053 (0.028-0.087)	0.071 (0.043-0.107)	0.099 (0.063-0.142)	0.128 (0.071-0.200)
Belarus	0.052 (0.020-0.104)	0.077 (0.042-0.124)	0.097 (0.059-0.147)	0.122 (0.079-0.173)	0.160 (0.099-0.228)
Belgium	0.082 (0.043-0.134)	0.103 (0.074-0.136)	0.129 (0.100-0.161)	0.159 (0.126-0.194)	0.180 (0.129-0.238)
Belize	0.035 (0.011-0.075)	0.047 (0.024-0.079)	0.063 (0.036-0.096)	0.088 (0.056-0.128)	0.112 (0.064-0.173)
Benin	0.005 (0.001-0.014)	0.008 (0.003-0.017)	0.011 (0.005-0.022)	0.017 (0.009-0.029)	0.024 (0.011-0.046)
Bermuda	0.105 (0.046-0.186)	0.134 (0.076-0.203)	0.160 (0.100-0.228)	0.180 (0.123-0.242)	0.206 (0.133-0.285)
Bhutan	0.004 (0.001-0.010)	0.006 (0.002-0.013)	0.011 (0.005-0.022)	0.021 (0.011-0.037)	0.038 (0.018-0.069)
Bolivia	0.030 (0.009-0.065)	0.044 (0.019-0.079)	0.060 (0.031-0.100)	0.082 (0.048-0.124)	0.107 (0.058-0.168)
Bosnia and Herzegovina	0.028 (0.006-0.074)	0.039 (0.014-0.079)	0.051 (0.024-0.090)	0.095 (0.057-0.141)	0.122 (0.074-0.180)
Botswana	0.006 (0.001-0.016)	0.012 (0.004-0.024)	0.028 (0.013-0.050)	0.046 (0.024-0.074)	0.072 (0.036-0.121)
Brazil	0.033 (0.013-0.064)	0.055 (0.033-0.082)	0.080 (0.056-0.107)	0.108 (0.083-0.134)	0.140 (0.098-0.191)
Brunei Darussalam	0.021 (0.004-0.059)	0.034 (0.011-0.074)	0.054 (0.025-0.096)	0.084 (0.046-0.134)	0.112 (0.057-0.185)
Bulgaria	0.068 (0.029-0.128)	0.101 (0.061-0.153)	0.125 (0.082-0.176)	0.141 (0.098-0.193)	0.168 (0.108-0.240)
Burkina Faso	0.004 (0.001-0.012)	0.005 (0.002-0.012)	0.008 (0.003-0.016)	0.014 (0.007-0.024)	0.022 (0.009-0.040)
Burundi	0.001 (0.000-0.005)	0.002 (0.000-0.006)	0.003 (0.001-0.009)	0.005 (0.002-0.010)	0.008 (0.002-0.018)
Cabo Verde	0.005 (0.001-0.015)	0.009 (0.003-0.019)	0.018 (0.008-0.033)	0.037 (0.020-0.061)	0.060 (0.030-0.101)
Cambodia	0.002 (0.000-0.005)	0.002 (0.001-0.005)	0.004 (0.001-0.007)	0.007 (0.003-0.013)	0.014 (0.006-0.028)
Cameroon	0.007 (0.002-0.018)	0.011 (0.005-0.022)	0.017 (0.009-0.030)	0.025 (0.015-0.040)	0.036 (0.018-0.063)
Canada	0.077 (0.043-0.125)	0.102 (0.072-0.138)	0.131 (0.098-0.169)	0.165 (0.128-0.204)	0.189 (0.134-0.246)
Central African Republic	0.004 (0.001-0.014)	0.006 (0.001-0.015)	0.008 (0.003-0.017)	0.010 (0.004-0.020)	0.015 (0.005-0.033)
Chad	0.005 (0.001-0.013)	0.006 (0.002-0.013)	0.009 (0.004-0.017)	0.014 (0.007-0.024)	0.022 (0.009-0.043)
Chile	0.081 (0.033-0.150)	0.102 (0.060-0.156)	0.128 (0.088-0.173)	0.161 (0.121-0.203)	0.189 (0.134-0.251)
China	0.003 (0.001-0.008)	0.005 (0.003-0.009)	0.013 (0.009-0.017)	0.033 (0.024-0.043)	0.067 (0.042-0.096)
China (Hong Kong SAR)	0.029 (0.008-0.066)	0.041 (0.018-0.075)	0.061 (0.033-0.098)	0.084 (0.046-0.131)	0.119 (0.058-0.195)
Colombia	0.038 (0.014-0.078)	0.056 (0.030-0.089)	0.078 (0.048-0.111)	0.102 (0.072-0.136)	0.129 (0.082-0.184)
Comoros	0.005 (0.001-0.014)	0.007 (0.002-0.015)	0.010 (0.004-0.019)	0.013 (0.006-0.024)	0.019 (0.008-0.036)
Congo	0.006 (0.001-0.016)	0.011 (0.004-0.022)	0.017 (0.007-0.032)	0.025 (0.012-0.044)	0.039 (0.016-0.075)
Cook Islands	0.166 (0.083-0.266)	0.193 (0.127-0.265)	0.224 (0.155-0.295)	0.245 (0.179-0.311)	0.255 (0.179-0.334)
Costa Rica	0.037 (0.013-0.076)	0.057 (0.029-0.093)	0.083 (0.051-0.125)	0.115 (0.080-0.158)	0.147 (0.096-0.207)
Cote d'Ivoire	0.008 (0.002-0.021)	0.012 (0.005-0.024)	0.017 (0.008-0.030)	0.024 (0.013-0.041)	0.034 (0.016-0.061)
Croatia	0.046 (0.017-0.094)	0.073 (0.040-0.117)	0.098 (0.061-0.143)	0.132 (0.089-0.182)	0.169 (0.108-0.237)
Cuba	0.041 (0.016-0.080)	0.066 (0.037-0.103)	0.084 (0.052-0.122)	0.098 (0.065-0.136)	0.128 (0.079-0.187)
Cyprus	0.039 (0.014-0.079)	0.067 (0.037-0.109)	0.107 (0.068-0.155)	0.142 (0.096-0.195)	0.164 (0.101-0.237)
Czech Republic	0.098 (0.049-0.163)	0.129 (0.087-0.180)	0.150 (0.107-0.198)	0.173 (0.129-0.220)	0.197 (0.136-0.263)
Denmark	0.062 (0.035-0.096)	0.084 (0.058-0.113)	0.113 (0.082-0.148)	0.144 (0.107-0.184)	0.163 (0.110-0.219)
Djibouti	0.017 (0.004-0.043)	0.020 (0.006-0.046)	0.026 (0.010-0.051)	0.031 (0.013-0.058)	0.043 (0.016-0.086)
Dominica	0.029 (0.009-0.061)	0.043 (0.021-0.074)	0.071 (0.041-0.111)	0.097 (0.061-0.143)	0.126 (0.074-0.190)
Dominican Republic	0.021 (0.007-0.046)	0.036 (0.018-0.062)	0.054 (0.031-0.083)	0.087 (0.057-0.124)	0.122 (0.074-0.181)
DR Congo	0.005 (0.001-0.013)	0.006 (0.002-0.015)	0.009 (0.003-0.018)	0.010 (0.004-0.020)	0.016 (0.005-0.035)
Ecuador	0.029 (0.009-0.063)	0.047 (0.022-0.083)	0.069 (0.038-0.111)	0.093 (0.057-0.137)	0.122 (0.071-0.186)
Egypt	0.050 (0.019-0.099)	0.072 (0.042-0.109)	0.095 (0.065-0.134)	0.122 (0.090-0.162)	0.152 (0.100-0.214)
El Salvador	0.036 (0.012-0.076)	0.051 (0.025-0.087)	0.071 (0.041-0.110)	0.100 (0.065-0.143)	0.125 (0.075-0.187)
Equatorial Guinea	0.004 (0.001-0.014)	0.007 (0.002-0.016)	0.011 (0.004-0.023)	0.037 (0.015-0.071)	0.068 (0.024-0.138)
Eritrea	0.004 (0.001-0.010)	0.005 (0.001-0.010)	0.007 (0.003-0.013)	0.010 (0.005-0.018)	0.014 (0.005-0.027)
Estonia	0.067 (0.032-0.120)	0.087 (0.057-0.125)	0.101 (0.071-0.136)	0.130 (0.101-0.162)	0.163 (0.118-0.216)
Ethiopia	0.003 (0.001-0.010)	0.004 (0.001-0.009)	0.005 (0.002-0.010)	0.007 (0.003-0.013)	0.011 (0.004-0.023)
Fiji	0.082 (0.029-0.165)	0.111 (0.055-0.184)	0.138 (0.078-0.210)	0.168 (0.107-0.236)	0.187 (0.117-0.264)
Finland	0.060 (0.032-0.097)	0.088 (0.063-0.116)	0.118 (0.091-0.150)	0.145 (0.117-0.176)	0.164 (0.117-0.215)
France	0.070 (0.036-0.119)	0.095 (0.062-0.136)	0.122 (0.086-0.162)	0.152 (0.112-0.194)	0.172 (0.116-0.235)
French Polynesia	0.202 (0.109-0.310)	0.230 (0.149-0.321)	0.253 (0.176-0.335)	0.268 (0.202-0.338)	0.279 (0.202-0.358)

Gabon	0.009 (0.002-0.025)	0.021 (0.007-0.045)	0.037 (0.015-0.069)	0.055 (0.026-0.095)	0.077 (0.035-0.136)
Gambia	0.007 (0.001-0.018)	0.009 (0.003-0.020)	0.016 (0.007-0.029)	0.024 (0.012-0.041)	0.035 (0.015-0.065)
Georgia	0.049 (0.016-0.103)	0.069 (0.033-0.121)	0.074 (0.038-0.126)	0.095 (0.055-0.148)	0.127 (0.073-0.196)
Germany	0.068 (0.037-0.108)	0.094 (0.071-0.121)	0.121 (0.097-0.149)	0.147 (0.119-0.179)	0.172 (0.120-0.226)
Ghana	0.007 (0.002-0.017)	0.009 (0.003-0.018)	0.013 (0.006-0.024)	0.021 (0.011-0.035)	0.033 (0.015-0.062)
Greece	0.063 (0.029-0.112)	0.092 (0.057-0.136)	0.119 (0.080-0.166)	0.151 (0.107-0.201)	0.174 (0.111-0.243)
Greenland	0.065 (0.031-0.112)	0.093 (0.058-0.136)	0.121 (0.083-0.167)	0.152 (0.110-0.201)	0.180 (0.120-0.246)
Grenada	0.024 (0.008-0.052)	0.035 (0.016-0.062)	0.056 (0.031-0.091)	0.084 (0.051-0.127)	0.116 (0.064-0.179)
Guatemala	0.031 (0.010-0.066)	0.043 (0.021-0.076)	0.060 (0.035-0.095)	0.085 (0.054-0.124)	0.110 (0.063-0.169)
Guinea	0.005 (0.001-0.015)	0.008 (0.002-0.017)	0.011 (0.005-0.021)	0.016 (0.008-0.027)	0.022 (0.009-0.041)
Guinea Bissau	0.006 (0.001-0.015)	0.008 (0.003-0.017)	0.014 (0.006-0.027)	0.018 (0.009-0.032)	0.024 (0.010-0.046)
Guyana	0.026 (0.009-0.057)	0.036 (0.017-0.063)	0.046 (0.025-0.076)	0.071 (0.041-0.110)	0.097 (0.051-0.158)
Haiti	0.012 (0.003-0.031)	0.018 (0.007-0.035)	0.025 (0.011-0.045)	0.039 (0.019-0.067)	0.056 (0.024-0.103)
Honduras	0.025 (0.007-0.054)	0.037 (0.017-0.066)	0.053 (0.029-0.086)	0.077 (0.047-0.114)	0.102 (0.057-0.159)
Hungary	0.090 (0.045-0.152)	0.120 (0.080-0.168)	0.143 (0.102-0.191)	0.164 (0.122-0.211)	0.189 (0.130-0.256)
Iceland	0.070 (0.034-0.118)	0.095 (0.058-0.138)	0.116 (0.077-0.158)	0.140 (0.097-0.187)	0.163 (0.102-0.225)
India	0.003 (0.001-0.006)	0.004 (0.002-0.007)	0.007 (0.004-0.010)	0.012 (0.008-0.017)	0.021 (0.012-0.034)
Indonesia	0.002 (0.000-0.006)	0.004 (0.001-0.007)	0.008 (0.004-0.013)	0.016 (0.009-0.024)	0.030 (0.016-0.052)
Iran	0.038 (0.015-0.076)	0.059 (0.037-0.087)	0.080 (0.058-0.106)	0.110 (0.088-0.133)	0.143 (0.102-0.190)
Iraq	0.057 (0.023-0.110)	0.080 (0.045-0.125)	0.094 (0.057-0.140)	0.113 (0.075-0.161)	0.146 (0.088-0.211)
Ireland	0.061 (0.028-0.109)	0.087 (0.052-0.129)	0.118 (0.079-0.163)	0.166 (0.122-0.213)	0.194 (0.132-0.260)
Israel	0.087 (0.042-0.153)	0.109 (0.069-0.158)	0.134 (0.092-0.180)	0.159 (0.116-0.208)	0.182 (0.122-0.252)
Italy	0.065 (0.035-0.109)	0.089 (0.065-0.118)	0.115 (0.091-0.143)	0.148 (0.120-0.179)	0.175 (0.125-0.232)
Jamaica	0.030 (0.011-0.061)	0.041 (0.022-0.066)	0.059 (0.037-0.086)	0.085 (0.057-0.118)	0.112 (0.066-0.168)
Japan	0.006 (0.003-0.011)	0.009 (0.006-0.013)	0.015 (0.011-0.020)	0.023 (0.017-0.031)	0.035 (0.021-0.054)
Jordan	0.060 (0.023-0.119)	0.090 (0.056-0.135)	0.122 (0.084-0.171)	0.149 (0.111-0.196)	0.177 (0.119-0.243)
Kazakhstan	0.052 (0.018-0.105)	0.072 (0.036-0.122)	0.092 (0.051-0.145)	0.119 (0.072-0.175)	0.162 (0.095-0.242)
Kenya	0.004 (0.001-0.012)	0.007 (0.002-0.015)	0.011 (0.005-0.021)	0.016 (0.008-0.029)	0.025 (0.010-0.049)
Kiribati	0.127 (0.062-0.217)	0.149 (0.090-0.219)	0.168 (0.107-0.237)	0.193 (0.131-0.259)	0.201 (0.124-0.288)
Kuwait	0.107 (0.048-0.186)	0.144 (0.099-0.196)	0.170 (0.128-0.215)	0.207 (0.165-0.251)	0.226 (0.165-0.288)
Kyrgyzstan	0.037 (0.011-0.083)	0.047 (0.021-0.086)	0.058 (0.029-0.099)	0.070 (0.038-0.113)	0.095 (0.048-0.154)
Lao PDR	0.002 (0.000-0.005)	0.002 (0.001-0.005)	0.004 (0.002-0.008)	0.008 (0.004-0.015)	0.018 (0.008-0.033)
Latvia	0.069 (0.029-0.132)	0.094 (0.054-0.144)	0.108 (0.067-0.158)	0.131 (0.088-0.182)	0.172 (0.107-0.242)
Lebanon	0.066 (0.029-0.122)	0.096 (0.056-0.146)	0.126 (0.081-0.180)	0.158 (0.107-0.215)	0.179 (0.120-0.244)
Lesotho	0.006 (0.001-0.016)	0.008 (0.003-0.018)	0.013 (0.005-0.026)	0.021 (0.010-0.037)	0.034 (0.015-0.062)
Liberia	0.008 (0.002-0.021)	0.012 (0.004-0.025)	0.012 (0.004-0.025)	0.015 (0.007-0.027)	0.024 (0.010-0.046)
Libya	0.062 (0.020-0.126)	0.106 (0.065-0.157)	0.130 (0.089-0.178)	0.153 (0.112-0.200)	0.174 (0.115-0.236)
Lithuania	0.065 (0.029-0.122)	0.094 (0.059-0.140)	0.114 (0.078-0.161)	0.140 (0.100-0.187)	0.178 (0.118-0.247)
Luxembourg	0.066 (0.030-0.114)	0.093 (0.053-0.141)	0.127 (0.078-0.184)	0.159 (0.112-0.213)	0.185 (0.123-0.254)
Macedonia (TFYR)	0.054 (0.021-0.107)	0.078 (0.043-0.126)	0.099 (0.059-0.150)	0.121 (0.078-0.172)	0.148 (0.091-0.218)
Madagascar	0.005 (0.001-0.015)	0.007 (0.002-0.015)	0.009 (0.004-0.018)	0.013 (0.006-0.022)	0.018 (0.008-0.036)
Malawi	0.003 (0.001-0.011)	0.004 (0.001-0.011)	0.006 (0.002-0.013)	0.009 (0.004-0.016)	0.012 (0.005-0.025)
Malaysia	0.007 (0.002-0.018)	0.014 (0.006-0.026)	0.028 (0.016-0.045)	0.054 (0.035-0.078)	0.083 (0.049-0.127)
Maldives	0.003 (0.001-0.009)	0.006 (0.002-0.013)	0.011 (0.005-0.022)	0.024 (0.011-0.045)	0.047 (0.021-0.084)
Mali	0.006 (0.001-0.017)	0.009 (0.003-0.018)	0.013 (0.006-0.024)	0.019 (0.010-0.033)	0.029 (0.012-0.053)
Malta	0.091 (0.035-0.178)	0.118 (0.072-0.173)	0.140 (0.094-0.192)	0.170 (0.122-0.226)	0.193 (0.126-0.267)
Marshall Islands	0.172 (0.085-0.281)	0.191 (0.118-0.274)	0.213 (0.145-0.284)	0.224 (0.158-0.293)	0.230 (0.145-0.323)
Mauritania	0.008 (0.002-0.021)	0.013 (0.005-0.026)	0.021 (0.009-0.037)	0.030 (0.016-0.051)	0.044 (0.020-0.079)
Mauritius	0.014 (0.004-0.033)	0.021 (0.010-0.039)	0.036 (0.020-0.059)	0.059 (0.035-0.091)	0.087 (0.044-0.143)
Mexico	0.056 (0.023-0.105)	0.085 (0.051-0.127)	0.117 (0.084-0.155)	0.148 (0.117-0.183)	0.174 (0.124-0.228)
Micronesia (Federated States of)	0.127 (0.063-0.219)	0.150 (0.093-0.220)	0.173 (0.116-0.240)	0.194 (0.141-0.254)	0.208 (0.141-0.288)
Moldova	0.039 (0.014-0.083)	0.056 (0.029-0.096)	0.067 (0.038-0.108)	0.076 (0.044-0.117)	0.105 (0.059-0.164)
Mongolia	0.038 (0.011-0.084)	0.052 (0.024-0.093)	0.065 (0.035-0.105)	0.083 (0.051-0.124)	0.116 (0.068-0.174)
Montenegro	0.050 (0.016-0.107)	0.082 (0.041-0.136)	0.109 (0.065-0.163)	0.138 (0.091-0.190)	0.164 (0.104-0.233)
Morocco	0.033 (0.010-0.071)	0.047 (0.023-0.080)	0.067 (0.039-0.103)	0.091 (0.058-0.133)	0.120 (0.070-0.180)
Mozambique	0.003 (0.000-0.010)	0.004 (0.001-0.010)	0.006 (0.002-0.012)	0.009 (0.004-0.017)	0.014 (0.005-0.027)
Myanmar	0.002 (0.000-0.008)	0.003 (0.001-0.007)	0.004 (0.001-0.008)	0.008 (0.004-0.014)	0.018 (0.008-0.035)
Namibia	0.010 (0.002-0.027)	0.015 (0.006-0.030)	0.022 (0.010-0.039)	0.036 (0.020-0.059)	0.057 (0.029-0.098)
Nauru	0.253 (0.161-0.356)	0.258 (0.191-0.329)	0.258 (0.198-0.323)	0.253 (0.187-0.321)	0.252 (0.161-0.348)
Nepal	0.003 (0.001-0.009)	0.005 (0.002-0.010)	0.008 (0.003-0.015)	0.013 (0.006-0.022)	0.020 (0.009-0.040)
Netherlands	0.047 (0.022-0.081)	0.066 (0.043-0.094)	0.092 (0.064-0.121)	0.127 (0.096-0.160)	0.151 (0.102-0.203)
New Zealand	0.087 (0.047-0.137)	0.110 (0.076-0.150)	0.136 (0.101-0.174)	0.164 (0.129-0.200)	0.191 (0.145-0.240)
Nicaragua	0.038 (0.013-0.078)	0.051 (0.025-0.088)	0.060 (0.033-0.096)	0.079 (0.049-0.118)	0.101 (0.055-0.161)
Niger	0.004 (0.001-0.012)	0.005 (0.002-0.012)	0.008 (0.003-0.015)	0.010 (0.005-0.019)	0.013 (0.005-0.027)
Nigeria	0.006 (0.001-0.015)	0.009 (0.004-0.018)	0.014 (0.008-0.023)	0.022 (0.014-0.034)	0.038 (0.020-0.061)
Niue	0.135 (0.061-0.238)	0.173 (0.104-0.259)	0.209 (0.144-0.286)	0.241 (0.180-0.310)	0.265 (0.187-0.348)
North Korea	0.007 (0.002-0.019)	0.011 (0.004-0.022)	0.014 (0.006-0.027)	0.015 (0.006-0.031)	0.019 (0.005-0.045)
Norway	0.053 (0.027-0.093)	0.082 (0.055-0.116)	0.112 (0.081-0.147)	0.148 (0.114-0.186)	0.174 (0.120-0.233)

Occupied Palestinian Territory	0.064 (0.025-0.126)	0.083 (0.046-0.133)	0.099 (0.062-0.146)	0.122 (0.084-0.170)	0.136 (0.080-0.207)
Oman	0.035 (0.011-0.077)	0.065 (0.036-0.101)	0.105 (0.069-0.143)	0.140 (0.100-0.181)	0.173 (0.114-0.235)
Pakistan	0.006 (0.002-0.015)	0.010 (0.004-0.019)	0.016 (0.008-0.029)	0.025 (0.013-0.042)	0.037 (0.017-0.067)
Palau	0.186 (0.096-0.294)	0.210 (0.141-0.287)	0.232 (0.166-0.303)	0.247 (0.183-0.314)	0.255 (0.177-0.336)
Panama	0.041 (0.015-0.084)	0.061 (0.031-0.099)	0.083 (0.050-0.123)	0.112 (0.075-0.154)	0.147 (0.093-0.208)
Papua New Guinea	0.050 (0.013-0.116)	0.062 (0.024-0.119)	0.079 (0.036-0.137)	0.095 (0.049-0.153)	0.117 (0.057-0.192)
Paraguay	0.029 (0.008-0.064)	0.043 (0.020-0.078)	0.068 (0.037-0.110)	0.096 (0.058-0.143)	0.123 (0.072-0.186)
Peru	0.039 (0.014-0.080)	0.057 (0.028-0.094)	0.074 (0.043-0.115)	0.094 (0.062-0.134)	0.124 (0.077-0.180)
Philippines	0.006 (0.001-0.014)	0.009 (0.004-0.016)	0.014 (0.007-0.023)	0.021 (0.012-0.034)	0.034 (0.017-0.061)
Poland	0.076 (0.036-0.131)	0.100 (0.069-0.139)	0.120 (0.088-0.156)	0.149 (0.117-0.183)	0.180 (0.129-0.236)
Portugal	0.037 (0.015-0.072)	0.055 (0.032-0.084)	0.086 (0.060-0.118)	0.128 (0.095-0.166)	0.160 (0.104-0.221)
Puerto Rico	0.051 (0.020-0.098)	0.084 (0.048-0.131)	0.126 (0.082-0.180)	0.158 (0.113-0.212)	0.178 (0.116-0.247)
Qatar	0.086 (0.037-0.153)	0.132 (0.077-0.195)	0.161 (0.111-0.215)	0.198 (0.147-0.253)	0.223 (0.155-0.292)
Romania	0.055 (0.022-0.106)	0.085 (0.050-0.130)	0.107 (0.068-0.150)	0.133 (0.090-0.178)	0.170 (0.108-0.238)
Russian Federation	0.058 (0.029-0.102)	0.080 (0.057-0.108)	0.096 (0.075-0.119)	0.117 (0.091-0.145)	0.156 (0.106-0.216)
Rwanda	0.001 (0.000-0.005)	0.002 (0.000-0.006)	0.003 (0.001-0.008)	0.005 (0.002-0.011)	0.009 (0.003-0.020)
Saint Kitts and Nevis	0.031 (0.011-0.067)	0.048 (0.023-0.085)	0.074 (0.042-0.116)	0.107 (0.067-0.156)	0.135 (0.073-0.211)
Saint Lucia	0.022 (0.007-0.050)	0.033 (0.017-0.058)	0.056 (0.033-0.088)	0.088 (0.055-0.131)	0.123 (0.066-0.198)
Saint Vincent and the Grenadines	0.023 (0.007-0.053)	0.035 (0.016-0.064)	0.056 (0.031-0.092)	0.088 (0.054-0.131)	0.120 (0.068-0.186)
Samoa	0.119 (0.055-0.209)	0.142 (0.085-0.212)	0.176 (0.118-0.241)	0.204 (0.148-0.266)	0.219 (0.147-0.299)
Sao Tome and Principe	0.008 (0.002-0.022)	0.012 (0.005-0.025)	0.018 (0.008-0.034)	0.026 (0.014-0.044)	0.038 (0.018-0.068)
Saudi Arabia	0.058 (0.020-0.118)	0.102 (0.066-0.147)	0.139 (0.105-0.177)	0.167 (0.131-0.207)	0.195 (0.139-0.258)
Senegal	0.009 (0.002-0.022)	0.012 (0.004-0.023)	0.016 (0.007-0.028)	0.022 (0.011-0.037)	0.030 (0.014-0.055)
Serbia	0.052 (0.021-0.099)	0.078 (0.048-0.118)	0.101 (0.068-0.141)	0.124 (0.090-0.163)	0.147 (0.098-0.201)
Seychelles	0.017 (0.005-0.039)	0.030 (0.014-0.055)	0.048 (0.026-0.080)	0.075 (0.044-0.115)	0.104 (0.054-0.167)
Sierra Leone	0.007 (0.001-0.019)	0.010 (0.003-0.020)	0.012 (0.005-0.022)	0.015 (0.008-0.025)	0.022 (0.010-0.040)
Singapore	0.007 (0.002-0.018)	0.014 (0.007-0.025)	0.025 (0.014-0.038)	0.037 (0.023-0.055)	0.055 (0.032-0.087)
Slovakia	0.061 (0.026-0.113)	0.089 (0.056-0.133)	0.117 (0.080-0.158)	0.143 (0.106-0.186)	0.179 (0.122-0.245)
Slovenia	0.052 (0.019-0.103)	0.082 (0.046-0.130)	0.110 (0.070-0.159)	0.143 (0.097-0.194)	0.172 (0.108-0.243)
Solomon Islands	0.038 (0.009-0.097)	0.053 (0.019-0.106)	0.068 (0.030-0.121)	0.080 (0.042-0.128)	0.097 (0.048-0.162)
Somalia	0.007 (0.001-0.021)	0.009 (0.003-0.021)	0.011 (0.004-0.023)	0.014 (0.005-0.027)	0.018 (0.006-0.037)
South Africa	0.026 (0.009-0.054)	0.036 (0.019-0.059)	0.050 (0.031-0.074)	0.070 (0.049-0.095)	0.098 (0.064-0.138)
South Korea	0.001 (0.000-0.003)	0.005 (0.003-0.010)	0.015 (0.008-0.024)	0.025 (0.016-0.036)	0.043 (0.025-0.068)
Spain	0.064 (0.031-0.110)	0.092 (0.065-0.125)	0.124 (0.097-0.154)	0.158 (0.129-0.190)	0.182 (0.129-0.240)
Sri Lanka	0.003 (0.001-0.007)	0.004 (0.001-0.008)	0.007 (0.003-0.012)	0.013 (0.007-0.022)	0.027 (0.011-0.052)
Sudan	0.007 (0.001-0.019)	0.010 (0.004-0.021)	0.015 (0.006-0.029)	0.024 (0.012-0.042)	0.037 (0.016-0.068)
Suriname	0.039 (0.014-0.082)	0.064 (0.033-0.106)	0.080 (0.046-0.124)	0.105 (0.066-0.152)	0.135 (0.080-0.201)
Swaziland	0.008 (0.002-0.021)	0.013 (0.005-0.028)	0.022 (0.010-0.042)	0.036 (0.019-0.060)	0.052 (0.024-0.094)
Sweden	0.066 (0.038-0.103)	0.085 (0.062-0.111)	0.108 (0.084-0.134)	0.138 (0.111-0.167)	0.163 (0.115-0.216)
Switzerland	0.056 (0.025-0.102)	0.082 (0.052-0.122)	0.118 (0.084-0.157)	0.143 (0.108-0.179)	0.165 (0.114-0.219)
Syrian Arab Republic	0.041 (0.015-0.085)	0.066 (0.035-0.107)	0.084 (0.051-0.126)	0.109 (0.072-0.152)	0.129 (0.078-0.190)
Taiwan	0.006 (0.002-0.014)	0.014 (0.007-0.026)	0.031 (0.018-0.048)	0.054 (0.032-0.080)	0.077 (0.039-0.125)
Tajikistan	0.036 (0.011-0.080)	0.045 (0.019-0.085)	0.050 (0.023-0.088)	0.053 (0.025-0.092)	0.076 (0.035-0.136)
Tanzania	0.005 (0.001-0.013)	0.006 (0.002-0.014)	0.009 (0.004-0.017)	0.014 (0.007-0.024)	0.022 (0.010-0.041)
Thailand	0.005 (0.001-0.011)	0.007 (0.003-0.014)	0.014 (0.008-0.023)	0.027 (0.017-0.042)	0.049 (0.026-0.084)
Timor-Leste	0.001 (0.000-0.004)	0.002 (0.001-0.004)	0.003 (0.001-0.006)	0.005 (0.002-0.010)	0.009 (0.003-0.020)
Togo	0.005 (0.001-0.014)	0.007 (0.002-0.015)	0.011 (0.005-0.020)	0.015 (0.008-0.026)	0.022 (0.009-0.041)
Tokelau	0.115 (0.034-0.244)	0.144 (0.067-0.246)	0.178 (0.108-0.262)	0.211 (0.142-0.286)	0.233 (0.144-0.330)
Tonga	0.135 (0.062-0.236)	0.162 (0.097-0.239)	0.191 (0.125-0.263)	0.216 (0.155-0.281)	0.230 (0.156-0.312)
Trinidad and Tobago	0.029 (0.006-0.075)	0.046 (0.018-0.092)	0.062 (0.033-0.105)	0.100 (0.057-0.156)	0.144 (0.067-0.243)
Tunisia	0.038 (0.014-0.079)	0.057 (0.031-0.091)	0.080 (0.050-0.116)	0.108 (0.074-0.144)	0.136 (0.088-0.193)
Turkey	0.049 (0.021-0.094)	0.075 (0.048-0.110)	0.111 (0.082-0.145)	0.146 (0.117-0.178)	0.176 (0.130-0.226)
Turkmenistan	0.044 (0.015-0.095)	0.056 (0.026-0.097)	0.073 (0.039-0.118)	0.091 (0.052-0.139)	0.132 (0.076-0.199)
Tuvalu	0.124 (0.057-0.219)	0.152 (0.090-0.228)	0.188 (0.125-0.260)	0.211 (0.148-0.278)	0.224 (0.145-0.310)
Uganda	0.002 (0.000-0.008)	0.003 (0.001-0.008)	0.004 (0.001-0.009)	0.007 (0.003-0.014)	0.012 (0.005-0.026)
Ukraine	0.060 (0.024-0.118)	0.082 (0.046-0.132)	0.098 (0.059-0.148)	0.109 (0.068-0.161)	0.140 (0.082-0.210)
United Arab Emirates	0.109 (0.039-0.208)	0.138 (0.082-0.204)	0.163 (0.114-0.218)	0.195 (0.145-0.247)	0.204 (0.139-0.274)
United Kingdom	0.080 (0.044-0.124)	0.097 (0.073-0.124)	0.123 (0.102-0.145)	0.158 (0.136-0.181)	0.189 (0.149-0.230)
United States of America	0.092 (0.057-0.137)	0.117 (0.088-0.150)	0.150 (0.118-0.184)	0.183 (0.153-0.217)	0.204 (0.156-0.253)
Uruguay	0.085 (0.033-0.159)	0.102 (0.057-0.158)	0.122 (0.079-0.174)	0.149 (0.104-0.201)	0.178 (0.116-0.249)
Uzbekistan	0.039 (0.012-0.088)	0.051 (0.021-0.095)	0.062 (0.030-0.107)	0.073 (0.040-0.118)	0.103 (0.052-0.170)
Vanuatu	0.058 (0.017-0.131)	0.072 (0.030-0.132)	0.089 (0.043-0.145)	0.109 (0.061-0.163)	0.130 (0.069-0.200)
Venezuela	0.063 (0.026-0.117)	0.091 (0.052-0.138)	0.114 (0.075-0.161)	0.136 (0.098-0.179)	0.163 (0.108-0.226)
Viet Nam	0.001 (0.000-0.004)	0.002 (0.001-0.003)	0.003 (0.001-0.005)	0.006 (0.003-0.011)	0.016 (0.007-0.030)
Yemen	0.020 (0.005-0.048)	0.028 (0.012-0.053)	0.041 (0.021-0.069)	0.058 (0.032-0.090)	0.075 (0.036-0.125)
Zambia	0.009 (0.002-0.023)	0.013 (0.005-0.026)	0.016 (0.007-0.029)	0.020 (0.010-0.034)	0.030 (0.013-0.056)
Zimbabwe	0.008 (0.002-0.021)	0.012 (0.005-0.024)	0.018 (0.009-0.031)	0.025 (0.013-0.041)	0.030 (0.013-0.056)

Women, 30 ≤ BMI < 35 kg/m²

Country	Year				
	1975	1985	1995	2005	2014
Afghanistan	0.008 (0.002-0.019)	0.012 (0.005-0.024)	0.017 (0.008-0.031)	0.024 (0.012-0.043)	0.041 (0.020-0.073)
Albania	0.093 (0.036-0.177)	0.099 (0.053-0.159)	0.098 (0.060-0.146)	0.111 (0.073-0.157)	0.122 (0.071-0.183)
Algeria	0.100 (0.044-0.182)	0.129 (0.079-0.190)	0.152 (0.107-0.203)	0.178 (0.131-0.227)	0.195 (0.134-0.263)
American Samoa	0.229 (0.146-0.321)	0.240 (0.174-0.310)	0.247 (0.186-0.313)	0.250 (0.187-0.317)	0.249 (0.169-0.340)
Andorra	0.126 (0.064-0.210)	0.140 (0.089-0.204)	0.149 (0.101-0.205)	0.166 (0.116-0.222)	0.170 (0.107-0.242)
Angola	0.019 (0.004-0.050)	0.027 (0.009-0.055)	0.038 (0.017-0.068)	0.058 (0.029-0.097)	0.088 (0.042-0.150)
Antigua and Barbuda	0.092 (0.037-0.173)	0.119 (0.066-0.185)	0.147 (0.094-0.212)	0.165 (0.109-0.231)	0.178 (0.102-0.270)
Argentina	0.102 (0.047-0.180)	0.118 (0.071-0.176)	0.137 (0.094-0.186)	0.155 (0.115-0.201)	0.176 (0.117-0.240)
Armenia	0.075 (0.023-0.160)	0.091 (0.044-0.155)	0.098 (0.058-0.147)	0.118 (0.078-0.166)	0.138 (0.082-0.209)
Australia	0.075 (0.044-0.115)	0.093 (0.068-0.120)	0.114 (0.089-0.141)	0.139 (0.112-0.169)	0.159 (0.117-0.207)
Austria	0.057 (0.028-0.097)	0.069 (0.044-0.101)	0.084 (0.057-0.115)	0.105 (0.074-0.141)	0.122 (0.076-0.179)
Azerbaijan	0.087 (0.031-0.175)	0.105 (0.053-0.171)	0.107 (0.064-0.158)	0.122 (0.078-0.173)	0.154 (0.092-0.229)
Bahamas	0.131 (0.062-0.223)	0.154 (0.093-0.225)	0.172 (0.117-0.236)	0.188 (0.132-0.250)	0.193 (0.125-0.271)
Bahrain	0.190 (0.099-0.296)	0.195 (0.131-0.265)	0.212 (0.157-0.268)	0.226 (0.173-0.280)	0.232 (0.162-0.306)
Bangladesh	0.006 (0.001-0.014)	0.009 (0.004-0.017)	0.015 (0.008-0.024)	0.025 (0.015-0.038)	0.041 (0.022-0.067)
Barbados	0.085 (0.035-0.156)	0.109 (0.064-0.167)	0.131 (0.087-0.183)	0.156 (0.109-0.212)	0.171 (0.104-0.252)
Belarus	0.110 (0.045-0.206)	0.125 (0.071-0.194)	0.132 (0.084-0.190)	0.140 (0.092-0.197)	0.150 (0.089-0.223)
Belgium	0.085 (0.043-0.140)	0.099 (0.069-0.134)	0.117 (0.090-0.147)	0.135 (0.105-0.171)	0.147 (0.101-0.202)
Belize	0.069 (0.025-0.139)	0.090 (0.048-0.143)	0.116 (0.074-0.167)	0.142 (0.097-0.193)	0.156 (0.096-0.227)
Benin	0.014 (0.004-0.034)	0.023 (0.011-0.042)	0.038 (0.022-0.059)	0.061 (0.041-0.086)	0.086 (0.054-0.126)
Bermuda	0.172 (0.081-0.282)	0.186 (0.110-0.269)	0.198 (0.130-0.269)	0.210 (0.148-0.277)	0.219 (0.144-0.306)
Bhutan	0.007 (0.001-0.018)	0.012 (0.005-0.025)	0.025 (0.012-0.044)	0.044 (0.025-0.070)	0.066 (0.036-0.109)
Bolivia	0.063 (0.021-0.128)	0.081 (0.041-0.134)	0.105 (0.065-0.155)	0.133 (0.091-0.181)	0.157 (0.100-0.227)
Bosnia and Herzegovina	0.056 (0.014-0.141)	0.067 (0.027-0.128)	0.082 (0.044-0.131)	0.119 (0.076-0.171)	0.115 (0.067-0.169)
Botswana	0.033 (0.008-0.083)	0.065 (0.029-0.119)	0.111 (0.065-0.170)	0.144 (0.093-0.202)	0.172 (0.107-0.248)
Brazil	0.062 (0.026-0.115)	0.089 (0.057-0.129)	0.111 (0.084-0.142)	0.131 (0.105-0.160)	0.151 (0.108-0.200)
Brunei Darussalam	0.067 (0.017-0.155)	0.082 (0.034-0.151)	0.095 (0.053-0.152)	0.113 (0.069-0.170)	0.123 (0.066-0.199)
Bulgaria	0.116 (0.052-0.207)	0.125 (0.073-0.189)	0.127 (0.082-0.180)	0.136 (0.092-0.189)	0.144 (0.087-0.212)
Burkina Faso	0.007 (0.001-0.018)	0.011 (0.004-0.022)	0.021 (0.011-0.033)	0.039 (0.024-0.057)	0.060 (0.034-0.092)
Burundi	0.004 (0.000-0.014)	0.008 (0.002-0.019)	0.014 (0.005-0.028)	0.024 (0.012-0.043)	0.042 (0.020-0.073)
Cabo Verde	0.011 (0.003-0.030)	0.023 (0.010-0.043)	0.044 (0.024-0.072)	0.081 (0.052-0.118)	0.111 (0.066-0.167)
Cambodia	0.002 (0.000-0.007)	0.005 (0.001-0.010)	0.009 (0.004-0.017)	0.018 (0.010-0.029)	0.034 (0.017-0.058)
Cameroon	0.019 (0.006-0.045)	0.035 (0.017-0.060)	0.049 (0.031-0.072)	0.073 (0.052-0.100)	0.101 (0.064-0.146)
Canada	0.077 (0.043-0.120)	0.103 (0.073-0.138)	0.128 (0.096-0.163)	0.152 (0.118-0.190)	0.168 (0.118-0.221)
Central African Republic	0.013 (0.003-0.034)	0.018 (0.006-0.038)	0.026 (0.012-0.047)	0.038 (0.020-0.065)	0.057 (0.026-0.100)
Chad	0.011 (0.002-0.027)	0.016 (0.006-0.031)	0.027 (0.014-0.044)	0.047 (0.028-0.070)	0.071 (0.039-0.112)
Chile	0.103 (0.044-0.186)	0.128 (0.076-0.191)	0.155 (0.111-0.205)	0.178 (0.136-0.222)	0.194 (0.137-0.257)
China	0.008 (0.002-0.019)	0.013 (0.008-0.020)	0.024 (0.018-0.031)	0.045 (0.034-0.058)	0.069 (0.042-0.102)
China (Hong Kong SAR)	0.056 (0.015-0.130)	0.065 (0.029-0.115)	0.073 (0.040-0.115)	0.073 (0.039-0.116)	0.073 (0.030-0.136)
Colombia	0.079 (0.030-0.150)	0.103 (0.060-0.157)	0.127 (0.089-0.169)	0.149 (0.115-0.186)	0.169 (0.119-0.225)
Comoros	0.019 (0.005-0.045)	0.026 (0.011-0.047)	0.038 (0.021-0.061)	0.056 (0.035-0.084)	0.080 (0.047-0.123)
Congo	0.023 (0.007-0.053)	0.040 (0.019-0.069)	0.057 (0.033-0.088)	0.075 (0.047-0.110)	0.097 (0.055-0.152)
Cook Islands	0.196 (0.105-0.304)	0.233 (0.161-0.313)	0.254 (0.183-0.329)	0.262 (0.193-0.333)	0.263 (0.181-0.351)
Costa Rica	0.073 (0.026-0.145)	0.096 (0.051-0.152)	0.125 (0.082-0.176)	0.155 (0.112-0.203)	0.178 (0.121-0.244)
Cote d'Ivoire	0.027 (0.008-0.061)	0.038 (0.018-0.066)	0.051 (0.031-0.077)	0.073 (0.049-0.102)	0.096 (0.060-0.141)
Croatia	0.096 (0.041-0.180)	0.114 (0.067-0.174)	0.121 (0.079-0.172)	0.131 (0.089-0.182)	0.135 (0.080-0.202)
Cuba	0.090 (0.040-0.159)	0.122 (0.074-0.180)	0.138 (0.093-0.188)	0.155 (0.112-0.203)	0.173 (0.114-0.239)
Cyprus	0.057 (0.022-0.113)	0.097 (0.054-0.150)	0.126 (0.082-0.179)	0.146 (0.099-0.201)	0.156 (0.094-0.227)
Czech Republic	0.142 (0.073-0.233)	0.151 (0.100-0.211)	0.150 (0.107-0.199)	0.150 (0.109-0.199)	0.149 (0.092-0.213)
Denmark	0.064 (0.031-0.107)	0.080 (0.051-0.115)	0.093 (0.063-0.127)	0.104 (0.071-0.140)	0.118 (0.073-0.169)
Djibouti	0.033 (0.008-0.077)	0.038 (0.013-0.077)	0.056 (0.028-0.094)	0.072 (0.039-0.114)	0.095 (0.048-0.158)
Dominica	0.079 (0.033-0.148)	0.102 (0.058-0.159)	0.132 (0.086-0.184)	0.152 (0.105-0.205)	0.170 (0.108-0.239)
Dominican Republic	0.058 (0.023-0.112)	0.087 (0.051-0.133)	0.111 (0.075-0.152)	0.144 (0.104-0.188)	0.166 (0.109-0.230)
DR Congo	0.014 (0.003-0.036)	0.019 (0.007-0.039)	0.027 (0.013-0.047)	0.036 (0.019-0.059)	0.054 (0.027-0.094)
Ecuador	0.065 (0.021-0.135)	0.092 (0.046-0.152)	0.119 (0.073-0.174)	0.142 (0.096-0.195)	0.164 (0.105-0.234)
Egypt	0.116 (0.050-0.202)	0.151 (0.100-0.211)	0.179 (0.137-0.225)	0.203 (0.164-0.247)	0.218 (0.159-0.278)
El Salvador	0.080 (0.031-0.158)	0.099 (0.055-0.158)	0.126 (0.084-0.177)	0.157 (0.115-0.207)	0.177 (0.117-0.246)
Equatorial Guinea	0.013 (0.003-0.036)	0.022 (0.008-0.044)	0.036 (0.017-0.064)	0.096 (0.051-0.151)	0.127 (0.059-0.212)
Eritrea	0.008 (0.002-0.021)	0.012 (0.005-0.023)	0.021 (0.011-0.034)	0.037 (0.021-0.055)	0.055 (0.029-0.089)
Estonia	0.137 (0.066-0.229)	0.141 (0.092-0.203)	0.136 (0.098-0.179)	0.135 (0.106-0.169)	0.132 (0.091-0.183)
Ethiopia	0.007 (0.001-0.019)	0.011 (0.004-0.021)	0.017 (0.008-0.029)	0.028 (0.016-0.044)	0.049 (0.025-0.080)
Fiji	0.127 (0.047-0.244)	0.153 (0.078-0.245)	0.176 (0.109-0.251)	0.198 (0.138-0.266)	0.211 (0.142-0.289)
Finland	0.054 (0.027-0.090)	0.075 (0.052-0.103)	0.099 (0.075-0.126)	0.122 (0.095-0.151)	0.135 (0.094-0.180)
France	0.077 (0.036-0.135)	0.090 (0.057-0.132)	0.106 (0.073-0.146)	0.125 (0.089-0.165)	0.140 (0.089-0.197)
French Polynesia	0.226 (0.125-0.343)	0.243 (0.156-0.337)	0.251 (0.176-0.334)	0.253 (0.184-0.326)	0.251 (0.171-0.335)
Gabon	0.041 (0.010-0.098)	0.068 (0.030-0.118)	0.090 (0.050-0.138)	0.117 (0.073-0.169)	0.133 (0.077-0.199)
Gambia	0.015 (0.004-0.036)	0.025 (0.011-0.046)	0.041 (0.023-0.066)	0.067 (0.043-0.096)	0.091 (0.054-0.138)
Georgia	0.079 (0.027-0.164)	0.104 (0.052-0.174)	0.101 (0.057-0.155)	0.115 (0.072-0.166)	0.134 (0.079-0.200)

Germany	0.068 (0.037-0.110)	0.084 (0.062-0.109)	0.102 (0.080-0.125)	0.123 (0.097-0.151)	0.138 (0.095-0.188)
Ghana	0.024 (0.007-0.053)	0.033 (0.017-0.056)	0.052 (0.033-0.077)	0.081 (0.056-0.110)	0.112 (0.071-0.160)
Greece	0.096 (0.045-0.166)	0.116 (0.072-0.169)	0.131 (0.089-0.179)	0.148 (0.105-0.197)	0.155 (0.098-0.222)
Greenland	0.072 (0.035-0.126)	0.093 (0.058-0.136)	0.108 (0.074-0.148)	0.128 (0.092-0.171)	0.144 (0.094-0.204)
Grenada	0.064 (0.024-0.126)	0.090 (0.049-0.143)	0.121 (0.078-0.172)	0.150 (0.102-0.203)	0.168 (0.104-0.245)
Guatemala	0.069 (0.025-0.139)	0.090 (0.049-0.145)	0.112 (0.075-0.158)	0.141 (0.101-0.186)	0.163 (0.105-0.228)
Guinea	0.010 (0.002-0.027)	0.019 (0.008-0.036)	0.031 (0.016-0.051)	0.049 (0.030-0.071)	0.069 (0.041-0.107)
Guinea Bissau	0.012 (0.003-0.031)	0.021 (0.009-0.040)	0.037 (0.019-0.062)	0.054 (0.033-0.081)	0.073 (0.042-0.115)
Guyana	0.070 (0.027-0.135)	0.092 (0.050-0.147)	0.116 (0.073-0.169)	0.148 (0.097-0.203)	0.166 (0.102-0.240)
Haiti	0.035 (0.011-0.076)	0.050 (0.025-0.087)	0.068 (0.041-0.104)	0.091 (0.059-0.131)	0.114 (0.064-0.175)
Honduras	0.055 (0.017-0.114)	0.078 (0.039-0.127)	0.103 (0.065-0.148)	0.131 (0.093-0.174)	0.157 (0.104-0.219)
Hungary	0.125 (0.062-0.209)	0.133 (0.086-0.188)	0.128 (0.088-0.175)	0.133 (0.092-0.181)	0.137 (0.081-0.202)
Iceland	0.066 (0.031-0.116)	0.091 (0.057-0.135)	0.105 (0.071-0.145)	0.122 (0.083-0.166)	0.134 (0.083-0.196)
India	0.005 (0.002-0.012)	0.010 (0.005-0.016)	0.017 (0.011-0.023)	0.027 (0.020-0.036)	0.042 (0.026-0.061)
Indonesia	0.006 (0.001-0.014)	0.012 (0.005-0.022)	0.024 (0.015-0.037)	0.042 (0.029-0.060)	0.064 (0.039-0.099)
Iran	0.112 (0.051-0.196)	0.136 (0.091-0.187)	0.157 (0.125-0.194)	0.184 (0.157-0.212)	0.204 (0.157-0.256)
Iraq	0.126 (0.058-0.216)	0.154 (0.097-0.219)	0.167 (0.116-0.222)	0.192 (0.140-0.247)	0.212 (0.144-0.288)
Ireland	0.044 (0.019-0.080)	0.066 (0.039-0.100)	0.095 (0.064-0.131)	0.128 (0.093-0.171)	0.154 (0.102-0.214)
Israel	0.115 (0.055-0.195)	0.135 (0.086-0.192)	0.154 (0.109-0.205)	0.165 (0.121-0.216)	0.175 (0.114-0.243)
Italy	0.086 (0.046-0.144)	0.103 (0.075-0.137)	0.119 (0.094-0.147)	0.136 (0.107-0.167)	0.147 (0.100-0.199)
Jamaica	0.095 (0.042-0.170)	0.112 (0.070-0.164)	0.139 (0.100-0.184)	0.162 (0.121-0.207)	0.178 (0.119-0.246)
Japan	0.015 (0.008-0.024)	0.017 (0.012-0.023)	0.020 (0.015-0.026)	0.024 (0.017-0.031)	0.026 (0.015-0.041)
Jordan	0.121 (0.053-0.213)	0.168 (0.116-0.229)	0.192 (0.147-0.242)	0.211 (0.170-0.255)	0.222 (0.161-0.285)
Kazakhstan	0.079 (0.028-0.161)	0.098 (0.049-0.160)	0.112 (0.067-0.166)	0.128 (0.082-0.181)	0.142 (0.081-0.216)
Kenya	0.010 (0.002-0.026)	0.019 (0.008-0.035)	0.031 (0.017-0.050)	0.051 (0.031-0.077)	0.079 (0.044-0.124)
Kiribati	0.179 (0.093-0.284)	0.196 (0.124-0.273)	0.216 (0.147-0.290)	0.236 (0.166-0.309)	0.241 (0.155-0.333)
Kuwait	0.225 (0.120-0.341)	0.221 (0.156-0.293)	0.231 (0.181-0.286)	0.240 (0.192-0.292)	0.245 (0.181-0.317)
Kyrgyzstan	0.050 (0.015-0.113)	0.063 (0.029-0.114)	0.076 (0.044-0.118)	0.089 (0.056-0.129)	0.109 (0.063-0.166)
Lao PDR	0.004 (0.001-0.012)	0.007 (0.002-0.014)	0.011 (0.006-0.021)	0.023 (0.013-0.038)	0.041 (0.022-0.068)
Latvia	0.141 (0.063-0.245)	0.154 (0.093-0.226)	0.148 (0.097-0.205)	0.150 (0.102-0.204)	0.148 (0.088-0.220)
Lebanon	0.128 (0.063-0.216)	0.155 (0.099-0.220)	0.176 (0.121-0.233)	0.197 (0.140-0.258)	0.212 (0.144-0.281)
Lesotho	0.036 (0.009-0.091)	0.056 (0.025-0.102)	0.086 (0.050-0.133)	0.121 (0.079-0.171)	0.152 (0.095-0.223)
Liberia	0.020 (0.005-0.046)	0.030 (0.014-0.056)	0.030 (0.013-0.055)	0.048 (0.028-0.072)	0.074 (0.043-0.114)
Libya	0.172 (0.078-0.289)	0.196 (0.129-0.268)	0.203 (0.150-0.261)	0.214 (0.164-0.269)	0.220 (0.154-0.291)
Lithuania	0.145 (0.071-0.244)	0.161 (0.105-0.226)	0.159 (0.113-0.212)	0.159 (0.115-0.211)	0.163 (0.102-0.235)
Luxembourg	0.059 (0.026-0.107)	0.071 (0.039-0.111)	0.089 (0.052-0.134)	0.115 (0.076-0.159)	0.131 (0.079-0.192)
Macedonia (TFYR)	0.114 (0.050-0.202)	0.122 (0.072-0.187)	0.127 (0.081-0.179)	0.126 (0.082-0.176)	0.128 (0.074-0.193)
Madagascar	0.011 (0.002-0.028)	0.015 (0.006-0.029)	0.023 (0.012-0.038)	0.037 (0.021-0.056)	0.058 (0.030-0.095)
Malawi	0.008 (0.002-0.022)	0.014 (0.005-0.028)	0.022 (0.012-0.037)	0.039 (0.024-0.058)	0.063 (0.035-0.100)
Malaysia	0.017 (0.005-0.039)	0.032 (0.016-0.057)	0.055 (0.034-0.083)	0.084 (0.059-0.114)	0.111 (0.071-0.162)
Maldives	0.008 (0.002-0.021)	0.018 (0.007-0.035)	0.034 (0.017-0.058)	0.060 (0.033-0.095)	0.082 (0.045-0.130)
Mali	0.011 (0.003-0.029)	0.019 (0.008-0.036)	0.031 (0.017-0.050)	0.052 (0.033-0.076)	0.074 (0.043-0.115)
Malta	0.097 (0.035-0.191)	0.125 (0.075-0.189)	0.143 (0.097-0.201)	0.163 (0.113-0.219)	0.173 (0.108-0.248)
Marshall Islands	0.196 (0.099-0.309)	0.218 (0.141-0.304)	0.240 (0.171-0.313)	0.246 (0.182-0.319)	0.249 (0.164-0.337)
Mauritania	0.016 (0.004-0.040)	0.027 (0.012-0.052)	0.045 (0.024-0.074)	0.067 (0.041-0.102)	0.092 (0.052-0.144)
Mauritius	0.033 (0.011-0.069)	0.050 (0.026-0.083)	0.077 (0.049-0.114)	0.112 (0.074-0.159)	0.146 (0.087-0.217)
Mexico	0.107 (0.046-0.187)	0.135 (0.086-0.193)	0.159 (0.121-0.200)	0.181 (0.148-0.218)	0.196 (0.144-0.256)
Micronesia (Federated States of)	0.173 (0.086-0.277)	0.196 (0.126-0.278)	0.214 (0.152-0.283)	0.224 (0.168-0.286)	0.228 (0.157-0.303)
Moldova	0.101 (0.040-0.195)	0.115 (0.064-0.178)	0.114 (0.072-0.165)	0.113 (0.073-0.160)	0.121 (0.070-0.181)
Mongolia	0.053 (0.015-0.124)	0.072 (0.034-0.128)	0.087 (0.051-0.132)	0.103 (0.067-0.146)	0.130 (0.079-0.190)
Montenegro	0.104 (0.038-0.200)	0.124 (0.067-0.196)	0.125 (0.078-0.182)	0.126 (0.082-0.178)	0.129 (0.074-0.196)
Morocco	0.079 (0.031-0.153)	0.109 (0.064-0.165)	0.137 (0.094-0.186)	0.164 (0.119-0.211)	0.187 (0.124-0.255)
Mozambique	0.007 (0.001-0.021)	0.012 (0.004-0.026)	0.022 (0.011-0.037)	0.040 (0.024-0.061)	0.063 (0.035-0.101)
Myanmar	0.003 (0.000-0.011)	0.005 (0.002-0.013)	0.010 (0.005-0.019)	0.022 (0.012-0.036)	0.042 (0.023-0.070)
Namibia	0.055 (0.016-0.124)	0.070 (0.033-0.122)	0.091 (0.055-0.136)	0.121 (0.084-0.165)	0.149 (0.095-0.210)
Nauru	0.254 (0.168-0.351)	0.263 (0.199-0.332)	0.261 (0.202-0.324)	0.252 (0.189-0.321)	0.249 (0.158-0.348)
Nepal	0.005 (0.001-0.013)	0.008 (0.003-0.017)	0.015 (0.008-0.026)	0.028 (0.016-0.043)	0.044 (0.024-0.071)
Netherlands	0.056 (0.027-0.098)	0.069 (0.044-0.100)	0.088 (0.063-0.117)	0.113 (0.085-0.143)	0.132 (0.089-0.179)
New Zealand	0.075 (0.039-0.122)	0.094 (0.064-0.130)	0.118 (0.088-0.152)	0.150 (0.119-0.185)	0.167 (0.126-0.214)
Nicaragua	0.079 (0.030-0.149)	0.095 (0.053-0.148)	0.111 (0.073-0.156)	0.139 (0.102-0.185)	0.161 (0.106-0.228)
Niger	0.009 (0.002-0.024)	0.013 (0.005-0.026)	0.021 (0.011-0.036)	0.034 (0.020-0.052)	0.051 (0.028-0.083)
Nigeria	0.018 (0.005-0.042)	0.027 (0.013-0.047)	0.041 (0.026-0.060)	0.066 (0.047-0.088)	0.098 (0.065-0.138)
Niue	0.183 (0.091-0.300)	0.223 (0.143-0.315)	0.244 (0.173-0.320)	0.254 (0.190-0.322)	0.259 (0.181-0.344)
North Korea	0.023 (0.006-0.056)	0.029 (0.012-0.055)	0.030 (0.014-0.054)	0.029 (0.012-0.055)	0.033 (0.009-0.073)
Norway	0.062 (0.031-0.107)	0.088 (0.059-0.123)	0.112 (0.082-0.148)	0.135 (0.101-0.171)	0.145 (0.097-0.200)
Occupied Palestinian Territory	0.120 (0.054-0.211)	0.140 (0.086-0.208)	0.159 (0.113-0.213)	0.185 (0.139-0.237)	0.199 (0.133-0.274)
Oman	0.097 (0.038-0.182)	0.138 (0.084-0.199)	0.175 (0.126-0.225)	0.197 (0.149-0.246)	0.212 (0.147-0.283)
Pakistan	0.013 (0.004-0.028)	0.021 (0.010-0.038)	0.032 (0.019-0.052)	0.045 (0.028-0.069)	0.061 (0.035-0.097)
Palau	0.251 (0.142-0.371)	0.254 (0.175-0.337)	0.261 (0.190-0.339)	0.264 (0.199-0.336)	0.265 (0.186-0.350)
Panama	0.087 (0.035-0.164)	0.111 (0.062-0.173)	0.133 (0.089-0.185)	0.162 (0.118-0.212)	0.188 (0.127-0.255)
Papua New Guinea	0.069 (0.019-0.154)	0.087 (0.036-0.156)	0.111 (0.061-0.174)	0.132 (0.081-0.193)	0.155 (0.088-0.237)

Paraguay	0.044 (0.014-0.092)	0.066 (0.033-0.111)	0.092 (0.054-0.139)	0.114 (0.071-0.164)	0.139 (0.083-0.207)
Peru	0.082 (0.031-0.157)	0.105 (0.060-0.161)	0.124 (0.085-0.168)	0.153 (0.119-0.189)	0.184 (0.137-0.238)
Philippines	0.010 (0.003-0.022)	0.018 (0.010-0.029)	0.027 (0.018-0.040)	0.039 (0.026-0.057)	0.055 (0.030-0.090)
Poland	0.128 (0.066-0.213)	0.134 (0.090-0.186)	0.138 (0.102-0.180)	0.148 (0.113-0.188)	0.152 (0.101-0.210)
Portugal	0.063 (0.025-0.119)	0.081 (0.047-0.124)	0.105 (0.071-0.146)	0.125 (0.090-0.168)	0.140 (0.087-0.199)
Puerto Rico	0.119 (0.054-0.203)	0.153 (0.097-0.221)	0.184 (0.130-0.246)	0.200 (0.149-0.256)	0.204 (0.141-0.275)
Qatar	0.175 (0.089-0.280)	0.230 (0.155-0.313)	0.233 (0.175-0.296)	0.248 (0.192-0.305)	0.251 (0.178-0.329)
Romania	0.109 (0.047-0.197)	0.124 (0.074-0.186)	0.127 (0.083-0.177)	0.131 (0.088-0.182)	0.136 (0.081-0.200)
Russian Federation	0.159 (0.090-0.246)	0.166 (0.126-0.215)	0.162 (0.135-0.191)	0.160 (0.127-0.195)	0.161 (0.106-0.223)
Rwanda	0.005 (0.001-0.015)	0.009 (0.002-0.021)	0.015 (0.006-0.029)	0.030 (0.016-0.050)	0.053 (0.028-0.087)
Saint Kitts and Nevis	0.079 (0.031-0.149)	0.113 (0.064-0.176)	0.140 (0.091-0.198)	0.160 (0.108-0.220)	0.174 (0.104-0.257)
Saint Lucia	0.063 (0.024-0.125)	0.085 (0.048-0.132)	0.115 (0.076-0.163)	0.138 (0.093-0.192)	0.159 (0.094-0.240)
Saint Vincent and the Grenadines	0.065 (0.024-0.129)	0.090 (0.048-0.144)	0.120 (0.076-0.171)	0.146 (0.099-0.201)	0.161 (0.101-0.234)
Samoa	0.187 (0.098-0.295)	0.208 (0.136-0.289)	0.225 (0.162-0.296)	0.236 (0.177-0.300)	0.236 (0.165-0.313)
Sao Tome and Principe	0.027 (0.007-0.061)	0.037 (0.016-0.066)	0.057 (0.032-0.091)	0.079 (0.050-0.115)	0.103 (0.062-0.153)
Saudi Arabia	0.165 (0.081-0.268)	0.196 (0.139-0.257)	0.209 (0.170-0.253)	0.221 (0.180-0.266)	0.233 (0.171-0.299)
Senegal	0.021 (0.006-0.048)	0.031 (0.015-0.053)	0.044 (0.025-0.068)	0.064 (0.041-0.093)	0.087 (0.052-0.133)
Serbia	0.099 (0.044-0.178)	0.110 (0.067-0.164)	0.115 (0.079-0.158)	0.116 (0.083-0.154)	0.122 (0.077-0.174)
Seychelles	0.052 (0.019-0.107)	0.082 (0.045-0.133)	0.113 (0.071-0.163)	0.147 (0.100-0.203)	0.176 (0.110-0.254)
Sierra Leone	0.018 (0.005-0.043)	0.027 (0.012-0.050)	0.037 (0.021-0.060)	0.054 (0.035-0.079)	0.082 (0.051-0.122)
Singapore	0.022 (0.007-0.048)	0.034 (0.018-0.056)	0.044 (0.028-0.066)	0.048 (0.031-0.070)	0.059 (0.033-0.091)
Slovakia	0.094 (0.040-0.173)	0.111 (0.067-0.165)	0.121 (0.081-0.163)	0.130 (0.092-0.171)	0.141 (0.090-0.202)
Slovenia	0.102 (0.043-0.192)	0.116 (0.068-0.180)	0.127 (0.083-0.181)	0.135 (0.092-0.188)	0.139 (0.084-0.205)
Solomon Islands	0.067 (0.017-0.152)	0.094 (0.039-0.168)	0.120 (0.067-0.186)	0.140 (0.089-0.199)	0.160 (0.095-0.237)
Somalia	0.011 (0.002-0.028)	0.016 (0.006-0.032)	0.024 (0.011-0.042)	0.035 (0.017-0.059)	0.054 (0.024-0.095)
South Africa	0.112 (0.047-0.206)	0.131 (0.079-0.196)	0.148 (0.107-0.195)	0.171 (0.136-0.209)	0.188 (0.141-0.241)
South Korea	0.003 (0.001-0.009)	0.012 (0.006-0.021)	0.028 (0.017-0.041)	0.035 (0.024-0.049)	0.053 (0.031-0.081)
Spain	0.089 (0.045-0.149)	0.107 (0.074-0.144)	0.127 (0.099-0.157)	0.146 (0.117-0.178)	0.159 (0.110-0.214)
Sri Lanka	0.008 (0.002-0.021)	0.014 (0.006-0.027)	0.025 (0.014-0.041)	0.043 (0.026-0.065)	0.068 (0.036-0.114)
Sudan	0.016 (0.004-0.039)	0.024 (0.010-0.046)	0.039 (0.021-0.064)	0.061 (0.035-0.094)	0.086 (0.047-0.138)
Suriname	0.108 (0.047-0.193)	0.138 (0.082-0.205)	0.148 (0.097-0.207)	0.165 (0.113-0.222)	0.181 (0.114-0.255)
Swaziland	0.045 (0.012-0.105)	0.069 (0.031-0.125)	0.103 (0.059-0.157)	0.133 (0.085-0.187)	0.158 (0.094-0.236)
Sweden	0.065 (0.032-0.109)	0.078 (0.052-0.108)	0.094 (0.069-0.121)	0.114 (0.085-0.145)	0.130 (0.086-0.180)
Switzerland	0.057 (0.025-0.103)	0.071 (0.042-0.108)	0.095 (0.063-0.131)	0.110 (0.078-0.147)	0.121 (0.077-0.173)
Syrian Arab Republic	0.092 (0.037-0.174)	0.124 (0.074-0.186)	0.144 (0.098-0.199)	0.168 (0.120-0.220)	0.184 (0.122-0.253)
Taiwan	0.022 (0.007-0.052)	0.037 (0.019-0.065)	0.052 (0.032-0.078)	0.062 (0.039-0.090)	0.069 (0.033-0.116)
Tajikistan	0.060 (0.018-0.129)	0.070 (0.031-0.123)	0.072 (0.039-0.115)	0.082 (0.049-0.123)	0.107 (0.059-0.167)
Tanzania	0.011 (0.003-0.027)	0.017 (0.008-0.032)	0.029 (0.017-0.046)	0.048 (0.032-0.069)	0.077 (0.048-0.115)
Thailand	0.011 (0.003-0.026)	0.021 (0.010-0.037)	0.036 (0.022-0.056)	0.057 (0.039-0.081)	0.081 (0.048-0.125)
Timor-Leste	0.003 (0.000-0.008)	0.005 (0.001-0.010)	0.009 (0.004-0.016)	0.014 (0.007-0.024)	0.025 (0.011-0.045)
Togo	0.015 (0.004-0.036)	0.023 (0.009-0.043)	0.035 (0.019-0.057)	0.056 (0.035-0.082)	0.080 (0.047-0.118)
Tokelau	0.171 (0.062-0.322)	0.197 (0.105-0.308)	0.216 (0.141-0.300)	0.227 (0.159-0.301)	0.231 (0.146-0.326)
Tonga	0.193 (0.097-0.313)	0.216 (0.139-0.306)	0.235 (0.167-0.313)	0.245 (0.184-0.313)	0.245 (0.172-0.323)
Trinidad and Tobago	0.079 (0.024-0.169)	0.102 (0.048-0.175)	0.120 (0.073-0.177)	0.151 (0.097-0.217)	0.172 (0.091-0.272)
Tunisia	0.095 (0.040-0.175)	0.128 (0.080-0.186)	0.156 (0.111-0.203)	0.181 (0.138-0.228)	0.198 (0.139-0.262)
Turkey	0.111 (0.052-0.190)	0.143 (0.097-0.196)	0.177 (0.141-0.216)	0.201 (0.168-0.235)	0.216 (0.166-0.269)
Turkmenistan	0.064 (0.020-0.137)	0.076 (0.036-0.133)	0.096 (0.055-0.148)	0.112 (0.070-0.161)	0.138 (0.083-0.205)
Tuvalu	0.160 (0.078-0.262)	0.193 (0.123-0.276)	0.226 (0.160-0.302)	0.240 (0.174-0.315)	0.245 (0.162-0.335)
Uganda	0.006 (0.001-0.018)	0.010 (0.003-0.022)	0.018 (0.008-0.032)	0.033 (0.018-0.054)	0.058 (0.032-0.094)
Ukraine	0.137 (0.062-0.238)	0.147 (0.089-0.216)	0.145 (0.096-0.204)	0.141 (0.093-0.196)	0.144 (0.082-0.216)
United Arab Emirates	0.217 (0.103-0.346)	0.223 (0.148-0.306)	0.228 (0.170-0.288)	0.243 (0.189-0.299)	0.240 (0.169-0.314)
United Kingdom	0.074 (0.041-0.117)	0.094 (0.070-0.121)	0.120 (0.100-0.142)	0.148 (0.128-0.170)	0.167 (0.131-0.204)
United States of America	0.094 (0.059-0.137)	0.115 (0.087-0.146)	0.138 (0.111-0.168)	0.159 (0.131-0.189)	0.170 (0.128-0.217)
Uruguay	0.099 (0.040-0.185)	0.119 (0.067-0.185)	0.140 (0.093-0.196)	0.158 (0.111-0.210)	0.181 (0.120-0.248)
Uzbekistan	0.051 (0.015-0.118)	0.063 (0.028-0.114)	0.078 (0.043-0.123)	0.094 (0.056-0.139)	0.120 (0.067-0.187)
Vanuatu	0.083 (0.024-0.177)	0.107 (0.049-0.183)	0.134 (0.080-0.200)	0.158 (0.105-0.218)	0.179 (0.109-0.257)
Venezuela	0.121 (0.053-0.211)	0.137 (0.082-0.203)	0.159 (0.110-0.212)	0.177 (0.133-0.224)	0.194 (0.133-0.260)
Viet Nam	0.002 (0.000-0.007)	0.004 (0.001-0.008)	0.007 (0.004-0.012)	0.015 (0.009-0.024)	0.028 (0.014-0.049)
Yemen	0.055 (0.018-0.119)	0.072 (0.037-0.122)	0.094 (0.057-0.139)	0.121 (0.081-0.167)	0.140 (0.086-0.203)
Zambia	0.025 (0.008-0.057)	0.036 (0.017-0.061)	0.046 (0.027-0.070)	0.065 (0.042-0.091)	0.096 (0.058-0.142)
Zimbabwe	0.059 (0.019-0.126)	0.080 (0.042-0.132)	0.105 (0.068-0.151)	0.128 (0.089-0.175)	0.144 (0.089-0.209)

Men, $35 \leq BMI < 40\ kg/m^2$

Country	Year				
	1975	1985	1995	2005	2014
Afghanistan	0.000 (0.000-0.000)	0.000 (0.000-0.000)	0.000 (0.000-0.001)	0.000 (0.000-0.001)	0.001 (0.000-0.003)
Albania	0.003 (0.000-0.013)	0.005 (0.001-0.015)	0.008 (0.002-0.018)	0.014 (0.006-0.029)	0.022 (0.008-0.047)
Algeria	0.002 (0.000-0.008)	0.004 (0.001-0.010)	0.007 (0.002-0.015)	0.012 (0.005-0.024)	0.020 (0.007-0.042)
American Samoa	0.065 (0.025-0.128)	0.090 (0.048-0.144)	0.116 (0.069-0.173)	0.132 (0.079-0.199)	0.142 (0.070-0.227)
Andorra	0.009 (0.002-0.025)	0.016 (0.005-0.036)	0.025 (0.009-0.050)	0.040 (0.018-0.074)	0.052 (0.019-0.102)
Angola	0.000 (0.000-0.001)	0.000 (0.000-0.001)	0.001 (0.000-0.002)	0.002 (0.000-0.006)	0.006 (0.001-0.019)
Antigua and Barbuda	0.002 (0.000-0.009)	0.005 (0.001-0.014)	0.012 (0.003-0.028)	0.023 (0.007-0.049)	0.040 (0.011-0.094)
Argentina	0.010 (0.002-0.028)	0.015 (0.005-0.032)	0.024 (0.011-0.043)	0.036 (0.020-0.060)	0.055 (0.026-0.097)
Armenia	0.005 (0.000-0.021)	0.007 (0.001-0.022)	0.009 (0.002-0.024)	0.014 (0.004-0.032)	0.024 (0.007-0.056)
Australia	0.009 (0.003-0.019)	0.013 (0.007-0.021)	0.022 (0.014-0.033)	0.040 (0.027-0.056)	0.060 (0.035-0.093)
Austria	0.005 (0.001-0.013)	0.008 (0.003-0.016)	0.014 (0.006-0.025)	0.024 (0.013-0.043)	0.037 (0.015-0.072)
Azerbaijan	0.004 (0.000-0.017)	0.007 (0.001-0.020)	0.009 (0.002-0.022)	0.013 (0.004-0.029)	0.029 (0.008-0.065)
Bahamas	0.006 (0.001-0.019)	0.011 (0.003-0.026)	0.021 (0.007-0.044)	0.036 (0.014-0.068)	0.050 (0.018-0.103)
Bahrain	0.012 (0.002-0.035)	0.016 (0.006-0.034)	0.025 (0.011-0.045)	0.036 (0.018-0.061)	0.048 (0.020-0.090)
Bangladesh	0.000 (0.000-0.000)	0.000 (0.000-0.000)	0.000 (0.000-0.000)	0.000 (0.000-0.001)	0.001 (0.000-0.003)
Barbados	0.002 (0.000-0.007)	0.004 (0.001-0.010)	0.008 (0.003-0.017)	0.017 (0.007-0.034)	0.032 (0.010-0.071)
Belarus	0.004 (0.000-0.014)	0.008 (0.002-0.020)	0.013 (0.004-0.031)	0.022 (0.008-0.046)	0.035 (0.011-0.076)
Belgium	0.005 (0.001-0.015)	0.008 (0.004-0.014)	0.013 (0.007-0.021)	0.023 (0.013-0.035)	0.035 (0.016-0.063)
Belize	0.002 (0.000-0.007)	0.003 (0.001-0.009)	0.007 (0.002-0.015)	0.014 (0.005-0.028)	0.023 (0.007-0.051)
Benin	0.000 (0.000-0.001)	0.000 (0.000-0.001)	0.001 (0.000-0.002)	0.002 (0.001-0.004)	0.004 (0.001-0.010)
Bermuda	0.013 (0.002-0.042)	0.020 (0.005-0.052)	0.031 (0.010-0.068)	0.048 (0.019-0.091)	0.072 (0.026-0.136)
Bhutan	0.000 (0.000-0.000)	0.000 (0.000-0.000)	0.000 (0.000-0.001)	0.001 (0.000-0.003)	0.003 (0.001-0.008)
Bolivia	0.001 (0.000-0.004)	0.002 (0.000-0.006)	0.004 (0.001-0.010)	0.007 (0.002-0.017)	0.013 (0.003-0.035)
Bosnia and Herzegovina	0.002 (0.000-0.008)	0.002 (0.000-0.008)	0.004 (0.001-0.011)	0.012 (0.004-0.024)	0.016 (0.005-0.033)
Botswana	0.000 (0.000-0.001)	0.000 (0.000-0.002)	0.002 (0.000-0.006)	0.006 (0.002-0.014)	0.013 (0.003-0.033)
Brazil	0.002 (0.000-0.005)	0.004 (0.001-0.009)	0.008 (0.004-0.014)	0.015 (0.009-0.023)	0.025 (0.012-0.045)
Brunei Darussalam	0.001 (0.000-0.007)	0.003 (0.000-0.010)	0.006 (0.001-0.017)	0.015 (0.004-0.035)	0.027 (0.007-0.065)
Bulgaria	0.006 (0.001-0.022)	0.012 (0.003-0.027)	0.018 (0.007-0.036)	0.025 (0.011-0.047)	0.036 (0.013-0.074)
Burkina Faso	0.000 (0.000-0.000)	0.000 (0.000-0.000)	0.000 (0.000-0.001)	0.001 (0.000-0.002)	0.002 (0.000-0.005)
Burundi	0.000 (0.000-0.000)	0.000 (0.000-0.000)	0.000 (0.000-0.000)	0.000 (0.000-0.001)	0.000 (0.000-0.002)
Cabo Verde	0.000 (0.000-0.001)	0.000 (0.000-0.001)	0.001 (0.000-0.003)	0.004 (0.001-0.009)	0.010 (0.003-0.025)
Cambodia	0.000 (0.000-0.000)	0.000 (0.000-0.000)	0.000 (0.000-0.000)	0.000 (0.000-0.001)	0.001 (0.000-0.003)
Cameroon	0.000 (0.000-0.001)	0.000 (0.000-0.001)	0.001 (0.000-0.003)	0.002 (0.001-0.005)	0.006 (0.001-0.014)
Canada	0.007 (0.002-0.017)	0.013 (0.006-0.023)	0.025 (0.014-0.040)	0.041 (0.024-0.062)	0.056 (0.027-0.095)
Central African Republic	0.000 (0.000-0.001)	0.000 (0.000-0.001)	0.000 (0.000-0.001)	0.001 (0.000-0.003)	0.002 (0.000-0.006)
Chad	0.000 (0.000-0.001)	0.000 (0.000-0.001)	0.000 (0.000-0.001)	0.001 (0.000-0.003)	0.003 (0.001-0.008)
Chile	0.006 (0.001-0.019)	0.010 (0.003-0.024)	0.019 (0.009-0.035)	0.032 (0.018-0.051)	0.047 (0.022-0.082)
China	0.000 (0.000-0.000)	0.000 (0.000-0.000)	0.000 (0.000-0.000)	0.001 (0.001-0.002)	0.006 (0.002-0.011)
China (Hong Kong SAR)	0.001 (0.000-0.003)	0.001 (0.000-0.004)	0.003 (0.001-0.008)	0.006 (0.002-0.015)	0.012 (0.002-0.035)
Colombia	0.002 (0.000-0.007)	0.004 (0.001-0.010)	0.008 (0.003-0.016)	0.014 (0.007-0.024)	0.023 (0.009-0.044)
Comoros	0.000 (0.000-0.001)	0.000 (0.000-0.001)	0.000 (0.000-0.001)	0.001 (0.000-0.002)	0.002 (0.000-0.005)
Congo	0.000 (0.000-0.001)	0.001 (0.000-0.003)	0.002 (0.000-0.005)	0.004 (0.001-0.009)	0.008 (0.002-0.022)
Cook Islands	0.035 (0.008-0.089)	0.058 (0.024-0.107)	0.090 (0.045-0.150)	0.119 (0.067-0.184)	0.139 (0.072-0.221)
Costa Rica	0.002 (0.000-0.007)	0.004 (0.001-0.011)	0.009 (0.003-0.020)	0.018 (0.008-0.034)	0.031 (0.013-0.062)
Cote d'Ivoire	0.000 (0.000-0.001)	0.001 (0.000-0.002)	0.001 (0.000-0.003)	0.002 (0.001-0.005)	0.005 (0.001-0.012)
Croatia	0.004 (0.000-0.014)	0.008 (0.002-0.020)	0.014 (0.005-0.029)	0.023 (0.010-0.044)	0.033 (0.011-0.068)
Cuba	0.003 (0.000-0.008)	0.006 (0.002-0.014)	0.011 (0.004-0.021)	0.017 (0.008-0.032)	0.031 (0.013-0.060)
Cyprus	0.002 (0.000-0.006)	0.006 (0.002-0.015)	0.016 (0.006-0.033)	0.030 (0.013-0.057)	0.045 (0.016-0.090)
Czech Republic	0.012 (0.003-0.035)	0.020 (0.008-0.038)	0.026 (0.013-0.044)	0.034 (0.018-0.056)	0.042 (0.017-0.080)
Denmark	0.005 (0.002-0.012)	0.009 (0.005-0.016)	0.016 (0.009-0.027)	0.025 (0.014-0.041)	0.036 (0.017-0.065)
Djibouti	0.001 (0.000-0.003)	0.001 (0.000-0.003)	0.002 (0.000-0.005)	0.003 (0.001-0.007)	0.005 (0.001-0.015)
Dominica	0.001 (0.000-0.005)	0.003 (0.001-0.008)	0.007 (0.002-0.016)	0.015 (0.005-0.029)	0.026 (0.009-0.057)
Dominican Republic	0.001 (0.000-0.003)	0.002 (0.000-0.006)	0.005 (0.002-0.010)	0.013 (0.006-0.025)	0.026 (0.009-0.055)
DR Congo	0.000 (0.000-0.001)	0.000 (0.000-0.001)	0.001 (0.000-0.002)	0.001 (0.000-0.003)	0.002 (0.000-0.007)
Ecuador	0.001 (0.000-0.004)	0.002 (0.000-0.007)	0.005 (0.001-0.013)	0.010 (0.003-0.021)	0.018 (0.005-0.041)
Egypt	0.006 (0.001-0.018)	0.010 (0.003-0.022)	0.018 (0.008-0.032)	0.028 (0.015-0.046)	0.043 (0.019-0.077)
El Salvador	0.002 (0.000-0.007)	0.003 (0.001-0.009)	0.007 (0.002-0.016)	0.014 (0.005-0.029)	0.023 (0.007-0.050)
Equatorial Guinea	0.000 (0.000-0.001)	0.000 (0.000-0.001)	0.001 (0.000-0.003)	0.006 (0.001-0.017)	0.017 (0.002-0.051)
Eritrea	0.000 (0.000-0.000)	0.000 (0.000-0.000)	0.000 (0.000-0.001)	0.000 (0.000-0.001)	0.001 (0.000-0.003)
Estonia	0.007 (0.001-0.021)	0.011 (0.005-0.024)	0.016 (0.008-0.028)	0.024 (0.015-0.036)	0.032 (0.015-0.054)
Ethiopia	0.000 (0.000-0.000)	0.000 (0.000-0.000)	0.000 (0.000-0.000)	0.000 (0.000-0.001)	0.001 (0.000-0.002)
Fiji	0.008 (0.001-0.034)	0.013 (0.002-0.038)	0.020 (0.006-0.047)	0.031 (0.012-0.061)	0.044 (0.016-0.089)
Finland	0.004 (0.001-0.010)	0.009 (0.004-0.015)	0.017 (0.010-0.027)	0.029 (0.019-0.042)	0.041 (0.022-0.067)
France	0.004 (0.001-0.013)	0.008 (0.003-0.016)	0.015 (0.007-0.025)	0.026 (0.014-0.042)	0.039 (0.017-0.069)
French Polynesia	0.044 (0.010-0.109)	0.065 (0.024-0.129)	0.089 (0.040-0.156)	0.110 (0.059-0.173)	0.127 (0.063-0.204)

Gabon	0.001 (0.000-0.003)	0.002 (0.000-0.006)	0.005 (0.001-0.014)	0.011 (0.003-0.026)	0.019 (0.004-0.048)
Gambia	0.000 (0.000-0.001)	0.000 (0.000-0.001)	0.001 (0.000-0.002)	0.002 (0.001-0.005)	0.005 (0.001-0.013)
Georgia	0.005 (0.000-0.020)	0.010 (0.002-0.026)	0.011 (0.003-0.028)	0.016 (0.005-0.034)	0.027 (0.009-0.059)
Germany	0.004 (0.001-0.010)	0.008 (0.005-0.013)	0.015 (0.010-0.022)	0.026 (0.017-0.038)	0.038 (0.018-0.066)
Ghana	0.000 (0.000-0.001)	0.001 (0.000-0.002)	0.001 (0.000-0.003)	0.004 (0.001-0.008)	0.009 (0.003-0.022)
Greece	0.004 (0.001-0.013)	0.008 (0.003-0.019)	0.015 (0.006-0.029)	0.026 (0.012-0.048)	0.039 (0.014-0.076)
Greenland	0.005 (0.001-0.014)	0.010 (0.003-0.022)	0.018 (0.007-0.034)	0.030 (0.014-0.052)	0.044 (0.018-0.082)
Grenada	0.001 (0.000-0.004)	0.002 (0.000-0.006)	0.006 (0.002-0.014)	0.013 (0.005-0.028)	0.026 (0.008-0.057)
Guatemala	0.001 (0.000-0.006)	0.003 (0.001-0.008)	0.005 (0.002-0.012)	0.011 (0.004-0.022)	0.018 (0.006-0.041)
Guinea	0.000 (0.000-0.000)	0.000 (0.000-0.001)	0.000 (0.000-0.001)	0.001 (0.000-0.003)	0.002 (0.001-0.007)
Guinea Bissau	0.000 (0.000-0.000)	0.000 (0.000-0.001)	0.001 (0.000-0.002)	0.001 (0.000-0.003)	0.003 (0.001-0.008)
Guyana	0.001 (0.000-0.005)	0.003 (0.001-0.008)	0.005 (0.002-0.013)	0.013 (0.004-0.028)	0.024 (0.007-0.054)
Haiti	0.000 (0.000-0.001)	0.001 (0.000-0.002)	0.001 (0.000-0.004)	0.003 (0.001-0.009)	0.008 (0.001-0.021)
Honduras	0.001 (0.000-0.004)	0.002 (0.000-0.006)	0.004 (0.001-0.010)	0.008 (0.003-0.018)	0.016 (0.004-0.037)
Hungary	0.010 (0.002-0.028)	0.017 (0.007-0.032)	0.023 (0.011-0.041)	0.030 (0.015-0.052)	0.039 (0.015-0.075)
Iceland	0.006 (0.001-0.016)	0.013 (0.005-0.025)	0.020 (0.009-0.036)	0.032 (0.016-0.055)	0.045 (0.019-0.084)
India	0.000 (0.000-0.000)	0.000 (0.000-0.000)	0.000 (0.000-0.001)	0.001 (0.000-0.001)	0.002 (0.001-0.003)
Indonesia	0.000 (0.000-0.000)	0.000 (0.000-0.000)	0.000 (0.000-0.000)	0.001 (0.000-0.002)	0.003 (0.001-0.008)
Iran	0.003 (0.000-0.010)	0.005 (0.002-0.011)	0.008 (0.004-0.013)	0.014 (0.009-0.020)	0.023 (0.012-0.039)
Iraq	0.006 (0.001-0.020)	0.009 (0.003-0.023)	0.012 (0.004-0.026)	0.019 (0.008-0.038)	0.031 (0.011-0.065)
Ireland	0.002 (0.000-0.008)	0.005 (0.002-0.011)	0.011 (0.005-0.022)	0.027 (0.013-0.047)	0.045 (0.020-0.082)
Israel	0.008 (0.002-0.023)	0.014 (0.005-0.028)	0.023 (0.011-0.041)	0.034 (0.018-0.057)	0.048 (0.020-0.090)
Italy	0.004 (0.001-0.009)	0.007 (0.004-0.012)	0.013 (0.008-0.020)	0.023 (0.014-0.033)	0.033 (0.016-0.056)
Jamaica	0.002 (0.000-0.006)	0.003 (0.001-0.007)	0.006 (0.003-0.012)	0.014 (0.006-0.025)	0.026 (0.010-0.055)
Japan	0.000 (0.000-0.000)	0.000 (0.000-0.000)	0.000 (0.000-0.001)	0.001 (0.001-0.002)	0.002 (0.001-0.005)
Jordan	0.005 (0.001-0.019)	0.011 (0.004-0.025)	0.020 (0.009-0.036)	0.031 (0.016-0.052)	0.046 (0.021-0.084)
Kazakhstan	0.005 (0.001-0.019)	0.009 (0.002-0.024)	0.015 (0.004-0.034)	0.023 (0.008-0.048)	0.037 (0.011-0.082)
Kenya	0.000 (0.000-0.000)	0.000 (0.000-0.001)	0.000 (0.000-0.001)	0.001 (0.000-0.003)	0.003 (0.001-0.008)
Kiribati	0.023 (0.005-0.059)	0.034 (0.012-0.068)	0.050 (0.021-0.091)	0.074 (0.035-0.124)	0.090 (0.037-0.165)
Kuwait	0.021 (0.004-0.057)	0.027 (0.012-0.050)	0.037 (0.020-0.059)	0.051 (0.031-0.078)	0.066 (0.033-0.111)
Kyrgyzstan	0.003 (0.000-0.012)	0.004 (0.001-0.013)	0.006 (0.002-0.016)	0.009 (0.003-0.022)	0.016 (0.004-0.039)
Lao PDR	0.000 (0.000-0.000)	0.000 (0.000-0.000)	0.000 (0.000-0.000)	0.000 (0.000-0.001)	0.002 (0.000-0.004)
Latvia	0.007 (0.001-0.022)	0.013 (0.004-0.029)	0.018 (0.007-0.038)	0.026 (0.011-0.050)	0.039 (0.014-0.078)
Lebanon	0.006 (0.001-0.021)	0.011 (0.004-0.026)	0.017 (0.007-0.035)	0.027 (0.011-0.050)	0.042 (0.017-0.079)
Lesotho	0.000 (0.000-0.001)	0.000 (0.000-0.001)	0.001 (0.000-0.002)	0.002 (0.001-0.005)	0.006 (0.001-0.014)
Liberia	0.000 (0.000-0.001)	0.000 (0.000-0.002)	0.001 (0.000-0.002)	0.001 (0.000-0.003)	0.003 (0.001-0.008)
Libya	0.008 (0.001-0.029)	0.015 (0.005-0.033)	0.021 (0.009-0.040)	0.029 (0.014-0.051)	0.039 (0.016-0.073)
Lithuania	0.006 (0.001-0.018)	0.012 (0.004-0.025)	0.019 (0.008-0.035)	0.028 (0.014-0.049)	0.044 (0.018-0.085)
Luxembourg	0.005 (0.001-0.014)	0.009 (0.003-0.021)	0.018 (0.006-0.039)	0.031 (0.014-0.057)	0.046 (0.019-0.087)
Macedonia (TFYR)	0.005 (0.001-0.019)	0.009 (0.002-0.022)	0.014 (0.004-0.030)	0.019 (0.007-0.039)	0.025 (0.008-0.057)
Madagascar	0.000 (0.000-0.000)	0.000 (0.000-0.001)	0.000 (0.000-0.001)	0.001 (0.000-0.002)	0.002 (0.000-0.005)
Malawi	0.000 (0.000-0.000)	0.000 (0.000-0.000)	0.000 (0.000-0.001)	0.000 (0.000-0.001)	0.001 (0.000-0.003)
Malaysia	0.000 (0.000-0.001)	0.001 (0.000-0.002)	0.002 (0.001-0.005)	0.008 (0.004-0.015)	0.020 (0.008-0.040)
Maldives	0.000 (0.000-0.000)	0.000 (0.000-0.000)	0.000 (0.000-0.001)	0.002 (0.000-0.006)	0.006 (0.001-0.015)
Mali	0.000 (0.000-0.001)	0.000 (0.000-0.001)	0.001 (0.000-0.002)	0.001 (0.000-0.004)	0.003 (0.001-0.010)
Malta	0.007 (0.001-0.025)	0.013 (0.004-0.030)	0.022 (0.009-0.044)	0.038 (0.017-0.068)	0.053 (0.020-0.103)
Marshall Islands	0.034 (0.007-0.088)	0.049 (0.017-0.099)	0.072 (0.034-0.125)	0.088 (0.045-0.147)	0.103 (0.042-0.185)
Mauritania	0.000 (0.000-0.001)	0.000 (0.000-0.001)	0.001 (0.000-0.002)	0.002 (0.000-0.005)	0.004 (0.001-0.012)
Mauritius	0.000 (0.000-0.002)	0.001 (0.000-0.003)	0.003 (0.001-0.006)	0.007 (0.003-0.015)	0.016 (0.005-0.039)
Mexico	0.005 (0.001-0.015)	0.010 (0.003-0.021)	0.019 (0.010-0.032)	0.032 (0.021-0.049)	0.048 (0.025-0.080)
Micronesia (Federated States of)	0.022 (0.004-0.060)	0.034 (0.013-0.071)	0.051 (0.024-0.091)	0.069 (0.038-0.109)	0.087 (0.042-0.149)
Moldova	0.003 (0.000-0.010)	0.005 (0.001-0.014)	0.008 (0.002-0.019)	0.011 (0.004-0.025)	0.019 (0.006-0.044)
Mongolia	0.003 (0.000-0.011)	0.004 (0.001-0.013)	0.007 (0.002-0.016)	0.010 (0.004-0.021)	0.019 (0.007-0.041)
Montenegro	0.004 (0.000-0.016)	0.008 (0.002-0.023)	0.014 (0.004-0.031)	0.021 (0.008-0.042)	0.029 (0.009-0.062)
Morocco	0.002 (0.000-0.008)	0.003 (0.001-0.010)	0.006 (0.002-0.014)	0.011 (0.004-0.022)	0.019 (0.006-0.042)
Mozambique	0.000 (0.000-0.000)	0.000 (0.000-0.000)	0.000 (0.000-0.001)	0.000 (0.000-0.001)	0.001 (0.000-0.003)
Myanmar	0.000 (0.000-0.000)	0.000 (0.000-0.000)	0.000 (0.000-0.000)	0.000 (0.000-0.001)	0.002 (0.000-0.004)
Namibia	0.000 (0.000-0.002)	0.001 (0.000-0.003)	0.002 (0.000-0.005)	0.005 (0.002-0.011)	0.012 (0.003-0.029)
Nauru	0.092 (0.037-0.170)	0.114 (0.067-0.171)	0.125 (0.079-0.180)	0.123 (0.074-0.184)	0.130 (0.056-0.220)
Nepal	0.000 (0.000-0.000)	0.000 (0.000-0.000)	0.000 (0.000-0.001)	0.001 (0.000-0.002)	0.002 (0.000-0.004)
Netherlands	0.002 (0.000-0.006)	0.004 (0.002-0.008)	0.008 (0.004-0.015)	0.018 (0.010-0.028)	0.027 (0.012-0.050)
New Zealand	0.007 (0.002-0.018)	0.012 (0.005-0.022)	0.022 (0.012-0.035)	0.040 (0.026-0.059)	0.062 (0.037-0.094)
Nicaragua	0.002 (0.000-0.007)	0.003 (0.001-0.009)	0.005 (0.001-0.012)	0.010 (0.003-0.021)	0.017 (0.005-0.039)
Niger	0.000 (0.000-0.000)	0.000 (0.000-0.000)	0.000 (0.000-0.001)	0.001 (0.000-0.002)	0.001 (0.000-0.003)
Nigeria	0.000 (0.000-0.001)	0.000 (0.000-0.001)	0.001 (0.000-0.002)	0.003 (0.001-0.005)	0.007 (0.003-0.016)
Niue	0.021 (0.004-0.061)	0.040 (0.013-0.085)	0.066 (0.028-0.119)	0.093 (0.048-0.149)	0.120 (0.059-0.193)
North Korea	0.000 (0.000-0.000)	0.000 (0.000-0.001)	0.000 (0.000-0.001)	0.001 (0.000-0.002)	0.001 (0.000-0.005)
Norway	0.004 (0.001-0.011)	0.010 (0.004-0.018)	0.020 (0.011-0.033)	0.036 (0.022-0.055)	0.050 (0.025-0.087)

Occupied Palestinian Territory	0.007 (0.001-0.022)	0.010 (0.003-0.025)	0.016 (0.006-0.031)	0.026 (0.012-0.047)	0.037 (0.013-0.075)
Oman	0.002 (0.000-0.009)	0.006 (0.002-0.015)	0.014 (0.006-0.025)	0.023 (0.012-0.039)	0.036 (0.015-0.068)
Pakistan	0.000 (0.000-0.001)	0.000 (0.000-0.001)	0.001 (0.000-0.002)	0.002 (0.000-0.004)	0.003 (0.001-0.009)
Palau	0.053 (0.013-0.125)	0.070 (0.029-0.128)	0.094 (0.046-0.153)	0.114 (0.063-0.174)	0.129 (0.064-0.205)
Panama	0.003 (0.000-0.010)	0.005 (0.001-0.014)	0.010 (0.003-0.022)	0.019 (0.008-0.036)	0.035 (0.013-0.069)
Papua New Guinea	0.003 (0.000-0.016)	0.005 (0.001-0.017)	0.008 (0.002-0.022)	0.012 (0.003-0.029)	0.019 (0.004-0.051)
Paraguay	0.001 (0.000-0.005)	0.003 (0.000-0.008)	0.006 (0.002-0.016)	0.012 (0.004-0.028)	0.021 (0.006-0.050)
Peru	0.002 (0.000-0.007)	0.003 (0.001-0.010)	0.006 (0.002-0.014)	0.011 (0.005-0.021)	0.021 (0.008-0.042)
Philippines	0.000 (0.000-0.000)	0.000 (0.000-0.001)	0.001 (0.000-0.002)	0.002 (0.001-0.004)	0.005 (0.001-0.012)
Poland	0.009 (0.002-0.026)	0.015 (0.007-0.027)	0.021 (0.012-0.034)	0.032 (0.020-0.047)	0.043 (0.021-0.073)
Portugal	0.001 (0.000-0.005)	0.003 (0.001-0.007)	0.008 (0.003-0.014)	0.017 (0.009-0.028)	0.029 (0.011-0.056)
Puerto Rico	0.004 (0.000-0.013)	0.010 (0.003-0.023)	0.024 (0.009-0.047)	0.041 (0.019-0.072)	0.057 (0.023-0.108)
Qatar	0.014 (0.002-0.042)	0.031 (0.011-0.067)	0.040 (0.019-0.072)	0.061 (0.033-0.099)	0.081 (0.040-0.137)
Romania	0.005 (0.001-0.018)	0.010 (0.003-0.023)	0.016 (0.006-0.030)	0.023 (0.010-0.041)	0.032 (0.011-0.067)
Russian Federation	0.005 (0.001-0.013)	0.009 (0.004-0.015)	0.014 (0.009-0.021)	0.022 (0.013-0.032)	0.034 (0.014-0.062)
Rwanda	0.000 (0.000-0.000)	0.000 (0.000-0.000)	0.000 (0.000-0.000)	0.000 (0.000-0.001)	0.001 (0.000-0.002)
Saint Kitts and Nevis	0.002 (0.000-0.007)	0.005 (0.001-0.013)	0.010 (0.003-0.024)	0.022 (0.009-0.044)	0.038 (0.012-0.084)
Saint Lucia	0.001 (0.000-0.004)	0.002 (0.000-0.005)	0.005 (0.002-0.012)	0.013 (0.005-0.027)	0.027 (0.008-0.063)
Saint Vincent and the Grenadines	0.001 (0.000-0.005)	0.002 (0.000-0.007)	0.006 (0.002-0.015)	0.014 (0.005-0.030)	0.026 (0.008-0.060)
Samoa	0.022 (0.005-0.059)	0.036 (0.014-0.072)	0.060 (0.029-0.103)	0.088 (0.050-0.137)	0.109 (0.054-0.179)
Sao Tome and Principe	0.000 (0.000-0.002)	0.001 (0.000-0.002)	0.002 (0.000-0.005)	0.004 (0.001-0.009)	0.008 (0.002-0.019)
Saudi Arabia	0.008 (0.001-0.027)	0.017 (0.007-0.033)	0.027 (0.016-0.041)	0.039 (0.024-0.058)	0.058 (0.029-0.098)
Senegal	0.000 (0.000-0.001)	0.000 (0.000-0.001)	0.001 (0.000-0.002)	0.002 (0.000-0.004)	0.004 (0.001-0.010)
Serbia	0.004 (0.001-0.014)	0.008 (0.003-0.018)	0.013 (0.006-0.024)	0.019 (0.010-0.032)	0.025 (0.011-0.047)
Seychelles	0.001 (0.000-0.003)	0.002 (0.000-0.005)	0.005 (0.001-0.011)	0.012 (0.004-0.025)	0.025 (0.008-0.056)
Sierra Leone	0.000 (0.000-0.001)	0.000 (0.000-0.001)	0.001 (0.000-0.002)	0.001 (0.000-0.003)	0.003 (0.001-0.008)
Singapore	0.000 (0.000-0.001)	0.000 (0.000-0.001)	0.001 (0.000-0.003)	0.003 (0.001-0.005)	0.005 (0.002-0.012)
Slovakia	0.004 (0.001-0.014)	0.008 (0.003-0.019)	0.014 (0.006-0.027)	0.021 (0.010-0.038)	0.032 (0.013-0.062)
Slovenia	0.004 (0.000-0.014)	0.008 (0.002-0.020)	0.014 (0.005-0.030)	0.022 (0.008-0.043)	0.029 (0.009-0.062)
Solomon Islands	0.003 (0.000-0.013)	0.004 (0.001-0.016)	0.008 (0.001-0.021)	0.011 (0.003-0.026)	0.016 (0.004-0.041)
Somalia	0.000 (0.000-0.001)	0.000 (0.000-0.001)	0.000 (0.000-0.001)	0.001 (0.000-0.002)	0.001 (0.000-0.004)
South Africa	0.002 (0.000-0.007)	0.004 (0.001-0.010)	0.008 (0.004-0.016)	0.016 (0.009-0.027)	0.031 (0.015-0.053)
South Korea	0.000 (0.000-0.000)	0.000 (0.000-0.000)	0.000 (0.000-0.001)	0.001 (0.000-0.002)	0.002 (0.001-0.005)
Spain	0.003 (0.001-0.009)	0.007 (0.003-0.013)	0.015 (0.009-0.023)	0.028 (0.018-0.041)	0.041 (0.019-0.073)
Sri Lanka	0.000 (0.000-0.000)	0.000 (0.000-0.000)	0.000 (0.000-0.001)	0.001 (0.000-0.002)	0.004 (0.001-0.011)
Sudan	0.000 (0.000-0.001)	0.000 (0.000-0.001)	0.001 (0.000-0.002)	0.002 (0.000-0.004)	0.004 (0.001-0.010)
Suriname	0.003 (0.000-0.010)	0.006 (0.001-0.016)	0.010 (0.003-0.023)	0.018 (0.006-0.038)	0.032 (0.010-0.068)
Swaziland	0.000 (0.000-0.001)	0.001 (0.000-0.002)	0.002 (0.000-0.005)	0.004 (0.001-0.011)	0.010 (0.002-0.026)
Sweden	0.006 (0.002-0.013)	0.010 (0.005-0.016)	0.017 (0.010-0.025)	0.029 (0.019-0.042)	0.042 (0.021-0.071)
Switzerland	0.004 (0.001-0.013)	0.008 (0.003-0.016)	0.018 (0.009-0.030)	0.028 (0.016-0.045)	0.040 (0.020-0.071)
Syrian Arab Republic	0.003 (0.000-0.011)	0.006 (0.001-0.014)	0.009 (0.003-0.020)	0.015 (0.006-0.031)	0.023 (0.008-0.051)
Taiwan	0.000 (0.000-0.000)	0.000 (0.000-0.001)	0.001 (0.000-0.002)	0.003 (0.001-0.006)	0.007 (0.002-0.017)
Tajikistan	0.003 (0.000-0.012)	0.004 (0.001-0.013)	0.005 (0.001-0.014)	0.006 (0.001-0.016)	0.012 (0.003-0.033)
Tanzania	0.000 (0.000-0.000)	0.000 (0.000-0.001)	0.000 (0.000-0.001)	0.001 (0.000-0.002)	0.003 (0.001-0.006)
Thailand	0.000 (0.000-0.000)	0.000 (0.000-0.000)	0.001 (0.000-0.001)	0.002 (0.001-0.005)	0.008 (0.002-0.018)
Timor-Leste	0.000 (0.000-0.000)	0.000 (0.000-0.000)	0.000 (0.000-0.000)	0.000 (0.000-0.000)	0.001 (0.000-0.002)
Togo	0.000 (0.000-0.001)	0.000 (0.000-0.001)	0.001 (0.000-0.002)	0.001 (0.000-0.003)	0.003 (0.001-0.008)
Tokelau	0.021 (0.002-0.074)	0.033 (0.007-0.086)	0.054 (0.019-0.109)	0.078 (0.035-0.136)	0.103 (0.040-0.191)
Tonga	0.027 (0.005-0.073)	0.043 (0.015-0.090)	0.067 (0.031-0.119)	0.093 (0.051-0.148)	0.114 (0.057-0.189)
Trinidad and Tobago	0.001 (0.000-0.007)	0.003 (0.000-0.010)	0.006 (0.002-0.015)	0.016 (0.005-0.035)	0.035 (0.008-0.087)
Tunisia	0.003 (0.000-0.010)	0.005 (0.001-0.011)	0.008 (0.003-0.016)	0.015 (0.007-0.027)	0.024 (0.009-0.048)
Turkey	0.003 (0.001-0.011)	0.006 (0.003-0.014)	0.013 (0.007-0.022)	0.023 (0.015-0.034)	0.037 (0.020-0.062)
Turkmenistan	0.003 (0.000-0.014)	0.005 (0.001-0.015)	0.009 (0.002-0.022)	0.013 (0.004-0.029)	0.025 (0.008-0.056)
Tuvalu	0.019 (0.004-0.053)	0.033 (0.011-0.071)	0.059 (0.025-0.109)	0.082 (0.040-0.141)	0.102 (0.042-0.182)
Uganda	0.000 (0.000-0.000)	0.000 (0.000-0.000)	0.000 (0.000-0.000)	0.000 (0.000-0.001)	0.001 (0.000-0.003)
Ukraine	0.005 (0.001-0.019)	0.010 (0.002-0.024)	0.015 (0.005-0.032)	0.019 (0.007-0.042)	0.029 (0.008-0.066)
United Arab Emirates	0.022 (0.003-0.070)	0.026 (0.008-0.057)	0.033 (0.015-0.059)	0.050 (0.026-0.082)	0.058 (0.024-0.106)
United Kingdom	0.006 (0.002-0.014)	0.010 (0.005-0.016)	0.018 (0.013-0.025)	0.036 (0.027-0.045)	0.056 (0.037-0.080)
United States of America	0.012 (0.005-0.025)	0.021 (0.012-0.033)	0.037 (0.024-0.052)	0.060 (0.042-0.081)	0.080 (0.048-0.116)
Uruguay	0.008 (0.001-0.026)	0.012 (0.003-0.030)	0.020 (0.008-0.040)	0.030 (0.014-0.055)	0.046 (0.019-0.087)
Uzbekistan	0.003 (0.000-0.011)	0.004 (0.001-0.013)	0.006 (0.001-0.016)	0.009 (0.003-0.022)	0.017 (0.004-0.043)
Vanuatu	0.005 (0.000-0.023)	0.007 (0.001-0.024)	0.012 (0.003-0.031)	0.019 (0.006-0.040)	0.029 (0.009-0.065)
Venezuela	0.005 (0.001-0.018)	0.010 (0.003-0.023)	0.017 (0.007-0.034)	0.027 (0.014-0.046)	0.040 (0.017-0.074)
Viet Nam	0.000 (0.000-0.000)	0.000 (0.000-0.000)	0.000 (0.000-0.000)	0.000 (0.000-0.000)	0.001 (0.000-0.002)
Yemen	0.001 (0.000-0.004)	0.002 (0.000-0.005)	0.003 (0.001-0.007)	0.005 (0.002-0.012)	0.009 (0.002-0.021)
Zambia	0.000 (0.000-0.001)	0.000 (0.000-0.002)	0.001 (0.000-0.002)	0.001 (0.000-0.004)	0.004 (0.001-0.010)
Zimbabwe	0.000 (0.000-0.001)	0.000 (0.000-0.001)	0.001 (0.000-0.003)	0.002 (0.001-0.006)	0.004 (0.001-0.011)

Women, 35 ≤ BMI < 40 kg/m^2

Country	Year				
	1975	1985	1995	2005	2014
Afghanistan	0.001 (0.000-0.003)	0.001 (0.000-0.004)	0.002 (0.001-0.006)	0.004 (0.001-0.009)	0.008 (0.002-0.018)
Albania	0.014 (0.003-0.039)	0.017 (0.005-0.038)	0.020 (0.008-0.040)	0.029 (0.014-0.052)	0.038 (0.015-0.073)
Algeria	0.017 (0.004-0.046)	0.028 (0.011-0.055)	0.040 (0.020-0.069)	0.056 (0.031-0.091)	0.073 (0.038-0.120)
American Samoa	0.141 (0.075-0.227)	0.155 (0.099-0.221)	0.167 (0.113-0.231)	0.175 (0.114-0.245)	0.180 (0.100-0.269)
Andorra	0.034 (0.010-0.078)	0.044 (0.018-0.082)	0.051 (0.024-0.089)	0.064 (0.033-0.107)	0.070 (0.031-0.127)
Angola	0.002 (0.000-0.008)	0.004 (0.001-0.011)	0.007 (0.002-0.017)	0.014 (0.004-0.031)	0.028 (0.009-0.064)
Antigua and Barbuda	0.022 (0.004-0.057)	0.036 (0.013-0.073)	0.059 (0.027-0.104)	0.082 (0.042-0.138)	0.112 (0.049-0.197)
Argentina	0.028 (0.008-0.064)	0.037 (0.017-0.069)	0.050 (0.028-0.080)	0.067 (0.042-0.098)	0.091 (0.052-0.142)
Armenia	0.015 (0.002-0.043)	0.021 (0.006-0.047)	0.025 (0.010-0.050)	0.037 (0.017-0.064)	0.054 (0.022-0.100)
Australia	0.018 (0.008-0.034)	0.028 (0.018-0.041)	0.041 (0.029-0.057)	0.059 (0.043-0.079)	0.076 (0.049-0.111)
Austria	0.012 (0.004-0.027)	0.017 (0.008-0.030)	0.024 (0.012-0.039)	0.033 (0.018-0.053)	0.041 (0.019-0.073)
Azerbaijan	0.018 (0.003-0.052)	0.026 (0.008-0.057)	0.031 (0.013-0.058)	0.042 (0.021-0.074)	0.069 (0.031-0.126)
Bahamas	0.040 (0.010-0.096)	0.056 (0.023-0.107)	0.075 (0.038-0.127)	0.096 (0.052-0.152)	0.113 (0.056-0.183)
Bahrain	0.050 (0.015-0.109)	0.056 (0.027-0.096)	0.070 (0.039-0.106)	0.086 (0.051-0.127)	0.101 (0.055-0.157)
Bangladesh	0.000 (0.000-0.001)	0.001 (0.000-0.001)	0.001 (0.000-0.003)	0.003 (0.001-0.005)	0.006 (0.002-0.012)
Barbados	0.019 (0.005-0.049)	0.032 (0.013-0.060)	0.047 (0.025-0.077)	0.071 (0.040-0.113)	0.098 (0.046-0.169)
Belarus	0.022 (0.004-0.061)	0.032 (0.011-0.067)	0.040 (0.017-0.075)	0.049 (0.023-0.087)	0.062 (0.026-0.116)
Belgium	0.017 (0.006-0.037)	0.023 (0.013-0.036)	0.030 (0.020-0.044)	0.038 (0.024-0.055)	0.042 (0.021-0.071)
Belize	0.015 (0.003-0.043)	0.024 (0.008-0.050)	0.038 (0.017-0.067)	0.057 (0.030-0.093)	0.077 (0.035-0.135)
Benin	0.002 (0.000-0.006)	0.004 (0.001-0.009)	0.008 (0.003-0.015)	0.016 (0.008-0.027)	0.027 (0.013-0.049)
Bermuda	0.066 (0.018-0.144)	0.079 (0.033-0.146)	0.093 (0.046-0.155)	0.108 (0.059-0.170)	0.133 (0.066-0.218)
Bhutan	0.000 (0.000-0.002)	0.001 (0.000-0.003)	0.003 (0.001-0.007)	0.007 (0.003-0.014)	0.013 (0.005-0.026)
Bolivia	0.008 (0.001-0.026)	0.014 (0.004-0.032)	0.022 (0.009-0.043)	0.036 (0.018-0.063)	0.055 (0.025-0.100)
Bosnia and Herzegovina	0.007 (0.001-0.025)	0.009 (0.002-0.024)	0.014 (0.005-0.029)	0.029 (0.014-0.051)	0.034 (0.015-0.062)
Botswana	0.007 (0.001-0.024)	0.017 (0.004-0.042)	0.037 (0.015-0.072)	0.054 (0.025-0.093)	0.075 (0.033-0.132)
Brazil	0.010 (0.002-0.026)	0.020 (0.009-0.034)	0.031 (0.020-0.046)	0.047 (0.034-0.063)	0.067 (0.041-0.100)
Brunei Darussalam	0.009 (0.001-0.035)	0.014 (0.003-0.039)	0.021 (0.007-0.046)	0.033 (0.013-0.063)	0.043 (0.015-0.090)
Bulgaria	0.022 (0.005-0.056)	0.030 (0.011-0.059)	0.035 (0.016-0.063)	0.041 (0.020-0.071)	0.050 (0.021-0.094)
Burkina Faso	0.001 (0.000-0.002)	0.001 (0.000-0.003)	0.003 (0.001-0.006)	0.008 (0.004-0.014)	0.015 (0.006-0.030)
Burundi	0.000 (0.000-0.001)	0.000 (0.000-0.002)	0.001 (0.000-0.003)	0.003 (0.001-0.007)	0.007 (0.002-0.017)
Cabo Verde	0.001 (0.000-0.005)	0.003 (0.001-0.009)	0.009 (0.003-0.019)	0.023 (0.010-0.043)	0.039 (0.016-0.074)
Cambodia	0.000 (0.000-0.000)	0.000 (0.000-0.001)	0.001 (0.000-0.002)	0.002 (0.001-0.004)	0.006 (0.002-0.013)
Cameroon	0.003 (0.000-0.008)	0.006 (0.002-0.013)	0.011 (0.005-0.020)	0.021 (0.012-0.034)	0.036 (0.018-0.063)
Canada	0.015 (0.006-0.030)	0.026 (0.015-0.042)	0.041 (0.026-0.061)	0.059 (0.038-0.084)	0.072 (0.041-0.113)
Central African Republic	0.001 (0.000-0.005)	0.002 (0.000-0.007)	0.004 (0.001-0.010)	0.008 (0.003-0.017)	0.015 (0.004-0.036)
Chad	0.001 (0.000-0.004)	0.002 (0.000-0.005)	0.005 (0.002-0.010)	0.011 (0.005-0.020)	0.022 (0.009-0.042)
Chile	0.023 (0.005-0.057)	0.034 (0.014-0.064)	0.050 (0.028-0.078)	0.070 (0.045-0.101)	0.093 (0.054-0.143)
China	0.000 (0.000-0.001)	0.000 (0.000-0.001)	0.001 (0.001-0.002)	0.004 (0.003-0.007)	0.011 (0.005-0.020)
China (Hong Kong SAR)	0.005 (0.001-0.020)	0.007 (0.002-0.018)	0.009 (0.003-0.021)	0.011 (0.004-0.025)	0.014 (0.003-0.038)
Colombia	0.011 (0.002-0.030)	0.019 (0.007-0.037)	0.029 (0.015-0.048)	0.043 (0.027-0.064)	0.060 (0.033-0.098)
Comoros	0.002 (0.000-0.007)	0.003 (0.001-0.008)	0.006 (0.002-0.012)	0.010 (0.004-0.020)	0.019 (0.008-0.038)
Congo	0.003 (0.000-0.008)	0.006 (0.002-0.013)	0.011 (0.005-0.021)	0.018 (0.008-0.032)	0.030 (0.012-0.060)
Cook Islands	0.095 (0.036-0.183)	0.124 (0.068-0.197)	0.150 (0.090-0.223)	0.168 (0.107-0.241)	0.180 (0.102-0.268)
Costa Rica	0.010 (0.002-0.030)	0.019 (0.006-0.040)	0.032 (0.015-0.058)	0.052 (0.029-0.082)	0.073 (0.039-0.120)
Cote d'Ivoire	0.003 (0.000-0.010)	0.006 (0.002-0.013)	0.010 (0.004-0.019)	0.017 (0.009-0.030)	0.027 (0.013-0.050)
Croatia	0.015 (0.003-0.042)	0.023 (0.008-0.048)	0.029 (0.013-0.055)	0.039 (0.018-0.068)	0.049 (0.020-0.093)
Cuba	0.021 (0.005-0.053)	0.036 (0.016-0.068)	0.048 (0.025-0.078)	0.059 (0.034-0.091)	0.079 (0.041-0.128)
Cyprus	0.009 (0.002-0.027)	0.021 (0.008-0.045)	0.038 (0.017-0.069)	0.053 (0.026-0.093)	0.065 (0.028-0.122)
Czech Republic	0.039 (0.013-0.086)	0.048 (0.025-0.082)	0.052 (0.030-0.080)	0.057 (0.033-0.087)	0.061 (0.029-0.105)
Denmark	0.014 (0.005-0.032)	0.021 (0.010-0.036)	0.028 (0.015-0.045)	0.035 (0.020-0.055)	0.041 (0.020-0.071)
Djibouti	0.006 (0.001-0.019)	0.007 (0.001-0.019)	0.011 (0.003-0.025)	0.016 (0.005-0.035)	0.025 (0.007-0.057)
Dominica	0.016 (0.003-0.044)	0.027 (0.010-0.056)	0.045 (0.022-0.080)	0.065 (0.035-0.106)	0.089 (0.045-0.149)
Dominican Republic	0.009 (0.002-0.026)	0.019 (0.008-0.037)	0.031 (0.017-0.052)	0.055 (0.032-0.084)	0.078 (0.040-0.128)
DR Congo	0.002 (0.000-0.006)	0.003 (0.001-0.007)	0.005 (0.001-0.010)	0.008 (0.003-0.016)	0.016 (0.005-0.035)
Ecuador	0.007 (0.001-0.023)	0.014 (0.004-0.034)	0.024 (0.009-0.048)	0.037 (0.018-0.065)	0.055 (0.025-0.099)
Egypt	0.027 (0.007-0.065)	0.043 (0.022-0.074)	0.062 (0.040-0.092)	0.086 (0.060-0.118)	0.113 (0.072-0.164)
El Salvador	0.011 (0.002-0.033)	0.018 (0.006-0.039)	0.030 (0.014-0.054)	0.049 (0.027-0.078)	0.068 (0.034-0.113)
Equatorial Guinea	0.001 (0.000-0.006)	0.003 (0.001-0.009)	0.007 (0.002-0.016)	0.030 (0.010-0.062)	0.053 (0.017-0.115)
Eritrea	0.001 (0.000-0.002)	0.001 (0.000-0.003)	0.002 (0.001-0.005)	0.006 (0.002-0.011)	0.012 (0.004-0.024)
Estonia	0.031 (0.009-0.075)	0.037 (0.018-0.065)	0.040 (0.023-0.062)	0.046 (0.031-0.065)	0.053 (0.029-0.082)
Ethiopia	0.000 (0.000-0.002)	0.001 (0.000-0.002)	0.002 (0.000-0.004)	0.004 (0.001-0.007)	0.009 (0.003-0.018)
Fiji	0.036 (0.007-0.104)	0.049 (0.016-0.109)	0.063 (0.029-0.114)	0.080 (0.044-0.129)	0.095 (0.047-0.156)
Finland	0.011 (0.004-0.023)	0.019 (0.011-0.031)	0.029 (0.019-0.042)	0.039 (0.027-0.054)	0.046 (0.026-0.072)
France	0.021 (0.007-0.048)	0.029 (0.014-0.050)	0.038 (0.022-0.060)	0.050 (0.030-0.076)	0.060 (0.031-0.099)
French Polynesia	0.116 (0.045-0.226)	0.135 (0.067-0.224)	0.151 (0.084-0.231)	0.164 (0.100-0.239)	0.175 (0.099-0.258)
Gabon	0.006 (0.001-0.022)	0.015 (0.004-0.036)	0.025 (0.009-0.050)	0.040 (0.019-0.072)	0.056 (0.025-0.103)
Gambia	0.002 (0.000-0.006)	0.004 (0.001-0.009)	0.008 (0.003-0.017)	0.017 (0.008-0.031)	0.029 (0.012-0.054)
Georgia	0.017 (0.003-0.052)	0.028 (0.009-0.064)	0.030 (0.012-0.060)	0.042 (0.020-0.075)	0.062 (0.027-0.111)

Germany	0.013 (0.005-0.026)	0.020 (0.013-0.030)	0.028 (0.020-0.039)	0.037 (0.026-0.052)	0.046 (0.025-0.073)
Ghana	0.003 (0.001-0.010)	0.006 (0.002-0.013)	0.012 (0.006-0.021)	0.024 (0.013-0.038)	0.040 (0.019-0.071)
Greece	0.021 (0.006-0.052)	0.031 (0.014-0.060)	0.040 (0.020-0.068)	0.053 (0.029-0.087)	0.062 (0.029-0.109)
Greenland	0.017 (0.005-0.039)	0.026 (0.011-0.050)	0.035 (0.018-0.061)	0.046 (0.024-0.075)	0.055 (0.027-0.097)
Grenada	0.012 (0.002-0.033)	0.021 (0.007-0.046)	0.039 (0.017-0.070)	0.063 (0.033-0.104)	0.091 (0.044-0.157)
Guatemala	0.009 (0.001-0.025)	0.014 (0.005-0.031)	0.023 (0.011-0.041)	0.039 (0.022-0.062)	0.057 (0.028-0.097)
Guinea	0.001 (0.000-0.004)	0.003 (0.001-0.007)	0.005 (0.002-0.011)	0.011 (0.005-0.020)	0.020 (0.008-0.038)
Guinea Bissau	0.001 (0.000-0.005)	0.003 (0.001-0.008)	0.007 (0.002-0.015)	0.013 (0.006-0.025)	0.022 (0.009-0.043)
Guyana	0.014 (0.003-0.039)	0.023 (0.008-0.050)	0.037 (0.017-0.069)	0.064 (0.032-0.108)	0.090 (0.042-0.156)
Haiti	0.004 (0.001-0.013)	0.008 (0.002-0.018)	0.014 (0.006-0.027)	0.025 (0.012-0.045)	0.040 (0.016-0.078)
Honduras	0.006 (0.001-0.021)	0.012 (0.003-0.027)	0.022 (0.009-0.040)	0.038 (0.020-0.062)	0.059 (0.030-0.102)
Hungary	0.029 (0.009-0.066)	0.037 (0.018-0.063)	0.040 (0.021-0.066)	0.045 (0.024-0.074)	0.051 (0.022-0.094)
Iceland	0.015 (0.005-0.035)	0.027 (0.013-0.048)	0.033 (0.017-0.055)	0.042 (0.022-0.069)	0.049 (0.023-0.089)
India	0.000 (0.000-0.001)	0.001 (0.000-0.002)	0.002 (0.001-0.003)	0.004 (0.003-0.006)	0.008 (0.004-0.014)
Indonesia	0.000 (0.000-0.001)	0.001 (0.000-0.001)	0.002 (0.001-0.004)	0.005 (0.003-0.009)	0.012 (0.006-0.024)
Iran	0.018 (0.005-0.044)	0.026 (0.013-0.046)	0.035 (0.023-0.051)	0.051 (0.039-0.065)	0.070 (0.044-0.102)
Iraq	0.028 (0.007-0.071)	0.040 (0.017-0.077)	0.047 (0.023-0.081)	0.063 (0.034-0.102)	0.085 (0.043-0.141)
Ireland	0.006 (0.002-0.016)	0.014 (0.006-0.026)	0.026 (0.013-0.043)	0.046 (0.026-0.071)	0.063 (0.032-0.103)
Israel	0.031 (0.009-0.071)	0.040 (0.019-0.071)	0.050 (0.028-0.080)	0.058 (0.034-0.089)	0.066 (0.032-0.113)
Italy	0.017 (0.006-0.037)	0.023 (0.014-0.036)	0.031 (0.021-0.043)	0.042 (0.028-0.057)	0.052 (0.028-0.084)
Jamaica	0.020 (0.005-0.049)	0.029 (0.014-0.053)	0.047 (0.027-0.072)	0.069 (0.043-0.100)	0.093 (0.050-0.147)
Japan	0.001 (0.000-0.002)	0.001 (0.001-0.002)	0.002 (0.001-0.003)	0.003 (0.002-0.004)	0.003 (0.002-0.006)
Jordan	0.028 (0.008-0.068)	0.052 (0.026-0.087)	0.071 (0.045-0.105)	0.093 (0.064-0.127)	0.113 (0.070-0.164)
Kazakhstan	0.015 (0.002-0.045)	0.024 (0.008-0.052)	0.033 (0.015-0.061)	0.046 (0.022-0.080)	0.065 (0.026-0.121)
Kenya	0.001 (0.000-0.003)	0.002 (0.001-0.005)	0.005 (0.002-0.010)	0.010 (0.004-0.019)	0.021 (0.008-0.042)
Kiribati	0.068 (0.023-0.140)	0.082 (0.038-0.143)	0.101 (0.053-0.161)	0.126 (0.071-0.192)	0.141 (0.071-0.225)
Kuwait	0.076 (0.025-0.162)	0.082 (0.045-0.134)	0.093 (0.060-0.134)	0.111 (0.076-0.152)	0.127 (0.077-0.186)
Kyrgyzstan	0.008 (0.001-0.026)	0.013 (0.004-0.030)	0.019 (0.008-0.036)	0.027 (0.013-0.048)	0.043 (0.019-0.079)
Lao PDR	0.000 (0.000-0.000)	0.000 (0.000-0.001)	0.001 (0.000-0.002)	0.002 (0.001-0.004)	0.005 (0.002-0.012)
Latvia	0.034 (0.008-0.086)	0.045 (0.018-0.087)	0.049 (0.023-0.087)	0.055 (0.028-0.092)	0.066 (0.029-0.119)
Lebanon	0.027 (0.008-0.064)	0.040 (0.017-0.075)	0.052 (0.026-0.086)	0.070 (0.037-0.112)	0.090 (0.047-0.142)
Lesotho	0.008 (0.001-0.028)	0.014 (0.004-0.034)	0.025 (0.011-0.051)	0.042 (0.021-0.072)	0.061 (0.028-0.107)
Liberia	0.003 (0.000-0.009)	0.005 (0.001-0.013)	0.006 (0.002-0.014)	0.012 (0.005-0.022)	0.023 (0.010-0.044)
Libya	0.043 (0.010-0.108)	0.060 (0.028-0.106)	0.069 (0.038-0.109)	0.083 (0.049-0.124)	0.099 (0.055-0.156)
Lithuania	0.032 (0.009-0.076)	0.045 (0.021-0.079)	0.051 (0.028-0.082)	0.059 (0.034-0.093)	0.075 (0.036-0.129)
Luxembourg	0.013 (0.003-0.033)	0.020 (0.007-0.039)	0.030 (0.012-0.058)	0.040 (0.019-0.069)	0.049 (0.021-0.088)
Macedonia (TFYR)	0.019 (0.004-0.051)	0.025 (0.010-0.052)	0.031 (0.013-0.058)	0.035 (0.016-0.064)	0.042 (0.017-0.082)
Madagascar	0.001 (0.000-0.004)	0.002 (0.000-0.004)	0.003 (0.001-0.007)	0.006 (0.003-0.012)	0.013 (0.005-0.027)
Malawi	0.001 (0.000-0.003)	0.001 (0.000-0.003)	0.003 (0.001-0.006)	0.006 (0.003-0.012)	0.014 (0.005-0.028)
Malaysia	0.001 (0.000-0.004)	0.003 (0.001-0.007)	0.008 (0.004-0.016)	0.019 (0.011-0.031)	0.033 (0.016-0.058)
Maldives	0.000 (0.000-0.001)	0.001 (0.000-0.003)	0.004 (0.001-0.009)	0.011 (0.005-0.023)	0.021 (0.008-0.042)
Mali	0.001 (0.000-0.004)	0.003 (0.001-0.006)	0.005 (0.002-0.011)	0.011 (0.005-0.020)	0.021 (0.009-0.040)
Malta	0.028 (0.006-0.074)	0.042 (0.018-0.078)	0.052 (0.026-0.090)	0.067 (0.035-0.109)	0.077 (0.034-0.135)
Marshall Islands	0.092 (0.032-0.183)	0.112 (0.056-0.188)	0.133 (0.077-0.201)	0.144 (0.086-0.212)	0.154 (0.080-0.239)
Mauritania	0.002 (0.000-0.008)	0.005 (0.001-0.013)	0.011 (0.004-0.023)	0.020 (0.009-0.036)	0.032 (0.013-0.062)
Mauritius	0.005 (0.001-0.015)	0.010 (0.003-0.020)	0.019 (0.009-0.035)	0.036 (0.018-0.062)	0.060 (0.026-0.109)
Mexico	0.019 (0.004-0.049)	0.031 (0.014-0.056)	0.048 (0.030-0.069)	0.068 (0.049-0.091)	0.091 (0.055-0.134)
Micronesia (Federated States of)	0.071 (0.024-0.145)	0.089 (0.044-0.152)	0.109 (0.063-0.166)	0.128 (0.082-0.183)	0.146 (0.086-0.216)
Moldova	0.017 (0.003-0.047)	0.024 (0.008-0.051)	0.028 (0.012-0.053)	0.032 (0.015-0.060)	0.044 (0.019-0.084)
Mongolia	0.008 (0.001-0.027)	0.013 (0.004-0.032)	0.019 (0.008-0.037)	0.027 (0.013-0.046)	0.041 (0.018-0.074)
Montenegro	0.017 (0.003-0.052)	0.027 (0.008-0.060)	0.032 (0.013-0.062)	0.039 (0.017-0.070)	0.046 (0.017-0.087)
Morocco	0.011 (0.002-0.032)	0.019 (0.007-0.039)	0.029 (0.014-0.051)	0.044 (0.023-0.071)	0.062 (0.030-0.108)
Mozambique	0.001 (0.000-0.002)	0.001 (0.000-0.004)	0.003 (0.001-0.006)	0.007 (0.003-0.013)	0.013 (0.005-0.028)
Myanmar	0.000 (0.000-0.001)	0.000 (0.000-0.001)	0.001 (0.000-0.002)	0.003 (0.001-0.006)	0.009 (0.003-0.018)
Namibia	0.013 (0.002-0.045)	0.019 (0.006-0.044)	0.029 (0.013-0.052)	0.045 (0.025-0.072)	0.061 (0.031-0.102)
Nauru	0.156 (0.084-0.245)	0.163 (0.107-0.225)	0.160 (0.110-0.219)	0.152 (0.098-0.214)	0.153 (0.079-0.245)
Nepal	0.000 (0.000-0.001)	0.001 (0.000-0.002)	0.002 (0.001-0.004)	0.004 (0.002-0.007)	0.007 (0.003-0.016)
Netherlands	0.010 (0.003-0.024)	0.015 (0.008-0.026)	0.024 (0.014-0.036)	0.035 (0.023-0.051)	0.043 (0.023-0.068)
New Zealand	0.018 (0.007-0.038)	0.029 (0.016-0.047)	0.044 (0.027-0.065)	0.065 (0.046-0.089)	0.085 (0.056-0.119)
Nicaragua	0.013 (0.002-0.037)	0.020 (0.007-0.041)	0.028 (0.014-0.050)	0.045 (0.026-0.071)	0.064 (0.032-0.110)
Niger	0.001 (0.000-0.004)	0.002 (0.000-0.005)	0.004 (0.001-0.008)	0.007 (0.003-0.014)	0.013 (0.005-0.026)
Nigeria	0.002 (0.000-0.006)	0.004 (0.001-0.009)	0.008 (0.004-0.013)	0.017 (0.010-0.026)	0.034 (0.018-0.055)
Niue	0.077 (0.024-0.168)	0.109 (0.051-0.187)	0.135 (0.077-0.210)	0.158 (0.101-0.228)	0.179 (0.108-0.263)
North Korea	0.001 (0.000-0.005)	0.002 (0.000-0.005)	0.002 (0.001-0.006)	0.002 (0.000-0.007)	0.004 (0.000-0.012)
Norway	0.012 (0.004-0.026)	0.022 (0.012-0.037)	0.034 (0.021-0.051)	0.048 (0.031-0.069)	0.055 (0.029-0.091)
Occupied Palestinian Territory	0.028 (0.008-0.068)	0.040 (0.018-0.075)	0.053 (0.029-0.086)	0.076 (0.045-0.114)	0.095 (0.048-0.154)
Oman	0.015 (0.003-0.039)	0.028 (0.012-0.054)	0.046 (0.025-0.072)	0.063 (0.037-0.094)	0.082 (0.043-0.132)
Pakistan	0.001 (0.000-0.004)	0.003 (0.001-0.006)	0.005 (0.002-0.010)	0.009 (0.004-0.017)	0.015 (0.006-0.030)
Palau	0.129 (0.051-0.238)	0.138 (0.073-0.217)	0.151 (0.090-0.225)	0.163 (0.100-0.233)	0.169 (0.095-0.258)
Panama	0.014 (0.002-0.038)	0.023 (0.007-0.049)	0.036 (0.016-0.064)	0.055 (0.031-0.087)	0.081 (0.043-0.131)
Papua New Guinea	0.015 (0.002-0.051)	0.021 (0.005-0.054)	0.032 (0.011-0.066)	0.043 (0.018-0.081)	0.061 (0.023-0.117)

Paraguay	0.006 (0.001-0.020)	0.012 (0.003-0.029)	0.024 (0.008-0.048)	0.039 (0.016-0.072)	0.058 (0.023-0.110)
Peru	0.010 (0.001-0.029)	0.016 (0.005-0.034)	0.023 (0.011-0.040)	0.035 (0.023-0.052)	0.055 (0.033-0.083)
Philippines	0.000 (0.000-0.001)	0.001 (0.000-0.002)	0.003 (0.001-0.005)	0.006 (0.003-0.010)	0.011 (0.004-0.023)
Poland	0.031 (0.010-0.068)	0.038 (0.021-0.064)	0.044 (0.027-0.066)	0.052 (0.034-0.075)	0.061 (0.032-0.099)
Portugal	0.008 (0.002-0.023)	0.013 (0.005-0.027)	0.022 (0.011-0.039)	0.034 (0.019-0.055)	0.044 (0.021-0.077)
Puerto Rico	0.032 (0.008-0.074)	0.054 (0.024-0.096)	0.079 (0.043-0.126)	0.101 (0.063-0.151)	0.121 (0.069-0.187)
Qatar	0.047 (0.014-0.105)	0.078 (0.036-0.137)	0.086 (0.049-0.132)	0.106 (0.066-0.155)	0.122 (0.071-0.186)
Romania	0.019 (0.004-0.049)	0.028 (0.011-0.056)	0.034 (0.016-0.059)	0.040 (0.019-0.069)	0.050 (0.021-0.092)
Russian Federation	0.038 (0.014-0.079)	0.049 (0.031-0.073)	0.055 (0.041-0.071)	0.062 (0.043-0.082)	0.072 (0.039-0.115)
Rwanda	0.000 (0.000-0.001)	0.000 (0.000-0.002)	0.001 (0.000-0.003)	0.003 (0.001-0.007)	0.008 (0.003-0.018)
Saint Kitts and Nevis	0.017 (0.004-0.045)	0.032 (0.012-0.066)	0.050 (0.023-0.088)	0.075 (0.039-0.123)	0.101 (0.047-0.174)
Saint Lucia	0.011 (0.002-0.029)	0.019 (0.007-0.038)	0.036 (0.018-0.061)	0.059 (0.032-0.096)	0.090 (0.042-0.157)
Saint Vincent and the Grenadines	0.012 (0.002-0.033)	0.022 (0.007-0.046)	0.039 (0.017-0.071)	0.061 (0.031-0.101)	0.083 (0.038-0.144)
Samoa	0.081 (0.030-0.160)	0.102 (0.053-0.165)	0.130 (0.080-0.193)	0.157 (0.107-0.217)	0.175 (0.108-0.249)
Sao Tome and Principe	0.004 (0.000-0.012)	0.007 (0.002-0.016)	0.013 (0.005-0.028)	0.022 (0.010-0.040)	0.034 (0.014-0.064)
Saudi Arabia	0.039 (0.011-0.090)	0.058 (0.032-0.094)	0.073 (0.050-0.101)	0.090 (0.063-0.122)	0.112 (0.070-0.163)
Senegal	0.003 (0.000-0.009)	0.005 (0.002-0.012)	0.009 (0.004-0.018)	0.017 (0.008-0.029)	0.028 (0.012-0.053)
Serbia	0.018 (0.004-0.045)	0.024 (0.011-0.046)	0.031 (0.017-0.050)	0.036 (0.021-0.057)	0.044 (0.021-0.075)
Seychelles	0.010 (0.002-0.029)	0.021 (0.008-0.043)	0.036 (0.017-0.063)	0.059 (0.031-0.095)	0.084 (0.042-0.144)
Sierra Leone	0.002 (0.000-0.008)	0.004 (0.001-0.010)	0.007 (0.003-0.015)	0.013 (0.006-0.024)	0.025 (0.011-0.046)
Singapore	0.002 (0.000-0.006)	0.004 (0.001-0.008)	0.006 (0.003-0.011)	0.008 (0.004-0.014)	0.011 (0.005-0.022)
Slovakia	0.016 (0.004-0.043)	0.025 (0.010-0.047)	0.032 (0.017-0.054)	0.041 (0.023-0.065)	0.053 (0.025-0.092)
Slovenia	0.018 (0.004-0.051)	0.027 (0.010-0.054)	0.035 (0.016-0.063)	0.044 (0.022-0.076)	0.053 (0.023-0.096)
Solomon Islands	0.013 (0.001-0.043)	0.020 (0.005-0.052)	0.031 (0.011-0.064)	0.041 (0.018-0.075)	0.055 (0.022-0.104)
Somalia	0.001 (0.000-0.005)	0.002 (0.000-0.006)	0.004 (0.001-0.009)	0.007 (0.002-0.015)	0.012 (0.003-0.029)
South Africa	0.046 (0.013-0.113)	0.058 (0.028-0.105)	0.071 (0.045-0.105)	0.088 (0.063-0.116)	0.106 (0.070-0.148)
South Korea	0.000 (0.000-0.000)	0.001 (0.000-0.001)	0.002 (0.001-0.004)	0.004 (0.002-0.006)	0.007 (0.003-0.014)
Spain	0.016 (0.005-0.036)	0.024 (0.013-0.039)	0.034 (0.023-0.048)	0.047 (0.033-0.064)	0.057 (0.031-0.090)
Sri Lanka	0.000 (0.000-0.001)	0.001 (0.000-0.002)	0.002 (0.001-0.005)	0.007 (0.003-0.012)	0.016 (0.006-0.034)
Sudan	0.002 (0.000-0.006)	0.003 (0.001-0.009)	0.007 (0.002-0.016)	0.014 (0.006-0.029)	0.025 (0.009-0.053)
Suriname	0.024 (0.005-0.061)	0.041 (0.015-0.080)	0.050 (0.023-0.089)	0.068 (0.035-0.113)	0.090 (0.042-0.152)
Swaziland	0.010 (0.001-0.034)	0.019 (0.005-0.045)	0.033 (0.013-0.064)	0.050 (0.024-0.088)	0.069 (0.030-0.124)
Sweden	0.012 (0.004-0.027)	0.018 (0.009-0.029)	0.024 (0.015-0.036)	0.033 (0.022-0.049)	0.041 (0.021-0.068)
Switzerland	0.010 (0.002-0.026)	0.016 (0.007-0.031)	0.026 (0.013-0.043)	0.033 (0.018-0.053)	0.039 (0.019-0.069)
Syrian Arab Republic	0.016 (0.003-0.044)	0.028 (0.011-0.056)	0.040 (0.019-0.069)	0.057 (0.030-0.093)	0.074 (0.036-0.129)
Taiwan	0.001 (0.000-0.003)	0.002 (0.001-0.006)	0.005 (0.002-0.009)	0.008 (0.003-0.014)	0.011 (0.003-0.025)
Tajikistan	0.010 (0.001-0.032)	0.013 (0.003-0.033)	0.016 (0.006-0.034)	0.022 (0.009-0.042)	0.037 (0.014-0.073)
Tanzania	0.001 (0.000-0.004)	0.002 (0.001-0.005)	0.005 (0.002-0.009)	0.010 (0.005-0.018)	0.021 (0.010-0.038)
Thailand	0.001 (0.000-0.003)	0.002 (0.001-0.005)	0.005 (0.002-0.010)	0.013 (0.007-0.022)	0.025 (0.011-0.048)
Timor-Leste	0.000 (0.000-0.000)	0.000 (0.000-0.001)	0.001 (0.000-0.001)	0.001 (0.000-0.003)	0.004 (0.001-0.009)
Togo	0.002 (0.000-0.006)	0.003 (0.001-0.008)	0.007 (0.003-0.014)	0.014 (0.006-0.025)	0.024 (0.011-0.045)
Tokelau	0.075 (0.015-0.189)	0.098 (0.036-0.191)	0.124 (0.063-0.206)	0.151 (0.089-0.225)	0.174 (0.093-0.267)
Tonga	0.088 (0.030-0.177)	0.111 (0.055-0.184)	0.136 (0.080-0.203)	0.160 (0.105-0.224)	0.176 (0.109-0.254)
Trinidad and Tobago	0.016 (0.002-0.052)	0.027 (0.008-0.062)	0.040 (0.018-0.075)	0.069 (0.034-0.119)	0.105 (0.041-0.192)
Tunisia	0.018 (0.004-0.045)	0.030 (0.013-0.056)	0.044 (0.024-0.071)	0.063 (0.039-0.094)	0.084 (0.046-0.132)
Turkey	0.021 (0.006-0.050)	0.035 (0.019-0.058)	0.054 (0.037-0.075)	0.076 (0.057-0.098)	0.098 (0.066-0.139)
Turkmenistan	0.012 (0.002-0.035)	0.017 (0.005-0.038)	0.026 (0.010-0.049)	0.037 (0.018-0.065)	0.059 (0.026-0.108)
Tuvalu	0.064 (0.021-0.135)	0.089 (0.042-0.156)	0.121 (0.067-0.191)	0.141 (0.083-0.212)	0.155 (0.082-0.242)
Uganda	0.000 (0.000-0.002)	0.001 (0.000-0.002)	0.002 (0.000-0.004)	0.005 (0.002-0.010)	0.012 (0.005-0.025)
Ukraine	0.029 (0.007-0.075)	0.038 (0.014-0.075)	0.043 (0.019-0.079)	0.046 (0.021-0.082)	0.056 (0.022-0.106)
United Arab Emirates	0.075 (0.019-0.172)	0.078 (0.036-0.135)	0.086 (0.050-0.130)	0.107 (0.068-0.154)	0.114 (0.063-0.179)
United Kingdom	0.016 (0.006-0.031)	0.025 (0.016-0.037)	0.039 (0.030-0.050)	0.058 (0.047-0.072)	0.075 (0.053-0.100)
United States of America	0.027 (0.013-0.046)	0.040 (0.026-0.057)	0.059 (0.043-0.079)	0.081 (0.061-0.102)	0.097 (0.064-0.135)
Uruguay	0.027 (0.006-0.070)	0.037 (0.013-0.075)	0.050 (0.024-0.088)	0.067 (0.037-0.106)	0.088 (0.045-0.144)
Uzbekistan	0.009 (0.001-0.029)	0.013 (0.004-0.032)	0.020 (0.008-0.041)	0.030 (0.013-0.054)	0.047 (0.019-0.091)
Vanuatu	0.019 (0.003-0.061)	0.028 (0.008-0.066)	0.040 (0.017-0.076)	0.054 (0.027-0.092)	0.071 (0.032-0.125)
Venezuela	0.022 (0.004-0.057)	0.031 (0.011-0.061)	0.043 (0.022-0.072)	0.059 (0.034-0.088)	0.078 (0.041-0.126)
Viet Nam	0.000 (0.000-0.000)	0.000 (0.000-0.000)	0.000 (0.000-0.001)	0.001 (0.001-0.002)	0.004 (0.001-0.008)
Yemen	0.006 (0.001-0.019)	0.010 (0.003-0.022)	0.017 (0.007-0.032)	0.028 (0.013-0.048)	0.040 (0.017-0.073)
Zambia	0.003 (0.001-0.011)	0.006 (0.002-0.013)	0.009 (0.004-0.017)	0.015 (0.008-0.027)	0.029 (0.013-0.052)
Zimbabwe	0.013 (0.002-0.039)	0.020 (0.008-0.043)	0.031 (0.015-0.052)	0.042 (0.023-0.067)	0.051 (0.023-0.090)

Men, BMI ≥ 40 kg/m^2

Country	Year				
	1975	1985	1995	2005	2014
Afghanistan	0.000 (0.000-0.000)	0.000 (0.000-0.000)	0.000 (0.000-0.000)	0.000 (0.000-0.000)	0.000 (0.000-0.001)
Albania	0.000 (0.000-0.002)	0.001 (0.000-0.002)	0.001 (0.000-0.003)	0.002 (0.000-0.005)	0.003 (0.000-0.010)
Algeria	0.000 (0.000-0.002)	0.001 (0.000-0.002)	0.001 (0.000-0.004)	0.003 (0.001-0.007)	0.005 (0.001-0.015)
American Samoa	0.027 (0.007-0.068)	0.044 (0.018-0.084)	0.064 (0.031-0.114)	0.078 (0.038-0.137)	0.089 (0.030-0.178)
Andorra	0.001 (0.000-0.003)	0.002 (0.000-0.006)	0.003 (0.001-0.011)	0.008 (0.002-0.023)	0.013 (0.002-0.040)
Angola	0.000 (0.000-0.000)	0.000 (0.000-0.000)	0.000 (0.000-0.000)	0.000 (0.000-0.002)	0.002 (0.000-0.010)
Antigua and Barbuda	0.000 (0.000-0.001)	0.001 (0.000-0.002)	0.002 (0.000-0.008)	0.009 (0.002-0.026)	0.032 (0.005-0.091)
Argentina	0.001 (0.000-0.004)	0.001 (0.000-0.005)	0.003 (0.001-0.008)	0.006 (0.002-0.013)	0.013 (0.003-0.032)
Armenia	0.002 (0.000-0.016)	0.003 (0.000-0.011)	0.003 (0.000-0.009)	0.004 (0.001-0.011)	0.007 (0.001-0.023)
Australia	0.001 (0.000-0.003)	0.002 (0.001-0.003)	0.004 (0.002-0.007)	0.011 (0.006-0.018)	0.022 (0.010-0.043)
Austria	0.000 (0.000-0.001)	0.001 (0.000-0.002)	0.002 (0.001-0.005)	0.005 (0.002-0.012)	0.010 (0.002-0.027)
Azerbaijan	0.001 (0.000-0.007)	0.001 (0.000-0.006)	0.002 (0.000-0.006)	0.003 (0.001-0.008)	0.009 (0.001-0.027)
Bahamas	0.001 (0.000-0.003)	0.001 (0.000-0.005)	0.004 (0.001-0.013)	0.013 (0.003-0.033)	0.029 (0.006-0.080)
Bahrain	0.002 (0.000-0.009)	0.002 (0.000-0.007)	0.004 (0.001-0.010)	0.008 (0.002-0.017)	0.013 (0.003-0.034)
Bangladesh	0.000 (0.000-0.000)	0.000 (0.000-0.000)	0.000 (0.000-0.000)	0.000 (0.000-0.000)	0.000 (0.000-0.001)
Barbados	0.000 (0.000-0.001)	0.001 (0.000-0.002)	0.002 (0.000-0.005)	0.008 (0.002-0.021)	0.027 (0.006-0.074)
Belarus	0.001 (0.000-0.004)	0.001 (0.000-0.005)	0.002 (0.000-0.008)	0.004 (0.001-0.014)	0.009 (0.001-0.031)
Belgium	0.000 (0.000-0.002)	0.001 (0.000-0.002)	0.001 (0.001-0.003)	0.003 (0.001-0.006)	0.005 (0.001-0.014)
Belize	0.000 (0.000-0.002)	0.000 (0.000-0.002)	0.001 (0.000-0.004)	0.005 (0.001-0.013)	0.015 (0.003-0.044)
Benin	0.000 (0.000-0.000)	0.000 (0.000-0.000)	0.000 (0.000-0.001)	0.001 (0.000-0.002)	0.003 (0.000-0.010)
Bermuda	0.002 (0.000-0.010)	0.003 (0.000-0.012)	0.007 (0.001-0.022)	0.019 (0.004-0.047)	0.048 (0.011-0.118)
Bhutan	0.000 (0.000-0.000)	0.000 (0.000-0.000)	0.000 (0.000-0.000)	0.000 (0.000-0.001)	0.001 (0.000-0.003)
Bolivia	0.000 (0.000-0.001)	0.000 (0.000-0.001)	0.000 (0.000-0.002)	0.001 (0.000-0.004)	0.004 (0.000-0.015)
Bosnia and Herzegovina	0.000 (0.000-0.002)	0.000 (0.000-0.002)	0.001 (0.000-0.002)	0.002 (0.000-0.005)	0.003 (0.000-0.008)
Botswana	0.000 (0.000-0.000)	0.000 (0.000-0.000)	0.001 (0.000-0.002)	0.003 (0.000-0.008)	0.009 (0.001-0.028)
Brazil	0.000 (0.000-0.001)	0.000 (0.000-0.001)	0.001 (0.000-0.002)	0.003 (0.001-0.005)	0.006 (0.002-0.015)
Brunei Darussalam	0.000 (0.000-0.001)	0.000 (0.000-0.002)	0.001 (0.000-0.003)	0.004 (0.001-0.013)	0.015 (0.002-0.050)
Bulgaria	0.001 (0.000-0.004)	0.001 (0.000-0.005)	0.002 (0.000-0.007)	0.004 (0.001-0.010)	0.006 (0.001-0.020)
Burkina Faso	0.000 (0.000-0.000)	0.000 (0.000-0.000)	0.000 (0.000-0.000)	0.000 (0.000-0.001)	0.002 (0.000-0.006)
Burundi	0.000 (0.000-0.000)	0.000 (0.000-0.000)	0.000 (0.000-0.000)	0.000 (0.000-0.000)	0.000 (0.000-0.001)
Cabo Verde	0.000 (0.000-0.000)	0.000 (0.000-0.000)	0.000 (0.000-0.001)	0.002 (0.000-0.005)	0.007 (0.001-0.023)
Cambodia	0.000 (0.000-0.000)	0.000 (0.000-0.000)	0.000 (0.000-0.000)	0.000 (0.000-0.000)	0.000 (0.000-0.002)
Cameroon	0.000 (0.000-0.000)	0.000 (0.000-0.000)	0.000 (0.000-0.001)	0.001 (0.000-0.003)	0.005 (0.001-0.014)
Canada	0.001 (0.000-0.002)	0.002 (0.001-0.004)	0.006 (0.003-0.012)	0.014 (0.007-0.024)	0.023 (0.007-0.047)
Central African Republic	0.000 (0.000-0.000)	0.000 (0.000-0.000)	0.000 (0.000-0.000)	0.000 (0.000-0.001)	0.000 (0.000-0.002)
Chad	0.000 (0.000-0.000)	0.000 (0.000-0.000)	0.000 (0.000-0.001)	0.001 (0.000-0.003)	0.005 (0.001-0.016)
Chile	0.001 (0.000-0.003)	0.001 (0.000-0.003)	0.002 (0.001-0.005)	0.005 (0.002-0.011)	0.012 (0.003-0.028)
China	0.000 (0.000-0.000)	0.000 (0.000-0.000)	0.000 (0.000-0.000)	0.000 (0.000-0.000)	0.001 (0.000-0.004)
China (Hong Kong SAR)	0.000 (0.000-0.001)	0.000 (0.000-0.001)	0.000 (0.000-0.001)	0.001 (0.000-0.003)	0.003 (0.000-0.012)
Colombia	0.000 (0.000-0.001)	0.000 (0.000-0.002)	0.001 (0.000-0.003)	0.003 (0.001-0.005)	0.006 (0.002-0.016)
Comoros	0.000 (0.000-0.000)	0.000 (0.000-0.000)	0.000 (0.000-0.000)	0.000 (0.000-0.001)	0.001 (0.000-0.003)
Congo	0.000 (0.000-0.000)	0.000 (0.000-0.000)	0.000 (0.000-0.001)	0.000 (0.000-0.002)	0.002 (0.000-0.008)
Cook Islands	0.012 (0.001-0.042)	0.021 (0.005-0.052)	0.042 (0.014-0.088)	0.068 (0.028-0.125)	0.092 (0.033-0.177)
Costa Rica	0.000 (0.000-0.001)	0.000 (0.000-0.002)	0.001 (0.000-0.005)	0.005 (0.001-0.011)	0.012 (0.003-0.032)
Cote d'Ivoire	0.000 (0.000-0.000)	0.000 (0.000-0.001)	0.000 (0.000-0.001)	0.001 (0.000-0.004)	0.005 (0.001-0.016)
Croatia	0.001 (0.000-0.003)	0.001 (0.000-0.004)	0.002 (0.000-0.007)	0.004 (0.001-0.012)	0.007 (0.001-0.023)
Cuba	0.000 (0.000-0.002)	0.001 (0.000-0.003)	0.002 (0.000-0.006)	0.006 (0.002-0.014)	0.019 (0.005-0.045)
Cyprus	0.000 (0.000-0.000)	0.000 (0.000-0.002)	0.002 (0.000-0.007)	0.007 (0.002-0.019)	0.014 (0.002-0.042)
Czech Republic	0.002 (0.000-0.007)	0.003 (0.001-0.007)	0.004 (0.001-0.008)	0.006 (0.002-0.012)	0.008 (0.002-0.023)
Denmark	0.000 (0.000-0.001)	0.001 (0.000-0.002)	0.002 (0.001-0.005)	0.005 (0.002-0.010)	0.008 (0.002-0.019)
Djibouti	0.000 (0.000-0.001)	0.000 (0.000-0.001)	0.000 (0.000-0.002)	0.001 (0.000-0.002)	0.002 (0.000-0.007)
Dominica	0.000 (0.000-0.001)	0.000 (0.000-0.001)	0.001 (0.000-0.004)	0.004 (0.001-0.012)	0.013 (0.002-0.039)
Dominican Republic	0.000 (0.000-0.000)	0.000 (0.000-0.001)	0.001 (0.000-0.002)	0.003 (0.001-0.008)	0.012 (0.003-0.032)
DR Congo	0.000 (0.000-0.000)	0.000 (0.000-0.000)	0.000 (0.000-0.000)	0.000 (0.000-0.000)	0.000 (0.000-0.002)
Ecuador	0.000 (0.000-0.001)	0.000 (0.000-0.001)	0.001 (0.000-0.002)	0.002 (0.000-0.005)	0.005 (0.001-0.016)
Egypt	0.001 (0.000-0.007)	0.002 (0.000-0.007)	0.005 (0.002-0.011)	0.010 (0.004-0.019)	0.018 (0.005-0.041)
El Salvador	0.000 (0.000-0.002)	0.000 (0.000-0.002)	0.001 (0.000-0.004)	0.004 (0.001-0.011)	0.009 (0.001-0.027)
Equatorial Guinea	0.000 (0.000-0.000)	0.000 (0.000-0.000)	0.000 (0.000-0.000)	0.002 (0.000-0.007)	0.009 (0.000-0.039)
Eritrea	0.000 (0.000-0.000)	0.000 (0.000-0.000)	0.000 (0.000-0.000)	0.000 (0.000-0.000)	0.000 (0.000-0.002)
Estonia	0.001 (0.000-0.006)	0.002 (0.000-0.006)	0.003 (0.001-0.007)	0.005 (0.003-0.010)	0.009 (0.003-0.020)
Ethiopia	0.000 (0.000-0.000)	0.000 (0.000-0.000)	0.000 (0.000-0.000)	0.000 (0.000-0.000)	0.000 (0.000-0.001)
Fiji	0.002 (0.000-0.016)	0.003 (0.000-0.016)	0.006 (0.001-0.018)	0.010 (0.003-0.025)	0.018 (0.004-0.046)
Finland	0.000 (0.000-0.001)	0.001 (0.000-0.002)	0.002 (0.001-0.005)	0.005 (0.003-0.009)	0.009 (0.003-0.019)
France	0.000 (0.000-0.001)	0.000 (0.000-0.001)	0.001 (0.000-0.003)	0.004 (0.001-0.008)	0.008 (0.002-0.021)
French Polynesia	0.012 (0.001-0.044)	0.021 (0.004-0.059)	0.038 (0.010-0.088)	0.059 (0.022-0.112)	0.082 (0.030-0.154)

Gabon	0.000 (0.000-0.000)	0.000 (0.000-0.001)	0.001 (0.000-0.003)	0.002 (0.000-0.007)	0.005 (0.000-0.020)
Gambia	0.000 (0.000-0.000)	0.000 (0.000-0.000)	0.000 (0.000-0.001)	0.001 (0.000-0.003)	0.003 (0.000-0.011)
Georgia	0.002 (0.000-0.011)	0.003 (0.000-0.011)	0.003 (0.000-0.010)	0.004 (0.001-0.012)	0.009 (0.001-0.026)
Germany	0.000 (0.000-0.001)	0.001 (0.000-0.001)	0.002 (0.001-0.004)	0.005 (0.003-0.009)	0.009 (0.003-0.021)
Ghana	0.000 (0.000-0.000)	0.000 (0.000-0.001)	0.000 (0.000-0.002)	0.002 (0.001-0.006)	0.010 (0.002-0.029)
Greece	0.000 (0.000-0.001)	0.001 (0.000-0.002)	0.002 (0.000-0.006)	0.005 (0.001-0.013)	0.010 (0.002-0.028)
Greenland	0.000 (0.000-0.002)	0.001 (0.000-0.003)	0.003 (0.001-0.008)	0.006 (0.002-0.015)	0.011 (0.002-0.030)
Grenada	0.000 (0.000-0.000)	0.000 (0.000-0.001)	0.001 (0.000-0.003)	0.004 (0.001-0.010)	0.013 (0.002-0.037)
Guatemala	0.000 (0.000-0.001)	0.000 (0.000-0.001)	0.001 (0.000-0.002)	0.002 (0.000-0.006)	0.006 (0.001-0.019)
Guinea	0.000 (0.000-0.000)	0.000 (0.000-0.000)	0.000 (0.000-0.001)	0.001 (0.000-0.002)	0.002 (0.000-0.007)
Guinea Bissau	0.000 (0.000-0.000)	0.000 (0.000-0.000)	0.000 (0.000-0.001)	0.001 (0.000-0.002)	0.003 (0.000-0.009)
Guyana	0.000 (0.000-0.001)	0.000 (0.000-0.002)	0.001 (0.000-0.004)	0.006 (0.001-0.015)	0.019 (0.004-0.053)
Haiti	0.000 (0.000-0.000)	0.000 (0.000-0.000)	0.000 (0.000-0.001)	0.001 (0.000-0.004)	0.004 (0.000-0.014)
Honduras	0.000 (0.000-0.001)	0.000 (0.000-0.001)	0.001 (0.000-0.002)	0.002 (0.000-0.005)	0.005 (0.001-0.017)
Hungary	0.002 (0.000-0.008)	0.003 (0.001-0.008)	0.005 (0.002-0.011)	0.007 (0.002-0.016)	0.010 (0.002-0.028)
Iceland	0.001 (0.000-0.002)	0.001 (0.000-0.004)	0.003 (0.001-0.007)	0.006 (0.002-0.014)	0.010 (0.002-0.028)
India	0.000 (0.000-0.000)	0.000 (0.000-0.000)	0.000 (0.000-0.000)	0.000 (0.000-0.000)	0.000 (0.000-0.001)
Indonesia	0.000 (0.000-0.000)	0.000 (0.000-0.000)	0.000 (0.000-0.000)	0.000 (0.000-0.001)	0.002 (0.000-0.005)
Iran	0.000 (0.000-0.002)	0.001 (0.000-0.002)	0.001 (0.000-0.002)	0.002 (0.001-0.004)	0.005 (0.002-0.011)
Iraq	0.001 (0.000-0.004)	0.001 (0.000-0.004)	0.001 (0.000-0.004)	0.003 (0.001-0.008)	0.006 (0.001-0.019)
Ireland	0.000 (0.000-0.001)	0.001 (0.000-0.002)	0.002 (0.001-0.006)	0.009 (0.003-0.019)	0.021 (0.006-0.046)
Israel	0.000 (0.000-0.002)	0.001 (0.000-0.003)	0.002 (0.000-0.005)	0.004 (0.001-0.009)	0.008 (0.001-0.022)
Italy	0.000 (0.000-0.000)	0.000 (0.000-0.001)	0.001 (0.000-0.002)	0.003 (0.001-0.006)	0.007 (0.002-0.016)
Jamaica	0.000 (0.000-0.001)	0.000 (0.000-0.001)	0.001 (0.000-0.002)	0.003 (0.001-0.008)	0.013 (0.003-0.037)
Japan	0.000 (0.000-0.000)	0.000 (0.000-0.000)	0.000 (0.000-0.000)	0.000 (0.000-0.000)	0.000 (0.000-0.000)
Jordan	0.001 (0.000-0.007)	0.002 (0.000-0.007)	0.005 (0.001-0.011)	0.008 (0.003-0.016)	0.014 (0.004-0.034)
Kazakhstan	0.002 (0.000-0.010)	0.003 (0.000-0.010)	0.004 (0.001-0.013)	0.007 (0.001-0.020)	0.014 (0.002-0.044)
Kenya	0.000 (0.000-0.000)	0.000 (0.000-0.000)	0.000 (0.000-0.000)	0.000 (0.000-0.001)	0.001 (0.000-0.005)
Kiribati	0.004 (0.000-0.016)	0.008 (0.002-0.023)	0.015 (0.004-0.037)	0.026 (0.008-0.058)	0.037 (0.008-0.093)
Kuwait	0.004 (0.000-0.019)	0.006 (0.002-0.015)	0.010 (0.004-0.020)	0.017 (0.008-0.031)	0.028 (0.009-0.059)
Kyrgyzstan	0.001 (0.000-0.005)	0.001 (0.000-0.004)	0.001 (0.000-0.004)	0.002 (0.000-0.006)	0.003 (0.000-0.011)
Lao PDR	0.000 (0.000-0.000)	0.000 (0.000-0.000)	0.000 (0.000-0.000)	0.000 (0.000-0.000)	0.001 (0.000-0.003)
Latvia	0.001 (0.000-0.007)	0.002 (0.000-0.008)	0.004 (0.001-0.010)	0.006 (0.002-0.015)	0.011 (0.002-0.032)
Lebanon	0.001 (0.000-0.005)	0.002 (0.000-0.006)	0.002 (0.000-0.007)	0.004 (0.001-0.012)	0.010 (0.002-0.026)
Lesotho	0.000 (0.000-0.000)	0.000 (0.000-0.000)	0.000 (0.000-0.001)	0.001 (0.000-0.003)	0.004 (0.001-0.011)
Liberia	0.000 (0.000-0.000)	0.000 (0.000-0.001)	0.000 (0.000-0.001)	0.001 (0.000-0.002)	0.003 (0.000-0.010)
Libya	0.001 (0.000-0.007)	0.002 (0.000-0.008)	0.004 (0.001-0.010)	0.006 (0.002-0.015)	0.011 (0.003-0.029)
Lithuania	0.001 (0.000-0.005)	0.002 (0.001-0.006)	0.004 (0.001-0.010)	0.007 (0.003-0.016)	0.015 (0.003-0.039)
Luxembourg	0.000 (0.000-0.002)	0.001 (0.000-0.003)	0.003 (0.000-0.008)	0.007 (0.002-0.016)	0.012 (0.003-0.033)
Macedonia (TFYR)	0.001 (0.000-0.004)	0.001 (0.000-0.004)	0.002 (0.000-0.007)	0.003 (0.001-0.009)	0.004 (0.001-0.014)
Madagascar	0.000 (0.000-0.000)	0.000 (0.000-0.000)	0.000 (0.000-0.000)	0.000 (0.000-0.001)	0.001 (0.000-0.004)
Malawi	0.000 (0.000-0.000)	0.000 (0.000-0.000)	0.000 (0.000-0.000)	0.000 (0.000-0.000)	0.000 (0.000-0.002)
Malaysia	0.000 (0.000-0.000)	0.000 (0.000-0.000)	0.000 (0.000-0.001)	0.002 (0.001-0.005)	0.011 (0.003-0.028)
Maldives	0.000 (0.000-0.000)	0.000 (0.000-0.000)	0.000 (0.000-0.000)	0.000 (0.000-0.002)	0.003 (0.000-0.009)
Mali	0.000 (0.000-0.000)	0.000 (0.000-0.000)	0.000 (0.000-0.001)	0.001 (0.000-0.003)	0.004 (0.000-0.012)
Malta	0.001 (0.000-0.006)	0.002 (0.000-0.006)	0.004 (0.001-0.012)	0.009 (0.002-0.023)	0.016 (0.003-0.046)
Marshall Islands	0.011 (0.001-0.042)	0.017 (0.003-0.047)	0.028 (0.008-0.063)	0.037 (0.012-0.077)	0.048 (0.011-0.112)
Mauritania	0.000 (0.000-0.000)	0.000 (0.000-0.000)	0.000 (0.000-0.001)	0.001 (0.000-0.004)	0.005 (0.001-0.015)
Mauritius	0.000 (0.000-0.000)	0.000 (0.000-0.000)	0.000 (0.000-0.001)	0.001 (0.000-0.004)	0.007 (0.001-0.021)
Mexico	0.001 (0.000-0.003)	0.001 (0.000-0.004)	0.003 (0.001-0.007)	0.008 (0.004-0.013)	0.016 (0.006-0.034)
Micronesia (Federated States of)	0.006 (0.000-0.024)	0.011 (0.003-0.031)	0.021 (0.007-0.046)	0.036 (0.016-0.066)	0.058 (0.022-0.113)
Moldova	0.000 (0.000-0.002)	0.001 (0.000-0.003)	0.001 (0.000-0.004)	0.002 (0.000-0.006)	0.004 (0.001-0.014)
Mongolia	0.001 (0.000-0.006)	0.001 (0.000-0.004)	0.001 (0.000-0.004)	0.002 (0.001-0.005)	0.005 (0.001-0.013)
Montenegro	0.000 (0.000-0.003)	0.001 (0.000-0.004)	0.002 (0.000-0.006)	0.003 (0.001-0.009)	0.005 (0.001-0.016)
Morocco	0.000 (0.000-0.002)	0.000 (0.000-0.002)	0.001 (0.000-0.003)	0.002 (0.000-0.005)	0.004 (0.001-0.014)
Mozambique	0.000 (0.000-0.000)	0.000 (0.000-0.000)	0.000 (0.000-0.000)	0.000 (0.000-0.000)	0.000 (0.000-0.001)
Myanmar	0.000 (0.000-0.000)	0.000 (0.000-0.000)	0.000 (0.000-0.000)	0.000 (0.000-0.000)	0.001 (0.000-0.002)
Namibia	0.000 (0.000-0.000)	0.000 (0.000-0.001)	0.000 (0.000-0.001)	0.002 (0.000-0.005)	0.006 (0.001-0.019)
Nauru	0.051 (0.013-0.114)	0.066 (0.031-0.116)	0.078 (0.041-0.127)	0.075 (0.037-0.127)	0.079 (0.023-0.164)
Nepal	0.000 (0.000-0.000)	0.000 (0.000-0.000)	0.000 (0.000-0.000)	0.000 (0.000-0.000)	0.000 (0.000-0.001)
Netherlands	0.000 (0.000-0.001)	0.000 (0.000-0.001)	0.001 (0.000-0.003)	0.003 (0.001-0.006)	0.005 (0.001-0.013)
New Zealand	0.001 (0.000-0.003)	0.002 (0.001-0.005)	0.005 (0.002-0.011)	0.014 (0.007-0.023)	0.026 (0.012-0.047)
Nicaragua	0.000 (0.000-0.002)	0.001 (0.000-0.003)	0.001 (0.000-0.005)	0.003 (0.001-0.010)	0.008 (0.001-0.025)
Niger	0.000 (0.000-0.000)	0.000 (0.000-0.000)	0.000 (0.000-0.001)	0.001 (0.000-0.002)	0.002 (0.000-0.007)
Nigeria	0.000 (0.000-0.000)	0.000 (0.000-0.000)	0.000 (0.000-0.001)	0.001 (0.000-0.003)	0.008 (0.002-0.020)
Niue	0.004 (0.000-0.020)	0.011 (0.002-0.033)	0.025 (0.006-0.060)	0.048 (0.018-0.093)	0.081 (0.030-0.154)
North Korea	0.000 (0.000-0.000)	0.000 (0.000-0.000)	0.000 (0.000-0.000)	0.000 (0.000-0.000)	0.000 (0.000-0.001)
Norway	0.000 (0.000-0.002)	0.001 (0.000-0.003)	0.004 (0.002-0.008)	0.010 (0.005-0.019)	0.018 (0.005-0.039)

Occupied Palestinian Territory	0.001 (0.000-0.005)	0.001 (0.000-0.005)	0.002 (0.000-0.006)	0.004 (0.001-0.010)	0.008 (0.002-0.022)
Oman	0.000 (0.000-0.002)	0.001 (0.000-0.004)	0.004 (0.001-0.008)	0.008 (0.003-0.017)	0.018 (0.005-0.043)
Pakistan	0.000 (0.000-0.000)	0.000 (0.000-0.000)	0.000 (0.000-0.001)	0.000 (0.000-0.001)	0.001 (0.000-0.004)
Palau	0.014 (0.001-0.055)	0.024 (0.005-0.061)	0.040 (0.013-0.090)	0.057 (0.022-0.109)	0.069 (0.023-0.137)
Panama	0.000 (0.000-0.002)	0.001 (0.000-0.003)	0.002 (0.000-0.006)	0.006 (0.002-0.013)	0.017 (0.004-0.043)
Papua New Guinea	0.001 (0.000-0.007)	0.001 (0.000-0.006)	0.002 (0.000-0.008)	0.004 (0.001-0.012)	0.008 (0.001-0.029)
Paraguay	0.000 (0.000-0.001)	0.000 (0.000-0.001)	0.001 (0.000-0.003)	0.002 (0.000-0.007)	0.006 (0.001-0.020)
Peru	0.000 (0.000-0.001)	0.000 (0.000-0.002)	0.001 (0.000-0.003)	0.002 (0.001-0.004)	0.005 (0.001-0.015)
Philippines	0.000 (0.000-0.000)	0.000 (0.000-0.000)	0.000 (0.000-0.000)	0.000 (0.000-0.001)	0.002 (0.000-0.006)
Poland	0.001 (0.000-0.004)	0.001 (0.000-0.003)	0.002 (0.001-0.004)	0.004 (0.002-0.007)	0.006 (0.002-0.015)
Portugal	0.000 (0.000-0.000)	0.000 (0.000-0.000)	0.001 (0.000-0.002)	0.002 (0.001-0.005)	0.006 (0.001-0.016)
Puerto Rico	0.000 (0.000-0.003)	0.002 (0.000-0.006)	0.006 (0.001-0.017)	0.018 (0.006-0.039)	0.038 (0.010-0.091)
Qatar	0.004 (0.000-0.017)	0.007 (0.001-0.023)	0.011 (0.003-0.026)	0.021 (0.008-0.043)	0.036 (0.012-0.078)
Romania	0.001 (0.000-0.004)	0.001 (0.000-0.005)	0.003 (0.001-0.007)	0.004 (0.001-0.011)	0.007 (0.001-0.021)
Russian Federation	0.000 (0.000-0.002)	0.001 (0.000-0.002)	0.002 (0.001-0.003)	0.003 (0.001-0.005)	0.006 (0.001-0.018)
Rwanda	0.000 (0.000-0.000)	0.000 (0.000-0.000)	0.000 (0.000-0.000)	0.000 (0.000-0.000)	0.000 (0.000-0.001)
Saint Kitts and Nevis	0.000 (0.000-0.001)	0.001 (0.000-0.003)	0.002 (0.000-0.007)	0.010 (0.002-0.025)	0.033 (0.007-0.084)
Saint Lucia	0.000 (0.000-0.000)	0.000 (0.000-0.001)	0.001 (0.000-0.003)	0.004 (0.001-0.012)	0.020 (0.004-0.055)
Saint Vincent and the Grenadines	0.000 (0.000-0.001)	0.000 (0.000-0.001)	0.001 (0.000-0.004)	0.005 (0.001-0.014)	0.015 (0.002-0.045)
Samoa	0.005 (0.000-0.019)	0.010 (0.002-0.025)	0.021 (0.007-0.043)	0.041 (0.019-0.072)	0.064 (0.025-0.122)
Sao Tome and Principe	0.000 (0.000-0.001)	0.000 (0.000-0.001)	0.001 (0.000-0.003)	0.002 (0.001-0.007)	0.008 (0.001-0.022)
Saudi Arabia	0.002 (0.000-0.008)	0.004 (0.001-0.010)	0.008 (0.004-0.014)	0.014 (0.007-0.025)	0.027 (0.010-0.057)
Senegal	0.000 (0.000-0.000)	0.000 (0.000-0.001)	0.000 (0.000-0.001)	0.001 (0.000-0.003)	0.004 (0.001-0.012)
Serbia	0.000 (0.000-0.002)	0.001 (0.000-0.002)	0.001 (0.000-0.003)	0.002 (0.001-0.005)	0.003 (0.001-0.009)
Seychelles	0.000 (0.000-0.001)	0.000 (0.000-0.001)	0.001 (0.000-0.003)	0.004 (0.001-0.009)	0.012 (0.002-0.033)
Sierra Leone	0.000 (0.000-0.000)	0.000 (0.000-0.001)	0.000 (0.000-0.001)	0.001 (0.000-0.003)	0.005 (0.001-0.013)
Singapore	0.000 (0.000-0.000)	0.000 (0.000-0.000)	0.000 (0.000-0.000)	0.000 (0.000-0.001)	0.001 (0.000-0.003)
Slovakia	0.000 (0.000-0.002)	0.001 (0.000-0.002)	0.001 (0.000-0.004)	0.003 (0.001-0.006)	0.006 (0.001-0.015)
Slovenia	0.000 (0.000-0.002)	0.001 (0.000-0.003)	0.002 (0.000-0.006)	0.003 (0.001-0.009)	0.006 (0.001-0.018)
Solomon Islands	0.001 (0.000-0.009)	0.002 (0.000-0.009)	0.003 (0.000-0.012)	0.006 (0.001-0.015)	0.010 (0.002-0.031)
Somalia	0.000 (0.000-0.000)	0.000 (0.000-0.000)	0.000 (0.000-0.000)	0.000 (0.000-0.001)	0.000 (0.000-0.002)
South Africa	0.000 (0.000-0.002)	0.001 (0.000-0.003)	0.003 (0.001-0.006)	0.008 (0.004-0.014)	0.017 (0.006-0.034)
South Korea	0.000 (0.000-0.000)	0.000 (0.000-0.000)	0.000 (0.000-0.000)	0.000 (0.000-0.000)	0.000 (0.000-0.001)
Spain	0.000 (0.000-0.001)	0.000 (0.000-0.001)	0.002 (0.001-0.003)	0.005 (0.002-0.008)	0.009 (0.002-0.022)
Sri Lanka	0.000 (0.000-0.000)	0.000 (0.000-0.000)	0.000 (0.000-0.000)	0.000 (0.000-0.001)	0.002 (0.000-0.009)
Sudan	0.000 (0.000-0.000)	0.000 (0.000-0.000)	0.000 (0.000-0.000)	0.000 (0.000-0.001)	0.001 (0.000-0.004)
Suriname	0.000 (0.000-0.002)	0.001 (0.000-0.003)	0.002 (0.000-0.007)	0.006 (0.001-0.017)	0.019 (0.003-0.057)
Swaziland	0.000 (0.000-0.000)	0.000 (0.000-0.001)	0.000 (0.000-0.002)	0.002 (0.000-0.005)	0.006 (0.001-0.021)
Sweden	0.000 (0.000-0.001)	0.001 (0.000-0.002)	0.002 (0.001-0.004)	0.005 (0.003-0.009)	0.009 (0.002-0.020)
Switzerland	0.000 (0.000-0.001)	0.001 (0.000-0.002)	0.002 (0.001-0.005)	0.006 (0.002-0.011)	0.011 (0.004-0.025)
Syrian Arab Republic	0.001 (0.000-0.003)	0.001 (0.000-0.003)	0.002 (0.000-0.005)	0.003 (0.001-0.009)	0.006 (0.001-0.019)
Taiwan	0.000 (0.000-0.000)	0.000 (0.000-0.000)	0.000 (0.000-0.000)	0.000 (0.000-0.001)	0.002 (0.000-0.007)
Tajikistan	0.001 (0.000-0.005)	0.001 (0.000-0.004)	0.001 (0.000-0.004)	0.001 (0.000-0.005)	0.003 (0.000-0.013)
Tanzania	0.000 (0.000-0.000)	0.000 (0.000-0.000)	0.000 (0.000-0.000)	0.000 (0.000-0.001)	0.001 (0.000-0.002)
Thailand	0.000 (0.000-0.000)	0.000 (0.000-0.000)	0.000 (0.000-0.000)	0.000 (0.000-0.001)	0.003 (0.000-0.008)
Timor-Leste	0.000 (0.000-0.000)	0.000 (0.000-0.000)	0.000 (0.000-0.000)	0.000 (0.000-0.000)	0.000 (0.000-0.001)
Togo	0.000 (0.000-0.000)	0.000 (0.000-0.000)	0.000 (0.000-0.001)	0.001 (0.000-0.002)	0.003 (0.000-0.009)
Tokelau	0.004 (0.000-0.023)	0.009 (0.001-0.032)	0.020 (0.004-0.052)	0.041 (0.013-0.087)	0.074 (0.020-0.162)
Tonga	0.005 (0.000-0.023)	0.011 (0.002-0.033)	0.022 (0.006-0.053)	0.040 (0.016-0.077)	0.061 (0.021-0.123)
Trinidad and Tobago	0.000 (0.000-0.001)	0.000 (0.000-0.001)	0.001 (0.000-0.003)	0.006 (0.001-0.018)	0.030 (0.004-0.092)
Tunisia	0.000 (0.000-0.002)	0.001 (0.000-0.002)	0.001 (0.000-0.003)	0.002 (0.001-0.006)	0.006 (0.001-0.015)
Turkey	0.000 (0.000-0.002)	0.001 (0.000-0.002)	0.002 (0.001-0.003)	0.004 (0.002-0.006)	0.008 (0.003-0.017)
Turkmenistan	0.001 (0.000-0.006)	0.001 (0.000-0.005)	0.002 (0.000-0.006)	0.003 (0.001-0.008)	0.007 (0.001-0.021)
Tuvalu	0.004 (0.000-0.018)	0.009 (0.001-0.027)	0.021 (0.005-0.052)	0.034 (0.010-0.075)	0.048 (0.012-0.112)
Uganda	0.000 (0.000-0.000)	0.000 (0.000-0.000)	0.000 (0.000-0.000)	0.000 (0.000-0.000)	0.001 (0.000-0.002)
Ukraine	0.001 (0.000-0.005)	0.002 (0.000-0.006)	0.003 (0.000-0.009)	0.004 (0.001-0.011)	0.007 (0.001-0.023)
United Arab Emirates	0.004 (0.000-0.021)	0.004 (0.000-0.013)	0.006 (0.001-0.015)	0.013 (0.004-0.029)	0.020 (0.005-0.049)
United Kingdom	0.001 (0.000-0.002)	0.001 (0.000-0.002)	0.003 (0.002-0.005)	0.009 (0.006-0.012)	0.017 (0.009-0.030)
United States of America	0.003 (0.001-0.007)	0.006 (0.003-0.010)	0.014 (0.008-0.023)	0.032 (0.020-0.047)	0.052 (0.026-0.088)
Uruguay	0.001 (0.000-0.005)	0.001 (0.000-0.005)	0.003 (0.000-0.008)	0.005 (0.001-0.014)	0.012 (0.002-0.032)
Uzbekistan	0.001 (0.000-0.006)	0.001 (0.000-0.005)	0.001 (0.000-0.005)	0.002 (0.000-0.007)	0.005 (0.001-0.019)
Vanuatu	0.002 (0.000-0.011)	0.002 (0.000-0.010)	0.004 (0.001-0.013)	0.007 (0.002-0.019)	0.014 (0.003-0.040)
Venezuela	0.001 (0.000-0.004)	0.001 (0.000-0.005)	0.003 (0.001-0.009)	0.007 (0.002-0.016)	0.015 (0.004-0.038)
Viet Nam	0.000 (0.000-0.000)	0.000 (0.000-0.000)	0.000 (0.000-0.000)	0.000 (0.000-0.000)	0.000 (0.000-0.001)
Yemen	0.000 (0.000-0.001)	0.000 (0.000-0.001)	0.000 (0.000-0.002)	0.001 (0.000-0.004)	0.003 (0.000-0.009)
Zambia	0.000 (0.000-0.000)	0.000 (0.000-0.000)	0.000 (0.000-0.001)	0.000 (0.000-0.001)	0.001 (0.000-0.005)
Zimbabwe	0.000 (0.000-0.000)	0.000 (0.000-0.000)	0.000 (0.000-0.001)	0.001 (0.000-0.003)	0.002 (0.000-0.008)

Women, BMI ≥ 40 kg/m^2

Country	Year				
	1975	1985	1995	2005	2014
Afghanistan	0.000 (0.000-0.000)	0.000 (0.000-0.000)	0.000 (0.000-0.001)	0.001 (0.000-0.002)	0.002 (0.000-0.006)
Albania	0.002 (0.000-0.009)	0.003 (0.000-0.010)	0.004 (0.001-0.011)	0.007 (0.002-0.018)	0.011 (0.002-0.028)
Algeria	0.003 (0.000-0.012)	0.006 (0.001-0.017)	0.011 (0.004-0.024)	0.019 (0.007-0.038)	0.029 (0.010-0.061)
American Samoa	0.065 (0.024-0.130)	0.089 (0.046-0.148)	0.117 (0.068-0.180)	0.138 (0.081-0.207)	0.154 (0.078-0.248)
Andorra	0.009 (0.001-0.031)	0.013 (0.003-0.035)	0.017 (0.004-0.040)	0.024 (0.007-0.055)	0.030 (0.007-0.072)
Angola	0.000 (0.000-0.002)	0.001 (0.000-0.003)	0.001 (0.000-0.005)	0.004 (0.001-0.012)	0.009 (0.001-0.030)
Antigua and Barbuda	0.008 (0.001-0.031)	0.016 (0.003-0.044)	0.031 (0.009-0.071)	0.047 (0.015-0.100)	0.066 (0.018-0.146)
Argentina	0.006 (0.001-0.020)	0.009 (0.003-0.022)	0.015 (0.006-0.029)	0.023 (0.011-0.040)	0.034 (0.014-0.066)
Armenia	0.003 (0.000-0.015)	0.005 (0.001-0.017)	0.007 (0.002-0.019)	0.013 (0.004-0.029)	0.021 (0.005-0.051)
Australia	0.005 (0.001-0.010)	0.009 (0.005-0.015)	0.016 (0.010-0.025)	0.029 (0.019-0.043)	0.043 (0.024-0.071)
Austria	0.002 (0.000-0.005)	0.003 (0.001-0.008)	0.006 (0.002-0.013)	0.011 (0.004-0.023)	0.017 (0.005-0.038)
Azerbaijan	0.005 (0.000-0.021)	0.008 (0.001-0.023)	0.010 (0.003-0.024)	0.015 (0.005-0.033)	0.029 (0.008-0.066)
Bahamas	0.018 (0.002-0.057)	0.029 (0.007-0.071)	0.041 (0.013-0.089)	0.055 (0.020-0.111)	0.066 (0.021-0.138)
Bahrain	0.015 (0.002-0.048)	0.016 (0.004-0.037)	0.022 (0.008-0.044)	0.029 (0.012-0.056)	0.038 (0.013-0.077)
Bangladesh	0.000 (0.000-0.000)	0.000 (0.000-0.000)	0.000 (0.000-0.000)	0.001 (0.000-0.001)	0.002 (0.000-0.005)
Barbados	0.007 (0.001-0.024)	0.014 (0.004-0.032)	0.024 (0.010-0.045)	0.041 (0.018-0.075)	0.059 (0.021-0.119)
Belarus	0.005 (0.000-0.021)	0.009 (0.002-0.027)	0.013 (0.003-0.036)	0.018 (0.005-0.044)	0.025 (0.006-0.063)
Belgium	0.003 (0.001-0.009)	0.005 (0.002-0.010)	0.009 (0.004-0.014)	0.014 (0.007-0.024)	0.019 (0.007-0.039)
Belize	0.005 (0.000-0.019)	0.010 (0.002-0.027)	0.020 (0.006-0.044)	0.035 (0.013-0.070)	0.050 (0.017-0.105)
Benin	0.000 (0.000-0.002)	0.001 (0.000-0.003)	0.003 (0.001-0.008)	0.009 (0.003-0.017)	0.018 (0.007-0.038)
Bermuda	0.034 (0.004-0.099)	0.042 (0.009-0.102)	0.050 (0.014-0.108)	0.062 (0.022-0.123)	0.075 (0.024-0.152)
Bhutan	0.000 (0.000-0.000)	0.000 (0.000-0.000)	0.000 (0.000-0.001)	0.001 (0.000-0.003)	0.003 (0.001-0.009)
Bolivia	0.001 (0.000-0.006)	0.002 (0.000-0.008)	0.005 (0.001-0.013)	0.010 (0.003-0.022)	0.018 (0.005-0.043)
Bosnia and Herzegovina	0.001 (0.000-0.003)	0.001 (0.000-0.004)	0.002 (0.000-0.007)	0.007 (0.002-0.017)	0.010 (0.003-0.023)
Botswana	0.002 (0.000-0.007)	0.006 (0.001-0.019)	0.017 (0.004-0.042)	0.030 (0.010-0.063)	0.045 (0.014-0.097)
Brazil	0.002 (0.000-0.005)	0.004 (0.001-0.009)	0.008 (0.004-0.013)	0.014 (0.008-0.021)	0.022 (0.010-0.039)
Brunei Darussalam	0.003 (0.000-0.015)	0.004 (0.000-0.016)	0.006 (0.001-0.020)	0.011 (0.002-0.031)	0.018 (0.003-0.051)
Bulgaria	0.004 (0.000-0.014)	0.006 (0.001-0.018)	0.009 (0.002-0.021)	0.012 (0.004-0.028)	0.016 (0.004-0.041)
Burkina Faso	0.000 (0.000-0.000)	0.000 (0.000-0.001)	0.001 (0.000-0.003)	0.004 (0.001-0.008)	0.009 (0.003-0.020)
Burundi	0.000 (0.000-0.000)	0.000 (0.000-0.001)	0.000 (0.000-0.002)	0.001 (0.000-0.004)	0.004 (0.001-0.011)
Cabo Verde	0.000 (0.000-0.001)	0.001 (0.000-0.003)	0.003 (0.001-0.009)	0.011 (0.003-0.025)	0.021 (0.006-0.049)
Cambodia	0.000 (0.000-0.000)	0.000 (0.000-0.000)	0.000 (0.000-0.000)	0.000 (0.000-0.001)	0.002 (0.000-0.005)
Cameroon	0.000 (0.000-0.002)	0.002 (0.000-0.005)	0.004 (0.001-0.008)	0.009 (0.004-0.018)	0.020 (0.008-0.040)
Canada	0.004 (0.001-0.011)	0.010 (0.004-0.018)	0.019 (0.010-0.032)	0.033 (0.019-0.052)	0.047 (0.022-0.080)
Central African Republic	0.000 (0.000-0.000)	0.000 (0.000-0.001)	0.001 (0.000-0.002)	0.001 (0.000-0.005)	0.004 (0.001-0.013)
Chad	0.000 (0.000-0.001)	0.000 (0.000-0.001)	0.001 (0.000-0.004)	0.004 (0.001-0.010)	0.011 (0.003-0.027)
Chile	0.005 (0.001-0.019)	0.010 (0.003-0.024)	0.018 (0.008-0.034)	0.029 (0.015-0.049)	0.041 (0.018-0.074)
China	0.000 (0.000-0.000)	0.000 (0.000-0.000)	0.000 (0.000-0.000)	0.001 (0.000-0.001)	0.002 (0.001-0.005)
China (Hong Kong SAR)	0.000 (0.000-0.002)	0.001 (0.000-0.002)	0.001 (0.000-0.004)	0.002 (0.000-0.005)	0.003 (0.000-0.010)
Colombia	0.001 (0.000-0.005)	0.003 (0.001-0.008)	0.006 (0.002-0.012)	0.010 (0.005-0.019)	0.017 (0.006-0.033)
Comoros	0.000 (0.000-0.002)	0.001 (0.000-0.003)	0.002 (0.000-0.005)	0.004 (0.001-0.009)	0.009 (0.002-0.021)
Congo	0.000 (0.000-0.001)	0.001 (0.000-0.003)	0.002 (0.000-0.005)	0.004 (0.001-0.010)	0.009 (0.002-0.024)
Cook Islands	0.029 (0.005-0.077)	0.058 (0.020-0.114)	0.091 (0.040-0.156)	0.117 (0.057-0.188)	0.135 (0.063-0.223)
Costa Rica	0.002 (0.000-0.010)	0.005 (0.001-0.015)	0.011 (0.004-0.025)	0.021 (0.009-0.042)	0.034 (0.013-0.067)
Cote d'Ivoire	0.001 (0.000-0.004)	0.002 (0.000-0.005)	0.004 (0.001-0.009)	0.008 (0.003-0.017)	0.015 (0.005-0.033)
Croatia	0.003 (0.000-0.012)	0.005 (0.001-0.015)	0.008 (0.002-0.020)	0.012 (0.003-0.028)	0.015 (0.003-0.040)
Cuba	0.006 (0.001-0.020)	0.013 (0.004-0.031)	0.020 (0.007-0.039)	0.029 (0.013-0.053)	0.042 (0.017-0.081)
Cyprus	0.001 (0.000-0.005)	0.005 (0.001-0.014)	0.011 (0.003-0.027)	0.018 (0.005-0.042)	0.026 (0.006-0.062)
Czech Republic	0.009 (0.002-0.026)	0.013 (0.004-0.026)	0.015 (0.006-0.028)	0.017 (0.007-0.033)	0.020 (0.006-0.044)
Denmark	0.002 (0.000-0.006)	0.004 (0.001-0.009)	0.007 (0.003-0.014)	0.011 (0.004-0.021)	0.015 (0.005-0.032)
Djibouti	0.001 (0.000-0.005)	0.002 (0.000-0.006)	0.003 (0.000-0.011)	0.006 (0.001-0.017)	0.010 (0.002-0.032)
Dominica	0.005 (0.000-0.017)	0.009 (0.002-0.026)	0.020 (0.006-0.045)	0.033 (0.012-0.068)	0.049 (0.016-0.100)
Dominican Republic	0.002 (0.000-0.008)	0.006 (0.001-0.014)	0.012 (0.005-0.024)	0.025 (0.012-0.046)	0.041 (0.015-0.080)
DR Congo	0.000 (0.000-0.001)	0.000 (0.000-0.001)	0.001 (0.000-0.003)	0.002 (0.000-0.005)	0.006 (0.001-0.016)
Ecuador	0.001 (0.000-0.005)	0.003 (0.000-0.009)	0.005 (0.001-0.015)	0.010 (0.003-0.023)	0.017 (0.005-0.039)
Egypt	0.007 (0.001-0.021)	0.015 (0.005-0.030)	0.026 (0.014-0.044)	0.044 (0.026-0.067)	0.066 (0.035-0.107)
El Salvador	0.002 (0.000-0.007)	0.003 (0.000-0.009)	0.007 (0.002-0.016)	0.014 (0.005-0.029)	0.023 (0.007-0.050)
Equatorial Guinea	0.000 (0.000-0.001)	0.000 (0.000-0.002)	0.001 (0.000-0.004)	0.010 (0.002-0.028)	0.020 (0.003-0.059)
Eritrea	0.000 (0.000-0.000)	0.000 (0.000-0.000)	0.000 (0.000-0.001)	0.001 (0.000-0.003)	0.003 (0.001-0.008)
Estonia	0.009 (0.001-0.026)	0.011 (0.004-0.025)	0.013 (0.006-0.023)	0.016 (0.009-0.026)	0.018 (0.008-0.035)
Ethiopia	0.000 (0.000-0.000)	0.000 (0.000-0.000)	0.000 (0.000-0.001)	0.001 (0.000-0.002)	0.003 (0.001-0.007)
Fiji	0.009 (0.001-0.038)	0.014 (0.003-0.042)	0.022 (0.006-0.052)	0.033 (0.013-0.065)	0.044 (0.016-0.089)
Finland	0.001 (0.000-0.004)	0.003 (0.001-0.007)	0.007 (0.004-0.013)	0.013 (0.008-0.021)	0.018 (0.008-0.032)
France	0.003 (0.000-0.009)	0.005 (0.002-0.011)	0.008 (0.003-0.016)	0.013 (0.006-0.023)	0.019 (0.006-0.038)
French Polynesia	0.053 (0.010-0.132)	0.074 (0.023-0.150)	0.094 (0.039-0.172)	0.111 (0.054-0.185)	0.126 (0.058-0.211)

Gabon	0.001 (0.000-0.005)	0.003 (0.000-0.009)	0.005 (0.001-0.014)	0.010 (0.003-0.023)	0.015 (0.004-0.038)
Gambia	0.000 (0.000-0.002)	0.001 (0.000-0.003)	0.003 (0.001-0.008)	0.008 (0.003-0.018)	0.018 (0.006-0.038)
Georgia	0.005 (0.000-0.023)	0.010 (0.002-0.032)	0.011 (0.003-0.029)	0.017 (0.005-0.038)	0.027 (0.008-0.063)
Germany	0.002 (0.000-0.005)	0.004 (0.002-0.006)	0.007 (0.004-0.011)	0.013 (0.007-0.019)	0.019 (0.008-0.036)
Ghana	0.001 (0.000-0.004)	0.002 (0.001-0.006)	0.006 (0.002-0.012)	0.015 (0.007-0.027)	0.031 (0.013-0.060)
Greece	0.004 (0.000-0.016)	0.008 (0.002-0.021)	0.012 (0.004-0.028)	0.020 (0.007-0.043)	0.026 (0.007-0.062)
Greenland	0.003 (0.000-0.008)	0.006 (0.001-0.014)	0.009 (0.003-0.021)	0.015 (0.005-0.032)	0.020 (0.006-0.045)
Grenada	0.003 (0.000-0.012)	0.007 (0.001-0.021)	0.017 (0.005-0.039)	0.032 (0.011-0.066)	0.051 (0.017-0.105)
Guatemala	0.002 (0.000-0.007)	0.003 (0.001-0.009)	0.007 (0.002-0.014)	0.013 (0.005-0.026)	0.023 (0.007-0.049)
Guinea	0.000 (0.000-0.001)	0.001 (0.000-0.002)	0.002 (0.000-0.004)	0.004 (0.001-0.010)	0.010 (0.003-0.023)
Guinea Bissau	0.000 (0.000-0.001)	0.001 (0.000-0.003)	0.002 (0.000-0.007)	0.006 (0.001-0.014)	0.012 (0.003-0.030)
Guyana	0.003 (0.000-0.014)	0.007 (0.001-0.021)	0.014 (0.004-0.035)	0.029 (0.009-0.063)	0.047 (0.015-0.100)
Haiti	0.001 (0.000-0.003)	0.001 (0.000-0.005)	0.003 (0.001-0.009)	0.008 (0.002-0.018)	0.017 (0.004-0.042)
Honduras	0.001 (0.000-0.004)	0.002 (0.000-0.007)	0.005 (0.001-0.013)	0.012 (0.004-0.024)	0.022 (0.007-0.047)
Hungary	0.006 (0.001-0.018)	0.008 (0.003-0.019)	0.010 (0.004-0.021)	0.012 (0.004-0.026)	0.015 (0.004-0.037)
Iceland	0.001 (0.000-0.004)	0.003 (0.001-0.008)	0.006 (0.002-0.012)	0.009 (0.003-0.020)	0.013 (0.003-0.031)
India	0.000 (0.000-0.000)	0.000 (0.000-0.000)	0.000 (0.000-0.000)	0.001 (0.000-0.001)	0.002 (0.001-0.003)
Indonesia	0.000 (0.000-0.000)	0.000 (0.000-0.000)	0.000 (0.000-0.001)	0.001 (0.000-0.002)	0.004 (0.001-0.009)
Iran	0.003 (0.000-0.011)	0.005 (0.001-0.010)	0.007 (0.004-0.012)	0.013 (0.009-0.019)	0.022 (0.011-0.039)
Iraq	0.004 (0.000-0.014)	0.006 (0.001-0.018)	0.008 (0.002-0.021)	0.014 (0.004-0.033)	0.024 (0.006-0.057)
Ireland	0.001 (0.000-0.004)	0.003 (0.001-0.008)	0.009 (0.003-0.019)	0.021 (0.009-0.039)	0.035 (0.014-0.067)
Israel	0.006 (0.001-0.019)	0.009 (0.003-0.022)	0.014 (0.005-0.029)	0.020 (0.008-0.038)	0.026 (0.008-0.056)
Italy	0.002 (0.000-0.007)	0.004 (0.002-0.008)	0.007 (0.004-0.011)	0.012 (0.006-0.018)	0.017 (0.007-0.033)
Jamaica	0.007 (0.001-0.023)	0.012 (0.004-0.027)	0.024 (0.011-0.042)	0.040 (0.021-0.067)	0.059 (0.025-0.108)
Japan	0.000 (0.000-0.000)	0.000 (0.000-0.000)	0.000 (0.000-0.000)	0.000 (0.000-0.000)	0.000 (0.000-0.000)
Jordan	0.006 (0.001-0.021)	0.016 (0.006-0.033)	0.026 (0.012-0.046)	0.041 (0.024-0.065)	0.061 (0.030-0.102)
Kazakhstan	0.005 (0.000-0.022)	0.009 (0.002-0.027)	0.015 (0.004-0.035)	0.022 (0.007-0.049)	0.032 (0.008-0.076)
Kenya	0.000 (0.000-0.001)	0.001 (0.000-0.002)	0.002 (0.000-0.004)	0.005 (0.001-0.011)	0.012 (0.003-0.029)
Kiribati	0.014 (0.002-0.041)	0.022 (0.007-0.051)	0.038 (0.014-0.077)	0.065 (0.027-0.117)	0.089 (0.034-0.166)
Kuwait	0.041 (0.008-0.109)	0.035 (0.014-0.069)	0.041 (0.021-0.069)	0.052 (0.028-0.082)	0.063 (0.030-0.110)
Kyrgyzstan	0.001 (0.000-0.007)	0.003 (0.000-0.009)	0.005 (0.001-0.012)	0.008 (0.003-0.019)	0.015 (0.004-0.035)
Lao PDR	0.000 (0.000-0.000)	0.000 (0.000-0.000)	0.000 (0.000-0.000)	0.000 (0.000-0.001)	0.001 (0.000-0.004)
Latvia	0.009 (0.001-0.032)	0.014 (0.003-0.037)	0.016 (0.005-0.038)	0.020 (0.007-0.044)	0.025 (0.007-0.060)
Lebanon	0.005 (0.001-0.018)	0.009 (0.002-0.023)	0.015 (0.005-0.032)	0.024 (0.008-0.050)	0.037 (0.013-0.076)
Lesotho	0.002 (0.000-0.007)	0.004 (0.001-0.013)	0.011 (0.003-0.026)	0.024 (0.009-0.048)	0.043 (0.016-0.085)
Liberia	0.001 (0.000-0.003)	0.002 (0.000-0.006)	0.002 (0.000-0.007)	0.006 (0.002-0.015)	0.016 (0.005-0.037)
Libya	0.018 (0.002-0.060)	0.023 (0.007-0.054)	0.027 (0.010-0.054)	0.035 (0.015-0.066)	0.047 (0.018-0.092)
Lithuania	0.009 (0.001-0.028)	0.014 (0.005-0.032)	0.018 (0.008-0.034)	0.023 (0.010-0.043)	0.030 (0.009-0.064)
Luxembourg	0.002 (0.000-0.006)	0.003 (0.001-0.009)	0.006 (0.001-0.017)	0.011 (0.003-0.026)	0.015 (0.004-0.038)
Macedonia (TFYR)	0.004 (0.000-0.016)	0.006 (0.001-0.018)	0.009 (0.002-0.022)	0.011 (0.003-0.027)	0.014 (0.003-0.036)
Madagascar	0.000 (0.000-0.000)	0.000 (0.000-0.001)	0.000 (0.000-0.001)	0.001 (0.000-0.003)	0.004 (0.001-0.010)
Malawi	0.000 (0.000-0.000)	0.000 (0.000-0.001)	0.001 (0.000-0.002)	0.002 (0.001-0.006)	0.007 (0.002-0.017)
Malaysia	0.000 (0.000-0.000)	0.000 (0.000-0.001)	0.002 (0.001-0.004)	0.006 (0.003-0.011)	0.015 (0.005-0.030)
Maldives	0.000 (0.000-0.000)	0.000 (0.000-0.000)	0.000 (0.000-0.001)	0.002 (0.000-0.005)	0.005 (0.001-0.015)
Mali	0.000 (0.000-0.001)	0.001 (0.000-0.002)	0.002 (0.000-0.004)	0.005 (0.002-0.011)	0.012 (0.004-0.027)
Malta	0.005 (0.000-0.020)	0.011 (0.003-0.027)	0.017 (0.005-0.039)	0.027 (0.009-0.056)	0.035 (0.009-0.081)
Marshall Islands	0.025 (0.004-0.071)	0.041 (0.012-0.090)	0.068 (0.027-0.125)	0.090 (0.041-0.156)	0.111 (0.046-0.198)
Mauritania	0.001 (0.000-0.003)	0.002 (0.000-0.006)	0.005 (0.001-0.013)	0.011 (0.004-0.025)	0.021 (0.006-0.048)
Mauritius	0.001 (0.000-0.004)	0.002 (0.001-0.006)	0.006 (0.002-0.014)	0.014 (0.005-0.028)	0.026 (0.008-0.057)
Mexico	0.005 (0.000-0.015)	0.009 (0.003-0.020)	0.016 (0.008-0.027)	0.026 (0.016-0.039)	0.038 (0.018-0.066)
Micronesia (Federated States of)	0.021 (0.004-0.061)	0.036 (0.012-0.076)	0.057 (0.026-0.103)	0.084 (0.048-0.133)	0.112 (0.057-0.178)
Moldova	0.004 (0.000-0.017)	0.007 (0.001-0.022)	0.009 (0.002-0.024)	0.012 (0.004-0.029)	0.019 (0.005-0.044)
Mongolia	0.001 (0.000-0.006)	0.002 (0.000-0.009)	0.004 (0.001-0.011)	0.007 (0.003-0.015)	0.013 (0.004-0.029)
Montenegro	0.004 (0.000-0.018)	0.007 (0.001-0.022)	0.009 (0.002-0.024)	0.011 (0.003-0.029)	0.014 (0.003-0.039)
Morocco	0.002 (0.000-0.008)	0.004 (0.001-0.012)	0.009 (0.003-0.019)	0.017 (0.006-0.034)	0.029 (0.009-0.062)
Mozambique	0.000 (0.000-0.000)	0.000 (0.000-0.001)	0.000 (0.000-0.001)	0.002 (0.000-0.004)	0.005 (0.001-0.012)
Myanmar	0.000 (0.000-0.000)	0.000 (0.000-0.000)	0.000 (0.000-0.000)	0.001 (0.000-0.002)	0.003 (0.001-0.009)
Namibia	0.004 (0.000-0.017)	0.006 (0.001-0.018)	0.011 (0.004-0.025)	0.021 (0.009-0.039)	0.034 (0.013-0.066)
Nauru	0.109 (0.048-0.191)	0.131 (0.078-0.192)	0.139 (0.089-0.196)	0.143 (0.085-0.210)	0.158 (0.076-0.256)
Nepal	0.000 (0.000-0.000)	0.000 (0.000-0.000)	0.000 (0.000-0.000)	0.001 (0.000-0.001)	0.001 (0.000-0.004)
Netherlands	0.001 (0.000-0.004)	0.002 (0.001-0.005)	0.005 (0.002-0.010)	0.009 (0.005-0.017)	0.013 (0.005-0.026)
New Zealand	0.005 (0.001-0.012)	0.009 (0.004-0.018)	0.018 (0.009-0.032)	0.035 (0.021-0.054)	0.052 (0.029-0.083)
Nicaragua	0.003 (0.000-0.011)	0.005 (0.001-0.013)	0.008 (0.003-0.018)	0.017 (0.007-0.033)	0.028 (0.010-0.060)
Niger	0.000 (0.000-0.001)	0.000 (0.000-0.001)	0.001 (0.000-0.003)	0.003 (0.001-0.007)	0.007 (0.002-0.017)
Nigeria	0.001 (0.000-0.003)	0.001 (0.000-0.004)	0.004 (0.001-0.007)	0.010 (0.005-0.017)	0.022 (0.010-0.041)
Niue	0.023 (0.003-0.071)	0.047 (0.013-0.105)	0.074 (0.029-0.141)	0.101 (0.048-0.170)	0.125 (0.058-0.210)
North Korea	0.000 (0.000-0.000)	0.000 (0.000-0.000)	0.000 (0.000-0.001)	0.000 (0.000-0.002)	0.001 (0.000-0.005)
Norway	0.002 (0.000-0.005)	0.005 (0.002-0.010)	0.009 (0.005-0.017)	0.016 (0.008-0.028)	0.022 (0.009-0.043)

Occupied Palestinian Territory	0.006 (0.001-0.020)	0.010 (0.003-0.024)	0.017 (0.007-0.034)	0.032 (0.015-0.058)	0.050 (0.019-0.099)
Oman	0.004 (0.000-0.016)	0.010 (0.003-0.023)	0.018 (0.008-0.034)	0.028 (0.013-0.050)	0.041 (0.017-0.082)
Pakistan	0.000 (0.000-0.000)	0.000 (0.000-0.001)	0.001 (0.000-0.003)	0.003 (0.001-0.006)	0.006 (0.002-0.013)
Palau	0.061 (0.013-0.148)	0.068 (0.023-0.134)	0.086 (0.036-0.153)	0.104 (0.049-0.173)	0.119 (0.053-0.203)
Panama	0.003 (0.000-0.012)	0.006 (0.001-0.017)	0.011 (0.004-0.026)	0.021 (0.009-0.042)	0.035 (0.013-0.072)
Papua New Guinea	0.002 (0.000-0.011)	0.003 (0.000-0.013)	0.007 (0.001-0.020)	0.012 (0.003-0.030)	0.020 (0.004-0.054)
Paraguay	0.001 (0.000-0.004)	0.003 (0.000-0.010)	0.007 (0.001-0.020)	0.013 (0.003-0.034)	0.022 (0.005-0.058)
Peru	0.001 (0.000-0.006)	0.003 (0.001-0.007)	0.004 (0.002-0.010)	0.008 (0.004-0.014)	0.015 (0.007-0.028)
Philippines	0.000 (0.000-0.000)	0.000 (0.000-0.000)	0.000 (0.000-0.001)	0.001 (0.000-0.003)	0.004 (0.001-0.010)
Poland	0.006 (0.001-0.019)	0.009 (0.003-0.018)	0.012 (0.006-0.021)	0.016 (0.008-0.028)	0.021 (0.008-0.041)
Portugal	0.001 (0.000-0.004)	0.002 (0.000-0.006)	0.005 (0.002-0.011)	0.009 (0.003-0.018)	0.014 (0.004-0.031)
Puerto Rico	0.013 (0.002-0.040)	0.025 (0.007-0.059)	0.043 (0.018-0.082)	0.057 (0.028-0.098)	0.068 (0.030-0.121)
Qatar	0.019 (0.003-0.057)	0.045 (0.014-0.095)	0.046 (0.020-0.085)	0.061 (0.030-0.104)	0.074 (0.034-0.133)
Romania	0.004 (0.000-0.015)	0.007 (0.002-0.018)	0.009 (0.003-0.021)	0.012 (0.004-0.029)	0.017 (0.004-0.041)
Russian Federation	0.011 (0.003-0.029)	0.016 (0.009-0.027)	0.020 (0.013-0.028)	0.024 (0.014-0.036)	0.029 (0.011-0.056)
Rwanda	0.000 (0.000-0.000)	0.000 (0.000-0.000)	0.000 (0.000-0.001)	0.001 (0.000-0.003)	0.004 (0.001-0.010)
Saint Kitts and Nevis	0.006 (0.001-0.021)	0.014 (0.003-0.038)	0.027 (0.008-0.060)	0.044 (0.016-0.088)	0.063 (0.021-0.131)
Saint Lucia	0.003 (0.000-0.012)	0.007 (0.002-0.019)	0.018 (0.006-0.037)	0.032 (0.013-0.061)	0.054 (0.018-0.111)
Saint Vincent and the Grenadines	0.003 (0.000-0.012)	0.007 (0.001-0.021)	0.017 (0.004-0.040)	0.031 (0.010-0.069)	0.046 (0.014-0.104)
Samoa	0.025 (0.005-0.067)	0.041 (0.015-0.080)	0.068 (0.034-0.116)	0.104 (0.060-0.157)	0.133 (0.072-0.206)
Sao Tome and Principe	0.001 (0.000-0.006)	0.003 (0.000-0.008)	0.007 (0.002-0.017)	0.014 (0.005-0.030)	0.025 (0.008-0.056)
Saudi Arabia	0.018 (0.003-0.052)	0.026 (0.011-0.049)	0.033 (0.019-0.051)	0.045 (0.027-0.068)	0.062 (0.031-0.106)
Senegal	0.001 (0.000-0.003)	0.002 (0.000-0.005)	0.004 (0.001-0.009)	0.009 (0.003-0.019)	0.018 (0.006-0.039)
Serbia	0.004 (0.000-0.013)	0.006 (0.002-0.014)	0.008 (0.003-0.016)	0.011 (0.005-0.020)	0.015 (0.005-0.032)
Seychelles	0.003 (0.000-0.012)	0.008 (0.002-0.021)	0.017 (0.006-0.034)	0.029 (0.013-0.055)	0.045 (0.017-0.090)
Sierra Leone	0.001 (0.000-0.003)	0.002 (0.000-0.005)	0.003 (0.001-0.009)	0.007 (0.002-0.017)	0.017 (0.006-0.038)
Singapore	0.000 (0.000-0.001)	0.000 (0.000-0.001)	0.001 (0.000-0.002)	0.001 (0.000-0.002)	0.001 (0.000-0.004)
Slovakia	0.002 (0.000-0.008)	0.004 (0.001-0.010)	0.006 (0.002-0.014)	0.009 (0.003-0.019)	0.013 (0.004-0.030)
Slovenia	0.003 (0.000-0.012)	0.005 (0.001-0.015)	0.008 (0.002-0.021)	0.011 (0.003-0.027)	0.014 (0.003-0.037)
Solomon Islands	0.002 (0.000-0.008)	0.003 (0.000-0.013)	0.007 (0.001-0.020)	0.012 (0.003-0.028)	0.020 (0.005-0.049)
Somalia	0.000 (0.000-0.001)	0.000 (0.000-0.001)	0.001 (0.000-0.003)	0.002 (0.000-0.005)	0.004 (0.001-0.014)
South Africa	0.025 (0.005-0.072)	0.036 (0.014-0.073)	0.049 (0.028-0.078)	0.068 (0.046-0.095)	0.087 (0.053-0.129)
South Korea	0.000 (0.000-0.000)	0.000 (0.000-0.000)	0.000 (0.000-0.000)	0.000 (0.000-0.000)	0.000 (0.000-0.001)
Spain	0.003 (0.001-0.009)	0.006 (0.002-0.011)	0.010 (0.006-0.016)	0.017 (0.010-0.027)	0.025 (0.010-0.049)
Sri Lanka	0.000 (0.000-0.000)	0.000 (0.000-0.000)	0.000 (0.000-0.001)	0.002 (0.000-0.004)	0.006 (0.001-0.017)
Sudan	0.000 (0.000-0.001)	0.000 (0.000-0.001)	0.001 (0.000-0.003)	0.003 (0.001-0.008)	0.006 (0.001-0.018)
Suriname	0.010 (0.001-0.035)	0.019 (0.004-0.048)	0.025 (0.006-0.058)	0.036 (0.011-0.077)	0.051 (0.016-0.109)
Swaziland	0.003 (0.000-0.012)	0.006 (0.001-0.021)	0.016 (0.004-0.039)	0.028 (0.009-0.062)	0.045 (0.013-0.098)
Sweden	0.002 (0.000-0.005)	0.003 (0.001-0.006)	0.006 (0.003-0.010)	0.010 (0.006-0.017)	0.015 (0.006-0.029)
Switzerland	0.001 (0.000-0.005)	0.003 (0.001-0.007)	0.006 (0.002-0.013)	0.010 (0.004-0.019)	0.013 (0.004-0.028)
Syrian Arab Republic	0.003 (0.000-0.012)	0.007 (0.001-0.019)	0.012 (0.004-0.028)	0.022 (0.007-0.045)	0.035 (0.011-0.076)
Taiwan	0.000 (0.000-0.000)	0.000 (0.000-0.000)	0.000 (0.000-0.001)	0.001 (0.000-0.002)	0.002 (0.000-0.006)
Tajikistan	0.002 (0.000-0.009)	0.003 (0.000-0.010)	0.004 (0.001-0.010)	0.006 (0.001-0.015)	0.013 (0.003-0.034)
Tanzania	0.000 (0.000-0.001)	0.000 (0.000-0.001)	0.001 (0.000-0.003)	0.004 (0.001-0.007)	0.009 (0.003-0.020)
Thailand	0.000 (0.000-0.000)	0.000 (0.000-0.000)	0.001 (0.000-0.002)	0.003 (0.001-0.006)	0.008 (0.002-0.018)
Timor-Leste	0.000 (0.000-0.000)	0.000 (0.000-0.000)	0.000 (0.000-0.000)	0.000 (0.000-0.001)	0.001 (0.000-0.004)
Togo	0.000 (0.000-0.002)	0.001 (0.000-0.003)	0.003 (0.001-0.007)	0.007 (0.002-0.015)	0.015 (0.005-0.032)
Tokelau	0.025 (0.002-0.090)	0.039 (0.008-0.104)	0.061 (0.020-0.126)	0.088 (0.038-0.159)	0.116 (0.045-0.210)
Tonga	0.025 (0.004-0.073)	0.041 (0.013-0.092)	0.067 (0.029-0.125)	0.096 (0.051-0.156)	0.121 (0.060-0.196)
Trinidad and Tobago	0.008 (0.000-0.031)	0.014 (0.003-0.040)	0.022 (0.007-0.050)	0.043 (0.015-0.088)	0.067 (0.018-0.149)
Tunisia	0.003 (0.000-0.010)	0.006 (0.002-0.015)	0.011 (0.004-0.023)	0.021 (0.009-0.039)	0.034 (0.013-0.067)
Turkey	0.005 (0.001-0.015)	0.009 (0.004-0.019)	0.018 (0.011-0.029)	0.031 (0.020-0.043)	0.048 (0.027-0.075)
Turkmenistan	0.002 (0.000-0.012)	0.004 (0.001-0.013)	0.007 (0.002-0.019)	0.012 (0.004-0.027)	0.021 (0.006-0.051)
Tuvalu	0.015 (0.002-0.045)	0.031 (0.009-0.072)	0.063 (0.024-0.124)	0.093 (0.041-0.166)	0.118 (0.049-0.209)
Uganda	0.000 (0.000-0.000)	0.000 (0.000-0.000)	0.000 (0.000-0.001)	0.001 (0.000-0.003)	0.004 (0.001-0.010)
Ukraine	0.008 (0.001-0.030)	0.012 (0.002-0.034)	0.015 (0.004-0.036)	0.017 (0.005-0.040)	0.022 (0.005-0.056)
United Arab Emirates	0.038 (0.005-0.113)	0.033 (0.010-0.074)	0.035 (0.015-0.064)	0.046 (0.022-0.080)	0.054 (0.021-0.105)
United Kingdom	0.003 (0.001-0.008)	0.007 (0.004-0.012)	0.014 (0.010-0.020)	0.028 (0.020-0.036)	0.042 (0.026-0.061)
United States of America	0.013 (0.005-0.027)	0.024 (0.014-0.037)	0.042 (0.028-0.059)	0.063 (0.045-0.085)	0.081 (0.049-0.121)
Uruguay	0.007 (0.001-0.027)	0.012 (0.003-0.033)	0.021 (0.007-0.045)	0.030 (0.012-0.059)	0.044 (0.016-0.089)
Uzbekistan	0.002 (0.000-0.008)	0.003 (0.000-0.010)	0.005 (0.001-0.015)	0.010 (0.003-0.024)	0.019 (0.004-0.049)
Vanuatu	0.004 (0.000-0.019)	0.007 (0.001-0.023)	0.014 (0.004-0.033)	0.024 (0.009-0.049)	0.038 (0.012-0.080)
Venezuela	0.004 (0.000-0.016)	0.006 (0.001-0.018)	0.010 (0.003-0.023)	0.016 (0.006-0.030)	0.022 (0.008-0.046)
Viet Nam	0.000 (0.000-0.000)	0.000 (0.000-0.000)	0.000 (0.000-0.000)	0.000 (0.000-0.000)	0.000 (0.000-0.001)
Yemen	0.001 (0.000-0.004)	0.002 (0.000-0.005)	0.003 (0.001-0.008)	0.007 (0.002-0.017)	0.014 (0.004-0.033)
Zambia	0.001 (0.000-0.002)	0.001 (0.000-0.003)	0.002 (0.001-0.005)	0.005 (0.002-0.010)	0.011 (0.003-0.024)
Zimbabwe	0.003 (0.000-0.012)	0.006 (0.002-0.016)	0.013 (0.005-0.025)	0.022 (0.009-0.040)	0.032 (0.011-0.066)

Appendix Table 6: Results of model validation. Test 1 and Test 2 are described in Appendix 3.

Test 1: Men, mean BMI

Data		No. of held out observations	Percent covered	Error (kg/m^2)†				Absolute error			
				Median	Q1	Q3	(p*)	Median	Q1	Q3	(p*)
All		4099	98	0.00	-0.36	0.32	0.30	0.34	0.15	0.66	0.30
Super-region	Central and Eastern Europe	328	99	0.04	-0.42	0.41	0.38	0.41	0.20	0.69	0.38
	Central Asia, Middle East and North Africa	310	98	0.00	-0.31	0.32	0.87	0.31	0.13	0.67	0.87
	East and South East Asia	569	99	0.00	-0.25	0.22	0.72	0.24	0.10	0.44	0.72
	High-income Asia Pacific	*No data from this region were among the held-out data*									
	High-income Western countries	1822	99	0.02	-0.39	0.34	0.10	0.36	0.16	0.67	0.10
	Latin America and Caribbean	379	98	-0.05	-0.46	0.27	0.58	0.35	0.17	0.75	0.58
	Oceania	163	82	0.01	-0.73	0.72	0.89	0.73	0.29	1.78	0.89
	South Asia	330	100	-0.03	-0.33	0.26	0.89	0.30	0.13	0.62	0.89
	Sub-Saharan Africa	198	100	0.01	-0.35	0.32	0.91	0.33	0.15	0.55	0.91
Urban or rural studies	Rural	469	98	0.00	-0.43	0.33	0.63	0.37	0.16	0.71	0.63
	Urban	1263	98	-0.01	-0.41	0.37	0.32	0.40	0.18	0.73	0.32
	Both rural and urban	2367	98	0.01	-0.32	0.30	0.63	0.31	0.14	0.60	0.63
Study representativeness	Community	1698	98	-0.02	-0.45	0.36	0.23	0.41	0.18	0.76	0.23
	Sub-national	626	99	0.01	-0.34	0.33	0.94	0.33	0.16	0.67	0.94
	National	1775	98	0.01	-0.31	0.28	0.74	0.29	0.13	0.55	0.74
Age band	18-40	1549	98	0.12	-0.20	0.49	0.15	0.36	0.15	0.68	0.15
	40-60	1300	98	0.02	-0.26	0.26	0.66	0.26	0.12	0.48	0.66
	60 and above	1250	98	-0.25	-0.68	0.14	0.00	0.44	0.21	0.81	0.00
Years	1975-1984	233	96	-0.05	-0.37	0.34	0.79	0.35	0.18	0.66	0.79
	1985-1994	1057	98	0.01	-0.34	0.32	0.52	0.33	0.13	0.66	0.52
	1995-2004	1423	99	0.01	-0.38	0.34	0.57	0.35	0.15	0.65	0.57
	2005-2014	1386	98	0.00	-0.37	0.31	0.49	0.33	0.15	0.67	0.49
Data density	Data poor	159	98	-0.07	-0.46	0.32	0.87	0.38	0.17	0.73	0.87
	Average data density	431	99	-0.01	-0.34	0.32	0.95	0.33	0.15	0.61	0.95
	Data rich	3509	98	0.01	-0.36	0.32	0.28	0.34	0.15	0.66	0.28

† Estimated values minus held out values.

* p-values for model error comparisons were calculated using the non-parametric Wilcoxon signed-rank test for paired data. The p-values are calculated assuming independence of the held-out observations. They should therefore be interpreted as an approximation because there is some dependence among the held-out observations, within each of the five repetitions for example.

Test 1: Men, BMI < 18.5 kg/m^2

Data		No. of held out observations	Percent covered	Error (percentage points)†				Absolute error			
				Median	Q1	Q3	(p*)	Median	Q1	Q3	(p*)
All		4099	78	-0.10	-1.72	0.62	0.02	1.08	0.34	3.79	0.02
Super-region	Central and Eastern Europe	328	78	-0.60	-1.36	0.23	0.00	0.79	0.35	1.64	0.00
	Central Asia, Middle East and North Africa	310	90	-1.30	-3.49	0.69	0.00	2.21	0.97	3.97	0.00
	East and South East Asia	569	97	1.88	-3.58	4.83	0.00	4.35	2.56	7.19	0.00
	High-income Asia Pacific	*No data from this region were among the held-out data*									
	High-income Western countries	1822	66	0.02	-0.55	0.38	0.00	0.44	0.20	0.97	0.00
	Latin America and Caribbean	379	80	-0.85	-3.07	0.43	0.00	1.40	0.57	3.24	0.00
	Oceania	163	37	0.19	-0.37	0.74	0.00	0.70	0.24	2.17	0.00
	South Asia	330	97	-10.24	-20.77	-1.37	0.00	10.34	4.77	20.84	0.00
	Sub-Saharan Africa	198	97	2.66	-3.88	6.31	0.00	6.09	3.06	9.21	0.00
Urban or rural studies	Rural	469	67	-0.02	-10.05	0.78	0.06	1.79	0.59	10.93	0.06
	Urban	1263	70	-0.11	-1.25	0.45	0.28	0.78	0.32	2.59	0.28
	Both rural and urban	2367	83	-0.09	-1.71	0.77	0.32	1.18	0.32	3.98	0.32
Study representativeness	Community	1698	70	-0.03	-1.53	0.62	0.00	1.00	0.37	3.77	0.00
	Sub-national	626	76	-0.12	-1.37	0.32	0.91	0.69	0.24	2.79	0.91
	National	1775	85	-0.12	-2.00	0.84	0.75	1.33	0.39	4.12	0.75
Age band	18-40	1549	80	-0.21	-2.47	1.04	0.41	1.69	0.60	4.44	0.41
	40-60	1300	75	-0.05	-0.95	0.36	0.52	0.62	0.23	2.98	0.52
	60 and above	1250	77	-0.05	-1.73	0.62	0.27	0.99	0.34	3.73	0.27
Years	1975-1984	233	62	0.27	-0.55	0.65	0.00	0.62	0.32	1.56	0.00
	1985-1994	1057	69	0.13	-0.98	0.69	0.00	0.81	0.33	2.67	0.00
	1995-2004	1423	85	-0.21	-1.93	0.52	0.48	1.16	0.34	4.01	0.48
	2005-2014	1386	79	-0.16	-3.01	0.69	0.35	1.49	0.39	4.91	0.35
Data density	Data poor	159	87	-0.02	-2.85	2.38	0.68	2.58	0.82	6.40	0.68
	Average data density	431	82	0.13	-2.01	1.85	0.25	1.92	0.62	4.91	0.25
	Data rich	3509	76	-0.12	-1.61	0.53	0.04	0.97	0.32	3.46	0.04

† Estimated values minus held out values.

***** p-values for model error comparisons were calculated using the non-parametric Wilcoxon signed-rank test for paired data. The p-values are calculated assuming independence of the held-out observations. They should therefore be interpreted as an approximation because there is some dependence among the held-out observations, within each of the five repetitions for example.

Test 1: Men, BMI 18.5 to <20 kg/m^2

Data		No. of held out observations	Percent covered	Error (percentage points)†				Absolute error			
				Median	Q1	Q3	(p*)	Median	Q1	Q3	(p*)
All		4099	89	-0.21	-2.21	1.21	0.85	1.58	0.65	3.85	0.85
Super-region	Central and Eastern Europe	328	94	-1.01	-2.28	0.49	0.00	1.56	0.77	2.66	0.00
	Central Asia, Middle East and North Africa	310	95	-1.56	-3.87	0.71	0.00	2.41	1.10	4.36	0.00
	East and South East Asia	569	99	0.48	-3.93	3.76	0.05	3.85	1.57	7.21	0.05
	High-income Asia Pacific	*No data from this region were among the held-out data*									
	High-income Western countries	1822	85	-0.06	-1.07	0.86	0.10	0.96	0.41	1.80	0.10
	Latin America and Caribbean	379	87	-1.18	-3.66	0.98	0.01	2.15	1.08	4.21	0.01
	Oceania	163	51	0.43	-0.64	1.79	0.00	1.14	0.52	3.23	0.00
	South Asia	330	98	-1.43	-5.75	1.95	0.00	4.00	1.70	6.91	0.00
	Sub-Saharan Africa	198	100	1.99	-2.78	5.78	0.02	4.88	2.45	8.10	0.02
Urban or rural studies	Rural	469	80	-0.41	-4.85	1.49	0.71	2.32	1.09	5.61	0.71
	Urban	1263	87	-0.32	-1.98	1.15	0.03	1.50	0.66	3.17	0.03
	Both rural and urban	2367	92	-0.13	-2.07	1.17	0.55	1.52	0.60	3.86	0.55
Study representativeness	Community	1698	85	-0.21	-2.13	1.39	0.93	1.71	0.80	4.11	0.93
	Sub-national	626	90	-0.06	-1.60	0.99	0.87	1.22	0.47	2.96	0.87
	National	1775	93	-0.29	-2.48	1.08	0.56	1.60	0.60	3.92	0.56
Age band	18-40	1549	92	-0.52	-3.11	1.58	0.52	2.37	1.05	5.32	0.52
	40-60	1300	89	-0.13	-1.66	1.00	0.21	1.26	0.52	2.88	0.21
	60 and above	1250	85	-0.06	-1.61	1.10	0.69	1.29	0.55	3.04	0.69
Years	1975-1984	233	82	0.31	-1.22	1.39	0.37	1.35	0.66	2.63	0.37
	1985-1994	1057	87	0.04	-1.63	1.41	0.75	1.49	0.67	3.35	0.75
	1995-2004	1423	92	-0.38	-2.41	1.03	0.37	1.60	0.65	3.70	0.37
	2005-2014	1386	89	-0.29	-2.78	1.07	0.64	1.78	0.64	4.51	0.64
Data density	Data poor	159	92	-0.01	-2.65	2.21	0.96	2.61	1.10	5.44	0.96
	Average data density	431	91	0.15	-2.57	2.39	0.83	2.43	1.00	5.22	0.83
	Data rich	3509	89	-0.25	-2.11	1.09	0.83	1.48	0.61	3.55	0.83

† Estimated values minus held out values.

* p-values for model error comparisons were calculated using the non-parametric Wilcoxon signed-rank test for paired data. The p-values are calculated assuming independence of the held-out observations. They should therefore be interpreted as an approximation because there is some dependence among the held-out observations, within each of the five repetitions for example.

Test 1: Men, BMI 20 to <25 kg/m^2

Data		No. of held out observations	Percent covered	Error (percentage points)†				Absolute error			
				Median	Q1	Q3	(p*)	Median	Q1	Q3	(p*)
All		4099	99	1.95	-3.76	8.52	0.00	6.33	2.75	11.41	0.00
Super-region	Central and Eastern Europe	328	100	-3.46	-9.74	3.21	0.01	6.68	3.39	11.67	0.01
	Central Asia, Middle East and North Africa	310	100	0.06	-7.48	9.16	0.70	8.10	4.06	12.59	0.70
	East and South East Asia	569	100	2.02	-2.71	8.04	0.00	5.05	2.49	9.18	0.00
	High-income Asia Pacific	*No data from this region were among the held-out data*									
	High-income Western countries	1822	99	2.01	-2.73	7.93	0.00	5.42	2.30	10.16	0.00
	Latin America and Caribbean	379	99	1.08	-5.80	7.51	0.20	6.66	3.31	11.14	0.20
	Oceania	163	93	4.26	-2.05	14.18	0.00	7.90	3.48	19.68	0.00
	South Asia	330	99	10.11	1.18	19.34	0.00	11.14	5.34	19.38	0.00
	Sub-Saharan Africa	198	99	0.51	-5.29	6.73	0.04	6.25	2.64	11.19	0.04
Urban or rural studies	Rural	469	98	7.77	-0.24	15.78	0.00	9.53	4.55	17.09	0.00
	Urban	1263	99	0.95	-4.92	7.25	0.00	6.21	2.86	11.13	0.00
	Both rural and urban	2367	99	1.66	-3.56	7.81	0.00	5.87	2.59	10.78	0.00
Study representativeness	Community	1698	99	2.48	-3.87	10.12	0.00	7.02	3.19	12.51	0.00
	Sub-national	626	100	2.99	-2.54	8.82	0.00	6.46	2.85	11.60	0.00
	National	1775	99	1.19	-4.01	7.11	0.00	5.56	2.51	10.26	0.00
Age band	18-40	1549	99	1.74	-3.68	9.02	0.00	6.37	2.63	11.90	0.00
	40-60	1300	100	1.29	-4.17	7.70	0.00	6.04	2.50	10.96	0.00
	60 and above	1250	99	2.97	-3.51	9.20	0.00	6.51	3.22	11.58	0.00
Years	1975-1984	233	98	2.24	-3.68	11.23	0.00	6.52	2.92	13.25	0.00
	1985-1994	1057	99	0.61	-5.36	7.87	0.00	6.50	3.07	11.29	0.00
	1995-2004	1423	99	2.38	-3.06	8.63	0.00	6.00	2.72	11.35	0.00
	2005-2014	1386	99	2.28	-3.05	8.75	0.00	6.42	2.64	11.32	0.00
Data density	Data poor	159	99	1.30	-6.76	8.55	0.36	7.60	3.56	12.14	0.36
	Average data density	431	99	0.83	-4.87	7.43	0.09	6.08	2.71	11.17	0.09
	Data rich	3509	99	2.07	-3.52	8.72	0.00	6.30	2.74	11.46	0.00

† Estimated values minus held out values.

***** p-values for model error comparisons were calculated using the non-parametric Wilcoxon signed-rank test for paired data. The p-values are calculated assuming independence of the held-out observations. They should therefore be interpreted as an approximation because there is some dependence among the held-out observations, within each of the five repetitions for example.

Test 1: Men, BMI 25 to <30 kg/m^2

Data		No. of held out observations	Percent covered	Error (percentage points)†				Absolute error			
				Median	Q1	Q3	(p*)	Median	Q1	Q3	(p*)
All		4099	98	0.70	-3.84	5.48	0.21	4.66	2.14	8.45	0.21
Super-region	Central and Eastern Europe	328	99	4.15	-1.16	7.58	0.00	5.88	3.12	8.22	0.00
	Central Asia, Middle East and North Africa	310	98	2.51	-2.27	7.38	0.00	4.99	2.47	9.09	0.00
	East and South East Asia	569	98	-0.86	-6.05	3.12	0.01	4.91	1.92	8.71	0.01
	High-income Asia Pacific	*No data from this region were among the held-out data*									
	High-income Western countries	1822	99	-0.39	-3.81	3.43	0.02	3.64	1.79	6.82	0.02
	Latin America and Caribbean	379	98	2.55	-2.85	8.52	0.00	6.05	2.66	10.37	0.00
	Oceania	163	94	1.28	-7.07	8.99	0.29	8.01	3.98	17.63	0.29
	South Asia	330	96	5.24	0.57	9.84	0.00	7.10	3.16	11.35	0.00
	Sub-Saharan Africa	198	96	-1.27	-6.65	3.31	0.61	5.33	2.51	7.98	0.61
Urban or rural studies	Rural	469	95	2.00	-3.97	8.45	0.05	6.55	2.83	10.98	0.05
	Urban	1263	99	0.72	-4.30	6.16	0.26	5.12	2.24	9.13	0.26
	Both rural and urban	2367	99	0.54	-3.69	4.67	0.66	4.16	1.97	7.60	0.66
Study representativeness	Community	1698	98	0.88	-4.69	6.56	0.36	5.70	2.49	10.21	0.36
	Sub-national	626	99	0.31	-3.77	4.55	0.97	4.17	1.93	7.55	0.97
	National	1775	98	0.68	-3.36	4.98	0.22	4.10	1.96	7.40	0.22
Age band	18-40	1549	97	0.86	-3.71	4.99	0.27	4.35	2.08	7.91	0.27
	40-60	1300	100	0.66	-3.68	5.88	0.53	4.66	2.04	8.59	0.53
	60 and above	1250	98	0.56	-4.23	5.85	0.65	4.98	2.28	9.01	0.65
Years	1975-1984	233	98	-0.66	-5.88	4.69	0.32	5.20	2.33	9.85	0.32
	1985-1994	1057	98	0.60	-4.46	5.22	0.79	4.85	2.05	8.73	0.79
	1995-2004	1423	98	0.70	-3.71	5.87	0.36	4.70	2.24	8.22	0.36
	2005-2014	1386	98	0.95	-3.15	5.33	0.08	4.36	2.03	8.17	0.08
Data density	Data poor	159	98	1.32	-3.85	6.47	0.30	5.17	2.20	10.70	0.30
	Average data density	431	98	-0.64	-5.99	5.42	0.77	5.74	2.93	8.79	0.77
	Data rich	3509	98	0.74	-3.62	5.47	0.31	4.48	2.07	8.26	0.31

† Estimated values minus held out values.

***** p-values for model error comparisons were calculated using the non-parametric Wilcoxon signed-rank test for paired data. The p-values are calculated assuming independence of the held-out observations. They should therefore be interpreted as an approximation because there is some dependence among the held-out observations, within each of the five repetitions for example.

Test 1: Men, BMI 30 to <35 kg/m^2

Data		No. of held out observations	Percent covered	Error (percentage points)†				Absolute error			
				Median	Q1	Q3	(p*)	Median	Q1	Q3	(p*)
All		4099	92	0.07	-2.66	2.17	0.64	2.40	0.90	5.05	0.64
Super-region	Central and Eastern Europe	328	91	2.51	-0.89	5.18	0.00	3.87	2.10	6.30	0.00
	Central Asia, Middle East and North Africa	310	96	1.50	-2.36	4.63	0.03	4.01	1.85	7.55	0.03
	East and South East Asia	569	85	-0.27	-1.48	0.38	0.00	0.83	0.33	1.76	0.00
	High-income Asia Pacific	*No data from this region were among the held-out data*									
	High-income Western countries	1822	96	-0.45	-3.65	2.12	0.00	2.95	1.26	5.44	0.00
	Latin America and Caribbean	379	95	1.25	-2.17	4.27	0.03	3.38	1.65	6.39	0.03
	Oceania	163	85	-1.28	-9.04	3.80	0.12	7.25	3.18	11.39	0.12
	South Asia	330	82	0.39	-0.61	1.03	0.00	0.93	0.44	1.66	0.00
	Sub-Saharan Africa	198	84	-0.30	-1.98	0.53	0.28	1.23	0.39	2.46	0.28
Urban or rural studies	Rural	469	82	-0.07	-3.97	1.23	0.66	2.08	0.81	5.42	0.66
	Urban	1263	93	0.25	-2.83	3.16	0.42	3.02	1.29	5.43	0.42
	Both rural and urban	2367	93	0.06	-2.29	1.91	0.69	2.12	0.77	4.81	0.69
Study representativeness	Community	1698	90	-0.24	-3.67	2.14	0.15	2.88	1.14	5.50	0.15
	Sub-national	626	95	-0.37	-3.78	1.69	0.31	2.64	1.04	6.03	0.31
	National	1775	92	0.22	-1.56	2.37	0.01	1.94	0.66	4.32	0.01
Age band	18-40	1549	86	0.15	-1.56	1.86	0.01	1.72	0.61	3.78	0.01
	40-60	1300	98	0.08	-3.13	2.64	0.76	2.88	1.18	6.06	0.76
	60 and above	1250	93	-0.39	-3.92	2.09	0.07	2.97	1.10	6.22	0.07
Years	1975-1984	233	90	-0.19	-5.05	2.14	0.34	2.85	1.23	7.47	0.34
	1985-1994	1057	91	0.05	-2.78	2.54	0.47	2.64	0.89	5.00	0.47
	1995-2004	1423	92	0.08	-2.23	1.96	0.75	2.09	0.83	4.77	0.75
	2005-2014	1386	93	0.13	-2.73	2.12	0.90	2.40	0.98	5.32	0.90
Data density	Data poor	159	89	-0.09	-2.49	1.70	0.93	2.25	0.76	4.67	0.93
	Average data density	431	91	0.10	-2.69	2.61	0.41	2.63	0.97	5.45	0.41
	Data rich	3509	92	0.07	-2.66	2.13	0.87	2.39	0.90	5.03	0.87

† Estimated values minus held out values.

* p-values for model error comparisons were calculated using the non-parametric Wilcoxon signed-rank test for paired data. The p-values are calculated assuming independence of the held-out observations. They should therefore be interpreted as an approximation because there is some dependence among the held-out observations, within each of the five repetitions for example.

Test 1: Men, BMI 35 to <40 kg/m^2

Data		No. of held out observations	Percent covered	Error (percentage points)[†]				Absolute error			
				Median	Q1	Q3	(p*)	Median	Q1	Q3	(p*)
All		4099	75	0.00	-0.72	0.50	0.00	0.59	0.17	1.40	0.00
Super-region	Central and Eastern Europe	328	77	0.61	-0.38	1.42	0.00	0.98	0.57	1.80	0.00
	Central Asia, Middle East and North Africa	310	87	0.26	-0.97	1.12	0.11	1.09	0.48	1.99	0.11
	East and South East Asia	569	61	-0.02	-0.19	0.01	0.02	0.07	0.01	0.21	0.02
	High-income Asia Pacific	*No data from this region were among the held-out data*									
	High-income Western countries	1822	79	-0.09	-1.06	0.60	0.01	0.79	0.39	1.54	0.01
	Latin America and Caribbean	379	82	-0.01	-1.04	0.52	0.89	0.77	0.32	1.64	0.89
	Oceania	163	71	0.60	-3.47	2.22	0.11	2.68	1.01	6.19	0.11
	South Asia	330	61	0.01	-0.18	0.05	0.05	0.07	0.03	0.24	0.05
	Sub-Saharan Africa	198	62	0.00	-0.45	0.09	0.22	0.18	0.06	0.59	0.22
Urban or rural studies	Rural	469	57	0.01	-0.66	0.25	0.00	0.38	0.08	1.37	0.00
	Urban	1263	73	-0.06	-0.89	0.55	0.12	0.69	0.27	1.45	0.12
	Both rural and urban	2367	79	0.00	-0.62	0.53	0.00	0.57	0.14	1.37	0.00
Study representativeness	Community	1698	69	-0.04	-0.88	0.40	0.00	0.60	0.20	1.40	0.00
	Sub-national	626	84	-0.07	-0.87	0.45	0.86	0.65	0.21	1.39	0.86
	National	1775	76	0.02	-0.50	0.59	0.00	0.57	0.13	1.40	0.00
Age band	18-40	1549	64	0.01	-0.54	0.41	0.00	0.48	0.13	1.05	0.00
	40-60	1300	87	-0.01	-0.80	0.64	0.92	0.73	0.23	1.63	0.92
	60 and above	1250	75	0.00	-0.88	0.53	0.01	0.69	0.19	1.58	0.01
Years	1975-1984	233	73	-0.12	-1.01	0.48	0.77	0.68	0.27	1.49	0.77
	1985-1994	1057	66	0.00	-0.61	0.48	0.00	0.54	0.15	1.14	0.00
	1995-2004	1423	78	-0.02	-0.60	0.42	0.10	0.50	0.15	1.26	0.10
	2005-2014	1386	78	0.02	-0.90	0.66	0.02	0.76	0.20	1.84	0.02
Data density	Data poor	159	69	0.03	-0.67	0.38	0.09	0.53	0.25	1.30	0.09
	Average data density	431	76	0.00	-0.71	0.48	0.16	0.61	0.18	1.45	0.16
	Data rich	3509	75	0.00	-0.72	0.51	0.00	0.59	0.16	1.40	0.00

[†] Estimated values minus held out values.

***** p-values for model error comparisons were calculated using the non-parametric Wilcoxon signed-rank test for paired data. The p-values are calculated assuming independence of the held-out observations. They should therefore be interpreted as an approximation because there is some dependence among the held-out observations, within each of the five repetitions for example.

Test 1: Men, BMI ≥ 40 kg/m^2

Data		No. of held out observations	Percent covered	Error (percentage points)[†]				Absolute error			
				Median	Q1	Q3	(p*)	Median	Q1	Q3	(p*)
All		4099	56	0.01	-0.23	0.13	0.00	0.16	0.04	0.48	0.00
Super-region	Central and Eastern Europe	328	50	0.14	-0.07	0.33	0.00	0.26	0.12	0.54	0.00
	Central Asia, Middle East and North Africa	310	74	0.04	-0.38	0.23	0.49	0.29	0.12	0.58	0.49
	East and South East Asia	569	51	0.00	-0.05	0.00	0.01	0.01	0.00	0.05	0.01
	High-income Asia Pacific	*No data from this region were among the held-out data*									
	High-income Western countries	1822	56	0.04	-0.32	0.18	0.00	0.23	0.08	0.52	0.00
	Latin America and Caribbean	379	68	-0.06	-0.48	0.12	0.99	0.24	0.10	0.68	0.99
	Oceania	163	60	0.21	-1.99	0.99	0.02	1.17	0.43	3.47	0.02
	South Asia	330	49	0.00	-0.08	0.01	0.00	0.02	0.00	0.08	0.00
	Sub-Saharan Africa	198	47	0.01	-0.19	0.05	0.00	0.06	0.02	0.39	0.00
Urban or rural studies	Rural	469	36	0.01	-0.02	0.10	0.00	0.07	0.02	0.30	0.00
	Urban	1263	53	0.00	-0.35	0.10	0.00	0.17	0.05	0.46	0.00
	Both rural and urban	2367	62	0.01	-0.21	0.18	0.00	0.19	0.05	0.53	0.00
Study representativeness	Community	1698	50	0.00	-0.28	0.08	0.00	0.14	0.04	0.38	0.00
	Sub-national	626	63	0.00	-0.35	0.11	0.01	0.20	0.05	0.48	0.01
	National	1775	60	0.02	-0.13	0.24	0.00	0.19	0.04	0.58	0.00
Age band	18-40	1549	47	0.02	-0.11	0.13	0.00	0.12	0.03	0.36	0.00
	40-60	1300	68	0.00	-0.34	0.16	0.00	0.24	0.06	0.60	0.00
	60 and above	1250	56	0.00	-0.28	0.12	0.00	0.17	0.04	0.49	0.00
Years	1975-1984	233	48	0.00	-0.34	0.07	0.00	0.17	0.03	0.58	0.00
	1985-1994	1057	44	0.01	-0.14	0.09	0.00	0.11	0.03	0.29	0.00
	1995-2004	1423	63	0.00	-0.19	0.12	0.00	0.14	0.04	0.41	0.00
	2005-2014	1386	61	0.02	-0.34	0.27	0.00	0.30	0.07	0.75	0.00
Data density	Data poor	159	49	0.03	-0.17	0.25	0.00	0.22	0.06	0.67	0.00
	Average data density	431	60	0.01	-0.42	0.19	0.00	0.29	0.07	0.69	0.00
	Data rich	3509	56	0.01	-0.21	0.12	0.00	0.15	0.04	0.45	0.00

[†] Estimated values minus held out values.

***** p-values for model error comparisons were calculated using the non-parametric Wilcoxon signed-rank test for paired data. The p-values are calculated assuming independence of the held-out observations. They should therefore be interpreted as an approximation because there is some dependence among the held-out observations, within each of the five repetitions for example.

Test 1: Women, mean BMI

Data		No. of held out observations	Percent covered	Error (kg/m²)†				Absolute error			
				Median	Q1	Q3	(p*)	Median	Q1	Q3	(p*)
All		4034	98	-0.02	-0.67	0.64	0.25	0.65	0.31	1.17	0.25
Super-region	Central and Eastern Europe	383	97	0.13	-0.99	1.13	0.60	1.07	0.58	1.50	0.60
	Central Asia, Middle East and North Africa	248	97	0.01	-0.52	0.59	0.92	0.55	0.32	1.10	0.92
	East and South East Asia	155	100	0.09	-0.69	0.74	0.97	0.71	0.32	1.13	0.97
	High-income Asia Pacific	53	100	0.04	-0.46	0.52	0.75	0.49	0.33	0.87	0.75
	High-income Western countries	1858	99	-0.02	-0.83	0.68	0.01	0.74	0.38	1.24	0.01
	Latin America and Caribbean	280	100	-0.07	-0.48	0.34	0.62	0.43	0.20	0.82	0.62
	Oceania	193	84	-0.11	-1.26	1.23	0.89	1.26	0.48	2.56	0.89
	South Asia	371	100	-0.02	-0.35	0.47	0.68	0.42	0.21	0.76	0.68
	Sub-Saharan Africa	493	100	-0.07	-0.39	0.37	0.94	0.38	0.19	0.70	0.94
Urban or rural studies	Rural	410	99	0.03	-0.78	0.85	0.92	0.79	0.36	1.43	0.92
	Urban	1085	99	0.04	-0.68	0.68	0.83	0.68	0.33	1.18	0.83
	Both rural and urban	2539	98	-0.06	-0.66	0.58	0.26	0.62	0.29	1.13	0.26
Study representativeness	Community	1412	99	0.00	-0.78	0.73	0.51	0.75	0.34	1.28	0.51
	Sub-national	644	97	0.01	-0.78	0.74	0.57	0.76	0.35	1.26	0.57
	National	1978	98	-0.05	-0.57	0.53	0.49	0.55	0.27	1.04	0.49
Age band	18-40	1647	99	0.17	-0.39	0.85	0.01	0.61	0.28	1.14	0.01
	40-60	1247	97	0.10	-0.41	0.64	0.26	0.53	0.26	0.87	0.26
	60 and above	1140	98	-0.63	-1.35	0.20	0.00	0.92	0.44	1.56	0.00
Years	1975-1984	218	95	0.12	-0.96	0.89	0.81	0.94	0.42	1.43	0.81
	1985-1994	1066	98	0.01	-0.68	0.70	0.93	0.69	0.32	1.18	0.93
	1995-2004	1379	98	-0.05	-0.67	0.66	0.44	0.66	0.30	1.20	0.44
	2005-2014	1371	98	-0.03	-0.62	0.53	0.27	0.58	0.28	1.09	0.27
Data density	Data poor	180	98	0.02	-0.79	0.53	0.70	0.67	0.29	1.35	0.70
	Average data density	786	98	-0.04	-0.52	0.54	0.94	0.53	0.27	1.17	0.94
	Data rich	3068	98	-0.02	-0.70	0.66	0.19	0.68	0.32	1.16	0.19

† Estimated values minus held out values.

***** p-values for model error comparisons were calculated using the non-parametric Wilcoxon signed-rank test for paired data. The p-values are calculated assuming independence of the held-out observations. They should therefore be interpreted as an approximation because there is some dependence among the held-out observations, within each of the five repetitions for example.

Test 1: Women, BMI < 18.5 kg/m^2

Data		No. of held out observations	Percent covered	Error (percentage points)[†]				Absolute error			
				Median	Q1	Q3	(p*)	Median	Q1	Q3	(p*)
All		4034	87	0.00	-1.71	1.15	0.00	1.37	0.56	3.71	0.00
Super-region	Central and Eastern Europe	383	85	0.08	-1.12	0.65	0.06	0.72	0.31	1.97	0.06
	Central Asia, Middle East and North Africa	248	96	-0.13	-1.98	1.17	0.57	1.42	0.63	2.76	0.57
	East and South East Asia	155	100	-0.85	-6.94	2.60	0.37	3.73	1.73	7.99	0.37
	High-income Asia Pacific	53	100	-1.87	-5.26	-0.39	0.01	2.23	1.09	5.26	0.01
	High-income Western countries	1858	83	0.04	-0.93	0.86	0.00	0.89	0.40	1.79	0.00
	Latin America and Caribbean	280	82	0.43	-0.68	1.68	0.01	1.16	0.52	2.76	0.01
	Oceania	193	55	0.18	-1.10	1.80	0.00	1.40	0.57	3.54	0.00
	South Asia	371	96	-6.96	-17.55	2.62	0.00	10.07	4.47	18.69	0.00
	Sub-Saharan Africa	493	98	0.72	-3.73	4.01	0.19	3.82	1.93	6.72	0.19
Urban or rural studies	Rural	410	78	-1.19	-10.93	1.11	0.64	3.30	1.14	11.51	0.64
	Urban	1085	79	-0.15	-1.49	0.79	0.98	1.10	0.52	2.61	0.98
	Both rural and urban	2539	92	0.10	-1.36	1.34	0.00	1.36	0.52	3.63	0.00
Study representativeness	Community	1412	78	-0.14	-2.09	0.89	0.04	1.38	0.61	3.61	0.04
	Sub-national	644	88	-0.13	-1.21	0.84	0.23	1.03	0.42	2.63	0.23
	National	1978	93	0.13	-1.66	1.49	0.00	1.54	0.57	4.05	0.00
Age band	18-40	1647	91	-0.05	-2.78	1.94	0.04	2.29	1.01	5.07	0.04
	40-60	1247	86	-0.05	-1.13	0.68	0.70	0.83	0.37	2.24	0.70
	60 and above	1140	82	0.11	-1.25	1.05	0.00	1.11	0.46	2.97	0.00
Years	1975-1984	218	79	-0.34	-2.10	0.97	0.71	1.42	0.71	3.18	0.71
	1985-1994	1066	80	0.08	-1.19	0.90	0.01	1.05	0.48	2.46	0.01
	1995-2004	1379	91	0.01	-1.96	1.19	0.01	1.45	0.57	3.61	0.01
	2005-2014	1371	89	-0.03	-2.14	1.37	0.11	1.68	0.63	4.98	0.11
Data density	Data poor	180	82	0.36	-1.62	2.38	0.01	2.12	0.76	3.92	0.01
	Average data density	786	89	0.30	-2.01	2.13	0.19	2.07	0.78	4.68	0.19
	Data rich	3068	86	-0.06	-1.68	0.95	0.00	1.22	0.51	3.33	0.00

[†] Estimated values minus held out values.

* p-values for model error comparisons were calculated using the non-parametric Wilcoxon signed-rank test for paired data. The p-values are calculated assuming independence of the held-out observations. They should therefore be interpreted as an approximation because there is some dependence among the held-out observations, within each of the five repetitions for example.

Test 1: Women, BMI 18.5 to <20 kg/m^2

Data		No. of held out observations	Percent covered	Error (percentage points)[†]				Absolute error			
				Median	Q1	Q3	(p*)	Median	Q1	Q3	(p*)
All		4034	92	0.07	-2.20	1.87	0.01	2.02	0.87	4.15	0.01
Super-region	Central and Eastern Europe	383	96	0.32	-1.93	1.20	0.05	1.40	0.67	2.67	0.05
	Central Asia, Middle East and North Africa	248	96	-0.22	-1.71	1.08	0.24	1.35	0.66	2.92	0.24
	East and South East Asia	155	99	0.30	-3.81	3.17	0.57	3.42	1.48	5.70	0.57
	High-income Asia Pacific	53	94	0.59	-2.62	2.32	0.13	2.62	1.01	5.52	0.13
	High-income Western countries	1858	91	-0.04	-1.88	1.56	0.00	1.74	0.76	3.34	0.00
	Latin America and Caribbean	280	91	0.38	-1.17	2.22	0.08	1.79	0.79	3.30	0.08
	Oceania	193	59	0.26	-1.74	1.77	0.01	1.77	0.56	4.03	0.01
	South Asia	371	98	0.76	-3.02	4.89	0.05	3.91	1.83	6.69	0.05
	Sub-Saharan Africa	493	100	-0.56	-4.14	3.37	0.70	3.64	1.80	6.26	0.70
Urban or rural studies	Rural	410	86	-0.53	-3.53	1.92	0.59	2.59	1.23	5.16	0.59
	Urban	1085	90	-0.44	-2.79	1.60	0.18	2.16	0.95	4.14	0.18
	Both rural and urban	2539	94	0.34	-1.69	2.01	0.00	1.87	0.80	4.02	0.00
Study representativeness	Community	1412	88	-0.13	-2.68	1.88	0.37	2.32	1.04	4.44	0.37
	Sub-national	644	92	0.03	-2.14	1.75	0.28	1.86	0.83	3.63	0.28
	National	1978	95	0.23	-1.78	1.92	0.02	1.88	0.77	4.10	0.02
Age band	18-40	1647	96	-0.10	-3.39	2.92	0.83	3.06	1.37	5.86	0.83
	40-60	1247	92	0.05	-1.65	1.43	0.99	1.51	0.63	2.98	0.99
	60 and above	1140	87	0.27	-1.45	1.62	0.00	1.57	0.72	3.12	0.00
Years	1975-1984	218	84	-0.67	-3.31	1.57	0.81	2.37	1.10	4.64	0.81
	1985-1994	1066	89	0.08	-2.18	1.71	0.39	1.90	0.91	3.60	0.39
	1995-2004	1379	94	0.25	-2.14	1.88	0.02	1.96	0.79	4.02	0.02
	2005-2014	1371	94	0.00	-2.13	2.17	0.18	2.15	0.91	4.58	0.18
Data density	Data poor	180	84	0.53	-1.88	2.92	0.11	2.43	0.89	4.85	0.11
	Average data density	786	94	-0.11	-2.31	2.23	0.96	2.28	1.00	4.60	0.96
	Data rich	3068	92	0.07	-2.17	1.79	0.01	1.93	0.84	4.03	0.01

[†] Estimated values minus held out values.

* p-values for model error comparisons were calculated using the non-parametric Wilcoxon signed-rank test for paired data. The p-values are calculated assuming independence of the held-out observations. They should therefore be interpreted as an approximation because there is some dependence among the held-out observations, within each of the five repetitions for example.

Test 1: Women, BMI 20 to <25 kg/m^2

Data		No. of held out observations	Percent covered	Error (percentage points)†				Absolute error			
				Median	Q1	Q3	(p*)	Median	Q1	Q3	(p*)
All		4034	99	1.80	-4.05	8.40	0.00	6.19	2.85	11.69	0.00
Super-region	Central and Eastern Europe	383	100	3.99	-1.74	9.34	0.00	6.28	2.77	9.92	0.00
	Central Asia, Middle East and North Africa	248	100	0.74	-4.13	5.61	0.95	4.81	2.39	8.11	0.95
	East and South East Asia	155	99	-0.61	-5.82	4.52	0.79	5.08	2.50	8.46	0.79
	High-income Asia Pacific	53	100	7.98	2.50	15.63	0.00	8.26	3.13	15.63	0.00
	High-income Western countries	1858	99	1.13	-4.56	8.03	0.00	6.18	2.89	11.83	0.00
	Latin America and Caribbean	280	100	1.51	-3.43	7.42	0.08	5.46	2.30	10.70	0.08
	Oceania	193	94	6.19	-3.11	14.21	0.00	9.32	4.71	17.38	0.00
	South Asia	371	98	7.35	0.22	15.75	0.00	9.08	4.12	17.72	0.00
	Sub-Saharan Africa	493	100	-0.37	-4.93	5.13	0.98	5.06	2.46	8.95	0.98
Urban or rural studies	Rural	410	97	6.29	-1.04	16.00	0.00	8.93	4.09	18.02	0.00
	Urban	1085	99	0.28	-6.61	7.70	0.53	7.34	3.61	13.19	0.53
	Both rural and urban	2539	99	1.82	-3.22	7.76	0.00	5.46	2.48	10.05	0.00
Study representativeness	Community	1412	99	2.45	-4.77	10.76	0.00	7.56	3.62	13.95	0.00
	Sub-national	644	99	0.59	-6.23	8.41	0.22	7.05	3.45	13.20	0.22
	National	1978	99	1.80	-2.87	7.30	0.00	5.14	2.33	9.39	0.00
Age band	18-40	1647	99	1.40	-4.34	8.24	0.00	6.02	2.72	11.73	0.00
	40-60	1247	100	1.19	-4.37	7.28	0.00	5.95	2.77	10.93	0.00
	60 and above	1140	99	3.22	-3.16	9.86	0.00	6.70	3.20	12.79	0.00
Years	1975-1984	218	99	0.89	-5.69	12.58	0.17	8.08	4.16	14.79	0.17
	1985-1994	1066	99	0.84	-5.15	8.31	0.07	6.50	3.21	12.37	0.07
	1995-2004	1379	99	2.31	-3.32	8.91	0.00	6.28	2.76	11.88	0.00
	2005-2014	1371	99	2.09	-3.47	7.74	0.00	5.67	2.55	10.25	0.00
Data density	Data poor	180	100	2.51	-3.17	9.98	0.02	6.42	2.81	12.29	0.02
	Average data density	786	99	1.28	-4.48	7.15	0.14	6.13	2.67	11.04	0.14
	Data rich	3068	99	1.87	-3.95	8.71	0.00	6.19	2.91	11.80	0.00

† Estimated values minus held out values.

* p-values for model error comparisons were calculated using the non-parametric Wilcoxon signed-rank test for paired data. The p-values are calculated assuming independence of the held-out observations. They should therefore be interpreted as an approximation because there is some dependence among the held-out observations, within each of the five repetitions for example.

Test 1: Women, BMI 25 to <30 kg/m^2

Data		No. of held out observations	Percent covered	Error (percentage points)†				Absolute error			
				Median	Q1	Q3	(p*)	Median	Q1	Q3	(p*)
All		4034	98	0.16	-4.04	5.08	0.59	4.55	2.09	8.44	0.59
Super-region	Central and Eastern Europe	383	99	1.05	-2.14	4.40	0.99	3.35	1.52	6.03	0.99
	Central Asia, Middle East and North Africa	248	100	0.41	-3.58	5.63	0.22	4.82	2.05	7.53	0.22
	East and South East Asia	155	99	2.31	-3.83	7.50	0.06	6.04	3.17	9.47	0.06
	High-income Asia Pacific	53	100	-1.13	-5.32	1.64	0.11	3.48	1.29	5.62	0.11
	High-income Western countries	1858	99	-0.76	-4.29	4.16	0.00	4.25	2.06	7.75	0.00
	Latin America and Caribbean	280	98	-0.84	-5.07	3.45	0.54	4.49	1.89	8.24	0.54
	Oceania	193	96	3.73	-4.00	14.18	0.00	10.48	3.95	17.50	0.00
	South Asia	371	95	1.75	-4.68	6.80	0.00	6.24	2.73	10.95	0.00
	Sub-Saharan Africa	493	99	1.39	-3.56	5.37	0.03	4.72	2.20	8.94	0.03
Urban or rural studies	Rural	410	91	2.86	-3.06	8.77	0.00	6.26	2.92	11.25	0.00
	Urban	1085	99	1.35	-3.28	6.78	0.02	5.24	2.33	9.36	0.02
	Both rural and urban	2539	99	-0.62	-4.33	3.91	0.05	4.14	1.96	7.56	0.05
Study representativeness	Community	1412	97	1.22	-3.67	7.01	0.05	5.43	2.39	9.84	0.05
	Sub-national	644	99	-0.07	-4.16	5.03	0.99	4.62	2.39	7.94	0.99
	National	1978	99	-0.49	-4.07	3.91	0.30	4.04	1.88	7.48	0.30
Age band	18-40	1647	97	0.76	-3.57	5.43	0.01	4.57	2.11	8.50	0.01
	40-60	1247	100	0.15	-3.77	4.91	0.79	4.31	2.08	8.02	0.79
	60 and above	1140	98	-0.72	-4.65	4.82	0.00	4.74	2.11	8.94	0.00
Years	1975-1984	218	99	2.11	-3.69	7.96	0.08	5.64	2.76	10.62	0.08
	1985-1994	1066	99	0.72	-3.56	5.72	0.27	4.59	2.18	8.73	0.27
	1995-2004	1379	97	0.03	-4.14	5.08	0.75	4.61	2.13	8.21	0.75
	2005-2014	1371	99	-0.38	-4.26	4.45	0.44	4.36	1.93	8.18	0.44
Data density	Data poor	180	96	0.14	-5.90	6.61	0.85	6.49	2.90	10.89	0.85
	Average data density	786	99	0.49	-4.14	5.04	0.12	4.61	2.18	8.77	0.12
	Data rich	3068	98	-0.02	-3.90	4.98	0.66	4.44	2.05	8.28	0.66

† Estimated values minus held out values.

* p-values for model error comparisons were calculated using the non-parametric Wilcoxon signed-rank test for paired data. The p-values are calculated assuming independence of the held-out observations. They should therefore be interpreted as an approximation because there is some dependence among the held-out observations, within each of the five repetitions for example.

Test 1: Women, BMI 30 to <35 kg/m²

Data		No. of held out observations	Percent covered	Error (percentage points)†				Absolute error			
				Median	Q1	Q3	(p*)	Median	Q1	Q3	(p*)
All		4034	94	0.23	-3.23	2.85	0.34	2.99	1.30	5.68	0.34
Super-region	Central and Eastern Europe	383	96	0.13	-4.94	2.80	0.15	3.52	1.78	5.94	0.15
	Central Asia, Middle East and North Africa	248	96	0.58	-3.26	3.01	0.39	3.16	1.45	6.08	0.39
	East and South East Asia	155	95	0.98	-0.96	3.95	0.00	2.16	0.97	4.25	0.00
	High-income Asia Pacific	53	92	-0.64	-3.59	0.15	0.02	1.25	0.41	3.64	0.02
	High-income Western countries	1858	96	0.33	-3.55	3.14	0.45	3.32	1.49	6.08	0.45
	Latin America and Caribbean	280	96	-0.96	-3.63	1.89	0.71	2.98	1.44	5.41	0.71
	Oceania	193	91	-0.52	-4.83	4.48	0.77	4.77	1.99	10.27	0.77
	South Asia	371	89	0.28	-1.53	2.19	0.00	1.98	0.76	3.88	0.00
	Sub-Saharan Africa	493	90	0.35	-1.97	2.42	0.06	2.22	0.66	4.67	0.06
Urban or rural studies	Rural	410	84	0.60	-3.34	2.70	0.03	2.99	1.34	6.12	0.03
	Urban	1085	95	0.94	-3.65	4.30	0.01	4.09	1.91	6.93	0.01
	Both rural and urban	2539	96	-0.02	-3.06	2.27	0.53	2.58	1.11	5.04	0.53
Study representativeness	Community	1412	92	0.36	-4.18	3.43	0.38	3.75	1.65	6.81	0.38
	Sub-national	644	96	0.60	-3.57	3.86	0.33	3.72	1.61	6.77	0.33
	National	1978	95	0.12	-2.52	2.20	0.87	2.34	1.00	4.63	0.87
Age band	18-40	1647	90	0.21	-2.04	1.98	0.16	2.02	0.83	3.93	0.16
	40-60	1247	99	0.65	-3.44	3.53	0.34	3.51	1.65	6.20	0.34
	60 and above	1140	96	-0.28	-5.32	3.69	0.00	4.42	1.99	7.85	0.00
Years	1975-1984	218	94	0.32	-5.15	4.28	0.78	4.42	2.07	8.19	0.78
	1985-1994	1066	95	0.61	-3.22	3.13	0.18	3.14	1.29	5.89	0.18
	1995-2004	1379	93	0.17	-3.19	2.56	0.71	2.82	1.25	5.45	0.71
	2005-2014	1371	95	0.02	-3.15	2.62	0.67	2.86	1.27	5.36	0.67
Data density	Data poor	180	93	-0.18	-5.25	2.52	0.40	3.66	1.89	7.96	0.40
	Average data density	786	93	-0.08	-3.42	2.89	0.28	3.17	1.21	5.82	0.28
	Data rich	3068	95	0.30	-2.99	2.85	0.50	2.90	1.30	5.59	0.50

† Estimated values minus held out values.

* p-values for model error comparisons were calculated using the non-parametric Wilcoxon signed-rank test for paired data. The p-values are calculated assuming independence of the held-out observations. They should therefore be interpreted as an approximation because there is some dependence among the held-out observations, within each of the five repetitions for example.

Test 1: Women, BMI 35 to <40 kg/m²

Data		No. of held out observations	Percent covered	Error (percentage points)†				Absolute error			
				Median	Q1	Q3	(p*)	Median	Q1	Q3	(p*)
All		4034	85	0.06	-1.40	1.09	0.10	1.20	0.42	2.72	0.10
Super-region	Central and Eastern Europe	383	89	-0.50	-2.84	0.66	0.01	1.47	0.63	3.23	0.01
	Central Asia, Middle East and North Africa	248	93	0.36	-1.34	1.75	0.08	1.57	0.67	3.42	0.08
	East and South East Asia	155	79	0.07	-0.23	0.42	0.02	0.34	0.14	0.78	0.02
	High-income Asia Pacific	53	83	-0.36	-1.26	-0.02	0.00	0.41	0.20	1.26	0.00
	High-income Western countries	1858	87	0.25	-1.48	1.51	0.89	1.50	0.67	2.83	0.89
	Latin America and Caribbean	280	91	0.07	-1.16	1.42	0.61	1.32	0.52	2.80	0.61
	Oceania	193	85	-0.52	-8.54	3.19	0.12	5.64	2.23	10.31	0.12
	South Asia	371	75	0.01	-0.54	0.24	0.24	0.35	0.10	0.80	0.24
	Sub-Saharan Africa	493	79	0.03	-0.72	0.52	0.10	0.59	0.15	1.73	0.10
Urban or rural studies	Rural	410	70	0.07	-1.02	0.49	0.00	0.65	0.20	2.22	0.00
	Urban	1085	86	0.32	-1.79	1.71	0.02	1.72	0.78	3.24	0.02
	Both rural and urban	2539	88	0.02	-1.36	0.92	0.90	1.09	0.38	2.49	0.90
Study representativeness	Community	1412	83	0.08	-1.90	1.14	0.33	1.41	0.52	2.96	0.33
	Sub-national	644	89	0.17	-1.43	1.54	0.13	1.47	0.63	3.17	0.13
	National	1978	86	0.04	-1.09	0.91	0.42	0.98	0.34	2.39	0.42
Age band	18-40	1647	74	0.04	-0.79	0.65	0.00	0.70	0.24	1.66	0.00
	40-60	1247	96	0.16	-1.82	1.60	0.73	1.69	0.66	3.27	0.73
	60 and above	1140	90	0.04	-2.33	1.37	0.37	1.72	0.67	3.49	0.37
Years	1975-1984	218	85	0.11	-2.64	1.32	0.39	1.77	0.76	4.62	0.39
	1985-1994	1066	83	0.06	-1.64	1.06	0.34	1.22	0.45	2.75	0.34
	1995-2004	1379	86	0.04	-1.30	0.89	0.28	1.04	0.35	2.46	0.28
	2005-2014	1371	86	0.11	-1.30	1.28	0.29	1.29	0.45	2.77	0.29
Data density	Data poor	180	87	-0.09	-3.10	1.01	0.50	1.74	0.56	4.73	0.50
	Average data density	786	85	0.07	-1.40	1.17	0.07	1.24	0.41	3.22	0.07
	Data rich	3068	85	0.07	-1.34	1.05	0.33	1.17	0.42	2.57	0.33

† Estimated values minus held out values.

* p-values for model error comparisons were calculated using the non-parametric Wilcoxon signed-rank test for paired data. The p-values are calculated assuming independence of the held-out observations. They should therefore be interpreted as an approximation because there is some dependence among the held-out observations, within each of the five repetitions for example.

Test 1: Women, BMI ≥ 40 kg/m^2

Data		No. of held out observations	Percent covered	Error (percentage points)[†]				Absolute error			
				Median	Q1	Q3	(p*)	Median	Q1	Q3	(p*)
All		4034	75	0.03	-0.72	0.44	0.00	0.55	0.17	1.42	0.00
Super-region	Central and Eastern Europe	383	80	-0.23	-1.33	0.19	0.01	0.64	0.20	1.49	0.01
	Central Asia, Middle East and North Africa	248	87	0.03	-1.30	0.78	0.90	0.99	0.33	2.08	0.90
	East and South East Asia	155	62	0.01	-0.08	0.12	0.00	0.10	0.04	0.26	0.00
	High-income Asia Pacific	53	60	-0.19	-0.40	0.01	0.03	0.19	0.03	0.40	0.03
	High-income Western countries	1858	75	0.11	-0.76	0.69	0.62	0.71	0.29	1.50	0.62
	Latin America and Caribbean	280	83	-0.03	-0.78	0.62	0.84	0.67	0.23	1.41	0.84
	Oceania	193	74	-0.38	-10.21	1.04	0.03	3.32	0.75	10.21	0.03
	South Asia	371	62	0.01	-0.14	0.07	0.00	0.08	0.03	0.25	0.00
	Sub-Saharan Africa	493	73	0.02	-0.43	0.32	0.01	0.35	0.08	1.02	0.01
Urban or rural studies	Rural	410	48	0.03	-0.48	0.26	0.00	0.33	0.06	1.17	0.00
	Urban	1085	73	0.10	-0.78	0.60	0.00	0.67	0.26	1.48	0.00
	Both rural and urban	2539	80	0.01	-0.71	0.40	0.48	0.55	0.15	1.42	0.48
Study representativeness	Community	1412	69	0.03	-0.90	0.40	0.01	0.57	0.19	1.40	0.01
	Sub-national	644	80	0.08	-0.63	0.72	0.01	0.68	0.22	1.67	0.01
	National	1978	77	0.02	-0.61	0.39	0.10	0.50	0.13	1.37	0.10
Age band	18-40	1647	62	0.02	-0.42	0.22	0.00	0.28	0.08	0.84	0.00
	40-60	1247	89	0.03	-0.91	0.73	0.79	0.80	0.33	1.84	0.79
	60 and above	1140	77	0.03	-0.99	0.64	0.29	0.78	0.29	1.77	0.29
Years	1975-1984	218	68	0.04	-0.69	0.48	0.11	0.61	0.24	1.70	0.11
	1985-1994	1066	71	0.02	-0.74	0.37	0.02	0.52	0.17	1.17	0.02
	1995-2004	1379	76	0.02	-0.63	0.39	0.11	0.49	0.13	1.30	0.11
	2005-2014	1371	77	0.04	-0.82	0.60	0.05	0.68	0.19	1.73	0.05
Data density	Data poor	180	72	0.06	-1.29	0.78	0.11	0.82	0.31	2.33	0.11
	Average data density	786	76	0.02	-0.75	0.40	0.04	0.54	0.13	1.65	0.04
	Data rich	3068	74	0.03	-0.70	0.43	0.01	0.55	0.17	1.35	0.01

[†] Estimated values minus held out values.

* p-values for model error comparisons were calculated using the non-parametric Wilcoxon signed-rank test for paired data. The p-values are calculated assuming independence of the held-out observations. They should therefore be interpreted as an approximation because there is some dependence among the held-out observations, within each of the five repetitions for example.

Test 2: Men, mean BMI

Data		No. of held out observations	Percent covered	Error (kg/m^2)[†]				Absolute error			
				Median	Q1	Q3	(p*)	Median	Q1	Q3	(p*)
All		3530	95	-0.01	-0.30	0.24	0.34	0.27	0.12	0.54	0.34
Super-region	Central and Eastern Europe	292	97	0.00	-0.27	0.22	0.78	0.24	0.10	0.50	0.78
	Central Asia, Middle East and North Africa	322	93	-0.01	-0.34	0.39	0.90	0.37	0.18	0.74	0.90
	East and South East Asia	376	94	0.00	-0.22	0.22	0.84	0.22	0.11	0.41	0.84
	High-income Asia Pacific	354	97	0.01	-0.19	0.18	0.60	0.18	0.08	0.37	0.60
	High-income Western countries	1140	95	-0.01	-0.28	0.23	0.38	0.25	0.10	0.49	0.38
	Latin America and Caribbean	426	96	0.00	-0.33	0.29	0.48	0.31	0.14	0.64	0.48
	Oceania	133	87	-0.05	-0.63	0.48	0.75	0.56	0.25	1.14	0.75
	South Asia	210	90	0.00	-0.35	0.27	0.97	0.29	0.15	0.55	0.97
	Sub-Saharan Africa	277	95	-0.08	-0.40	0.28	0.55	0.33	0.18	0.65	0.55
Urban or rural studies	Rural	455	93	-0.04	-0.42	0.29	0.50	0.34	0.15	0.65	0.50
	Urban	822	94	-0.02	-0.37	0.29	0.28	0.32	0.14	0.62	0.28
	Both rural and urban	2253	95	0.00	-0.26	0.23	0.69	0.24	0.11	0.48	0.69
Study representativeness	Community	1279	94	-0.03	-0.41	0.29	0.20	0.33	0.14	0.66	0.20
	Sub-national	466	94	0.02	-0.29	0.24	0.96	0.25	0.13	0.48	0.96
	National	1785	96	-0.01	-0.25	0.23	0.78	0.24	0.10	0.48	0.78
Age band	18-40	1287	93	-0.03	-0.33	0.24	0.27	0.28	0.13	0.58	0.27
	40-60	1043	97	0.02	-0.21	0.23	0.98	0.22	0.11	0.43	0.98
	60 and above	1200	94	-0.02	-0.35	0.26	0.35	0.30	0.12	0.62	0.35
Years	1975-1984	136	100	-0.02	-0.21	0.14	0.87	0.19	0.08	0.38	0.87
	1985-1994	569	97	-0.01	-0.28	0.22	0.67	0.23	0.11	0.49	0.67
	1995-2004	1246	94	0.01	-0.28	0.26	0.69	0.27	0.12	0.54	0.69
	2005-2014	1579	94	-0.01	-0.33	0.25	0.38	0.28	0.13	0.58	0.38
Data density	Data poor	*Not applicable for male data in source-based test*									
	Average data density	460	95	-0.03	-0.37	0.34	0.80	0.36	0.17	0.69	0.80
	Data rich	3070	95	-0.01	-0.28	0.24	0.37	0.26	0.11	0.51	0.37
Hold out pattern	Post-2000 data removed	1183	95	-0.02	-0.29	0.24	0.55	0.27	0.11	0.54	0.55
	Random set of data removed	2347	94	0.00	-0.30	0.24	0.44	0.27	0.12	0.53	0.44

[†] Estimated values minus held out values.

***** p-values for model error comparisons were calculated using the non-parametric Wilcoxon signed-rank test for paired data. The p-values are calculated assuming independence of the held-out observations. They should therefore be interpreted as an approximation because there is some dependence among the held-out observations, within each of the five repetitions for example.

Test 2: Men, BMI < 18.5 kg/m^2

Data		No. of held out observations	Percent covered	Error (percentage points)[†]				Absolute error			
				Median	Q1	Q3	(p*)	Median	Q1	Q3	(p*)
All		3530	81	-0.10	-1.36	0.67	0.09	1.02	0.32	3.34	0.09
Super-region	Central and Eastern Europe	292	70	-0.24	-1.23	0.27	0.66	0.61	0.26	1.54	0.66
	Central Asia, Middle East and North Africa	322	83	0.01	-1.28	0.90	0.22	1.14	0.48	2.64	0.22
	East and South East Asia	376	98	-1.11	-4.09	1.42	0.34	3.08	1.19	5.35	0.34
	High-income Asia Pacific	354	96	-0.30	-2.17	1.35	0.42	1.69	0.80	3.62	0.42
	High-income Western countries	1140	71	-0.10	-0.63	0.21	0.80	0.38	0.17	0.86	0.80
	Latin America and Caribbean	426	80	-0.05	-1.39	0.69	0.96	0.99	0.36	2.74	0.96
	Oceania	133	44	0.04	-1.94	0.45	0.00	0.86	0.21	3.26	0.00
	South Asia	210	98	0.57	-8.79	9.07	0.45	8.87	4.58	14.81	0.45
	Sub-Saharan Africa	277	97	1.50	-2.39	5.19	0.00	3.88	1.73	7.91	0.00
Urban or rural studies	Rural	455	71	-0.11	-4.55	0.84	0.06	1.77	0.44	5.65	0.06
	Urban	822	75	-0.16	-1.20	0.50	0.88	0.80	0.31	2.77	0.88
	Both rural and urban	2253	85	-0.08	-1.21	0.77	0.28	0.99	0.31	3.06	0.28
Study representativeness	Community	1279	72	-0.15	-1.66	0.53	0.03	0.98	0.34	3.51	0.03
	Sub-national	466	78	0.01	-1.09	0.70	0.61	0.85	0.24	3.92	0.61
	National	1785	88	-0.11	-1.30	0.83	0.32	1.07	0.35	3.04	0.32
Age band	18-40	1287	84	-0.22	-2.09	1.02	0.47	1.56	0.56	4.21	0.47
	40-60	1043	79	-0.07	-0.82	0.35	0.84	0.61	0.20	1.93	0.84
	60 and above	1200	80	-0.06	-1.40	0.62	0.25	0.95	0.29	3.21	0.25
Years	1975-1984	136	88	-0.17	-1.02	0.52	0.11	0.84	0.36	1.81	0.11
	1985-1994	569	75	0.05	-1.10	0.63	0.14	0.82	0.32	2.39	0.14
	1995-2004	1246	84	-0.21	-1.33	0.46	0.70	0.90	0.30	2.87	0.70
	2005-2014	1579	80	-0.06	-1.54	0.91	0.03	1.26	0.34	4.03	0.03
Data density	Data poor	*Not applicable for male data in source-based test*									
	Average data density	460	81	0.20	-1.31	2.73	0.02	2.00	0.67	5.64	0.02
	Data rich	3070	81	-0.14	-1.37	0.53	0.38	0.91	0.30	3.03	0.38
Hold out pattern	Post-2000 data removed	1183	80	-0.06	-1.18	0.89	0.07	1.03	0.34	2.82	0.07
	Random set of data removed	2347	81	-0.13	-1.47	0.56	0.55	1.01	0.31	3.55	0.55

[†] Estimated values minus held out values.

***** p-values for model error comparisons were calculated using the non-parametric Wilcoxon signed-rank test for paired data. The p-values are calculated assuming independence of the held-out observations. They should therefore be interpreted as an approximation because there is some dependence among the held-out observations, within each of the five repetitions for example.

Test 2: Men, BMI 18.5 to <20 kg/m^2

Data		No. of held out observations	Percent covered	Error (percentage points)[†]				Absolute error			
				Median	Q1	Q3	(p*)	Median	Q1	Q3	(p*)
All		3530	90	-0.24	-2.04	1.06	0.90	1.52	0.61	3.65	0.90
Super-region	Central and Eastern Europe	292	88	-0.22	-1.64	0.83	0.59	1.18	0.57	2.42	0.59
	Central Asia, Middle East and North Africa	322	89	-0.28	-2.00	1.39	0.93	1.77	0.73	3.26	0.93
	East and South East Asia	376	100	-1.60	-4.47	1.51	0.04	3.31	1.59	5.56	0.04
	High-income Asia Pacific	354	97	-0.13	-2.61	1.58	0.54	1.97	0.80	4.61	0.54
	High-income Western countries	1140	85	-0.21	-1.02	0.58	0.39	0.82	0.37	1.60	0.39
	Latin America and Caribbean	426	88	-0.11	-2.31	1.21	0.99	1.68	0.69	3.14	0.99
	Oceania	133	56	0.19	-1.08	1.05	0.01	1.06	0.36	3.35	0.01
	South Asia	210	99	-1.11	-5.52	2.51	0.05	4.18	1.78	6.87	0.05
	Sub-Saharan Africa	277	99	0.74	-3.34	4.30	0.14	3.90	1.83	6.98	0.14
Urban or rural studies	Rural	455	82	-0.39	-4.43	1.31	0.67	2.45	0.97	6.76	0.67
	Urban	822	87	-0.39	-1.97	0.94	0.39	1.48	0.63	3.22	0.39
	Both rural and urban	2253	92	-0.16	-1.76	1.06	0.64	1.39	0.56	3.43	0.64
Study representativeness	Community	1279	85	-0.30	-2.35	1.13	0.74	1.68	0.75	3.99	0.74
	Sub-national	466	90	-0.16	-1.96	0.86	1.00	1.21	0.49	3.90	1.00
	National	1785	93	-0.21	-1.88	1.06	0.81	1.46	0.57	3.45	0.81
Age band	18-40	1287	92	-0.39	-2.91	1.69	0.97	2.21	0.92	5.30	0.97
	40-60	1043	90	-0.18	-1.52	0.80	0.14	1.08	0.47	2.73	0.14
	60 and above	1200	86	-0.21	-1.79	0.94	0.75	1.27	0.50	3.02	0.75
Years	1975-1984	136	90	-0.08	-1.20	1.10	0.56	1.18	0.45	2.68	0.56
	1985-1994	569	88	-0.14	-1.48	1.00	0.95	1.25	0.53	3.08	0.95
	1995-2004	1246	91	-0.29	-2.09	0.89	0.82	1.41	0.59	3.33	0.82
	2005-2014	1579	89	-0.24	-2.35	1.27	0.66	1.78	0.67	4.21	0.66
Data density	Data poor	*Not applicable for male data in source-based test*									
	Average data density	460	88	0.27	-2.43	2.76	0.25	2.55	0.89	5.59	0.25
	Data rich	3070	90	-0.28	-2.01	0.92	0.78	1.40	0.58	3.35	0.78
Hold out pattern	Post-2000 data removed	1183	89	-0.02	-1.77	1.55	0.09	1.65	0.63	3.36	0.09
	Random set of data removed	2347	90	-0.36	-2.24	0.89	0.27	1.43	0.60	3.81	0.27

[†] Estimated values minus held out values.

***** p-values for model error comparisons were calculated using the non-parametric Wilcoxon signed-rank test for paired data. The p-values are calculated assuming independence of the held-out observations. They should therefore be interpreted as an approximation because there is some dependence among the held-out observations, within each of the five repetitions for example.

Test 2: Men, BMI 20 to <25 kg/m^2

Data		No. of held out observations	Percent covered	Error (percentage points)[†]				Absolute error			
				Median	Q1	Q3	(p*)	Median	Q1	Q3	(p*)
All		3530	99	1.38	-3.51	7.10	0.00	5.25	2.33	10.16	0.00
Super-region	Central and Eastern Europe	292	100	0.41	-4.18	6.41	0.15	5.34	2.17	9.31	0.15
	Central Asia, Middle East and North Africa	322	98	2.22	-4.32	7.96	0.03	5.86	2.98	11.68	0.03
	East and South East Asia	376	100	1.96	-2.89	8.46	0.00	5.10	2.53	9.60	0.00
	High-income Asia Pacific	354	100	-0.26	-2.65	4.26	0.03	2.96	1.46	6.41	0.03
	High-income Western countries	1140	99	1.32	-3.13	6.76	0.00	4.75	2.23	9.50	0.00
	Latin America and Caribbean	426	99	1.14	-4.42	6.50	0.10	5.41	2.53	10.00	0.10
	Oceania	133	93	1.71	-7.52	10.25	0.37	9.16	3.86	19.80	0.37
	South Asia	210	100	4.72	-2.87	13.26	0.00	8.74	4.23	14.69	0.00
	Sub-Saharan Africa	277	99	1.97	-3.10	8.11	0.00	6.05	2.50	10.56	0.00
Urban or rural studies	Rural	455	98	4.70	-1.66	12.97	0.00	7.88	3.81	14.94	0.00
	Urban	822	99	0.06	-5.33	6.08	0.10	5.80	2.62	10.33	0.10
	Both rural and urban	2253	99	1.27	-3.02	6.53	0.00	4.77	2.10	9.15	0.00
Study representativeness	Community	1279	99	1.72	-3.90	9.06	0.00	6.25	2.78	11.92	0.00
	Sub-national	466	99	1.25	-4.25	6.68	0.54	5.74	2.71	10.31	0.54
	National	1785	99	1.25	-2.91	6.25	0.00	4.69	2.01	8.84	0.00
Age band	18-40	1287	99	2.57	-2.29	8.74	0.00	5.64	2.40	11.38	0.00
	40-60	1043	100	-0.06	-4.29	5.20	0.35	4.81	2.15	9.30	0.35
	60 and above	1200	99	1.30	-3.85	7.06	0.00	5.37	2.35	10.01	0.00
Years	1975-1984	136	99	-0.76	-3.62	4.67	0.66	4.00	1.52	8.99	0.66
	1985-1994	569	99	-0.07	-3.90	5.91	0.12	4.73	2.11	9.60	0.12
	1995-2004	1246	99	1.53	-3.28	7.16	0.00	5.29	2.37	9.82	0.00
	2005-2014	1579	99	1.86	-3.17	7.78	0.00	5.54	2.48	10.69	0.00
Data density	Data poor	*Not applicable for male data in source-based test*									
	Average data density	460	98	1.38	-6.41	7.78	0.21	7.24	3.11	12.22	0.21
	Data rich	3070	99	1.38	-3.13	7.02	0.00	5.08	2.23	9.72	0.00
Hold out pattern	Post-2000 data removed	1183	99	1.30	-2.91	7.25	0.00	5.12	2.03	9.75	0.00
	Random set of data removed	2347	99	1.40	-3.73	7.05	0.00	5.35	2.47	10.32	0.00

[†] Estimated values minus held out values.

***** p-values for model error comparisons were calculated using the non-parametric Wilcoxon signed-rank test for paired data. The p-values are calculated assuming independence of the held-out observations. They should therefore be interpreted as an approximation because there is some dependence among the held-out observations, within each of the five repetitions for example.

Test 2: Men, BMI 25 to <30 kg/m^2

Data		No. of held out observations	Percent covered	Error (percentage points)†				Absolute error			
				Median	Q1	Q3	(p*)	Median	Q1	Q3	(p*)
All		3530	98	0.75	-3.14	4.79	0.04	4.03	1.82	7.56	0.04
Super-region	Central and Eastern Europe	292	98	2.26	-2.90	6.11	0.53	5.02	2.61	8.14	0.53
	Central Asia, Middle East and North Africa	322	98	0.42	-4.20	5.65	0.36	4.86	2.42	9.13	0.36
	East and South East Asia	376	98	0.72	-3.40	4.61	0.34	3.81	1.76	7.08	0.34
	High-income Asia Pacific	354	98	0.49	-1.86	4.16	0.12	2.88	1.17	5.80	0.12
	High-income Western countries	1140	99	0.56	-2.55	3.88	0.07	3.23	1.57	6.23	0.07
	Latin America and Caribbean	426	98	0.79	-3.95	5.47	0.48	4.66	2.41	8.35	0.48
	Oceania	133	92	4.66	-3.02	12.46	0.00	8.97	4.10	17.25	0.00
	South Asia	210	95	1.10	-4.57	5.33	0.35	5.04	2.33	8.58	0.35
	Sub-Saharan Africa	277	96	-0.26	-5.95	4.03	0.57	5.15	2.40	8.19	0.57
Urban or rural studies	Rural	455	93	1.69	-3.47	6.88	0.07	5.54	2.51	10.03	0.07
	Urban	822	98	1.37	-3.95	6.30	0.24	5.09	2.45	9.05	0.24
	Both rural and urban	2253	99	0.45	-2.91	4.01	0.19	3.56	1.62	6.48	0.19
Study representativeness	Community	1279	97	1.40	-3.52	6.41	0.08	5.12	2.42	9.35	0.08
	Sub-national	466	99	0.79	-3.78	5.64	0.49	4.48	2.04	8.25	0.49
	National	1785	98	0.41	-2.81	3.79	0.31	3.40	1.54	6.17	0.31
Age band	18-40	1287	96	0.15	-3.69	3.95	0.50	3.82	1.80	7.42	0.50
	40-60	1043	100	1.00	-2.71	5.24	0.05	4.10	1.73	7.36	0.05
	60 and above	1200	98	1.15	-2.79	5.52	0.34	4.15	1.90	7.89	0.34
Years	1975-1984	136	98	0.64	-2.16	4.77	0.21	2.91	1.39	6.40	0.21
	1985-1994	569	97	1.18	-2.67	5.40	0.15	4.20	1.89	7.60	0.15
	1995-2004	1246	98	0.85	-2.92	5.16	0.16	3.86	1.73	7.48	0.16
	2005-2014	1579	98	0.51	-3.82	4.37	0.57	4.13	1.93	7.65	0.57
Data density	Data poor	*Not applicable for male data in source-based test*									
	Average data density	460	96	0.40	-5.75	6.12	0.33	5.83	2.89	9.93	0.33
	Data rich	3070	98	0.81	-2.80	4.69	0.05	3.83	1.72	7.13	0.05
Hold out pattern	Post-2000 data removed	1183	98	0.45	-2.94	4.34	0.29	3.83	1.71	7.22	0.29
	Random set of data removed	2347	98	0.89	-3.23	5.04	0.07	4.16	1.87	7.63	0.07

† Estimated values minus held out values.

***** p-values for model error comparisons were calculated using the non-parametric Wilcoxon signed-rank test for paired data. The p-values are calculated assuming independence of the held-out observations. They should therefore be interpreted as an approximation because there is some dependence among the held-out observations, within each of the five repetitions for example.

Test 2: Men, BMI 30 to <35 kg/m^2

Data		No. of held out observations	Percent covered	Error (percentage points)†				Absolute error			
				Median	Q1	Q3	(p*)	Median	Q1	Q3	(p*)
All		3530	91	0.06	-2.61	1.73	0.96	2.11	0.81	4.72	0.96
Super-region	Central and Eastern Europe	292	93	0.55	-3.09	2.49	0.78	2.55	1.23	5.07	0.78
	Central Asia, Middle East and North Africa	322	95	-0.31	-4.76	3.10	0.41	3.84	1.60	7.28	0.41
	East and South East Asia	376	89	0.29	-0.62	1.18	0.11	1.00	0.38	2.71	0.11
	High-income Asia Pacific	354	76	0.02	-0.95	0.57	0.36	0.74	0.35	1.42	0.36
	High-income Western countries	1140	96	-0.23	-3.34	2.41	0.08	2.80	1.19	5.34	0.08
	Latin America and Caribbean	426	95	0.40	-3.02	2.92	0.81	2.95	1.34	5.21	0.81
	Oceania	133	83	1.38	-6.33	8.31	0.44	6.86	3.68	11.18	0.44
	South Asia	210	85	0.07	-1.42	0.62	0.71	0.88	0.34	1.82	0.71
	Sub-Saharan Africa	277	86	-0.54	-3.15	0.71	0.10	1.54	0.69	3.45	0.10
Urban or rural studies	Rural	455	76	0.14	-3.53	1.27	0.09	1.90	0.72	4.93	0.09
	Urban	822	93	0.57	-2.59	3.23	0.25	2.97	1.20	5.70	0.25
	Both rural and urban	2253	93	-0.09	-2.46	1.40	0.34	1.90	0.71	4.31	0.34
Study representativeness	Community	1279	88	0.13	-3.67	1.91	0.30	2.66	1.00	5.78	0.30
	Sub-national	466	94	0.04	-2.74	2.19	0.22	2.50	1.03	4.82	0.22
	National	1785	92	0.04	-2.00	1.43	0.86	1.68	0.63	4.03	0.86
Age band	18-40	1287	86	-0.17	-2.18	1.16	0.26	1.63	0.65	3.65	0.26
	40-60	1043	97	0.40	-2.85	2.46	0.54	2.63	1.11	5.56	0.54
	60 and above	1200	92	0.09	-2.98	1.92	0.89	2.42	0.73	5.38	0.89
Years	1975-1984	136	88	0.09	-1.27	1.67	0.68	1.52	0.33	4.95	0.68
	1985-1994	569	87	0.08	-1.76	1.48	0.53	1.69	0.57	3.97	0.53
	1995-2004	1246	93	-0.02	-2.42	1.67	0.66	2.05	0.80	4.49	0.66
	2005-2014	1579	91	0.09	-3.04	1.87	0.78	2.36	0.95	5.07	0.78
Data density	Data poor	*Not applicable for male data in source-based test*									
	Average data density	460	87	0.19	-3.34	2.49	0.30	2.90	1.09	6.43	0.30
	Data rich	3070	92	0.04	-2.48	1.63	0.60	2.04	0.77	4.53	0.60
Hold out pattern	Post-2000 data removed	1183	89	-0.28	-3.34	1.32	0.19	2.14	0.76	5.00	0.19
	Random set of data removed	2347	92	0.16	-2.25	1.95	0.34	2.10	0.82	4.62	0.34

† Estimated values minus held out values.

***** p-values for model error comparisons were calculated using the non-parametric Wilcoxon signed-rank test for paired data. The p-values are calculated assuming independence of the held-out observations. They should therefore be interpreted as an approximation because there is some dependence among the held-out observations, within each of the five repetitions for example.

Test 2: Men, BMI 35 to <40 kg/m^2

Data		No. of held out observations	Percent covered	Error (percentage points)[†]				Absolute error			
				Median	Q1	Q3	(p*)	Median	Q1	Q3	(p*)
All		3530	75	-0.02	-0.77	0.32	0.01	0.51	0.14	1.36	0.01
Super-region	Central and Eastern Europe	292	80	-0.03	-0.98	0.53	0.51	0.72	0.32	1.53	0.51
	Central Asia, Middle East and North Africa	322	86	-0.03	-1.22	0.85	0.86	0.98	0.43	1.93	0.86
	East and South East Asia	376	71	0.00	-0.17	0.05	0.97	0.10	0.03	0.29	0.97
	High-income Asia Pacific	354	43	0.00	-0.18	0.01	0.00	0.03	0.01	0.20	0.00
	High-income Western countries	1140	83	-0.15	-1.07	0.61	0.10	0.86	0.39	1.61	0.10
	Latin America and Caribbean	426	84	0.05	-0.92	0.55	0.82	0.66	0.26	1.51	0.82
	Oceania	133	69	0.67	-3.73	3.02	0.09	3.20	1.64	6.60	0.09
	South Asia	210	64	-0.03	-0.39	0.03	0.40	0.11	0.03	0.41	0.40
	Sub-Saharan Africa	277	72	-0.12	-0.78	0.06	0.07	0.23	0.08	0.87	0.07
Urban or rural studies	Rural	455	55	0.00	-0.52	0.18	0.00	0.29	0.05	1.20	0.00
	Urban	822	78	-0.03	-0.87	0.54	0.52	0.68	0.20	1.43	0.52
	Both rural and urban	2253	79	-0.05	-0.75	0.29	0.26	0.50	0.15	1.36	0.26
Study representativeness	Community	1279	71	-0.04	-0.99	0.28	0.18	0.60	0.15	1.41	0.18
	Sub-national	466	82	0.02	-0.64	0.54	0.04	0.58	0.21	1.41	0.04
	National	1785	77	-0.02	-0.65	0.29	0.13	0.45	0.12	1.30	0.13
Age band	18-40	1287	68	-0.01	-0.63	0.24	0.01	0.38	0.12	1.04	0.01
	40-60	1043	87	-0.03	-0.75	0.59	0.58	0.65	0.19	1.63	0.58
	60 and above	1200	74	-0.02	-0.92	0.29	0.04	0.58	0.13	1.46	0.04
Years	1975-1984	136	68	-0.07	-0.69	0.01	0.96	0.44	0.02	1.12	0.96
	1985-1994	569	66	-0.02	-0.58	0.16	0.02	0.34	0.08	0.99	0.02
	1995-2004	1246	80	-0.06	-0.72	0.27	0.75	0.49	0.14	1.26	0.75
	2005-2014	1579	76	0.01	-0.89	0.46	0.03	0.63	0.19	1.68	0.03
Data density	Data poor	*Not applicable for male data in source-based test*									
	Average data density	460	73	-0.01	-0.88	0.37	0.10	0.63	0.17	1.87	0.10
	Data rich	3070	76	-0.02	-0.75	0.31	0.03	0.51	0.14	1.31	0.03
Hold out pattern	Post-2000 data removed	1183	70	-0.02	-0.72	0.28	0.03	0.49	0.11	1.34	0.03
	Random set of data removed	2347	78	-0.02	-0.80	0.34	0.05	0.54	0.15	1.37	0.05

[†] Estimated values minus held out values.

***** p-values for model error comparisons were calculated using the non-parametric Wilcoxon signed-rank test for paired data. The p-values are calculated assuming independence of the held-out observations. They should therefore be interpreted as an approximation because there is some dependence among the held-out observations, within each of the five repetitions for example.

Test 2: Men, BMI ≥ 40 kg/m^2

Data		No. of held out observations	Percent covered	Error (percentage points)[†]				Absolute error			
				Median	Q1	Q3	(p*)	Median	Q1	Q3	(p*)
All		3530	59	0.00	-0.27	0.08	0.00	0.15	0.03	0.50	0.00
Super-region	Central and Eastern Europe	292	52	0.03	-0.34	0.12	0.01	0.16	0.06	0.47	0.01
	Central Asia, Middle East and North Africa	322	74	0.02	-0.34	0.22	0.25	0.29	0.12	0.64	0.25
	East and South East Asia	376	58	0.00	-0.06	0.01	0.09	0.03	0.01	0.10	0.09
	High-income Asia Pacific	354	19	0.00	0.00	0.00	0.00	0.00	0.00	0.02	0.00
	High-income Western countries	1140	66	-0.02	-0.41	0.12	0.68	0.24	0.08	0.63	0.68
	Latin America and Caribbean	426	70	0.02	-0.34	0.21	0.09	0.25	0.09	0.56	0.09
	Oceania	133	61	0.29	-2.46	1.08	0.06	1.43	0.61	3.08	0.06
	South Asia	210	47	0.00	-0.13	0.02	0.00	0.04	0.01	0.13	0.00
	Sub-Saharan Africa	277	58	0.00	-0.32	0.05	0.07	0.15	0.04	0.43	0.07
Urban or rural studies	Rural	455	37	0.01	-0.03	0.07	0.00	0.05	0.01	0.24	0.00
	Urban	822	63	-0.03	-0.34	0.08	0.16	0.18	0.06	0.50	0.16
	Both rural and urban	2253	62	0.00	-0.29	0.09	0.00	0.17	0.03	0.53	0.00
Study representativeness	Community	1279	54	0.00	-0.33	0.06	0.00	0.14	0.03	0.47	0.00
	Sub-national	466	63	0.01	-0.20	0.17	0.00	0.18	0.05	0.53	0.00
	National	1785	61	0.00	-0.25	0.09	0.00	0.15	0.03	0.51	0.00
Age band	18-40	1287	51	0.00	-0.21	0.07	0.00	0.12	0.02	0.40	0.00
	40-60	1043	72	-0.01	-0.35	0.15	0.09	0.25	0.06	0.65	0.09
	60 and above	1200	55	0.00	-0.26	0.08	0.00	0.14	0.02	0.45	0.00
Years	1975-1984	136	50	0.00	-0.33	0.00	0.15	0.10	0.00	0.47	0.15
	1985-1994	569	48	0.00	-0.14	0.04	0.00	0.07	0.01	0.25	0.00
	1995-2004	1246	64	-0.01	-0.25	0.05	0.00	0.13	0.03	0.39	0.00
	2005-2014	1579	59	0.01	-0.34	0.18	0.00	0.24	0.05	0.70	0.00
Data density	Data poor	*Not applicable for male data in source-based test*									
	Average data density	460	57	0.01	-0.35	0.17	0.00	0.23	0.07	0.74	0.00
	Data rich	3070	59	0.00	-0.26	0.07	0.00	0.14	0.03	0.47	0.00
Hold out pattern	Post-2000 data removed	1183	53	0.00	-0.30	0.05	0.00	0.14	0.01	0.46	0.00
	Random set of data removed	2347	62	0.00	-0.26	0.09	0.00	0.16	0.04	0.52	0.00

[†] Estimated values minus held out values.

* p-values for model error comparisons were calculated using the non-parametric Wilcoxon signed-rank test for paired data. The p-values are calculated assuming independence of the held-out observations. They should therefore be interpreted as an approximation because there is some dependence among the held-out observations, within each of the five repetitions for example.

Test 2: Women, mean BMI

Data		No. of held out observations	Percent covered	Error (kg/m²)†				Absolute error			
				Median	Q1	Q3	(p*)	Median	Q1	Q3	(p*)
All		4047	94	-0.01	-0.36	0.33	0.95	0.35	0.16	0.67	0.95
Super-region	Central and Eastern Europe	207	97	0.01	-0.44	0.41	0.83	0.43	0.15	0.70	0.83
	Central Asia, Middle East and North Africa	522	89	-0.01	-0.51	0.55	0.92	0.54	0.25	0.95	0.92
	East and South East Asia	499	94	0.00	-0.27	0.33	0.90	0.31	0.14	0.57	0.90
	High-income Asia Pacific	239	94	0.03	-0.40	0.30	0.60	0.32	0.16	0.58	0.60
	High-income Western countries	1162	96	-0.01	-0.34	0.28	0.54	0.31	0.15	0.57	0.54
	Latin America and Caribbean	523	96	-0.05	-0.40	0.30	0.82	0.35	0.14	0.78	0.82
	Oceania	117	95	-0.04	-0.55	0.50	0.99	0.53	0.26	0.80	0.99
	South Asia	225	93	-0.02	-0.23	0.26	0.97	0.25	0.12	0.48	0.97
	Sub-Saharan Africa	553	94	-0.01	-0.33	0.35	0.39	0.33	0.13	0.67	0.39
Urban or rural studies	Rural	381	91	0.04	-0.40	0.46	0.97	0.43	0.18	0.92	0.97
	Urban	981	95	-0.01	-0.38	0.37	0.66	0.38	0.17	0.74	0.66
	Both rural and urban	2685	94	-0.02	-0.35	0.31	0.93	0.33	0.15	0.62	0.93
Study representativeness	Community	1292	93	0.00	-0.43	0.38	0.68	0.40	0.18	0.83	0.68
	Sub-national	517	94	-0.01	-0.32	0.36	0.83	0.33	0.15	0.60	0.83
	National	2238	95	-0.02	-0.34	0.31	0.82	0.32	0.15	0.61	0.82
Age band	18-40	1695	94	0.00	-0.33	0.35	0.76	0.33	0.15	0.67	0.76
	40-60	1211	97	-0.02	-0.33	0.28	0.71	0.31	0.14	0.54	0.71
	60 and above	1141	92	-0.03	-0.45	0.39	0.87	0.42	0.19	0.86	0.87
Years	1975-1984	166	94	0.01	-0.35	0.36	0.97	0.36	0.14	0.64	0.97
	1985-1994	634	97	-0.01	-0.33	0.33	0.90	0.33	0.15	0.61	0.90
	1995-2004	1515	94	-0.02	-0.34	0.32	0.92	0.33	0.15	0.64	0.92
	2005-2014	1732	93	-0.01	-0.40	0.36	0.94	0.37	0.17	0.73	0.94
Data density	Data poor	116	97	0.02	-0.59	0.46	0.94	0.52	0.21	1.05	0.94
	Average data density	723	95	-0.01	-0.35	0.39	0.75	0.36	0.17	0.72	0.75
	Data rich	3208	94	-0.01	-0.36	0.32	0.83	0.34	0.15	0.65	0.83
Hold out pattern	Post-2000 data removed	1406	95	0.00	-0.34	0.31	0.93	0.32	0.15	0.60	0.93
	Random set of data removed	2641	94	-0.02	-0.37	0.36	0.92	0.36	0.16	0.72	0.92

† Estimated values minus held out values.

***** p-values for model error comparisons were calculated using the non-parametric Wilcoxon signed-rank test for paired data. The p-values are calculated assuming independence of the held-out observations. They should therefore be interpreted as an approximation because there is some dependence among the held-out observations, within each of the five repetitions for example.

Test 2: Women, BMI < 18.5 kg/m^2

Data		No. of held out observations	Percent covered	Error (percentage points)†				Absolute error			
				Median	Q1	Q3	(p*)	Median	Q1	Q3	(p*)
All		4047	90	-0.15	-2.03	1.14	0.71	1.57	0.57	4.06	0.71
Super-region	Central and Eastern Europe	207	88	-0.25	-1.71	0.38	0.98	0.85	0.31	2.32	0.98
	Central Asia, Middle East and North Africa	522	88	0.25	-1.29	1.16	0.00	1.21	0.54	2.83	0.00
	East and South East Asia	499	98	-0.15	-4.43	3.76	0.27	4.06	1.92	6.69	0.27
	High-income Asia Pacific	239	100	-0.48	-5.77	1.95	0.01	2.87	1.36	6.66	0.01
	High-income Western countries	1162	88	-0.32	-1.35	0.35	0.00	0.82	0.34	1.89	0.00
	Latin America and Caribbean	523	86	0.10	-1.09	0.89	0.44	0.98	0.46	2.02	0.44
	Oceania	117	52	0.09	-1.07	0.49	0.07	0.86	0.19	2.31	0.07
	South Asia	225	96	-4.31	-16.58	5.02	0.00	9.39	4.86	17.62	0.00
	Sub-Saharan Africa	553	98	0.70	-2.46	3.69	0.00	3.25	1.55	6.16	0.00
Urban or rural studies	Rural	381	80	-1.30	-8.16	0.85	0.38	2.72	1.01	8.48	0.38
	Urban	981	84	-0.53	-2.04	0.52	0.00	1.23	0.52	2.97	0.00
	Both rural and urban	2685	94	0.06	-1.64	1.51	0.02	1.58	0.56	4.13	0.02
Study representativeness	Community	1292	83	-0.49	-2.43	0.85	0.53	1.66	0.63	4.09	0.53
	Sub-national	517	92	-0.28	-1.71	0.63	0.14	1.17	0.42	3.78	0.14
	National	2238	94	0.04	-1.77	1.43	0.10	1.60	0.57	4.15	0.10
Age band	18-40	1695	94	-0.17	-3.34	2.05	0.94	2.59	1.03	5.64	0.94
	40-60	1211	89	-0.22	-1.37	0.53	0.02	0.91	0.34	2.59	0.02
	60 and above	1141	87	-0.01	-1.41	0.99	0.52	1.20	0.49	3.30	0.52
Years	1975-1984	166	86	-1.01	-3.18	0.09	0.00	1.51	0.55	3.79	0.00
	1985-1994	634	87	-0.14	-1.87	1.07	0.54	1.45	0.54	3.71	0.54
	1995-2004	1515	93	-0.14	-1.95	1.10	0.52	1.44	0.53	4.12	0.52
	2005-2014	1732	90	-0.06	-2.02	1.38	0.31	1.70	0.62	4.23	0.31
Data density	Data poor	116	94	-0.14	-2.41	1.50	0.73	2.06	0.85	4.52	0.73
	Average data density	723	89	0.10	-1.67	1.71	0.36	1.70	0.63	4.30	0.36
	Data rich	3208	91	-0.21	-2.09	1.03	0.93	1.50	0.55	3.96	0.93
Hold out pattern	Post-2000 data removed	1406	94	-0.27	-2.29	1.17	0.26	1.74	0.66	4.06	0.26
	Random set of data removed	2641	89	-0.09	-1.86	1.13	0.22	1.48	0.53	4.06	0.22

† Estimated values minus held out values.

***** p-values for model error comparisons were calculated using the non-parametric Wilcoxon signed-rank test for paired data. The p-values are calculated assuming independence of the held-out observations. They should therefore be interpreted as an approximation because there is some dependence among the held-out observations, within each of the five repetitions for example.

Test 2: Women, BMI 18.5 to <20 kg/m^2

Data		No. of held out observations	Percent covered	Error (percentage points)[†]				Absolute error			
				Median	Q1	Q3	(p*)	Median	Q1	Q3	(p*)
All		4047	94	-0.16	-2.54	1.67	0.78	2.09	0.87	4.39	0.78
Super-region	Central and Eastern Europe	207	99	-0.05	-2.14	0.94	0.22	1.45	0.56	3.50	0.22
	Central Asia, Middle East and North Africa	522	91	0.09	-1.38	1.53	0.13	1.47	0.69	3.20	0.13
	East and South East Asia	499	99	-0.15	-4.21	3.22	0.73	3.79	1.82	6.44	0.73
	High-income Asia Pacific	239	100	-0.66	-5.70	1.69	0.20	3.13	1.34	6.29	0.20
	High-income Western countries	1162	93	-0.38	-2.28	1.03	0.18	1.56	0.69	3.14	0.18
	Latin America and Caribbean	523	92	-0.13	-1.64	1.36	0.77	1.48	0.61	2.65	0.77
	Oceania	117	62	0.18	-1.23	1.13	0.20	1.18	0.38	3.80	0.20
	South Asia	225	98	-1.55	-5.04	3.23	0.16	4.28	2.29	6.54	0.16
	Sub-Saharan Africa	553	99	0.45	-3.19	3.39	0.59	3.32	1.60	5.73	0.59
Urban or rural studies	Rural	381	87	-1.55	-5.43	1.32	0.04	3.15	1.41	6.57	0.04
	Urban	981	91	-0.56	-2.67	1.12	0.05	1.91	0.82	3.82	0.05
	Both rural and urban	2685	96	0.09	-2.14	1.96	0.23	2.06	0.84	4.32	0.23
Study representativeness	Community	1292	90	-0.33	-2.87	1.59	0.67	2.24	0.97	4.72	0.67
	Sub-national	517	94	-0.35	-2.46	1.26	0.37	1.95	0.70	4.19	0.37
	National	2238	96	0.04	-2.29	1.77	0.80	2.06	0.87	4.32	0.80
Age band	18-40	1695	97	-0.04	-3.61	2.71	0.66	3.05	1.48	5.88	0.66
	40-60	1211	94	-0.31	-2.13	1.06	0.09	1.51	0.64	3.40	0.09
	60 and above	1141	91	-0.09	-1.98	1.24	0.99	1.48	0.66	3.23	0.99
Years	1975-1984	166	91	-0.81	-2.78	0.98	0.11	2.23	0.97	3.81	0.11
	1985-1994	634	91	-0.01	-2.43	1.98	0.84	2.16	0.90	4.48	0.84
	1995-2004	1515	95	-0.03	-2.29	1.61	0.43	1.93	0.81	4.24	0.43
	2005-2014	1732	95	-0.28	-2.81	1.63	0.47	2.21	0.90	4.63	0.47
Data density	Data poor	116	93	-0.40	-3.11	1.12	0.77	2.14	0.89	4.24	0.77
	Average data density	723	94	-0.02	-2.30	1.97	0.66	2.13	0.87	4.65	0.66
	Data rich	3208	94	-0.19	-2.60	1.60	0.99	2.08	0.87	4.37	0.99
Hold out pattern	Post-2000 data removed	1406	96	-0.31	-2.82	1.68	0.23	2.24	0.99	4.41	0.23
	Random set of data removed	2641	93	-0.08	-2.37	1.67	0.47	2.05	0.81	4.37	0.47

[†] Estimated values minus held out values.

* p-values for model error comparisons were calculated using the non-parametric Wilcoxon signed-rank test for paired data. The p-values are calculated assuming independence of the held-out observations. They should therefore be interpreted as an approximation because there is some dependence among the held-out observations, within each of the five repetitions for example.

Test 2: Women, BMI 20 to <25 kg/m^2

Data		No. of held out observations	Percent covered	Error (percentage points)†				Absolute error			
				Median	Q1	Q3	(p*)	Median	Q1	Q3	(p*)
All		4047	99	1.53	-3.63	7.12	0.00	5.40	2.51	10.10	0.00
Super-region	Central and Eastern Europe	207	100	3.45	-2.84	7.98	0.01	6.35	3.21	9.03	0.01
	Central Asia, Middle East and North Africa	522	100	2.81	-2.42	8.15	0.00	5.46	2.71	10.02	0.00
	East and South East Asia	499	99	1.43	-3.26	6.59	0.00	4.95	2.24	9.28	0.00
	High-income Asia Pacific	239	100	0.10	-3.05	8.69	0.00	4.54	2.24	9.61	0.00
	High-income Western countries	1162	99	0.98	-4.42	6.44	0.01	5.44	2.45	10.36	0.01
	Latin America and Caribbean	523	99	0.77	-3.37	5.65	0.22	4.46	1.83	8.54	0.22
	Oceania	117	93	2.91	-2.73	10.41	0.08	7.95	2.91	16.96	0.08
	South Asia	225	97	5.81	-0.81	14.31	0.00	8.09	4.09	17.13	0.00
	Sub-Saharan Africa	553	100	-0.30	-5.25	5.49	0.73	5.36	2.63	9.35	0.73
Urban or rural studies	Rural	381	96	4.86	-2.37	12.67	0.00	8.26	3.85	15.62	0.00
	Urban	981	99	0.36	-5.32	5.78	0.55	5.61	2.62	10.54	0.55
	Both rural and urban	2685	100	1.60	-3.30	6.98	0.00	5.09	2.35	9.32	0.00
Study representativeness	Community	1292	98	2.25	-3.90	8.84	0.00	6.35	2.89	12.27	0.00
	Sub-national	517	99	0.00	-5.67	5.70	0.83	5.70	2.69	10.80	0.83
	National	2238	100	1.52	-3.18	6.62	0.00	4.91	2.26	8.80	0.00
Age band	18-40	1695	99	1.92	-3.47	8.30	0.00	5.80	2.63	10.63	0.00
	40-60	1211	100	1.39	-3.52	5.95	0.06	4.81	2.29	9.04	0.06
	60 and above	1141	98	1.25	-3.90	6.96	0.00	5.38	2.57	10.15	0.00
Years	1975-1984	166	99	2.29	-5.47	8.12	0.28	6.82	3.63	10.92	0.28
	1985-1994	634	99	0.68	-4.76	6.65	0.35	5.78	2.90	10.79	0.35
	1995-2004	1515	99	2.05	-2.60	7.48	0.00	5.19	2.30	9.82	0.00
	2005-2014	1732	99	1.17	-3.97	6.79	0.00	5.30	2.53	9.93	0.00
Data density	Data poor	116	100	0.94	-5.28	5.21	0.92	5.29	2.31	9.65	0.92
	Average data density	723	99	1.15	-4.79	6.61	0.34	5.52	2.65	9.66	0.34
	Data rich	3208	99	1.63	-3.42	7.36	0.00	5.38	2.49	10.16	0.00
Hold out pattern	Post-2000 data removed	1406	99	1.07	-3.56	6.46	0.00	5.01	2.31	9.65	0.00
	Random set of data removed	2641	99	1.74	-3.68	7.32	0.00	5.62	2.61	10.30	0.00

† Estimated values minus held out values.

* p-values for model error comparisons were calculated using the non-parametric Wilcoxon signed-rank test for paired data. The p-values are calculated assuming independence of the held-out observations. They should therefore be interpreted as an approximation because there is some dependence among the held-out observations, within each of the five repetitions for example.

Test 2: Women, BMI 25 to <30 kg/m^2

Data		No. of held out observations	Percent covered	Error (percentage points)†				Absolute error			
				Median	Q1	Q3	(p*)	Median	Q1	Q3	(p*)
All		4047	98	0.88	-3.36	5.41	0.00	4.37	2.02	7.99	0.00
Super-region	Central and Eastern Europe	207	98	2.08	-1.55	4.98	0.51	3.52	1.90	6.14	0.51
	Central Asia, Middle East and North Africa	522	99	1.40	-3.30	5.62	0.03	4.62	2.11	8.59	0.03
	East and South East Asia	499	98	0.17	-5.11	5.44	0.52	5.30	2.50	9.37	0.52
	High-income Asia Pacific	239	97	2.47	-0.75	5.61	0.01	4.17	1.92	6.39	0.01
	High-income Western countries	1162	98	0.15	-3.31	4.94	0.85	3.96	1.90	7.16	0.85
	Latin America and Caribbean	523	99	0.22	-3.60	3.77	0.43	3.64	1.58	6.66	0.43
	Oceania	117	96	5.43	-1.44	13.51	0.00	9.80	4.16	16.06	0.00
	South Asia	225	93	2.87	-3.26	8.47	0.00	6.58	3.12	11.31	0.00
	Sub-Saharan Africa	553	98	0.79	-3.50	5.22	0.04	4.35	1.84	8.14	0.04
Urban or rural studies	Rural	381	90	3.23	-3.28	9.22	0.00	6.34	3.28	11.07	0.00
	Urban	981	98	1.78	-2.82	6.97	0.00	4.71	2.20	8.48	0.00
	Both rural and urban	2685	99	0.43	-3.54	4.57	0.36	4.09	1.84	7.39	0.36
Study representativeness	Community	1292	96	1.57	-3.67	7.16	0.00	5.31	2.41	9.37	0.00
	Sub-national	517	98	0.85	-3.57	5.76	0.46	4.37	2.30	8.35	0.46
	National	2238	99	0.64	-3.08	4.62	0.06	3.95	1.75	7.14	0.06
Age band	18-40	1695	97	0.64	-3.95	5.02	0.01	4.53	2.02	7.92	0.01
	40-60	1211	100	1.34	-2.97	5.48	0.01	4.36	2.08	7.88	0.01
	60 and above	1141	97	0.79	-3.03	5.91	0.05	4.17	1.94	8.30	0.05
Years	1975-1984	166	98	2.18	-1.90	5.17	0.10	4.17	2.17	8.29	0.10
	1985-1994	634	98	1.01	-3.61	6.44	0.07	4.88	2.35	9.10	0.07
	1995-2004	1515	98	0.74	-3.24	5.03	0.26	4.27	1.94	7.60	0.26
	2005-2014	1732	98	0.83	-3.39	5.41	0.01	4.34	1.97	7.95	0.01
Data density	Data poor	116	97	2.71	-0.85	7.23	0.05	4.41	1.94	8.39	0.05
	Average data density	723	99	0.92	-3.52	5.38	0.05	4.54	2.12	7.96	0.05
	Data rich	3208	98	0.82	-3.39	5.35	0.01	4.33	1.99	7.98	0.01
Hold out pattern	Post-2000 data removed	1406	98	0.77	-3.63	4.76	0.09	4.33	1.95	7.42	0.09
	Random set of data removed	2641	98	0.98	-3.22	5.82	0.00	4.42	2.06	8.34	0.00

† Estimated values minus held out values.

***** p-values for model error comparisons were calculated using the non-parametric Wilcoxon signed-rank test for paired data. The p-values are calculated assuming independence of the held-out observations. They should therefore be interpreted as an approximation because there is some dependence among the held-out observations, within each of the five repetitions for example.

Test 2: Women, BMI 30 to <35 kg/m^2

Data		No. of held out observations	Percent covered	Error (percentage points)[†]				Absolute error			
				Median	Q1	Q3	(p*)	Median	Q1	Q3	(p*)
All		4047	93	0.18	-2.44	2.48	0.03	2.46	0.96	4.95	0.03
Super-region	Central and Eastern Europe	207	92	0.18	-4.44	3.20	0.45	3.67	2.08	5.75	0.45
	Central Asia, Middle East and North Africa	522	96	-0.45	-4.79	2.05	0.05	3.66	1.50	6.60	0.05
	East and South East Asia	499	90	0.21	-1.51	2.02	0.09	1.82	0.66	3.40	0.09
	High-income Asia Pacific	239	88	0.17	-0.74	0.76	0.92	0.75	0.32	1.63	0.92
	High-income Western countries	1162	96	0.65	-2.26	3.34	0.05	2.84	1.25	5.47	0.05
	Latin America and Caribbean	523	96	0.08	-2.89	2.99	0.51	2.96	1.45	5.55	0.51
	Oceania	117	91	-0.44	-4.21	3.90	0.90	3.96	1.63	9.09	0.90
	South Asia	225	85	0.29	-1.47	2.07	0.02	1.89	0.75	3.71	0.02
	Sub-Saharan Africa	553	92	0.01	-1.91	1.62	0.40	1.71	0.54	3.87	0.40
Urban or rural studies	Rural	381	82	0.68	-1.97	3.03	0.00	2.51	1.26	5.81	0.00
	Urban	981	95	0.89	-2.84	3.74	0.01	3.38	1.64	6.10	0.01
	Both rural and urban	2685	94	0.03	-2.38	1.87	0.79	2.10	0.77	4.30	0.79
Study representativeness	Community	1292	91	0.33	-3.21	3.09	0.11	3.13	1.38	6.03	0.11
	Sub-national	517	96	0.90	-1.84	3.86	0.01	3.21	1.35	5.98	0.01
	National	2238	94	0.08	-2.14	1.84	0.48	1.98	0.73	3.98	0.48
Age band	18-40	1695	88	0.10	-1.69	1.64	0.08	1.65	0.62	3.49	0.08
	40-60	1211	99	0.42	-2.94	2.80	0.36	2.87	1.32	5.47	0.36
	60 and above	1141	95	0.27	-3.26	3.54	0.35	3.46	1.47	6.27	0.35
Years	1975-1984	166	92	0.02	-3.27	3.24	0.42	3.30	0.95	6.09	0.42
	1985-1994	634	92	0.17	-2.23	2.56	0.24	2.39	0.91	5.36	0.24
	1995-2004	1515	94	0.07	-2.85	2.19	0.65	2.49	0.98	4.69	0.65
	2005-2014	1732	94	0.34	-2.10	2.56	0.03	2.43	0.96	4.89	0.03
Data density	Data poor	116	85	0.33	-3.11	3.18	0.43	3.19	1.56	5.04	0.43
	Average data density	723	93	0.14	-2.83	2.56	0.10	2.71	0.97	5.33	0.10
	Data rich	3208	94	0.19	-2.38	2.42	0.13	2.39	0.94	4.87	0.13
Hold out pattern	Post-2000 data removed	1406	94	0.22	-1.80	2.32	0.16	2.08	0.73	4.46	0.16
	Random set of data removed	2641	93	0.16	-2.86	2.54	0.17	2.65	1.09	5.20	0.17

[†] Estimated values minus held out values.

***** p-values for model error comparisons were calculated using the non-parametric Wilcoxon signed-rank test for paired data. The p-values are calculated assuming independence of the held-out observations. They should therefore be interpreted as an approximation because there is some dependence among the held-out observations, within each of the five repetitions for example.

Test 2: Women, BMI 35 to <40 kg/m^2

Data		No. of held out observations	Percent covered	Error (percentage points)[†]				Absolute error			
				Median	Q1	Q3	(p*)	Median	Q1	Q3	(p*)
All		4047	84	0.01	-1.19	0.71	0.05	0.90	0.26	2.32	0.05
Super-region	Central and Eastern Europe	207	86	0.08	-2.35	0.98	0.33	1.43	0.68	3.10	0.33
	Central Asia, Middle East and North Africa	522	91	-0.09	-2.95	0.92	0.12	1.79	0.61	3.96	0.12
	East and South East Asia	499	75	0.00	-0.34	0.20	0.24	0.26	0.08	0.65	0.24
	High-income Asia Pacific	239	70	0.00	-0.23	0.08	0.34	0.13	0.04	0.32	0.34
	High-income Western countries	1162	89	0.10	-1.45	1.42	0.78	1.45	0.62	2.73	0.78
	Latin America and Caribbean	523	93	0.08	-1.27	1.34	0.34	1.29	0.48	2.62	0.34
	Oceania	117	86	-1.92	-7.38	2.71	0.25	5.53	2.00	9.45	0.25
	South Asia	225	74	0.01	-0.31	0.30	0.02	0.30	0.09	0.74	0.02
	Sub-Saharan Africa	553	78	0.00	-0.89	0.29	0.41	0.58	0.14	1.63	0.41
Urban or rural studies	Rural	381	68	0.12	-0.59	0.74	0.00	0.68	0.21	2.22	0.00
	Urban	981	87	0.15	-1.41	1.46	0.04	1.46	0.52	2.98	0.04
	Both rural and urban	2685	85	-0.02	-1.18	0.47	0.73	0.77	0.23	2.04	0.73
Study representativeness	Community	1292	81	0.02	-1.39	0.92	0.03	1.16	0.34	2.71	0.03
	Sub-national	517	90	0.11	-0.88	1.25	0.05	1.10	0.41	2.61	0.05
	National	2238	85	0.00	-1.14	0.47	0.86	0.75	0.23	2.03	0.86
Age band	18-40	1695	75	0.00	-0.73	0.39	0.03	0.53	0.14	1.41	0.03
	40-60	1211	95	0.02	-1.72	1.11	0.83	1.36	0.46	3.07	0.83
	60 and above	1141	87	0.00	-1.60	1.00	0.36	1.27	0.38	3.04	0.36
Years	1975-1984	166	80	0.00	-1.70	1.11	0.27	1.34	0.46	2.81	0.27
	1985-1994	634	81	-0.02	-1.23	0.61	0.29	0.87	0.28	2.33	0.29
	1995-2004	1515	85	-0.02	-1.26	0.45	0.95	0.79	0.22	2.05	0.95
	2005-2014	1732	85	0.05	-1.10	0.91	0.03	0.98	0.30	2.50	0.03
Data density	Data poor	116	78	0.09	-1.29	0.92	0.44	1.23	0.44	3.06	0.44
	Average data density	723	84	0.01	-1.39	0.73	0.14	0.99	0.28	2.53	0.14
	Data rich	3208	85	0.00	-1.16	0.70	0.17	0.87	0.26	2.23	0.17
Hold out pattern	Post-2000 data removed	1406	84	0.00	-0.78	0.48	0.77	0.63	0.16	1.91	0.77
	Random set of data removed	2641	84	0.01	-1.46	0.79	0.12	1.06	0.36	2.55	0.12

[†] Estimated values minus held out values.

***** p-values for model error comparisons were calculated using the non-parametric Wilcoxon signed-rank test for paired data. The p-values are calculated assuming independence of the held-out observations. They should therefore be interpreted as an approximation because there is some dependence among the held-out observations, within each of the five repetitions for example.

Test 2: Women, BMI ≥ 40 kg/m^2

Data		No. of held out observations	Percent covered	Error (percentage points)[†]				Absolute error			
				Median	Q1	Q3	(p*)	Median	Q1	Q3	(p*)
All		4047	72	0.00	-0.56	0.26	0.00	0.38	0.09	1.12	0.00
Super-region	Central and Eastern Europe	207	77	-0.06	-1.11	0.24	0.27	0.51	0.15	1.43	0.27
	Central Asia, Middle East and North Africa	522	84	0.04	-1.09	0.56	0.92	0.74	0.22	1.82	0.92
	East and South East Asia	499	59	0.00	-0.14	0.03	0.08	0.08	0.02	0.19	0.08
	High-income Asia Pacific	239	37	0.00	-0.11	0.01	0.00	0.01	0.00	0.12	0.00
	High-income Western countries	1162	77	0.04	-0.86	0.47	0.46	0.60	0.25	1.27	0.46
	Latin America and Caribbean	523	83	0.01	-0.84	0.45	0.65	0.60	0.23	1.36	0.65
	Oceania	117	77	-0.96	-8.56	0.89	0.73	3.24	0.96	8.72	0.73
	South Asia	225	57	0.00	-0.10	0.05	0.00	0.06	0.02	0.20	0.00
	Sub-Saharan Africa	553	72	0.01	-0.53	0.22	0.05	0.35	0.08	1.01	0.05
Urban or rural studies	Rural	381	50	0.03	-0.26	0.27	0.00	0.27	0.05	1.16	0.00
	Urban	981	76	0.00	-0.61	0.49	0.01	0.53	0.16	1.27	0.01
	Both rural and urban	2685	74	0.00	-0.55	0.17	0.02	0.33	0.08	1.05	0.02
Study representativeness	Community	1292	68	0.00	-0.67	0.31	0.00	0.43	0.11	1.20	0.00
	Sub-national	517	80	0.04	-0.35	0.51	0.00	0.44	0.13	1.11	0.00
	National	2238	73	0.00	-0.55	0.17	0.01	0.33	0.08	1.05	0.01
Age band	18-40	1695	62	0.00	-0.33	0.13	0.00	0.20	0.05	0.61	0.00
	40-60	1211	85	-0.02	-0.83	0.51	0.44	0.61	0.17	1.51	0.44
	60 and above	1141	74	0.00	-0.79	0.37	0.02	0.51	0.14	1.39	0.02
Years	1975-1984	166	61	0.00	-0.66	0.27	0.23	0.50	0.15	1.05	0.23
	1985-1994	634	69	0.00	-0.54	0.22	0.01	0.35	0.08	0.91	0.01
	1995-2004	1515	74	0.00	-0.50	0.15	0.06	0.31	0.07	0.95	0.06
	2005-2014	1732	73	0.01	-0.59	0.36	0.00	0.45	0.11	1.36	0.00
Data density	Data poor	116	66	0.03	-1.13	0.41	0.49	0.61	0.17	1.60	0.49
	Average data density	723	75	0.01	-0.55	0.25	0.03	0.40	0.10	1.25	0.03
	Data rich	3208	72	0.00	-0.55	0.25	0.00	0.37	0.09	1.08	0.00
Hold out pattern	Post-2000 data removed	1406	69	0.00	-0.27	0.16	0.00	0.23	0.04	0.88	0.00
	Random set of data removed	2641	74	0.01	-0.74	0.29	0.00	0.46	0.12	1.24	0.00

[†] Estimated values minus held out values.

* p-values for model error comparisons were calculated using the non-parametric Wilcoxon signed-rank test for paired data. The p-values are calculated assuming independence of the held-out observations. They should therefore be interpreted as an approximation because there is some dependence among the held-out observations, within each of the five repetitions for example.

Appendix Figure 1: Flowchart of secondary search for data sources.

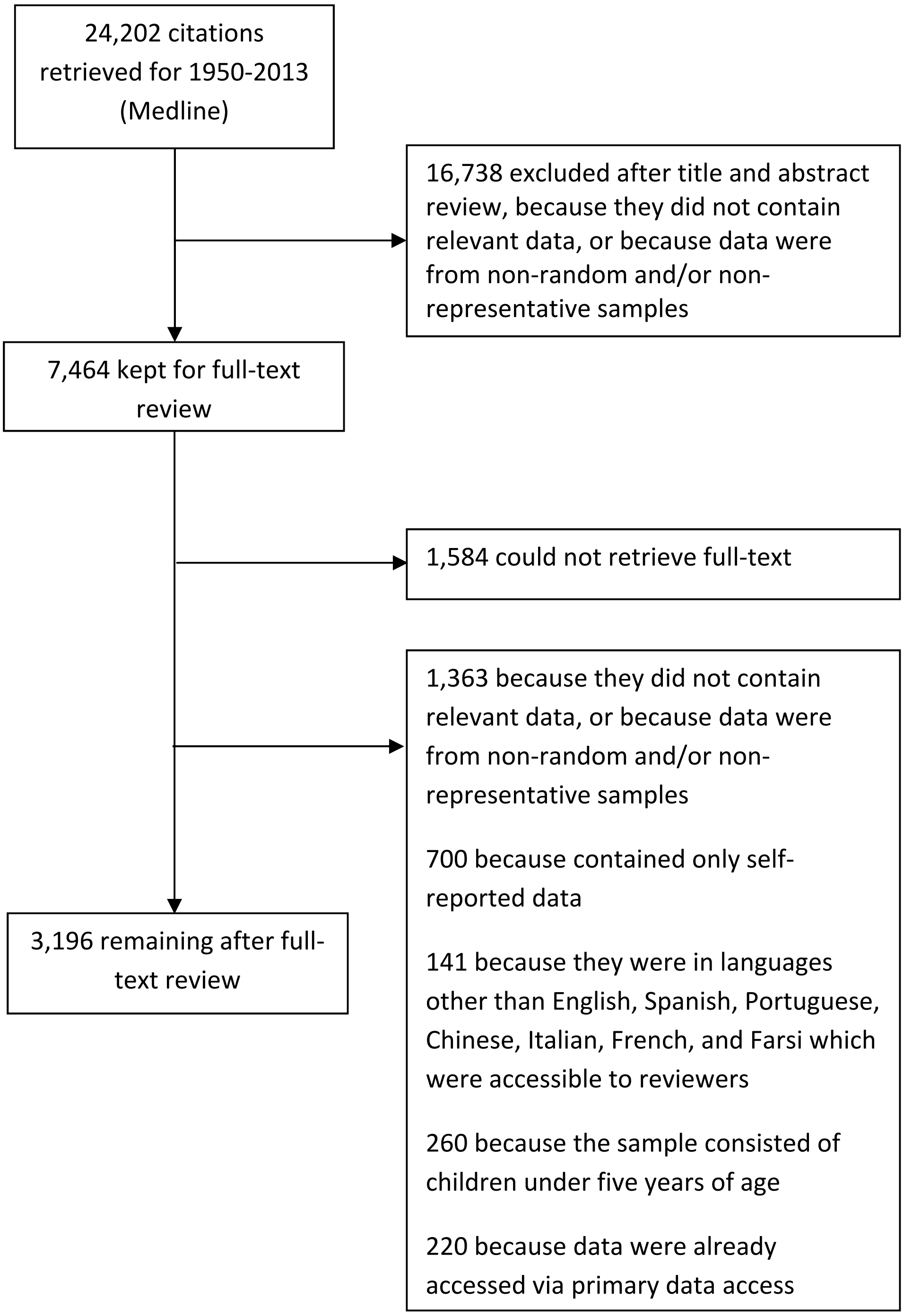
24,202 citations retrieved for 1950-2013 (Medline)
16,738 excluded after title and abstract review, because they did not contain relevant data, or because data were from non-random and/or non-representative samples
7,464 kept for full-text review
1,584 could not retrieve full-text
1,363 because they did not contain relevant data, or because data were from non-random and/or non-representative samples
700 because contained only self-reported data
141 because they were in languages other than English, Spanish, Portuguese, Chinese, Italian, French, and Farsi which were accessible to reviewers
260 because the sample consisted of children under five years of age
220 because data were already accessed via primary data access
3,196 remaining after full-text review

Appendix Figure 2: Number of data sources used in the analysis, by country.

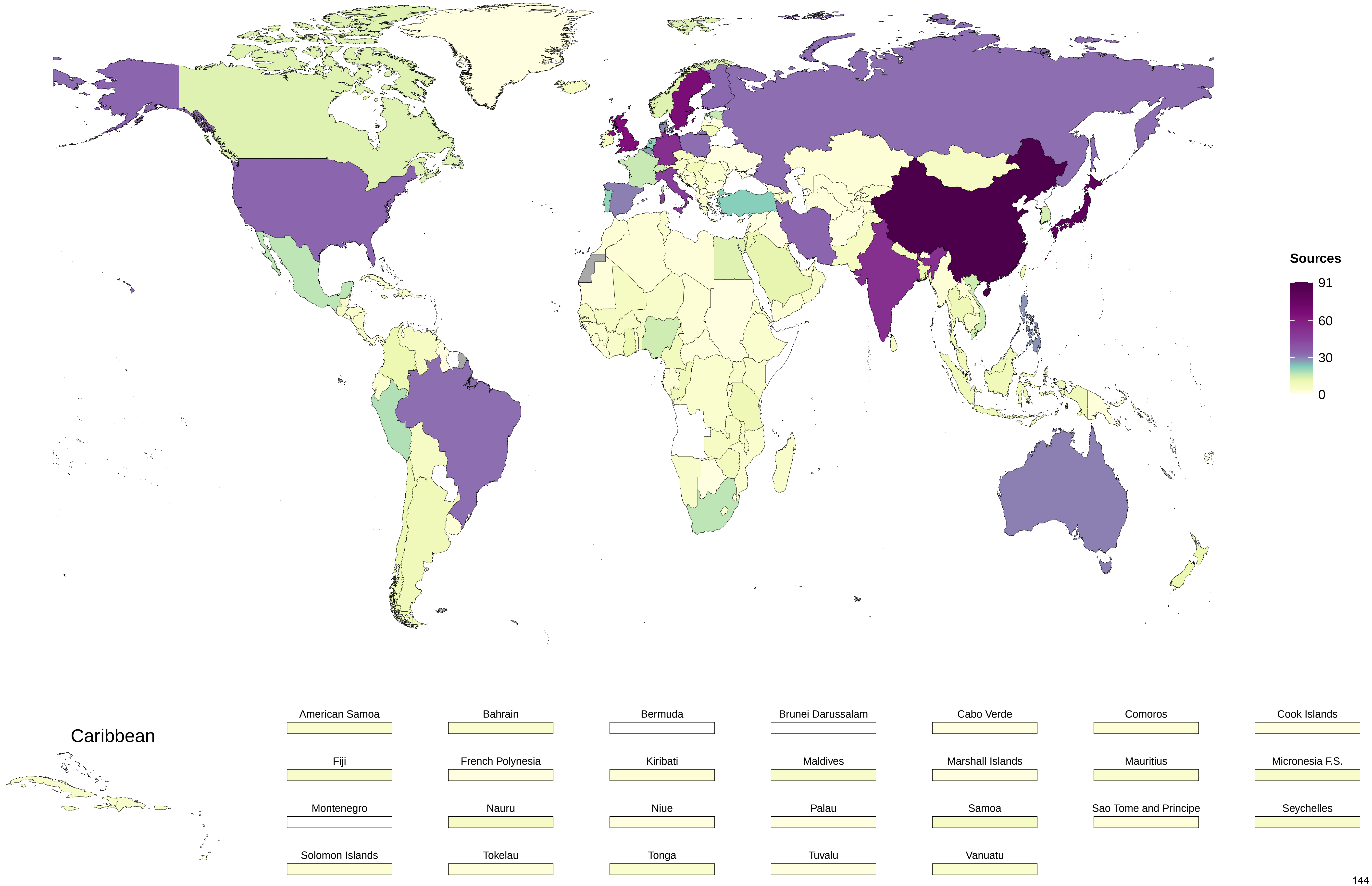
Sources
91
60
30
0
Caribbean
American Samoa
Bahrain
Bermuda
Brunei Darussalam
Cabo Verde
Comoros
Cook Islands
Fiji
French Polynesia
Kiribati
Maldives
Marshall Islands
Mauritius
Micronesia F.S.
Montenegro
Nauru
Niue
Palau
Samoa
Sao Tome and Principe
Seychelles
Solomon Islands
Tokelau
Tonga
Tuvalu
Vanuatu

Appendix Figure 3: Number of data sources by region and year.

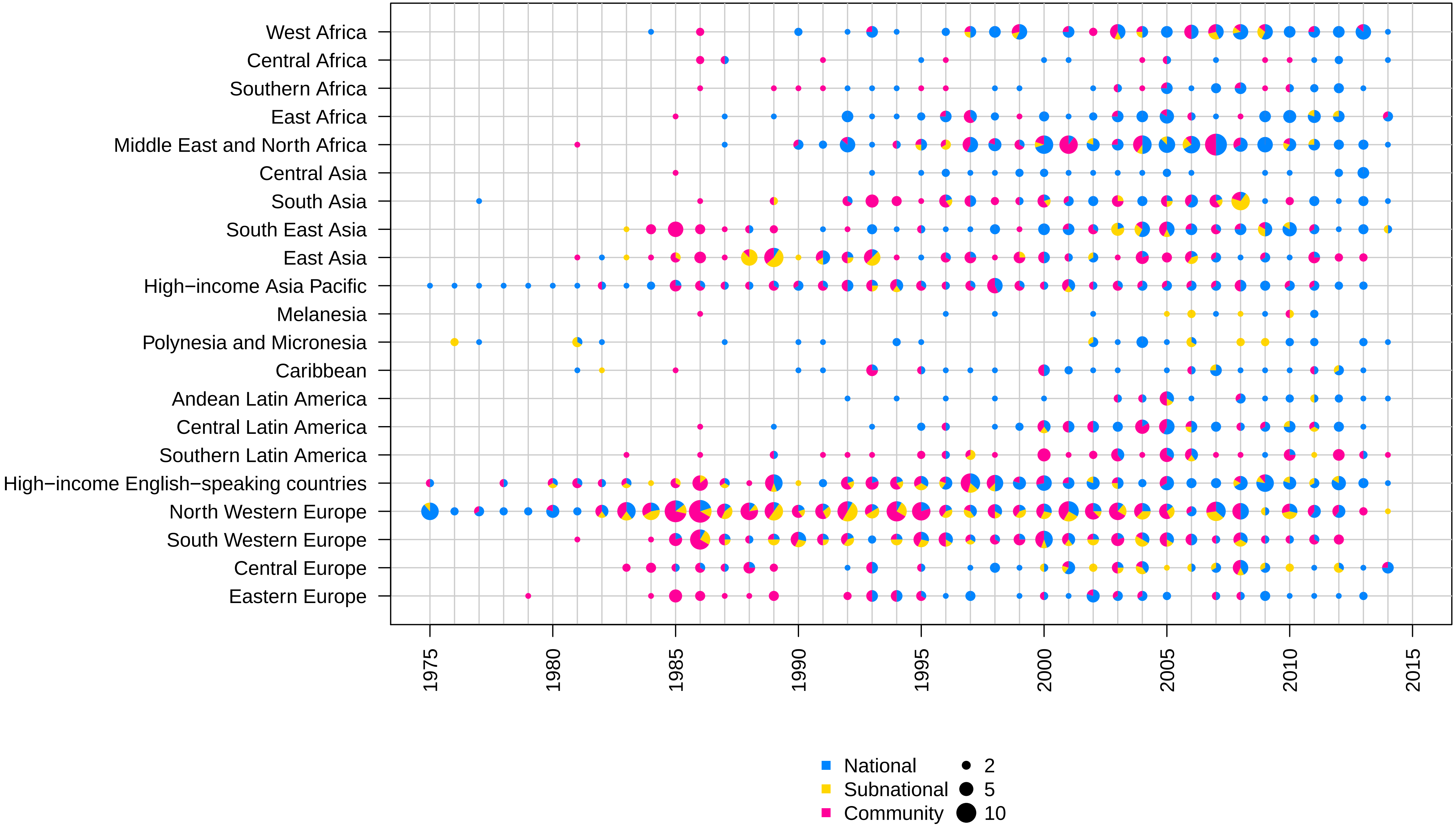

West Africa
Central Africa
Southern Africa
East Africa
Middle East and North Africa
Central Asia
South Asia
South East Asia
East Asia
High−income Asia Pacific
Melanesia
Polynesia and Micronesia
Caribbean
Andean Latin America
Central Latin America
Southern Latin America
High−income English−speaking countries
North Western Europe
South Western Europe
Central Europe
Eastern Europe
1975
1980
1985
1990
1995
2000
2005
2010
2015
National
Subnational
Community
2
5
10

Appendix Figure 4: Comparison of age-standardised mean BMI estimated using models with and without covariates.

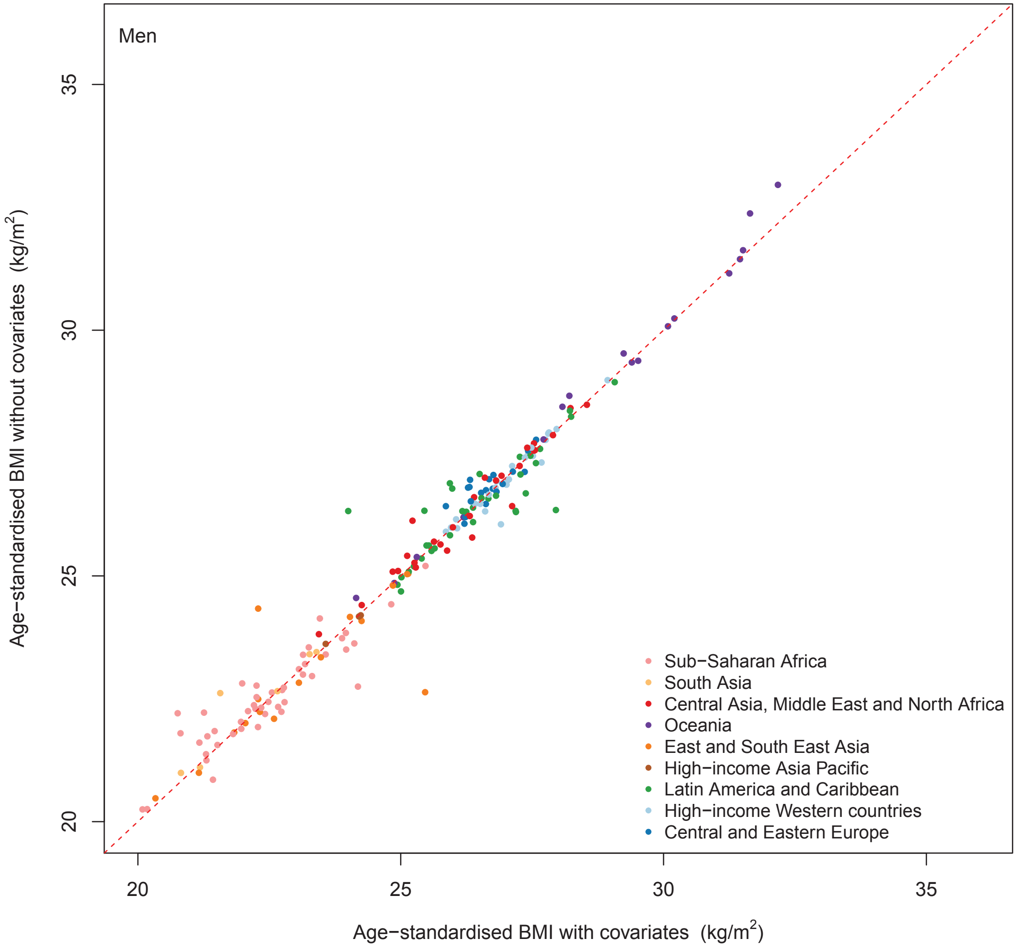
Men
Age−standardised BMI without covariates (kg/m2)
Age−standardised BMI with covariates (kg/m2)
20
25
30
35
Sub−Saharan Africa
South Asia
Central Asia, Middle East and North Africa
Oceania
East and South East Asia
High−income Asia Pacific
Latin America and Caribbean
High−income Western countries
Central and Eastern Europe

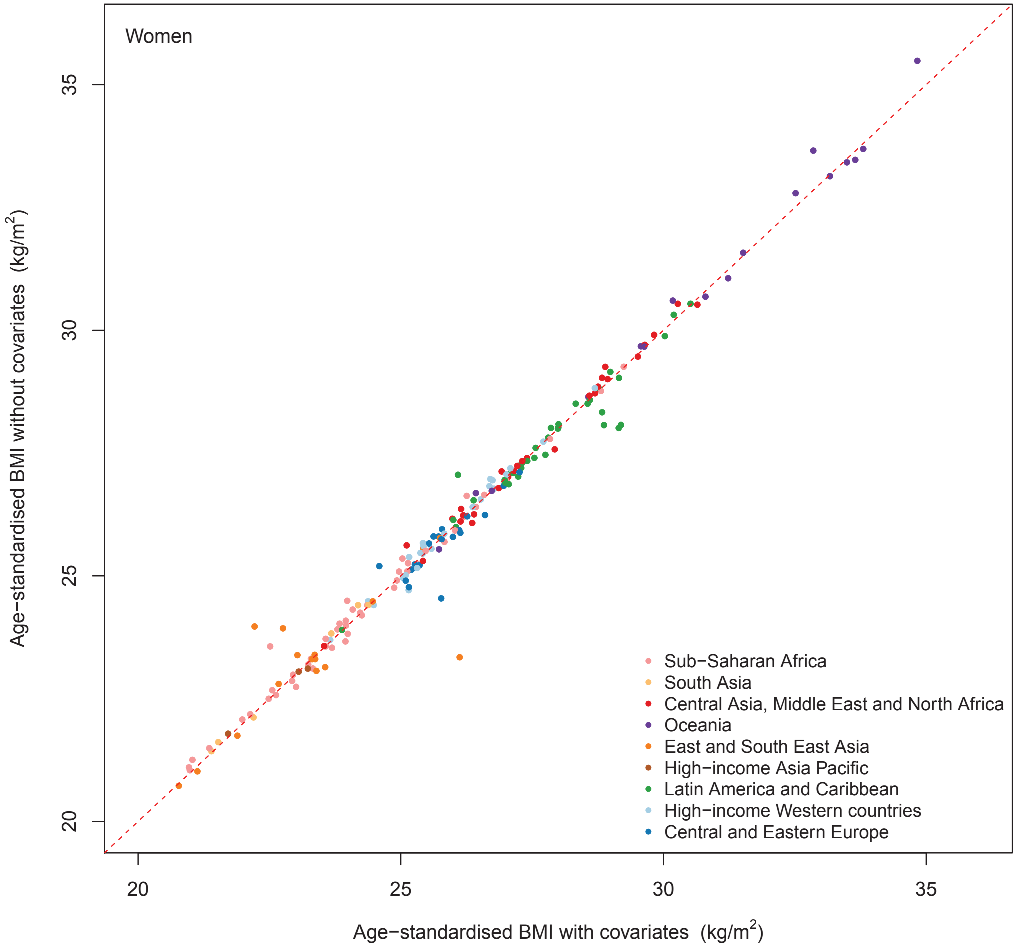
Women
Age−standardised BMI without covariates (kg/m2)
Age−standardised BMI with covariates (kg/m2)
20
25
30
35
Sub−Saharan Africa
South Asia
Central Asia, Middle East and North Africa
Oceania
East and South East Asia
High−income Asia Pacific
Latin America and Caribbean
High−income Western countries
Central and Eastern Europe

Appendix Figure 5: Standard deviation (SD) of BMI in 1975 vs. 2014, for ages 20-24 years, 45-49 years and 70-74 years. Each point shows one country.

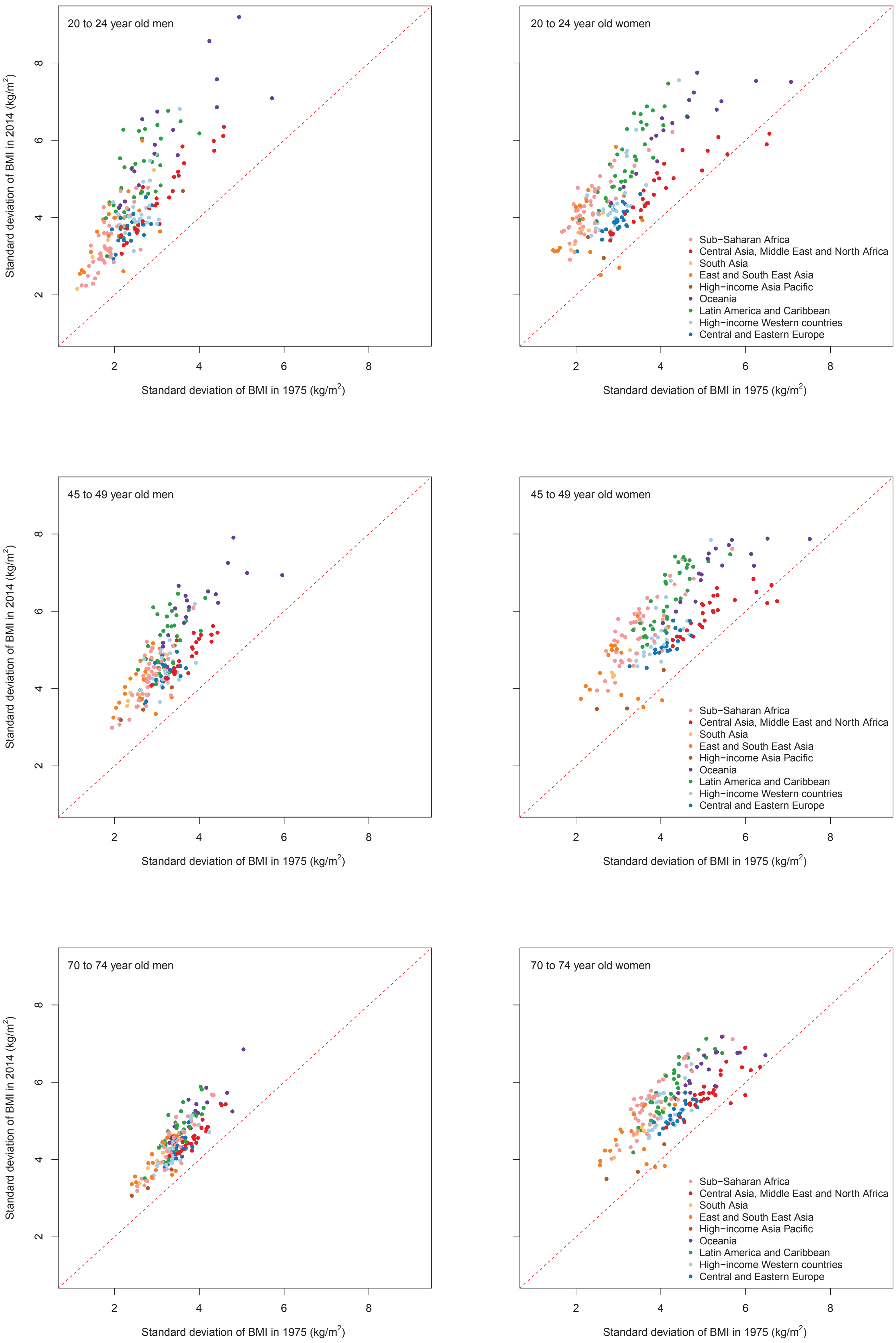
20 to 24 year old men
Standard deviation of BMI in 2014 (kg/m²)
Standard deviation of BMI in 1975 (kg/m²)
20 to 24 year old women
Sub−Saharan Africa
Central Asia, Middle East and North Africa
South Asia
East and South East Asia
High−income Asia Pacific
Oceania
Latin America and Caribbean
High−income Western countries
Central and Eastern Europe
Standard deviation of BMI in 1975 (kg/m²)
45 to 49 year old men
Standard deviation of BMI in 2014 (kg/m²)
Standard deviation of BMI in 1975 (kg/m²)
45 to 49 year old women
Sub−Saharan Africa
Central Asia, Middle East and North Africa
South Asia
East and South East Asia
High−income Asia Pacific
Oceania
Latin America and Caribbean
High−income Western countries
Central and Eastern Europe
Standard deviation of BMI in 1975 (kg/m²)
70 to 74 year old men
Standard deviation of BMI in 2014 (kg/m²)
Standard deviation of BMI in 1975 (kg/m²)
70 to 74 year old women
Sub−Saharan Africa
Central Asia, Middle East and North Africa
South Asia
East and South East Asia
High−income Asia Pacific
Oceania
Latin America and Caribbean
High−income Western countries
Central and Eastern Europe
Standard deviation of BMI in 1975 (kg/m²)

Appendix Figure 6: Male vs. female age standardised mean BMI in 1975 and 2014, and change from 1975 to 2014.

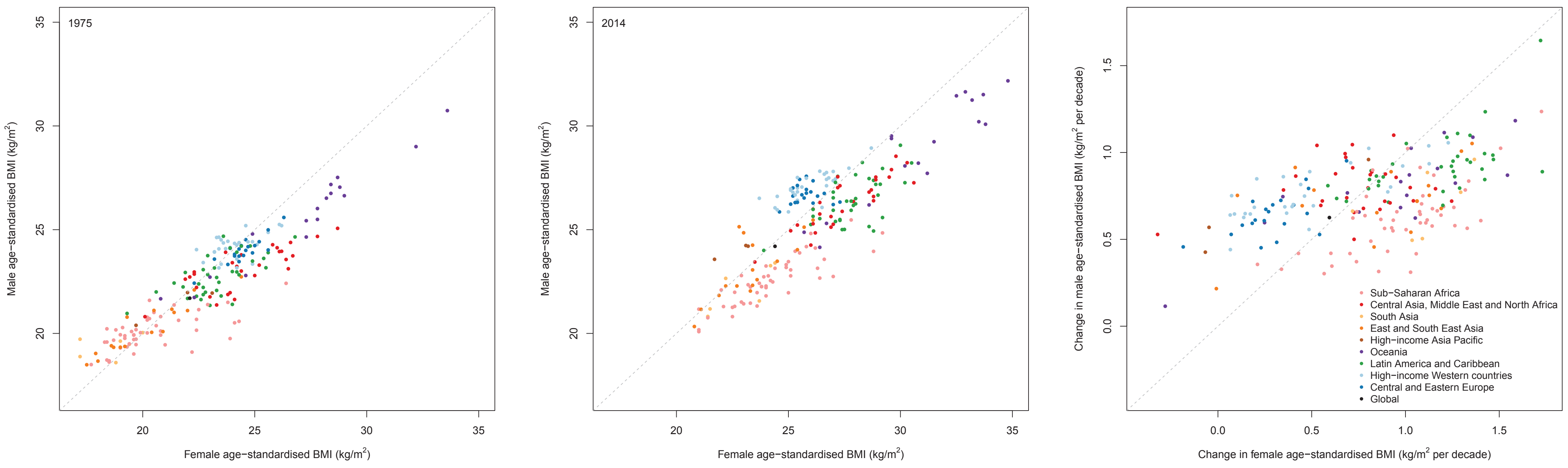
1975
2014
Male age−standardised BMI (kg/m^2)
Female age−standardised BMI (kg/m^2)
Change in male age−standardised BMI (kg/m^2 per decade)
Change in female age−standardised BMI (kg/m^2 per decade)
Sub−Saharan Africa
Central Asia, Middle East and North Africa
South Asia
East and South East Asia
High−income Asia Pacific
Oceania
Latin America and Caribbean
High−income Western countries
Central and Eastern Europe
Global

Appendix Figure 7: Probability of achieving the target of halting the rise of obesity compared to its 2010 levels by country if post-2000 trends continue.

Men

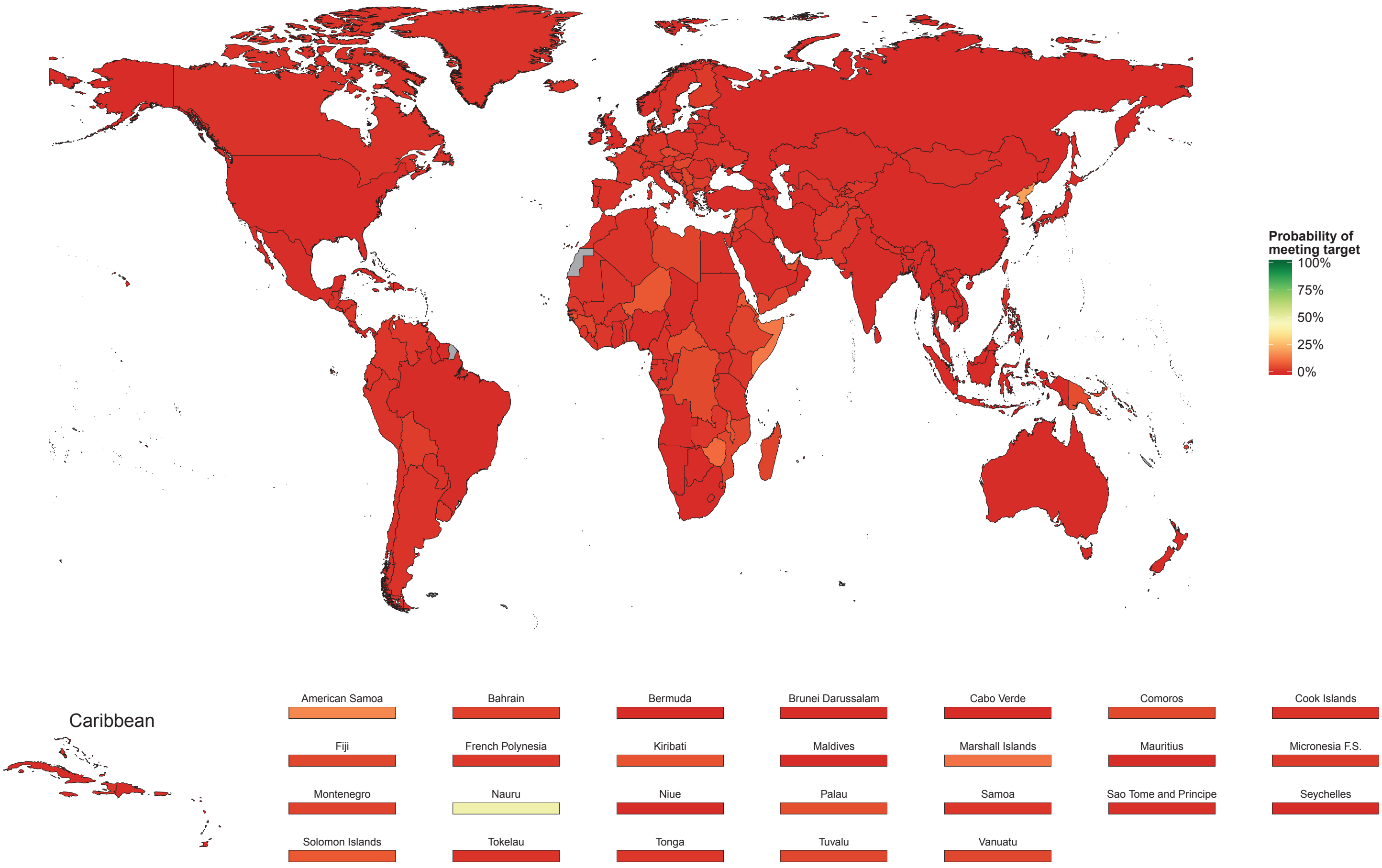
Probability of meeting target
100%
75%
50%
25%
0%
Caribbean
American Samoa
Bahrain
Bermuda
Brunei Darussalam
Cabo Verde
Comoros
Cook Islands
Fiji
French Polynesia
Kiribati
Maldives
Marshall Islands
Mauritius
Micronesia F.S.
Montenegro
Nauru
Niue
Palau
Samoa
Sao Tome and Principe
Seychelles
Solomon Islands
Tokelau
Tonga
Tuvalu
Vanuatu

Women

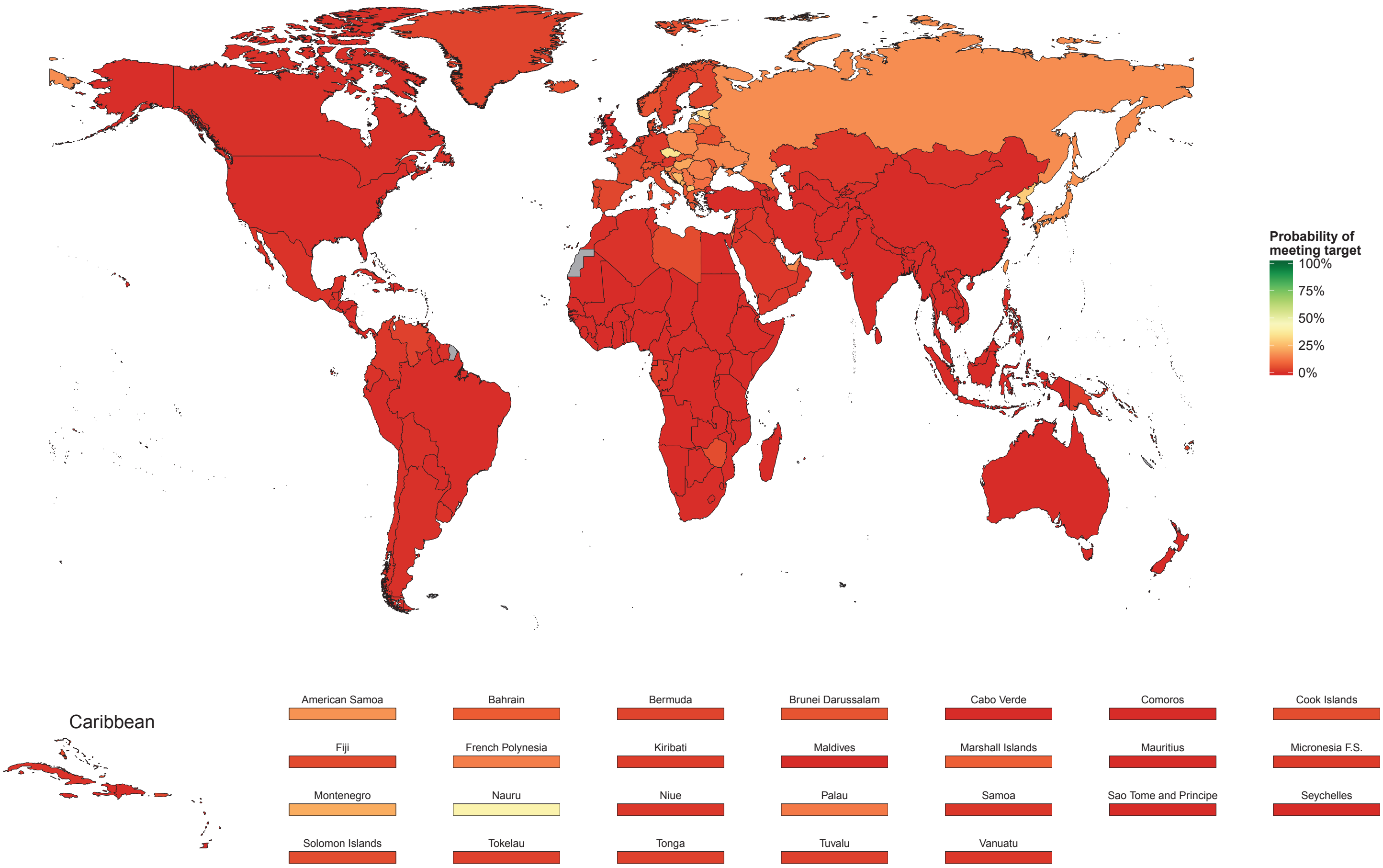
Probability of meeting target
100%
75%
50%
25%
0%
Caribbean
American Samoa
Bahrain
Bermuda
Brunei Darussalam
Cabo Verde
Comoros
Cook Islands
Fiji
French Polynesia
Kiribati
Maldives
Marshall Islands
Mauritius
Micronesia F.S.
Montenegro
Nauru
Niue
Palau
Samoa
Sao Tome and Principe
Seychelles
Solomon Islands
Tokelau
Tonga
Tuvalu
Vanuatu

Appendix Figure 8: Trends in age-standardised mean BMI and prevalences of BMI categories by sex and country.

Afghanistan
South Asia

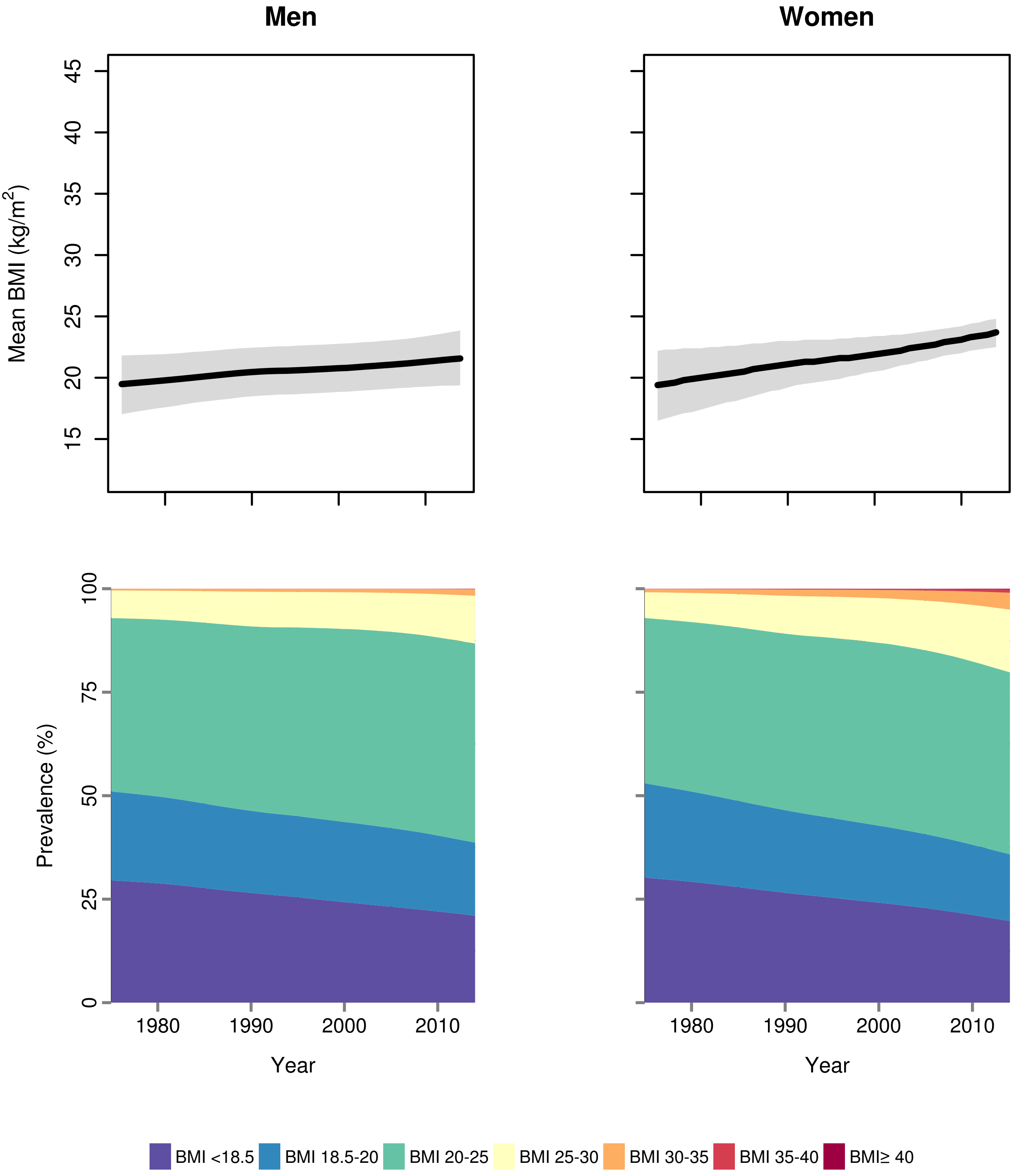

Albania
Central Europe

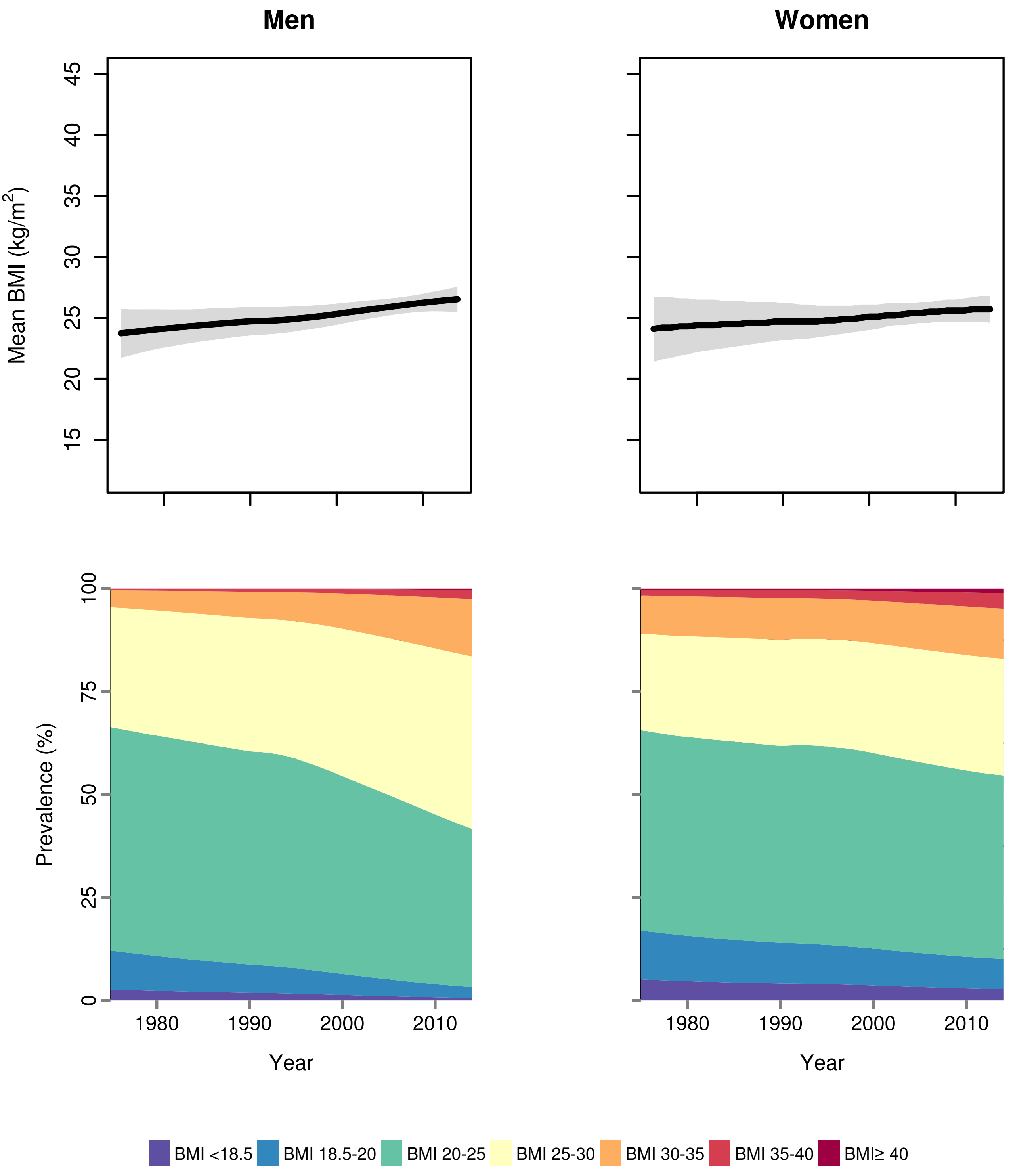

Algeria

Middle East and North Africa

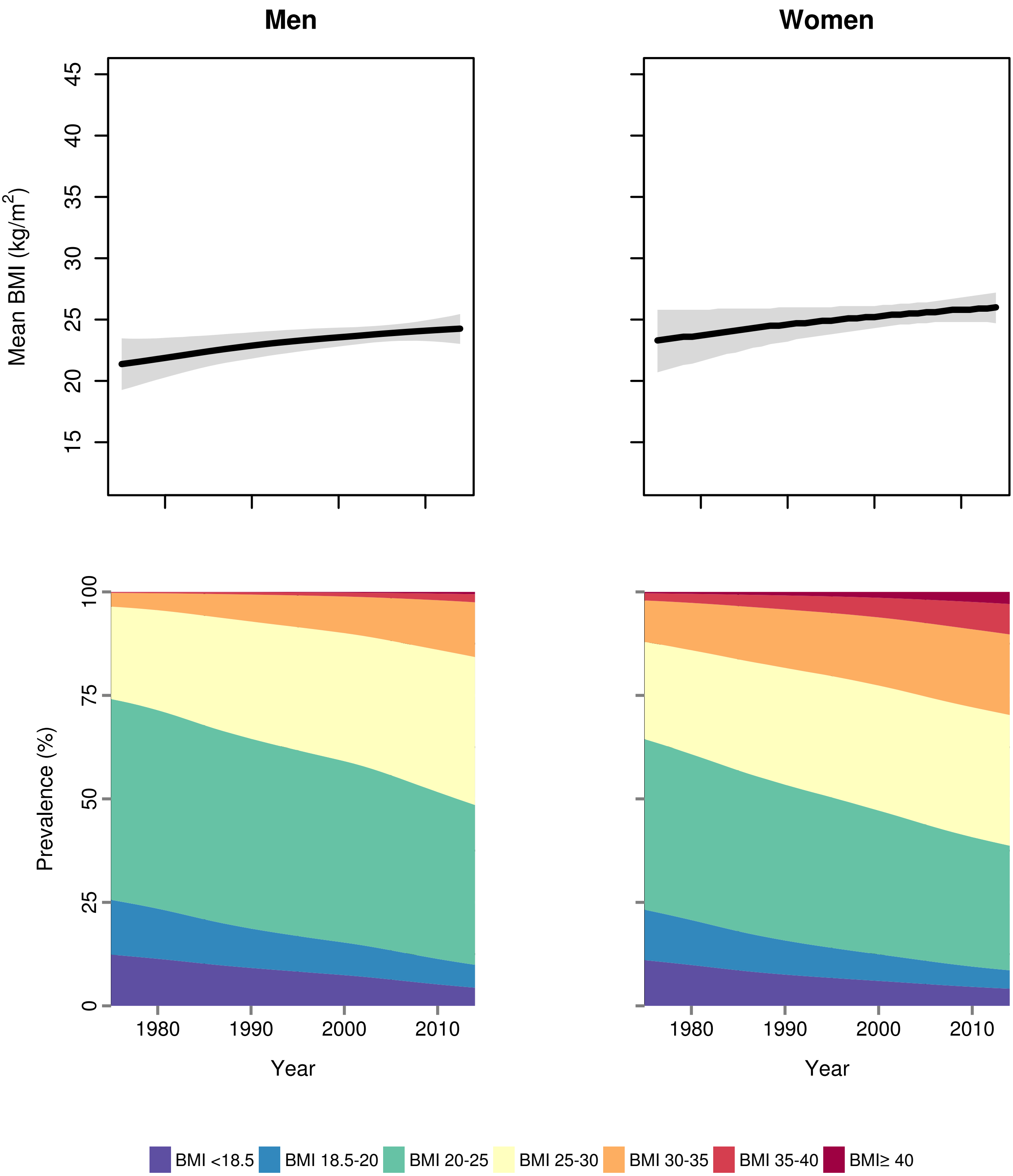

American Samoa

Polynesia and Micronesia

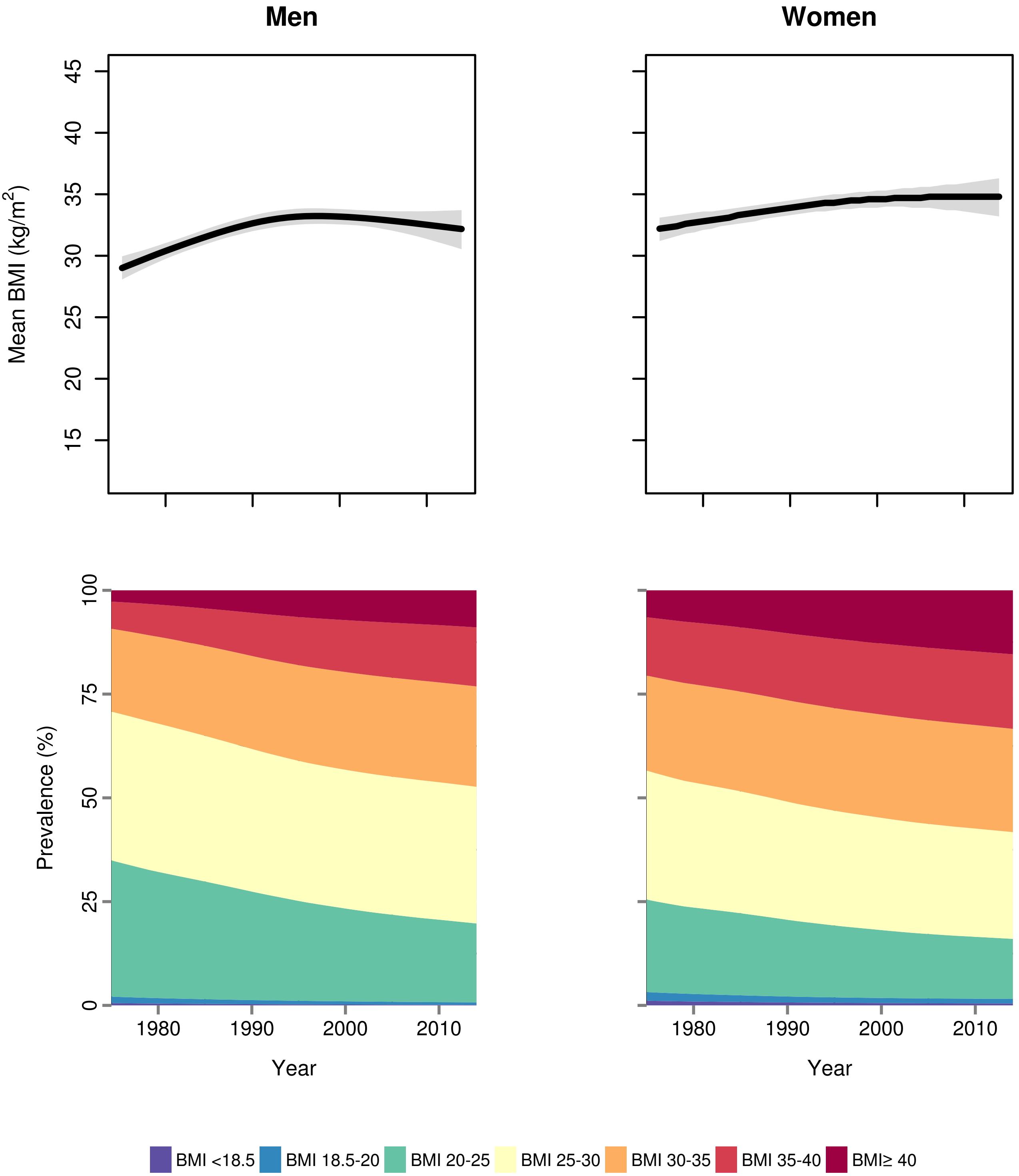

Andorra

South Western Europe

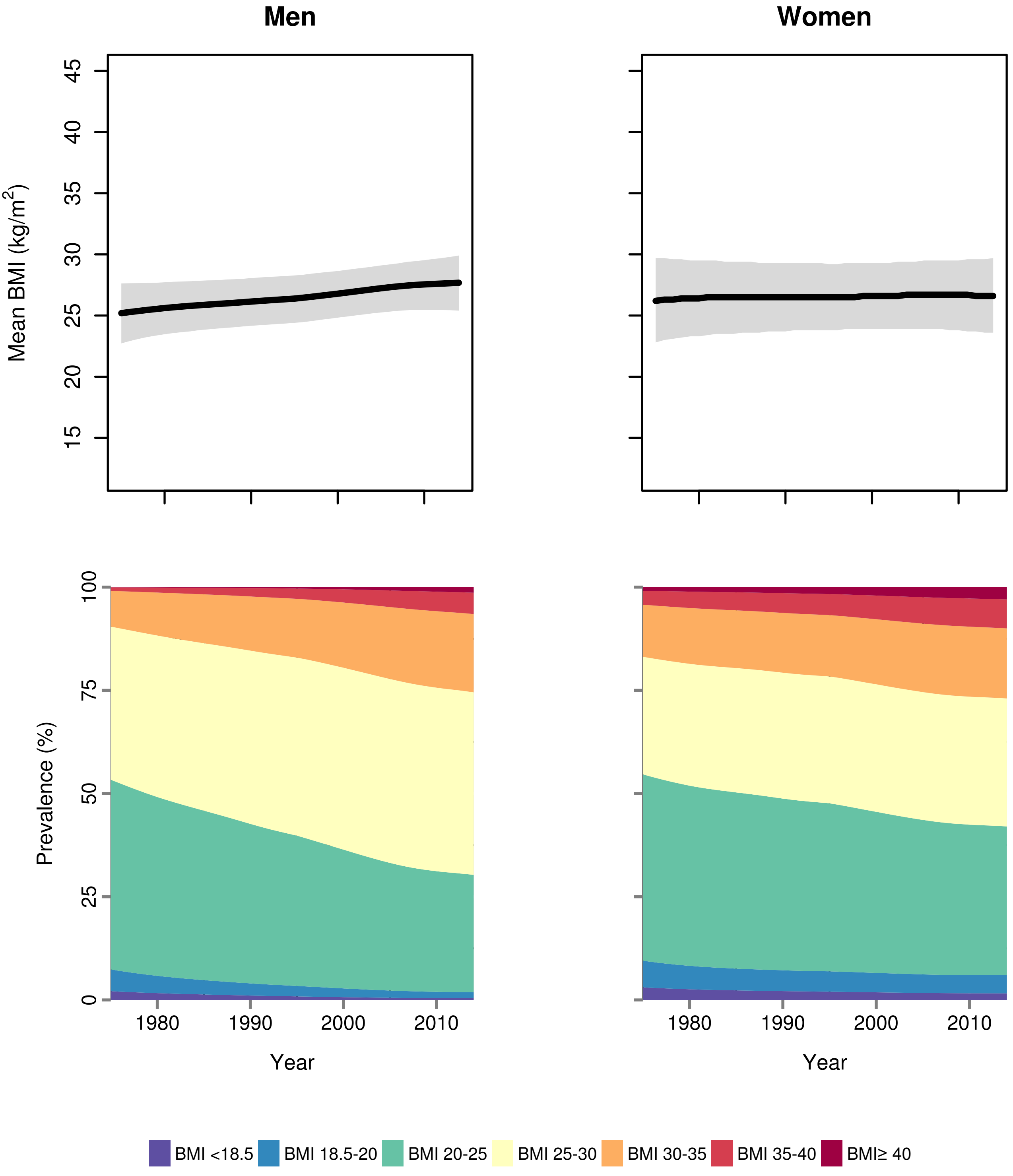

Angola
Central Africa

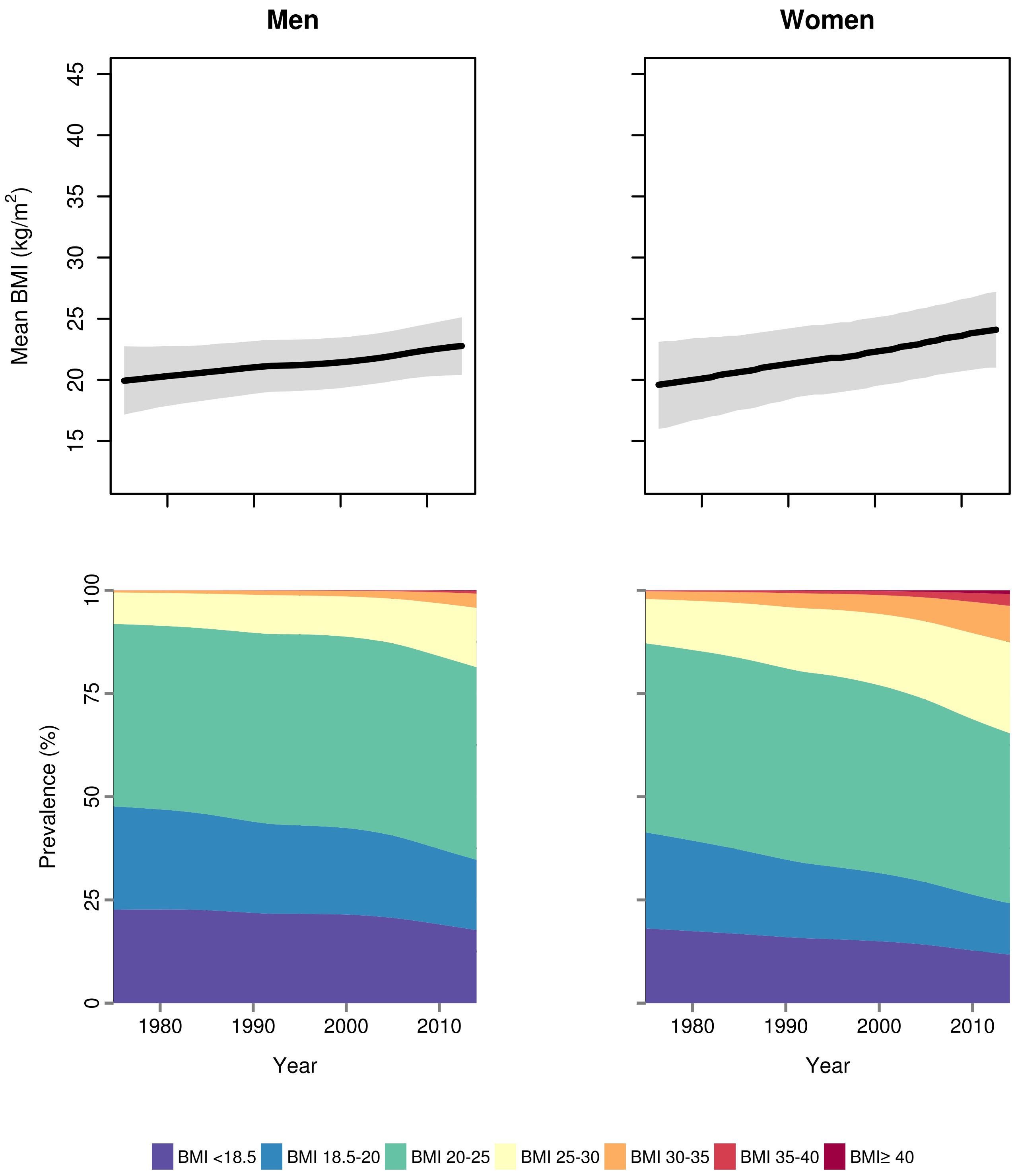

Antigua and Barbuda

Caribbean

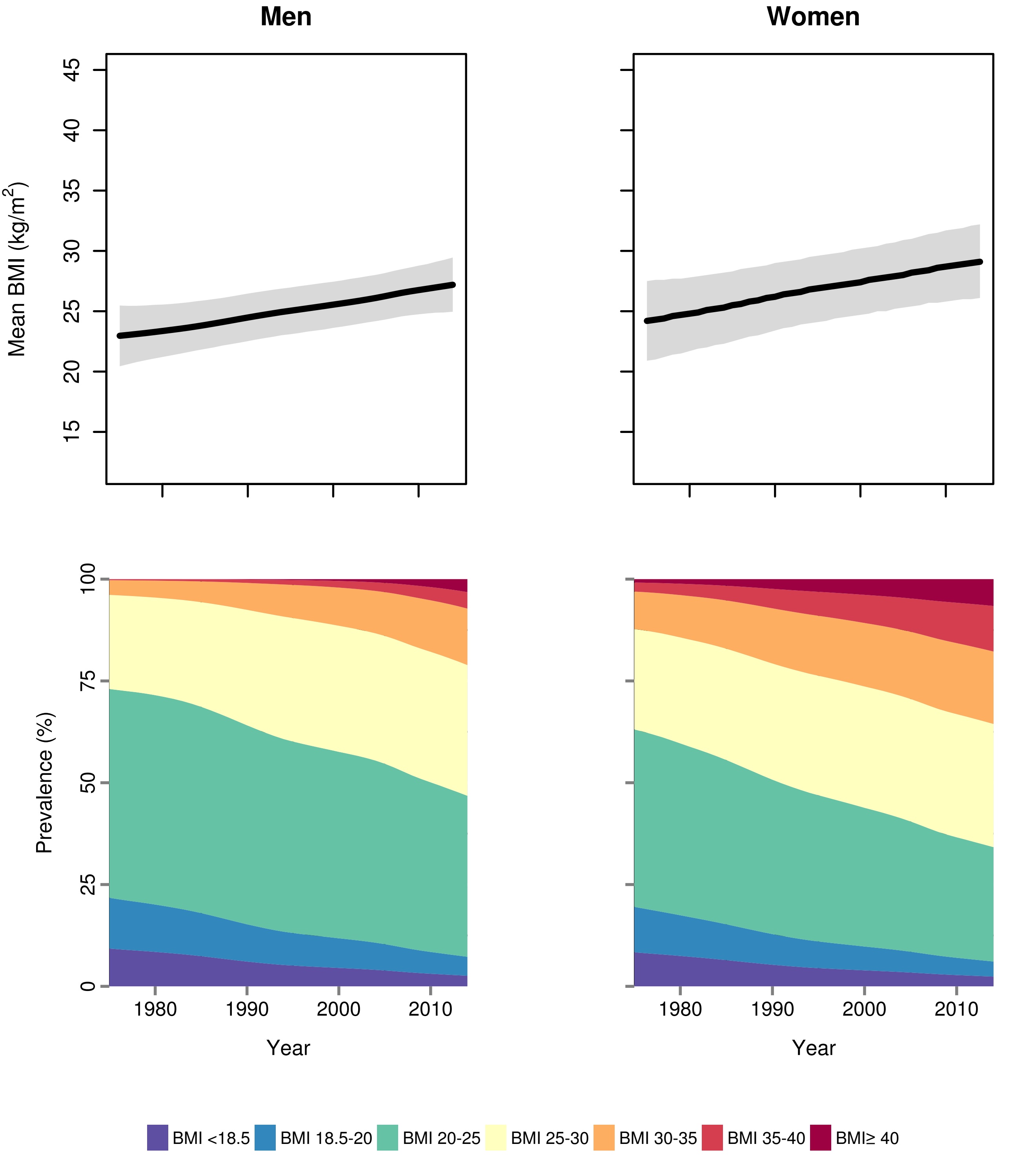

Argentina

Southern Latin America

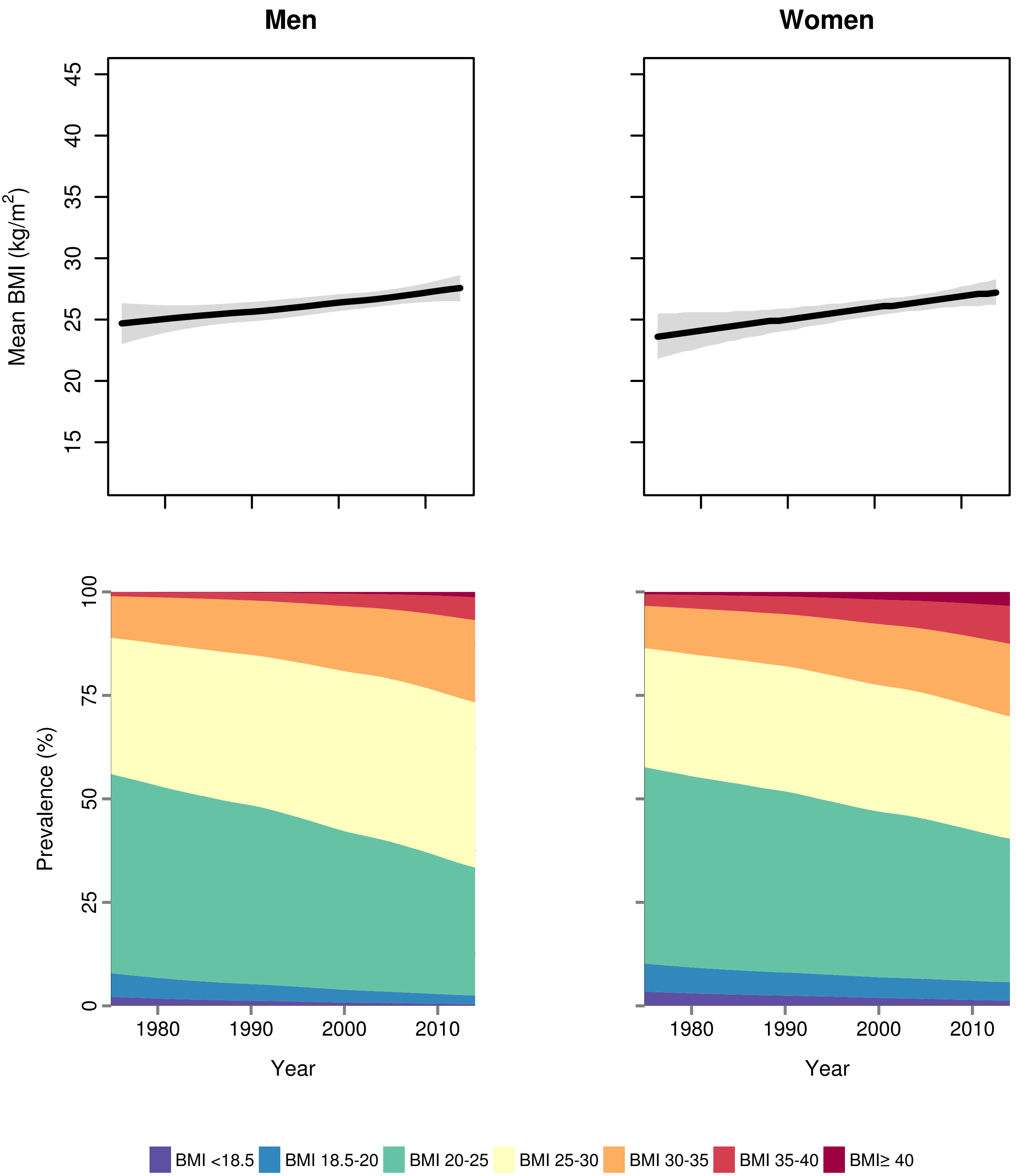

Armenia
Central Asia

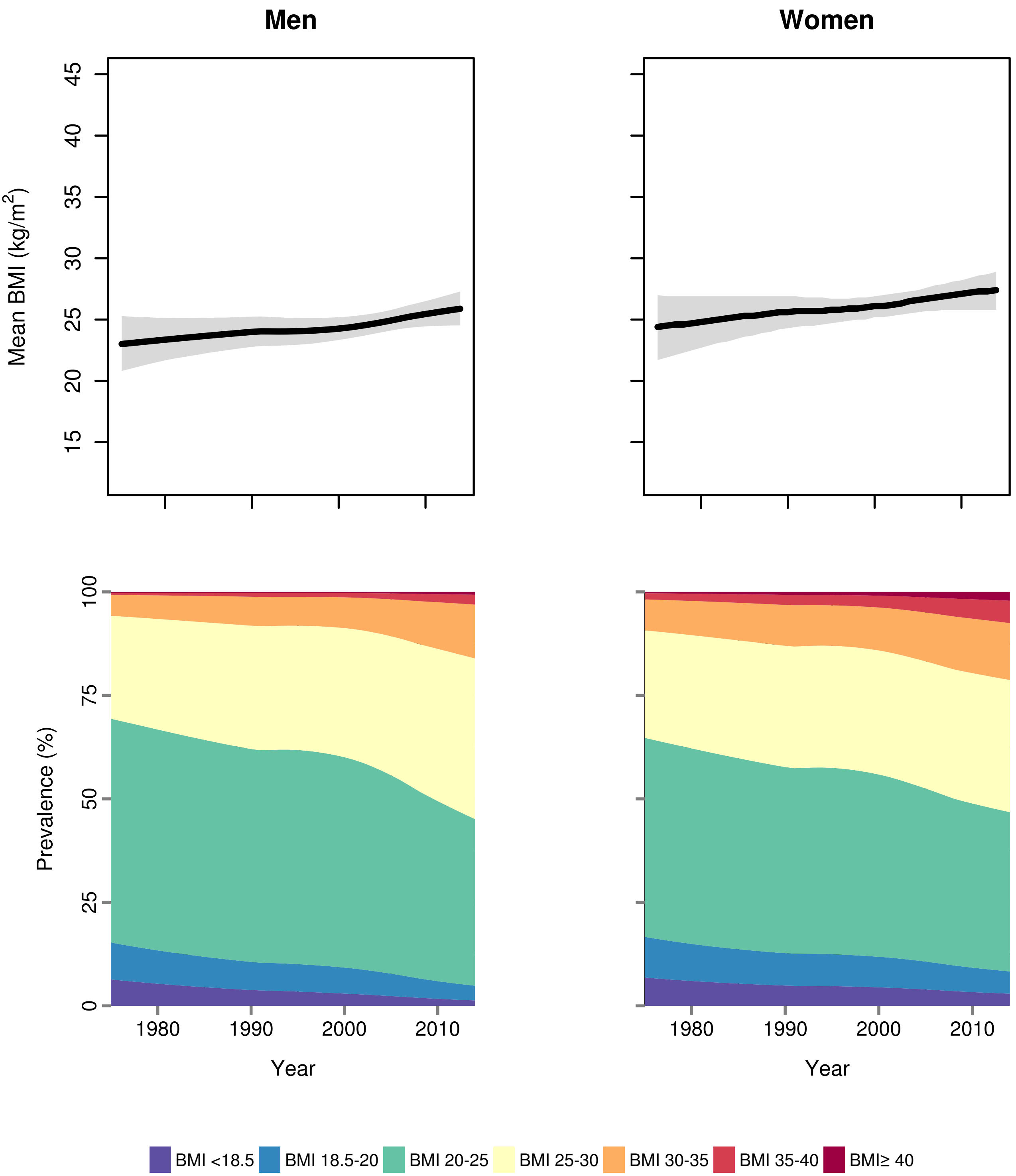

Australia

High-income English-speaking countries

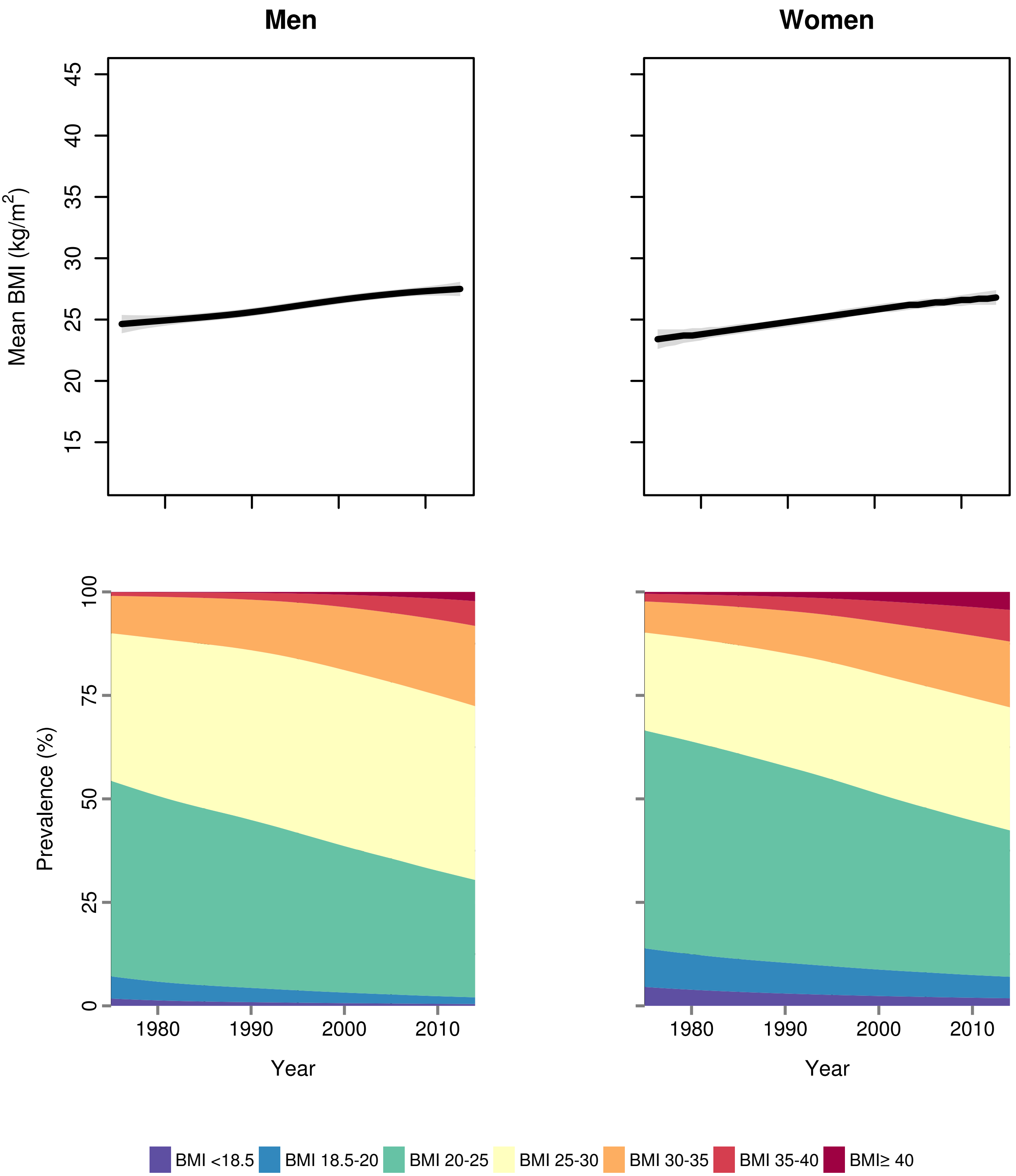

Austria

North Western Europe

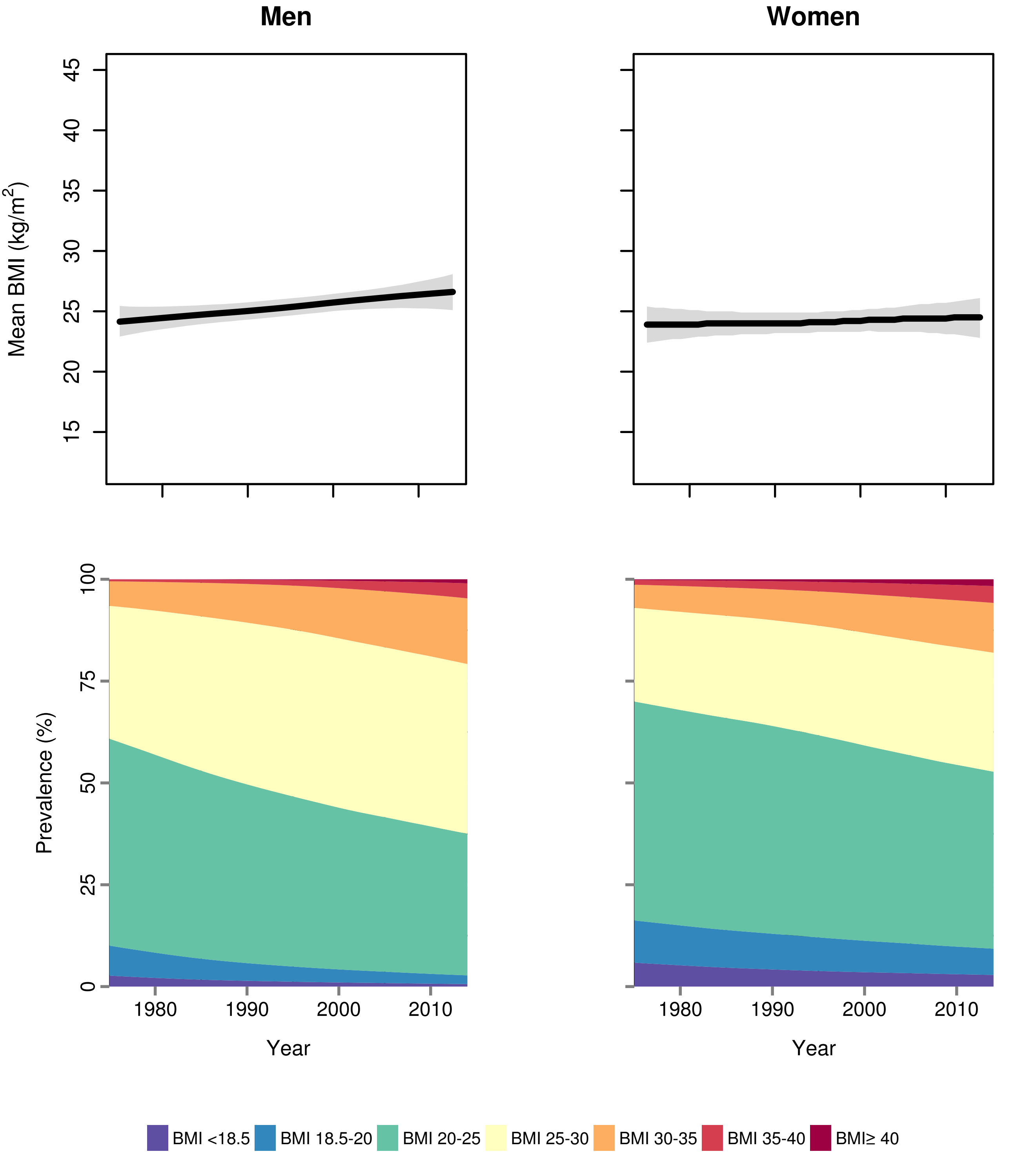

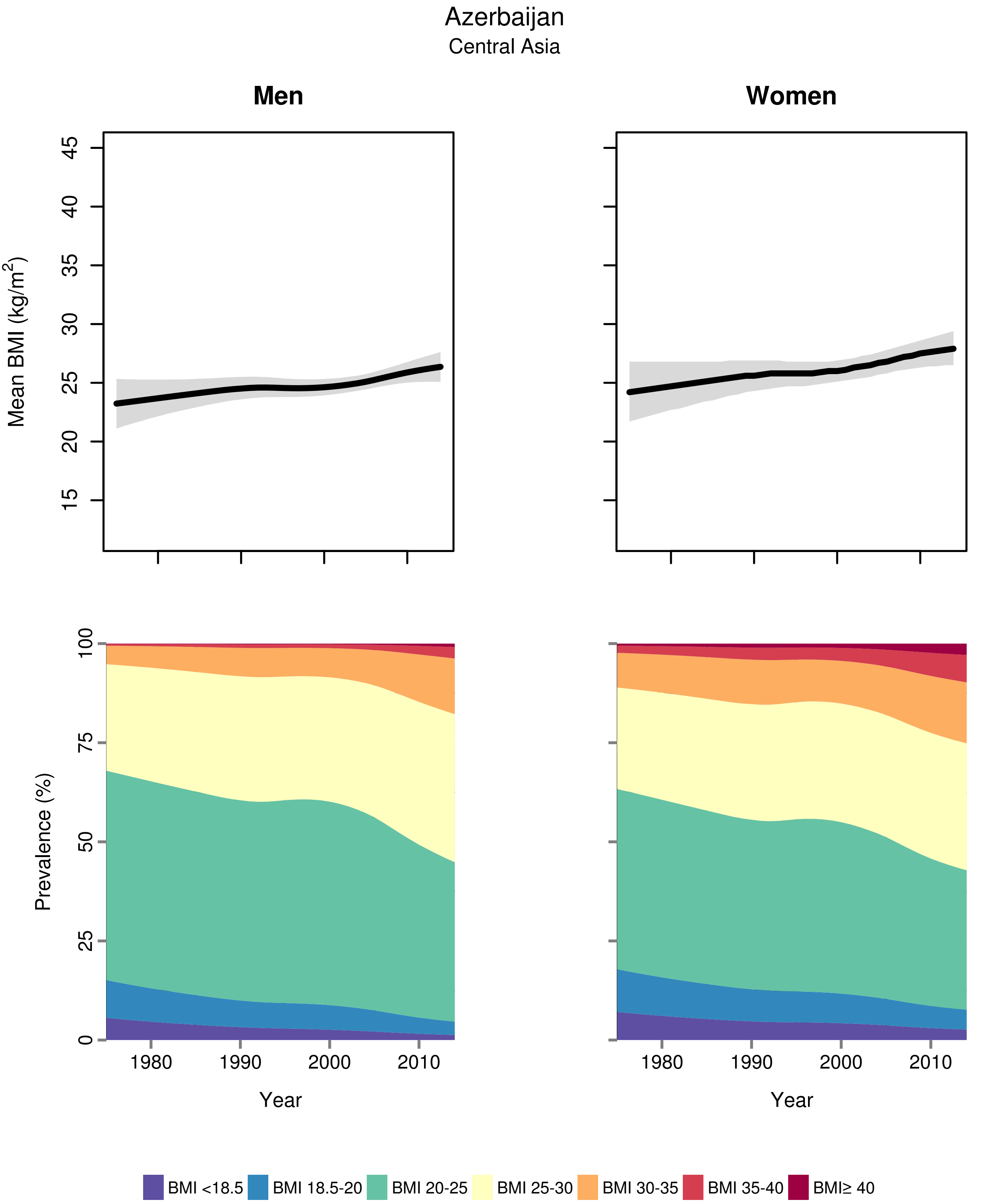
Azerbaijan
Central Asia
Men
Women
Mean BMI (kg/m²)
45
40
35
30
25
20
15
Prevalence (%)
100
75
50
25
0
1980
1990
2000
2010
Year
BMI <18.5
BMI 18.5-20
BMI 20-25
BMI 25-30
BMI 30-35
BMI 35-40
BMI≥ 40

Bahamas

Caribbean

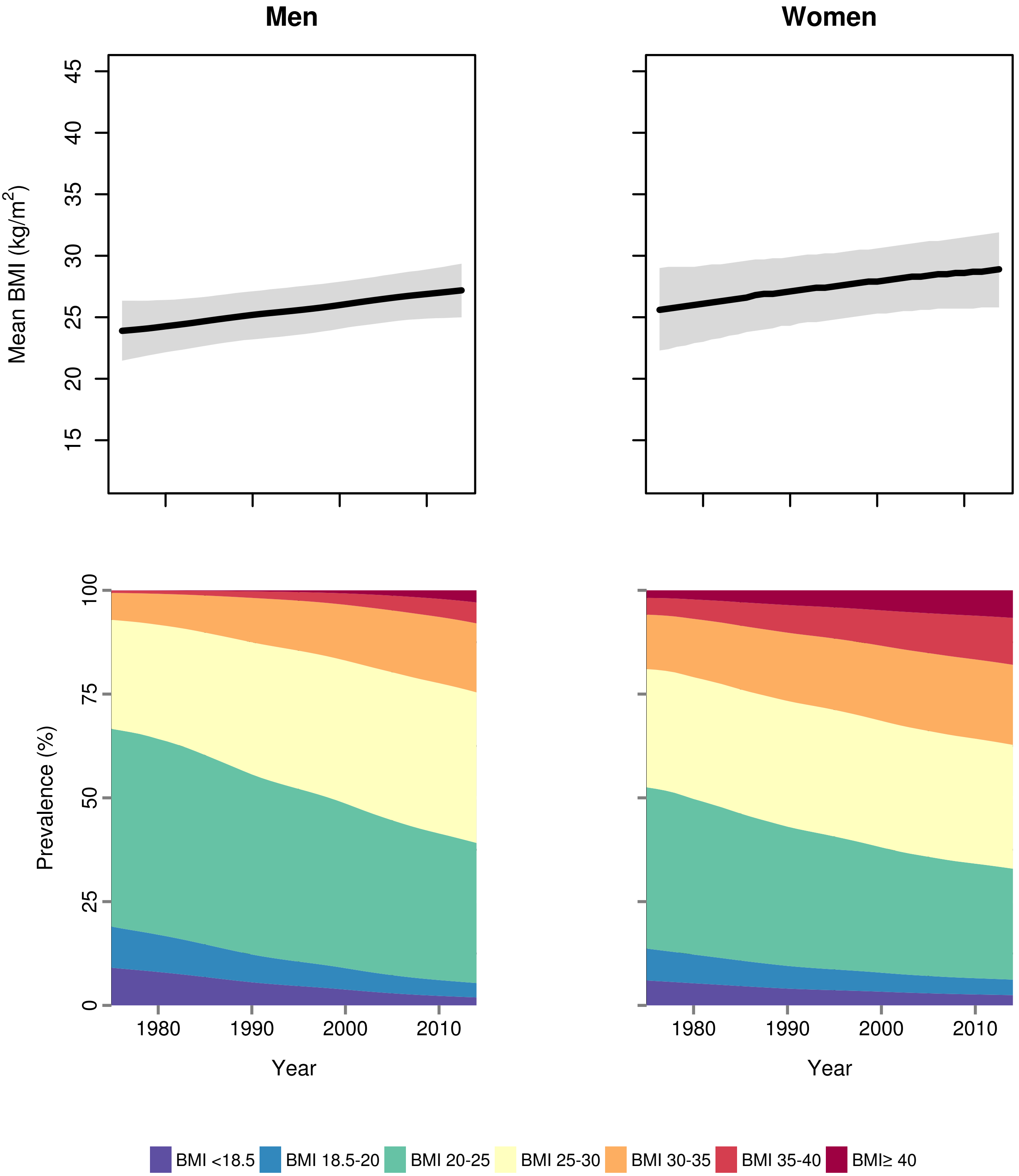

Bahrain

Middle East and North Africa

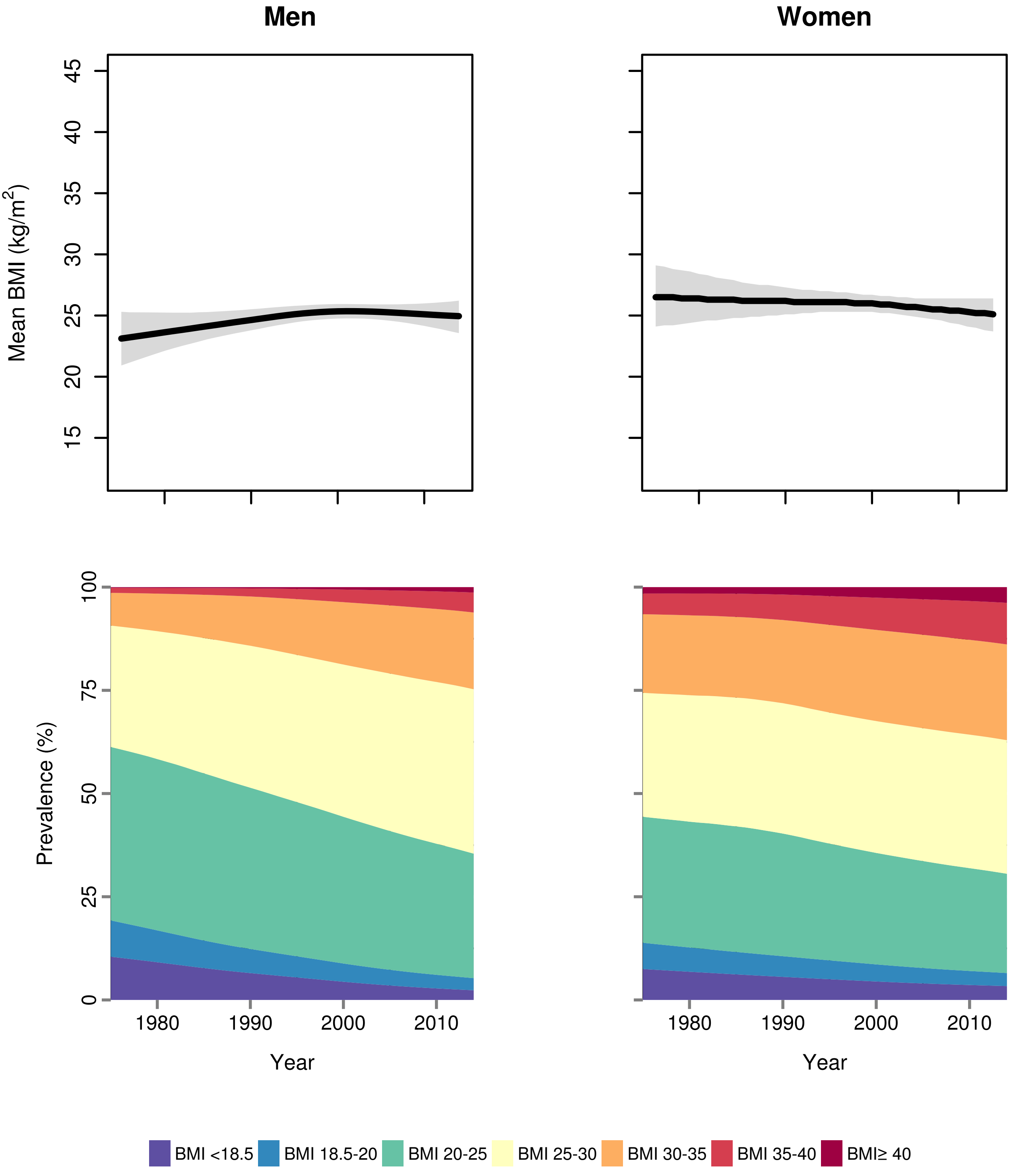

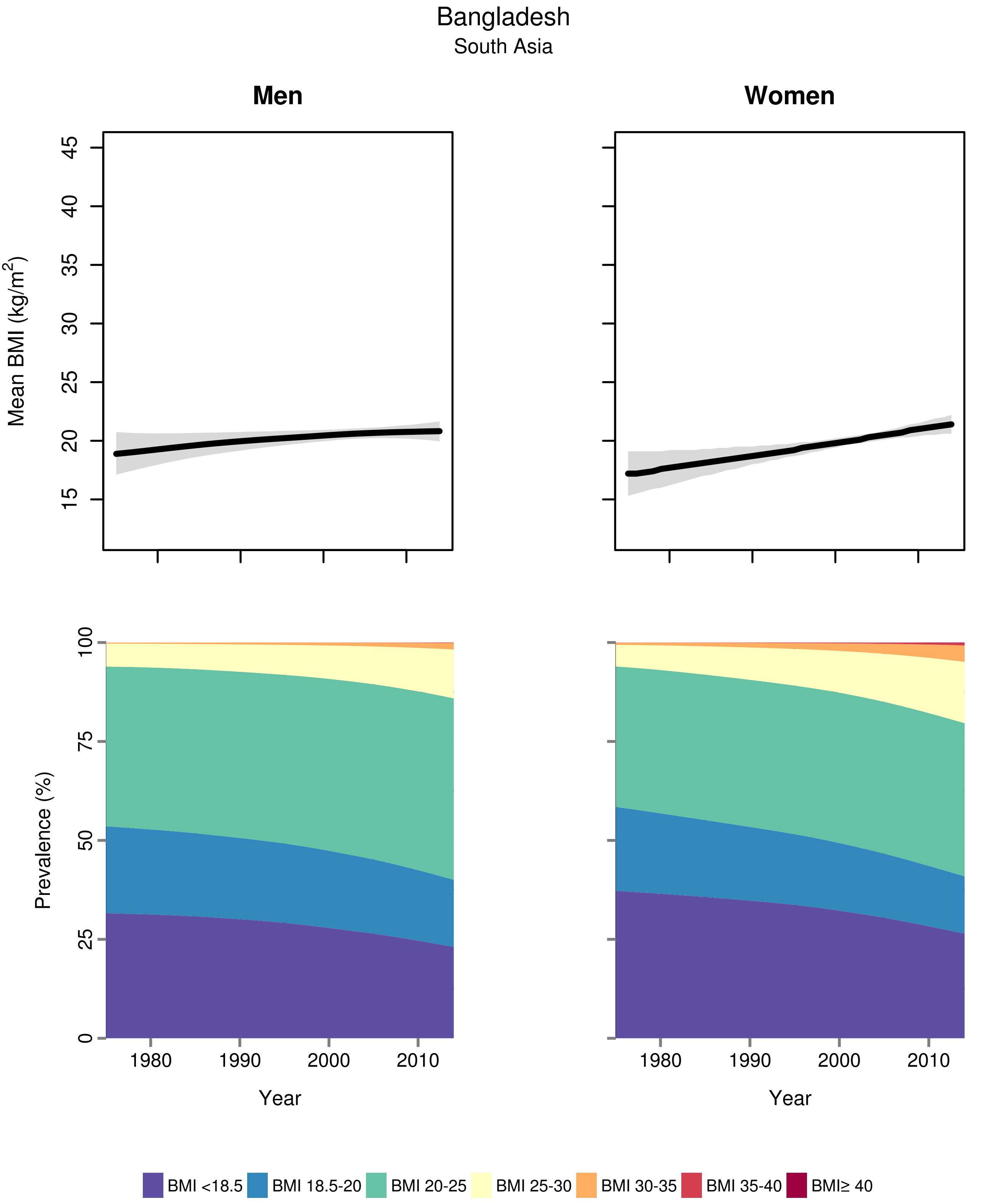

Bangladesh
South Asia
Men
Women
Mean BMI (kg/m²)
45
40
35
30
25
20
15
Prevalence (%)
100
75
50
25
0
1980
1990
2000
2010
Year
BMI <18.5
BMI 18.5-20
BMI 20-25
BMI 25-30
BMI 30-35
BMI 35-40
BMI≥ 40

Barbados
Caribbean

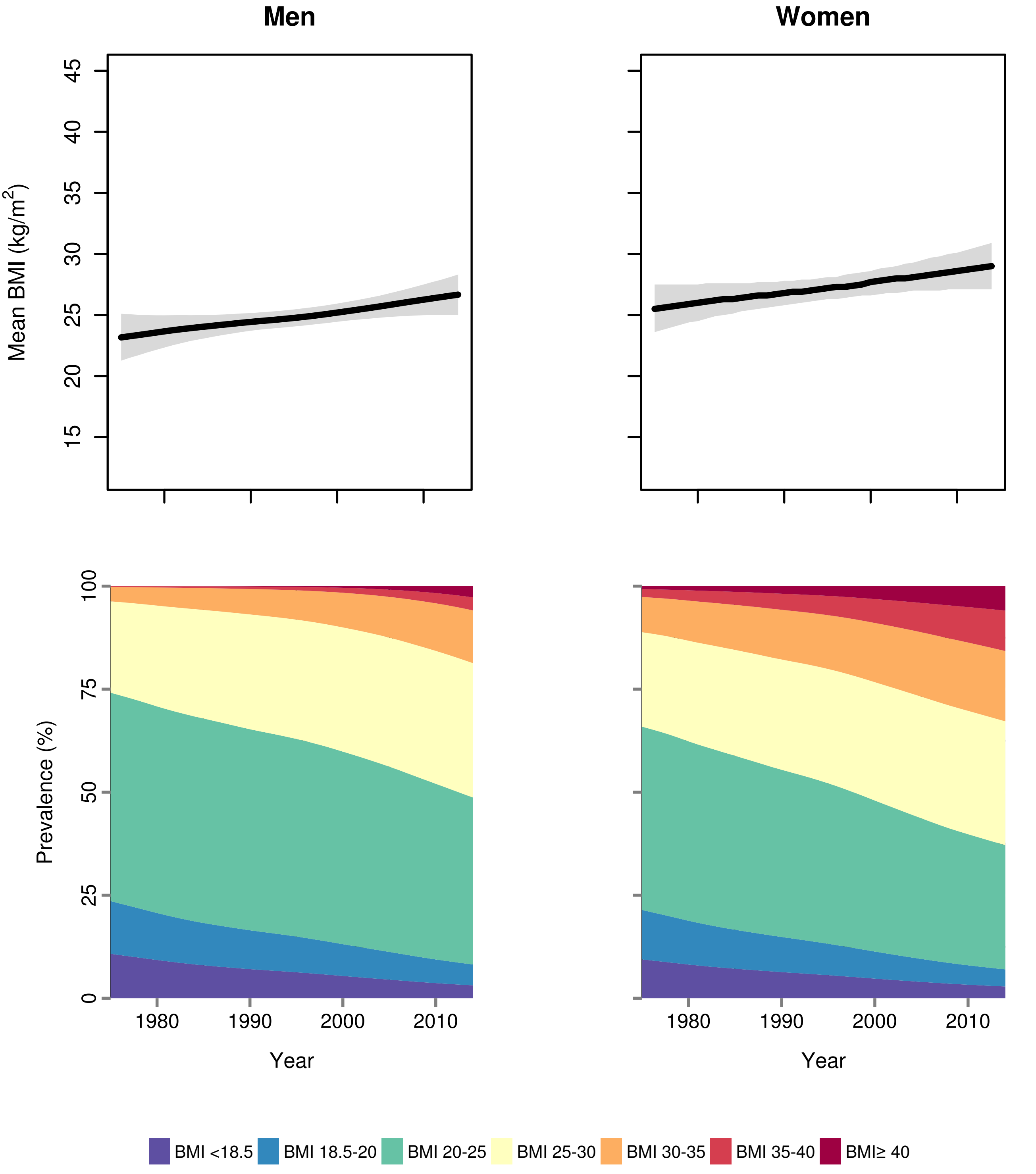

Belarus

Eastern Europe

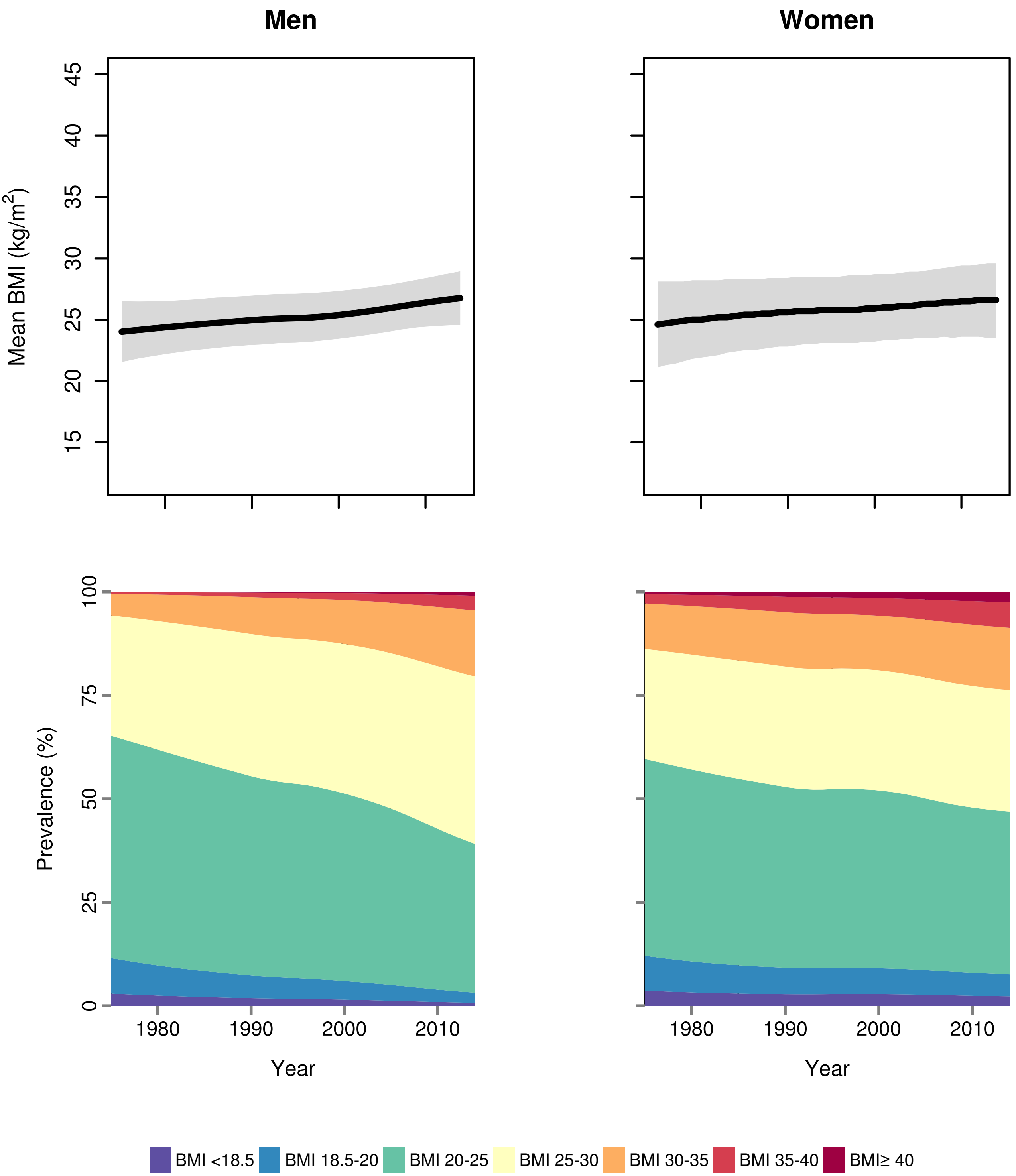

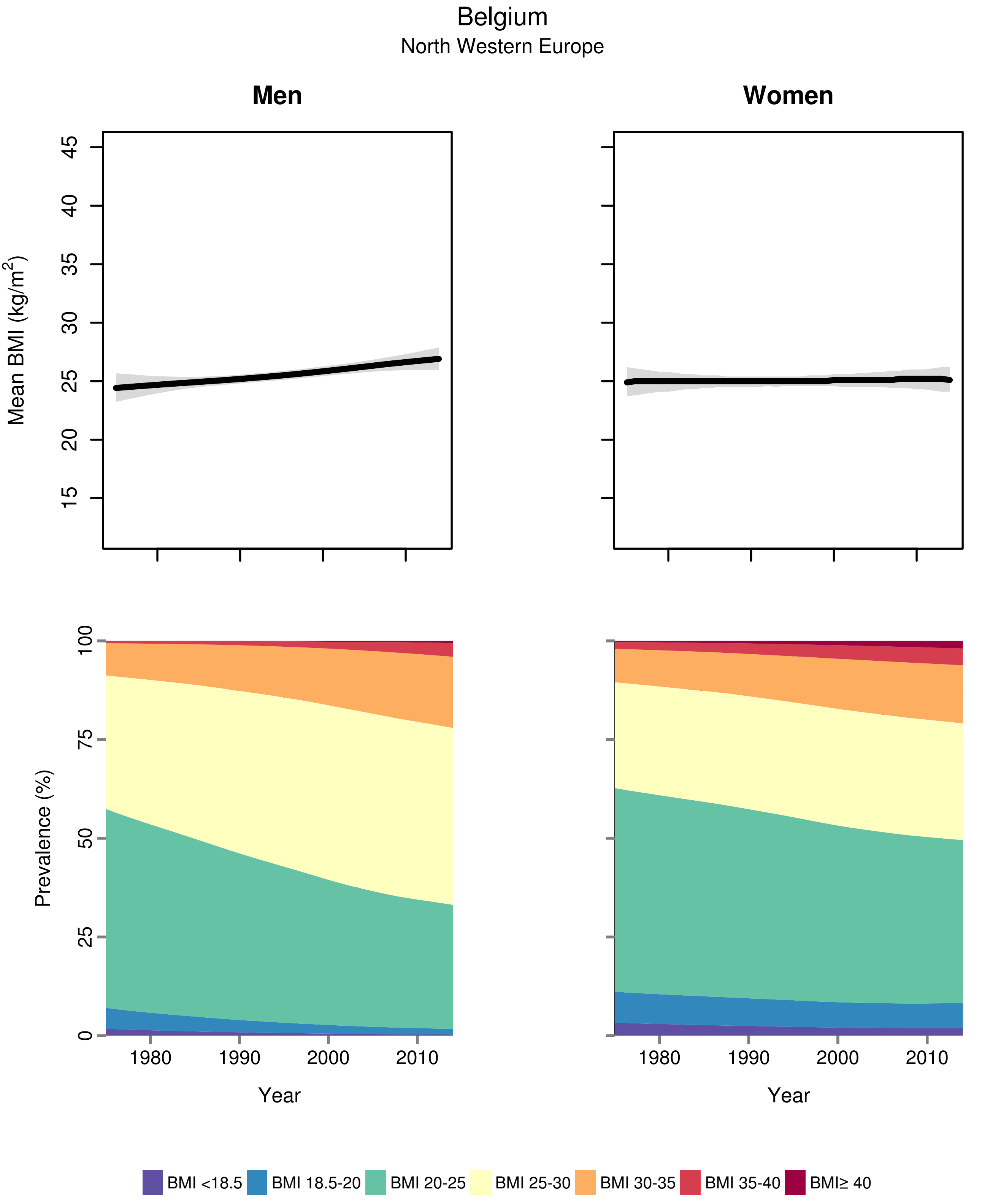
Belgium
North Western Europe
Men
Women
Mean BMI (kg/m2)
45
40
35
30
25
20
15
Prevalence (%)
100
75
50
25
0
1980
1990
2000
2010
Year
BMI <18.5
BMI 18.5-20
BMI 20-25
BMI 25-30
BMI 30-35
BMI 35-40
BMI≥ 40

Belize

Caribbean

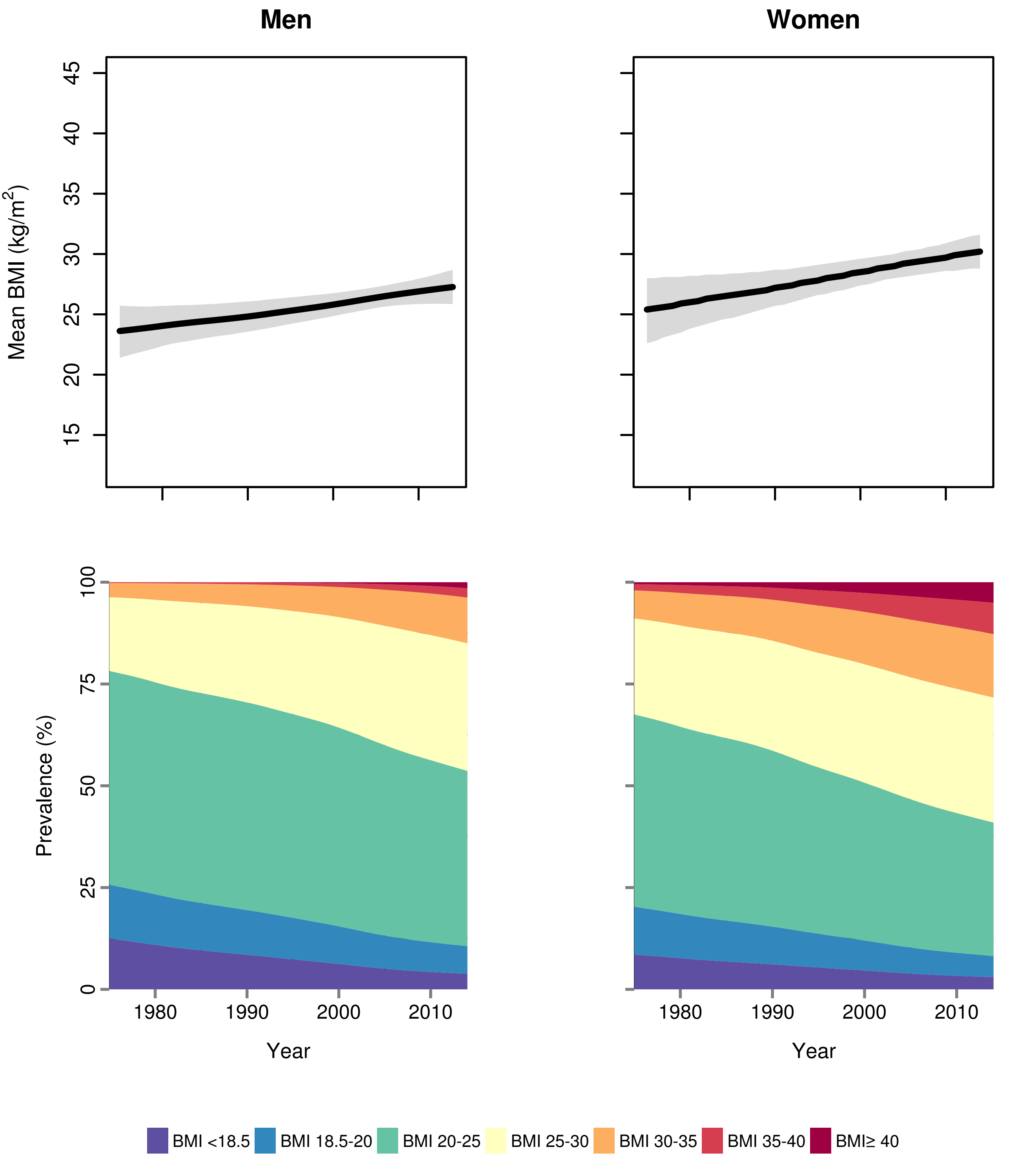

Benin

West Africa

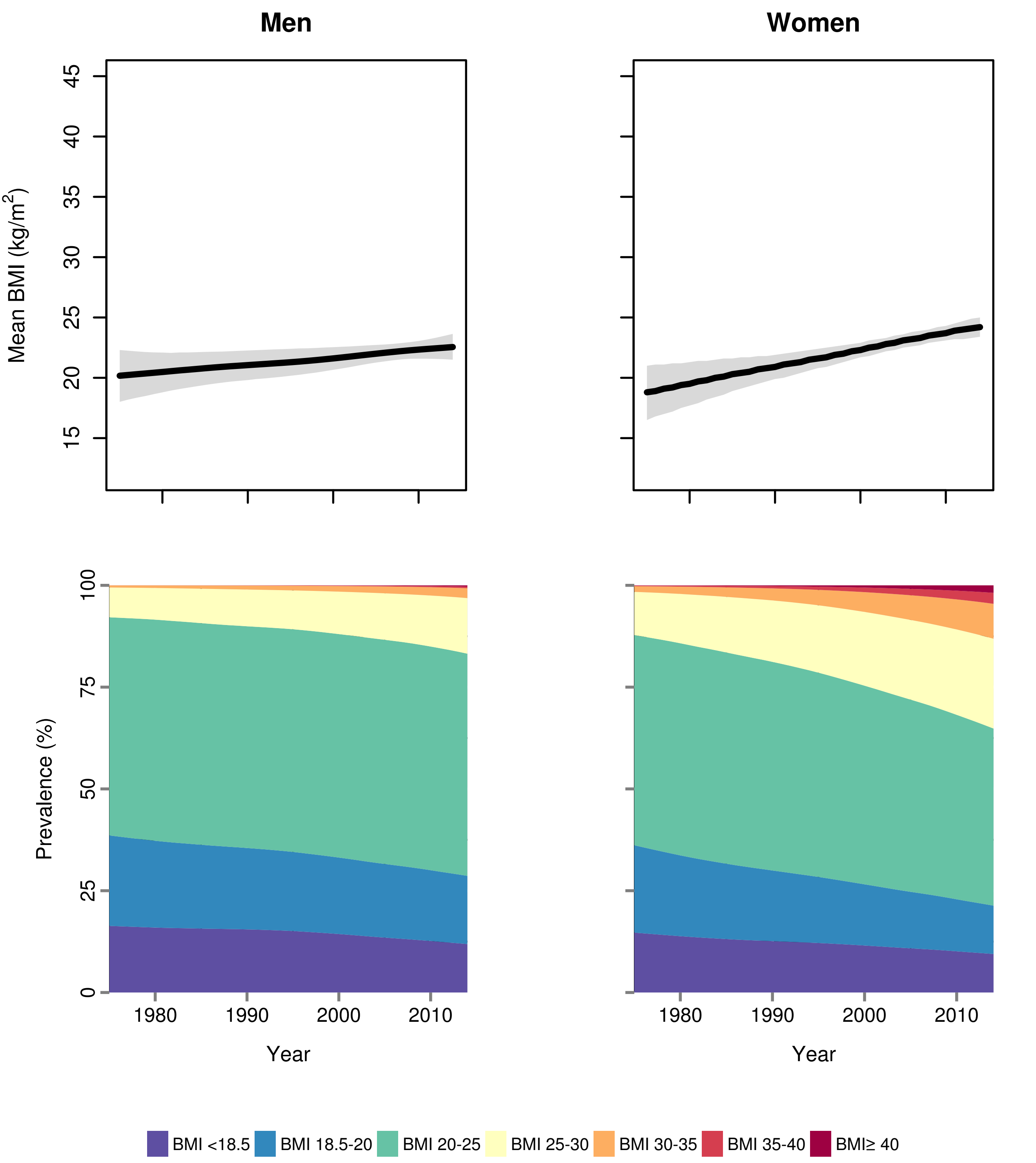

Bermuda

Caribbean

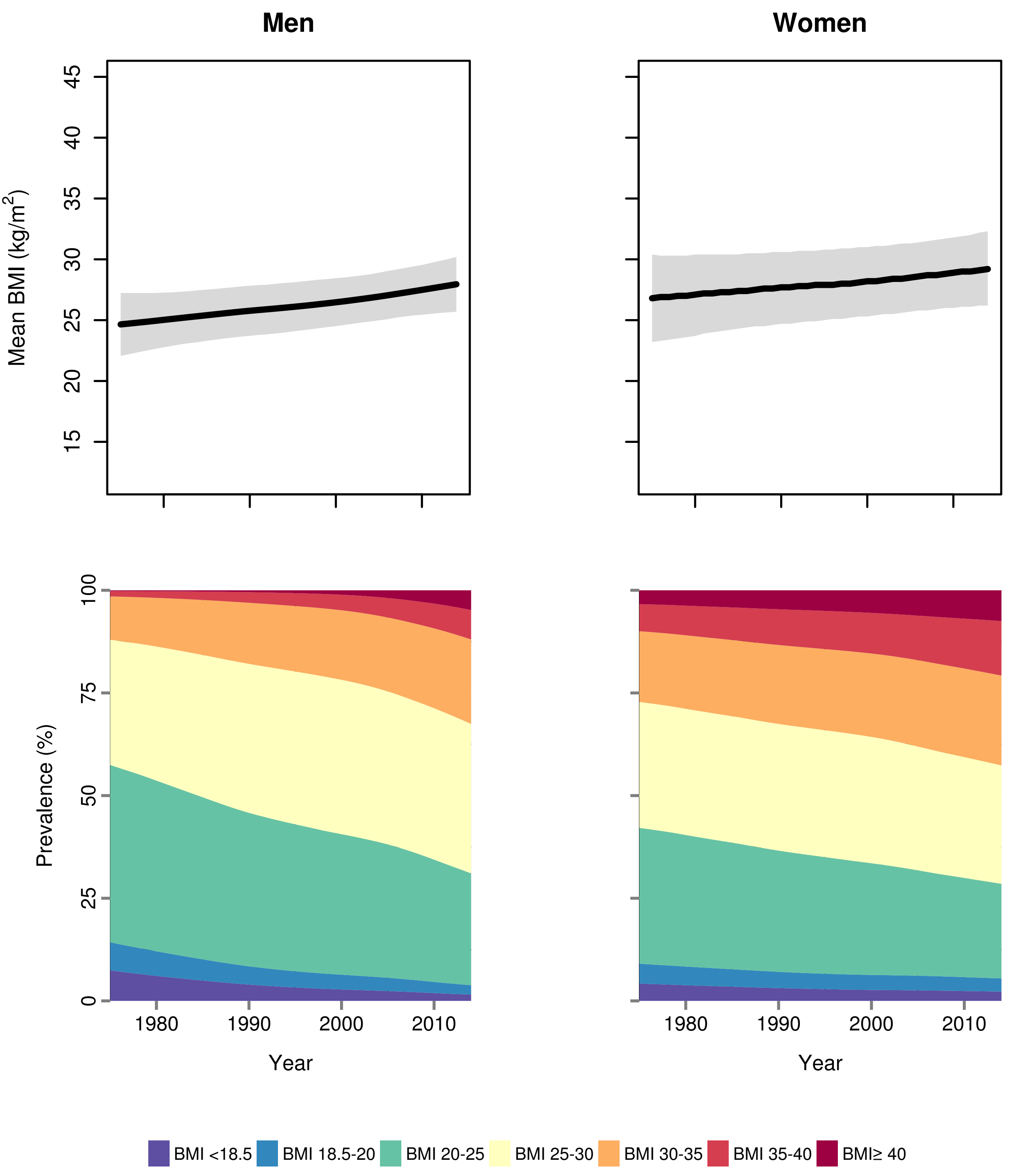

Bhutan

South Asia

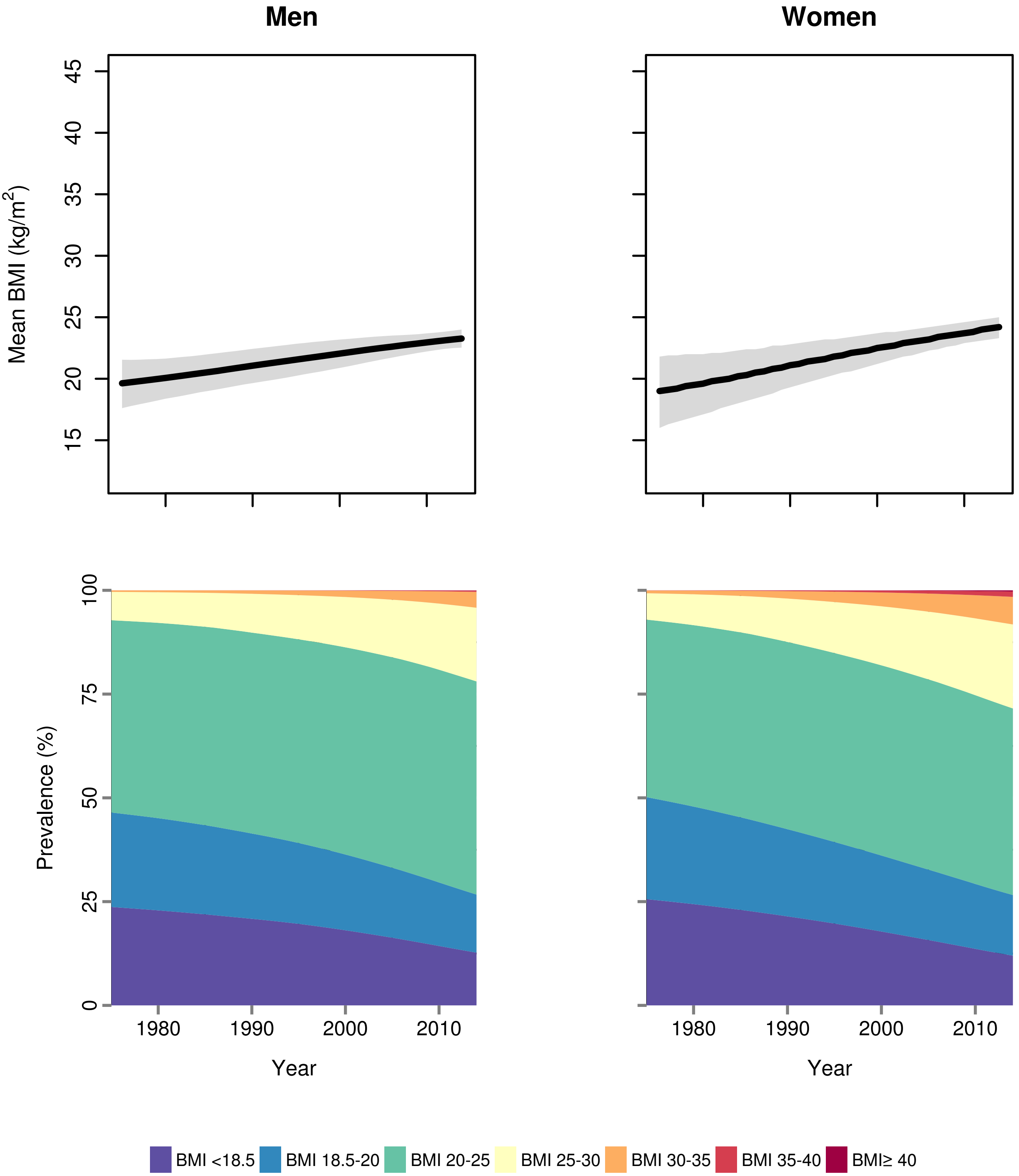

Bolivia

Andean Latin America

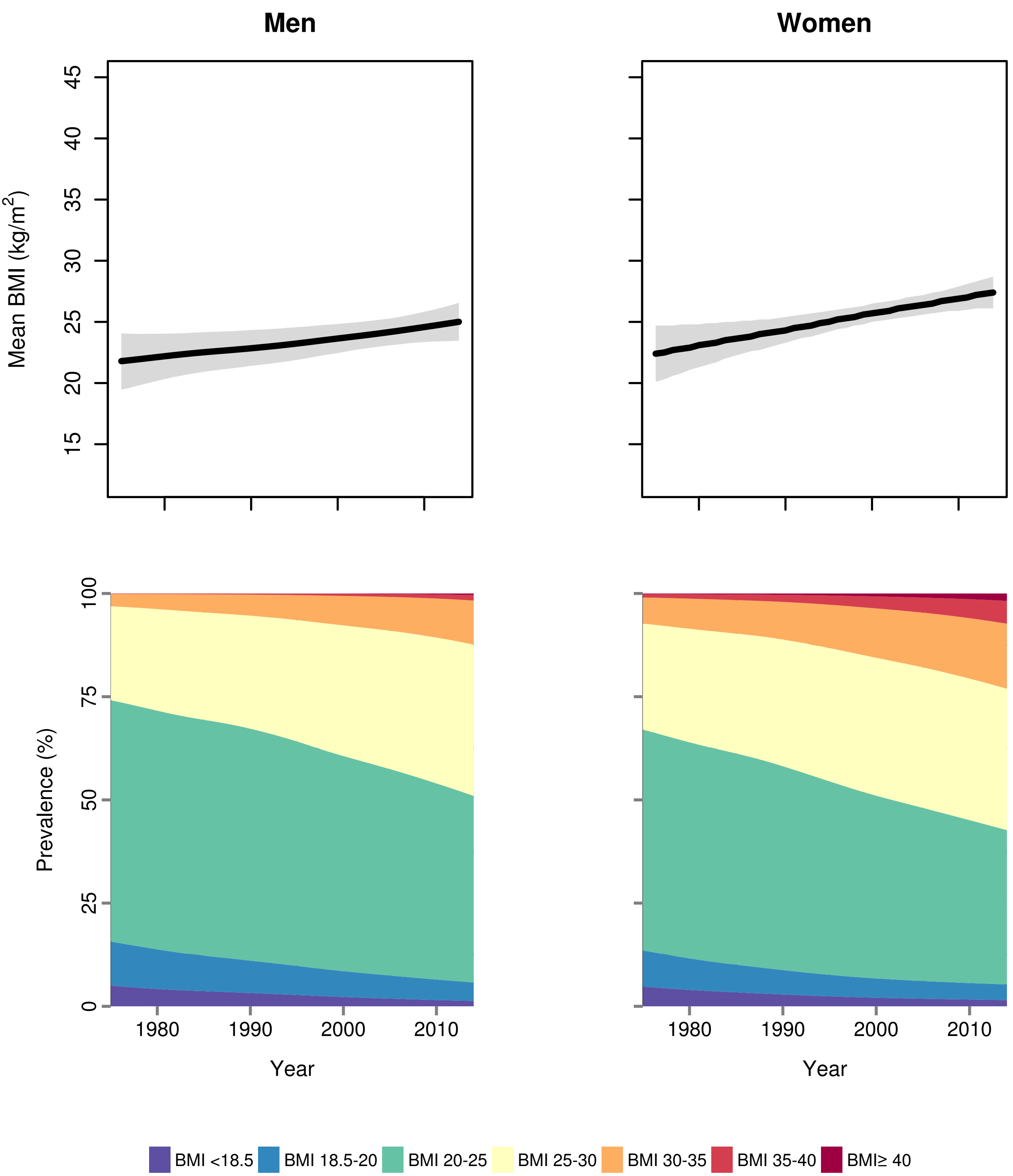

Bosnia and Herzegovina

Central Europe

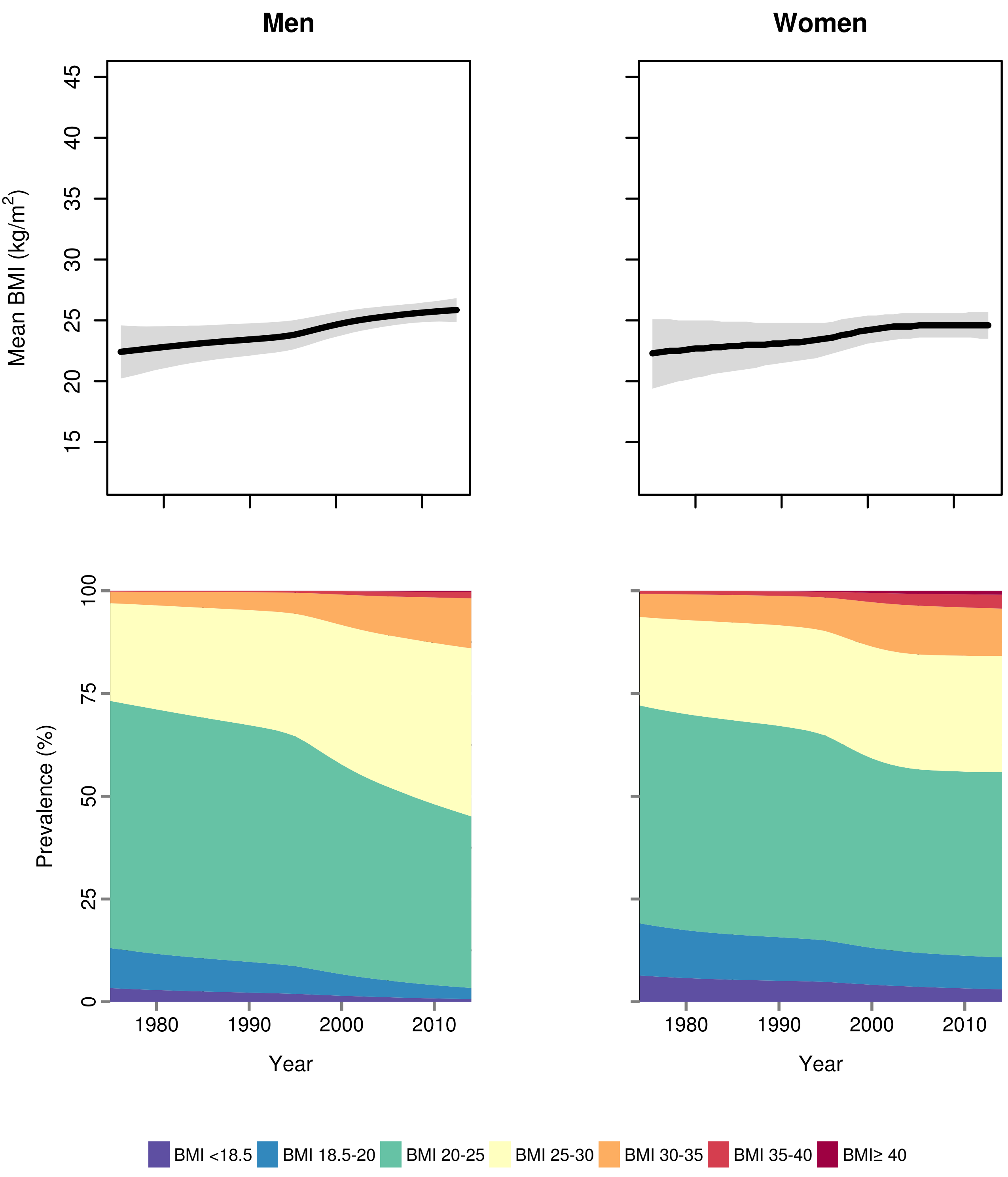

Botswana

Southern Africa

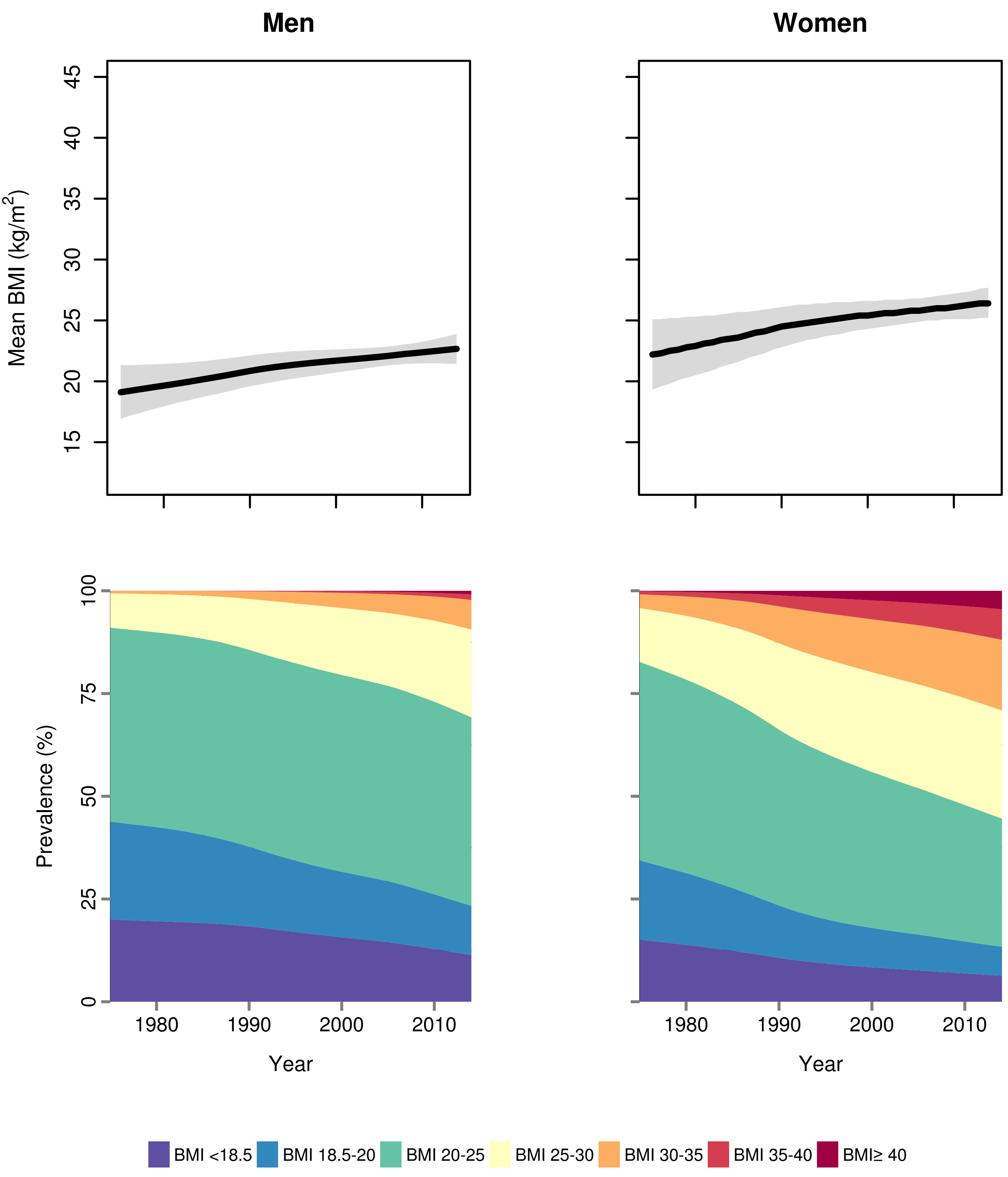

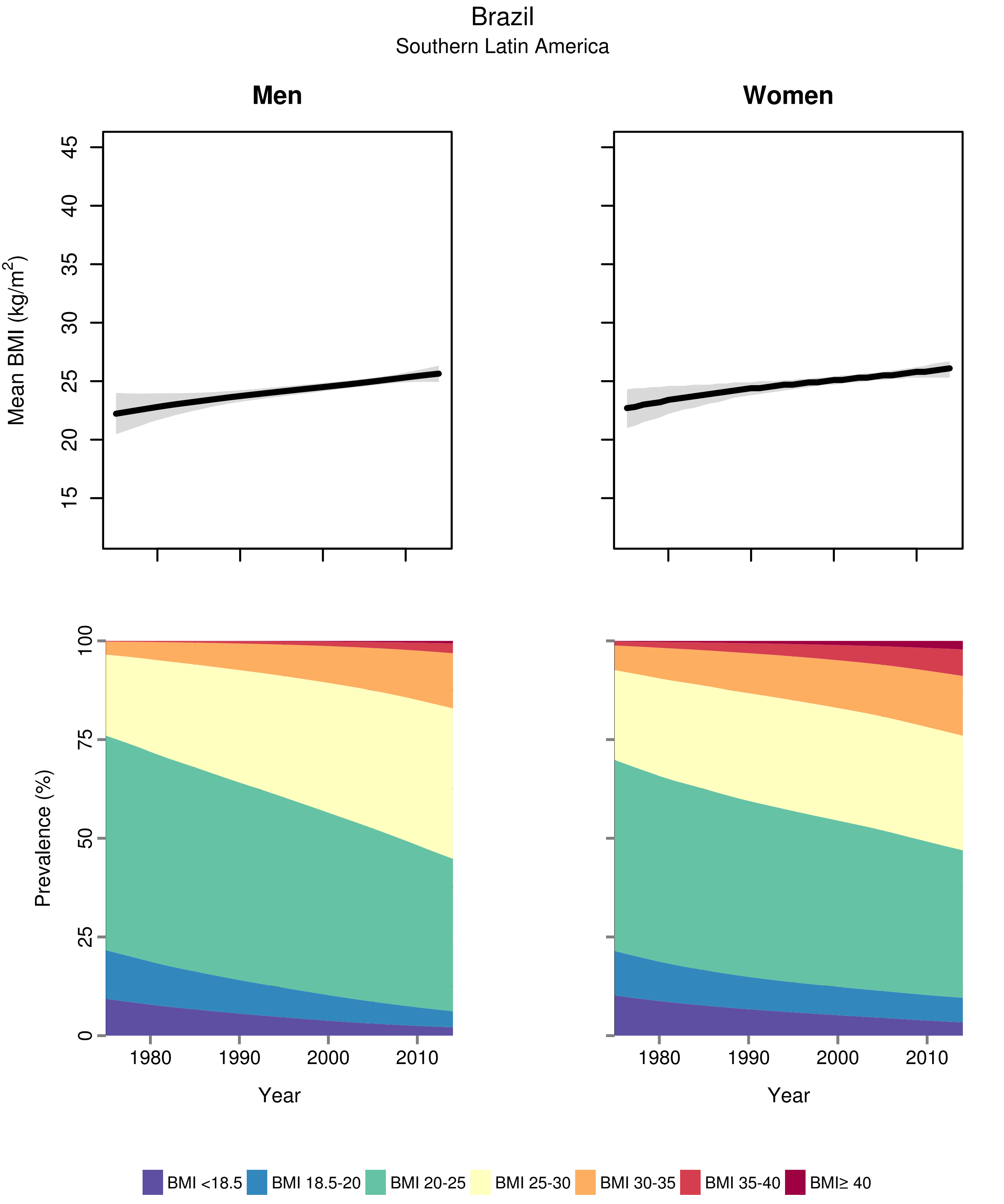

Brazil
Southern Latin America
Men
Women
Mean BMI (kg/m²)
45
40
35
30
25
20
15
Prevalence (%)
100
75
50
25
0
1980
1990
2000
2010
Year
BMI <18.5
BMI 18.5-20
BMI 20-25
BMI 25-30
BMI 30-35
BMI 35-40
BMI≥ 40

Brunei Darussalam

South East Asia

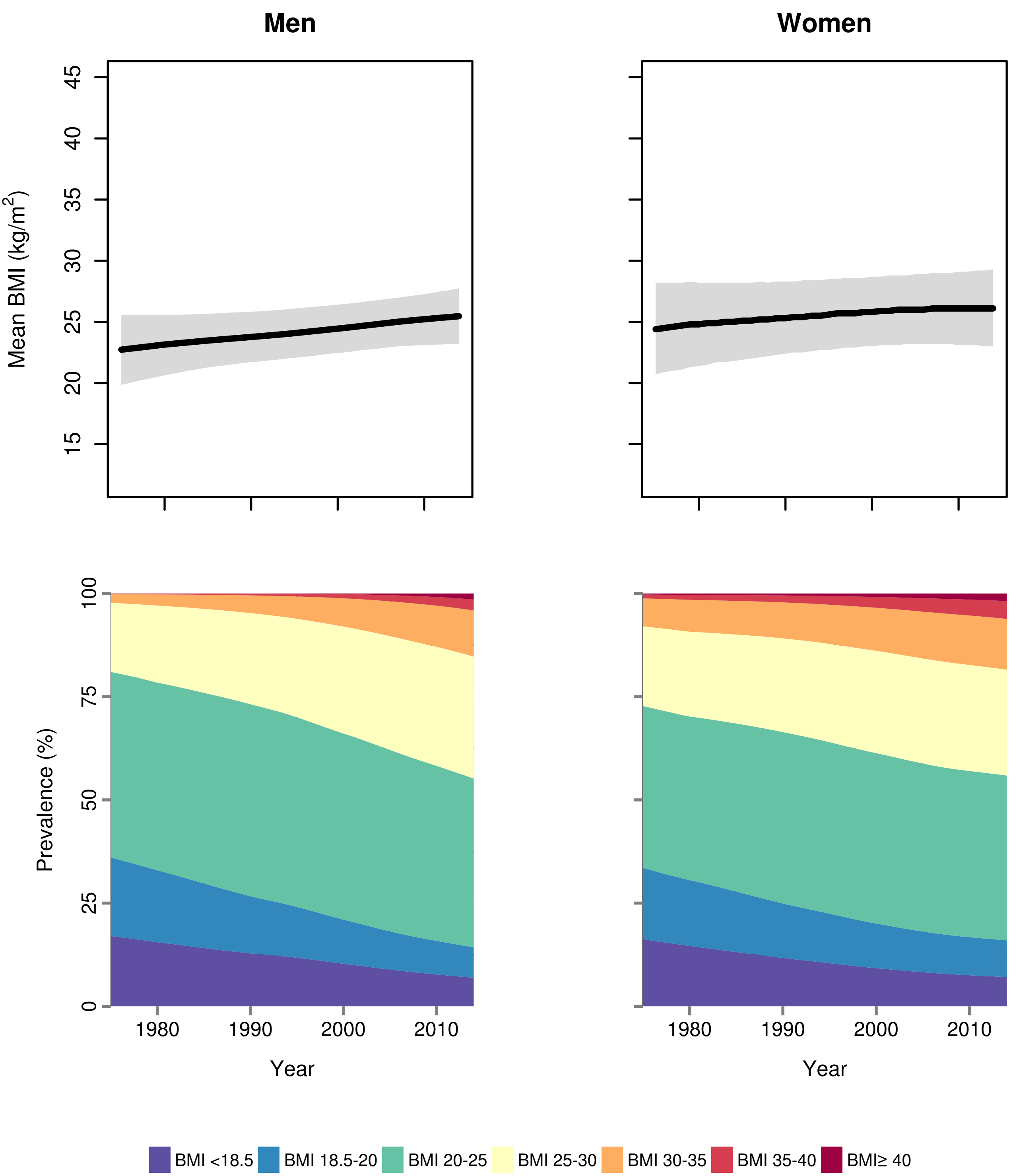

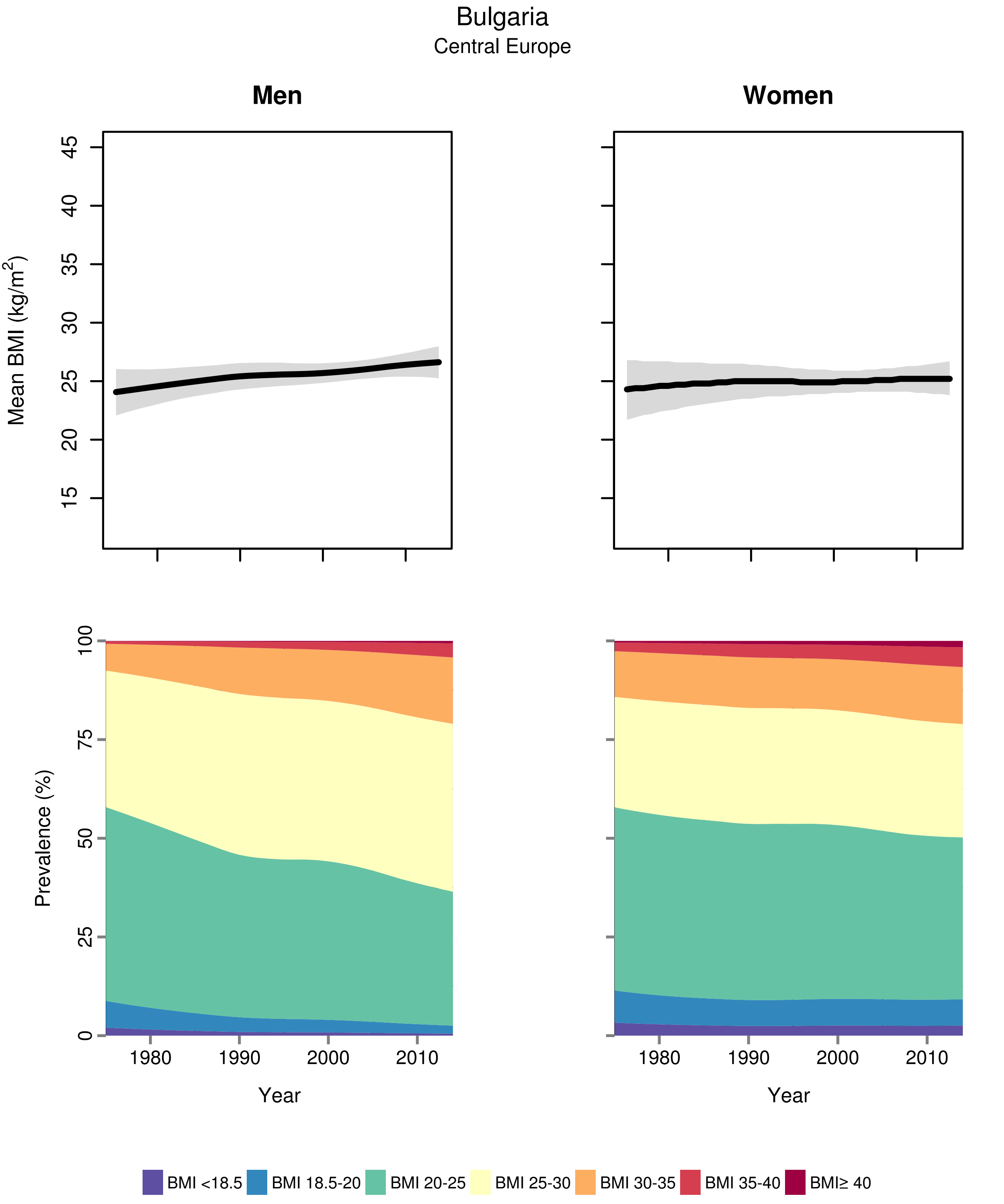

Bulgaria
Central Europe
Men
Women
Mean BMI (kg/m²)
45
40
35
30
25
20
15
Prevalence (%)
100
75
50
25
0
1980
1990
2000
2010
Year
BMI <18.5
BMI 18.5-20
BMI 20-25
BMI 25-30
BMI 30-35
BMI 35-40
BMI≥ 40

Burkina Faso
West Africa

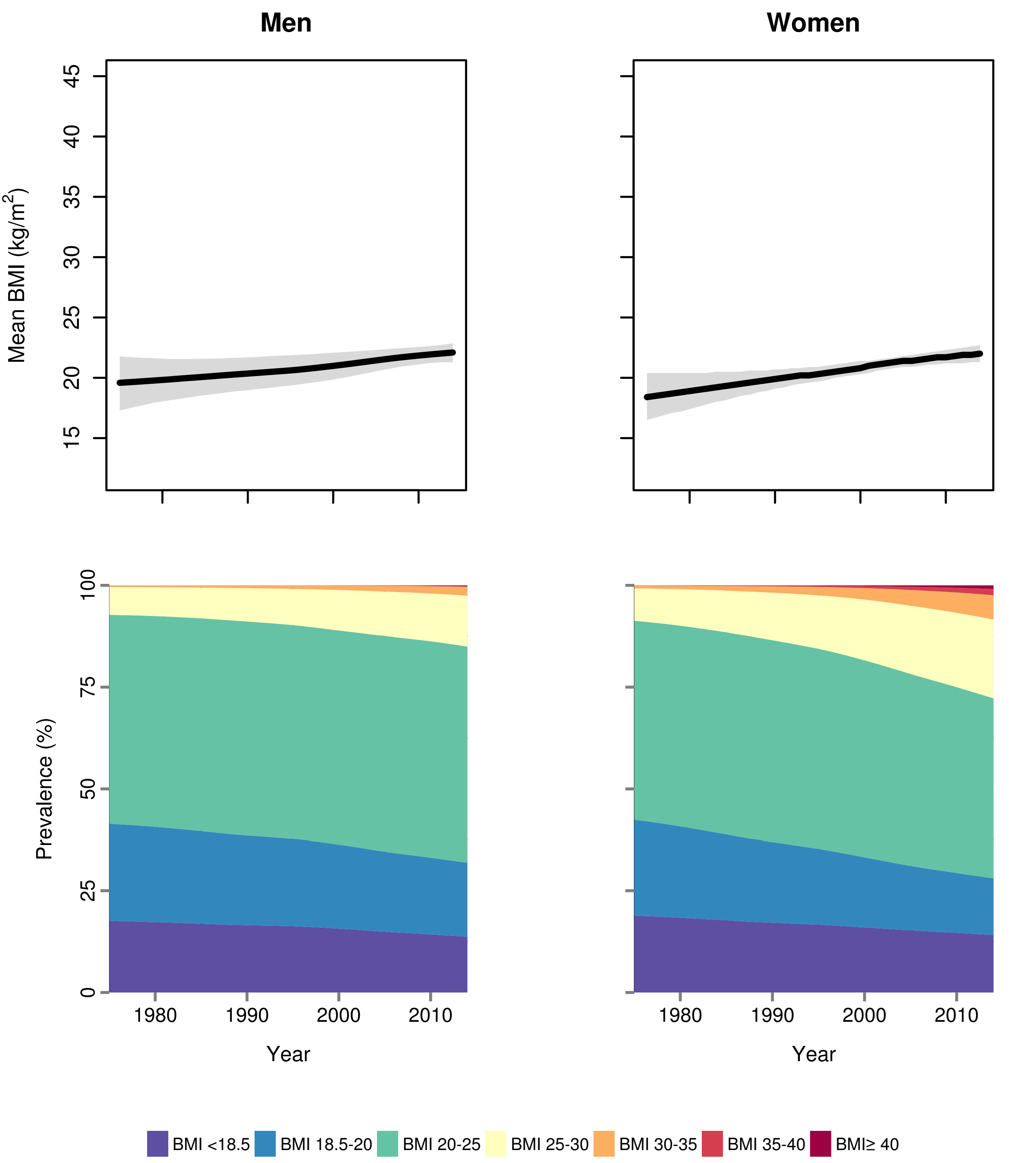

Burundi

East Africa

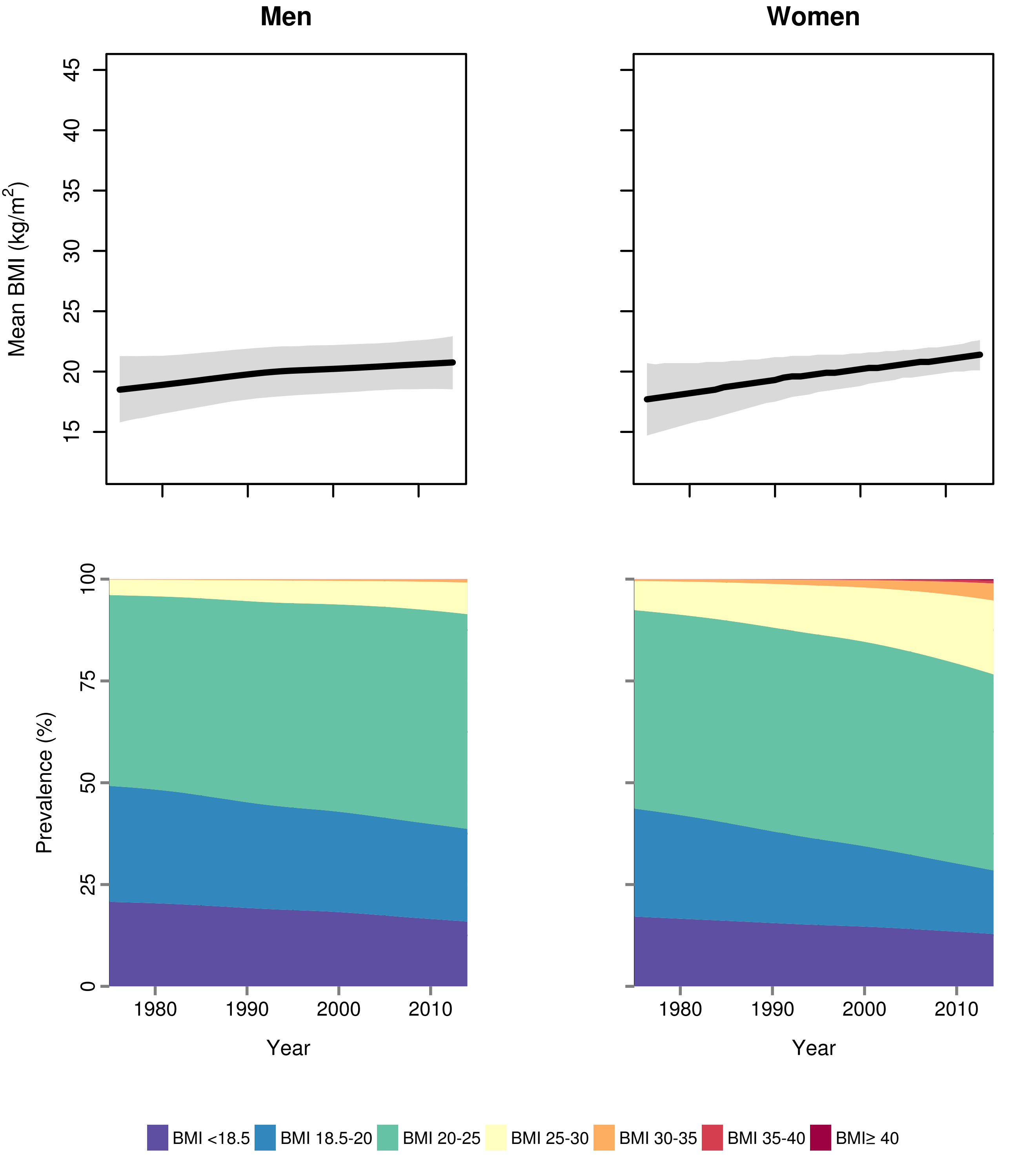

Cabo Verde
West Africa

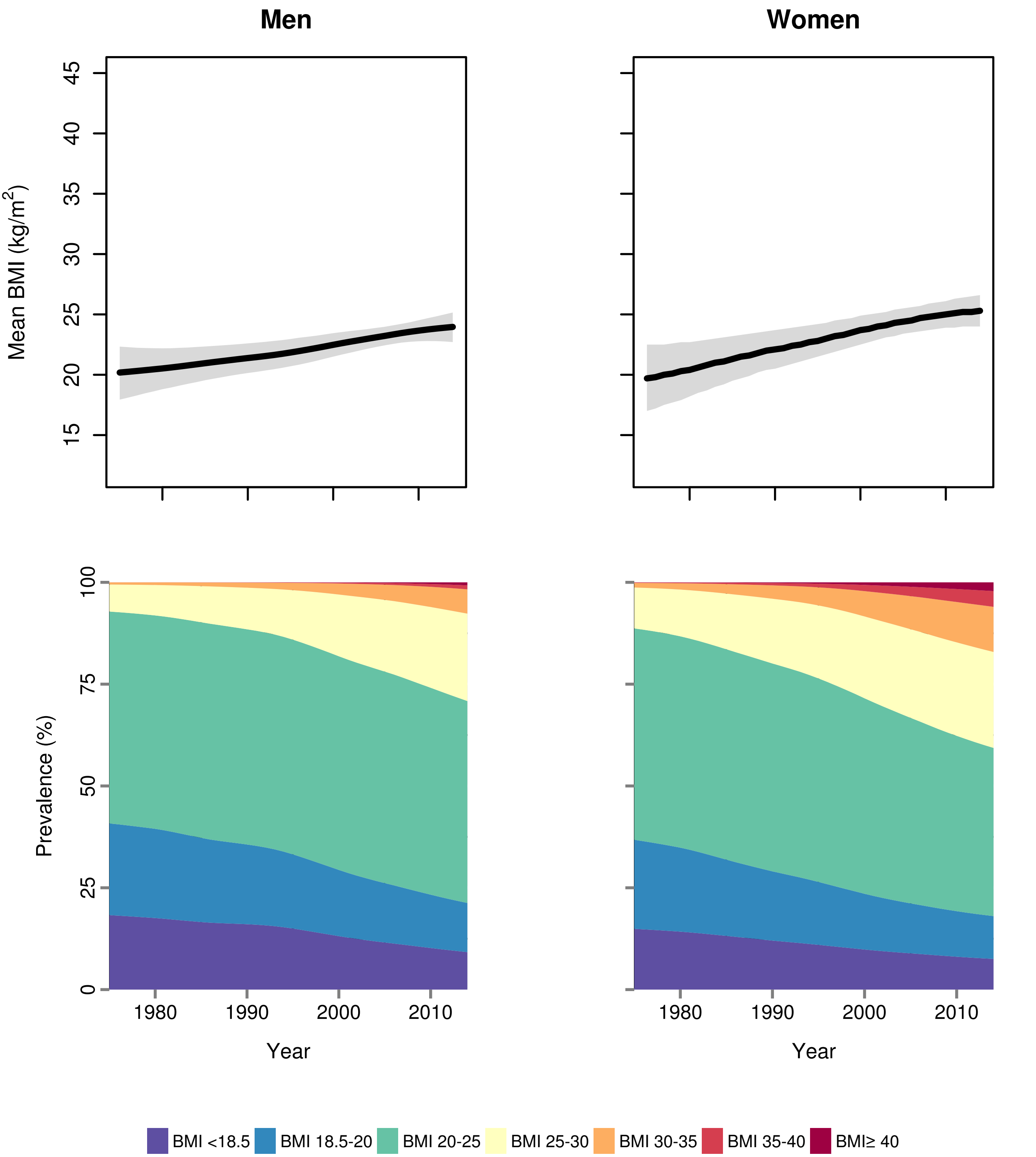

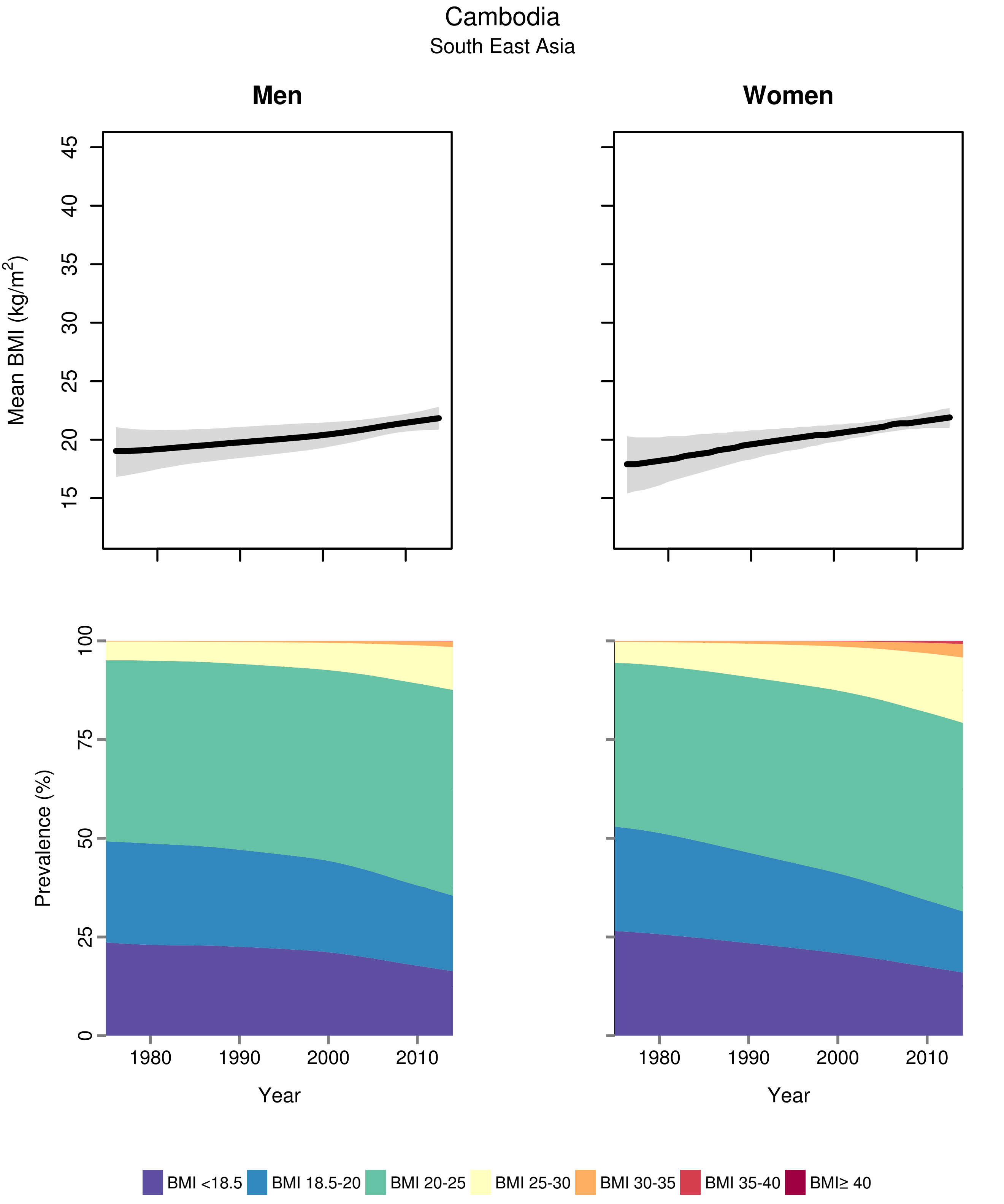

Cambodia
South East Asia
Men
Women
Mean BMI (kg/m²)
45
40
35
30
25
20
15
Prevalence (%)
100
75
50
25
0
1980
1990
2000
2010
Year
BMI <18.5
BMI 18.5-20
BMI 20-25
BMI 25-30
BMI 30-35
BMI 35-40
BMI≥ 40

Cameroon
West Africa

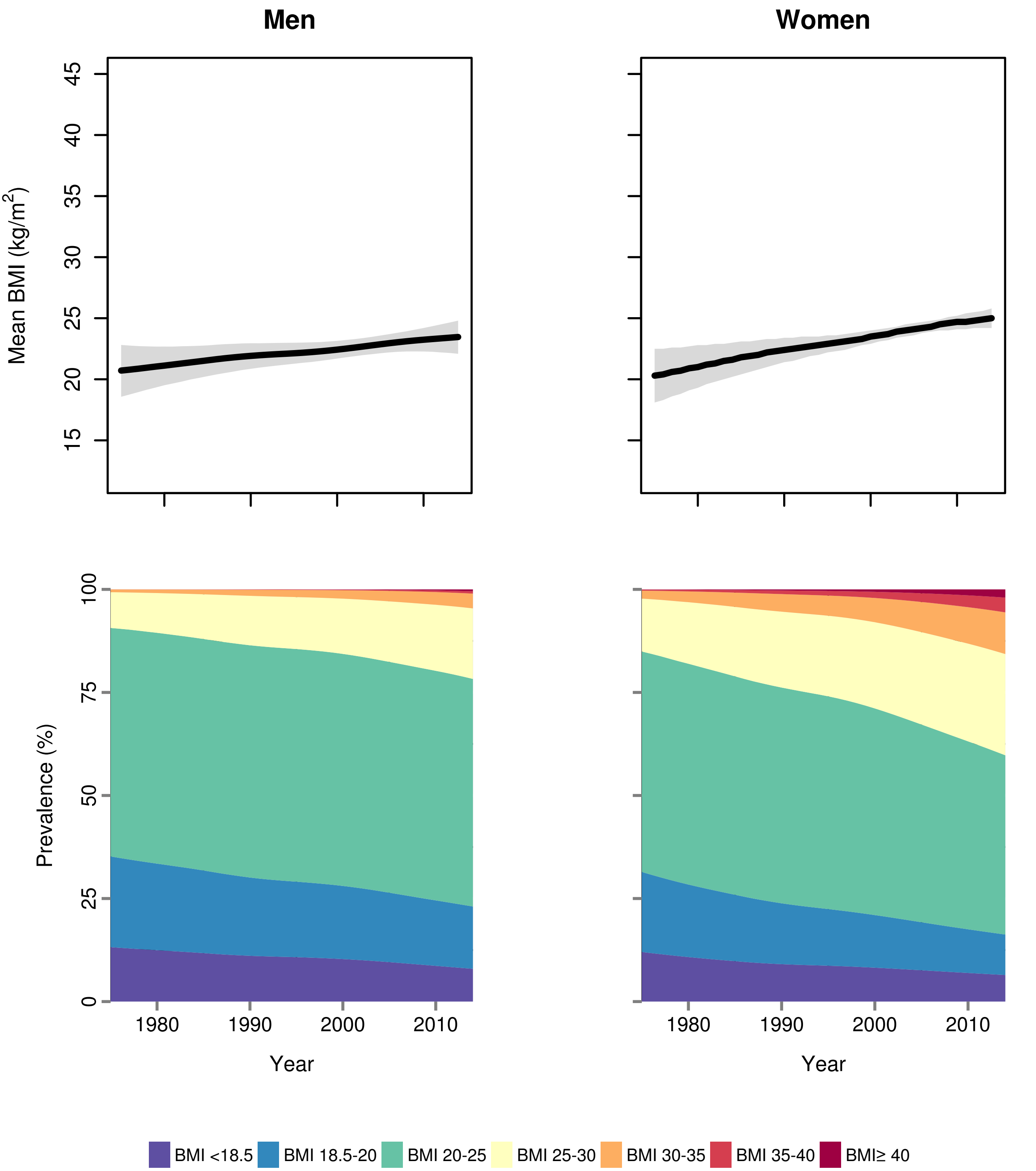

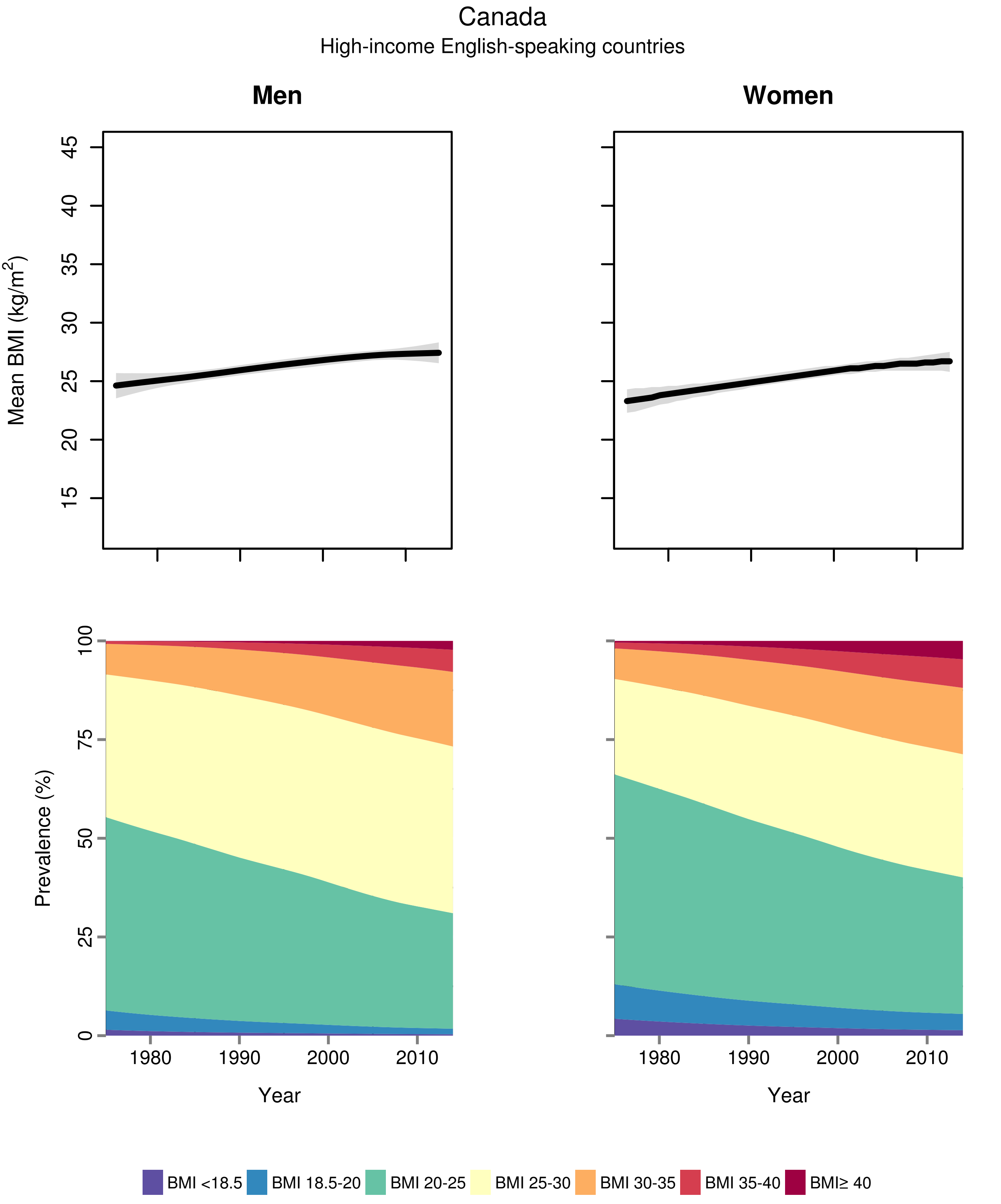
Canada
High-income English-speaking countries
Men
Women
Mean BMI (kg/m2)
45
40
35
30
25
20
15
Prevalence (%)
100
75
50
25
0
1980
1990
2000
2010
Year
BMI <18.5
BMI 18.5-20
BMI 20-25
BMI 25-30
BMI 30-35
BMI 35-40
BMI≥ 40

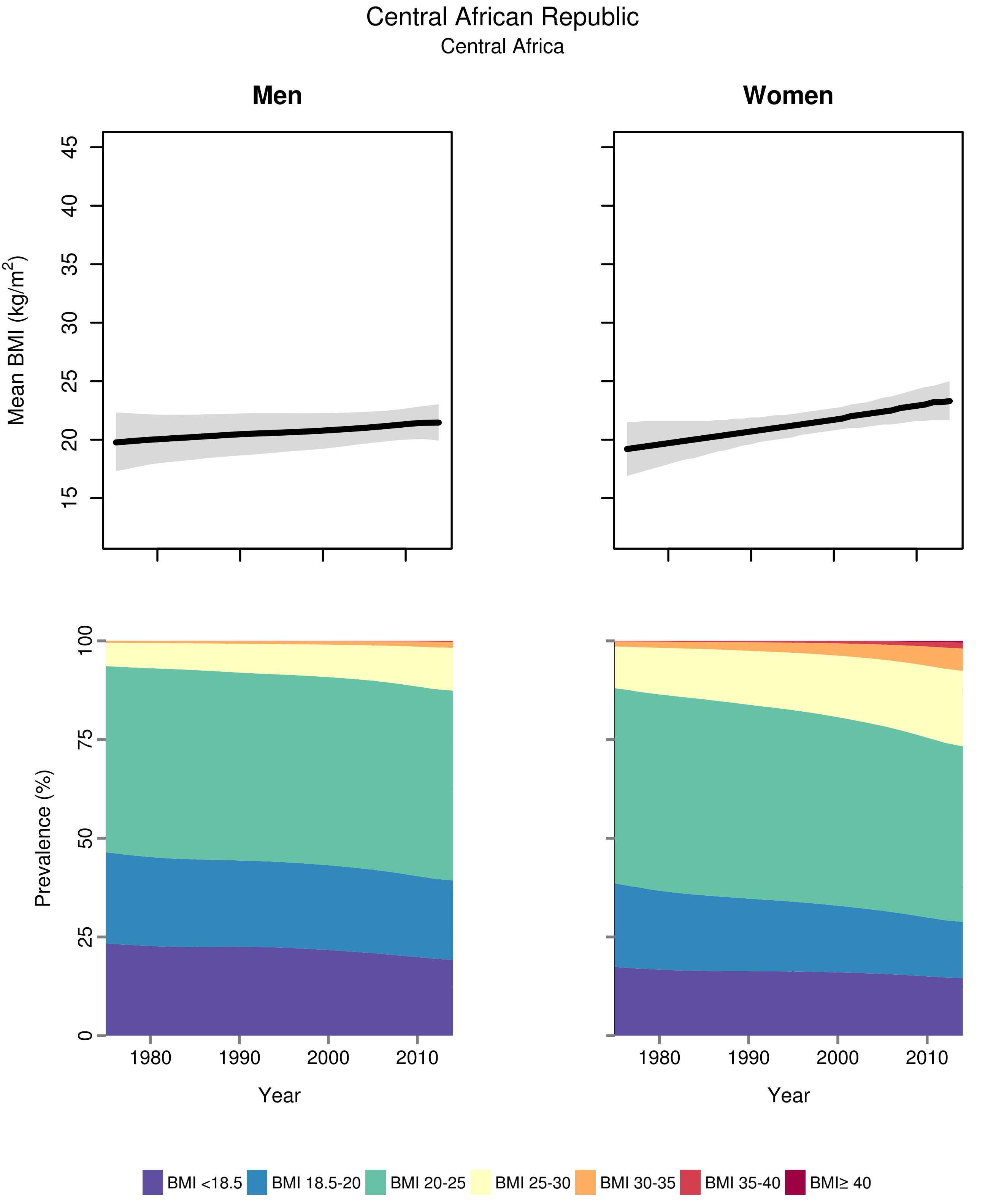
Central African Republic
Central Africa
Men
Women
Mean BMI (kg/m2)
45
40
35
30
25
20
15
Prevalence (%)
100
75
50
25
0
1980
1990
2000
2010
Year
BMI <18.5
BMI 18.5-20
BMI 20-25
BMI 25-30
BMI 30-35
BMI 35-40
BMI≥ 40

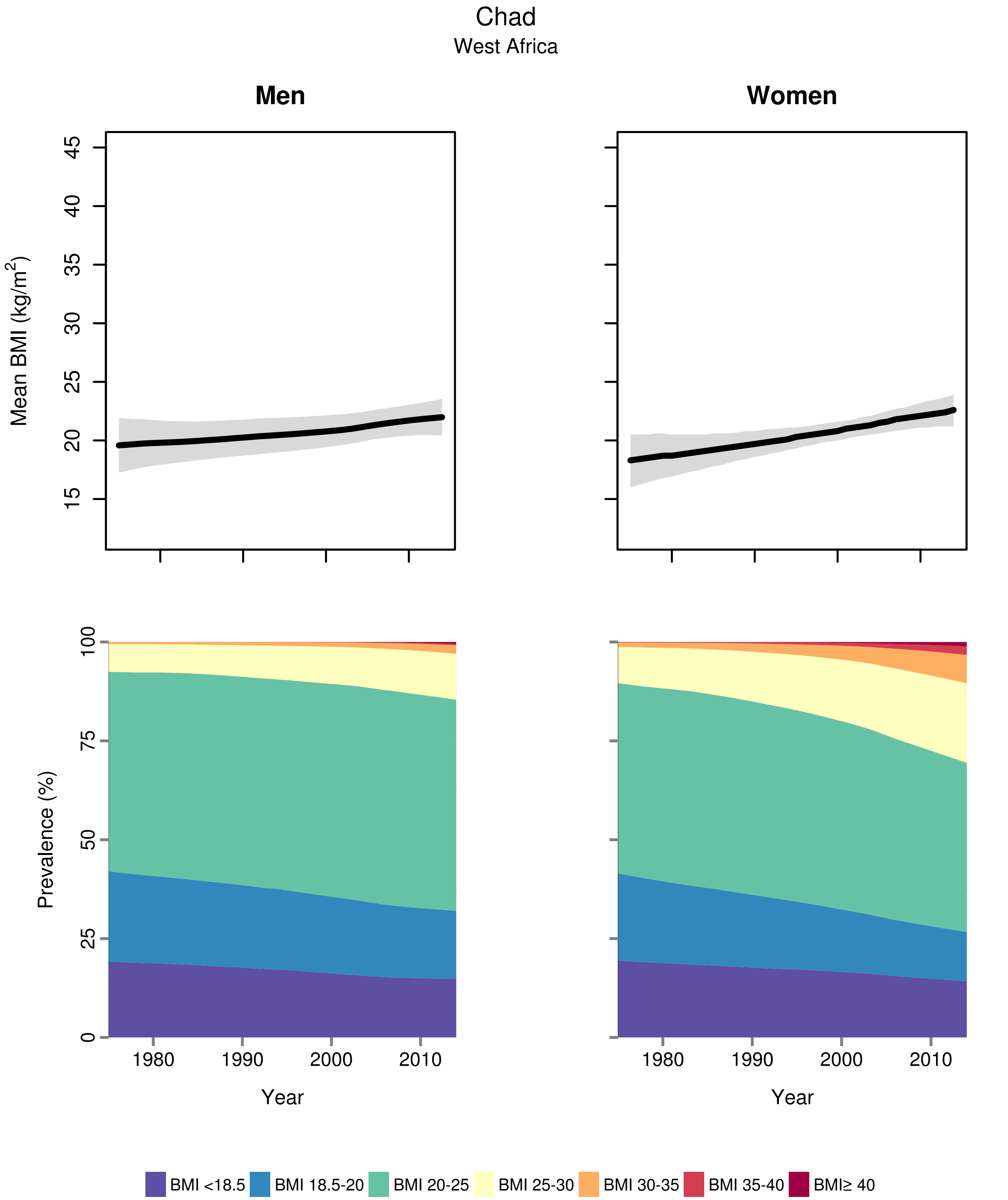

Chad
West Africa
Men
Women
Mean BMI (kg/m2)
45
40
35
30
25
20
15
Prevalence (%)
100
75
50
25
0
1980
1990
2000
2010
Year
BMI <18.5
BMI 18.5-20
BMI 20-25
BMI 25-30
BMI 30-35
BMI 35-40
BMI≥ 40

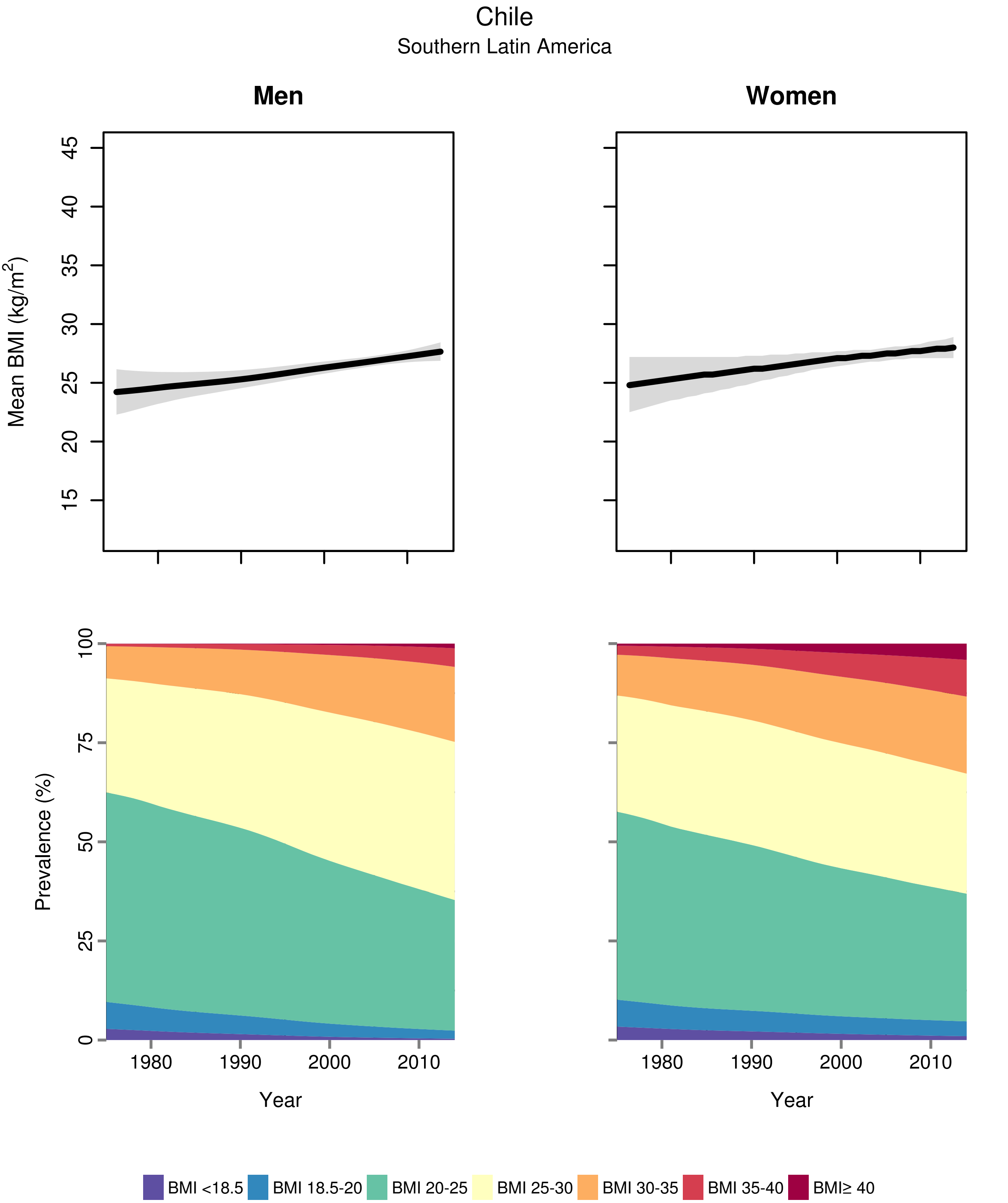

Chile
Southern Latin America
Men
Women
Mean BMI (kg/m2)
45
40
35
30
25
20
15
Prevalence (%)
100
75
50
25
0
1980
1990
2000
2010
Year
BMI <18.5
BMI 18.5-20
BMI 20-25
BMI 25-30
BMI 30-35
BMI 35-40
BMI≥ 40

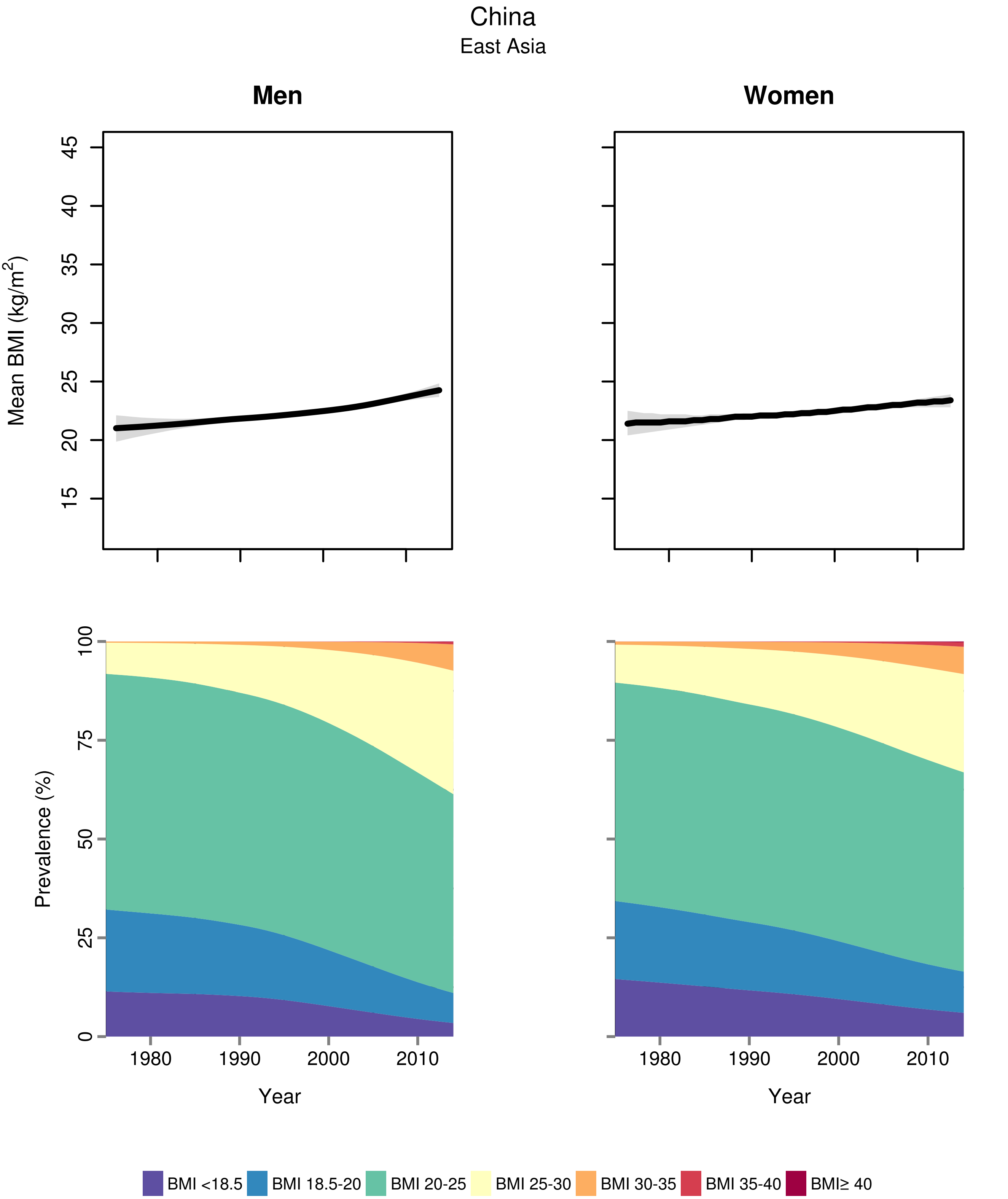
China
East Asia
Men
Women
Mean BMI (kg/m2)
45
40
35
30
25
20
15
Prevalence (%)
100
75
50
25
0
1980
1990
2000
2010
Year
BMI <18.5
BMI 18.5-20
BMI 20-25
BMI 25-30
BMI 30-35
BMI 35-40
BMI≥ 40

China (Hong Kong SAR)

East Asia

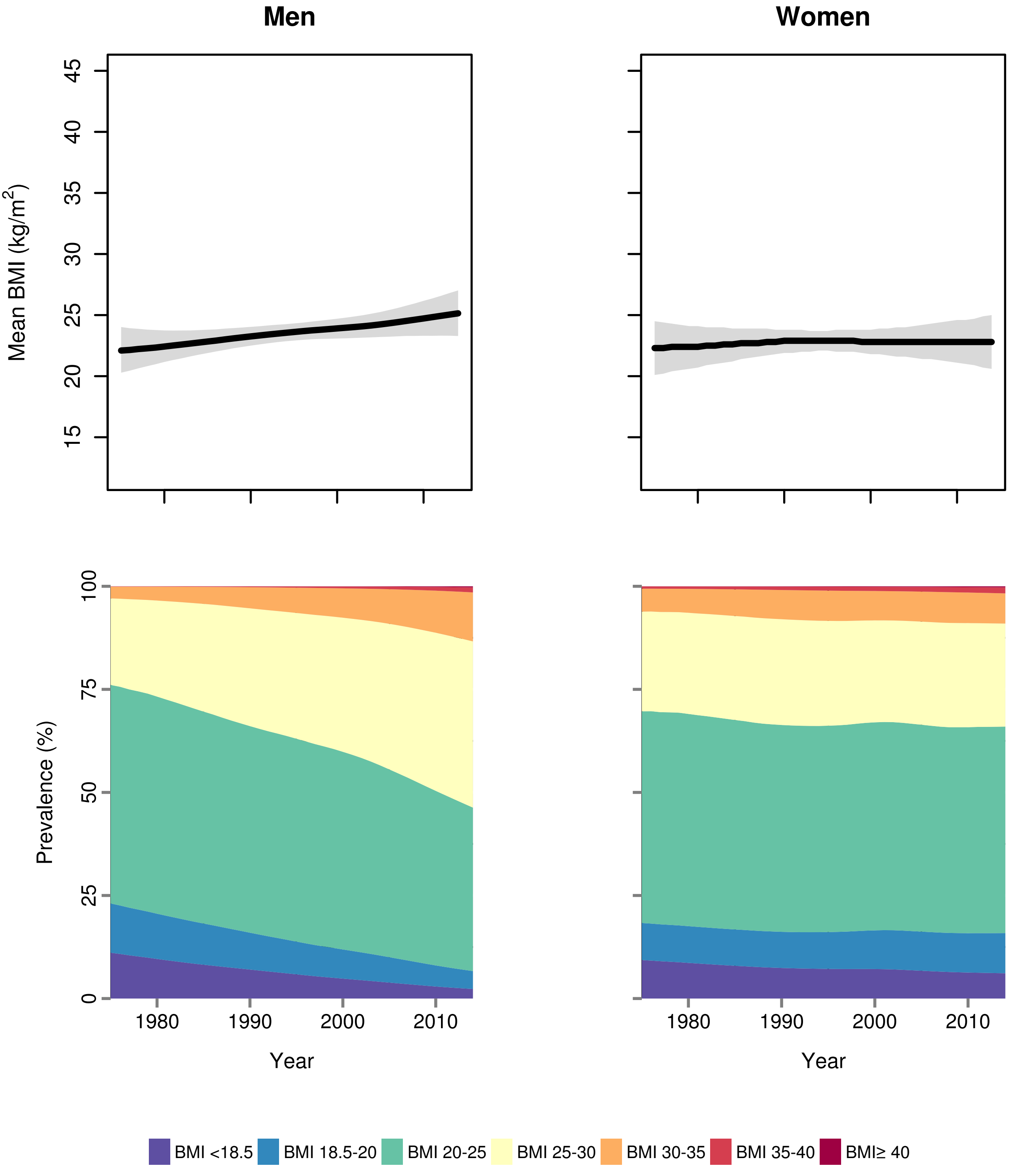

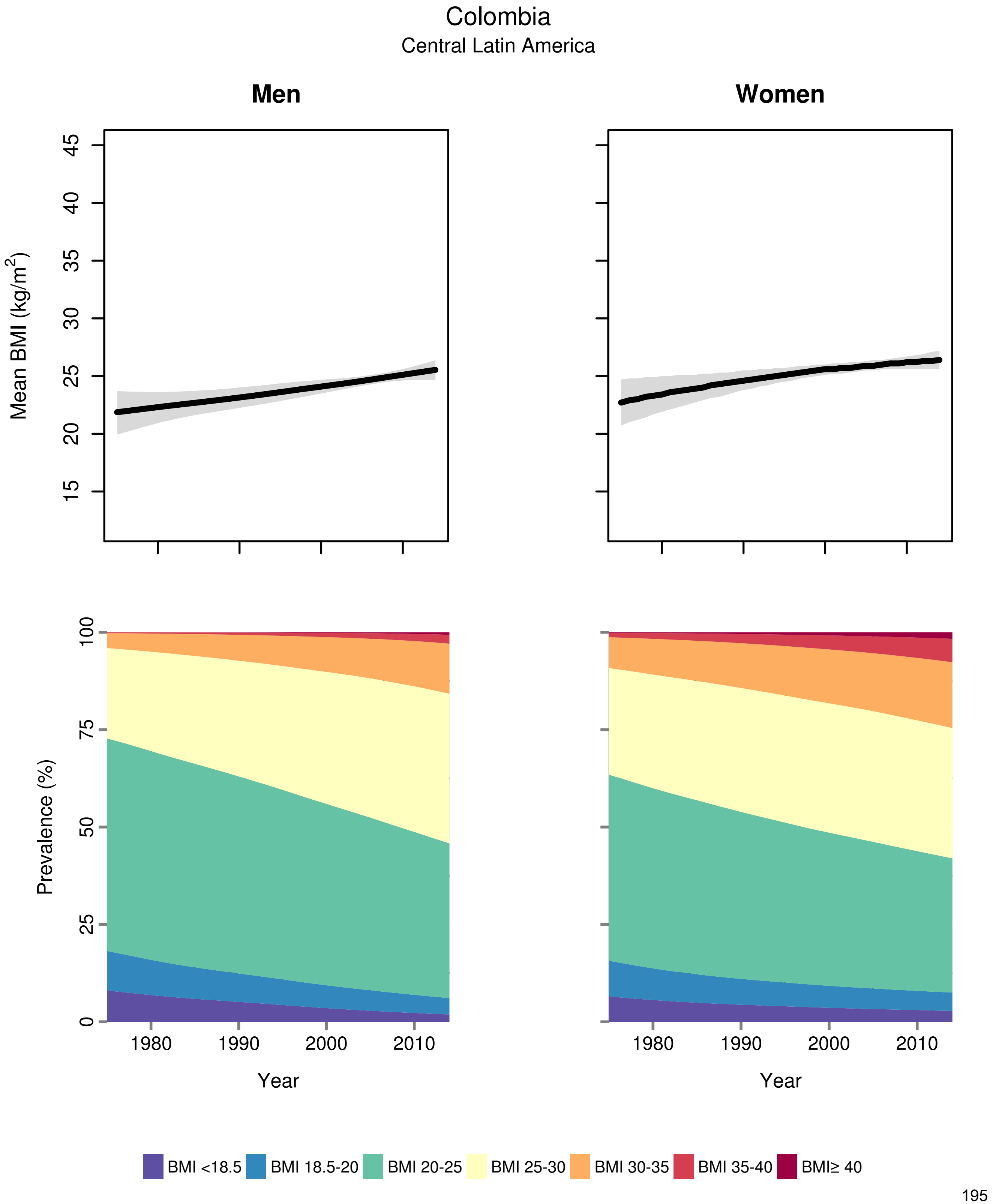

Colombia
Central Latin America
Men
Women
Mean BMI (kg/m2)
45
40
35
30
25
20
15
Prevalence (%)
100
75
50
25
0
1980
1990
2000
2010
Year
BMI <18.5
BMI 18.5-20
BMI 20-25
BMI 25-30
BMI 30-35
BMI 35-40
BMI≥ 40

Comoros

East Africa

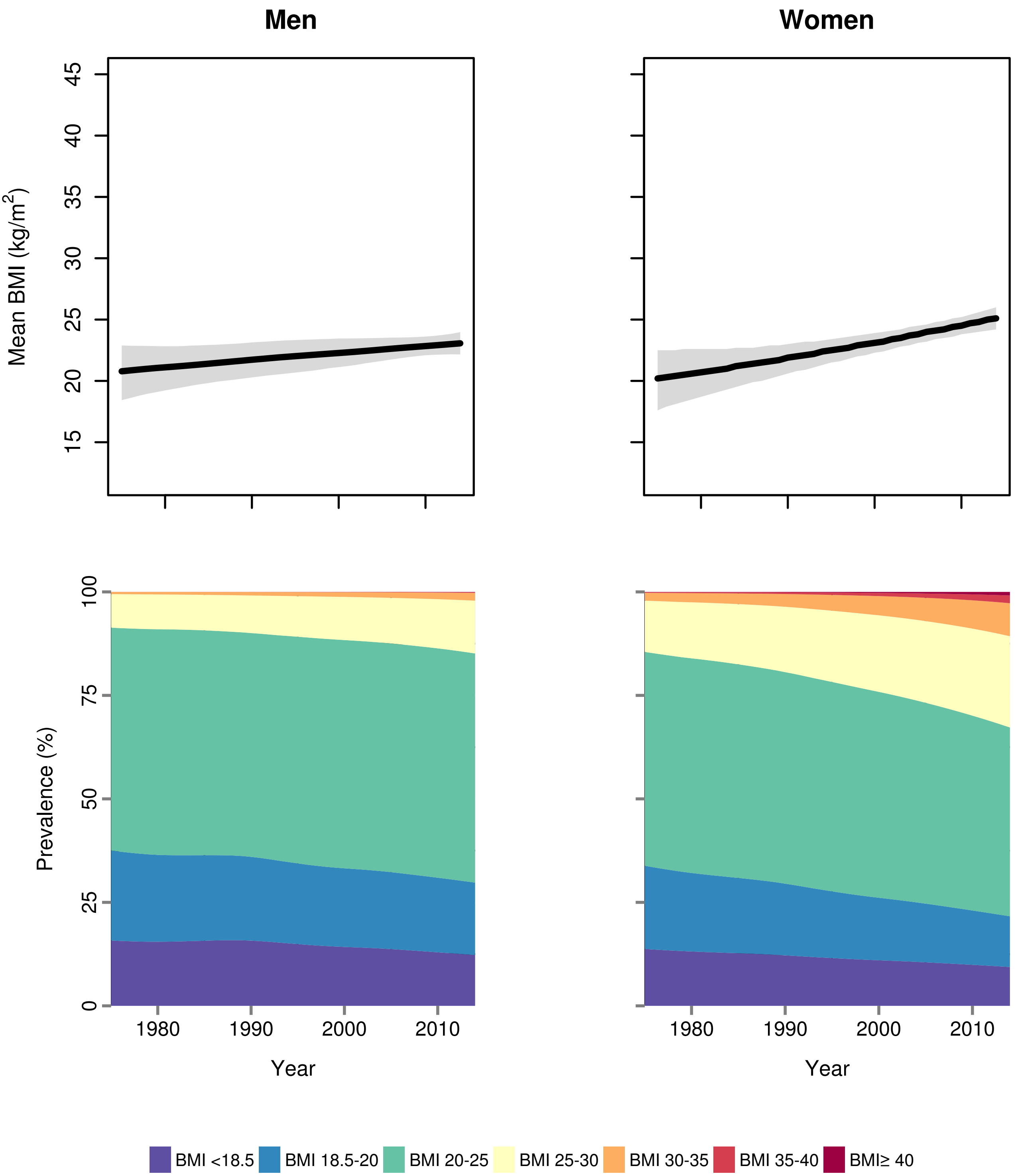

Congo
Central Africa

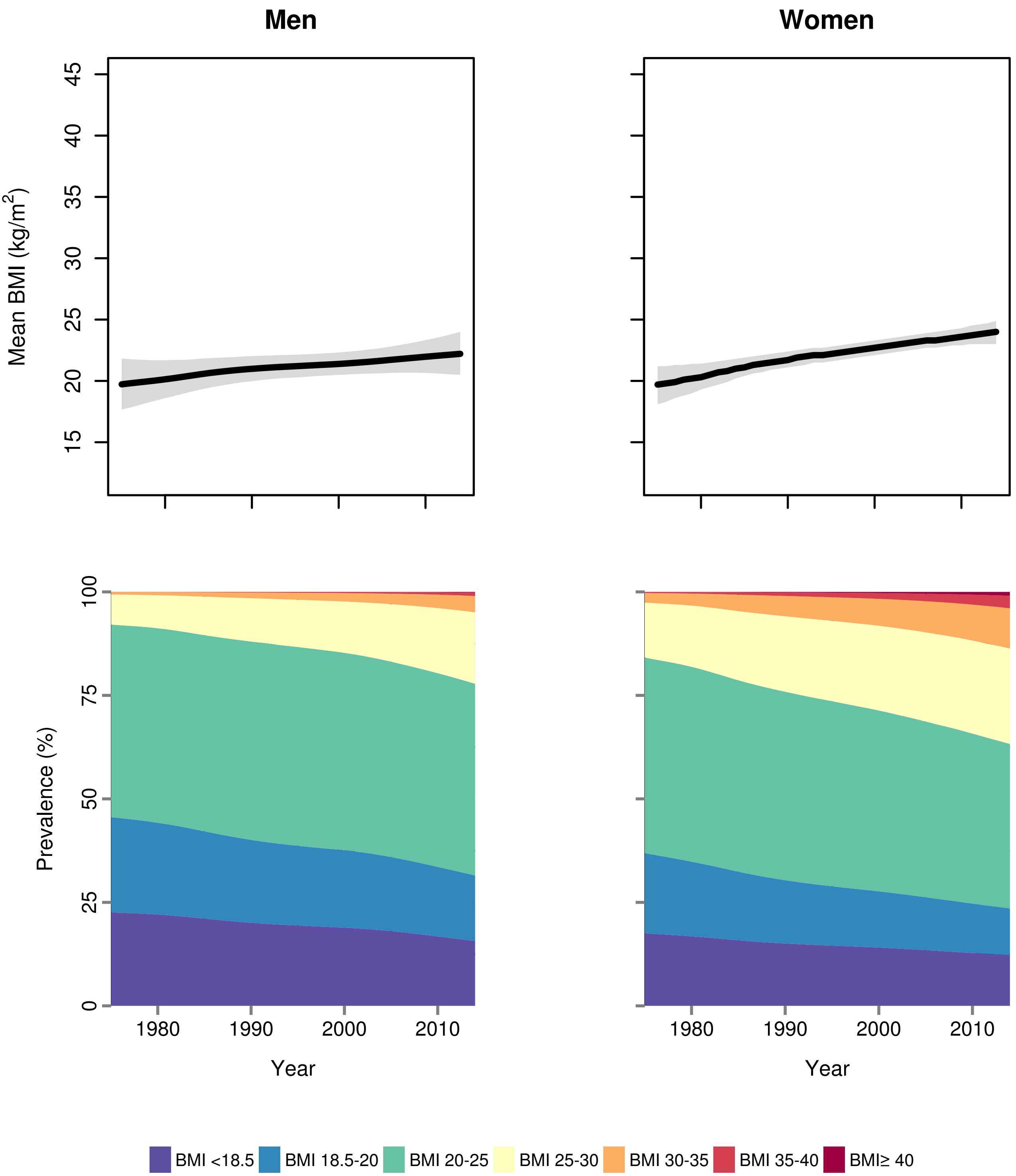

Cook Islands

Polynesia and Micronesia

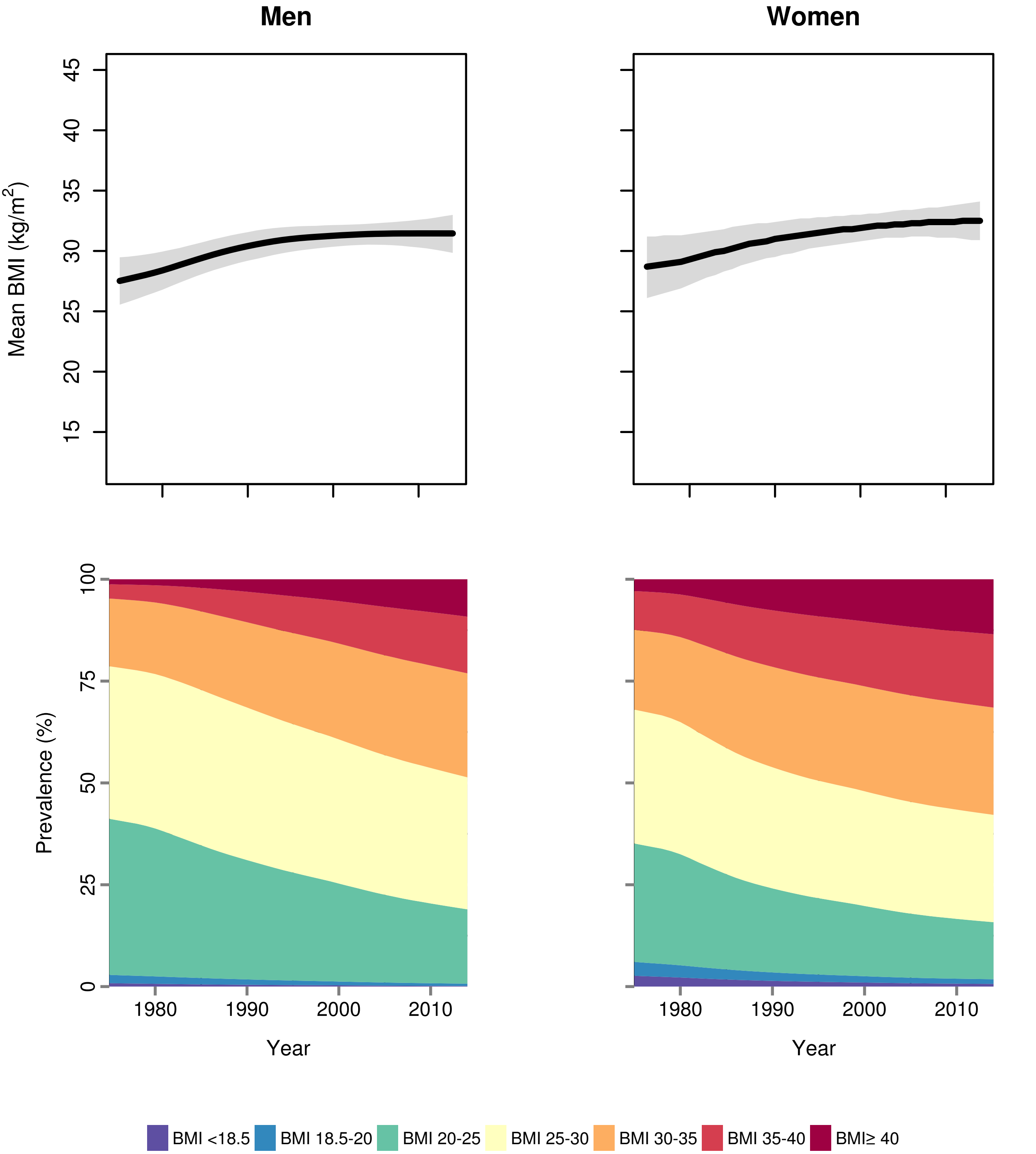

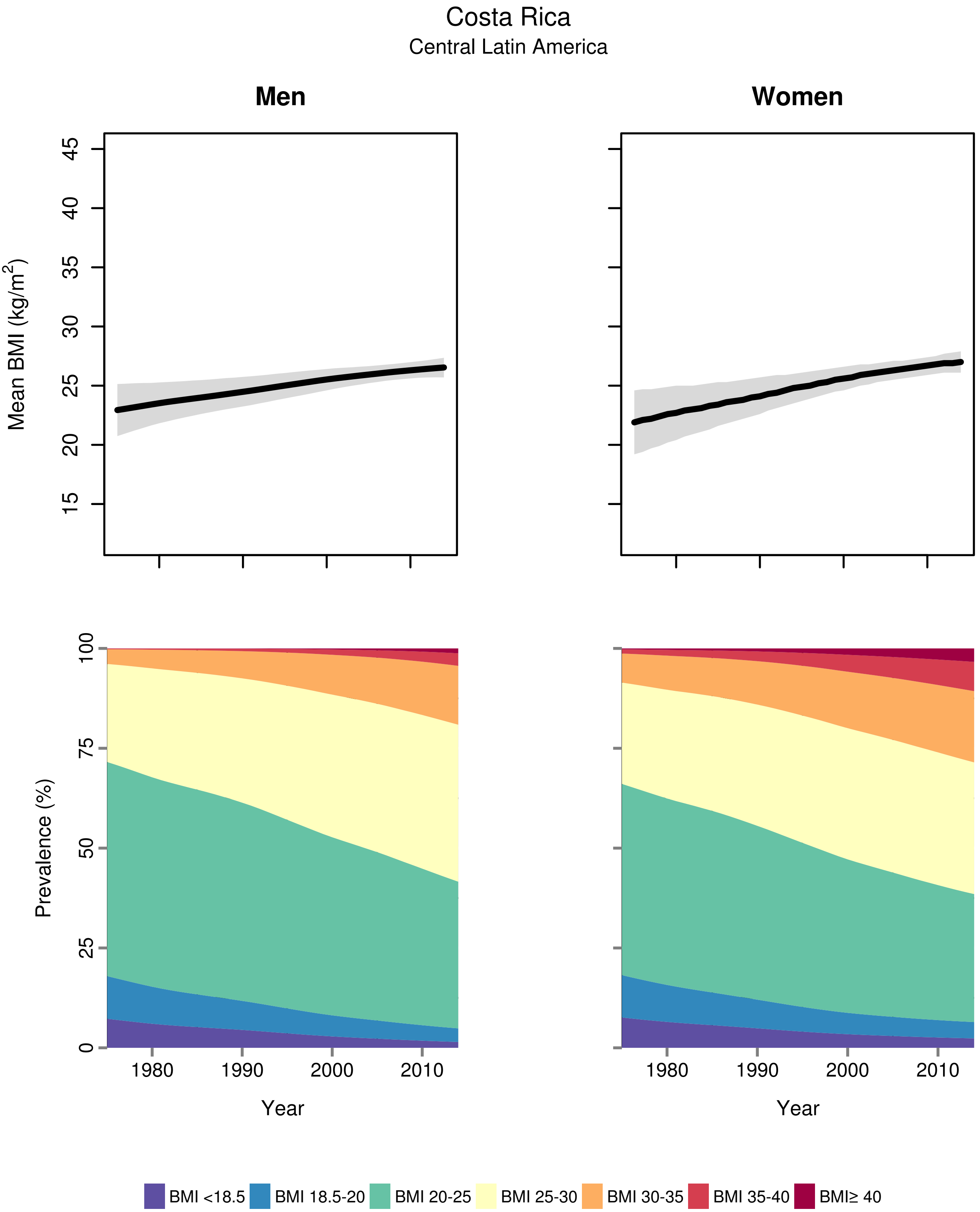

Costa Rica
Central Latin America
Men
Women
Mean BMI (kg/m2)
45
40
35
30
25
20
15
Prevalence (%)
100
75
50
25
0
1980
1990
2000
2010
Year
BMI <18.5
BMI 18.5-20
BMI 20-25
BMI 25-30
BMI 30-35
BMI 35-40
BMI≥ 40

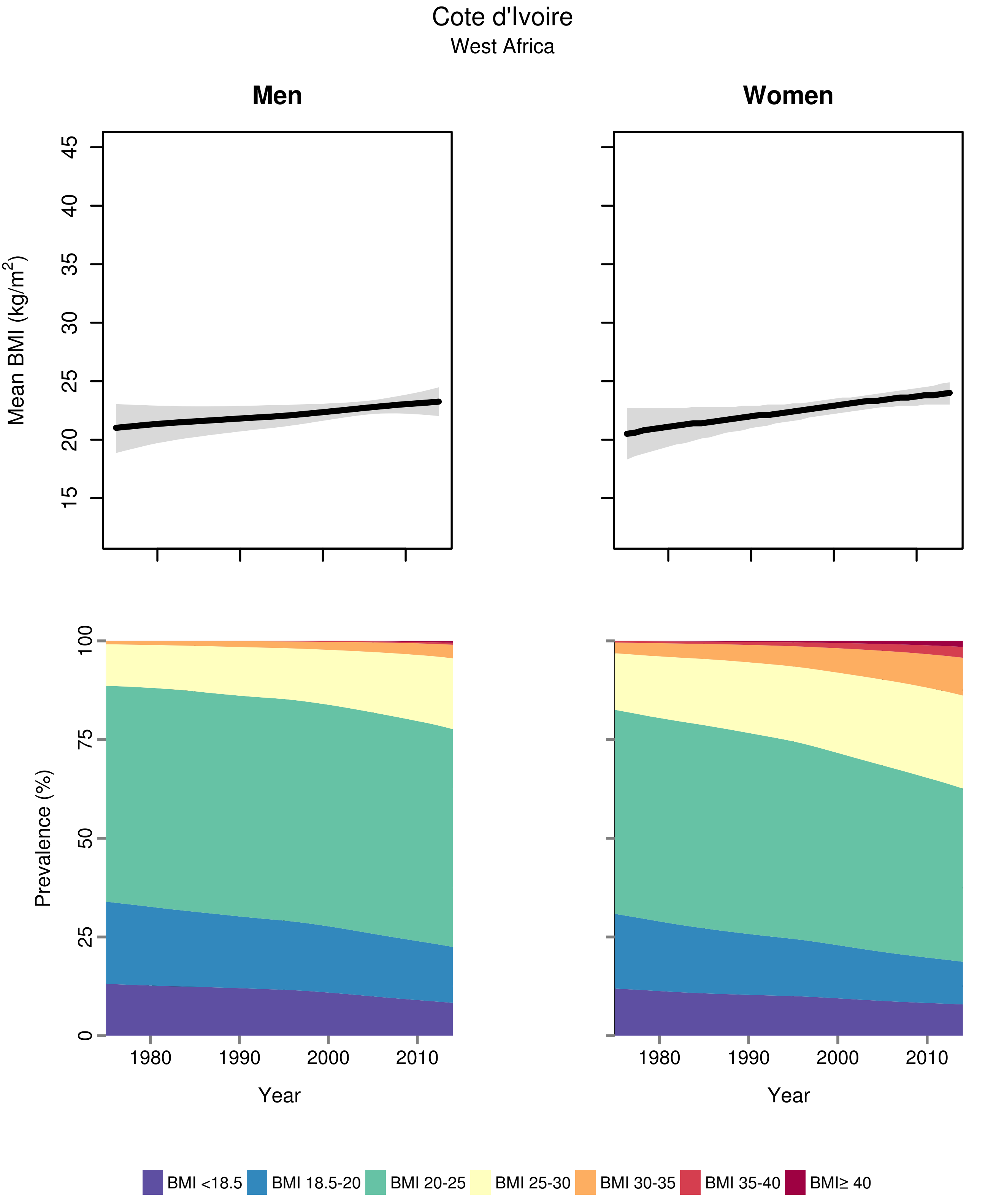

Cote d'Ivoire
West Africa
Men
Women
Mean BMI (kg/m2)
45
40
35
30
25
20
15
100
75
50
25
0
Prevalence (%)
1980
1990
2000
2010
Year
BMI <18.5
BMI 18.5-20
BMI 20-25
BMI 25-30
BMI 30-35
BMI 35-40
BMI≥ 40

Croatia
Central Europe

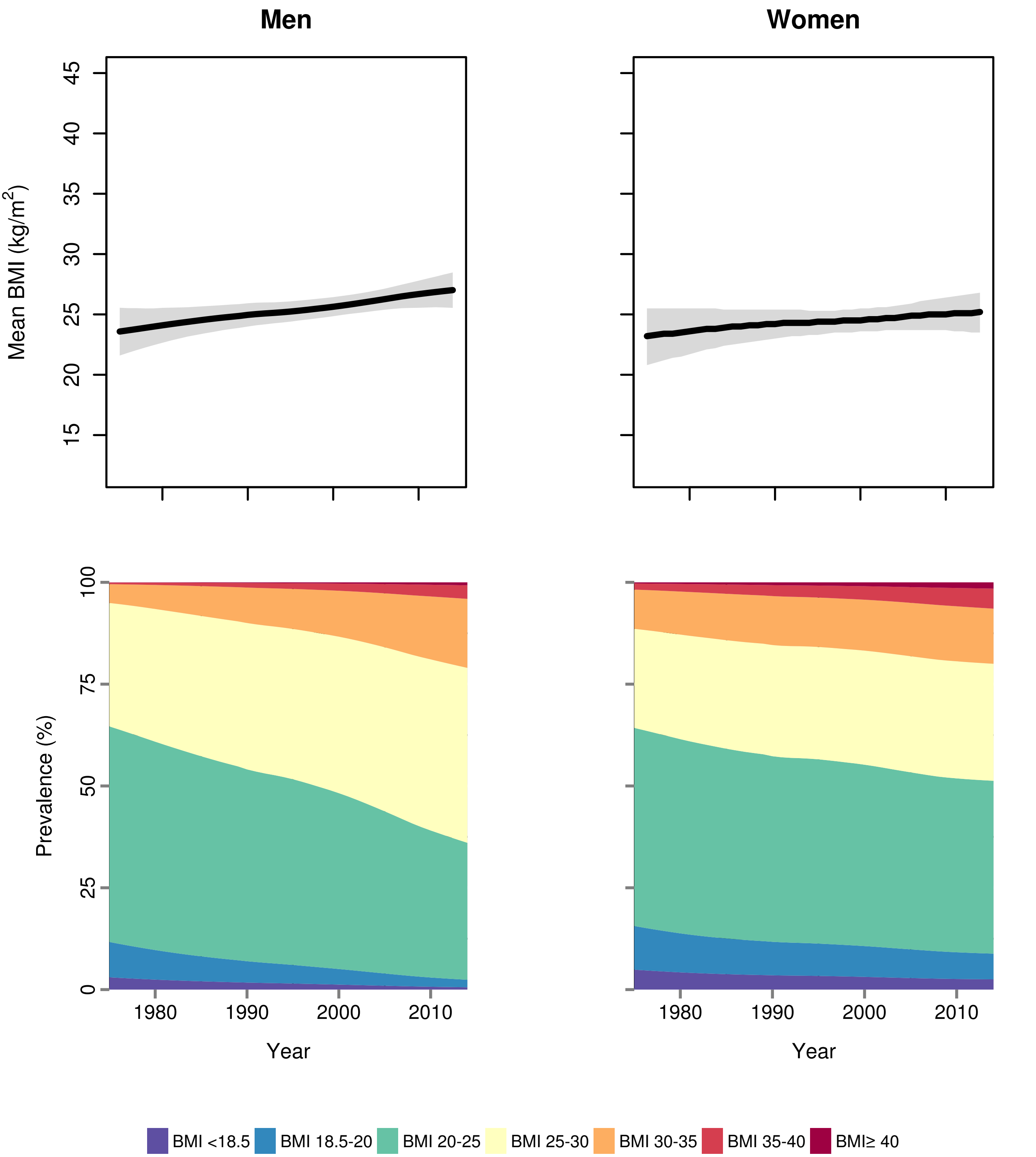

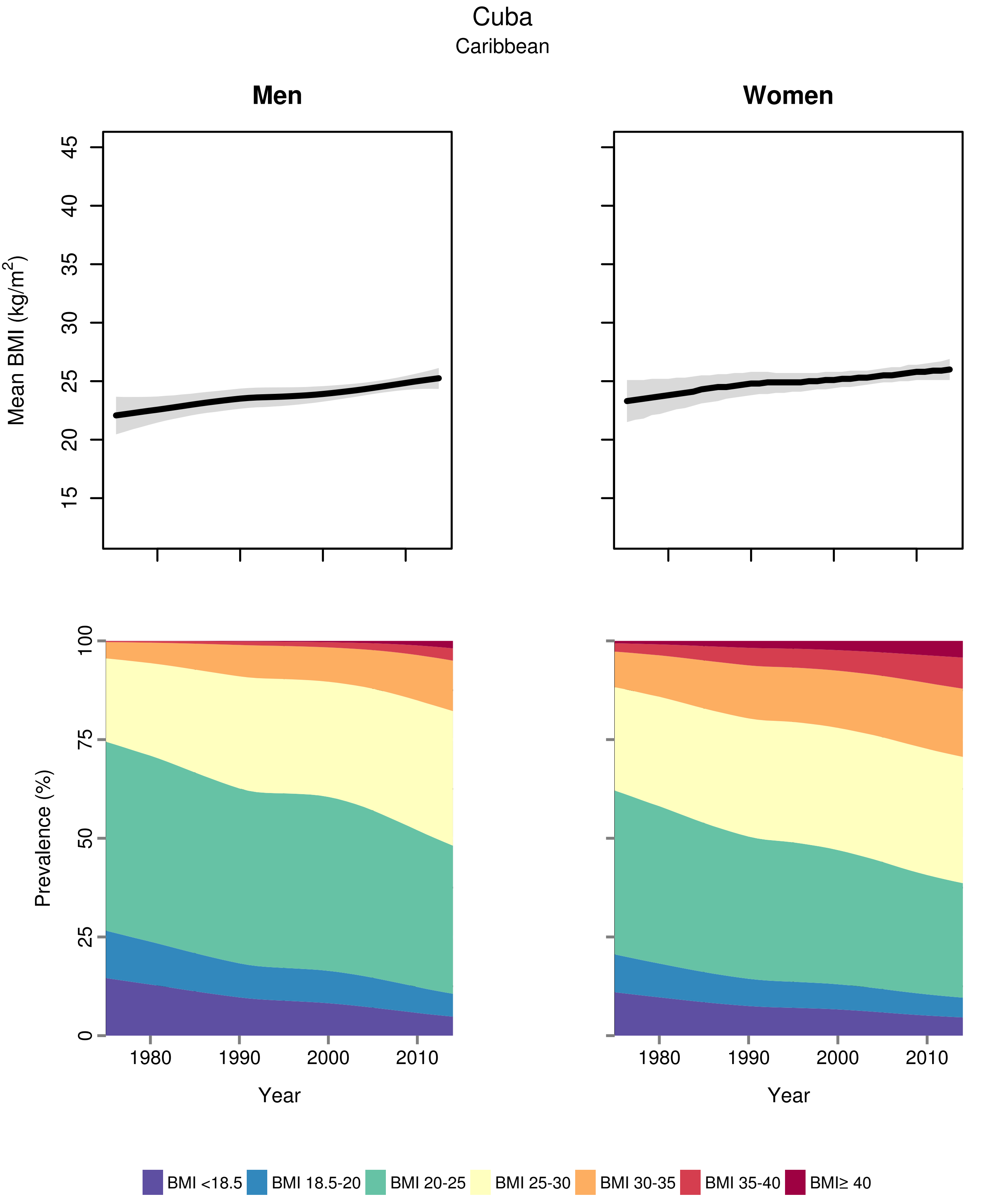

Cuba
Caribbean
Men
Women
Mean BMI (kg/m2)
45
40
35
30
25
20
15
Prevalence (%)
100
75
50
25
0
1980
1990
2000
2010
Year
BMI <18.5
BMI 18.5-20
BMI 20-25
BMI 25-30
BMI 30-35
BMI 35-40
BMI≥ 40

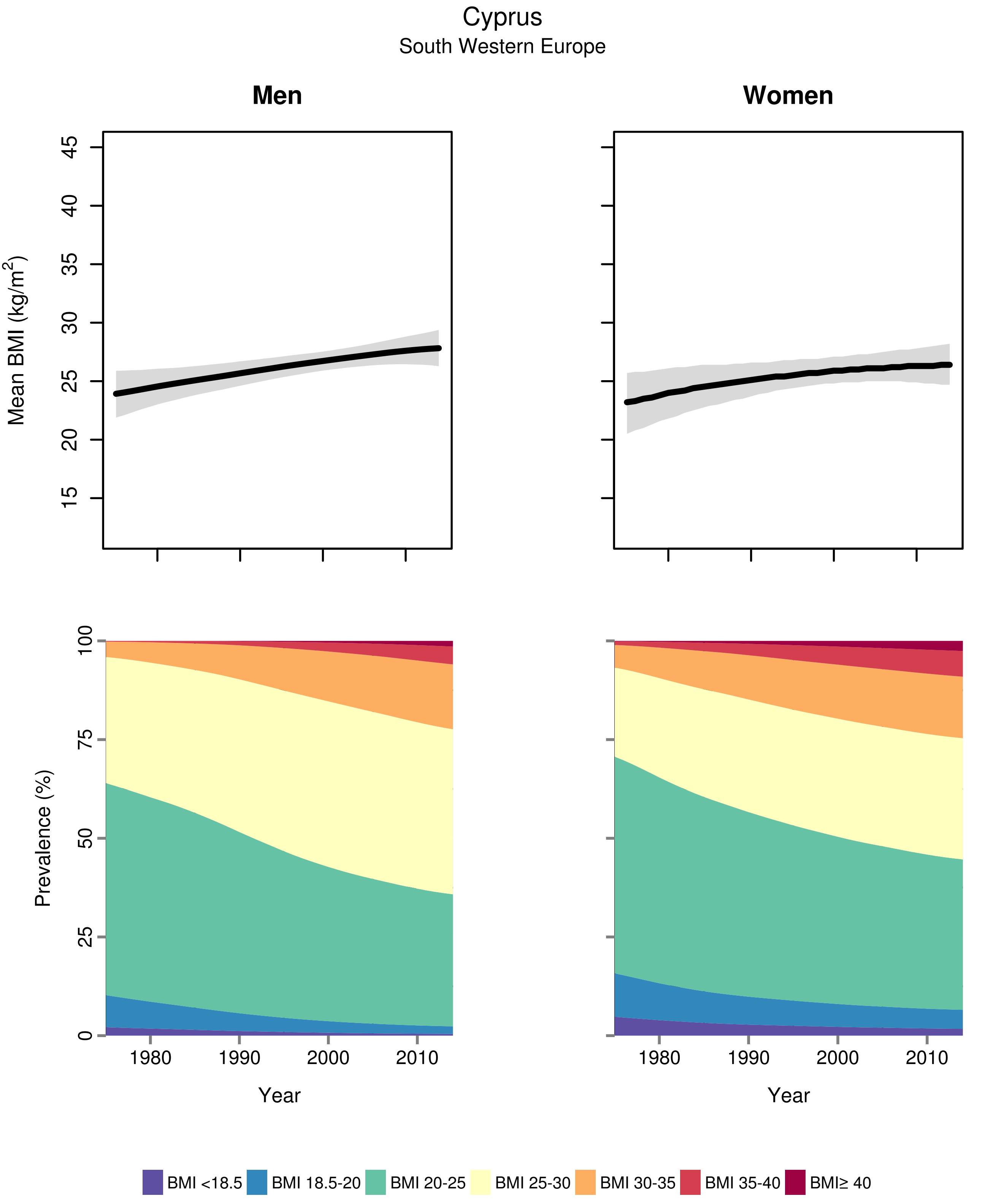

Cyprus
South Western Europe
Men
Women
Mean BMI (kg/m2)
45
40
35
30
25
20
15
Prevalence (%)
100
75
50
25
0
1980
1990
2000
2010
Year
Year
BMI <18.5
BMI 18.5-20
BMI 20-25
BMI 25-30
BMI 30-35
BMI 35-40
BMI≥ 40

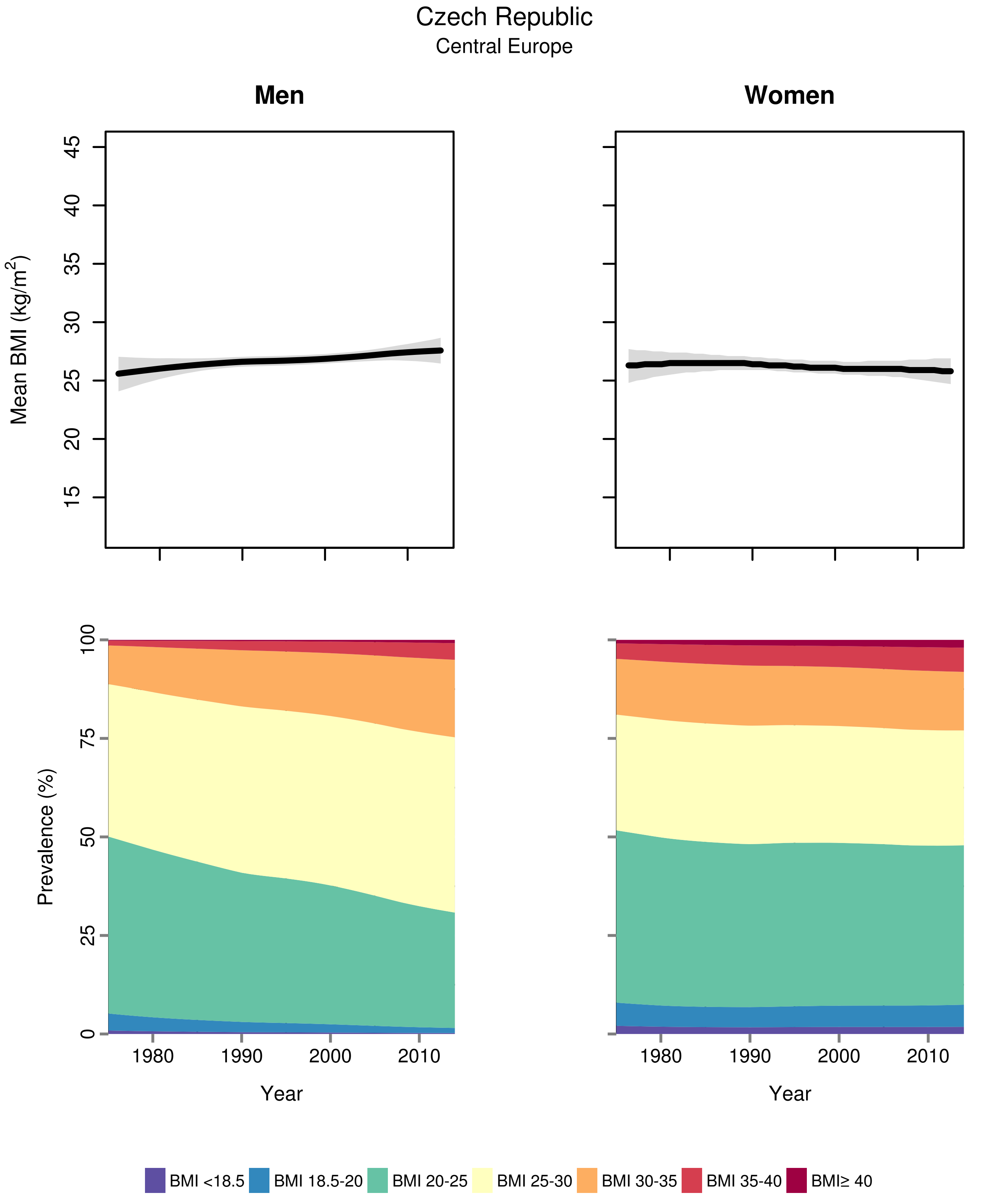
Czech Republic
Central Europe
Men
Women
Mean BMI (kg/m2)
45
40
35
30
25
20
15
Prevalence (%)
100
75
50
25
0
1980
1990
2000
2010
Year
BMI <18.5
BMI 18.5-20
BMI 20-25
BMI 25-30
BMI 30-35
BMI 35-40
BMI≥ 40

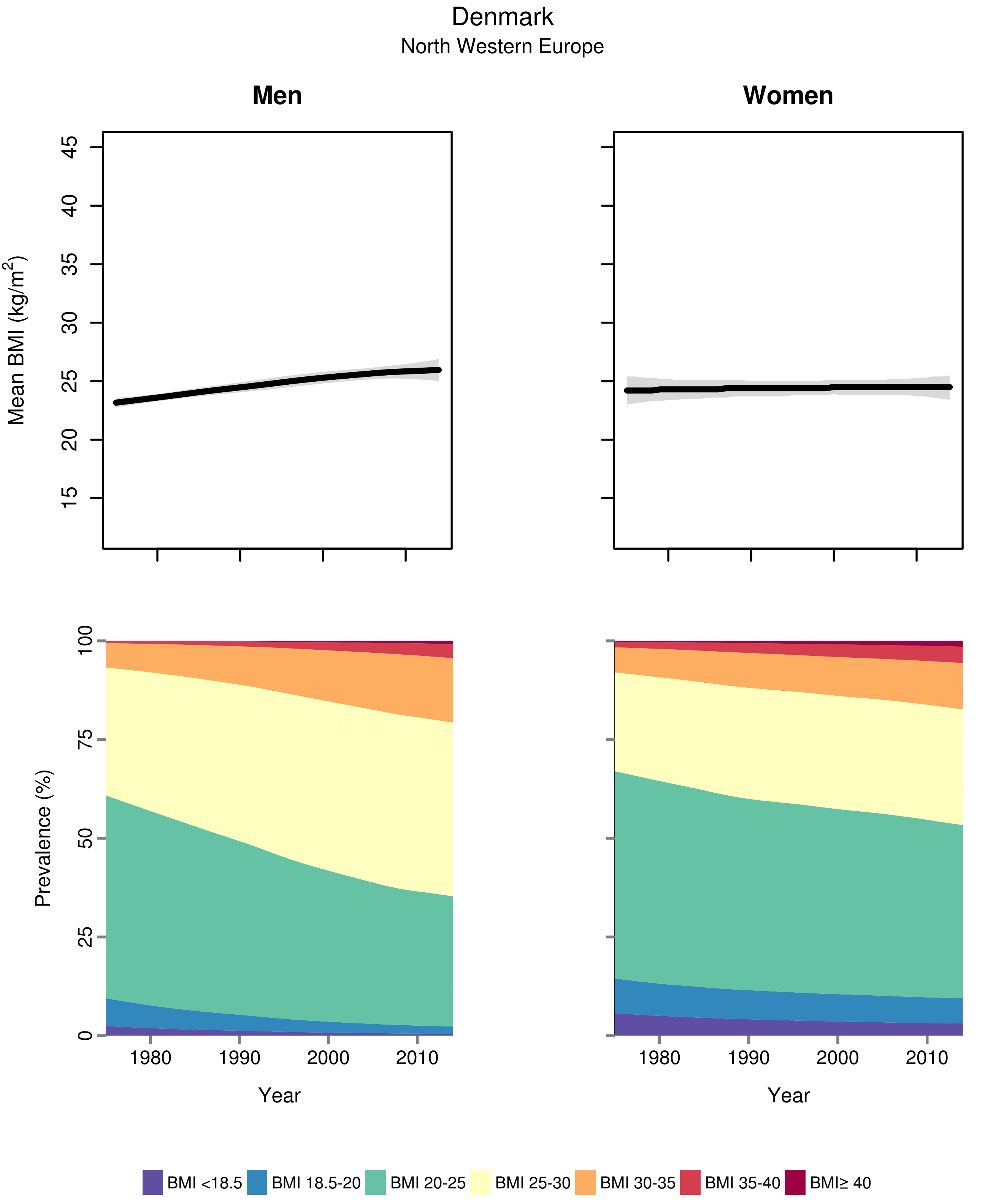
Denmark
North Western Europe
Men
Women
Mean BMI (kg/m2)
45
40
35
30
25
20
15
Prevalence (%)
100
75
50
25
0
1980
1990
2000
2010
Year
BMI <18.5
BMI 18.5-20
BMI 20-25
BMI 25-30
BMI 30-35
BMI 35-40
BMI≥ 40

Djibouti

East Africa

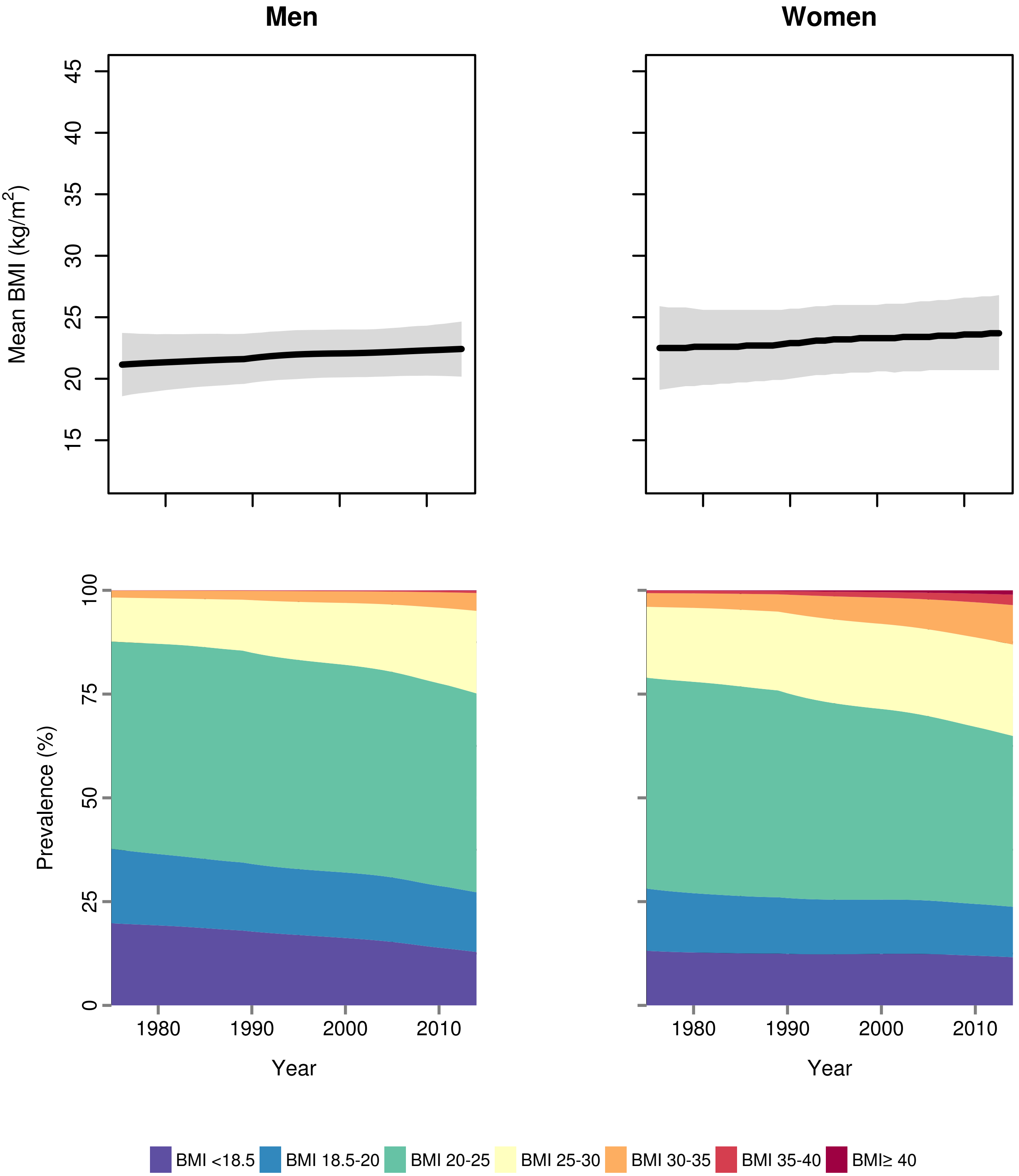

Dominica
Caribbean

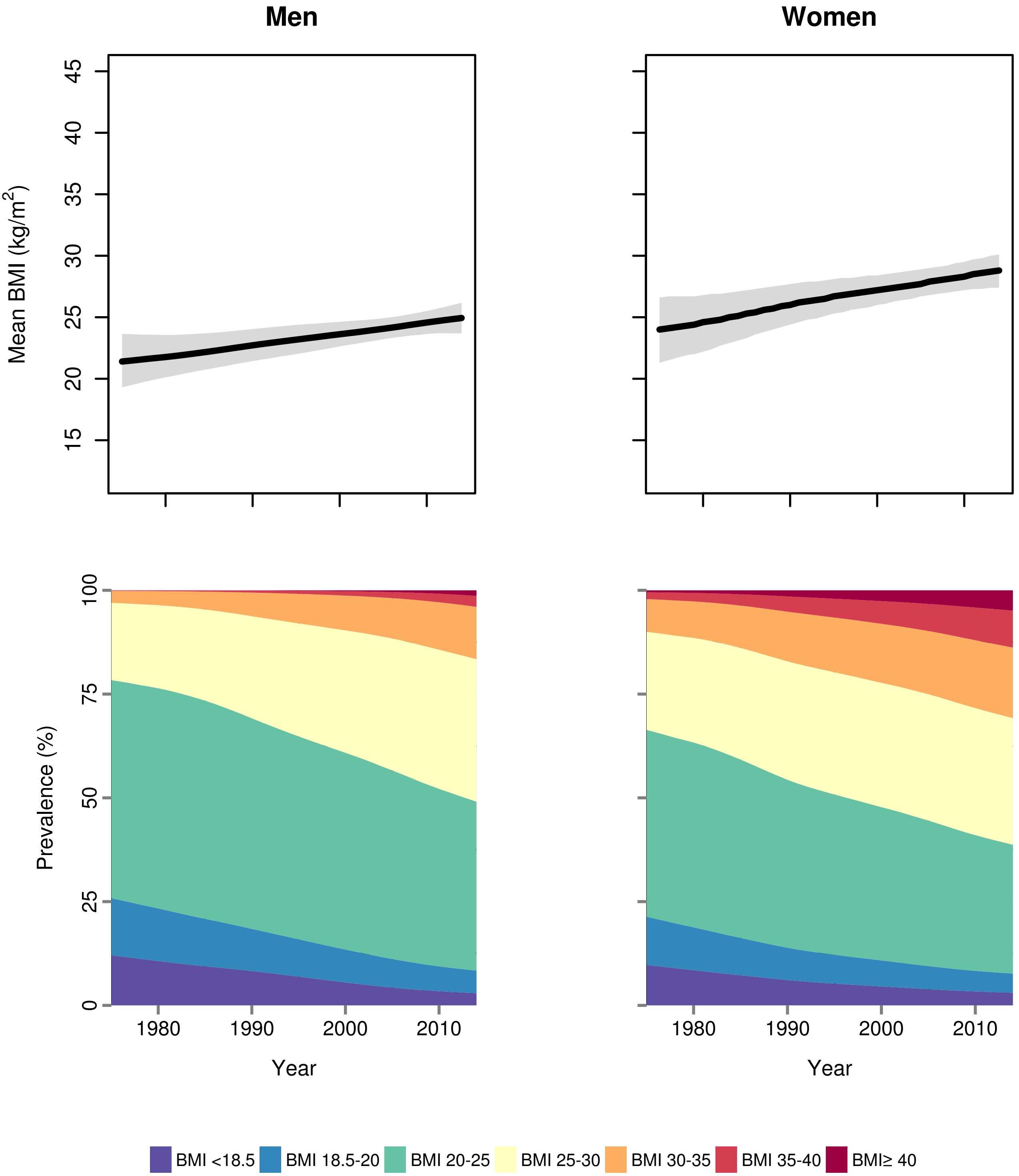

Dominican Republic
Caribbean

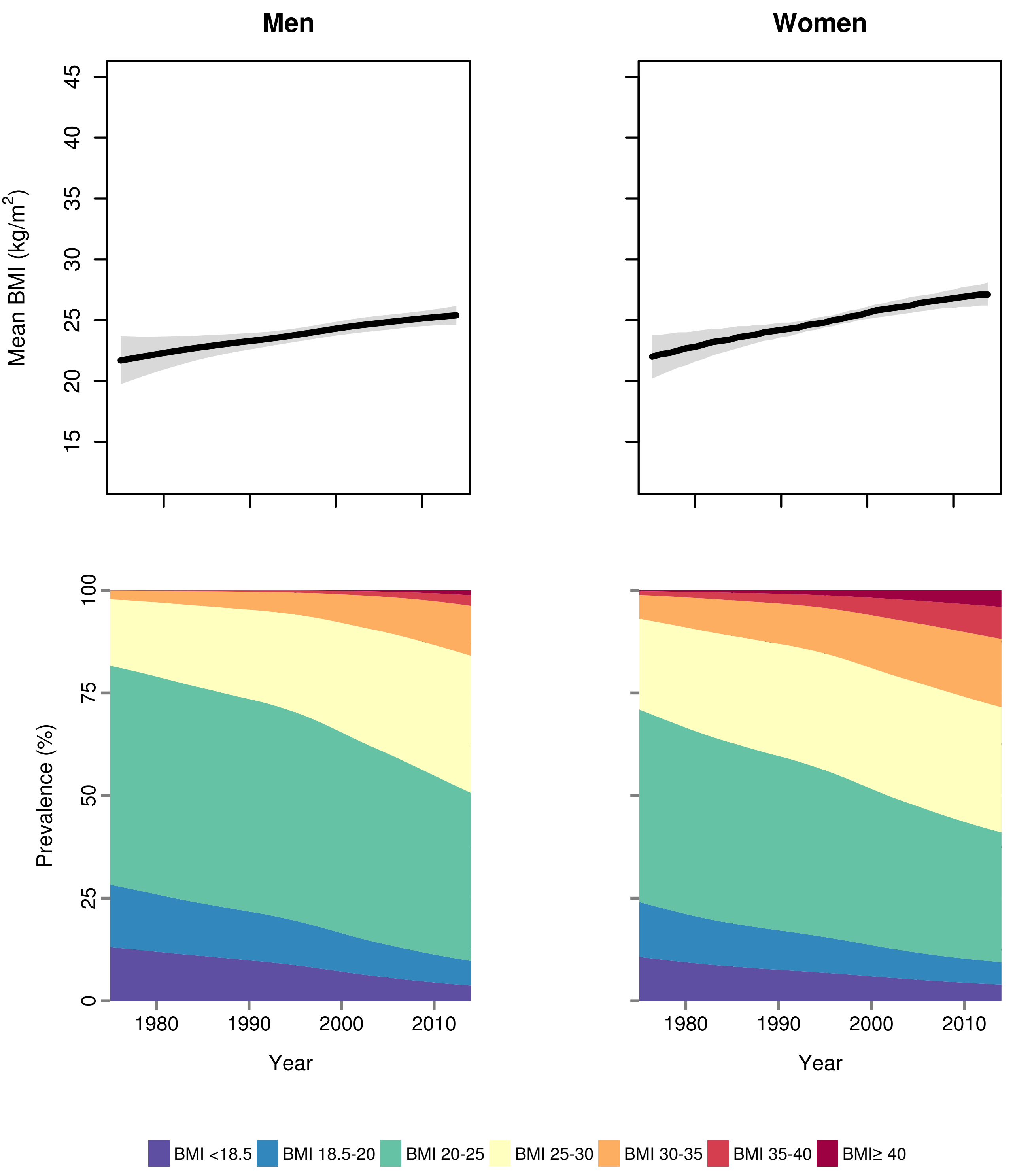

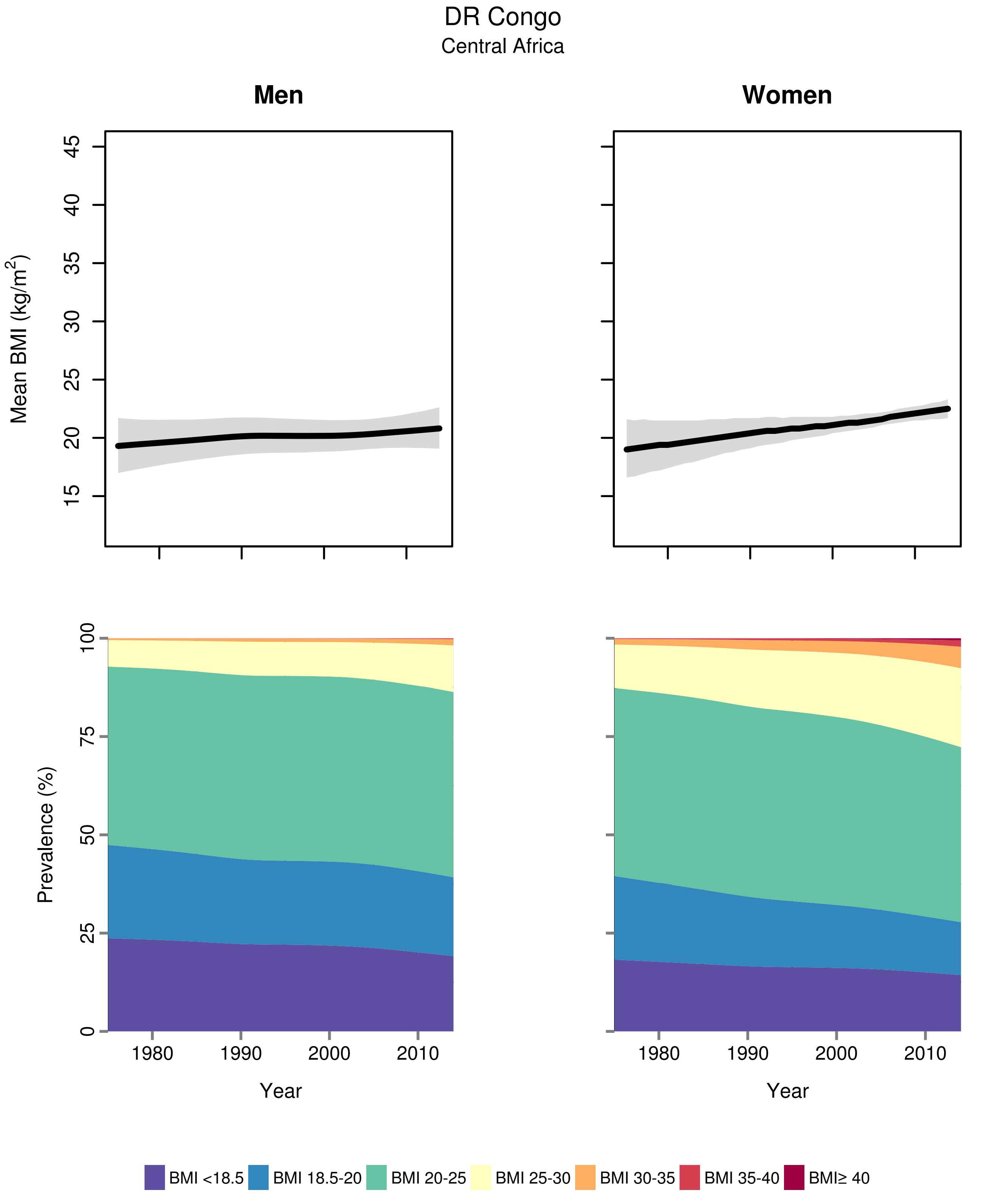

DR Congo
Central Africa
Men
Women
Mean BMI (kg/m^2)
45
40
35
30
25
20
15
Prevalence (%)
100
75
50
25
0
1980
1990
2000
2010
Year
BMI <18.5
BMI 18.5-20
BMI 20-25
BMI 25-30
BMI 30-35
BMI 35-40
BMI≥ 40

Ecuador

Andean Latin America

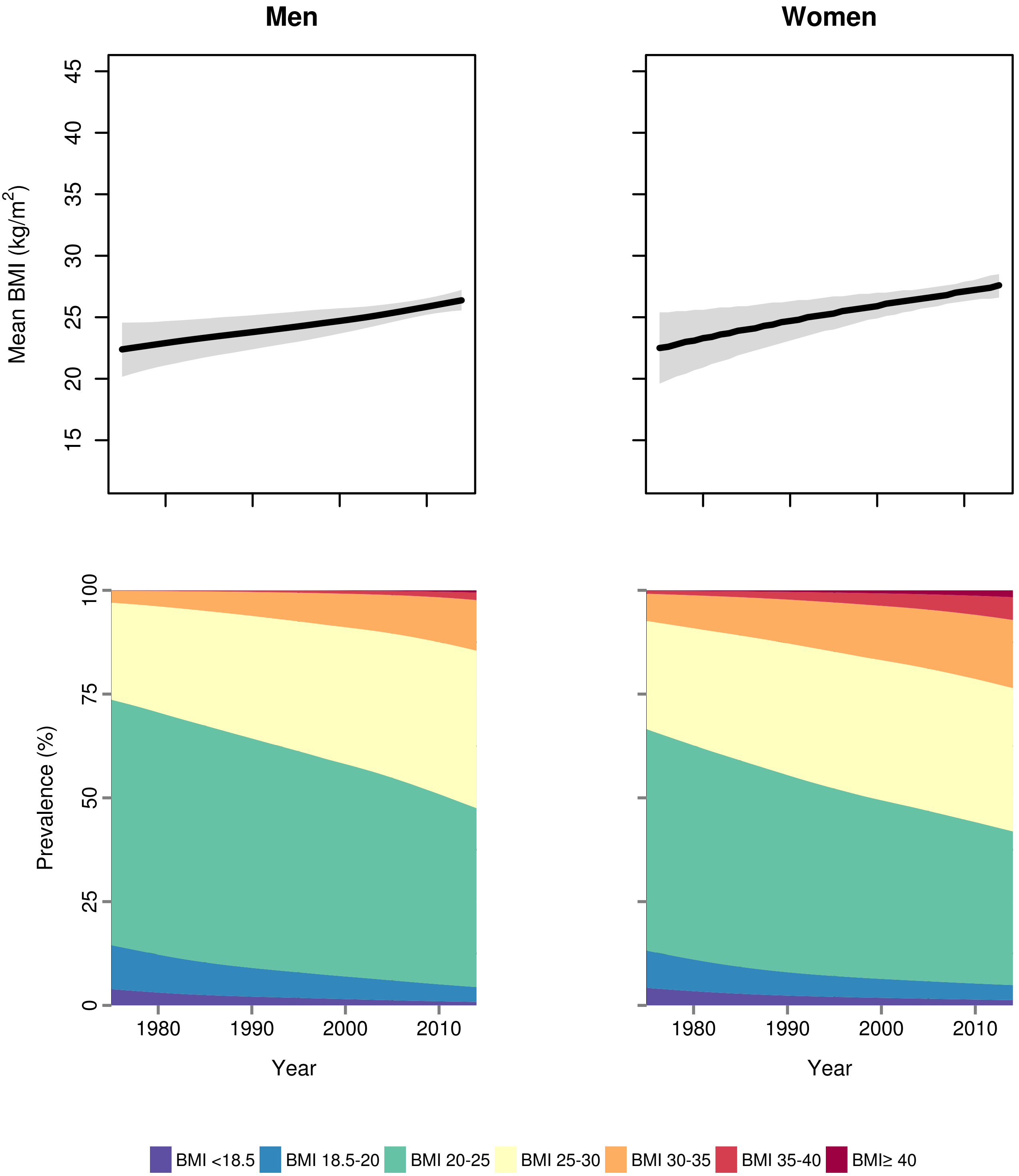

Egypt

Middle East and North Africa

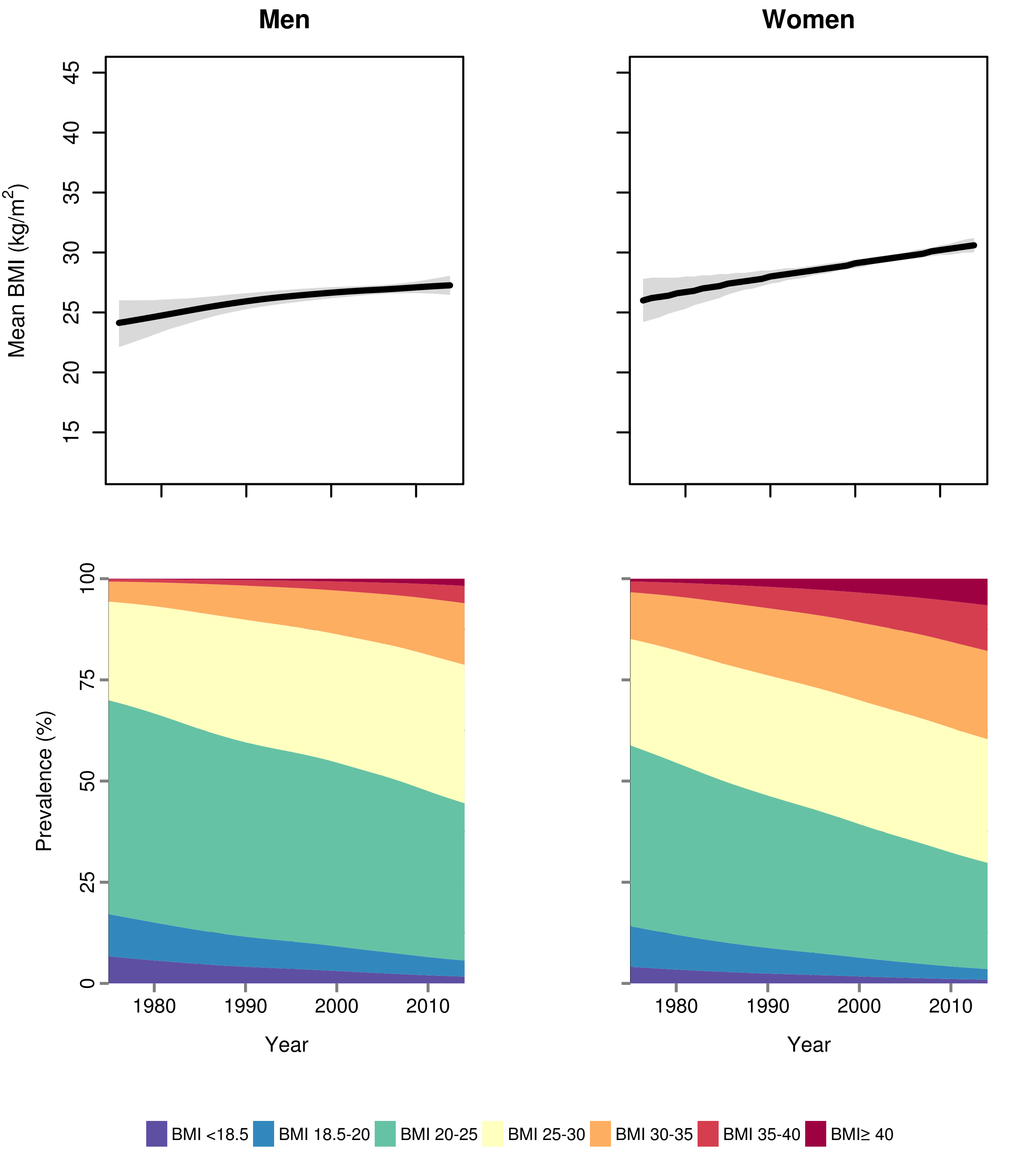

El Salvador

Central Latin America

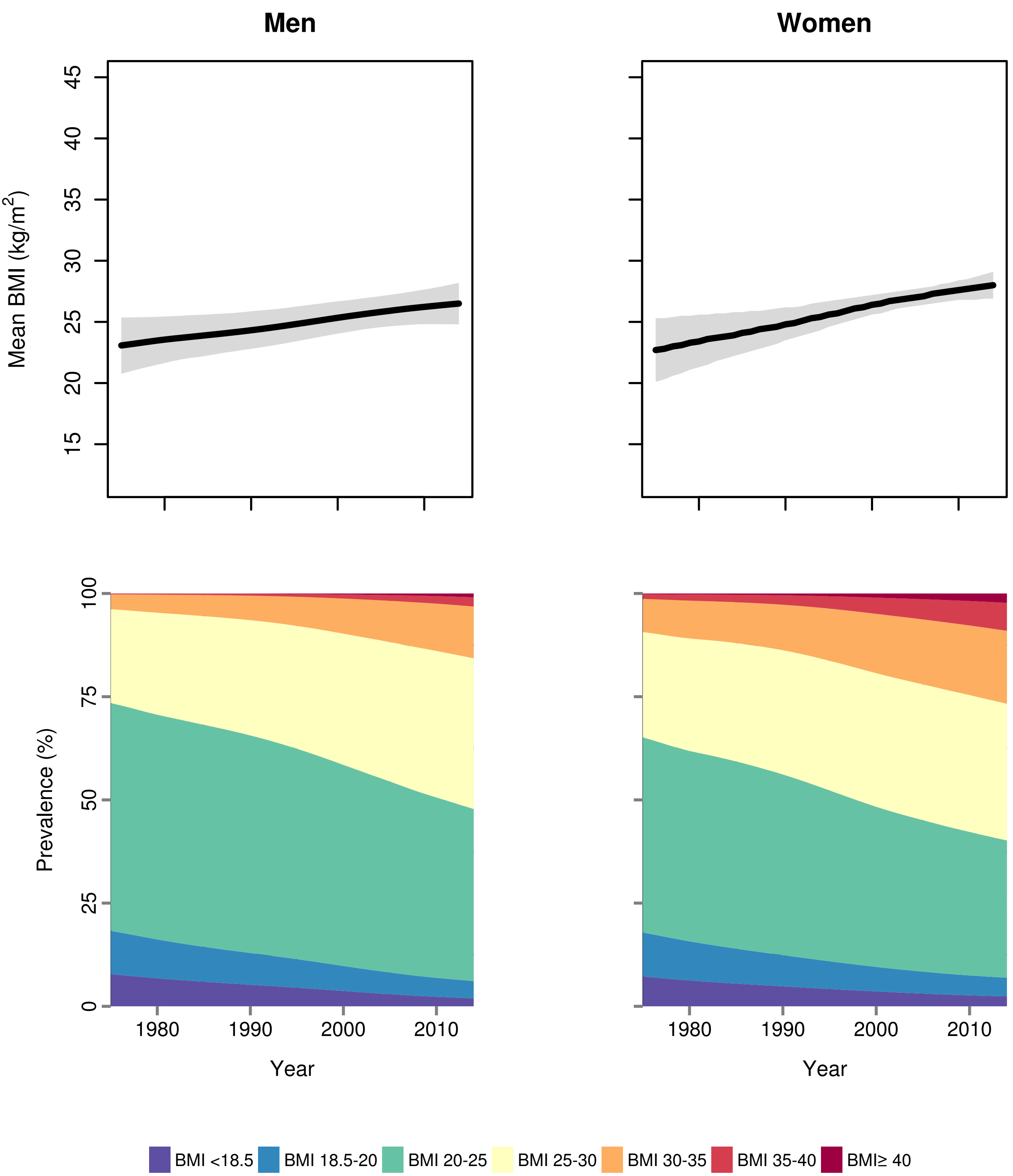

Equatorial Guinea
Central Africa

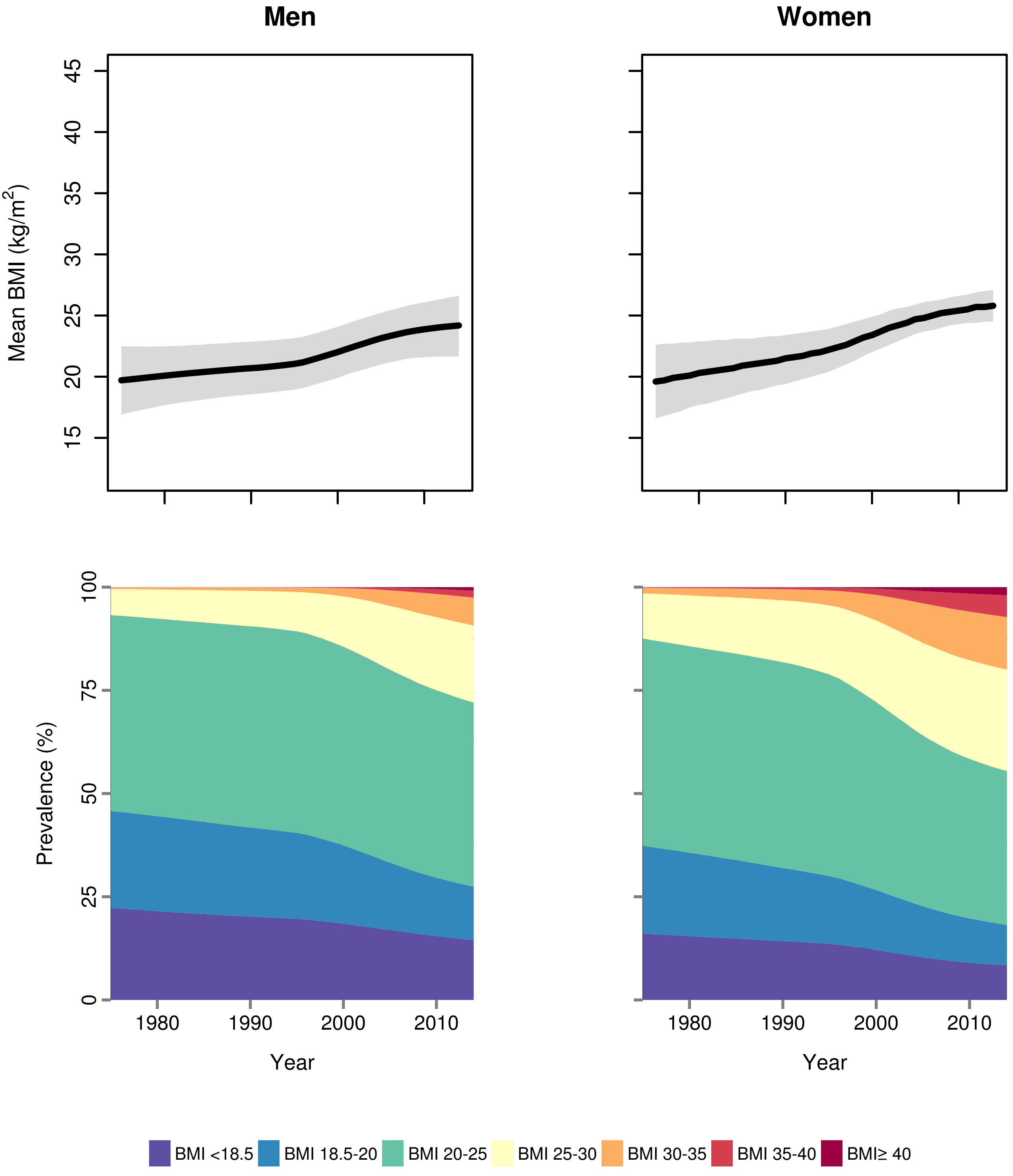

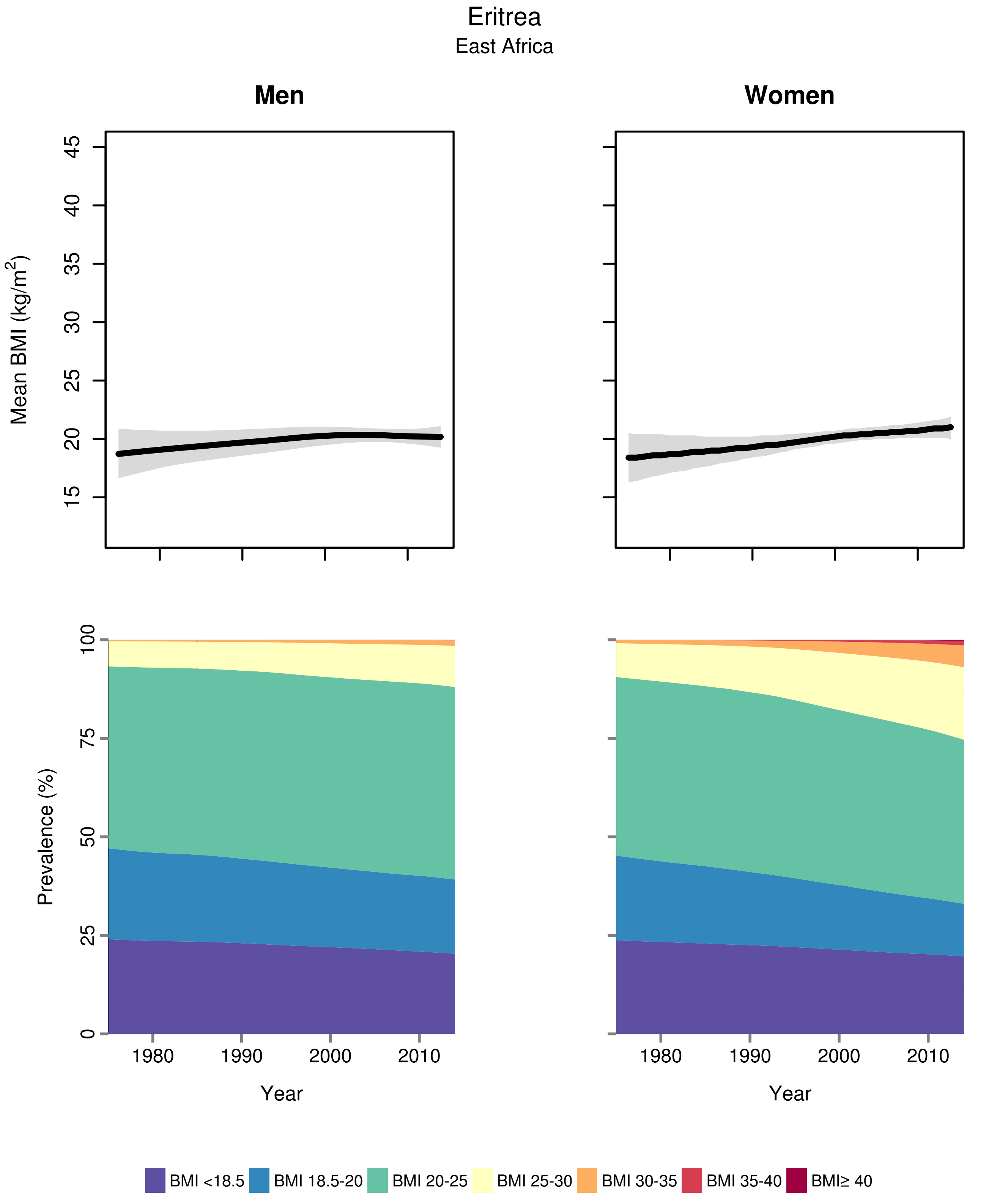
Eritrea
East Africa
Men
Women
Mean BMI (kg/m2)
45
40
35
30
25
20
15
Prevalence (%)
100
75
50
25
0
1980
1990
2000
2010
Year
BMI <18.5
BMI 18.5-20
BMI 20-25
BMI 25-30
BMI 30-35
BMI 35-40
BMI≥ 40

Estonia

Eastern Europe

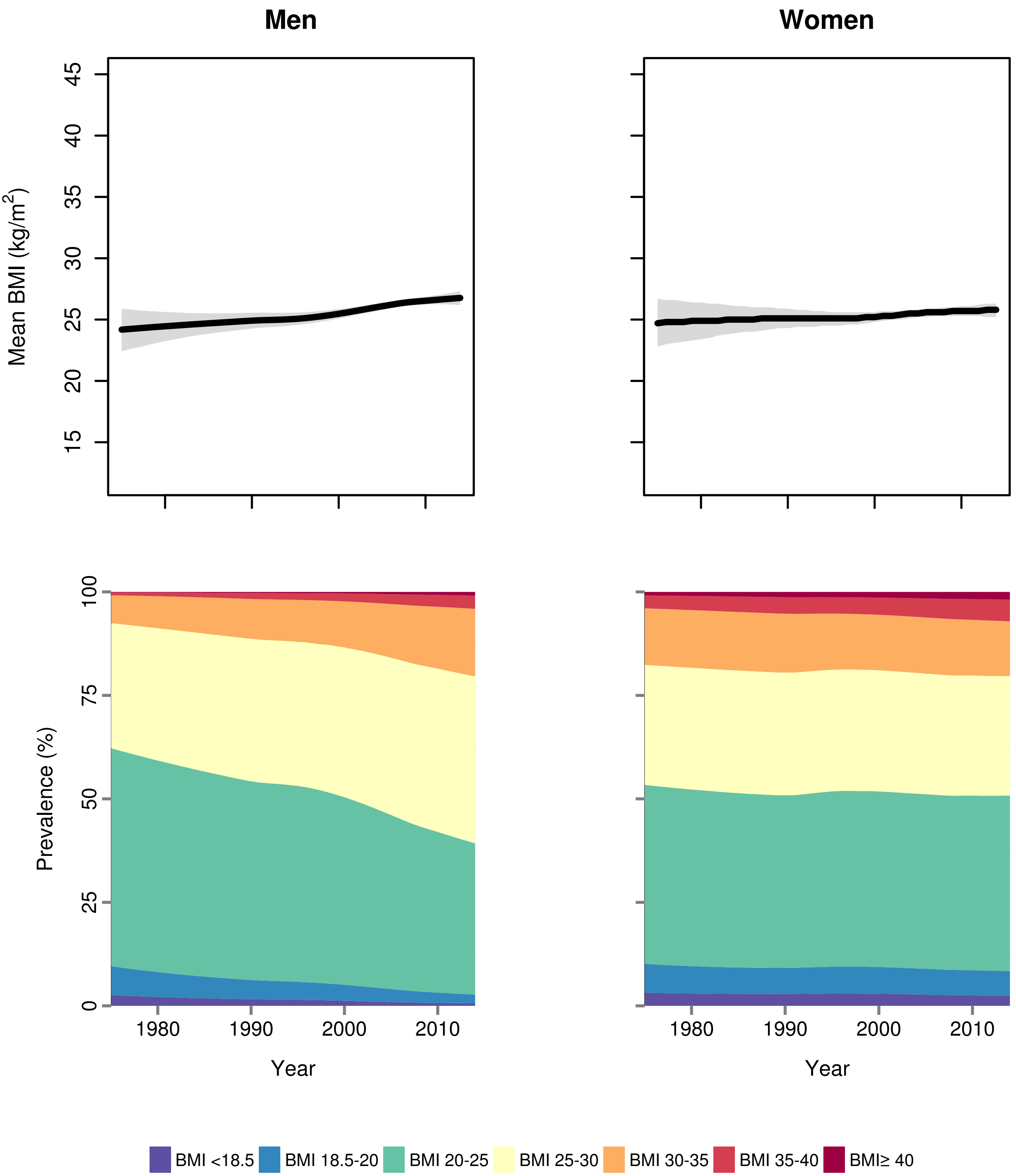

Ethiopia
East Africa

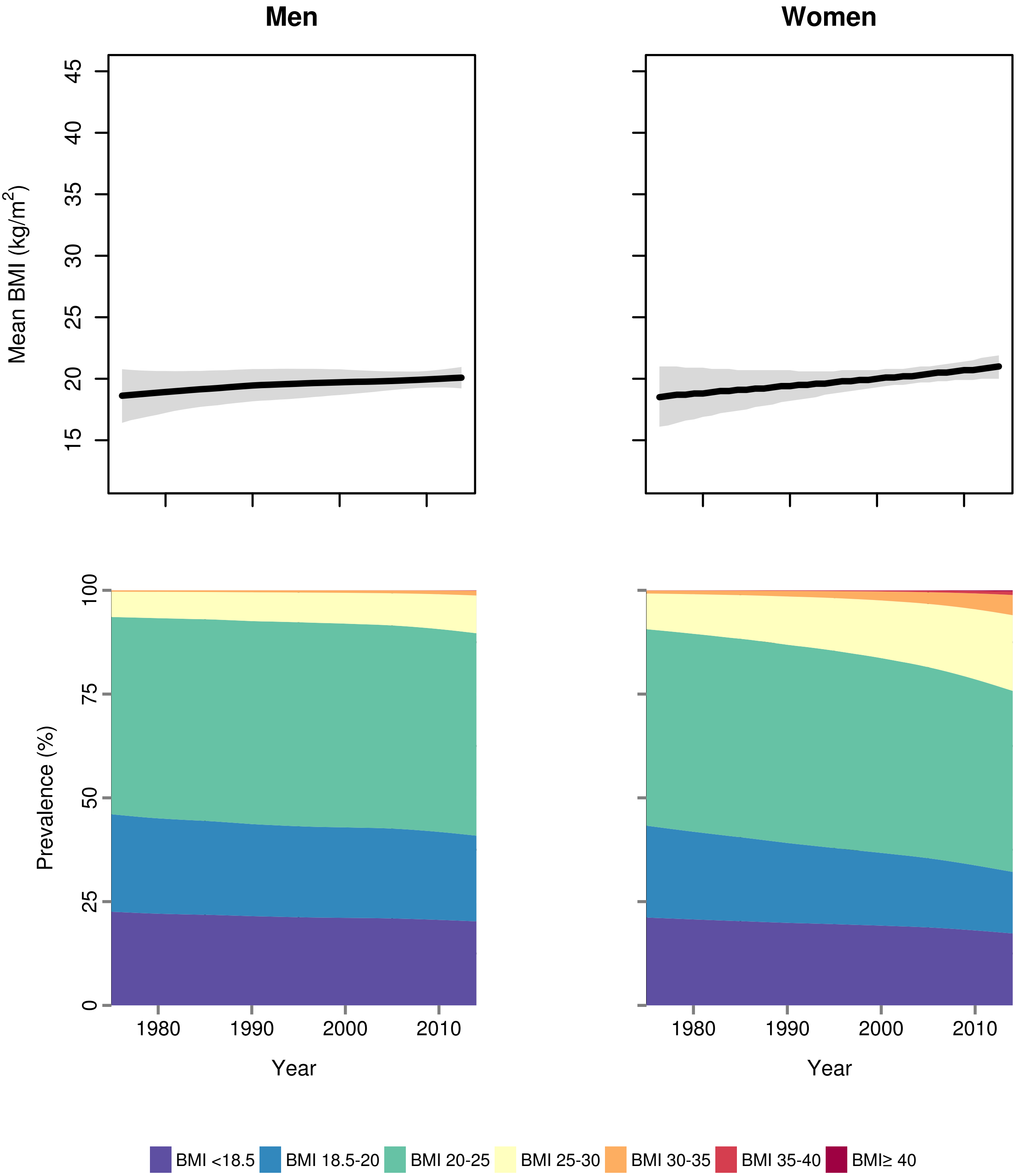

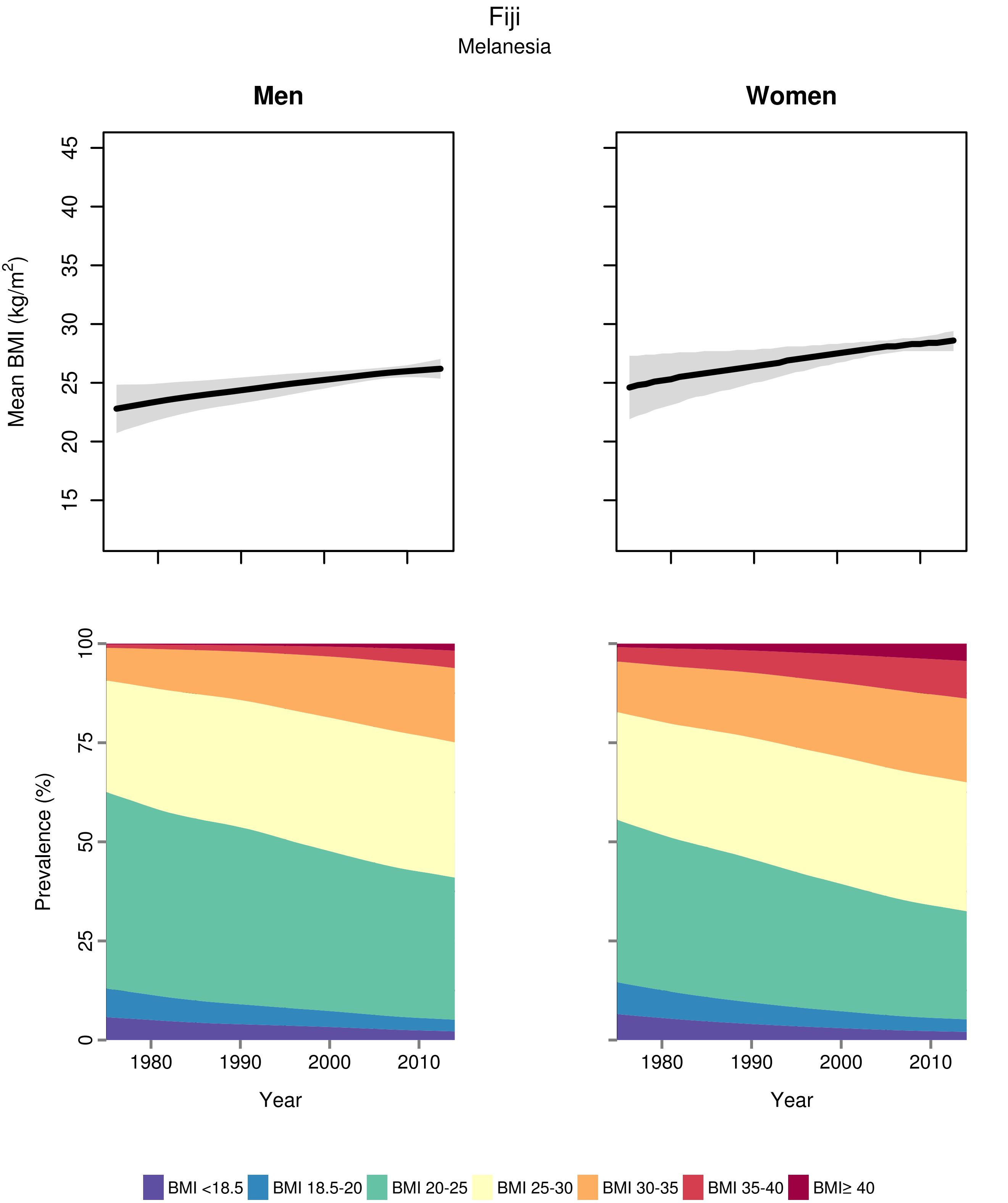

Fiji
Melanesia
Men
Women
Mean BMI (kg/m2)
45
40
35
30
25
20
15
100
75
50
25
0
Prevalence (%)
1980
1990
2000
2010
Year
BMI <18.5
BMI 18.5-20
BMI 20-25
BMI 25-30
BMI 30-35
BMI 35-40
BMI≥ 40

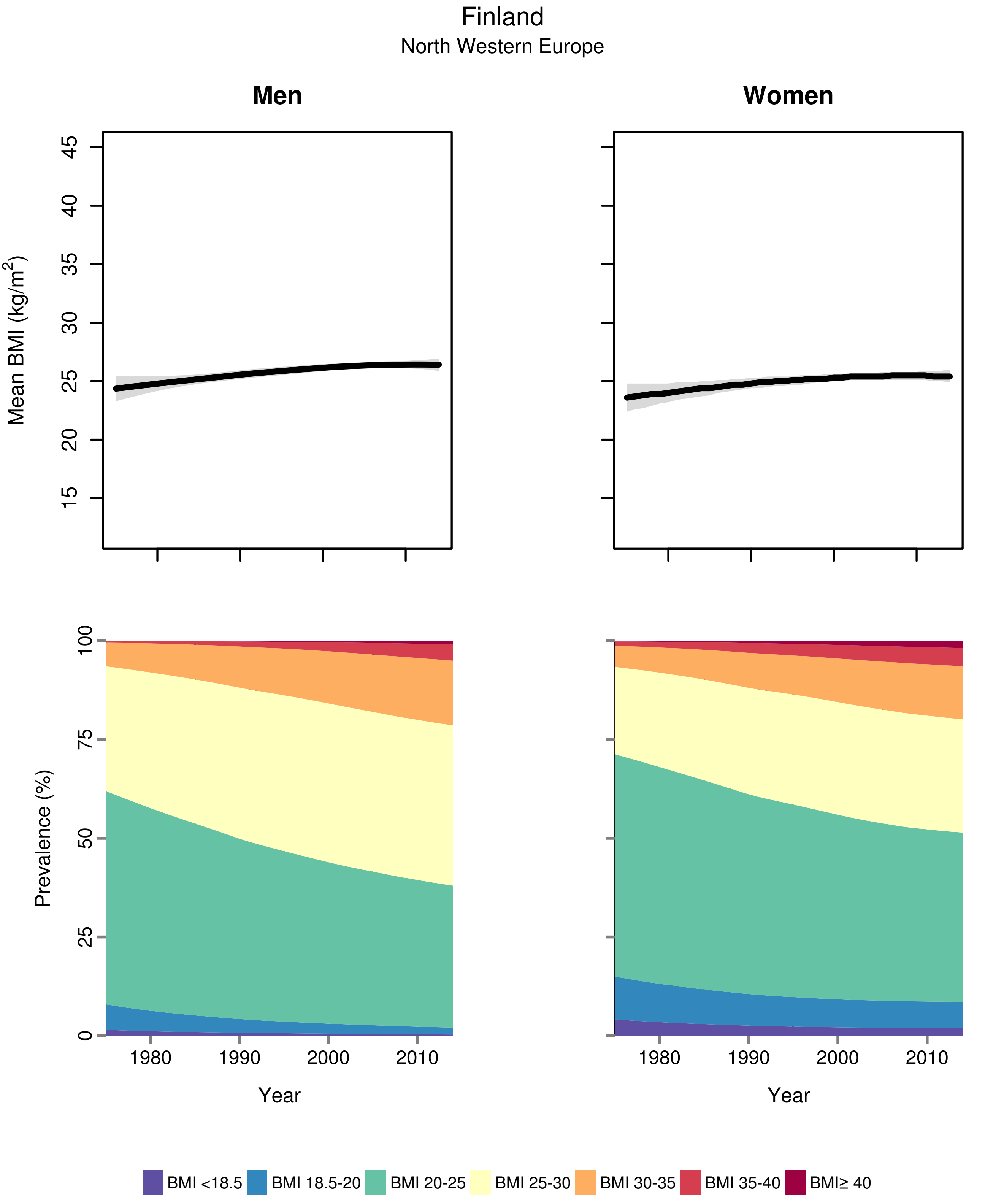
Finland
North Western Europe
Men
Women
Mean BMI (kg/m²)
45
40
35
30
25
20
15
100
75
50
25
0
Prevalence (%)
1980
1990
2000
2010
Year
BMI <18.5
BMI 18.5-20
BMI 20-25
BMI 25-30
BMI 30-35
BMI 35-40
BMI≥ 40

France

South Western Europe

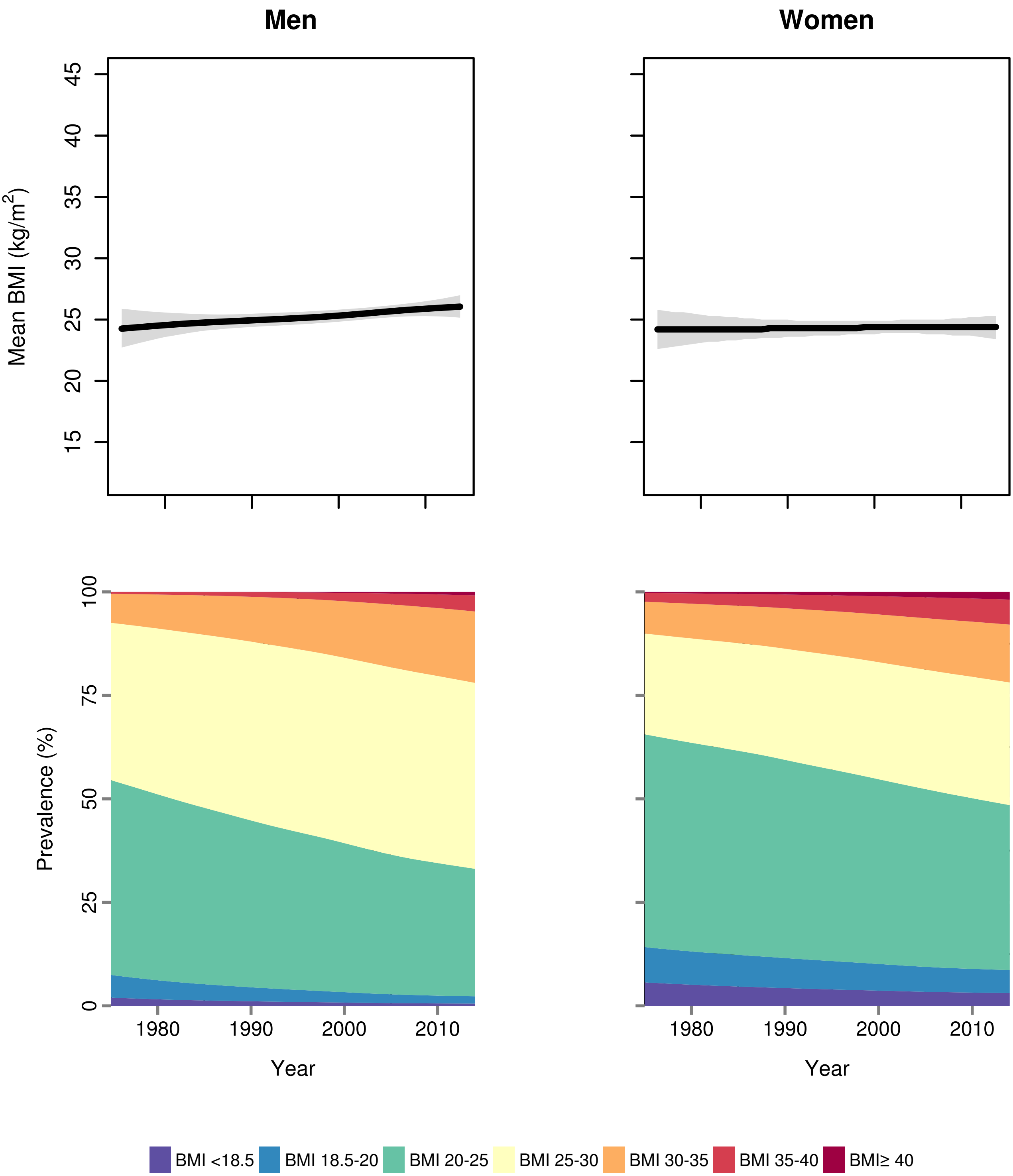

French Polynesia

Polynesia and Micronesia

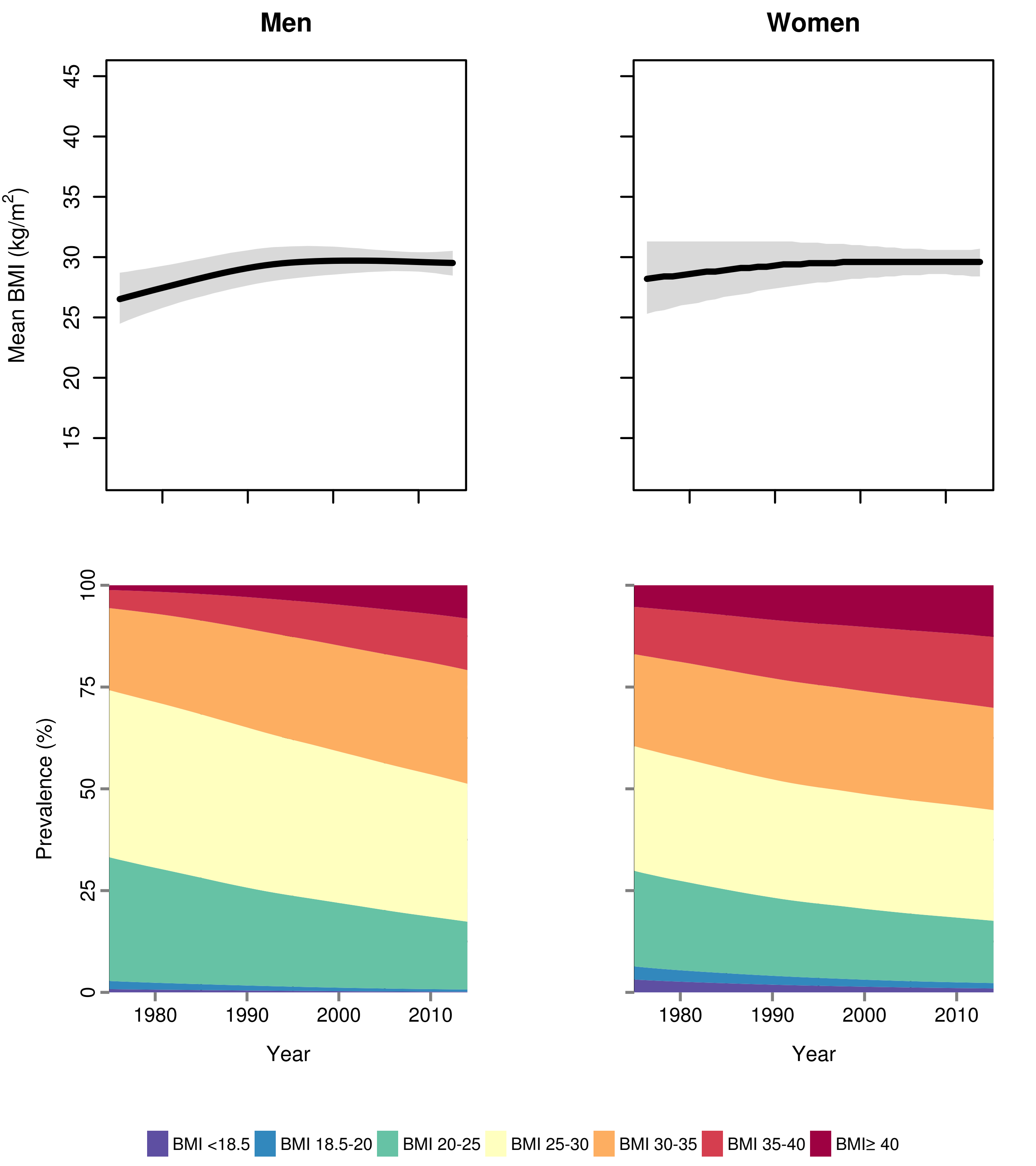

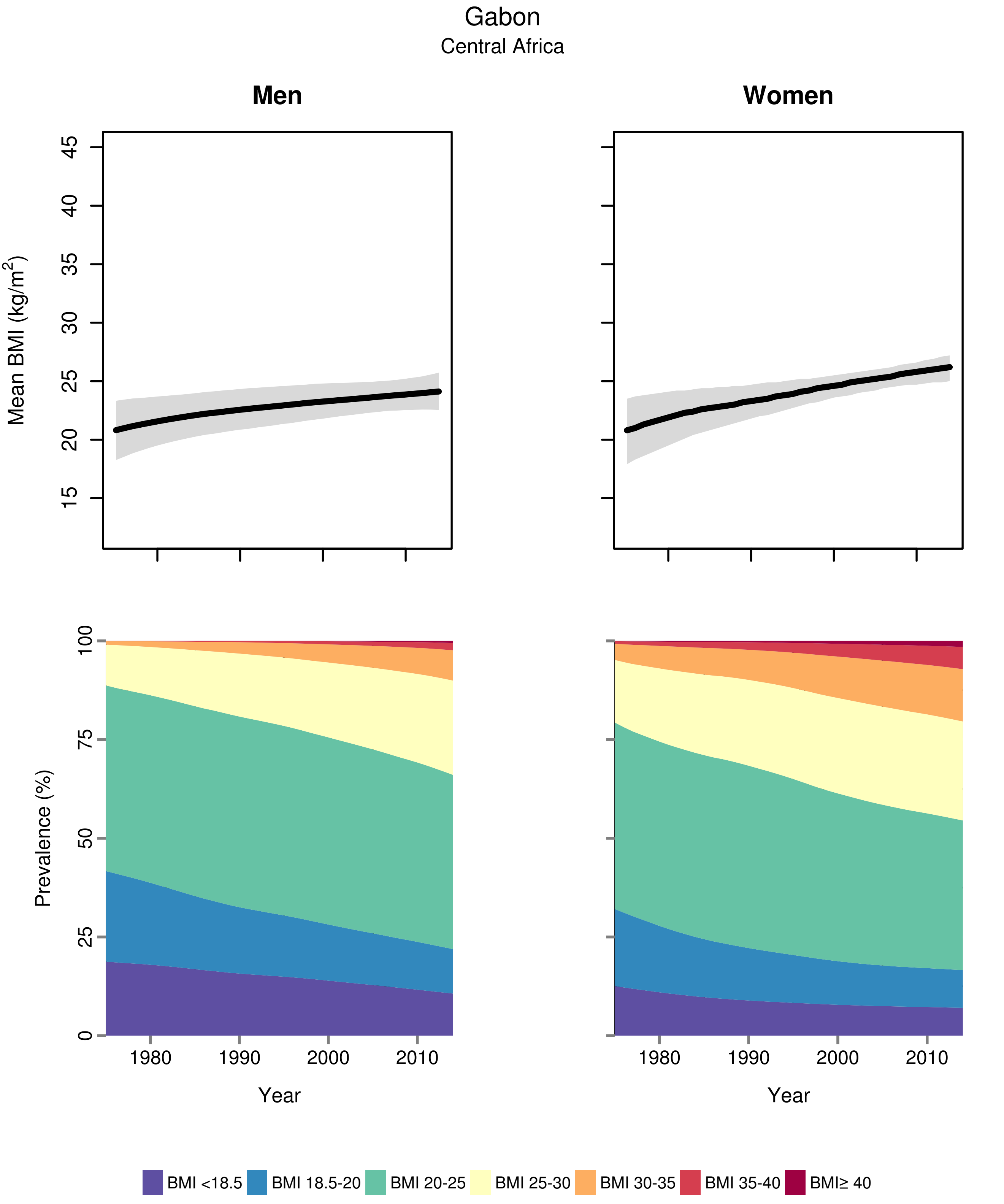

Gabon
Central Africa
Men
Women
Mean BMI (kg/m2)
45
40
35
30
25
20
15
Prevalence (%)
100
75
50
25
0
1980
1990
2000
2010
Year
BMI <18.5
BMI 18.5-20
BMI 20-25
BMI 25-30
BMI 30-35
BMI 35-40
BMI≥ 40

Gambia

West Africa

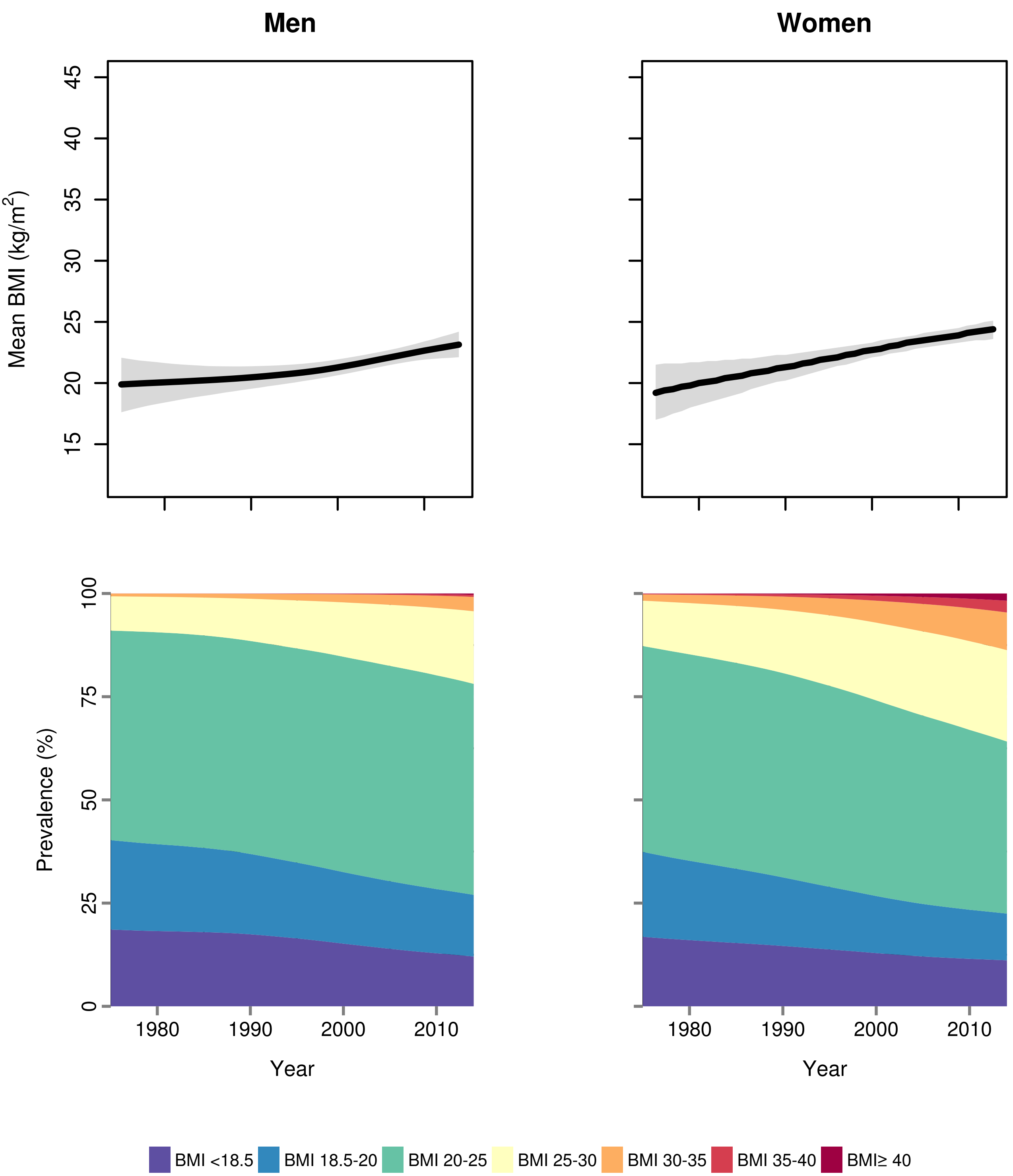

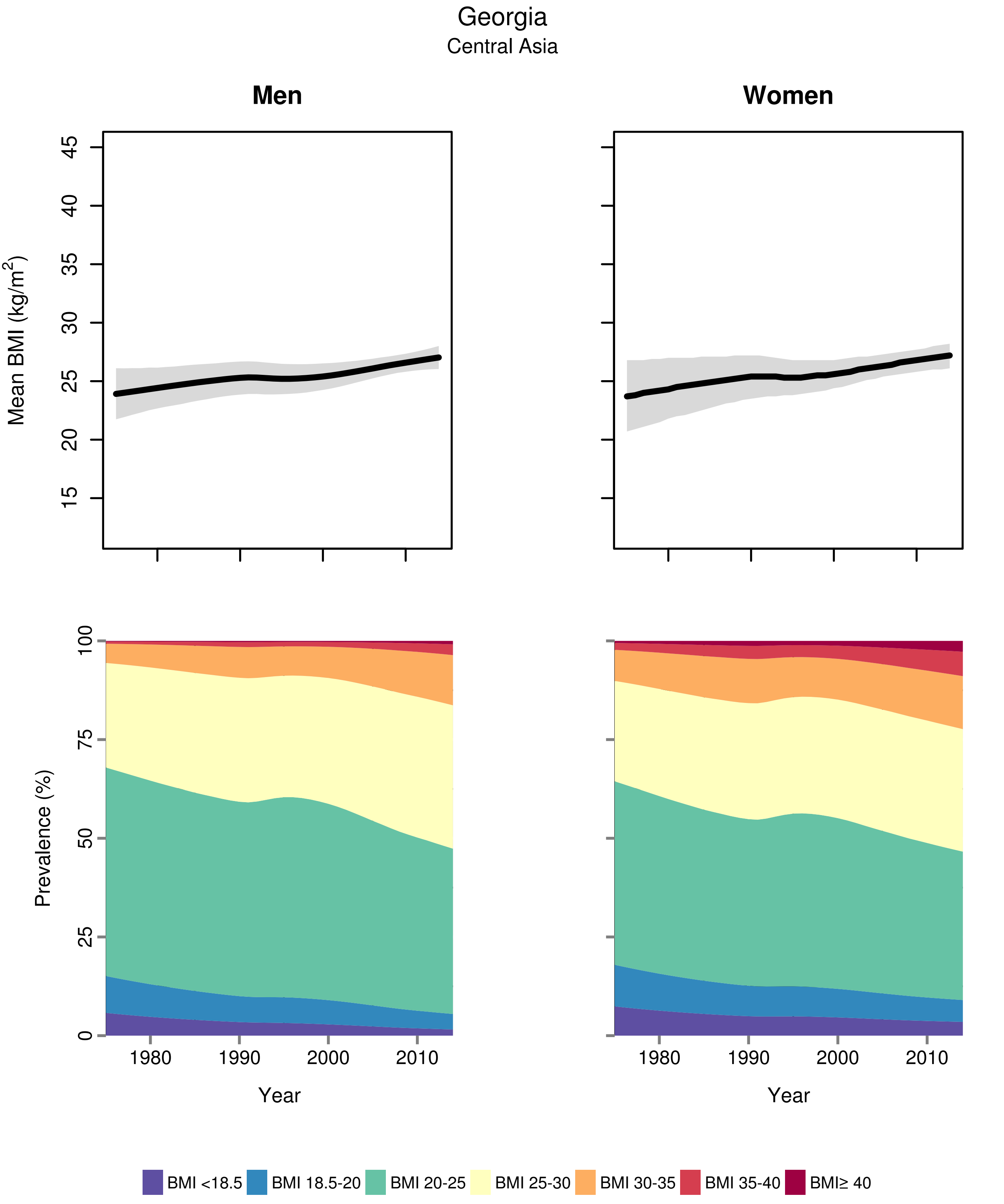

Georgia
Central Asia
Men
Women
Mean BMI (kg/m2)
45
40
35
30
25
20
15
Prevalence (%)
100
75
50
25
0
1980
1990
2000
2010
Year
BMI <18.5
BMI 18.5-20
BMI 20-25
BMI 25-30
BMI 30-35
BMI 35-40
BMI≥ 40

Germany

North Western Europe

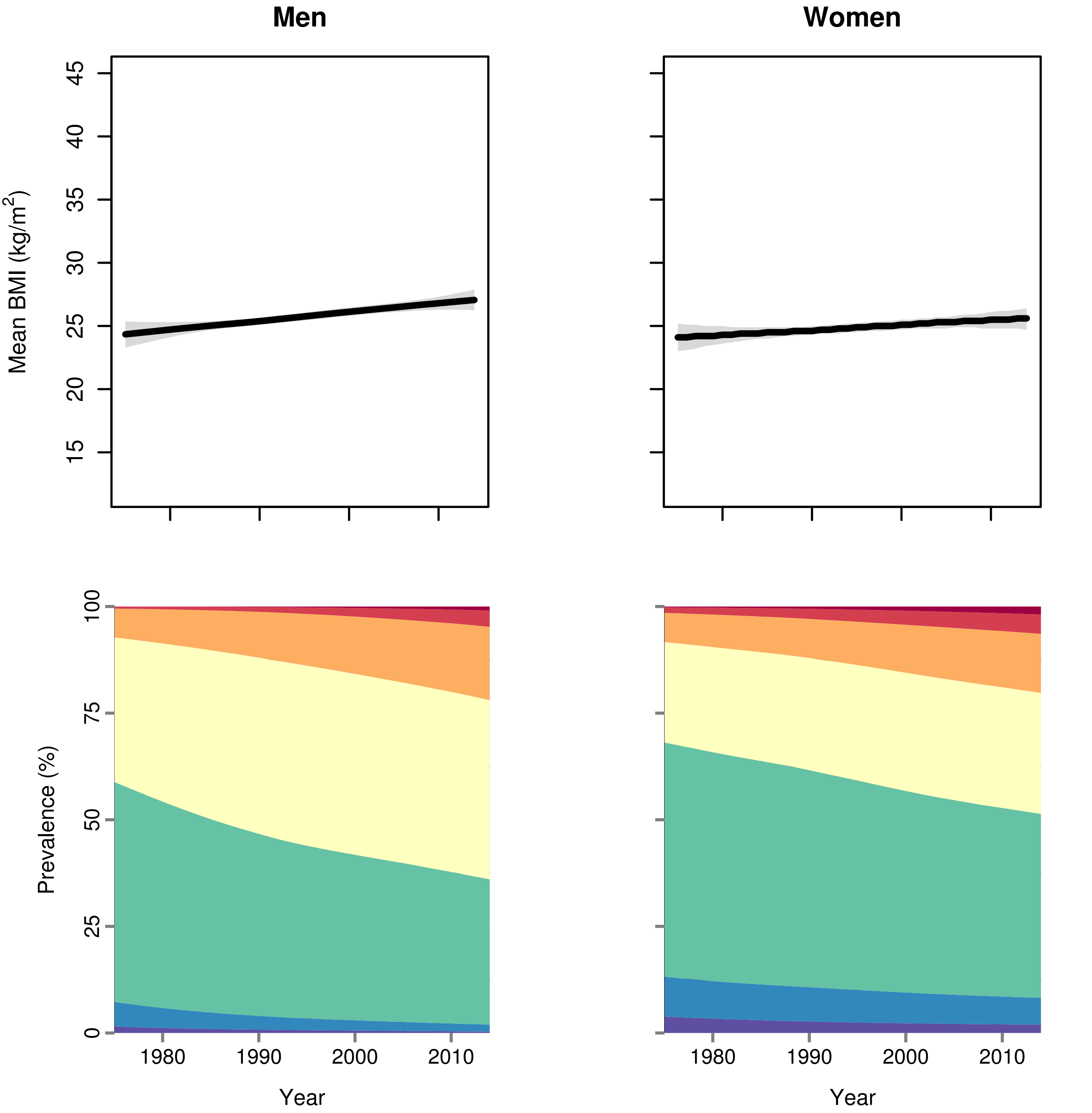

BMI <18.5 | BMI 18.5-20 | BMI 20-25 | BMI 25-30 | BMI 30-35 | BMI 35-40 | BMI≥ 40

Ghana

West Africa

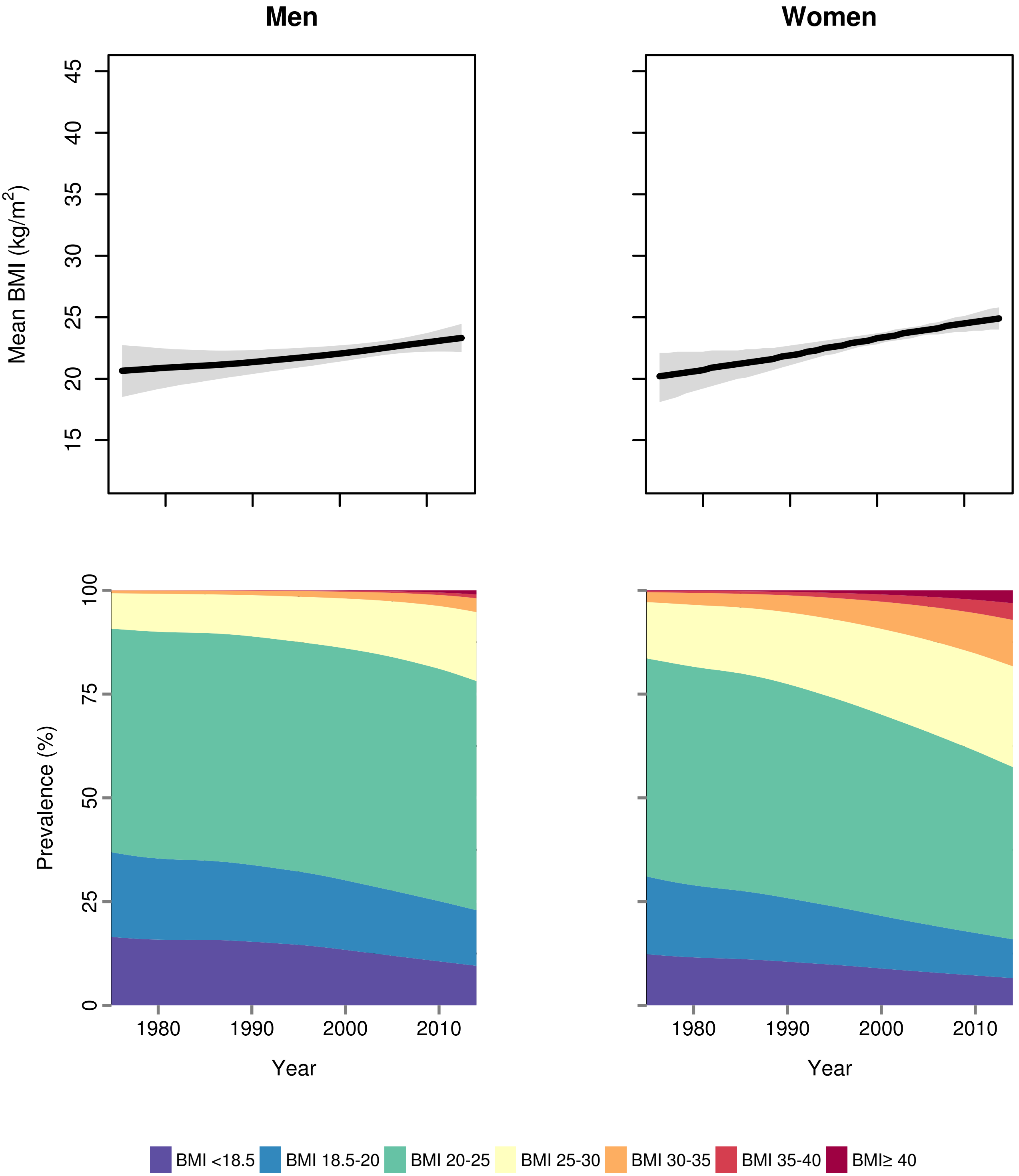

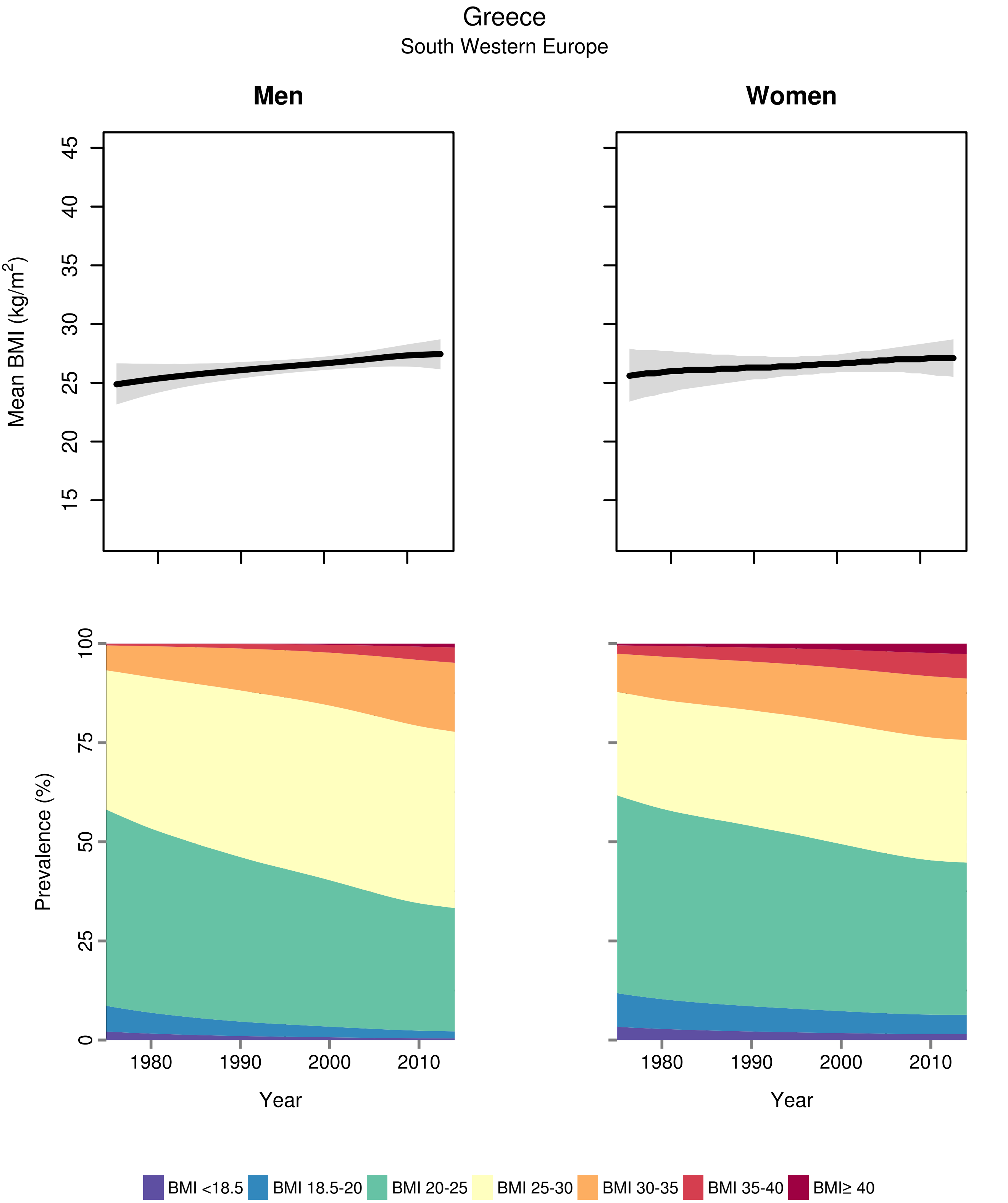

Greece
South Western Europe
Men
Women
Mean BMI (kg/m2)
45
40
35
30
25
20
15
Prevalence (%)
100
75
50
25
0
1980
1990
2000
2010
Year
BMI <18.5
BMI 18.5-20
BMI 20-25
BMI 25-30
BMI 30-35
BMI 35-40
BMI≥ 40

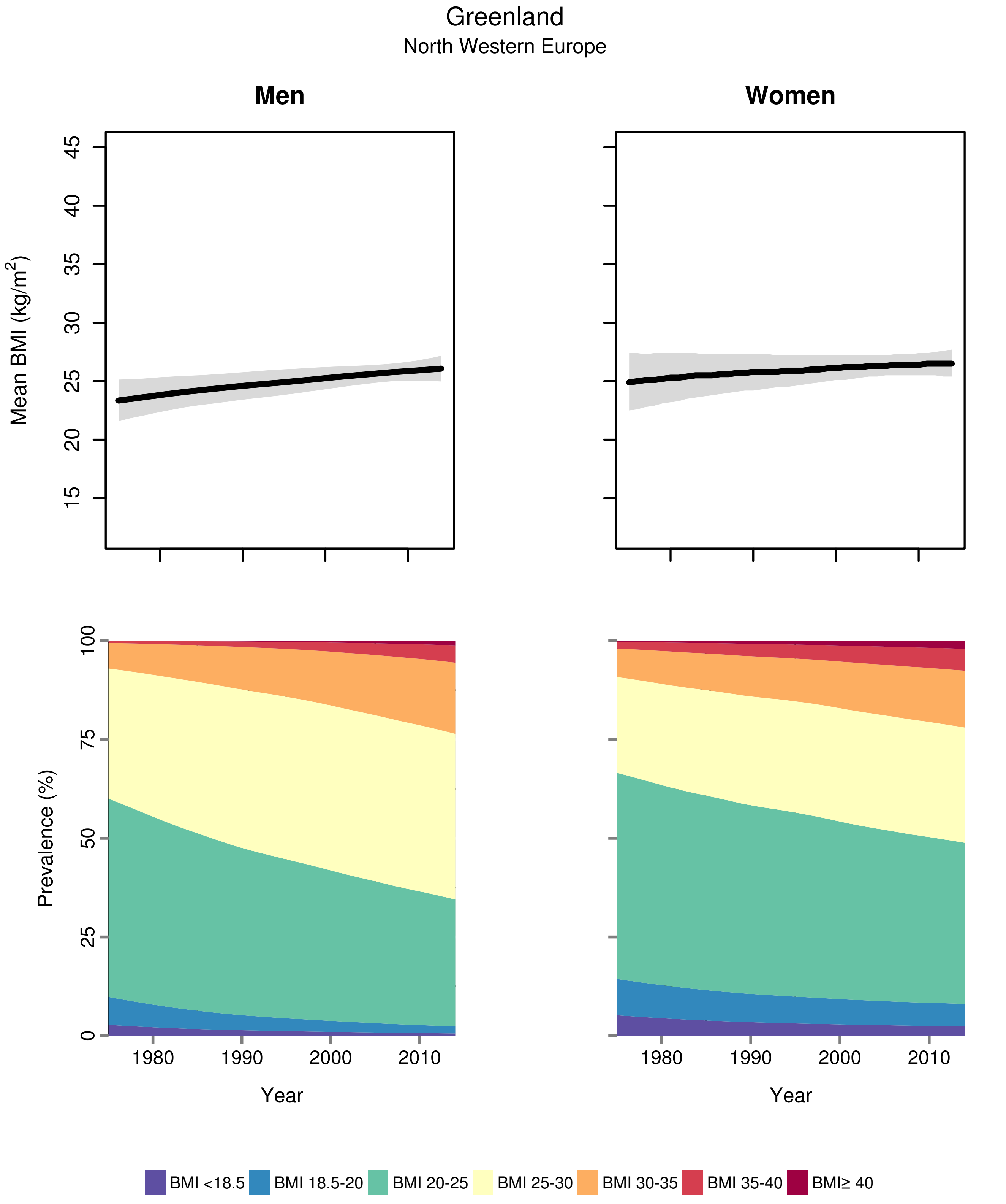
Greenland
North Western Europe
Men
Women
Mean BMI (kg/m2)
45
40
35
30
25
20
15
Prevalence (%)
100
75
50
25
0
1980
1990
2000
2010
Year
BMI <18.5
BMI 18.5-20
BMI 20-25
BMI 25-30
BMI 30-35
BMI 35-40
BMI≥ 40

Grenada
Caribbean

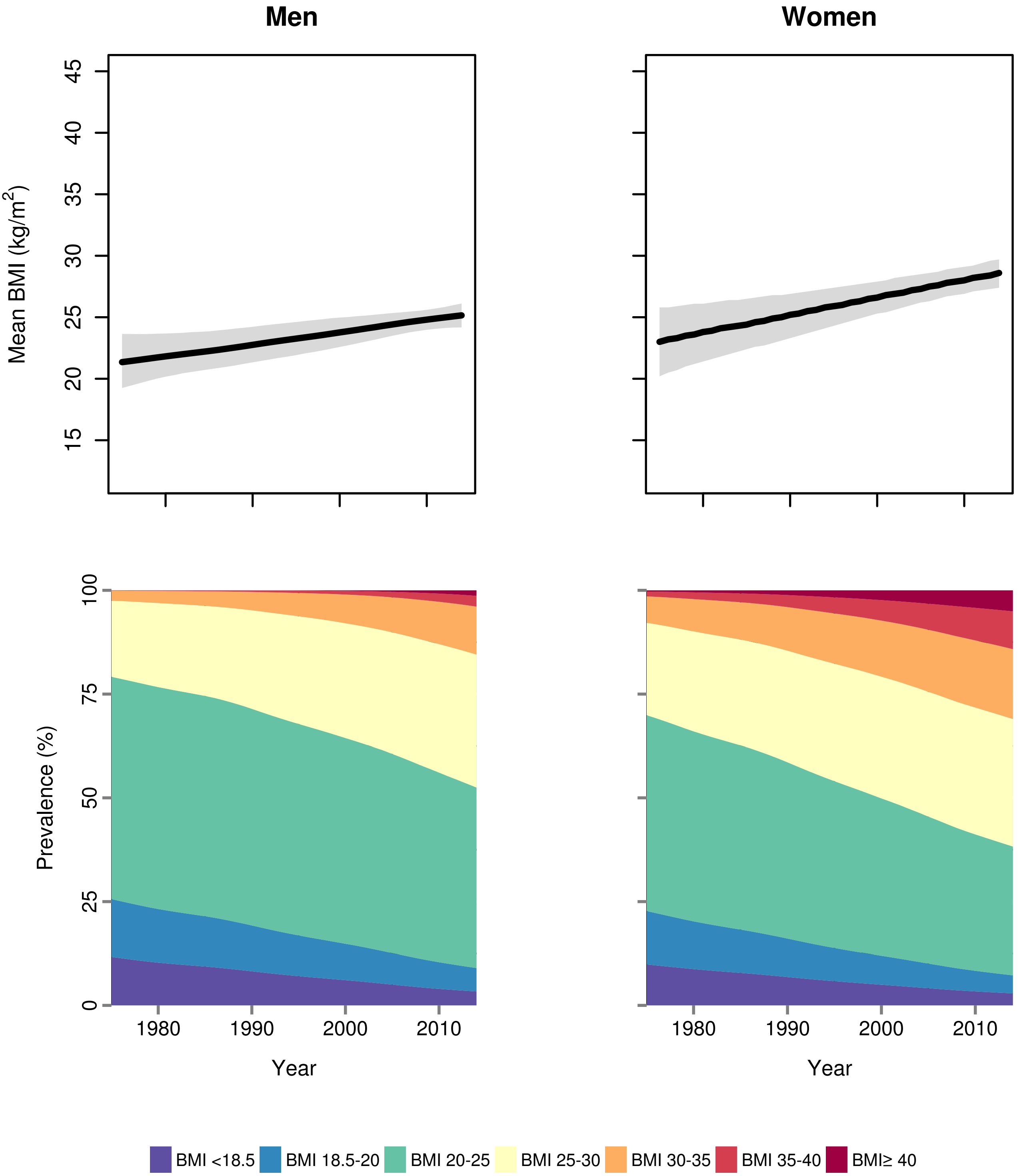

Guatemala

Central Latin America

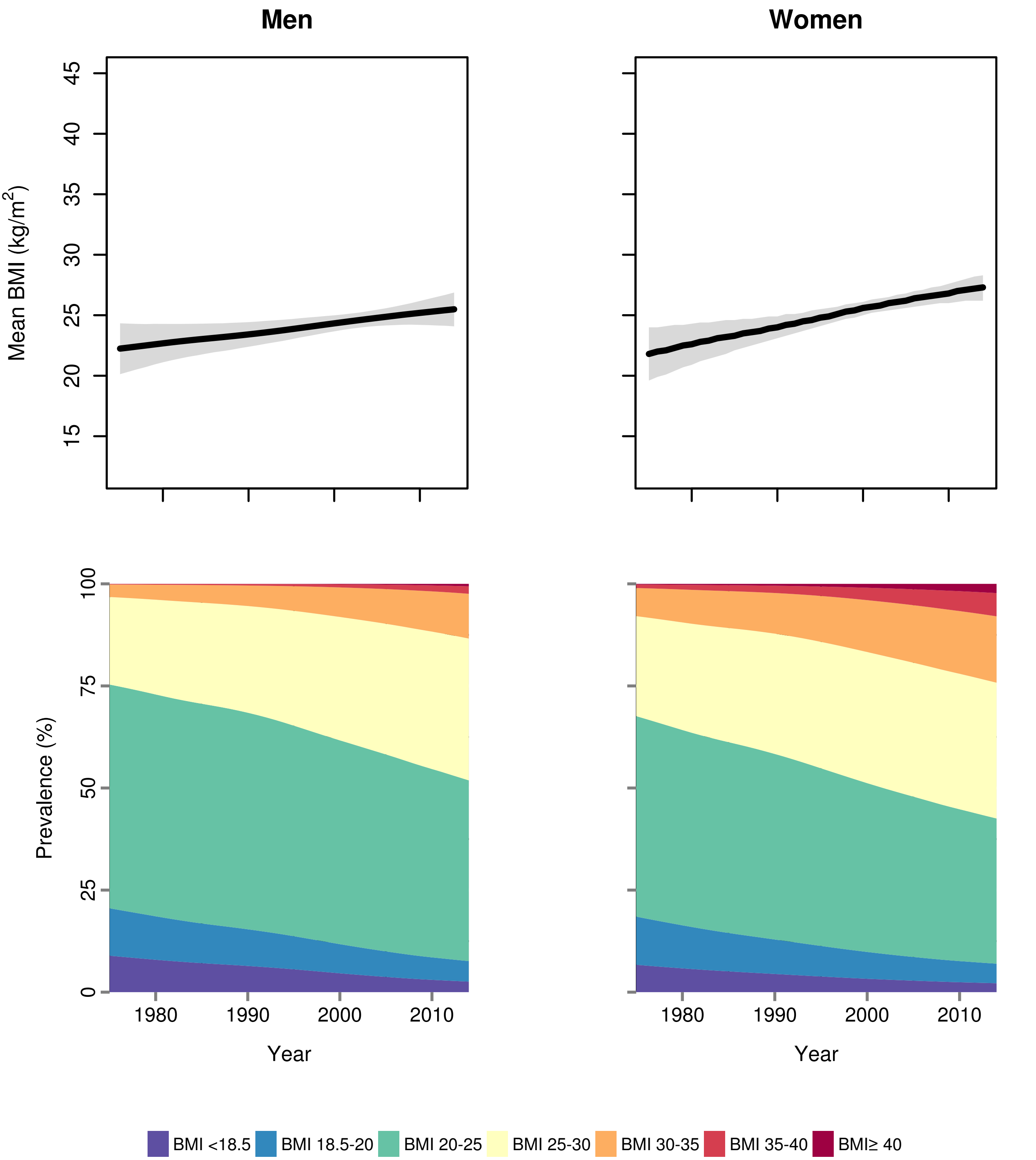

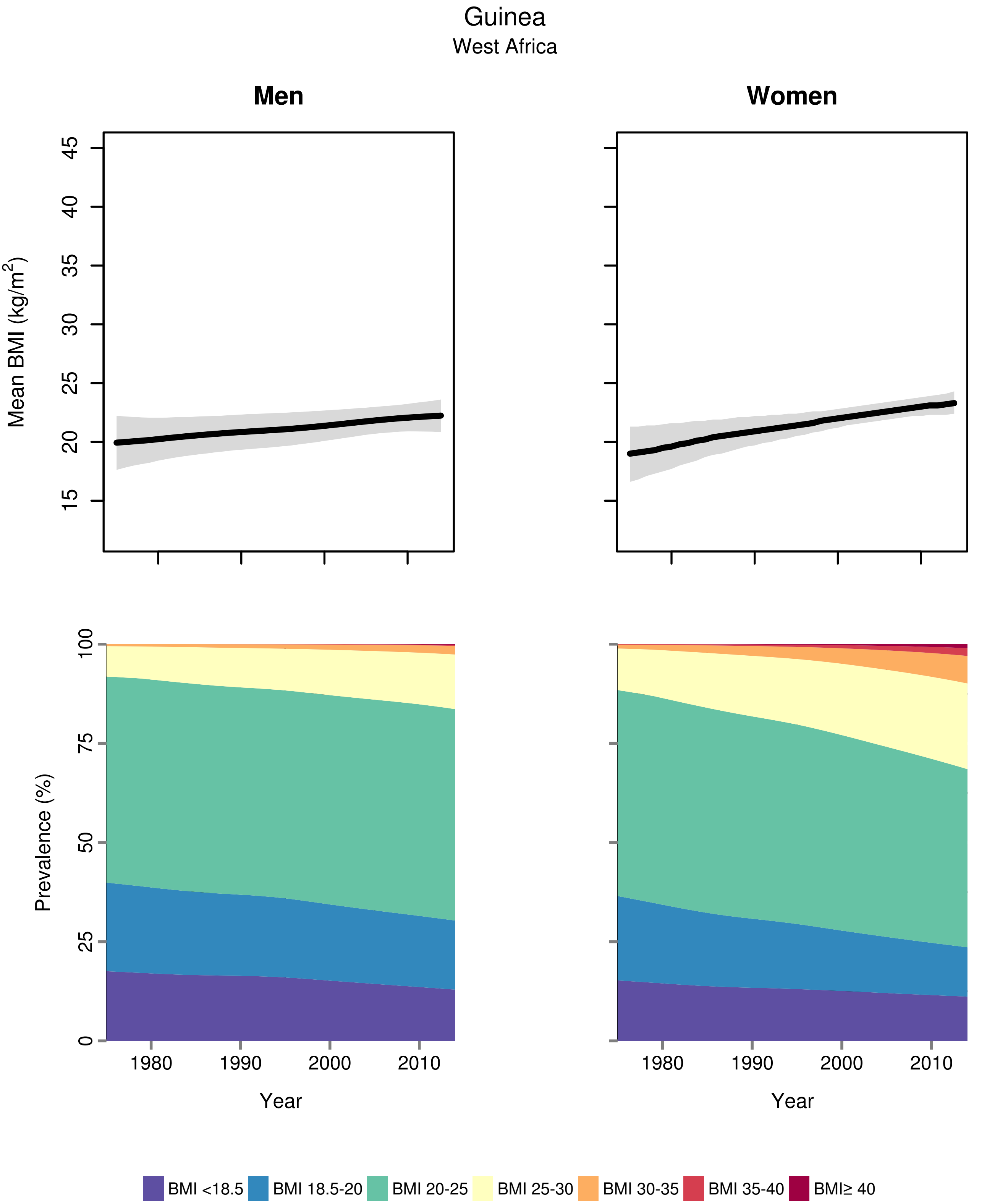

Guinea
West Africa
Men
Women
Mean BMI (kg/m²)
45
40
35
30
25
20
15
Prevalence (%)
100
75
50
25
0
1980
1990
2000
2010
Year
BMI <18.5
BMI 18.5-20
BMI 20-25
BMI 25-30
BMI 30-35
BMI 35-40
BMI≥ 40

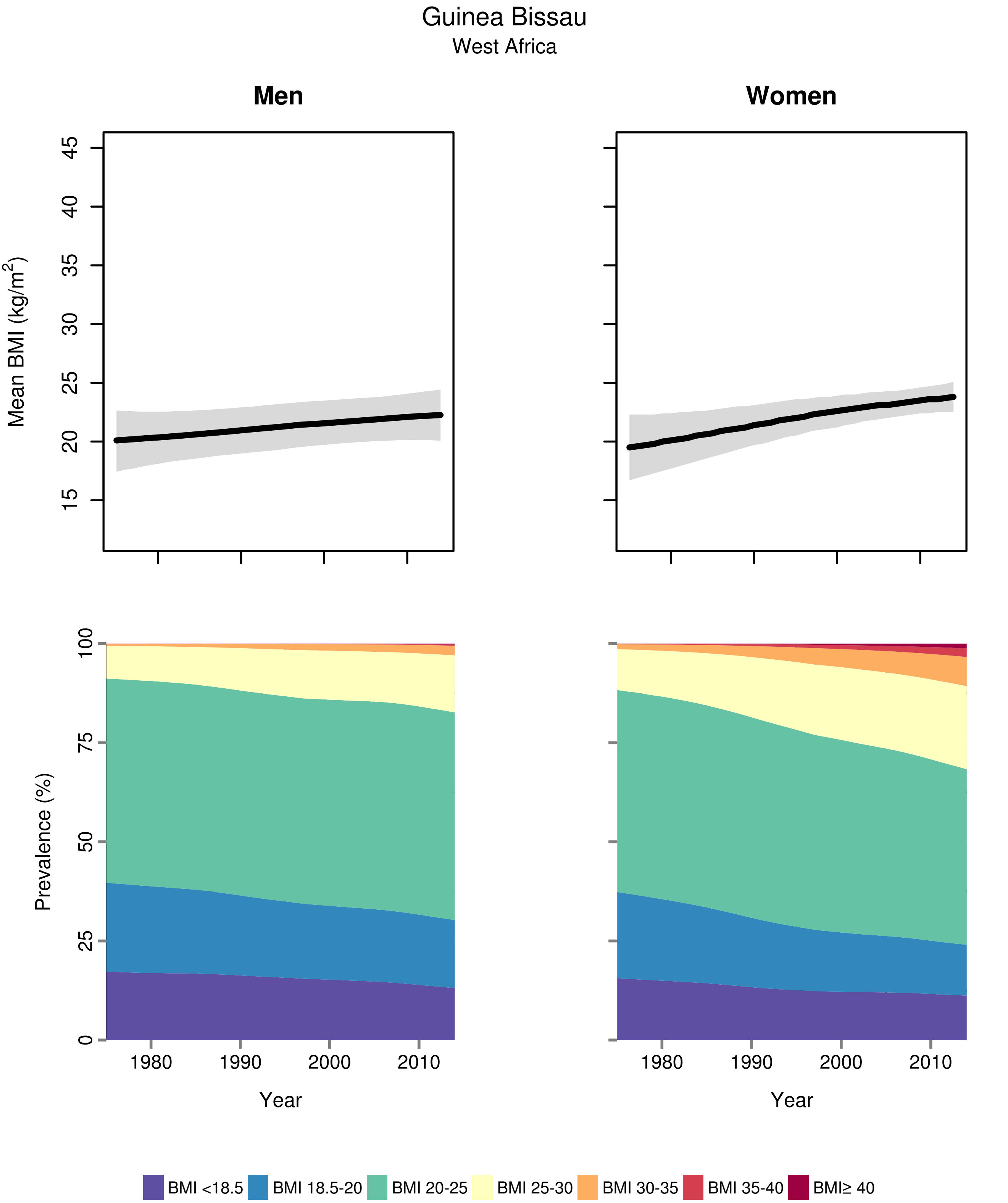

Guinea Bissau
West Africa
Men
Women
Mean BMI (kg/m2)
45
40
35
30
25
20
15
Prevalence (%)
100
75
50
25
0
1980
1990
2000
2010
Year
BMI <18.5
BMI 18.5-20
BMI 20-25
BMI 25-30
BMI 30-35
BMI 35-40
BMI≥ 40

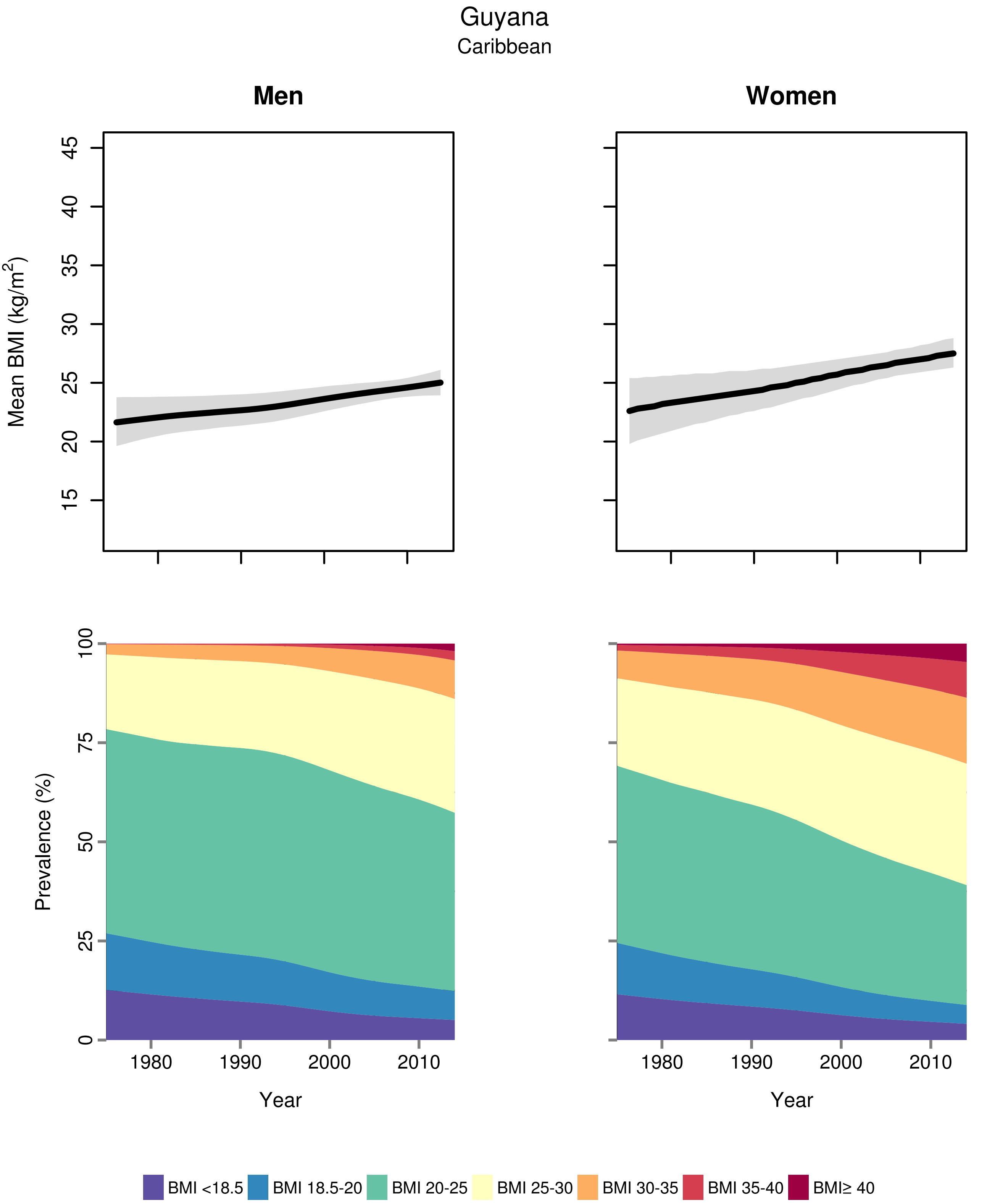
Guyana
Caribbean
Men
Women
Mean BMI (kg/m2)
45
40
35
30
25
20
15
Prevalence (%)
100
75
50
25
0
1980
1990
2000
2010
Year
BMI <18.5
BMI 18.5-20
BMI 20-25
BMI 25-30
BMI 30-35
BMI 35-40
BMI≥ 40

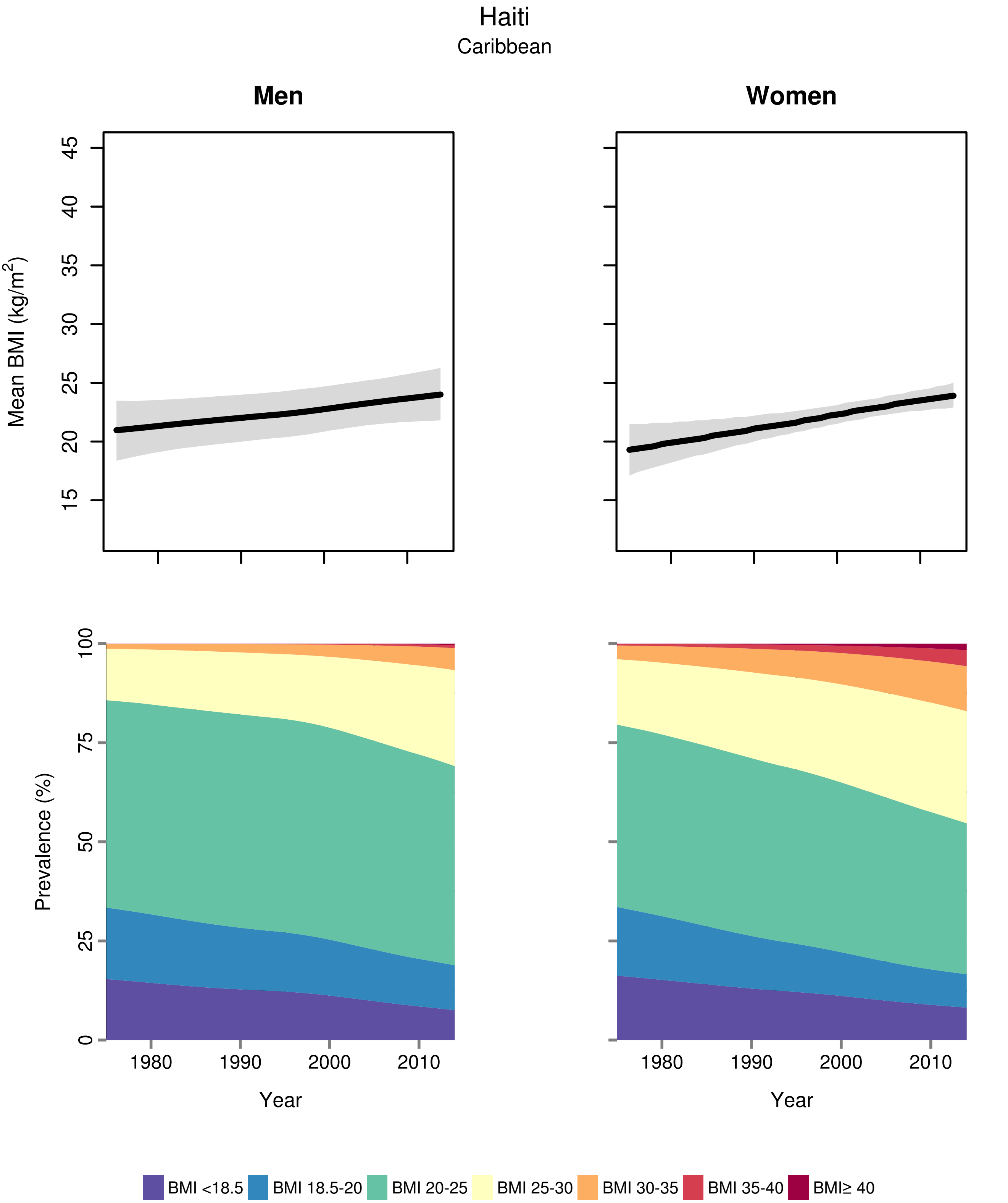

Haiti
Caribbean
Men
Women
Mean BMI (kg/m2)
45
40
35
30
25
20
15
Prevalence (%)
100
75
50
25
0
1980
1990
2000
2010
Year
BMI <18.5
BMI 18.5-20
BMI 20-25
BMI 25-30
BMI 30-35
BMI 35-40
BMI≥ 40

Honduras

Central Latin America

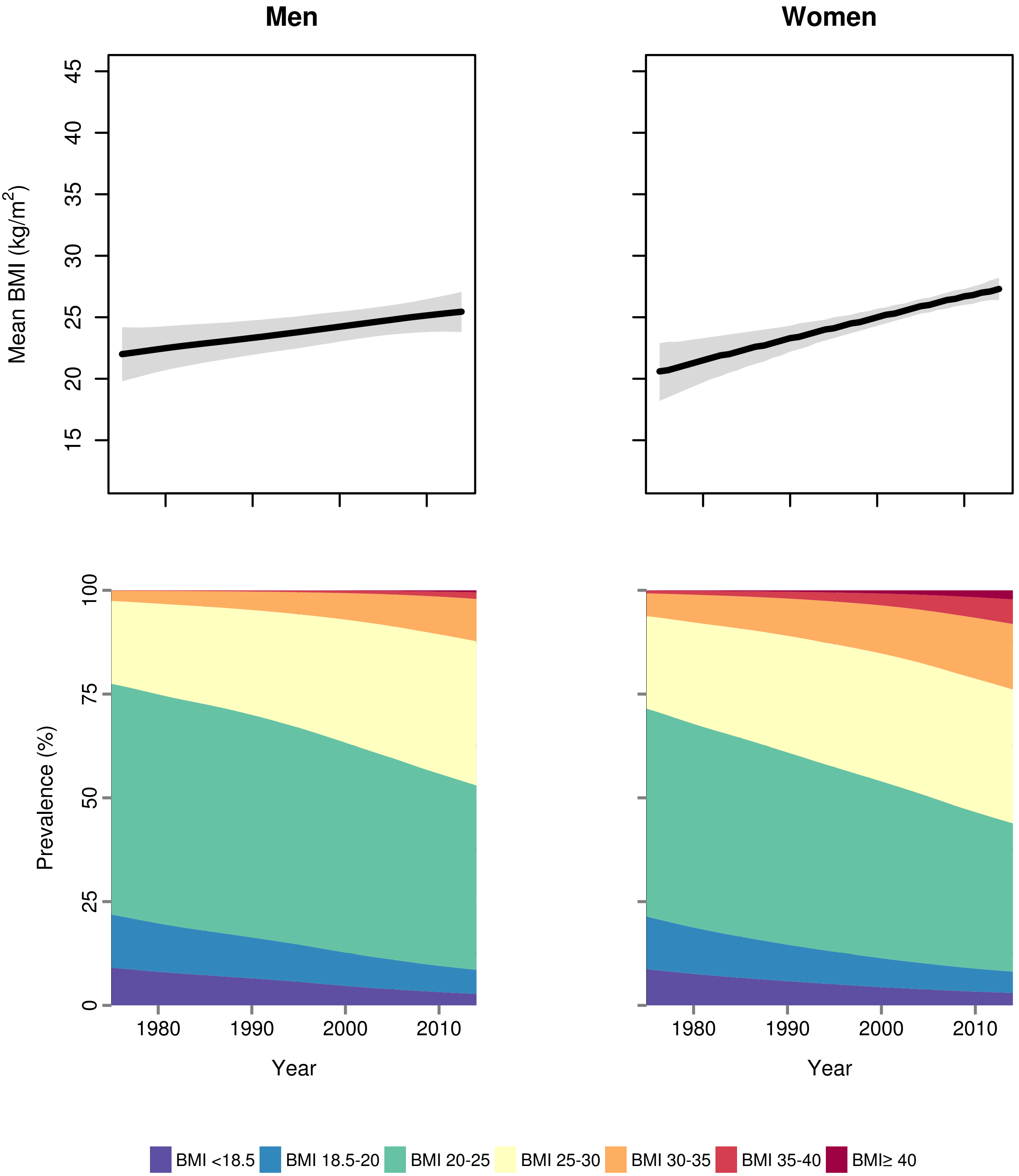

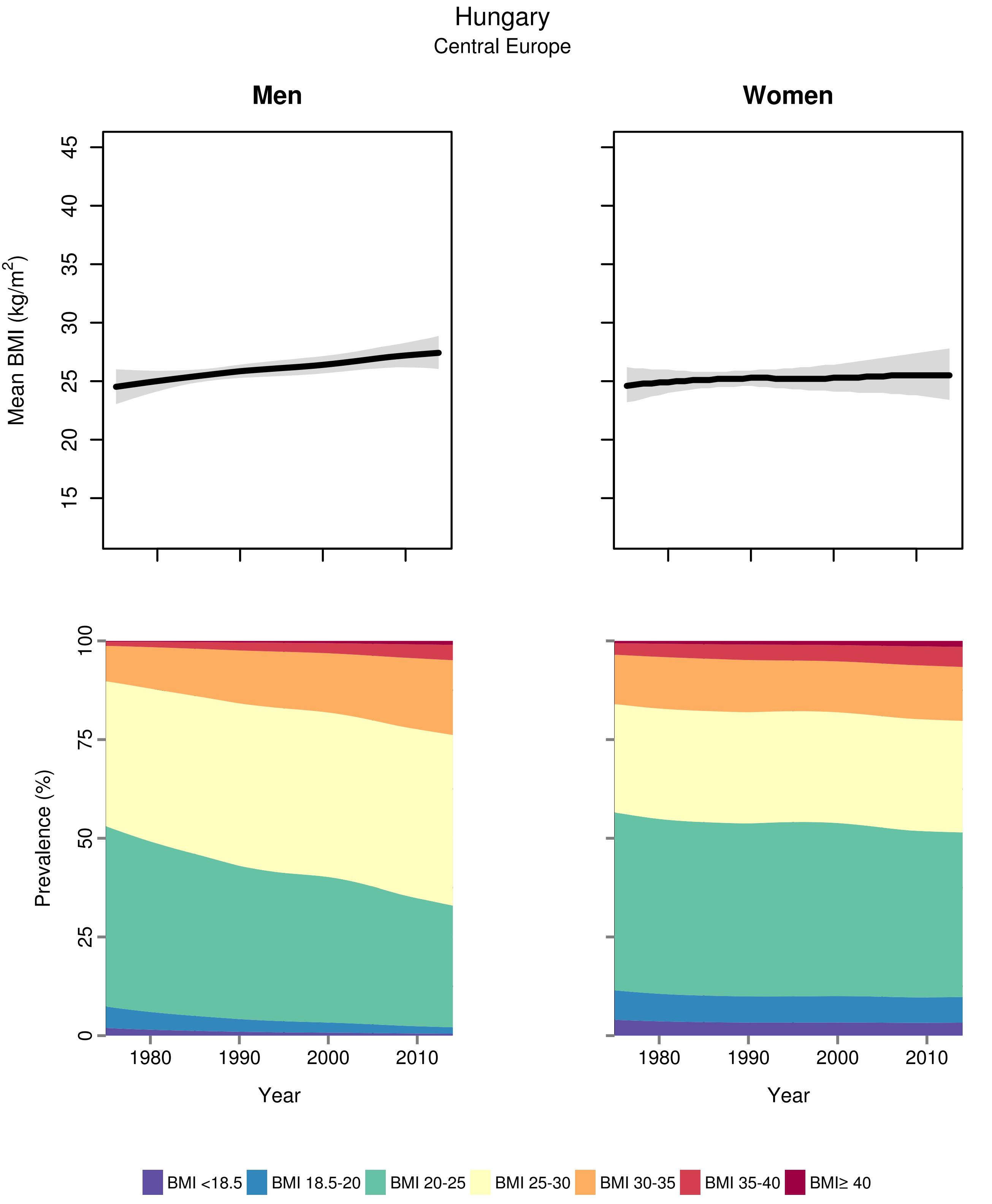

Hungary
Central Europe
Men
Women
Mean BMI (kg/m2)
15
20
25
30
35
40
45
Prevalence (%)
0
25
50
75
100
1980
1990
2000
2010
Year
BMI <18.5
BMI 18.5-20
BMI 20-25
BMI 25-30
BMI 30-35
BMI 35-40
BMI≥ 40

Iceland

North Western Europe

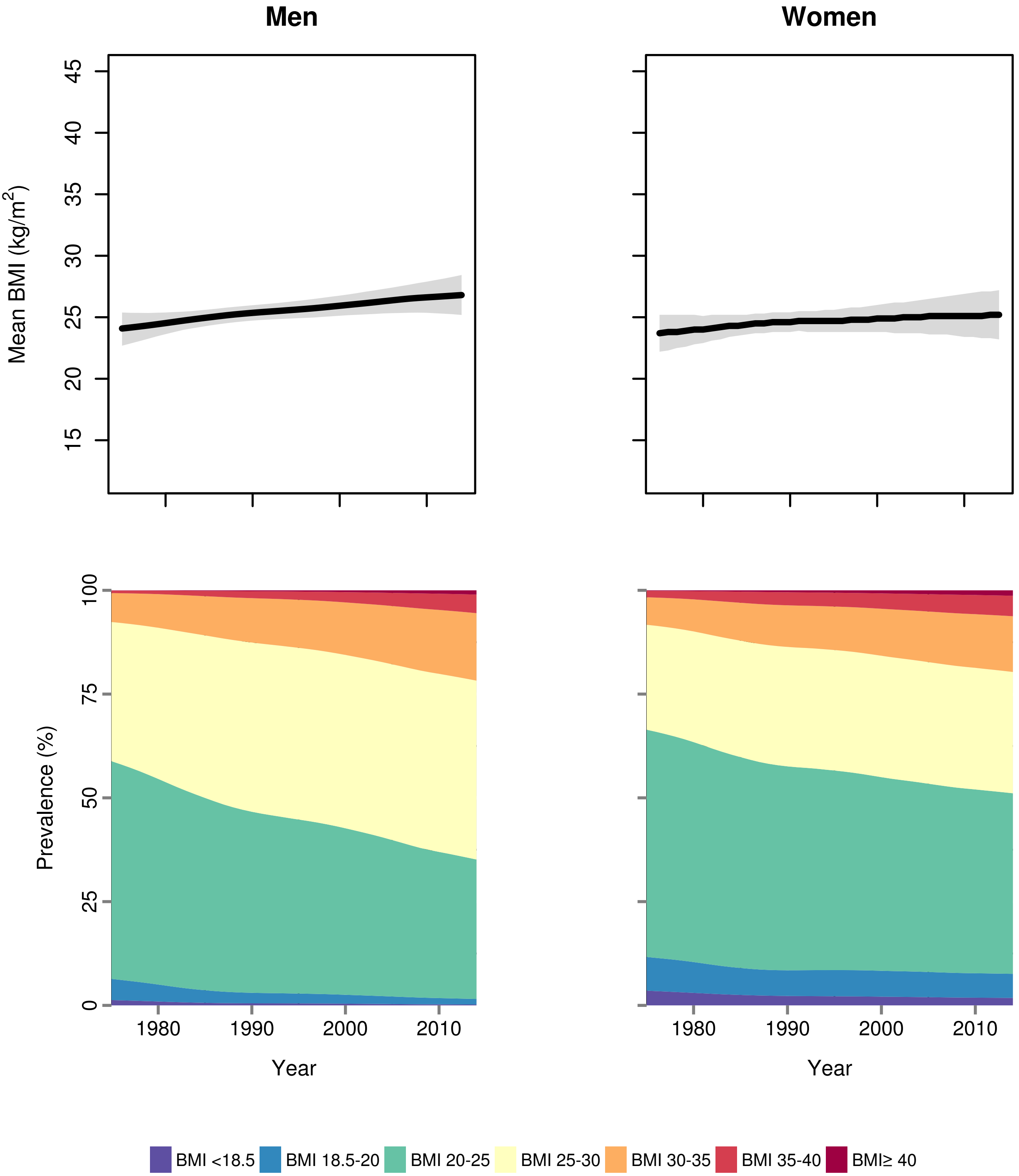

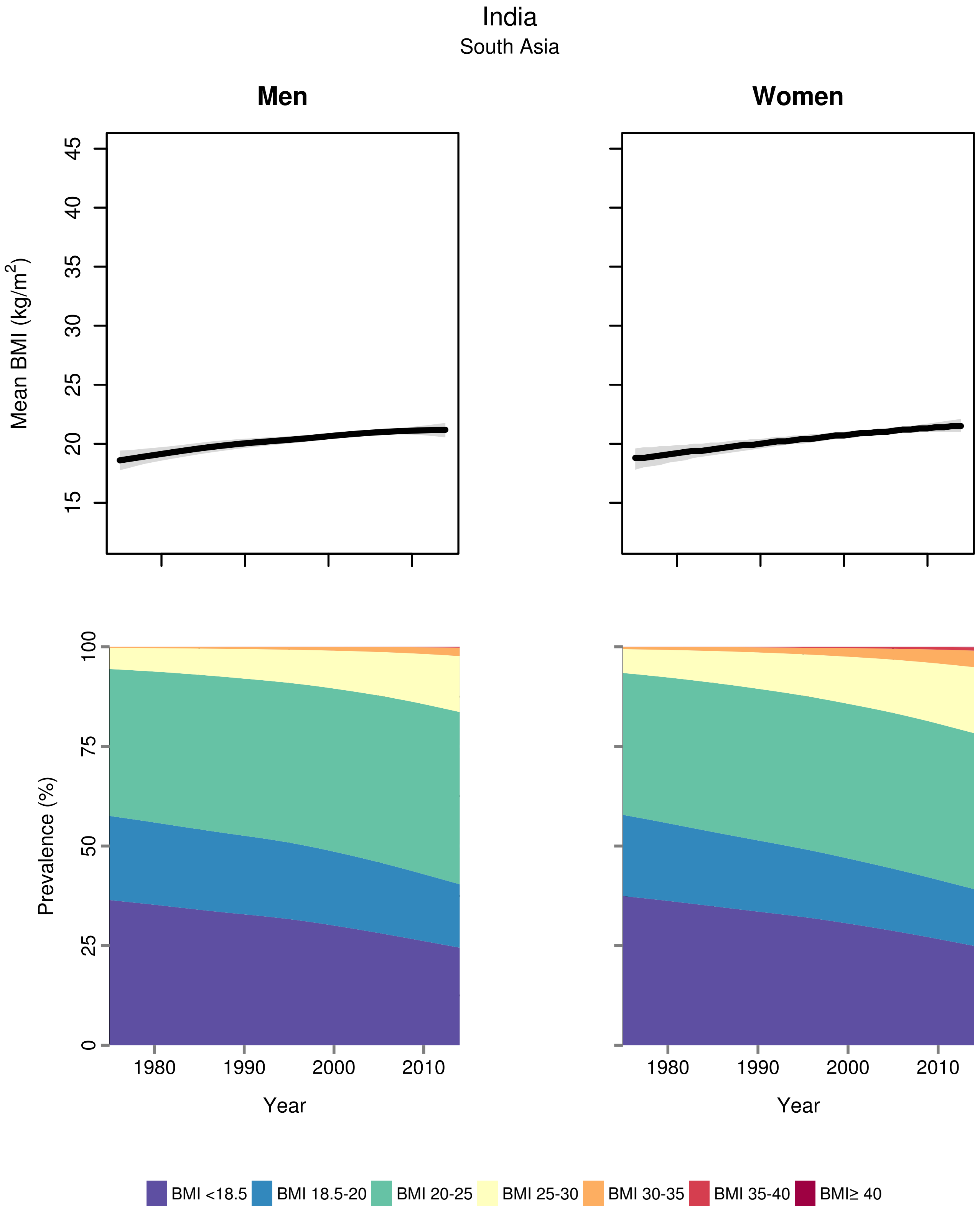
India
South Asia
Men
Women
Mean BMI (kg/m2)
45
40
35
30
25
20
15
Prevalence (%)
100
75
50
25
0
1980
1990
2000
2010
Year
BMI <18.5
BMI 18.5-20
BMI 20-25
BMI 25-30
BMI 30-35
BMI 35-40
BMI≥ 40

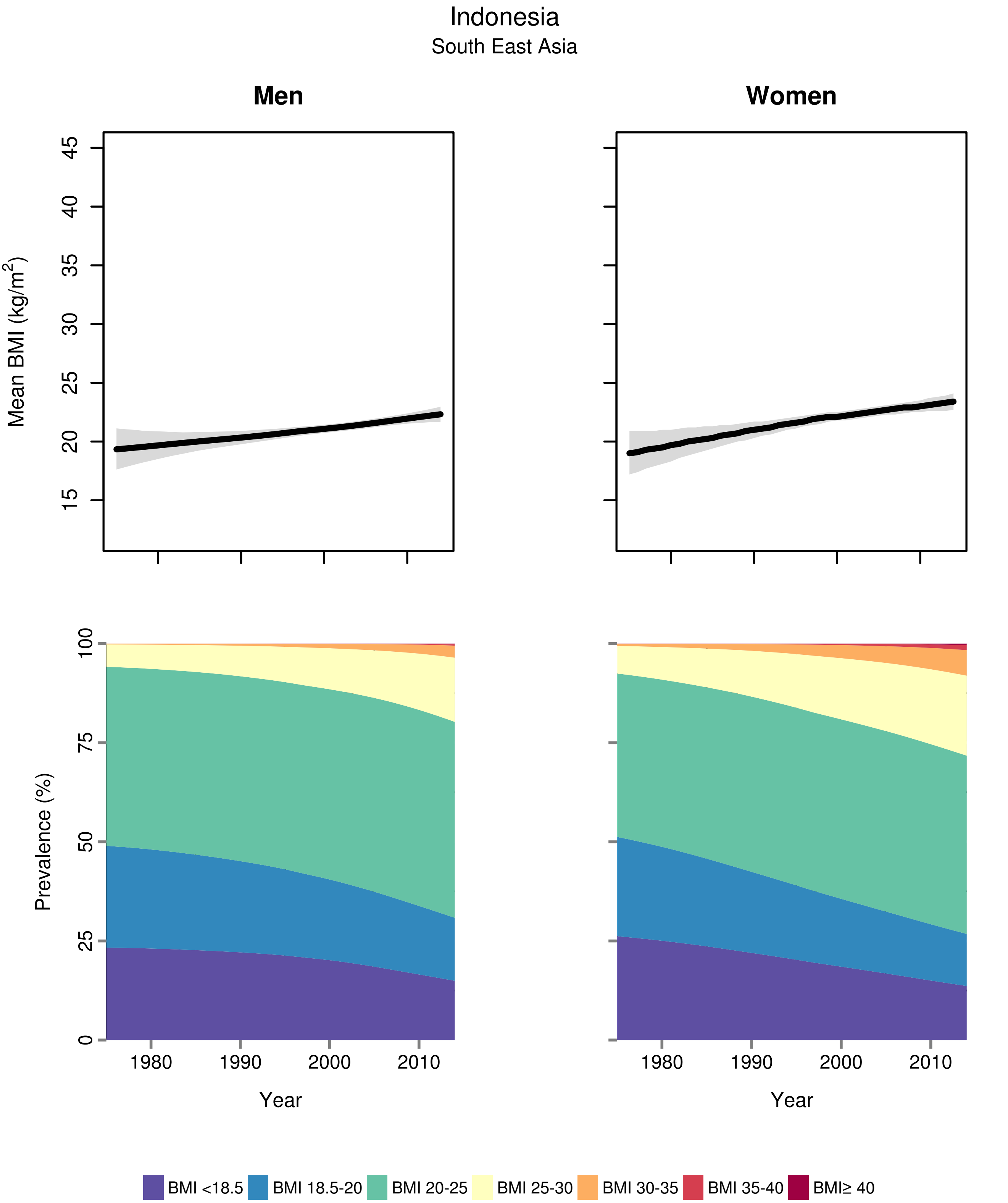
Indonesia
South East Asia
Men
Women
Mean BMI (kg/m2)
45
40
35
30
25
20
15
Prevalence (%)
100
75
50
25
0
1980
1990
2000
2010
Year
BMI <18.5
BMI 18.5-20
BMI 20-25
BMI 25-30
BMI 30-35
BMI 35-40
BMI≥ 40

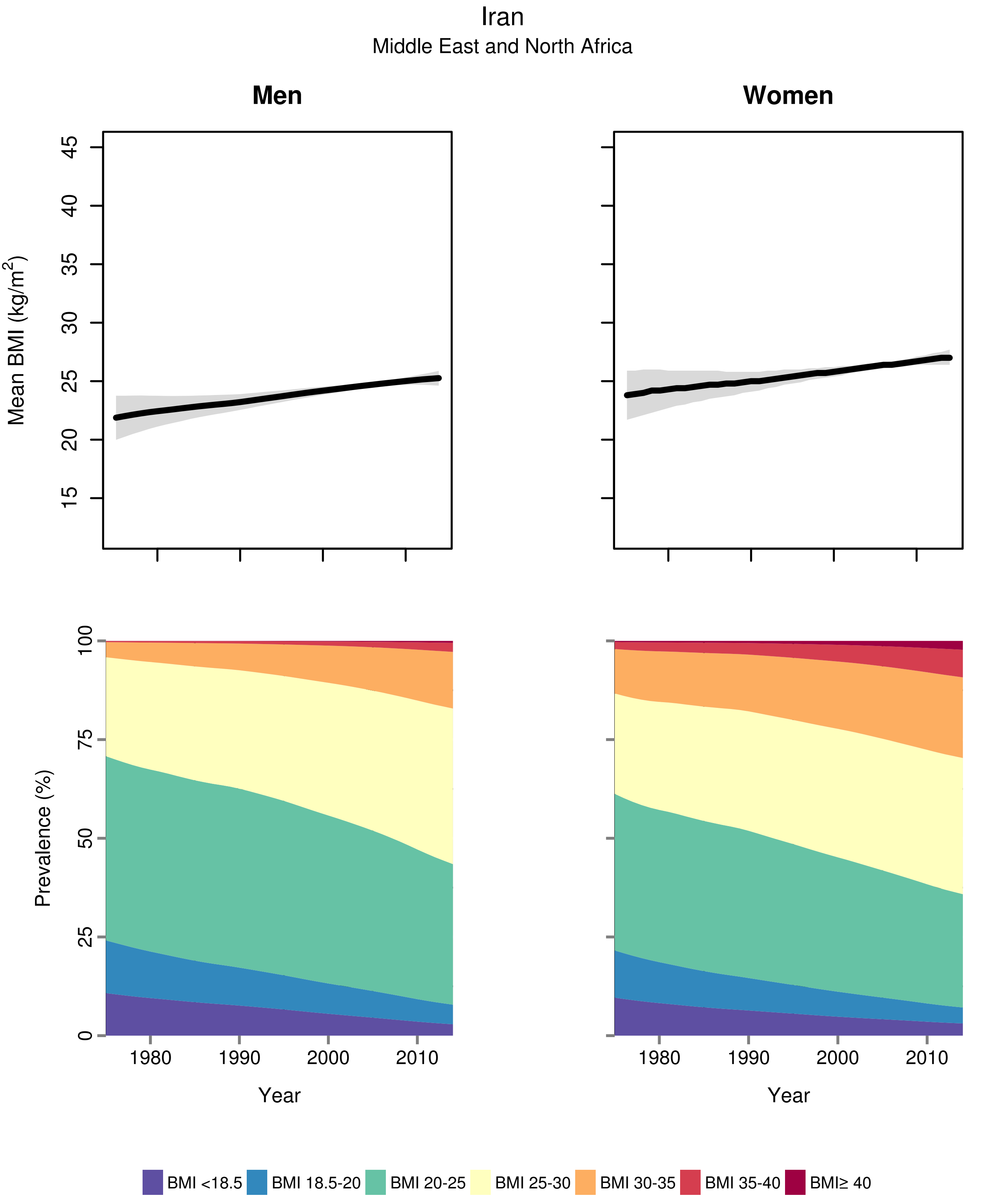
Iran
Middle East and North Africa
Men
Women
Mean BMI (kg/m²)
45
40
35
30
25
20
15
Prevalence (%)
100
75
50
25
0
1980
1990
2000
2010
Year
BMI <18.5
BMI 18.5-20
BMI 20-25
BMI 25-30
BMI 30-35
BMI 35-40
BMI≥ 40

Iraq

Middle East and North Africa

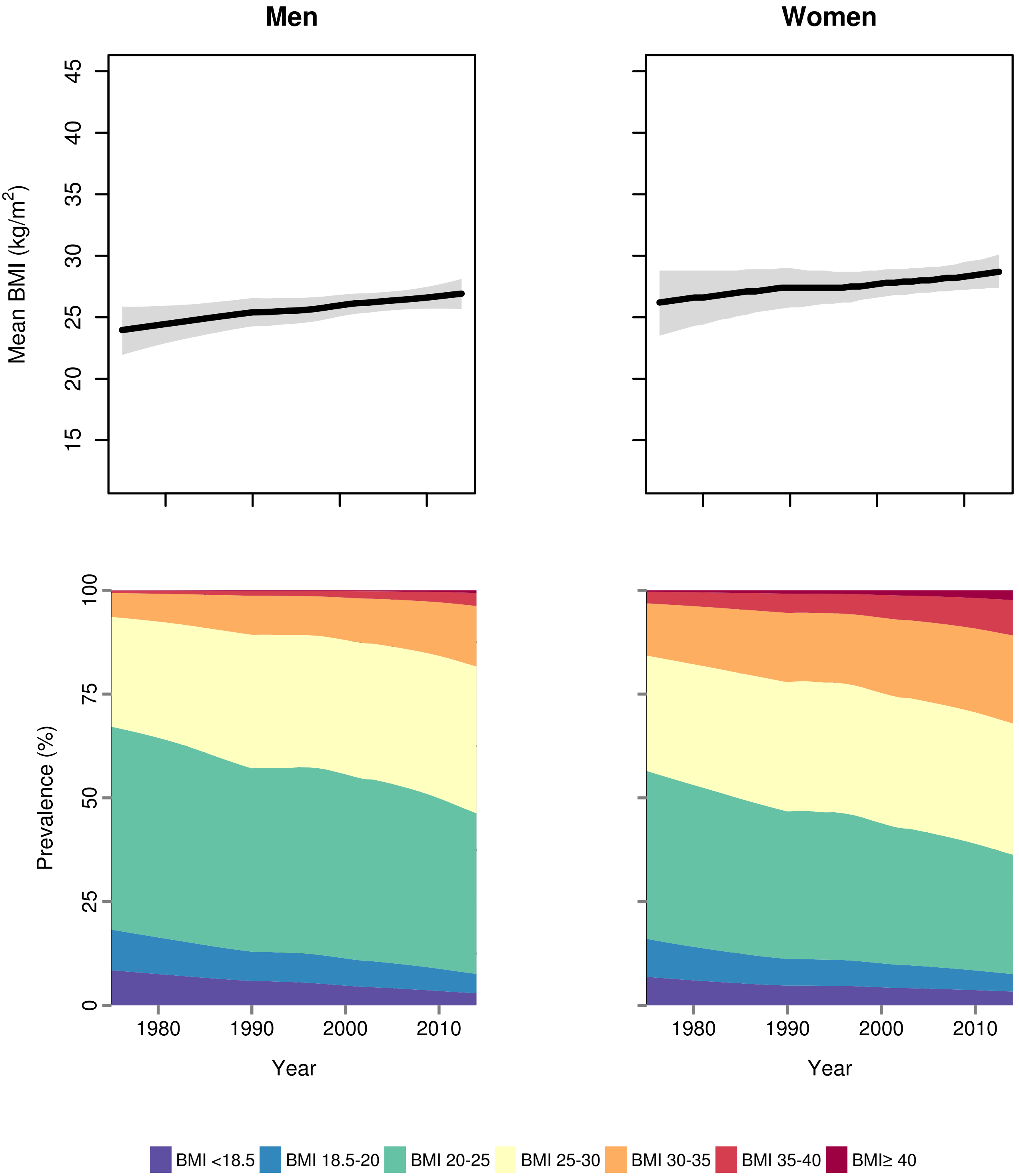

Ireland

High-income English-speaking countries

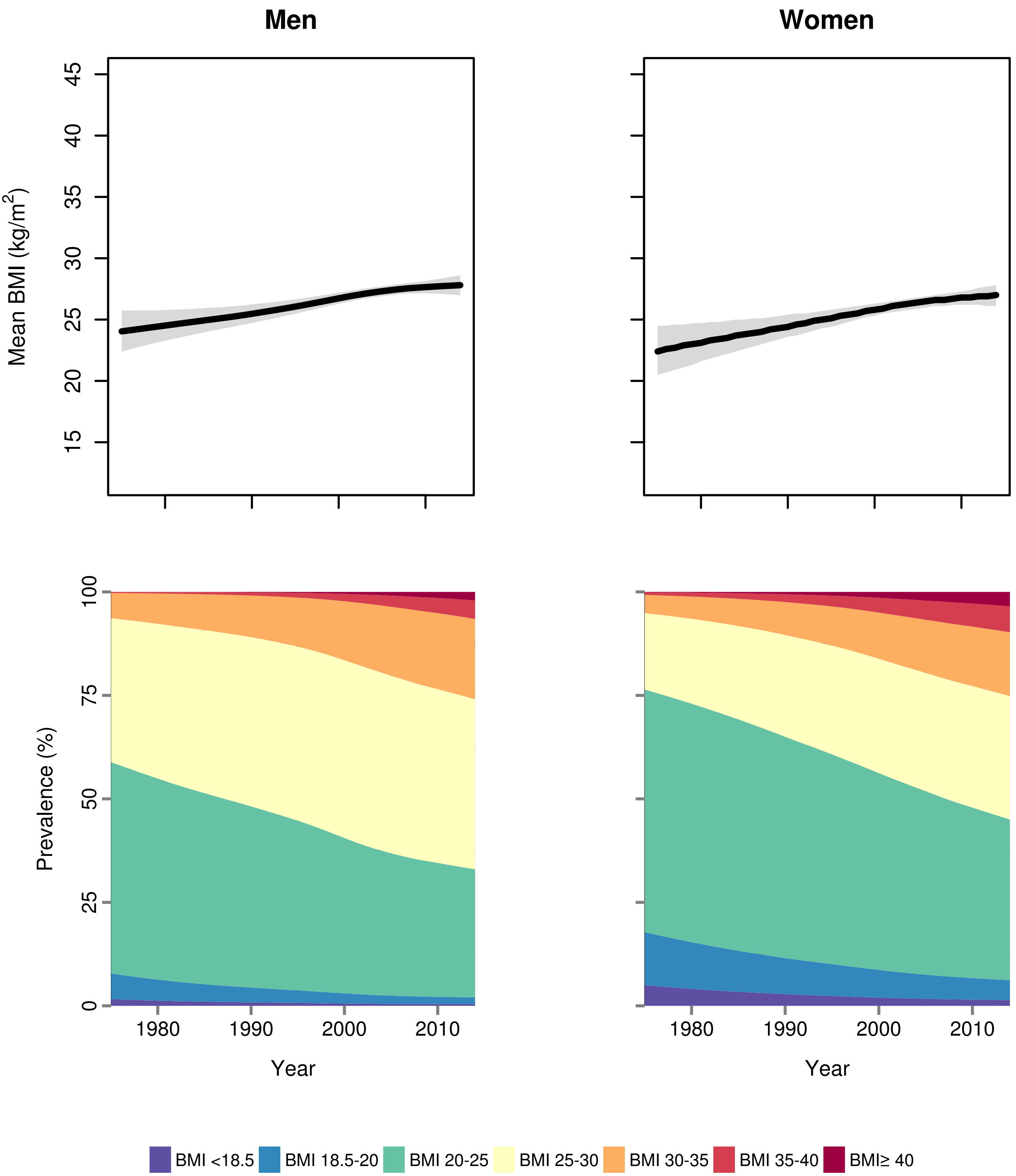

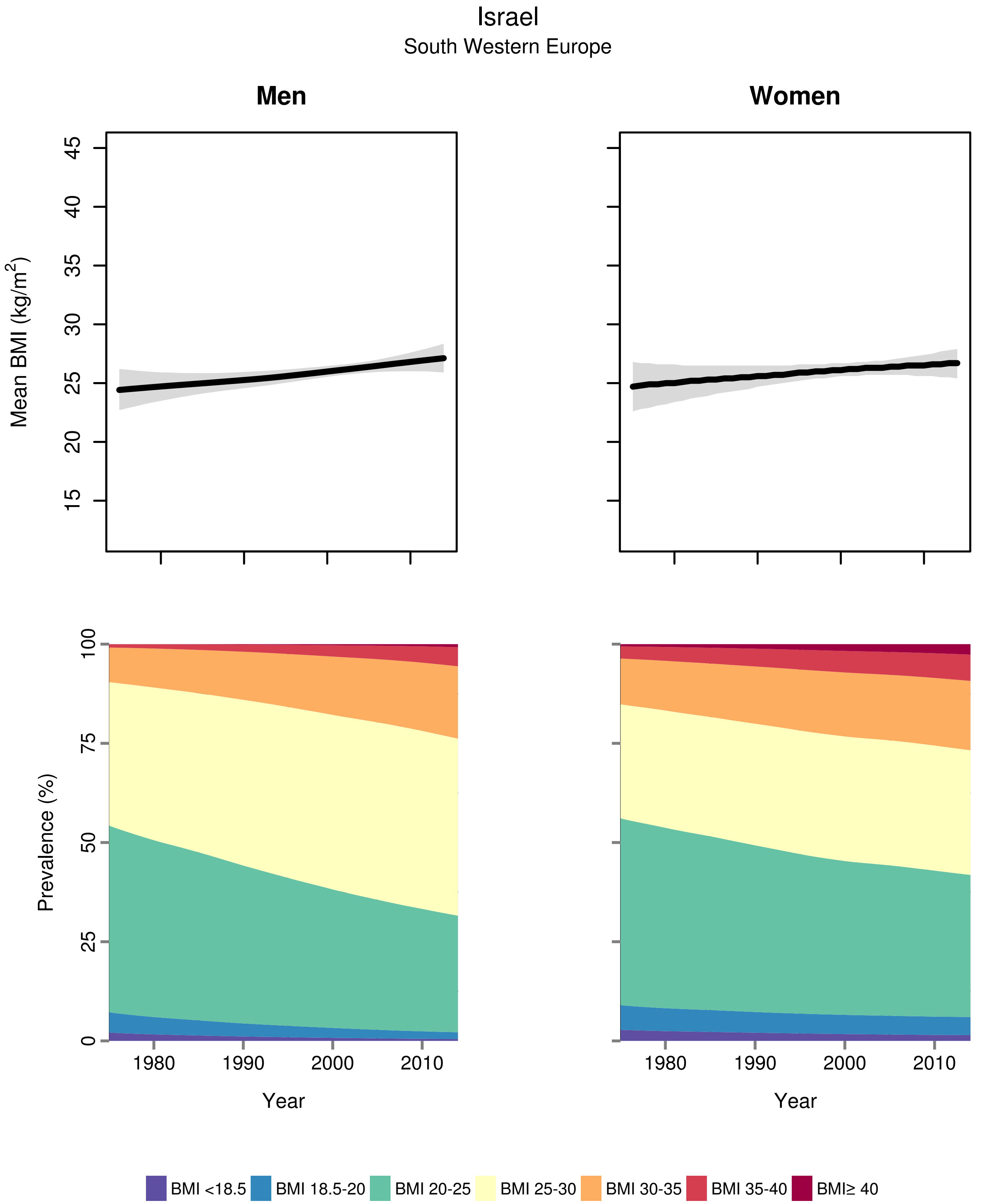
Israel
South Western Europe
Men
Women
Mean BMI (kg/m2)
45
40
35
30
25
20
15
Prevalence (%)
100
75
50
25
0
1980
1990
2000
2010
Year
BMI <18.5
BMI 18.5-20
BMI 20-25
BMI 25-30
BMI 30-35
BMI 35-40
BMI≥ 40

Italy

South Western Europe

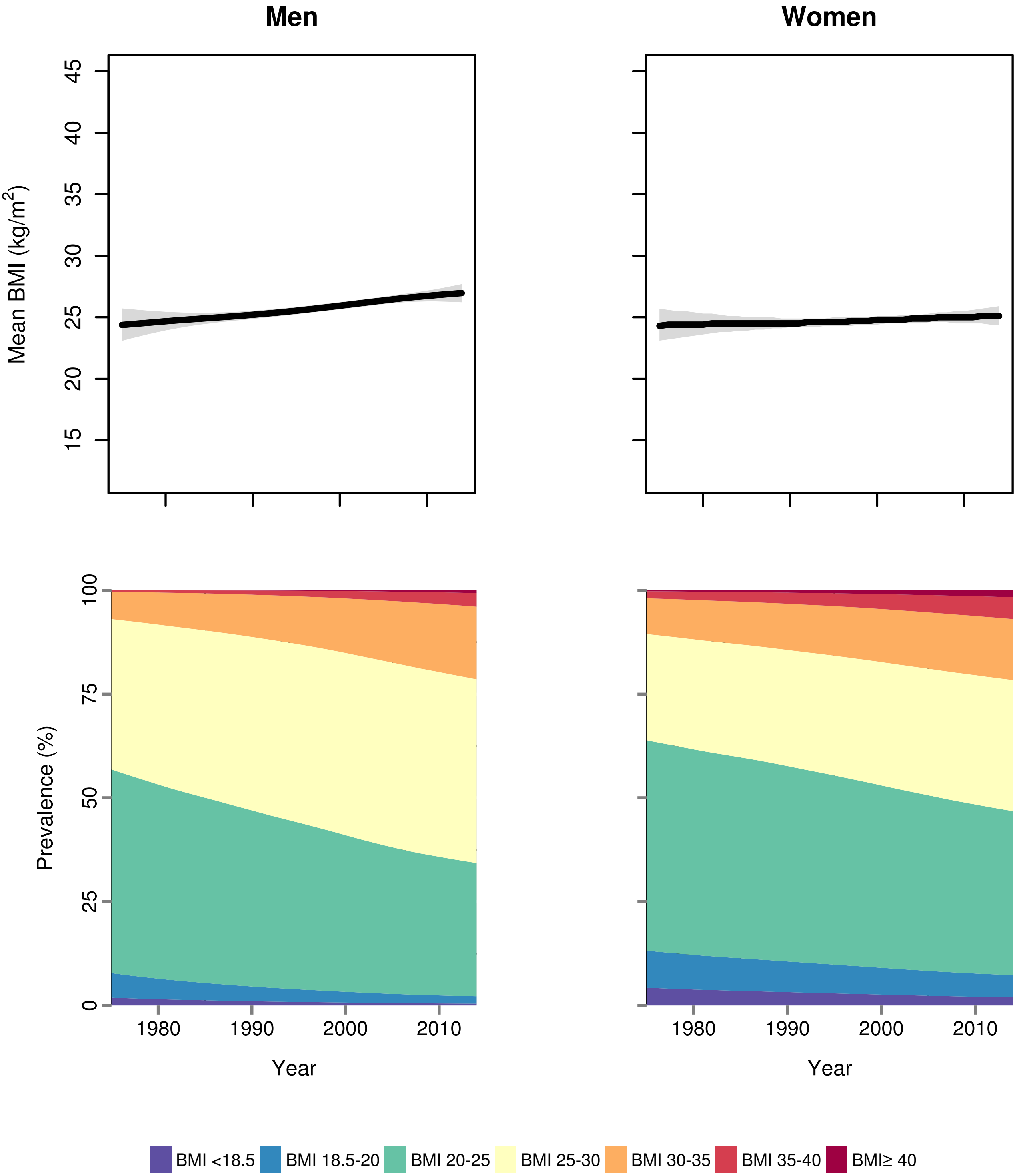

Jamaica

Caribbean

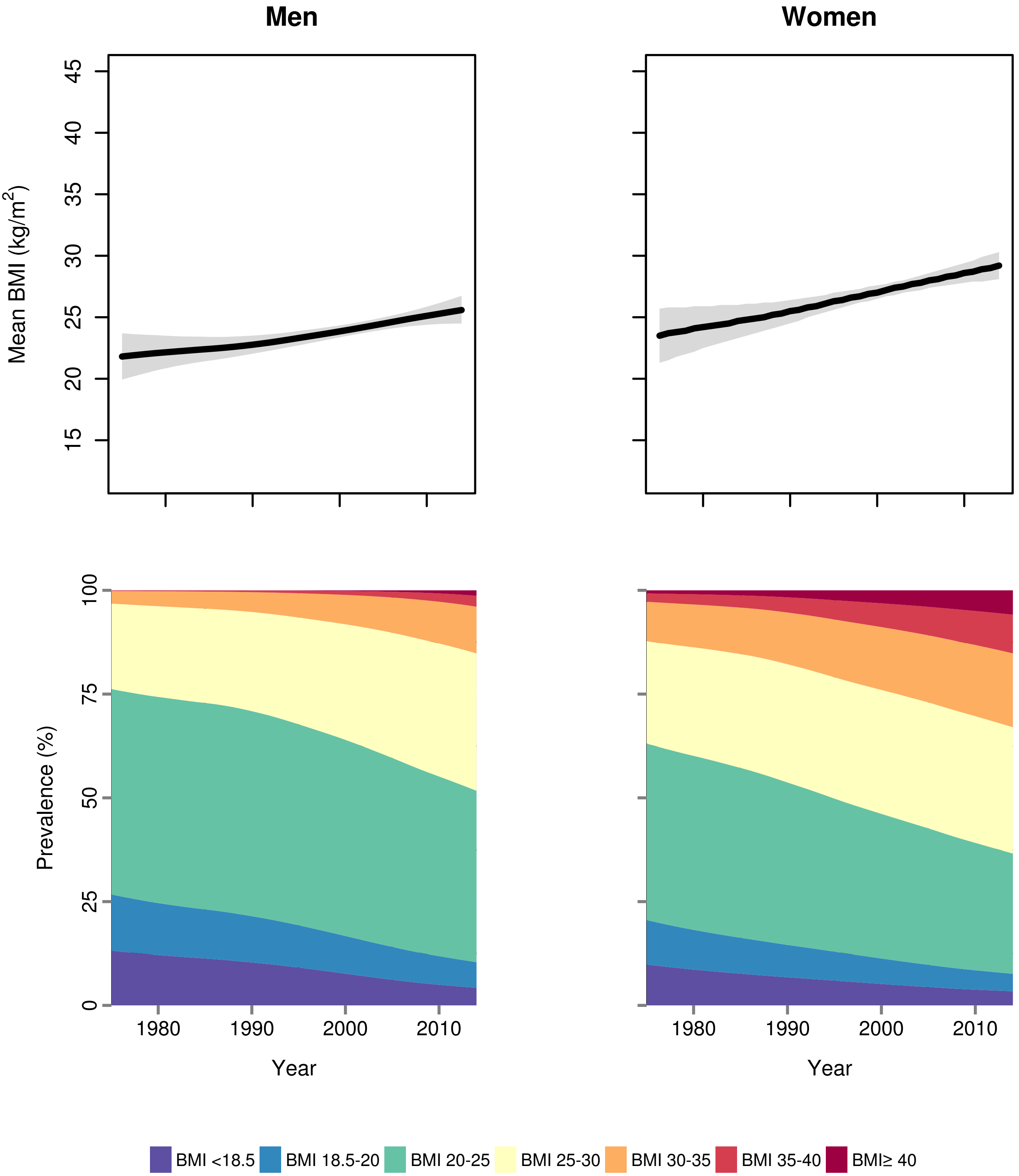

Japan

High-income Asia Pacific

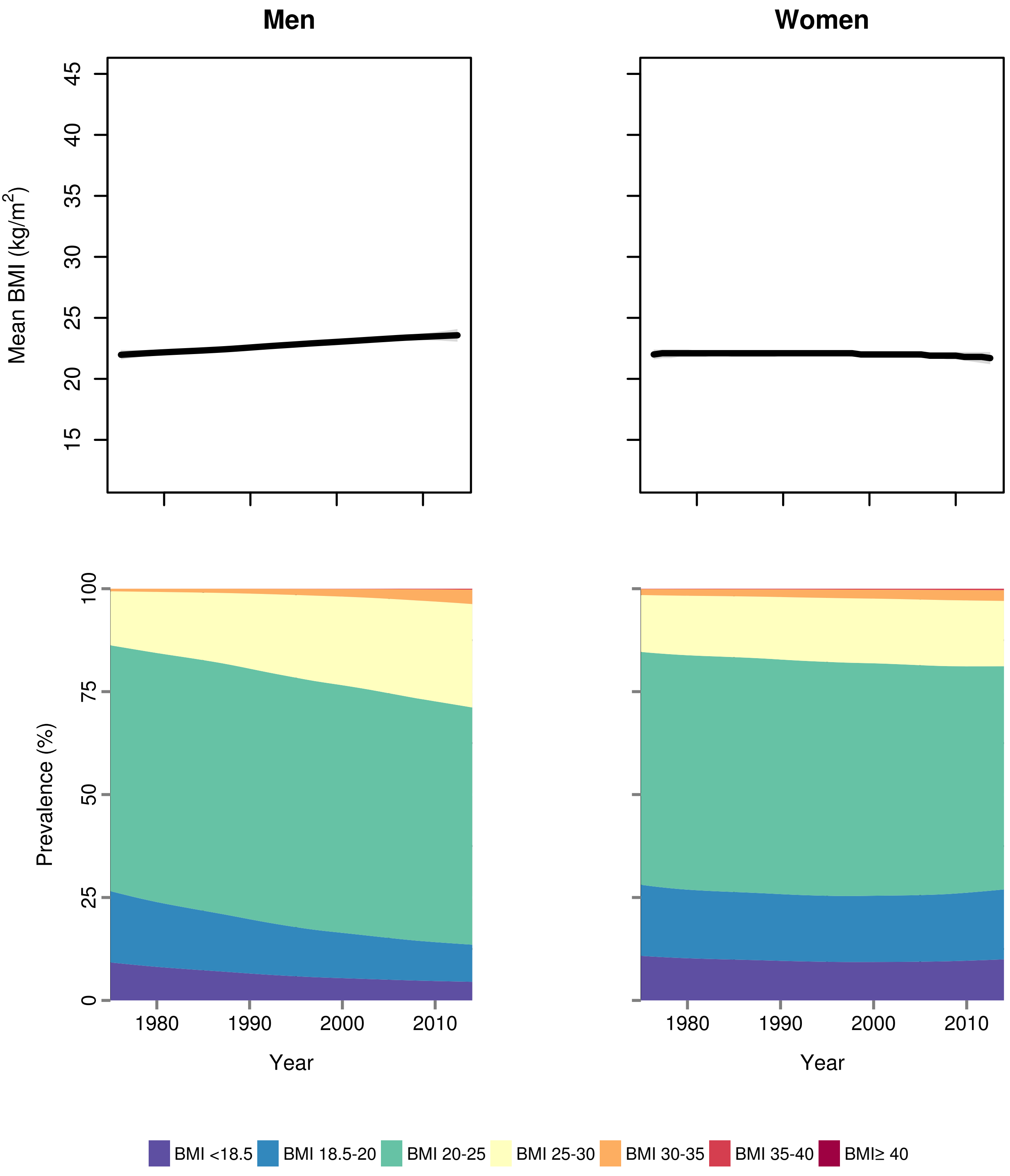

Jordan

Middle East and North Africa

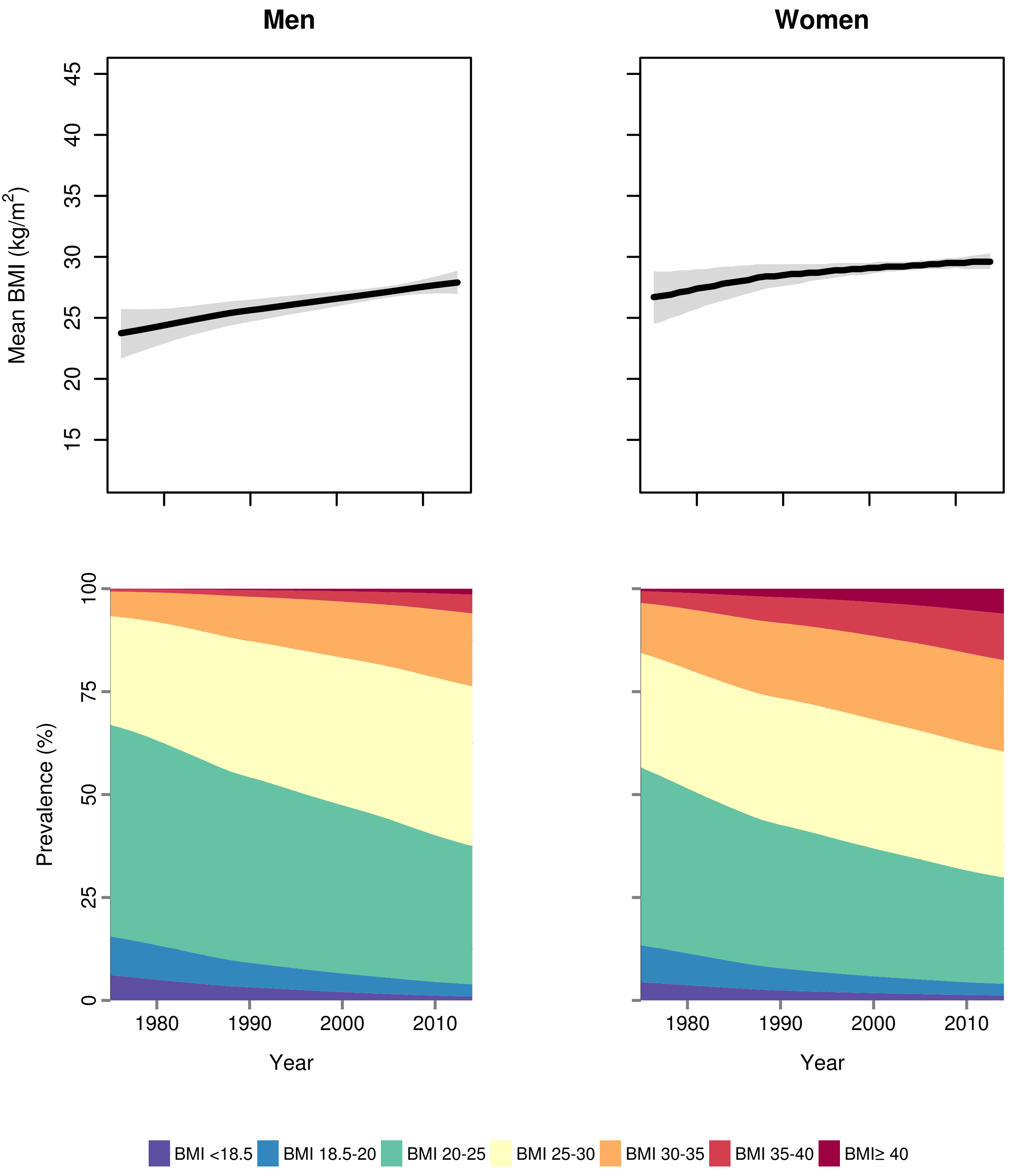

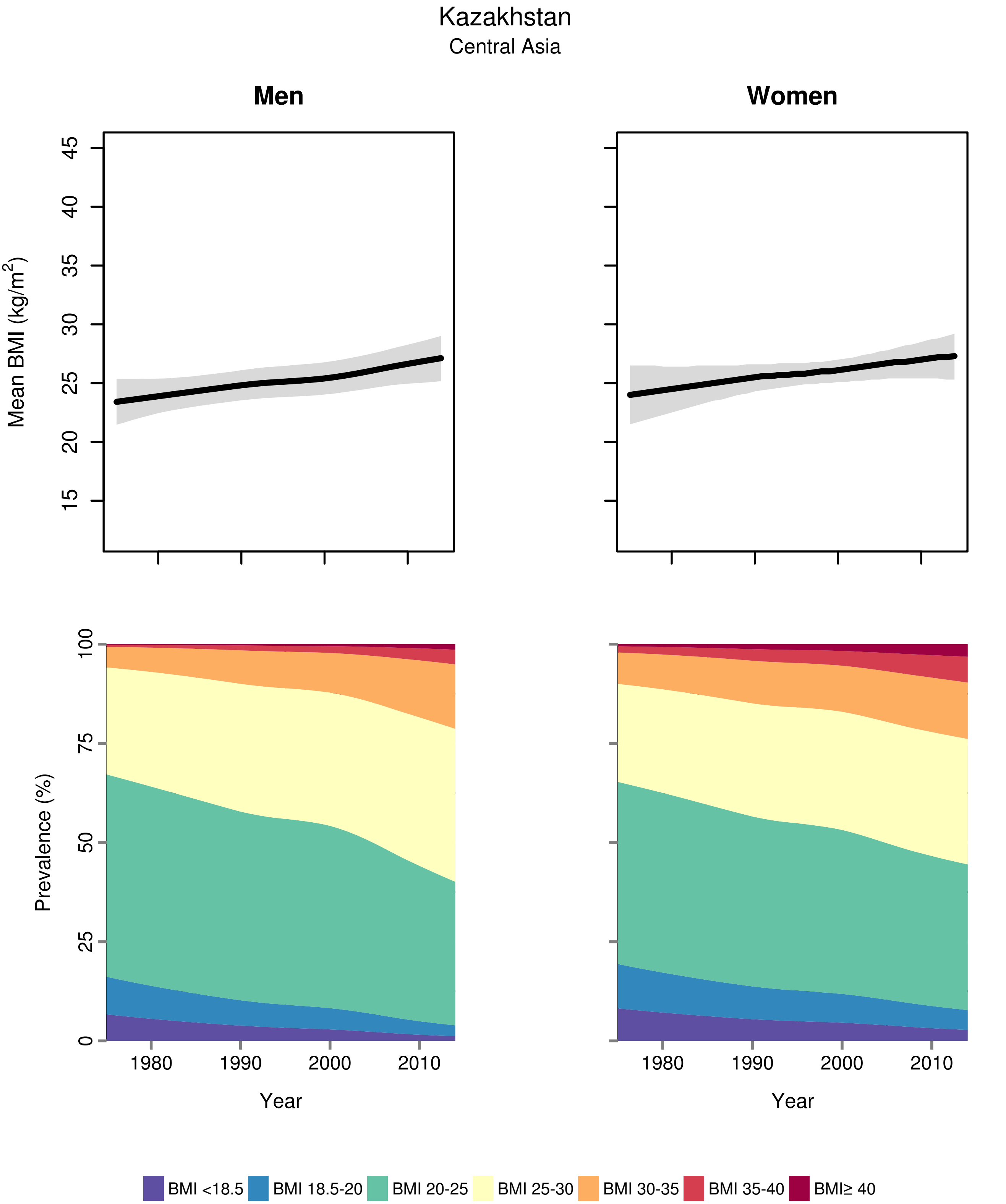

Kazakhstan
Central Asia
Men
Women
Mean BMI (kg/m2)
45
40
35
30
25
20
15
Prevalence (%)
100
75
50
25
0
1980
1990
2000
2010
Year
BMI <18.5
BMI 18.5-20
BMI 20-25
BMI 25-30
BMI 30-35
BMI 35-40
BMI≥ 40

Kenya
East Africa

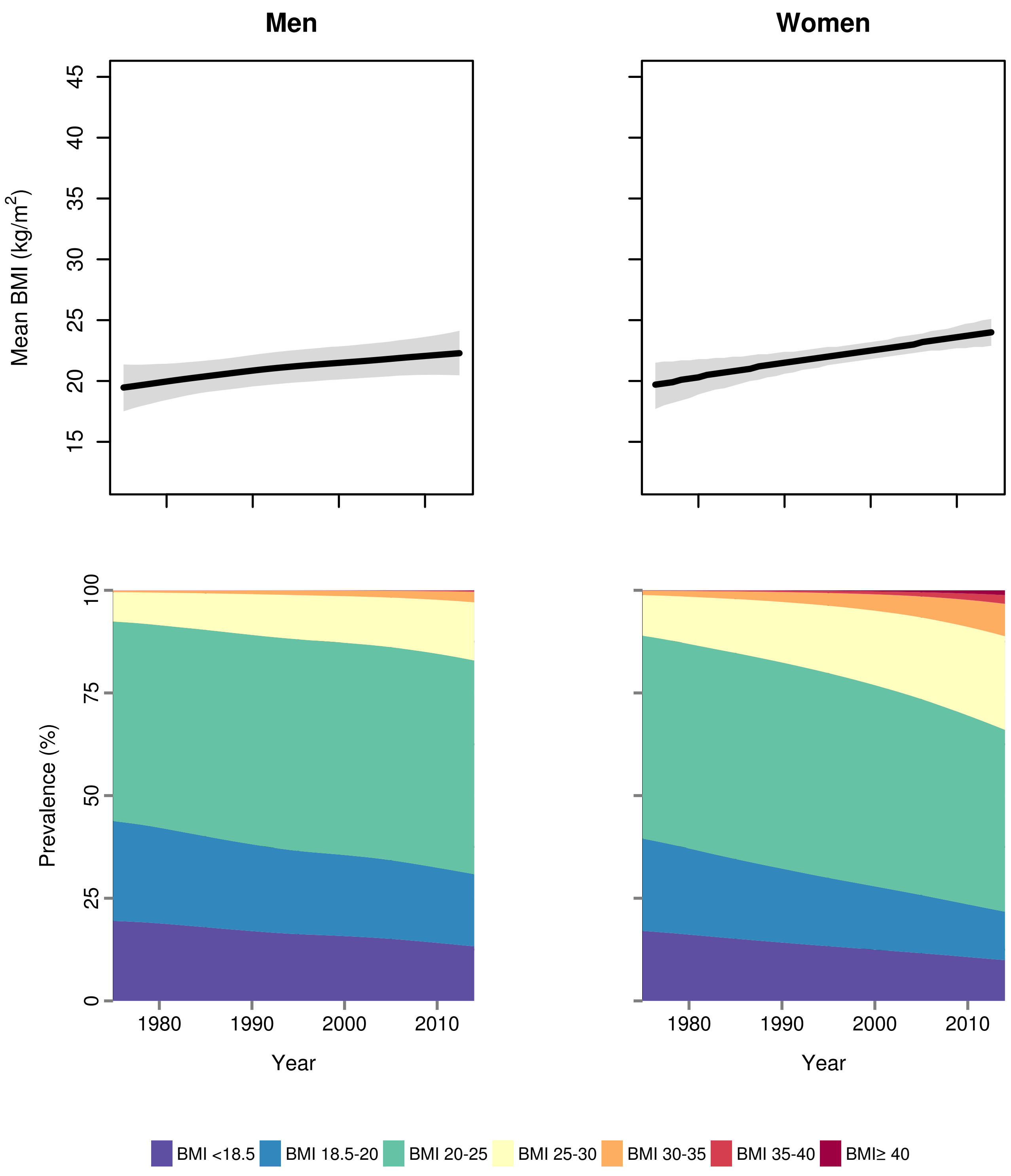

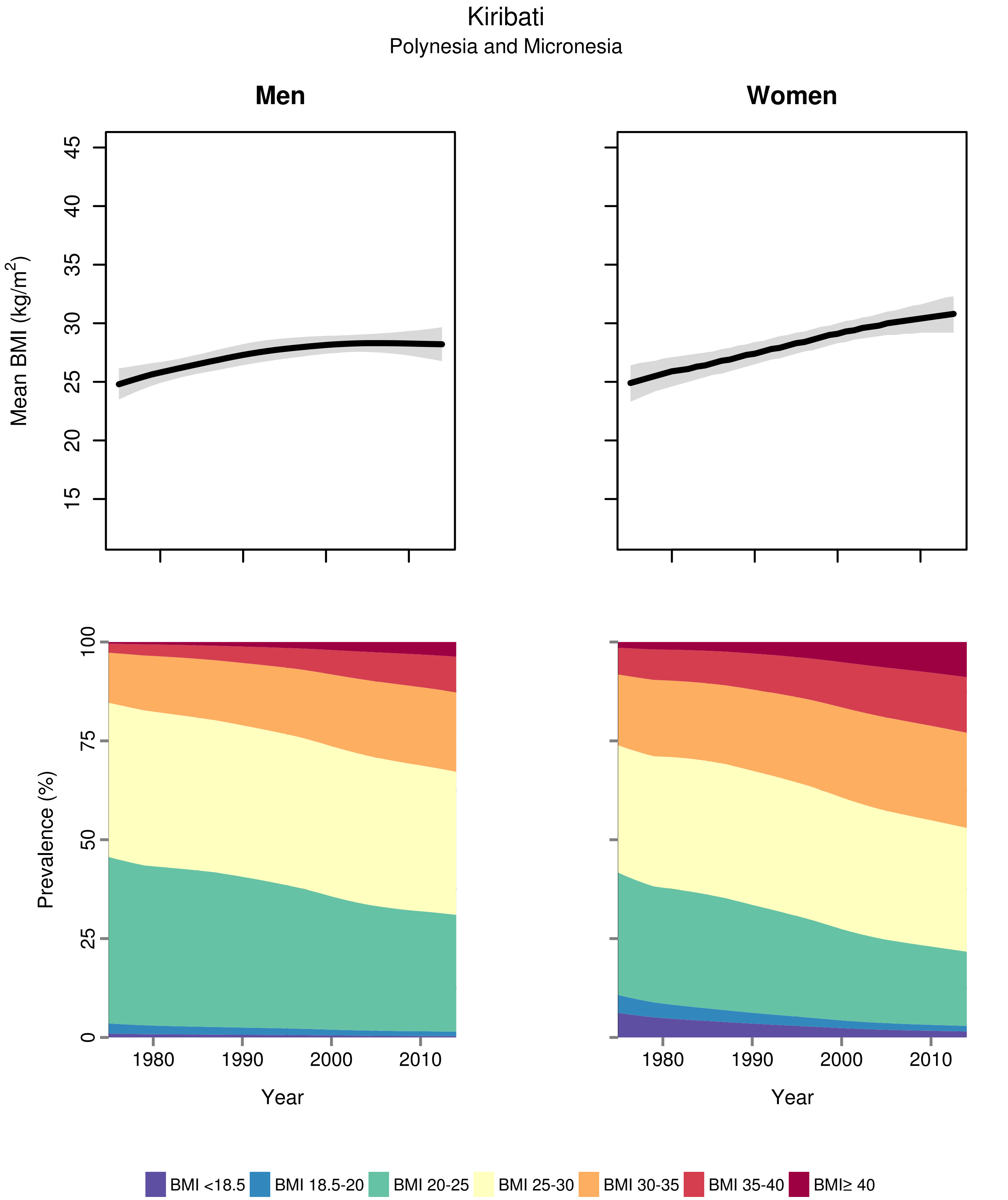
Kiribati
Polynesia and Micronesia
Men
Women
Mean BMI (kg/m^2)
45
40
35
30
25
20
15
Prevalence (%)
100
75
50
25
0
1980
1990
2000
2010
Year
BMI <18.5
BMI 18.5-20
BMI 20-25
BMI 25-30
BMI 30-35
BMI 35-40
BMI≥ 40

Kuwait

Middle East and North Africa

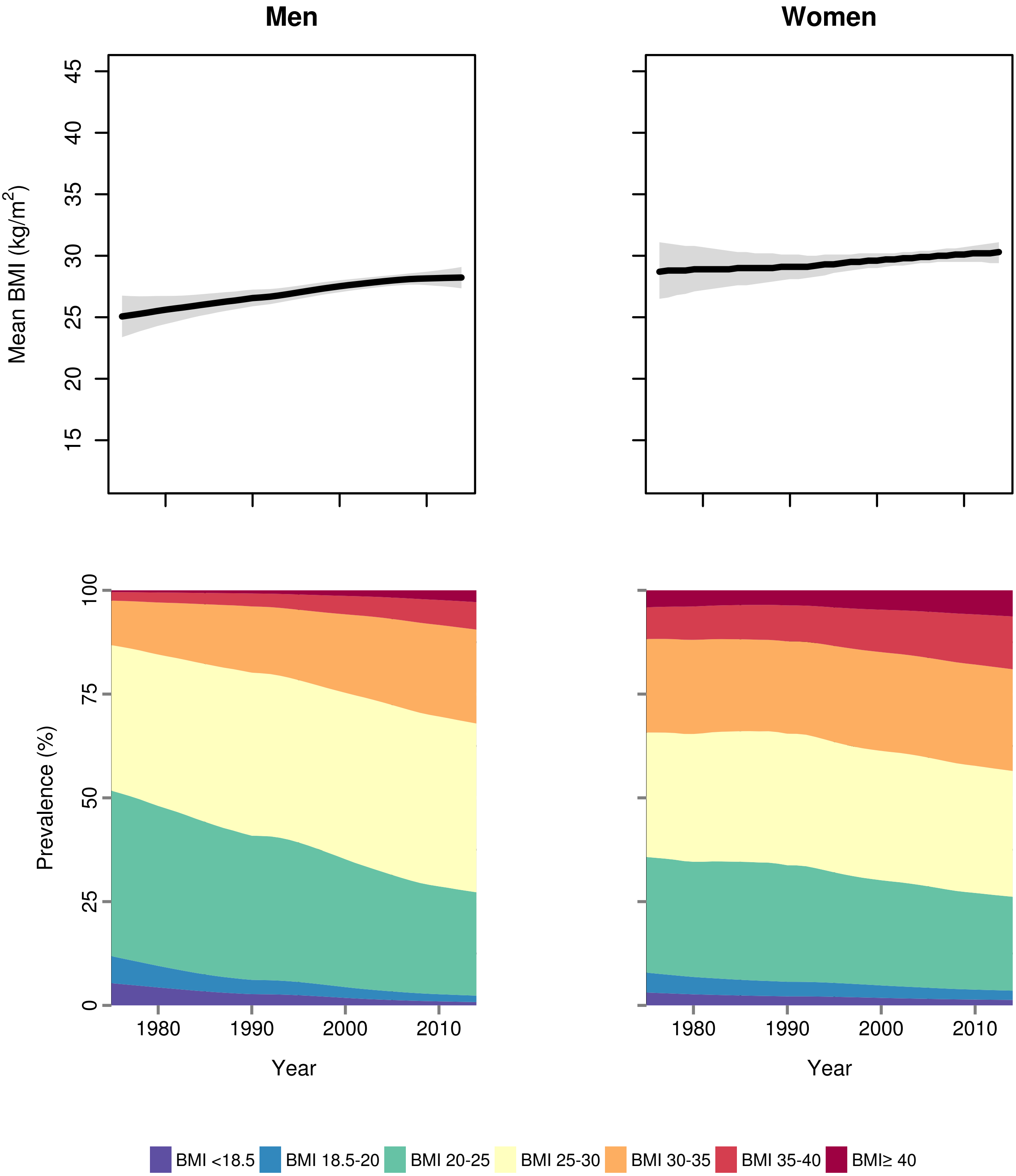

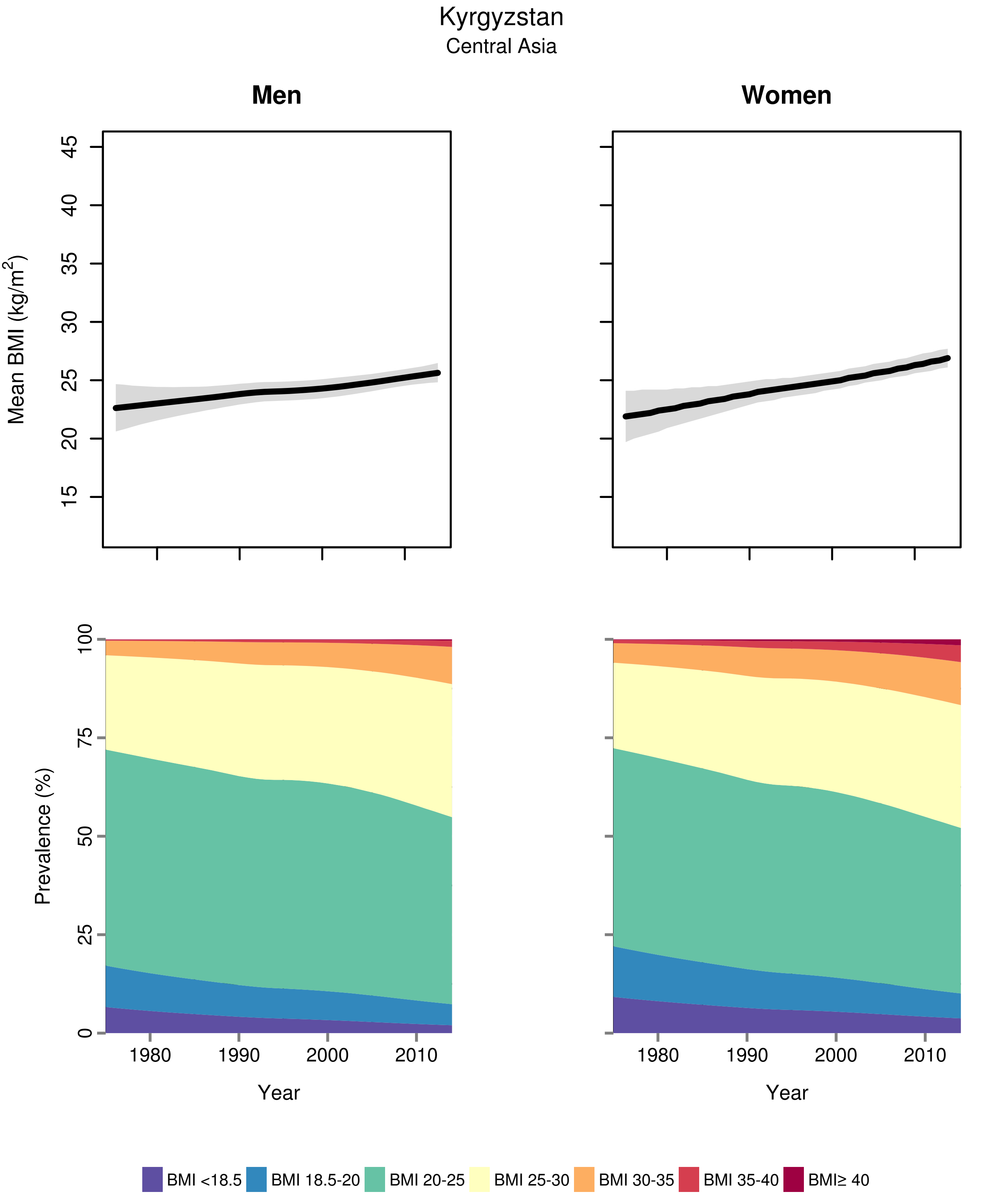

Kyrgyzstan
Central Asia
Men
Women
Mean BMI (kg/m2)
45
40
35
30
25
20
15
Prevalence (%)
100
75
50
25
0
1980
1990
2000
2010
Year
BMI <18.5
BMI 18.5-20
BMI 20-25
BMI 25-30
BMI 30-35
BMI 35-40
BMI≥ 40

Lao PDR

South East Asia

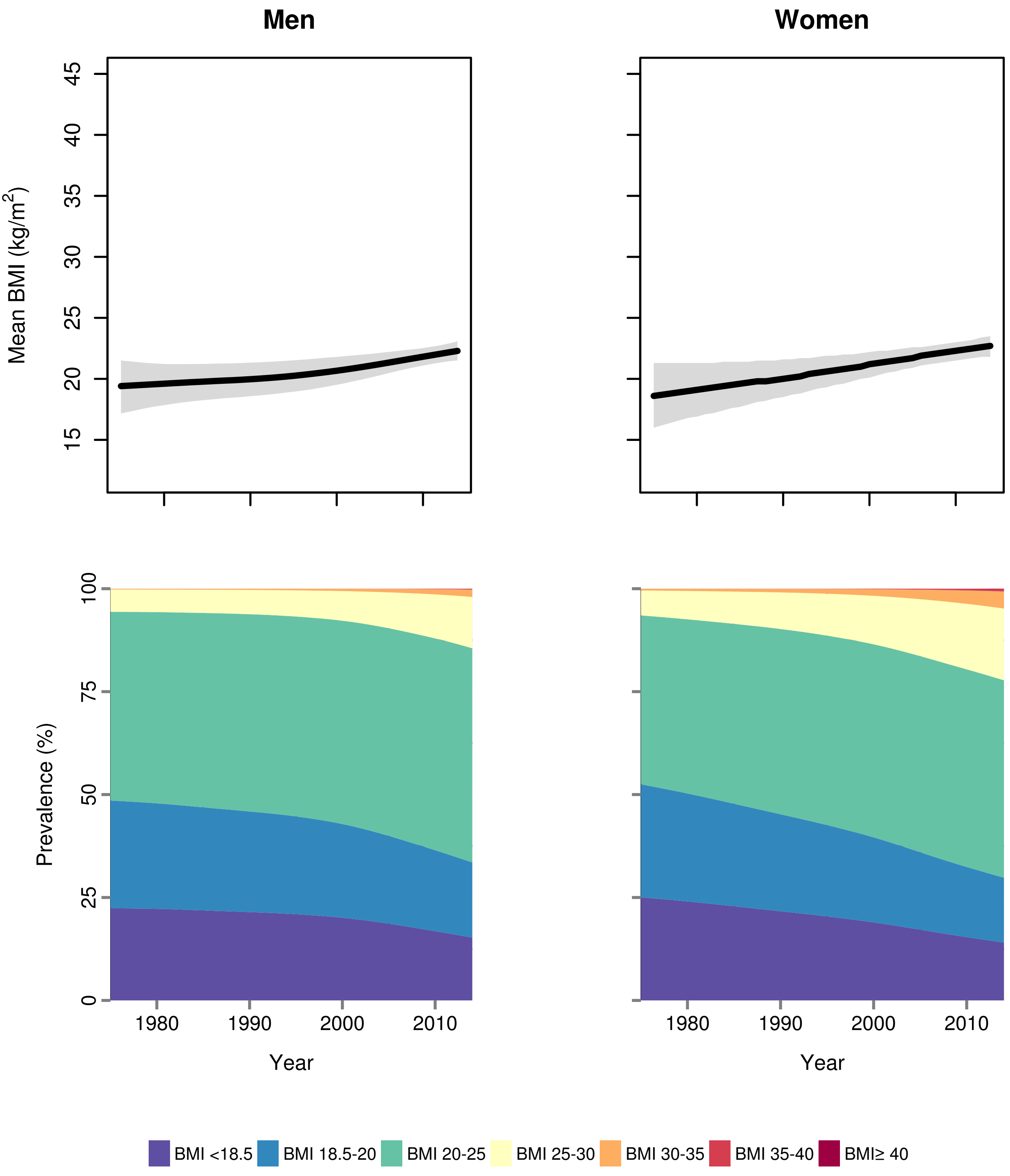

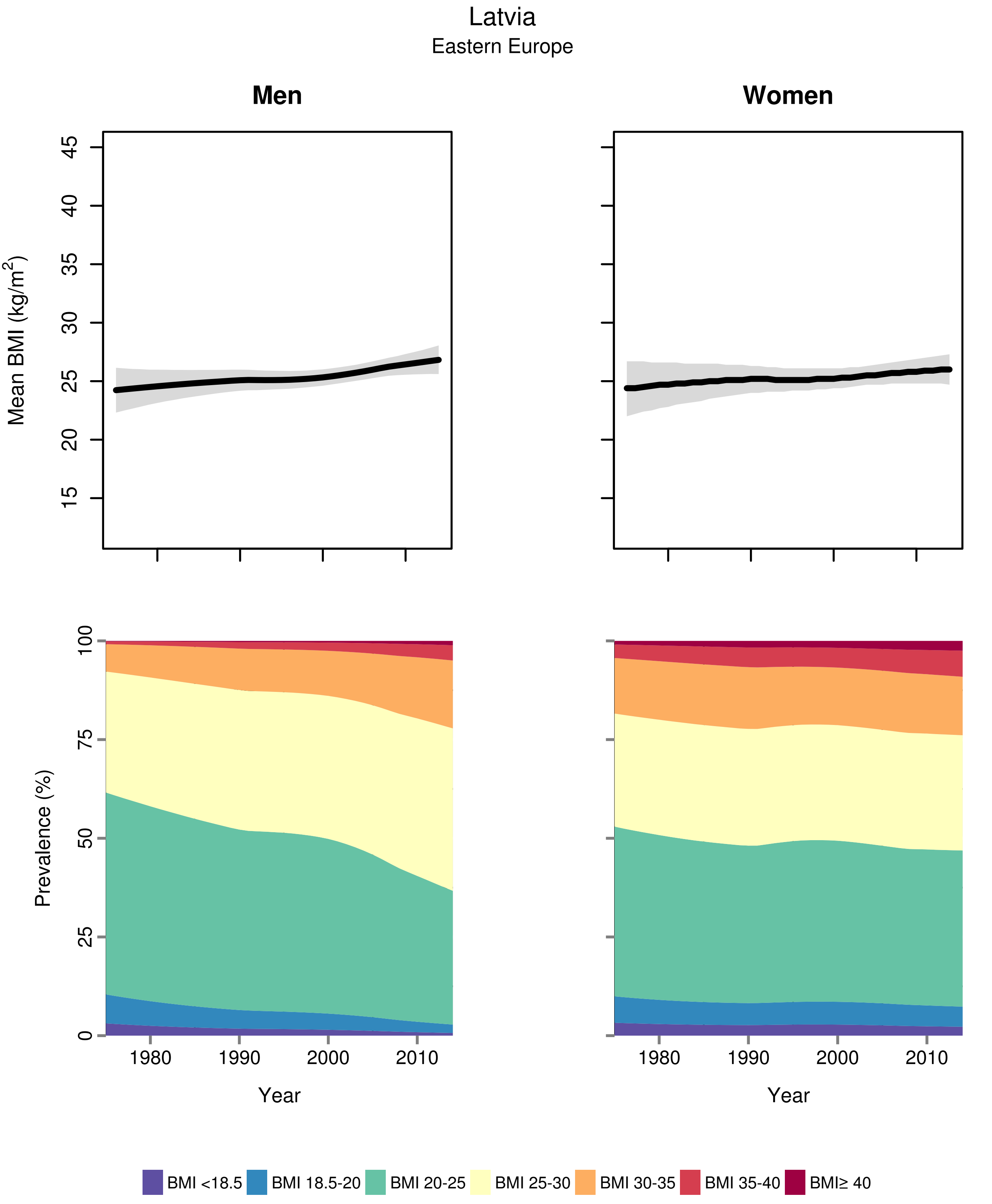

Latvia
Eastern Europe
Men
Women
Mean BMI (kg/m2)
45
40
35
30
25
20
15
Prevalence (%)
100
75
50
25
0
1980
1990
2000
2010
Year
BMI <18.5
BMI 18.5-20
BMI 20-25
BMI 25-30
BMI 30-35
BMI 35-40
BMI≥ 40

Lebanon

Middle East and North Africa

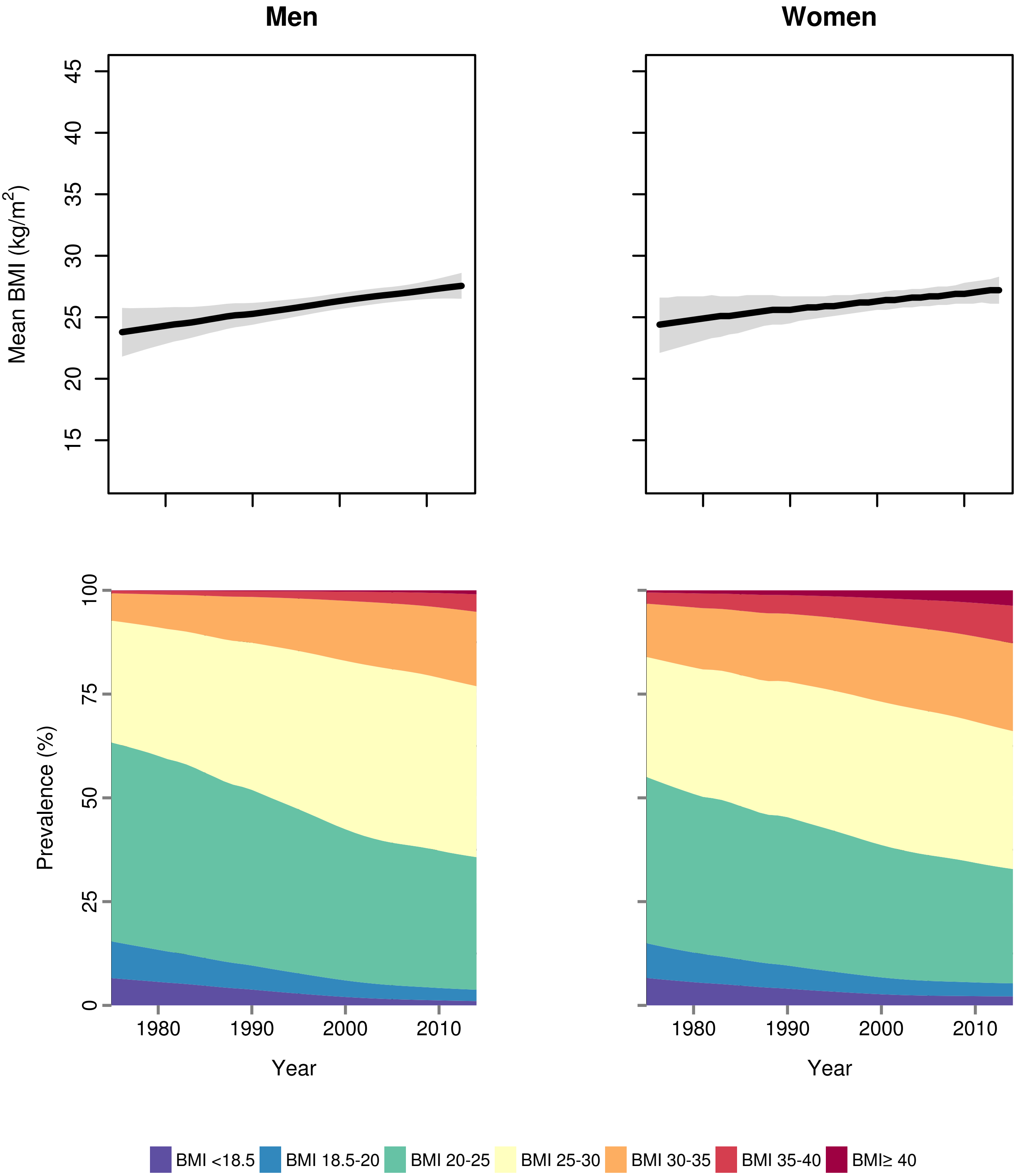

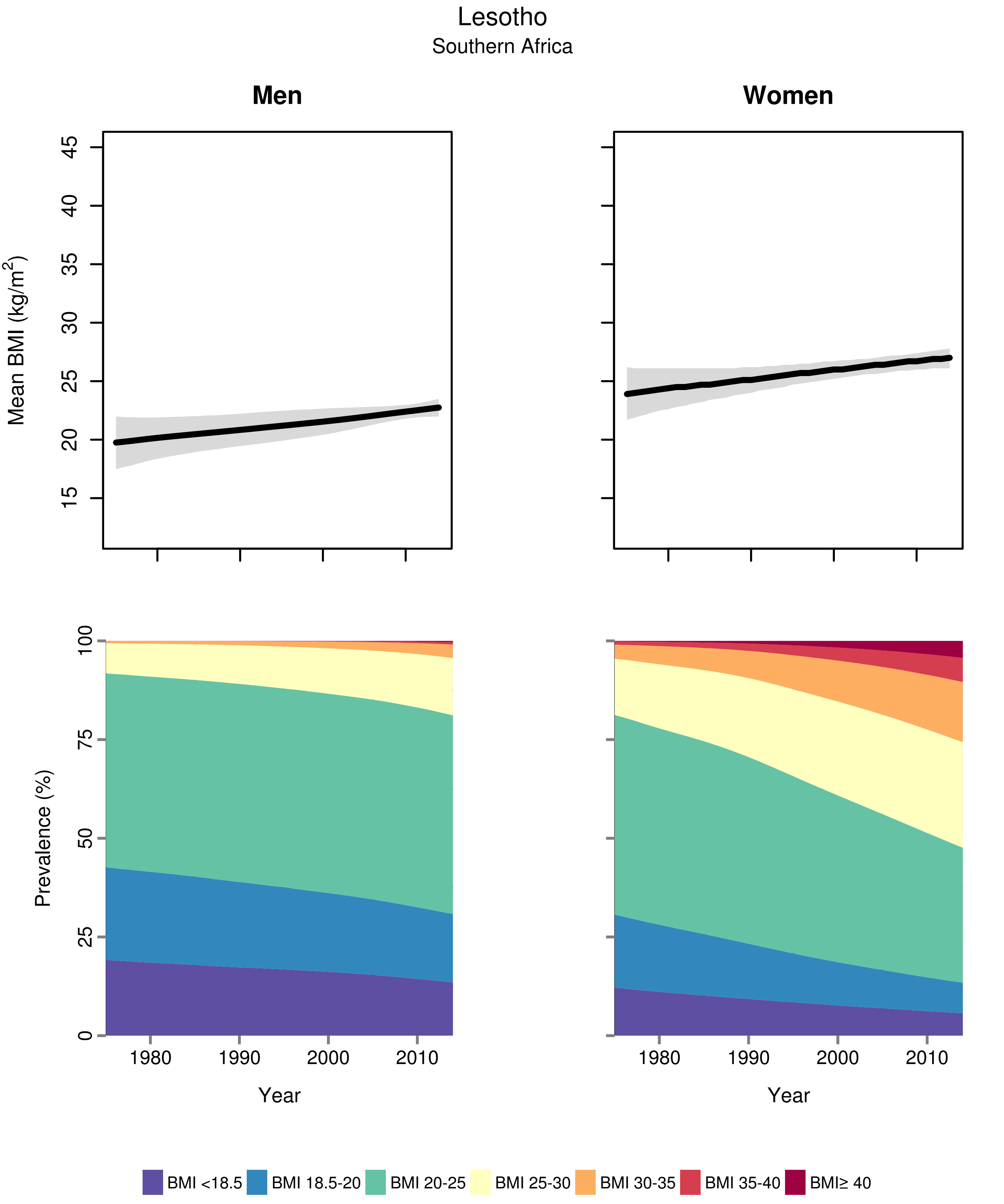
Lesotho
Southern Africa
Men
Women
Mean BMI (kg/m2)
45
40
35
30
25
20
15
Prevalence (%)
100
75
50
25
0
1980
1990
2000
2010
Year
BMI <18.5
BMI 18.5-20
BMI 20-25
BMI 25-30
BMI 30-35
BMI 35-40
BMI≥ 40

Liberia

West Africa

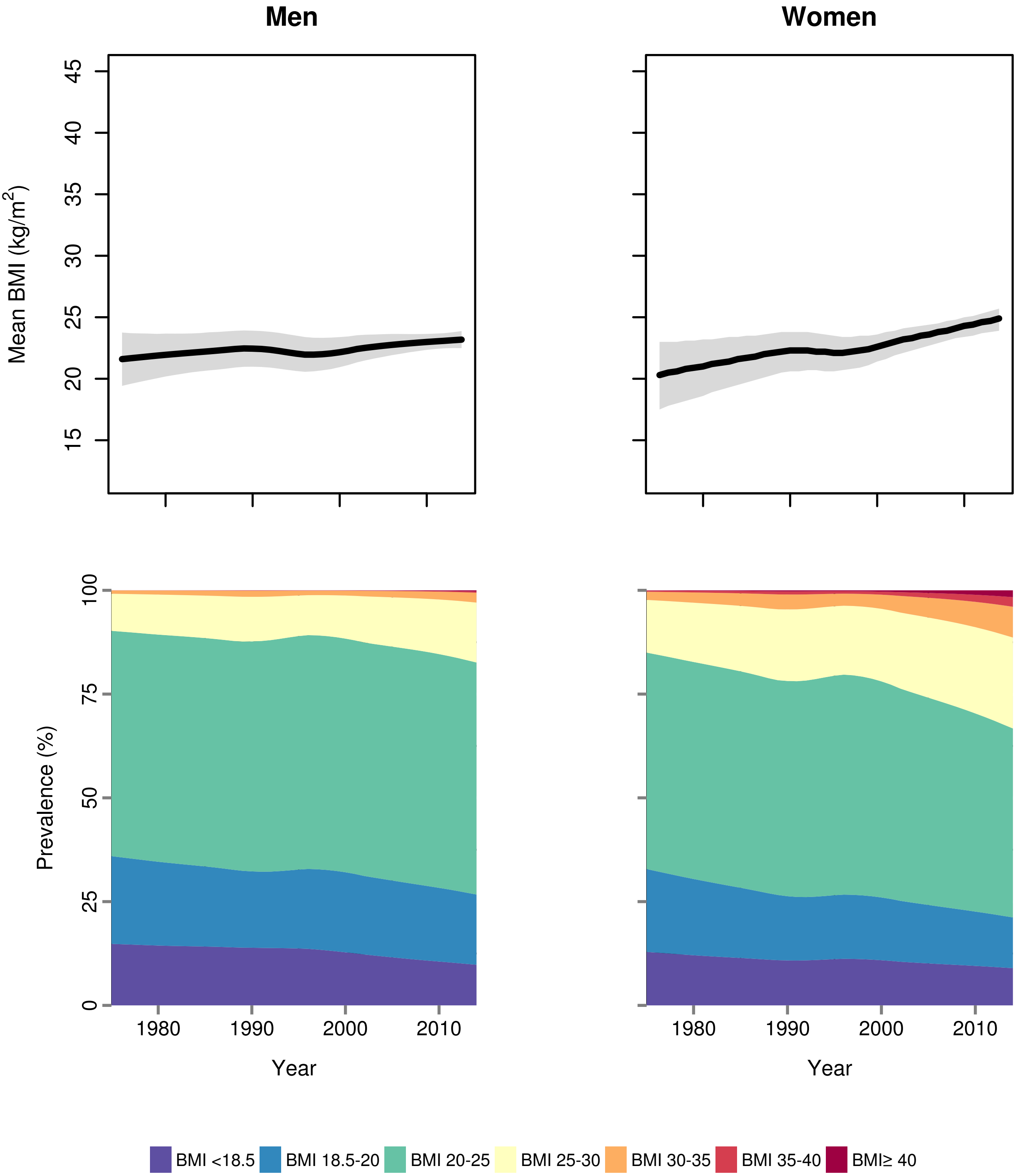

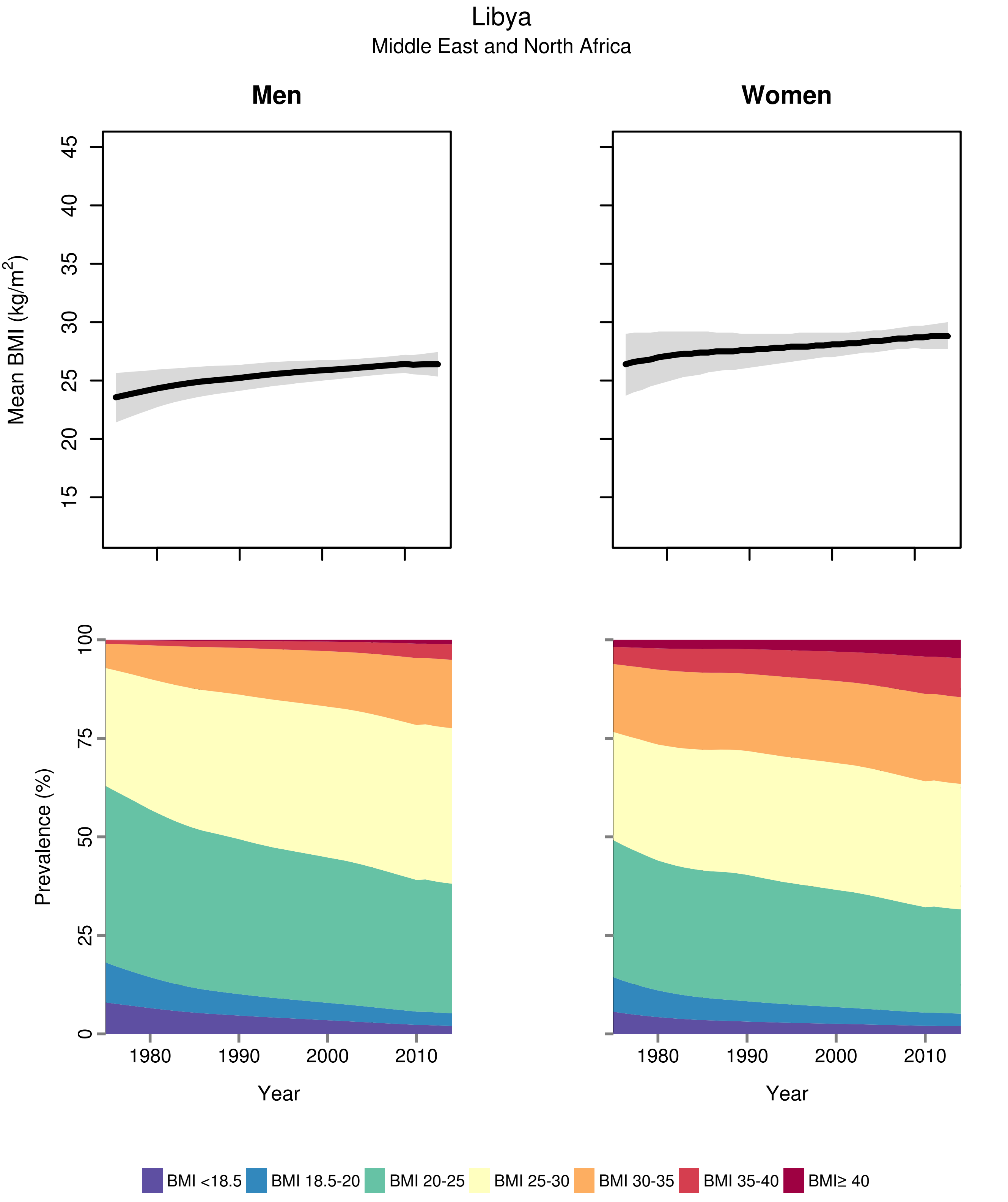

Libya
Middle East and North Africa
Men
Women
Mean BMI (kg/m2)
45
40
35
30
25
20
15
Prevalence (%)
100
75
50
25
0
1980
1990
2000
2010
Year
BMI <18.5
BMI 18.5-20
BMI 20-25
BMI 25-30
BMI 30-35
BMI 35-40
BMI≥ 40

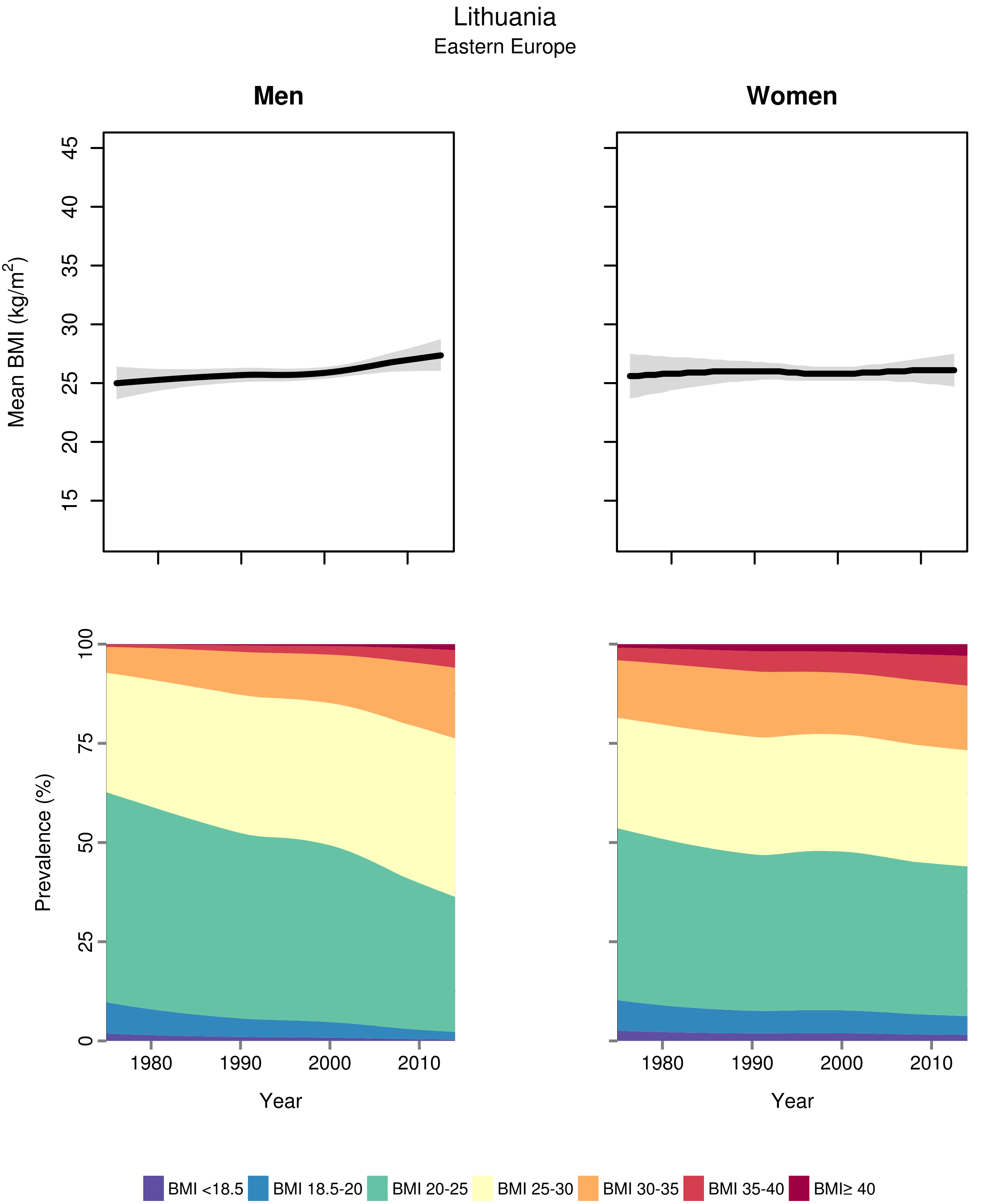

Lithuania
Eastern Europe
Men
Women
Mean BMI (kg/m²)
45
40
35
30
25
20
15
100
75
50
25
0
Prevalence (%)
1980
1990
2000
2010
Year
BMI <18.5
BMI 18.5-20
BMI 20-25
BMI 25-30
BMI 30-35
BMI 35-40
BMI≥ 40

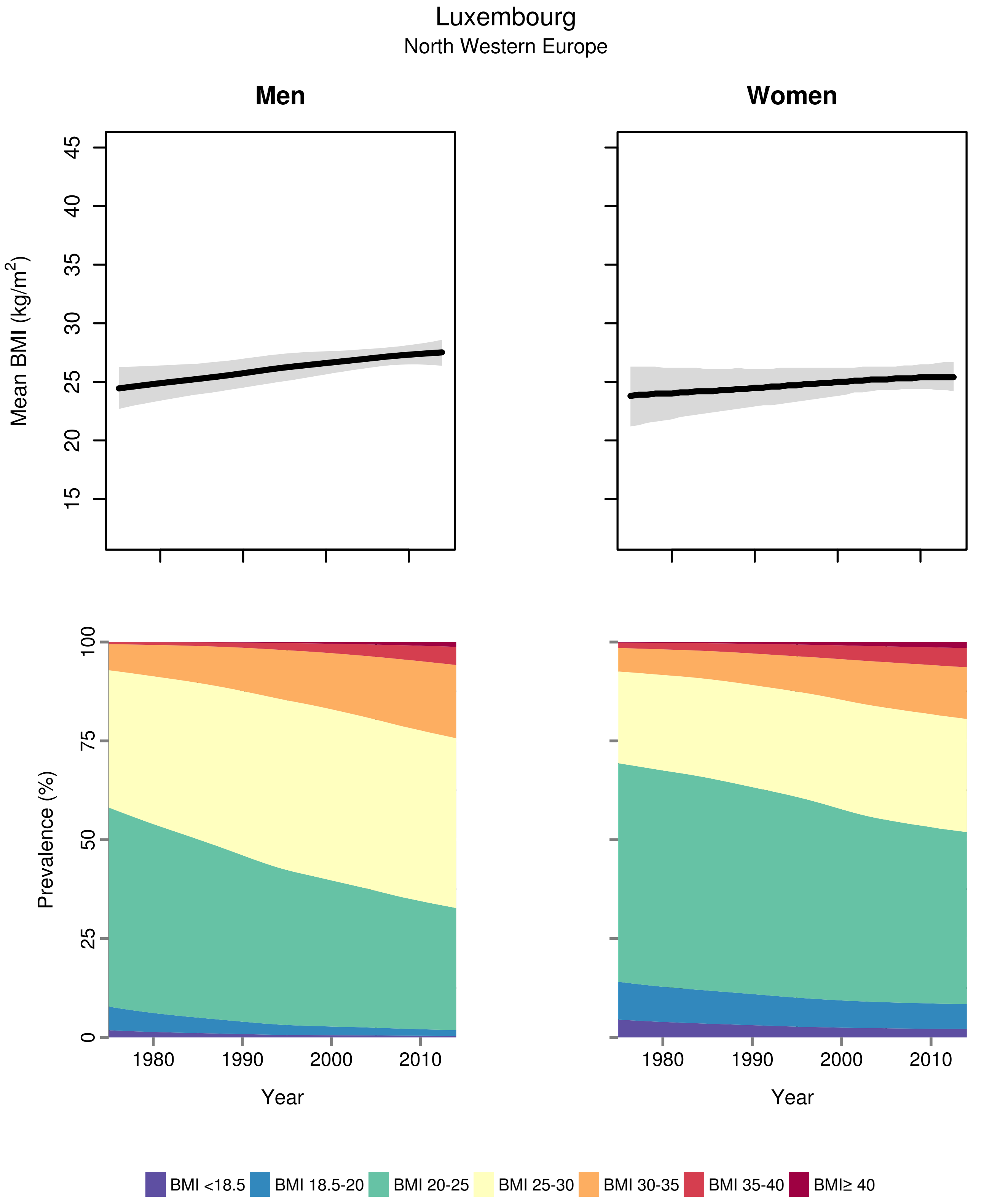
Luxembourg
North Western Europe
Men
Women
Mean BMI (kg/m2)
45
40
35
30
25
20
15
Prevalence (%)
100
75
50
25
0
1980
1990
2000
2010
Year
BMI <18.5
BMI 18.5-20
BMI 20-25
BMI 25-30
BMI 30-35
BMI 35-40
BMI≥ 40

Macedonia (TFYR)

Central Europe

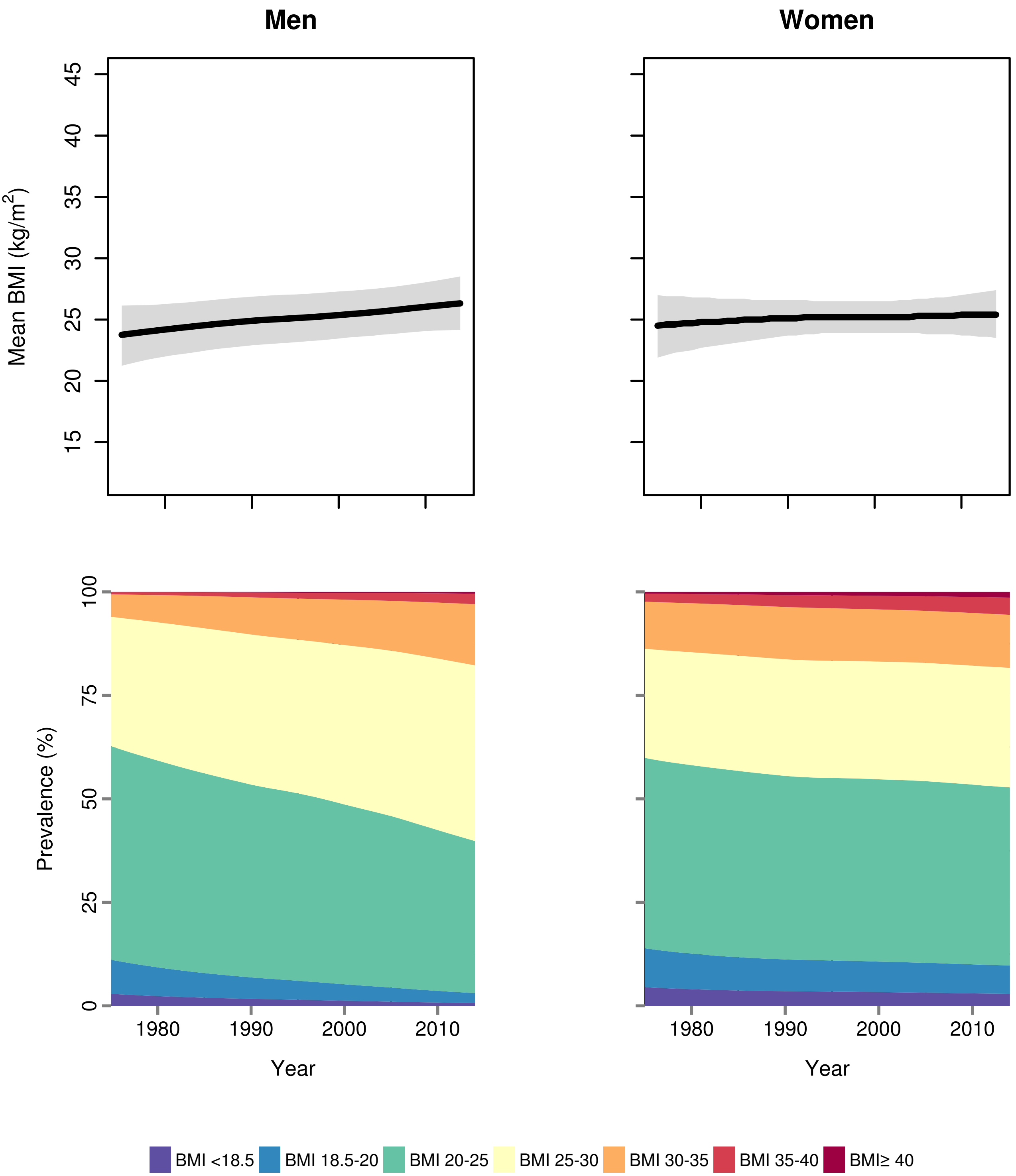

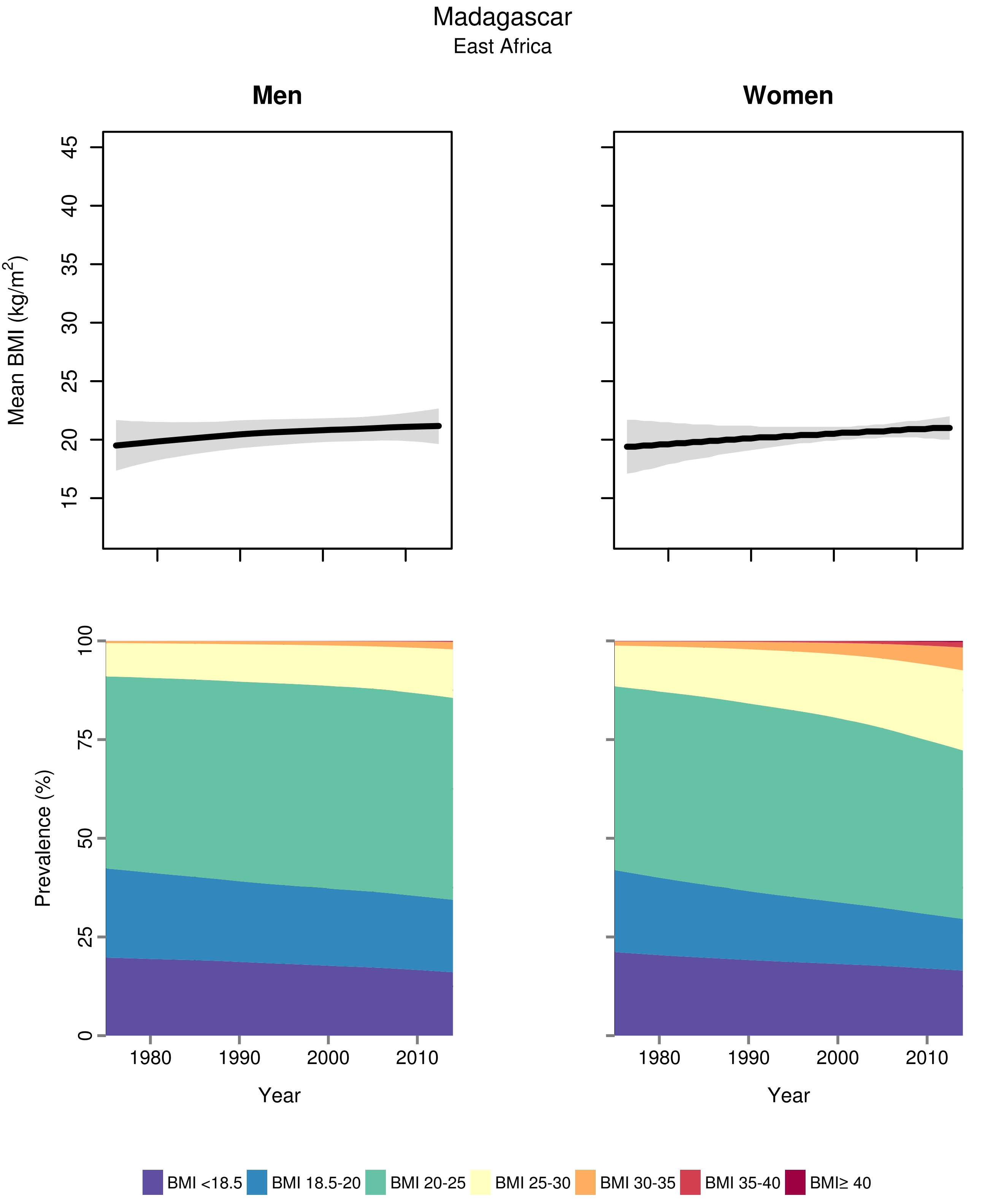
Madagascar
East Africa
Men
Women
Mean BMI (kg/m2)
45
40
35
30
25
20
15
100
75
50
25
0
Prevalence (%)
1980
1990
2000
2010
Year
BMI <18.5
BMI 18.5-20
BMI 20-25
BMI 25-30
BMI 30-35
BMI 35-40
BMI≥ 40

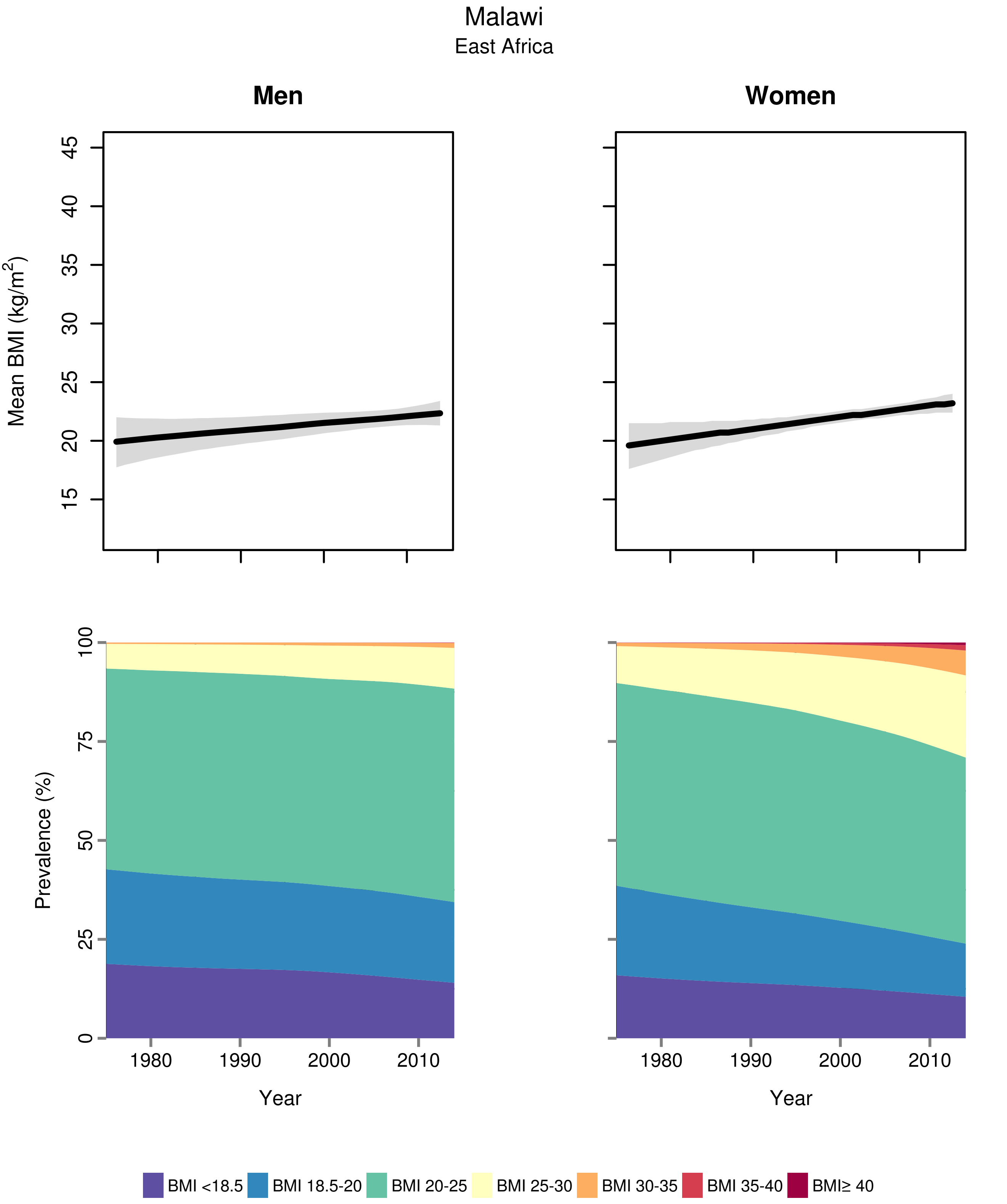
Malawi
East Africa
Men
Women
Mean BMI (kg/m^2)
15
20
25
30
35
40
45
Prevalence (%)
0
25
50
75
100
1980
1990
2000
2010
Year
BMI <18.5
BMI 18.5-20
BMI 20-25
BMI 25-30
BMI 30-35
BMI 35-40
BMI≥ 40

Malaysia
South East Asia

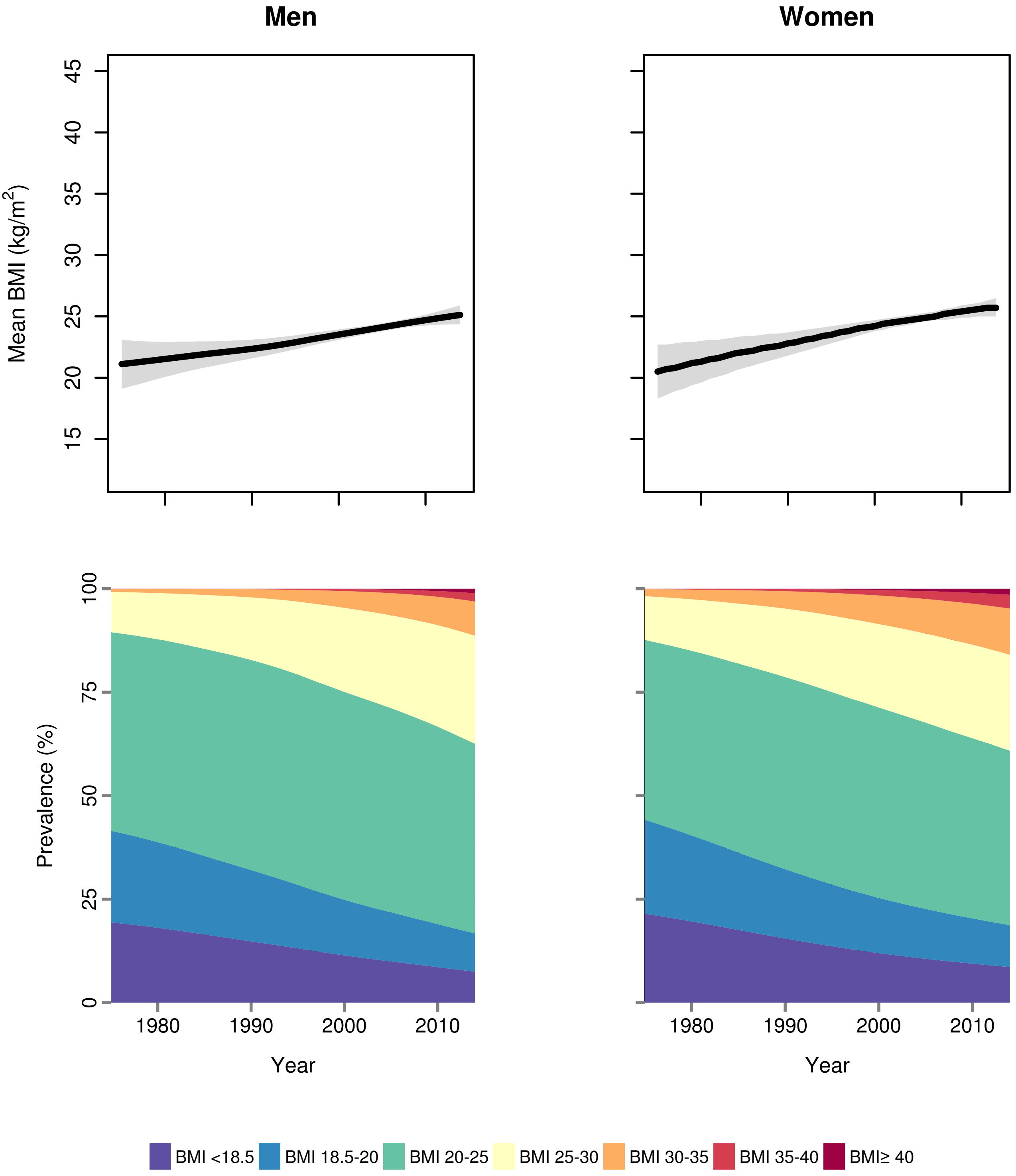

Maldives

South East Asia

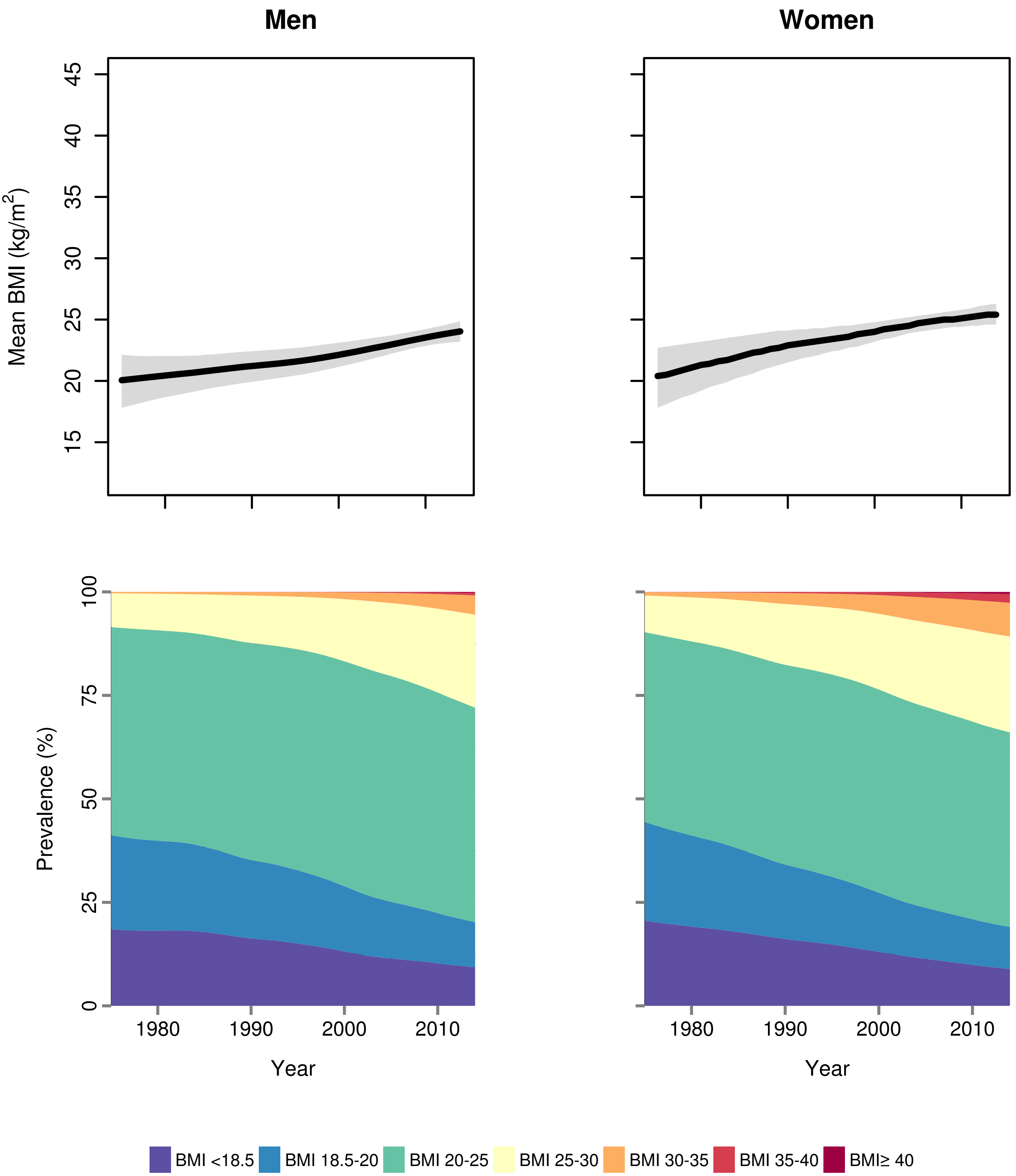

Mali

West Africa

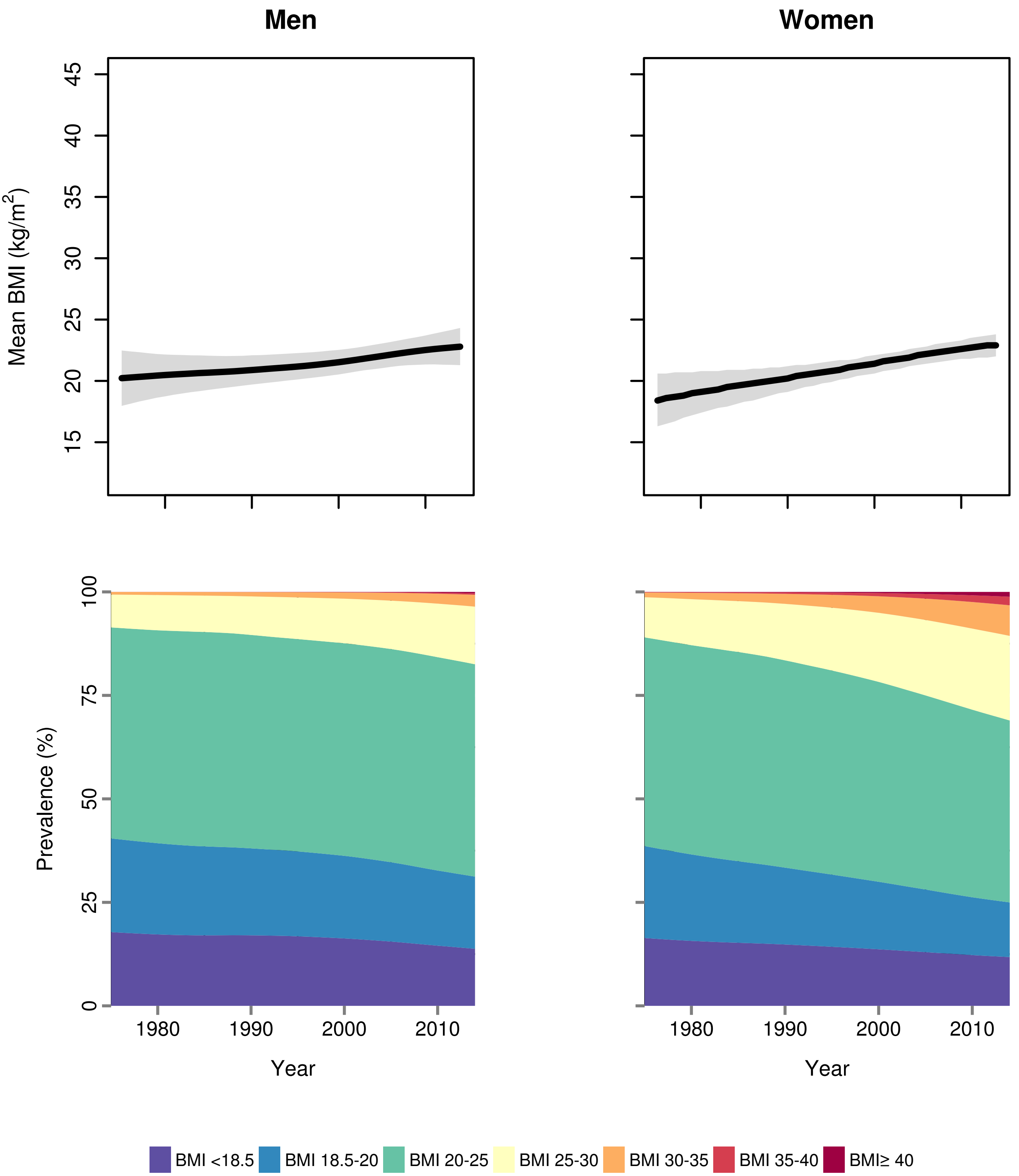

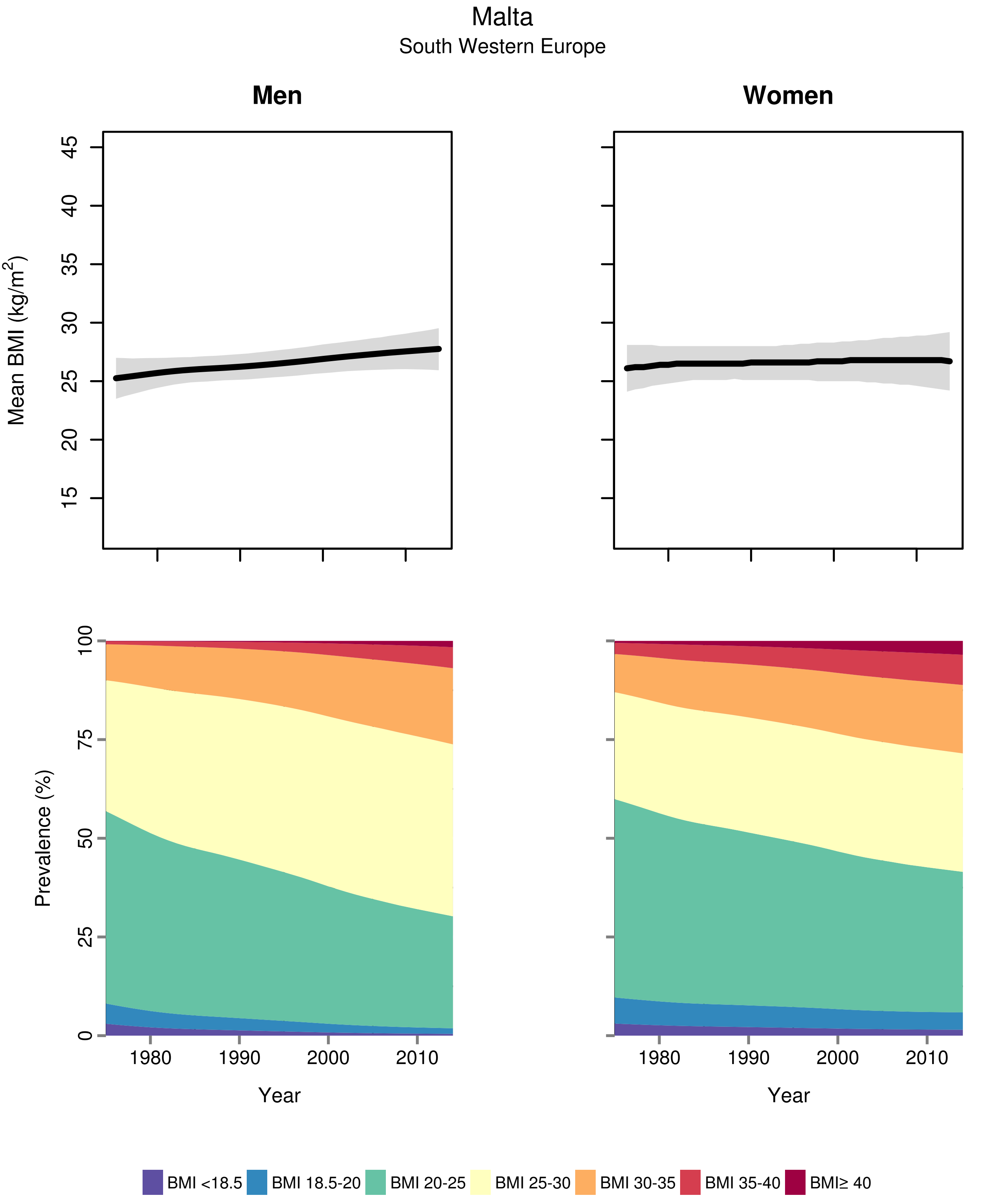

Malta
South Western Europe
Men
Women
Mean BMI (kg/m²)
45
40
35
30
25
20
15
Prevalence (%)
100
75
50
25
0
1980
1990
2000
2010
Year
BMI <18.5
BMI 18.5-20
BMI 20-25
BMI 25-30
BMI 30-35
BMI 35-40
BMI≥ 40

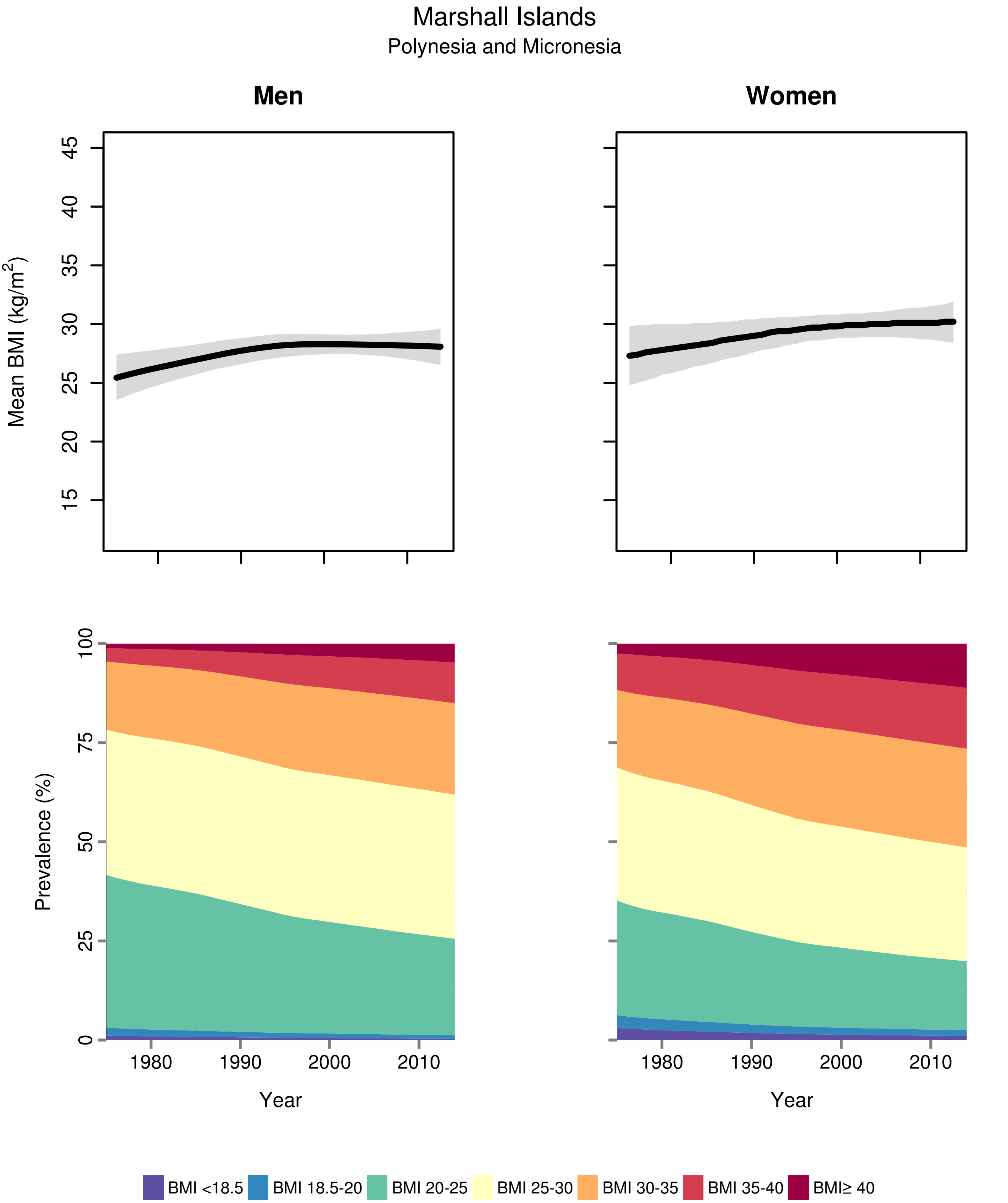
Marshall Islands
Polynesia and Micronesia
Men
Women
Mean BMI (kg/m2)
45
40
35
30
25
20
15
Prevalence (%)
100
75
50
25
0
1980
1990
2000
2010
Year
BMI <18.5
BMI 18.5-20
BMI 20-25
BMI 25-30
BMI 30-35
BMI 35-40
BMI≥ 40

Mauritania

West Africa

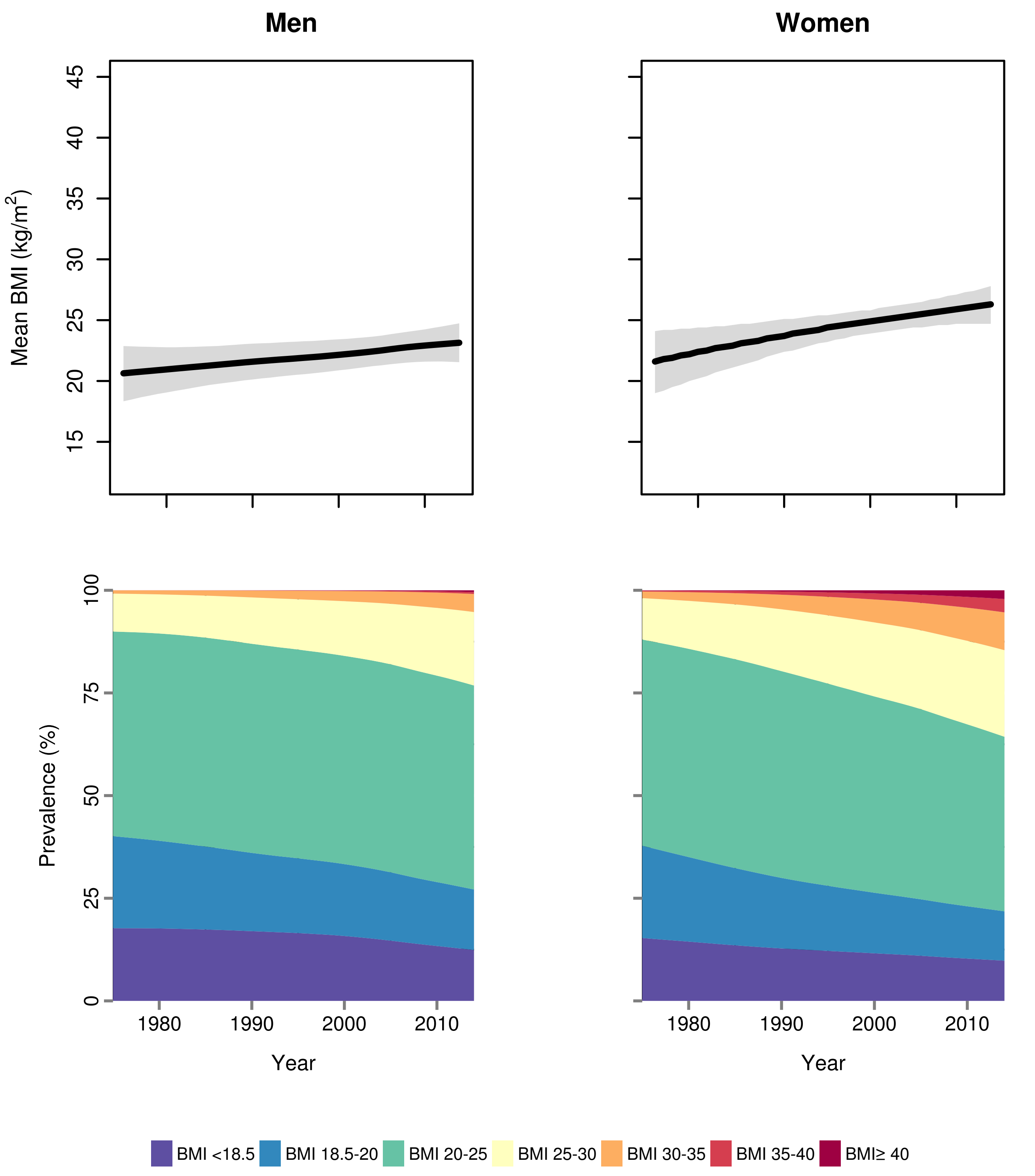

Mauritius
East Africa

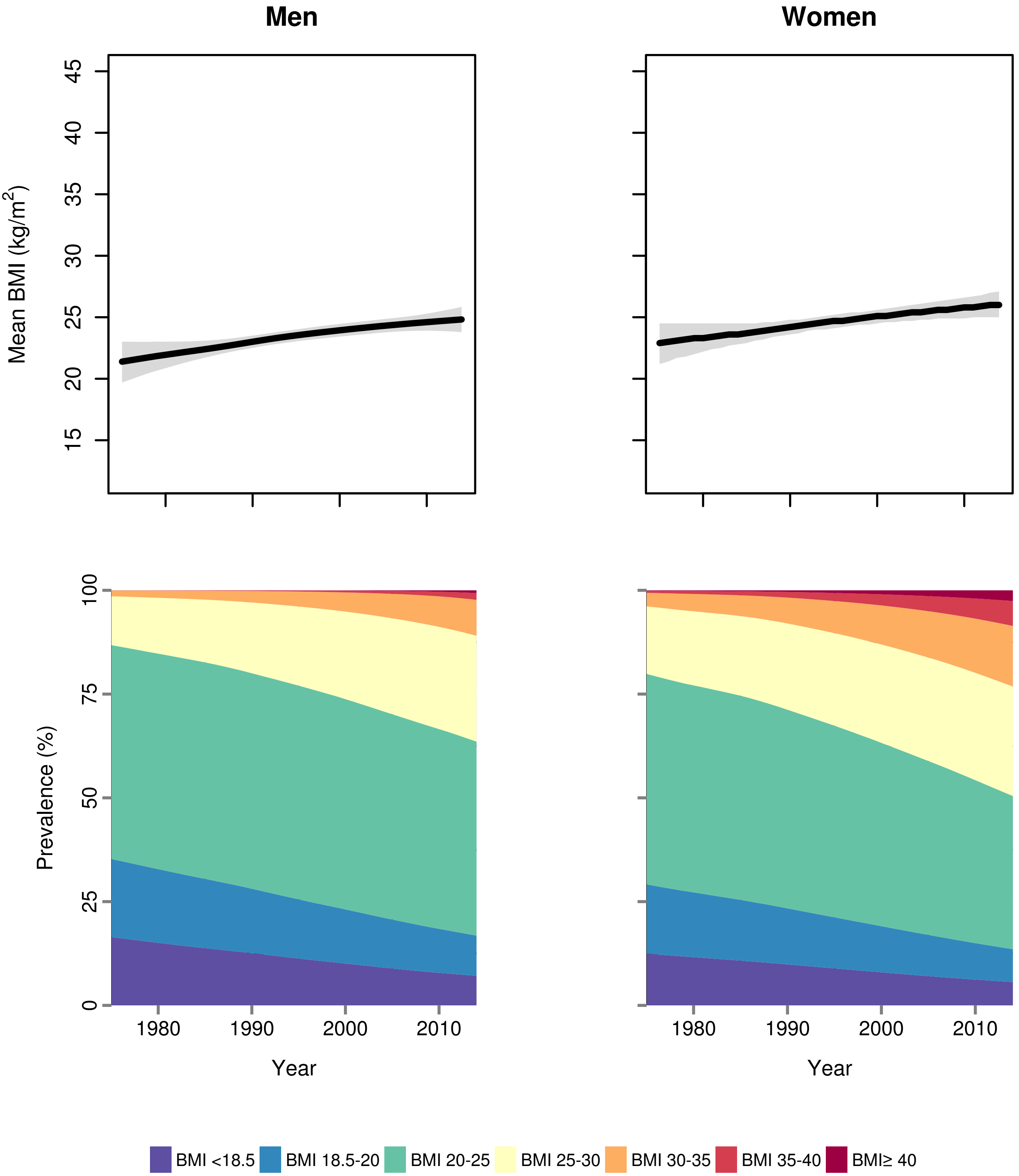

Mexico
Central Latin America

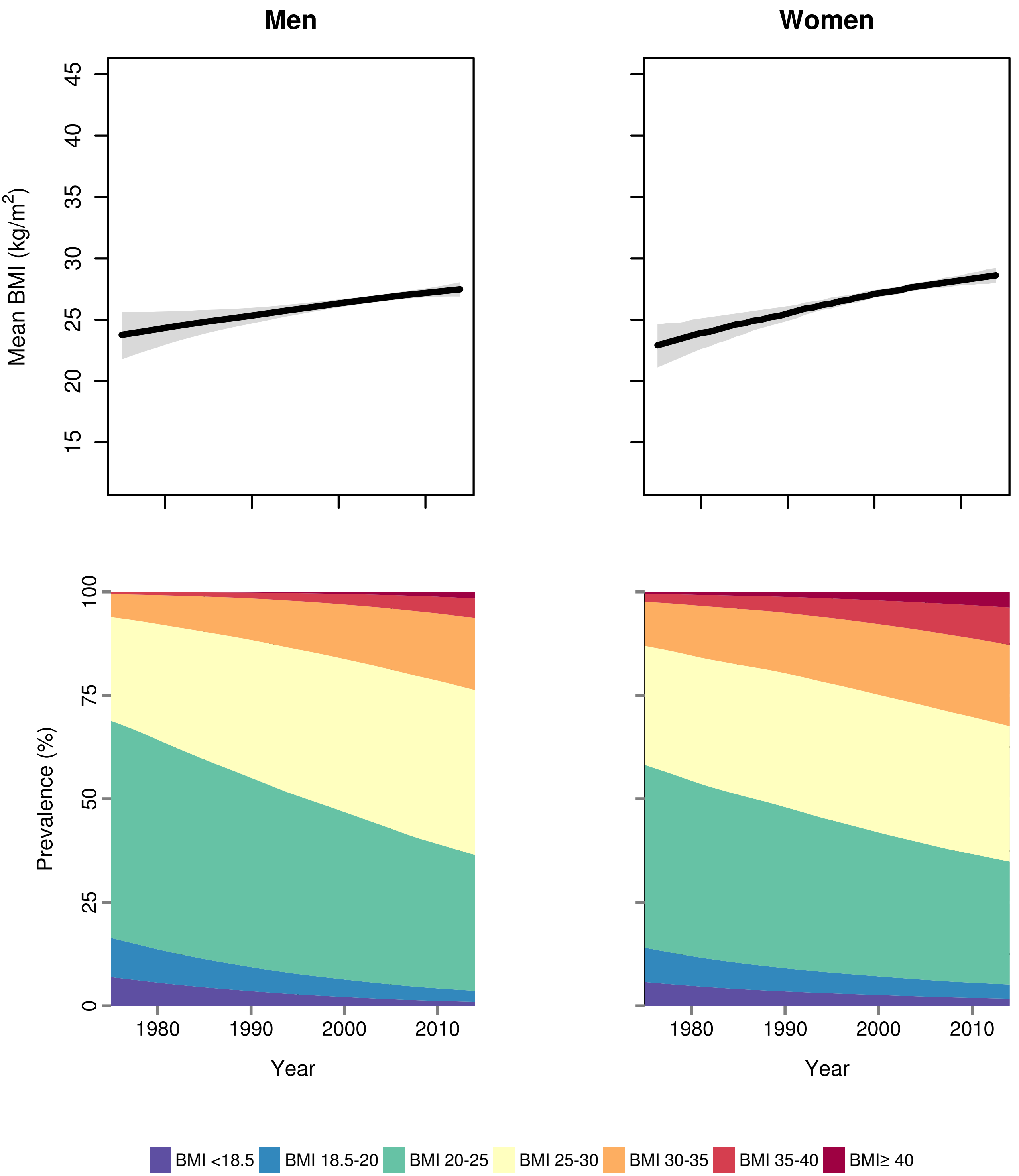

Micronesia (Federated States of)

Polynesia and Micronesia

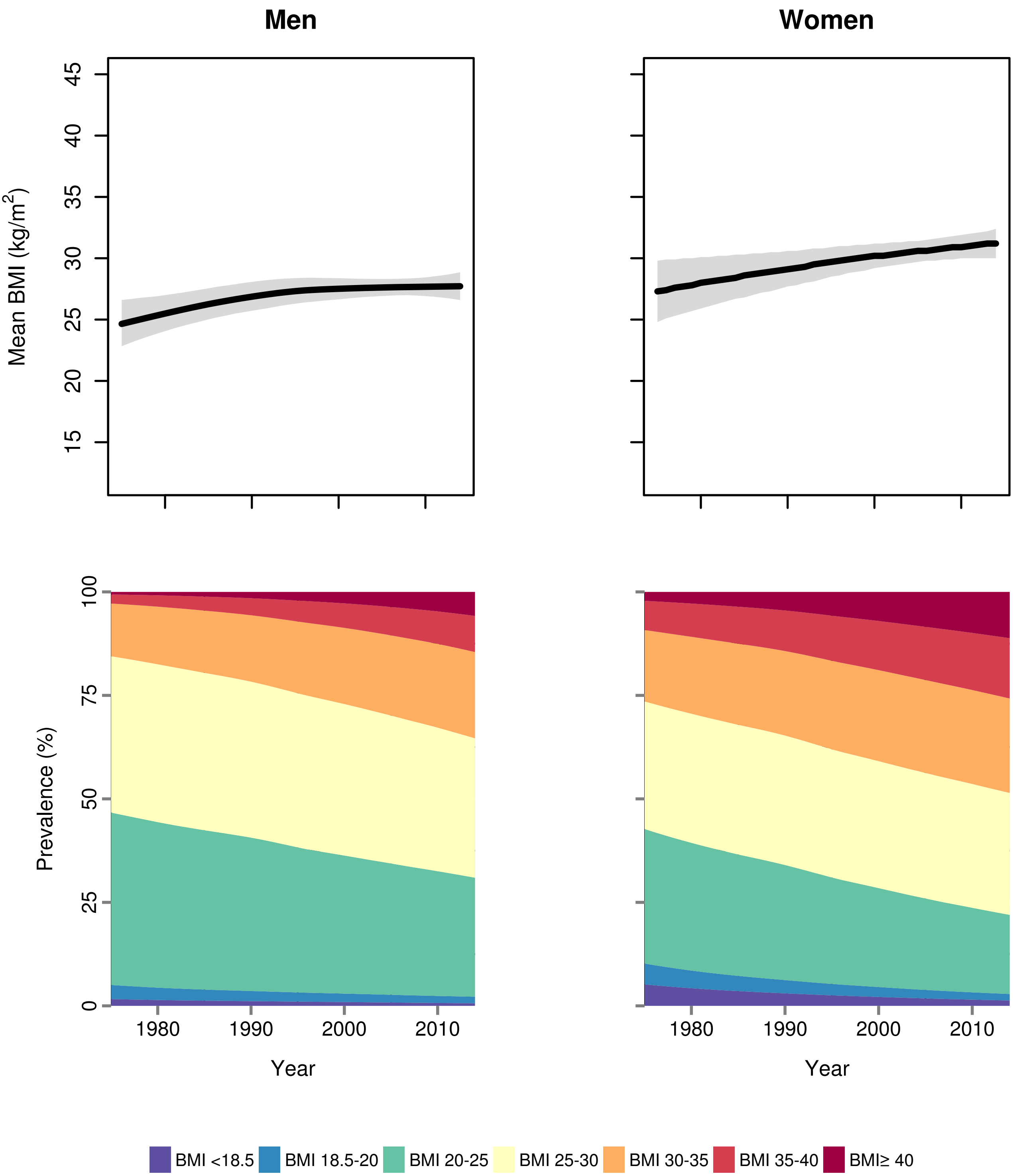

Moldova
Eastern Europe

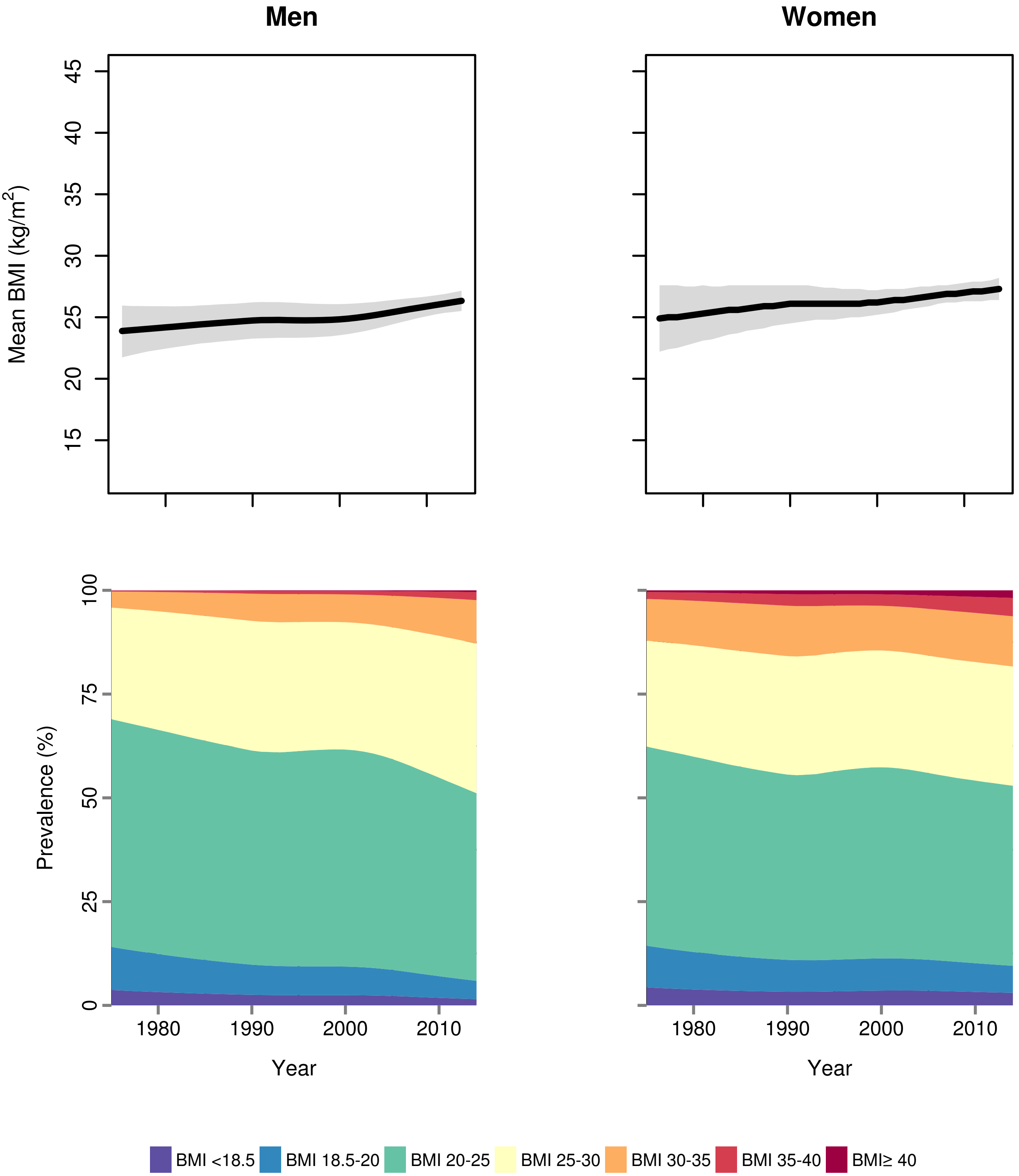

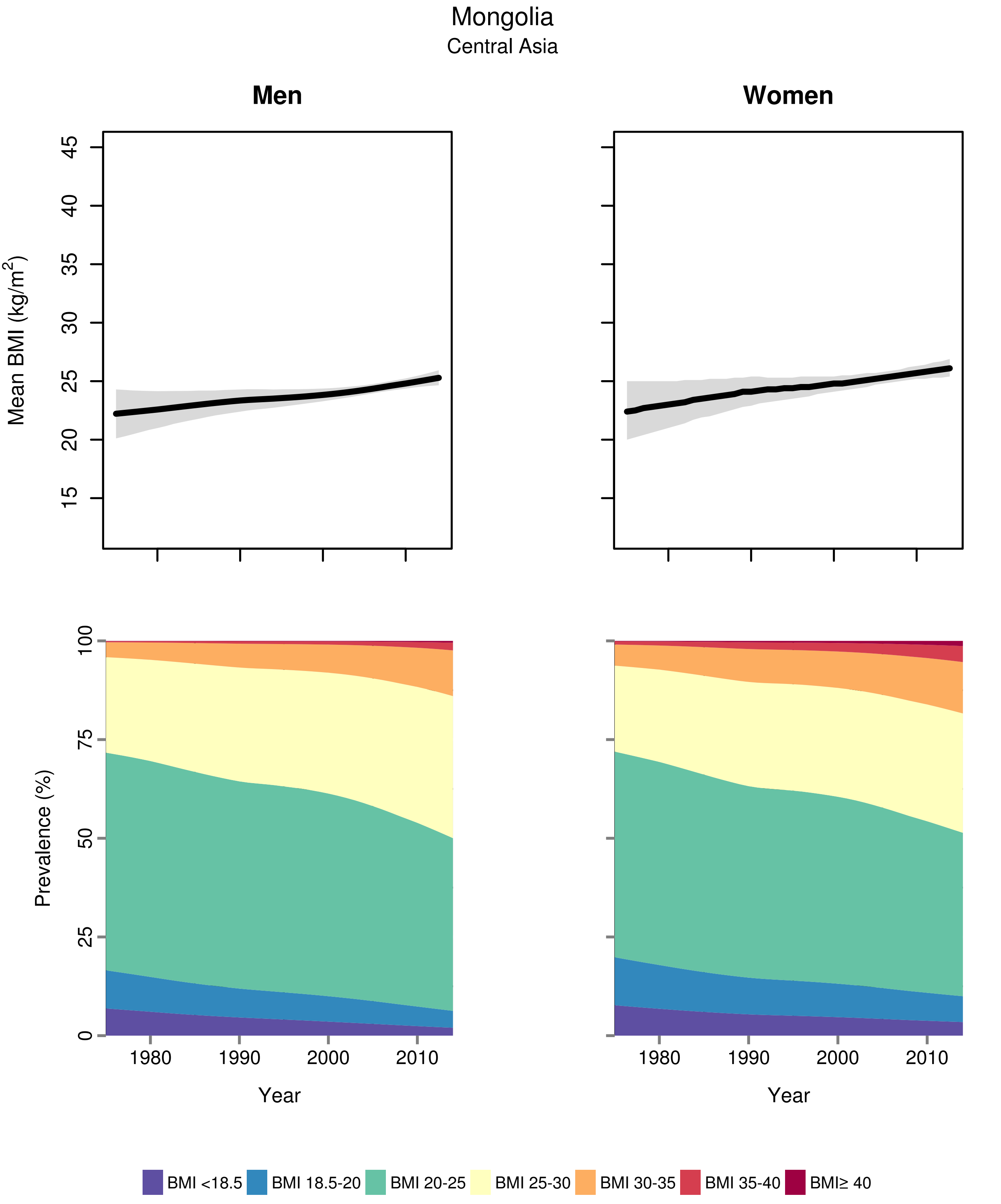
Mongolia
Central Asia
Men
Women
Mean BMI (kg/m2)
45
40
35
30
25
20
15
Prevalence (%)
100
75
50
25
0
1980
1990
2000
2010
Year
BMI <18.5
BMI 18.5-20
BMI 20-25
BMI 25-30
BMI 30-35
BMI 35-40
BMI≥ 40

Montenegro
Central Europe

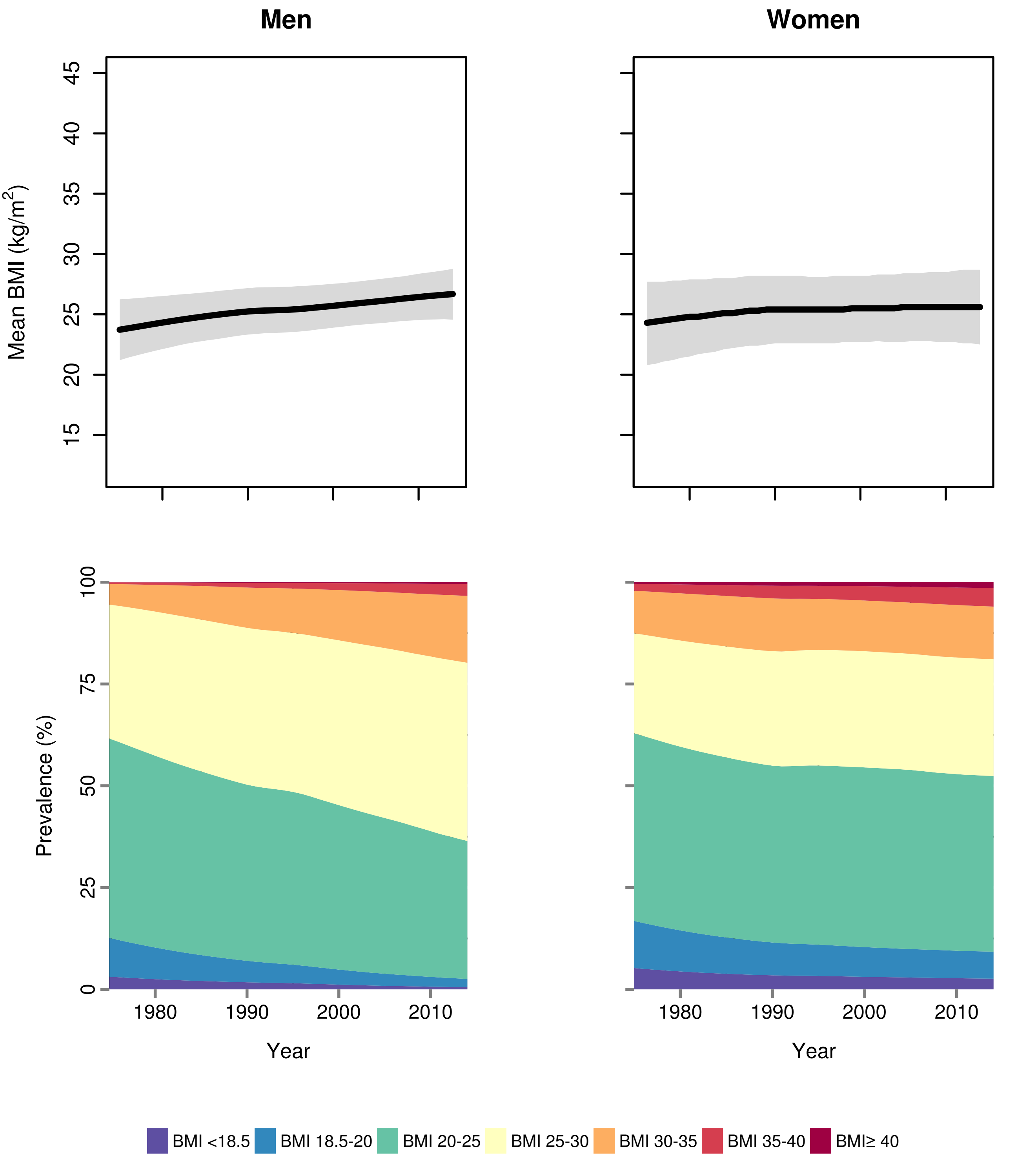

Morocco

Middle East and North Africa

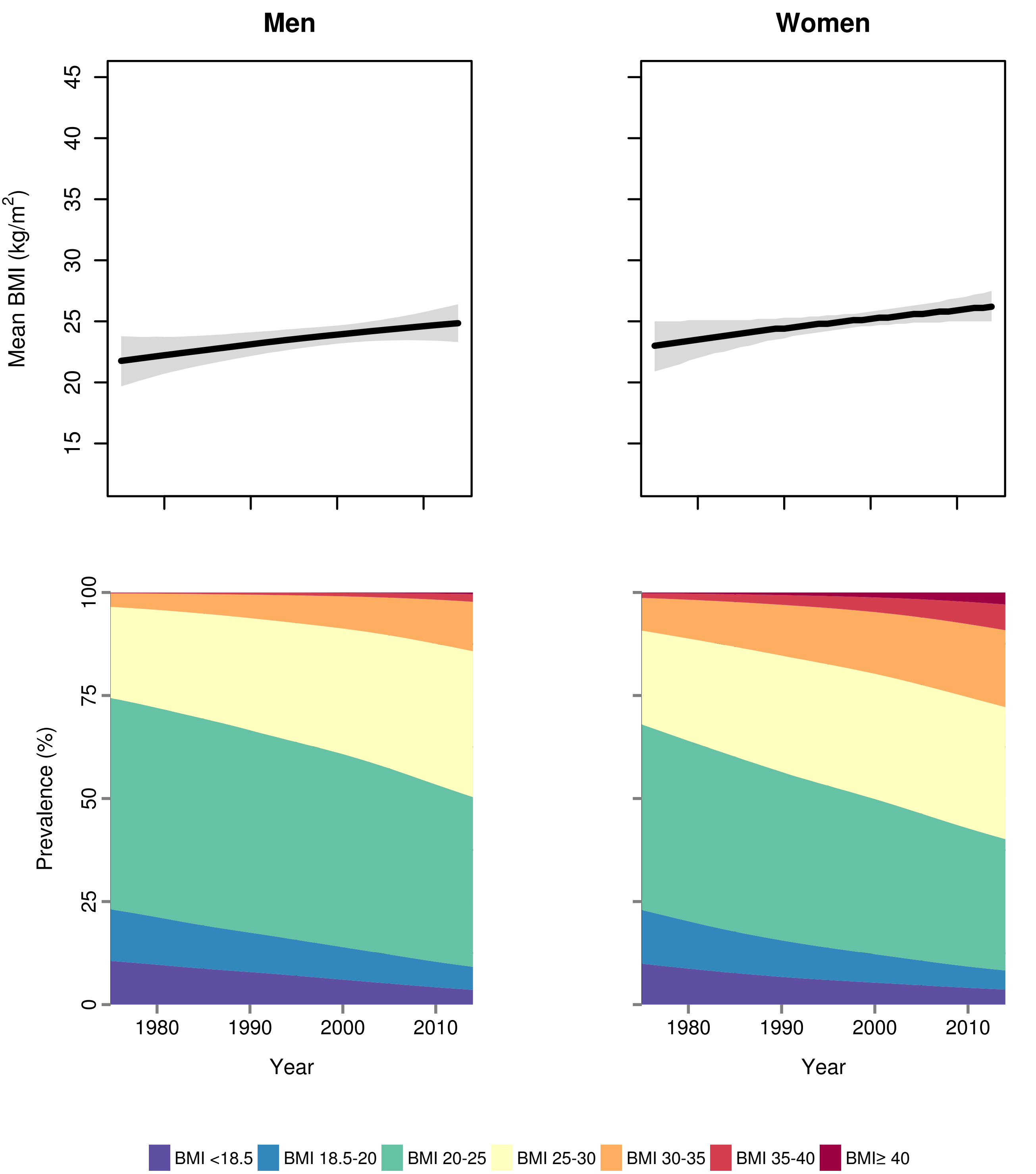

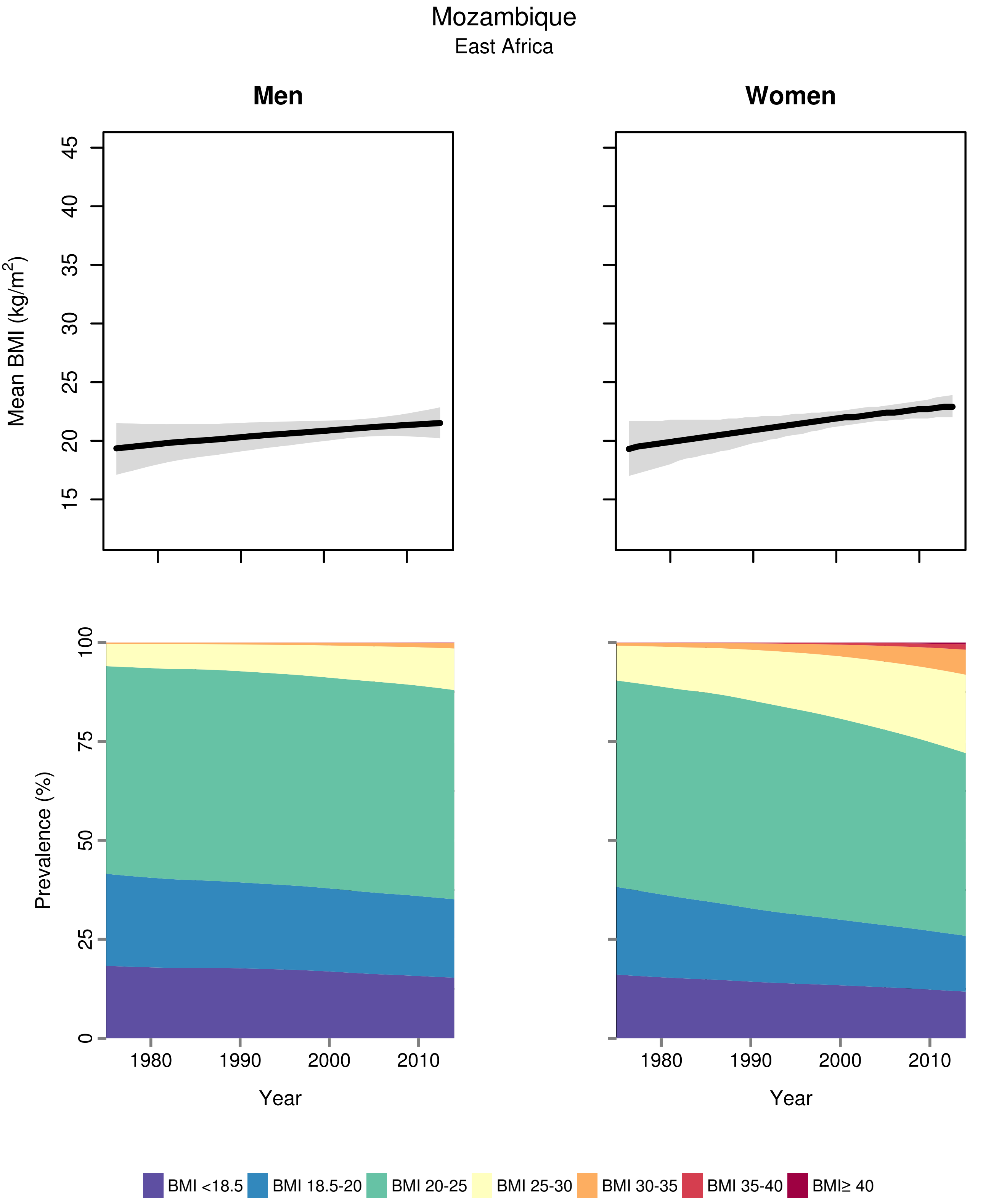
Mozambique
East Africa
Men
Women
Mean BMI (kg/m2)
45
40
35
30
25
20
15
Prevalence (%)
100
75
50
25
0
1980
1990
2000
2010
Year
BMI <18.5
BMI 18.5-20
BMI 20-25
BMI 25-30
BMI 30-35
BMI 35-40
BMI≥ 40

Myanmar

South East Asia

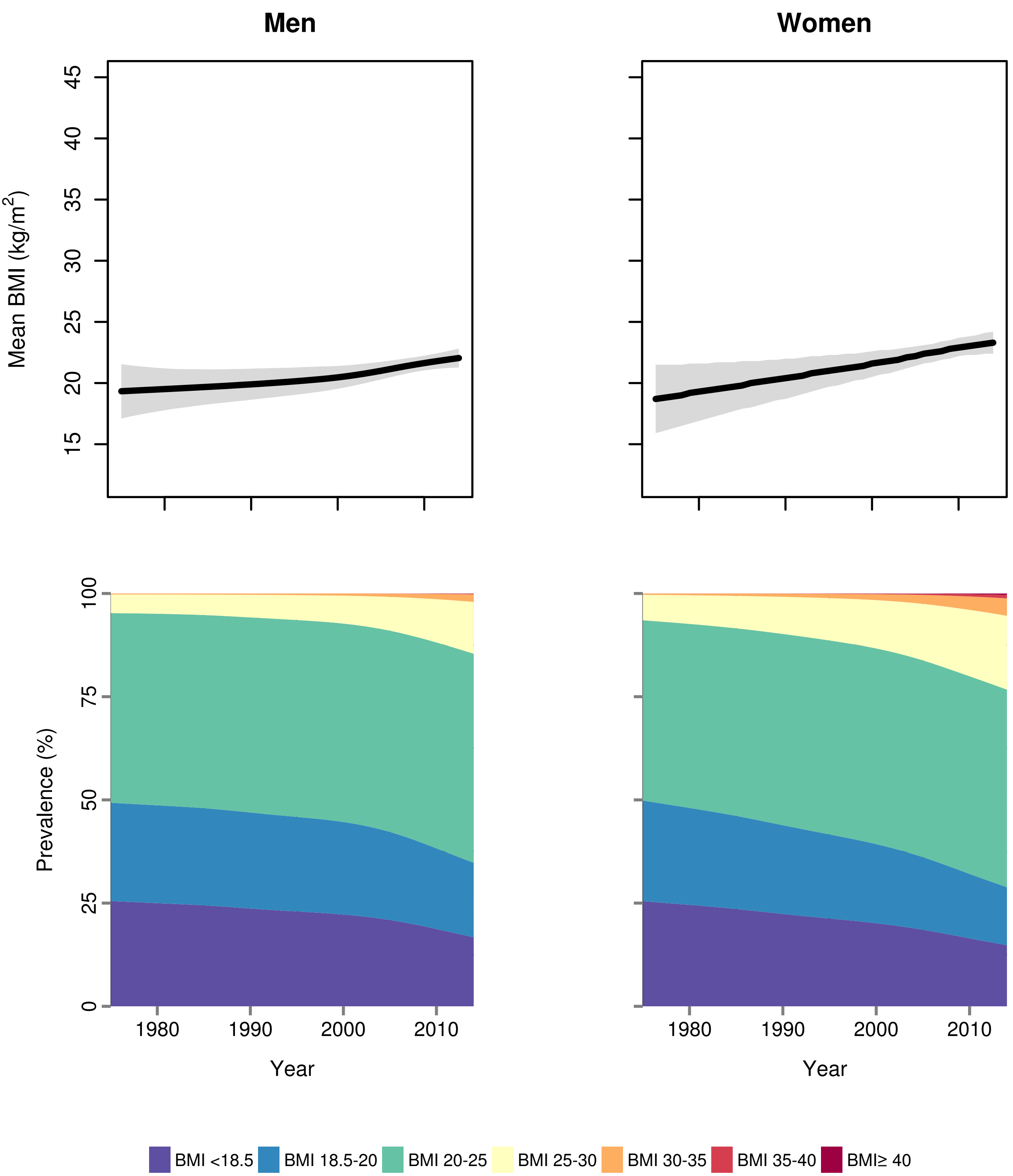

Namibia

Southern Africa

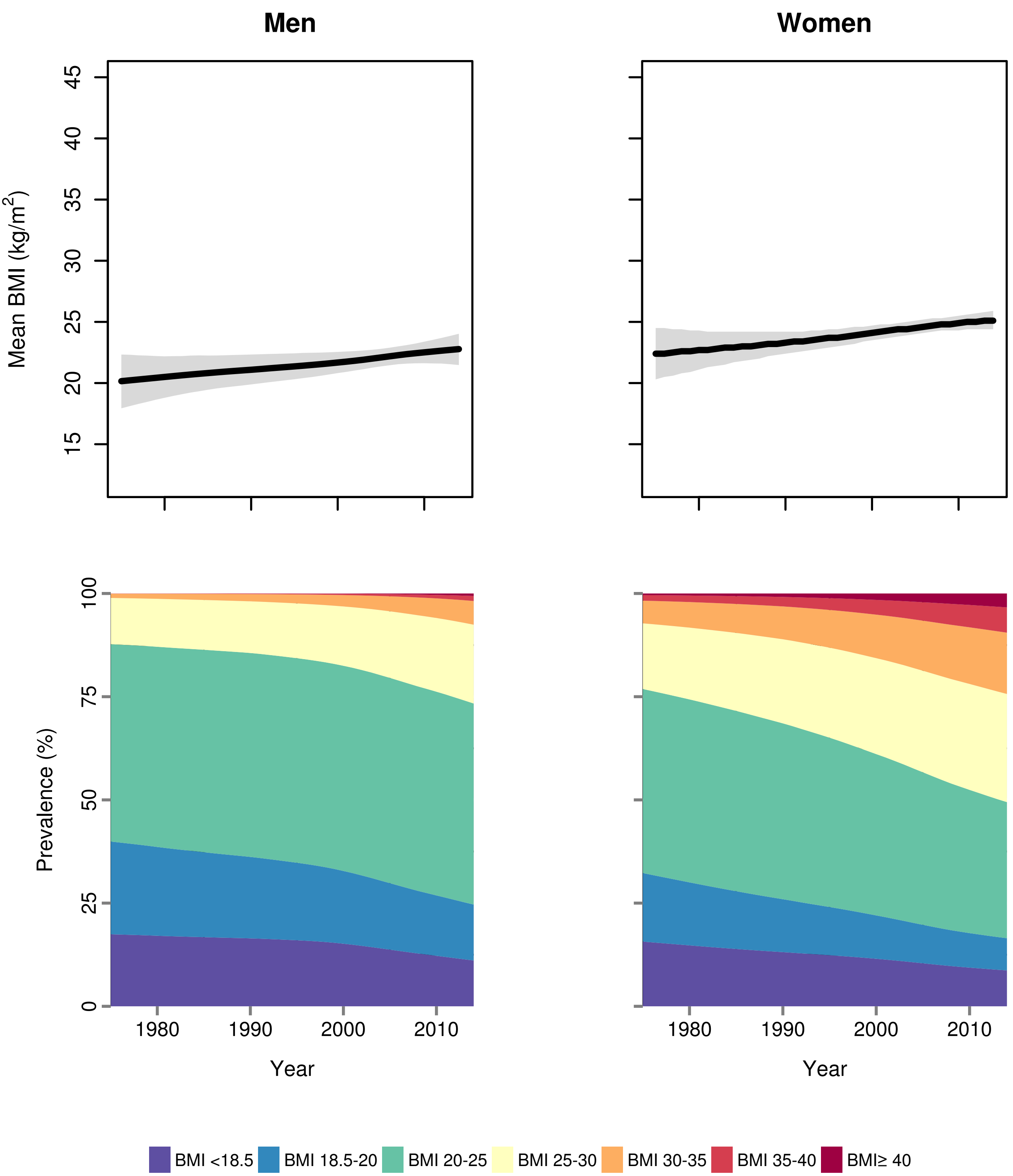

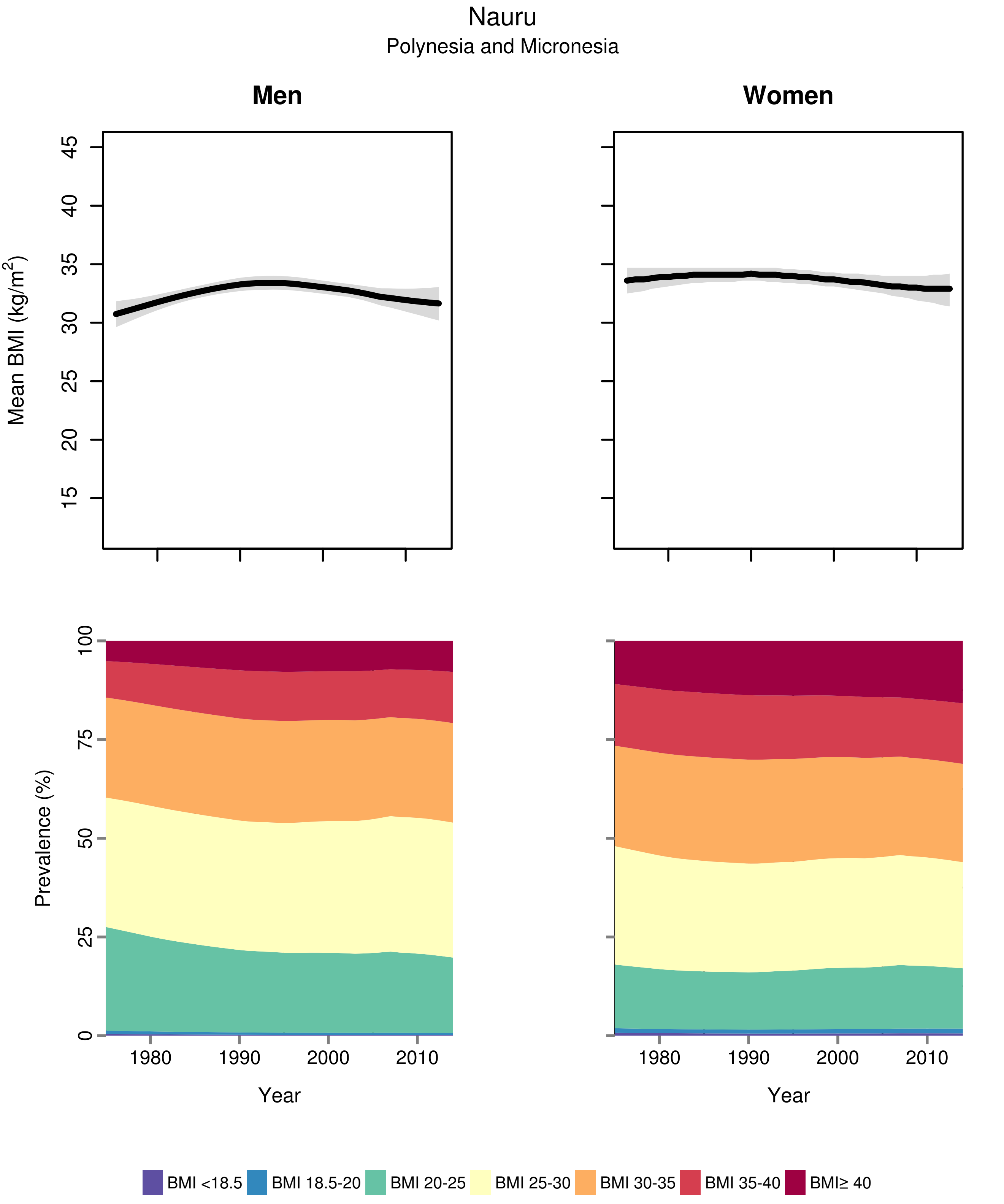
Nauru
Polynesia and Micronesia
Men
Women
Mean BMI (kg/m2)
45
40
35
30
25
20
15
Prevalence (%)
100
75
50
25
0
1980
1990
2000
2010
Year
BMI <18.5
BMI 18.5-20
BMI 20-25
BMI 25-30
BMI 30-35
BMI 35-40
BMI≥ 40

Nepal

South Asia

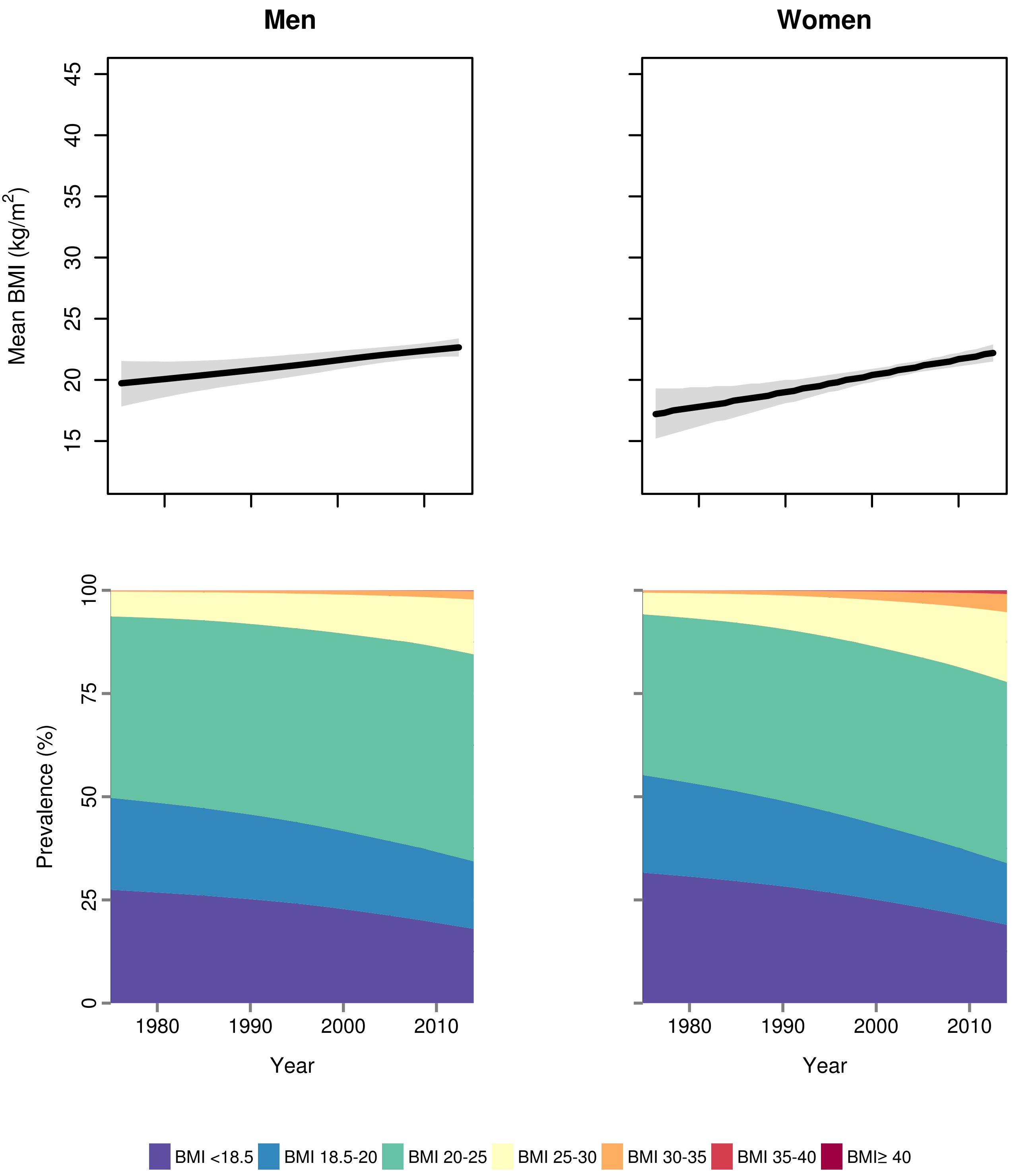

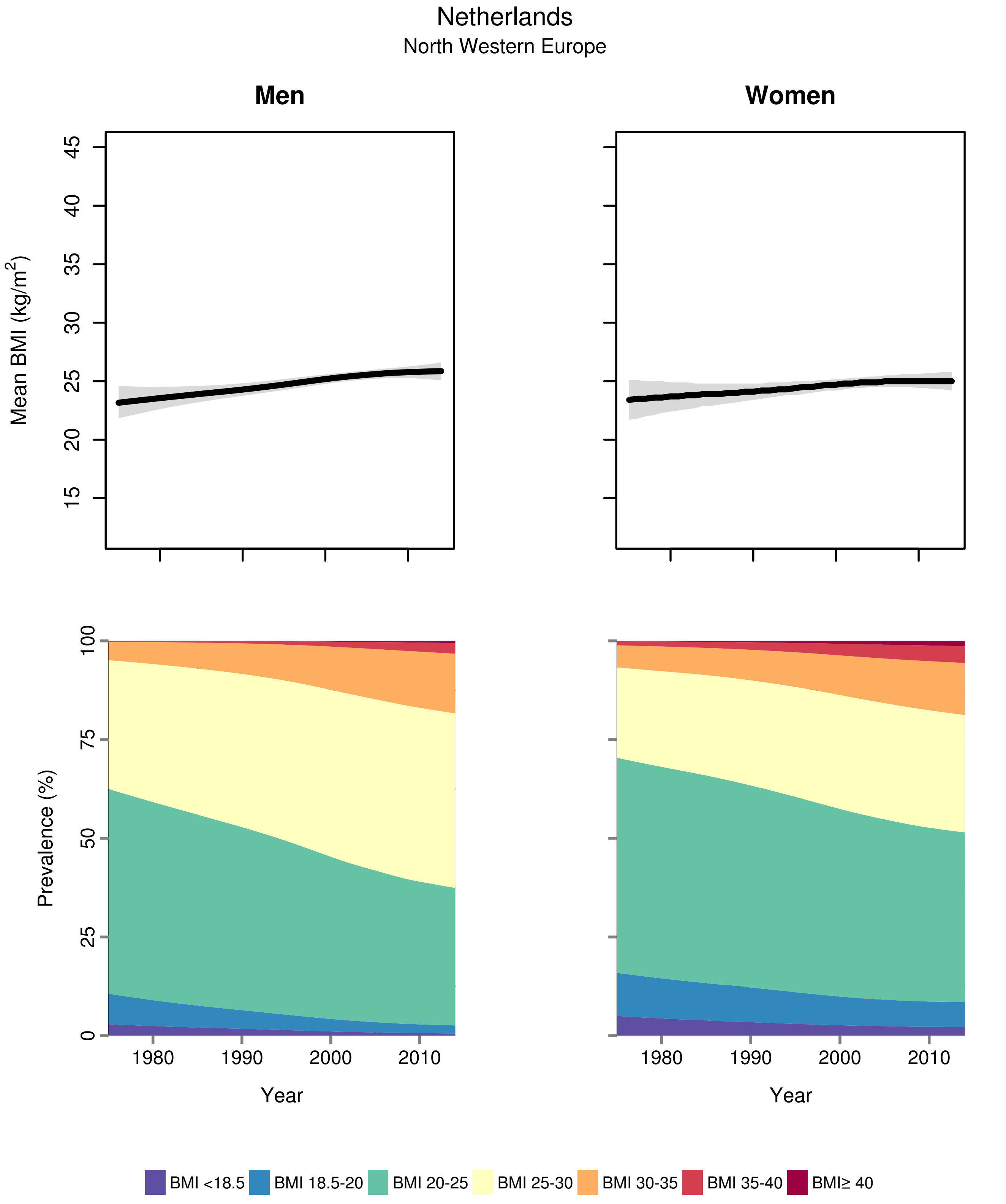
Netherlands
North Western Europe
Men
Women
Mean BMI (kg/m2)
45
40
35
30
25
20
15
Prevalence (%)
100
75
50
25
0
1980
1990
2000
2010
Year
BMI <18.5
BMI 18.5-20
BMI 20-25
BMI 25-30
BMI 30-35
BMI 35-40
BMI≥ 40

New Zealand

High-income English-speaking countries

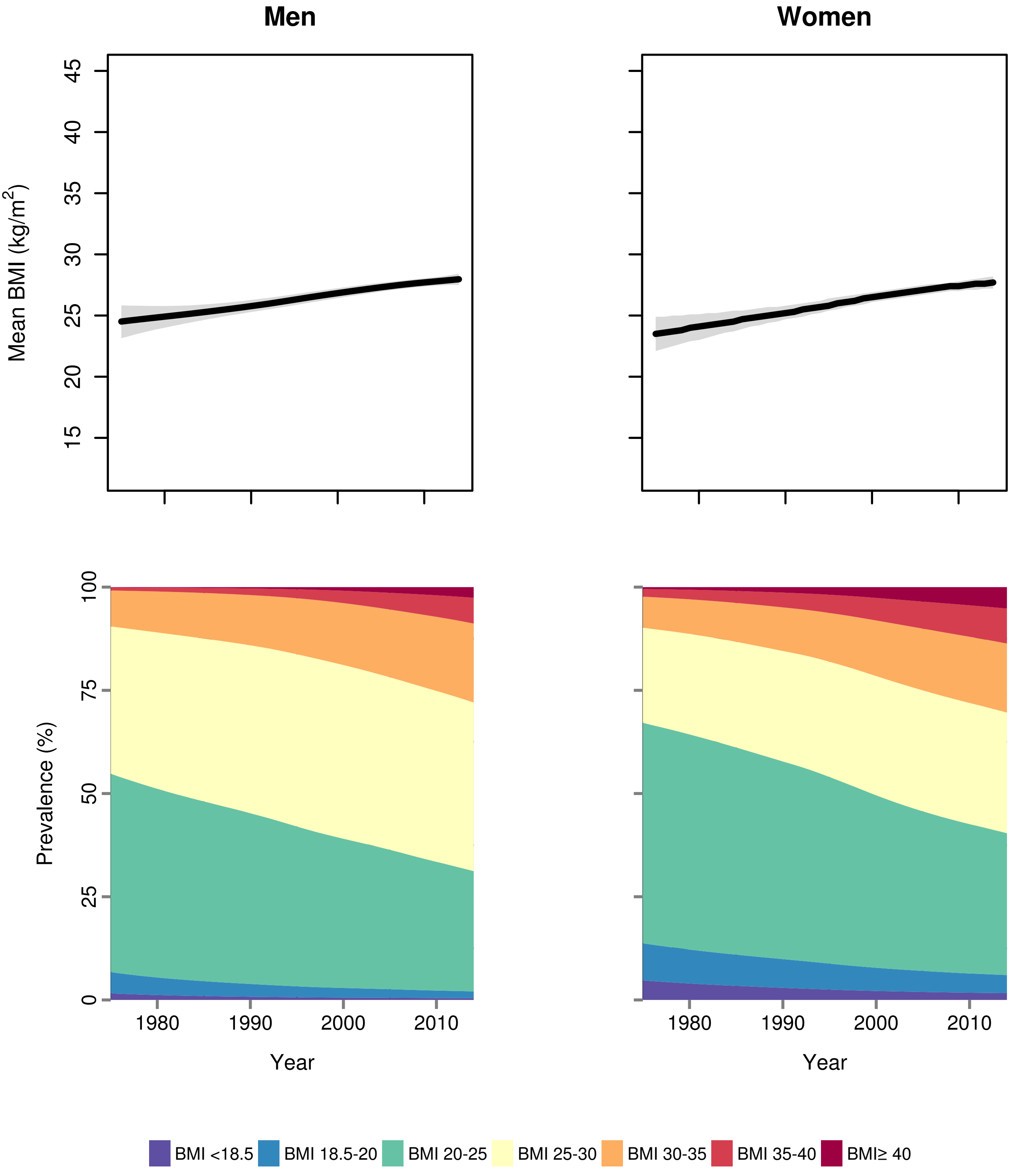

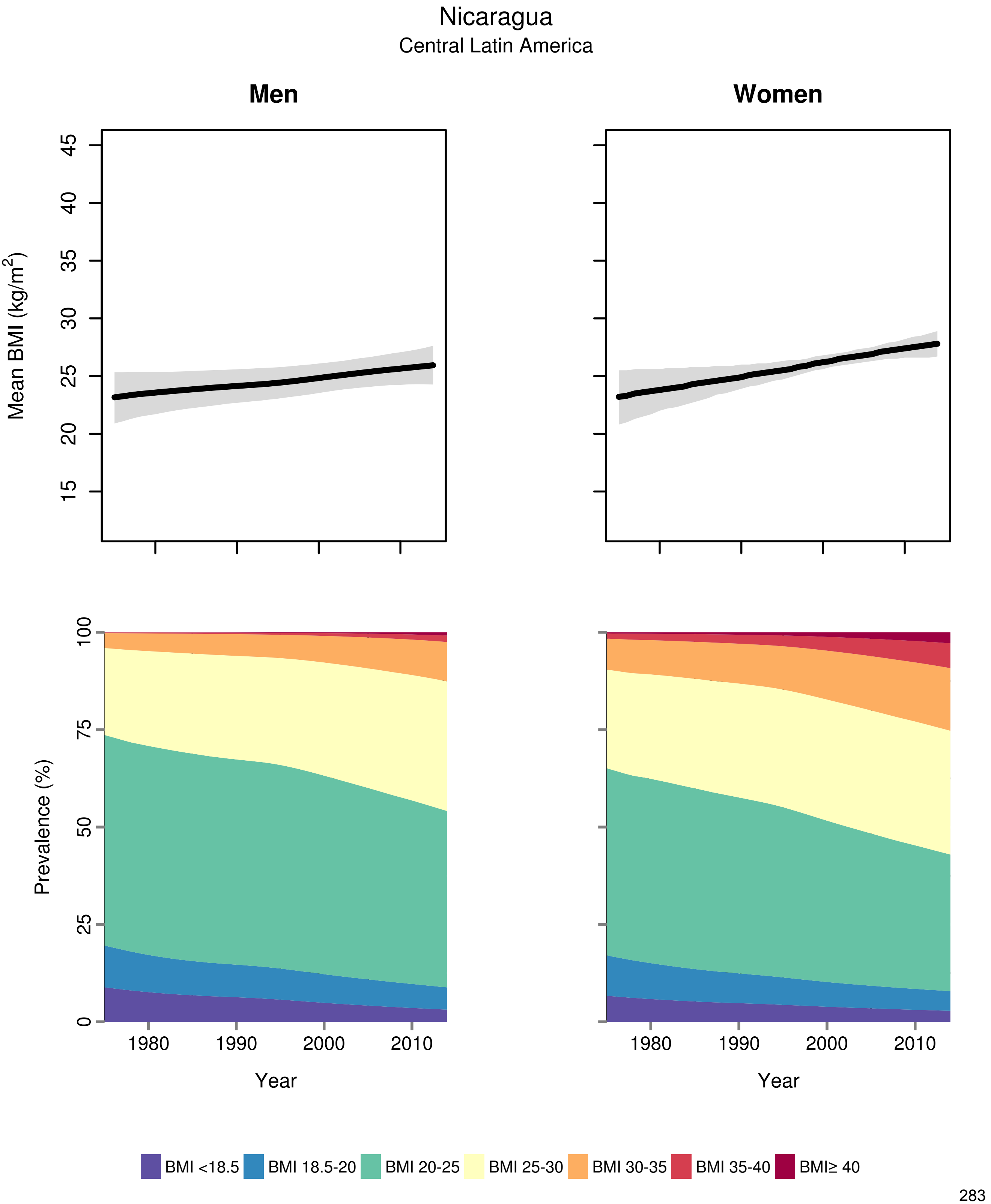
Nicaragua
Central Latin America
Men
Women
Mean BMI (kg/m²)
45
40
35
30
25
20
15
Prevalence (%)
100
75
50
25
0
1980
1990
2000
2010
Year
BMI <18.5
BMI 18.5-20
BMI 20-25
BMI 25-30
BMI 30-35
BMI 35-40
BMI≥ 40

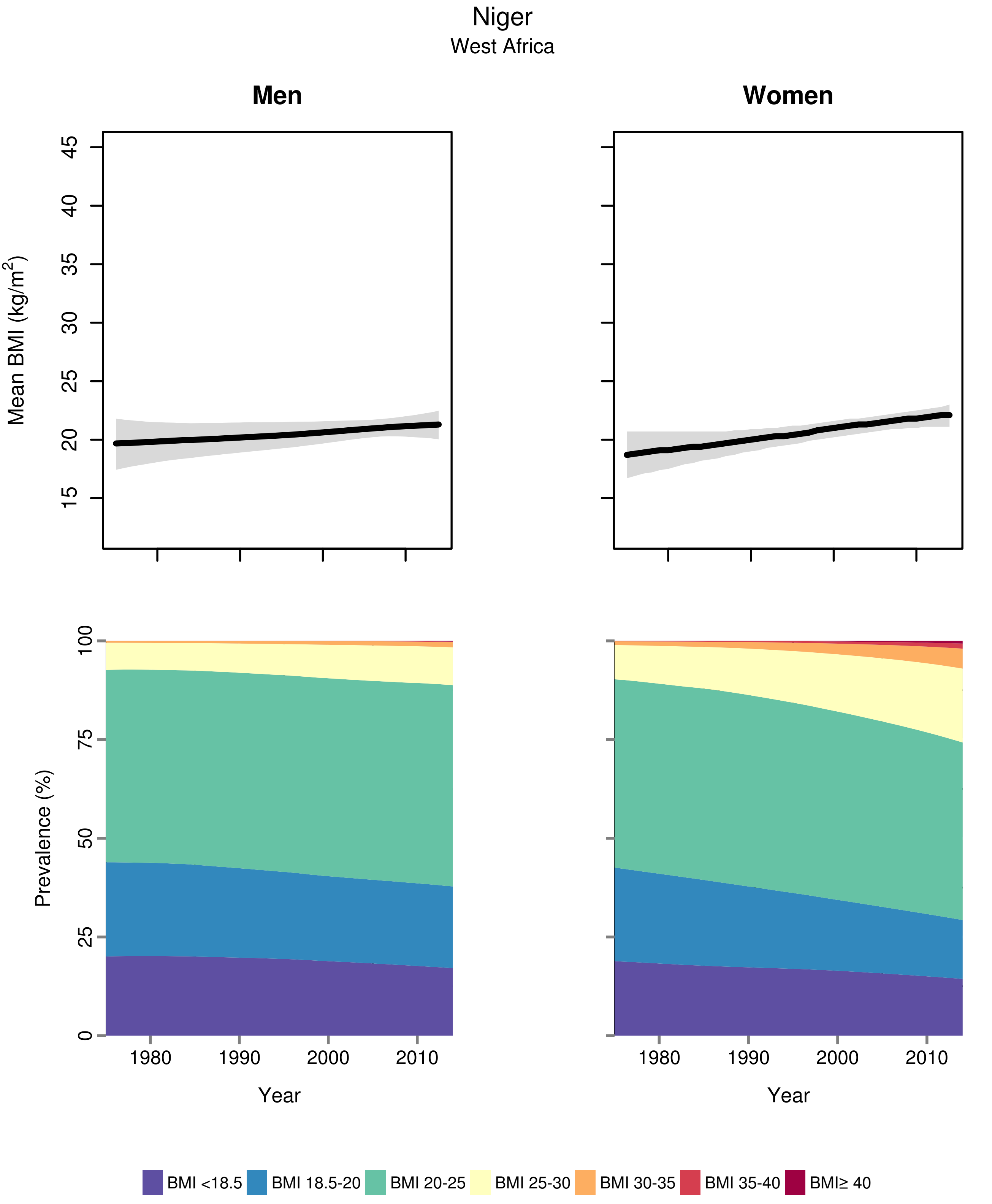
Niger
West Africa
Men
Women
Mean BMI (kg/m²)
45
40
35
30
25
20
15
100
75
50
25
0
Prevalence (%)
1980
1990
2000
2010
Year
BMI <18.5
BMI 18.5-20
BMI 20-25
BMI 25-30
BMI 30-35
BMI 35-40
BMI≥ 40

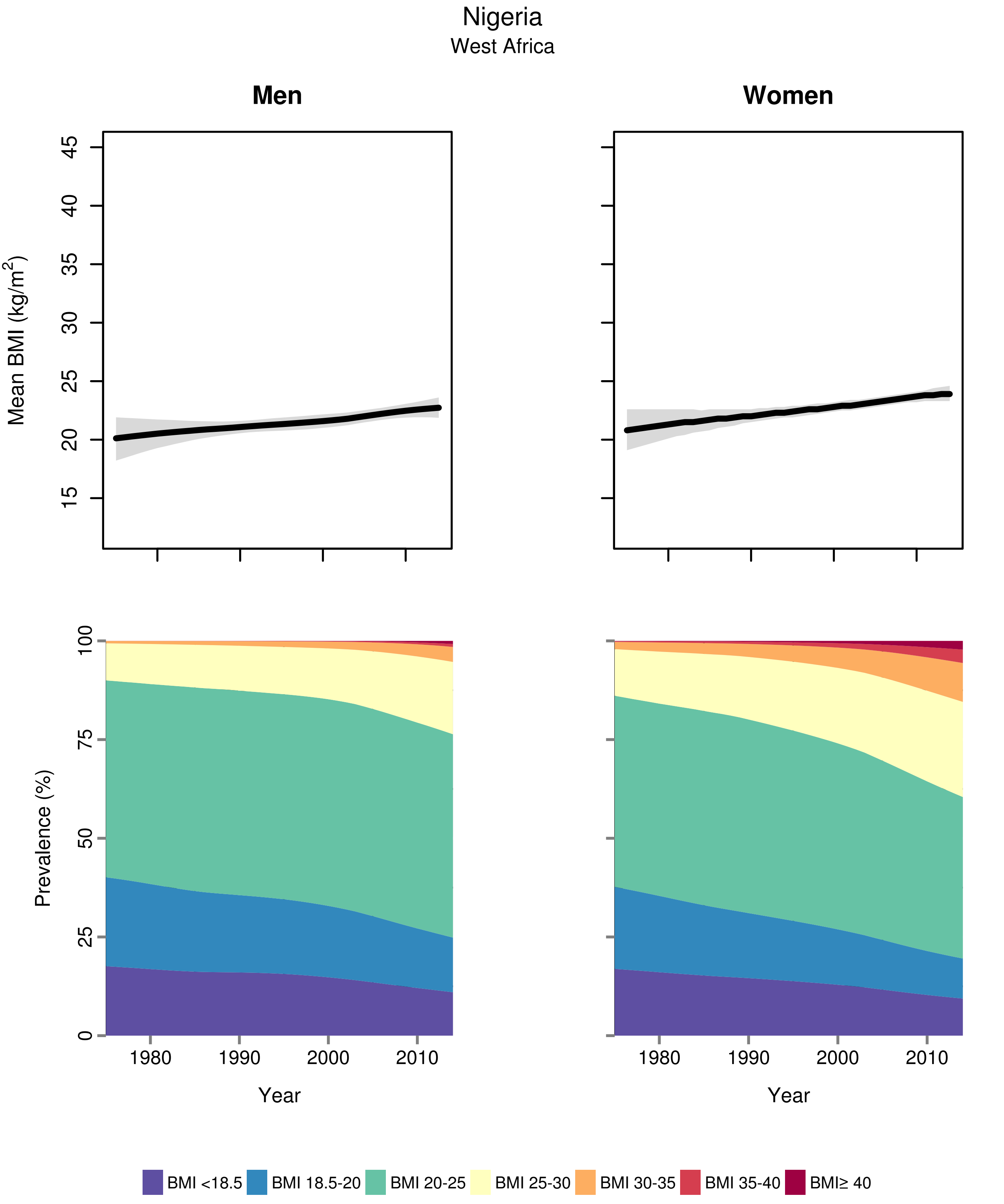

Nigeria
West Africa
Men
Women
Mean BMI (kg/m2)
45
40
35
30
25
20
15
Prevalence (%)
100
75
50
25
0
1980
1990
2000
2010
Year
BMI <18.5
BMI 18.5-20
BMI 20-25
BMI 25-30
BMI 30-35
BMI 35-40
BMI≥ 40

Niue

Polynesia and Micronesia

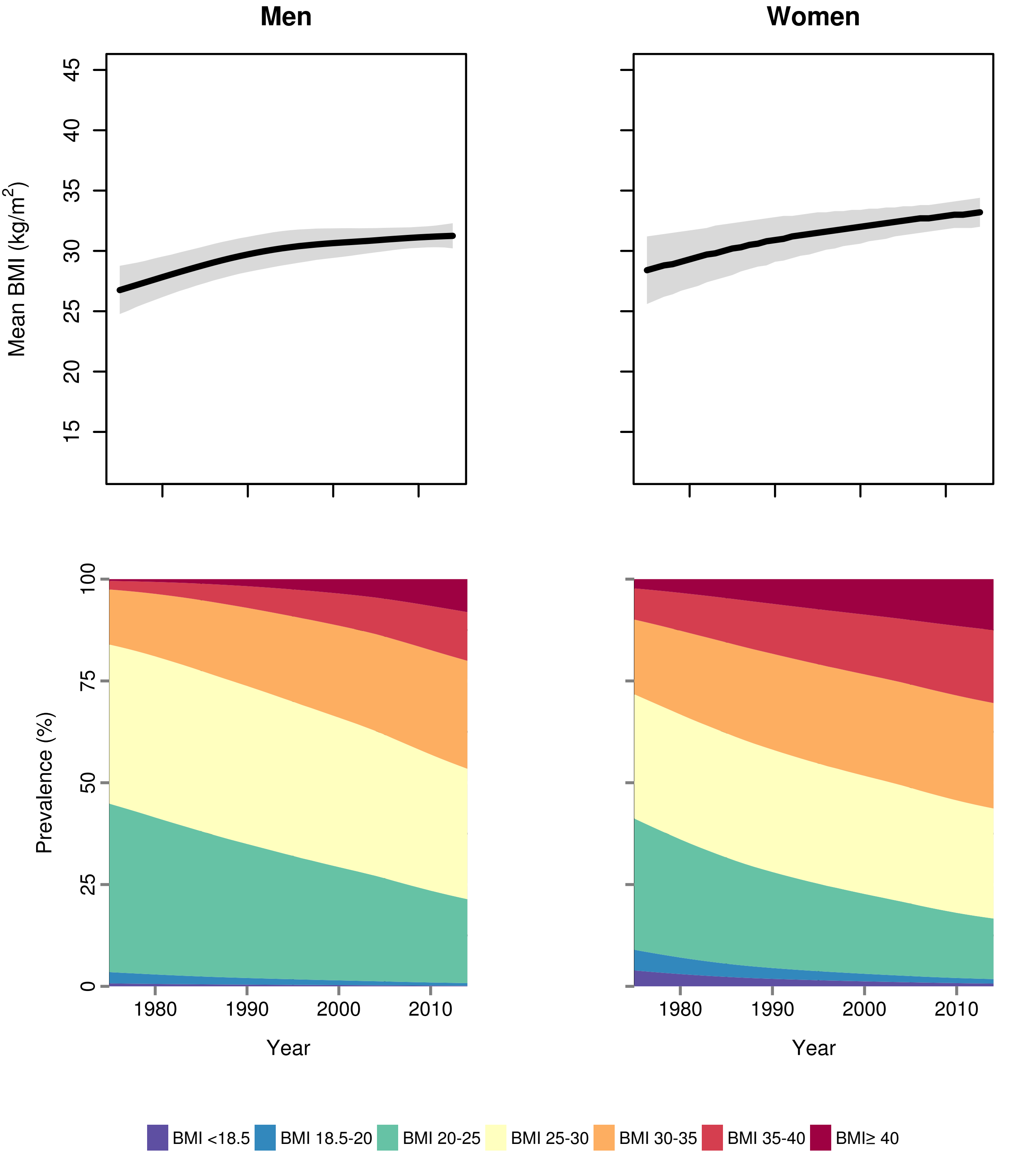

North Korea

East Asia

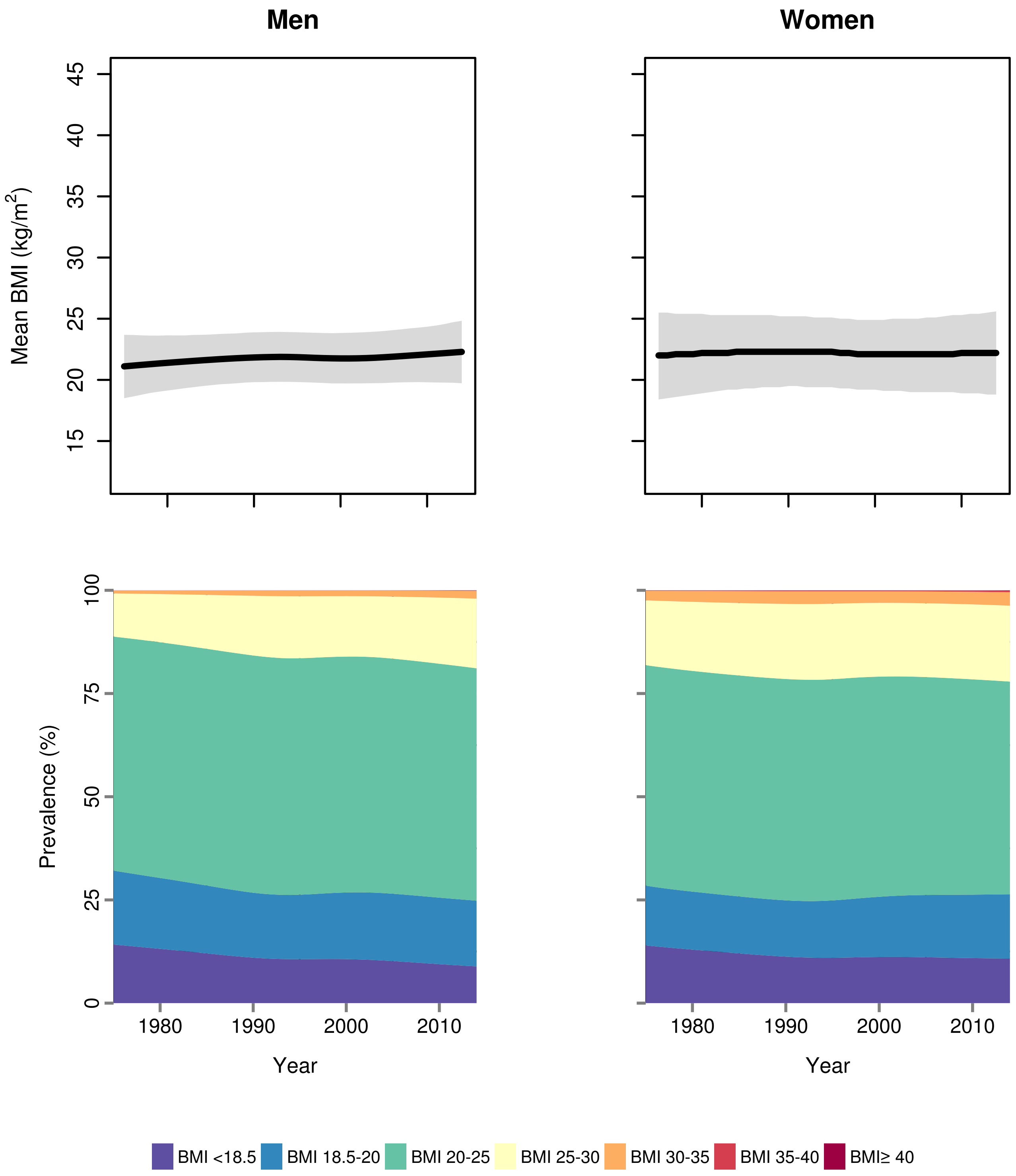

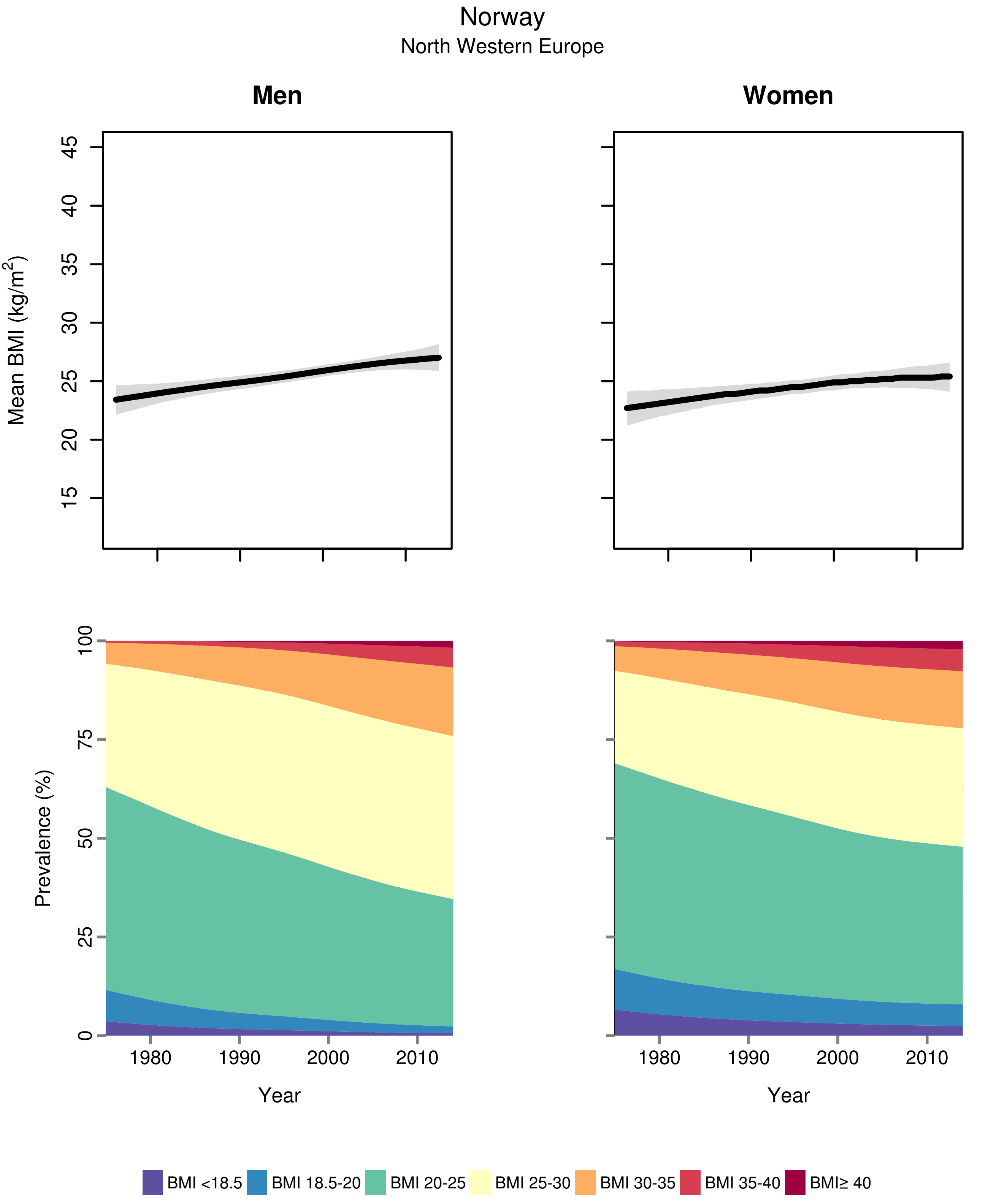
Norway
North Western Europe
Men
Women
Mean BMI (kg/m2)
45
40
35
30
25
20
15
Prevalence (%)
100
75
50
25
0
1980
1990
2000
2010
Year
BMI <18.5
BMI 18.5-20
BMI 20-25
BMI 25-30
BMI 30-35
BMI 35-40
BMI≥ 40

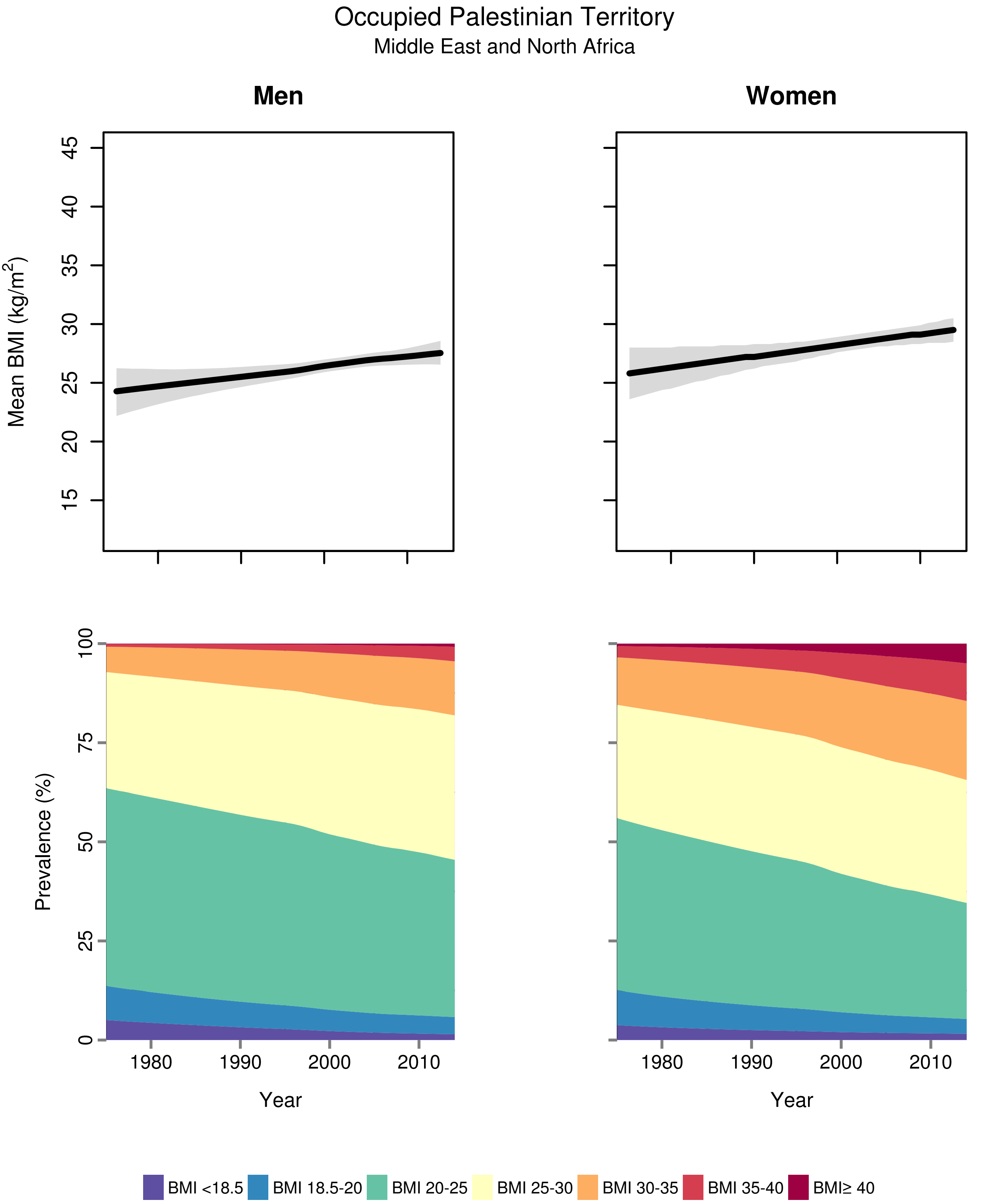

Occupied Palestinian Territory
Middle East and North Africa
Men
Women
Mean BMI (kg/m2)
45
40
35
30
25
20
15
100
75
50
25
0
Prevalence (%)
1980
1990
2000
2010
Year
BMI <18.5
BMI 18.5-20
BMI 20-25
BMI 25-30
BMI 30-35
BMI 35-40
BMI≥ 40

Oman

Middle East and North Africa

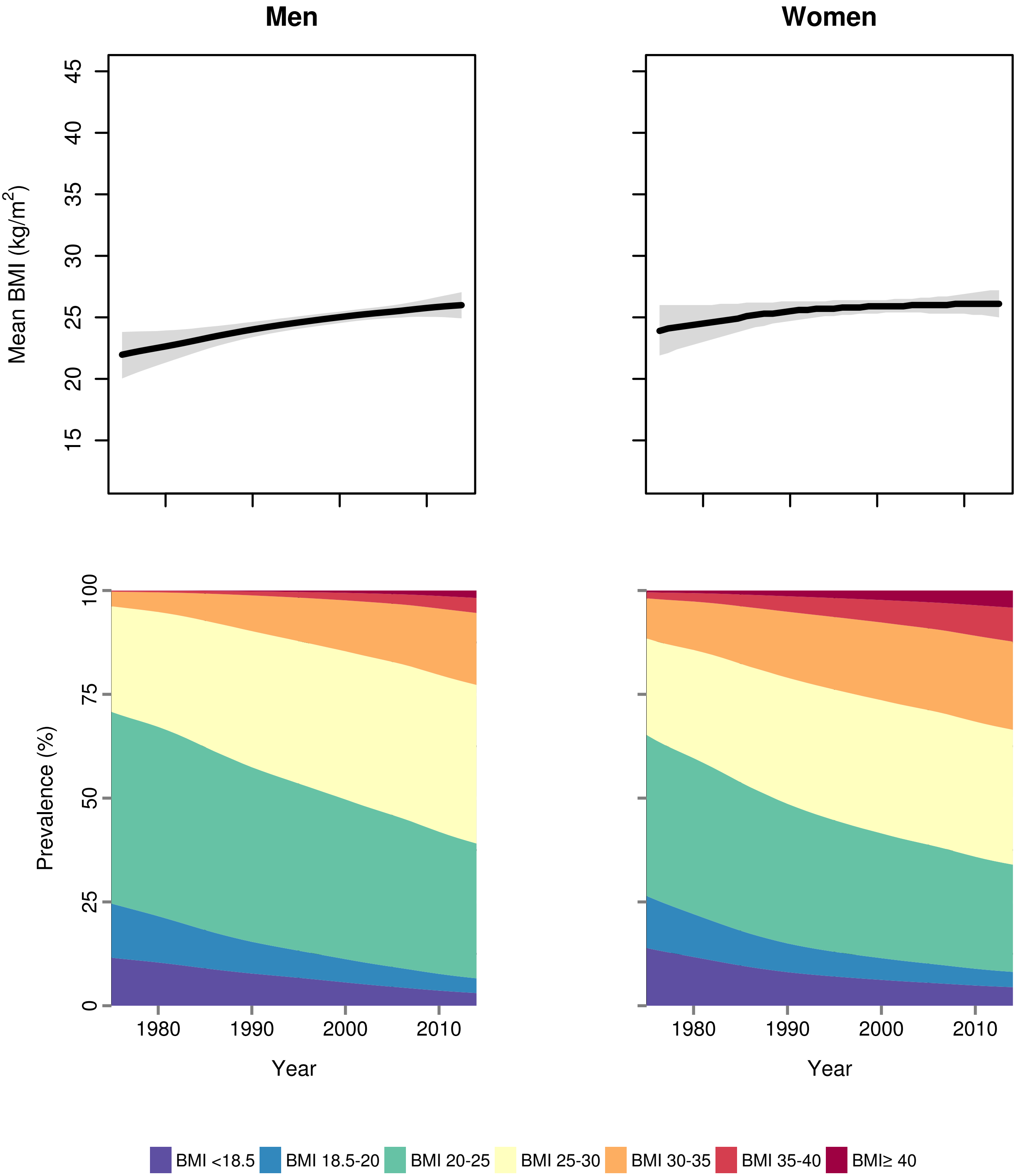

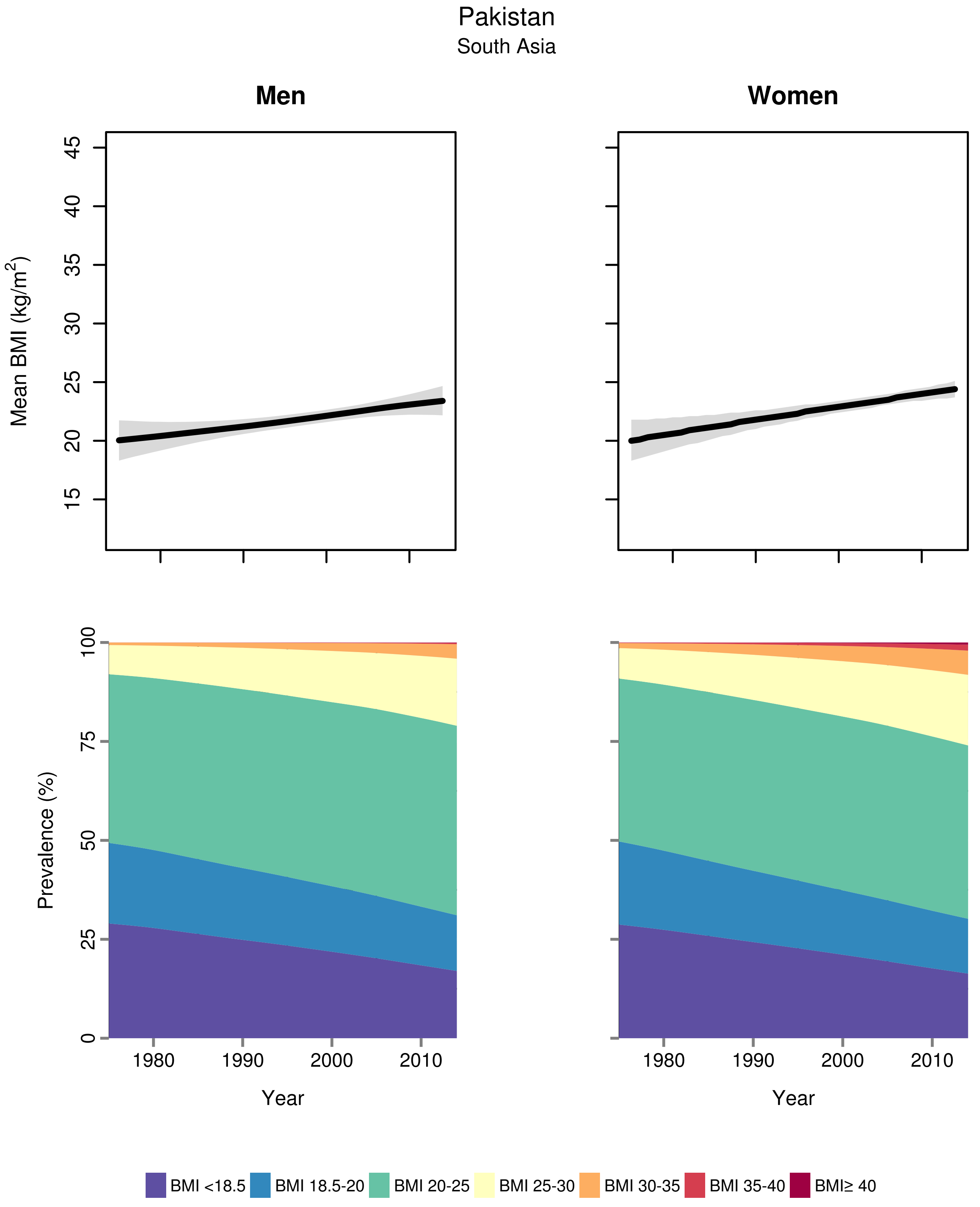

Pakistan
South Asia
Men
Women
Mean BMI (kg/m2)
45
40
35
30
25
20
15
Prevalence (%)
100
75
50
25
0
1980
1990
2000
2010
Year
BMI <18.5
BMI 18.5-20
BMI 20-25
BMI 25-30
BMI 30-35
BMI 35-40
BMI≥ 40

Palau

Polynesia and Micronesia

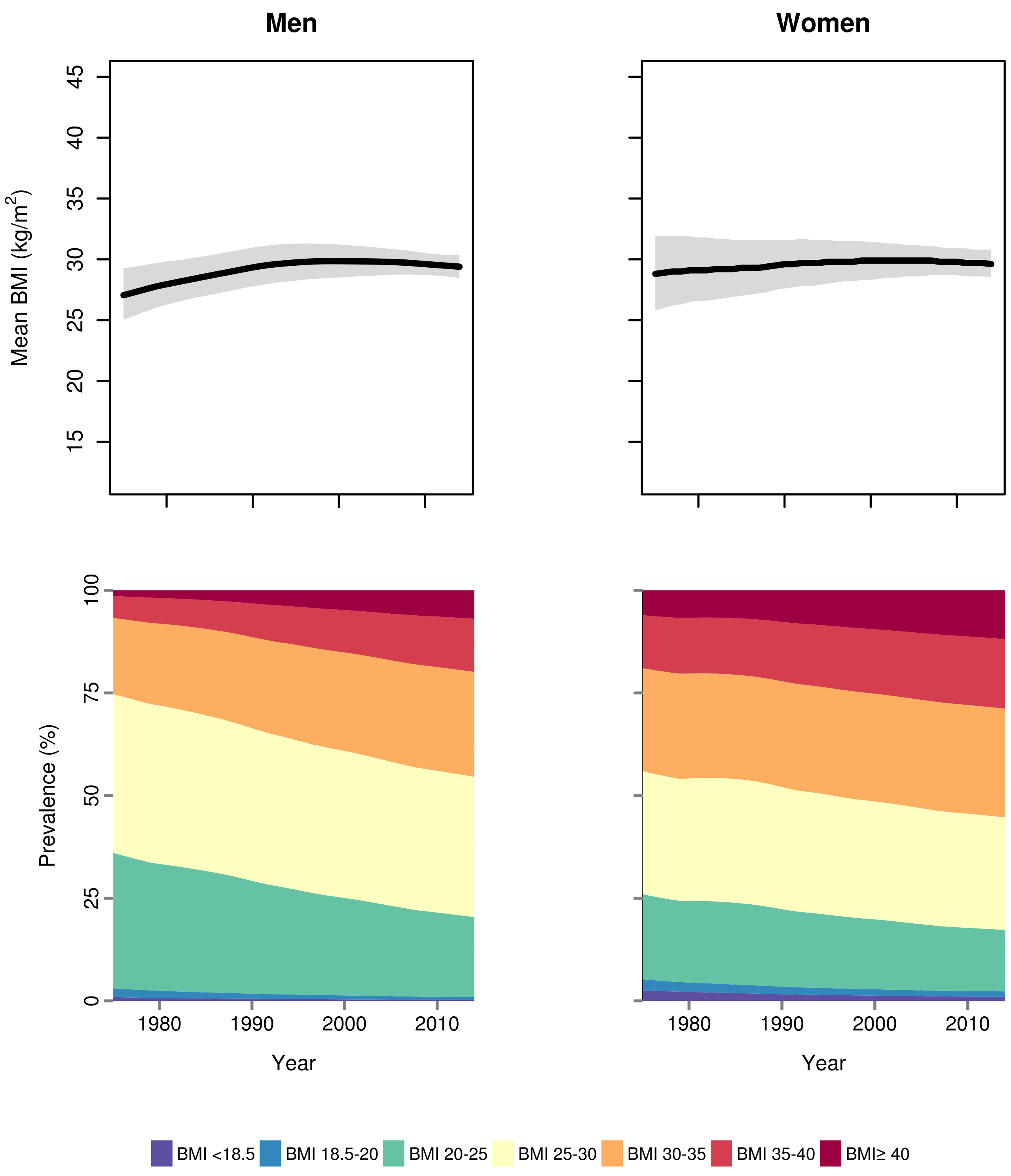

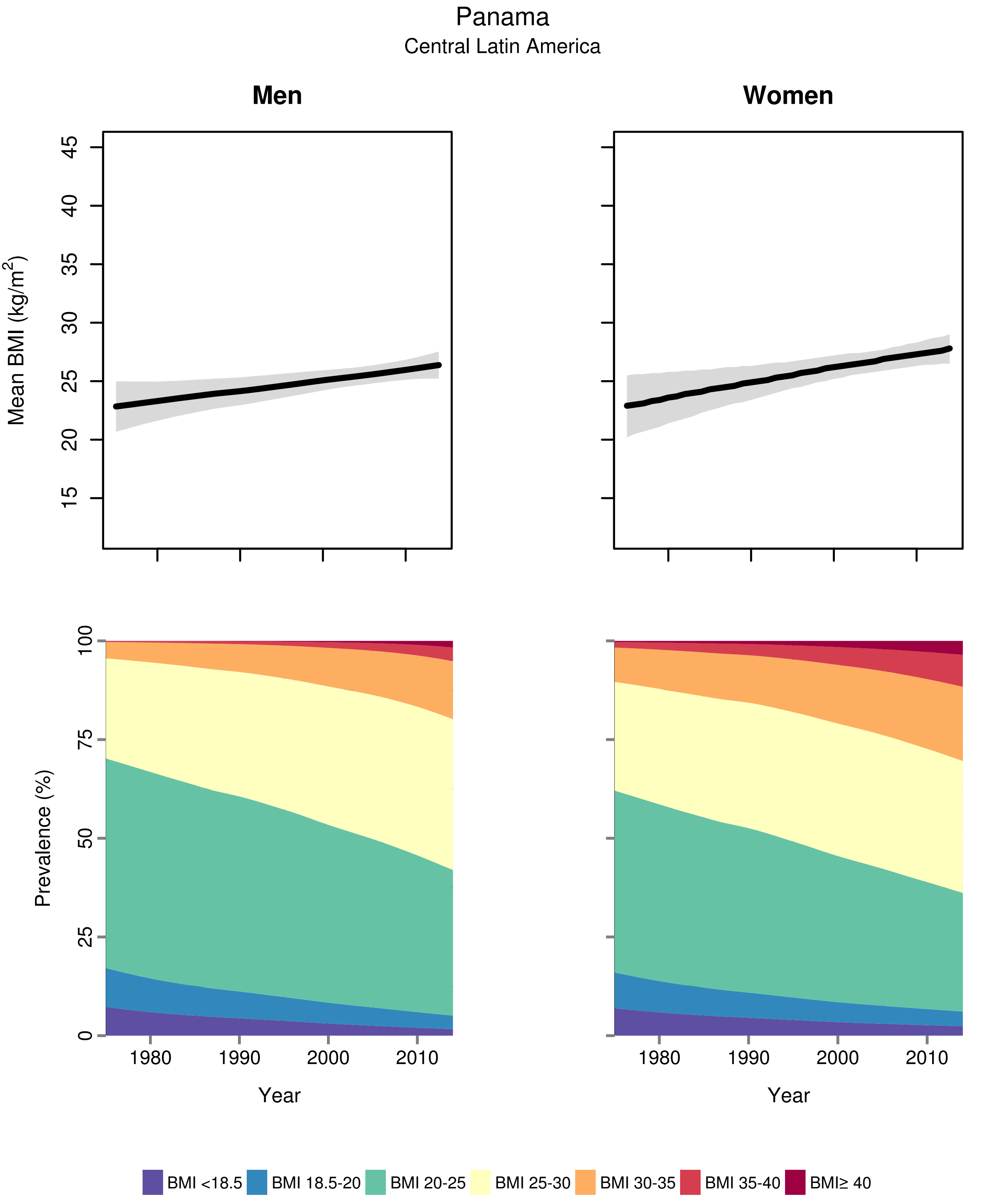
Panama
Central Latin America
Men
Women
Mean BMI (kg/m2)
45
40
35
30
25
20
15
100
75
50
25
0
Prevalence (%)
1980
1990
2000
2010
Year
BMI <18.5
BMI 18.5-20
BMI 20-25
BMI 25-30
BMI 30-35
BMI 35-40
BMI≥ 40

Papua New Guinea

Melanesia

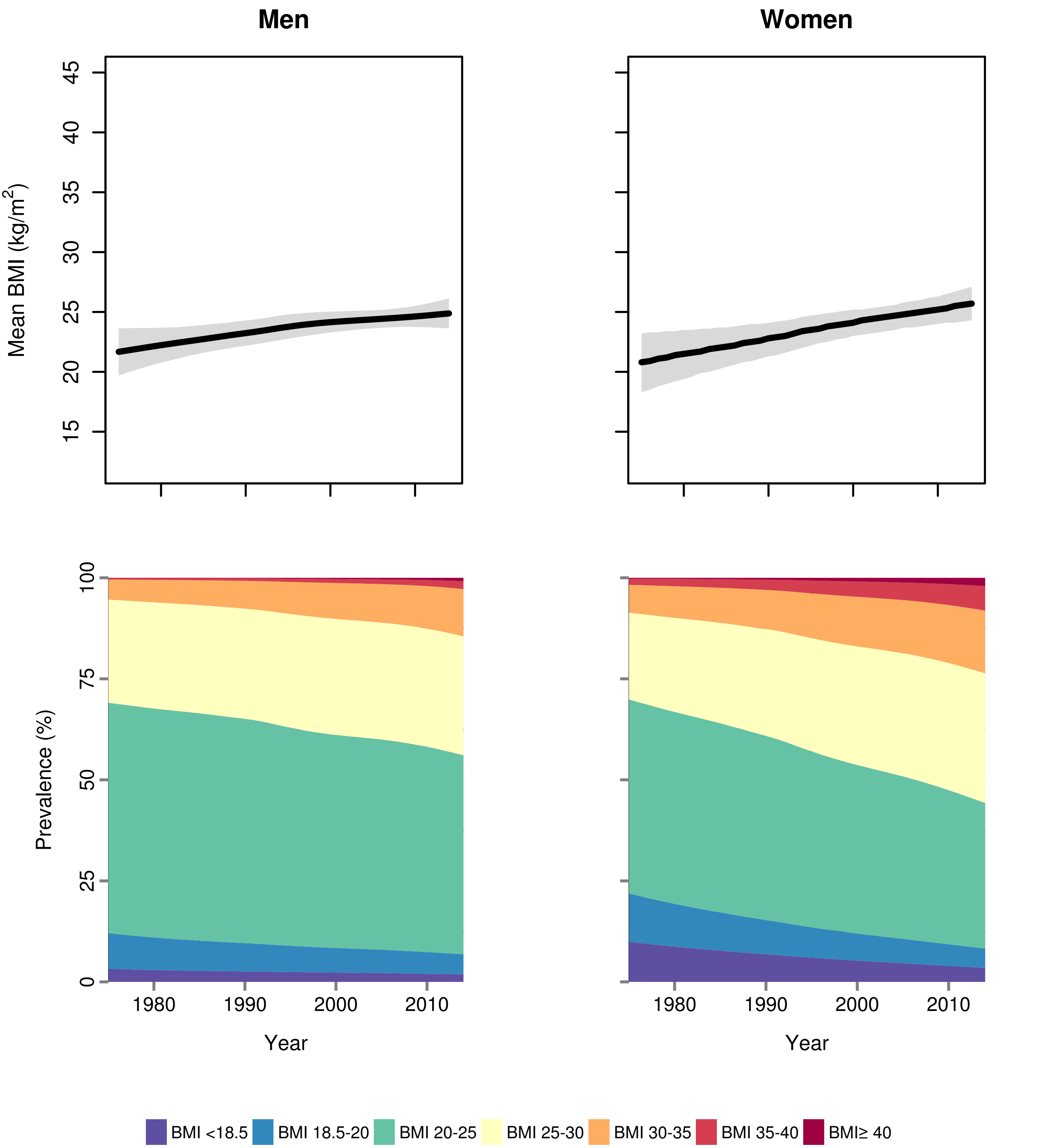

Paraguay

Southern Latin America

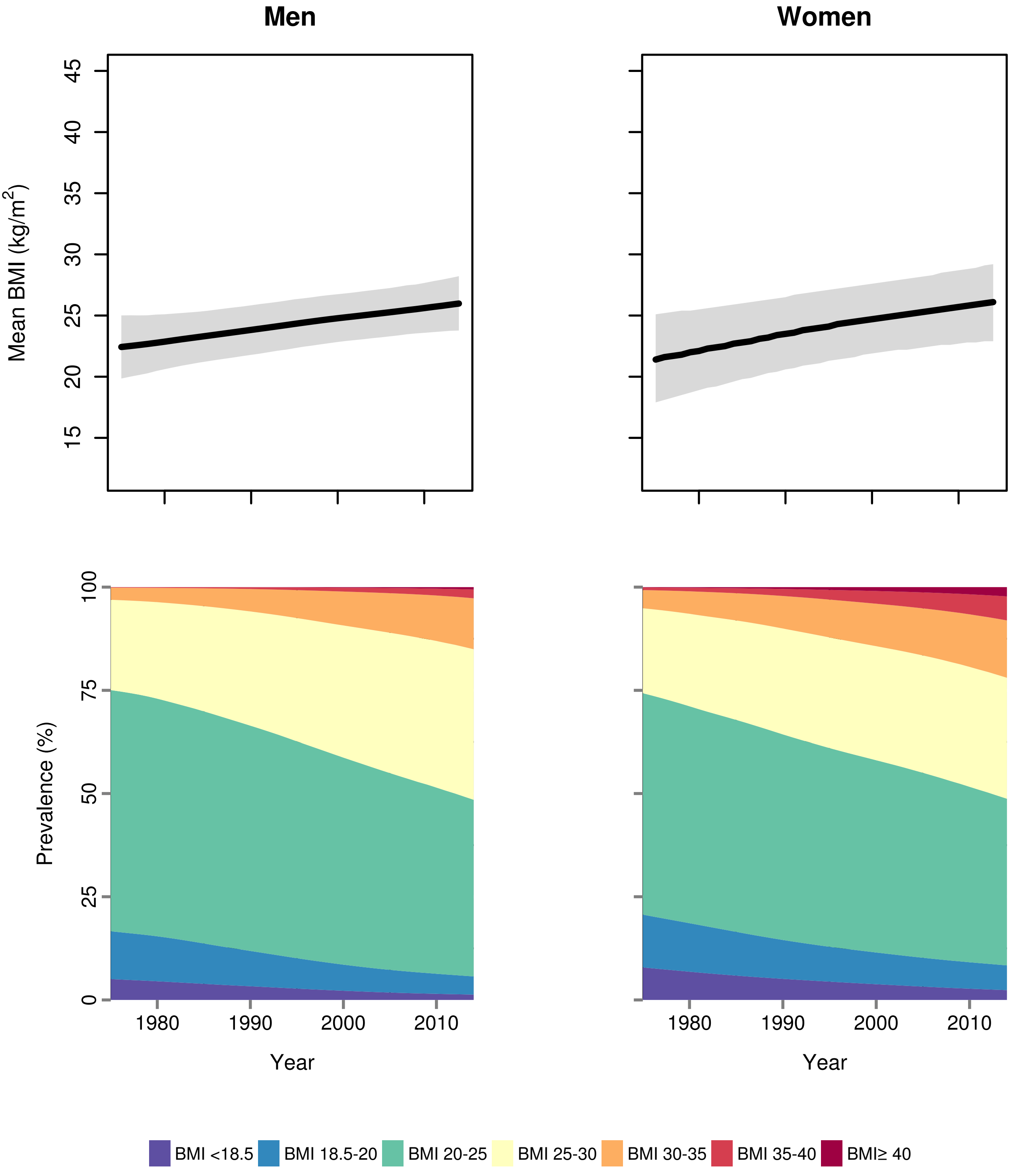

Peru

Andean Latin America

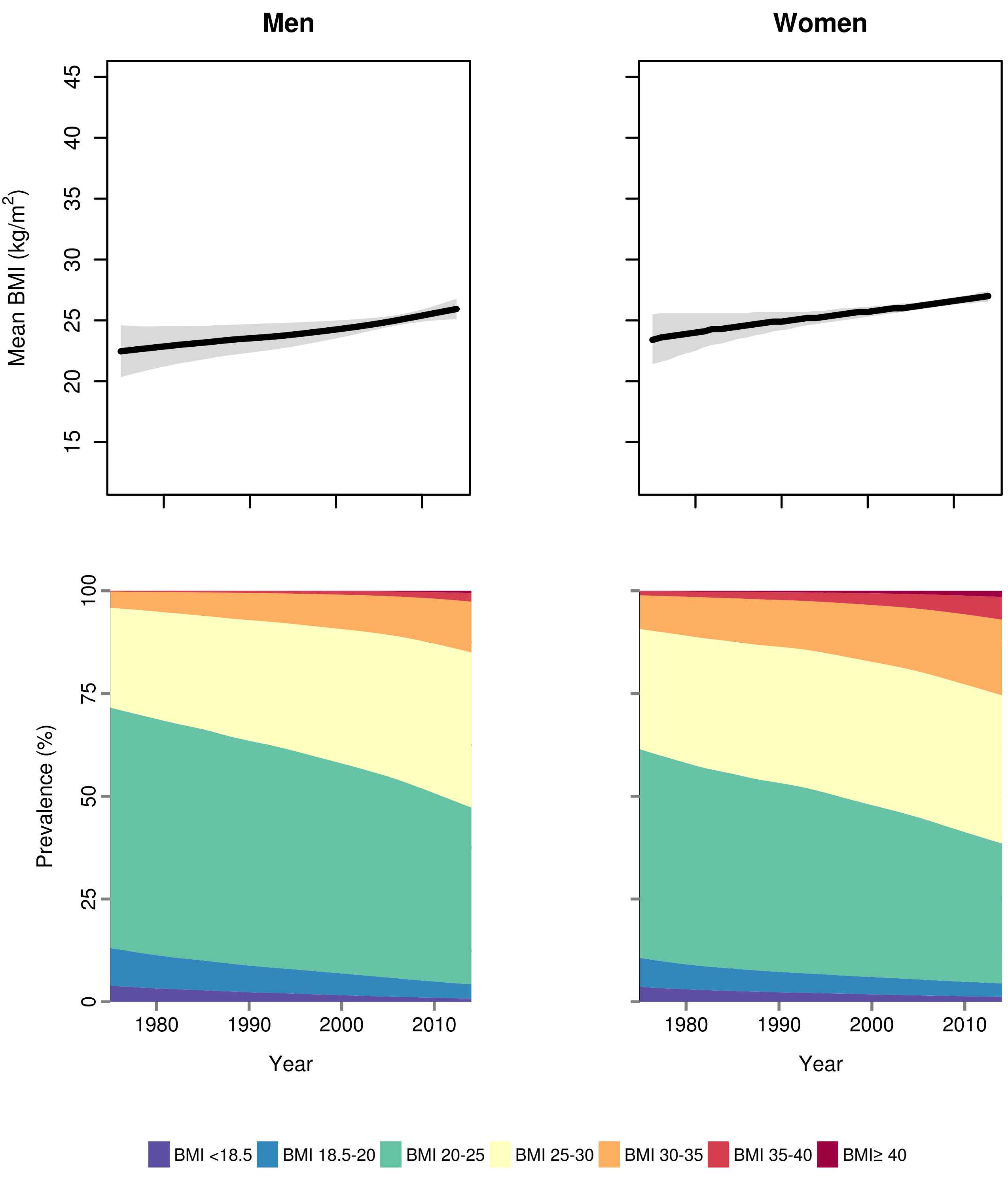

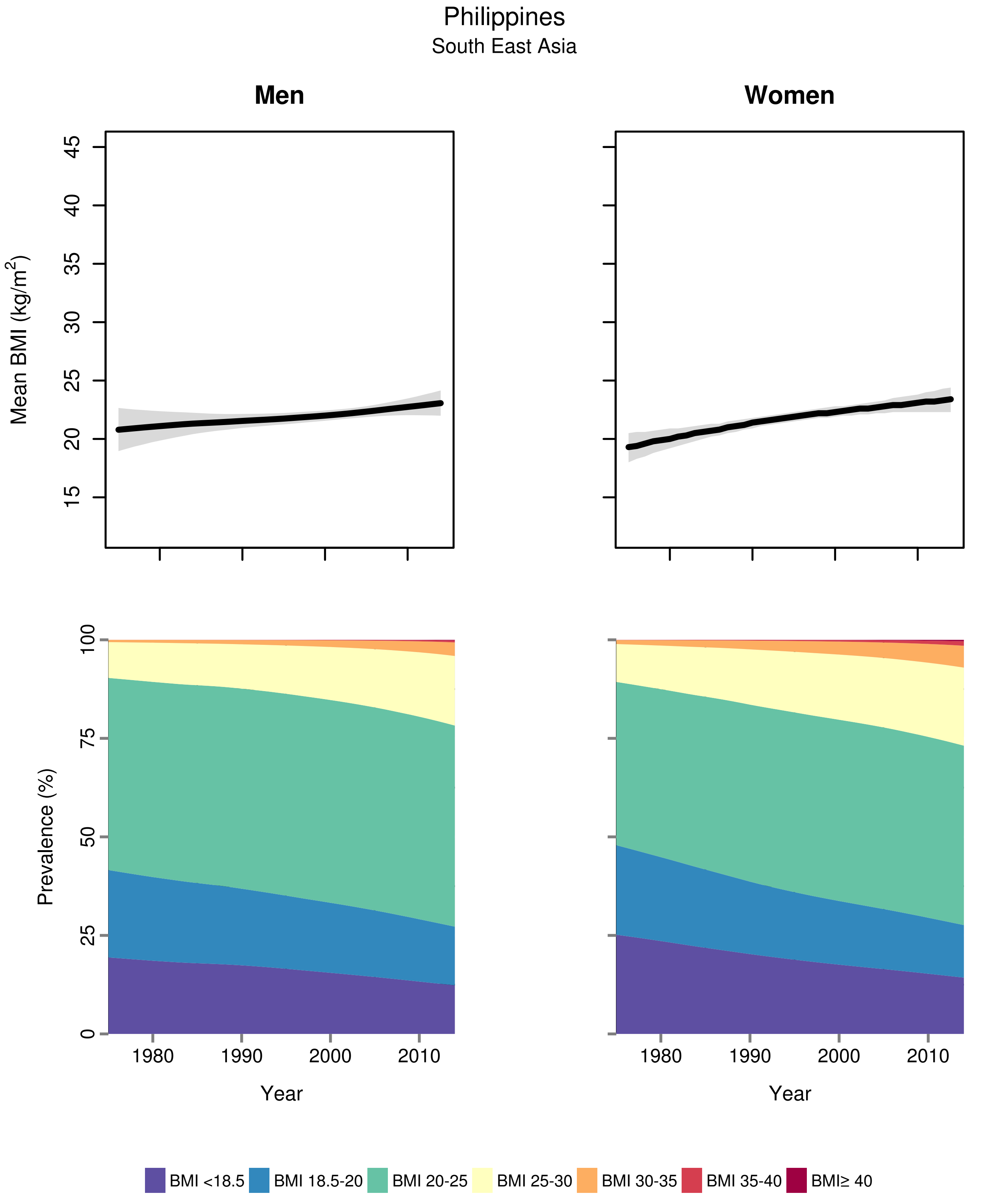

Philippines
South East Asia
Men
Women
Mean BMI (kg/m2)
45
40
35
30
25
20
15
Prevalence (%)
100
75
50
25
0
1980
1990
2000
2010
Year
BMI <18.5
BMI 18.5-20
BMI 20-25
BMI 25-30
BMI 30-35
BMI 35-40
BMI≥ 40

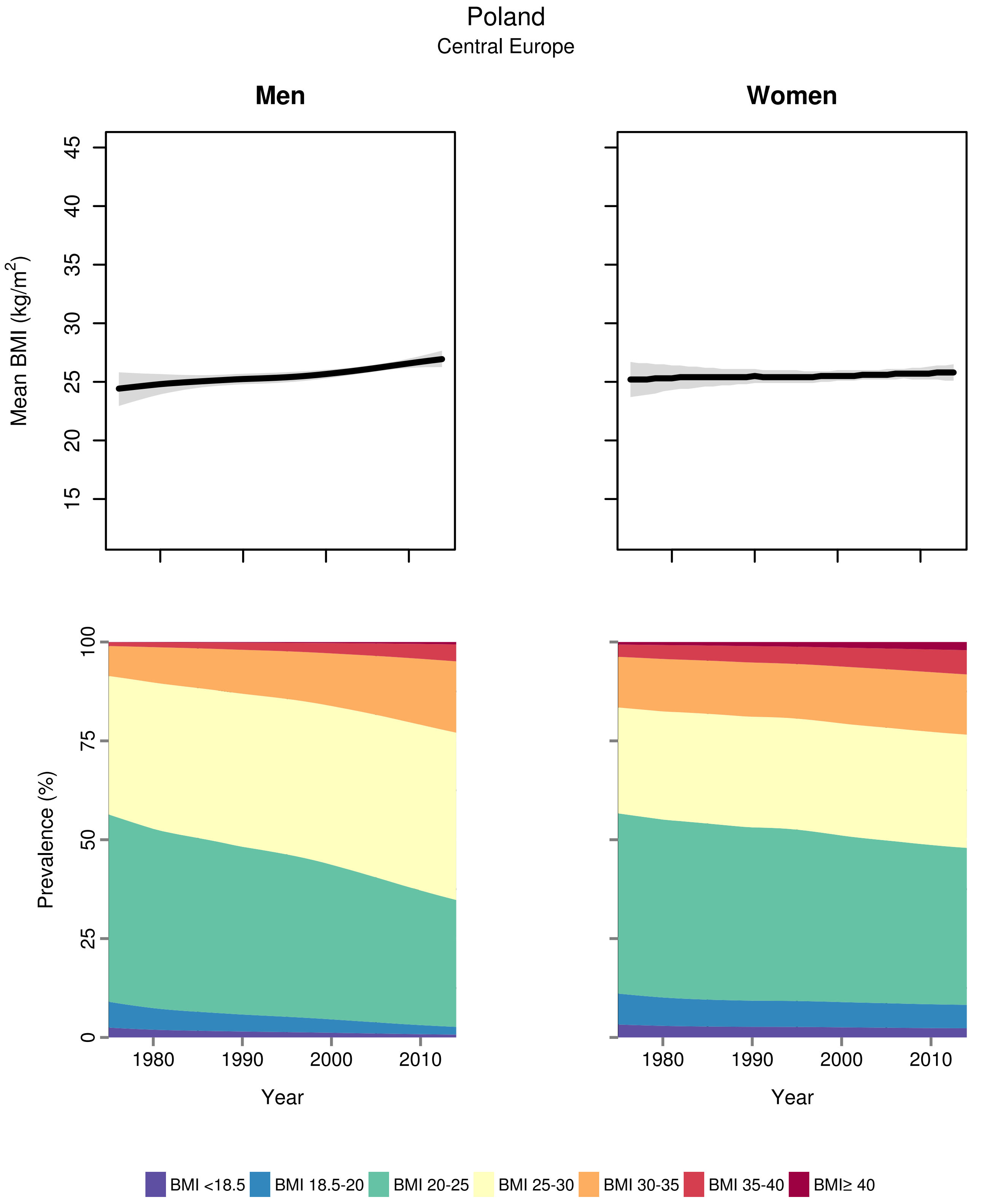

Poland
Central Europe
Men
Women
Mean BMI (kg/m2)
45
40
35
30
25
20
15
Prevalence (%)
100
75
50
25
0
1980
1990
2000
2010
Year
BMI <18.5
BMI 18.5-20
BMI 20-25
BMI 25-30
BMI 30-35
BMI 35-40
BMI≥ 40

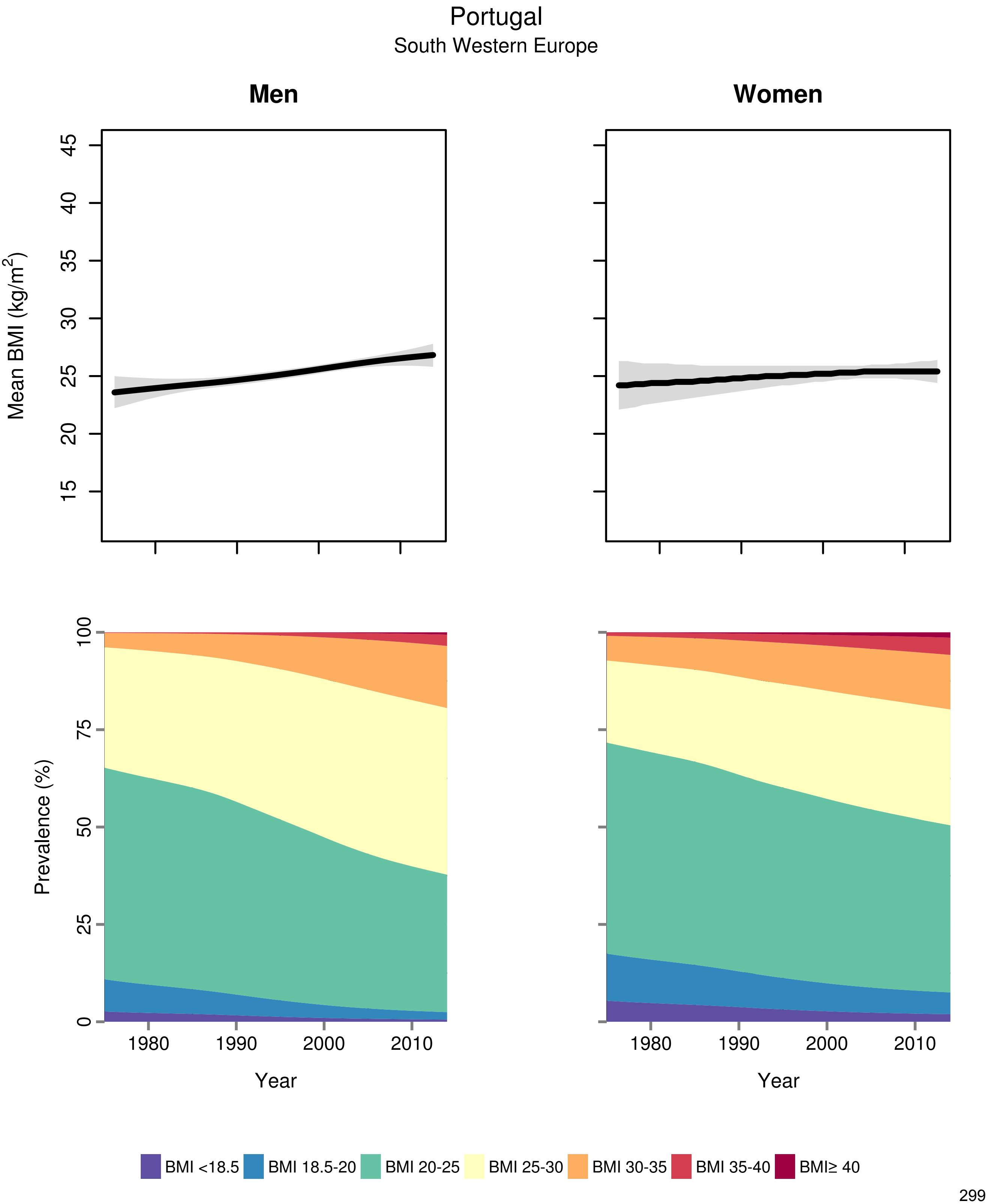
Portugal
South Western Europe
Men
Women
Mean BMI (kg/m²)
45
40
35
30
25
20
15
Prevalence (%)
100
75
50
25
0
1980
1990
2000
2010
Year
BMI <18.5
BMI 18.5-20
BMI 20-25
BMI 25-30
BMI 30-35
BMI 35-40
BMI≥ 40

Puerto Rico

Caribbean

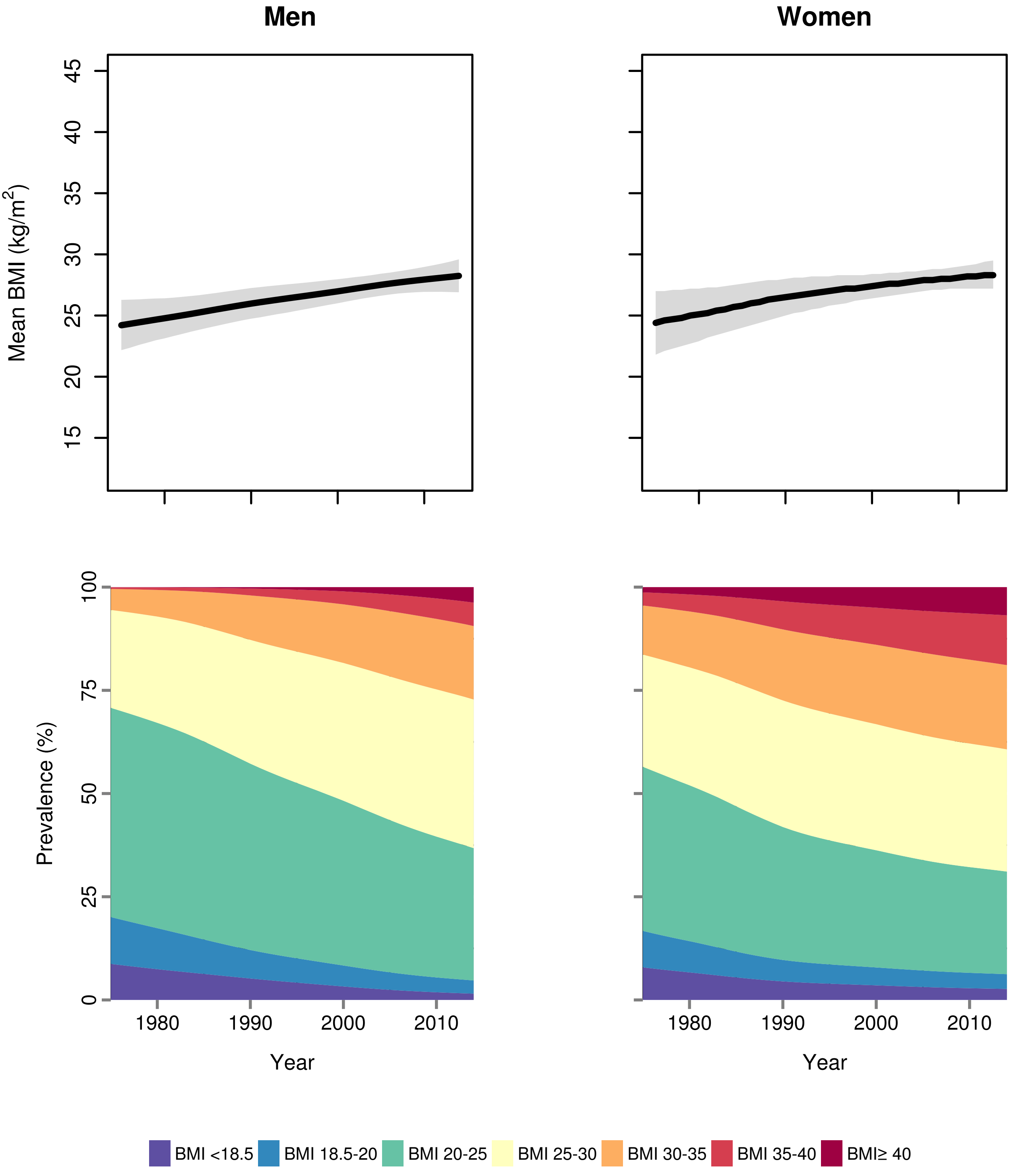

Qatar

Middle East and North Africa

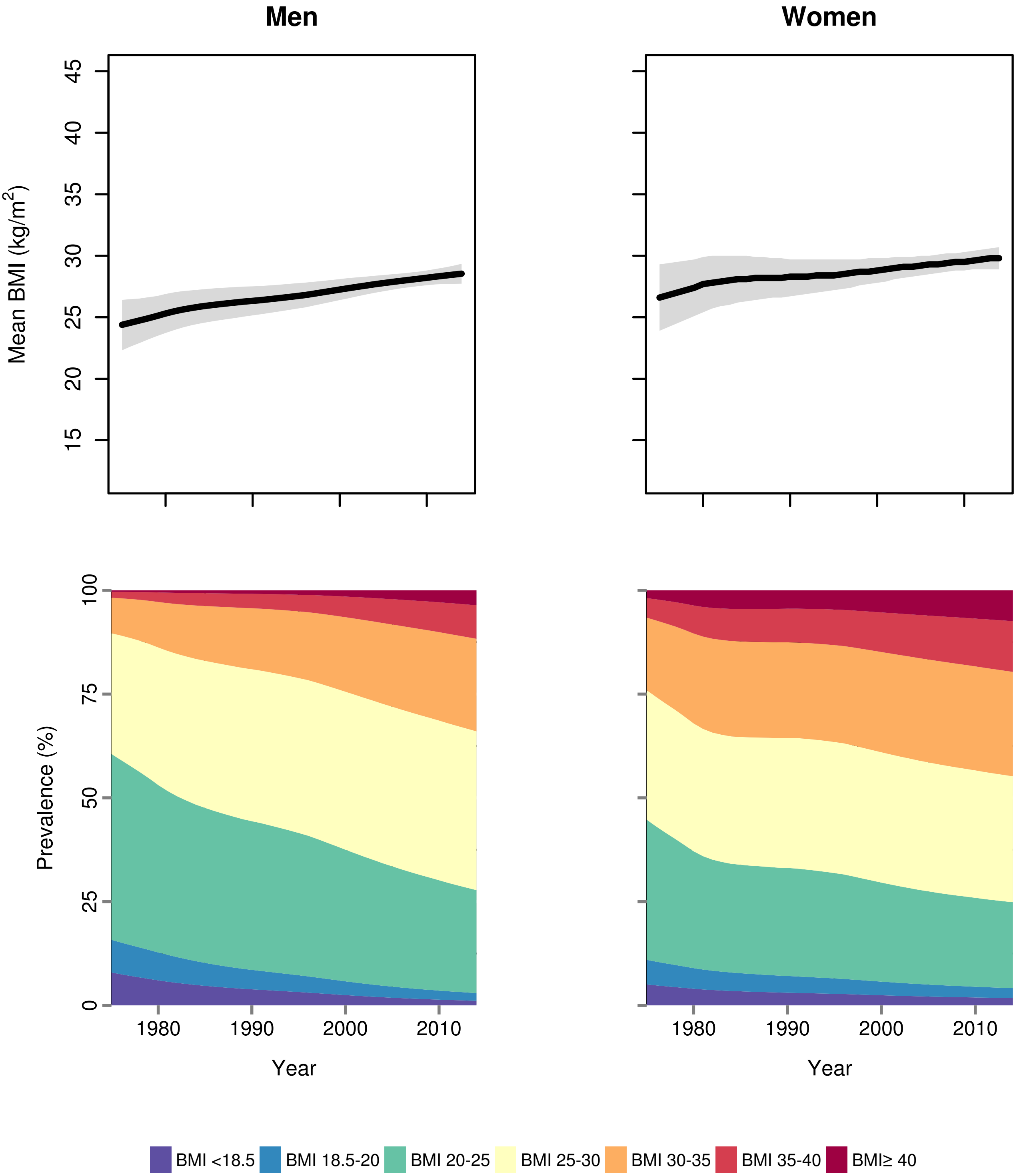

Romania

Central Europe

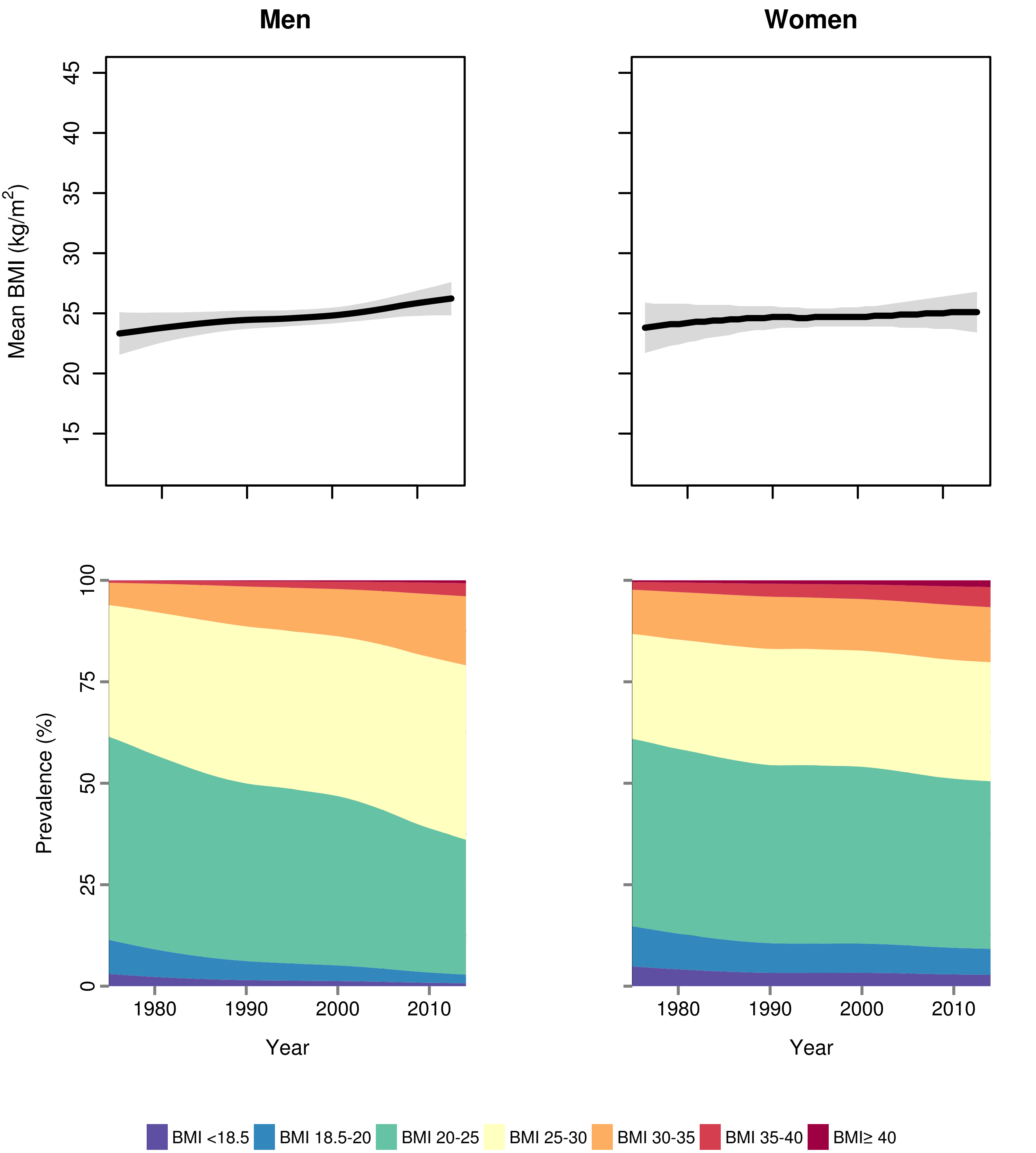

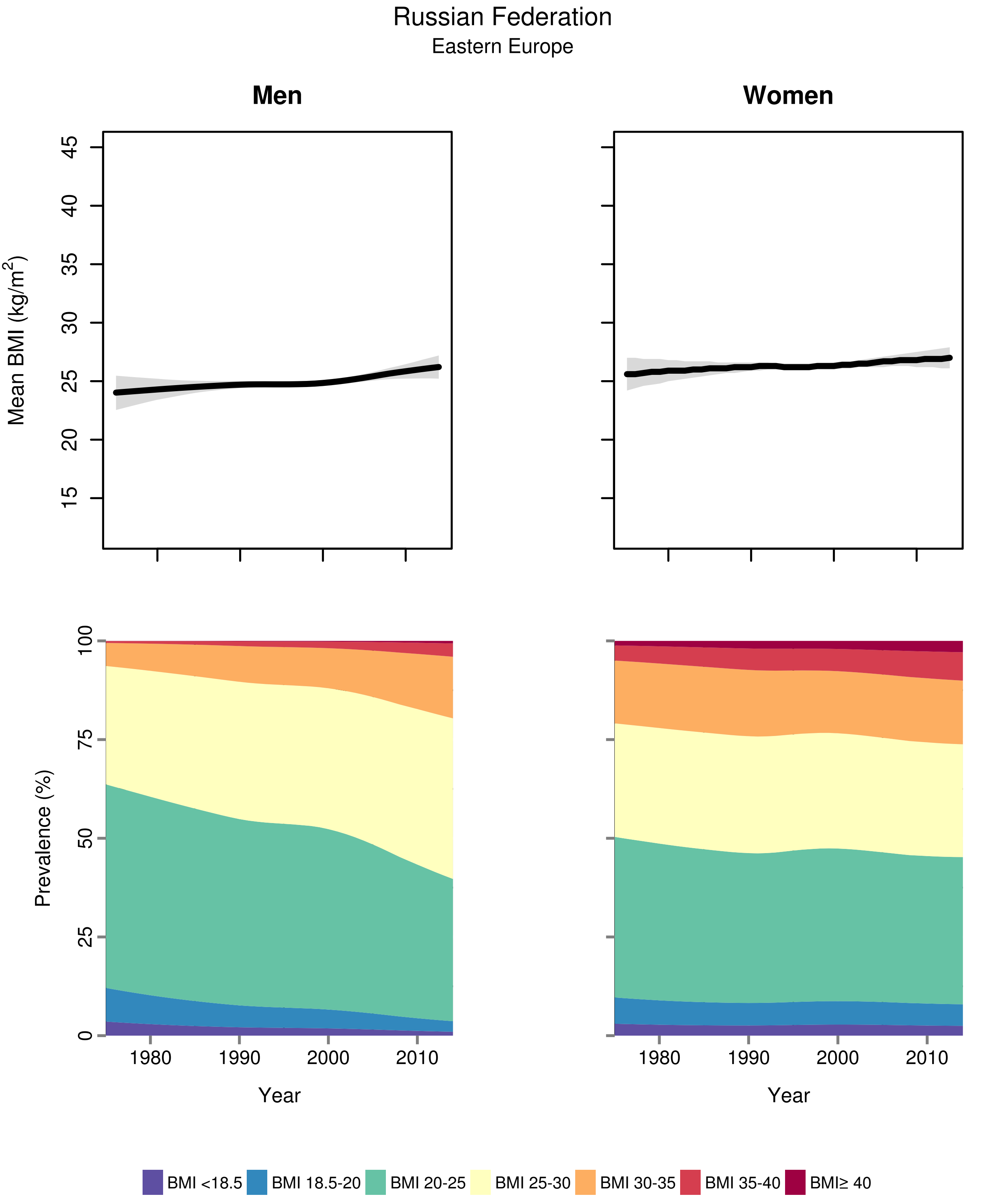

Russian Federation
Eastern Europe
Men
Women
Mean BMI (kg/m2)
15
20
25
30
35
40
45
Prevalence (%)
0
25
50
75
100
1980
1990
2000
2010
Year
BMI <18.5
BMI 18.5-20
BMI 20-25
BMI 25-30
BMI 30-35
BMI 35-40
BMI≥ 40

Rwanda

East Africa

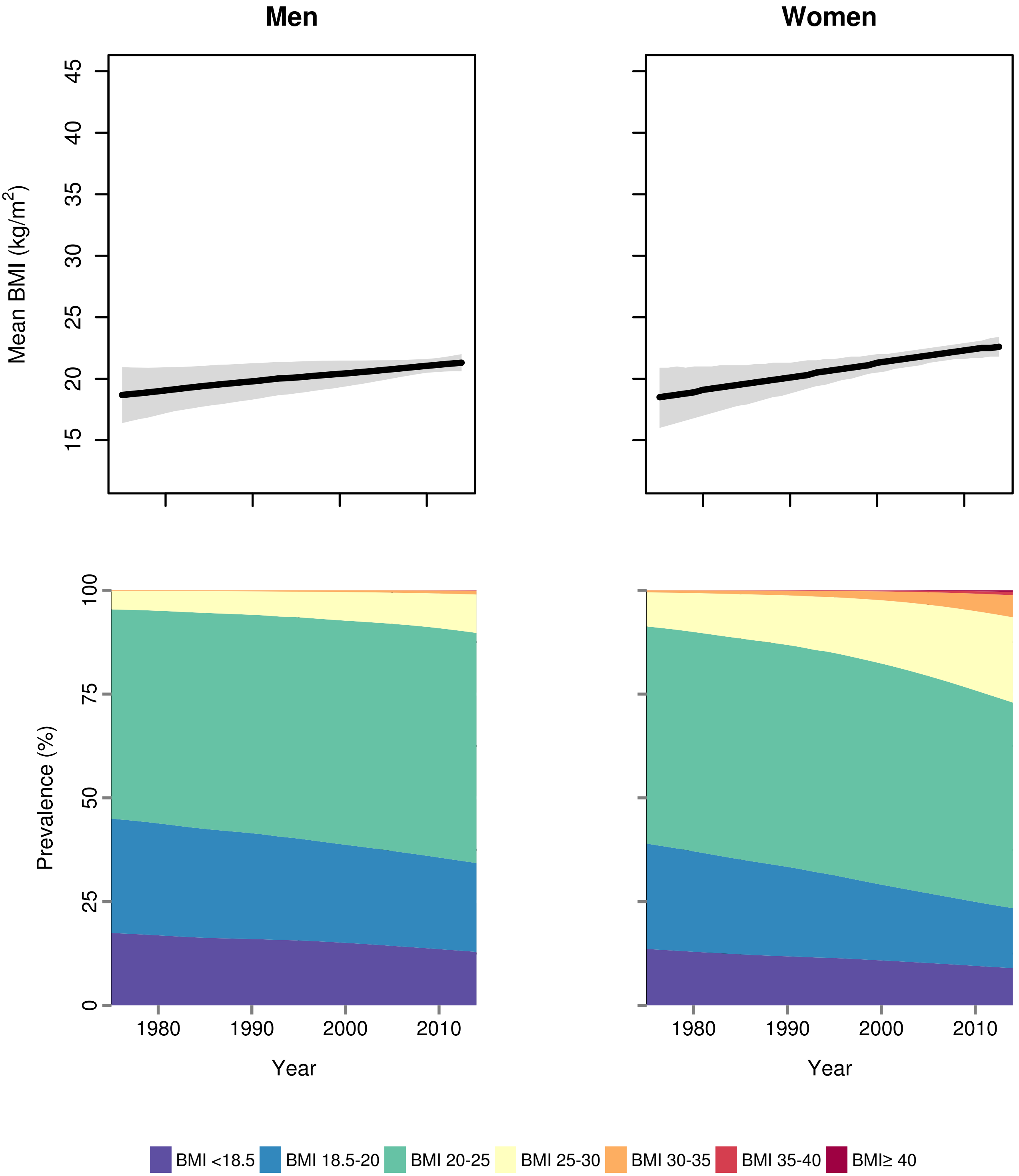

Saint Kitts and Nevis

Caribbean

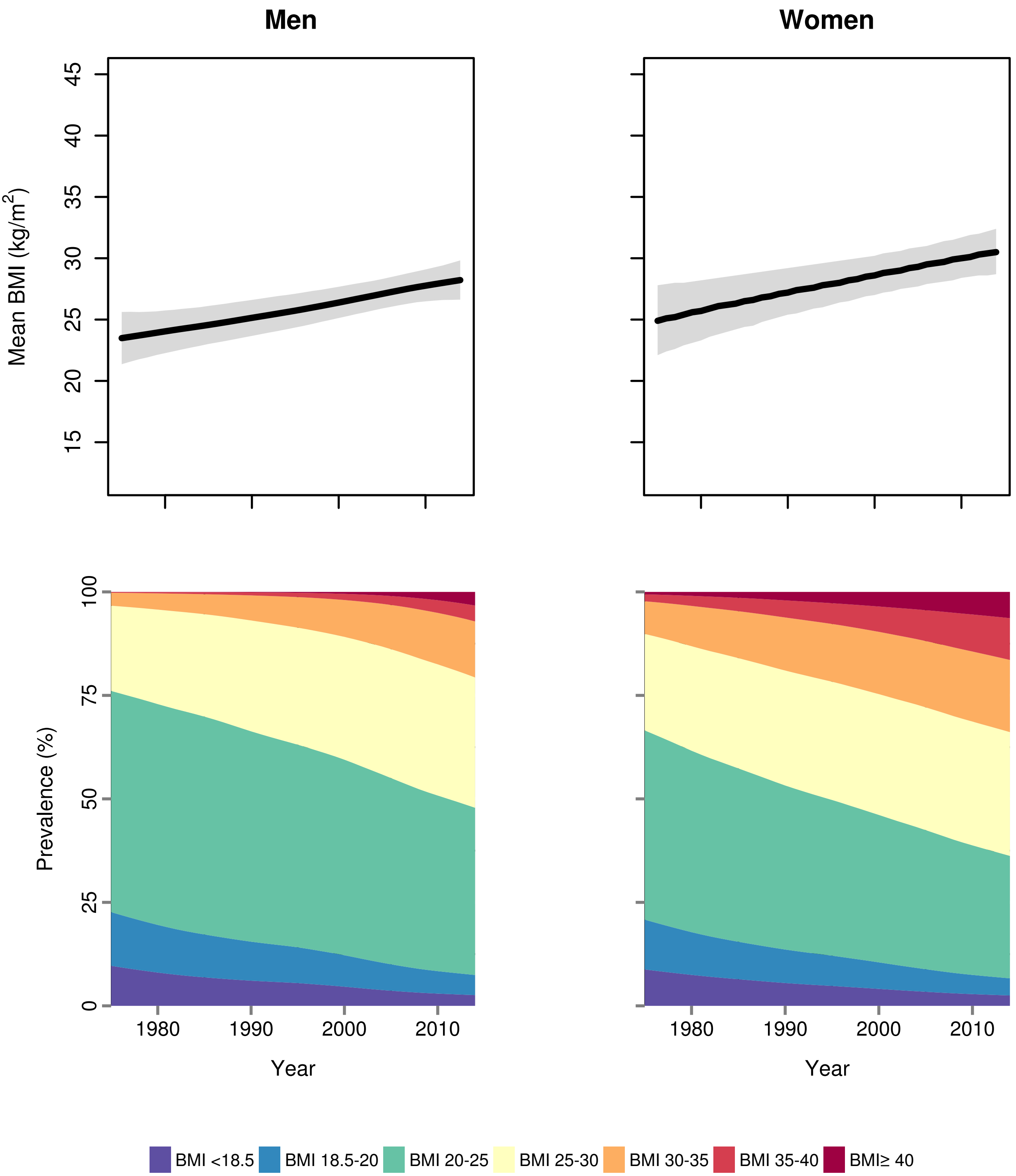

Saint Lucia
Caribbean

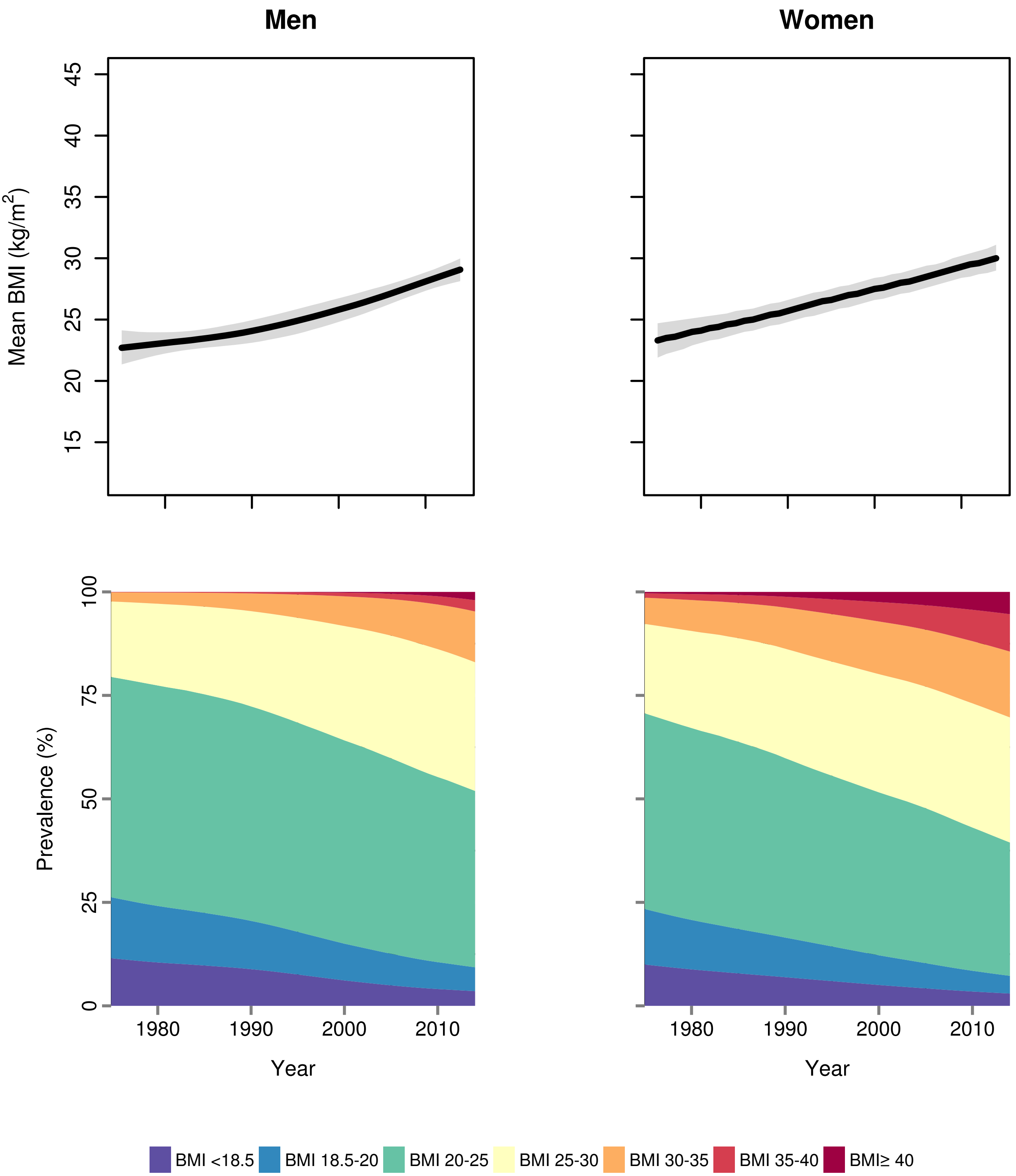

Saint Vincent and the Grenadines

Caribbean

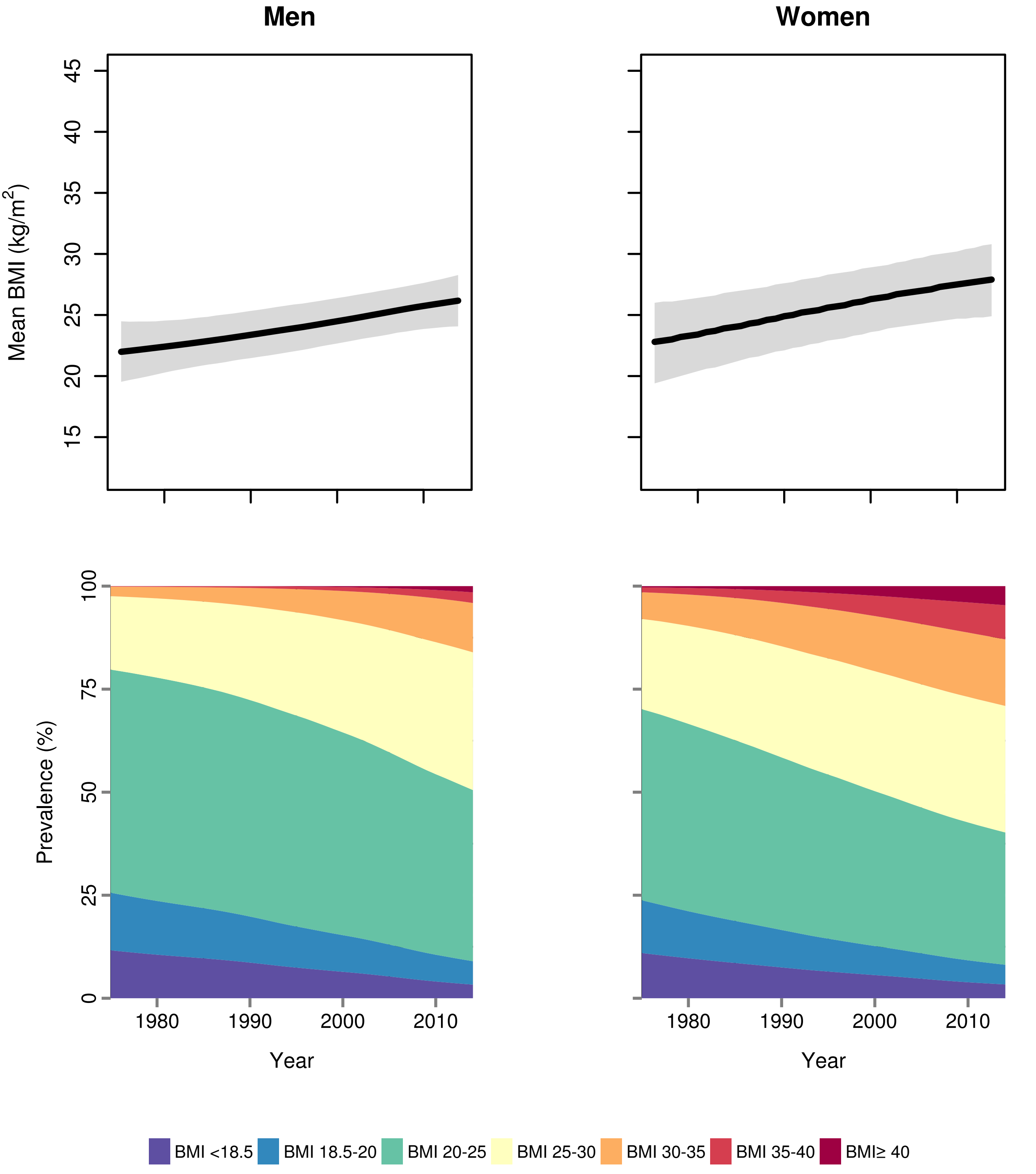

Samoa

Polynesia and Micronesia

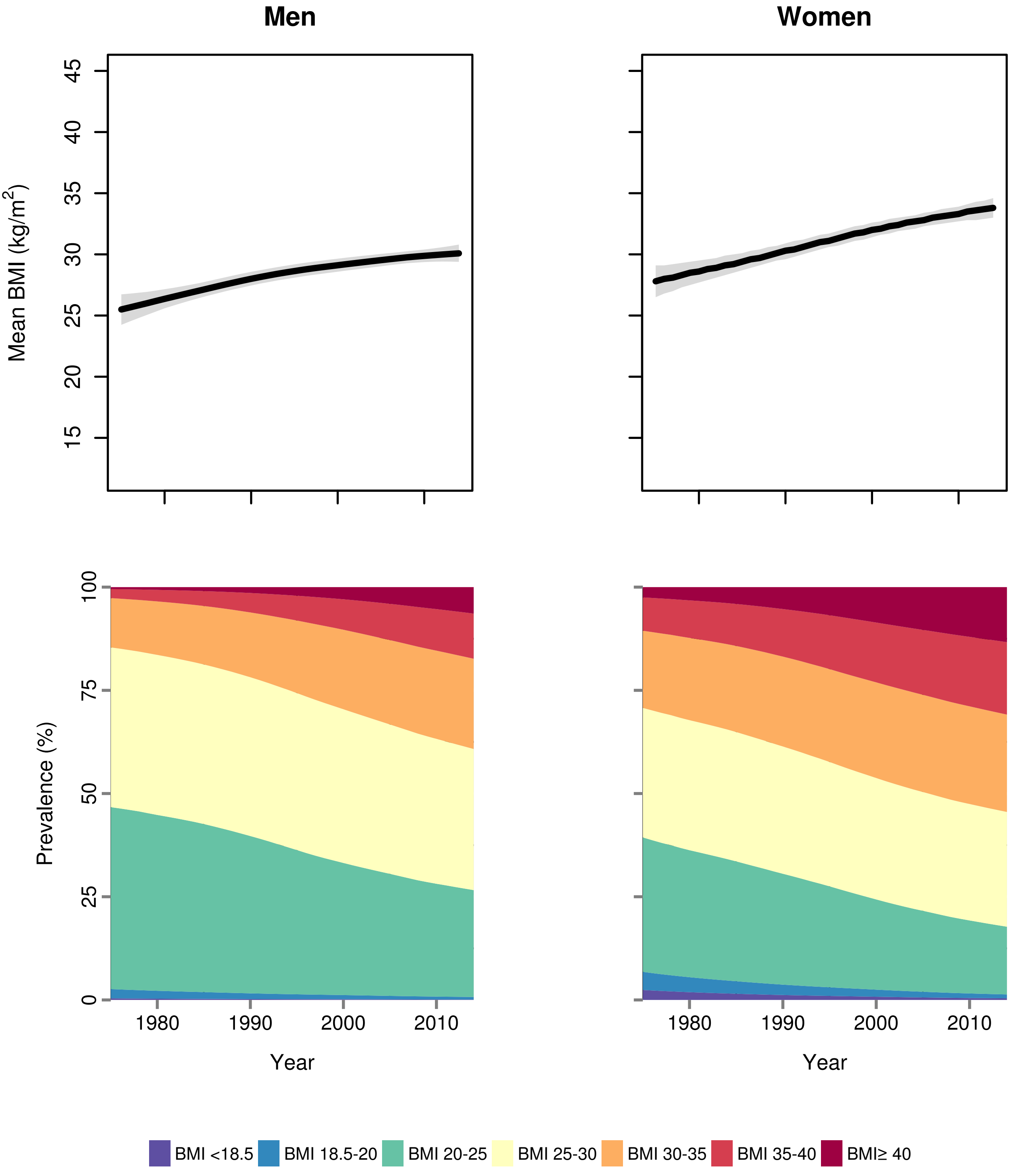

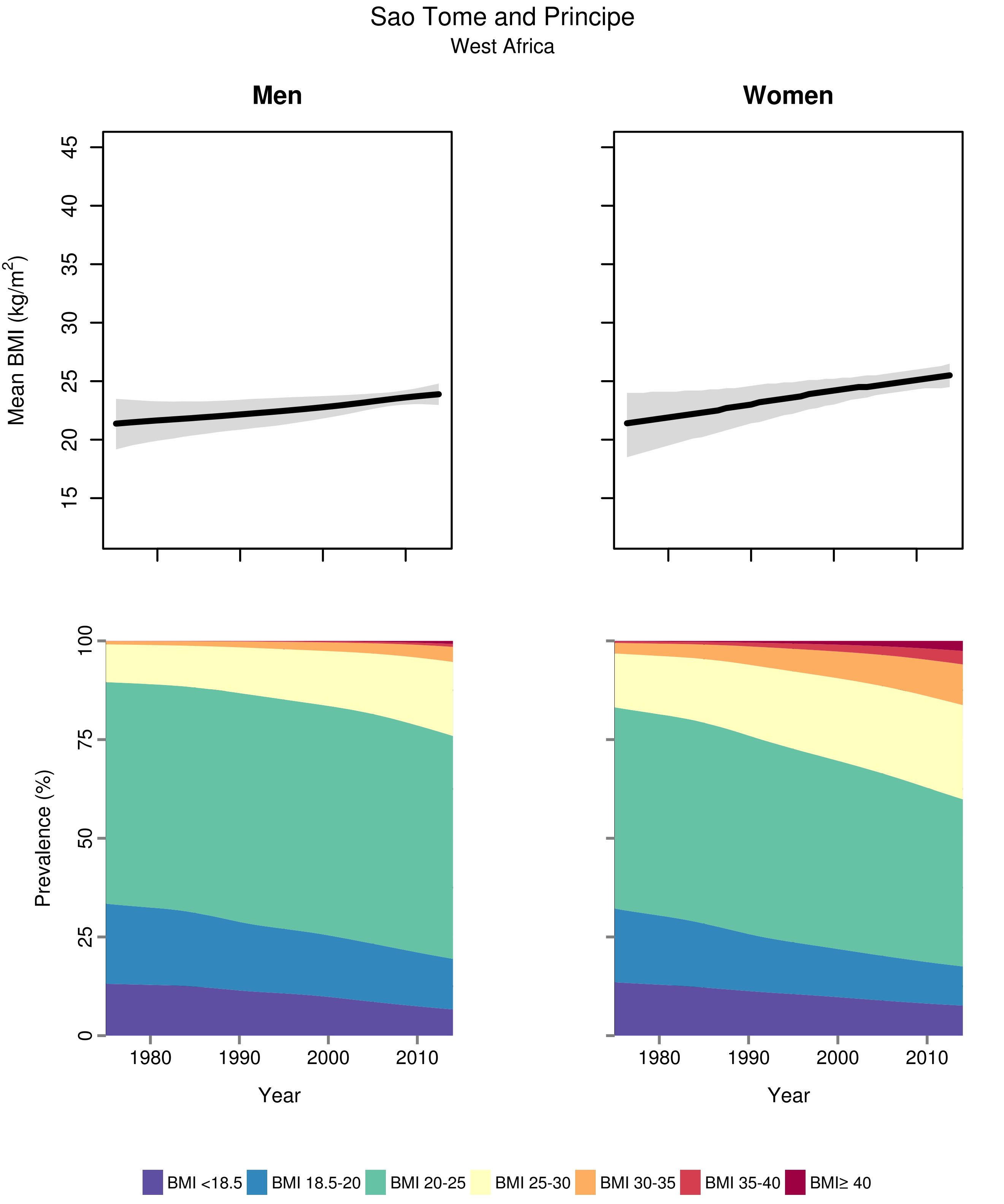

Sao Tome and Principe
West Africa
Men
Women
Mean BMI (kg/m2)
45
40
35
30
25
20
15
Prevalence (%)
100
75
50
25
0
1980
1990
2000
2010
Year
BMI <18.5
BMI 18.5-20
BMI 20-25
BMI 25-30
BMI 30-35
BMI 35-40
BMI≥ 40

Saudi Arabia

Middle East and North Africa

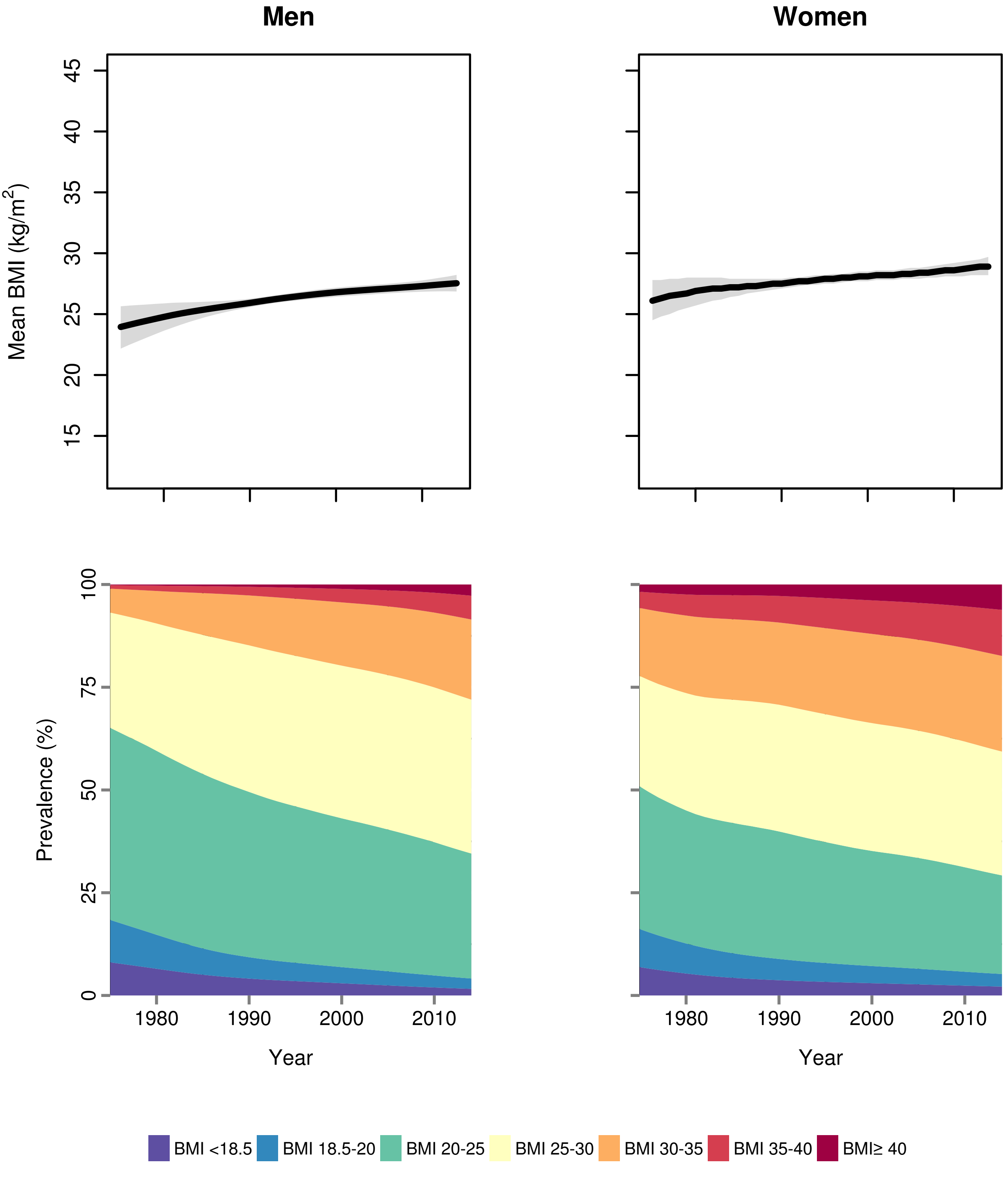

Senegal
West Africa

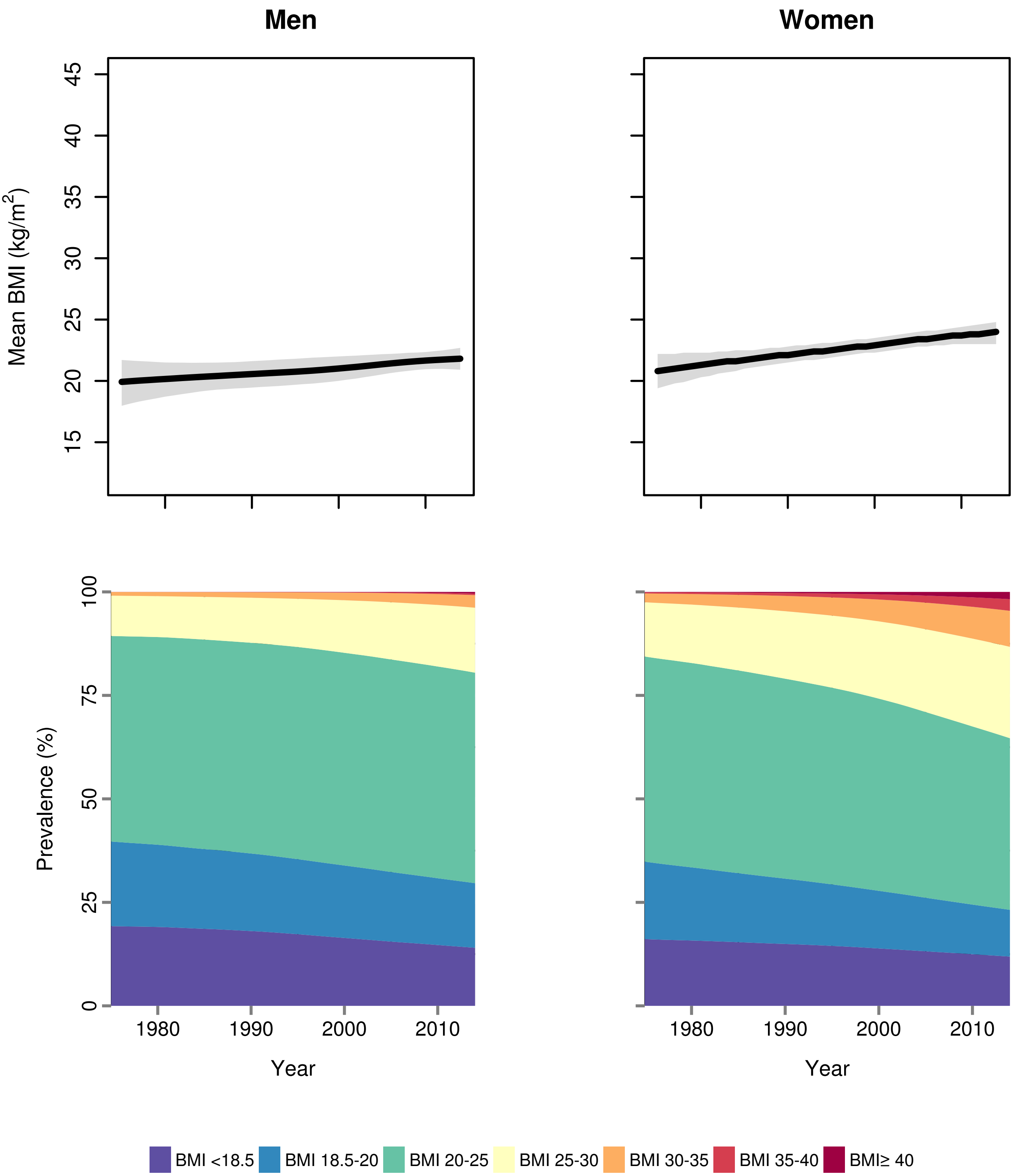

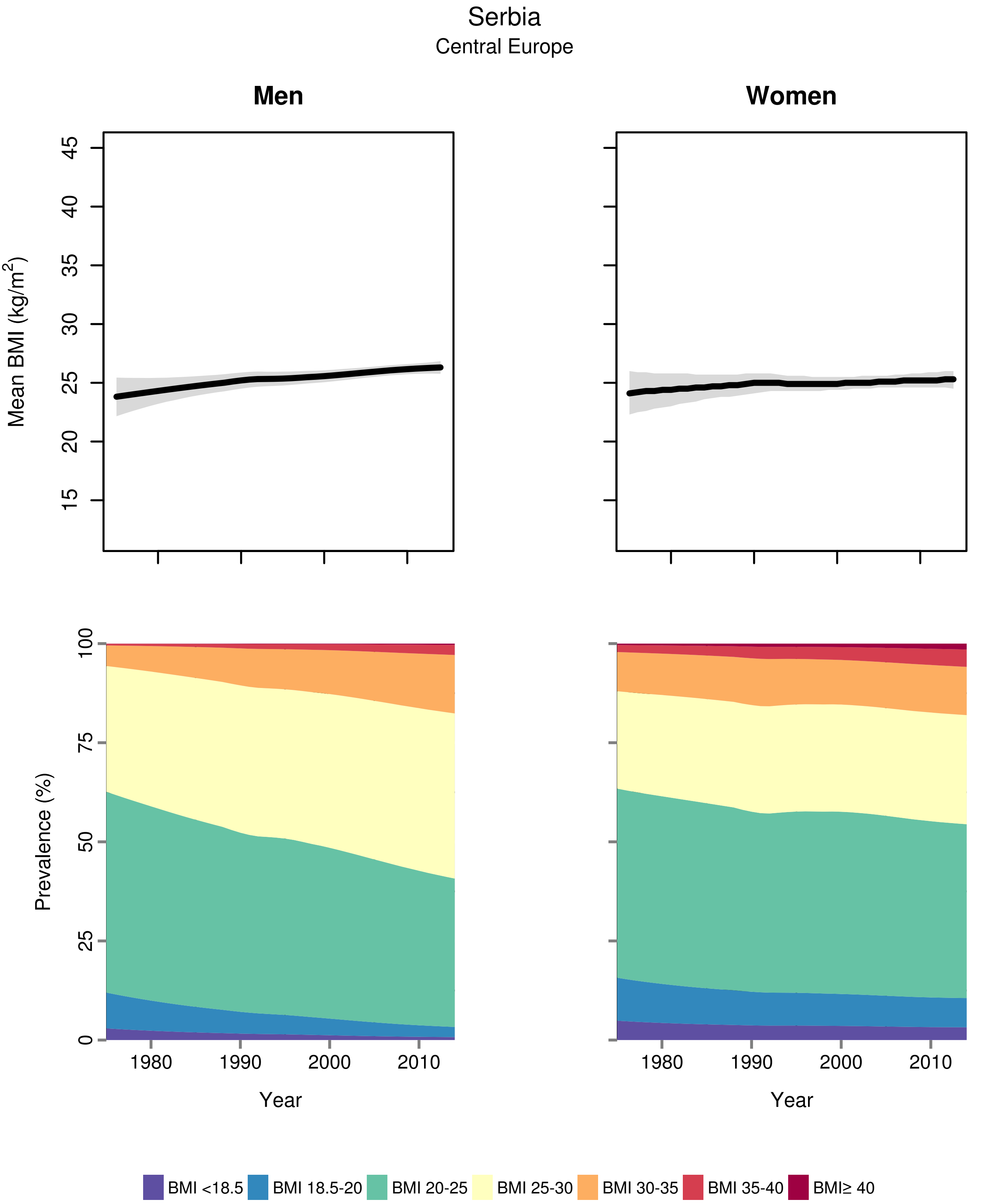
Serbia
Central Europe
Men
Women
Mean BMI (kg/m2)
45
40
35
30
25
20
15
Prevalence (%)
100
75
50
25
0
1980
1990
2000
2010
Year
BMI <18.5
BMI 18.5-20
BMI 20-25
BMI 25-30
BMI 30-35
BMI 35-40
BMI≥ 40

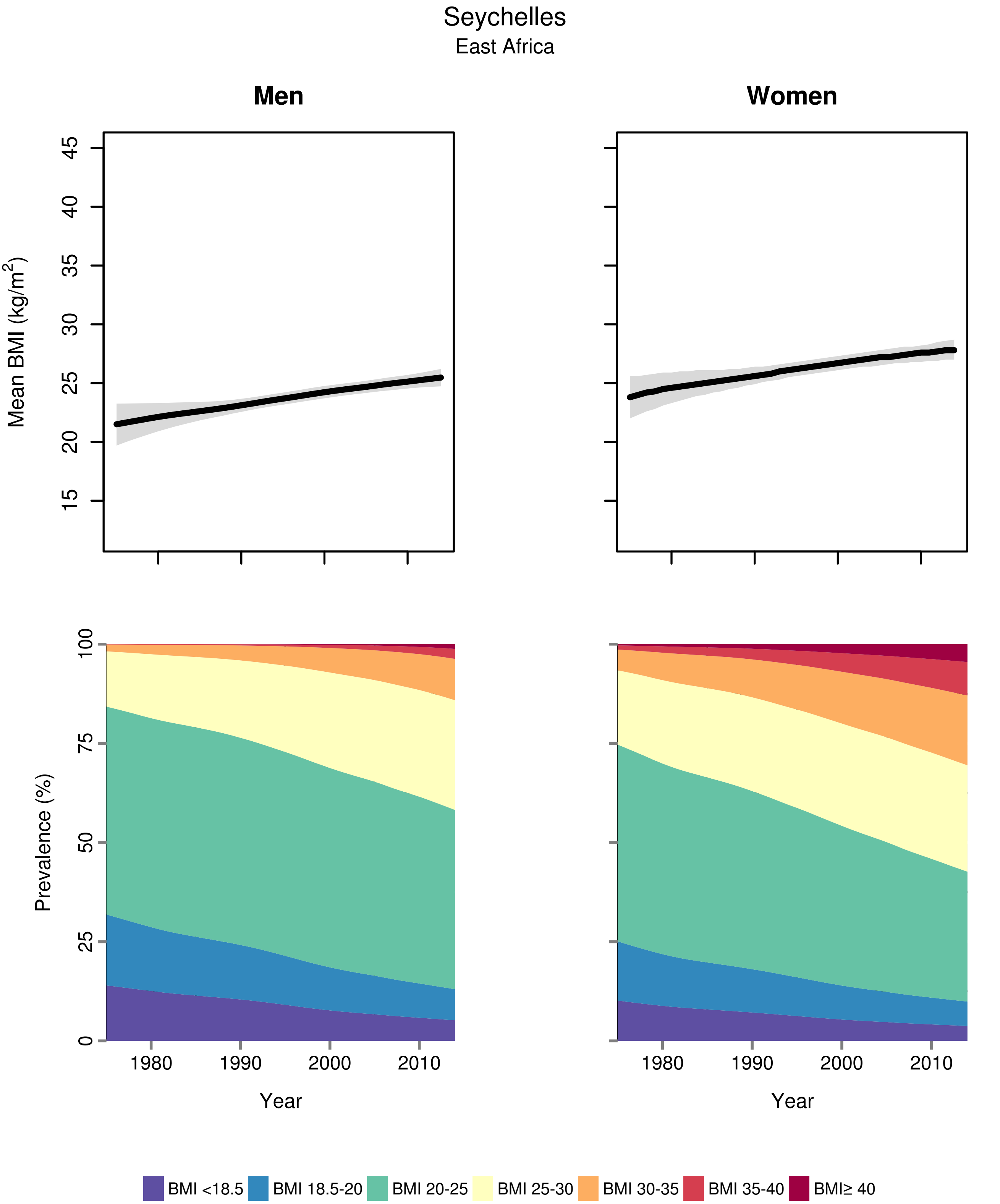
Seychelles
East Africa
Men
Women
Mean BMI (kg/m²)
45
40
35
30
25
20
15
Prevalence (%)
100
75
50
25
0
1980
1990
2000
2010
Year
BMI <18.5
BMI 18.5-20
BMI 20-25
BMI 25-30
BMI 30-35
BMI 35-40
BMI≥ 40

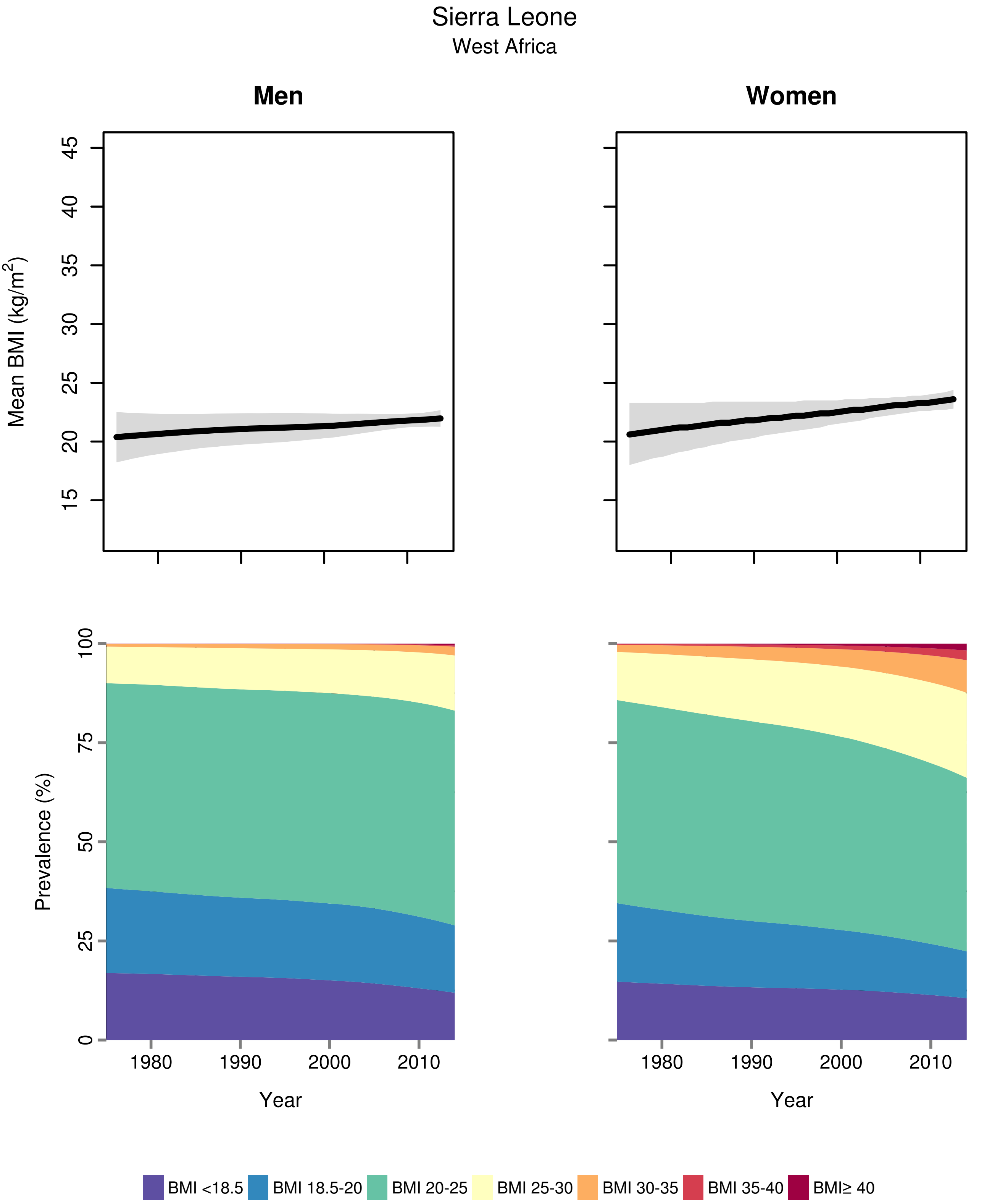
Sierra Leone
West Africa
Men
Women
Mean BMI (kg/m2)
45
40
35
30
25
20
15
Prevalence (%)
100
75
50
25
0
1980
1990
2000
2010
Year
BMI <18.5
BMI 18.5-20
BMI 20-25
BMI 25-30
BMI 30-35
BMI 35-40
BMI≥ 40

Singapore

High-income Asia Pacific

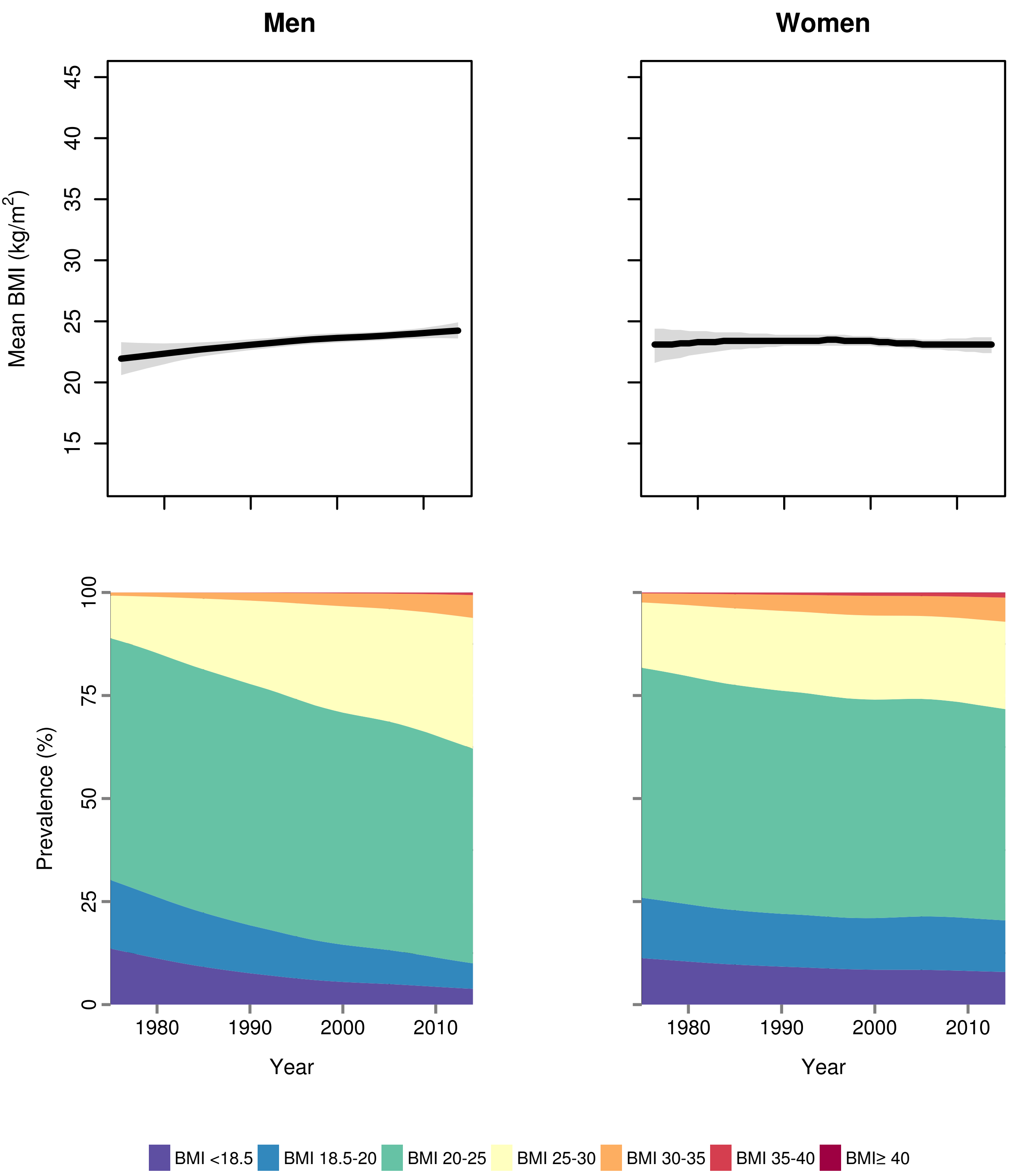

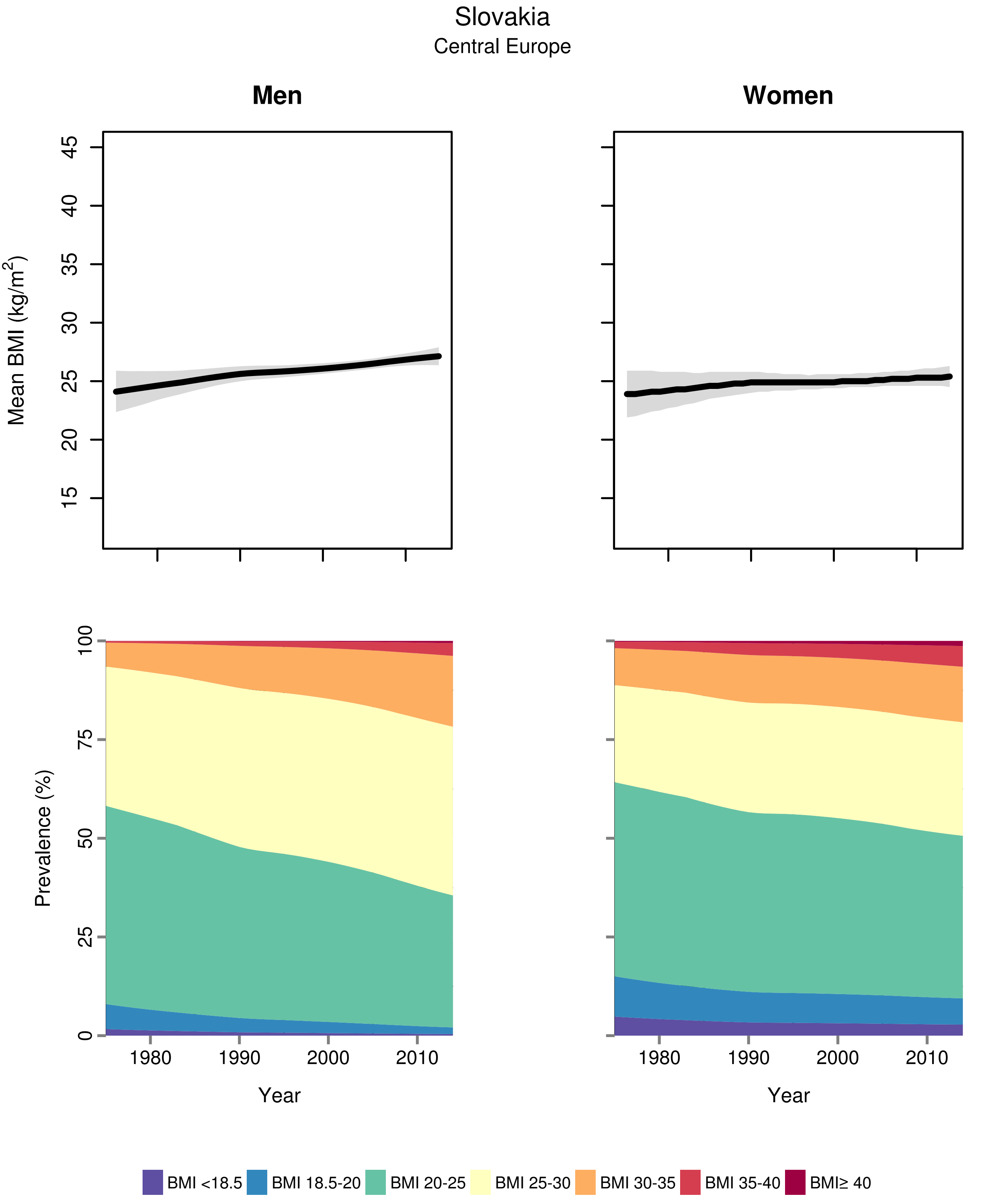

Slovakia
Central Europe
Men
Women
Mean BMI (kg/m2)
45
40
35
30
25
20
15
Prevalence (%)
100
75
50
25
0
1980
1990
2000
2010
Year
BMI <18.5
BMI 18.5-20
BMI 20-25
BMI 25-30
BMI 30-35
BMI 35-40
BMI≥ 40

Slovenia

Central Europe

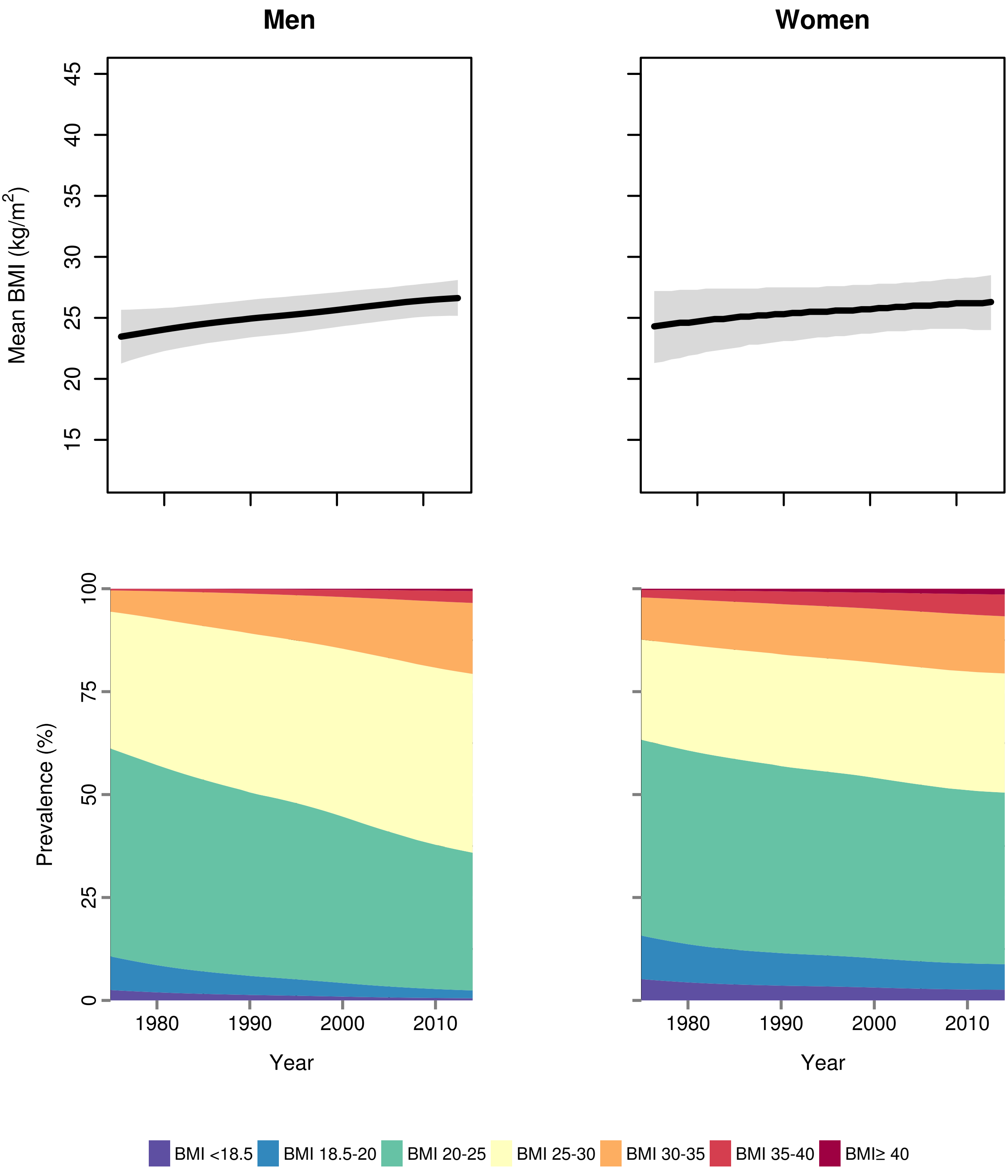

Solomon Islands
Melanesia

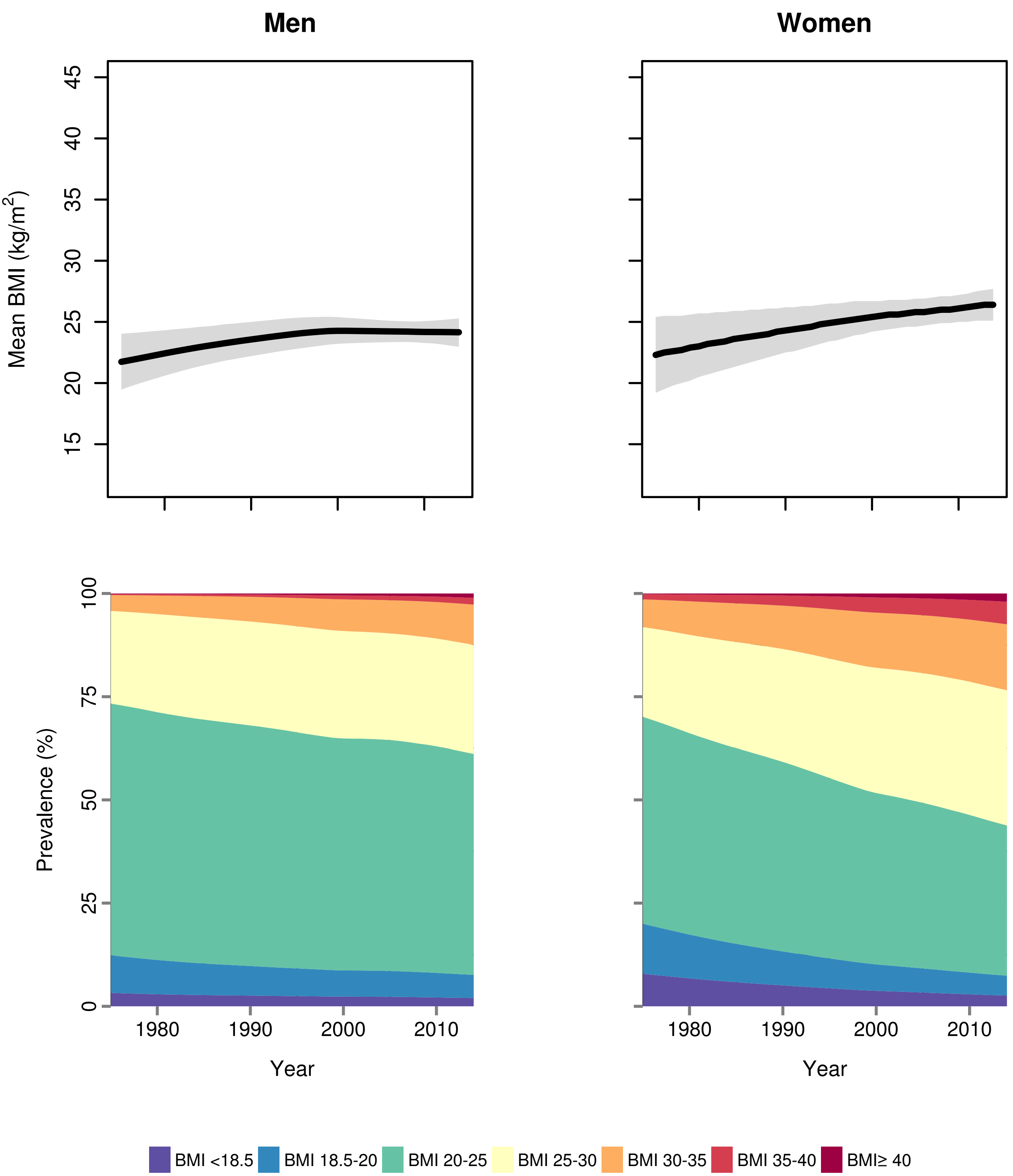

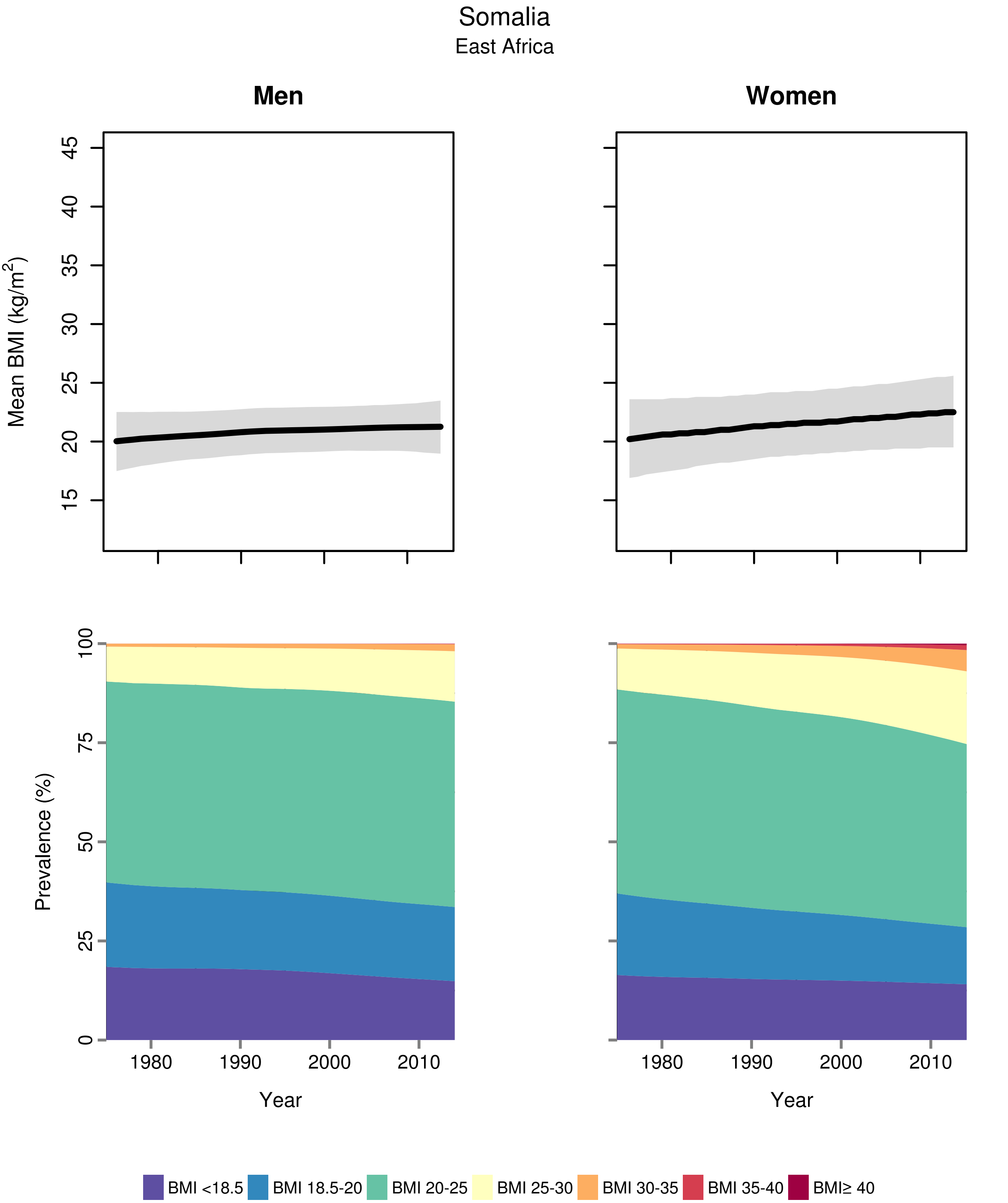

Somalia
East Africa
Men
Women
Mean BMI (kg/m²)
15
20
25
30
35
40
45
Prevalence (%)
0
25
50
75
100
1980
1990
2000
2010
Year
BMI <18.5
BMI 18.5-20
BMI 20-25
BMI 25-30
BMI 30-35
BMI 35-40
BMI≥ 40

South Africa

Southern Africa

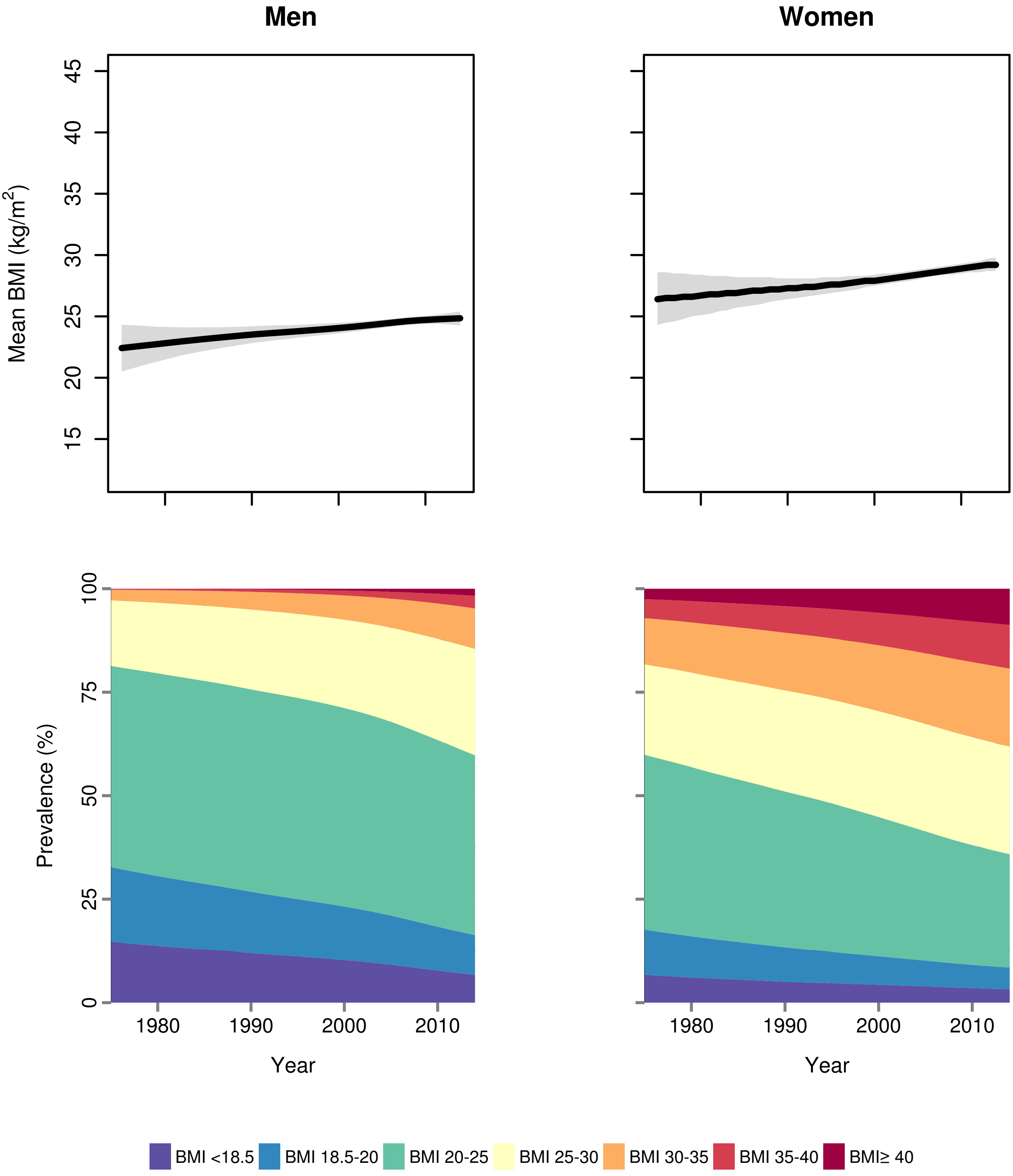

South Korea

High-income Asia Pacific

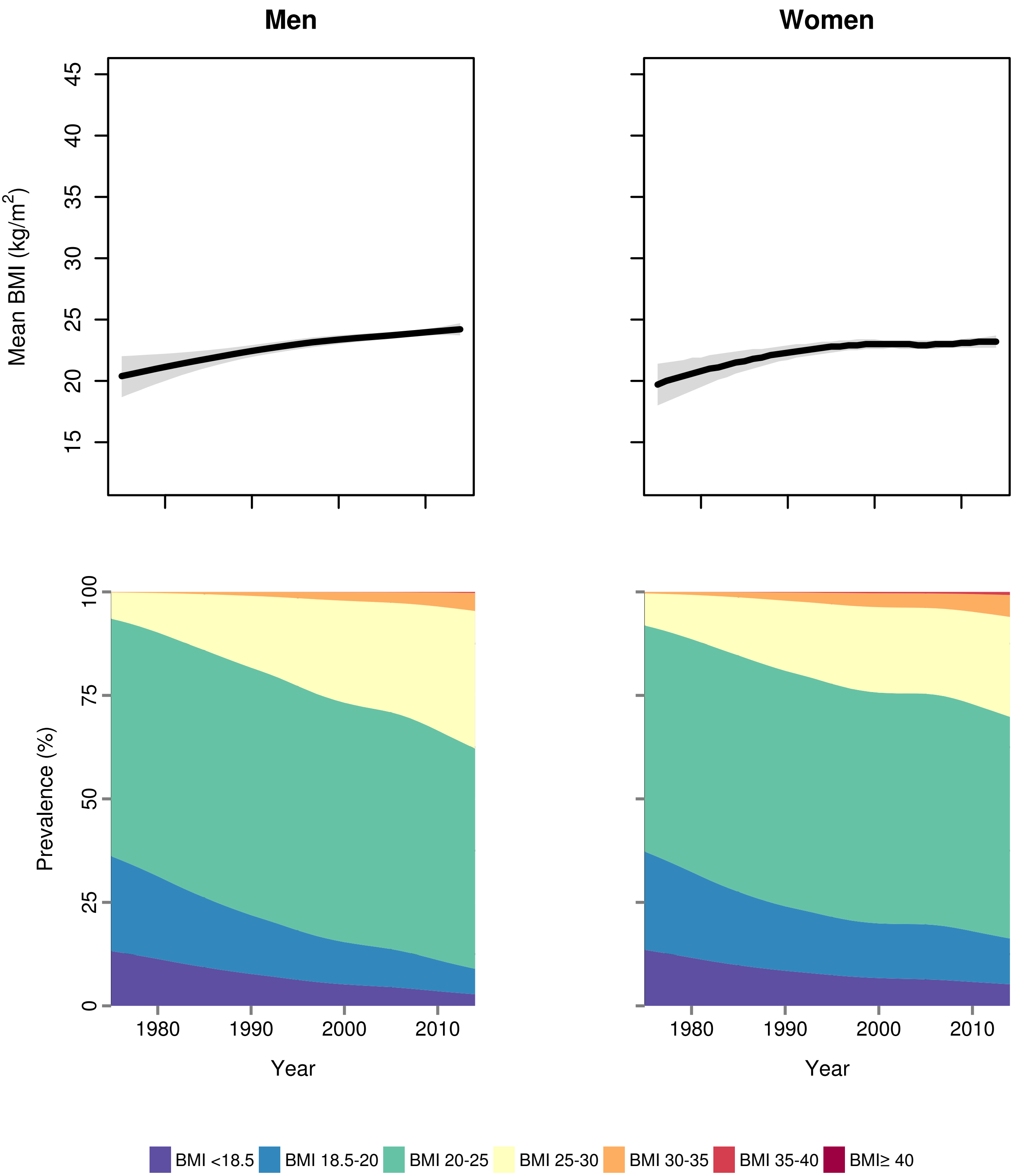

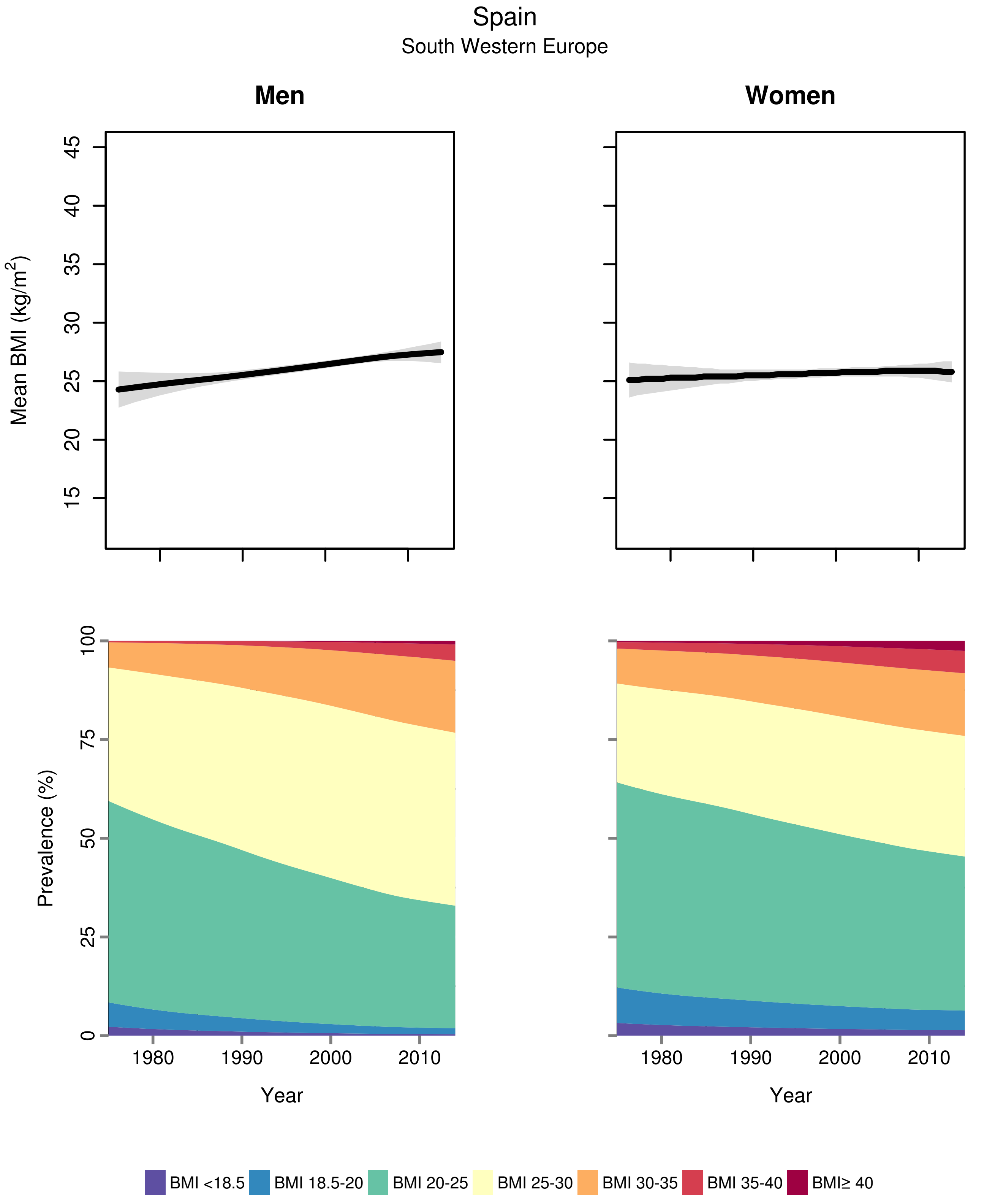

Spain
South Western Europe
Men
Women
Mean BMI (kg/m2)
45
40
35
30
25
20
15
Prevalence (%)
100
75
50
25
0
1980
1990
2000
2010
Year
BMI <18.5
BMI 18.5-20
BMI 20-25
BMI 25-30
BMI 30-35
BMI 35-40
BMI≥ 40

Sri Lanka

South East Asia

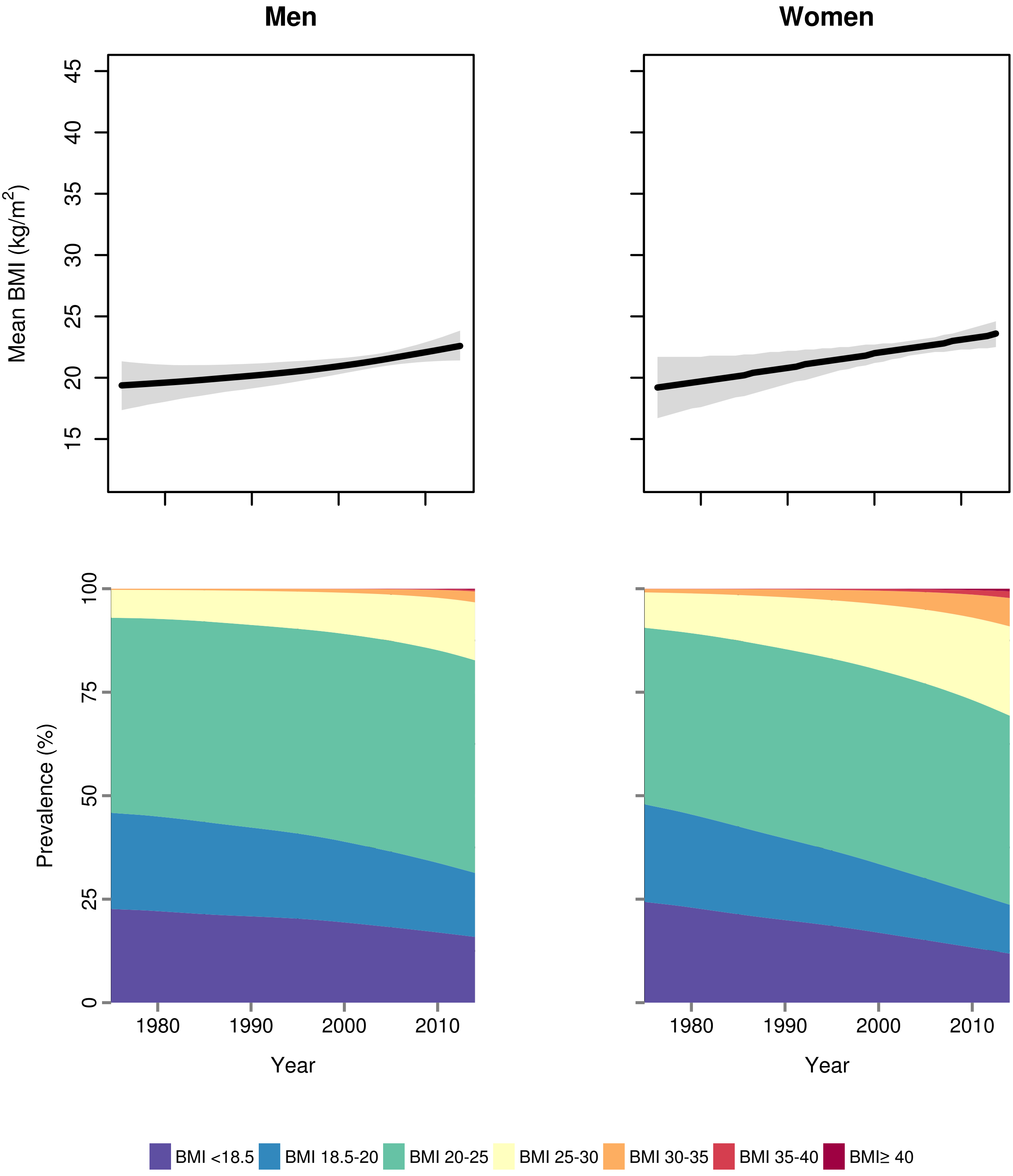

Sudan

East Africa

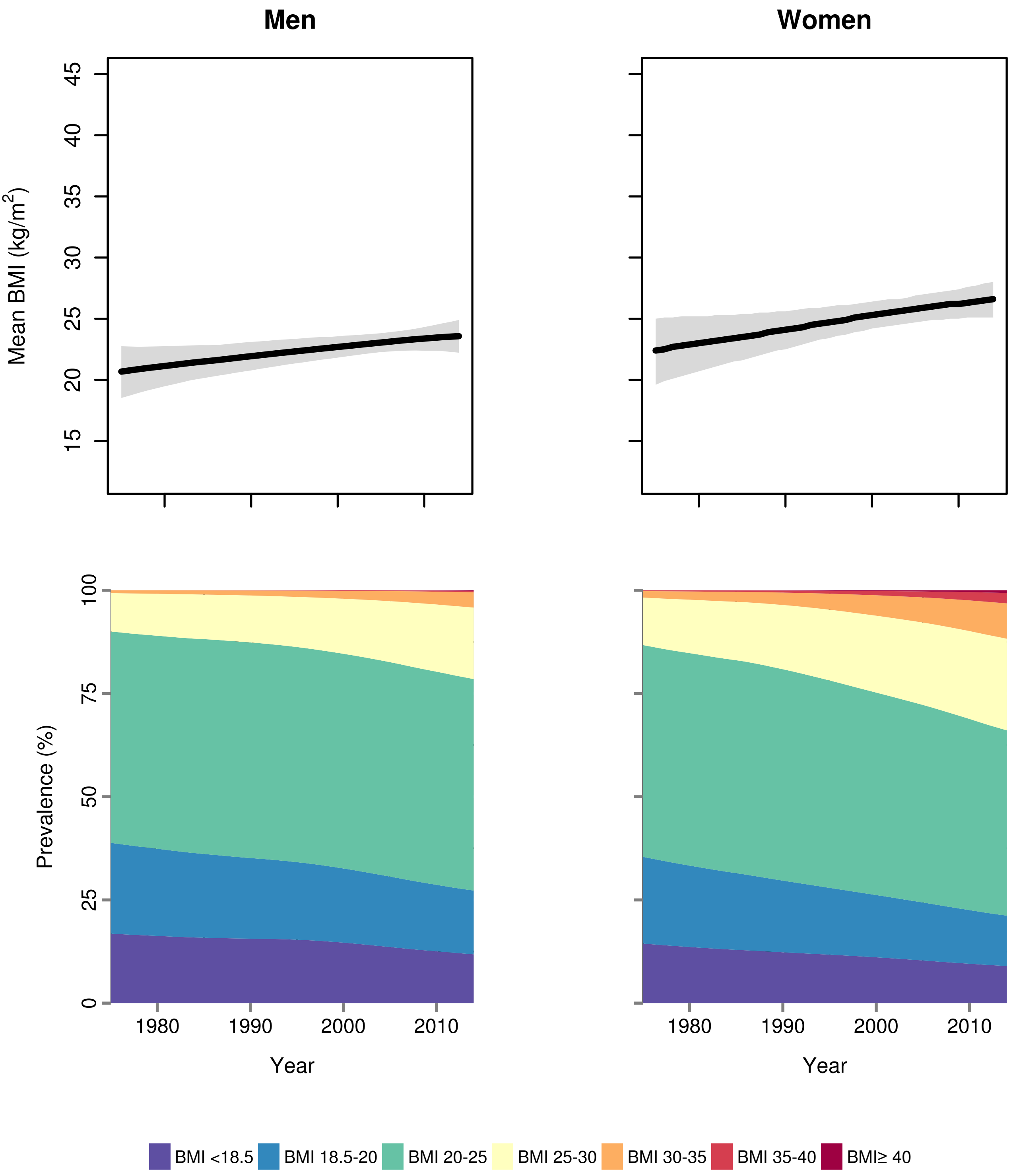

Suriname
Caribbean

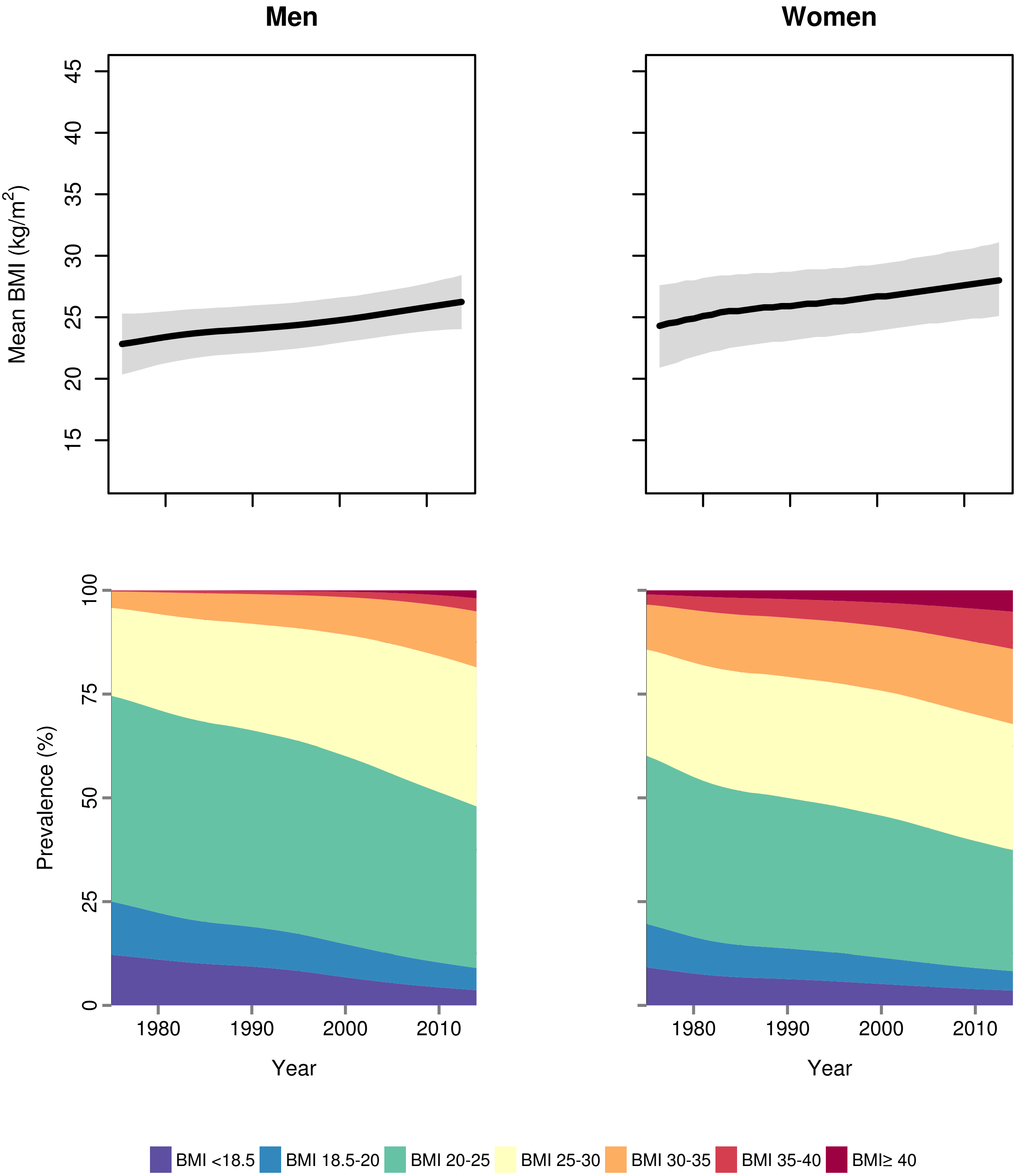

Swaziland

Southern Africa

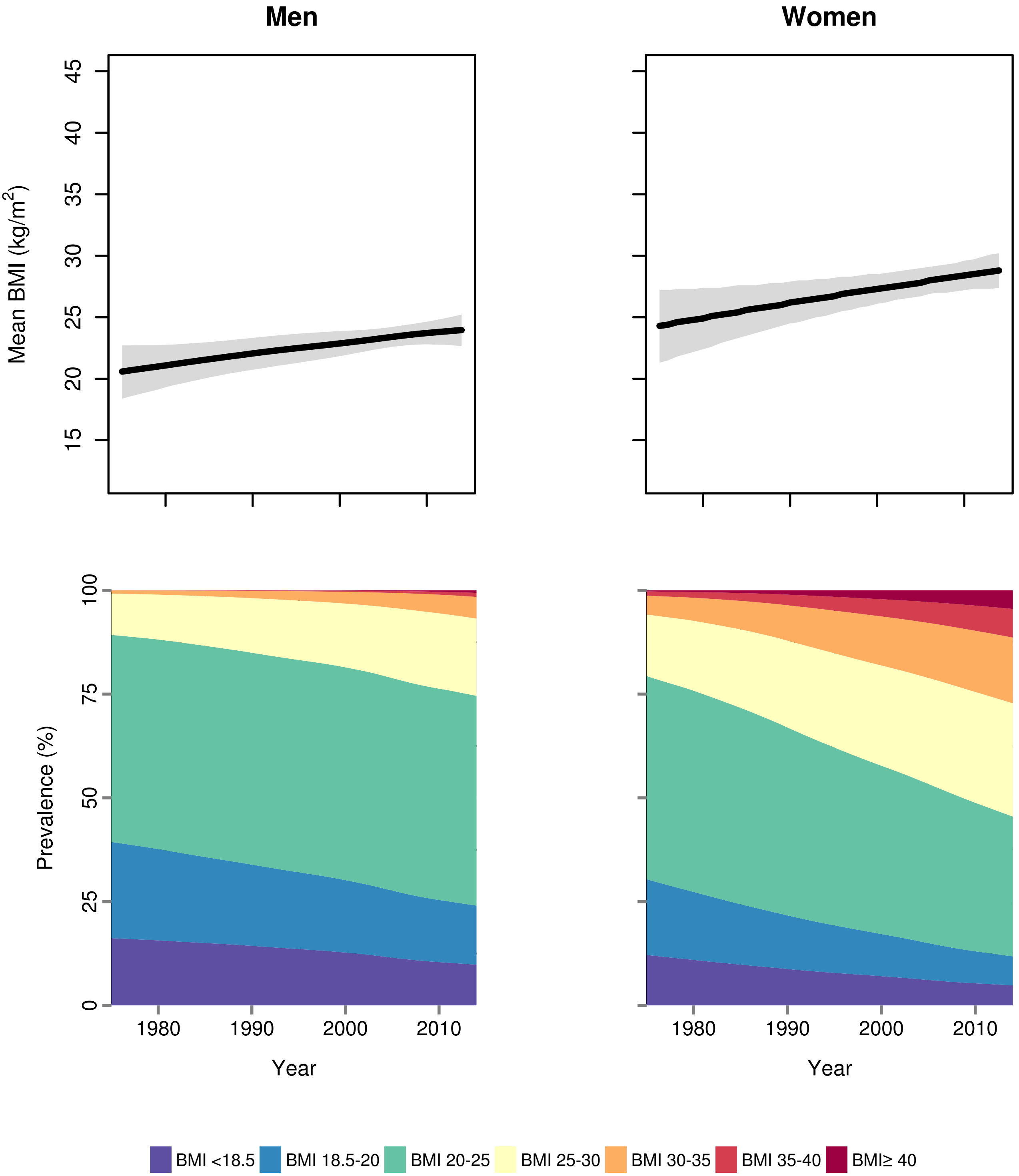

Sweden

North Western Europe

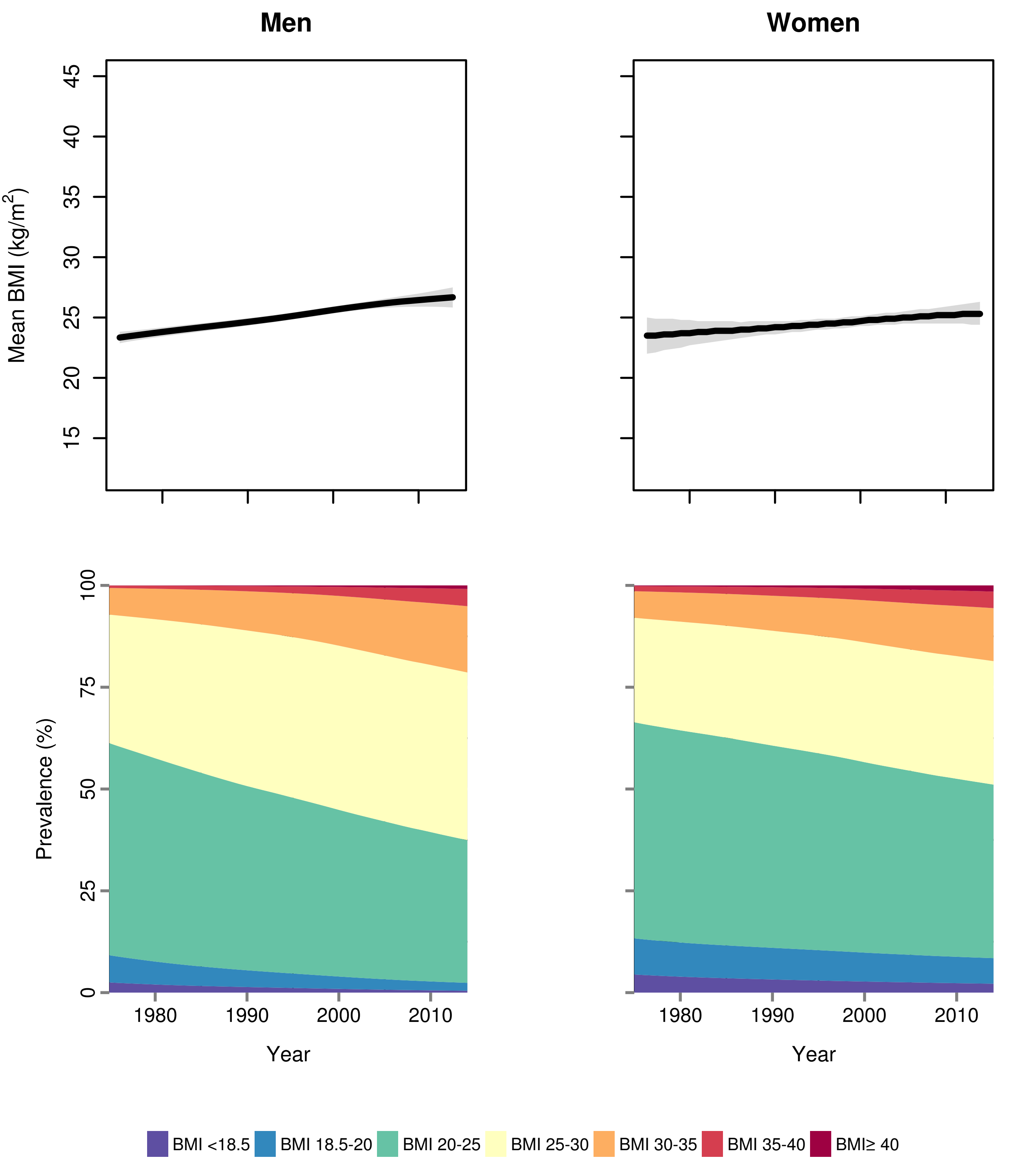

Switzerland

North Western Europe

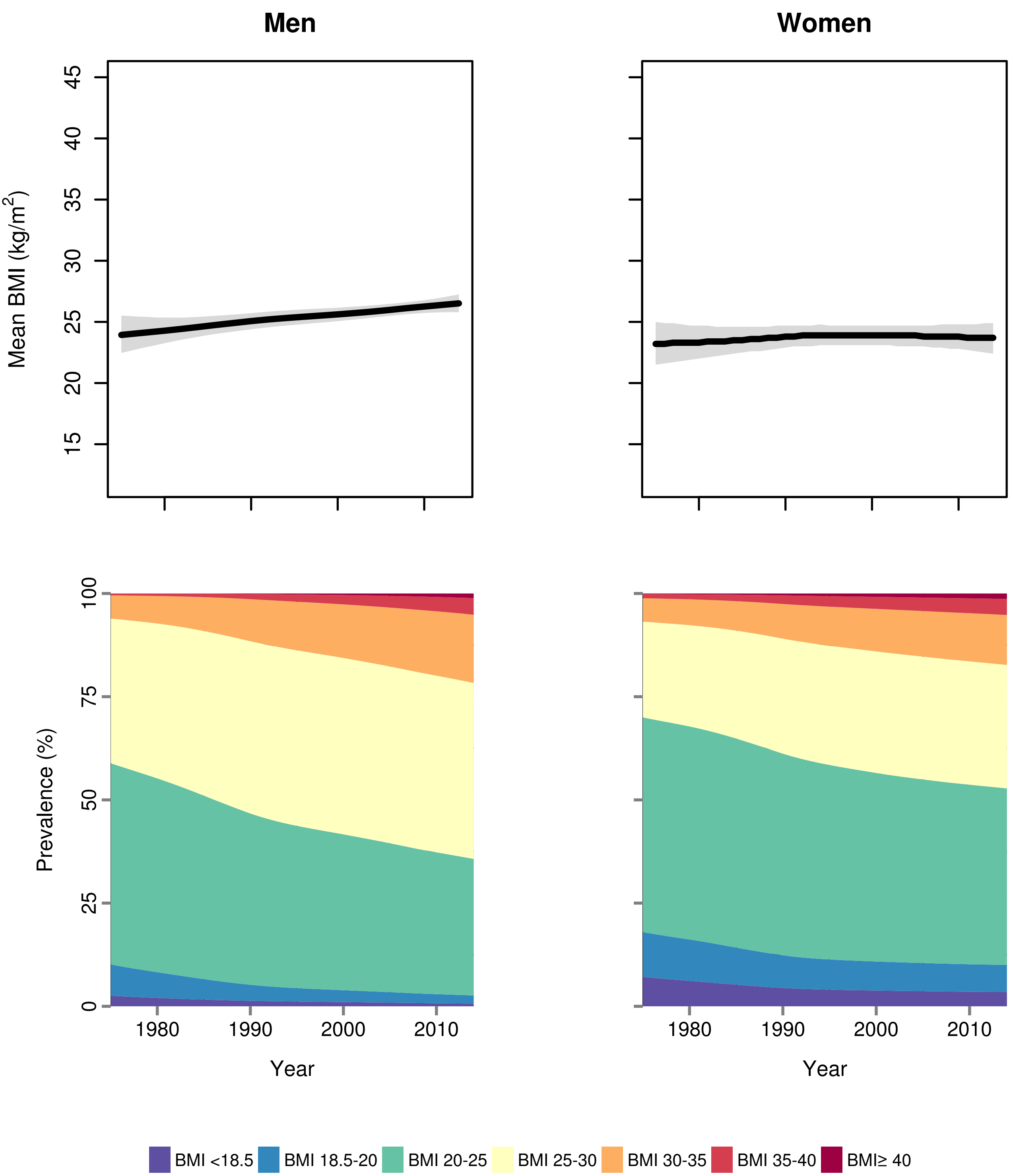

Syrian Arab Republic
Middle East and North Africa

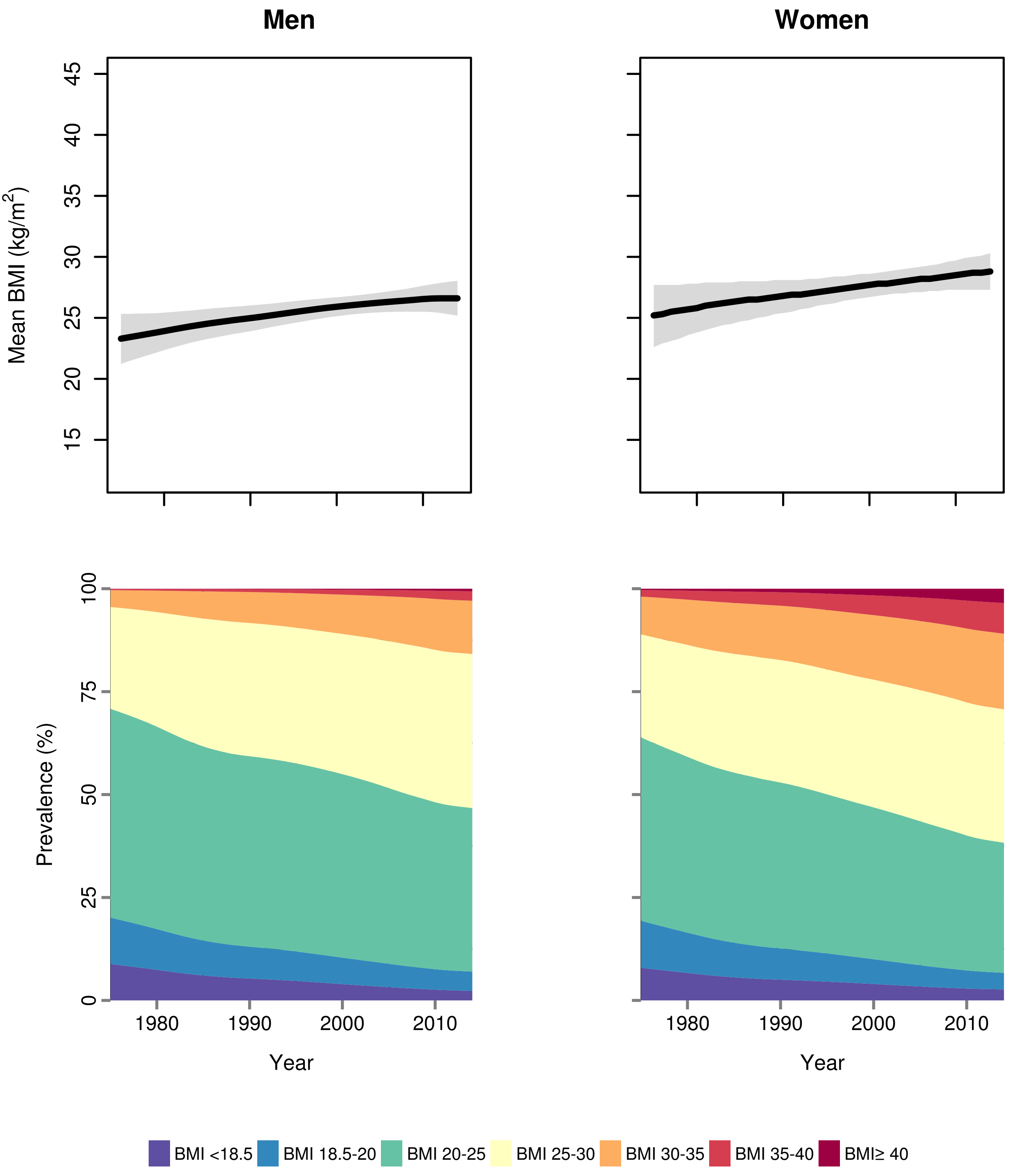

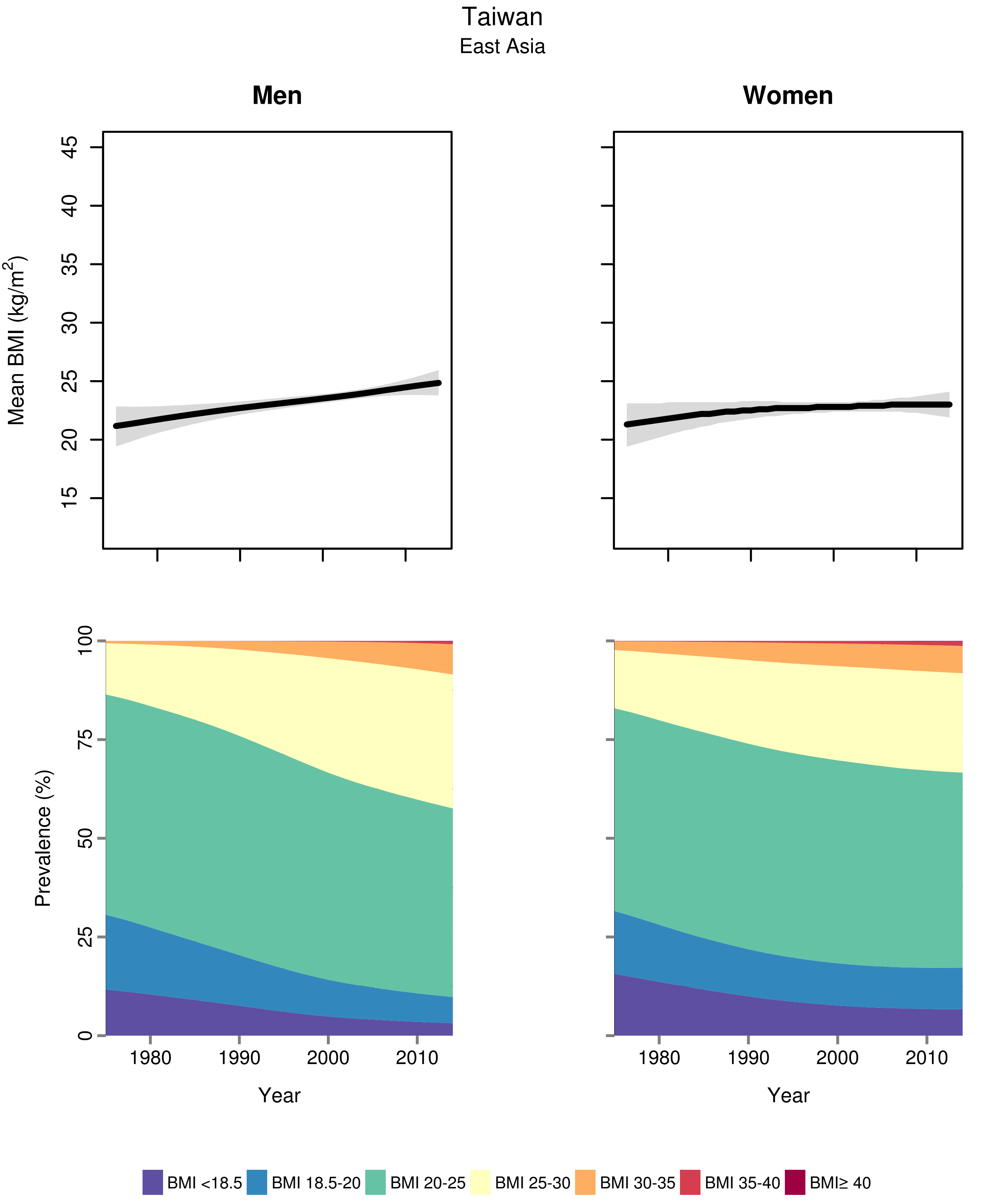
Taiwan
East Asia
Men
Women
Mean BMI (kg/m2)
45
40
35
30
25
20
15
100
75
50
25
0
Prevalence (%)
1980
1990
2000
2010
Year
BMI <18.5
BMI 18.5-20
BMI 20-25
BMI 25-30
BMI 30-35
BMI 35-40
BMI≥ 40

Tajikistan
Central Asia

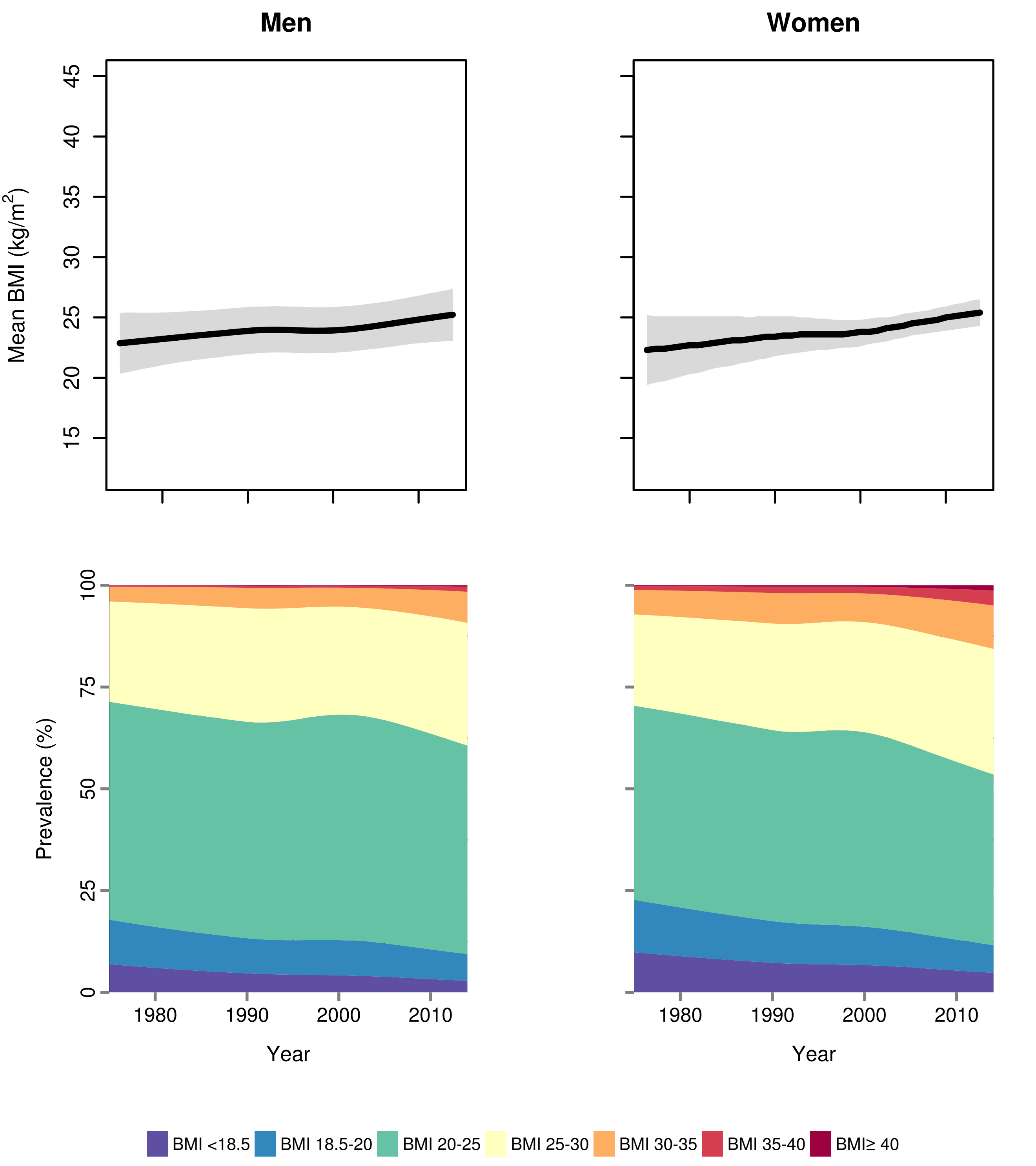

Tanzania
East Africa

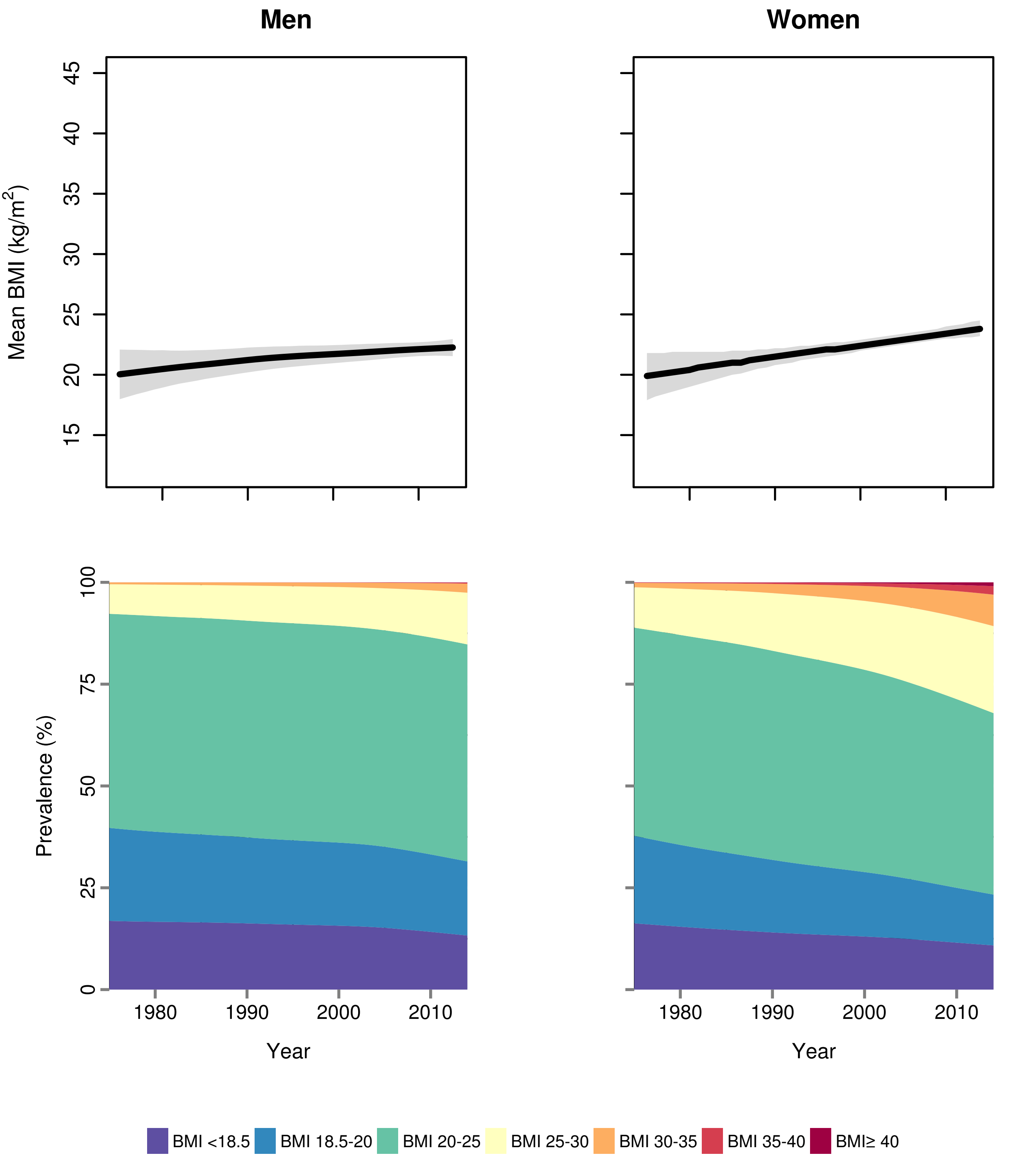

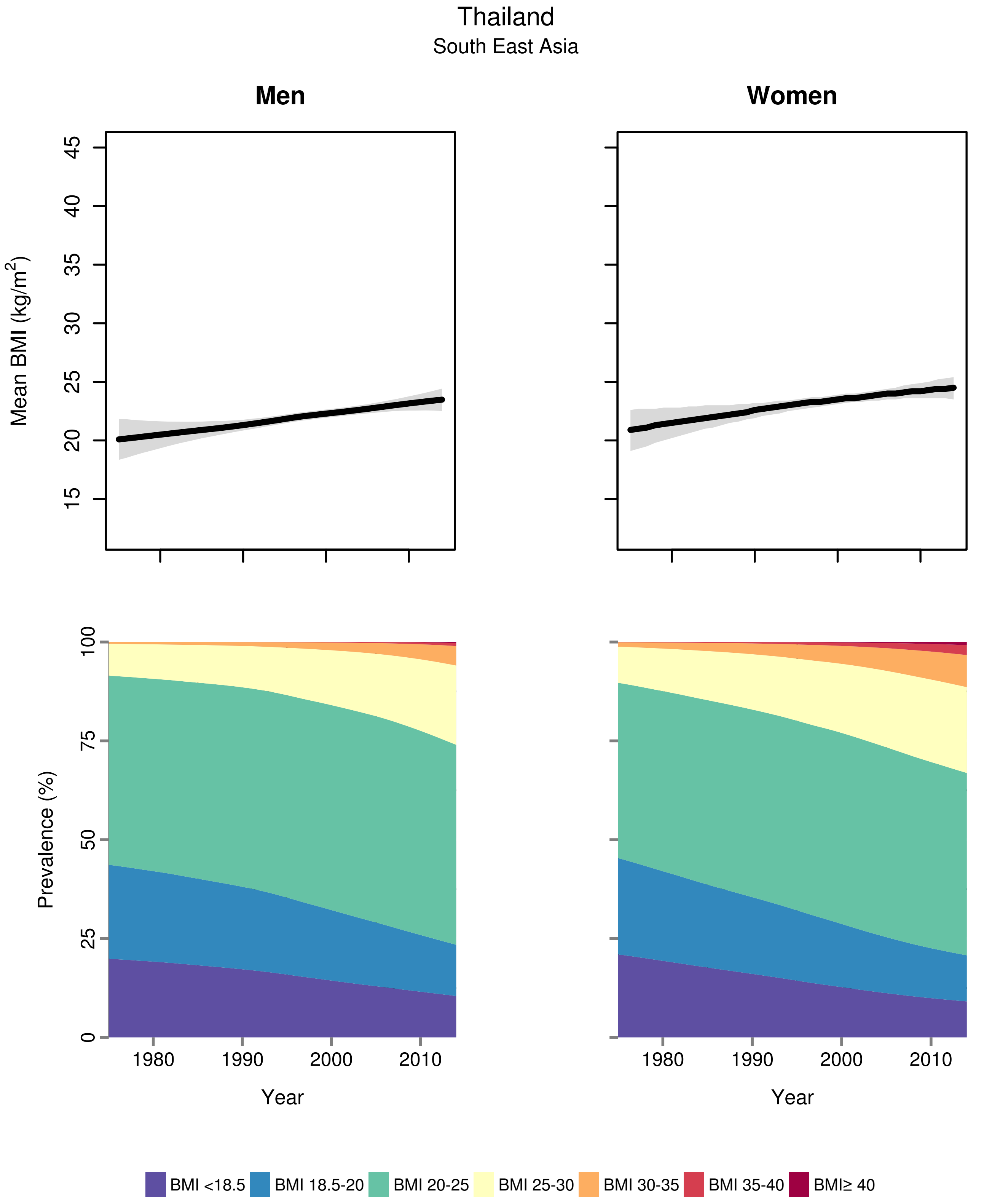

Thailand
South East Asia
Men
Women
Mean BMI (kg/m²)
45
40
35
30
25
20
15
Prevalence (%)
100
75
50
25
0
1980
1990
2000
2010
Year
BMI <18.5
BMI 18.5-20
BMI 20-25
BMI 25-30
BMI 30-35
BMI 35-40
BMI≥ 40

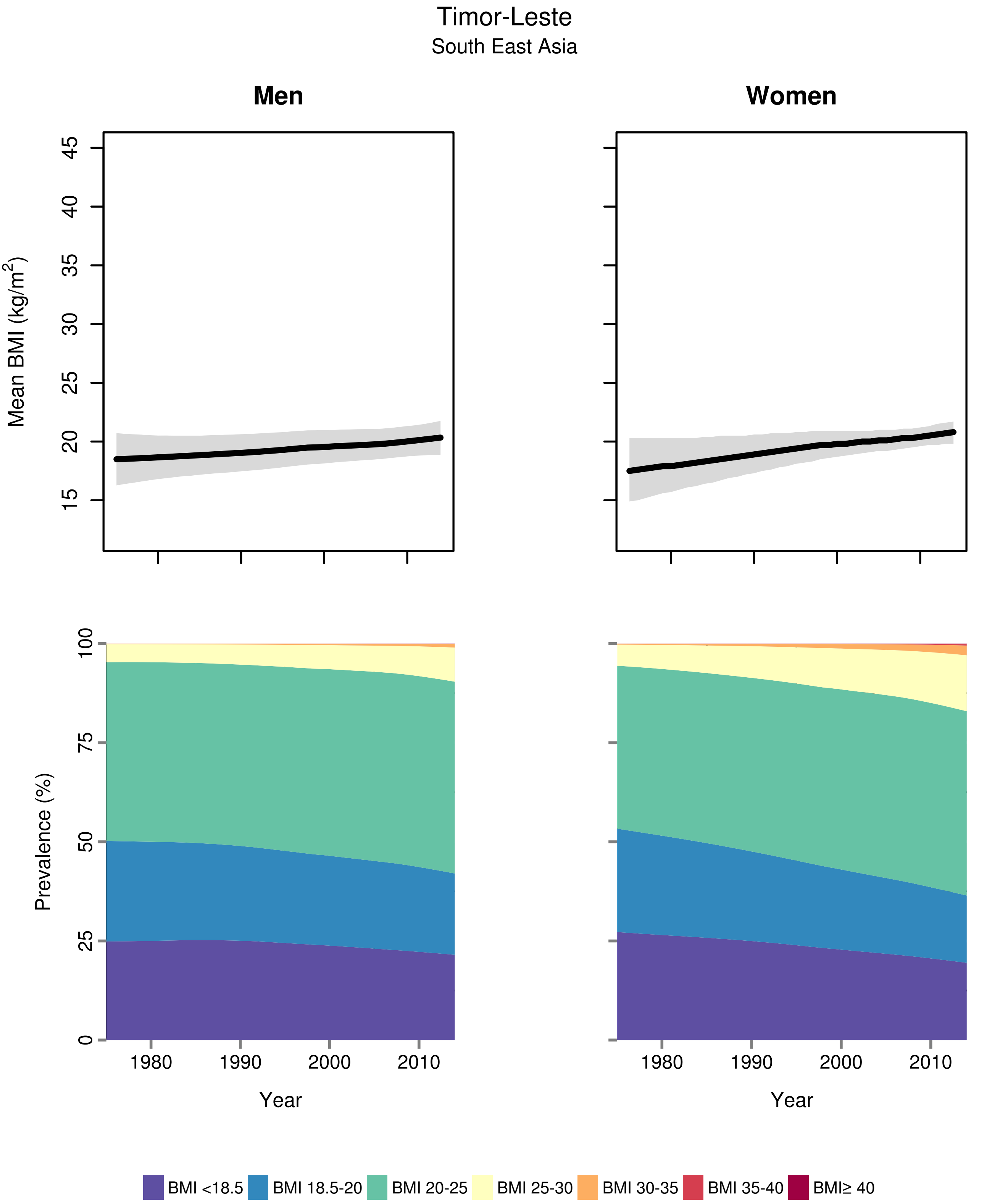

Timor-Leste
South East Asia
Men
Women
Mean BMI (kg/m2)
45
40
35
30
25
20
15
100
75
50
25
0
Prevalence (%)
1980
1990
2000
2010
Year
BMI <18.5
BMI 18.5-20
BMI 20-25
BMI 25-30
BMI 30-35
BMI 35-40
BMI≥ 40

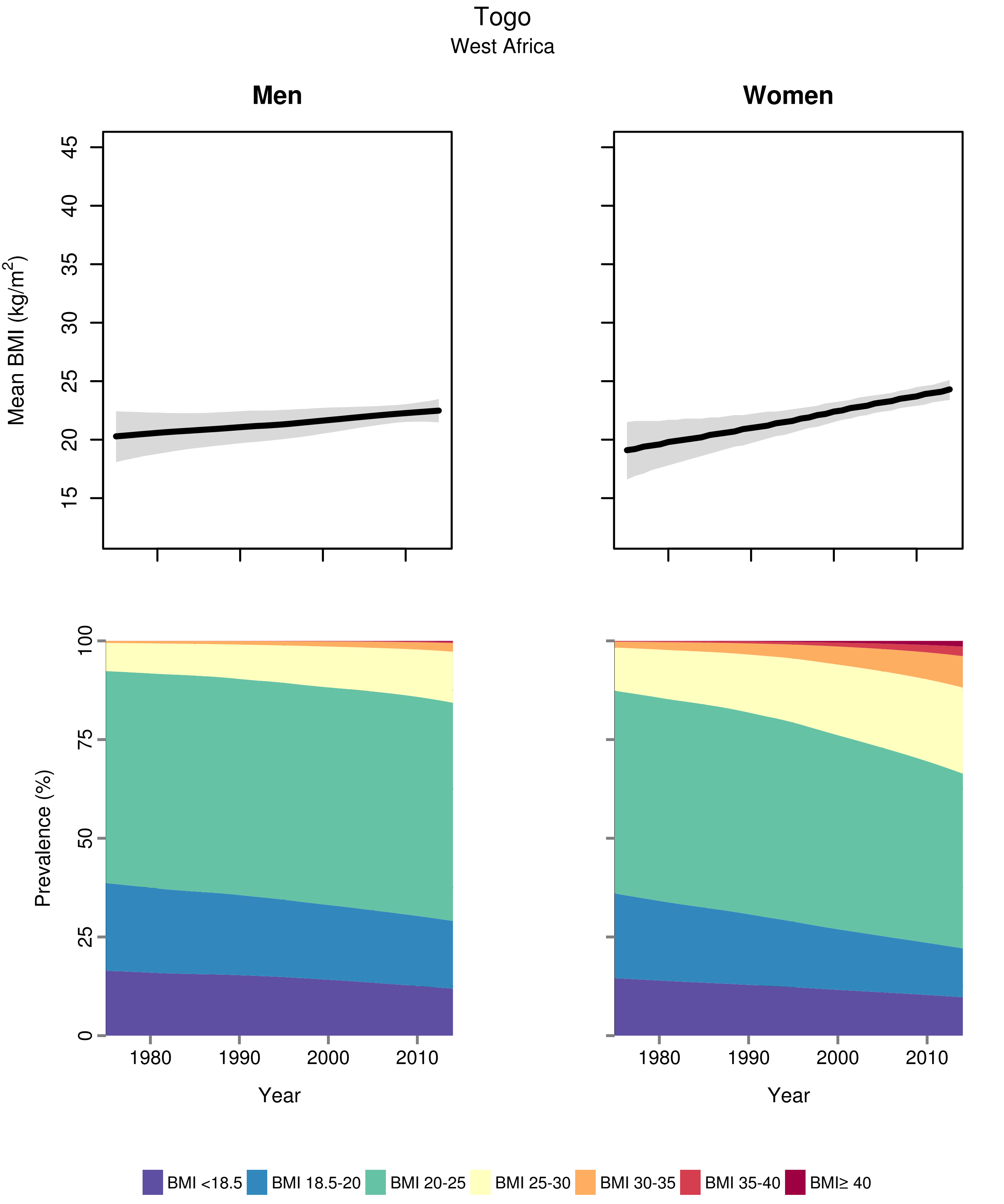

Togo
West Africa
Men
Women
Mean BMI (kg/m2)
45
40
35
30
25
20
15
Prevalence (%)
100
75
50
25
0
1980
1990
2000
2010
Year
BMI <18.5
BMI 18.5-20
BMI 20-25
BMI 25-30
BMI 30-35
BMI 35-40
BMI≥ 40

Tokelau

Polynesia and Micronesia

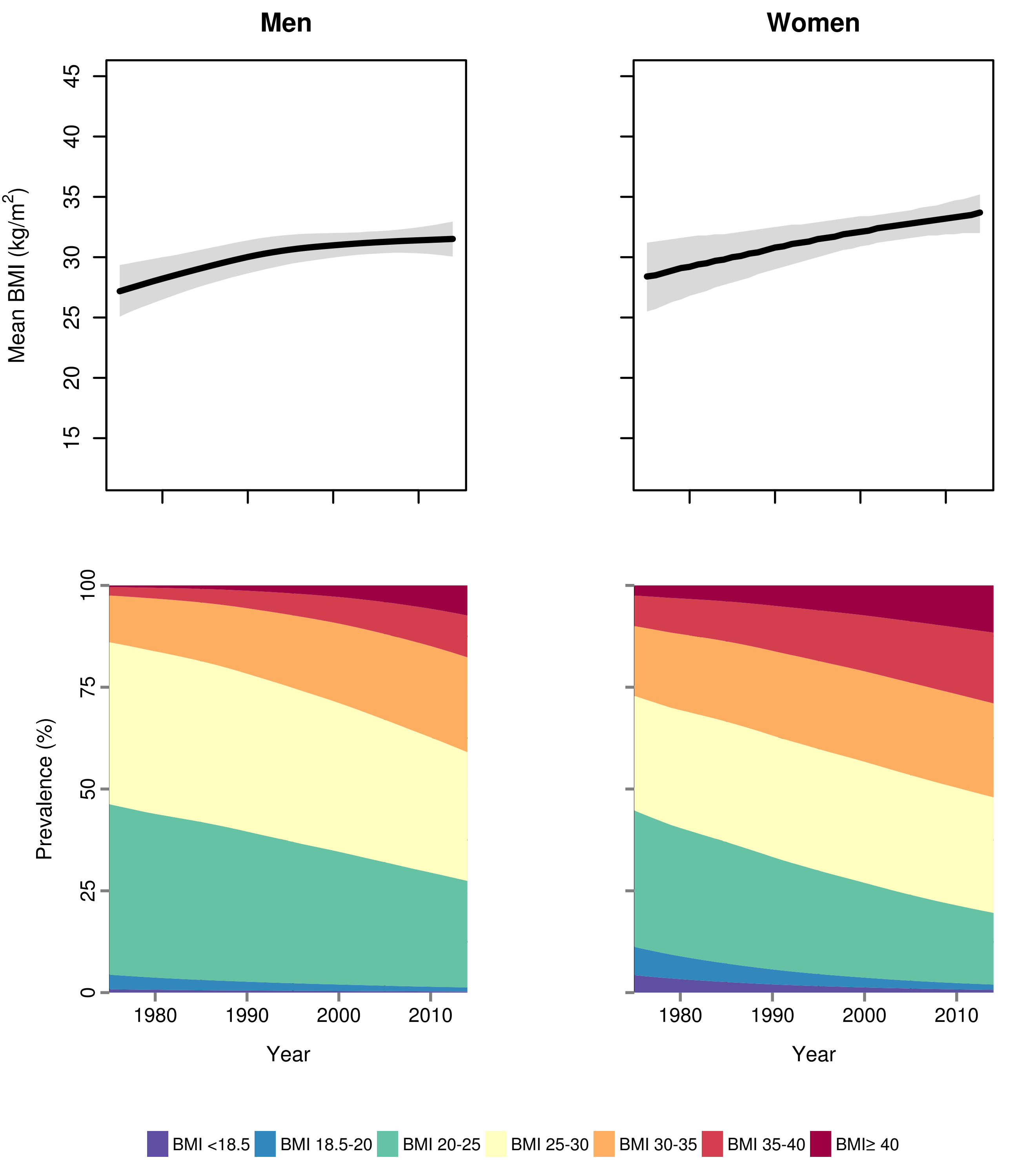

Tonga

Polynesia and Micronesia

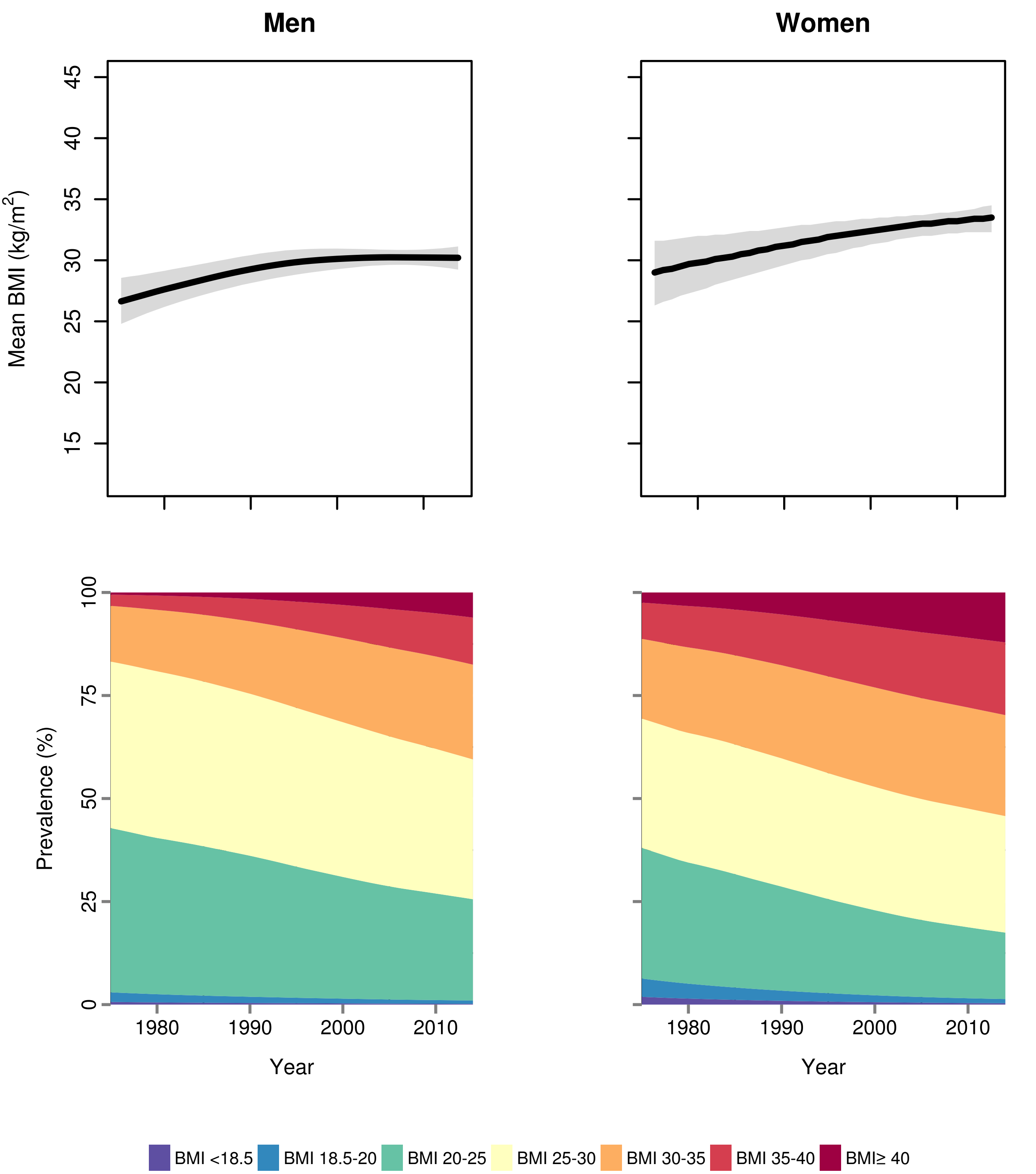

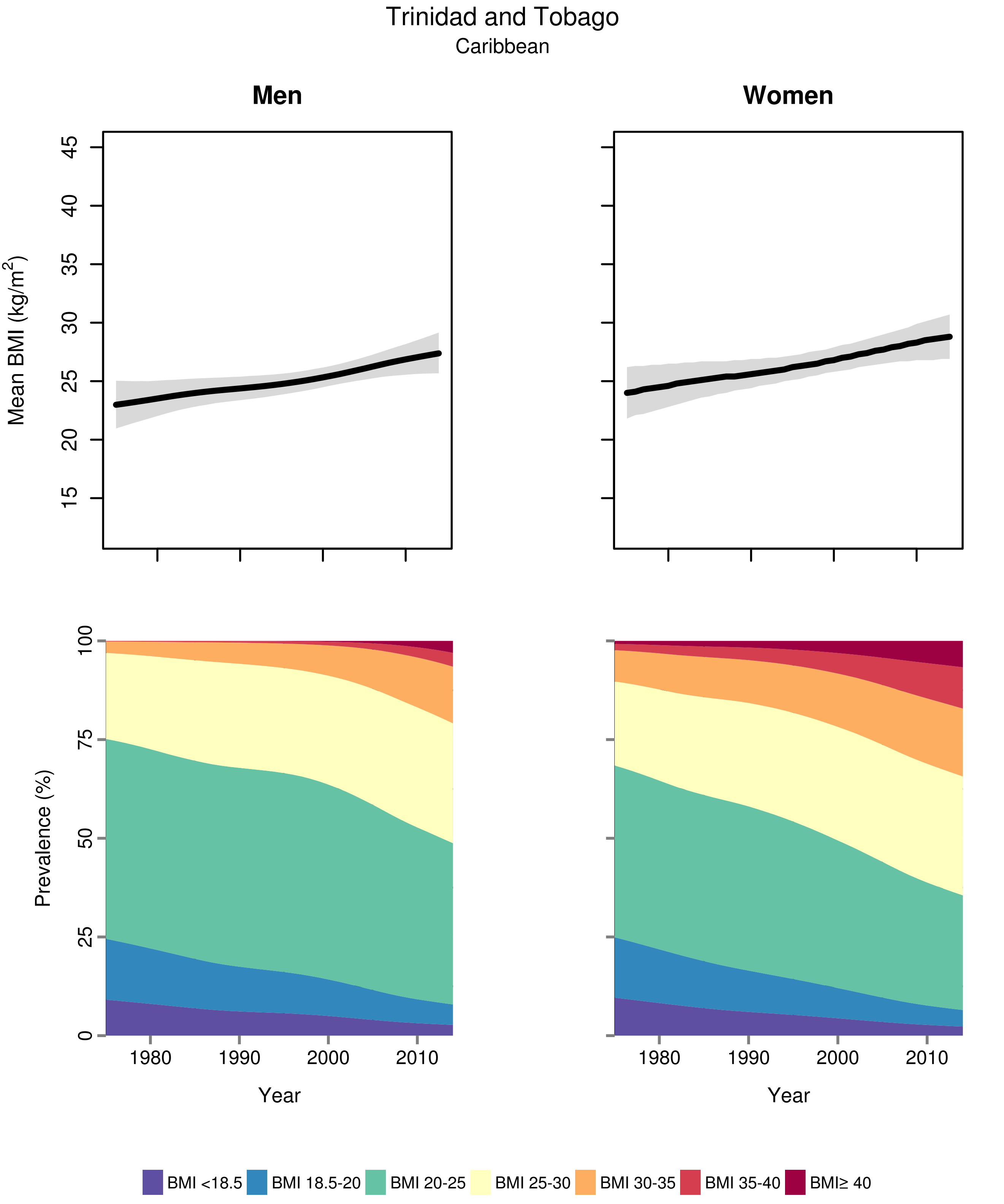
Trinidad and Tobago
Caribbean
Men
Women
Mean BMI (kg/m2)
45
40
35
30
25
20
15
Prevalence (%)
100
75
50
25
0
1980
1990
2000
2010
Year
BMI <18.5
BMI 18.5-20
BMI 20-25
BMI 25-30
BMI 30-35
BMI 35-40
BMI≥ 40

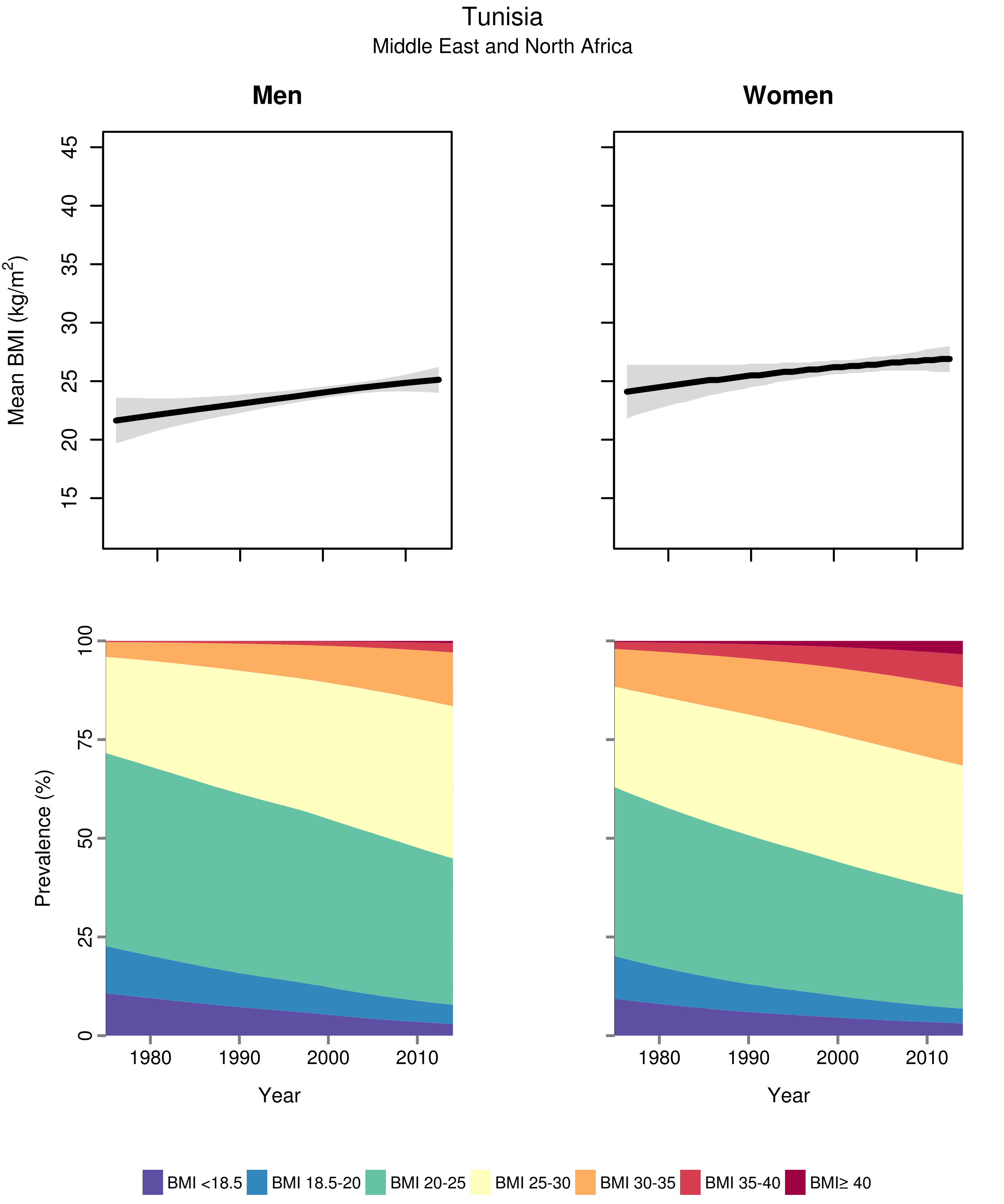
Tunisia
Middle East and North Africa
Men
Women
Mean BMI (kg/m2)
15
20
25
30
35
40
45
Prevalence (%)
0
25
50
75
100
1980
1990
2000
2010
Year
BMI <18.5
BMI 18.5-20
BMI 20-25
BMI 25-30
BMI 30-35
BMI 35-40
BMI≥ 40

Turkey

Middle East and North Africa

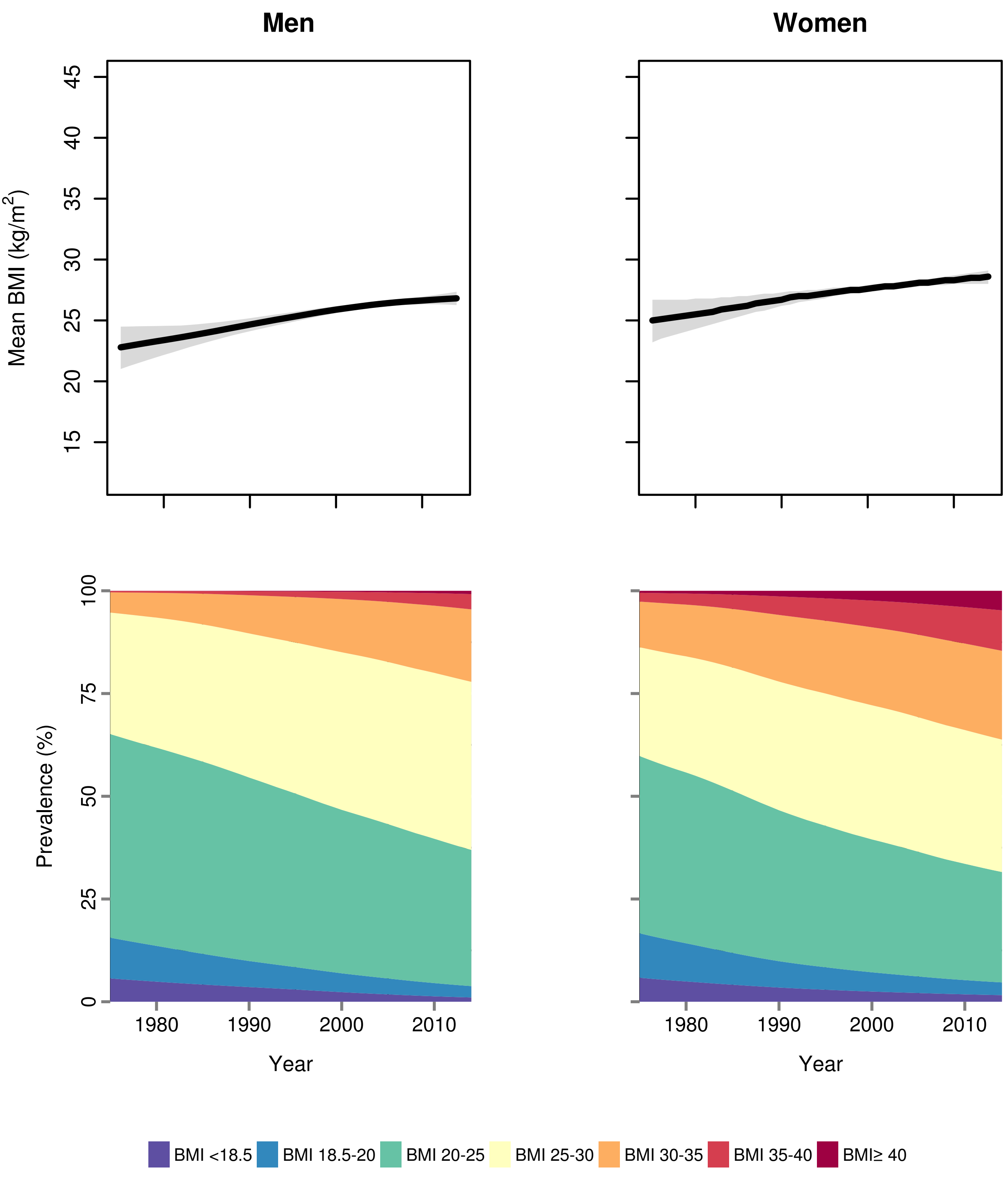

Turkmenistan
Central Asia

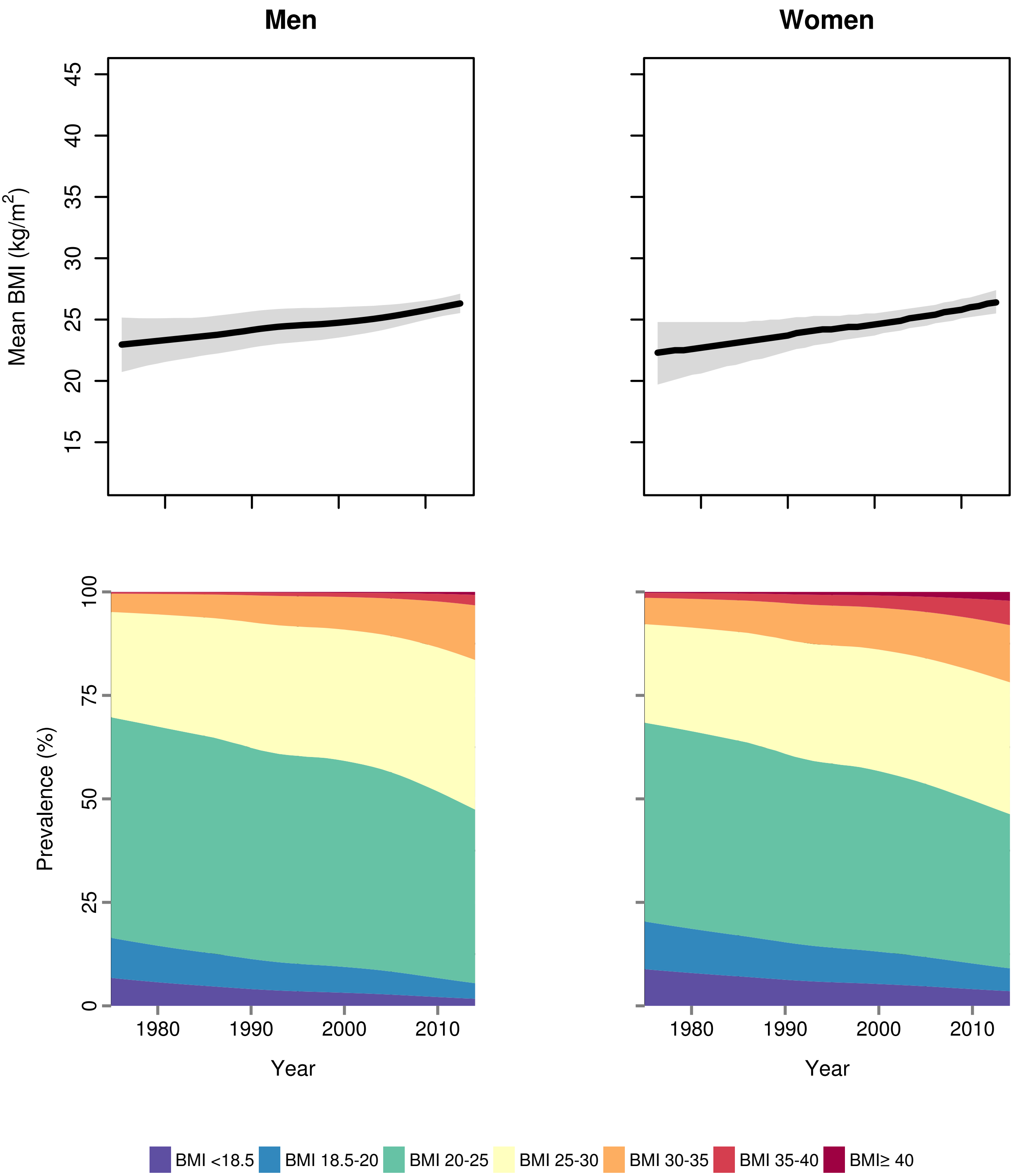

Tuvalu

Polynesia and Micronesia

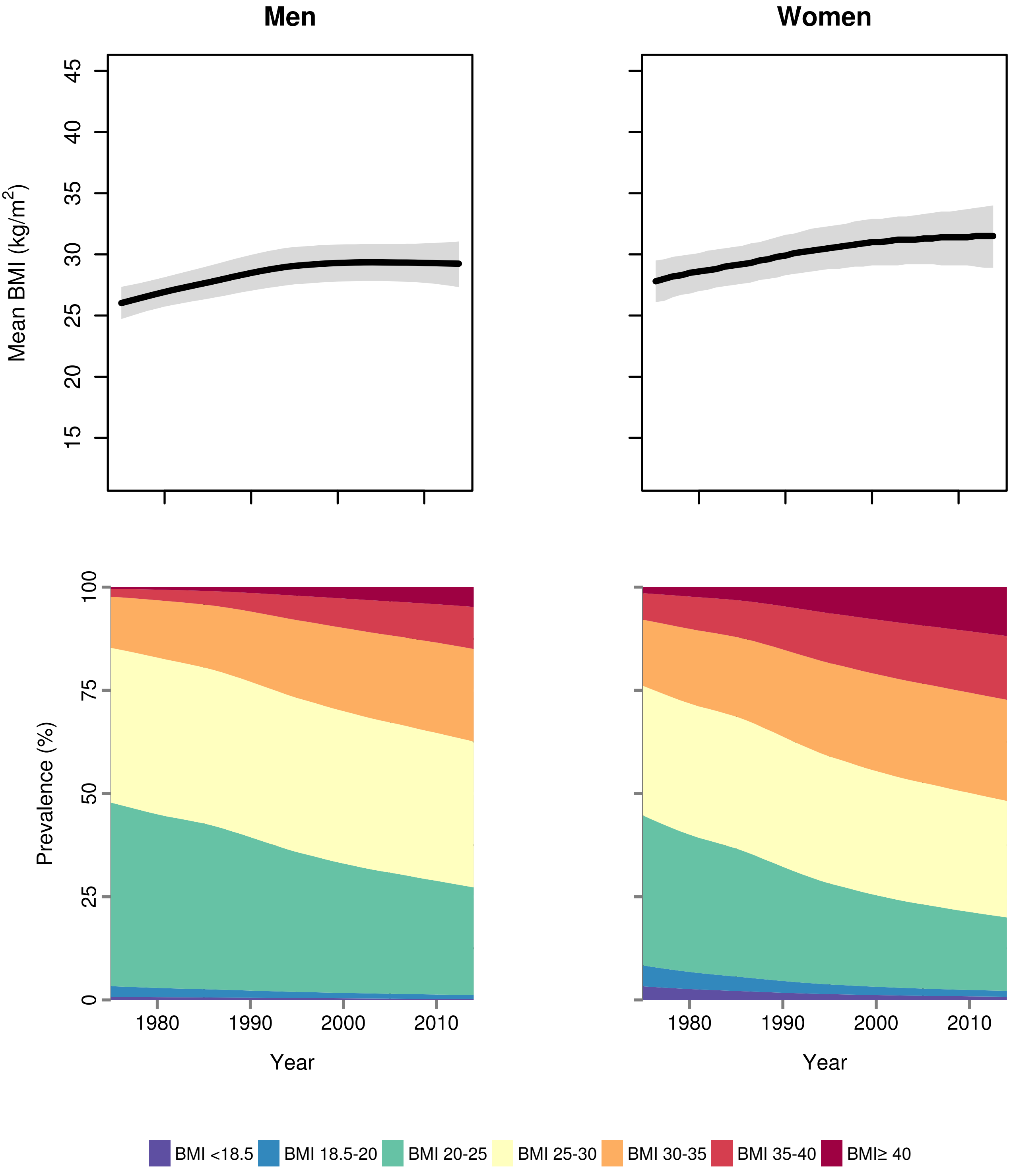

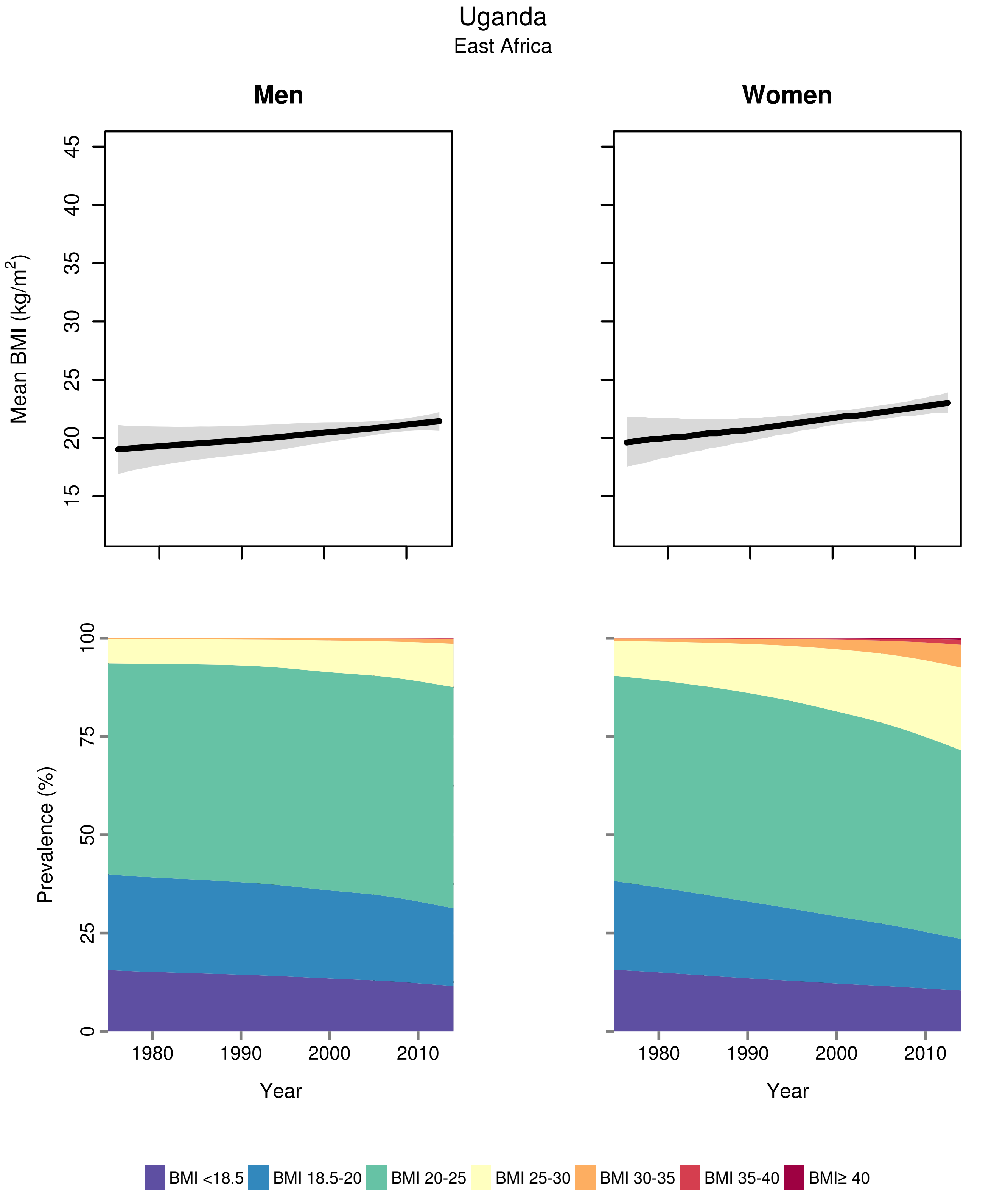

Uganda
East Africa
Men
Women
Mean BMI (kg/m²)
45
40
35
30
25
20
15
Prevalence (%)
100
75
50
25
0
1980
1990
2000
2010
Year
BMI <18.5
BMI 18.5-20
BMI 20-25
BMI 25-30
BMI 30-35
BMI 35-40
BMI≥ 40

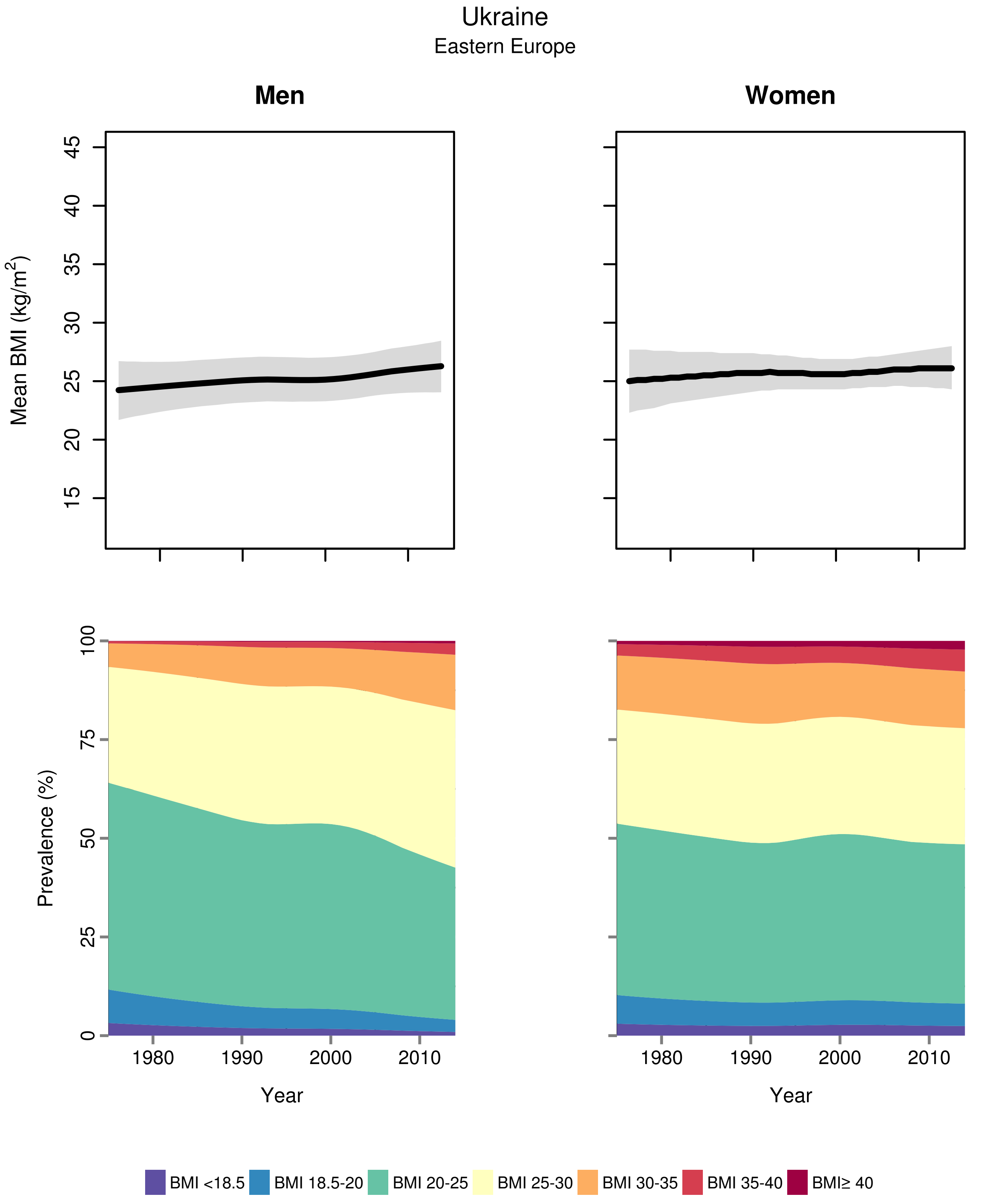
Ukraine
Eastern Europe
Men
Women
Mean BMI (kg/m2)
45
40
35
30
25
20
15
Prevalence (%)
100
75
50
25
0
1980
1990
2000
2010
Year
BMI <18.5
BMI 18.5-20
BMI 20-25
BMI 25-30
BMI 30-35
BMI 35-40
BMI≥ 40

United Arab Emirates
Middle East and North Africa

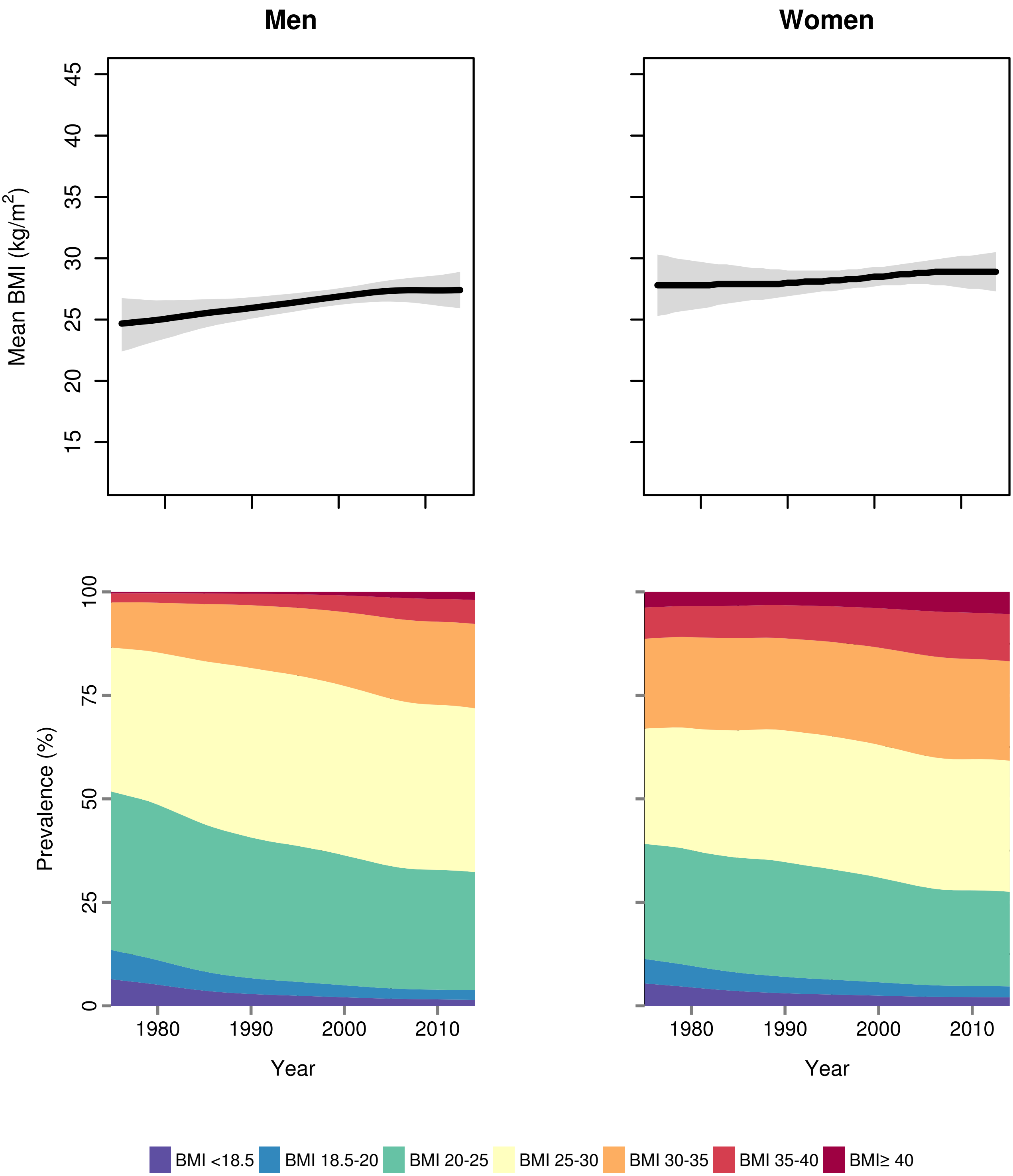

United Kingdom

High-income English-speaking countries

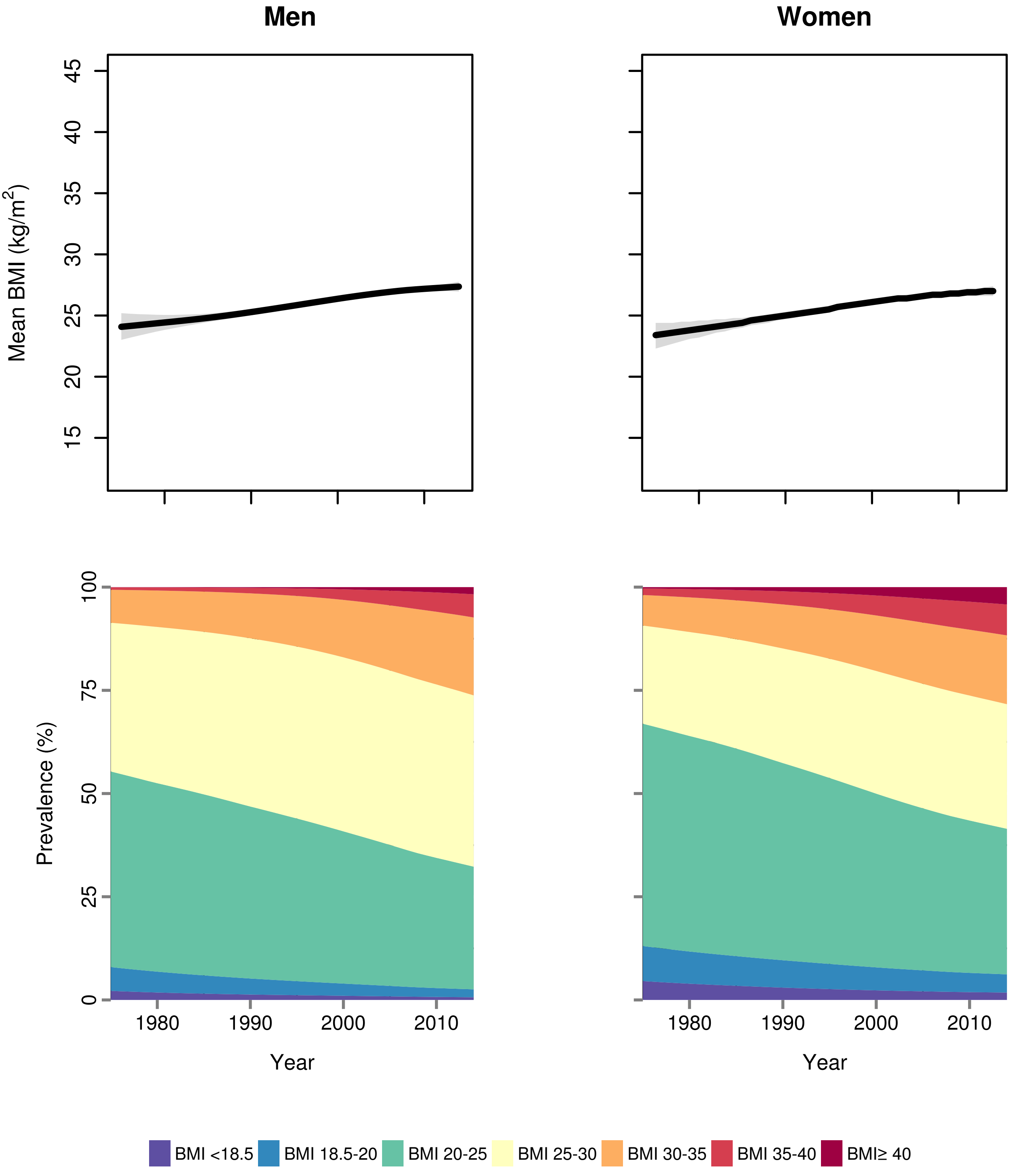

United States of America

High-income English-speaking countries

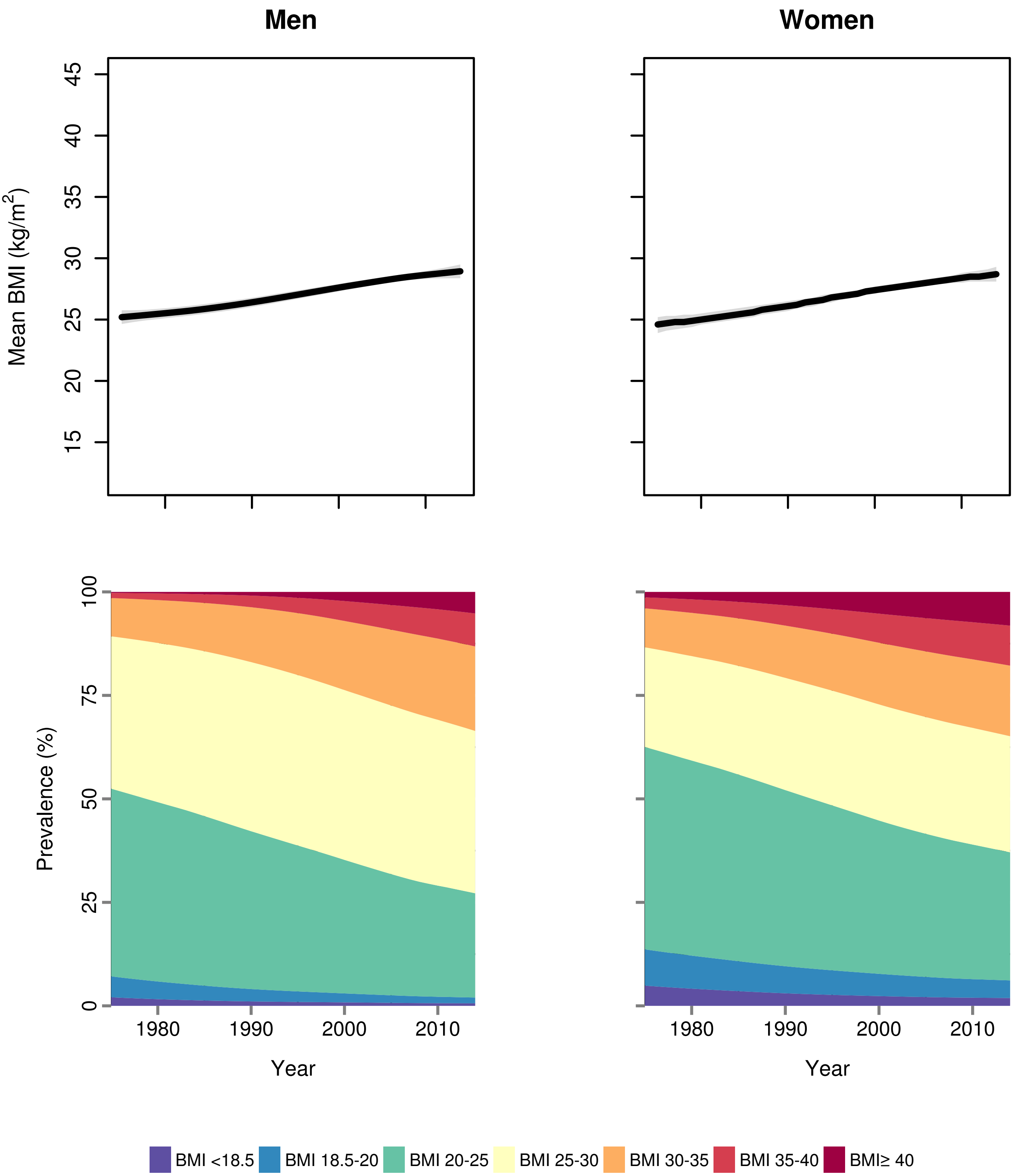

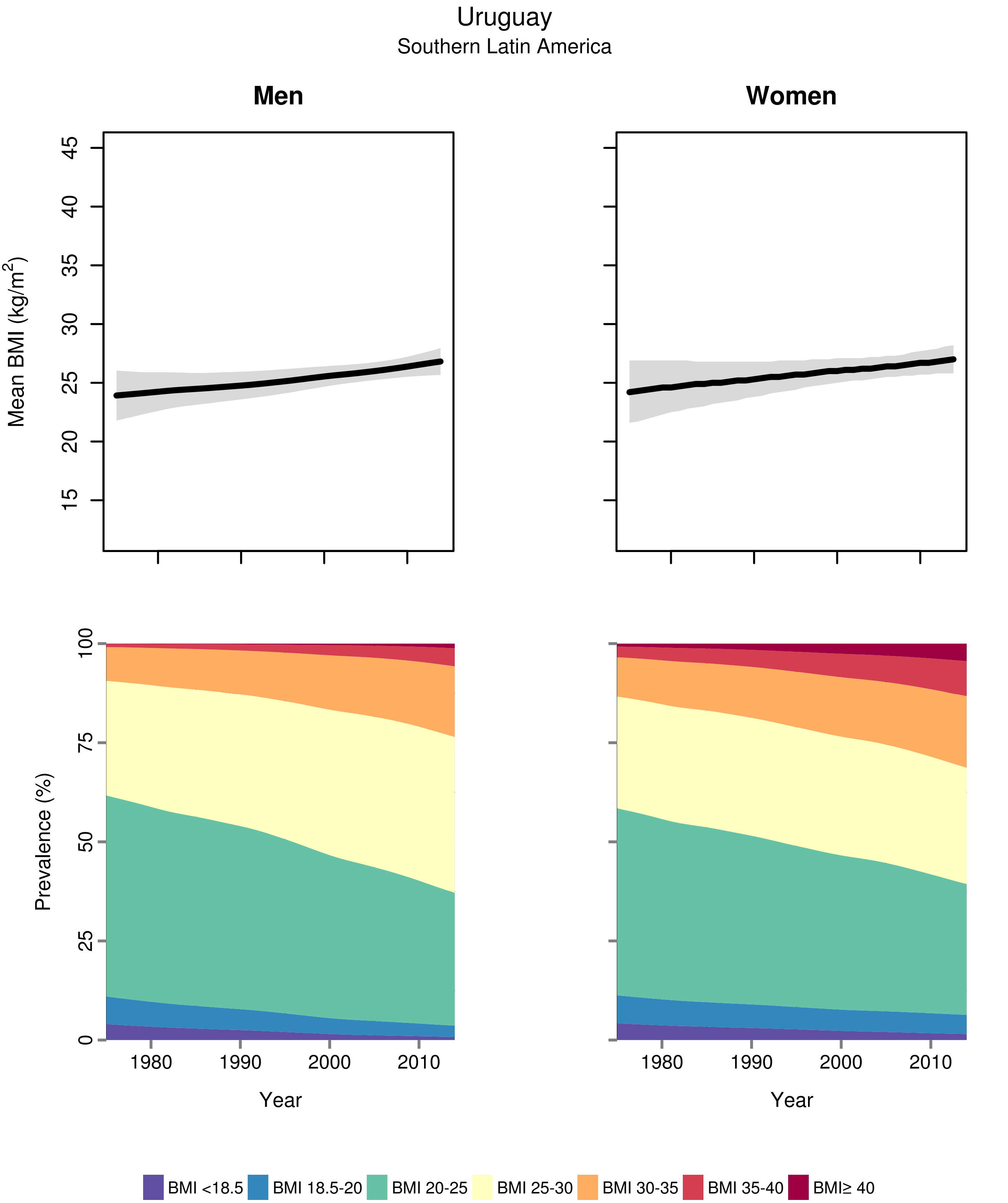

Uruguay
Southern Latin America
Men
Women
Mean BMI (kg/m2)
45
40
35
30
25
20
15
Prevalence (%)
100
75
50
25
0
1980
1990
2000
2010
Year
BMI <18.5
BMI 18.5-20
BMI 20-25
BMI 25-30
BMI 30-35
BMI 35-40
BMI≥ 40

Uzbekistan

Central Asia

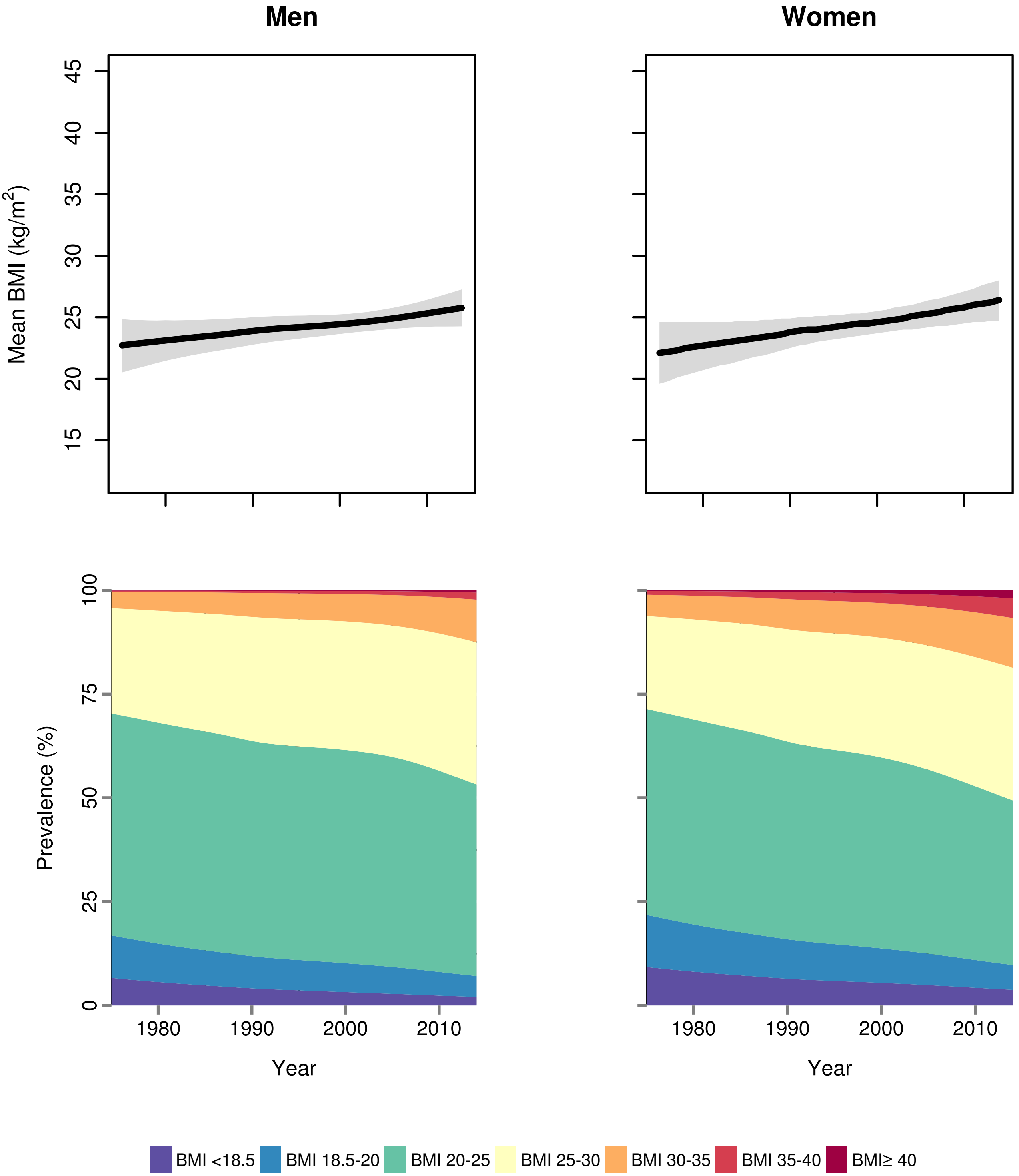

Vanuatu

Melanesia

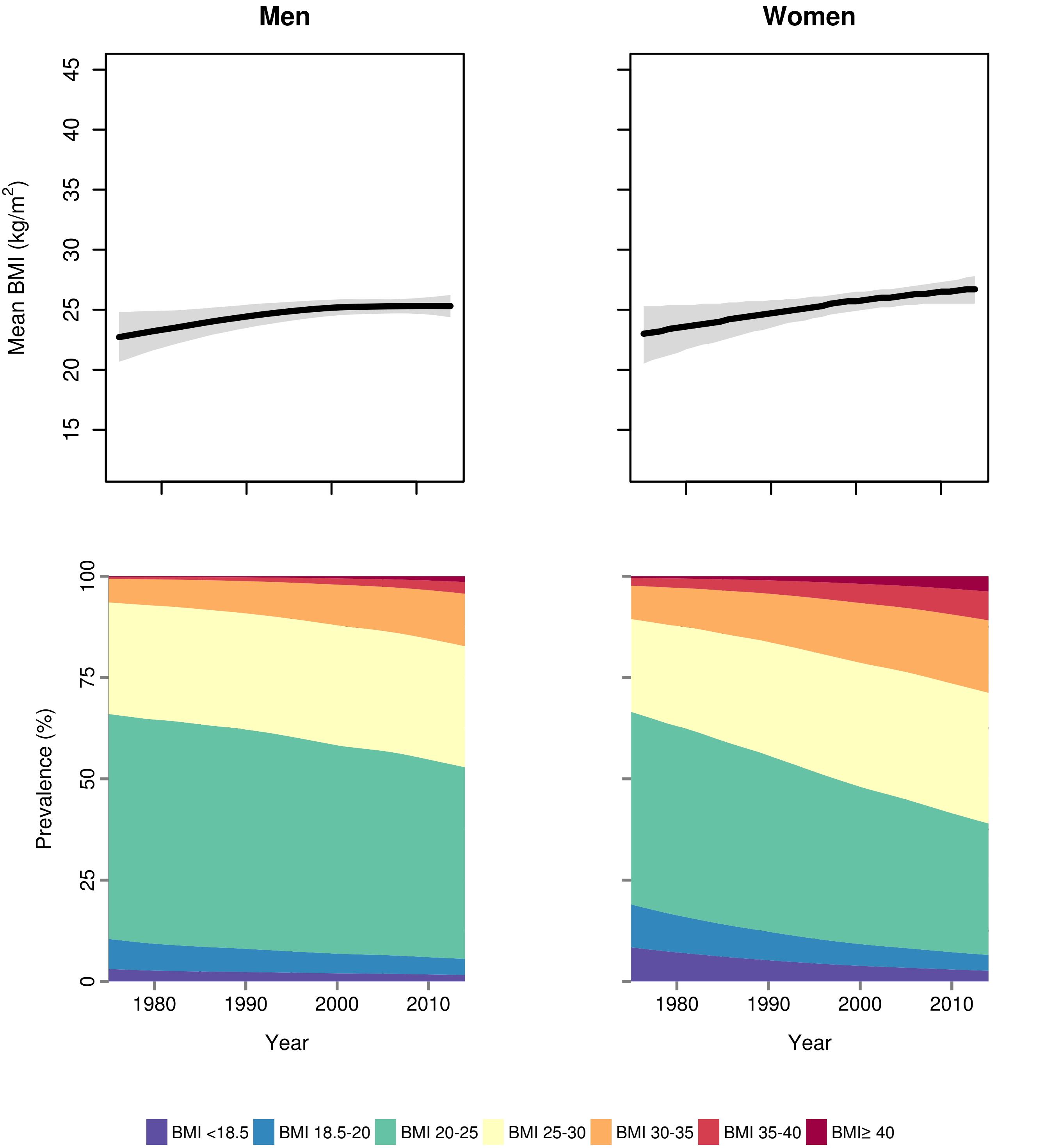

Venezuela

Central Latin America

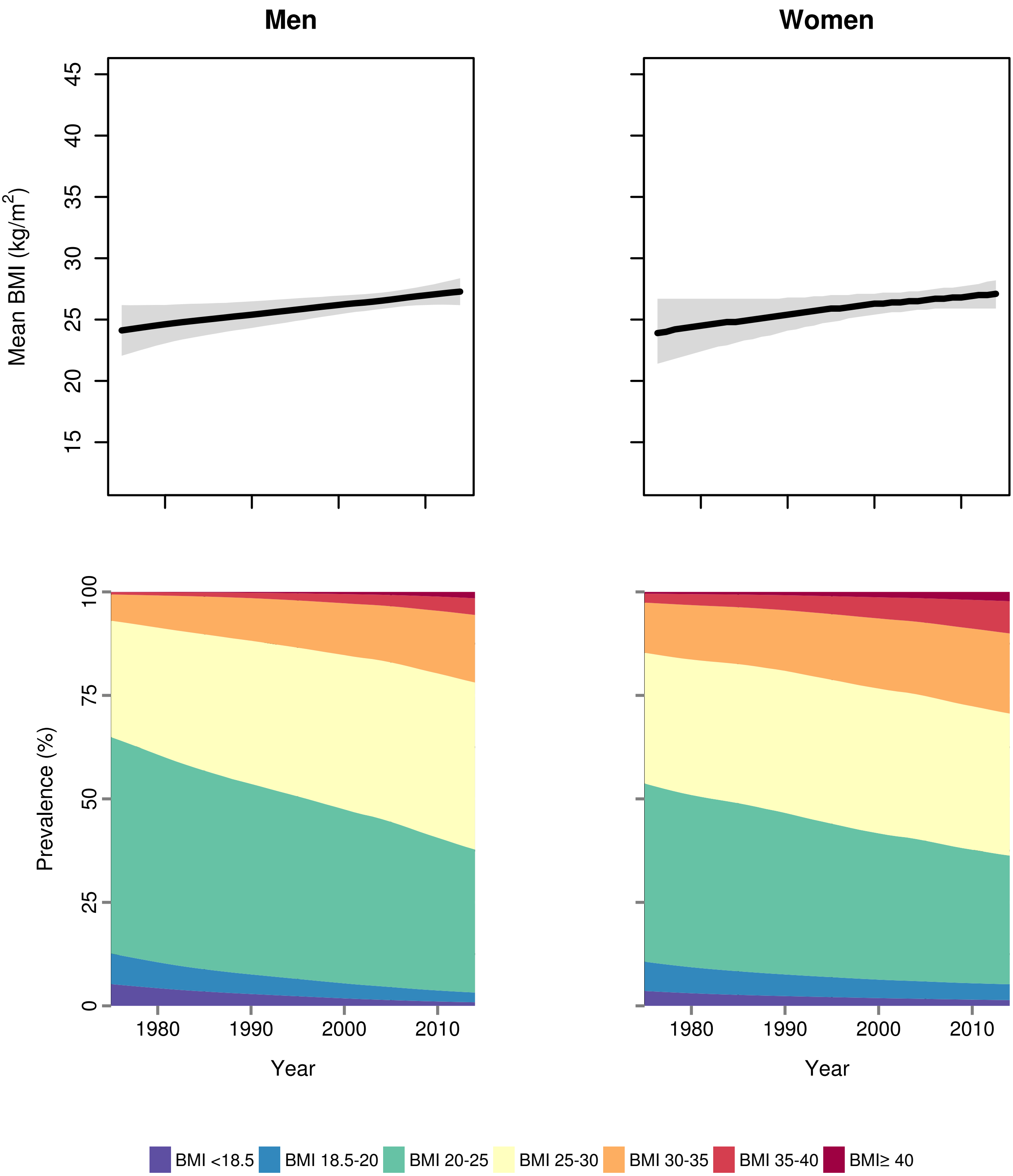

Viet Nam

South East Asia

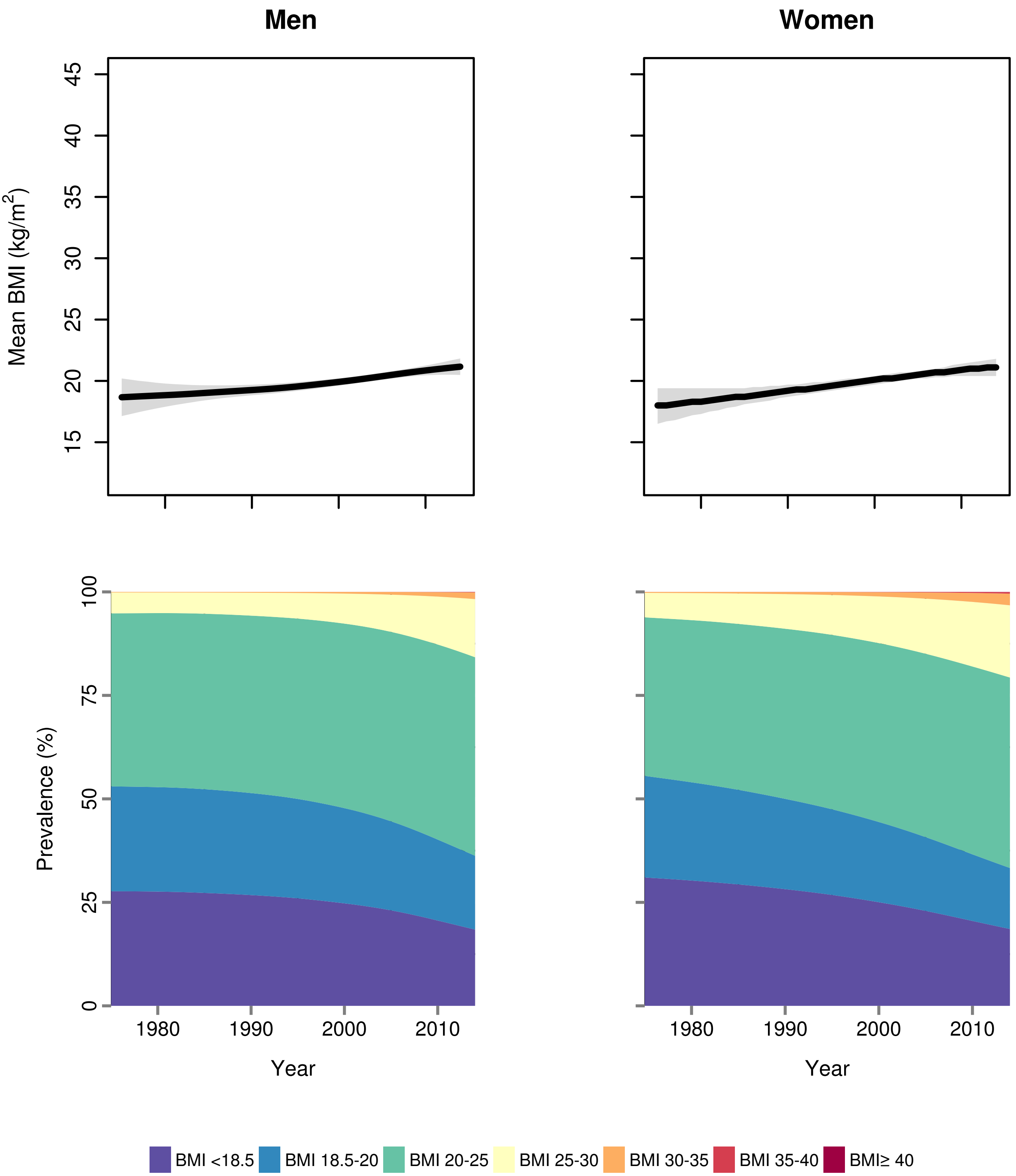

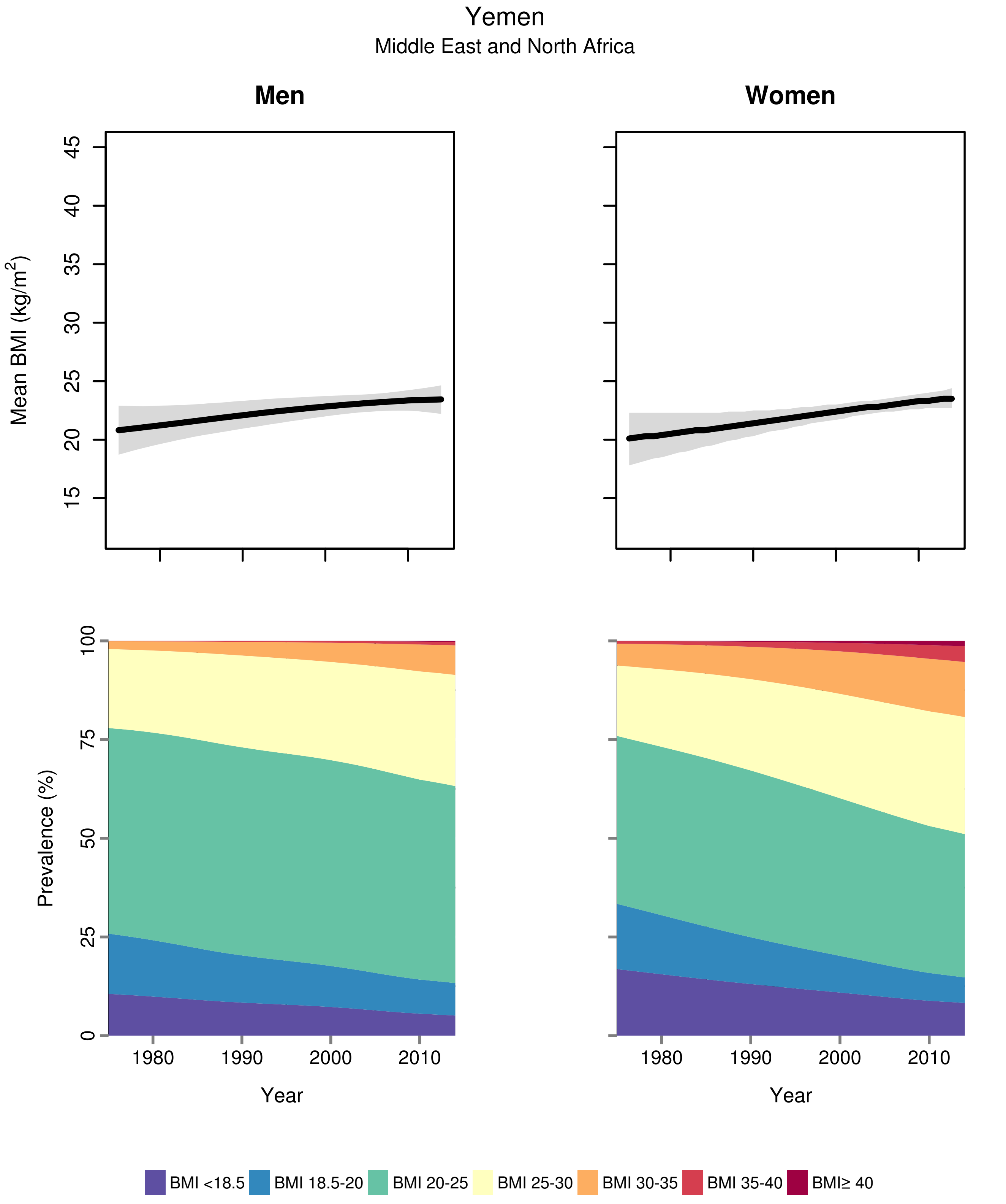
Yemen
Middle East and North Africa
Men
Women
Mean BMI (kg/m2)
45
40
35
30
25
20
15
Prevalence (%)
100
75
50
25
0
1980
1990
2000
2010
Year
BMI <18.5
BMI 18.5-20
BMI 20-25
BMI 25-30
BMI 30-35
BMI 35-40
BMI≥ 40

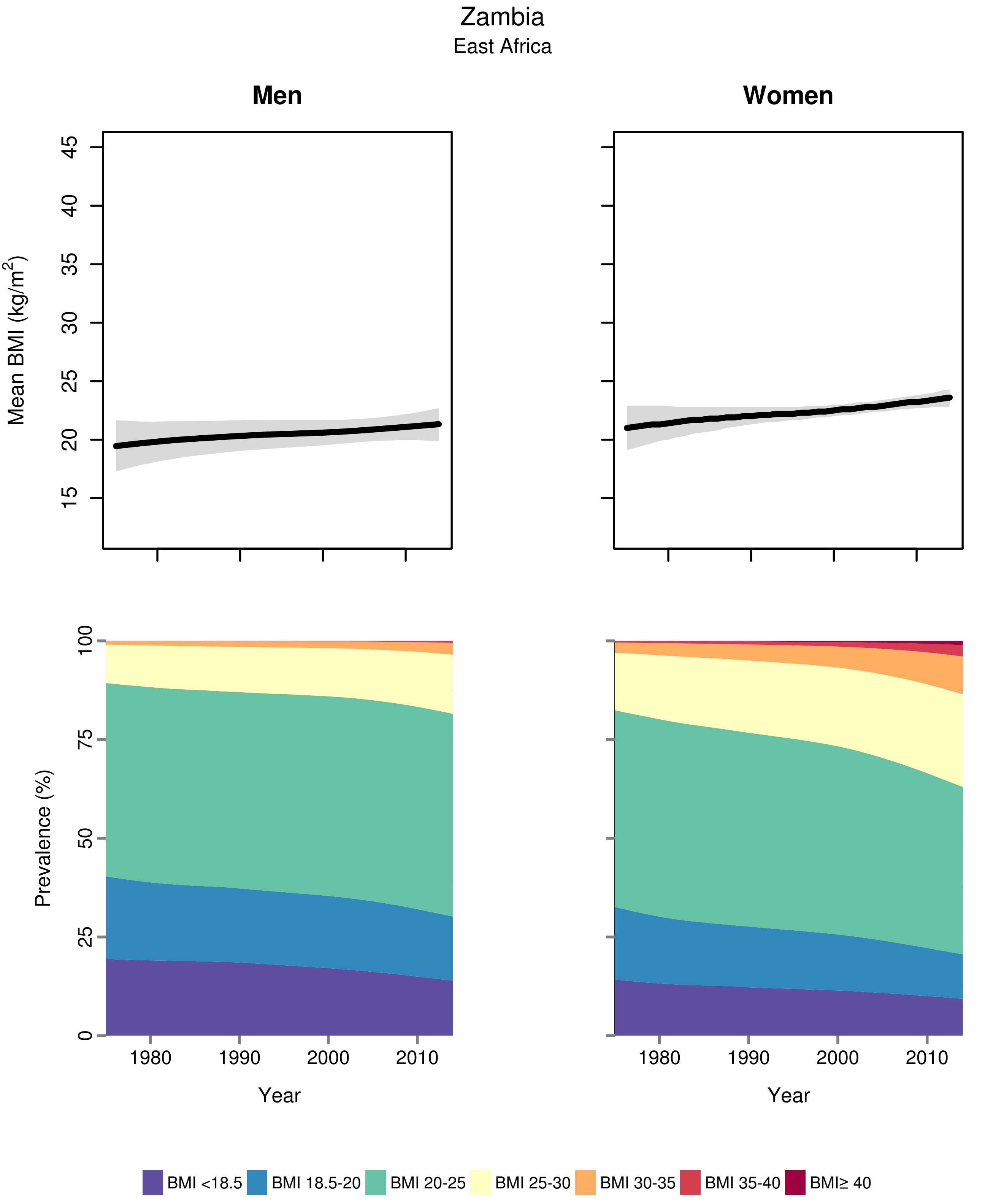

Zambia
East Africa
Men
Women
Mean BMI (kg/m2)
45
40
35
30
25
20
15
Prevalence (%)
100
75
50
25
0
1980
1990
2000
2010
Year
BMI <18.5
BMI 18.5-20
BMI 20-25
BMI 25-30
BMI 30-35
BMI 35-40
BMI≥ 40

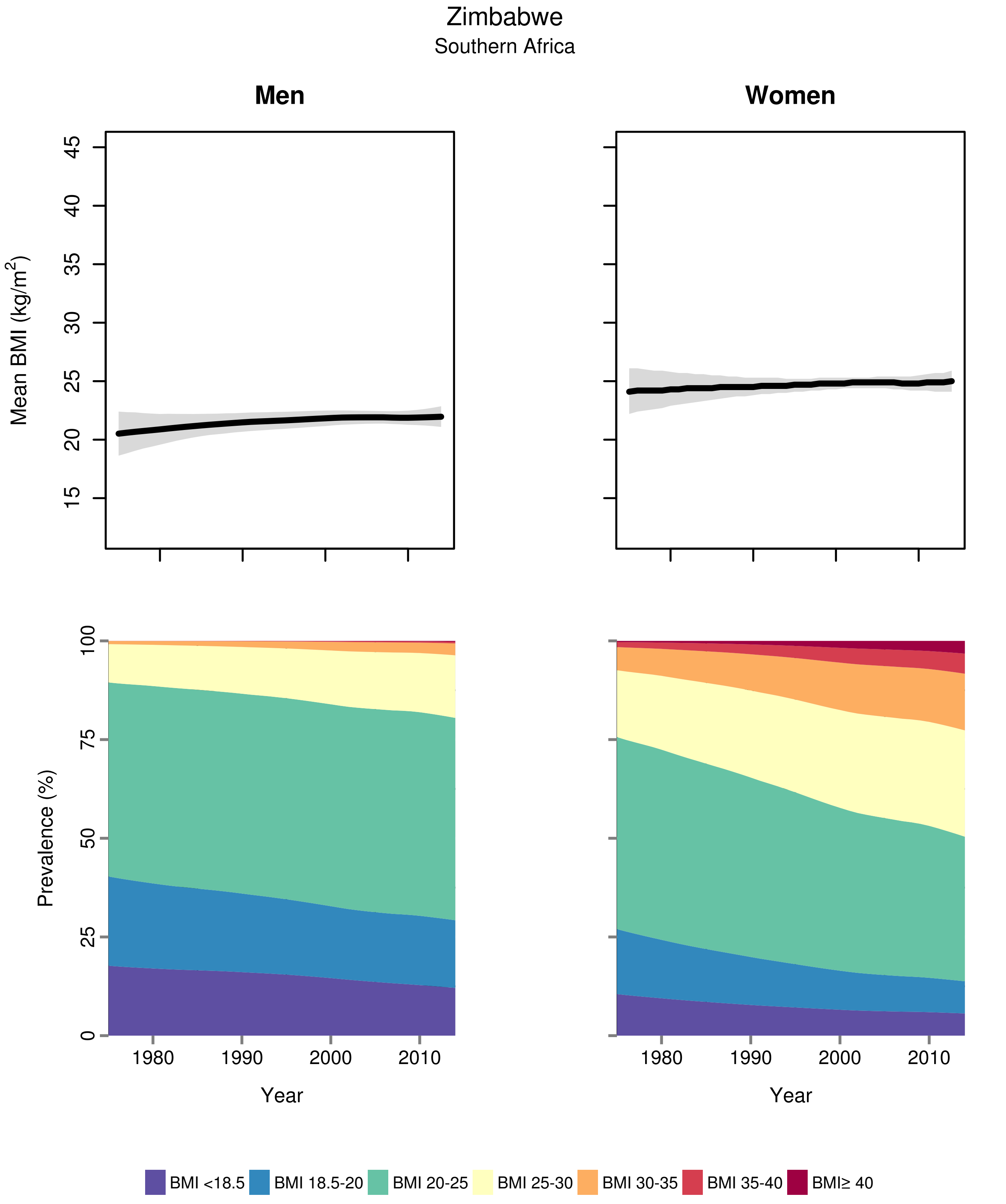
Zimbabwe
Southern Africa
Men
Women
Mean BMI (kg/m2)
45
40
35
30
25
20
15
Prevalence (%)
100
75
50
25
0
1980
1990
2000
2010
Year
BMI <18.5
BMI 18.5-20
BMI 20-25
BMI 25-30
BMI 30-35
BMI 35-40
BMI≥ 40
